# Identification of *Dirofilaria immitis* miRNA using illumina deep sequencing

**DOI:** 10.1186/1297-9716-44-3

**Published:** 2013-01-18

**Authors:** Yan Fu, Jingchao Lan, Xuhang Wu, Deying Yang, Zhihe Zhang, Huaming Nie, Rong Hou, Runhui Zhang, Wanpeng Zheng, Yue Xie, Ning Yan, Zhi Yang, Chengdong Wang, Li Luo, Li Liu, Xiaobin Gu, Shuxian Wang, Xuerong Peng, Guangyou Yang

**Affiliations:** 1 Department of Parasitology, College of Veterinary Medicine, Sichuan Agricultural University, 625014, Ya’an, China; 2 The Sichuan Key Laboratory for Conservation Biology on Endangered Wildlife – Developing toward a State Key Laboratory for China, Chengdu Research Base of Giant Panda Breeding, 610081, Chengdu, Sichuan, China; 3 Department of Chemistry, College of Life and Basic Science, Sichuan Agricultural University, 625014, Ya’an, China

## Abstract

The heartworm *Dirofilaria immitis* is the causative agent of cardiopulmonary dirofilariosis in dogs and cats, which also infects a wide range of wild mammals and humans. The complex life cycle of *D. immitis* with several developmental stages in its invertebrate mosquito vectors and its vertebrate hosts indicates the importance of miRNA in growth and development, and their ability to regulate infection of mammalian hosts. This study identified the miRNA profiles of *D. immitis* of zoonotic significance by deep sequencing. A total of 1063 conserved miRNA candidates, including 68 anti-sense miRNA (miRNA*) sequences, were predicted by computational methods and could be grouped into 808 miRNA families. A significant bias towards family members, family abundance and sequence nucleotides was observed. Thirteen novel miRNA candidates were predicted by alignment with the *Brugia malayi* genome. Eleven out of 13 predicted miRNA candidates were verified by using a PCR-based method. Target genes of the novel miRNA candidates were predicted by using the heartworm transcriptome dataset. To our knowledge, this is the first report of miRNA profiles in *D. immitis*, which will contribute to a better understanding of the complex biology of this zoonotic filarial nematode and the molecular regulation roles of miRNA involved. Our findings may also become a useful resource for small RNA studies in other filarial parasitic nematodes.

## Introduction

The heartworm *Dirofilaria immitis*, a member of the clade III of filarial parasitic nematodes [1], is the causative agent of cardiopulmonary dirofilariosis. It affects domestic dogs, cats and various wild mammals with increasing incidence in temperate and tropical areas [2,3]. As mosquito-borne zoonotic pathogens, heartworms can also be transmitted to humans, where they cause pulmonary dirofilariosis, since some mosquito vectors feed indistinctly on animal reservoirs and humans [4]. Human dirofilariosis occurs at an increasing rate and hundreds of clinical cases have been reported to date. Due to the population dynamics of humans and animal reservoirs and to climate change, the accelerated introduction of newly competent *Dirofilaria* vectors in non-endemic areas can increase the distribution of this zoonosis [5,6]. To date, the most practical and effective control strategy is chemotherapy against both microfilariae and adult worms, but this still carries potential risks for severe thromboembolism and perivascular inflammation [7,8]. While avermectin-class drugs are widely used for prevention, the American Heartworm Society estimates that 27 million dogs are untreated in the US, as described in Nematode.net [9]. Besides, little progress has been made in the development of vaccines against *D. immitis*[3].

In recent years, the discovery of numerous small endogenous non-coding RNA (sncRNA) has aroused interest in their valuable function in both transcriptional and post-transcriptional gene regulation as well as genome stability and chromatin [10]. According to their biogenesis and Argonaute protein binding partners, small RNA can be simply grouped into three major classes: microRNA (miRNA), small interfering RNA (siRNA), and Piwi-associated RNA (piRNA) [11]. Amongst these, miRNA (20 ~ 24 nt) have recently received special attention since they generally function in post-transcriptional gene silencing in diverse eumetazoans, acting as rheostats to make fine-scale adjustments to the protein output [12,13]. Precursor miRNA are cleaved from the primary transcripts by Drosha and DGCR8 [14-16] in the nucleus to generate a pre-miRNA containing an imperfect stem loop or “hairpin” structure of 50 ~ 80 nt, which is exported to the cytoplasm and subsequently processed by Dicer, a dsRNA-specific endonuclease to produce mature miRNA:miRNA* duplexes [17]. As one of the two arms of the miRNA duplexes, the mature miRNA are loaded onto an RNA-induced silencing complex (RISC) containing an Argonaute protein. By contrast, the non-incorporated miRNA* is subsequently degraded. The RISC then binds imperfectly to the target sites of mRNA, predominately in the untranslated region of the target mRNA for translational repression or mRNA cleavage [18-20]. The nucleotides 2–7 of mature miRNA, known as the “seed region”, play a crucial role in miRNA target recognition [21].

Nematodes, one of the most divergent phyla, have adapted to all environments. Over 23 000 species out of an estimated 100 000 to 10 million species have been described [22]. Since the first small RNA, *lin-4*, was identified in the nematode *Caenorhabditis elegans*[23], the combination of available reference genomes with experimental and computational approaches has boosted the discovery of abundant miRNA in this species [24]. Recently, the availability of high throughput sequencing technology has facilitated new discoveries of species-specific or lowly expressed miRNA possible [25]. It has greatly accelerated the establishment of small RNA repertoires of both free-living nematodes (e.g. *Caenorhabditis briggsae*, *C. remanei* and *Pristionchus pacificus*) [26], and of parasitic nematodes (e.g. *Ascaris suum* and the plant-parasitic nematode *Bursaphelenchus xylophilus*) [27,28]. One important group of parasitic nematodes is the superfamily of filaria, which belongs to the clade III nematodes. They include several pathogenic worms causing various tropical diseases both in humans and in animals of economical value [29]. Little was known about small RNA in filarial nematodes until the description of 32 miRNA in *B. malayi* was achieved by adopting RNA cloning and bioinformatics approaches [30]. Due to the ability of miRNA to respond to environmental and developmental signals [31], they are essential for the complex life cycle of filarial worms and are now considered as a key mechanism of gene regulation. However, these miRNA have been identified using traditional cloning and sequencing methods, which were not exhaustive, and additional filarial-specific or lowly expressed small RNA remain to be identified. Recently, the miRNA profiles of *B. pahangi* were detected by Illumina deep sequencing [32]. However, little information on small RNA of *D. immitis* is currently available.

Here, we describe the characterization of small RNA in adult *D. immitis* by combining Illumina/Solexa deep-sequencing technology with computational approaches. The small RNA profile of *D. immitis* will provide new insight into the control of these parasites and the pathways that could lead to the identification of new potential vaccine candidates. Moreover, due to the similar morphology, life cycle and modes of transmission among filaria, our results may become a useful resource for small RNA studies in other filarial parasitic nematodes. In addition, combining the study of small RNA in parasitic nematodes with those in free-living nematodes could help improve our understanding of the evolution and function of small RNA in this fascinating animal phylum.

## Materials and methods

### Source material

Live adult heartworms were collected by necropsy of an adult dog with sudden death, obtained from a veterinary hospital in Ya’an, Sichuan, China. Phosphate-buffered saline (PBS; pH 7.4; 37°C) was used five times to wash the live worms. The worms were immediately frozen in liquid nitrogen and stored at −80°C until further use.

The animal from which the specimens were collected was handled in accordance with animal protection laws of the People’s Republic of China (draft of an animal protection law in China released on September 18, 2009). The owner of the dead dog gave permission to use its tissue. This study was approved by the National Institute of Animal Health Animal Care and Use Committee at Sichuan Agricultural University, China (approval number 2010–020).

### Small RNA library preparation and high-throughput sequencing

Total RNA was extracted from four mixed-sex adult whole worms using Trizol (Invitrogen, Carlsbad, California, USA) according to the manufacturer’s instructions. The total RNA were then subjected to 15% denaturing polyacrylamide gel electrophoresis, and the 18–30 nt size range of RNA was isolated from the gel and purified. Next, proprietary (Solexa) adapters were sequentially ligated to the 5^′^- and 3^′^-termini of these small RNA. The gel-purified ligation products were converted to DNA and amplified by RT-PCR with 18 PCR cycles to produce libraries that were sequenced using a Solexa sequencer at Huada Genomics Institute Co. Ltd, Shenzhen, China.

### Computational methods to search conserved and novel miRNA

The adapter sequences were removed, low quality tags cleaned up and contamination formed by adapter-adapter ligation as well as reads containing poly A tails was filtered out. Afterwards, modified sequences (clean reads) from 18 nt to 30 nt were screened against the GenBank noncoding RNA database [33] and Rfam database (version 10.0) [34] to remove rRNA, tRNA, snRNA and snoRNA, as well as other ncRNA. Repeat overlapping sequences were annotated as repeat-associated small RNA using the tag2repeat software developed by BGI (Beijing Genome Institute, Beijing, China). After eliminating repetitive sequences, clean small RNA sequences were aligned with the miRNA precursor/mature miRNA of all animals in miRBase 17.0 [24] to identify conserved miRNA in *D. immitis*. Only perfectly or near-perfectly (1–2 mismatches) matching sequences were considered as conserved miRNA. All remaining clean sequences were considered as non-annotation reads. It was not possible to confirm that the miRNA identified by screening of the MirBase are all true miRNA in *D. immitis* without access to a genome.

To discover the potential miRNA precursors (with hairpin structure), all clean small RNA sequences were mapped to the *B*. *malayi* genome assembly (Brugia_assembly.ghedin.fasta), which was obtained from the Sanger Institute FTP site [35], using SOAP (Short Oligonucleotide Alignment Program) [36]. To obtain all candidate miRNA precursors with hairpin-like structures, the Mireap algorithm [37] was used. The parameters were set for animals to explore the secondary structure, the dicer cleavage site and the minimum free energy of the non-annotated small RNA tags that could be mapped to the genome. According to a previous study on plant miRNA [38], the minimal folding free energy index (MFEI) is a useful criterion for distinguishing miRNA from other types of coding or non-coding RNA, because 90% of miRNA precursors have an MFEI > 0.85, and no other RNA have an MFEI higher than 0.85 (MFEI = [MFEI/length of the RNA sequence × 100]/(G+C)%). The MFEI was calculated and potential novel heartworm miRNA candidates were further screened.

### PCR verification of miRNA precursors

To verify the predicted miRNA precursors based on the experimental approaches, we extracted genomic DNA from adult *D. immitis* using a Gentra Puregene Tissue Kit (Qiagen, Valencia, CA, USA) according to the manufacturer’s instruction. By using Oligo 6 software, we designed primers for the predicted 13 candidate miRNA precursors based on the *B. malayi* genome. In addition, the four novel miRNA/miRNA* duplex sequences were also used to design primers. The details of these primers are shown in Additional file 1. The PCR procedure was designed as previously described [28]. The length of amplification products was examined by 3.5% agarose gels. Fragments (70–100 nucleotides in length) were subcloned into a pMD18-T vector (Takara, Dalian, Liaoning, China) for sequencing analysis.

### Target gene prediction

To predict the targets of novel miRNA, we chose 20 810 unigene sequences from the transcriptome database of adult heartworms obtained by our lab. The unigenes have been uploaded into the Transcriptome Shotgun Assembly Sequence Database (TSA) at NCBI with the accession numbers: JR895929 - JR916738 [39]. RNAhybrid was employed as the programme for target prediction with the main parameters of –f (helix constraint) 2,8; –v (max bulge loop size) 3; –u (max internal loop size (per side)) 3 [40,41].

A gene ontology (GO) enrichment analysis [42] was used to reveal the functions significantly related to predicted target gene candidates of novel miRNA, as well as to recognize the main biological functions of the target gene candidates. The Kyoto encyclopedia of gene and genome (KEGG) pathway database [43] was also used for the target gene candidates to reveal the main pathways (including metabolic and signal transduction pathways) the target gene candidates are involved in [44].

## Results

### Deep sequencing of heartworm short RNA

**Figure 1:**
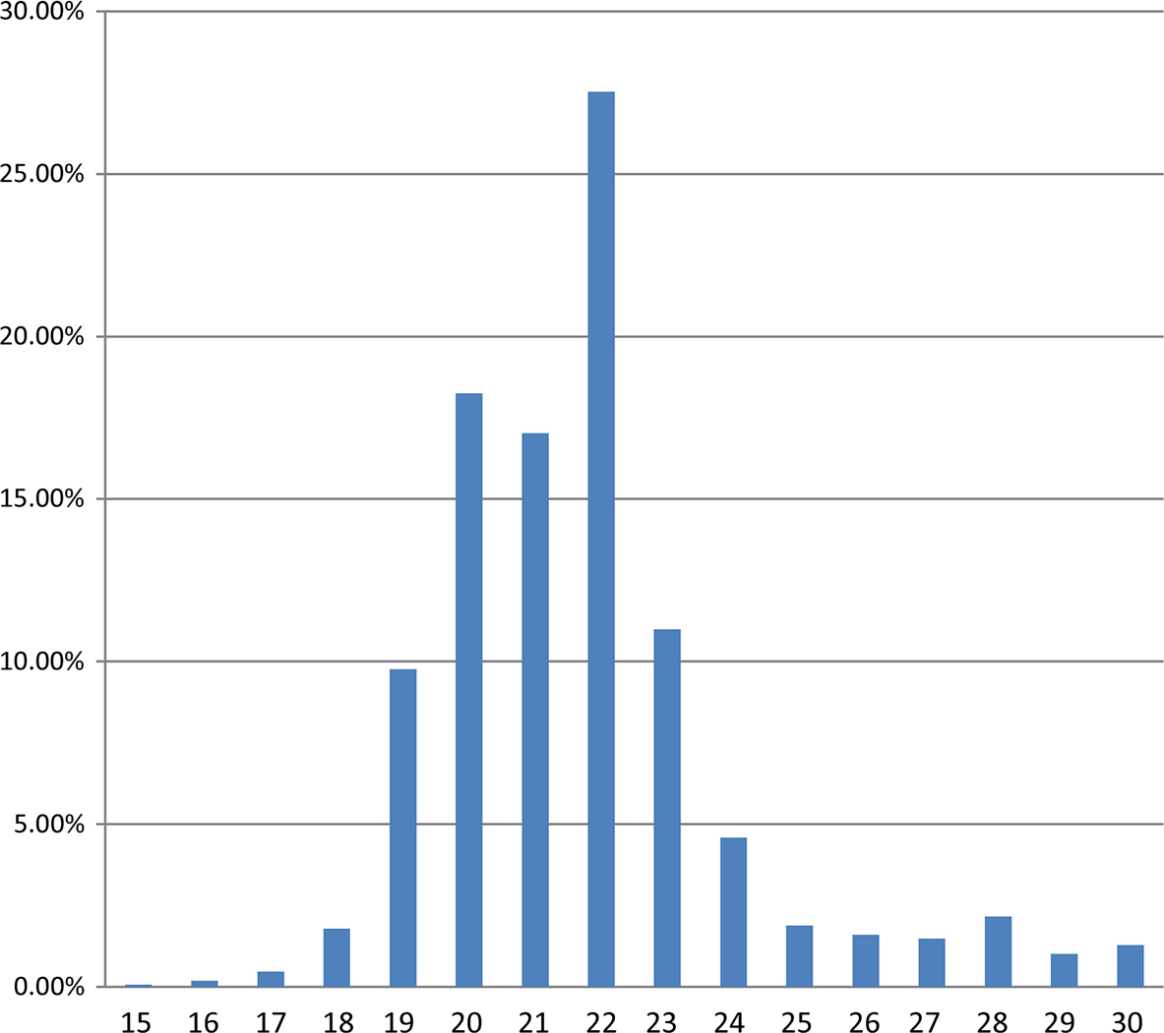
**Length distribution of sequenced small RNA.** The horizontal axis means the length (nt) of sequence, and the vertical means the frequence percentage (%).

**Figure 2:**
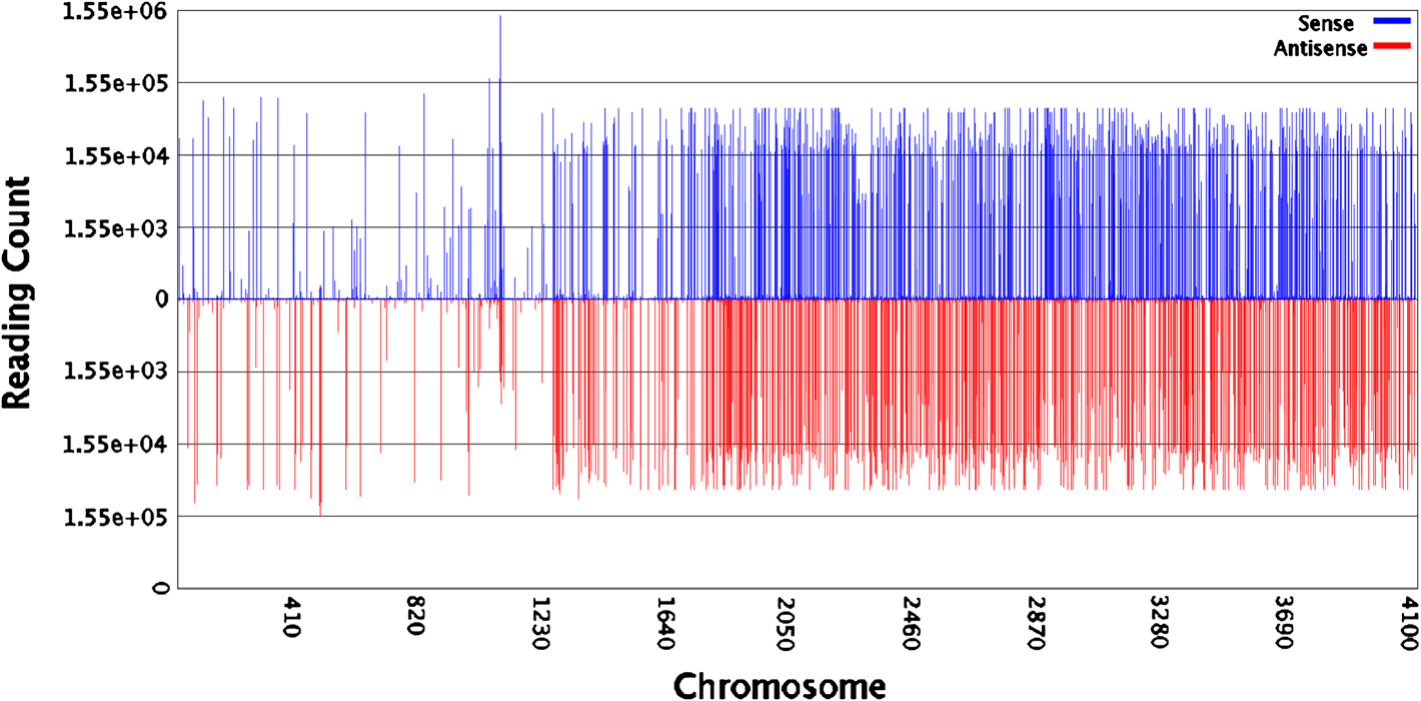
***D. immitis *small RNA distribution across different chromosomes of *Brugia malayi*.** “sense” and “anti-sense” stand for “+” and “–” strands of Chromosomes respectively.

**Figure 3:**
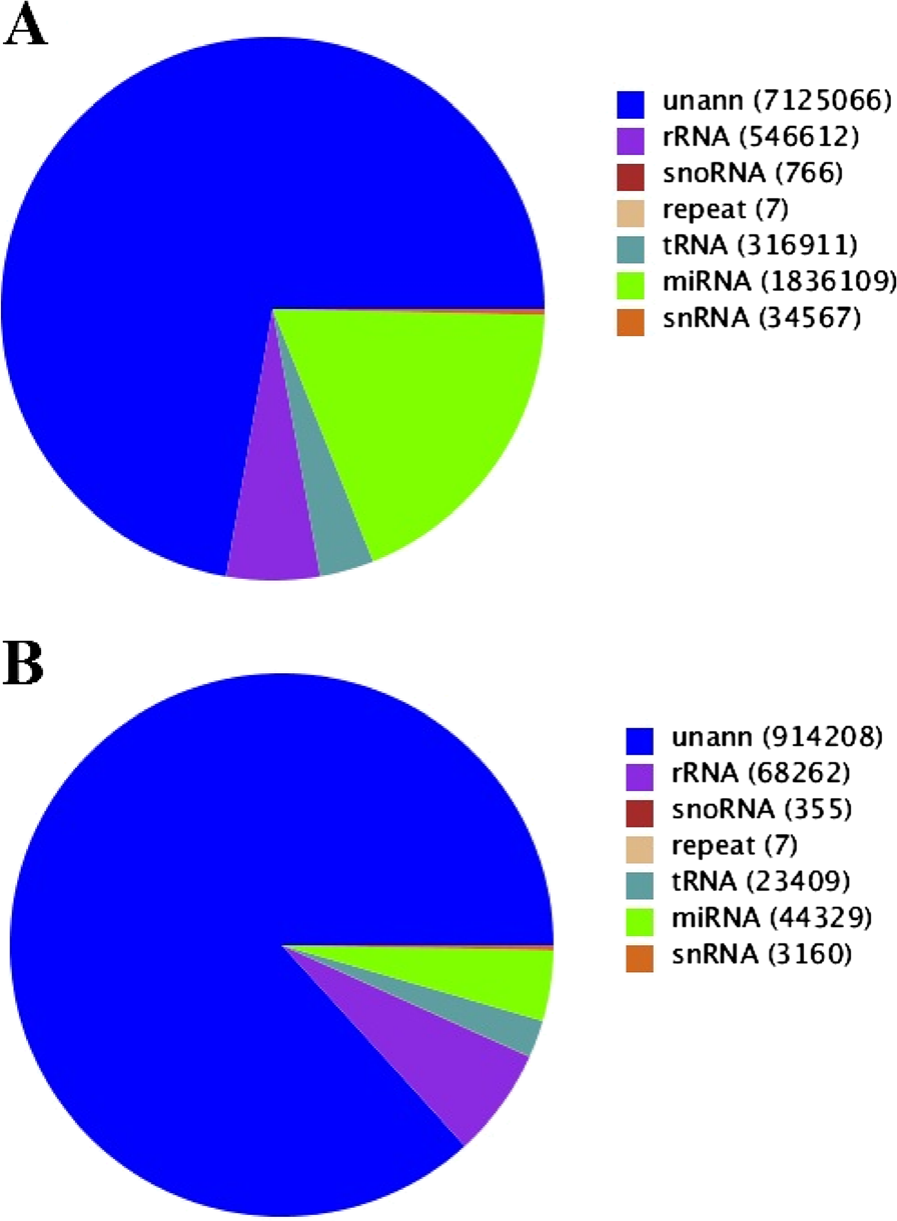
**Coverage of small RNA in *D. immitis *by Illumina sequencing. A**: total reads; **B**: unique reads. One unique read represents all the reads with the same sequence. “unann” means unannotated reads/unique reads. “repeat” means repeat-associated small RNA.

To identify miRNA active in heartworms, we used Illumina sequencing technology on the small RNA library from adult worms. We obtained 10 266 697 raw reads, which were deposited in the GEO of NCBI [45] under the accession number GSE35646. Low quality reads, reads with 5^′^ primer contaminants, reads without 3^′^ primers, reads without the inert tags and reads with Poly-A were discarded. The size distribution of all sRNA is summarized in Figure 1. The majority of the heartworm sRNA were in the range between 19 nt and 23 nt in length, with 20 nt and 22 nt as the two major size groups (Figure 1). After removing reads shorter than 18 nt, we obtained 9.86 million clean reads (18–30 nt in length) with 1.05 million unique sequences (clean reads). One unique sequence represents all the reads with the same sequence. Among them, 2.67 million (27.05%) reads were perfectly mapped to the *B*. *malayi* genome (Figure 2), including 34 494 (3.37%) unique sequences. This proportion (27.05%) was higher than that of *Clonorchis sinensis* small RNA reads mapped to the *Schistosoma japonicum* genome (19.46%), but lower than that of *B*. *pahangi* small RNA reads mapped to the *B. malayi* genome (62.3%) [32,46]. After further removal of tRNA, rRNA, snRNA, snoRNA and repeat-associated small RNA, a total of 8.96 million small RNA sequences were obtained. The distribution of reads/unique miRNA is shown in Figure 3. Among them, the percentage of known miRNA was 18.62% with 1 836 109 reads, which included 44 329 unique sequences. The miRNA contribution was lower than that of adult *B. pahangi*[32], which was a consequence of a higher percentage of non-annotated reads.

### Identification of conserved heartworm miRNA

To identify conserved miRNA in our dataset, all small RNA sequences were aligned to the miRNA precursor/mature miRNA of all animals in miRBase 17.0 [24]. Blastn searches showed that 1 836 109 total reads representing 44 329 unique sRNA matched to 1063 conserved miRNA that belonged to 808 miRNA families (Additional file 2). Sixty-eight unique miRNA* of these miRNA were also sequenced. The high throughput sequencing technology has been shown to determine the abundance of various miRNA families and even distinguish between different members of one family of one organism, which represents an alternative way to estimate the expression profiles of miRNA genes [47,48]. Interestingly, heartworm miRNA families varied significantly in their relative abundance. For instance, some types of miRNA were expressed with high predominance, with miR-1 accounting for 39.1% (718 580) of the total reads. It was followed by miR-71 with a percentage of 15.8% (289 744). The ten most abundant miRNA families are shown in Table 1. In comparison to *B. xylophilus*, miR-100, miR-1, miR-71 and let-7 were the most frequently sequenced miRNA in both species, while miR-279, miR-9, miR-101, miR-2 and miR-769 exhibited high abundance in *D. immitis* only. The miRNA miR-72, miR-34 and miR-252, which are highly expressed in *B. xylophilus*[28], were only detected 147, 1107 and 467 times in *D. immitis*, respectively. A comparison of the *D. immitis* miRNA presented here with the 32 miRNA identified previously in *B. malayi* suggested that all miRNA except miR-92 were identified in our analysis [30]. This suggests a species-specific expression profile for miRNA. A total of 703 types of *D. immitis* miRNA were sequenced fewer than 1000 times, including 200 types of miRNA with less than 10 copies. Diversity of *D. immitis* miRNA could also be found in the number of members they contained (Additional file 2). The largest miRNA family identified was let-7 consisting of 13 members, followed by miR-30, miR-2, miR-9, miR-92 and miR-548, which processed 10, 9, 8, 8 and 8 members, respectively. Other miRNA families, such as miR-228, miR-8 and miR-769 had only one member detected in this study. However, the number of members that a given miRNA family possessed had no relationship with the abundance of the miRNA family. For example, the miR-548 family possessed eight members with only 102 sequenced reads in total, whereas the miR-228 family had only one member with 241 479 sequenced reads. The size of miRNA families may be indicative of their function in this stage of the parasite. Different family members displayed drastically different expression levels. For instance, the abundance of the miR-100 family varied from 110 reads (miR-100b) to 68 557 reads (miR-100) in deep sequencing. The same observation was made in the case of other families, such as miR-1 (ranging from 17 to 283 242 reads) and let-7 (between 1 and 115 197 reads). This could suggest that the dominant member of a miRNA family performed a regulatory role in this family at the developmental stage when the samples were collected for RNA extraction.

**Table 1:** Sequences and abundance of top ten predicted conserved miRNA families of heartworms

**Name**	**Sequence**^**a**^	**Count**
miR-1	TGGAATGTAAAGAAGTATGT	718580
miR-71	TGAAAGACATGGGTAGTGAGACG	289744
miR-228	AATGGCACTAGATGAATTCACGG	241479
miR-100	TACCCGTAGCTCCGAATATGTGT	230351
let-7	TGAGGTAGTAGGTTGTATAGTT	229047
miR-279	TGACTAGAACCATACTCAGCT	114228
miR-9	TCTTTGGTTATCTAGCTGTATGA	106805
miR-101	TACATACTGGAGGAGCTGAA	99626
miR-2	TATCACAGGCCTGATGCAGCGAG	78387
miR-769	TGAGATTCTGGGTTTGAAC	77756

^a^ denotes the miRNA sequence with the highest count of the entire miRNA family.

**Figure 4:**
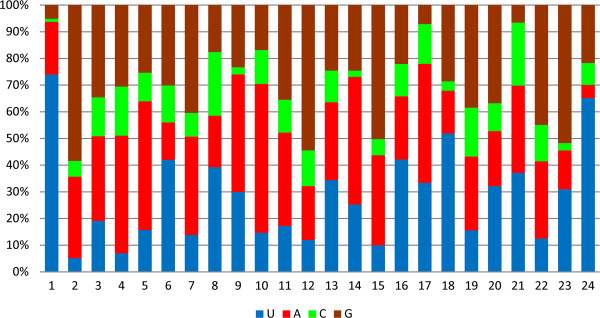
**Analysis of nucleotide bias percentage at each position in miRNA of *D. immitis*.** The horizontal axis means the nucleotide position in miRNA of *D. immitis*, and the vertical means the frequence percentage (%).

Nucleotide bias analysis at each position indicated that (A+U) dominated in the miRNA sequences and occupied a very high percentage at the start and the ends of reads, while a relatively high frequency of (G+C) appeared mostly at the middle of the reads (Figure 4 and Additional file 3). A first nucleotide bias analysis showed that uracil (U) was the most frequently used first nucleotide in miRNA of *D. immitis* with 74.02% incidence. The highest percentage of (A+U) was also located at the first nucleotide position with a percentage of 93.70%, followed by rates of 78.03%, 74.02%, 70.47% and 70.16% at the 17^th^, 9^th^, 10^th^ and the last position, respectively. The analysis showed that U also had a high frequency at the 18^th^ and 24^th^ positions with percentages of 51.92% and 65.25%, but the lowest rates of 5.13% and 6.98% at the 2^nd^ and 4^th^ positions, respectively. At the 12^th^ position, the (G+C) content reached its maximum at 67.85%, followed by 64.32% at the 2^nd^ position. G had high frequencies of 58.41%, 54.44%, 51.64% at the 2^nd^, 12^th^ and 23^rd^ positions respectively, while it rarely appeared at the first position (5.09%).

### Identification of novel heartworm miRNA

**Figure 5:**
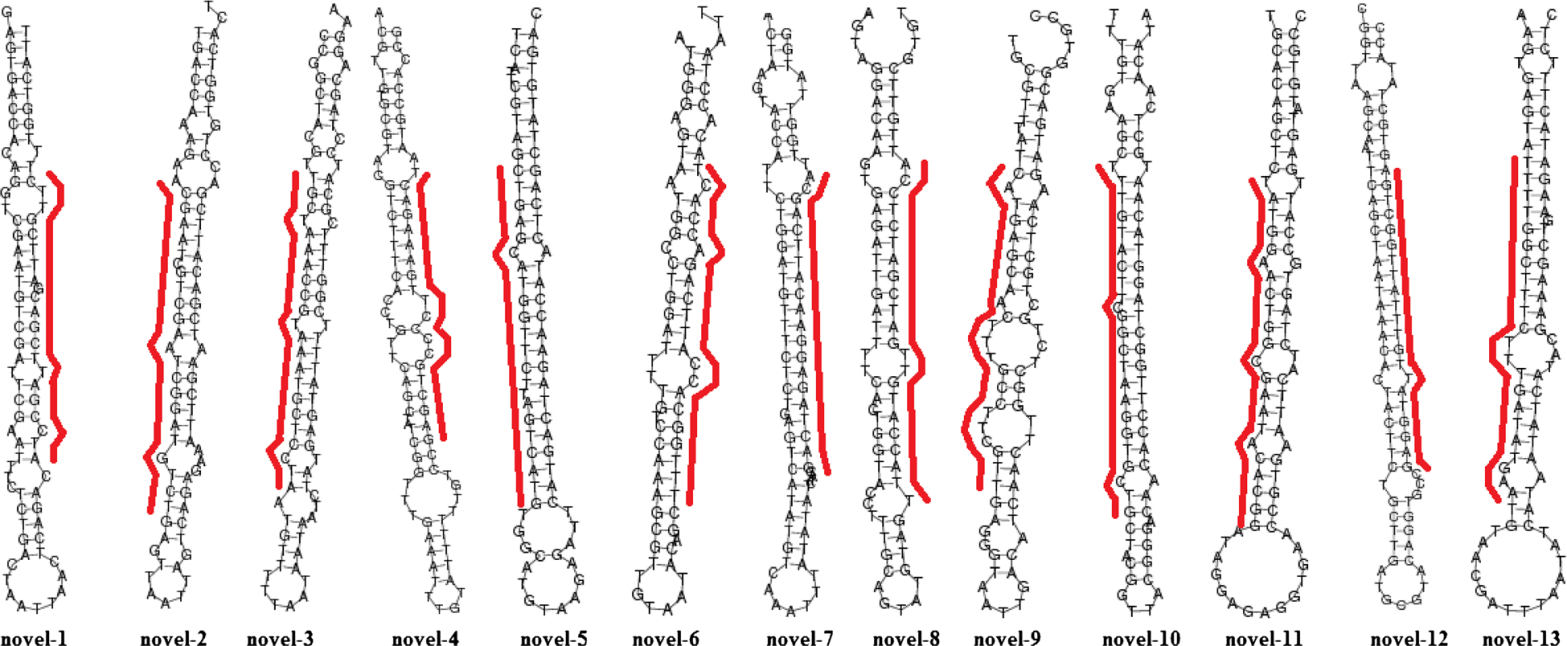
**Predicted hairpin secondary structures for the novel miRNA precursors of *D. immitis*.** Nucleotide bases of mature miRNA are highlighted in red. The actual size of each putative precursor might differ slightly from its shown length since it was not identified experimentally. RNA-fold software was employed to evaluate the stem-loop structure.

Apart from the conserved miRNA, we also discovered 13 novel miRNA by deep sequencing. Although the full genome sequence of *D. immitis* is currently not available, the genome of the closely related species *B*. *malayi* is an alternative source for precursor identification. A total of 1 479 929 non-annotated reads (out of 2.67 million reads) were marked as potential novel miRNA candidates. The secondary structure, the dicer cleavage site and the minimum free energy predicted by Mireap showed that 13 unique sequences were found. All of them have only one location on the *B. malayi* genome with the exception of dim-novel-013, which was found in two locations of the genome. The most abundant novel miRNA, dim-novel-12 was represented by 1.4 M reads (16.0% of the total). The high expression level of dim-novel-12 may indicate its significant role in the gene regulatory network. More importantly, the identification of a miRNA* from four novel miRNA candidates provided more support to consider them as novel miRNA. The expression levels of most miRNA* were much lower than their complementary sequences (sequencing frequency < 10), which is consistent with the hypothesis that a fast degradation mechanism of miRNA* exists in parasites [46]. Information about the sequences, expression levels, locations of the novel miRNA candidates and the corresponding miRNA* sequences are shown in Additional file 4. The typical secondary structures of characteristic stem-loop hairpins for the predicted precursors are shown in Figure 5. Previous studies on plant miRNA showed that MFEI was a sufficient criterion to distinguish miRNA from all coding and non-coding RNA (*P* < 0.001), suggesting that RNA sequences with MFEI larger than 0.85 are most likely to be miRNA [38]. Additional file 4 shows that almost all novel candidates had an MFEI > 0.85, except dim-novel-004 (0.65), dim-novel-009 (0.69) and dim-novel-010 (0.81).

### Experimental verification of miRNA precursors

**Figure 6:**
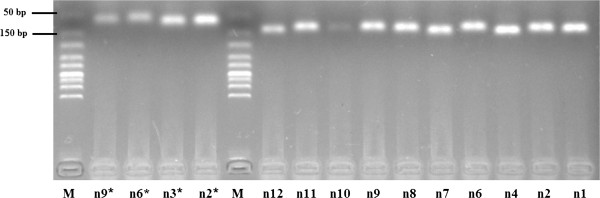
**Electrophoretic analysis of novel miRNA precursor PCR products.** “M” represents the marker (50–500 bp), “n” denotes the novel miRNA precursor candidate.

To verify the existence of the 13 novel miRNA (including the four novel miRNA/miRNA* pairs) in *D. immitis*, we amplified corresponding fragments from *D. immitis* genomic DNA for 13 of our predicted miRNA precursors (70–100 bp) as well as the four novel miRNA/miRNA* duplexes (50–70 bp). Products of expected length were successfully amplified from 10 out of the 13 candidates and all four duplexes (Figure 6), indicating that almost all of the predicted novel miRNA were canonical miRNA of *D. immitis* except dim-novel-005 and dim-novel-013. However, it is still possible that the failure to validate these miRNA precursors might be due to inappropriate primer design. Alignment analyses showed that the majority of pre-miRNA sequences of *D. immitis*, especially the mature miRNA sequences, contained high similarities with the corresponding predicted sequences from the *B. malayi* genome (identity > 93%), indicating the conservation of miRNA precursors in these two nematode species (Additional file 4). However, dim-novel-2 and dim-novel-4 had lower similarity with the predicted pre-miRNA from *B. malayi* (identity = 76.52% and 69.05%, respectively), suggesting they may have species-specific function roles in *D. immitis*.

### Prediction of novel miRNA target genes

Identification of direct miRNA targets is crucial for the characterization of miRNA functions [49]. To better understand the biological function of the newly identified miRNA in *D. immitis*, the putative target sites of the miRNA candidates were predicted using the described criteria and methods. However, bioinformatics predictions of target mRNA need to be further experimentally validated. Although in animals the miRNA target sites have been shown to be primarily located in the 3^′^ untranslated region (UTR) [50], the existence of miRNA-binding sites in protein coding regions has also been experimentally demonstrated [51,52]. According to these findings, we used 20 810 adult *D. immitis* unigenes, which were obtained by Illumina sequencing and Trinity assembling to predict miRNA targets. The thirteen novel miRNA candidates obtained 88 104 target-sites from our unigenes (Additional file 5). Most of the miRNA had multiple target-sites, while some unigenes could be targeted by more than one miRNA. This phenomenon indicates that multi-functional miRNA exist in heartworms. Similarly, the expression of some genes was also regulated by multiple miRNA.

According to the GO analysis on putative target genes (see Additional files 6, 7, 8), they appear to be involved in a wide variety of physiological processes, including molecular functions, cellular components and biological processes. The majority of the predicted targets fall into the categories of “binding”, “intracellular”, “intracellular part”, “catalytic activity”, “cellular process” and “metabolic process”. A KEGG pathway analysis was also used for the target gene candidates. In organisms, genes usually interact with each other to play different roles in certain biological functions. An analysis based on pathways could facilitate the understanding of biological functions of putative target genes. Additional file 9 shows that putative target unigenes participated in many important biological pathways. Our findings here might provide some clues for further investigation of the miRNA targets.

## Discussion

The complex life cycle of *D. immitis* with several developmental stages in its invertebrate mosquito vectors and its vertebrate hosts indicates the important roles of miRNA in the growth and development of heartworms and their ability to regulate infections of mammalian hosts. Identification of the whole expression map of miRNA in *D. immitis* is necessary for elucidating their roles, both in the normal functioning of eukaryotic cells and also in disturbances during disease.

Only 13 novel heartworm miRNA candidates were predicted in our study. The reason for this could be the low percentage of non-annotated reads matching the *B. malayi* genome (27.05%). Another reason might be that the reference genome used for matching analysis was the *B. malayi* genome rather than that of *D. immitis*, which is currently not published.

Unlike the miRNA dataset of *C. sinensis*[46], we not only found 68 homologs of known miRNA* of other organisms, but also obtained four miRNA* sequences of novel miRNA in the *D. immitis* dataset. Amongst these novel miRNA* sequences, most had no more than 10 copies, except dim-novel*-9 with 56 copies, while the copies of conserved miRNA* sequences ranged from 1 to 700 (miR-100-3p). There are also abundant miRNA* sequences in other nematodes, including *C. elegans*, *B. pahangi*, *H. contortus* and *B. xylophilus*[28,32,53]. It has been demonstrated that miRNA* function has, beyond the maintenance of precursor secondary structures, measurable effects on gene regulatory networks in living animals and during the course of species evolution [54,55]. Further investigation of these less stable sequences in *D. immitis* might reveal endogenous regulatory networks that miRNA* species are involved in [56]. Interestingly, we also found several miRNA* in our dataset with much higher expression levels than their mature partners, such as dim-miR-153* (immature/mature sequence read ratio: ~2/1), dim-miR-1422i* (~39/1), dim-miR-144* (~9/1), miR-200a* (~42/1), miR-9b* (~118/1). This phenomenon has also been observed in other nematode species, which supports the arm-switching hypothesis that a number of miRNA families have undergone transition from miRNA* to mature sequences or are still in the process of switching [26,57].

Nucleotide bias analysis showed that uracil was the most prominent nucleotide in *D. immitis* miRNA, particularly at the beginning and the end of the conserved miRNA, similar to miRNA of other parasites [28,46,58]. The second highest percentage of (G+C) was located at the beginning of seed region with 64.32%. However, the mean percentage of (G+C) at the positions 6–9 within the seed region was only 40.16%, lower than in other helminths [46,58,59]. It has been suggested that the positions 2–8 are the “seed region” of an miRNA, which is responsible for binding with the target genes for gene regulation [60]. The observation of relatively low frequency of (G+C) in the “seed regions” seems inconsistent with the mechanisms of miRNA action. One explanation for this might be possible species-specific differences, such as a different or shifted “seed region” for miRNA in *D. immitis* compared with previously analyzed vertebrate species [60].

The conserved miRNA in the adult stage of *D. immitis*, such as miR-1, miR-71, miR-228, miR-100, let-7, miR-279 and miR-9, had very high copy numbers, suggesting that they fulfill an essential function. In comparison with miRNA of other parasites, miR-71 was expressed abundantly among nematodes (*D. immitis*, *B. pahangi*, *H. contortus* and *B. xylophilus*), trematodes (*C. sinensis*) and cestodes (*T. saginata*), indicating that it might be essential for parasite survival [28,32,46,58]. Recent research suggested that miR-71 might positively influence the lifespan of worms, since it was shown to increase longevity in *C. elegans*[61]. Additionally, miR-100 and let-7 had very high copy numbers in nematode species but not in trematodes or cestodes [28,32,46,58,59]. They were sometimes absent in the latter two helminth groups, indicating that they might be especially involved in the regulation of metabolism in nematodes. Previous studies demonstrated that miRNA let-7 is a critical regulator of developmental timing events at the L4-to-adult transition [62,63], supported by the abundant expression of let-7 in most adult nematodes. The miRNA miR-48, miR-84 and miR-241, which were grouped into the family let-7 according to the identity of their seed sequences [64], were demonstrated to control developmental timing at the L2-to-L3 transition [62]. However, it was surprising to find that miR-84 had a very high number of copies (15 713) unlike miR-241 with a very low expression (87 copies) or miR-48, which was absent in our dataset. The discrepancy between its high expression level in adult heartworms and its recognized function involved in larva-to-larva transition indicates that miR-84 may have multiple roles in different stages of *D. immitis*. Given that most of the heartworm miRNA had multiple target-sites, while some unigenes could be targeted by more than one miRNA, we suggest that miRNA regulate various aspects of development and metabolism in diverse stages of *D. immitis* by functioning together with different, unrelated miRNA in regulatory networks.

To estimate the validity of the predicted miRNA candidates, we employed a PCR-based method to determine their existence in the *D. immitis* genome based on the secondary structure characters of their precursors. All PCR products showed a high degree of homology with the corresponding fragments in the *B. malayi* genome, indicating that these miRNA are conserved between the two species. A previous study on plant miRNA suggested that MFIE was a sufficient criterion to distinguish miRNA from all coding and non-coding RNA with a much higher MFEI (> 0.85) [38]. Surprisingly, our PCR results demonstrated the validity of dim-novel-004, dim-novel-009 and dim-novel-010, which had a MFEI < 0.85. This observation might indicate the difference between plant and animal miRNA precursors in their minimal negative folding free energy. At least it shows that MFEI should not be used as the only criterion to designate miRNA in animals.

Identification of potential targets may be of significance in elucidating the miRNA regulatory network. Due to a lack of detailed knowledge of the interaction mechanisms between miRNA and its target transcripts, it is difficult to predict miRNA targets in animals [65]. Most miRNA usually act upon their target genes by binding to sequences in the 3^′^ untranslated regions (UTR) [66]. However, little information about *D. immitis* 3^′^ UTR is available. Depending on a heartworm transcriptome dataset to predict the targets of its miRNA might find some canonical targets of miRNA, but would lead to higher false positive rates compared with a prediction using the 3^′^ UTR database [65]. Therefore, more experimental evidence is needed to validate the relationships between miRNA and the putative target transcripts.

In conclusion, our findings in this study illustrated for the first time the expression profiles of miRNA in the zoonotic nematode *D. immitis* by Illumina deep sequencing combined with bioinformatics analysis. This study led to the discovery of a large number of miRNA in the heartworm, especially 11 verified novel miRNA candidates, despite the lack of a completely published heartworm genome. This conserved and novel miRNA resource could be used as a new platform to study gene regulation in *D. immitis*. We also used a computational approach to predict the targeted genes of the novel miRNA with a wide range of functions in a variety of biological processes and metabolism pathways. Further experimental investigations are necessary to analyze the functional categories suggested by our computational approach to elucidate any significant correlation between miRNA and their targets.

## Competing interests

The authors declare that they have no competing interests.

## Authors’ contributions

YF, JCL and GYY conceived and designed the whole experiment. XHW, DYY, RHZ and WPZ performed the experiments of verification. YX, ZY, CDW and LLiu analyzed the data. LLuo, XBG, SXW and XRP contributed reagents/materials/analysis tools. YF, NY and HMN drafted the manuscript. ZHZ and RH revised the manuscript critically for important intellectual content. All authors read and approved the final manuscript.

## Supplementary Material

Additional file 1 **List of PCR primers for miRNA precursors and the PCR product sequence.** All 13 candidate miRNA precursors and four novel miRNA/miRNA* duplex sequences were used to design primers. Click here for file

Additional file 2 **List of *D. immitis *conserved miRNA based on miRBase 17.0.** Each “miRNA family” is not limited to one specific species. “Count” means the amount of miRNA of a family in our sample. “Sequence” represents a miRNA who has the highest count in the whole family. Click here for file

Additional file 3 **The nucleotide bias percentage at each position in miRNA of *D. immitis.*** The highest percentage at each column is highlighted. Click here for file

Additional file 4 **Details of the 13 novel miRNA precursors of *D. immitis.*** (A) miRNA precursor information (in order): sequence, name, length. (B) miRNA precursor information (in order): hairpin structure, structure, MFE. (C) Mature miRNA information (in order): sequence, name, length. (D) Star miRNA (if any) information (in order): sequence, name, length. Click here for file

Additional file 5 **List of predicted target unigenes of novel miRNA candidates.** 88 104 target-sites are predicted by mapping the thirteen novel miRNA to 20,810 adult *D. immitis* unigenes, which were obtained by Illumina sequencing and Trinity assembling. Click here for file

Additional file 6 **The GO annotations on putative target genes (Cellular component).** 7 722 target genes were assigned to 339 Go-terms which belong to “Cellular component” ontology. “Gene Ontology term” means GO terms with P-value as good or better than 1. “Cluster frequency” represents number and frequency of target genes related to this term. “Genome frequency of use” means number and frequency of coding genes related to this term. Click here for file

Additional file 7 **The GO annotations on putative target genes (Molecular function).** 10 541 target genes were assigned to 652 Go-terms which belong to “Molecular function” ontology. “Gene Ontology term” means GO terms with P-value as good or better than 1. “Cluster frequency” represents number and frequency of target genes related to this term. “Genome frequency of use” means number and frequency of coding genes related to this term. Click here for file

Additional file 8 **The GO annotations on putative target genes (Biological process).** 9 357 target genes were assigned to 2 235 Go-terms which belong to “Biological process” ontology. “Gene Ontology term” means GO terms with P-value as good or better than 1. “Cluster frequency” represents number and frequency of target genes related to this term. “Genome frequency of use” represents number and frequency of coding genes related to this term. Click here for file

Additional file 9 **KEGG pathway of predicted target genes.** 9 661 target genes were assigned to 250 KEGG pathways. “Target genes with pathway annotation” represents number and frequency of target genes related to this pathway. “All genes of the species with pathway annotation” represents number and frequency of reference genes related to this pathway. “Pvalue” and “Qvalue” represent *P*-value before correction and corrected *P*-value, respectively. Click here for file
