# Supplementary material for: Identification of Dirofilaria immitis miRNA using illumina deep sequencing
Source: Vet Res. 2013 Jan 18;44(1):3. doi: 10.1186/1297-9716-44-3 (PMC3598945; doi:10.1186/1297-9716-44-3)
Supplement: Additional file 3 — The nucleotide bias percentage at each position in miRNA of D. immitis. The highest percentage at each column is highlighted. [file 1297-9716-44-3-S3.pdf]

Additional file 3. The nucleotide bias percentage at each position in miRNAs of *D. immitis*.

Position	A(%)	U（%）	C（%）	G(%)	A+U(%)	G+C(%)
1	19.68	74.02	1.21	5.09	93.70	6.30
2	30.55	5.13	5.92	58.41	35.68	64.32
3	31.75	19.09	14.60	34.56	50.84	49.16
4	44.07	6.98	18.39	30.55	51.06	48.94
5	48.27	15.67	10.73	25.33	63.94	36.06
6	13.99	42.06	13.82	30.14	56.04	43.96
7	36.89	13.84	8.83	40.44	50.73	49.27
8	19.32	39.23	23.87	17.59	58.55	41.45
9	44.00	30.02	2.67	23.31	74.02	25.98
10	55.83	14.64	12.68	16.84	70.47	29.53
11	34.95	17.27	12.35	35.43	52.22	47.78
12	20.19	11.96	13.41	54.44	32.15	67.85
13	29.26	34.37	11.83	24.54	63.63	36.37
14	47.81	25.29	2.37	24.53	73.10	26.90
15	33.79	9.93	6.13	50.15	43.72	56.28
16	23.73	42.10	12.16	22.00	65.84	34.16
17	44.61	33.42	14.94	7.03	78.03	21.97
18	16.00	51.92	3.50	28.57	67.93	32.07
19	27.66	15.59	18.30	38.44	43.25	56.75
20	20.60	32.16	10.50	36.74	52.76	47.24
21	32.70	37.15	23.65	6.50	69.85	30.15
22	28.92	12.50	13.67	44.91	41.41	58.59
23	14.57	30.93	2.85	51.64	45.51	54.49
24	4.91	65.25	8.21	21.64	70.16	29.84
