# Supplementary material for: Identification of Dirofilaria immitis miRNA using illumina deep sequencing
Source: Vet Res. 2013 Jan 18;44(1):3. doi: 10.1186/1297-9716-44-3 (PMC3598945; doi:10.1186/1297-9716-44-3)
Supplement: Additional file 4 — Details of the 13 novel miRNA precursors of D. immitis. (A) miRNA precursor information (in order): sequence, name, length. (B) miRNA precursor information (in order): hairpin structure, structure, MFE. (C) Mature miRNA information (in order): sequence, name, length. (D) Star miRNA (if any) information (in order): sequence, name, length. [file 1297-9716-44-3-S4.pdf]

mireap

Dirofilaria_immites-novel-1 gi|170571255|ref|NW_001890621.1|:56293:56377:+ 85(nt) -31.70(kcal/mol) MFEI=0.88

(A) GAGTGACCACAGGTCGAATGTCGATTCGAATTTCTCTGACTAATTAACTCAGACATCCGATTCGACGATTCGTTCTTTGGTCATT Dirofilaria_immites-novel-1 8

(B) .((((((((.((..((((((((((.(((.((...(((((.........))))).)).))).))))).)))))..)).))))))))

(C) **ACATCCGATTCGACGATTCGTTC********** Dirofilaria_immites-novel-1-3p 8

--ACATCCGATTCGACGATTCGTT----------- t0230722 2

--ACATCCGATTCGACGATTCGTTC---------- t0095495 6

//

mireap

Dirofilaria_immites-novel-2 gi|170571255|ref|NW_001890621.1|:56294:56375:- 82(nt) -34.40(kcal/mol) MFEI=0.98

(A) TGACCAAAGAACGAATCGTCGAATCGGATGTCTGAGTTAATTAGTCAGAGAAATTCGAATCGACATTCGACCTGTGGTCACT Dirofilaria_immites-novel-2 24312

(B) ((((((.((..(((((.(((((.((((((.(((((.(.....).)))))...)))))).))))))))))..)).))))))..

(C) **********ACGAATCGTCGAATCGGATGTCT*** Dirofilaria_immites-novel-2-5p 24311

(D) **AAATTCGAATCGACATTCGACC********** Dirofilaria_immites-novel-2-3p 1

----------ACGAATCGTCGAATCGGA-- t0106330 5

----------ACGAATCGTCGAATCGGAT--- t0048850 13

----------ACGAATCGTCGAATCGGATG-- t0025821 27

----------ACGAATCGTCGAATCGGATGT--- t0011534 66

----------ACGAATCGTCGAATCGGATGTC-- t0001428 542

----------ACGAATCGTCGAATCGGATGTCT--- t0000022 23500

----------ACGAATCGTCGAATCGGATGTCTG-- t0011113 69

----------ACGAATCGTCGAATCGGATGTCTGA--- t0114964 5

-----------CGAATCGTCGAATCGGATGTC-- t0229608 2

-----------CGAATCGTCGAATCGGATGTCT--- t0013877 54

------------GAATCGTCGAATCGGATGTCT--- t0061564 10

-------------AATCGTCGAATCGGATGT--- t0592465 1

-------------AATCGTCGAATCGGATGTCT--- t0044407 14

--------------ATCGTCGAATCGGATGTCT--- t0161220 3

--AAATTCGAATCGACATTCGACC---------- t1044402 1
//
mireap
Dirofilaria_immites-novel-3 gi|170571286|ref|NW_001890630.1|:744:827:- 84(nt) -27.40(kcal/mol) MFEI=0.86
(A) CCGGCTACGTTGCTAAACCGTAAATGCTCCTAATGTTTTTAATAATAATCTATGAGTATTTTCGGTTTCGCATCCTAGCAGGAA Dirofilaria_immites-novel-3 43
(B) ((.((((.(.(((.((((((.((((((((.((.((((......))))...)).))))))))).)))))).))).).)))).))..
(C) **********TGCTAAACCGTAAATGCTCCTA** Dirofilaria_immites-novel-3-5p 42
(D) **TGAGTATTTTCGGTTTCGCATC********** Dirofilaria_immites-novel-3-3p 1
----------TGCTAAACCGTAAATGCTCCT--- t0064577 9
----------TGCTAAACCGTAAATGCTCCTA-- t0021897 33
--TGAGTATTTTCGGTTTCGCATC---------- t0786590 1
//
mireap
Dirofilaria_immites-novel-4 gi|170573109|ref|NW_001891087.1|:2287:2376:+ 90(nt) -26.70(kcal/mol) MFEI=0.65
(A) ACGTTGTGCGTACGTCTTTCACCTGTTCAGCTATCGGTTTGAAATTTGTATTTTTTGTCCGAGCTGCCCCTTGAAAGACTAATGCCACCG Dirofilaria_immites-novel-4 18
(B) .((.((.((((..((((((((...(..((((..((((...(((((....)))))....))))))))..)..))))))))..)))))).))
(C) **GAGCTGCCCCTTGAAAGACT********** Dirofilaria_immites-novel-4-3p 18
--GAGCTGCCCCTTGAAAGACT---------- t0060352 10
--GAGCTGCCCCTTGAAAGACTA--------- t0118190 4
--GAGCTGCCCCTTGAAAGACTAA-------- t0171985 3
--GAGCTGCCCCTTGAAAGACTAAT------- t0881577 1
//
mireap
Dirofilaria_immites-novel-5 gi|170575627|ref|NW_001891541.1|:19512:19589:+ 78(nt) -37.70(kcal/mol) MFEI=1.14
(A) TCATTCGTAGCTGAGCATGGTTCTTAGTCATGTGGCATGTAAGAGATTCATGACTAGAACCATACTCAGCTATGTGAC Dirofilaria_immites-novel-5 86
(B) (((..((((((((((.((((((((.(((((((...(........)...))))))))))))))).))))))))))))).
(C) **********CTGAGCATGGTTCTTAGTCATG** Dirofilaria_immites-novel-5-5p 86
----------CTGAGCATGGTTCTTAGTCAT--- t1010127 1

```
    ----------CTGAGCATGGTTCTTAGTCATG---------------------------------------------- t0009186 84
    -----------TGAGCATGGTTCTTAGTCATG---------------------------------------------- t0359615 1
//
mireap
    Dirofilaria_immites-novel-6 gi|170575627|ref|NW_001891541.1|:21719:21794:+ 76(nt) -29.60(kcal/mol) MFEI=0.9
(A) ATGGGAGTAATGGCCTGGATTTTGTCCAAAGCGTTGTAATACAGCTTTGGCACCATTCAGACCACTACACCTAATT Dirofilaria_immites-novel-6 823
(B) .((((.(((.(((.((((((..((.(((((((((......)).)))))))))..)))))).))).))).))))...
(C) *********************************************TTTGGCACCATTCAGACCACT********** Dirofilaria_immites-novel-6-3p 814
(D) **********TGGCCTGGATTTTGTCCAAAGC******************************************** Dirofilaria_immites-novel-6-5p 9
    ----------TGGCCTGGATTTTGTCCAAAGC-------------------------------------------- t0063401 9
    ---------------------------------------------TTTGGCACCATTCAGACC------------- t0920597 1
    ---------------------------------------------TTTGGCACCATTCAGACCA------------ t0191576 2
    ---------------------------------------------TTTGGCACCATTCAGACCAC----------- t0145036 3
    ---------------------------------------------TTTGGCACCATTCAGACCACT---------- t0001733 446
    ---------------------------------------------TTTGGCACCATTCAGACCACTA--------- t0002241 349
    ----------------------------------------------TTGGCACCATTCAGACCACT---------- t0124724 4
    ----------------------------------------------TTGGCACCATTCAGACCACTA--------- t0064940 9
//
mireap
    Dirofilaria_immites-novel-7 gi|170575775|ref|NW_001891561.1|:10393:10477:+ 85(nt) -28.70(kcal/mol) MFEI=0.96
(A) ACTAAGTACCATTCTGGATGTTTCTCTGAGTCATATGTCAAATTTATATATCTACGACTAGAGGAACATTCAGCATTGGTTATGG Dirofilaria_immites-novel-7 22
(B) .(((...((((..((((((((((((((.((((((((((.......))))))....))))))))))))))))))...))))..)))
(C) ******************************************************CGACTAGAGGAACATTCAGCA********** Dirofilaria_immites-novel-7-3p 22
    ------------------------------------------------------CGACTAGAGGAACATTCAGC----------- t0054122 11
    ------------------------------------------------------CGACTAGAGGAACATTCAGCA---------- t0053684 11
//
mireap
    Dirofilaria_immites-novel-8 gi|170585917|ref|NW_001893004.1|:593289:593368:+ 80(nt) -28.50(kcal/mol) MFEI=0.89
```

(A) AGTAGGACAAGTGAGATTGATTTTCACTGGTACCTTTGCAGTATGTAGTTACCATGTTGATCGATCTCCATTGTTCGTGT Dirofilaria_immites-novel-8 2754
(B) ....((((((..((((((((((..((.(((((...((((.....)))).)))))))..))))))))))..))))))....
(C) **TTACCATGTTGATCGATCTCCA********** Dirofilaria_immites-novel-8-3p 2754
--TTACCATGTTGATCGATC-------------- t0143697 3
--TTACCATGTTGATCGATCT------------- t0176884 3
--TTACCATGTTGATCGATCTC------------ t0016255 45
--TTACCATGTTGATCGATCTCC----------- t0001990 389
--TTACCATGTTGATCGATCTCCA---------- t0000287 2299
--TTACCATGTTGATCGATCTCCAT--------- t0060833 10
---TACCATGTTGATCGATCTCCA---------- t0180484 3
---CCATGTTGATCGATCTCCA---------- t0728218 1
--CATGTTGATCGATCTCCA---------- t0675113 1
//
mireap
Dirofilaria_immites-novel-9 gi|170589762|ref|NW_001893021.1|:5975911:5975989:+ 79(nt) -26.10(kcal/mol) MFEI=0.69
(A) TGCGTTTATCATGAGCAACTTTGCCTTCGTTGAGGGTAATTGACATCAACTTGGCTCTGCTGCTCAAGATGACGGTGCC Dirofilaria_immites-novel-9 4277
(B) ..((((.(((.((((((.(...(((...(((((..((.....)).)))))..)))...).)))))).))))))).....
(C) **********ATGAGCAACTTTGCCTTCGTTGA** Dirofilaria_immites-novel-9-5p 4221
(D) ***AACTTGGCTCTGCTGCTCAAGA********** Dirofilaria_immites-novel-9-3p 56
---------CATGAGCAACTTTGCCTTCGTT-- t0804900 1
---------CATGAGCAACTTTGCCTTCGTTGA-- t0112797 5
----------ATGAGCAACTTTGCCTTC--- t0725680 1
----------ATGAGCAACTTTGCCTTCGT--- t0027090 26
----------ATGAGCAACTTTGCCTTCGTT-- t0018134 40
----------ATGAGCAACTTTGCCTTCGTTG--- t0000767 965
----------ATGAGCAACTTTGCCTTCGTTGA-- t0000199 3122
----------ATGAGCAACTTTGCCTTCGTTGAG--- t0040616 16
----------ATGAGCAACTTTGCCTTCGTTGAGG-- t0173325 3

```
     -----------TGAGCAACTTTGCCTTCGTT------------------------------------------------ t0819747 1
     -----------TGAGCAACTTTGCCTTCGTTG----------------------------------------------- t0082180 7
     -----------TGAGCAACTTTGCCTTCGTTGA---------------------------------------------- t0024374 29
     -------------AGCAACTTTGCCTTCGTTG----------------------------------------------- t0696321 1
     -------------AGCAACTTTGCCTTCGTTGA---------------------------------------------- t0489639 1
     --------------GCAACTTTGCCTTCGTTGA---------------------------------------------- t0144719 3
     -----------------------------------------------AACTTGGCTCTGCTGCTCA------------- t0991968 1
     -----------------------------------------------AACTTGGCTCTGCTGCTCAAG----------- t0078829 7
     -----------------------------------------------AACTTGGCTCTGCTGCTCAAGA---------- t0020870 34
     ------------------------------------------------ACTTGGCTCTGCTGCTCAAGA---------- t0047055 13
     ------------------------------------------------ACTTGGCTCTGCTGCTCAAGAT--------- t0826064 1
//
mireap
     Dirofilaria_immites-novel-10 gi|170589762|ref|NW_001893021.1|:6353991:6354069:+ 79(nt) -35.10(kcal/mol) MFEI=0.95
(A)  TTTGTGAAGCTTTGTACTTCGGCTAAGGTGCTGCTACGGTTACGGGACAACACCTTGGCTAGGTACAATGCTCAACATA Dirofilaria_immites-novel-10 15896
(B)  ..(((..(((.((((((((.((((((((((.((((.((....)))).)).))))))))))))))))))).)))..)))..
(C)  **********TTTGTACTTCGGCTAAGGTGCTG********************************************** Dirofilaria_immites-novel-10-5p 15896
     ---------CTTTGTACTTCGGCTAAGGTGCTG---------------------------------------------- t0007086 111
     ----------TTTGTACTTCGGCTAAGGTGCTG---------------------------------------------- t0000034 15702
     ----------TTTGTACTTCGGCTAAGGTGCTGC--------------------------------------------- t0024354 29
     -----------TTGTACTTCGGCTAAGGTGCTG---------------------------------------------- t0017148 43
     ------------TGTACTTCGGCTAAGGTGCTG---------------------------------------------- t0053772 11
//
mireap
     Dirofilaria_immites-novel-11 gi|170589762|ref|NW_001893021.1|:62365:62444:- 80(nt) -31.44(kcal/mol) MFEI=0.81
(A)  TGCACAGCTCTATGGAACTGGCGAATACACGGATAGGAGAGGTGAACCGTGAATTCATCTAGTGCCATTGAGTATGTGCC Dirofilaria_immites-novel-11 8
(B)  .(((((((((.((((.(((((.((((.(((((..............))))).))))..))))).)))).)))).))))).
(C)  **********TATGGAACTGGCGAATACACGG************************************************ Dirofilaria_immites-novel-11-5p 8
```

```
    ----------TATGGAACTGGCGAATACACGG------------------------------------------------ t0068659 8
//
mireap
    Dirofilaria_immites-novel-12 gi|170590627|ref|NW_001893024.1|:500433:500517:+ 85(nt) -37.20(kcal/mol) MFEI=1.01
(A) CGGTTAAGCAATTCAGCTAATAAACACTACTTCTGCTTGATGCGTACAGGTGCCGAGGTATTGTTTATTGGCTGAGTGCTATACC Dirofilaria_immites-novel-12 1431640
(B) .(((..((((.(((((((((((((((.((((((.(((((.......)))))...)))))).)))))))))))))))))))..)))
(C) *****************************************************CGAGGTATTGTTTATTGGCTGA********** Dirofilaria_immites-novel-12-3p
1431640
    ----------------------------------------------------CCGAGGTATTGTTTATTGGC------------- t0299575 1
    ----------------------------------------------------CCGAGGTATTGTTTATTGGCT------------ t0563825 1
    ----------------------------------------------------CCGAGGTATTGTTTATTGGCTG----------- t0147594 3
    ----------------------------------------------------CCGAGGTATTGTTTATTGGCTGA---------- t0007370 106
    -----------------------------------------------------CGAGGTATTGTTTATTGG-------------- t0005303 148
    -----------------------------------------------------CGAGGTATTGTTTATTGGC------------- t0000183 3295
    -----------------------------------------------------CGAGGTATTGTTTATTGGCT------------ t0000076 7116
    -----------------------------------------------------CGAGGTATTGTTTATTGGCTG----------- t0000020 25340
    -----------------------------------------------------CGAGGTATTGTTTATTGGCTGA---------- t0000001 1386004
    -----------------------------------------------------CGAGGTATTGTTTATTGGCTGAG--------- t0000108 5033
    -----------------------------------------------------CGAGGTATTGTTTATTGGCTGAGT-------- t0081392 7
    ------------------------------------------------------GAGGTATTGTTTATTGGC------------- t0075426 8
    ------------------------------------------------------GAGGTATTGTTTATTGGCT------------ t0052999 12
    ------------------------------------------------------GAGGTATTGTTTATTGGCTG----------- t0022648 31
    ------------------------------------------------------GAGGTATTGTTTATTGGCTGA---------- t0000248 2637
    ------------------------------------------------------GAGGTATTGTTTATTGGCTGAG--------- t0047501 13
    -------------------------------------------------------AGGTATTGTTTATTGGCT------------ t0083213 7
    -------------------------------------------------------AGGTATTGTTTATTGGCTG----------- t0022285 32
    -------------------------------------------------------AGGTATTGTTTATTGGCTGA---------- t0000465 1524
    -------------------------------------------------------AGGTATTGTTTATTGGCTGAG--------- t0262231 2
```

```
-------------------------------------------------------AGGTATTGTTTATTGGCTGAGT-------- t0586900 1
--------------------------------------------------------GGTATTGTTTATTGGCTG----------- t0098810 5
--------------------------------------------------------GGTATTGTTTATTGGCTGA---------- t0004740 166
--------------------------------------------------------GGTATTGTTTATTGGCTGAG--------- t0165444 3
---------------------------------------------------------GTATTGTTTATTGGCTGA---------- t0005462 144
---------------------------------------------------------GTATTGTTTATTGGCTGAG--------- t0395726 1
//
mireap
    Dirofilaria_immites-novel-13(1) gi|170593506|ref|NW_001893039.1|:535278:535358:+ 81(nt) -21.74(kcal/mol) MFEI=0.95
(A) AAGTGAGTATTTTGGCTTTCTTTGATATGAATGTAACGATTTAATATCATAATATCATACGAAAGCTGAAGATACTTTCTC Dirofilaria_immites-novel-13 21
(B) .((.((((((((((((((((..((((((..(((..............))).))))))...))))))).))))))))).)).
(C) **********TTTGGCTTTCTTTGATATGAATG************************************************ Dirofilaria_immites-novel-13-5p 21
    ----------TTTGGCTTTCTTTGATATGAA-------------------------------------------------- t0227204 2
    ----------TTTGGCTTTCTTTGATATGAAT------------------------------------------------- t0122991 4
    ----------TTTGGCTTTCTTTGATATGAATG------------------------------------------------ t0042627 15
//
mireap
    Dirofilaria_immites-novel-13(2) gi|170648757|ref|NW_001914025.1|:283:363:- 81(nt) -21.74(kcal/mol) MFEI=0.95
(A) AAGTGAGTATTTTGGCTTTCTTTGATATGAATGTAACGATTTAATATCATAATATCATACGAAAGCTGAAGATACTTTCTC Dirofilaria_immites-novel-13 21
(B) .((.((((((((((((((((..((((((..(((..............))).))))))...))))))).))))))))).)).
(C) **********TTTGGCTTTCTTTGATATGAATG************************************************ Dirofilaria_immites-novel-13-5p 21
    ----------TTTGGCTTTCTTTGATATGAA-------------------------------------------------- t0227204 2
    ----------TTTGGCTTTCTTTGATATGAAT------------------------------------------------- t0122991 4
    ----------TTTGGCTTTCTTTGATATGAATG------------------------------------------------ t0042627 15
//
```
