# Supplementary material for: Identification of Dirofilaria immitis miRNA using illumina deep sequencing
Source: Vet Res. 2013 Jan 18;44(1):3. doi: 10.1186/1297-9716-44-3 (PMC3598945; doi:10.1186/1297-9716-44-3)
Supplement: Additional file 6 — The GO annotations on putative target genes (Cellular component). 7 722 target genes were assigned to 339 Go-terms which belong to “Cellular component” ontology. “Gene Ontology term” means GO terms with P-value as good or better than 1. “Cluster frequency” represents number and frequency of target genes related to this term. “Genome frequency of use” means number and frequency of coding genes related to this term. [file 1297-9716-44-3-S6.zip › index.htm/Additional file 6. Cellular component.html]

Terms for Dirofilaria\_immites\_C


## Terms for Dirofilaria\_immites\_C

---


### Result Table

|  |
| --- |
| **Terms from the Component Ontology with p-value as good or better than 1** |

| Gene Ontology term | Cluster frequency | Genome frequency of use | Corrected P-value |
| --- | --- | --- | --- |
| chromosome | 246 out of 7722 genes, 3.2% | 270 out of 9057 genes, 3.0% | 0.83427 |
| membrane-bounded organelle | 3308 out of 7722 genes, 42.8% | 3829 out of 9057 genes, 42.3% | 1 |
| nucleus | 754 out of 7722 genes, 9.8% | 855 out of 9057 genes, 9.4% | 1 |
| nuclear chromosome | 44 out of 7722 genes, 0.6% | 45 out of 9057 genes, 0.5% | 1 |
| clathrin-coated vesicle | 83 out of 7722 genes, 1.1% | 88 out of 9057 genes, 1.0% | 1 |
| intracellular membrane-bounded organelle | 3219 out of 7722 genes, 41.7% | 3728 out of 9057 genes, 41.2% | 1 |
| nuclear ubiquitin ligase complex | 27 out of 7722 genes, 0.3% | 27 out of 9057 genes, 0.3% | 1 |
| chromosomal part | 198 out of 7722 genes, 2.6% | 219 out of 9057 genes, 2.4% | 1 |
| ubiquitin ligase complex | 72 out of 7722 genes, 0.9% | 77 out of 9057 genes, 0.9% | 1 |
| intracellular | 4995 out of 7722 genes, 64.7% | 5821 out of 9057 genes, 64.3% | 1 |
| intracellular part | 4931 out of 7722 genes, 63.9% | 5746 out of 9057 genes, 63.4% | 1 |
| organelle | 3977 out of 7722 genes, 51.5% | 4626 out of 9057 genes, 51.1% | 1 |
| intracellular organelle | 3909 out of 7722 genes, 50.6% | 4547 out of 9057 genes, 50.2% | 1 |
| nuclear chromosome part | 20 out of 7722 genes, 0.3% | 20 out of 9057 genes, 0.2% | 1 |
| coated vesicle | 118 out of 7722 genes, 1.5% | 130 out of 9057 genes, 1.4% | 1 |
| insoluble fraction | 146 out of 7722 genes, 1.9% | 162 out of 9057 genes, 1.8% | 1 |
| nuclear part | 659 out of 7722 genes, 8.5% | 755 out of 9057 genes, 8.3% | 1 |
| condensed nuclear chromosome | 18 out of 7722 genes, 0.2% | 18 out of 9057 genes, 0.2% | 1 |
| spliceosomal complex | 67 out of 7722 genes, 0.9% | 73 out of 9057 genes, 0.8% | 1 |
| clathrin coated vesicle membrane | 26 out of 7722 genes, 0.3% | 27 out of 9057 genes, 0.3% | 1 |
| chromatin remodeling complex | 34 out of 7722 genes, 0.4% | 36 out of 9057 genes, 0.4% | 1 |
| lysosome | 15 out of 7722 genes, 0.2% | 15 out of 9057 genes, 0.2% | 1 |
| centrosome | 15 out of 7722 genes, 0.2% | 15 out of 9057 genes, 0.2% | 1 |
| synapse | 64 out of 7722 genes, 0.8% | 70 out of 9057 genes, 0.8% | 1 |
| intracellular organelle part | 1616 out of 7722 genes, 20.9% | 1875 out of 9057 genes, 20.7% | 1 |
| chromatin | 46 out of 7722 genes, 0.6% | 50 out of 9057 genes, 0.6% | 1 |
| nuclear periphery | 13 out of 7722 genes, 0.2% | 13 out of 9057 genes, 0.1% | 1 |
| cytoplasmic vesicle membrane | 67 out of 7722 genes, 0.9% | 74 out of 9057 genes, 0.8% | 1 |
| cytoplasmic vesicle part | 67 out of 7722 genes, 0.9% | 74 out of 9057 genes, 0.8% | 1 |
| organelle part | 1791 out of 7722 genes, 23.2% | 2082 out of 9057 genes, 23.0% | 1 |
| nuclear chromatin | 12 out of 7722 genes, 0.2% | 12 out of 9057 genes, 0.1% | 1 |
| transport vesicle | 36 out of 7722 genes, 0.5% | 39 out of 9057 genes, 0.4% | 1 |
| membrane fraction | 64 out of 7722 genes, 0.8% | 71 out of 9057 genes, 0.8% | 1 |
| lytic vacuole | 50 out of 7722 genes, 0.6% | 55 out of 9057 genes, 0.6% | 1 |
| condensed chromosome | 28 out of 7722 genes, 0.4% | 30 out of 9057 genes, 0.3% | 1 |
| trans-Golgi network transport vesicle membrane | 20 out of 7722 genes, 0.3% | 21 out of 9057 genes, 0.2% | 1 |
| trans-Golgi network transport vesicle | 20 out of 7722 genes, 0.3% | 21 out of 9057 genes, 0.2% | 1 |
| Golgi-associated vesicle membrane | 20 out of 7722 genes, 0.3% | 21 out of 9057 genes, 0.2% | 1 |
| nonmotile primary cilium | 11 out of 7722 genes, 0.1% | 11 out of 9057 genes, 0.1% | 1 |
| cell projection | 345 out of 7722 genes, 4.5% | 397 out of 9057 genes, 4.4% | 1 |
| histone deacetylase complex | 10 out of 7722 genes, 0.1% | 10 out of 9057 genes, 0.1% | 1 |
| transcription elongation factor complex | 10 out of 7722 genes, 0.1% | 10 out of 9057 genes, 0.1% | 1 |
| cell fraction | 226 out of 7722 genes, 2.9% | 259 out of 9057 genes, 2.9% | 1 |
| nuclear lumen | 446 out of 7722 genes, 5.8% | 515 out of 9057 genes, 5.7% | 1 |
| coated vesicle membrane | 60 out of 7722 genes, 0.8% | 67 out of 9057 genes, 0.7% | 1 |
| nuclear body | 53 out of 7722 genes, 0.7% | 59 out of 9057 genes, 0.7% | 1 |
| cell projection membrane | 25 out of 7722 genes, 0.3% | 27 out of 9057 genes, 0.3% | 1 |
| transport vesicle membrane | 32 out of 7722 genes, 0.4% | 35 out of 9057 genes, 0.4% | 1 |
| protein complex | 1063 out of 7722 genes, 13.8% | 1236 out of 9057 genes, 13.6% | 1 |
| vesicular fraction | 45 out of 7722 genes, 0.6% | 50 out of 9057 genes, 0.6% | 1 |
| endocytic vesicle membrane | 9 out of 7722 genes, 0.1% | 9 out of 9057 genes, 0.1% | 1 |
| AP-type membrane coat adaptor complex | 38 out of 7722 genes, 0.5% | 42 out of 9057 genes, 0.5% | 1 |
| organelle inner membrane | 141 out of 7722 genes, 1.8% | 161 out of 9057 genes, 1.8% | 1 |
| cell projection part | 58 out of 7722 genes, 0.8% | 65 out of 9057 genes, 0.7% | 1 |
| cell periphery | 504 out of 7722 genes, 6.5% | 584 out of 9057 genes, 6.4% | 1 |
| vesicle membrane | 70 out of 7722 genes, 0.9% | 79 out of 9057 genes, 0.9% | 1 |
| macromolecular complex | 1520 out of 7722 genes, 19.7% | 1772 out of 9057 genes, 19.6% | 1 |
| vacuole | 76 out of 7722 genes, 1.0% | 86 out of 9057 genes, 0.9% | 1 |
| Golgi-associated vesicle | 30 out of 7722 genes, 0.4% | 33 out of 9057 genes, 0.4% | 1 |
| neuron projection | 194 out of 7722 genes, 2.5% | 223 out of 9057 genes, 2.5% | 1 |
| plasma membrane | 490 out of 7722 genes, 6.3% | 568 out of 9057 genes, 6.3% | 1 |
| dendrite | 23 out of 7722 genes, 0.3% | 25 out of 9057 genes, 0.3% | 1 |
| cell body | 156 out of 7722 genes, 2.0% | 179 out of 9057 genes, 2.0% | 1 |
| sex chromosome | 8 out of 7722 genes, 0.1% | 8 out of 9057 genes, 0.1% | 1 |
| clathrin coat of trans-Golgi network vesicle | 8 out of 7722 genes, 0.1% | 8 out of 9057 genes, 0.1% | 1 |
| integral to membrane | 241 out of 7722 genes, 3.1% | 278 out of 9057 genes, 3.1% | 1 |
| cytoplasmic membrane-bounded vesicle | 192 out of 7722 genes, 2.5% | 221 out of 9057 genes, 2.4% | 1 |
| membrane coat | 68 out of 7722 genes, 0.9% | 77 out of 9057 genes, 0.9% | 1 |
| coated membrane | 68 out of 7722 genes, 0.9% | 77 out of 9057 genes, 0.9% | 1 |
| cilium | 29 out of 7722 genes, 0.4% | 32 out of 9057 genes, 0.4% | 1 |
| cullin-RING ubiquitin ligase complex | 29 out of 7722 genes, 0.4% | 32 out of 9057 genes, 0.4% | 1 |
| organelle lumen | 533 out of 7722 genes, 6.9% | 619 out of 9057 genes, 6.8% | 1 |
| intracellular organelle lumen | 533 out of 7722 genes, 6.9% | 619 out of 9057 genes, 6.8% | 1 |
| clathrin coat | 15 out of 7722 genes, 0.2% | 16 out of 9057 genes, 0.2% | 1 |
| ribonucleoprotein complex | 364 out of 7722 genes, 4.7% | 422 out of 9057 genes, 4.7% | 1 |
| membrane-enclosed lumen | 536 out of 7722 genes, 6.9% | 623 out of 9057 genes, 6.9% | 1 |
| respiratory chain | 28 out of 7722 genes, 0.4% | 31 out of 9057 genes, 0.3% | 1 |
| apicolateral plasma membrane | 21 out of 7722 genes, 0.3% | 23 out of 9057 genes, 0.3% | 1 |
| apical junction complex | 21 out of 7722 genes, 0.3% | 23 out of 9057 genes, 0.3% | 1 |
| clathrin coat of endocytic vesicle | 7 out of 7722 genes, 0.1% | 7 out of 9057 genes, 0.1% | 1 |
| clathrin-coated endocytic vesicle membrane | 7 out of 7722 genes, 0.1% | 7 out of 9057 genes, 0.1% | 1 |
| clathrin-coated endocytic vesicle | 7 out of 7722 genes, 0.1% | 7 out of 9057 genes, 0.1% | 1 |
| spindle microtubule | 14 out of 7722 genes, 0.2% | 15 out of 9057 genes, 0.2% | 1 |
| cytoplasmic vesicle | 211 out of 7722 genes, 2.7% | 244 out of 9057 genes, 2.7% | 1 |
| microtubule associated complex | 58 out of 7722 genes, 0.8% | 66 out of 9057 genes, 0.7% | 1 |
| extrinsic to membrane | 20 out of 7722 genes, 0.3% | 22 out of 9057 genes, 0.2% | 1 |
| membrane-bounded vesicle | 196 out of 7722 genes, 2.5% | 227 out of 9057 genes, 2.5% | 1 |
| coated pit | 13 out of 7722 genes, 0.2% | 14 out of 9057 genes, 0.2% | 1 |
| clathrin vesicle coat | 13 out of 7722 genes, 0.2% | 14 out of 9057 genes, 0.2% | 1 |
| neuronal cell body | 13 out of 7722 genes, 0.2% | 14 out of 9057 genes, 0.2% | 1 |
| cell | 7421 out of 7722 genes, 96.1% | 8701 out of 9057 genes, 96.1% | 1 |
| Golgi apparatus | 123 out of 7722 genes, 1.6% | 142 out of 9057 genes, 1.6% | 1 |
| preribosome | 19 out of 7722 genes, 0.2% | 21 out of 9057 genes, 0.2% | 1 |
| mitochondrial inner membrane | 50 out of 7722 genes, 0.6% | 57 out of 9057 genes, 0.6% | 1 |
| chromosome, telomeric region | 6 out of 7722 genes, 0.1% | 6 out of 9057 genes, 0.1% | 1 |
| heterochromatin | 6 out of 7722 genes, 0.1% | 6 out of 9057 genes, 0.1% | 1 |
| cell surface | 6 out of 7722 genes, 0.1% | 6 out of 9057 genes, 0.1% | 1 |
| integral to endoplasmic reticulum membrane | 6 out of 7722 genes, 0.1% | 6 out of 9057 genes, 0.1% | 1 |
| cell envelope | 6 out of 7722 genes, 0.1% | 6 out of 9057 genes, 0.1% | 1 |
| anchored to membrane | 6 out of 7722 genes, 0.1% | 6 out of 9057 genes, 0.1% | 1 |
| signal recognition particle | 6 out of 7722 genes, 0.1% | 6 out of 9057 genes, 0.1% | 1 |
| cell part | 7419 out of 7722 genes, 96.1% | 8699 out of 9057 genes, 96.0% | 1 |
| microtubule cytoskeleton | 328 out of 7722 genes, 4.2% | 382 out of 9057 genes, 4.2% | 1 |
| replication fork | 12 out of 7722 genes, 0.2% | 13 out of 9057 genes, 0.1% | 1 |
| Golgi apparatus part | 114 out of 7722 genes, 1.5% | 132 out of 9057 genes, 1.5% | 1 |
| plasma membrane part | 454 out of 7722 genes, 5.9% | 530 out of 9057 genes, 5.9% | 1 |
| mitochondrial matrix | 30 out of 7722 genes, 0.4% | 34 out of 9057 genes, 0.4% | 1 |
| synaptonemal complex | 5 out of 7722 genes, 0.1% | 5 out of 9057 genes, 0.1% | 1 |
| Golgi stack | 5 out of 7722 genes, 0.1% | 5 out of 9057 genes, 0.1% | 1 |
| mRNA cleavage factor complex | 5 out of 7722 genes, 0.1% | 5 out of 9057 genes, 0.1% | 1 |
| vesicle coat | 35 out of 7722 genes, 0.5% | 40 out of 9057 genes, 0.4% | 1 |
| mitochondrial respiratory chain | 23 out of 7722 genes, 0.3% | 26 out of 9057 genes, 0.3% | 1 |
| endocytic vesicle | 23 out of 7722 genes, 0.3% | 26 out of 9057 genes, 0.3% | 1 |
| extrinsic to plasma membrane | 17 out of 7722 genes, 0.2% | 19 out of 9057 genes, 0.2% | 1 |
| cytoplasm | 1622 out of 7722 genes, 21.0% | 1900 out of 9057 genes, 21.0% | 1 |
| non-membrane-bounded organelle | 1013 out of 7722 genes, 13.1% | 1186 out of 9057 genes, 13.1% | 1 |
| intracellular non-membrane-bounded organelle | 1013 out of 7722 genes, 13.1% | 1186 out of 9057 genes, 13.1% | 1 |
| endoplasmic reticulum part | 92 out of 7722 genes, 1.2% | 107 out of 9057 genes, 1.2% | 1 |
| basolateral plasma membrane | 57 out of 7722 genes, 0.7% | 66 out of 9057 genes, 0.7% | 1 |
| microtubule | 22 out of 7722 genes, 0.3% | 25 out of 9057 genes, 0.3% | 1 |
| cell-cell junction | 80 out of 7722 genes, 1.0% | 93 out of 9057 genes, 1.0% | 1 |
| myofibril | 103 out of 7722 genes, 1.3% | 120 out of 9057 genes, 1.3% | 1 |
| integral to organelle membrane | 10 out of 7722 genes, 0.1% | 11 out of 9057 genes, 0.1% | 1 |
| SWI/SNF-type complex | 10 out of 7722 genes, 0.1% | 11 out of 9057 genes, 0.1% | 1 |
| vesicle | 235 out of 7722 genes, 3.0% | 275 out of 9057 genes, 3.0% | 1 |
| proton-transporting V-type ATPase complex | 21 out of 7722 genes, 0.3% | 24 out of 9057 genes, 0.3% | 1 |
| nuclear heterochromatin | 4 out of 7722 genes, 0.1% | 4 out of 9057 genes, 0.0% | 1 |
| cis-Golgi network | 4 out of 7722 genes, 0.1% | 4 out of 9057 genes, 0.0% | 1 |
| unconventional myosin complex | 4 out of 7722 genes, 0.1% | 4 out of 9057 genes, 0.0% | 1 |
| nuclear speck | 4 out of 7722 genes, 0.1% | 4 out of 9057 genes, 0.0% | 1 |
| cell leading edge | 32 out of 7722 genes, 0.4% | 37 out of 9057 genes, 0.4% | 1 |
| small nuclear ribonucleoprotein complex | 26 out of 7722 genes, 0.3% | 30 out of 9057 genes, 0.3% | 1 |
| intrinsic to plasma membrane | 65 out of 7722 genes, 0.8% | 76 out of 9057 genes, 0.8% | 1 |
| axon part | 20 out of 7722 genes, 0.3% | 23 out of 9057 genes, 0.3% | 1 |
| actin filament | 9 out of 7722 genes, 0.1% | 10 out of 9057 genes, 0.1% | 1 |
| vacuolar proton-transporting V-type ATPase complex | 9 out of 7722 genes, 0.1% | 10 out of 9057 genes, 0.1% | 1 |
| vacuolar membrane | 25 out of 7722 genes, 0.3% | 29 out of 9057 genes, 0.3% | 1 |
| intrinsic to endoplasmic reticulum membrane | 25 out of 7722 genes, 0.3% | 29 out of 9057 genes, 0.3% | 1 |
| vacuolar part | 25 out of 7722 genes, 0.3% | 29 out of 9057 genes, 0.3% | 1 |
| stored secretory granule | 14 out of 7722 genes, 0.2% | 16 out of 9057 genes, 0.2% | 1 |
| contractile fiber | 114 out of 7722 genes, 1.5% | 134 out of 9057 genes, 1.5% | 1 |
| membrane | 2520 out of 7722 genes, 32.6% | 2959 out of 9057 genes, 32.7% | 1 |
| cytoplasmic part | 1534 out of 7722 genes, 19.9% | 1802 out of 9057 genes, 19.9% | 1 |
| basement membrane | 19 out of 7722 genes, 0.2% | 22 out of 9057 genes, 0.2% | 1 |
| endosome | 63 out of 7722 genes, 0.8% | 74 out of 9057 genes, 0.8% | 1 |
| integral to plasma membrane | 63 out of 7722 genes, 0.8% | 74 out of 9057 genes, 0.8% | 1 |
| anchoring junction | 63 out of 7722 genes, 0.8% | 74 out of 9057 genes, 0.8% | 1 |
| endoplasmic reticulum membrane | 74 out of 7722 genes, 1.0% | 87 out of 9057 genes, 1.0% | 1 |
| A band | 35 out of 7722 genes, 0.5% | 41 out of 9057 genes, 0.5% | 1 |
| mitochondrial membrane part | 35 out of 7722 genes, 0.5% | 41 out of 9057 genes, 0.5% | 1 |
| nucleolus | 8 out of 7722 genes, 0.1% | 9 out of 9057 genes, 0.1% | 1 |
| cell-cell adherens junction | 8 out of 7722 genes, 0.1% | 9 out of 9057 genes, 0.1% | 1 |
| plastid | 13 out of 7722 genes, 0.2% | 15 out of 9057 genes, 0.2% | 1 |
| ER to Golgi transport vesicle | 13 out of 7722 genes, 0.2% | 15 out of 9057 genes, 0.2% | 1 |
| cyclin-dependent protein kinase holoenzyme complex | 3 out of 7722 genes, 0.0% | 3 out of 9057 genes, 0.0% | 1 |
| sex chromatin | 3 out of 7722 genes, 0.0% | 3 out of 9057 genes, 0.0% | 1 |
| ER-Golgi intermediate compartment | 3 out of 7722 genes, 0.0% | 3 out of 9057 genes, 0.0% | 1 |
| septate junction | 3 out of 7722 genes, 0.0% | 3 out of 9057 genes, 0.0% | 1 |
| cohesin complex | 3 out of 7722 genes, 0.0% | 3 out of 9057 genes, 0.0% | 1 |
| plastid stroma | 3 out of 7722 genes, 0.0% | 3 out of 9057 genes, 0.0% | 1 |
| internal side of plasma membrane | 3 out of 7722 genes, 0.0% | 3 out of 9057 genes, 0.0% | 1 |
| inclusion body | 3 out of 7722 genes, 0.0% | 3 out of 9057 genes, 0.0% | 1 |
| secretory granule membrane | 3 out of 7722 genes, 0.0% | 3 out of 9057 genes, 0.0% | 1 |
| pseudopodium | 3 out of 7722 genes, 0.0% | 3 out of 9057 genes, 0.0% | 1 |
| receptor complex | 3 out of 7722 genes, 0.0% | 3 out of 9057 genes, 0.0% | 1 |
| pronucleus | 3 out of 7722 genes, 0.0% | 3 out of 9057 genes, 0.0% | 1 |
| occluding junction | 3 out of 7722 genes, 0.0% | 3 out of 9057 genes, 0.0% | 1 |
| Sin3-type complex | 3 out of 7722 genes, 0.0% | 3 out of 9057 genes, 0.0% | 1 |
| mitochondrial membrane | 78 out of 7722 genes, 1.0% | 92 out of 9057 genes, 1.0% | 1 |
| endomembrane system | 347 out of 7722 genes, 4.5% | 409 out of 9057 genes, 4.5% | 1 |
| I band | 23 out of 7722 genes, 0.3% | 27 out of 9057 genes, 0.3% | 1 |
| mitochondrial lumen | 88 out of 7722 genes, 1.1% | 104 out of 9057 genes, 1.1% | 1 |
| proteasome complex | 44 out of 7722 genes, 0.6% | 52 out of 9057 genes, 0.6% | 1 |
| chromosome, centromeric region | 44 out of 7722 genes, 0.6% | 52 out of 9057 genes, 0.6% | 1 |
| sarcomere | 93 out of 7722 genes, 1.2% | 110 out of 9057 genes, 1.2% | 1 |
| contractile fiber part | 104 out of 7722 genes, 1.3% | 123 out of 9057 genes, 1.4% | 1 |
| nuclear membrane-endoplasmic reticulum network | 76 out of 7722 genes, 1.0% | 90 out of 9057 genes, 1.0% | 1 |
| microbody | 38 out of 7722 genes, 0.5% | 45 out of 9057 genes, 0.5% | 1 |
| ER to Golgi transport vesicle membrane | 12 out of 7722 genes, 0.2% | 14 out of 9057 genes, 0.2% | 1 |
| ion channel complex | 12 out of 7722 genes, 0.2% | 14 out of 9057 genes, 0.2% | 1 |
| Golgi membrane | 54 out of 7722 genes, 0.7% | 64 out of 9057 genes, 0.7% | 1 |
| cytoskeletal part | 270 out of 7722 genes, 3.5% | 319 out of 9057 genes, 3.5% | 1 |
| spindle pole | 7 out of 7722 genes, 0.1% | 8 out of 9057 genes, 0.1% | 1 |
| axoneme | 7 out of 7722 genes, 0.1% | 8 out of 9057 genes, 0.1% | 1 |
| PcG protein complex | 7 out of 7722 genes, 0.1% | 8 out of 9057 genes, 0.1% | 1 |
| nucleolar part | 7 out of 7722 genes, 0.1% | 8 out of 9057 genes, 0.1% | 1 |
| endoplasmic reticulum | 119 out of 7722 genes, 1.5% | 141 out of 9057 genes, 1.6% | 1 |
| cytoskeleton | 517 out of 7722 genes, 6.7% | 610 out of 9057 genes, 6.7% | 1 |
| cell junction | 151 out of 7722 genes, 2.0% | 179 out of 9057 genes, 2.0% | 1 |
| proteasome accessory complex | 21 out of 7722 genes, 0.3% | 25 out of 9057 genes, 0.3% | 1 |
| axon | 21 out of 7722 genes, 0.3% | 25 out of 9057 genes, 0.3% | 1 |
| synapse part | 16 out of 7722 genes, 0.2% | 19 out of 9057 genes, 0.2% | 1 |
| nucleoplasm part | 166 out of 7722 genes, 2.1% | 197 out of 9057 genes, 2.2% | 1 |
| proteasome regulatory particle | 11 out of 7722 genes, 0.1% | 13 out of 9057 genes, 0.1% | 1 |
| proteasome core complex | 11 out of 7722 genes, 0.1% | 13 out of 9057 genes, 0.1% | 1 |
| nucleoplasm | 170 out of 7722 genes, 2.2% | 202 out of 9057 genes, 2.2% | 1 |
| exosome (RNase complex) | 6 out of 7722 genes, 0.1% | 7 out of 9057 genes, 0.1% | 1 |
| small nucleolar ribonucleoprotein complex | 6 out of 7722 genes, 0.1% | 7 out of 9057 genes, 0.1% | 1 |
| rough endoplasmic reticulum | 6 out of 7722 genes, 0.1% | 7 out of 9057 genes, 0.1% | 1 |
| kinesin complex | 6 out of 7722 genes, 0.1% | 7 out of 9057 genes, 0.1% | 1 |
| rough endoplasmic reticulum membrane | 6 out of 7722 genes, 0.1% | 7 out of 9057 genes, 0.1% | 1 |
| intrinsic to mitochondrial outer membrane | 6 out of 7722 genes, 0.1% | 7 out of 9057 genes, 0.1% | 1 |
| organelle subcompartment | 6 out of 7722 genes, 0.1% | 7 out of 9057 genes, 0.1% | 1 |
| mismatch repair complex | 6 out of 7722 genes, 0.1% | 7 out of 9057 genes, 0.1% | 1 |
| H4/H2A histone acetyltransferase complex | 6 out of 7722 genes, 0.1% | 7 out of 9057 genes, 0.1% | 1 |
| plastid part | 6 out of 7722 genes, 0.1% | 7 out of 9057 genes, 0.1% | 1 |
| CUL4 RING ubiquitin ligase complex | 6 out of 7722 genes, 0.1% | 7 out of 9057 genes, 0.1% | 1 |
| SSL2-core TFIIH complex | 2 out of 7722 genes, 0.0% | 2 out of 9057 genes, 0.0% | 1 |
| origin recognition complex | 2 out of 7722 genes, 0.0% | 2 out of 9057 genes, 0.0% | 1 |
| acrosomal vesicle | 2 out of 7722 genes, 0.0% | 2 out of 9057 genes, 0.0% | 1 |
| microsome | 2 out of 7722 genes, 0.0% | 2 out of 9057 genes, 0.0% | 1 |
| microvillus | 2 out of 7722 genes, 0.0% | 2 out of 9057 genes, 0.0% | 1 |
| ionotropic glutamate receptor complex | 2 out of 7722 genes, 0.0% | 2 out of 9057 genes, 0.0% | 1 |
| rhabdomere | 2 out of 7722 genes, 0.0% | 2 out of 9057 genes, 0.0% | 1 |
| filopodium | 2 out of 7722 genes, 0.0% | 2 out of 9057 genes, 0.0% | 1 |
| RNA polymerase complex | 2 out of 7722 genes, 0.0% | 2 out of 9057 genes, 0.0% | 1 |
| ISWI complex | 2 out of 7722 genes, 0.0% | 2 out of 9057 genes, 0.0% | 1 |
| Golgi cisterna | 2 out of 7722 genes, 0.0% | 2 out of 9057 genes, 0.0% | 1 |
| sarcolemma | 2 out of 7722 genes, 0.0% | 2 out of 9057 genes, 0.0% | 1 |
| plasma membrane-derived chromatophore | 2 out of 7722 genes, 0.0% | 2 out of 9057 genes, 0.0% | 1 |
| anchored to plasma membrane | 2 out of 7722 genes, 0.0% | 2 out of 9057 genes, 0.0% | 1 |
| envelope | 244 out of 7722 genes, 3.2% | 290 out of 9057 genes, 3.2% | 1 |
| kinetochore | 10 out of 7722 genes, 0.1% | 12 out of 9057 genes, 0.1% | 1 |
| proton-transporting two-sector ATPase complex, catalytic domain | 10 out of 7722 genes, 0.1% | 12 out of 9057 genes, 0.1% | 1 |
| cell cortex | 24 out of 7722 genes, 0.3% | 29 out of 9057 genes, 0.3% | 1 |
| cell division site | 24 out of 7722 genes, 0.3% | 29 out of 9057 genes, 0.3% | 1 |
| cell division site part | 24 out of 7722 genes, 0.3% | 29 out of 9057 genes, 0.3% | 1 |
| transcription factor complex | 44 out of 7722 genes, 0.6% | 53 out of 9057 genes, 0.6% | 1 |
| proton-transporting two-sector ATPase complex | 44 out of 7722 genes, 0.6% | 53 out of 9057 genes, 0.6% | 1 |
| organelle membrane | 469 out of 7722 genes, 6.1% | 556 out of 9057 genes, 6.1% | 1 |
| protein serine/threonine phosphatase complex | 19 out of 7722 genes, 0.2% | 23 out of 9057 genes, 0.3% | 1 |
| cell cortex part | 23 out of 7722 genes, 0.3% | 28 out of 9057 genes, 0.3% | 1 |
| germ cell nucleus | 5 out of 7722 genes, 0.1% | 6 out of 9057 genes, 0.1% | 1 |
| intercellular bridge | 5 out of 7722 genes, 0.1% | 6 out of 9057 genes, 0.1% | 1 |
| small ribosomal subunit | 18 out of 7722 genes, 0.2% | 22 out of 9057 genes, 0.2% | 1 |
| polytene chromosome | 9 out of 7722 genes, 0.1% | 11 out of 9057 genes, 0.1% | 1 |
| cortical cytoskeleton | 9 out of 7722 genes, 0.1% | 11 out of 9057 genes, 0.1% | 1 |
| organelle envelope | 238 out of 7722 genes, 3.1% | 284 out of 9057 genes, 3.1% | 1 |
| adherens junction | 37 out of 7722 genes, 0.5% | 45 out of 9057 genes, 0.5% | 1 |
| pigment granule | 13 out of 7722 genes, 0.2% | 16 out of 9057 genes, 0.2% | 1 |
| site of polarized growth | 17 out of 7722 genes, 0.2% | 21 out of 9057 genes, 0.2% | 1 |
| basal plasma membrane | 8 out of 7722 genes, 0.1% | 10 out of 9057 genes, 0.1% | 1 |
| cortical actin cytoskeleton | 8 out of 7722 genes, 0.1% | 10 out of 9057 genes, 0.1% | 1 |
| basal part of cell | 8 out of 7722 genes, 0.1% | 10 out of 9057 genes, 0.1% | 1 |
| extracellular region part | 188 out of 7722 genes, 2.4% | 226 out of 9057 genes, 2.5% | 1 |
| peroxisome | 16 out of 7722 genes, 0.2% | 20 out of 9057 genes, 0.2% | 1 |
| ruffle | 4 out of 7722 genes, 0.1% | 5 out of 9057 genes, 0.1% | 1 |
| mitochondrial intermembrane space | 4 out of 7722 genes, 0.1% | 5 out of 9057 genes, 0.1% | 1 |
| peroxisomal membrane | 4 out of 7722 genes, 0.1% | 5 out of 9057 genes, 0.1% | 1 |
| synaptic vesicle | 4 out of 7722 genes, 0.1% | 5 out of 9057 genes, 0.1% | 1 |
| growth cone | 4 out of 7722 genes, 0.1% | 5 out of 9057 genes, 0.1% | 1 |
| intrinsic to peroxisomal membrane | 4 out of 7722 genes, 0.1% | 5 out of 9057 genes, 0.1% | 1 |
| intrinsic to mitochondrial inner membrane | 4 out of 7722 genes, 0.1% | 5 out of 9057 genes, 0.1% | 1 |
| microbody membrane | 4 out of 7722 genes, 0.1% | 5 out of 9057 genes, 0.1% | 1 |
| organelle envelope lumen | 4 out of 7722 genes, 0.1% | 5 out of 9057 genes, 0.1% | 1 |
| female germline ring canal | 4 out of 7722 genes, 0.1% | 5 out of 9057 genes, 0.1% | 1 |
| germline ring canal | 4 out of 7722 genes, 0.1% | 5 out of 9057 genes, 0.1% | 1 |
| tricarboxylic acid cycle enzyme complex | 4 out of 7722 genes, 0.1% | 5 out of 9057 genes, 0.1% | 1 |
| membrane part | 1862 out of 7722 genes, 24.1% | 2201 out of 9057 genes, 24.3% | 1 |
| extracellular matrix | 112 out of 7722 genes, 1.5% | 136 out of 9057 genes, 1.5% | 1 |
| late endosome | 11 out of 7722 genes, 0.1% | 14 out of 9057 genes, 0.2% | 1 |
| histone methyltransferase complex | 11 out of 7722 genes, 0.1% | 14 out of 9057 genes, 0.2% | 1 |
| mitochondrial outer membrane | 7 out of 7722 genes, 0.1% | 9 out of 9057 genes, 0.1% | 1 |
| aster | 7 out of 7722 genes, 0.1% | 9 out of 9057 genes, 0.1% | 1 |
| endosome membrane | 7 out of 7722 genes, 0.1% | 9 out of 9057 genes, 0.1% | 1 |
| outer membrane | 7 out of 7722 genes, 0.1% | 9 out of 9057 genes, 0.1% | 1 |
| RNAi effector complex | 7 out of 7722 genes, 0.1% | 9 out of 9057 genes, 0.1% | 1 |
| organelle outer membrane | 7 out of 7722 genes, 0.1% | 9 out of 9057 genes, 0.1% | 1 |
| cation channel complex | 7 out of 7722 genes, 0.1% | 9 out of 9057 genes, 0.1% | 1 |
| endosomal part | 7 out of 7722 genes, 0.1% | 9 out of 9057 genes, 0.1% | 1 |
| microtubule organizing center | 71 out of 7722 genes, 0.9% | 87 out of 9057 genes, 1.0% | 1 |
| intrinsic to organelle membrane | 51 out of 7722 genes, 0.7% | 63 out of 9057 genes, 0.7% | 1 |
| proteinaceous extracellular matrix | 85 out of 7722 genes, 1.1% | 104 out of 9057 genes, 1.1% | 1 |
| histone acetyltransferase complex | 14 out of 7722 genes, 0.2% | 18 out of 9057 genes, 0.2% | 1 |
| microbody part | 14 out of 7722 genes, 0.2% | 18 out of 9057 genes, 0.2% | 1 |
| peroxisomal part | 14 out of 7722 genes, 0.2% | 18 out of 9057 genes, 0.2% | 1 |
| chloroplast | 3 out of 7722 genes, 0.0% | 4 out of 9057 genes, 0.0% | 1 |
| photosystem | 3 out of 7722 genes, 0.0% | 4 out of 9057 genes, 0.0% | 1 |
| chloroplast thylakoid | 3 out of 7722 genes, 0.0% | 4 out of 9057 genes, 0.0% | 1 |
| thylakoid | 3 out of 7722 genes, 0.0% | 4 out of 9057 genes, 0.0% | 1 |
| dystrophin-associated glycoprotein complex | 3 out of 7722 genes, 0.0% | 4 out of 9057 genes, 0.0% | 1 |
| dystroglycan complex | 3 out of 7722 genes, 0.0% | 4 out of 9057 genes, 0.0% | 1 |
| transcriptional repressor complex | 3 out of 7722 genes, 0.0% | 4 out of 9057 genes, 0.0% | 1 |
| dynein complex | 3 out of 7722 genes, 0.0% | 4 out of 9057 genes, 0.0% | 1 |
| intraflagellar transport particle | 3 out of 7722 genes, 0.0% | 4 out of 9057 genes, 0.0% | 1 |
| nuclear membrane | 3 out of 7722 genes, 0.0% | 4 out of 9057 genes, 0.0% | 1 |
| plastid thylakoid | 3 out of 7722 genes, 0.0% | 4 out of 9057 genes, 0.0% | 1 |
| septin cytoskeleton | 3 out of 7722 genes, 0.0% | 4 out of 9057 genes, 0.0% | 1 |
| photosynthetic membrane | 3 out of 7722 genes, 0.0% | 4 out of 9057 genes, 0.0% | 1 |
| chloroplast part | 3 out of 7722 genes, 0.0% | 4 out of 9057 genes, 0.0% | 1 |
| thylakoid part | 3 out of 7722 genes, 0.0% | 4 out of 9057 genes, 0.0% | 1 |
| axoneme part | 3 out of 7722 genes, 0.0% | 4 out of 9057 genes, 0.0% | 1 |
| dihydrolipoyl dehydrogenase complex | 3 out of 7722 genes, 0.0% | 4 out of 9057 genes, 0.0% | 1 |
| proton-transporting ATP synthase complex | 3 out of 7722 genes, 0.0% | 4 out of 9057 genes, 0.0% | 1 |
| spindle | 49 out of 7722 genes, 0.6% | 61 out of 9057 genes, 0.7% | 1 |
| cell-substrate adherens junction | 26 out of 7722 genes, 0.3% | 33 out of 9057 genes, 0.4% | 1 |
| myosin complex | 26 out of 7722 genes, 0.3% | 33 out of 9057 genes, 0.4% | 1 |
| cell-substrate junction | 26 out of 7722 genes, 0.3% | 33 out of 9057 genes, 0.4% | 1 |
| organellar small ribosomal subunit | 6 out of 7722 genes, 0.1% | 8 out of 9057 genes, 0.1% | 1 |
| nuclear envelope | 52 out of 7722 genes, 0.7% | 65 out of 9057 genes, 0.7% | 1 |
| large ribosomal subunit | 9 out of 7722 genes, 0.1% | 12 out of 9057 genes, 0.1% | 1 |
| mitochondrial envelope | 99 out of 7722 genes, 1.3% | 122 out of 9057 genes, 1.3% | 1 |
| extracellular region | 199 out of 7722 genes, 2.6% | 242 out of 9057 genes, 2.7% | 1 |
| mitochondrial part | 183 out of 7722 genes, 2.4% | 223 out of 9057 genes, 2.5% | 1 |
| methyltransferase complex | 12 out of 7722 genes, 0.2% | 16 out of 9057 genes, 0.2% | 1 |
| mitochondrion | 208 out of 7722 genes, 2.7% | 253 out of 9057 genes, 2.8% | 1 |
| ribosome | 50 out of 7722 genes, 0.6% | 63 out of 9057 genes, 0.7% | 1 |
| organellar large ribosomal subunit | 5 out of 7722 genes, 0.1% | 7 out of 9057 genes, 0.1% | 1 |
| basal lamina | 5 out of 7722 genes, 0.1% | 7 out of 9057 genes, 0.1% | 1 |
| actin filament bundle | 5 out of 7722 genes, 0.1% | 7 out of 9057 genes, 0.1% | 1 |
| cytoplasmic ubiquitin ligase complex | 2 out of 7722 genes, 0.0% | 3 out of 9057 genes, 0.0% | 1 |
| transcription export complex | 2 out of 7722 genes, 0.0% | 3 out of 9057 genes, 0.0% | 1 |
| collagen | 2 out of 7722 genes, 0.0% | 3 out of 9057 genes, 0.0% | 1 |
| nuclear pore | 2 out of 7722 genes, 0.0% | 3 out of 9057 genes, 0.0% | 1 |
| mitochondrial proton-transporting ATP synthase complex | 2 out of 7722 genes, 0.0% | 3 out of 9057 genes, 0.0% | 1 |
| endoplasmic reticulum lumen | 2 out of 7722 genes, 0.0% | 3 out of 9057 genes, 0.0% | 1 |
| striated muscle thin filament | 2 out of 7722 genes, 0.0% | 3 out of 9057 genes, 0.0% | 1 |
| palmitoyltransferase complex | 2 out of 7722 genes, 0.0% | 3 out of 9057 genes, 0.0% | 1 |
| pore complex | 2 out of 7722 genes, 0.0% | 3 out of 9057 genes, 0.0% | 1 |
| muscle myosin complex | 7 out of 7722 genes, 0.1% | 10 out of 9057 genes, 0.1% | 1 |
| myosin II complex | 7 out of 7722 genes, 0.1% | 10 out of 9057 genes, 0.1% | 1 |
| calcium channel complex | 4 out of 7722 genes, 0.1% | 6 out of 9057 genes, 0.1% | 1 |
| laminin complex | 4 out of 7722 genes, 0.1% | 6 out of 9057 genes, 0.1% | 1 |
| ribosomal subunit | 46 out of 7722 genes, 0.6% | 59 out of 9057 genes, 0.7% | 1 |
| early endosome | 6 out of 7722 genes, 0.1% | 9 out of 9057 genes, 0.1% | 1 |
| RNA cap binding complex | 6 out of 7722 genes, 0.1% | 9 out of 9057 genes, 0.1% | 1 |
| actin cytoskeleton | 102 out of 7722 genes, 1.3% | 128 out of 9057 genes, 1.4% | 1 |
| extracellular matrix part | 23 out of 7722 genes, 0.3% | 31 out of 9057 genes, 0.3% | 1 |
| COPI-coated vesicle | 3 out of 7722 genes, 0.0% | 5 out of 9057 genes, 0.1% | 1 |
| intrinsic to Golgi membrane | 8 out of 7722 genes, 0.1% | 12 out of 9057 genes, 0.1% | 1 |
| SAGA-type complex | 5 out of 7722 genes, 0.1% | 8 out of 9057 genes, 0.1% | 1 |
| intrinsic to membrane | 1327 out of 7722 genes, 17.2% | 1588 out of 9057 genes, 17.5% | 1 |
| condensed chromosome kinetochore | 2 out of 7722 genes, 0.0% | 4 out of 9057 genes, 0.0% | 1 |
| condensed chromosome, centromeric region | 2 out of 7722 genes, 0.0% | 4 out of 9057 genes, 0.0% | 1 |
| intermediate filament | 2 out of 7722 genes, 0.0% | 4 out of 9057 genes, 0.0% | 1 |
| trailing edge | 2 out of 7722 genes, 0.0% | 4 out of 9057 genes, 0.0% | 1 |
| intermediate filament cytoskeleton | 2 out of 7722 genes, 0.0% | 4 out of 9057 genes, 0.0% | 1 |
| organellar ribosome | 14 out of 7722 genes, 0.2% | 21 out of 9057 genes, 0.2% | 1 |
| DNA-directed RNA polymerase II, holoenzyme | 17 out of 7722 genes, 0.2% | 25 out of 9057 genes, 0.3% | 1 |

| Gene Ontology term | Genes annotated to the term |
| --- | --- |
| chromosome | Unigene38023\_Sample\_011046840, Unigene29269\_Sample\_011046840, Unigene39900\_Sample\_011046840, Unigene18228\_Sample\_011046840, Unigene28409\_Sample\_011046840, Unigene368\_Sample\_011046840, Unigene13117\_Sample\_011046840, Unigene41910\_Sample\_011046840, Unigene40049\_Sample\_011046840, Unigene40685\_Sample\_011046840, Unigene18212\_Sample\_011046840, Unigene28265\_Sample\_011046840, Unigene25871\_Sample\_011046840, Unigene19499\_Sample\_011046840, Unigene33322\_Sample\_011046840, Unigene6293\_Sample\_011046840, Unigene38540\_Sample\_011046840, Unigene33344\_Sample\_011046840, Unigene33482\_Sample\_011046840, Unigene32488\_Sample\_011046840, Unigene42166\_Sample\_011046840, Unigene5867\_Sample\_011046840, Unigene39391\_Sample\_011046840, Unigene31572\_Sample\_011046840, Unigene35789\_Sample\_011046840, Unigene39649\_Sample\_011046840, Unigene40868\_Sample\_011046840, Unigene27358\_Sample\_011046840, Unigene41153\_Sample\_011046840, Unigene41588\_Sample\_011046840, Unigene43486\_Sample\_011046840, Unigene43277\_Sample\_011046840, Unigene40602\_Sample\_011046840, Unigene43606\_Sample\_011046840, Unigene15570\_Sample\_011046840, Unigene6276\_Sample\_011046840, Unigene7677\_Sample\_011046840, Unigene43397\_Sample\_011046840, Unigene41263\_Sample\_011046840, Unigene33395\_Sample\_011046840, Unigene43453\_Sample\_011046840, Unigene14648\_Sample\_011046840, Unigene42168\_Sample\_011046840, Unigene20982\_Sample\_011046840, Unigene42030\_Sample\_011046840, Unigene29635\_Sample\_011046840, Unigene30865\_Sample\_011046840, Unigene17494\_Sample\_011046840, Unigene28451\_Sample\_011046840, Unigene9704\_Sample\_011046840, Unigene15120\_Sample\_011046840, Unigene30920\_Sample\_011046840, Unigene34036\_Sample\_011046840, Unigene25063\_Sample\_011046840, Unigene20033\_Sample\_011046840, Unigene30715\_Sample\_011046840, Unigene4344\_Sample\_011046840, Unigene16618\_Sample\_011046840, Unigene25680\_Sample\_011046840, Unigene37236\_Sample\_011046840, Unigene32390\_Sample\_011046840, Unigene38690\_Sample\_011046840, Unigene14511\_Sample\_011046840, Unigene14750\_Sample\_011046840, Unigene40503\_Sample\_011046840, Unigene42626\_Sample\_011046840, Unigene32118\_Sample\_011046840, Unigene38463\_Sample\_011046840, Unigene18128\_Sample\_011046840, Unigene16850\_Sample\_011046840, Unigene22159\_Sample\_011046840, Unigene42944\_Sample\_011046840, Unigene42103\_Sample\_011046840, Unigene41018\_Sample\_011046840, Unigene28415\_Sample\_011046840, Unigene4497\_Sample\_011046840, Unigene20549\_Sample\_011046840, Unigene5782\_Sample\_011046840, Unigene16716\_Sample\_011046840, Unigene40545\_Sample\_011046840, Unigene4652\_Sample\_011046840, Unigene9962\_Sample\_011046840, Unigene40452\_Sample\_011046840, Unigene41742\_Sample\_011046840, Unigene41562\_Sample\_011046840, Unigene23252\_Sample\_011046840, Unigene14078\_Sample\_011046840, Unigene14195\_Sample\_011046840, Unigene29958\_Sample\_011046840, Unigene19982\_Sample\_011046840, Unigene38606\_Sample\_011046840, Unigene42803\_Sample\_011046840, Unigene36063\_Sample\_011046840, Unigene33611\_Sample\_011046840, Unigene40638\_Sample\_011046840, Unigene6426\_Sample\_011046840, Unigene35699\_Sample\_011046840, Unigene41052\_Sample\_011046840, Unigene12511\_Sample\_011046840, Unigene13197\_Sample\_011046840, Unigene28581\_Sample\_011046840, Unigene15140\_Sample\_011046840, Unigene27604\_Sample\_011046840, Unigene14307\_Sample\_011046840, Unigene39540\_Sample\_011046840, Unigene34752\_Sample\_011046840, Unigene40753\_Sample\_011046840, Unigene31306\_Sample\_011046840, Unigene4842\_Sample\_011046840, Unigene11967\_Sample\_011046840, Unigene32518\_Sample\_011046840, Unigene24731\_Sample\_011046840, Unigene32844\_Sample\_011046840, Unigene35895\_Sample\_011046840, Unigene10553\_Sample\_011046840, Unigene20800\_Sample\_011046840, Unigene34436\_Sample\_011046840, Unigene4723\_Sample\_011046840, Unigene37470\_Sample\_011046840, Unigene30691\_Sample\_011046840, Unigene41441\_Sample\_011046840, Unigene27185\_Sample\_011046840, Unigene43414\_Sample\_011046840, Unigene31527\_Sample\_011046840, Unigene38595\_Sample\_011046840, Unigene42502\_Sample\_011046840, Unigene41104\_Sample\_011046840, Unigene40424\_Sample\_011046840, Unigene36973\_Sample\_011046840, Unigene31513\_Sample\_011046840, Unigene12913\_Sample\_011046840, Unigene2626\_Sample\_011046840, Unigene5383\_Sample\_011046840, Unigene40411\_Sample\_011046840, Unigene22810\_Sample\_011046840, Unigene28616\_Sample\_011046840, Unigene38121\_Sample\_011046840, Unigene37826\_Sample\_011046840, Unigene25298\_Sample\_011046840, Unigene41124\_Sample\_011046840, Unigene34703\_Sample\_011046840, Unigene8006\_Sample\_011046840, Unigene25450\_Sample\_011046840, Unigene39688\_Sample\_011046840, Unigene21415\_Sample\_011046840, Unigene30760\_Sample\_011046840, Unigene40281\_Sample\_011046840, Unigene25972\_Sample\_011046840, Unigene42985\_Sample\_011046840, Unigene39836\_Sample\_011046840, Unigene39948\_Sample\_011046840, Unigene37283\_Sample\_011046840, Unigene11613\_Sample\_011046840, Unigene5720\_Sample\_011046840, Unigene31822\_Sample\_011046840, Unigene42659\_Sample\_011046840, Unigene29471\_Sample\_011046840, Unigene30185\_Sample\_011046840, Unigene27677\_Sample\_011046840, Unigene41412\_Sample\_011046840, Unigene7983\_Sample\_011046840, Unigene36915\_Sample\_011046840, Unigene20485\_Sample\_011046840, Unigene39602\_Sample\_011046840, Unigene29411\_Sample\_011046840, Unigene2740\_Sample\_011046840, Unigene28315\_Sample\_011046840, Unigene43525\_Sample\_011046840, Unigene23923\_Sample\_011046840, Unigene27478\_Sample\_011046840, Unigene40204\_Sample\_011046840, Unigene41709\_Sample\_011046840, Unigene27932\_Sample\_011046840, Unigene29328\_Sample\_011046840, Unigene29253\_Sample\_011046840, Unigene29661\_Sample\_011046840, Unigene41218\_Sample\_011046840, Unigene40061\_Sample\_011046840, Unigene25413\_Sample\_011046840, Unigene28259\_Sample\_011046840, Unigene42459\_Sample\_011046840, Unigene32304\_Sample\_011046840, Unigene3842\_Sample\_011046840, Unigene38753\_Sample\_011046840, Unigene30608\_Sample\_011046840, Unigene34046\_Sample\_011046840, Unigene28397\_Sample\_011046840, Unigene26453\_Sample\_011046840, Unigene4498\_Sample\_011046840, Unigene43273\_Sample\_011046840, Unigene41000\_Sample\_011046840, Unigene43405\_Sample\_011046840, Unigene14154\_Sample\_011046840, Unigene34459\_Sample\_011046840, Unigene29802\_Sample\_011046840, Unigene27950\_Sample\_011046840, Unigene21949\_Sample\_011046840, Unigene41899\_Sample\_011046840, Unigene36338\_Sample\_011046840, Unigene38904\_Sample\_011046840, Unigene33129\_Sample\_011046840, Unigene5197\_Sample\_011046840, Unigene41119\_Sample\_011046840, Unigene5710\_Sample\_011046840, Unigene42866\_Sample\_011046840, Unigene17585\_Sample\_011046840, Unigene22186\_Sample\_011046840, Unigene30343\_Sample\_011046840, Unigene41512\_Sample\_011046840, Unigene32413\_Sample\_011046840, Unigene43165\_Sample\_011046840, Unigene14037\_Sample\_011046840, Unigene43450\_Sample\_011046840, Unigene41339\_Sample\_011046840, Unigene39446\_Sample\_011046840, Unigene14685\_Sample\_011046840, Unigene22688\_Sample\_011046840, Unigene41383\_Sample\_011046840, Unigene32099\_Sample\_011046840, Unigene17881\_Sample\_011046840, Unigene40614\_Sample\_011046840, Unigene18463\_Sample\_011046840, Unigene26022\_Sample\_011046840, Unigene42374\_Sample\_011046840, Unigene40004\_Sample\_011046840, Unigene35170\_Sample\_011046840, Unigene43289\_Sample\_011046840, Unigene41997\_Sample\_011046840, Unigene28153\_Sample\_011046840, Unigene15302\_Sample\_011046840, Unigene22606\_Sample\_011046840, Unigene21063\_Sample\_011046840, Unigene15681\_Sample\_011046840, Unigene30599\_Sample\_011046840, Unigene15608\_Sample\_011046840, Unigene38453\_Sample\_011046840, Unigene8155\_Sample\_011046840, Unigene32050\_Sample\_011046840, Unigene24019\_Sample\_011046840, Unigene40998\_Sample\_011046840, Unigene30813\_Sample\_011046840, Unigene41337\_Sample\_011046840, Unigene33527\_Sample\_011046840, Unigene39406\_Sample\_011046840, Unigene34028\_Sample\_011046840, Unigene39591\_Sample\_011046840 |
| membrane-bounded organelle | Unigene43390\_Sample\_011046840, Unigene10428\_Sample\_011046840, Unigene29380\_Sample\_011046840, Unigene368\_Sample\_011046840, Unigene6640\_Sample\_011046840, Unigene37021\_Sample\_011046840, Unigene41047\_Sample\_011046840, Unigene37093\_Sample\_011046840, Unigene34041\_Sample\_011046840, Unigene39952\_Sample\_011046840, Unigene34674\_Sample\_011046840, Unigene28519\_Sample\_011046840, Unigene41867\_Sample\_011046840, Unigene37584\_Sample\_011046840, Unigene23713\_Sample\_011046840, Unigene5341\_Sample\_011046840, Unigene5625\_Sample\_011046840, Unigene33631\_Sample\_011046840, Unigene39857\_Sample\_011046840, Unigene4650\_Sample\_011046840, Unigene7455\_Sample\_011046840, Unigene42372\_Sample\_011046840, Unigene15271\_Sample\_011046840, Unigene42642\_Sample\_011046840, Unigene33388\_Sample\_011046840, Unigene35265\_Sample\_011046840, Unigene42712\_Sample\_011046840, Unigene21420\_Sample\_011046840, Unigene29813\_Sample\_011046840, Unigene40446\_Sample\_011046840, Unigene28087\_Sample\_011046840, Unigene32526\_Sample\_011046840, Unigene42508\_Sample\_011046840, Unigene2135\_Sample\_011046840, Unigene34971\_Sample\_011046840, Unigene30321\_Sample\_011046840, Unigene29635\_Sample\_011046840, Unigene43070\_Sample\_011046840, Unigene37375\_Sample\_011046840, Unigene40497\_Sample\_011046840, Unigene39997\_Sample\_011046840, Unigene24030\_Sample\_011046840, Unigene10975\_Sample\_011046840, Unigene7475\_Sample\_011046840, Unigene4147\_Sample\_011046840, Unigene88\_Sample\_011046840, Unigene1132\_Sample\_011046840, Unigene19291\_Sample\_011046840, Unigene31238\_Sample\_011046840, Unigene20033\_Sample\_011046840, Unigene2743\_Sample\_011046840, Unigene2196\_Sample\_011046840, Unigene25680\_Sample\_011046840, Unigene32390\_Sample\_011046840, Unigene38610\_Sample\_011046840, Unigene24844\_Sample\_011046840, Unigene19368\_Sample\_011046840, Unigene35531\_Sample\_011046840, Unigene4570\_Sample\_011046840, Unigene43241\_Sample\_011046840, Unigene27851\_Sample\_011046840, Unigene43118\_Sample\_011046840, Unigene38179\_Sample\_011046840, Unigene20209\_Sample\_011046840, Unigene38463\_Sample\_011046840, Unigene42371\_Sample\_011046840, Unigene30678\_Sample\_011046840, Unigene42381\_Sample\_011046840, Unigene14294\_Sample\_011046840, Unigene36764\_Sample\_011046840, Unigene14077\_Sample\_011046840, Unigene33318\_Sample\_011046840, Unigene2419\_Sample\_011046840, Unigene38403\_Sample\_011046840, Unigene7904\_Sample\_011046840, Unigene27749\_Sample\_011046840, Unigene3107\_Sample\_011046840, Unigene30831\_Sample\_011046840, Unigene38560\_Sample\_011046840, Unigene5804\_Sample\_011046840, Unigene41107\_Sample\_011046840, Unigene24103\_Sample\_011046840, Unigene10585\_Sample\_011046840, Unigene12511\_Sample\_011046840, Unigene7468\_Sample\_011046840, Unigene893\_Sample\_011046840, Unigene38321\_Sample\_011046840, Unigene39349\_Sample\_011046840, Unigene12001\_Sample\_011046840, Unigene4204\_Sample\_011046840, Unigene42153\_Sample\_011046840, Unigene23870\_Sample\_011046840, Unigene19033\_Sample\_011046840, Unigene25022\_Sample\_011046840, Unigene31067\_Sample\_011046840, Unigene11967\_Sample\_011046840, Unigene21399\_Sample\_011046840, Unigene5389\_Sample\_011046840, Unigene33001\_Sample\_011046840, Unigene38464\_Sample\_011046840, Unigene13449\_Sample\_011046840, Unigene22269\_Sample\_011046840, Unigene22041\_Sample\_011046840, Unigene4830\_Sample\_011046840, Unigene21911\_Sample\_011046840, Unigene9326\_Sample\_011046840, Unigene6747\_Sample\_011046840, Unigene22988\_Sample\_011046840, Unigene3983\_Sample\_011046840, Unigene12913\_Sample\_011046840, Unigene34035\_Sample\_011046840, Unigene36028\_Sample\_011046840, Unigene28548\_Sample\_011046840, Unigene25830\_Sample\_011046840, Unigene16538\_Sample\_011046840, Unigene19915\_Sample\_011046840, Unigene39197\_Sample\_011046840, Unigene38605\_Sample\_011046840, Unigene31274\_Sample\_011046840, Unigene12101\_Sample\_011046840, Unigene4094\_Sample\_011046840, Unigene22810\_Sample\_011046840, Unigene14350\_Sample\_011046840, Unigene40478\_Sample\_011046840, Unigene6665\_Sample\_011046840, Unigene37582\_Sample\_011046840, Unigene36893\_Sample\_011046840, Unigene23457\_Sample\_011046840, Unigene41939\_Sample\_011046840, Unigene27300\_Sample\_011046840, Unigene27232\_Sample\_011046840, Unigene880\_Sample\_011046840, Unigene18239\_Sample\_011046840, Unigene4578\_Sample\_011046840, Unigene43324\_Sample\_011046840, Unigene40406\_Sample\_011046840, Unigene5888\_Sample\_011046840, Unigene24953\_Sample\_011046840, Unigene18001\_Sample\_011046840, Unigene12328\_Sample\_011046840, Unigene33106\_Sample\_011046840, Unigene41412\_Sample\_011046840, Unigene42453\_Sample\_011046840, Unigene42971\_Sample\_011046840, Unigene42395\_Sample\_011046840, Unigene39502\_Sample\_011046840, Unigene6724\_Sample\_011046840, Unigene28730\_Sample\_011046840, Unigene35033\_Sample\_011046840, Unigene34460\_Sample\_011046840, Unigene41357\_Sample\_011046840, Unigene41218\_Sample\_011046840, Unigene20251\_Sample\_011046840, Unigene43537\_Sample\_011046840, Unigene19832\_Sample\_011046840, Unigene27721\_Sample\_011046840, Unigene41048\_Sample\_011046840, Unigene41135\_Sample\_011046840, Unigene22924\_Sample\_011046840, Unigene4498\_Sample\_011046840, Unigene38847\_Sample\_011046840, Unigene26711\_Sample\_011046840, Unigene37389\_Sample\_011046840, Unigene3415\_Sample\_011046840, Unigene32337\_Sample\_011046840, Unigene5388\_Sample\_011046840, Unigene42724\_Sample\_011046840, Unigene30184\_Sample\_011046840, Unigene25515\_Sample\_011046840, Unigene43261\_Sample\_011046840, Unigene9112\_Sample\_011046840, Unigene8219\_Sample\_011046840, Unigene41425\_Sample\_011046840, Unigene18719\_Sample\_011046840, Unigene43039\_Sample\_011046840, Unigene21949\_Sample\_011046840, Unigene976\_Sample\_011046840, Unigene11948\_Sample\_011046840, Unigene42609\_Sample\_011046840, Unigene17567\_Sample\_011046840, Unigene37502\_Sample\_011046840, Unigene42125\_Sample\_011046840, Unigene30343\_Sample\_011046840, Unigene38965\_Sample\_011046840, Unigene31553\_Sample\_011046840, Unigene31038\_Sample\_011046840, Unigene29171\_Sample\_011046840, Unigene41743\_Sample\_011046840, Unigene24814\_Sample\_011046840, Unigene42671\_Sample\_011046840, Unigene37931\_Sample\_011046840, Unigene41759\_Sample\_011046840, Unigene26324\_Sample\_011046840, Unigene865\_Sample\_011046840, Unigene20161\_Sample\_011046840, Unigene42374\_Sample\_011046840, Unigene23958\_Sample\_011046840, Unigene39024\_Sample\_011046840, Unigene40141\_Sample\_011046840, Unigene37680\_Sample\_011046840, Unigene40045\_Sample\_011046840, Unigene25202\_Sample\_011046840, Unigene41161\_Sample\_011046840, Unigene34519\_Sample\_011046840, Unigene1151\_Sample\_011046840, Unigene21061\_Sample\_011046840, Unigene38222\_Sample\_011046840, Unigene35917\_Sample\_011046840, Unigene29051\_Sample\_011046840, Unigene522\_Sample\_011046840, Unigene42301\_Sample\_011046840, Unigene15944\_Sample\_011046840, Unigene2457\_Sample\_011046840, Unigene18087\_Sample\_011046840, Unigene43099\_Sample\_011046840, Unigene1344\_Sample\_011046840, Unigene42759\_Sample\_011046840, Unigene40217\_Sample\_011046840, Unigene2592\_Sample\_011046840, Unigene21362\_Sample\_011046840, Unigene35200\_Sample\_011046840, Unigene41500\_Sample\_011046840, Unigene13035\_Sample\_011046840, Unigene43379\_Sample\_011046840, Unigene35913\_Sample\_011046840, Unigene32333\_Sample\_011046840, Unigene32679\_Sample\_011046840, Unigene40216\_Sample\_011046840, Unigene41431\_Sample\_011046840, Unigene42859\_Sample\_011046840, Unigene30600\_Sample\_011046840, Unigene37153\_Sample\_011046840, Unigene19499\_Sample\_011046840, Unigene7625\_Sample\_011046840, Unigene18272\_Sample\_011046840, Unigene32197\_Sample\_011046840, Unigene32957\_Sample\_011046840, Unigene38737\_Sample\_011046840, Unigene33680\_Sample\_011046840, Unigene35688\_Sample\_011046840, Unigene37178\_Sample\_011046840, Unigene39096\_Sample\_011046840, Unigene23285\_Sample\_011046840, Unigene25343\_Sample\_011046840, Unigene39752\_Sample\_011046840, Unigene23186\_Sample\_011046840, Unigene2780\_Sample\_011046840, Unigene30568\_Sample\_011046840, Unigene43606\_Sample\_011046840, Unigene38853\_Sample\_011046840, Unigene41263\_Sample\_011046840, Unigene18600\_Sample\_011046840, Unigene7873\_Sample\_011046840, Unigene33171\_Sample\_011046840, Unigene14498\_Sample\_011046840, Unigene42807\_Sample\_011046840, Unigene32767\_Sample\_011046840, Unigene42388\_Sample\_011046840, Unigene21040\_Sample\_011046840, Unigene30889\_Sample\_011046840, Unigene37714\_Sample\_011046840, Unigene19369\_Sample\_011046840, Unigene37214\_Sample\_011046840, Unigene35063\_Sample\_011046840, Unigene2861\_Sample\_011046840, Unigene41074\_Sample\_011046840, Unigene4937\_Sample\_011046840, Unigene3914\_Sample\_011046840, Unigene16153\_Sample\_011046840, Unigene5310\_Sample\_011046840, Unigene38690\_Sample\_011046840, Unigene7028\_Sample\_011046840, Unigene35755\_Sample\_011046840, Unigene8313\_Sample\_011046840, Unigene32292\_Sample\_011046840, Unigene39014\_Sample\_011046840, Unigene10043\_Sample\_011046840, Unigene39286\_Sample\_011046840, Unigene1311\_Sample\_011046840, Unigene26740\_Sample\_011046840, Unigene40853\_Sample\_011046840, Unigene37870\_Sample\_011046840, Unigene40018\_Sample\_011046840, Unigene40545\_Sample\_011046840, Unigene30817\_Sample\_011046840, Unigene31401\_Sample\_011046840, Unigene41742\_Sample\_011046840, Unigene28429\_Sample\_011046840, Unigene40523\_Sample\_011046840, Unigene33611\_Sample\_011046840, Unigene32108\_Sample\_011046840, Unigene41631\_Sample\_011046840, Unigene30934\_Sample\_011046840, Unigene3206\_Sample\_011046840, Unigene39687\_Sample\_011046840, Unigene38386\_Sample\_011046840, Unigene7097\_Sample\_011046840, Unigene1884\_Sample\_011046840, Unigene34458\_Sample\_011046840, Unigene7405\_Sample\_011046840, Unigene31007\_Sample\_011046840, Unigene22051\_Sample\_011046840, Unigene40876\_Sample\_011046840, Unigene19732\_Sample\_011046840, Unigene35650\_Sample\_011046840, Unigene39854\_Sample\_011046840, Unigene22177\_Sample\_011046840, Unigene38624\_Sample\_011046840, Unigene41826\_Sample\_011046840, Unigene33927\_Sample\_011046840, Unigene6653\_Sample\_011046840, Unigene32146\_Sample\_011046840, Unigene37462\_Sample\_011046840, Unigene10704\_Sample\_011046840, Unigene7521\_Sample\_011046840, Unigene36759\_Sample\_011046840, Unigene27239\_Sample\_011046840, Unigene43135\_Sample\_011046840, Unigene10229\_Sample\_011046840, Unigene34663\_Sample\_011046840, Unigene36973\_Sample\_011046840, Unigene34833\_Sample\_011046840, Unigene37242\_Sample\_011046840, Unigene39749\_Sample\_011046840, Unigene30058\_Sample\_011046840, Unigene32101\_Sample\_011046840, Unigene40324\_Sample\_011046840, Unigene42897\_Sample\_011046840, Unigene39121\_Sample\_011046840, Unigene5383\_Sample\_011046840, Unigene42927\_Sample\_011046840, Unigene20798\_Sample\_011046840, Unigene36773\_Sample\_011046840, Unigene30089\_Sample\_011046840, Unigene6753\_Sample\_011046840, Unigene14139\_Sample\_011046840, Unigene8006\_Sample\_011046840, Unigene40979\_Sample\_011046840, Unigene40427\_Sample\_011046840, Unigene34604\_Sample\_011046840, Unigene2829\_Sample\_011046840, Unigene33029\_Sample\_011046840, Unigene8255\_Sample\_011046840, Unigene27359\_Sample\_011046840, Unigene41487\_Sample\_011046840, Unigene32157\_Sample\_011046840, Unigene42429\_Sample\_011046840, Unigene21710\_Sample\_011046840, Unigene40648\_Sample\_011046840, Unigene42157\_Sample\_011046840, Unigene43475\_Sample\_011046840, Unigene1561\_Sample\_011046840, Unigene5909\_Sample\_011046840, Unigene30384\_Sample\_011046840, Unigene30201\_Sample\_011046840, Unigene16873\_Sample\_011046840, Unigene38074\_Sample\_011046840, Unigene36820\_Sample\_011046840, Unigene3753\_Sample\_011046840, Unigene33714\_Sample\_011046840, Unigene5564\_Sample\_011046840, Unigene43304\_Sample\_011046840, Unigene29050\_Sample\_011046840, Unigene15148\_Sample\_011046840, Unigene34885\_Sample\_011046840, Unigene27478\_Sample\_011046840, Unigene34174\_Sample\_011046840, Unigene4032\_Sample\_011046840, Unigene41747\_Sample\_011046840, Unigene42706\_Sample\_011046840, Unigene2963\_Sample\_011046840, Unigene39263\_Sample\_011046840, Unigene29328\_Sample\_011046840, Unigene3538\_Sample\_011046840, Unigene33197\_Sample\_011046840, Unigene32426\_Sample\_011046840, Unigene43045\_Sample\_011046840, Unigene7925\_Sample\_011046840, Unigene32304\_Sample\_011046840, Unigene8203\_Sample\_011046840, Unigene1573\_Sample\_011046840, Unigene38680\_Sample\_011046840, Unigene32944\_Sample\_011046840, Unigene33852\_Sample\_011046840, Unigene38501\_Sample\_011046840, Unigene10921\_Sample\_011046840, Unigene42557\_Sample\_011046840, Unigene15163\_Sample\_011046840, Unigene37223\_Sample\_011046840, Unigene25628\_Sample\_011046840, Unigene23926\_Sample\_011046840, Unigene16252\_Sample\_011046840, Unigene6845\_Sample\_011046840, Unigene39264\_Sample\_011046840, Unigene40761\_Sample\_011046840, Unigene42169\_Sample\_011046840, Unigene40541\_Sample\_011046840, Unigene37939\_Sample\_011046840, Unigene41399\_Sample\_011046840, Unigene40590\_Sample\_011046840, Unigene42075\_Sample\_011046840, Unigene42241\_Sample\_011046840, Unigene21120\_Sample\_011046840, Unigene35690\_Sample\_011046840, Unigene12818\_Sample\_011046840, Unigene40961\_Sample\_011046840, Unigene33706\_Sample\_011046840, Unigene5464\_Sample\_011046840, Unigene29348\_Sample\_011046840, Unigene42276\_Sample\_011046840, Unigene28965\_Sample\_011046840, Unigene25584\_Sample\_011046840, Unigene40068\_Sample\_011046840, Unigene38031\_Sample\_011046840, Unigene35632\_Sample\_011046840, Unigene37162\_Sample\_011046840, Unigene2808\_Sample\_011046840, Unigene39700\_Sample\_011046840, Unigene34416\_Sample\_011046840, Unigene8583\_Sample\_011046840, Unigene972\_Sample\_011046840, Unigene41191\_Sample\_011046840, Unigene26022\_Sample\_011046840, Unigene38774\_Sample\_011046840, Unigene36461\_Sample\_011046840, Unigene38393\_Sample\_011046840, Unigene21226\_Sample\_011046840, Unigene38071\_Sample\_011046840, Unigene33115\_Sample\_011046840, Unigene31663\_Sample\_011046840, Unigene38550\_Sample\_011046840, Unigene41037\_Sample\_011046840, Unigene11726\_Sample\_011046840, Unigene14072\_Sample\_011046840, Unigene34844\_Sample\_011046840, Unigene5034\_Sample\_011046840, Unigene38761\_Sample\_011046840, Unigene38416\_Sample\_011046840, Unigene19411\_Sample\_011046840, Unigene32050\_Sample\_011046840, Unigene39949\_Sample\_011046840, Unigene37483\_Sample\_011046840, Unigene15222\_Sample\_011046840, Unigene41600\_Sample\_011046840, Unigene6401\_Sample\_011046840, Unigene37785\_Sample\_011046840, Unigene38523\_Sample\_011046840, Unigene34241\_Sample\_011046840, Unigene29391\_Sample\_011046840, Unigene37310\_Sample\_011046840, Unigene1085\_Sample\_011046840, Unigene26624\_Sample\_011046840, Unigene43441\_Sample\_011046840, Unigene29269\_Sample\_011046840, Unigene14018\_Sample\_011046840, Unigene17267\_Sample\_011046840, Unigene31672\_Sample\_011046840, Unigene33597\_Sample\_011046840, Unigene14882\_Sample\_011046840, Unigene13721\_Sample\_011046840, Unigene11049\_Sample\_011046840, Unigene42104\_Sample\_011046840, Unigene43470\_Sample\_011046840, Unigene33915\_Sample\_011046840, Unigene24938\_Sample\_011046840, Unigene36655\_Sample\_011046840, Unigene453\_Sample\_011046840, Unigene42187\_Sample\_011046840, Unigene35644\_Sample\_011046840, Unigene37629\_Sample\_011046840, Unigene36166\_Sample\_011046840, Unigene40915\_Sample\_011046840, Unigene8290\_Sample\_011046840, Unigene40868\_Sample\_011046840, Unigene40283\_Sample\_011046840, Unigene42264\_Sample\_011046840, Unigene991\_Sample\_011046840, Unigene38314\_Sample\_011046840, Unigene42160\_Sample\_011046840, Unigene42892\_Sample\_011046840, Unigene39443\_Sample\_011046840, Unigene37361\_Sample\_011046840, Unigene32227\_Sample\_011046840, Unigene24453\_Sample\_011046840, Unigene36896\_Sample\_011046840, Unigene28006\_Sample\_011046840, Unigene19793\_Sample\_011046840, Unigene15047\_Sample\_011046840, Unigene8223\_Sample\_011046840, Unigene42923\_Sample\_011046840, Unigene7174\_Sample\_011046840, Unigene30381\_Sample\_011046840, Unigene31928\_Sample\_011046840, Unigene25793\_Sample\_011046840, Unigene36522\_Sample\_011046840, Unigene15213\_Sample\_011046840, Unigene23572\_Sample\_011046840, Unigene35864\_Sample\_011046840, Unigene36191\_Sample\_011046840, Unigene21670\_Sample\_011046840, Unigene36589\_Sample\_011046840, Unigene42744\_Sample\_011046840, Unigene25557\_Sample\_011046840, Unigene39049\_Sample\_011046840, Unigene42280\_Sample\_011046840, Unigene33136\_Sample\_011046840, Unigene8045\_Sample\_011046840, Unigene36492\_Sample\_011046840, Unigene9944\_Sample\_011046840, Unigene6129\_Sample\_011046840, Unigene33969\_Sample\_011046840, Unigene29982\_Sample\_011046840, Unigene30879\_Sample\_011046840, Unigene16314\_Sample\_011046840, Unigene39491\_Sample\_011046840, Unigene22159\_Sample\_011046840, Unigene30576\_Sample\_011046840, Unigene23397\_Sample\_011046840, Unigene24277\_Sample\_011046840, Unigene26297\_Sample\_011046840, Unigene33275\_Sample\_011046840, Unigene8181\_Sample\_011046840, Unigene37274\_Sample\_011046840, Unigene6641\_Sample\_011046840, Unigene17700\_Sample\_011046840, Unigene24914\_Sample\_011046840, Unigene40859\_Sample\_011046840, Unigene39265\_Sample\_011046840, Unigene35677\_Sample\_011046840, Unigene4464\_Sample\_011046840, Unigene9962\_Sample\_011046840, Unigene39237\_Sample\_011046840, Unigene41136\_Sample\_011046840, Unigene7402\_Sample\_011046840, Unigene23730\_Sample\_011046840, Unigene31057\_Sample\_011046840, Unigene43387\_Sample\_011046840, Unigene37570\_Sample\_011046840, Unigene31458\_Sample\_011046840, Unigene41268\_Sample\_011046840, Unigene36089\_Sample\_011046840, Unigene30456\_Sample\_011046840, Unigene41678\_Sample\_011046840, Unigene35699\_Sample\_011046840, Unigene27865\_Sample\_011046840, Unigene36246\_Sample\_011046840, Unigene36967\_Sample\_011046840, Unigene34997\_Sample\_011046840, Unigene41128\_Sample\_011046840, Unigene1619\_Sample\_011046840, Unigene10148\_Sample\_011046840, Unigene489\_Sample\_011046840, Unigene7785\_Sample\_011046840, Unigene37688\_Sample\_011046840, Unigene18985\_Sample\_011046840, Unigene40020\_Sample\_011046840, Unigene41142\_Sample\_011046840, Unigene3897\_Sample\_011046840, Unigene25605\_Sample\_011046840, Unigene5969\_Sample\_011046840, Unigene38025\_Sample\_011046840, Unigene19052\_Sample\_011046840, Unigene22635\_Sample\_011046840, Unigene2383\_Sample\_011046840, Unigene27993\_Sample\_011046840, Unigene39020\_Sample\_011046840, Unigene7818\_Sample\_011046840, Unigene8465\_Sample\_011046840, Unigene24917\_Sample\_011046840, Unigene36737\_Sample\_011046840, Unigene32843\_Sample\_011046840, Unigene5257\_Sample\_011046840, Unigene8975\_Sample\_011046840, Unigene32360\_Sample\_011046840, Unigene10202\_Sample\_011046840, Unigene35359\_Sample\_011046840, Unigene42224\_Sample\_011046840, Unigene38351\_Sample\_011046840, Unigene16893\_Sample\_011046840, Unigene29994\_Sample\_011046840, Unigene28620\_Sample\_011046840, Unigene37826\_Sample\_011046840, Unigene16218\_Sample\_011046840, Unigene37581\_Sample\_011046840, Unigene36659\_Sample\_011046840, Unigene23761\_Sample\_011046840, Unigene41424\_Sample\_011046840, Unigene33024\_Sample\_011046840, Unigene36819\_Sample\_011046840, Unigene40437\_Sample\_011046840, Unigene29267\_Sample\_011046840, Unigene40661\_Sample\_011046840, Unigene14485\_Sample\_011046840, Unigene37664\_Sample\_011046840, Unigene19235\_Sample\_011046840, Unigene27831\_Sample\_011046840, Unigene16385\_Sample\_011046840, Unigene38248\_Sample\_011046840, Unigene40727\_Sample\_011046840, Unigene20304\_Sample\_011046840, Unigene13223\_Sample\_011046840, Unigene22789\_Sample\_011046840, Unigene29294\_Sample\_011046840, Unigene3713\_Sample\_011046840, Unigene32431\_Sample\_011046840, Unigene33393\_Sample\_011046840, Unigene21033\_Sample\_011046840, Unigene42484\_Sample\_011046840, Unigene40993\_Sample\_011046840, Unigene39796\_Sample\_011046840, Unigene4692\_Sample\_011046840, Unigene7287\_Sample\_011046840, Unigene5245\_Sample\_011046840, Unigene33196\_Sample\_011046840, Unigene6912\_Sample\_011046840, Unigene27570\_Sample\_011046840, Unigene40222\_Sample\_011046840, Unigene7760\_Sample\_011046840, Unigene4955\_Sample\_011046840, Unigene36846\_Sample\_011046840, Unigene41068\_Sample\_011046840, Unigene26550\_Sample\_011046840, Unigene26453\_Sample\_011046840, Unigene32803\_Sample\_011046840, Unigene25853\_Sample\_011046840, Unigene35874\_Sample\_011046840, Unigene38065\_Sample\_011046840, Unigene41941\_Sample\_011046840, Unigene43009\_Sample\_011046840, Unigene41616\_Sample\_011046840, Unigene40052\_Sample\_011046840, Unigene34927\_Sample\_011046840, Unigene40368\_Sample\_011046840, Unigene10913\_Sample\_011046840, Unigene28253\_Sample\_011046840, Unigene26424\_Sample\_011046840, Unigene22186\_Sample\_011046840, Unigene41177\_Sample\_011046840, Unigene14590\_Sample\_011046840, Unigene30365\_Sample\_011046840, Unigene12728\_Sample\_011046840, Unigene7589\_Sample\_011046840, Unigene31564\_Sample\_011046840, Unigene3029\_Sample\_011046840, Unigene32515\_Sample\_011046840, Unigene473\_Sample\_011046840, Unigene20351\_Sample\_011046840, Unigene12616\_Sample\_011046840, Unigene31408\_Sample\_011046840, Unigene40614\_Sample\_011046840, Unigene42951\_Sample\_011046840, Unigene17674\_Sample\_011046840, Unigene4307\_Sample\_011046840, Unigene38150\_Sample\_011046840, Unigene40026\_Sample\_011046840, Unigene33692\_Sample\_011046840, Unigene42407\_Sample\_011046840, Unigene40573\_Sample\_011046840, Unigene3098\_Sample\_011046840, Unigene15855\_Sample\_011046840, Unigene38116\_Sample\_011046840, Unigene443\_Sample\_011046840, Unigene39360\_Sample\_011046840, Unigene2342\_Sample\_011046840, Unigene3664\_Sample\_011046840, Unigene40697\_Sample\_011046840, Unigene39420\_Sample\_011046840, Unigene24457\_Sample\_011046840, Unigene700\_Sample\_011046840, Unigene38370\_Sample\_011046840, Unigene37533\_Sample\_011046840, Unigene30813\_Sample\_011046840, Unigene42620\_Sample\_011046840, Unigene22254\_Sample\_011046840, Unigene13304\_Sample\_011046840, Unigene28405\_Sample\_011046840, Unigene32263\_Sample\_011046840, Unigene42627\_Sample\_011046840, Unigene37020\_Sample\_011046840, Unigene42997\_Sample\_011046840, Unigene23433\_Sample\_011046840, Unigene17432\_Sample\_011046840, Unigene38844\_Sample\_011046840, Unigene37883\_Sample\_011046840, Unigene35646\_Sample\_011046840, Unigene8665\_Sample\_011046840, Unigene35835\_Sample\_011046840, Unigene42131\_Sample\_011046840, Unigene39107\_Sample\_011046840, Unigene1399\_Sample\_011046840, Unigene27153\_Sample\_011046840, Unigene24223\_Sample\_011046840, Unigene7363\_Sample\_011046840, Unigene31743\_Sample\_011046840, Unigene6293\_Sample\_011046840, Unigene36580\_Sample\_011046840, Unigene25822\_Sample\_011046840, Unigene31431\_Sample\_011046840, Unigene24719\_Sample\_011046840, Unigene52\_Sample\_011046840, Unigene30604\_Sample\_011046840, Unigene39413\_Sample\_011046840, Unigene41588\_Sample\_011046840, Unigene43176\_Sample\_011046840, Unigene28437\_Sample\_011046840, Unigene26884\_Sample\_011046840, Unigene40410\_Sample\_011046840, Unigene31033\_Sample\_011046840, Unigene27425\_Sample\_011046840, Unigene22528\_Sample\_011046840, Unigene11853\_Sample\_011046840, Unigene7819\_Sample\_011046840, Unigene30784\_Sample\_011046840, Unigene34657\_Sample\_011046840, Unigene42286\_Sample\_011046840, Unigene15696\_Sample\_011046840, Unigene29131\_Sample\_011046840, Unigene33772\_Sample\_011046840, Unigene42227\_Sample\_011046840, Unigene42813\_Sample\_011046840, Unigene40918\_Sample\_011046840, Unigene26969\_Sample\_011046840, Unigene5227\_Sample\_011046840, Unigene37937\_Sample\_011046840, Unigene37229\_Sample\_011046840, Unigene19320\_Sample\_011046840, Unigene25223\_Sample\_011046840, Unigene32118\_Sample\_011046840, Unigene2929\_Sample\_011046840, Unigene37721\_Sample\_011046840, Unigene30611\_Sample\_011046840, Unigene13770\_Sample\_011046840, Unigene26260\_Sample\_011046840, Unigene40565\_Sample\_011046840, Unigene28467\_Sample\_011046840, Unigene24618\_Sample\_011046840, Unigene36400\_Sample\_011046840, Unigene43084\_Sample\_011046840, Unigene24149\_Sample\_011046840, Unigene1814\_Sample\_011046840, Unigene30176\_Sample\_011046840, Unigene41712\_Sample\_011046840, Unigene41078\_Sample\_011046840, Unigene23155\_Sample\_011046840, Unigene28822\_Sample\_011046840, Unigene22122\_Sample\_011046840, Unigene32717\_Sample\_011046840, Unigene19982\_Sample\_011046840, Unigene34464\_Sample\_011046840, Unigene1404\_Sample\_011046840, Unigene6426\_Sample\_011046840, Unigene13980\_Sample\_011046840, Unigene557\_Sample\_011046840, Unigene29367\_Sample\_011046840, Unigene23570\_Sample\_011046840, Unigene31658\_Sample\_011046840, Unigene39522\_Sample\_011046840, Unigene7891\_Sample\_011046840, Unigene31412\_Sample\_011046840, Unigene12130\_Sample\_011046840, Unigene4723\_Sample\_011046840, Unigene29352\_Sample\_011046840, Unigene30691\_Sample\_011046840, Unigene43414\_Sample\_011046840, Unigene7503\_Sample\_011046840, Unigene6926\_Sample\_011046840, Unigene43021\_Sample\_011046840, Unigene27887\_Sample\_011046840, Unigene41062\_Sample\_011046840, Unigene36349\_Sample\_011046840, Unigene38630\_Sample\_011046840, Unigene26953\_Sample\_011046840, Unigene23108\_Sample\_011046840, Unigene39865\_Sample\_011046840, Unigene40273\_Sample\_011046840, Unigene28061\_Sample\_011046840, Unigene34121\_Sample\_011046840, Unigene32375\_Sample\_011046840, Unigene40057\_Sample\_011046840, Unigene34281\_Sample\_011046840, Unigene39556\_Sample\_011046840, Unigene40411\_Sample\_011046840, Unigene43249\_Sample\_011046840, Unigene23390\_Sample\_011046840, Unigene34655\_Sample\_011046840, Unigene34703\_Sample\_011046840, Unigene32984\_Sample\_011046840, Unigene25956\_Sample\_011046840, Unigene5491\_Sample\_011046840, Unigene7291\_Sample\_011046840, Unigene31889\_Sample\_011046840, Unigene21072\_Sample\_011046840, Unigene35505\_Sample\_011046840, Unigene33626\_Sample\_011046840, Unigene3345\_Sample\_011046840, Unigene16778\_Sample\_011046840, Unigene38559\_Sample\_011046840, Unigene34658\_Sample\_011046840, Unigene34653\_Sample\_011046840, Unigene2822\_Sample\_011046840, Unigene18790\_Sample\_011046840, Unigene30185\_Sample\_011046840, Unigene42517\_Sample\_011046840, Unigene4332\_Sample\_011046840, Unigene20838\_Sample\_011046840, Unigene7868\_Sample\_011046840, Unigene35883\_Sample\_011046840, Unigene37041\_Sample\_011046840, Unigene42114\_Sample\_011046840, Unigene23630\_Sample\_011046840, Unigene41653\_Sample\_011046840, Unigene30946\_Sample\_011046840, Unigene36203\_Sample\_011046840, Unigene30963\_Sample\_011046840, Unigene26759\_Sample\_011046840, Unigene35002\_Sample\_011046840, Unigene42943\_Sample\_011046840, Unigene22644\_Sample\_011046840, Unigene27654\_Sample\_011046840, Unigene17719\_Sample\_011046840, Unigene10241\_Sample\_011046840, Unigene43251\_Sample\_011046840, Unigene42171\_Sample\_011046840, Unigene22998\_Sample\_011046840, Unigene23890\_Sample\_011046840, Unigene35348\_Sample\_011046840, Unigene41125\_Sample\_011046840, Unigene39117\_Sample\_011046840, Unigene36935\_Sample\_011046840, Unigene36917\_Sample\_011046840, Unigene13452\_Sample\_011046840, Unigene41204\_Sample\_011046840, Unigene36900\_Sample\_011046840, Unigene29654\_Sample\_011046840, Unigene37187\_Sample\_011046840, Unigene33682\_Sample\_011046840, Unigene14568\_Sample\_011046840, Unigene41887\_Sample\_011046840, Unigene25041\_Sample\_011046840, Unigene38780\_Sample\_011046840, Unigene43555\_Sample\_011046840, Unigene22688\_Sample\_011046840, Unigene16247\_Sample\_011046840, Unigene32285\_Sample\_011046840, Unigene40391\_Sample\_011046840, Unigene28898\_Sample\_011046840, Unigene1089\_Sample\_011046840, Unigene39732\_Sample\_011046840, Unigene7134\_Sample\_011046840, Unigene32923\_Sample\_011046840, Unigene8142\_Sample\_011046840, Unigene20821\_Sample\_011046840, Unigene19481\_Sample\_011046840, Unigene36458\_Sample\_011046840, Unigene5846\_Sample\_011046840, Unigene36991\_Sample\_011046840, Unigene14274\_Sample\_011046840, Unigene28668\_Sample\_011046840, Unigene43275\_Sample\_011046840, Unigene28414\_Sample\_011046840, Unigene6878\_Sample\_011046840, Unigene37126\_Sample\_011046840, Unigene36326\_Sample\_011046840, Unigene21804\_Sample\_011046840, Unigene37908\_Sample\_011046840, Unigene40449\_Sample\_011046840, Unigene29198\_Sample\_011046840, Unigene30757\_Sample\_011046840, Unigene32056\_Sample\_011046840, Unigene43276\_Sample\_011046840, Unigene39406\_Sample\_011046840, Unigene25951\_Sample\_011046840, Unigene36621\_Sample\_011046840, Unigene27601\_Sample\_011046840, Unigene23176\_Sample\_011046840, Unigene14188\_Sample\_011046840, Unigene38001\_Sample\_011046840, Unigene19274\_Sample\_011046840, Unigene42387\_Sample\_011046840, Unigene7407\_Sample\_011046840, Unigene38261\_Sample\_011046840, Unigene40049\_Sample\_011046840, Unigene23126\_Sample\_011046840, Unigene30862\_Sample\_011046840, Unigene7533\_Sample\_011046840, Unigene42369\_Sample\_011046840, Unigene15803\_Sample\_011046840, Unigene4082\_Sample\_011046840, Unigene8152\_Sample\_011046840, Unigene38115\_Sample\_011046840, Unigene36462\_Sample\_011046840, Unigene33808\_Sample\_011046840, Unigene34932\_Sample\_011046840, Unigene42090\_Sample\_011046840, Unigene42430\_Sample\_011046840, Unigene19425\_Sample\_011046840, Unigene29121\_Sample\_011046840, Unigene26987\_Sample\_011046840, Unigene30393\_Sample\_011046840, Unigene27428\_Sample\_011046840, Unigene19859\_Sample\_011046840, Unigene28282\_Sample\_011046840, Unigene40806\_Sample\_011046840, Unigene33616\_Sample\_011046840, Unigene29480\_Sample\_011046840, Unigene42030\_Sample\_011046840, Unigene20517\_Sample\_011046840, Unigene14012\_Sample\_011046840, Unigene20681\_Sample\_011046840, Unigene39011\_Sample\_011046840, Unigene28316\_Sample\_011046840, Unigene16978\_Sample\_011046840, Unigene6139\_Sample\_011046840, Unigene2234\_Sample\_011046840, Unigene35314\_Sample\_011046840, Unigene19370\_Sample\_011046840, Unigene39978\_Sample\_011046840, Unigene38021\_Sample\_011046840, Unigene34684\_Sample\_011046840, Unigene28200\_Sample\_011046840, Unigene35485\_Sample\_011046840, Unigene26967\_Sample\_011046840, Unigene34960\_Sample\_011046840, Unigene36437\_Sample\_011046840, Unigene6495\_Sample\_011046840, Unigene23988\_Sample\_011046840, Unigene38381\_Sample\_011046840, Unigene22660\_Sample\_011046840, Unigene30195\_Sample\_011046840, Unigene5190\_Sample\_011046840, Unigene39647\_Sample\_011046840, Unigene1246\_Sample\_011046840, Unigene39272\_Sample\_011046840, Unigene41702\_Sample\_011046840, Unigene30851\_Sample\_011046840, Unigene21295\_Sample\_011046840, Unigene35151\_Sample\_011046840, Unigene12570\_Sample\_011046840, Unigene27922\_Sample\_011046840, Unigene26575\_Sample\_011046840, Unigene24067\_Sample\_011046840, Unigene36910\_Sample\_011046840, Unigene42047\_Sample\_011046840, Unigene18403\_Sample\_011046840, Unigene15252\_Sample\_011046840, Unigene24706\_Sample\_011046840, Unigene40705\_Sample\_011046840, Unigene15165\_Sample\_011046840, Unigene38478\_Sample\_011046840, Unigene41361\_Sample\_011046840, Unigene28163\_Sample\_011046840, Unigene18649\_Sample\_011046840, Unigene38571\_Sample\_011046840, Unigene43591\_Sample\_011046840, Unigene24532\_Sample\_011046840, Unigene15446\_Sample\_011046840, Unigene6254\_Sample\_011046840, Unigene35118\_Sample\_011046840, Unigene36251\_Sample\_011046840, Unigene42209\_Sample\_011046840, Unigene38245\_Sample\_011046840, Unigene24921\_Sample\_011046840, Unigene2615\_Sample\_011046840, Unigene39540\_Sample\_011046840, Unigene35939\_Sample\_011046840, Unigene38775\_Sample\_011046840, Unigene4842\_Sample\_011046840, Unigene15455\_Sample\_011046840, Unigene20828\_Sample\_011046840, Unigene37077\_Sample\_011046840, Unigene31322\_Sample\_011046840, Unigene24172\_Sample\_011046840, Unigene25348\_Sample\_011046840, Unigene37535\_Sample\_011046840, Unigene13078\_Sample\_011046840, Unigene3144\_Sample\_011046840, Unigene23304\_Sample\_011046840, Unigene43464\_Sample\_011046840, Unigene42786\_Sample\_011046840, Unigene8165\_Sample\_011046840, Unigene37362\_Sample\_011046840, Unigene35015\_Sample\_011046840, Unigene35395\_Sample\_011046840, Unigene13399\_Sample\_011046840, Unigene32907\_Sample\_011046840, Unigene38121\_Sample\_011046840, Unigene13285\_Sample\_011046840, Unigene29658\_Sample\_011046840, Unigene32316\_Sample\_011046840, Unigene32939\_Sample\_011046840, Unigene22061\_Sample\_011046840, Unigene23873\_Sample\_011046840, Unigene35564\_Sample\_011046840, Unigene35642\_Sample\_011046840, Unigene5081\_Sample\_011046840, Unigene37015\_Sample\_011046840, Unigene4025\_Sample\_011046840, Unigene12150\_Sample\_011046840, Unigene10106\_Sample\_011046840, Unigene38091\_Sample\_011046840, Unigene31822\_Sample\_011046840, Unigene41582\_Sample\_011046840, Unigene23401\_Sample\_011046840, Unigene43223\_Sample\_011046840, Unigene42903\_Sample\_011046840, Unigene23636\_Sample\_011046840, Unigene29411\_Sample\_011046840, Unigene4076\_Sample\_011046840, Unigene40284\_Sample\_011046840, Unigene25873\_Sample\_011046840, Unigene12776\_Sample\_011046840, Unigene29626\_Sample\_011046840, Unigene17079\_Sample\_011046840, Unigene2127\_Sample\_011046840, Unigene33260\_Sample\_011046840, Unigene7943\_Sample\_011046840, Unigene32288\_Sample\_011046840, Unigene7806\_Sample\_011046840, Unigene40465\_Sample\_011046840, Unigene1224\_Sample\_011046840, Unigene26940\_Sample\_011046840, Unigene42459\_Sample\_011046840, Unigene43153\_Sample\_011046840, Unigene35284\_Sample\_011046840, Unigene30608\_Sample\_011046840, Unigene40223\_Sample\_011046840, Unigene31862\_Sample\_011046840, Unigene34227\_Sample\_011046840, Unigene3279\_Sample\_011046840, Unigene39179\_Sample\_011046840, Unigene3934\_Sample\_011046840, Unigene40087\_Sample\_011046840, Unigene42127\_Sample\_011046840, Unigene41531\_Sample\_011046840, Unigene31248\_Sample\_011046840, Unigene36075\_Sample\_011046840, Unigene26432\_Sample\_011046840, Unigene18248\_Sample\_011046840, Unigene40210\_Sample\_011046840, Unigene35834\_Sample\_011046840, Unigene31503\_Sample\_011046840, Unigene39894\_Sample\_011046840, Unigene12033\_Sample\_011046840, Unigene23557\_Sample\_011046840, Unigene35302\_Sample\_011046840, Unigene27260\_Sample\_011046840, Unigene25330\_Sample\_011046840, Unigene37432\_Sample\_011046840, Unigene41034\_Sample\_011046840, Unigene6551\_Sample\_011046840, Unigene38376\_Sample\_011046840, Unigene41339\_Sample\_011046840, Unigene841\_Sample\_011046840, Unigene43346\_Sample\_011046840, Unigene41418\_Sample\_011046840, Unigene36772\_Sample\_011046840, Unigene11410\_Sample\_011046840, Unigene25018\_Sample\_011046840, Unigene42861\_Sample\_011046840, Unigene35797\_Sample\_011046840, Unigene35977\_Sample\_011046840, Unigene18463\_Sample\_011046840, Unigene19271\_Sample\_011046840, Unigene27895\_Sample\_011046840, Unigene17153\_Sample\_011046840, Unigene34652\_Sample\_011046840, Unigene12534\_Sample\_011046840, Unigene25826\_Sample\_011046840, Unigene29316\_Sample\_011046840, Unigene35743\_Sample\_011046840, Unigene12229\_Sample\_011046840, Unigene34059\_Sample\_011046840, Unigene33700\_Sample\_011046840, Unigene32253\_Sample\_011046840, Unigene12171\_Sample\_011046840, Unigene37637\_Sample\_011046840, Unigene22059\_Sample\_011046840, Unigene5098\_Sample\_011046840, Unigene7572\_Sample\_011046840, Unigene36485\_Sample\_011046840, Unigene10383\_Sample\_011046840, Unigene7727\_Sample\_011046840, Unigene30059\_Sample\_011046840, Unigene7391\_Sample\_011046840, Unigene41083\_Sample\_011046840, Unigene29913\_Sample\_011046840, Unigene39200\_Sample\_011046840, Unigene40849\_Sample\_011046840, Unigene37666\_Sample\_011046840, Unigene25292\_Sample\_011046840, Unigene25488\_Sample\_011046840, Unigene30695\_Sample\_011046840, Unigene39829\_Sample\_011046840, Unigene41935\_Sample\_011046840, Unigene32106\_Sample\_011046840, Unigene14473\_Sample\_011046840, Unigene34896\_Sample\_011046840, Unigene23121\_Sample\_011046840, Unigene41164\_Sample\_011046840, Unigene36257\_Sample\_011046840, Unigene38770\_Sample\_011046840, Unigene32268\_Sample\_011046840, Unigene39804\_Sample\_011046840, Unigene39391\_Sample\_011046840, Unigene24375\_Sample\_011046840, Unigene13729\_Sample\_011046840, Unigene41850\_Sample\_011046840, Unigene6860\_Sample\_011046840, Unigene41225\_Sample\_011046840, Unigene40017\_Sample\_011046840, Unigene21487\_Sample\_011046840, Unigene25938\_Sample\_011046840, Unigene33952\_Sample\_011046840, Unigene33427\_Sample\_011046840, Unigene39631\_Sample\_011046840, Unigene25767\_Sample\_011046840, Unigene42768\_Sample\_011046840, Unigene32790\_Sample\_011046840, Unigene41855\_Sample\_011046840, Unigene41007\_Sample\_011046840, Unigene10729\_Sample\_011046840, Unigene24420\_Sample\_011046840, Unigene10318\_Sample\_011046840, Unigene38034\_Sample\_011046840, Unigene41324\_Sample\_011046840, Unigene37439\_Sample\_011046840, Unigene29161\_Sample\_011046840, Unigene39464\_Sample\_011046840, Unigene25595\_Sample\_011046840, Unigene4724\_Sample\_011046840, Unigene43260\_Sample\_011046840, Unigene3510\_Sample\_011046840, Unigene6500\_Sample\_011046840, Unigene14750\_Sample\_011046840, Unigene40503\_Sample\_011046840, Unigene42626\_Sample\_011046840, Unigene21366\_Sample\_011046840, Unigene5335\_Sample\_011046840, Unigene24612\_Sample\_011046840, Unigene8162\_Sample\_011046840, Unigene41966\_Sample\_011046840, Unigene4686\_Sample\_011046840, Unigene41261\_Sample\_011046840, Unigene35610\_Sample\_011046840, Unigene36726\_Sample\_011046840, Unigene4101\_Sample\_011046840, Unigene29660\_Sample\_011046840, Unigene12353\_Sample\_011046840, Unigene41784\_Sample\_011046840, Unigene27261\_Sample\_011046840, Unigene41113\_Sample\_011046840, Unigene40088\_Sample\_011046840, Unigene33984\_Sample\_011046840, Unigene32791\_Sample\_011046840, Unigene37631\_Sample\_011046840, Unigene11943\_Sample\_011046840, Unigene39527\_Sample\_011046840, Unigene40562\_Sample\_011046840, Unigene33954\_Sample\_011046840, Unigene39720\_Sample\_011046840, Unigene29794\_Sample\_011046840, Unigene38919\_Sample\_011046840, Unigene15316\_Sample\_011046840, Unigene39458\_Sample\_011046840, Unigene26807\_Sample\_011046840, Unigene32518\_Sample\_011046840, Unigene38914\_Sample\_011046840, Unigene24898\_Sample\_011046840, Unigene4898\_Sample\_011046840, Unigene19593\_Sample\_011046840, Unigene38495\_Sample\_011046840, Unigene42729\_Sample\_011046840, Unigene42963\_Sample\_011046840, Unigene41810\_Sample\_011046840, Unigene30989\_Sample\_011046840, Unigene31513\_Sample\_011046840, Unigene24411\_Sample\_011046840, Unigene13278\_Sample\_011046840, Unigene30236\_Sample\_011046840, Unigene41870\_Sample\_011046840, Unigene1077\_Sample\_011046840, Unigene14093\_Sample\_011046840, Unigene27305\_Sample\_011046840, Unigene16797\_Sample\_011046840, Unigene30162\_Sample\_011046840, Unigene17730\_Sample\_011046840, Unigene37338\_Sample\_011046840, Unigene28124\_Sample\_011046840, Unigene2498\_Sample\_011046840, Unigene41882\_Sample\_011046840, Unigene37773\_Sample\_011046840, Unigene40686\_Sample\_011046840, Unigene29570\_Sample\_011046840, Unigene26719\_Sample\_011046840, Unigene42930\_Sample\_011046840, Unigene42643\_Sample\_011046840, Unigene43155\_Sample\_011046840, Unigene41637\_Sample\_011046840, Unigene39931\_Sample\_011046840, Unigene42544\_Sample\_011046840, Unigene4520\_Sample\_011046840, Unigene1740\_Sample\_011046840, Unigene28339\_Sample\_011046840, Unigene42230\_Sample\_011046840, Unigene42798\_Sample\_011046840, Unigene40899\_Sample\_011046840, Unigene35692\_Sample\_011046840, Unigene39235\_Sample\_011046840, Unigene27745\_Sample\_011046840, Unigene10754\_Sample\_011046840, Unigene5720\_Sample\_011046840, Unigene9889\_Sample\_011046840, Unigene38857\_Sample\_011046840, Unigene20264\_Sample\_011046840, Unigene42293\_Sample\_011046840, Unigene42024\_Sample\_011046840, Unigene40316\_Sample\_011046840, Unigene23234\_Sample\_011046840, Unigene20485\_Sample\_011046840, Unigene29743\_Sample\_011046840, Unigene38612\_Sample\_011046840, Unigene28315\_Sample\_011046840, Unigene2740\_Sample\_011046840, Unigene14963\_Sample\_011046840, Unigene34975\_Sample\_011046840, Unigene24318\_Sample\_011046840, Unigene39777\_Sample\_011046840, Unigene31453\_Sample\_011046840, Unigene29661\_Sample\_011046840, Unigene35086\_Sample\_011046840, Unigene42709\_Sample\_011046840, Unigene5006\_Sample\_011046840, Unigene31648\_Sample\_011046840, Unigene31407\_Sample\_011046840, Unigene42255\_Sample\_011046840, Unigene22119\_Sample\_011046840, Unigene43273\_Sample\_011046840, Unigene42020\_Sample\_011046840, Unigene29398\_Sample\_011046840, Unigene14412\_Sample\_011046840, Unigene8353\_Sample\_011046840, Unigene22727\_Sample\_011046840, Unigene30631\_Sample\_011046840, Unigene7348\_Sample\_011046840, Unigene35051\_Sample\_011046840, Unigene6849\_Sample\_011046840, Unigene8569\_Sample\_011046840, Unigene34380\_Sample\_011046840, Unigene39701\_Sample\_011046840, Unigene20616\_Sample\_011046840, Unigene41119\_Sample\_011046840, Unigene4922\_Sample\_011046840, Unigene9782\_Sample\_011046840, Unigene2039\_Sample\_011046840, Unigene29712\_Sample\_011046840, Unigene6017\_Sample\_011046840, Unigene33956\_Sample\_011046840, Unigene40428\_Sample\_011046840, Unigene4300\_Sample\_011046840, Unigene28019\_Sample\_011046840, Unigene14037\_Sample\_011046840, Unigene11150\_Sample\_011046840, Unigene21352\_Sample\_011046840, Unigene13251\_Sample\_011046840, Unigene32546\_Sample\_011046840, Unigene39745\_Sample\_011046840, Unigene15931\_Sample\_011046840, Unigene36603\_Sample\_011046840, Unigene4572\_Sample\_011046840, Unigene31444\_Sample\_011046840, Unigene30936\_Sample\_011046840, Unigene24314\_Sample\_011046840, Unigene43217\_Sample\_011046840, Unigene42317\_Sample\_011046840, Unigene2237\_Sample\_011046840, Unigene11994\_Sample\_011046840, Unigene41108\_Sample\_011046840, Unigene22809\_Sample\_011046840, Unigene25745\_Sample\_011046840, Unigene22078\_Sample\_011046840, Unigene42628\_Sample\_011046840, Unigene26460\_Sample\_011046840, Unigene38313\_Sample\_011046840, Unigene39818\_Sample\_011046840, Unigene13083\_Sample\_011046840, Unigene34838\_Sample\_011046840, Unigene42039\_Sample\_011046840, Unigene35414\_Sample\_011046840, Unigene41216\_Sample\_011046840, Unigene5003\_Sample\_011046840, Unigene42549\_Sample\_011046840, Unigene35820\_Sample\_011046840, Unigene42434\_Sample\_011046840, Unigene22537\_Sample\_011046840, Unigene10050\_Sample\_011046840, Unigene13921\_Sample\_011046840, Unigene9447\_Sample\_011046840, Unigene20272\_Sample\_011046840, Unigene27020\_Sample\_011046840, Unigene38482\_Sample\_011046840, Unigene38449\_Sample\_011046840, Unigene37787\_Sample\_011046840, Unigene38540\_Sample\_011046840, Unigene40676\_Sample\_011046840, Unigene26563\_Sample\_011046840, Unigene3134\_Sample\_011046840, Unigene37146\_Sample\_011046840, Unigene11875\_Sample\_011046840, Unigene16690\_Sample\_011046840, Unigene23601\_Sample\_011046840, Unigene18027\_Sample\_011046840, Unigene33901\_Sample\_011046840, Unigene37789\_Sample\_011046840, Unigene41638\_Sample\_011046840, Unigene33395\_Sample\_011046840, Unigene39987\_Sample\_011046840, Unigene42168\_Sample\_011046840, Unigene29670\_Sample\_011046840, Unigene27635\_Sample\_011046840, Unigene14977\_Sample\_011046840, Unigene5557\_Sample\_011046840, Unigene31260\_Sample\_011046840, Unigene28451\_Sample\_011046840, Unigene29095\_Sample\_011046840, Unigene30975\_Sample\_011046840, Unigene15120\_Sample\_011046840, Unigene17564\_Sample\_011046840, Unigene39606\_Sample\_011046840, Unigene43449\_Sample\_011046840, Unigene37815\_Sample\_011046840, Unigene26646\_Sample\_011046840, Unigene41252\_Sample\_011046840, Unigene32397\_Sample\_011046840, Unigene26435\_Sample\_011046840, Unigene10332\_Sample\_011046840, Unigene5425\_Sample\_011046840, Unigene4108\_Sample\_011046840, Unigene40703\_Sample\_011046840, Unigene34817\_Sample\_011046840, Unigene7299\_Sample\_011046840, Unigene26640\_Sample\_011046840, Unigene36817\_Sample\_011046840, Unigene4595\_Sample\_011046840, Unigene40609\_Sample\_011046840, Unigene28024\_Sample\_011046840, Unigene1830\_Sample\_011046840, Unigene37014\_Sample\_011046840, Unigene20572\_Sample\_011046840, Unigene38677\_Sample\_011046840, Unigene38254\_Sample\_011046840, Unigene40019\_Sample\_011046840, Unigene40031\_Sample\_011046840, Unigene7463\_Sample\_011046840, Unigene41720\_Sample\_011046840, Unigene14995\_Sample\_011046840, Unigene1355\_Sample\_011046840, Unigene41102\_Sample\_011046840, Unigene32521\_Sample\_011046840, Unigene33524\_Sample\_011046840, Unigene9789\_Sample\_011046840, Unigene38827\_Sample\_011046840, Unigene25963\_Sample\_011046840, Unigene33567\_Sample\_011046840, Unigene41559\_Sample\_011046840, Unigene29238\_Sample\_011046840, Unigene6929\_Sample\_011046840, Unigene36638\_Sample\_011046840, Unigene496\_Sample\_011046840, Unigene2002\_Sample\_011046840, Unigene32884\_Sample\_011046840, Unigene30166\_Sample\_011046840, Unigene38801\_Sample\_011046840, Unigene24559\_Sample\_011046840, Unigene37919\_Sample\_011046840, Unigene34752\_Sample\_011046840, Unigene31306\_Sample\_011046840, Unigene40496\_Sample\_011046840, Unigene41694\_Sample\_011046840, Unigene11931\_Sample\_011046840, Unigene36123\_Sample\_011046840, Unigene5385\_Sample\_011046840, Unigene40745\_Sample\_011046840, Unigene7125\_Sample\_011046840, Unigene28038\_Sample\_011046840, Unigene37398\_Sample\_011046840, Unigene13786\_Sample\_011046840, Unigene21551\_Sample\_011046840, Unigene5962\_Sample\_011046840, Unigene36779\_Sample\_011046840, Unigene42979\_Sample\_011046840, Unigene28616\_Sample\_011046840, Unigene26707\_Sample\_011046840, Unigene6626\_Sample\_011046840, Unigene5200\_Sample\_011046840, Unigene6861\_Sample\_011046840, Unigene25450\_Sample\_011046840, Unigene6991\_Sample\_011046840, Unigene38328\_Sample\_011046840, Unigene23645\_Sample\_011046840, Unigene39232\_Sample\_011046840, Unigene32668\_Sample\_011046840, Unigene13656\_Sample\_011046840, Unigene43358\_Sample\_011046840, Unigene39608\_Sample\_011046840, Unigene23032\_Sample\_011046840, Unigene39480\_Sample\_011046840, Unigene40491\_Sample\_011046840, Unigene25194\_Sample\_011046840, Unigene33508\_Sample\_011046840, Unigene37796\_Sample\_011046840, Unigene10108\_Sample\_011046840, Unigene19985\_Sample\_011046840, Unigene39905\_Sample\_011046840, Unigene4100\_Sample\_011046840, Unigene31024\_Sample\_011046840, Unigene43502\_Sample\_011046840, Unigene27982\_Sample\_011046840, Unigene33048\_Sample\_011046840, Unigene42038\_Sample\_011046840, Unigene14245\_Sample\_011046840, Unigene43519\_Sample\_011046840, Unigene36293\_Sample\_011046840, Unigene41873\_Sample\_011046840, Unigene32377\_Sample\_011046840, Unigene43410\_Sample\_011046840, Unigene39202\_Sample\_011046840, Unigene6873\_Sample\_011046840, Unigene35609\_Sample\_011046840, Unigene38940\_Sample\_011046840, Unigene41283\_Sample\_011046840, Unigene8658\_Sample\_011046840, Unigene26198\_Sample\_011046840, Unigene34730\_Sample\_011046840, Unigene23012\_Sample\_011046840, Unigene37892\_Sample\_011046840, Unigene36547\_Sample\_011046840, Unigene34200\_Sample\_011046840, Unigene39956\_Sample\_011046840, Unigene20538\_Sample\_011046840, Unigene35877\_Sample\_011046840, Unigene18965\_Sample\_011046840, Unigene33873\_Sample\_011046840, Unigene23494\_Sample\_011046840, Unigene1379\_Sample\_011046840, Unigene43023\_Sample\_011046840, Unigene41968\_Sample\_011046840, Unigene6784\_Sample\_011046840, Unigene25891\_Sample\_011046840, Unigene26491\_Sample\_011046840, Unigene29963\_Sample\_011046840, Unigene37790\_Sample\_011046840, Unigene18925\_Sample\_011046840, Unigene41072\_Sample\_011046840, Unigene6705\_Sample\_011046840, Unigene33046\_Sample\_011046840, Unigene36229\_Sample\_011046840, Unigene42700\_Sample\_011046840, Unigene706\_Sample\_011046840, Unigene27243\_Sample\_011046840, Unigene7553\_Sample\_011046840, Unigene35495\_Sample\_011046840, Unigene21063\_Sample\_011046840, Unigene9937\_Sample\_011046840, Unigene13581\_Sample\_011046840, Unigene43567\_Sample\_011046840, Unigene26863\_Sample\_011046840, Unigene36780\_Sample\_011046840, Unigene42404\_Sample\_011046840, Unigene36977\_Sample\_011046840, Unigene16992\_Sample\_011046840, Unigene30289\_Sample\_011046840, Unigene36232\_Sample\_011046840, Unigene19207\_Sample\_011046840, Unigene24096\_Sample\_011046840, Unigene4855\_Sample\_011046840, Unigene43373\_Sample\_011046840, Unigene23833\_Sample\_011046840, Unigene9558\_Sample\_011046840, Unigene40875\_Sample\_011046840, Unigene43487\_Sample\_011046840, Unigene32576\_Sample\_011046840, Unigene4849\_Sample\_011046840, Unigene34163\_Sample\_011046840, Unigene33344\_Sample\_011046840, Unigene39032\_Sample\_011046840, Unigene40781\_Sample\_011046840, Unigene2216\_Sample\_011046840, Unigene41670\_Sample\_011046840, Unigene36747\_Sample\_011046840, Unigene8605\_Sample\_011046840, Unigene33148\_Sample\_011046840, Unigene752\_Sample\_011046840, Unigene42875\_Sample\_011046840, Unigene35307\_Sample\_011046840, Unigene10443\_Sample\_011046840, Unigene11035\_Sample\_011046840, Unigene14602\_Sample\_011046840, Unigene11279\_Sample\_011046840, Unigene24744\_Sample\_011046840, Unigene19882\_Sample\_011046840, Unigene20463\_Sample\_011046840, Unigene42337\_Sample\_011046840, Unigene17478\_Sample\_011046840, Unigene41722\_Sample\_011046840, Unigene41299\_Sample\_011046840, Unigene42966\_Sample\_011046840, Unigene43521\_Sample\_011046840, Unigene1568\_Sample\_011046840, Unigene34785\_Sample\_011046840, Unigene38468\_Sample\_011046840, Unigene30715\_Sample\_011046840, Unigene42761\_Sample\_011046840, Unigene599\_Sample\_011046840, Unigene18264\_Sample\_011046840, Unigene37236\_Sample\_011046840, Unigene42685\_Sample\_011046840, Unigene38203\_Sample\_011046840, Unigene13335\_Sample\_011046840, Unigene27924\_Sample\_011046840, Unigene10706\_Sample\_011046840, Unigene41121\_Sample\_011046840, Unigene40533\_Sample\_011046840, Unigene24635\_Sample\_011046840, Unigene18128\_Sample\_011046840, Unigene22945\_Sample\_011046840, Unigene19049\_Sample\_011046840, Unigene37255\_Sample\_011046840, Unigene18691\_Sample\_011046840, Unigene24400\_Sample\_011046840, Unigene19287\_Sample\_011046840, Unigene24518\_Sample\_011046840, Unigene27725\_Sample\_011046840, Unigene7588\_Sample\_011046840, Unigene14847\_Sample\_011046840, Unigene42103\_Sample\_011046840, Unigene41785\_Sample\_011046840, Unigene35673\_Sample\_011046840, Unigene22486\_Sample\_011046840, Unigene36628\_Sample\_011046840, Unigene39912\_Sample\_011046840, Unigene4368\_Sample\_011046840, Unigene36382\_Sample\_011046840, Unigene22482\_Sample\_011046840, Unigene6634\_Sample\_011046840, Unigene41391\_Sample\_011046840, Unigene43511\_Sample\_011046840, Unigene28177\_Sample\_011046840, Unigene32361\_Sample\_011046840, Unigene33015\_Sample\_011046840, Unigene32026\_Sample\_011046840, Unigene9678\_Sample\_011046840, Unigene15903\_Sample\_011046840, Unigene40257\_Sample\_011046840, Unigene8931\_Sample\_011046840, Unigene28117\_Sample\_011046840, Unigene43514\_Sample\_011046840, Unigene13197\_Sample\_011046840, Unigene41937\_Sample\_011046840, Unigene33737\_Sample\_011046840, Unigene15140\_Sample\_011046840, Unigene38469\_Sample\_011046840, Unigene32866\_Sample\_011046840, Unigene14307\_Sample\_011046840, Unigene20643\_Sample\_011046840, Unigene39579\_Sample\_011046840, Unigene5097\_Sample\_011046840, Unigene32770\_Sample\_011046840, Unigene37589\_Sample\_011046840, Unigene11155\_Sample\_011046840, Unigene30337\_Sample\_011046840, Unigene39571\_Sample\_011046840, Unigene2160\_Sample\_011046840, Unigene39057\_Sample\_011046840, Unigene32171\_Sample\_011046840, Unigene40083\_Sample\_011046840, Unigene23676\_Sample\_011046840, Unigene25874\_Sample\_011046840, Unigene41236\_Sample\_011046840, Unigene36770\_Sample\_011046840, Unigene35486\_Sample\_011046840, Unigene15713\_Sample\_011046840, Unigene14623\_Sample\_011046840, Unigene37474\_Sample\_011046840, Unigene34254\_Sample\_011046840, Unigene43374\_Sample\_011046840, Unigene17541\_Sample\_011046840, Unigene17049\_Sample\_011046840, Unigene7014\_Sample\_011046840, Unigene41042\_Sample\_011046840, Unigene4577\_Sample\_011046840, Unigene27233\_Sample\_011046840, Unigene5650\_Sample\_011046840, Unigene6209\_Sample\_011046840, Unigene38242\_Sample\_011046840, Unigene38803\_Sample\_011046840, Unigene22036\_Sample\_011046840, Unigene25298\_Sample\_011046840, Unigene41727\_Sample\_011046840, Unigene41469\_Sample\_011046840, Unigene40813\_Sample\_011046840, Unigene7752\_Sample\_011046840, Unigene21046\_Sample\_011046840, Unigene15743\_Sample\_011046840, Unigene34777\_Sample\_011046840, Unigene19991\_Sample\_011046840, Unigene37995\_Sample\_011046840, Unigene38701\_Sample\_011046840, Unigene42622\_Sample\_011046840, Unigene38323\_Sample\_011046840, Unigene8388\_Sample\_011046840, Unigene8213\_Sample\_011046840, Unigene16359\_Sample\_011046840, Unigene40247\_Sample\_011046840, Unigene6608\_Sample\_011046840, Unigene33090\_Sample\_011046840, Unigene25203\_Sample\_011046840, Unigene5772\_Sample\_011046840, Unigene35507\_Sample\_011046840, Unigene43051\_Sample\_011046840, Unigene31151\_Sample\_011046840, Unigene4412\_Sample\_011046840, Unigene37414\_Sample\_011046840, Unigene40399\_Sample\_011046840, Unigene20960\_Sample\_011046840, Unigene5037\_Sample\_011046840, Unigene33982\_Sample\_011046840, Unigene20699\_Sample\_011046840, Unigene40807\_Sample\_011046840, Unigene1879\_Sample\_011046840, Unigene37529\_Sample\_011046840, Unigene42653\_Sample\_011046840, Unigene1535\_Sample\_011046840, Unigene37694\_Sample\_011046840, Unigene6457\_Sample\_011046840, Unigene641\_Sample\_011046840, Unigene11516\_Sample\_011046840, Unigene39750\_Sample\_011046840, Unigene34208\_Sample\_011046840, Unigene22265\_Sample\_011046840, Unigene38279\_Sample\_011046840, Unigene28752\_Sample\_011046840, Unigene25915\_Sample\_011046840, Unigene37799\_Sample\_011046840, Unigene26572\_Sample\_011046840, Unigene42972\_Sample\_011046840, Unigene43364\_Sample\_011046840, Unigene2284\_Sample\_011046840, Unigene33129\_Sample\_011046840, Unigene41067\_Sample\_011046840, Unigene19092\_Sample\_011046840, Unigene36364\_Sample\_011046840, Unigene28943\_Sample\_011046840, Unigene30501\_Sample\_011046840, Unigene25408\_Sample\_011046840, Unigene17585\_Sample\_011046840, Unigene4920\_Sample\_011046840, Unigene36504\_Sample\_011046840, Unigene23249\_Sample\_011046840, Unigene7708\_Sample\_011046840, Unigene40839\_Sample\_011046840, Unigene19948\_Sample\_011046840, Unigene39661\_Sample\_011046840, Unigene41436\_Sample\_011046840, Unigene13336\_Sample\_011046840, Unigene30481\_Sample\_011046840, Unigene39751\_Sample\_011046840, Unigene8000\_Sample\_011046840, Unigene37684\_Sample\_011046840, Unigene38450\_Sample\_011046840, Unigene42250\_Sample\_011046840, Unigene33105\_Sample\_011046840, Unigene2066\_Sample\_011046840, Unigene39916\_Sample\_011046840, Unigene41869\_Sample\_011046840, Unigene39979\_Sample\_011046840, Unigene16302\_Sample\_011046840, Unigene2783\_Sample\_011046840, Unigene41379\_Sample\_011046840, Unigene33011\_Sample\_011046840, Unigene30994\_Sample\_011046840, Unigene33465\_Sample\_011046840, Unigene14238\_Sample\_011046840, Unigene31397\_Sample\_011046840, Unigene36103\_Sample\_011046840, Unigene41546\_Sample\_011046840, Unigene33527\_Sample\_011046840, Unigene7054\_Sample\_011046840, Unigene15710\_Sample\_011046840, Unigene28952\_Sample\_011046840, Unigene34615\_Sample\_011046840, Unigene32859\_Sample\_011046840, Unigene34686\_Sample\_011046840, Unigene34028\_Sample\_011046840, Unigene39604\_Sample\_011046840, Unigene43264\_Sample\_011046840, Unigene39889\_Sample\_011046840, Unigene38023\_Sample\_011046840, Unigene26178\_Sample\_011046840, Unigene26641\_Sample\_011046840, Unigene34267\_Sample\_011046840, Unigene7902\_Sample\_011046840, Unigene42776\_Sample\_011046840, Unigene37568\_Sample\_011046840, Unigene26763\_Sample\_011046840, Unigene41910\_Sample\_011046840, Unigene19401\_Sample\_011046840, Unigene39840\_Sample\_011046840, Unigene40636\_Sample\_011046840, Unigene43154\_Sample\_011046840, Unigene7132\_Sample\_011046840, Unigene30892\_Sample\_011046840, Unigene313\_Sample\_011046840, Unigene43100\_Sample\_011046840, Unigene2493\_Sample\_011046840, Unigene2917\_Sample\_011046840, Unigene908\_Sample\_011046840, Unigene32380\_Sample\_011046840, Unigene39302\_Sample\_011046840, Unigene23411\_Sample\_011046840, Unigene32488\_Sample\_011046840, Unigene41580\_Sample\_011046840, Unigene41153\_Sample\_011046840, Unigene35962\_Sample\_011046840, Unigene32455\_Sample\_011046840, Unigene33356\_Sample\_011046840, Unigene32335\_Sample\_011046840, Unigene42949\_Sample\_011046840, Unigene20979\_Sample\_011046840, Unigene40972\_Sample\_011046840, Unigene6748\_Sample\_011046840, Unigene42607\_Sample\_011046840, Unigene5545\_Sample\_011046840, Unigene40625\_Sample\_011046840, Unigene40773\_Sample\_011046840, Unigene20982\_Sample\_011046840, Unigene32089\_Sample\_011046840, Unigene31072\_Sample\_011046840, Unigene30370\_Sample\_011046840, Unigene41318\_Sample\_011046840, Unigene39708\_Sample\_011046840, Unigene17494\_Sample\_011046840, Unigene34499\_Sample\_011046840, Unigene23767\_Sample\_011046840, Unigene36931\_Sample\_011046840, Unigene35607\_Sample\_011046840, Unigene41473\_Sample\_011046840, Unigene42635\_Sample\_011046840, Unigene5219\_Sample\_011046840, Unigene7352\_Sample\_011046840, Unigene18500\_Sample\_011046840, Unigene31129\_Sample\_011046840, Unigene34236\_Sample\_011046840, Unigene42762\_Sample\_011046840, Unigene34068\_Sample\_011046840, Unigene39858\_Sample\_011046840, Unigene177\_Sample\_011046840, Unigene41833\_Sample\_011046840, Unigene11899\_Sample\_011046840, Unigene35615\_Sample\_011046840, Unigene38504\_Sample\_011046840, Unigene40734\_Sample\_011046840, Unigene15902\_Sample\_011046840, Unigene36848\_Sample\_011046840, Unigene36463\_Sample\_011046840, Unigene38201\_Sample\_011046840, Unigene37095\_Sample\_011046840, Unigene30026\_Sample\_011046840, Unigene18797\_Sample\_011046840, Unigene36359\_Sample\_011046840, Unigene15951\_Sample\_011046840, Unigene1709\_Sample\_011046840, Unigene5621\_Sample\_011046840, Unigene15791\_Sample\_011046840, Unigene39666\_Sample\_011046840, Unigene35437\_Sample\_011046840, Unigene37505\_Sample\_011046840, Unigene14153\_Sample\_011046840, Unigene24478\_Sample\_011046840, Unigene30203\_Sample\_011046840, Unigene43435\_Sample\_011046840, Unigene39812\_Sample\_011046840, Unigene40047\_Sample\_011046840, Unigene39711\_Sample\_011046840, Unigene30726\_Sample\_011046840, Unigene17955\_Sample\_011046840, Unigene11041\_Sample\_011046840, Unigene33707\_Sample\_011046840, Unigene41104\_Sample\_011046840, Unigene20573\_Sample\_011046840, Unigene18097\_Sample\_011046840, Unigene34842\_Sample\_011046840, Unigene36005\_Sample\_011046840, Unigene17299\_Sample\_011046840, Unigene37066\_Sample\_011046840, Unigene607\_Sample\_011046840, Unigene41965\_Sample\_011046840, Unigene40796\_Sample\_011046840, Unigene4663\_Sample\_011046840, Unigene26766\_Sample\_011046840, Unigene24013\_Sample\_011046840, Unigene34514\_Sample\_011046840, Unigene26168\_Sample\_011046840, Unigene37186\_Sample\_011046840, Unigene43122\_Sample\_011046840, Unigene41124\_Sample\_011046840, Unigene30959\_Sample\_011046840, Unigene33208\_Sample\_011046840, Unigene7795\_Sample\_011046840, Unigene41096\_Sample\_011046840, Unigene37358\_Sample\_011046840, Unigene29560\_Sample\_011046840, Unigene25972\_Sample\_011046840, Unigene35878\_Sample\_011046840, Unigene18599\_Sample\_011046840, Unigene43230\_Sample\_011046840, Unigene5654\_Sample\_011046840, Unigene8445\_Sample\_011046840, Unigene25570\_Sample\_011046840, Unigene35629\_Sample\_011046840, Unigene37311\_Sample\_011046840, Unigene22613\_Sample\_011046840, Unigene34070\_Sample\_011046840, Unigene41932\_Sample\_011046840, Unigene32833\_Sample\_011046840, Unigene20100\_Sample\_011046840, Unigene39763\_Sample\_011046840, Unigene41229\_Sample\_011046840, Unigene42995\_Sample\_011046840, Unigene43297\_Sample\_011046840, Unigene39589\_Sample\_011046840, Unigene37863\_Sample\_011046840, Unigene39982\_Sample\_011046840, Unigene11151\_Sample\_011046840, Unigene24036\_Sample\_011046840, Unigene573\_Sample\_011046840, Unigene3770\_Sample\_011046840, Unigene17734\_Sample\_011046840, Unigene10992\_Sample\_011046840, Unigene29802\_Sample\_011046840, Unigene13794\_Sample\_011046840, Unigene24934\_Sample\_011046840, Unigene33749\_Sample\_011046840, Unigene33913\_Sample\_011046840, Unigene24856\_Sample\_011046840, Unigene37444\_Sample\_011046840, Unigene7472\_Sample\_011046840, Unigene4414\_Sample\_011046840, Unigene40877\_Sample\_011046840, Unigene28836\_Sample\_011046840, Unigene5146\_Sample\_011046840, Unigene37933\_Sample\_011046840, Unigene35780\_Sample\_011046840, Unigene10927\_Sample\_011046840, Unigene31301\_Sample\_011046840, Unigene42698\_Sample\_011046840, Unigene41406\_Sample\_011046840, Unigene11913\_Sample\_011046840, Unigene36035\_Sample\_011046840, Unigene29953\_Sample\_011046840, Unigene30142\_Sample\_011046840, Unigene12220\_Sample\_011046840, Unigene11742\_Sample\_011046840, Unigene7137\_Sample\_011046840, Unigene32099\_Sample\_011046840, Unigene1345\_Sample\_011046840, Unigene4825\_Sample\_011046840, Unigene37899\_Sample\_011046840, Unigene38733\_Sample\_011046840, Unigene37451\_Sample\_011046840, Unigene14113\_Sample\_011046840, Unigene3990\_Sample\_011046840, Unigene40004\_Sample\_011046840, Unigene3003\_Sample\_011046840, Unigene41997\_Sample\_011046840, Unigene41587\_Sample\_011046840, Unigene38687\_Sample\_011046840, Unigene35271\_Sample\_011046840, Unigene15302\_Sample\_011046840, Unigene30924\_Sample\_011046840, Unigene38655\_Sample\_011046840, Unigene43473\_Sample\_011046840, Unigene29985\_Sample\_011046840, Unigene34435\_Sample\_011046840, Unigene15608\_Sample\_011046840, Unigene33768\_Sample\_011046840, Unigene26813\_Sample\_011046840, Unigene30528\_Sample\_011046840, Unigene38331\_Sample\_011046840, Unigene26216\_Sample\_011046840, Unigene40015\_Sample\_011046840, Unigene28979\_Sample\_011046840, Unigene42775\_Sample\_011046840, Unigene4744\_Sample\_011046840, Unigene41926\_Sample\_011046840, Unigene1549\_Sample\_011046840, Unigene38671\_Sample\_011046840, Unigene31629\_Sample\_011046840, Unigene24418\_Sample\_011046840, Unigene5087\_Sample\_011046840, Unigene22095\_Sample\_011046840, Unigene30478\_Sample\_011046840, Unigene13562\_Sample\_011046840, Unigene26994\_Sample\_011046840, Unigene39554\_Sample\_011046840, Unigene5867\_Sample\_011046840, Unigene42338\_Sample\_011046840, Unigene6313\_Sample\_011046840, Unigene32005\_Sample\_011046840, Unigene31572\_Sample\_011046840, Unigene28410\_Sample\_011046840, Unigene23029\_Sample\_011046840, Unigene39061\_Sample\_011046840, Unigene33657\_Sample\_011046840, Unigene10533\_Sample\_011046840, Unigene37904\_Sample\_011046840, Unigene34854\_Sample\_011046840, Unigene18334\_Sample\_011046840, Unigene40825\_Sample\_011046840, Unigene37545\_Sample\_011046840, Unigene36084\_Sample\_011046840, Unigene8222\_Sample\_011046840, Unigene39570\_Sample\_011046840, Unigene39888\_Sample\_011046840, Unigene41368\_Sample\_011046840, Unigene34316\_Sample\_011046840, Unigene1485\_Sample\_011046840, Unigene42378\_Sample\_011046840, Unigene35961\_Sample\_011046840, Unigene11174\_Sample\_011046840, Unigene8137\_Sample\_011046840, Unigene17146\_Sample\_011046840, Unigene38709\_Sample\_011046840, Unigene38720\_Sample\_011046840, Unigene31864\_Sample\_011046840, Unigene4344\_Sample\_011046840, Unigene29502\_Sample\_011046840, Unigene39243\_Sample\_011046840, Unigene36654\_Sample\_011046840, Unigene37871\_Sample\_011046840, Unigene2762\_Sample\_011046840, Unigene32612\_Sample\_011046840, Unigene8160\_Sample\_011046840, Unigene32209\_Sample\_011046840, Unigene16376\_Sample\_011046840, Unigene34278\_Sample\_011046840, Unigene39923\_Sample\_011046840, Unigene17019\_Sample\_011046840, Unigene37653\_Sample\_011046840, Unigene24390\_Sample\_011046840, Unigene39165\_Sample\_011046840, Unigene24220\_Sample\_011046840, Unigene13861\_Sample\_011046840, Unigene5541\_Sample\_011046840, Unigene1008\_Sample\_011046840, Unigene26070\_Sample\_011046840, Unigene2863\_Sample\_011046840, Unigene30575\_Sample\_011046840, Unigene31634\_Sample\_011046840, Unigene27458\_Sample\_011046840, Unigene28335\_Sample\_011046840, Unigene35240\_Sample\_011046840, Unigene31901\_Sample\_011046840, Unigene29526\_Sample\_011046840, Unigene32895\_Sample\_011046840, Unigene41934\_Sample\_011046840, Unigene11454\_Sample\_011046840, Unigene34669\_Sample\_011046840, Unigene25402\_Sample\_011046840, Unigene27955\_Sample\_011046840, Unigene8253\_Sample\_011046840, Unigene34052\_Sample\_011046840, Unigene33222\_Sample\_011046840, Unigene17426\_Sample\_011046840, Unigene43404\_Sample\_011046840, Unigene38467\_Sample\_011046840, Unigene29615\_Sample\_011046840, Unigene35066\_Sample\_011046840, Unigene41540\_Sample\_011046840, Unigene34301\_Sample\_011046840, Unigene29499\_Sample\_011046840, Unigene42019\_Sample\_011046840, Unigene35346\_Sample\_011046840, Unigene789\_Sample\_011046840, Unigene43488\_Sample\_011046840, Unigene35895\_Sample\_011046840, Unigene32844\_Sample\_011046840, Unigene14007\_Sample\_011046840, Unigene1375\_Sample\_011046840, Unigene34436\_Sample\_011046840, Unigene39224\_Sample\_011046840, Unigene43413\_Sample\_011046840, Unigene43492\_Sample\_011046840, Unigene16409\_Sample\_011046840, Unigene7545\_Sample\_011046840, Unigene39296\_Sample\_011046840, Unigene341\_Sample\_011046840, Unigene30024\_Sample\_011046840, Unigene38077\_Sample\_011046840, Unigene7175\_Sample\_011046840, Unigene7113\_Sample\_011046840, Unigene20160\_Sample\_011046840, Unigene42272\_Sample\_011046840, Unigene8086\_Sample\_011046840, Unigene15260\_Sample\_011046840, Unigene42713\_Sample\_011046840, Unigene2820\_Sample\_011046840, Unigene40131\_Sample\_011046840, Unigene14074\_Sample\_011046840, Unigene29764\_Sample\_011046840, Unigene29769\_Sample\_011046840, Unigene40658\_Sample\_011046840, Unigene15680\_Sample\_011046840, Unigene35547\_Sample\_011046840, Unigene22514\_Sample\_011046840, Unigene27933\_Sample\_011046840, Unigene35731\_Sample\_011046840, Unigene33431\_Sample\_011046840, Unigene4839\_Sample\_011046840, Unigene35206\_Sample\_011046840, Unigene42012\_Sample\_011046840, Unigene35159\_Sample\_011046840, Unigene7427\_Sample\_011046840, Unigene27496\_Sample\_011046840, Unigene42659\_Sample\_011046840, Unigene37034\_Sample\_011046840, Unigene18009\_Sample\_011046840, Unigene38768\_Sample\_011046840, Unigene43318\_Sample\_011046840, Unigene32425\_Sample\_011046840, Unigene20494\_Sample\_011046840, Unigene41585\_Sample\_011046840, Unigene25046\_Sample\_011046840, Unigene28695\_Sample\_011046840, Unigene36393\_Sample\_011046840, Unigene5809\_Sample\_011046840, Unigene35693\_Sample\_011046840, Unigene6088\_Sample\_011046840, Unigene17840\_Sample\_011046840, Unigene20467\_Sample\_011046840, Unigene34194\_Sample\_011046840, Unigene42575\_Sample\_011046840, Unigene9133\_Sample\_011046840, Unigene37766\_Sample\_011046840, Unigene30495\_Sample\_011046840, Unigene27213\_Sample\_011046840, Unigene28516\_Sample\_011046840, Unigene34588\_Sample\_011046840, Unigene34929\_Sample\_011046840, Unigene34046\_Sample\_011046840, Unigene19619\_Sample\_011046840, Unigene40793\_Sample\_011046840, Unigene28684\_Sample\_011046840, Unigene4979\_Sample\_011046840, Unigene2123\_Sample\_011046840, Unigene22165\_Sample\_011046840, Unigene24711\_Sample\_011046840, Unigene24034\_Sample\_011046840, Unigene4822\_Sample\_011046840, Unigene4085\_Sample\_011046840, Unigene41899\_Sample\_011046840, Unigene36997\_Sample\_011046840, Unigene8956\_Sample\_011046840, Unigene38357\_Sample\_011046840, Unigene16941\_Sample\_011046840, Unigene8240\_Sample\_011046840, Unigene27829\_Sample\_011046840, Unigene12936\_Sample\_011046840, Unigene4591\_Sample\_011046840, Unigene43073\_Sample\_011046840, Unigene42283\_Sample\_011046840, Unigene40123\_Sample\_011046840, Unigene38028\_Sample\_011046840, Unigene40677\_Sample\_011046840, Unigene35179\_Sample\_011046840, Unigene40861\_Sample\_011046840, Unigene8019\_Sample\_011046840, Unigene39223\_Sample\_011046840, Unigene39446\_Sample\_011046840, Unigene18919\_Sample\_011046840, Unigene5953\_Sample\_011046840, Unigene43278\_Sample\_011046840, Unigene42206\_Sample\_011046840, Unigene420\_Sample\_011046840, Unigene10640\_Sample\_011046840, Unigene16082\_Sample\_011046840, Unigene20936\_Sample\_011046840, Unigene17259\_Sample\_011046840, Unigene35286\_Sample\_011046840, Unigene15508\_Sample\_011046840, Unigene39151\_Sample\_011046840, Unigene38104\_Sample\_011046840, Unigene43236\_Sample\_011046840, Unigene43127\_Sample\_011046840, Unigene20070\_Sample\_011046840, Unigene7669\_Sample\_011046840, Unigene3517\_Sample\_011046840, Unigene36700\_Sample\_011046840, Unigene9113\_Sample\_011046840, Unigene22229\_Sample\_011046840, Unigene27942\_Sample\_011046840, Unigene29255\_Sample\_011046840, Unigene36048\_Sample\_011046840, Unigene30713\_Sample\_011046840, Unigene36942\_Sample\_011046840, Unigene16663\_Sample\_011046840, Unigene23409\_Sample\_011046840, Unigene39199\_Sample\_011046840, Unigene32899\_Sample\_011046840, Unigene36459\_Sample\_011046840, Unigene36813\_Sample\_011046840, Unigene39361\_Sample\_011046840, Unigene28409\_Sample\_011046840, Unigene2111\_Sample\_011046840, Unigene31120\_Sample\_011046840, Unigene40942\_Sample\_011046840, Unigene22310\_Sample\_011046840, Unigene14947\_Sample\_011046840, Unigene22599\_Sample\_011046840, Unigene9531\_Sample\_011046840, Unigene43610\_Sample\_011046840, Unigene40822\_Sample\_011046840, Unigene42964\_Sample\_011046840, Unigene13511\_Sample\_011046840, Unigene21015\_Sample\_011046840, Unigene43624\_Sample\_011046840, Unigene42483\_Sample\_011046840, Unigene16898\_Sample\_011046840, Unigene37888\_Sample\_011046840, Unigene3682\_Sample\_011046840, Unigene35244\_Sample\_011046840, Unigene41101\_Sample\_011046840, Unigene42596\_Sample\_011046840, Unigene40511\_Sample\_011046840, Unigene7067\_Sample\_011046840, Unigene32822\_Sample\_011046840, Unigene34033\_Sample\_011046840, Unigene28597\_Sample\_011046840, Unigene39581\_Sample\_011046840, Unigene41233\_Sample\_011046840, Unigene8118\_Sample\_011046840, Unigene31168\_Sample\_011046840, Unigene26581\_Sample\_011046840, Unigene33837\_Sample\_011046840, Unigene29655\_Sample\_011046840, Unigene41011\_Sample\_011046840, Unigene39690\_Sample\_011046840, Unigene40422\_Sample\_011046840, Unigene39503\_Sample\_011046840, Unigene24344\_Sample\_011046840, Unigene28415\_Sample\_011046840, Unigene10804\_Sample\_011046840, Unigene18504\_Sample\_011046840, Unigene11629\_Sample\_011046840, Unigene34862\_Sample\_011046840, Unigene65\_Sample\_011046840, Unigene2454\_Sample\_011046840, Unigene26060\_Sample\_011046840, Unigene8043\_Sample\_011046840, Unigene40564\_Sample\_011046840, Unigene39271\_Sample\_011046840, Unigene32436\_Sample\_011046840, Unigene35396\_Sample\_011046840, Unigene8372\_Sample\_011046840, Unigene42455\_Sample\_011046840, Unigene39178\_Sample\_011046840, Unigene28581\_Sample\_011046840, Unigene40966\_Sample\_011046840, Unigene13884\_Sample\_011046840, Unigene43423\_Sample\_011046840, Unigene34790\_Sample\_011046840, Unigene39962\_Sample\_011046840, Unigene37935\_Sample\_011046840, Unigene13388\_Sample\_011046840, Unigene36114\_Sample\_011046840, Unigene6445\_Sample\_011046840, Unigene30903\_Sample\_011046840, Unigene42097\_Sample\_011046840, Unigene5915\_Sample\_011046840, Unigene41398\_Sample\_011046840, Unigene41081\_Sample\_011046840, Unigene43361\_Sample\_011046840, Unigene28840\_Sample\_011046840, Unigene43533\_Sample\_011046840, Unigene34839\_Sample\_011046840, Unigene38011\_Sample\_011046840, Unigene7280\_Sample\_011046840, Unigene39389\_Sample\_011046840, Unigene18382\_Sample\_011046840, Unigene21475\_Sample\_011046840, Unigene38159\_Sample\_011046840, Unigene33673\_Sample\_011046840, Unigene4387\_Sample\_011046840, Unigene40560\_Sample\_011046840, Unigene22669\_Sample\_011046840, Unigene5733\_Sample\_011046840, Unigene29895\_Sample\_011046840, Unigene26628\_Sample\_011046840, Unigene11583\_Sample\_011046840, Unigene39063\_Sample\_011046840, Unigene37873\_Sample\_011046840, Unigene32695\_Sample\_011046840, Unigene10569\_Sample\_011046840, Unigene33052\_Sample\_011046840, Unigene25735\_Sample\_011046840, Unigene37090\_Sample\_011046840, Unigene34637\_Sample\_011046840, Unigene1215\_Sample\_011046840, Unigene34855\_Sample\_011046840, Unigene15111\_Sample\_011046840, Unigene26170\_Sample\_011046840, Unigene1398\_Sample\_011046840, Unigene29397\_Sample\_011046840, Unigene8123\_Sample\_011046840, Unigene32834\_Sample\_011046840, Unigene39279\_Sample\_011046840, Unigene42650\_Sample\_011046840, Unigene28450\_Sample\_011046840, Unigene32858\_Sample\_011046840, Unigene38944\_Sample\_011046840, Unigene32857\_Sample\_011046840, Unigene22531\_Sample\_011046840, Unigene42585\_Sample\_011046840, Unigene8205\_Sample\_011046840, Unigene15594\_Sample\_011046840, Unigene9954\_Sample\_011046840, Unigene7974\_Sample\_011046840, Unigene3810\_Sample\_011046840, Unigene38003\_Sample\_011046840, Unigene38402\_Sample\_011046840, Unigene19563\_Sample\_011046840, Unigene29159\_Sample\_011046840, Unigene40629\_Sample\_011046840, Unigene41502\_Sample\_011046840, Unigene30226\_Sample\_011046840, Unigene7294\_Sample\_011046840, Unigene7892\_Sample\_011046840, Unigene38207\_Sample\_011046840, Unigene18685\_Sample\_011046840, Unigene42359\_Sample\_011046840, Unigene41314\_Sample\_011046840, Unigene24259\_Sample\_011046840, Unigene17569\_Sample\_011046840, Unigene4443\_Sample\_011046840, Unigene42802\_Sample\_011046840, Unigene25769\_Sample\_011046840, Unigene41000\_Sample\_011046840, Unigene37514\_Sample\_011046840, Unigene36854\_Sample\_011046840, Unigene42988\_Sample\_011046840, Unigene32735\_Sample\_011046840, Unigene39086\_Sample\_011046840, Unigene35969\_Sample\_011046840, Unigene43066\_Sample\_011046840, Unigene10054\_Sample\_011046840, Unigene38822\_Sample\_011046840, Unigene9950\_Sample\_011046840, Unigene38088\_Sample\_011046840, Unigene34784\_Sample\_011046840, Unigene38534\_Sample\_011046840, Unigene41512\_Sample\_011046840, Unigene2789\_Sample\_011046840, Unigene8373\_Sample\_011046840, Unigene20630\_Sample\_011046840, Unigene40856\_Sample\_011046840, Unigene30467\_Sample\_011046840, Unigene40811\_Sample\_011046840, Unigene39951\_Sample\_011046840, Unigene38730\_Sample\_011046840, Unigene43450\_Sample\_011046840, Unigene35452\_Sample\_011046840, Unigene40192\_Sample\_011046840, Unigene42554\_Sample\_011046840, Unigene38699\_Sample\_011046840, Unigene42105\_Sample\_011046840, Unigene27254\_Sample\_011046840, Unigene23020\_Sample\_011046840, Unigene42287\_Sample\_011046840, Unigene4612\_Sample\_011046840, Unigene34973\_Sample\_011046840, Unigene43494\_Sample\_011046840, Unigene37369\_Sample\_011046840, Unigene41679\_Sample\_011046840, Unigene41716\_Sample\_011046840, Unigene1226\_Sample\_011046840, Unigene37469\_Sample\_011046840, Unigene36671\_Sample\_011046840, Unigene11390\_Sample\_011046840, Unigene35859\_Sample\_011046840, Unigene14242\_Sample\_011046840, Unigene12277\_Sample\_011046840, Unigene1252\_Sample\_011046840, Unigene41439\_Sample\_011046840, Unigene32598\_Sample\_011046840, Unigene20078\_Sample\_011046840, Unigene23797\_Sample\_011046840, Unigene40439\_Sample\_011046840, Unigene11208\_Sample\_011046840, Unigene43019\_Sample\_011046840, Unigene1967\_Sample\_011046840, Unigene42448\_Sample\_011046840, Unigene34992\_Sample\_011046840, Unigene6181\_Sample\_011046840, Unigene29823\_Sample\_011046840, Unigene28676\_Sample\_011046840, Unigene9833\_Sample\_011046840, Unigene43336\_Sample\_011046840, Unigene38363\_Sample\_011046840, Unigene5316\_Sample\_011046840, Unigene19756\_Sample\_011046840, Unigene18015\_Sample\_011046840, Unigene36713\_Sample\_011046840, Unigene41507\_Sample\_011046840, Unigene29814\_Sample\_011046840, Unigene42849\_Sample\_011046840, Unigene31212\_Sample\_011046840, Unigene31960\_Sample\_011046840, Unigene34158\_Sample\_011046840, Unigene4234\_Sample\_011046840, Unigene34532\_Sample\_011046840, Unigene27358\_Sample\_011046840, Unigene13113\_Sample\_011046840, Unigene39033\_Sample\_011046840, Unigene19258\_Sample\_011046840, Unigene20546\_Sample\_011046840, Unigene42782\_Sample\_011046840, Unigene2439\_Sample\_011046840, Unigene20430\_Sample\_011046840, Unigene35676\_Sample\_011046840, Unigene42514\_Sample\_011046840, Unigene34471\_Sample\_011046840, Unigene37359\_Sample\_011046840, Unigene9750\_Sample\_011046840, Unigene39080\_Sample\_011046840, Unigene18477\_Sample\_011046840, Unigene43197\_Sample\_011046840, Unigene30634\_Sample\_011046840, Unigene5774\_Sample\_011046840, Unigene39136\_Sample\_011046840, Unigene36248\_Sample\_011046840, Unigene13709\_Sample\_011046840, Unigene37738\_Sample\_011046840, Unigene39725\_Sample\_011046840, Unigene7583\_Sample\_011046840, Unigene26517\_Sample\_011046840, Unigene27975\_Sample\_011046840, Unigene43263\_Sample\_011046840, Unigene32145\_Sample\_011046840, Unigene14511\_Sample\_011046840, Unigene8171\_Sample\_011046840, Unigene32749\_Sample\_011046840, Unigene14894\_Sample\_011046840, Unigene5322\_Sample\_011046840, Unigene12521\_Sample\_011046840, Unigene19469\_Sample\_011046840, Unigene12964\_Sample\_011046840, Unigene42377\_Sample\_011046840, Unigene16269\_Sample\_011046840, Unigene2536\_Sample\_011046840, Unigene41114\_Sample\_011046840, Unigene32865\_Sample\_011046840, Unigene32172\_Sample\_011046840, Unigene32463\_Sample\_011046840, Unigene40065\_Sample\_011046840, Unigene4481\_Sample\_011046840, Unigene4583\_Sample\_011046840, Unigene42935\_Sample\_011046840, Unigene39866\_Sample\_011046840, Unigene41562\_Sample\_011046840, Unigene23252\_Sample\_011046840, Unigene14195\_Sample\_011046840, Unigene32781\_Sample\_011046840, Unigene34547\_Sample\_011046840, Unigene13360\_Sample\_011046840, Unigene43314\_Sample\_011046840, Unigene34481\_Sample\_011046840, Unigene11179\_Sample\_011046840, Unigene40994\_Sample\_011046840, Unigene18714\_Sample\_011046840, Unigene38990\_Sample\_011046840, Unigene38406\_Sample\_011046840, Unigene36936\_Sample\_011046840, Unigene38017\_Sample\_011046840, Unigene5461\_Sample\_011046840, Unigene31532\_Sample\_011046840, Unigene23357\_Sample\_011046840, Unigene39992\_Sample\_011046840, Unigene7665\_Sample\_011046840, Unigene8218\_Sample\_011046840, Unigene38459\_Sample\_011046840, Unigene33047\_Sample\_011046840, Unigene24731\_Sample\_011046840, Unigene41792\_Sample\_011046840, Unigene41441\_Sample\_011046840, Unigene40136\_Sample\_011046840, Unigene40202\_Sample\_011046840, Unigene33426\_Sample\_011046840, Unigene14381\_Sample\_011046840, Unigene6486\_Sample\_011046840, Unigene2480\_Sample\_011046840, Unigene41914\_Sample\_011046840, Unigene9299\_Sample\_011046840, Unigene28958\_Sample\_011046840, Unigene40424\_Sample\_011046840, Unigene3599\_Sample\_011046840, Unigene25502\_Sample\_011046840, Unigene37081\_Sample\_011046840, Unigene11225\_Sample\_011046840, Unigene39665\_Sample\_011046840, Unigene30457\_Sample\_011046840, Unigene14002\_Sample\_011046840, Unigene5038\_Sample\_011046840, Unigene21444\_Sample\_011046840, Unigene43427\_Sample\_011046840, Unigene40124\_Sample\_011046840, Unigene36626\_Sample\_011046840, Unigene31258\_Sample\_011046840, Unigene43431\_Sample\_011046840, Unigene30067\_Sample\_011046840, Unigene33634\_Sample\_011046840, Unigene17531\_Sample\_011046840, Unigene37708\_Sample\_011046840, Unigene36139\_Sample\_011046840, Unigene29297\_Sample\_011046840, Unigene35421\_Sample\_011046840, Unigene24992\_Sample\_011046840, Unigene9448\_Sample\_011046840, Unigene37830\_Sample\_011046840, Unigene30708\_Sample\_011046840, Unigene33613\_Sample\_011046840, Unigene42985\_Sample\_011046840, Unigene42582\_Sample\_011046840, Unigene41740\_Sample\_011046840, Unigene20296\_Sample\_011046840, Unigene37841\_Sample\_011046840, Unigene40346\_Sample\_011046840, Unigene32864\_Sample\_011046840, Unigene38418\_Sample\_011046840, Unigene21000\_Sample\_011046840, Unigene30151\_Sample\_011046840, Unigene27532\_Sample\_011046840, Unigene6114\_Sample\_011046840, Unigene30699\_Sample\_011046840, Unigene1920\_Sample\_011046840, Unigene2470\_Sample\_011046840, Unigene3842\_Sample\_011046840, Unigene12743\_Sample\_011046840, Unigene41990\_Sample\_011046840, Unigene34257\_Sample\_011046840, Unigene11707\_Sample\_011046840, Unigene10770\_Sample\_011046840, Unigene22560\_Sample\_011046840, Unigene32952\_Sample\_011046840, Unigene41139\_Sample\_011046840, Unigene38322\_Sample\_011046840, Unigene41158\_Sample\_011046840, Unigene4492\_Sample\_011046840, Unigene16736\_Sample\_011046840, Unigene7963\_Sample\_011046840, Unigene34625\_Sample\_011046840, Unigene20308\_Sample\_011046840, Unigene33698\_Sample\_011046840, Unigene31578\_Sample\_011046840, Unigene38344\_Sample\_011046840, Unigene31239\_Sample\_011046840, Unigene37858\_Sample\_011046840, Unigene40137\_Sample\_011046840, Unigene16329\_Sample\_011046840, Unigene31582\_Sample\_011046840, Unigene17214\_Sample\_011046840, Unigene22216\_Sample\_011046840, Unigene31392\_Sample\_011046840, Unigene34877\_Sample\_011046840, Unigene30518\_Sample\_011046840, Unigene28689\_Sample\_011046840, Unigene10135\_Sample\_011046840, Unigene15362\_Sample\_011046840, Unigene17976\_Sample\_011046840, Unigene5517\_Sample\_011046840, Unigene33227\_Sample\_011046840, Unigene35723\_Sample\_011046840, Unigene23571\_Sample\_011046840, Unigene3826\_Sample\_011046840, Unigene32059\_Sample\_011046840, Unigene42977\_Sample\_011046840, Unigene37209\_Sample\_011046840, Unigene24484\_Sample\_011046840, Unigene30332\_Sample\_011046840, Unigene886\_Sample\_011046840, Unigene2053\_Sample\_011046840, Unigene42591\_Sample\_011046840, Unigene30221\_Sample\_011046840, Unigene37891\_Sample\_011046840, Unigene43457\_Sample\_011046840, Unigene14666\_Sample\_011046840, Unigene27879\_Sample\_011046840, Unigene40973\_Sample\_011046840, Unigene41654\_Sample\_011046840, Unigene43146\_Sample\_011046840, Unigene23684\_Sample\_011046840, Unigene39591\_Sample\_011046840, Unigene42683\_Sample\_011046840, Unigene29099\_Sample\_011046840, Unigene19383\_Sample\_011046840, Unigene34242\_Sample\_011046840, Unigene5289\_Sample\_011046840, Unigene37452\_Sample\_011046840, Unigene25871\_Sample\_011046840, Unigene42725\_Sample\_011046840, Unigene30573\_Sample\_011046840, Unigene34746\_Sample\_011046840, Unigene4863\_Sample\_011046840, Unigene24267\_Sample\_011046840, Unigene26273\_Sample\_011046840, Unigene33040\_Sample\_011046840, Unigene40812\_Sample\_011046840, Unigene39303\_Sample\_011046840, Unigene40787\_Sample\_011046840, Unigene22327\_Sample\_011046840, Unigene24674\_Sample\_011046840, Unigene18900\_Sample\_011046840, Unigene4494\_Sample\_011046840, Unigene36969\_Sample\_011046840, Unigene33889\_Sample\_011046840, Unigene1815\_Sample\_011046840, Unigene37365\_Sample\_011046840, Unigene25523\_Sample\_011046840, Unigene31910\_Sample\_011046840, Unigene43453\_Sample\_011046840, Unigene41479\_Sample\_011046840, Unigene14648\_Sample\_011046840, Unigene6446\_Sample\_011046840, Unigene28929\_Sample\_011046840, Unigene38951\_Sample\_011046840, Unigene13717\_Sample\_011046840, Unigene8520\_Sample\_011046840, Unigene39795\_Sample\_011046840, Unigene9027\_Sample\_011046840, Unigene26557\_Sample\_011046840, Unigene43031\_Sample\_011046840, Unigene34704\_Sample\_011046840, Unigene41868\_Sample\_011046840, Unigene42068\_Sample\_011046840, Unigene27364\_Sample\_011046840, Unigene40774\_Sample\_011046840, Unigene41561\_Sample\_011046840, Unigene7884\_Sample\_011046840, Unigene18132\_Sample\_011046840, Unigene40246\_Sample\_011046840, Unigene40731\_Sample\_011046840, Unigene37244\_Sample\_011046840, Unigene28923\_Sample\_011046840, Unigene42201\_Sample\_011046840, Unigene3161\_Sample\_011046840, Unigene40387\_Sample\_011046840, Unigene42944\_Sample\_011046840, Unigene4541\_Sample\_011046840, Unigene24509\_Sample\_011046840, Unigene35324\_Sample\_011046840, Unigene10956\_Sample\_011046840, Unigene5724\_Sample\_011046840, Unigene20549\_Sample\_011046840, Unigene7952\_Sample\_011046840, Unigene40924\_Sample\_011046840, Unigene5782\_Sample\_011046840, Unigene14078\_Sample\_011046840, Unigene35663\_Sample\_011046840, Unigene42803\_Sample\_011046840, Unigene37573\_Sample\_011046840, Unigene6894\_Sample\_011046840, Unigene31793\_Sample\_011046840, Unigene37879\_Sample\_011046840, Unigene42475\_Sample\_011046840, Unigene23365\_Sample\_011046840, Unigene39986\_Sample\_011046840, Unigene30897\_Sample\_011046840, Unigene41783\_Sample\_011046840, Unigene39416\_Sample\_011046840, Unigene34233\_Sample\_011046840, Unigene20783\_Sample\_011046840, Unigene42252\_Sample\_011046840, Unigene40728\_Sample\_011046840, Unigene31324\_Sample\_011046840, Unigene41821\_Sample\_011046840, Unigene37470\_Sample\_011046840, Unigene42530\_Sample\_011046840, Unigene40878\_Sample\_011046840, Unigene43616\_Sample\_011046840, Unigene29952\_Sample\_011046840, Unigene35996\_Sample\_011046840, Unigene43562\_Sample\_011046840, Unigene28531\_Sample\_011046840, Unigene14237\_Sample\_011046840, Unigene41778\_Sample\_011046840, Unigene2626\_Sample\_011046840, Unigene3395\_Sample\_011046840, Unigene37277\_Sample\_011046840, Unigene19433\_Sample\_011046840, Unigene9361\_Sample\_011046840, Unigene39318\_Sample\_011046840, Unigene35817\_Sample\_011046840, Unigene33515\_Sample\_011046840, Unigene31044\_Sample\_011046840, Unigene33378\_Sample\_011046840, Unigene2332\_Sample\_011046840, Unigene3164\_Sample\_011046840, Unigene42335\_Sample\_011046840, Unigene35417\_Sample\_011046840, Unigene27779\_Sample\_011046840, Unigene40490\_Sample\_011046840, Unigene24936\_Sample\_011046840, Unigene10460\_Sample\_011046840, Unigene26781\_Sample\_011046840, Unigene34355\_Sample\_011046840, Unigene40333\_Sample\_011046840, Unigene36929\_Sample\_011046840, Unigene7983\_Sample\_011046840, Unigene41606\_Sample\_011046840, Unigene32385\_Sample\_011046840, Unigene37702\_Sample\_011046840, Unigene8751\_Sample\_011046840, Unigene20612\_Sample\_011046840, Unigene43243\_Sample\_011046840, Unigene10540\_Sample\_011046840, Unigene31911\_Sample\_011046840, Unigene43525\_Sample\_011046840, Unigene4020\_Sample\_011046840, Unigene3241\_Sample\_011046840, Unigene39639\_Sample\_011046840, Unigene25328\_Sample\_011046840, Unigene22550\_Sample\_011046840, Unigene32853\_Sample\_011046840, Unigene12730\_Sample\_011046840, Unigene28259\_Sample\_011046840, Unigene37149\_Sample\_011046840, Unigene38753\_Sample\_011046840, Unigene5305\_Sample\_011046840, Unigene38000\_Sample\_011046840, Unigene35724\_Sample\_011046840, Unigene41092\_Sample\_011046840, Unigene8864\_Sample\_011046840, Unigene28670\_Sample\_011046840, Unigene12191\_Sample\_011046840, Unigene35234\_Sample\_011046840, Unigene34850\_Sample\_011046840, Unigene14154\_Sample\_011046840, Unigene32278\_Sample\_011046840, Unigene27950\_Sample\_011046840, Unigene21660\_Sample\_011046840, Unigene29386\_Sample\_011046840, Unigene15458\_Sample\_011046840, Unigene30466\_Sample\_011046840, Unigene7536\_Sample\_011046840, Unigene242\_Sample\_011046840, Unigene5710\_Sample\_011046840, Unigene34508\_Sample\_011046840, Unigene17239\_Sample\_011046840, Unigene143\_Sample\_011046840, Unigene42215\_Sample\_011046840, Unigene16751\_Sample\_011046840, Unigene33538\_Sample\_011046840, Unigene29117\_Sample\_011046840, Unigene23811\_Sample\_011046840, Unigene38199\_Sample\_011046840, Unigene7643\_Sample\_011046840, Unigene41574\_Sample\_011046840, Unigene15420\_Sample\_011046840, Unigene33085\_Sample\_011046840, Unigene40362\_Sample\_011046840, Unigene33736\_Sample\_011046840, Unigene17665\_Sample\_011046840, Unigene37299\_Sample\_011046840, Unigene28039\_Sample\_011046840, Unigene32875\_Sample\_011046840, Unigene12062\_Sample\_011046840, Unigene36669\_Sample\_011046840, Unigene42386\_Sample\_011046840, Unigene7734\_Sample\_011046840, Unigene14924\_Sample\_011046840, Unigene34509\_Sample\_011046840, Unigene36260\_Sample\_011046840, Unigene40376\_Sample\_011046840, Unigene16236\_Sample\_011046840, Unigene29599\_Sample\_011046840, Unigene38444\_Sample\_011046840, Unigene29696\_Sample\_011046840, Unigene7189\_Sample\_011046840, Unigene8155\_Sample\_011046840, Unigene39526\_Sample\_011046840, Unigene19098\_Sample\_011046840, Unigene39429\_Sample\_011046840, Unigene39339\_Sample\_011046840, Unigene15504\_Sample\_011046840, Unigene13340\_Sample\_011046840, Unigene27682\_Sample\_011046840, Unigene6624\_Sample\_011046840, Unigene40042\_Sample\_011046840, Unigene28521\_Sample\_011046840, Unigene32354\_Sample\_011046840, Unigene39248\_Sample\_011046840, Unigene42569\_Sample\_011046840, Unigene36007\_Sample\_011046840, Unigene8002\_Sample\_011046840, Unigene40462\_Sample\_011046840, Unigene25024\_Sample\_011046840, Unigene6711\_Sample\_011046840, Unigene23891\_Sample\_011046840, Unigene23122\_Sample\_011046840, Unigene37267\_Sample\_011046840, Unigene26865\_Sample\_011046840, Unigene32927\_Sample\_011046840, Unigene23169\_Sample\_011046840, Unigene33810\_Sample\_011046840, Unigene41818\_Sample\_011046840, Unigene16223\_Sample\_011046840, Unigene12911\_Sample\_011046840, Unigene41980\_Sample\_011046840, Unigene42325\_Sample\_011046840, Unigene40919\_Sample\_011046840, Unigene14503\_Sample\_011046840, Unigene22739\_Sample\_011046840, Unigene38058\_Sample\_011046840, Unigene39304\_Sample\_011046840, Unigene33699\_Sample\_011046840, Unigene36391\_Sample\_011046840, Unigene38722\_Sample\_011046840, Unigene3192\_Sample\_011046840, Unigene40469\_Sample\_011046840, Unigene12359\_Sample\_011046840, Unigene12476\_Sample\_011046840, Unigene23566\_Sample\_011046840, Unigene37124\_Sample\_011046840, Unigene32723\_Sample\_011046840, Unigene13989\_Sample\_011046840, Unigene27850\_Sample\_011046840, Unigene40826\_Sample\_011046840, Unigene32621\_Sample\_011046840, Unigene23801\_Sample\_011046840, Unigene36342\_Sample\_011046840, Unigene42634\_Sample\_011046840, Unigene29076\_Sample\_011046840, Unigene43560\_Sample\_011046840, Unigene34605\_Sample\_011046840, Unigene3752\_Sample\_011046840, Unigene35459\_Sample\_011046840, Unigene6135\_Sample\_011046840, Unigene16550\_Sample\_011046840, Unigene16850\_Sample\_011046840, Unigene13232\_Sample\_011046840, Unigene28549\_Sample\_011046840, Unigene37057\_Sample\_011046840, Unigene14853\_Sample\_011046840, Unigene42994\_Sample\_011046840, Unigene37045\_Sample\_011046840, Unigene4497\_Sample\_011046840, Unigene12675\_Sample\_011046840, Unigene188\_Sample\_011046840, Unigene6461\_Sample\_011046840, Unigene27316\_Sample\_011046840, Unigene24183\_Sample\_011046840, Unigene30537\_Sample\_011046840, Unigene43282\_Sample\_011046840, Unigene22747\_Sample\_011046840, Unigene22665\_Sample\_011046840, Unigene38851\_Sample\_011046840, Unigene20154\_Sample\_011046840, Unigene2690\_Sample\_011046840, Unigene41052\_Sample\_011046840, Unigene33392\_Sample\_011046840, Unigene16079\_Sample\_011046840, Unigene3068\_Sample\_011046840, Unigene43238\_Sample\_011046840, Unigene41943\_Sample\_011046840, Unigene37309\_Sample\_011046840, Unigene28312\_Sample\_011046840, Unigene40753\_Sample\_011046840, Unigene27006\_Sample\_011046840, Unigene20800\_Sample\_011046840, Unigene27473\_Sample\_011046840, Unigene26705\_Sample\_011046840, Unigene1620\_Sample\_011046840, Unigene31527\_Sample\_011046840, Unigene22626\_Sample\_011046840, Unigene141\_Sample\_011046840, Unigene35146\_Sample\_011046840, Unigene38900\_Sample\_011046840, Unigene8166\_Sample\_011046840, Unigene13275\_Sample\_011046840, Unigene9759\_Sample\_011046840, Unigene31035\_Sample\_011046840, Unigene21132\_Sample\_011046840, Unigene34039\_Sample\_011046840, Unigene6582\_Sample\_011046840, Unigene31560\_Sample\_011046840, Unigene40885\_Sample\_011046840, Unigene34017\_Sample\_011046840, Unigene41752\_Sample\_011046840, Unigene21415\_Sample\_011046840, Unigene38412\_Sample\_011046840, Unigene29975\_Sample\_011046840, Unigene25346\_Sample\_011046840, Unigene42297\_Sample\_011046840, Unigene23889\_Sample\_011046840, Unigene41446\_Sample\_011046840, Unigene43444\_Sample\_011046840, Unigene30704\_Sample\_011046840, Unigene30564\_Sample\_011046840, Unigene42481\_Sample\_011046840, Unigene40738\_Sample\_011046840, Unigene32589\_Sample\_011046840, Unigene7448\_Sample\_011046840, Unigene38998\_Sample\_011046840, Unigene13731\_Sample\_011046840, Unigene31084\_Sample\_011046840, Unigene5453\_Sample\_011046840, Unigene36253\_Sample\_011046840, Unigene12207\_Sample\_011046840, Unigene8026\_Sample\_011046840, Unigene41196\_Sample\_011046840, Unigene29253\_Sample\_011046840, Unigene38935\_Sample\_011046840, Unigene42550\_Sample\_011046840, Unigene6938\_Sample\_011046840, Unigene6651\_Sample\_011046840, Unigene41405\_Sample\_011046840, Unigene11522\_Sample\_011046840, Unigene28397\_Sample\_011046840, Unigene36899\_Sample\_011046840, Unigene36299\_Sample\_011046840, Unigene31914\_Sample\_011046840, Unigene6987\_Sample\_011046840, Unigene16486\_Sample\_011046840, Unigene26212\_Sample\_011046840, Unigene42202\_Sample\_011046840, Unigene40593\_Sample\_011046840, Unigene7169\_Sample\_011046840, Unigene6075\_Sample\_011046840, Unigene23026\_Sample\_011046840, Unigene2068\_Sample\_011046840, Unigene333\_Sample\_011046840, Unigene41982\_Sample\_011046840, Unigene35105\_Sample\_011046840, Unigene27013\_Sample\_011046840, Unigene30486\_Sample\_011046840, Unigene21529\_Sample\_011046840, Unigene1926\_Sample\_011046840, Unigene30435\_Sample\_011046840, Unigene8059\_Sample\_011046840, Unigene42320\_Sample\_011046840, Unigene26065\_Sample\_011046840, Unigene39582\_Sample\_011046840, Unigene3769\_Sample\_011046840, Unigene41799\_Sample\_011046840, Unigene25305\_Sample\_011046840, Unigene35170\_Sample\_011046840, Unigene19905\_Sample\_011046840, Unigene18537\_Sample\_011046840, Unigene22397\_Sample\_011046840, Unigene26110\_Sample\_011046840, Unigene20109\_Sample\_011046840, Unigene11458\_Sample\_011046840, Unigene20540\_Sample\_011046840, Unigene26932\_Sample\_011046840, Unigene31695\_Sample\_011046840, Unigene42116\_Sample\_011046840, Unigene42354\_Sample\_011046840, Unigene1456\_Sample\_011046840, Unigene43014\_Sample\_011046840, Unigene34328\_Sample\_011046840, Unigene34437\_Sample\_011046840, Unigene39254\_Sample\_011046840, Unigene15681\_Sample\_011046840, Unigene21699\_Sample\_011046840, Unigene7058\_Sample\_011046840, Unigene38329\_Sample\_011046840, Unigene41337\_Sample\_011046840, Unigene36070\_Sample\_011046840, Unigene41563\_Sample\_011046840, Unigene34874\_Sample\_011046840, Unigene39657\_Sample\_011046840, Unigene7525\_Sample\_011046840, Unigene40064\_Sample\_011046840, Unigene43080\_Sample\_011046840, Unigene39628\_Sample\_011046840, Unigene24129\_Sample\_011046840, Unigene26217\_Sample\_011046840, Unigene31662\_Sample\_011046840, Unigene13805\_Sample\_011046840, Unigene42955\_Sample\_011046840, Unigene13117\_Sample\_011046840, Unigene4807\_Sample\_011046840, Unigene15087\_Sample\_011046840, Unigene35427\_Sample\_011046840, Unigene39204\_Sample\_011046840, Unigene39588\_Sample\_011046840, Unigene5908\_Sample\_011046840, Unigene1271\_Sample\_011046840, Unigene13202\_Sample\_011046840, Unigene35008\_Sample\_011046840, Unigene11193\_Sample\_011046840, Unigene29870\_Sample\_011046840, Unigene41672\_Sample\_011046840, Unigene14816\_Sample\_011046840, Unigene6438\_Sample\_011046840, Unigene11100\_Sample\_011046840, Unigene28055\_Sample\_011046840, Unigene11043\_Sample\_011046840, Unigene11820\_Sample\_011046840, Unigene39649\_Sample\_011046840, Unigene2986\_Sample\_011046840, Unigene31576\_Sample\_011046840, Unigene43486\_Sample\_011046840, Unigene35141\_Sample\_011046840, Unigene37516\_Sample\_011046840, Unigene43218\_Sample\_011046840, Unigene18284\_Sample\_011046840, Unigene10850\_Sample\_011046840, Unigene7429\_Sample\_011046840, Unigene7825\_Sample\_011046840, Unigene3974\_Sample\_011046840, Unigene34610\_Sample\_011046840, Unigene11221\_Sample\_011046840, Unigene40602\_Sample\_011046840, Unigene43040\_Sample\_011046840, Unigene32583\_Sample\_011046840, Unigene35195\_Sample\_011046840, Unigene35735\_Sample\_011046840, Unigene31646\_Sample\_011046840, Unigene33659\_Sample\_011046840, Unigene34909\_Sample\_011046840, Unigene41427\_Sample\_011046840, Unigene6899\_Sample\_011046840, Unigene21598\_Sample\_011046840, Unigene34036\_Sample\_011046840, Unigene7221\_Sample\_011046840, Unigene42376\_Sample\_011046840, Unigene39974\_Sample\_011046840, Unigene39644\_Sample\_011046840, Unigene16618\_Sample\_011046840, Unigene3258\_Sample\_011046840, Unigene40298\_Sample\_011046840, Unigene9238\_Sample\_011046840, Unigene13322\_Sample\_011046840, Unigene34023\_Sample\_011046840, Unigene3320\_Sample\_011046840, Unigene32533\_Sample\_011046840, Unigene35568\_Sample\_011046840, Unigene34987\_Sample\_011046840, Unigene1475\_Sample\_011046840, Unigene41978\_Sample\_011046840, Unigene38773\_Sample\_011046840, Unigene4652\_Sample\_011046840, Unigene40452\_Sample\_011046840, Unigene13531\_Sample\_011046840, Unigene34137\_Sample\_011046840, Unigene29734\_Sample\_011046840, Unigene37194\_Sample\_011046840, Unigene23472\_Sample\_011046840, Unigene41524\_Sample\_011046840, Unigene19865\_Sample\_011046840, Unigene31278\_Sample\_011046840, Unigene38669\_Sample\_011046840, Unigene31320\_Sample\_011046840, Unigene38106\_Sample\_011046840, Unigene37234\_Sample\_011046840, Unigene43569\_Sample\_011046840, Unigene22770\_Sample\_011046840, Unigene39686\_Sample\_011046840, Unigene43214\_Sample\_011046840, Unigene39412\_Sample\_011046840, Unigene2864\_Sample\_011046840, Unigene34806\_Sample\_011046840, Unigene42211\_Sample\_011046840, Unigene29758\_Sample\_011046840, Unigene25902\_Sample\_011046840, Unigene37578\_Sample\_011046840, Unigene2180\_Sample\_011046840, Unigene41811\_Sample\_011046840, Unigene28270\_Sample\_011046840, Unigene7124\_Sample\_011046840, Unigene37716\_Sample\_011046840, Unigene39234\_Sample\_011046840, Unigene32740\_Sample\_011046840, Unigene8191\_Sample\_011046840, Unigene3608\_Sample\_011046840, Unigene41055\_Sample\_011046840, Unigene42939\_Sample\_011046840, Unigene40104\_Sample\_011046840, Unigene30051\_Sample\_011046840, Unigene42983\_Sample\_011046840, Unigene8633\_Sample\_011046840, Unigene24747\_Sample\_011046840, Unigene32240\_Sample\_011046840, Unigene37285\_Sample\_011046840, Unigene1389\_Sample\_011046840, Unigene5682\_Sample\_011046840, Unigene33166\_Sample\_011046840, Unigene32573\_Sample\_011046840, Unigene41250\_Sample\_011046840, Unigene8024\_Sample\_011046840, Unigene36981\_Sample\_011046840, Unigene43573\_Sample\_011046840, Unigene38113\_Sample\_011046840, Unigene35784\_Sample\_011046840, Unigene43315\_Sample\_011046840, Unigene17678\_Sample\_011046840, Unigene35174\_Sample\_011046840, Unigene3204\_Sample\_011046840, Unigene39836\_Sample\_011046840, Unigene31899\_Sample\_011046840, Unigene19860\_Sample\_011046840, Unigene41693\_Sample\_011046840, Unigene30970\_Sample\_011046840, Unigene21108\_Sample\_011046840, Unigene42142\_Sample\_011046840, Unigene40883\_Sample\_011046840, Unigene43002\_Sample\_011046840, Unigene35126\_Sample\_011046840, Unigene43003\_Sample\_011046840, Unigene39602\_Sample\_011046840, Unigene42765\_Sample\_011046840, Unigene38429\_Sample\_011046840, Unigene38841\_Sample\_011046840, Unigene10437\_Sample\_011046840, Unigene5746\_Sample\_011046840, Unigene8072\_Sample\_011046840, Unigene26556\_Sample\_011046840, Unigene31611\_Sample\_011046840, Unigene27932\_Sample\_011046840, Unigene31669\_Sample\_011046840, Unigene31198\_Sample\_011046840, Unigene43301\_Sample\_011046840, Unigene17816\_Sample\_011046840, Unigene31457\_Sample\_011046840, Unigene28065\_Sample\_011046840, Unigene21513\_Sample\_011046840, Unigene8187\_Sample\_011046840, Unigene16413\_Sample\_011046840, Unigene33061\_Sample\_011046840, Unigene37048\_Sample\_011046840, Unigene42730\_Sample\_011046840, Unigene15916\_Sample\_011046840, Unigene34917\_Sample\_011046840, Unigene31482\_Sample\_011046840, Unigene43405\_Sample\_011046840, Unigene36223\_Sample\_011046840, Unigene30033\_Sample\_011046840, Unigene16228\_Sample\_011046840, Unigene36338\_Sample\_011046840, Unigene43408\_Sample\_011046840, Unigene40895\_Sample\_011046840, Unigene6950\_Sample\_011046840, Unigene42043\_Sample\_011046840, Unigene36018\_Sample\_011046840, Unigene20869\_Sample\_011046840, Unigene16462\_Sample\_011046840, Unigene24879\_Sample\_011046840, Unigene41957\_Sample\_011046840, Unigene41383\_Sample\_011046840, Unigene34317\_Sample\_011046840, Unigene27840\_Sample\_011046840, Unigene30256\_Sample\_011046840, Unigene18542\_Sample\_011046840, Unigene3046\_Sample\_011046840, Unigene29507\_Sample\_011046840, Unigene42098\_Sample\_011046840, Unigene37486\_Sample\_011046840, Unigene32591\_Sample\_011046840, Unigene4932\_Sample\_011046840, Unigene13413\_Sample\_011046840, Unigene29334\_Sample\_011046840, Unigene43289\_Sample\_011046840, Unigene31376\_Sample\_011046840, Unigene28459\_Sample\_011046840, Unigene34268\_Sample\_011046840, Unigene27274\_Sample\_011046840, Unigene15290\_Sample\_011046840, Unigene28153\_Sample\_011046840, Unigene30083\_Sample\_011046840, Unigene39546\_Sample\_011046840, Unigene24336\_Sample\_011046840, Unigene30720\_Sample\_011046840, Unigene23318\_Sample\_011046840, Unigene42219\_Sample\_011046840, Unigene22066\_Sample\_011046840, Unigene7813\_Sample\_011046840, Unigene38232\_Sample\_011046840, Unigene42130\_Sample\_011046840, Unigene7388\_Sample\_011046840, Unigene40082\_Sample\_011046840, Unigene13841\_Sample\_011046840, Unigene1154\_Sample\_011046840, Unigene36762\_Sample\_011046840, Unigene18637\_Sample\_011046840, Unigene42645\_Sample\_011046840, Unigene23400\_Sample\_011046840, Unigene24482\_Sample\_011046840, Unigene26756\_Sample\_011046840, Unigene32130\_Sample\_011046840, Unigene33441\_Sample\_011046840, Unigene40483\_Sample\_011046840, Unigene40113\_Sample\_011046840, Unigene39900\_Sample\_011046840, Unigene18228\_Sample\_011046840, Unigene35365\_Sample\_011046840, Unigene33705\_Sample\_011046840, Unigene9985\_Sample\_011046840, Unigene6177\_Sample\_011046840, Unigene34118\_Sample\_011046840, Unigene5336\_Sample\_011046840, Unigene11684\_Sample\_011046840, Unigene36996\_Sample\_011046840, Unigene16687\_Sample\_011046840, Unigene4601\_Sample\_011046840, Unigene42166\_Sample\_011046840, Unigene5223\_Sample\_011046840, Unigene36120\_Sample\_011046840, Unigene37825\_Sample\_011046840, Unigene38826\_Sample\_011046840, Unigene36211\_Sample\_011046840, Unigene11392\_Sample\_011046840, Unigene43372\_Sample\_011046840, Unigene11686\_Sample\_011046840, Unigene34757\_Sample\_011046840, Unigene2724\_Sample\_011046840, Unigene33942\_Sample\_011046840, Unigene39734\_Sample\_011046840, Unigene39481\_Sample\_011046840, Unigene35544\_Sample\_011046840, Unigene29542\_Sample\_011046840, Unigene41433\_Sample\_011046840, Unigene34096\_Sample\_011046840, Unigene32555\_Sample\_011046840, Unigene13188\_Sample\_011046840, Unigene34205\_Sample\_011046840, Unigene6276\_Sample\_011046840, Unigene7677\_Sample\_011046840, Unigene43579\_Sample\_011046840, Unigene34048\_Sample\_011046840, Unigene2706\_Sample\_011046840, Unigene34219\_Sample\_011046840, Unigene26614\_Sample\_011046840, Unigene31898\_Sample\_011046840, Unigene28263\_Sample\_011046840, Unigene21413\_Sample\_011046840, Unigene25381\_Sample\_011046840, Unigene27385\_Sample\_011046840, Unigene38430\_Sample\_011046840, Unigene38085\_Sample\_011046840, Unigene16387\_Sample\_011046840, Unigene41917\_Sample\_011046840, Unigene33126\_Sample\_011046840, Unigene36446\_Sample\_011046840, Unigene37480\_Sample\_011046840, Unigene40479\_Sample\_011046840, Unigene38460\_Sample\_011046840, Unigene34336\_Sample\_011046840, Unigene36661\_Sample\_011046840, Unigene40495\_Sample\_011046840, Unigene19409\_Sample\_011046840, Unigene36494\_Sample\_011046840, Unigene6905\_Sample\_011046840, Unigene38491\_Sample\_011046840, Unigene28160\_Sample\_011046840, Unigene43177\_Sample\_011046840, Unigene42533\_Sample\_011046840, Unigene39609\_Sample\_011046840, Unigene31985\_Sample\_011046840, Unigene26402\_Sample\_011046840, Unigene11248\_Sample\_011046840, Unigene15043\_Sample\_011046840, Unigene15915\_Sample\_011046840, Unigene5876\_Sample\_011046840, Unigene36912\_Sample\_011046840, Unigene12550\_Sample\_011046840, Unigene36063\_Sample\_011046840, Unigene18251\_Sample\_011046840, Unigene43463\_Sample\_011046840, Unigene15017\_Sample\_011046840, Unigene41545\_Sample\_011046840, Unigene31980\_Sample\_011046840, Unigene28456\_Sample\_011046840, Unigene31283\_Sample\_011046840, Unigene38265\_Sample\_011046840, Unigene30230\_Sample\_011046840, Unigene9307\_Sample\_011046840, Unigene42403\_Sample\_011046840, Unigene28754\_Sample\_011046840, Unigene33408\_Sample\_011046840, Unigene39671\_Sample\_011046840, Unigene40741\_Sample\_011046840, Unigene43357\_Sample\_011046840, Unigene34984\_Sample\_011046840, Unigene3201\_Sample\_011046840, Unigene30293\_Sample\_011046840, Unigene1504\_Sample\_011046840, Unigene34015\_Sample\_011046840, Unigene20007\_Sample\_011046840, Unigene32180\_Sample\_011046840, Unigene2754\_Sample\_011046840, Unigene40922\_Sample\_011046840, Unigene39941\_Sample\_011046840, Unigene5991\_Sample\_011046840, Unigene20151\_Sample\_011046840, Unigene35511\_Sample\_011046840, Unigene35637\_Sample\_011046840, Unigene16641\_Sample\_011046840, Unigene39706\_Sample\_011046840, Unigene12175\_Sample\_011046840, Unigene40576\_Sample\_011046840, Unigene34977\_Sample\_011046840, Unigene43360\_Sample\_011046840, Unigene39095\_Sample\_011046840, Unigene1722\_Sample\_011046840, Unigene41292\_Sample\_011046840, Unigene38713\_Sample\_011046840, Unigene1454\_Sample\_011046840, Unigene35227\_Sample\_011046840, Unigene17098\_Sample\_011046840, Unigene34431\_Sample\_011046840, Unigene16879\_Sample\_011046840, Unigene43064\_Sample\_011046840, Unigene40520\_Sample\_011046840, Unigene2418\_Sample\_011046840, Unigene20967\_Sample\_011046840, Unigene41902\_Sample\_011046840, Unigene39983\_Sample\_011046840, Unigene12669\_Sample\_011046840, Unigene34505\_Sample\_011046840, Unigene16402\_Sample\_011046840, Unigene5094\_Sample\_011046840, Unigene20050\_Sample\_011046840, Unigene2367\_Sample\_011046840, Unigene31240\_Sample\_011046840, Unigene29471\_Sample\_011046840, Unigene13641\_Sample\_011046840, Unigene36915\_Sample\_011046840, Unigene1533\_Sample\_011046840, Unigene37963\_Sample\_011046840, Unigene5705\_Sample\_011046840, Unigene26417\_Sample\_011046840, Unigene37975\_Sample\_011046840, Unigene19272\_Sample\_011046840, Unigene26868\_Sample\_011046840, Unigene40061\_Sample\_011046840, Unigene40099\_Sample\_011046840, Unigene34573\_Sample\_011046840, Unigene8565\_Sample\_011046840, Unigene32901\_Sample\_011046840, Unigene33172\_Sample\_011046840, Unigene28488\_Sample\_011046840, Unigene33585\_Sample\_011046840, Unigene20014\_Sample\_011046840, Unigene31973\_Sample\_011046840, Unigene8102\_Sample\_011046840, Unigene12710\_Sample\_011046840, Unigene40116\_Sample\_011046840, Unigene22322\_Sample\_011046840, Unigene30400\_Sample\_011046840, Unigene27036\_Sample\_011046840, Unigene41523\_Sample\_011046840, Unigene6611\_Sample\_011046840, Unigene39017\_Sample\_011046840, Unigene36640\_Sample\_011046840, Unigene35183\_Sample\_011046840, Unigene21879\_Sample\_011046840, Unigene25369\_Sample\_011046840, Unigene34638\_Sample\_011046840, Unigene42866\_Sample\_011046840, Unigene32413\_Sample\_011046840, Unigene20370\_Sample\_011046840, Unigene14271\_Sample\_011046840, Unigene32814\_Sample\_011046840, Unigene42353\_Sample\_011046840, Unigene7507\_Sample\_011046840, Unigene7499\_Sample\_011046840, Unigene38409\_Sample\_011046840, Unigene27885\_Sample\_011046840, Unigene18168\_Sample\_011046840, Unigene11142\_Sample\_011046840, Unigene32307\_Sample\_011046840, Unigene38902\_Sample\_011046840, Unigene1881\_Sample\_011046840, Unigene21770\_Sample\_011046840, Unigene30430\_Sample\_011046840, Unigene41632\_Sample\_011046840, Unigene42022\_Sample\_011046840, Unigene40396\_Sample\_011046840, Unigene43096\_Sample\_011046840, Unigene27512\_Sample\_011046840, Unigene21541\_Sample\_011046840, Unigene7609\_Sample\_011046840, Unigene37781\_Sample\_011046840, Unigene26597\_Sample\_011046840, Unigene31886\_Sample\_011046840, Unigene5913\_Sample\_011046840, Unigene36477\_Sample\_011046840, Unigene35140\_Sample\_011046840, Unigene40836\_Sample\_011046840, Unigene33008\_Sample\_011046840, Unigene7829\_Sample\_011046840, Unigene29606\_Sample\_011046840, Unigene38794\_Sample\_011046840, Unigene40998\_Sample\_011046840, Unigene31286\_Sample\_011046840, Unigene41281\_Sample\_011046840, Unigene27683\_Sample\_011046840, Unigene5893\_Sample\_011046840, Unigene39705\_Sample\_011046840 |
| nucleus | Unigene34267\_Sample\_011046840, Unigene368\_Sample\_011046840, Unigene41910\_Sample\_011046840, Unigene40049\_Sample\_011046840, Unigene37584\_Sample\_011046840, Unigene42369\_Sample\_011046840, Unigene25871\_Sample\_011046840, Unigene42725\_Sample\_011046840, Unigene38115\_Sample\_011046840, Unigene2917\_Sample\_011046840, Unigene24267\_Sample\_011046840, Unigene42090\_Sample\_011046840, Unigene41580\_Sample\_011046840, Unigene42372\_Sample\_011046840, Unigene41153\_Sample\_011046840, Unigene26987\_Sample\_011046840, Unigene33388\_Sample\_011046840, Unigene42712\_Sample\_011046840, Unigene27428\_Sample\_011046840, Unigene36969\_Sample\_011046840, Unigene21420\_Sample\_011046840, Unigene42949\_Sample\_011046840, Unigene1815\_Sample\_011046840, Unigene37365\_Sample\_011046840, Unigene20979\_Sample\_011046840, Unigene33616\_Sample\_011046840, Unigene31910\_Sample\_011046840, Unigene43453\_Sample\_011046840, Unigene40625\_Sample\_011046840, Unigene20517\_Sample\_011046840, Unigene41318\_Sample\_011046840, Unigene29635\_Sample\_011046840, Unigene38951\_Sample\_011046840, Unigene34499\_Sample\_011046840, Unigene23767\_Sample\_011046840, Unigene20681\_Sample\_011046840, Unigene9027\_Sample\_011046840, Unigene26557\_Sample\_011046840, Unigene35314\_Sample\_011046840, Unigene1132\_Sample\_011046840, Unigene19291\_Sample\_011046840, Unigene41868\_Sample\_011046840, Unigene31238\_Sample\_011046840, Unigene20033\_Sample\_011046840, Unigene42068\_Sample\_011046840, Unigene32390\_Sample\_011046840, Unigene42762\_Sample\_011046840, Unigene40774\_Sample\_011046840, Unigene7884\_Sample\_011046840, Unigene36437\_Sample\_011046840, Unigene34960\_Sample\_011046840, Unigene6495\_Sample\_011046840, Unigene27851\_Sample\_011046840, Unigene23988\_Sample\_011046840, Unigene5190\_Sample\_011046840, Unigene38463\_Sample\_011046840, Unigene177\_Sample\_011046840, Unigene1246\_Sample\_011046840, Unigene30851\_Sample\_011046840, Unigene38504\_Sample\_011046840, Unigene33318\_Sample\_011046840, Unigene15902\_Sample\_011046840, Unigene36848\_Sample\_011046840, Unigene5724\_Sample\_011046840, Unigene5782\_Sample\_011046840, Unigene15951\_Sample\_011046840, Unigene40705\_Sample\_011046840, Unigene15791\_Sample\_011046840, Unigene18649\_Sample\_011046840, Unigene38571\_Sample\_011046840, Unigene3107\_Sample\_011046840, Unigene30831\_Sample\_011046840, Unigene42803\_Sample\_011046840, Unigene43591\_Sample\_011046840, Unigene35437\_Sample\_011046840, Unigene37505\_Sample\_011046840, Unigene24532\_Sample\_011046840, Unigene42209\_Sample\_011046840, Unigene30897\_Sample\_011046840, Unigene41783\_Sample\_011046840, Unigene24921\_Sample\_011046840, Unigene39540\_Sample\_011046840, Unigene4842\_Sample\_011046840, Unigene11967\_Sample\_011046840, Unigene37470\_Sample\_011046840, Unigene43616\_Sample\_011046840, Unigene29952\_Sample\_011046840, Unigene41104\_Sample\_011046840, Unigene37077\_Sample\_011046840, Unigene35996\_Sample\_011046840, Unigene22269\_Sample\_011046840, Unigene22041\_Sample\_011046840, Unigene4830\_Sample\_011046840, Unigene13078\_Sample\_011046840, Unigene22988\_Sample\_011046840, Unigene28531\_Sample\_011046840, Unigene37066\_Sample\_011046840, Unigene12913\_Sample\_011046840, Unigene2626\_Sample\_011046840, Unigene607\_Sample\_011046840, Unigene37277\_Sample\_011046840, Unigene35015\_Sample\_011046840, Unigene40796\_Sample\_011046840, Unigene22810\_Sample\_011046840, Unigene32907\_Sample\_011046840, Unigene26168\_Sample\_011046840, Unigene37186\_Sample\_011046840, Unigene41124\_Sample\_011046840, Unigene36893\_Sample\_011046840, Unigene29658\_Sample\_011046840, Unigene37358\_Sample\_011046840, Unigene25972\_Sample\_011046840, Unigene43324\_Sample\_011046840, Unigene24936\_Sample\_011046840, Unigene40406\_Sample\_011046840, Unigene35642\_Sample\_011046840, Unigene25570\_Sample\_011046840, Unigene22613\_Sample\_011046840, Unigene31822\_Sample\_011046840, Unigene34070\_Sample\_011046840, Unigene34355\_Sample\_011046840, Unigene41412\_Sample\_011046840, Unigene7983\_Sample\_011046840, Unigene42903\_Sample\_011046840, Unigene23636\_Sample\_011046840, Unigene29411\_Sample\_011046840, Unigene4076\_Sample\_011046840, Unigene40284\_Sample\_011046840, Unigene42453\_Sample\_011046840, Unigene39763\_Sample\_011046840, Unigene43525\_Sample\_011046840, Unigene42971\_Sample\_011046840, Unigene42995\_Sample\_011046840, Unigene29626\_Sample\_011046840, Unigene39589\_Sample\_011046840, Unigene39639\_Sample\_011046840, Unigene35033\_Sample\_011046840, Unigene32853\_Sample\_011046840, Unigene43537\_Sample\_011046840, Unigene42459\_Sample\_011046840, Unigene573\_Sample\_011046840, Unigene30608\_Sample\_011046840, Unigene38847\_Sample\_011046840, Unigene41092\_Sample\_011046840, Unigene3934\_Sample\_011046840, Unigene42127\_Sample\_011046840, Unigene8219\_Sample\_011046840, Unigene27950\_Sample\_011046840, Unigene36075\_Sample\_011046840, Unigene976\_Sample\_011046840, Unigene42609\_Sample\_011046840, Unigene17567\_Sample\_011046840, Unigene24856\_Sample\_011046840, Unigene7472\_Sample\_011046840, Unigene42215\_Sample\_011046840, Unigene37502\_Sample\_011046840, Unigene23557\_Sample\_011046840, Unigene35780\_Sample\_011046840, Unigene27260\_Sample\_011046840, Unigene33538\_Sample\_011046840, Unigene38965\_Sample\_011046840, Unigene6551\_Sample\_011046840, Unigene41339\_Sample\_011046840, Unigene41574\_Sample\_011046840, Unigene43346\_Sample\_011046840, Unigene42671\_Sample\_011046840, Unigene37931\_Sample\_011046840, Unigene7137\_Sample\_011046840, Unigene41759\_Sample\_011046840, Unigene32099\_Sample\_011046840, Unigene26324\_Sample\_011046840, Unigene18463\_Sample\_011046840, Unigene20161\_Sample\_011046840, Unigene42374\_Sample\_011046840, Unigene41997\_Sample\_011046840, Unigene35271\_Sample\_011046840, Unigene17153\_Sample\_011046840, Unigene15302\_Sample\_011046840, Unigene34509\_Sample\_011046840, Unigene38655\_Sample\_011046840, Unigene34519\_Sample\_011046840, Unigene1151\_Sample\_011046840, Unigene35743\_Sample\_011046840, Unigene8155\_Sample\_011046840, Unigene42301\_Sample\_011046840, Unigene32253\_Sample\_011046840, Unigene37637\_Sample\_011046840, Unigene27682\_Sample\_011046840, Unigene40042\_Sample\_011046840, Unigene36485\_Sample\_011046840, Unigene7727\_Sample\_011046840, Unigene28979\_Sample\_011046840, Unigene4744\_Sample\_011046840, Unigene41500\_Sample\_011046840, Unigene35913\_Sample\_011046840, Unigene42569\_Sample\_011046840, Unigene29913\_Sample\_011046840, Unigene32333\_Sample\_011046840, Unigene1549\_Sample\_011046840, Unigene41431\_Sample\_011046840, Unigene38671\_Sample\_011046840, Unigene37153\_Sample\_011046840, Unigene5087\_Sample\_011046840, Unigene34896\_Sample\_011046840, Unigene26865\_Sample\_011046840, Unigene5867\_Sample\_011046840, Unigene38770\_Sample\_011046840, Unigene39391\_Sample\_011046840, Unigene31572\_Sample\_011046840, Unigene41818\_Sample\_011046840, Unigene33680\_Sample\_011046840, Unigene39061\_Sample\_011046840, Unigene33657\_Sample\_011046840, Unigene40919\_Sample\_011046840, Unigene14503\_Sample\_011046840, Unigene37904\_Sample\_011046840, Unigene25938\_Sample\_011046840, Unigene40825\_Sample\_011046840, Unigene41263\_Sample\_011046840, Unigene25767\_Sample\_011046840, Unigene7873\_Sample\_011046840, Unigene36391\_Sample\_011046840, Unigene24420\_Sample\_011046840, Unigene42807\_Sample\_011046840, Unigene42388\_Sample\_011046840, Unigene27850\_Sample\_011046840, Unigene37214\_Sample\_011046840, Unigene32621\_Sample\_011046840, Unigene29161\_Sample\_011046840, Unigene25595\_Sample\_011046840, Unigene43260\_Sample\_011046840, Unigene37871\_Sample\_011046840, Unigene4937\_Sample\_011046840, Unigene38690\_Sample\_011046840, Unigene34605\_Sample\_011046840, Unigene8313\_Sample\_011046840, Unigene40503\_Sample\_011046840, Unigene42626\_Sample\_011046840, Unigene32292\_Sample\_011046840, Unigene24612\_Sample\_011046840, Unigene8162\_Sample\_011046840, Unigene41966\_Sample\_011046840, Unigene37653\_Sample\_011046840, Unigene4497\_Sample\_011046840, Unigene188\_Sample\_011046840, Unigene41784\_Sample\_011046840, Unigene2863\_Sample\_011046840, Unigene30817\_Sample\_011046840, Unigene30537\_Sample\_011046840, Unigene41742\_Sample\_011046840, Unigene28429\_Sample\_011046840, Unigene40088\_Sample\_011046840, Unigene43282\_Sample\_011046840, Unigene27458\_Sample\_011046840, Unigene33984\_Sample\_011046840, Unigene32791\_Sample\_011046840, Unigene28335\_Sample\_011046840, Unigene11454\_Sample\_011046840, Unigene33611\_Sample\_011046840, Unigene34669\_Sample\_011046840, Unigene33954\_Sample\_011046840, Unigene38851\_Sample\_011046840, Unigene27955\_Sample\_011046840, Unigene41631\_Sample\_011046840, Unigene33222\_Sample\_011046840, Unigene41052\_Sample\_011046840, Unigene39687\_Sample\_011046840, Unigene35066\_Sample\_011046840, Unigene34458\_Sample\_011046840, Unigene41943\_Sample\_011046840, Unigene29499\_Sample\_011046840, Unigene39458\_Sample\_011046840, Unigene40753\_Sample\_011046840, Unigene7405\_Sample\_011046840, Unigene22051\_Sample\_011046840, Unigene35895\_Sample\_011046840, Unigene40876\_Sample\_011046840, Unigene34436\_Sample\_011046840, Unigene38914\_Sample\_011046840, Unigene39224\_Sample\_011046840, Unigene1620\_Sample\_011046840, Unigene31527\_Sample\_011046840, Unigene16409\_Sample\_011046840, Unigene37462\_Sample\_011046840, Unigene7545\_Sample\_011046840, Unigene41810\_Sample\_011046840, Unigene31513\_Sample\_011046840, Unigene8166\_Sample\_011046840, Unigene8086\_Sample\_011046840, Unigene40324\_Sample\_011046840, Unigene39121\_Sample\_011046840, Unigene5383\_Sample\_011046840, Unigene42927\_Sample\_011046840, Unigene16797\_Sample\_011046840, Unigene36773\_Sample\_011046840, Unigene31560\_Sample\_011046840, Unigene40885\_Sample\_011046840, Unigene29764\_Sample\_011046840, Unigene8006\_Sample\_011046840, Unigene35547\_Sample\_011046840, Unigene21415\_Sample\_011046840, Unigene43155\_Sample\_011046840, Unigene41637\_Sample\_011046840, Unigene40427\_Sample\_011046840, Unigene34604\_Sample\_011046840, Unigene33431\_Sample\_011046840, Unigene25346\_Sample\_011046840, Unigene42230\_Sample\_011046840, Unigene35206\_Sample\_011046840, Unigene21710\_Sample\_011046840, Unigene1561\_Sample\_011046840, Unigene35692\_Sample\_011046840, Unigene7427\_Sample\_011046840, Unigene43444\_Sample\_011046840, Unigene5720\_Sample\_011046840, Unigene42659\_Sample\_011046840, Unigene9889\_Sample\_011046840, Unigene42481\_Sample\_011046840, Unigene36820\_Sample\_011046840, Unigene32589\_Sample\_011046840, Unigene42024\_Sample\_011046840, Unigene38998\_Sample\_011046840, Unigene23234\_Sample\_011046840, Unigene5564\_Sample\_011046840, Unigene29050\_Sample\_011046840, Unigene41585\_Sample\_011046840, Unigene28315\_Sample\_011046840, Unigene2740\_Sample\_011046840, Unigene5809\_Sample\_011046840, Unigene27478\_Sample\_011046840, Unigene31453\_Sample\_011046840, Unigene12207\_Sample\_011046840, Unigene39263\_Sample\_011046840, Unigene29328\_Sample\_011046840, Unigene8026\_Sample\_011046840, Unigene29253\_Sample\_011046840, Unigene42550\_Sample\_011046840, Unigene33197\_Sample\_011046840, Unigene43045\_Sample\_011046840, Unigene31648\_Sample\_011046840, Unigene32304\_Sample\_011046840, Unigene31407\_Sample\_011046840, Unigene1573\_Sample\_011046840, Unigene34929\_Sample\_011046840, Unigene11522\_Sample\_011046840, Unigene34046\_Sample\_011046840, Unigene19619\_Sample\_011046840, Unigene38501\_Sample\_011046840, Unigene42255\_Sample\_011046840, Unigene31914\_Sample\_011046840, Unigene29398\_Sample\_011046840, Unigene35051\_Sample\_011046840, Unigene34380\_Sample\_011046840, Unigene41899\_Sample\_011046840, Unigene42169\_Sample\_011046840, Unigene37939\_Sample\_011046840, Unigene41119\_Sample\_011046840, Unigene14037\_Sample\_011046840, Unigene21352\_Sample\_011046840, Unigene43278\_Sample\_011046840, Unigene28965\_Sample\_011046840, Unigene8583\_Sample\_011046840, Unigene24314\_Sample\_011046840, Unigene38104\_Sample\_011046840, Unigene35170\_Sample\_011046840, Unigene19905\_Sample\_011046840, Unigene43127\_Sample\_011046840, Unigene43217\_Sample\_011046840, Unigene22397\_Sample\_011046840, Unigene42317\_Sample\_011046840, Unigene7669\_Sample\_011046840, Unigene38071\_Sample\_011046840, Unigene11994\_Sample\_011046840, Unigene3517\_Sample\_011046840, Unigene31663\_Sample\_011046840, Unigene42354\_Sample\_011046840, Unigene22078\_Sample\_011046840, Unigene9113\_Sample\_011046840, Unigene1456\_Sample\_011046840, Unigene39254\_Sample\_011046840, Unigene38313\_Sample\_011046840, Unigene14072\_Sample\_011046840, Unigene38329\_Sample\_011046840, Unigene38416\_Sample\_011046840, Unigene39949\_Sample\_011046840, Unigene41337\_Sample\_011046840, Unigene41563\_Sample\_011046840, Unigene34874\_Sample\_011046840, Unigene38523\_Sample\_011046840, Unigene35414\_Sample\_011046840, Unigene34241\_Sample\_011046840, Unigene7525\_Sample\_011046840, Unigene32899\_Sample\_011046840, Unigene10050\_Sample\_011046840, Unigene31672\_Sample\_011046840, Unigene36813\_Sample\_011046840, Unigene39361\_Sample\_011046840, Unigene13805\_Sample\_011046840, Unigene28409\_Sample\_011046840, Unigene42104\_Sample\_011046840, Unigene20272\_Sample\_011046840, Unigene33915\_Sample\_011046840, Unigene39588\_Sample\_011046840, Unigene38449\_Sample\_011046840, Unigene36655\_Sample\_011046840, Unigene11193\_Sample\_011046840, Unigene38540\_Sample\_011046840, Unigene40676\_Sample\_011046840, Unigene36166\_Sample\_011046840, Unigene43610\_Sample\_011046840, Unigene11820\_Sample\_011046840, Unigene2986\_Sample\_011046840, Unigene43218\_Sample\_011046840, Unigene43624\_Sample\_011046840, Unigene42892\_Sample\_011046840, Unigene16898\_Sample\_011046840, Unigene11875\_Sample\_011046840, Unigene37361\_Sample\_011046840, Unigene32227\_Sample\_011046840, Unigene32583\_Sample\_011046840, Unigene35195\_Sample\_011046840, Unigene33395\_Sample\_011046840, Unigene29670\_Sample\_011046840, Unigene40511\_Sample\_011046840, Unigene7174\_Sample\_011046840, Unigene30381\_Sample\_011046840, Unigene15120\_Sample\_011046840, Unigene28451\_Sample\_011046840, Unigene7067\_Sample\_011046840, Unigene26646\_Sample\_011046840, Unigene16618\_Sample\_011046840, Unigene26435\_Sample\_011046840, Unigene42744\_Sample\_011046840, Unigene39049\_Sample\_011046840, Unigene40298\_Sample\_011046840, Unigene4108\_Sample\_011046840, Unigene33136\_Sample\_011046840, Unigene33837\_Sample\_011046840, Unigene33969\_Sample\_011046840, Unigene3320\_Sample\_011046840, Unigene30576\_Sample\_011046840, Unigene38254\_Sample\_011046840, Unigene8181\_Sample\_011046840, Unigene40019\_Sample\_011046840, Unigene4652\_Sample\_011046840, Unigene40452\_Sample\_011046840, Unigene13531\_Sample\_011046840, Unigene34137\_Sample\_011046840, Unigene11629\_Sample\_011046840, Unigene1355\_Sample\_011046840, Unigene23472\_Sample\_011046840, Unigene25963\_Sample\_011046840, Unigene41524\_Sample\_011046840, Unigene2454\_Sample\_011046840, Unigene8043\_Sample\_011046840, Unigene38669\_Sample\_011046840, Unigene6929\_Sample\_011046840, Unigene31458\_Sample\_011046840, Unigene40564\_Sample\_011046840, Unigene35699\_Sample\_011046840, Unigene36967\_Sample\_011046840, Unigene39686\_Sample\_011046840, Unigene43214\_Sample\_011046840, Unigene42211\_Sample\_011046840, Unigene13884\_Sample\_011046840, Unigene18985\_Sample\_011046840, Unigene2180\_Sample\_011046840, Unigene36114\_Sample\_011046840, Unigene40496\_Sample\_011046840, Unigene25605\_Sample\_011046840, Unigene37716\_Sample\_011046840, Unigene39020\_Sample\_011046840, Unigene32843\_Sample\_011046840, Unigene39389\_Sample\_011046840, Unigene24747\_Sample\_011046840, Unigene35359\_Sample\_011046840, Unigene1389\_Sample\_011046840, Unigene33673\_Sample\_011046840, Unigene5682\_Sample\_011046840, Unigene13786\_Sample\_011046840, Unigene26628\_Sample\_011046840, Unigene36779\_Sample\_011046840, Unigene32573\_Sample\_011046840, Unigene28616\_Sample\_011046840, Unigene1215\_Sample\_011046840, Unigene6861\_Sample\_011046840, Unigene36659\_Sample\_011046840, Unigene25450\_Sample\_011046840, Unigene15111\_Sample\_011046840, Unigene38113\_Sample\_011046840, Unigene23761\_Sample\_011046840, Unigene26170\_Sample\_011046840, Unigene23645\_Sample\_011046840, Unigene35784\_Sample\_011046840, Unigene39232\_Sample\_011046840, Unigene40661\_Sample\_011046840, Unigene43358\_Sample\_011046840, Unigene32858\_Sample\_011046840, Unigene38944\_Sample\_011046840, Unigene33508\_Sample\_011046840, Unigene39836\_Sample\_011046840, Unigene19985\_Sample\_011046840, Unigene32431\_Sample\_011046840, Unigene21033\_Sample\_011046840, Unigene42038\_Sample\_011046840, Unigene40883\_Sample\_011046840, Unigene35126\_Sample\_011046840, Unigene42765\_Sample\_011046840, Unigene33196\_Sample\_011046840, Unigene26556\_Sample\_011046840, Unigene32377\_Sample\_011046840, Unigene6912\_Sample\_011046840, Unigene43301\_Sample\_011046840, Unigene27570\_Sample\_011046840, Unigene38940\_Sample\_011046840, Unigene41283\_Sample\_011046840, Unigene8187\_Sample\_011046840, Unigene36846\_Sample\_011046840, Unigene26550\_Sample\_011046840, Unigene33061\_Sample\_011046840, Unigene31482\_Sample\_011046840, Unigene43405\_Sample\_011046840, Unigene41941\_Sample\_011046840, Unigene30033\_Sample\_011046840, Unigene16228\_Sample\_011046840, Unigene36223\_Sample\_011046840, Unigene36338\_Sample\_011046840, Unigene43066\_Sample\_011046840, Unigene10054\_Sample\_011046840, Unigene35969\_Sample\_011046840, Unigene39956\_Sample\_011046840, Unigene9950\_Sample\_011046840, Unigene22186\_Sample\_011046840, Unigene30365\_Sample\_011046840, Unigene40856\_Sample\_011046840, Unigene20869\_Sample\_011046840, Unigene16462\_Sample\_011046840, Unigene24879\_Sample\_011046840, Unigene39951\_Sample\_011046840, Unigene35452\_Sample\_011046840, Unigene40192\_Sample\_011046840, Unigene41383\_Sample\_011046840, Unigene41968\_Sample\_011046840, Unigene38699\_Sample\_011046840, Unigene20351\_Sample\_011046840, Unigene27840\_Sample\_011046840, Unigene26491\_Sample\_011046840, Unigene42105\_Sample\_011046840, Unigene41072\_Sample\_011046840, Unigene40614\_Sample\_011046840, Unigene42951\_Sample\_011046840, Unigene43494\_Sample\_011046840, Unigene37486\_Sample\_011046840, Unigene33046\_Sample\_011046840, Unigene41679\_Sample\_011046840, Unigene43289\_Sample\_011046840, Unigene41716\_Sample\_011046840, Unigene28153\_Sample\_011046840, Unigene27274\_Sample\_011046840, Unigene39546\_Sample\_011046840, Unigene443\_Sample\_011046840, Unigene1252\_Sample\_011046840, Unigene7813\_Sample\_011046840, Unigene43567\_Sample\_011046840, Unigene39420\_Sample\_011046840, Unigene24457\_Sample\_011046840, Unigene38370\_Sample\_011046840, Unigene40082\_Sample\_011046840, Unigene37533\_Sample\_011046840, Unigene42404\_Sample\_011046840, Unigene26756\_Sample\_011046840, Unigene40483\_Sample\_011046840, Unigene5316\_Sample\_011046840, Unigene23433\_Sample\_011046840, Unigene7363\_Sample\_011046840, Unigene6293\_Sample\_011046840, Unigene5336\_Sample\_011046840, Unigene42849\_Sample\_011046840, Unigene31212\_Sample\_011046840, Unigene33344\_Sample\_011046840, Unigene34158\_Sample\_011046840, Unigene39032\_Sample\_011046840, Unigene4601\_Sample\_011046840, Unigene42166\_Sample\_011046840, Unigene5223\_Sample\_011046840, Unigene27358\_Sample\_011046840, Unigene38826\_Sample\_011046840, Unigene36211\_Sample\_011046840, Unigene30604\_Sample\_011046840, Unigene43372\_Sample\_011046840, Unigene41588\_Sample\_011046840, Unigene39734\_Sample\_011046840, Unigene8605\_Sample\_011046840, Unigene20430\_Sample\_011046840, Unigene35544\_Sample\_011046840, Unigene29542\_Sample\_011046840, Unigene35307\_Sample\_011046840, Unigene37359\_Sample\_011046840, Unigene11853\_Sample\_011046840, Unigene34657\_Sample\_011046840, Unigene42286\_Sample\_011046840, Unigene43197\_Sample\_011046840, Unigene26614\_Sample\_011046840, Unigene15696\_Sample\_011046840, Unigene29131\_Sample\_011046840, Unigene38085\_Sample\_011046840, Unigene43521\_Sample\_011046840, Unigene34785\_Sample\_011046840, Unigene36446\_Sample\_011046840, Unigene26517\_Sample\_011046840, Unigene30715\_Sample\_011046840, Unigene37937\_Sample\_011046840, Unigene14511\_Sample\_011046840, Unigene40495\_Sample\_011046840, Unigene8171\_Sample\_011046840, Unigene12521\_Sample\_011046840, Unigene27924\_Sample\_011046840, Unigene32118\_Sample\_011046840, Unigene38491\_Sample\_011046840, Unigene42533\_Sample\_011046840, Unigene26402\_Sample\_011046840, Unigene41114\_Sample\_011046840, Unigene11248\_Sample\_011046840, Unigene24518\_Sample\_011046840, Unigene27725\_Sample\_011046840, Unigene42103\_Sample\_011046840, Unigene40065\_Sample\_011046840, Unigene41562\_Sample\_011046840, Unigene28822\_Sample\_011046840, Unigene22122\_Sample\_011046840, Unigene32717\_Sample\_011046840, Unigene36063\_Sample\_011046840, Unigene11179\_Sample\_011046840, Unigene13980\_Sample\_011046840, Unigene557\_Sample\_011046840, Unigene28456\_Sample\_011046840, Unigene38990\_Sample\_011046840, Unigene13197\_Sample\_011046840, Unigene15140\_Sample\_011046840, Unigene31283\_Sample\_011046840, Unigene14307\_Sample\_011046840, Unigene38265\_Sample\_011046840, Unigene5097\_Sample\_011046840, Unigene36936\_Sample\_011046840, Unigene7891\_Sample\_011046840, Unigene42403\_Sample\_011046840, Unigene31532\_Sample\_011046840, Unigene40741\_Sample\_011046840, Unigene8218\_Sample\_011046840, Unigene3201\_Sample\_011046840, Unigene12130\_Sample\_011046840, Unigene41792\_Sample\_011046840, Unigene4723\_Sample\_011046840, Unigene41441\_Sample\_011046840, Unigene5991\_Sample\_011046840, Unigene25874\_Sample\_011046840, Unigene20151\_Sample\_011046840, Unigene40424\_Sample\_011046840, Unigene15713\_Sample\_011046840, Unigene16641\_Sample\_011046840, Unigene23108\_Sample\_011046840, Unigene14623\_Sample\_011046840, Unigene43374\_Sample\_011046840, Unigene28061\_Sample\_011046840, Unigene1722\_Sample\_011046840, Unigene32375\_Sample\_011046840, Unigene27233\_Sample\_011046840, Unigene43427\_Sample\_011046840, Unigene43249\_Sample\_011046840, Unigene40411\_Sample\_011046840, Unigene6209\_Sample\_011046840, Unigene23390\_Sample\_011046840, Unigene25298\_Sample\_011046840, Unigene37708\_Sample\_011046840, Unigene35421\_Sample\_011046840, Unigene30708\_Sample\_011046840, Unigene31889\_Sample\_011046840, Unigene41902\_Sample\_011046840, Unigene37995\_Sample\_011046840, Unigene42622\_Sample\_011046840, Unigene8213\_Sample\_011046840, Unigene6608\_Sample\_011046840, Unigene42517\_Sample\_011046840, Unigene36915\_Sample\_011046840, Unigene37414\_Sample\_011046840, Unigene20838\_Sample\_011046840, Unigene35883\_Sample\_011046840, Unigene26417\_Sample\_011046840, Unigene23630\_Sample\_011046840, Unigene36203\_Sample\_011046840, Unigene33982\_Sample\_011046840, Unigene30151\_Sample\_011046840, Unigene30963\_Sample\_011046840, Unigene42653\_Sample\_011046840, Unigene1920\_Sample\_011046840, Unigene2470\_Sample\_011046840, Unigene3842\_Sample\_011046840, Unigene12743\_Sample\_011046840, Unigene28488\_Sample\_011046840, Unigene11516\_Sample\_011046840, Unigene641\_Sample\_011046840, Unigene39750\_Sample\_011046840, Unigene17719\_Sample\_011046840, Unigene11707\_Sample\_011046840, Unigene31973\_Sample\_011046840, Unigene10770\_Sample\_011046840, Unigene22560\_Sample\_011046840, Unigene40116\_Sample\_011046840, Unigene25915\_Sample\_011046840, Unigene34625\_Sample\_011046840, Unigene23890\_Sample\_011046840, Unigene26572\_Sample\_011046840, Unigene27036\_Sample\_011046840, Unigene35348\_Sample\_011046840, Unigene41523\_Sample\_011046840, Unigene39017\_Sample\_011046840, Unigene41125\_Sample\_011046840, Unigene42866\_Sample\_011046840, Unigene4920\_Sample\_011046840, Unigene40839\_Sample\_011046840, Unigene30518\_Sample\_011046840, Unigene7507\_Sample\_011046840, Unigene13336\_Sample\_011046840, Unigene39732\_Sample\_011046840, Unigene32923\_Sample\_011046840, Unigene8142\_Sample\_011046840, Unigene38902\_Sample\_011046840, Unigene37684\_Sample\_011046840, Unigene36458\_Sample\_011046840, Unigene5846\_Sample\_011046840, Unigene36991\_Sample\_011046840, Unigene42022\_Sample\_011046840, Unigene33105\_Sample\_011046840, Unigene2066\_Sample\_011046840, Unigene43096\_Sample\_011046840, Unigene39979\_Sample\_011046840, Unigene37781\_Sample\_011046840, Unigene37908\_Sample\_011046840, Unigene30332\_Sample\_011046840, Unigene31886\_Sample\_011046840, Unigene40449\_Sample\_011046840, Unigene30994\_Sample\_011046840, Unigene29198\_Sample\_011046840, Unigene42591\_Sample\_011046840, Unigene35140\_Sample\_011046840, Unigene33527\_Sample\_011046840, Unigene43146\_Sample\_011046840, Unigene39406\_Sample\_011046840, Unigene39705\_Sample\_011046840, Unigene34028\_Sample\_011046840, Unigene34686\_Sample\_011046840, Unigene39889\_Sample\_011046840, Unigene39591\_Sample\_011046840, Unigene39604\_Sample\_011046840 |
| nuclear chromosome | Unigene38463\_Sample\_011046840, Unigene5383\_Sample\_011046840, Unigene22810\_Sample\_011046840, Unigene28409\_Sample\_011046840, Unigene25298\_Sample\_011046840, Unigene40049\_Sample\_011046840, Unigene8006\_Sample\_011046840, Unigene25450\_Sample\_011046840, Unigene42866\_Sample\_011046840, Unigene38540\_Sample\_011046840, Unigene41562\_Sample\_011046840, Unigene41339\_Sample\_011046840, Unigene31572\_Sample\_011046840, Unigene39836\_Sample\_011046840, Unigene27358\_Sample\_011046840, Unigene41588\_Sample\_011046840, Unigene36063\_Sample\_011046840, Unigene33611\_Sample\_011046840, Unigene31822\_Sample\_011046840, Unigene40614\_Sample\_011046840, Unigene35699\_Sample\_011046840, Unigene41052\_Sample\_011046840, Unigene42374\_Sample\_011046840, Unigene13197\_Sample\_011046840, Unigene2740\_Sample\_011046840, Unigene27478\_Sample\_011046840, Unigene40753\_Sample\_011046840, Unigene11967\_Sample\_011046840, Unigene35895\_Sample\_011046840, Unigene34436\_Sample\_011046840, Unigene15120\_Sample\_011046840, Unigene28451\_Sample\_011046840, Unigene4723\_Sample\_011046840, Unigene42459\_Sample\_011046840, Unigene32304\_Sample\_011046840, Unigene3842\_Sample\_011046840, Unigene20033\_Sample\_011046840, Unigene16618\_Sample\_011046840, Unigene41104\_Sample\_011046840, Unigene41337\_Sample\_011046840, Unigene38690\_Sample\_011046840, Unigene12913\_Sample\_011046840, Unigene42626\_Sample\_011046840, Unigene32118\_Sample\_011046840 |
| clathrin-coated vesicle | Unigene43390\_Sample\_011046840, Unigene21362\_Sample\_011046840, Unigene39556\_Sample\_011046840, Unigene39063\_Sample\_011046840, Unigene32695\_Sample\_011046840, Unigene28676\_Sample\_011046840, Unigene28124\_Sample\_011046840, Unigene4807\_Sample\_011046840, Unigene31044\_Sample\_011046840, Unigene34041\_Sample\_011046840, Unigene41424\_Sample\_011046840, Unigene31743\_Sample\_011046840, Unigene4520\_Sample\_011046840, Unigene880\_Sample\_011046840, Unigene4849\_Sample\_011046840, Unigene34932\_Sample\_011046840, Unigene40648\_Sample\_011046840, Unigene42157\_Sample\_011046840, Unigene41850\_Sample\_011046840, Unigene38314\_Sample\_011046840, Unigene35507\_Sample\_011046840, Unigene11853\_Sample\_011046840, Unigene7868\_Sample\_011046840, Unigene36253\_Sample\_011046840, Unigene18477\_Sample\_011046840, Unigene36084\_Sample\_011046840, Unigene7287\_Sample\_011046840, Unigene41653\_Sample\_011046840, Unigene6899\_Sample\_011046840, Unigene35086\_Sample\_011046840, Unigene21040\_Sample\_011046840, Unigene39725\_Sample\_011046840, Unigene35284\_Sample\_011046840, Unigene6457\_Sample\_011046840, Unigene24259\_Sample\_011046840, Unigene10332\_Sample\_011046840, Unigene37048\_Sample\_011046840, Unigene18500\_Sample\_011046840, Unigene35485\_Sample\_011046840, Unigene39179\_Sample\_011046840, Unigene41158\_Sample\_011046840, Unigene3934\_Sample\_011046840, Unigene9112\_Sample\_011046840, Unigene30611\_Sample\_011046840, Unigene29386\_Sample\_011046840, Unigene30678\_Sample\_011046840, Unigene26402\_Sample\_011046840, Unigene11899\_Sample\_011046840, Unigene43408\_Sample\_011046840, Unigene4101\_Sample\_011046840, Unigene37933\_Sample\_011046840, Unigene38201\_Sample\_011046840, Unigene4481\_Sample\_011046840, Unigene8019\_Sample\_011046840, Unigene39866\_Sample\_011046840, Unigene7589\_Sample\_011046840, Unigene35690\_Sample\_011046840, Unigene6784\_Sample\_011046840, Unigene5464\_Sample\_011046840, Unigene27885\_Sample\_011046840, Unigene42320\_Sample\_011046840, Unigene35396\_Sample\_011046840, Unigene38106\_Sample\_011046840, Unigene37451\_Sample\_011046840, Unigene41545\_Sample\_011046840, Unigene35797\_Sample\_011046840, Unigene31980\_Sample\_011046840, Unigene35723\_Sample\_011046840, Unigene41128\_Sample\_011046840, Unigene33692\_Sample\_011046840, Unigene28459\_Sample\_011046840, Unigene37469\_Sample\_011046840, Unigene14007\_Sample\_011046840, Unigene34844\_Sample\_011046840, Unigene7058\_Sample\_011046840, Unigene3608\_Sample\_011046840, Unigene29255\_Sample\_011046840, Unigene35486\_Sample\_011046840, Unigene43099\_Sample\_011046840, Unigene12175\_Sample\_011046840, Unigene18382\_Sample\_011046840, Unigene32240\_Sample\_011046840, Unigene13275\_Sample\_011046840 |
| intracellular membrane-bounded organelle | Unigene43390\_Sample\_011046840, Unigene10428\_Sample\_011046840, Unigene29380\_Sample\_011046840, Unigene368\_Sample\_011046840, Unigene6640\_Sample\_011046840, Unigene37021\_Sample\_011046840, Unigene41047\_Sample\_011046840, Unigene37093\_Sample\_011046840, Unigene34041\_Sample\_011046840, Unigene39952\_Sample\_011046840, Unigene34674\_Sample\_011046840, Unigene28519\_Sample\_011046840, Unigene41867\_Sample\_011046840, Unigene37584\_Sample\_011046840, Unigene23713\_Sample\_011046840, Unigene5341\_Sample\_011046840, Unigene5625\_Sample\_011046840, Unigene33631\_Sample\_011046840, Unigene39857\_Sample\_011046840, Unigene4650\_Sample\_011046840, Unigene7455\_Sample\_011046840, Unigene42372\_Sample\_011046840, Unigene15271\_Sample\_011046840, Unigene42642\_Sample\_011046840, Unigene33388\_Sample\_011046840, Unigene35265\_Sample\_011046840, Unigene42712\_Sample\_011046840, Unigene21420\_Sample\_011046840, Unigene40446\_Sample\_011046840, Unigene28087\_Sample\_011046840, Unigene32526\_Sample\_011046840, Unigene42508\_Sample\_011046840, Unigene2135\_Sample\_011046840, Unigene34971\_Sample\_011046840, Unigene30321\_Sample\_011046840, Unigene29635\_Sample\_011046840, Unigene43070\_Sample\_011046840, Unigene37375\_Sample\_011046840, Unigene40497\_Sample\_011046840, Unigene39997\_Sample\_011046840, Unigene24030\_Sample\_011046840, Unigene10975\_Sample\_011046840, Unigene7475\_Sample\_011046840, Unigene4147\_Sample\_011046840, Unigene88\_Sample\_011046840, Unigene1132\_Sample\_011046840, Unigene19291\_Sample\_011046840, Unigene31238\_Sample\_011046840, Unigene20033\_Sample\_011046840, Unigene2743\_Sample\_011046840, Unigene2196\_Sample\_011046840, Unigene25680\_Sample\_011046840, Unigene32390\_Sample\_011046840, Unigene38610\_Sample\_011046840, Unigene24844\_Sample\_011046840, Unigene19368\_Sample\_011046840, Unigene35531\_Sample\_011046840, Unigene4570\_Sample\_011046840, Unigene43241\_Sample\_011046840, Unigene27851\_Sample\_011046840, Unigene43118\_Sample\_011046840, Unigene38179\_Sample\_011046840, Unigene20209\_Sample\_011046840, Unigene38463\_Sample\_011046840, Unigene42371\_Sample\_011046840, Unigene30678\_Sample\_011046840, Unigene42381\_Sample\_011046840, Unigene14294\_Sample\_011046840, Unigene36764\_Sample\_011046840, Unigene14077\_Sample\_011046840, Unigene33318\_Sample\_011046840, Unigene2419\_Sample\_011046840, Unigene38403\_Sample\_011046840, Unigene7904\_Sample\_011046840, Unigene27749\_Sample\_011046840, Unigene3107\_Sample\_011046840, Unigene30831\_Sample\_011046840, Unigene38560\_Sample\_011046840, Unigene5804\_Sample\_011046840, Unigene41107\_Sample\_011046840, Unigene24103\_Sample\_011046840, Unigene10585\_Sample\_011046840, Unigene12511\_Sample\_011046840, Unigene7468\_Sample\_011046840, Unigene893\_Sample\_011046840, Unigene38321\_Sample\_011046840, Unigene39349\_Sample\_011046840, Unigene12001\_Sample\_011046840, Unigene4204\_Sample\_011046840, Unigene42153\_Sample\_011046840, Unigene23870\_Sample\_011046840, Unigene19033\_Sample\_011046840, Unigene25022\_Sample\_011046840, Unigene31067\_Sample\_011046840, Unigene11967\_Sample\_011046840, Unigene21399\_Sample\_011046840, Unigene5389\_Sample\_011046840, Unigene33001\_Sample\_011046840, Unigene38464\_Sample\_011046840, Unigene13449\_Sample\_011046840, Unigene22269\_Sample\_011046840, Unigene22041\_Sample\_011046840, Unigene4830\_Sample\_011046840, Unigene21911\_Sample\_011046840, Unigene9326\_Sample\_011046840, Unigene22988\_Sample\_011046840, Unigene3983\_Sample\_011046840, Unigene12913\_Sample\_011046840, Unigene34035\_Sample\_011046840, Unigene36028\_Sample\_011046840, Unigene28548\_Sample\_011046840, Unigene25830\_Sample\_011046840, Unigene16538\_Sample\_011046840, Unigene19915\_Sample\_011046840, Unigene39197\_Sample\_011046840, Unigene38605\_Sample\_011046840, Unigene31274\_Sample\_011046840, Unigene12101\_Sample\_011046840, Unigene4094\_Sample\_011046840, Unigene22810\_Sample\_011046840, Unigene14350\_Sample\_011046840, Unigene40478\_Sample\_011046840, Unigene6665\_Sample\_011046840, Unigene37582\_Sample\_011046840, Unigene36893\_Sample\_011046840, Unigene23457\_Sample\_011046840, Unigene41939\_Sample\_011046840, Unigene27300\_Sample\_011046840, Unigene27232\_Sample\_011046840, Unigene880\_Sample\_011046840, Unigene18239\_Sample\_011046840, Unigene4578\_Sample\_011046840, Unigene43324\_Sample\_011046840, Unigene40406\_Sample\_011046840, Unigene24953\_Sample\_011046840, Unigene18001\_Sample\_011046840, Unigene12328\_Sample\_011046840, Unigene33106\_Sample\_011046840, Unigene41412\_Sample\_011046840, Unigene42453\_Sample\_011046840, Unigene42971\_Sample\_011046840, Unigene42395\_Sample\_011046840, Unigene39502\_Sample\_011046840, Unigene6724\_Sample\_011046840, Unigene28730\_Sample\_011046840, Unigene35033\_Sample\_011046840, Unigene34460\_Sample\_011046840, Unigene41357\_Sample\_011046840, Unigene41218\_Sample\_011046840, Unigene20251\_Sample\_011046840, Unigene43537\_Sample\_011046840, Unigene27721\_Sample\_011046840, Unigene41048\_Sample\_011046840, Unigene41135\_Sample\_011046840, Unigene22924\_Sample\_011046840, Unigene4498\_Sample\_011046840, Unigene38847\_Sample\_011046840, Unigene26711\_Sample\_011046840, Unigene37389\_Sample\_011046840, Unigene3415\_Sample\_011046840, Unigene32337\_Sample\_011046840, Unigene5388\_Sample\_011046840, Unigene42724\_Sample\_011046840, Unigene30184\_Sample\_011046840, Unigene25515\_Sample\_011046840, Unigene43261\_Sample\_011046840, Unigene9112\_Sample\_011046840, Unigene8219\_Sample\_011046840, Unigene41425\_Sample\_011046840, Unigene18719\_Sample\_011046840, Unigene43039\_Sample\_011046840, Unigene21949\_Sample\_011046840, Unigene976\_Sample\_011046840, Unigene11948\_Sample\_011046840, Unigene42609\_Sample\_011046840, Unigene17567\_Sample\_011046840, Unigene37502\_Sample\_011046840, Unigene42125\_Sample\_011046840, Unigene30343\_Sample\_011046840, Unigene38965\_Sample\_011046840, Unigene31553\_Sample\_011046840, Unigene31038\_Sample\_011046840, Unigene29171\_Sample\_011046840, Unigene41743\_Sample\_011046840, Unigene24814\_Sample\_011046840, Unigene42671\_Sample\_011046840, Unigene37931\_Sample\_011046840, Unigene41759\_Sample\_011046840, Unigene26324\_Sample\_011046840, Unigene865\_Sample\_011046840, Unigene20161\_Sample\_011046840, Unigene42374\_Sample\_011046840, Unigene23958\_Sample\_011046840, Unigene39024\_Sample\_011046840, Unigene40141\_Sample\_011046840, Unigene37680\_Sample\_011046840, Unigene40045\_Sample\_011046840, Unigene25202\_Sample\_011046840, Unigene41161\_Sample\_011046840, Unigene34519\_Sample\_011046840, Unigene1151\_Sample\_011046840, Unigene21061\_Sample\_011046840, Unigene38222\_Sample\_011046840, Unigene35917\_Sample\_011046840, Unigene29051\_Sample\_011046840, Unigene522\_Sample\_011046840, Unigene42301\_Sample\_011046840, Unigene15944\_Sample\_011046840, Unigene2457\_Sample\_011046840, Unigene18087\_Sample\_011046840, Unigene43099\_Sample\_011046840, Unigene1344\_Sample\_011046840, Unigene42759\_Sample\_011046840, Unigene40217\_Sample\_011046840, Unigene2592\_Sample\_011046840, Unigene21362\_Sample\_011046840, Unigene35200\_Sample\_011046840, Unigene41500\_Sample\_011046840, Unigene13035\_Sample\_011046840, Unigene43379\_Sample\_011046840, Unigene35913\_Sample\_011046840, Unigene32333\_Sample\_011046840, Unigene32679\_Sample\_011046840, Unigene40216\_Sample\_011046840, Unigene41431\_Sample\_011046840, Unigene42859\_Sample\_011046840, Unigene37153\_Sample\_011046840, Unigene19499\_Sample\_011046840, Unigene7625\_Sample\_011046840, Unigene18272\_Sample\_011046840, Unigene32197\_Sample\_011046840, Unigene32957\_Sample\_011046840, Unigene38737\_Sample\_011046840, Unigene33680\_Sample\_011046840, Unigene37178\_Sample\_011046840, Unigene39096\_Sample\_011046840, Unigene23285\_Sample\_011046840, Unigene25343\_Sample\_011046840, Unigene39752\_Sample\_011046840, Unigene23186\_Sample\_011046840, Unigene2780\_Sample\_011046840, Unigene43606\_Sample\_011046840, Unigene38853\_Sample\_011046840, Unigene41263\_Sample\_011046840, Unigene18600\_Sample\_011046840, Unigene7873\_Sample\_011046840, Unigene33171\_Sample\_011046840, Unigene14498\_Sample\_011046840, Unigene42807\_Sample\_011046840, Unigene32767\_Sample\_011046840, Unigene42388\_Sample\_011046840, Unigene21040\_Sample\_011046840, Unigene30889\_Sample\_011046840, Unigene37714\_Sample\_011046840, Unigene19369\_Sample\_011046840, Unigene37214\_Sample\_011046840, Unigene35063\_Sample\_011046840, Unigene2861\_Sample\_011046840, Unigene41074\_Sample\_011046840, Unigene4937\_Sample\_011046840, Unigene3914\_Sample\_011046840, Unigene16153\_Sample\_011046840, Unigene38690\_Sample\_011046840, Unigene7028\_Sample\_011046840, Unigene35755\_Sample\_011046840, Unigene8313\_Sample\_011046840, Unigene32292\_Sample\_011046840, Unigene39014\_Sample\_011046840, Unigene10043\_Sample\_011046840, Unigene39286\_Sample\_011046840, Unigene1311\_Sample\_011046840, Unigene26740\_Sample\_011046840, Unigene40853\_Sample\_011046840, Unigene37870\_Sample\_011046840, Unigene40018\_Sample\_011046840, Unigene40545\_Sample\_011046840, Unigene30817\_Sample\_011046840, Unigene31401\_Sample\_011046840, Unigene41742\_Sample\_011046840, Unigene28429\_Sample\_011046840, Unigene40523\_Sample\_011046840, Unigene33611\_Sample\_011046840, Unigene32108\_Sample\_011046840, Unigene41631\_Sample\_011046840, Unigene30934\_Sample\_011046840, Unigene3206\_Sample\_011046840, Unigene39687\_Sample\_011046840, Unigene38386\_Sample\_011046840, Unigene7097\_Sample\_011046840, Unigene1884\_Sample\_011046840, Unigene34458\_Sample\_011046840, Unigene7405\_Sample\_011046840, Unigene31007\_Sample\_011046840, Unigene22051\_Sample\_011046840, Unigene40876\_Sample\_011046840, Unigene19732\_Sample\_011046840, Unigene35650\_Sample\_011046840, Unigene39854\_Sample\_011046840, Unigene22177\_Sample\_011046840, Unigene41826\_Sample\_011046840, Unigene33927\_Sample\_011046840, Unigene6653\_Sample\_011046840, Unigene32146\_Sample\_011046840, Unigene37462\_Sample\_011046840, Unigene10704\_Sample\_011046840, Unigene7521\_Sample\_011046840, Unigene36759\_Sample\_011046840, Unigene27239\_Sample\_011046840, Unigene43135\_Sample\_011046840, Unigene10229\_Sample\_011046840, Unigene34663\_Sample\_011046840, Unigene36973\_Sample\_011046840, Unigene34833\_Sample\_011046840, Unigene37242\_Sample\_011046840, Unigene39749\_Sample\_011046840, Unigene30058\_Sample\_011046840, Unigene32101\_Sample\_011046840, Unigene40324\_Sample\_011046840, Unigene39121\_Sample\_011046840, Unigene5383\_Sample\_011046840, Unigene42927\_Sample\_011046840, Unigene20798\_Sample\_011046840, Unigene36773\_Sample\_011046840, Unigene30089\_Sample\_011046840, Unigene6753\_Sample\_011046840, Unigene14139\_Sample\_011046840, Unigene8006\_Sample\_011046840, Unigene40979\_Sample\_011046840, Unigene40427\_Sample\_011046840, Unigene34604\_Sample\_011046840, Unigene2829\_Sample\_011046840, Unigene33029\_Sample\_011046840, Unigene8255\_Sample\_011046840, Unigene27359\_Sample\_011046840, Unigene41487\_Sample\_011046840, Unigene32157\_Sample\_011046840, Unigene42429\_Sample\_011046840, Unigene21710\_Sample\_011046840, Unigene40648\_Sample\_011046840, Unigene42157\_Sample\_011046840, Unigene43475\_Sample\_011046840, Unigene1561\_Sample\_011046840, Unigene5909\_Sample\_011046840, Unigene30384\_Sample\_011046840, Unigene30201\_Sample\_011046840, Unigene16873\_Sample\_011046840, Unigene38074\_Sample\_011046840, Unigene36820\_Sample\_011046840, Unigene3753\_Sample\_011046840, Unigene33714\_Sample\_011046840, Unigene5564\_Sample\_011046840, Unigene43304\_Sample\_011046840, Unigene29050\_Sample\_011046840, Unigene15148\_Sample\_011046840, Unigene34885\_Sample\_011046840, Unigene27478\_Sample\_011046840, Unigene34174\_Sample\_011046840, Unigene4032\_Sample\_011046840, Unigene41747\_Sample\_011046840, Unigene42706\_Sample\_011046840, Unigene2963\_Sample\_011046840, Unigene39263\_Sample\_011046840, Unigene29328\_Sample\_011046840, Unigene3538\_Sample\_011046840, Unigene33197\_Sample\_011046840, Unigene32426\_Sample\_011046840, Unigene43045\_Sample\_011046840, Unigene7925\_Sample\_011046840, Unigene32304\_Sample\_011046840, Unigene8203\_Sample\_011046840, Unigene1573\_Sample\_011046840, Unigene38680\_Sample\_011046840, Unigene32944\_Sample\_011046840, Unigene33852\_Sample\_011046840, Unigene38501\_Sample\_011046840, Unigene10921\_Sample\_011046840, Unigene42557\_Sample\_011046840, Unigene15163\_Sample\_011046840, Unigene37223\_Sample\_011046840, Unigene25628\_Sample\_011046840, Unigene23926\_Sample\_011046840, Unigene16252\_Sample\_011046840, Unigene6845\_Sample\_011046840, Unigene39264\_Sample\_011046840, Unigene40761\_Sample\_011046840, Unigene42169\_Sample\_011046840, Unigene40541\_Sample\_011046840, Unigene37939\_Sample\_011046840, Unigene41399\_Sample\_011046840, Unigene40590\_Sample\_011046840, Unigene42075\_Sample\_011046840, Unigene42241\_Sample\_011046840, Unigene21120\_Sample\_011046840, Unigene35690\_Sample\_011046840, Unigene12818\_Sample\_011046840, Unigene40961\_Sample\_011046840, Unigene33706\_Sample\_011046840, Unigene5464\_Sample\_011046840, Unigene29348\_Sample\_011046840, Unigene42276\_Sample\_011046840, Unigene28965\_Sample\_011046840, Unigene25584\_Sample\_011046840, Unigene40068\_Sample\_011046840, Unigene38031\_Sample\_011046840, Unigene35632\_Sample\_011046840, Unigene37162\_Sample\_011046840, Unigene2808\_Sample\_011046840, Unigene39700\_Sample\_011046840, Unigene34416\_Sample\_011046840, Unigene8583\_Sample\_011046840, Unigene972\_Sample\_011046840, Unigene41191\_Sample\_011046840, Unigene26022\_Sample\_011046840, Unigene38774\_Sample\_011046840, Unigene36461\_Sample\_011046840, Unigene38393\_Sample\_011046840, Unigene21226\_Sample\_011046840, Unigene38071\_Sample\_011046840, Unigene33115\_Sample\_011046840, Unigene31663\_Sample\_011046840, Unigene38550\_Sample\_011046840, Unigene41037\_Sample\_011046840, Unigene11726\_Sample\_011046840, Unigene14072\_Sample\_011046840, Unigene34844\_Sample\_011046840, Unigene5034\_Sample\_011046840, Unigene38761\_Sample\_011046840, Unigene38416\_Sample\_011046840, Unigene19411\_Sample\_011046840, Unigene32050\_Sample\_011046840, Unigene39949\_Sample\_011046840, Unigene37483\_Sample\_011046840, Unigene15222\_Sample\_011046840, Unigene41600\_Sample\_011046840, Unigene6401\_Sample\_011046840, Unigene37785\_Sample\_011046840, Unigene38523\_Sample\_011046840, Unigene34241\_Sample\_011046840, Unigene29391\_Sample\_011046840, Unigene37310\_Sample\_011046840, Unigene1085\_Sample\_011046840, Unigene26624\_Sample\_011046840, Unigene43441\_Sample\_011046840, Unigene29269\_Sample\_011046840, Unigene14018\_Sample\_011046840, Unigene17267\_Sample\_011046840, Unigene31672\_Sample\_011046840, Unigene33597\_Sample\_011046840, Unigene14882\_Sample\_011046840, Unigene13721\_Sample\_011046840, Unigene11049\_Sample\_011046840, Unigene42104\_Sample\_011046840, Unigene43470\_Sample\_011046840, Unigene33915\_Sample\_011046840, Unigene24938\_Sample\_011046840, Unigene36655\_Sample\_011046840, Unigene453\_Sample\_011046840, Unigene42187\_Sample\_011046840, Unigene35644\_Sample\_011046840, Unigene37629\_Sample\_011046840, Unigene36166\_Sample\_011046840, Unigene40915\_Sample\_011046840, Unigene8290\_Sample\_011046840, Unigene40868\_Sample\_011046840, Unigene40283\_Sample\_011046840, Unigene42264\_Sample\_011046840, Unigene991\_Sample\_011046840, Unigene38314\_Sample\_011046840, Unigene42160\_Sample\_011046840, Unigene42892\_Sample\_011046840, Unigene39443\_Sample\_011046840, Unigene37361\_Sample\_011046840, Unigene32227\_Sample\_011046840, Unigene24453\_Sample\_011046840, Unigene36896\_Sample\_011046840, Unigene28006\_Sample\_011046840, Unigene19793\_Sample\_011046840, Unigene15047\_Sample\_011046840, Unigene8223\_Sample\_011046840, Unigene42923\_Sample\_011046840, Unigene7174\_Sample\_011046840, Unigene30381\_Sample\_011046840, Unigene31928\_Sample\_011046840, Unigene25793\_Sample\_011046840, Unigene36522\_Sample\_011046840, Unigene15213\_Sample\_011046840, Unigene23572\_Sample\_011046840, Unigene35864\_Sample\_011046840, Unigene36191\_Sample\_011046840, Unigene36589\_Sample\_011046840, Unigene42744\_Sample\_011046840, Unigene25557\_Sample\_011046840, Unigene39049\_Sample\_011046840, Unigene33136\_Sample\_011046840, Unigene8045\_Sample\_011046840, Unigene36492\_Sample\_011046840, Unigene9944\_Sample\_011046840, Unigene6129\_Sample\_011046840, Unigene33969\_Sample\_011046840, Unigene29982\_Sample\_011046840, Unigene30879\_Sample\_011046840, Unigene16314\_Sample\_011046840, Unigene39491\_Sample\_011046840, Unigene22159\_Sample\_011046840, Unigene30576\_Sample\_011046840, Unigene23397\_Sample\_011046840, Unigene26297\_Sample\_011046840, Unigene8181\_Sample\_011046840, Unigene37274\_Sample\_011046840, Unigene6641\_Sample\_011046840, Unigene17700\_Sample\_011046840, Unigene24914\_Sample\_011046840, Unigene40859\_Sample\_011046840, Unigene39265\_Sample\_011046840, Unigene35677\_Sample\_011046840, Unigene4464\_Sample\_011046840, Unigene9962\_Sample\_011046840, Unigene39237\_Sample\_011046840, Unigene41136\_Sample\_011046840, Unigene7402\_Sample\_011046840, Unigene23730\_Sample\_011046840, Unigene31057\_Sample\_011046840, Unigene43387\_Sample\_011046840, Unigene37570\_Sample\_011046840, Unigene31458\_Sample\_011046840, Unigene41268\_Sample\_011046840, Unigene36089\_Sample\_011046840, Unigene30456\_Sample\_011046840, Unigene41678\_Sample\_011046840, Unigene35699\_Sample\_011046840, Unigene27865\_Sample\_011046840, Unigene36246\_Sample\_011046840, Unigene36967\_Sample\_011046840, Unigene34997\_Sample\_011046840, Unigene41128\_Sample\_011046840, Unigene1619\_Sample\_011046840, Unigene10148\_Sample\_011046840, Unigene489\_Sample\_011046840, Unigene7785\_Sample\_011046840, Unigene37688\_Sample\_011046840, Unigene18985\_Sample\_011046840, Unigene40020\_Sample\_011046840, Unigene41142\_Sample\_011046840, Unigene3897\_Sample\_011046840, Unigene25605\_Sample\_011046840, Unigene5969\_Sample\_011046840, Unigene38025\_Sample\_011046840, Unigene19052\_Sample\_011046840, Unigene2383\_Sample\_011046840, Unigene27993\_Sample\_011046840, Unigene39020\_Sample\_011046840, Unigene7818\_Sample\_011046840, Unigene8465\_Sample\_011046840, Unigene24917\_Sample\_011046840, Unigene36737\_Sample\_011046840, Unigene32843\_Sample\_011046840, Unigene5257\_Sample\_011046840, Unigene8975\_Sample\_011046840, Unigene32360\_Sample\_011046840, Unigene10202\_Sample\_011046840, Unigene35359\_Sample\_011046840, Unigene42224\_Sample\_011046840, Unigene38351\_Sample\_011046840, Unigene16893\_Sample\_011046840, Unigene29994\_Sample\_011046840, Unigene28620\_Sample\_011046840, Unigene37826\_Sample\_011046840, Unigene16218\_Sample\_011046840, Unigene37581\_Sample\_011046840, Unigene36659\_Sample\_011046840, Unigene23761\_Sample\_011046840, Unigene41424\_Sample\_011046840, Unigene33024\_Sample\_011046840, Unigene36819\_Sample\_011046840, Unigene40437\_Sample\_011046840, Unigene29267\_Sample\_011046840, Unigene40661\_Sample\_011046840, Unigene14485\_Sample\_011046840, Unigene37664\_Sample\_011046840, Unigene19235\_Sample\_011046840, Unigene27831\_Sample\_011046840, Unigene16385\_Sample\_011046840, Unigene40727\_Sample\_011046840, Unigene20304\_Sample\_011046840, Unigene13223\_Sample\_011046840, Unigene22789\_Sample\_011046840, Unigene29294\_Sample\_011046840, Unigene3713\_Sample\_011046840, Unigene32431\_Sample\_011046840, Unigene33393\_Sample\_011046840, Unigene21033\_Sample\_011046840, Unigene42484\_Sample\_011046840, Unigene40993\_Sample\_011046840, Unigene39796\_Sample\_011046840, Unigene4692\_Sample\_011046840, Unigene7287\_Sample\_011046840, Unigene5245\_Sample\_011046840, Unigene33196\_Sample\_011046840, Unigene6912\_Sample\_011046840, Unigene27570\_Sample\_011046840, Unigene40222\_Sample\_011046840, Unigene7760\_Sample\_011046840, Unigene4955\_Sample\_011046840, Unigene36846\_Sample\_011046840, Unigene26550\_Sample\_011046840, Unigene26453\_Sample\_011046840, Unigene32803\_Sample\_011046840, Unigene25853\_Sample\_011046840, Unigene35874\_Sample\_011046840, Unigene38065\_Sample\_011046840, Unigene41941\_Sample\_011046840, Unigene43009\_Sample\_011046840, Unigene41616\_Sample\_011046840, Unigene40052\_Sample\_011046840, Unigene34927\_Sample\_011046840, Unigene40368\_Sample\_011046840, Unigene10913\_Sample\_011046840, Unigene28253\_Sample\_011046840, Unigene26424\_Sample\_011046840, Unigene22186\_Sample\_011046840, Unigene41177\_Sample\_011046840, Unigene14590\_Sample\_011046840, Unigene30365\_Sample\_011046840, Unigene12728\_Sample\_011046840, Unigene7589\_Sample\_011046840, Unigene31564\_Sample\_011046840, Unigene3029\_Sample\_011046840, Unigene32515\_Sample\_011046840, Unigene473\_Sample\_011046840, Unigene20351\_Sample\_011046840, Unigene12616\_Sample\_011046840, Unigene31408\_Sample\_011046840, Unigene40614\_Sample\_011046840, Unigene42951\_Sample\_011046840, Unigene17674\_Sample\_011046840, Unigene4307\_Sample\_011046840, Unigene38150\_Sample\_011046840, Unigene40026\_Sample\_011046840, Unigene33692\_Sample\_011046840, Unigene42407\_Sample\_011046840, Unigene40573\_Sample\_011046840, Unigene3098\_Sample\_011046840, Unigene15855\_Sample\_011046840, Unigene38116\_Sample\_011046840, Unigene443\_Sample\_011046840, Unigene39360\_Sample\_011046840, Unigene2342\_Sample\_011046840, Unigene3664\_Sample\_011046840, Unigene40697\_Sample\_011046840, Unigene39420\_Sample\_011046840, Unigene24457\_Sample\_011046840, Unigene700\_Sample\_011046840, Unigene38370\_Sample\_011046840, Unigene37533\_Sample\_011046840, Unigene30813\_Sample\_011046840, Unigene42620\_Sample\_011046840, Unigene22254\_Sample\_011046840, Unigene13304\_Sample\_011046840, Unigene28405\_Sample\_011046840, Unigene32263\_Sample\_011046840, Unigene42627\_Sample\_011046840, Unigene37020\_Sample\_011046840, Unigene42997\_Sample\_011046840, Unigene23433\_Sample\_011046840, Unigene17432\_Sample\_011046840, Unigene38844\_Sample\_011046840, Unigene37883\_Sample\_011046840, Unigene35646\_Sample\_011046840, Unigene8665\_Sample\_011046840, Unigene35835\_Sample\_011046840, Unigene42131\_Sample\_011046840, Unigene39107\_Sample\_011046840, Unigene1399\_Sample\_011046840, Unigene24223\_Sample\_011046840, Unigene7363\_Sample\_011046840, Unigene31743\_Sample\_011046840, Unigene6293\_Sample\_011046840, Unigene36580\_Sample\_011046840, Unigene25822\_Sample\_011046840, Unigene24719\_Sample\_011046840, Unigene52\_Sample\_011046840, Unigene30604\_Sample\_011046840, Unigene39413\_Sample\_011046840, Unigene41588\_Sample\_011046840, Unigene43176\_Sample\_011046840, Unigene26884\_Sample\_011046840, Unigene40410\_Sample\_011046840, Unigene31033\_Sample\_011046840, Unigene27425\_Sample\_011046840, Unigene22528\_Sample\_011046840, Unigene11853\_Sample\_011046840, Unigene7819\_Sample\_011046840, Unigene30784\_Sample\_011046840, Unigene34657\_Sample\_011046840, Unigene42286\_Sample\_011046840, Unigene15696\_Sample\_011046840, Unigene29131\_Sample\_011046840, Unigene33772\_Sample\_011046840, Unigene42227\_Sample\_011046840, Unigene42813\_Sample\_011046840, Unigene40918\_Sample\_011046840, Unigene26969\_Sample\_011046840, Unigene5227\_Sample\_011046840, Unigene37937\_Sample\_011046840, Unigene37229\_Sample\_011046840, Unigene19320\_Sample\_011046840, Unigene25223\_Sample\_011046840, Unigene32118\_Sample\_011046840, Unigene37721\_Sample\_011046840, Unigene30611\_Sample\_011046840, Unigene13770\_Sample\_011046840, Unigene40565\_Sample\_011046840, Unigene28467\_Sample\_011046840, Unigene24618\_Sample\_011046840, Unigene36400\_Sample\_011046840, Unigene43084\_Sample\_011046840, Unigene24149\_Sample\_011046840, Unigene1814\_Sample\_011046840, Unigene30176\_Sample\_011046840, Unigene41712\_Sample\_011046840, Unigene41078\_Sample\_011046840, Unigene23155\_Sample\_011046840, Unigene28822\_Sample\_011046840, Unigene22122\_Sample\_011046840, Unigene32717\_Sample\_011046840, Unigene19982\_Sample\_011046840, Unigene34464\_Sample\_011046840, Unigene1404\_Sample\_011046840, Unigene6426\_Sample\_011046840, Unigene13980\_Sample\_011046840, Unigene557\_Sample\_011046840, Unigene29367\_Sample\_011046840, Unigene23570\_Sample\_011046840, Unigene31658\_Sample\_011046840, Unigene39522\_Sample\_011046840, Unigene7891\_Sample\_011046840, Unigene31412\_Sample\_011046840, Unigene12130\_Sample\_011046840, Unigene4723\_Sample\_011046840, Unigene29352\_Sample\_011046840, Unigene30691\_Sample\_011046840, Unigene43414\_Sample\_011046840, Unigene7503\_Sample\_011046840, Unigene6926\_Sample\_011046840, Unigene43021\_Sample\_011046840, Unigene27887\_Sample\_011046840, Unigene41062\_Sample\_011046840, Unigene36349\_Sample\_011046840, Unigene38630\_Sample\_011046840, Unigene26953\_Sample\_011046840, Unigene23108\_Sample\_011046840, Unigene39865\_Sample\_011046840, Unigene40273\_Sample\_011046840, Unigene28061\_Sample\_011046840, Unigene34121\_Sample\_011046840, Unigene32375\_Sample\_011046840, Unigene40057\_Sample\_011046840, Unigene34281\_Sample\_011046840, Unigene39556\_Sample\_011046840, Unigene40411\_Sample\_011046840, Unigene43249\_Sample\_011046840, Unigene23390\_Sample\_011046840, Unigene34655\_Sample\_011046840, Unigene34703\_Sample\_011046840, Unigene32984\_Sample\_011046840, Unigene25956\_Sample\_011046840, Unigene5491\_Sample\_011046840, Unigene7291\_Sample\_011046840, Unigene31889\_Sample\_011046840, Unigene21072\_Sample\_011046840, Unigene35505\_Sample\_011046840, Unigene33626\_Sample\_011046840, Unigene3345\_Sample\_011046840, Unigene16778\_Sample\_011046840, Unigene38559\_Sample\_011046840, Unigene34658\_Sample\_011046840, Unigene34653\_Sample\_011046840, Unigene2822\_Sample\_011046840, Unigene18790\_Sample\_011046840, Unigene30185\_Sample\_011046840, Unigene42517\_Sample\_011046840, Unigene4332\_Sample\_011046840, Unigene20838\_Sample\_011046840, Unigene7868\_Sample\_011046840, Unigene35883\_Sample\_011046840, Unigene37041\_Sample\_011046840, Unigene42114\_Sample\_011046840, Unigene23630\_Sample\_011046840, Unigene41653\_Sample\_011046840, Unigene30946\_Sample\_011046840, Unigene36203\_Sample\_011046840, Unigene30963\_Sample\_011046840, Unigene26759\_Sample\_011046840, Unigene35002\_Sample\_011046840, Unigene42943\_Sample\_011046840, Unigene22644\_Sample\_011046840, Unigene27654\_Sample\_011046840, Unigene17719\_Sample\_011046840, Unigene10241\_Sample\_011046840, Unigene43251\_Sample\_011046840, Unigene42171\_Sample\_011046840, Unigene22998\_Sample\_011046840, Unigene23890\_Sample\_011046840, Unigene35348\_Sample\_011046840, Unigene41125\_Sample\_011046840, Unigene39117\_Sample\_011046840, Unigene36935\_Sample\_011046840, Unigene36917\_Sample\_011046840, Unigene13452\_Sample\_011046840, Unigene41204\_Sample\_011046840, Unigene36900\_Sample\_011046840, Unigene29654\_Sample\_011046840, Unigene37187\_Sample\_011046840, Unigene33682\_Sample\_011046840, Unigene14568\_Sample\_011046840, Unigene41887\_Sample\_011046840, Unigene25041\_Sample\_011046840, Unigene43555\_Sample\_011046840, Unigene22688\_Sample\_011046840, Unigene16247\_Sample\_011046840, Unigene32285\_Sample\_011046840, Unigene40391\_Sample\_011046840, Unigene28898\_Sample\_011046840, Unigene1089\_Sample\_011046840, Unigene39732\_Sample\_011046840, Unigene7134\_Sample\_011046840, Unigene32923\_Sample\_011046840, Unigene8142\_Sample\_011046840, Unigene20821\_Sample\_011046840, Unigene19481\_Sample\_011046840, Unigene36458\_Sample\_011046840, Unigene5846\_Sample\_011046840, Unigene36991\_Sample\_011046840, Unigene14274\_Sample\_011046840, Unigene28668\_Sample\_011046840, Unigene43275\_Sample\_011046840, Unigene28414\_Sample\_011046840, Unigene37126\_Sample\_011046840, Unigene36326\_Sample\_011046840, Unigene21804\_Sample\_011046840, Unigene37908\_Sample\_011046840, Unigene40449\_Sample\_011046840, Unigene29198\_Sample\_011046840, Unigene30757\_Sample\_011046840, Unigene32056\_Sample\_011046840, Unigene43276\_Sample\_011046840, Unigene39406\_Sample\_011046840, Unigene25951\_Sample\_011046840, Unigene36621\_Sample\_011046840, Unigene27601\_Sample\_011046840, Unigene14188\_Sample\_011046840, Unigene38001\_Sample\_011046840, Unigene19274\_Sample\_011046840, Unigene42387\_Sample\_011046840, Unigene7407\_Sample\_011046840, Unigene38261\_Sample\_011046840, Unigene40049\_Sample\_011046840, Unigene23126\_Sample\_011046840, Unigene30862\_Sample\_011046840, Unigene7533\_Sample\_011046840, Unigene42369\_Sample\_011046840, Unigene15803\_Sample\_011046840, Unigene4082\_Sample\_011046840, Unigene8152\_Sample\_011046840, Unigene38115\_Sample\_011046840, Unigene36462\_Sample\_011046840, Unigene33808\_Sample\_011046840, Unigene34932\_Sample\_011046840, Unigene42090\_Sample\_011046840, Unigene42430\_Sample\_011046840, Unigene19425\_Sample\_011046840, Unigene29121\_Sample\_011046840, Unigene26987\_Sample\_011046840, Unigene30393\_Sample\_011046840, Unigene27428\_Sample\_011046840, Unigene19859\_Sample\_011046840, Unigene28282\_Sample\_011046840, Unigene40806\_Sample\_011046840, Unigene33616\_Sample\_011046840, Unigene29480\_Sample\_011046840, Unigene42030\_Sample\_011046840, Unigene20517\_Sample\_011046840, Unigene20681\_Sample\_011046840, Unigene39011\_Sample\_011046840, Unigene28316\_Sample\_011046840, Unigene16978\_Sample\_011046840, Unigene6139\_Sample\_011046840, Unigene2234\_Sample\_011046840, Unigene35314\_Sample\_011046840, Unigene19370\_Sample\_011046840, Unigene39978\_Sample\_011046840, Unigene38021\_Sample\_011046840, Unigene34684\_Sample\_011046840, Unigene28200\_Sample\_011046840, Unigene35485\_Sample\_011046840, Unigene26967\_Sample\_011046840, Unigene34960\_Sample\_011046840, Unigene36437\_Sample\_011046840, Unigene6495\_Sample\_011046840, Unigene23988\_Sample\_011046840, Unigene38381\_Sample\_011046840, Unigene22660\_Sample\_011046840, Unigene30195\_Sample\_011046840, Unigene5190\_Sample\_011046840, Unigene39647\_Sample\_011046840, Unigene1246\_Sample\_011046840, Unigene39272\_Sample\_011046840, Unigene41702\_Sample\_011046840, Unigene30851\_Sample\_011046840, Unigene21295\_Sample\_011046840, Unigene35151\_Sample\_011046840, Unigene12570\_Sample\_011046840, Unigene26575\_Sample\_011046840, Unigene24067\_Sample\_011046840, Unigene36910\_Sample\_011046840, Unigene42047\_Sample\_011046840, Unigene18403\_Sample\_011046840, Unigene15252\_Sample\_011046840, Unigene24706\_Sample\_011046840, Unigene40705\_Sample\_011046840, Unigene15165\_Sample\_011046840, Unigene38478\_Sample\_011046840, Unigene41361\_Sample\_011046840, Unigene28163\_Sample\_011046840, Unigene18649\_Sample\_011046840, Unigene38571\_Sample\_011046840, Unigene43591\_Sample\_011046840, Unigene24532\_Sample\_011046840, Unigene15446\_Sample\_011046840, Unigene6254\_Sample\_011046840, Unigene35118\_Sample\_011046840, Unigene36251\_Sample\_011046840, Unigene42209\_Sample\_011046840, Unigene38245\_Sample\_011046840, Unigene24921\_Sample\_011046840, Unigene2615\_Sample\_011046840, Unigene39540\_Sample\_011046840, Unigene35939\_Sample\_011046840, Unigene38775\_Sample\_011046840, Unigene4842\_Sample\_011046840, Unigene15455\_Sample\_011046840, Unigene20828\_Sample\_011046840, Unigene37077\_Sample\_011046840, Unigene31322\_Sample\_011046840, Unigene24172\_Sample\_011046840, Unigene25348\_Sample\_011046840, Unigene37535\_Sample\_011046840, Unigene13078\_Sample\_011046840, Unigene3144\_Sample\_011046840, Unigene23304\_Sample\_011046840, Unigene43464\_Sample\_011046840, Unigene42786\_Sample\_011046840, Unigene8165\_Sample\_011046840, Unigene37362\_Sample\_011046840, Unigene35015\_Sample\_011046840, Unigene13399\_Sample\_011046840, Unigene32907\_Sample\_011046840, Unigene38121\_Sample\_011046840, Unigene13285\_Sample\_011046840, Unigene29658\_Sample\_011046840, Unigene32316\_Sample\_011046840, Unigene32939\_Sample\_011046840, Unigene22061\_Sample\_011046840, Unigene23873\_Sample\_011046840, Unigene35564\_Sample\_011046840, Unigene35642\_Sample\_011046840, Unigene5081\_Sample\_011046840, Unigene37015\_Sample\_011046840, Unigene4025\_Sample\_011046840, Unigene12150\_Sample\_011046840, Unigene10106\_Sample\_011046840, Unigene38091\_Sample\_011046840, Unigene31822\_Sample\_011046840, Unigene41582\_Sample\_011046840, Unigene23401\_Sample\_011046840, Unigene43223\_Sample\_011046840, Unigene42903\_Sample\_011046840, Unigene23636\_Sample\_011046840, Unigene29411\_Sample\_011046840, Unigene4076\_Sample\_011046840, Unigene40284\_Sample\_011046840, Unigene25873\_Sample\_011046840, Unigene12776\_Sample\_011046840, Unigene29626\_Sample\_011046840, Unigene17079\_Sample\_011046840, Unigene2127\_Sample\_011046840, Unigene33260\_Sample\_011046840, Unigene7943\_Sample\_011046840, Unigene32288\_Sample\_011046840, Unigene7806\_Sample\_011046840, Unigene40465\_Sample\_011046840, Unigene1224\_Sample\_011046840, Unigene26940\_Sample\_011046840, Unigene42459\_Sample\_011046840, Unigene35284\_Sample\_011046840, Unigene30608\_Sample\_011046840, Unigene40223\_Sample\_011046840, Unigene31862\_Sample\_011046840, Unigene34227\_Sample\_011046840, Unigene3279\_Sample\_011046840, Unigene39179\_Sample\_011046840, Unigene3934\_Sample\_011046840, Unigene40087\_Sample\_011046840, Unigene42127\_Sample\_011046840, Unigene41531\_Sample\_011046840, Unigene31248\_Sample\_011046840, Unigene36075\_Sample\_011046840, Unigene18248\_Sample\_011046840, Unigene40210\_Sample\_011046840, Unigene35834\_Sample\_011046840, Unigene39894\_Sample\_011046840, Unigene12033\_Sample\_011046840, Unigene23557\_Sample\_011046840, Unigene35302\_Sample\_011046840, Unigene27260\_Sample\_011046840, Unigene25330\_Sample\_011046840, Unigene37432\_Sample\_011046840, Unigene41034\_Sample\_011046840, Unigene6551\_Sample\_011046840, Unigene38376\_Sample\_011046840, Unigene41339\_Sample\_011046840, Unigene841\_Sample\_011046840, Unigene43346\_Sample\_011046840, Unigene41418\_Sample\_011046840, Unigene36772\_Sample\_011046840, Unigene11410\_Sample\_011046840, Unigene25018\_Sample\_011046840, Unigene42861\_Sample\_011046840, Unigene35797\_Sample\_011046840, Unigene35977\_Sample\_011046840, Unigene18463\_Sample\_011046840, Unigene19271\_Sample\_011046840, Unigene17153\_Sample\_011046840, Unigene34652\_Sample\_011046840, Unigene12534\_Sample\_011046840, Unigene25826\_Sample\_011046840, Unigene29316\_Sample\_011046840, Unigene35743\_Sample\_011046840, Unigene12229\_Sample\_011046840, Unigene34059\_Sample\_011046840, Unigene33700\_Sample\_011046840, Unigene32253\_Sample\_011046840, Unigene12171\_Sample\_011046840, Unigene37637\_Sample\_011046840, Unigene22059\_Sample\_011046840, Unigene5098\_Sample\_011046840, Unigene7572\_Sample\_011046840, Unigene36485\_Sample\_011046840, Unigene10383\_Sample\_011046840, Unigene7727\_Sample\_011046840, Unigene30059\_Sample\_011046840, Unigene7391\_Sample\_011046840, Unigene41083\_Sample\_011046840, Unigene29913\_Sample\_011046840, Unigene39200\_Sample\_011046840, Unigene40849\_Sample\_011046840, Unigene37666\_Sample\_011046840, Unigene25292\_Sample\_011046840, Unigene25488\_Sample\_011046840, Unigene30695\_Sample\_011046840, Unigene39829\_Sample\_011046840, Unigene41935\_Sample\_011046840, Unigene32106\_Sample\_011046840, Unigene14473\_Sample\_011046840, Unigene34896\_Sample\_011046840, Unigene23121\_Sample\_011046840, Unigene41164\_Sample\_011046840, Unigene36257\_Sample\_011046840, Unigene38770\_Sample\_011046840, Unigene32268\_Sample\_011046840, Unigene39804\_Sample\_011046840, Unigene39391\_Sample\_011046840, Unigene24375\_Sample\_011046840, Unigene13729\_Sample\_011046840, Unigene41850\_Sample\_011046840, Unigene6860\_Sample\_011046840, Unigene41225\_Sample\_011046840, Unigene40017\_Sample\_011046840, Unigene21487\_Sample\_011046840, Unigene25938\_Sample\_011046840, Unigene33952\_Sample\_011046840, Unigene33427\_Sample\_011046840, Unigene39631\_Sample\_011046840, Unigene25767\_Sample\_011046840, Unigene42768\_Sample\_011046840, Unigene32790\_Sample\_011046840, Unigene41855\_Sample\_011046840, Unigene41007\_Sample\_011046840, Unigene10729\_Sample\_011046840, Unigene24420\_Sample\_011046840, Unigene10318\_Sample\_011046840, Unigene38034\_Sample\_011046840, Unigene41324\_Sample\_011046840, Unigene37439\_Sample\_011046840, Unigene29161\_Sample\_011046840, Unigene39464\_Sample\_011046840, Unigene25595\_Sample\_011046840, Unigene4724\_Sample\_011046840, Unigene43260\_Sample\_011046840, Unigene3510\_Sample\_011046840, Unigene6500\_Sample\_011046840, Unigene14750\_Sample\_011046840, Unigene40503\_Sample\_011046840, Unigene42626\_Sample\_011046840, Unigene21366\_Sample\_011046840, Unigene5335\_Sample\_011046840, Unigene24612\_Sample\_011046840, Unigene8162\_Sample\_011046840, Unigene41966\_Sample\_011046840, Unigene4686\_Sample\_011046840, Unigene41261\_Sample\_011046840, Unigene35610\_Sample\_011046840, Unigene36726\_Sample\_011046840, Unigene4101\_Sample\_011046840, Unigene29660\_Sample\_011046840, Unigene41784\_Sample\_011046840, Unigene27261\_Sample\_011046840, Unigene41113\_Sample\_011046840, Unigene40088\_Sample\_011046840, Unigene33984\_Sample\_011046840, Unigene32791\_Sample\_011046840, Unigene37631\_Sample\_011046840, Unigene11943\_Sample\_011046840, Unigene39527\_Sample\_011046840, Unigene40562\_Sample\_011046840, Unigene33954\_Sample\_011046840, Unigene39720\_Sample\_011046840, Unigene29794\_Sample\_011046840, Unigene38919\_Sample\_011046840, Unigene15316\_Sample\_011046840, Unigene39458\_Sample\_011046840, Unigene26807\_Sample\_011046840, Unigene32518\_Sample\_011046840, Unigene38914\_Sample\_011046840, Unigene24898\_Sample\_011046840, Unigene4898\_Sample\_011046840, Unigene19593\_Sample\_011046840, Unigene38495\_Sample\_011046840, Unigene42729\_Sample\_011046840, Unigene42963\_Sample\_011046840, Unigene41810\_Sample\_011046840, Unigene30989\_Sample\_011046840, Unigene31513\_Sample\_011046840, Unigene24411\_Sample\_011046840, Unigene13278\_Sample\_011046840, Unigene30236\_Sample\_011046840, Unigene41870\_Sample\_011046840, Unigene1077\_Sample\_011046840, Unigene14093\_Sample\_011046840, Unigene27305\_Sample\_011046840, Unigene16797\_Sample\_011046840, Unigene30162\_Sample\_011046840, Unigene17730\_Sample\_011046840, Unigene37338\_Sample\_011046840, Unigene28124\_Sample\_011046840, Unigene2498\_Sample\_011046840, Unigene41882\_Sample\_011046840, Unigene37773\_Sample\_011046840, Unigene40686\_Sample\_011046840, Unigene29570\_Sample\_011046840, Unigene26719\_Sample\_011046840, Unigene42930\_Sample\_011046840, Unigene42643\_Sample\_011046840, Unigene43155\_Sample\_011046840, Unigene41637\_Sample\_011046840, Unigene39931\_Sample\_011046840, Unigene42544\_Sample\_011046840, Unigene4520\_Sample\_011046840, Unigene1740\_Sample\_011046840, Unigene28339\_Sample\_011046840, Unigene42230\_Sample\_011046840, Unigene42798\_Sample\_011046840, Unigene40899\_Sample\_011046840, Unigene35692\_Sample\_011046840, Unigene39235\_Sample\_011046840, Unigene27745\_Sample\_011046840, Unigene10754\_Sample\_011046840, Unigene5720\_Sample\_011046840, Unigene9889\_Sample\_011046840, Unigene38857\_Sample\_011046840, Unigene20264\_Sample\_011046840, Unigene42293\_Sample\_011046840, Unigene42024\_Sample\_011046840, Unigene40316\_Sample\_011046840, Unigene23234\_Sample\_011046840, Unigene20485\_Sample\_011046840, Unigene29743\_Sample\_011046840, Unigene38612\_Sample\_011046840, Unigene28315\_Sample\_011046840, Unigene2740\_Sample\_011046840, Unigene14963\_Sample\_011046840, Unigene34975\_Sample\_011046840, Unigene24318\_Sample\_011046840, Unigene39777\_Sample\_011046840, Unigene31453\_Sample\_011046840, Unigene29661\_Sample\_011046840, Unigene35086\_Sample\_011046840, Unigene5006\_Sample\_011046840, Unigene31648\_Sample\_011046840, Unigene31407\_Sample\_011046840, Unigene42255\_Sample\_011046840, Unigene22119\_Sample\_011046840, Unigene43273\_Sample\_011046840, Unigene42020\_Sample\_011046840, Unigene29398\_Sample\_011046840, Unigene14412\_Sample\_011046840, Unigene8353\_Sample\_011046840, Unigene22727\_Sample\_011046840, Unigene30631\_Sample\_011046840, Unigene7348\_Sample\_011046840, Unigene35051\_Sample\_011046840, Unigene6849\_Sample\_011046840, Unigene8569\_Sample\_011046840, Unigene34380\_Sample\_011046840, Unigene20616\_Sample\_011046840, Unigene41119\_Sample\_011046840, Unigene4922\_Sample\_011046840, Unigene9782\_Sample\_011046840, Unigene2039\_Sample\_011046840, Unigene29712\_Sample\_011046840, Unigene6017\_Sample\_011046840, Unigene33956\_Sample\_011046840, Unigene40428\_Sample\_011046840, Unigene4300\_Sample\_011046840, Unigene28019\_Sample\_011046840, Unigene14037\_Sample\_011046840, Unigene11150\_Sample\_011046840, Unigene21352\_Sample\_011046840, Unigene13251\_Sample\_011046840, Unigene32546\_Sample\_011046840, Unigene39745\_Sample\_011046840, Unigene15931\_Sample\_011046840, Unigene36603\_Sample\_011046840, Unigene4572\_Sample\_011046840, Unigene31444\_Sample\_011046840, Unigene30936\_Sample\_011046840, Unigene24314\_Sample\_011046840, Unigene43217\_Sample\_011046840, Unigene42317\_Sample\_011046840, Unigene2237\_Sample\_011046840, Unigene11994\_Sample\_011046840, Unigene41108\_Sample\_011046840, Unigene22809\_Sample\_011046840, Unigene25745\_Sample\_011046840, Unigene22078\_Sample\_011046840, Unigene42628\_Sample\_011046840, Unigene26460\_Sample\_011046840, Unigene38313\_Sample\_011046840, Unigene39818\_Sample\_011046840, Unigene13083\_Sample\_011046840, Unigene34838\_Sample\_011046840, Unigene42039\_Sample\_011046840, Unigene35414\_Sample\_011046840, Unigene41216\_Sample\_011046840, Unigene5003\_Sample\_011046840, Unigene42549\_Sample\_011046840, Unigene35820\_Sample\_011046840, Unigene42434\_Sample\_011046840, Unigene22537\_Sample\_011046840, Unigene10050\_Sample\_011046840, Unigene13921\_Sample\_011046840, Unigene9447\_Sample\_011046840, Unigene20272\_Sample\_011046840, Unigene27020\_Sample\_011046840, Unigene38482\_Sample\_011046840, Unigene38449\_Sample\_011046840, Unigene37787\_Sample\_011046840, Unigene38540\_Sample\_011046840, Unigene40676\_Sample\_011046840, Unigene26563\_Sample\_011046840, Unigene3134\_Sample\_011046840, Unigene11875\_Sample\_011046840, Unigene16690\_Sample\_011046840, Unigene23601\_Sample\_011046840, Unigene33901\_Sample\_011046840, Unigene37789\_Sample\_011046840, Unigene41638\_Sample\_011046840, Unigene33395\_Sample\_011046840, Unigene39987\_Sample\_011046840, Unigene42168\_Sample\_011046840, Unigene29670\_Sample\_011046840, Unigene27635\_Sample\_011046840, Unigene14977\_Sample\_011046840, Unigene5557\_Sample\_011046840, Unigene31260\_Sample\_011046840, Unigene28451\_Sample\_011046840, Unigene29095\_Sample\_011046840, Unigene30975\_Sample\_011046840, Unigene15120\_Sample\_011046840, Unigene17564\_Sample\_011046840, Unigene39606\_Sample\_011046840, Unigene43449\_Sample\_011046840, Unigene37815\_Sample\_011046840, Unigene26646\_Sample\_011046840, Unigene41252\_Sample\_011046840, Unigene32397\_Sample\_011046840, Unigene26435\_Sample\_011046840, Unigene10332\_Sample\_011046840, Unigene5425\_Sample\_011046840, Unigene4108\_Sample\_011046840, Unigene40703\_Sample\_011046840, Unigene34817\_Sample\_011046840, Unigene26640\_Sample\_011046840, Unigene36817\_Sample\_011046840, Unigene4595\_Sample\_011046840, Unigene40609\_Sample\_011046840, Unigene28024\_Sample\_011046840, Unigene1830\_Sample\_011046840, Unigene37014\_Sample\_011046840, Unigene20572\_Sample\_011046840, Unigene38677\_Sample\_011046840, Unigene38254\_Sample\_011046840, Unigene40019\_Sample\_011046840, Unigene40031\_Sample\_011046840, Unigene7463\_Sample\_011046840, Unigene41720\_Sample\_011046840, Unigene14995\_Sample\_011046840, Unigene1355\_Sample\_011046840, Unigene41102\_Sample\_011046840, Unigene32521\_Sample\_011046840, Unigene33524\_Sample\_011046840, Unigene9789\_Sample\_011046840, Unigene38827\_Sample\_011046840, Unigene25963\_Sample\_011046840, Unigene33567\_Sample\_011046840, Unigene41559\_Sample\_011046840, Unigene6929\_Sample\_011046840, Unigene36638\_Sample\_011046840, Unigene496\_Sample\_011046840, Unigene2002\_Sample\_011046840, Unigene32884\_Sample\_011046840, Unigene30166\_Sample\_011046840, Unigene38801\_Sample\_011046840, Unigene24559\_Sample\_011046840, Unigene37919\_Sample\_011046840, Unigene34752\_Sample\_011046840, Unigene31306\_Sample\_011046840, Unigene40496\_Sample\_011046840, Unigene41694\_Sample\_011046840, Unigene11931\_Sample\_011046840, Unigene36123\_Sample\_011046840, Unigene5385\_Sample\_011046840, Unigene40745\_Sample\_011046840, Unigene7125\_Sample\_011046840, Unigene28038\_Sample\_011046840, Unigene37398\_Sample\_011046840, Unigene13786\_Sample\_011046840, Unigene21551\_Sample\_011046840, Unigene5962\_Sample\_011046840, Unigene36779\_Sample\_011046840, Unigene42979\_Sample\_011046840, Unigene28616\_Sample\_011046840, Unigene26707\_Sample\_011046840, Unigene6626\_Sample\_011046840, Unigene5200\_Sample\_011046840, Unigene6861\_Sample\_011046840, Unigene25450\_Sample\_011046840, Unigene6991\_Sample\_011046840, Unigene38328\_Sample\_011046840, Unigene23645\_Sample\_011046840, Unigene39232\_Sample\_011046840, Unigene32668\_Sample\_011046840, Unigene13656\_Sample\_011046840, Unigene43358\_Sample\_011046840, Unigene23032\_Sample\_011046840, Unigene39480\_Sample\_011046840, Unigene40491\_Sample\_011046840, Unigene25194\_Sample\_011046840, Unigene33508\_Sample\_011046840, Unigene37796\_Sample\_011046840, Unigene10108\_Sample\_011046840, Unigene19985\_Sample\_011046840, Unigene39905\_Sample\_011046840, Unigene4100\_Sample\_011046840, Unigene31024\_Sample\_011046840, Unigene43502\_Sample\_011046840, Unigene27982\_Sample\_011046840, Unigene33048\_Sample\_011046840, Unigene42038\_Sample\_011046840, Unigene14245\_Sample\_011046840, Unigene43519\_Sample\_011046840, Unigene36293\_Sample\_011046840, Unigene41873\_Sample\_011046840, Unigene32377\_Sample\_011046840, Unigene43410\_Sample\_011046840, Unigene39202\_Sample\_011046840, Unigene6873\_Sample\_011046840, Unigene35609\_Sample\_011046840, Unigene38940\_Sample\_011046840, Unigene41283\_Sample\_011046840, Unigene8658\_Sample\_011046840, Unigene26198\_Sample\_011046840, Unigene34730\_Sample\_011046840, Unigene23012\_Sample\_011046840, Unigene37892\_Sample\_011046840, Unigene36547\_Sample\_011046840, Unigene34200\_Sample\_011046840, Unigene39956\_Sample\_011046840, Unigene20538\_Sample\_011046840, Unigene35877\_Sample\_011046840, Unigene18965\_Sample\_011046840, Unigene33873\_Sample\_011046840, Unigene23494\_Sample\_011046840, Unigene1379\_Sample\_011046840, Unigene43023\_Sample\_011046840, Unigene41968\_Sample\_011046840, Unigene6784\_Sample\_011046840, Unigene25891\_Sample\_011046840, Unigene26491\_Sample\_011046840, Unigene29963\_Sample\_011046840, Unigene37790\_Sample\_011046840, Unigene18925\_Sample\_011046840, Unigene41072\_Sample\_011046840, Unigene6705\_Sample\_011046840, Unigene33046\_Sample\_011046840, Unigene36229\_Sample\_011046840, Unigene42700\_Sample\_011046840, Unigene706\_Sample\_011046840, Unigene27243\_Sample\_011046840, Unigene7553\_Sample\_011046840, Unigene35495\_Sample\_011046840, Unigene21063\_Sample\_011046840, Unigene9937\_Sample\_011046840, Unigene13581\_Sample\_011046840, Unigene43567\_Sample\_011046840, Unigene26863\_Sample\_011046840, Unigene36780\_Sample\_011046840, Unigene42404\_Sample\_011046840, Unigene36977\_Sample\_011046840, Unigene16992\_Sample\_011046840, Unigene30289\_Sample\_011046840, Unigene36232\_Sample\_011046840, Unigene19207\_Sample\_011046840, Unigene24096\_Sample\_011046840, Unigene4855\_Sample\_011046840, Unigene43373\_Sample\_011046840, Unigene23833\_Sample\_011046840, Unigene9558\_Sample\_011046840, Unigene40875\_Sample\_011046840, Unigene32576\_Sample\_011046840, Unigene4849\_Sample\_011046840, Unigene34163\_Sample\_011046840, Unigene33344\_Sample\_011046840, Unigene39032\_Sample\_011046840, Unigene40781\_Sample\_011046840, Unigene2216\_Sample\_011046840, Unigene41670\_Sample\_011046840, Unigene8605\_Sample\_011046840, Unigene33148\_Sample\_011046840, Unigene752\_Sample\_011046840, Unigene42875\_Sample\_011046840, Unigene35307\_Sample\_011046840, Unigene10443\_Sample\_011046840, Unigene11035\_Sample\_011046840, Unigene14602\_Sample\_011046840, Unigene11279\_Sample\_011046840, Unigene24744\_Sample\_011046840, Unigene19882\_Sample\_011046840, Unigene20463\_Sample\_011046840, Unigene42337\_Sample\_011046840, Unigene17478\_Sample\_011046840, Unigene41722\_Sample\_011046840, Unigene41299\_Sample\_011046840, Unigene42966\_Sample\_011046840, Unigene43521\_Sample\_011046840, Unigene1568\_Sample\_011046840, Unigene34785\_Sample\_011046840, Unigene38468\_Sample\_011046840, Unigene30715\_Sample\_011046840, Unigene42761\_Sample\_011046840, Unigene599\_Sample\_011046840, Unigene18264\_Sample\_011046840, Unigene37236\_Sample\_011046840, Unigene42685\_Sample\_011046840, Unigene38203\_Sample\_011046840, Unigene13335\_Sample\_011046840, Unigene27924\_Sample\_011046840, Unigene10706\_Sample\_011046840, Unigene41121\_Sample\_011046840, Unigene40533\_Sample\_011046840, Unigene24635\_Sample\_011046840, Unigene18128\_Sample\_011046840, Unigene22945\_Sample\_011046840, Unigene19049\_Sample\_011046840, Unigene37255\_Sample\_011046840, Unigene18691\_Sample\_011046840, Unigene24400\_Sample\_011046840, Unigene19287\_Sample\_011046840, Unigene24518\_Sample\_011046840, Unigene27725\_Sample\_011046840, Unigene7588\_Sample\_011046840, Unigene14847\_Sample\_011046840, Unigene42103\_Sample\_011046840, Unigene41785\_Sample\_011046840, Unigene35673\_Sample\_011046840, Unigene22486\_Sample\_011046840, Unigene36628\_Sample\_011046840, Unigene39912\_Sample\_011046840, Unigene4368\_Sample\_011046840, Unigene36382\_Sample\_011046840, Unigene22482\_Sample\_011046840, Unigene6634\_Sample\_011046840, Unigene43511\_Sample\_011046840, Unigene28177\_Sample\_011046840, Unigene32361\_Sample\_011046840, Unigene33015\_Sample\_011046840, Unigene32026\_Sample\_011046840, Unigene9678\_Sample\_011046840, Unigene15903\_Sample\_011046840, Unigene8931\_Sample\_011046840, Unigene28117\_Sample\_011046840, Unigene43514\_Sample\_011046840, Unigene13197\_Sample\_011046840, Unigene41937\_Sample\_011046840, Unigene33737\_Sample\_011046840, Unigene15140\_Sample\_011046840, Unigene38469\_Sample\_011046840, Unigene32866\_Sample\_011046840, Unigene14307\_Sample\_011046840, Unigene20643\_Sample\_011046840, Unigene39579\_Sample\_011046840, Unigene5097\_Sample\_011046840, Unigene32770\_Sample\_011046840, Unigene37589\_Sample\_011046840, Unigene11155\_Sample\_011046840, Unigene30337\_Sample\_011046840, Unigene39571\_Sample\_011046840, Unigene2160\_Sample\_011046840, Unigene39057\_Sample\_011046840, Unigene40083\_Sample\_011046840, Unigene23676\_Sample\_011046840, Unigene25874\_Sample\_011046840, Unigene41236\_Sample\_011046840, Unigene36770\_Sample\_011046840, Unigene35486\_Sample\_011046840, Unigene15713\_Sample\_011046840, Unigene14623\_Sample\_011046840, Unigene37474\_Sample\_011046840, Unigene34254\_Sample\_011046840, Unigene43374\_Sample\_011046840, Unigene17541\_Sample\_011046840, Unigene17049\_Sample\_011046840, Unigene7014\_Sample\_011046840, Unigene41042\_Sample\_011046840, Unigene4577\_Sample\_011046840, Unigene27233\_Sample\_011046840, Unigene5650\_Sample\_011046840, Unigene6209\_Sample\_011046840, Unigene38242\_Sample\_011046840, Unigene38803\_Sample\_011046840, Unigene25298\_Sample\_011046840, Unigene41727\_Sample\_011046840, Unigene41469\_Sample\_011046840, Unigene40813\_Sample\_011046840, Unigene7752\_Sample\_011046840, Unigene21046\_Sample\_011046840, Unigene15743\_Sample\_011046840, Unigene34777\_Sample\_011046840, Unigene19991\_Sample\_011046840, Unigene37995\_Sample\_011046840, Unigene38701\_Sample\_011046840, Unigene42622\_Sample\_011046840, Unigene38323\_Sample\_011046840, Unigene8388\_Sample\_011046840, Unigene8213\_Sample\_011046840, Unigene16359\_Sample\_011046840, Unigene40247\_Sample\_011046840, Unigene6608\_Sample\_011046840, Unigene33090\_Sample\_011046840, Unigene25203\_Sample\_011046840, Unigene5772\_Sample\_011046840, Unigene35507\_Sample\_011046840, Unigene43051\_Sample\_011046840, Unigene31151\_Sample\_011046840, Unigene4412\_Sample\_011046840, Unigene37414\_Sample\_011046840, Unigene40399\_Sample\_011046840, Unigene20960\_Sample\_011046840, Unigene5037\_Sample\_011046840, Unigene33982\_Sample\_011046840, Unigene20699\_Sample\_011046840, Unigene40807\_Sample\_011046840, Unigene1879\_Sample\_011046840, Unigene37529\_Sample\_011046840, Unigene42653\_Sample\_011046840, Unigene1535\_Sample\_011046840, Unigene37694\_Sample\_011046840, Unigene6457\_Sample\_011046840, Unigene641\_Sample\_011046840, Unigene11516\_Sample\_011046840, Unigene39750\_Sample\_011046840, Unigene34208\_Sample\_011046840, Unigene22265\_Sample\_011046840, Unigene38279\_Sample\_011046840, Unigene28752\_Sample\_011046840, Unigene25915\_Sample\_011046840, Unigene37799\_Sample\_011046840, Unigene26572\_Sample\_011046840, Unigene42972\_Sample\_011046840, Unigene43364\_Sample\_011046840, Unigene2284\_Sample\_011046840, Unigene33129\_Sample\_011046840, Unigene19092\_Sample\_011046840, Unigene36364\_Sample\_011046840, Unigene28943\_Sample\_011046840, Unigene30501\_Sample\_011046840, Unigene17585\_Sample\_011046840, Unigene4920\_Sample\_011046840, Unigene36504\_Sample\_011046840, Unigene23249\_Sample\_011046840, Unigene7708\_Sample\_011046840, Unigene40839\_Sample\_011046840, Unigene19948\_Sample\_011046840, Unigene39661\_Sample\_011046840, Unigene41436\_Sample\_011046840, Unigene13336\_Sample\_011046840, Unigene30481\_Sample\_011046840, Unigene39751\_Sample\_011046840, Unigene8000\_Sample\_011046840, Unigene37684\_Sample\_011046840, Unigene38450\_Sample\_011046840, Unigene42250\_Sample\_011046840, Unigene33105\_Sample\_011046840, Unigene2066\_Sample\_011046840, Unigene39916\_Sample\_011046840, Unigene41869\_Sample\_011046840, Unigene39979\_Sample\_011046840, Unigene16302\_Sample\_011046840, Unigene2783\_Sample\_011046840, Unigene33011\_Sample\_011046840, Unigene30994\_Sample\_011046840, Unigene33465\_Sample\_011046840, Unigene14238\_Sample\_011046840, Unigene31397\_Sample\_011046840, Unigene36103\_Sample\_011046840, Unigene41546\_Sample\_011046840, Unigene33527\_Sample\_011046840, Unigene7054\_Sample\_011046840, Unigene15710\_Sample\_011046840, Unigene28952\_Sample\_011046840, Unigene34615\_Sample\_011046840, Unigene32859\_Sample\_011046840, Unigene34686\_Sample\_011046840, Unigene34028\_Sample\_011046840, Unigene39604\_Sample\_011046840, Unigene43264\_Sample\_011046840, Unigene39889\_Sample\_011046840, Unigene38023\_Sample\_011046840, Unigene26641\_Sample\_011046840, Unigene34267\_Sample\_011046840, Unigene7902\_Sample\_011046840, Unigene42776\_Sample\_011046840, Unigene37568\_Sample\_011046840, Unigene26763\_Sample\_011046840, Unigene41910\_Sample\_011046840, Unigene19401\_Sample\_011046840, Unigene39840\_Sample\_011046840, Unigene40636\_Sample\_011046840, Unigene43154\_Sample\_011046840, Unigene7132\_Sample\_011046840, Unigene30892\_Sample\_011046840, Unigene313\_Sample\_011046840, Unigene43100\_Sample\_011046840, Unigene2493\_Sample\_011046840, Unigene2917\_Sample\_011046840, Unigene908\_Sample\_011046840, Unigene32380\_Sample\_011046840, Unigene39302\_Sample\_011046840, Unigene23411\_Sample\_011046840, Unigene32488\_Sample\_011046840, Unigene41580\_Sample\_011046840, Unigene41153\_Sample\_011046840, Unigene35962\_Sample\_011046840, Unigene32455\_Sample\_011046840, Unigene33356\_Sample\_011046840, Unigene32335\_Sample\_011046840, Unigene42949\_Sample\_011046840, Unigene20979\_Sample\_011046840, Unigene40972\_Sample\_011046840, Unigene6748\_Sample\_011046840, Unigene42607\_Sample\_011046840, Unigene5545\_Sample\_011046840, Unigene40625\_Sample\_011046840, Unigene40773\_Sample\_011046840, Unigene20982\_Sample\_011046840, Unigene32089\_Sample\_011046840, Unigene31072\_Sample\_011046840, Unigene30370\_Sample\_011046840, Unigene41318\_Sample\_011046840, Unigene39708\_Sample\_011046840, Unigene17494\_Sample\_011046840, Unigene34499\_Sample\_011046840, Unigene23767\_Sample\_011046840, Unigene36931\_Sample\_011046840, Unigene35607\_Sample\_011046840, Unigene41473\_Sample\_011046840, Unigene42635\_Sample\_011046840, Unigene5219\_Sample\_011046840, Unigene7352\_Sample\_011046840, Unigene18500\_Sample\_011046840, Unigene31129\_Sample\_011046840, Unigene34236\_Sample\_011046840, Unigene42762\_Sample\_011046840, Unigene34068\_Sample\_011046840, Unigene39858\_Sample\_011046840, Unigene177\_Sample\_011046840, Unigene41833\_Sample\_011046840, Unigene11899\_Sample\_011046840, Unigene35615\_Sample\_011046840, Unigene38504\_Sample\_011046840, Unigene40734\_Sample\_011046840, Unigene15902\_Sample\_011046840, Unigene36848\_Sample\_011046840, Unigene36463\_Sample\_011046840, Unigene38201\_Sample\_011046840, Unigene37095\_Sample\_011046840, Unigene30026\_Sample\_011046840, Unigene18797\_Sample\_011046840, Unigene36359\_Sample\_011046840, Unigene15951\_Sample\_011046840, Unigene1709\_Sample\_011046840, Unigene5621\_Sample\_011046840, Unigene15791\_Sample\_011046840, Unigene39666\_Sample\_011046840, Unigene35437\_Sample\_011046840, Unigene37505\_Sample\_011046840, Unigene24478\_Sample\_011046840, Unigene30203\_Sample\_011046840, Unigene43435\_Sample\_011046840, Unigene39812\_Sample\_011046840, Unigene40047\_Sample\_011046840, Unigene39711\_Sample\_011046840, Unigene30726\_Sample\_011046840, Unigene17955\_Sample\_011046840, Unigene11041\_Sample\_011046840, Unigene33707\_Sample\_011046840, Unigene41104\_Sample\_011046840, Unigene20573\_Sample\_011046840, Unigene18097\_Sample\_011046840, Unigene34842\_Sample\_011046840, Unigene36005\_Sample\_011046840, Unigene17299\_Sample\_011046840, Unigene37066\_Sample\_011046840, Unigene607\_Sample\_011046840, Unigene41965\_Sample\_011046840, Unigene40796\_Sample\_011046840, Unigene4663\_Sample\_011046840, Unigene26766\_Sample\_011046840, Unigene24013\_Sample\_011046840, Unigene34514\_Sample\_011046840, Unigene26168\_Sample\_011046840, Unigene37186\_Sample\_011046840, Unigene43122\_Sample\_011046840, Unigene41124\_Sample\_011046840, Unigene30959\_Sample\_011046840, Unigene33208\_Sample\_011046840, Unigene7795\_Sample\_011046840, Unigene41096\_Sample\_011046840, Unigene37358\_Sample\_011046840, Unigene29560\_Sample\_011046840, Unigene25972\_Sample\_011046840, Unigene35878\_Sample\_011046840, Unigene18599\_Sample\_011046840, Unigene43230\_Sample\_011046840, Unigene5654\_Sample\_011046840, Unigene8445\_Sample\_011046840, Unigene25570\_Sample\_011046840, Unigene35629\_Sample\_011046840, Unigene37311\_Sample\_011046840, Unigene22613\_Sample\_011046840, Unigene34070\_Sample\_011046840, Unigene41932\_Sample\_011046840, Unigene32833\_Sample\_011046840, Unigene20100\_Sample\_011046840, Unigene39763\_Sample\_011046840, Unigene41229\_Sample\_011046840, Unigene42995\_Sample\_011046840, Unigene43297\_Sample\_011046840, Unigene39589\_Sample\_011046840, Unigene37863\_Sample\_011046840, Unigene39982\_Sample\_011046840, Unigene11151\_Sample\_011046840, Unigene24036\_Sample\_011046840, Unigene573\_Sample\_011046840, Unigene3770\_Sample\_011046840, Unigene17734\_Sample\_011046840, Unigene10992\_Sample\_011046840, Unigene29802\_Sample\_011046840, Unigene13794\_Sample\_011046840, Unigene24934\_Sample\_011046840, Unigene33749\_Sample\_011046840, Unigene33913\_Sample\_011046840, Unigene24856\_Sample\_011046840, Unigene37444\_Sample\_011046840, Unigene7472\_Sample\_011046840, Unigene4414\_Sample\_011046840, Unigene40877\_Sample\_011046840, Unigene28836\_Sample\_011046840, Unigene5146\_Sample\_011046840, Unigene37933\_Sample\_011046840, Unigene35780\_Sample\_011046840, Unigene10927\_Sample\_011046840, Unigene31301\_Sample\_011046840, Unigene42698\_Sample\_011046840, Unigene41406\_Sample\_011046840, Unigene11913\_Sample\_011046840, Unigene36035\_Sample\_011046840, Unigene29953\_Sample\_011046840, Unigene30142\_Sample\_011046840, Unigene12220\_Sample\_011046840, Unigene11742\_Sample\_011046840, Unigene7137\_Sample\_011046840, Unigene32099\_Sample\_011046840, Unigene1345\_Sample\_011046840, Unigene4825\_Sample\_011046840, Unigene37899\_Sample\_011046840, Unigene38733\_Sample\_011046840, Unigene37451\_Sample\_011046840, Unigene14113\_Sample\_011046840, Unigene3990\_Sample\_011046840, Unigene40004\_Sample\_011046840, Unigene3003\_Sample\_011046840, Unigene41997\_Sample\_011046840, Unigene41587\_Sample\_011046840, Unigene38687\_Sample\_011046840, Unigene35271\_Sample\_011046840, Unigene15302\_Sample\_011046840, Unigene30924\_Sample\_011046840, Unigene38655\_Sample\_011046840, Unigene43473\_Sample\_011046840, Unigene29985\_Sample\_011046840, Unigene34435\_Sample\_011046840, Unigene15608\_Sample\_011046840, Unigene33768\_Sample\_011046840, Unigene26813\_Sample\_011046840, Unigene30528\_Sample\_011046840, Unigene38331\_Sample\_011046840, Unigene26216\_Sample\_011046840, Unigene40015\_Sample\_011046840, Unigene28979\_Sample\_011046840, Unigene42775\_Sample\_011046840, Unigene4744\_Sample\_011046840, Unigene41926\_Sample\_011046840, Unigene1549\_Sample\_011046840, Unigene38671\_Sample\_011046840, Unigene31629\_Sample\_011046840, Unigene24418\_Sample\_011046840, Unigene5087\_Sample\_011046840, Unigene22095\_Sample\_011046840, Unigene30478\_Sample\_011046840, Unigene13562\_Sample\_011046840, Unigene26994\_Sample\_011046840, Unigene39554\_Sample\_011046840, Unigene5867\_Sample\_011046840, Unigene42338\_Sample\_011046840, Unigene6313\_Sample\_011046840, Unigene32005\_Sample\_011046840, Unigene31572\_Sample\_011046840, Unigene28410\_Sample\_011046840, Unigene23029\_Sample\_011046840, Unigene39061\_Sample\_011046840, Unigene33657\_Sample\_011046840, Unigene10533\_Sample\_011046840, Unigene37904\_Sample\_011046840, Unigene34854\_Sample\_011046840, Unigene18334\_Sample\_011046840, Unigene40825\_Sample\_011046840, Unigene37545\_Sample\_011046840, Unigene36084\_Sample\_011046840, Unigene8222\_Sample\_011046840, Unigene39570\_Sample\_011046840, Unigene39888\_Sample\_011046840, Unigene41368\_Sample\_011046840, Unigene34316\_Sample\_011046840, Unigene1485\_Sample\_011046840, Unigene42378\_Sample\_011046840, Unigene35961\_Sample\_011046840, Unigene11174\_Sample\_011046840, Unigene8137\_Sample\_011046840, Unigene17146\_Sample\_011046840, Unigene38709\_Sample\_011046840, Unigene38720\_Sample\_011046840, Unigene31864\_Sample\_011046840, Unigene4344\_Sample\_011046840, Unigene29502\_Sample\_011046840, Unigene39243\_Sample\_011046840, Unigene36654\_Sample\_011046840, Unigene37871\_Sample\_011046840, Unigene2762\_Sample\_011046840, Unigene32612\_Sample\_011046840, Unigene8160\_Sample\_011046840, Unigene32209\_Sample\_011046840, Unigene16376\_Sample\_011046840, Unigene34278\_Sample\_011046840, Unigene39923\_Sample\_011046840, Unigene17019\_Sample\_011046840, Unigene37653\_Sample\_011046840, Unigene24390\_Sample\_011046840, Unigene39165\_Sample\_011046840, Unigene24220\_Sample\_011046840, Unigene13861\_Sample\_011046840, Unigene5541\_Sample\_011046840, Unigene1008\_Sample\_011046840, Unigene26070\_Sample\_011046840, Unigene2863\_Sample\_011046840, Unigene30575\_Sample\_011046840, Unigene31634\_Sample\_011046840, Unigene27458\_Sample\_011046840, Unigene28335\_Sample\_011046840, Unigene31901\_Sample\_011046840, Unigene29526\_Sample\_011046840, Unigene32895\_Sample\_011046840, Unigene41934\_Sample\_011046840, Unigene11454\_Sample\_011046840, Unigene34669\_Sample\_011046840, Unigene25402\_Sample\_011046840, Unigene27955\_Sample\_011046840, Unigene8253\_Sample\_011046840, Unigene34052\_Sample\_011046840, Unigene33222\_Sample\_011046840, Unigene17426\_Sample\_011046840, Unigene43404\_Sample\_011046840, Unigene38467\_Sample\_011046840, Unigene29615\_Sample\_011046840, Unigene35066\_Sample\_011046840, Unigene41540\_Sample\_011046840, Unigene34301\_Sample\_011046840, Unigene29499\_Sample\_011046840, Unigene42019\_Sample\_011046840, Unigene35346\_Sample\_011046840, Unigene789\_Sample\_011046840, Unigene43488\_Sample\_011046840, Unigene35895\_Sample\_011046840, Unigene32844\_Sample\_011046840, Unigene14007\_Sample\_011046840, Unigene1375\_Sample\_011046840, Unigene34436\_Sample\_011046840, Unigene39224\_Sample\_011046840, Unigene43413\_Sample\_011046840, Unigene43492\_Sample\_011046840, Unigene16409\_Sample\_011046840, Unigene7545\_Sample\_011046840, Unigene39296\_Sample\_011046840, Unigene341\_Sample\_011046840, Unigene30024\_Sample\_011046840, Unigene38077\_Sample\_011046840, Unigene7175\_Sample\_011046840, Unigene7113\_Sample\_011046840, Unigene20160\_Sample\_011046840, Unigene42272\_Sample\_011046840, Unigene8086\_Sample\_011046840, Unigene15260\_Sample\_011046840, Unigene42713\_Sample\_011046840, Unigene2820\_Sample\_011046840, Unigene40131\_Sample\_011046840, Unigene14074\_Sample\_011046840, Unigene29764\_Sample\_011046840, Unigene29769\_Sample\_011046840, Unigene40658\_Sample\_011046840, Unigene15680\_Sample\_011046840, Unigene35547\_Sample\_011046840, Unigene22514\_Sample\_011046840, Unigene27933\_Sample\_011046840, Unigene35731\_Sample\_011046840, Unigene33431\_Sample\_011046840, Unigene4839\_Sample\_011046840, Unigene35206\_Sample\_011046840, Unigene42012\_Sample\_011046840, Unigene35159\_Sample\_011046840, Unigene7427\_Sample\_011046840, Unigene27496\_Sample\_011046840, Unigene42659\_Sample\_011046840, Unigene37034\_Sample\_011046840, Unigene18009\_Sample\_011046840, Unigene38768\_Sample\_011046840, Unigene43318\_Sample\_011046840, Unigene32425\_Sample\_011046840, Unigene20494\_Sample\_011046840, Unigene41585\_Sample\_011046840, Unigene25046\_Sample\_011046840, Unigene28695\_Sample\_011046840, Unigene36393\_Sample\_011046840, Unigene5809\_Sample\_011046840, Unigene35693\_Sample\_011046840, Unigene6088\_Sample\_011046840, Unigene17840\_Sample\_011046840, Unigene20467\_Sample\_011046840, Unigene34194\_Sample\_011046840, Unigene42575\_Sample\_011046840, Unigene9133\_Sample\_011046840, Unigene37766\_Sample\_011046840, Unigene30495\_Sample\_011046840, Unigene27213\_Sample\_011046840, Unigene28516\_Sample\_011046840, Unigene34588\_Sample\_011046840, Unigene34929\_Sample\_011046840, Unigene34046\_Sample\_011046840, Unigene19619\_Sample\_011046840, Unigene40793\_Sample\_011046840, Unigene28684\_Sample\_011046840, Unigene4979\_Sample\_011046840, Unigene2123\_Sample\_011046840, Unigene22165\_Sample\_011046840, Unigene24711\_Sample\_011046840, Unigene24034\_Sample\_011046840, Unigene4822\_Sample\_011046840, Unigene4085\_Sample\_011046840, Unigene41899\_Sample\_011046840, Unigene36997\_Sample\_011046840, Unigene8956\_Sample\_011046840, Unigene38357\_Sample\_011046840, Unigene16941\_Sample\_011046840, Unigene8240\_Sample\_011046840, Unigene27829\_Sample\_011046840, Unigene12936\_Sample\_011046840, Unigene4591\_Sample\_011046840, Unigene43073\_Sample\_011046840, Unigene42283\_Sample\_011046840, Unigene40123\_Sample\_011046840, Unigene38028\_Sample\_011046840, Unigene40677\_Sample\_011046840, Unigene35179\_Sample\_011046840, Unigene40861\_Sample\_011046840, Unigene8019\_Sample\_011046840, Unigene39223\_Sample\_011046840, Unigene39446\_Sample\_011046840, Unigene18919\_Sample\_011046840, Unigene5953\_Sample\_011046840, Unigene43278\_Sample\_011046840, Unigene42206\_Sample\_011046840, Unigene420\_Sample\_011046840, Unigene10640\_Sample\_011046840, Unigene16082\_Sample\_011046840, Unigene20936\_Sample\_011046840, Unigene17259\_Sample\_011046840, Unigene35286\_Sample\_011046840, Unigene15508\_Sample\_011046840, Unigene39151\_Sample\_011046840, Unigene38104\_Sample\_011046840, Unigene43236\_Sample\_011046840, Unigene43127\_Sample\_011046840, Unigene7669\_Sample\_011046840, Unigene3517\_Sample\_011046840, Unigene36700\_Sample\_011046840, Unigene9113\_Sample\_011046840, Unigene22229\_Sample\_011046840, Unigene27942\_Sample\_011046840, Unigene29255\_Sample\_011046840, Unigene36048\_Sample\_011046840, Unigene30713\_Sample\_011046840, Unigene36942\_Sample\_011046840, Unigene16663\_Sample\_011046840, Unigene23409\_Sample\_011046840, Unigene39199\_Sample\_011046840, Unigene32899\_Sample\_011046840, Unigene36459\_Sample\_011046840, Unigene36813\_Sample\_011046840, Unigene39361\_Sample\_011046840, Unigene28409\_Sample\_011046840, Unigene2111\_Sample\_011046840, Unigene31120\_Sample\_011046840, Unigene40942\_Sample\_011046840, Unigene22310\_Sample\_011046840, Unigene14947\_Sample\_011046840, Unigene22599\_Sample\_011046840, Unigene9531\_Sample\_011046840, Unigene43610\_Sample\_011046840, Unigene40822\_Sample\_011046840, Unigene42964\_Sample\_011046840, Unigene13511\_Sample\_011046840, Unigene21015\_Sample\_011046840, Unigene43624\_Sample\_011046840, Unigene42483\_Sample\_011046840, Unigene16898\_Sample\_011046840, Unigene35244\_Sample\_011046840, Unigene41101\_Sample\_011046840, Unigene42596\_Sample\_011046840, Unigene40511\_Sample\_011046840, Unigene7067\_Sample\_011046840, Unigene32822\_Sample\_011046840, Unigene34033\_Sample\_011046840, Unigene28597\_Sample\_011046840, Unigene39581\_Sample\_011046840, Unigene41233\_Sample\_011046840, Unigene31168\_Sample\_011046840, Unigene26581\_Sample\_011046840, Unigene33837\_Sample\_011046840, Unigene29655\_Sample\_011046840, Unigene39690\_Sample\_011046840, Unigene40422\_Sample\_011046840, Unigene39503\_Sample\_011046840, Unigene24344\_Sample\_011046840, Unigene28415\_Sample\_011046840, Unigene10804\_Sample\_011046840, Unigene18504\_Sample\_011046840, Unigene11629\_Sample\_011046840, Unigene34862\_Sample\_011046840, Unigene2454\_Sample\_011046840, Unigene26060\_Sample\_011046840, Unigene8043\_Sample\_011046840, Unigene40564\_Sample\_011046840, Unigene39271\_Sample\_011046840, Unigene32436\_Sample\_011046840, Unigene35396\_Sample\_011046840, Unigene8372\_Sample\_011046840, Unigene42455\_Sample\_011046840, Unigene39178\_Sample\_011046840, Unigene28581\_Sample\_011046840, Unigene40966\_Sample\_011046840, Unigene13884\_Sample\_011046840, Unigene43423\_Sample\_011046840, Unigene34790\_Sample\_011046840, Unigene39962\_Sample\_011046840, Unigene37935\_Sample\_011046840, Unigene13388\_Sample\_011046840, Unigene36114\_Sample\_011046840, Unigene6445\_Sample\_011046840, Unigene30903\_Sample\_011046840, Unigene42097\_Sample\_011046840, Unigene5915\_Sample\_011046840, Unigene41398\_Sample\_011046840, Unigene41081\_Sample\_011046840, Unigene43361\_Sample\_011046840, Unigene28840\_Sample\_011046840, Unigene43533\_Sample\_011046840, Unigene34839\_Sample\_011046840, Unigene38011\_Sample\_011046840, Unigene7280\_Sample\_011046840, Unigene39389\_Sample\_011046840, Unigene18382\_Sample\_011046840, Unigene21475\_Sample\_011046840, Unigene38159\_Sample\_011046840, Unigene33673\_Sample\_011046840, Unigene4387\_Sample\_011046840, Unigene40560\_Sample\_011046840, Unigene22669\_Sample\_011046840, Unigene5733\_Sample\_011046840, Unigene29895\_Sample\_011046840, Unigene26628\_Sample\_011046840, Unigene11583\_Sample\_011046840, Unigene39063\_Sample\_011046840, Unigene37873\_Sample\_011046840, Unigene32695\_Sample\_011046840, Unigene33052\_Sample\_011046840, Unigene25735\_Sample\_011046840, Unigene34637\_Sample\_011046840, Unigene1215\_Sample\_011046840, Unigene34855\_Sample\_011046840, Unigene15111\_Sample\_011046840, Unigene26170\_Sample\_011046840, Unigene1398\_Sample\_011046840, Unigene29397\_Sample\_011046840, Unigene8123\_Sample\_011046840, Unigene32834\_Sample\_011046840, Unigene39279\_Sample\_011046840, Unigene42650\_Sample\_011046840, Unigene28450\_Sample\_011046840, Unigene32858\_Sample\_011046840, Unigene38944\_Sample\_011046840, Unigene32857\_Sample\_011046840, Unigene22531\_Sample\_011046840, Unigene42585\_Sample\_011046840, Unigene8205\_Sample\_011046840, Unigene15594\_Sample\_011046840, Unigene9954\_Sample\_011046840, Unigene7974\_Sample\_011046840, Unigene3810\_Sample\_011046840, Unigene38003\_Sample\_011046840, Unigene38402\_Sample\_011046840, Unigene19563\_Sample\_011046840, Unigene40629\_Sample\_011046840, Unigene41502\_Sample\_011046840, Unigene30226\_Sample\_011046840, Unigene7294\_Sample\_011046840, Unigene7892\_Sample\_011046840, Unigene38207\_Sample\_011046840, Unigene18685\_Sample\_011046840, Unigene42359\_Sample\_011046840, Unigene41314\_Sample\_011046840, Unigene24259\_Sample\_011046840, Unigene17569\_Sample\_011046840, Unigene4443\_Sample\_011046840, Unigene25769\_Sample\_011046840, Unigene41000\_Sample\_011046840, Unigene37514\_Sample\_011046840, Unigene36854\_Sample\_011046840, Unigene42988\_Sample\_011046840, Unigene32735\_Sample\_011046840, Unigene39086\_Sample\_011046840, Unigene35969\_Sample\_011046840, Unigene43066\_Sample\_011046840, Unigene10054\_Sample\_011046840, Unigene38822\_Sample\_011046840, Unigene9950\_Sample\_011046840, Unigene38088\_Sample\_011046840, Unigene34784\_Sample\_011046840, Unigene38534\_Sample\_011046840, Unigene41512\_Sample\_011046840, Unigene2789\_Sample\_011046840, Unigene8373\_Sample\_011046840, Unigene40856\_Sample\_011046840, Unigene30467\_Sample\_011046840, Unigene40811\_Sample\_011046840, Unigene39951\_Sample\_011046840, Unigene38730\_Sample\_011046840, Unigene43450\_Sample\_011046840, Unigene35452\_Sample\_011046840, Unigene40192\_Sample\_011046840, Unigene42554\_Sample\_011046840, Unigene38699\_Sample\_011046840, Unigene42105\_Sample\_011046840, Unigene27254\_Sample\_011046840, Unigene23020\_Sample\_011046840, Unigene42287\_Sample\_011046840, Unigene4612\_Sample\_011046840, Unigene34973\_Sample\_011046840, Unigene43494\_Sample\_011046840, Unigene41679\_Sample\_011046840, Unigene41716\_Sample\_011046840, Unigene1226\_Sample\_011046840, Unigene37469\_Sample\_011046840, Unigene36671\_Sample\_011046840, Unigene11390\_Sample\_011046840, Unigene35859\_Sample\_011046840, Unigene14242\_Sample\_011046840, Unigene12277\_Sample\_011046840, Unigene1252\_Sample\_011046840, Unigene41439\_Sample\_011046840, Unigene32598\_Sample\_011046840, Unigene20078\_Sample\_011046840, Unigene23797\_Sample\_011046840, Unigene40439\_Sample\_011046840, Unigene11208\_Sample\_011046840, Unigene43019\_Sample\_011046840, Unigene1967\_Sample\_011046840, Unigene34992\_Sample\_011046840, Unigene6181\_Sample\_011046840, Unigene29823\_Sample\_011046840, Unigene28676\_Sample\_011046840, Unigene9833\_Sample\_011046840, Unigene43336\_Sample\_011046840, Unigene38363\_Sample\_011046840, Unigene5316\_Sample\_011046840, Unigene19756\_Sample\_011046840, Unigene18015\_Sample\_011046840, Unigene36713\_Sample\_011046840, Unigene41507\_Sample\_011046840, Unigene29814\_Sample\_011046840, Unigene42849\_Sample\_011046840, Unigene31212\_Sample\_011046840, Unigene31960\_Sample\_011046840, Unigene34158\_Sample\_011046840, Unigene4234\_Sample\_011046840, Unigene34532\_Sample\_011046840, Unigene27358\_Sample\_011046840, Unigene13113\_Sample\_011046840, Unigene39033\_Sample\_011046840, Unigene19258\_Sample\_011046840, Unigene20546\_Sample\_011046840, Unigene42782\_Sample\_011046840, Unigene2439\_Sample\_011046840, Unigene20430\_Sample\_011046840, Unigene35676\_Sample\_011046840, Unigene42514\_Sample\_011046840, Unigene34471\_Sample\_011046840, Unigene37359\_Sample\_011046840, Unigene9750\_Sample\_011046840, Unigene39080\_Sample\_011046840, Unigene18477\_Sample\_011046840, Unigene43197\_Sample\_011046840, Unigene30634\_Sample\_011046840, Unigene5774\_Sample\_011046840, Unigene39136\_Sample\_011046840, Unigene36248\_Sample\_011046840, Unigene13709\_Sample\_011046840, Unigene37738\_Sample\_011046840, Unigene39725\_Sample\_011046840, Unigene26517\_Sample\_011046840, Unigene27975\_Sample\_011046840, Unigene43263\_Sample\_011046840, Unigene32145\_Sample\_011046840, Unigene14511\_Sample\_011046840, Unigene8171\_Sample\_011046840, Unigene32749\_Sample\_011046840, Unigene14894\_Sample\_011046840, Unigene5322\_Sample\_011046840, Unigene12521\_Sample\_011046840, Unigene19469\_Sample\_011046840, Unigene12964\_Sample\_011046840, Unigene42377\_Sample\_011046840, Unigene16269\_Sample\_011046840, Unigene2536\_Sample\_011046840, Unigene41114\_Sample\_011046840, Unigene32865\_Sample\_011046840, Unigene32172\_Sample\_011046840, Unigene32463\_Sample\_011046840, Unigene40065\_Sample\_011046840, Unigene4481\_Sample\_011046840, Unigene4583\_Sample\_011046840, Unigene42935\_Sample\_011046840, Unigene39866\_Sample\_011046840, Unigene41562\_Sample\_011046840, Unigene23252\_Sample\_011046840, Unigene14195\_Sample\_011046840, Unigene32781\_Sample\_011046840, Unigene34547\_Sample\_011046840, Unigene13360\_Sample\_011046840, Unigene43314\_Sample\_011046840, Unigene34481\_Sample\_011046840, Unigene11179\_Sample\_011046840, Unigene40994\_Sample\_011046840, Unigene18714\_Sample\_011046840, Unigene38990\_Sample\_011046840, Unigene38406\_Sample\_011046840, Unigene36936\_Sample\_011046840, Unigene38017\_Sample\_011046840, Unigene5461\_Sample\_011046840, Unigene31532\_Sample\_011046840, Unigene23357\_Sample\_011046840, Unigene39992\_Sample\_011046840, Unigene8218\_Sample\_011046840, Unigene38459\_Sample\_011046840, Unigene33047\_Sample\_011046840, Unigene24731\_Sample\_011046840, Unigene41792\_Sample\_011046840, Unigene41441\_Sample\_011046840, Unigene40136\_Sample\_011046840, Unigene40202\_Sample\_011046840, Unigene33426\_Sample\_011046840, Unigene14381\_Sample\_011046840, Unigene6486\_Sample\_011046840, Unigene2480\_Sample\_011046840, Unigene41914\_Sample\_011046840, Unigene9299\_Sample\_011046840, Unigene28958\_Sample\_011046840, Unigene40424\_Sample\_011046840, Unigene25502\_Sample\_011046840, Unigene37081\_Sample\_011046840, Unigene11225\_Sample\_011046840, Unigene39665\_Sample\_011046840, Unigene30457\_Sample\_011046840, Unigene14002\_Sample\_011046840, Unigene5038\_Sample\_011046840, Unigene21444\_Sample\_011046840, Unigene43427\_Sample\_011046840, Unigene40124\_Sample\_011046840, Unigene36626\_Sample\_011046840, Unigene31258\_Sample\_011046840, Unigene43431\_Sample\_011046840, Unigene30067\_Sample\_011046840, Unigene33634\_Sample\_011046840, Unigene17531\_Sample\_011046840, Unigene37708\_Sample\_011046840, Unigene36139\_Sample\_011046840, Unigene29297\_Sample\_011046840, Unigene35421\_Sample\_011046840, Unigene24992\_Sample\_011046840, Unigene9448\_Sample\_011046840, Unigene37830\_Sample\_011046840, Unigene30708\_Sample\_011046840, Unigene33613\_Sample\_011046840, Unigene42985\_Sample\_011046840, Unigene42582\_Sample\_011046840, Unigene41740\_Sample\_011046840, Unigene20296\_Sample\_011046840, Unigene37841\_Sample\_011046840, Unigene40346\_Sample\_011046840, Unigene32864\_Sample\_011046840, Unigene38418\_Sample\_011046840, Unigene21000\_Sample\_011046840, Unigene30151\_Sample\_011046840, Unigene27532\_Sample\_011046840, Unigene6114\_Sample\_011046840, Unigene30699\_Sample\_011046840, Unigene1920\_Sample\_011046840, Unigene2470\_Sample\_011046840, Unigene3842\_Sample\_011046840, Unigene12743\_Sample\_011046840, Unigene41990\_Sample\_011046840, Unigene34257\_Sample\_011046840, Unigene11707\_Sample\_011046840, Unigene10770\_Sample\_011046840, Unigene22560\_Sample\_011046840, Unigene32952\_Sample\_011046840, Unigene41139\_Sample\_011046840, Unigene41158\_Sample\_011046840, Unigene4492\_Sample\_011046840, Unigene16736\_Sample\_011046840, Unigene34625\_Sample\_011046840, Unigene20308\_Sample\_011046840, Unigene33698\_Sample\_011046840, Unigene31578\_Sample\_011046840, Unigene31239\_Sample\_011046840, Unigene37858\_Sample\_011046840, Unigene40137\_Sample\_011046840, Unigene16329\_Sample\_011046840, Unigene31582\_Sample\_011046840, Unigene17214\_Sample\_011046840, Unigene22216\_Sample\_011046840, Unigene31392\_Sample\_011046840, Unigene34877\_Sample\_011046840, Unigene30518\_Sample\_011046840, Unigene28689\_Sample\_011046840, Unigene10135\_Sample\_011046840, Unigene15362\_Sample\_011046840, Unigene17976\_Sample\_011046840, Unigene5517\_Sample\_011046840, Unigene33227\_Sample\_011046840, Unigene35723\_Sample\_011046840, Unigene23571\_Sample\_011046840, Unigene3826\_Sample\_011046840, Unigene32059\_Sample\_011046840, Unigene42977\_Sample\_011046840, Unigene37209\_Sample\_011046840, Unigene24484\_Sample\_011046840, Unigene30332\_Sample\_011046840, Unigene886\_Sample\_011046840, Unigene2053\_Sample\_011046840, Unigene42591\_Sample\_011046840, Unigene30221\_Sample\_011046840, Unigene37891\_Sample\_011046840, Unigene43457\_Sample\_011046840, Unigene14666\_Sample\_011046840, Unigene27879\_Sample\_011046840, Unigene40973\_Sample\_011046840, Unigene41654\_Sample\_011046840, Unigene43146\_Sample\_011046840, Unigene23684\_Sample\_011046840, Unigene39591\_Sample\_011046840, Unigene42683\_Sample\_011046840, Unigene29099\_Sample\_011046840, Unigene19383\_Sample\_011046840, Unigene34242\_Sample\_011046840, Unigene5289\_Sample\_011046840, Unigene37452\_Sample\_011046840, Unigene25871\_Sample\_011046840, Unigene42725\_Sample\_011046840, Unigene30573\_Sample\_011046840, Unigene34746\_Sample\_011046840, Unigene4863\_Sample\_011046840, Unigene24267\_Sample\_011046840, Unigene26273\_Sample\_011046840, Unigene33040\_Sample\_011046840, Unigene40812\_Sample\_011046840, Unigene39303\_Sample\_011046840, Unigene40787\_Sample\_011046840, Unigene22327\_Sample\_011046840, Unigene24674\_Sample\_011046840, Unigene18900\_Sample\_011046840, Unigene4494\_Sample\_011046840, Unigene36969\_Sample\_011046840, Unigene33889\_Sample\_011046840, Unigene1815\_Sample\_011046840, Unigene37365\_Sample\_011046840, Unigene25523\_Sample\_011046840, Unigene31910\_Sample\_011046840, Unigene43453\_Sample\_011046840, Unigene41479\_Sample\_011046840, Unigene14648\_Sample\_011046840, Unigene6446\_Sample\_011046840, Unigene28929\_Sample\_011046840, Unigene38951\_Sample\_011046840, Unigene13717\_Sample\_011046840, Unigene8520\_Sample\_011046840, Unigene39795\_Sample\_011046840, Unigene9027\_Sample\_011046840, Unigene26557\_Sample\_011046840, Unigene43031\_Sample\_011046840, Unigene34704\_Sample\_011046840, Unigene41868\_Sample\_011046840, Unigene42068\_Sample\_011046840, Unigene27364\_Sample\_011046840, Unigene40774\_Sample\_011046840, Unigene41561\_Sample\_011046840, Unigene7884\_Sample\_011046840, Unigene18132\_Sample\_011046840, Unigene40246\_Sample\_011046840, Unigene40731\_Sample\_011046840, Unigene37244\_Sample\_011046840, Unigene28923\_Sample\_011046840, Unigene42201\_Sample\_011046840, Unigene3161\_Sample\_011046840, Unigene40387\_Sample\_011046840, Unigene42944\_Sample\_011046840, Unigene4541\_Sample\_011046840, Unigene24509\_Sample\_011046840, Unigene35324\_Sample\_011046840, Unigene10956\_Sample\_011046840, Unigene5724\_Sample\_011046840, Unigene20549\_Sample\_011046840, Unigene7952\_Sample\_011046840, Unigene40924\_Sample\_011046840, Unigene5782\_Sample\_011046840, Unigene14078\_Sample\_011046840, Unigene35663\_Sample\_011046840, Unigene42803\_Sample\_011046840, Unigene37573\_Sample\_011046840, Unigene6894\_Sample\_011046840, Unigene31793\_Sample\_011046840, Unigene37879\_Sample\_011046840, Unigene42475\_Sample\_011046840, Unigene39986\_Sample\_011046840, Unigene30897\_Sample\_011046840, Unigene41783\_Sample\_011046840, Unigene39416\_Sample\_011046840, Unigene34233\_Sample\_011046840, Unigene20783\_Sample\_011046840, Unigene42252\_Sample\_011046840, Unigene40728\_Sample\_011046840, Unigene31324\_Sample\_011046840, Unigene41821\_Sample\_011046840, Unigene37470\_Sample\_011046840, Unigene42530\_Sample\_011046840, Unigene40878\_Sample\_011046840, Unigene43616\_Sample\_011046840, Unigene29952\_Sample\_011046840, Unigene35996\_Sample\_011046840, Unigene43562\_Sample\_011046840, Unigene28531\_Sample\_011046840, Unigene14237\_Sample\_011046840, Unigene41778\_Sample\_011046840, Unigene2626\_Sample\_011046840, Unigene3395\_Sample\_011046840, Unigene37277\_Sample\_011046840, Unigene19433\_Sample\_011046840, Unigene39318\_Sample\_011046840, Unigene35817\_Sample\_011046840, Unigene33515\_Sample\_011046840, Unigene31044\_Sample\_011046840, Unigene33378\_Sample\_011046840, Unigene2332\_Sample\_011046840, Unigene3164\_Sample\_011046840, Unigene42335\_Sample\_011046840, Unigene35417\_Sample\_011046840, Unigene40490\_Sample\_011046840, Unigene24936\_Sample\_011046840, Unigene10460\_Sample\_011046840, Unigene26781\_Sample\_011046840, Unigene34355\_Sample\_011046840, Unigene40333\_Sample\_011046840, Unigene36929\_Sample\_011046840, Unigene7983\_Sample\_011046840, Unigene41606\_Sample\_011046840, Unigene32385\_Sample\_011046840, Unigene37702\_Sample\_011046840, Unigene8751\_Sample\_011046840, Unigene20612\_Sample\_011046840, Unigene43243\_Sample\_011046840, Unigene10540\_Sample\_011046840, Unigene31911\_Sample\_011046840, Unigene43525\_Sample\_011046840, Unigene4020\_Sample\_011046840, Unigene3241\_Sample\_011046840, Unigene39639\_Sample\_011046840, Unigene25328\_Sample\_011046840, Unigene22550\_Sample\_011046840, Unigene32853\_Sample\_011046840, Unigene12730\_Sample\_011046840, Unigene28259\_Sample\_011046840, Unigene37149\_Sample\_011046840, Unigene38753\_Sample\_011046840, Unigene5305\_Sample\_011046840, Unigene38000\_Sample\_011046840, Unigene35724\_Sample\_011046840, Unigene41092\_Sample\_011046840, Unigene8864\_Sample\_011046840, Unigene28670\_Sample\_011046840, Unigene12191\_Sample\_011046840, Unigene35234\_Sample\_011046840, Unigene34850\_Sample\_011046840, Unigene14154\_Sample\_011046840, Unigene32278\_Sample\_011046840, Unigene27950\_Sample\_011046840, Unigene21660\_Sample\_011046840, Unigene29386\_Sample\_011046840, Unigene15458\_Sample\_011046840, Unigene30466\_Sample\_011046840, Unigene7536\_Sample\_011046840, Unigene242\_Sample\_011046840, Unigene5710\_Sample\_011046840, Unigene34508\_Sample\_011046840, Unigene17239\_Sample\_011046840, Unigene143\_Sample\_011046840, Unigene42215\_Sample\_011046840, Unigene16751\_Sample\_011046840, Unigene33538\_Sample\_011046840, Unigene29117\_Sample\_011046840, Unigene23811\_Sample\_011046840, Unigene38199\_Sample\_011046840, Unigene7643\_Sample\_011046840, Unigene41574\_Sample\_011046840, Unigene15420\_Sample\_011046840, Unigene33085\_Sample\_011046840, Unigene40362\_Sample\_011046840, Unigene33736\_Sample\_011046840, Unigene17665\_Sample\_011046840, Unigene37299\_Sample\_011046840, Unigene28039\_Sample\_011046840, Unigene32875\_Sample\_011046840, Unigene12062\_Sample\_011046840, Unigene36669\_Sample\_011046840, Unigene42386\_Sample\_011046840, Unigene7734\_Sample\_011046840, Unigene34509\_Sample\_011046840, Unigene36260\_Sample\_011046840, Unigene40376\_Sample\_011046840, Unigene16236\_Sample\_011046840, Unigene29599\_Sample\_011046840, Unigene38444\_Sample\_011046840, Unigene29696\_Sample\_011046840, Unigene7189\_Sample\_011046840, Unigene8155\_Sample\_011046840, Unigene39526\_Sample\_011046840, Unigene19098\_Sample\_011046840, Unigene39429\_Sample\_011046840, Unigene39339\_Sample\_011046840, Unigene15504\_Sample\_011046840, Unigene13340\_Sample\_011046840, Unigene27682\_Sample\_011046840, Unigene6624\_Sample\_011046840, Unigene40042\_Sample\_011046840, Unigene28521\_Sample\_011046840, Unigene32354\_Sample\_011046840, Unigene39248\_Sample\_011046840, Unigene42569\_Sample\_011046840, Unigene36007\_Sample\_011046840, Unigene8002\_Sample\_011046840, Unigene40462\_Sample\_011046840, Unigene25024\_Sample\_011046840, Unigene6711\_Sample\_011046840, Unigene23891\_Sample\_011046840, Unigene23122\_Sample\_011046840, Unigene37267\_Sample\_011046840, Unigene26865\_Sample\_011046840, Unigene32927\_Sample\_011046840, Unigene23169\_Sample\_011046840, Unigene33810\_Sample\_011046840, Unigene41818\_Sample\_011046840, Unigene16223\_Sample\_011046840, Unigene12911\_Sample\_011046840, Unigene41980\_Sample\_011046840, Unigene42325\_Sample\_011046840, Unigene40919\_Sample\_011046840, Unigene14503\_Sample\_011046840, Unigene22739\_Sample\_011046840, Unigene38058\_Sample\_011046840, Unigene39304\_Sample\_011046840, Unigene33699\_Sample\_011046840, Unigene36391\_Sample\_011046840, Unigene38722\_Sample\_011046840, Unigene3192\_Sample\_011046840, Unigene40469\_Sample\_011046840, Unigene12359\_Sample\_011046840, Unigene12476\_Sample\_011046840, Unigene23566\_Sample\_011046840, Unigene37124\_Sample\_011046840, Unigene32723\_Sample\_011046840, Unigene13989\_Sample\_011046840, Unigene27850\_Sample\_011046840, Unigene40826\_Sample\_011046840, Unigene32621\_Sample\_011046840, Unigene23801\_Sample\_011046840, Unigene36342\_Sample\_011046840, Unigene42634\_Sample\_011046840, Unigene29076\_Sample\_011046840, Unigene43560\_Sample\_011046840, Unigene34605\_Sample\_011046840, Unigene3752\_Sample\_011046840, Unigene35459\_Sample\_011046840, Unigene6135\_Sample\_011046840, Unigene16550\_Sample\_011046840, Unigene16850\_Sample\_011046840, Unigene13232\_Sample\_011046840, Unigene28549\_Sample\_011046840, Unigene37057\_Sample\_011046840, Unigene14853\_Sample\_011046840, Unigene42994\_Sample\_011046840, Unigene37045\_Sample\_011046840, Unigene4497\_Sample\_011046840, Unigene12675\_Sample\_011046840, Unigene188\_Sample\_011046840, Unigene6461\_Sample\_011046840, Unigene27316\_Sample\_011046840, Unigene24183\_Sample\_011046840, Unigene30537\_Sample\_011046840, Unigene43282\_Sample\_011046840, Unigene22747\_Sample\_011046840, Unigene22665\_Sample\_011046840, Unigene38851\_Sample\_011046840, Unigene20154\_Sample\_011046840, Unigene2690\_Sample\_011046840, Unigene41052\_Sample\_011046840, Unigene33392\_Sample\_011046840, Unigene16079\_Sample\_011046840, Unigene3068\_Sample\_011046840, Unigene43238\_Sample\_011046840, Unigene41943\_Sample\_011046840, Unigene37309\_Sample\_011046840, Unigene28312\_Sample\_011046840, Unigene40753\_Sample\_011046840, Unigene27006\_Sample\_011046840, Unigene20800\_Sample\_011046840, Unigene27473\_Sample\_011046840, Unigene26705\_Sample\_011046840, Unigene1620\_Sample\_011046840, Unigene31527\_Sample\_011046840, Unigene22626\_Sample\_011046840, Unigene141\_Sample\_011046840, Unigene35146\_Sample\_011046840, Unigene38900\_Sample\_011046840, Unigene8166\_Sample\_011046840, Unigene13275\_Sample\_011046840, Unigene9759\_Sample\_011046840, Unigene31035\_Sample\_011046840, Unigene21132\_Sample\_011046840, Unigene34039\_Sample\_011046840, Unigene6582\_Sample\_011046840, Unigene31560\_Sample\_011046840, Unigene40885\_Sample\_011046840, Unigene41752\_Sample\_011046840, Unigene21415\_Sample\_011046840, Unigene38412\_Sample\_011046840, Unigene29975\_Sample\_011046840, Unigene25346\_Sample\_011046840, Unigene42297\_Sample\_011046840, Unigene23889\_Sample\_011046840, Unigene43444\_Sample\_011046840, Unigene30704\_Sample\_011046840, Unigene30564\_Sample\_011046840, Unigene42481\_Sample\_011046840, Unigene40738\_Sample\_011046840, Unigene32589\_Sample\_011046840, Unigene7448\_Sample\_011046840, Unigene38998\_Sample\_011046840, Unigene13731\_Sample\_011046840, Unigene31084\_Sample\_011046840, Unigene5453\_Sample\_011046840, Unigene36253\_Sample\_011046840, Unigene12207\_Sample\_011046840, Unigene8026\_Sample\_011046840, Unigene41196\_Sample\_011046840, Unigene29253\_Sample\_011046840, Unigene38935\_Sample\_011046840, Unigene42550\_Sample\_011046840, Unigene6938\_Sample\_011046840, Unigene6651\_Sample\_011046840, Unigene41405\_Sample\_011046840, Unigene11522\_Sample\_011046840, Unigene28397\_Sample\_011046840, Unigene36899\_Sample\_011046840, Unigene36299\_Sample\_011046840, Unigene31914\_Sample\_011046840, Unigene6987\_Sample\_011046840, Unigene16486\_Sample\_011046840, Unigene26212\_Sample\_011046840, Unigene42202\_Sample\_011046840, Unigene40593\_Sample\_011046840, Unigene7169\_Sample\_011046840, Unigene6075\_Sample\_011046840, Unigene23026\_Sample\_011046840, Unigene2068\_Sample\_011046840, Unigene333\_Sample\_011046840, Unigene41982\_Sample\_011046840, Unigene35105\_Sample\_011046840, Unigene27013\_Sample\_011046840, Unigene30486\_Sample\_011046840, Unigene21529\_Sample\_011046840, Unigene1926\_Sample\_011046840, Unigene30435\_Sample\_011046840, Unigene8059\_Sample\_011046840, Unigene42320\_Sample\_011046840, Unigene26065\_Sample\_011046840, Unigene39582\_Sample\_011046840, Unigene3769\_Sample\_011046840, Unigene41799\_Sample\_011046840, Unigene25305\_Sample\_011046840, Unigene35170\_Sample\_011046840, Unigene19905\_Sample\_011046840, Unigene18537\_Sample\_011046840, Unigene22397\_Sample\_011046840, Unigene26110\_Sample\_011046840, Unigene20109\_Sample\_011046840, Unigene11458\_Sample\_011046840, Unigene20540\_Sample\_011046840, Unigene26932\_Sample\_011046840, Unigene31695\_Sample\_011046840, Unigene42116\_Sample\_011046840, Unigene42354\_Sample\_011046840, Unigene1456\_Sample\_011046840, Unigene43014\_Sample\_011046840, Unigene34328\_Sample\_011046840, Unigene34437\_Sample\_011046840, Unigene39254\_Sample\_011046840, Unigene15681\_Sample\_011046840, Unigene21699\_Sample\_011046840, Unigene7058\_Sample\_011046840, Unigene38329\_Sample\_011046840, Unigene41337\_Sample\_011046840, Unigene36070\_Sample\_011046840, Unigene41563\_Sample\_011046840, Unigene34874\_Sample\_011046840, Unigene39657\_Sample\_011046840, Unigene7525\_Sample\_011046840, Unigene40064\_Sample\_011046840, Unigene43080\_Sample\_011046840, Unigene39628\_Sample\_011046840, Unigene24129\_Sample\_011046840, Unigene26217\_Sample\_011046840, Unigene31662\_Sample\_011046840, Unigene13805\_Sample\_011046840, Unigene42955\_Sample\_011046840, Unigene13117\_Sample\_011046840, Unigene4807\_Sample\_011046840, Unigene15087\_Sample\_011046840, Unigene35427\_Sample\_011046840, Unigene39204\_Sample\_011046840, Unigene39588\_Sample\_011046840, Unigene5908\_Sample\_011046840, Unigene1271\_Sample\_011046840, Unigene35008\_Sample\_011046840, Unigene11193\_Sample\_011046840, Unigene29870\_Sample\_011046840, Unigene41672\_Sample\_011046840, Unigene14816\_Sample\_011046840, Unigene6438\_Sample\_011046840, Unigene11100\_Sample\_011046840, Unigene28055\_Sample\_011046840, Unigene11043\_Sample\_011046840, Unigene11820\_Sample\_011046840, Unigene39649\_Sample\_011046840, Unigene2986\_Sample\_011046840, Unigene31576\_Sample\_011046840, Unigene43486\_Sample\_011046840, Unigene35141\_Sample\_011046840, Unigene37516\_Sample\_011046840, Unigene43218\_Sample\_011046840, Unigene18284\_Sample\_011046840, Unigene10850\_Sample\_011046840, Unigene7429\_Sample\_011046840, Unigene7825\_Sample\_011046840, Unigene3974\_Sample\_011046840, Unigene34610\_Sample\_011046840, Unigene11221\_Sample\_011046840, Unigene40602\_Sample\_011046840, Unigene43040\_Sample\_011046840, Unigene32583\_Sample\_011046840, Unigene35195\_Sample\_011046840, Unigene35735\_Sample\_011046840, Unigene31646\_Sample\_011046840, Unigene33659\_Sample\_011046840, Unigene34909\_Sample\_011046840, Unigene41427\_Sample\_011046840, Unigene6899\_Sample\_011046840, Unigene21598\_Sample\_011046840, Unigene34036\_Sample\_011046840, Unigene7221\_Sample\_011046840, Unigene42376\_Sample\_011046840, Unigene39974\_Sample\_011046840, Unigene39644\_Sample\_011046840, Unigene16618\_Sample\_011046840, Unigene3258\_Sample\_011046840, Unigene40298\_Sample\_011046840, Unigene13322\_Sample\_011046840, Unigene34023\_Sample\_011046840, Unigene3320\_Sample\_011046840, Unigene32533\_Sample\_011046840, Unigene35568\_Sample\_011046840, Unigene34987\_Sample\_011046840, Unigene41978\_Sample\_011046840, Unigene38773\_Sample\_011046840, Unigene4652\_Sample\_011046840, Unigene40452\_Sample\_011046840, Unigene13531\_Sample\_011046840, Unigene34137\_Sample\_011046840, Unigene29734\_Sample\_011046840, Unigene37194\_Sample\_011046840, Unigene23472\_Sample\_011046840, Unigene41524\_Sample\_011046840, Unigene19865\_Sample\_011046840, Unigene31278\_Sample\_011046840, Unigene38669\_Sample\_011046840, Unigene31320\_Sample\_011046840, Unigene38106\_Sample\_011046840, Unigene37234\_Sample\_011046840, Unigene43569\_Sample\_011046840, Unigene22770\_Sample\_011046840, Unigene39686\_Sample\_011046840, Unigene43214\_Sample\_011046840, Unigene39412\_Sample\_011046840, Unigene2864\_Sample\_011046840, Unigene34806\_Sample\_011046840, Unigene42211\_Sample\_011046840, Unigene29758\_Sample\_011046840, Unigene25902\_Sample\_011046840, Unigene37578\_Sample\_011046840, Unigene2180\_Sample\_011046840, Unigene41811\_Sample\_011046840, Unigene28270\_Sample\_011046840, Unigene7124\_Sample\_011046840, Unigene37716\_Sample\_011046840, Unigene39234\_Sample\_011046840, Unigene32740\_Sample\_011046840, Unigene8191\_Sample\_011046840, Unigene3608\_Sample\_011046840, Unigene41055\_Sample\_011046840, Unigene42939\_Sample\_011046840, Unigene40104\_Sample\_011046840, Unigene30051\_Sample\_011046840, Unigene42983\_Sample\_011046840, Unigene8633\_Sample\_011046840, Unigene24747\_Sample\_011046840, Unigene32240\_Sample\_011046840, Unigene37285\_Sample\_011046840, Unigene1389\_Sample\_011046840, Unigene5682\_Sample\_011046840, Unigene33166\_Sample\_011046840, Unigene32573\_Sample\_011046840, Unigene41250\_Sample\_011046840, Unigene8024\_Sample\_011046840, Unigene43573\_Sample\_011046840, Unigene38113\_Sample\_011046840, Unigene35784\_Sample\_011046840, Unigene43315\_Sample\_011046840, Unigene17678\_Sample\_011046840, Unigene35174\_Sample\_011046840, Unigene3204\_Sample\_011046840, Unigene39836\_Sample\_011046840, Unigene31899\_Sample\_011046840, Unigene19860\_Sample\_011046840, Unigene41693\_Sample\_011046840, Unigene30970\_Sample\_011046840, Unigene21108\_Sample\_011046840, Unigene42142\_Sample\_011046840, Unigene40883\_Sample\_011046840, Unigene43002\_Sample\_011046840, Unigene35126\_Sample\_011046840, Unigene43003\_Sample\_011046840, Unigene39602\_Sample\_011046840, Unigene42765\_Sample\_011046840, Unigene38429\_Sample\_011046840, Unigene38841\_Sample\_011046840, Unigene10437\_Sample\_011046840, Unigene5746\_Sample\_011046840, Unigene8072\_Sample\_011046840, Unigene26556\_Sample\_011046840, Unigene31611\_Sample\_011046840, Unigene27932\_Sample\_011046840, Unigene31669\_Sample\_011046840, Unigene31198\_Sample\_011046840, Unigene43301\_Sample\_011046840, Unigene17816\_Sample\_011046840, Unigene31457\_Sample\_011046840, Unigene28065\_Sample\_011046840, Unigene21513\_Sample\_011046840, Unigene8187\_Sample\_011046840, Unigene16413\_Sample\_011046840, Unigene33061\_Sample\_011046840, Unigene37048\_Sample\_011046840, Unigene42730\_Sample\_011046840, Unigene15916\_Sample\_011046840, Unigene34917\_Sample\_011046840, Unigene31482\_Sample\_011046840, Unigene43405\_Sample\_011046840, Unigene36223\_Sample\_011046840, Unigene30033\_Sample\_011046840, Unigene16228\_Sample\_011046840, Unigene36338\_Sample\_011046840, Unigene43408\_Sample\_011046840, Unigene40895\_Sample\_011046840, Unigene6950\_Sample\_011046840, Unigene42043\_Sample\_011046840, Unigene36018\_Sample\_011046840, Unigene20869\_Sample\_011046840, Unigene16462\_Sample\_011046840, Unigene24879\_Sample\_011046840, Unigene41957\_Sample\_011046840, Unigene41383\_Sample\_011046840, Unigene34317\_Sample\_011046840, Unigene27840\_Sample\_011046840, Unigene30256\_Sample\_011046840, Unigene18542\_Sample\_011046840, Unigene3046\_Sample\_011046840, Unigene29507\_Sample\_011046840, Unigene42098\_Sample\_011046840, Unigene37486\_Sample\_011046840, Unigene32591\_Sample\_011046840, Unigene4932\_Sample\_011046840, Unigene13413\_Sample\_011046840, Unigene29334\_Sample\_011046840, Unigene43289\_Sample\_011046840, Unigene31376\_Sample\_011046840, Unigene28459\_Sample\_011046840, Unigene34268\_Sample\_011046840, Unigene27274\_Sample\_011046840, Unigene15290\_Sample\_011046840, Unigene28153\_Sample\_011046840, Unigene30083\_Sample\_011046840, Unigene39546\_Sample\_011046840, Unigene24336\_Sample\_011046840, Unigene30720\_Sample\_011046840, Unigene23318\_Sample\_011046840, Unigene42219\_Sample\_011046840, Unigene22066\_Sample\_011046840, Unigene7813\_Sample\_011046840, Unigene38232\_Sample\_011046840, Unigene42130\_Sample\_011046840, Unigene7388\_Sample\_011046840, Unigene40082\_Sample\_011046840, Unigene13841\_Sample\_011046840, Unigene1154\_Sample\_011046840, Unigene36762\_Sample\_011046840, Unigene18637\_Sample\_011046840, Unigene23400\_Sample\_011046840, Unigene24482\_Sample\_011046840, Unigene26756\_Sample\_011046840, Unigene32130\_Sample\_011046840, Unigene33441\_Sample\_011046840, Unigene40483\_Sample\_011046840, Unigene40113\_Sample\_011046840, Unigene39900\_Sample\_011046840, Unigene18228\_Sample\_011046840, Unigene35365\_Sample\_011046840, Unigene33705\_Sample\_011046840, Unigene9985\_Sample\_011046840, Unigene6177\_Sample\_011046840, Unigene34118\_Sample\_011046840, Unigene5336\_Sample\_011046840, Unigene11684\_Sample\_011046840, Unigene36996\_Sample\_011046840, Unigene16687\_Sample\_011046840, Unigene4601\_Sample\_011046840, Unigene42166\_Sample\_011046840, Unigene5223\_Sample\_011046840, Unigene36120\_Sample\_011046840, Unigene37825\_Sample\_011046840, Unigene38826\_Sample\_011046840, Unigene36211\_Sample\_011046840, Unigene11392\_Sample\_011046840, Unigene43372\_Sample\_011046840, Unigene11686\_Sample\_011046840, Unigene34757\_Sample\_011046840, Unigene2724\_Sample\_011046840, Unigene33942\_Sample\_011046840, Unigene39734\_Sample\_011046840, Unigene39481\_Sample\_011046840, Unigene35544\_Sample\_011046840, Unigene29542\_Sample\_011046840, Unigene41433\_Sample\_011046840, Unigene34096\_Sample\_011046840, Unigene32555\_Sample\_011046840, Unigene13188\_Sample\_011046840, Unigene34205\_Sample\_011046840, Unigene6276\_Sample\_011046840, Unigene7677\_Sample\_011046840, Unigene43579\_Sample\_011046840, Unigene34048\_Sample\_011046840, Unigene2706\_Sample\_011046840, Unigene34219\_Sample\_011046840, Unigene26614\_Sample\_011046840, Unigene31898\_Sample\_011046840, Unigene28263\_Sample\_011046840, Unigene21413\_Sample\_011046840, Unigene25381\_Sample\_011046840, Unigene27385\_Sample\_011046840, Unigene38430\_Sample\_011046840, Unigene38085\_Sample\_011046840, Unigene16387\_Sample\_011046840, Unigene41917\_Sample\_011046840, Unigene33126\_Sample\_011046840, Unigene36446\_Sample\_011046840, Unigene37480\_Sample\_011046840, Unigene40479\_Sample\_011046840, Unigene38460\_Sample\_011046840, Unigene34336\_Sample\_011046840, Unigene36661\_Sample\_011046840, Unigene40495\_Sample\_011046840, Unigene19409\_Sample\_011046840, Unigene36494\_Sample\_011046840, Unigene38491\_Sample\_011046840, Unigene28160\_Sample\_011046840, Unigene43177\_Sample\_011046840, Unigene42533\_Sample\_011046840, Unigene39609\_Sample\_011046840, Unigene26402\_Sample\_011046840, Unigene11248\_Sample\_011046840, Unigene15043\_Sample\_011046840, Unigene15915\_Sample\_011046840, Unigene5876\_Sample\_011046840, Unigene36912\_Sample\_011046840, Unigene12550\_Sample\_011046840, Unigene36063\_Sample\_011046840, Unigene18251\_Sample\_011046840, Unigene43463\_Sample\_011046840, Unigene15017\_Sample\_011046840, Unigene41545\_Sample\_011046840, Unigene31980\_Sample\_011046840, Unigene28456\_Sample\_011046840, Unigene31283\_Sample\_011046840, Unigene38265\_Sample\_011046840, Unigene30230\_Sample\_011046840, Unigene9307\_Sample\_011046840, Unigene42403\_Sample\_011046840, Unigene28754\_Sample\_011046840, Unigene33408\_Sample\_011046840, Unigene39671\_Sample\_011046840, Unigene40741\_Sample\_011046840, Unigene43357\_Sample\_011046840, Unigene34984\_Sample\_011046840, Unigene3201\_Sample\_011046840, Unigene30293\_Sample\_011046840, Unigene1504\_Sample\_011046840, Unigene34015\_Sample\_011046840, Unigene32180\_Sample\_011046840, Unigene2754\_Sample\_011046840, Unigene40922\_Sample\_011046840, Unigene39941\_Sample\_011046840, Unigene5991\_Sample\_011046840, Unigene20151\_Sample\_011046840, Unigene35511\_Sample\_011046840, Unigene35637\_Sample\_011046840, Unigene16641\_Sample\_011046840, Unigene39706\_Sample\_011046840, Unigene12175\_Sample\_011046840, Unigene40576\_Sample\_011046840, Unigene34977\_Sample\_011046840, Unigene43360\_Sample\_011046840, Unigene39095\_Sample\_011046840, Unigene1722\_Sample\_011046840, Unigene38713\_Sample\_011046840, Unigene35227\_Sample\_011046840, Unigene17098\_Sample\_011046840, Unigene34431\_Sample\_011046840, Unigene16879\_Sample\_011046840, Unigene43064\_Sample\_011046840, Unigene40520\_Sample\_011046840, Unigene2418\_Sample\_011046840, Unigene20967\_Sample\_011046840, Unigene41902\_Sample\_011046840, Unigene39983\_Sample\_011046840, Unigene12669\_Sample\_011046840, Unigene34505\_Sample\_011046840, Unigene16402\_Sample\_011046840, Unigene5094\_Sample\_011046840, Unigene20050\_Sample\_011046840, Unigene2367\_Sample\_011046840, Unigene31240\_Sample\_011046840, Unigene29471\_Sample\_011046840, Unigene36915\_Sample\_011046840, Unigene1533\_Sample\_011046840, Unigene37963\_Sample\_011046840, Unigene5705\_Sample\_011046840, Unigene26417\_Sample\_011046840, Unigene37975\_Sample\_011046840, Unigene19272\_Sample\_011046840, Unigene26868\_Sample\_011046840, Unigene40061\_Sample\_011046840, Unigene40099\_Sample\_011046840, Unigene34573\_Sample\_011046840, Unigene8565\_Sample\_011046840, Unigene32901\_Sample\_011046840, Unigene33172\_Sample\_011046840, Unigene28488\_Sample\_011046840, Unigene33585\_Sample\_011046840, Unigene20014\_Sample\_011046840, Unigene31973\_Sample\_011046840, Unigene8102\_Sample\_011046840, Unigene12710\_Sample\_011046840, Unigene40116\_Sample\_011046840, Unigene22322\_Sample\_011046840, Unigene30400\_Sample\_011046840, Unigene27036\_Sample\_011046840, Unigene41523\_Sample\_011046840, Unigene6611\_Sample\_011046840, Unigene39017\_Sample\_011046840, Unigene36640\_Sample\_011046840, Unigene35183\_Sample\_011046840, Unigene21879\_Sample\_011046840, Unigene25369\_Sample\_011046840, Unigene34638\_Sample\_011046840, Unigene42866\_Sample\_011046840, Unigene32413\_Sample\_011046840, Unigene20370\_Sample\_011046840, Unigene14271\_Sample\_011046840, Unigene32814\_Sample\_011046840, Unigene42353\_Sample\_011046840, Unigene7507\_Sample\_011046840, Unigene7499\_Sample\_011046840, Unigene38409\_Sample\_011046840, Unigene27885\_Sample\_011046840, Unigene18168\_Sample\_011046840, Unigene11142\_Sample\_011046840, Unigene32307\_Sample\_011046840, Unigene38902\_Sample\_011046840, Unigene1881\_Sample\_011046840, Unigene21770\_Sample\_011046840, Unigene30430\_Sample\_011046840, Unigene41632\_Sample\_011046840, Unigene42022\_Sample\_011046840, Unigene40396\_Sample\_011046840, Unigene43096\_Sample\_011046840, Unigene27512\_Sample\_011046840, Unigene21541\_Sample\_011046840, Unigene37781\_Sample\_011046840, Unigene26597\_Sample\_011046840, Unigene31886\_Sample\_011046840, Unigene5913\_Sample\_011046840, Unigene36477\_Sample\_011046840, Unigene35140\_Sample\_011046840, Unigene40836\_Sample\_011046840, Unigene33008\_Sample\_011046840, Unigene7829\_Sample\_011046840, Unigene29606\_Sample\_011046840, Unigene38794\_Sample\_011046840, Unigene40998\_Sample\_011046840, Unigene31286\_Sample\_011046840, Unigene41281\_Sample\_011046840, Unigene27683\_Sample\_011046840, Unigene5893\_Sample\_011046840, Unigene39705\_Sample\_011046840 |
| nuclear ubiquitin ligase complex | Unigene1722\_Sample\_011046840, Unigene38902\_Sample\_011046840, Unigene10050\_Sample\_011046840, Unigene557\_Sample\_011046840, Unigene36779\_Sample\_011046840, Unigene13805\_Sample\_011046840, Unigene41125\_Sample\_011046840, Unigene41114\_Sample\_011046840, Unigene38254\_Sample\_011046840, Unigene40019\_Sample\_011046840, Unigene7174\_Sample\_011046840, Unigene27260\_Sample\_011046840, Unigene43358\_Sample\_011046840, Unigene39224\_Sample\_011046840, Unigene24936\_Sample\_011046840, Unigene35206\_Sample\_011046840, Unigene36846\_Sample\_011046840, Unigene1561\_Sample\_011046840, Unigene6608\_Sample\_011046840, Unigene39734\_Sample\_011046840, Unigene24747\_Sample\_011046840, Unigene20430\_Sample\_011046840, Unigene36437\_Sample\_011046840, Unigene8171\_Sample\_011046840, Unigene1389\_Sample\_011046840, Unigene33222\_Sample\_011046840, Unigene39889\_Sample\_011046840 |
| chromosomal part | Unigene38023\_Sample\_011046840, Unigene29269\_Sample\_011046840, Unigene39900\_Sample\_011046840, Unigene18228\_Sample\_011046840, Unigene28409\_Sample\_011046840, Unigene368\_Sample\_011046840, Unigene13117\_Sample\_011046840, Unigene40049\_Sample\_011046840, Unigene40685\_Sample\_011046840, Unigene18212\_Sample\_011046840, Unigene28265\_Sample\_011046840, Unigene25871\_Sample\_011046840, Unigene19499\_Sample\_011046840, Unigene33322\_Sample\_011046840, Unigene38540\_Sample\_011046840, Unigene33482\_Sample\_011046840, Unigene32488\_Sample\_011046840, Unigene42166\_Sample\_011046840, Unigene5867\_Sample\_011046840, Unigene39391\_Sample\_011046840, Unigene31572\_Sample\_011046840, Unigene39649\_Sample\_011046840, Unigene40868\_Sample\_011046840, Unigene27358\_Sample\_011046840, Unigene43486\_Sample\_011046840, Unigene43277\_Sample\_011046840, Unigene15570\_Sample\_011046840, Unigene6276\_Sample\_011046840, Unigene7677\_Sample\_011046840, Unigene43397\_Sample\_011046840, Unigene41263\_Sample\_011046840, Unigene33395\_Sample\_011046840, Unigene14648\_Sample\_011046840, Unigene42168\_Sample\_011046840, Unigene20982\_Sample\_011046840, Unigene29635\_Sample\_011046840, Unigene30865\_Sample\_011046840, Unigene17494\_Sample\_011046840, Unigene28451\_Sample\_011046840, Unigene9704\_Sample\_011046840, Unigene15120\_Sample\_011046840, Unigene34036\_Sample\_011046840, Unigene25063\_Sample\_011046840, Unigene20033\_Sample\_011046840, Unigene30715\_Sample\_011046840, Unigene4344\_Sample\_011046840, Unigene16618\_Sample\_011046840, Unigene25680\_Sample\_011046840, Unigene37236\_Sample\_011046840, Unigene32390\_Sample\_011046840, Unigene38690\_Sample\_011046840, Unigene14511\_Sample\_011046840, Unigene14750\_Sample\_011046840, Unigene40503\_Sample\_011046840, Unigene42626\_Sample\_011046840, Unigene18128\_Sample\_011046840, Unigene16850\_Sample\_011046840, Unigene22159\_Sample\_011046840, Unigene42944\_Sample\_011046840, Unigene42103\_Sample\_011046840, Unigene28415\_Sample\_011046840, Unigene4497\_Sample\_011046840, Unigene20549\_Sample\_011046840, Unigene5782\_Sample\_011046840, Unigene16716\_Sample\_011046840, Unigene40545\_Sample\_011046840, Unigene4652\_Sample\_011046840, Unigene9962\_Sample\_011046840, Unigene41742\_Sample\_011046840, Unigene41562\_Sample\_011046840, Unigene23252\_Sample\_011046840, Unigene14078\_Sample\_011046840, Unigene14195\_Sample\_011046840, Unigene29958\_Sample\_011046840, Unigene19982\_Sample\_011046840, Unigene42803\_Sample\_011046840, Unigene36063\_Sample\_011046840, Unigene33611\_Sample\_011046840, Unigene40638\_Sample\_011046840, Unigene6426\_Sample\_011046840, Unigene41052\_Sample\_011046840, Unigene12511\_Sample\_011046840, Unigene28581\_Sample\_011046840, Unigene15140\_Sample\_011046840, Unigene27604\_Sample\_011046840, Unigene39540\_Sample\_011046840, Unigene34752\_Sample\_011046840, Unigene40753\_Sample\_011046840, Unigene31306\_Sample\_011046840, Unigene4842\_Sample\_011046840, Unigene11967\_Sample\_011046840, Unigene32518\_Sample\_011046840, Unigene24731\_Sample\_011046840, Unigene32844\_Sample\_011046840, Unigene35895\_Sample\_011046840, Unigene10553\_Sample\_011046840, Unigene20800\_Sample\_011046840, Unigene34436\_Sample\_011046840, Unigene4723\_Sample\_011046840, Unigene37470\_Sample\_011046840, Unigene30691\_Sample\_011046840, Unigene27185\_Sample\_011046840, Unigene43414\_Sample\_011046840, Unigene38595\_Sample\_011046840, Unigene42502\_Sample\_011046840, Unigene41104\_Sample\_011046840, Unigene40424\_Sample\_011046840, Unigene36973\_Sample\_011046840, Unigene31513\_Sample\_011046840, Unigene12913\_Sample\_011046840, Unigene2626\_Sample\_011046840, Unigene5383\_Sample\_011046840, Unigene22810\_Sample\_011046840, Unigene28616\_Sample\_011046840, Unigene38121\_Sample\_011046840, Unigene37826\_Sample\_011046840, Unigene25298\_Sample\_011046840, Unigene41124\_Sample\_011046840, Unigene34703\_Sample\_011046840, Unigene25450\_Sample\_011046840, Unigene39688\_Sample\_011046840, Unigene21415\_Sample\_011046840, Unigene30760\_Sample\_011046840, Unigene25972\_Sample\_011046840, Unigene42985\_Sample\_011046840, Unigene39836\_Sample\_011046840, Unigene39948\_Sample\_011046840, Unigene11613\_Sample\_011046840, Unigene37283\_Sample\_011046840, Unigene5720\_Sample\_011046840, Unigene31822\_Sample\_011046840, Unigene42659\_Sample\_011046840, Unigene29471\_Sample\_011046840, Unigene30185\_Sample\_011046840, Unigene27677\_Sample\_011046840, Unigene41412\_Sample\_011046840, Unigene36915\_Sample\_011046840, Unigene20485\_Sample\_011046840, Unigene39602\_Sample\_011046840, Unigene29411\_Sample\_011046840, Unigene2740\_Sample\_011046840, Unigene43525\_Sample\_011046840, Unigene27478\_Sample\_011046840, Unigene40204\_Sample\_011046840, Unigene41709\_Sample\_011046840, Unigene27932\_Sample\_011046840, Unigene29253\_Sample\_011046840, Unigene29661\_Sample\_011046840, Unigene25413\_Sample\_011046840, Unigene28259\_Sample\_011046840, Unigene38753\_Sample\_011046840, Unigene34046\_Sample\_011046840, Unigene28397\_Sample\_011046840, Unigene26453\_Sample\_011046840, Unigene4498\_Sample\_011046840, Unigene43273\_Sample\_011046840, Unigene41000\_Sample\_011046840, Unigene14154\_Sample\_011046840, Unigene34459\_Sample\_011046840, Unigene29802\_Sample\_011046840, Unigene21949\_Sample\_011046840, Unigene41899\_Sample\_011046840, Unigene36338\_Sample\_011046840, Unigene38904\_Sample\_011046840, Unigene33129\_Sample\_011046840, Unigene5710\_Sample\_011046840, Unigene5197\_Sample\_011046840, Unigene42866\_Sample\_011046840, Unigene22186\_Sample\_011046840, Unigene30343\_Sample\_011046840, Unigene41512\_Sample\_011046840, Unigene43165\_Sample\_011046840, Unigene14037\_Sample\_011046840, Unigene43450\_Sample\_011046840, Unigene39446\_Sample\_011046840, Unigene22688\_Sample\_011046840, Unigene41383\_Sample\_011046840, Unigene32099\_Sample\_011046840, Unigene26022\_Sample\_011046840, Unigene40004\_Sample\_011046840, Unigene35170\_Sample\_011046840, Unigene41997\_Sample\_011046840, Unigene28153\_Sample\_011046840, Unigene22606\_Sample\_011046840, Unigene21063\_Sample\_011046840, Unigene15681\_Sample\_011046840, Unigene30599\_Sample\_011046840, Unigene15608\_Sample\_011046840, Unigene38453\_Sample\_011046840, Unigene8155\_Sample\_011046840, Unigene32050\_Sample\_011046840, Unigene24019\_Sample\_011046840, Unigene30813\_Sample\_011046840, Unigene41337\_Sample\_011046840, Unigene33527\_Sample\_011046840, Unigene40998\_Sample\_011046840, Unigene34028\_Sample\_011046840, Unigene39591\_Sample\_011046840 |
| ubiquitin ligase complex | Unigene1722\_Sample\_011046840, Unigene13786\_Sample\_011046840, Unigene10050\_Sample\_011046840, Unigene39121\_Sample\_011046840, Unigene39623\_Sample\_011046840, Unigene42927\_Sample\_011046840, Unigene26628\_Sample\_011046840, Unigene27305\_Sample\_011046840, Unigene34267\_Sample\_011046840, Unigene36779\_Sample\_011046840, Unigene13805\_Sample\_011046840, Unigene29240\_Sample\_011046840, Unigene18354\_Sample\_011046840, Unigene29658\_Sample\_011046840, Unigene43358\_Sample\_011046840, Unigene24936\_Sample\_011046840, Unigene35206\_Sample\_011046840, Unigene1561\_Sample\_011046840, Unigene6608\_Sample\_011046840, Unigene39734\_Sample\_011046840, Unigene20430\_Sample\_011046840, Unigene36800\_Sample\_011046840, Unigene26069\_Sample\_011046840, Unigene31354\_Sample\_011046840, Unigene38912\_Sample\_011046840, Unigene32620\_Sample\_011046840, Unigene5037\_Sample\_011046840, Unigene34513\_Sample\_011046840, Unigene11174\_Sample\_011046840, Unigene7174\_Sample\_011046840, Unigene14598\_Sample\_011046840, Unigene36846\_Sample\_011046840, Unigene41252\_Sample\_011046840, Unigene42629\_Sample\_011046840, Unigene16406\_Sample\_011046840, Unigene41139\_Sample\_011046840, Unigene36437\_Sample\_011046840, Unigene37207\_Sample\_011046840, Unigene8171\_Sample\_011046840, Unigene18691\_Sample\_011046840, Unigene41125\_Sample\_011046840, Unigene42169\_Sample\_011046840, Unigene41114\_Sample\_011046840, Unigene38254\_Sample\_011046840, Unigene40019\_Sample\_011046840, Unigene27260\_Sample\_011046840, Unigene29541\_Sample\_011046840, Unigene34731\_Sample\_011046840, Unigene20330\_Sample\_011046840, Unigene37890\_Sample\_011046840, Unigene33222\_Sample\_011046840, Unigene30694\_Sample\_011046840, Unigene38902\_Sample\_011046840, Unigene557\_Sample\_011046840, Unigene43371\_Sample\_011046840, Unigene21541\_Sample\_011046840, Unigene41373\_Sample\_011046840, Unigene19732\_Sample\_011046840, Unigene39224\_Sample\_011046840, Unigene28840\_Sample\_011046840, Unigene42173\_Sample\_011046840, Unigene25325\_Sample\_011046840, Unigene10229\_Sample\_011046840, Unigene26863\_Sample\_011046840, Unigene28531\_Sample\_011046840, Unigene37474\_Sample\_011046840, Unigene11650\_Sample\_011046840, Unigene24747\_Sample\_011046840, Unigene5009\_Sample\_011046840, Unigene1389\_Sample\_011046840, Unigene11225\_Sample\_011046840, Unigene39889\_Sample\_011046840 |
[truncated: 2,177,970 more chars]
