# Supplementary material for: Identification of Dirofilaria immitis miRNA using illumina deep sequencing
Source: Vet Res. 2013 Jan 18;44(1):3. doi: 10.1186/1297-9716-44-3 (PMC3598945; doi:10.1186/1297-9716-44-3)
Supplement: Additional file 7 — The GO annotations on putative target genes (Molecular function). 10 541 target genes were assigned to 652 Go-terms which belong to “Molecular function” ontology. “Gene Ontology term” means GO terms with P-value as good or better than 1. “Cluster frequency” represents number and frequency of target genes related to this term. “Genome frequency of use” means number and frequency of coding genes related to this term. [file 1297-9716-44-3-S7.zip › index.htm/Additional file 7. Molecular function.html]

Terms for Dirofilaria\_immites\_F


## Terms for Dirofilaria\_immites\_F

---


### Result Table

|  |
| --- |
| **Terms from the Function Ontology with p-value as good or better than 1** |

| Gene Ontology term | Cluster frequency | Genome frequency of use | Corrected P-value |
| --- | --- | --- | --- |
| transferase activity, transferring hexosyl groups | 123 out of 10541 genes, 1.2% | 133 out of 12397 genes, 1.1% | 1 |
| phosphoprotein phosphatase activity | 169 out of 10541 genes, 1.6% | 188 out of 12397 genes, 1.5% | 1 |
| active transmembrane transporter activity | 236 out of 10541 genes, 2.2% | 265 out of 12397 genes, 2.1% | 1 |
| phospholipid binding | 66 out of 10541 genes, 0.6% | 71 out of 12397 genes, 0.6% | 1 |
| oxidoreductase activity, acting on paired donors, with incorporation or reduction of molecular oxygen, 2-oxoglutarate as one donor, and incorporation of one atom each of oxygen into both donors | 31 out of 10541 genes, 0.3% | 32 out of 12397 genes, 0.3% | 1 |
| amine transmembrane transporter activity | 30 out of 10541 genes, 0.3% | 31 out of 12397 genes, 0.3% | 1 |
| vitamin binding | 39 out of 10541 genes, 0.4% | 41 out of 12397 genes, 0.3% | 1 |
| nucleoside binding | 1761 out of 10541 genes, 16.7% | 2041 out of 12397 genes, 16.5% | 1 |
| transmembrane transporter activity | 642 out of 10541 genes, 6.1% | 736 out of 12397 genes, 5.9% | 1 |
| phosphoinositide binding | 29 out of 10541 genes, 0.3% | 30 out of 12397 genes, 0.2% | 1 |
| phosphoric ester hydrolase activity | 333 out of 10541 genes, 3.2% | 378 out of 12397 genes, 3.0% | 1 |
| purine nucleoside binding | 1746 out of 10541 genes, 16.6% | 2025 out of 12397 genes, 16.3% | 1 |
| receptor activity | 253 out of 10541 genes, 2.4% | 286 out of 12397 genes, 2.3% | 1 |
| enzyme binding | 207 out of 10541 genes, 2.0% | 233 out of 12397 genes, 1.9% | 1 |
| RNA helicase activity | 27 out of 10541 genes, 0.3% | 28 out of 12397 genes, 0.2% | 1 |
| transferase activity, transferring one-carbon groups | 185 out of 10541 genes, 1.8% | 208 out of 12397 genes, 1.7% | 1 |
| procollagen-proline dioxygenase activity | 17 out of 10541 genes, 0.2% | 17 out of 12397 genes, 0.1% | 1 |
| peptidyl-proline dioxygenase activity | 17 out of 10541 genes, 0.2% | 17 out of 12397 genes, 0.1% | 1 |
| adenyl nucleotide binding | 1729 out of 10541 genes, 16.4% | 2007 out of 12397 genes, 16.2% | 1 |
| adenyl ribonucleotide binding | 1645 out of 10541 genes, 15.6% | 1909 out of 12397 genes, 15.4% | 1 |
| transferase activity | 1964 out of 10541 genes, 18.6% | 2283 out of 12397 genes, 18.4% | 1 |
| coenzyme binding | 16 out of 10541 genes, 0.2% | 16 out of 12397 genes, 0.1% | 1 |
| G-protein coupled receptor activity | 42 out of 10541 genes, 0.4% | 45 out of 12397 genes, 0.4% | 1 |
| transferase activity, transferring nitrogenous groups | 25 out of 10541 genes, 0.2% | 26 out of 12397 genes, 0.2% | 1 |
| ligase activity, forming carbon-oxygen bonds | 127 out of 10541 genes, 1.2% | 142 out of 12397 genes, 1.1% | 1 |
| ligase activity, forming aminoacyl-tRNA and related compounds | 127 out of 10541 genes, 1.2% | 142 out of 12397 genes, 1.1% | 1 |
| phospholipid transporter activity | 15 out of 10541 genes, 0.1% | 15 out of 12397 genes, 0.1% | 1 |
| DNA binding | 355 out of 10541 genes, 3.4% | 406 out of 12397 genes, 3.3% | 1 |
| primary active transmembrane transporter activity | 131 out of 10541 genes, 1.2% | 147 out of 12397 genes, 1.2% | 1 |
| P-P-bond-hydrolysis-driven transmembrane transporter activity | 131 out of 10541 genes, 1.2% | 147 out of 12397 genes, 1.2% | 1 |
| phospholipase activity | 40 out of 10541 genes, 0.4% | 43 out of 12397 genes, 0.3% | 1 |
| lipase activity | 40 out of 10541 genes, 0.4% | 43 out of 12397 genes, 0.3% | 1 |
| neurotransmitter transporter activity | 14 out of 10541 genes, 0.1% | 14 out of 12397 genes, 0.1% | 1 |
| binding | 7989 out of 10541 genes, 75.8% | 9370 out of 12397 genes, 75.6% | 1 |
| UDP-glycosyltransferase activity | 68 out of 10541 genes, 0.6% | 75 out of 12397 genes, 0.6% | 1 |
| transmembrane receptor activity | 173 out of 10541 genes, 1.6% | 196 out of 12397 genes, 1.6% | 1 |
| metal cluster binding | 67 out of 10541 genes, 0.6% | 74 out of 12397 genes, 0.6% | 1 |
| cell adhesion molecule binding | 13 out of 10541 genes, 0.1% | 13 out of 12397 genes, 0.1% | 1 |
| structure-specific DNA binding | 59 out of 10541 genes, 0.6% | 65 out of 12397 genes, 0.5% | 1 |
| phosphatase activity | 263 out of 10541 genes, 2.5% | 301 out of 12397 genes, 2.4% | 1 |
| signal transducer activity | 452 out of 10541 genes, 4.3% | 521 out of 12397 genes, 4.2% | 1 |
| sequence-specific DNA binding RNA polymerase II transcription factor activity | 12 out of 10541 genes, 0.1% | 12 out of 12397 genes, 0.1% | 1 |
| G-protein-coupled receptor binding | 12 out of 10541 genes, 0.1% | 12 out of 12397 genes, 0.1% | 1 |
| sequence-specific DNA binding transcription factor activity | 12 out of 10541 genes, 0.1% | 12 out of 12397 genes, 0.1% | 1 |
| calmodulin-dependent protein kinase activity | 12 out of 10541 genes, 0.1% | 12 out of 12397 genes, 0.1% | 1 |
| myosin binding | 12 out of 10541 genes, 0.1% | 12 out of 12397 genes, 0.1% | 1 |
| transporter activity | 755 out of 10541 genes, 7.2% | 875 out of 12397 genes, 7.1% | 1 |
| transaminase activity | 20 out of 10541 genes, 0.2% | 21 out of 12397 genes, 0.2% | 1 |
| ligand-dependent nuclear receptor activity | 56 out of 10541 genes, 0.5% | 62 out of 12397 genes, 0.5% | 1 |
| transferase activity, transferring glycosyl groups | 190 out of 10541 genes, 1.8% | 217 out of 12397 genes, 1.8% | 1 |
| aminoacyl-tRNA ligase activity | 114 out of 10541 genes, 1.1% | 129 out of 12397 genes, 1.0% | 1 |
| methyltransferase activity | 120 out of 10541 genes, 1.1% | 136 out of 12397 genes, 1.1% | 1 |
| ribonucleotide binding | 1933 out of 10541 genes, 18.3% | 2256 out of 12397 genes, 18.2% | 1 |
| purine ribonucleotide binding | 1933 out of 10541 genes, 18.3% | 2256 out of 12397 genes, 18.2% | 1 |
| carbohydrate binding | 81 out of 10541 genes, 0.8% | 91 out of 12397 genes, 0.7% | 1 |
| purine nucleotide binding | 2017 out of 10541 genes, 19.1% | 2355 out of 12397 genes, 19.0% | 1 |
| oligosaccharyl transferase activity | 10 out of 10541 genes, 0.1% | 10 out of 12397 genes, 0.1% | 1 |
| inorganic anion transmembrane transporter activity | 10 out of 10541 genes, 0.1% | 10 out of 12397 genes, 0.1% | 1 |
| sulfurtransferase activity | 10 out of 10541 genes, 0.1% | 10 out of 12397 genes, 0.1% | 1 |
| disulfide oxidoreductase activity | 18 out of 10541 genes, 0.2% | 19 out of 12397 genes, 0.2% | 1 |
| hydrolase activity, acting on acid anhydrides, catalyzing transmembrane movement of substances | 98 out of 10541 genes, 0.9% | 111 out of 12397 genes, 0.9% | 1 |
| kinase activity | 852 out of 10541 genes, 8.1% | 991 out of 12397 genes, 8.0% | 1 |
| transferase activity, transferring phosphorus-containing groups | 1179 out of 10541 genes, 11.2% | 1374 out of 12397 genes, 11.1% | 1 |
| molecular transducer activity | 555 out of 10541 genes, 5.3% | 644 out of 12397 genes, 5.2% | 1 |
| ATPase activity, coupled | 352 out of 10541 genes, 3.3% | 407 out of 12397 genes, 3.3% | 1 |
| substrate-specific transmembrane transporter activity | 547 out of 10541 genes, 5.2% | 635 out of 12397 genes, 5.1% | 1 |
| transcription cofactor activity | 51 out of 10541 genes, 0.5% | 57 out of 12397 genes, 0.5% | 1 |
| oxidoreductase activity, acting on single donors with incorporation of molecular oxygen | 24 out of 10541 genes, 0.2% | 26 out of 12397 genes, 0.2% | 1 |
| hydrolase activity, acting on carbon-nitrogen (but not peptide) bonds, in linear amides | 24 out of 10541 genes, 0.2% | 26 out of 12397 genes, 0.2% | 1 |
| hexosaminidase activity | 9 out of 10541 genes, 0.1% | 9 out of 12397 genes, 0.1% | 1 |
| deacetylase activity | 9 out of 10541 genes, 0.1% | 9 out of 12397 genes, 0.1% | 1 |
| nucleic acid binding | 1553 out of 10541 genes, 14.7% | 1814 out of 12397 genes, 14.6% | 1 |
| channel activity | 248 out of 10541 genes, 2.4% | 286 out of 12397 genes, 2.3% | 1 |
| passive transmembrane transporter activity | 248 out of 10541 genes, 2.4% | 286 out of 12397 genes, 2.3% | 1 |
| helicase activity | 120 out of 10541 genes, 1.1% | 137 out of 12397 genes, 1.1% | 1 |
| ATPase activity, coupled to movement of substances | 95 out of 10541 genes, 0.9% | 108 out of 12397 genes, 0.9% | 1 |
| phosphotransferase activity, alcohol group as acceptor | 654 out of 10541 genes, 6.2% | 761 out of 12397 genes, 6.1% | 1 |
| voltage-gated potassium channel activity | 16 out of 10541 genes, 0.2% | 17 out of 12397 genes, 0.1% | 1 |
| cation:cation antiporter activity | 16 out of 10541 genes, 0.2% | 17 out of 12397 genes, 0.1% | 1 |
| glucosyltransferase activity | 16 out of 10541 genes, 0.2% | 17 out of 12397 genes, 0.1% | 1 |
| adenylyltransferase activity | 16 out of 10541 genes, 0.2% | 17 out of 12397 genes, 0.1% | 1 |
| protein domain specific binding | 56 out of 10541 genes, 0.5% | 63 out of 12397 genes, 0.5% | 1 |
| phospholipase C activity | 23 out of 10541 genes, 0.2% | 25 out of 12397 genes, 0.2% | 1 |
| ATPase activity | 364 out of 10541 genes, 3.5% | 422 out of 12397 genes, 3.4% | 1 |
| kinase regulator activity | 36 out of 10541 genes, 0.3% | 40 out of 12397 genes, 0.3% | 1 |
| protein binding | 1434 out of 10541 genes, 13.6% | 1676 out of 12397 genes, 13.5% | 1 |
| 3',5'-cyclic-nucleotide phosphodiesterase activity | 8 out of 10541 genes, 0.1% | 8 out of 12397 genes, 0.1% | 1 |
| isocitrate dehydrogenase activity | 8 out of 10541 genes, 0.1% | 8 out of 12397 genes, 0.1% | 1 |
| glutamate receptor activity | 8 out of 10541 genes, 0.1% | 8 out of 12397 genes, 0.1% | 1 |
| oxidoreductase activity, acting on NADH or NADPH, heme protein as acceptor | 8 out of 10541 genes, 0.1% | 8 out of 12397 genes, 0.1% | 1 |
| hydrolase activity, hydrolyzing N-glycosyl compounds | 8 out of 10541 genes, 0.1% | 8 out of 12397 genes, 0.1% | 1 |
| protein deacetylase activity | 8 out of 10541 genes, 0.1% | 8 out of 12397 genes, 0.1% | 1 |
| NAD or NADH binding | 8 out of 10541 genes, 0.1% | 8 out of 12397 genes, 0.1% | 1 |
| microtubule motor activity | 29 out of 10541 genes, 0.3% | 32 out of 12397 genes, 0.3% | 1 |
| hydrolase activity, acting on acid anhydrides | 968 out of 10541 genes, 9.2% | 1130 out of 12397 genes, 9.1% | 1 |
| carbohydrate kinase activity | 15 out of 10541 genes, 0.1% | 16 out of 12397 genes, 0.1% | 1 |
| cation channel activity | 122 out of 10541 genes, 1.2% | 140 out of 12397 genes, 1.1% | 1 |
| nucleotide binding | 2251 out of 10541 genes, 21.4% | 2636 out of 12397 genes, 21.3% | 1 |
| potassium channel activity | 54 out of 10541 genes, 0.5% | 61 out of 12397 genes, 0.5% | 1 |
| protein kinase activity | 606 out of 10541 genes, 5.7% | 706 out of 12397 genes, 5.7% | 1 |
| catalytic activity | 5517 out of 10541 genes, 52.3% | 6475 out of 12397 genes, 52.2% | 1 |
| ATPase activity, coupled to transmembrane movement of substances | 91 out of 10541 genes, 0.9% | 104 out of 12397 genes, 0.8% | 1 |
| oxidoreductase activity, acting on paired donors, with incorporation or reduction of molecular oxygen | 60 out of 10541 genes, 0.6% | 68 out of 12397 genes, 0.5% | 1 |
| nucleoside-triphosphatase activity | 884 out of 10541 genes, 8.4% | 1032 out of 12397 genes, 8.3% | 1 |
| extracellular ligand-gated ion channel activity | 28 out of 10541 genes, 0.3% | 31 out of 12397 genes, 0.3% | 1 |
| oxidoreductase activity, acting on the aldehyde or oxo group of donors | 28 out of 10541 genes, 0.3% | 31 out of 12397 genes, 0.3% | 1 |
| nucleic acid binding transcription factor activity | 276 out of 10541 genes, 2.6% | 320 out of 12397 genes, 2.6% | 1 |
| ion channel activity | 240 out of 10541 genes, 2.3% | 278 out of 12397 genes, 2.2% | 1 |
| substrate-specific channel activity | 240 out of 10541 genes, 2.3% | 278 out of 12397 genes, 2.2% | 1 |
| pyrophosphatase activity | 962 out of 10541 genes, 9.1% | 1124 out of 12397 genes, 9.1% | 1 |
| hydrolase activity, acting on acid anhydrides, in phosphorus-containing anhydrides | 962 out of 10541 genes, 9.1% | 1124 out of 12397 genes, 9.1% | 1 |
| transferase activity, transferring sulfur-containing groups | 21 out of 10541 genes, 0.2% | 23 out of 12397 genes, 0.2% | 1 |
| carbon-oxygen lyase activity | 40 out of 10541 genes, 0.4% | 45 out of 12397 genes, 0.4% | 1 |
| small GTPase regulator activity | 185 out of 10541 genes, 1.8% | 214 out of 12397 genes, 1.7% | 1 |
| kinase binding | 83 out of 10541 genes, 0.8% | 95 out of 12397 genes, 0.8% | 1 |
| deoxyribonuclease activity | 14 out of 10541 genes, 0.1% | 15 out of 12397 genes, 0.1% | 1 |
| calcium activated cation channel activity | 14 out of 10541 genes, 0.1% | 15 out of 12397 genes, 0.1% | 1 |
| phosphoric diester hydrolase activity | 46 out of 10541 genes, 0.4% | 52 out of 12397 genes, 0.4% | 1 |
| iron-sulfur cluster binding | 46 out of 10541 genes, 0.4% | 52 out of 12397 genes, 0.4% | 1 |
| receptor binding | 101 out of 10541 genes, 1.0% | 116 out of 12397 genes, 0.9% | 1 |
| pyruvate dehydrogenase activity | 7 out of 10541 genes, 0.1% | 7 out of 12397 genes, 0.1% | 1 |
| sulfate adenylyltransferase activity | 7 out of 10541 genes, 0.1% | 7 out of 12397 genes, 0.1% | 1 |
| transmembrane receptor protein serine/threonine kinase signaling protein activity | 7 out of 10541 genes, 0.1% | 7 out of 12397 genes, 0.1% | 1 |
| transforming growth factor beta receptor, cytoplasmic mediator activity | 7 out of 10541 genes, 0.1% | 7 out of 12397 genes, 0.1% | 1 |
| sodium channel activity | 7 out of 10541 genes, 0.1% | 7 out of 12397 genes, 0.1% | 1 |
| organic acid:sodium symporter activity | 7 out of 10541 genes, 0.1% | 7 out of 12397 genes, 0.1% | 1 |
| calcium:cation antiporter activity | 7 out of 10541 genes, 0.1% | 7 out of 12397 genes, 0.1% | 1 |
| DNA N-glycosylase activity | 7 out of 10541 genes, 0.1% | 7 out of 12397 genes, 0.1% | 1 |
| nucleoside kinase activity | 7 out of 10541 genes, 0.1% | 7 out of 12397 genes, 0.1% | 1 |
| steroid hormone receptor binding | 7 out of 10541 genes, 0.1% | 7 out of 12397 genes, 0.1% | 1 |
| SMAD binding | 7 out of 10541 genes, 0.1% | 7 out of 12397 genes, 0.1% | 1 |
| inositol trisphosphate kinase activity | 7 out of 10541 genes, 0.1% | 7 out of 12397 genes, 0.1% | 1 |
| guanylyltransferase activity | 7 out of 10541 genes, 0.1% | 7 out of 12397 genes, 0.1% | 1 |
| G-protein coupled amine receptor activity | 20 out of 10541 genes, 0.2% | 22 out of 12397 genes, 0.2% | 1 |
| metalloexopeptidase activity | 20 out of 10541 genes, 0.2% | 22 out of 12397 genes, 0.2% | 1 |
| ion transmembrane transporter activity | 480 out of 10541 genes, 4.6% | 560 out of 12397 genes, 4.5% | 1 |
| cis-trans isomerase activity | 32 out of 10541 genes, 0.3% | 36 out of 12397 genes, 0.3% | 1 |
| protein serine/threonine kinase activity | 140 out of 10541 genes, 1.3% | 162 out of 12397 genes, 1.3% | 1 |
| small conjugating protein binding | 13 out of 10541 genes, 0.1% | 14 out of 12397 genes, 0.1% | 1 |
| UDP-glucosyltransferase activity | 13 out of 10541 genes, 0.1% | 14 out of 12397 genes, 0.1% | 1 |
| protein kinase binding | 38 out of 10541 genes, 0.4% | 43 out of 12397 genes, 0.3% | 1 |
| cation-transporting ATPase activity | 44 out of 10541 genes, 0.4% | 50 out of 12397 genes, 0.4% | 1 |
| ribonucleoprotein binding | 19 out of 10541 genes, 0.2% | 21 out of 12397 genes, 0.2% | 1 |
| glycoprotein binding | 6 out of 10541 genes, 0.1% | 6 out of 12397 genes, 0.0% | 1 |
| glycogen debranching enzyme activity | 6 out of 10541 genes, 0.1% | 6 out of 12397 genes, 0.0% | 1 |
| histone deacetylase activity | 6 out of 10541 genes, 0.1% | 6 out of 12397 genes, 0.0% | 1 |
| acetylgalactosaminyltransferase activity | 6 out of 10541 genes, 0.1% | 6 out of 12397 genes, 0.0% | 1 |
| galactosyltransferase activity | 6 out of 10541 genes, 0.1% | 6 out of 12397 genes, 0.0% | 1 |
| peptide transporter activity | 6 out of 10541 genes, 0.1% | 6 out of 12397 genes, 0.0% | 1 |
| oxidoreductase activity, acting on the CH-CH group of donors, oxygen as acceptor | 6 out of 10541 genes, 0.1% | 6 out of 12397 genes, 0.0% | 1 |
| phosphotransferase activity, for other substituted phosphate groups | 6 out of 10541 genes, 0.1% | 6 out of 12397 genes, 0.0% | 1 |
| transmitter-gated ion channel activity | 6 out of 10541 genes, 0.1% | 6 out of 12397 genes, 0.0% | 1 |
| transmitter-gated channel activity | 6 out of 10541 genes, 0.1% | 6 out of 12397 genes, 0.0% | 1 |
| alkali metal ion binding | 6 out of 10541 genes, 0.1% | 6 out of 12397 genes, 0.0% | 1 |
| histone binding | 6 out of 10541 genes, 0.1% | 6 out of 12397 genes, 0.0% | 1 |
| secondary active transmembrane transporter activity | 79 out of 10541 genes, 0.7% | 91 out of 12397 genes, 0.7% | 1 |
| acetylglucosaminyltransferase activity | 31 out of 10541 genes, 0.3% | 35 out of 12397 genes, 0.3% | 1 |
| substrate-specific transporter activity | 659 out of 10541 genes, 6.3% | 771 out of 12397 genes, 6.2% | 1 |
| transcription regulator activity | 237 out of 10541 genes, 2.2% | 276 out of 12397 genes, 2.2% | 1 |
| N-methyltransferase activity | 43 out of 10541 genes, 0.4% | 49 out of 12397 genes, 0.4% | 1 |
| S-adenosylmethionine-dependent methyltransferase activity | 49 out of 10541 genes, 0.5% | 56 out of 12397 genes, 0.5% | 1 |
| peptide receptor activity | 12 out of 10541 genes, 0.1% | 13 out of 12397 genes, 0.1% | 1 |
| DNA ligase activity | 12 out of 10541 genes, 0.1% | 13 out of 12397 genes, 0.1% | 1 |
| ligase activity, forming phosphoric ester bonds | 12 out of 10541 genes, 0.1% | 13 out of 12397 genes, 0.1% | 1 |
| small conjugating protein ligase activity | 113 out of 10541 genes, 1.1% | 131 out of 12397 genes, 1.1% | 1 |
| protein tyrosine kinase activity | 54 out of 10541 genes, 0.5% | 62 out of 12397 genes, 0.5% | 1 |
| sugar binding | 24 out of 10541 genes, 0.2% | 27 out of 12397 genes, 0.2% | 1 |
| lysine N-methyltransferase activity | 30 out of 10541 genes, 0.3% | 34 out of 12397 genes, 0.3% | 1 |
| protein-lysine N-methyltransferase activity | 30 out of 10541 genes, 0.3% | 34 out of 12397 genes, 0.3% | 1 |
| enzyme regulator activity | 390 out of 10541 genes, 3.7% | 456 out of 12397 genes, 3.7% | 1 |
| Ras guanyl-nucleotide exchange factor activity | 83 out of 10541 genes, 0.8% | 96 out of 12397 genes, 0.8% | 1 |
| exopeptidase activity | 71 out of 10541 genes, 0.7% | 82 out of 12397 genes, 0.7% | 1 |
| ATPase activity, coupled to transmembrane movement of ions | 82 out of 10541 genes, 0.8% | 95 out of 12397 genes, 0.8% | 1 |
| protein methyltransferase activity | 41 out of 10541 genes, 0.4% | 47 out of 12397 genes, 0.4% | 1 |
| enzyme inhibitor activity | 35 out of 10541 genes, 0.3% | 40 out of 12397 genes, 0.3% | 1 |
| lipid transporter activity | 35 out of 10541 genes, 0.3% | 40 out of 12397 genes, 0.3% | 1 |
| small GTPase binding | 29 out of 10541 genes, 0.3% | 33 out of 12397 genes, 0.3% | 1 |
| GTPase binding | 29 out of 10541 genes, 0.3% | 33 out of 12397 genes, 0.3% | 1 |
| sequence-specific DNA binding | 23 out of 10541 genes, 0.2% | 26 out of 12397 genes, 0.2% | 1 |
| solute:cation antiporter activity | 17 out of 10541 genes, 0.2% | 19 out of 12397 genes, 0.2% | 1 |
| voltage-gated cation channel activity | 17 out of 10541 genes, 0.2% | 19 out of 12397 genes, 0.2% | 1 |
| oxidoreductase activity, acting on the CH-CH group of donors | 58 out of 10541 genes, 0.6% | 67 out of 12397 genes, 0.5% | 1 |
| cytokine receptor binding | 5 out of 10541 genes, 0.0% | 5 out of 12397 genes, 0.0% | 1 |
| RNA guanylyltransferase activity | 5 out of 10541 genes, 0.0% | 5 out of 12397 genes, 0.0% | 1 |
| glyceraldehyde-3-phosphate dehydrogenase activity | 5 out of 10541 genes, 0.0% | 5 out of 12397 genes, 0.0% | 1 |
| oligopeptide transporter activity | 5 out of 10541 genes, 0.0% | 5 out of 12397 genes, 0.0% | 1 |
| acylglycerol O-acyltransferase activity | 5 out of 10541 genes, 0.0% | 5 out of 12397 genes, 0.0% | 1 |
| C-acetyltransferase activity | 5 out of 10541 genes, 0.0% | 5 out of 12397 genes, 0.0% | 1 |
| exonuclease activity, active with either ribo- or deoxyribonucleic acids and producing 5'-phosphomonoesters | 5 out of 10541 genes, 0.0% | 5 out of 12397 genes, 0.0% | 1 |
| nucleoside-diphosphatase activity | 5 out of 10541 genes, 0.0% | 5 out of 12397 genes, 0.0% | 1 |
| wide pore channel activity | 5 out of 10541 genes, 0.0% | 5 out of 12397 genes, 0.0% | 1 |
| UDP-galactosyltransferase activity | 5 out of 10541 genes, 0.0% | 5 out of 12397 genes, 0.0% | 1 |
| alcohol binding | 5 out of 10541 genes, 0.0% | 5 out of 12397 genes, 0.0% | 1 |
| proteoglycan binding | 5 out of 10541 genes, 0.0% | 5 out of 12397 genes, 0.0% | 1 |
| vitamin transporter activity | 5 out of 10541 genes, 0.0% | 5 out of 12397 genes, 0.0% | 1 |
| mannosyltransferase activity | 11 out of 10541 genes, 0.1% | 12 out of 12397 genes, 0.1% | 1 |
| transmembrane receptor protein tyrosine kinase activity | 11 out of 10541 genes, 0.1% | 12 out of 12397 genes, 0.1% | 1 |
| signal sequence binding | 11 out of 10541 genes, 0.1% | 12 out of 12397 genes, 0.1% | 1 |
| oxidoreductase activity, acting on the aldehyde or oxo group of donors, NAD or NADP as acceptor | 11 out of 10541 genes, 0.1% | 12 out of 12397 genes, 0.1% | 1 |
| phosphatase binding | 11 out of 10541 genes, 0.1% | 12 out of 12397 genes, 0.1% | 1 |
| hydrolase activity, hydrolyzing O-glycosyl compounds | 92 out of 10541 genes, 0.9% | 107 out of 12397 genes, 0.9% | 1 |
| antiporter activity | 28 out of 10541 genes, 0.3% | 32 out of 12397 genes, 0.3% | 1 |
| solute:solute antiporter activity | 28 out of 10541 genes, 0.3% | 32 out of 12397 genes, 0.3% | 1 |
| translation factor activity, nucleic acid binding | 177 out of 10541 genes, 1.7% | 207 out of 12397 genes, 1.7% | 1 |
| excitatory extracellular ligand-gated ion channel activity | 22 out of 10541 genes, 0.2% | 25 out of 12397 genes, 0.2% | 1 |
| heme-copper terminal oxidase activity | 22 out of 10541 genes, 0.2% | 25 out of 12397 genes, 0.2% | 1 |
| polysaccharide binding | 22 out of 10541 genes, 0.2% | 25 out of 12397 genes, 0.2% | 1 |
| cofactor binding | 125 out of 10541 genes, 1.2% | 146 out of 12397 genes, 1.2% | 1 |
| hydrolase activity, acting on carbon-nitrogen (but not peptide) bonds | 39 out of 10541 genes, 0.4% | 45 out of 12397 genes, 0.4% | 1 |
| organic acid transmembrane transporter activity | 16 out of 10541 genes, 0.2% | 18 out of 12397 genes, 0.1% | 1 |
| RNA methyltransferase activity | 16 out of 10541 genes, 0.2% | 18 out of 12397 genes, 0.1% | 1 |
| transmembrane receptor protein kinase activity | 16 out of 10541 genes, 0.2% | 18 out of 12397 genes, 0.1% | 1 |
| transferase activity, transferring pentosyl groups | 33 out of 10541 genes, 0.3% | 38 out of 12397 genes, 0.3% | 1 |
| peptidase activity, acting on L-amino acid peptides | 403 out of 10541 genes, 3.8% | 473 out of 12397 genes, 3.8% | 1 |
| protein tyrosine phosphatase activity | 10 out of 10541 genes, 0.1% | 11 out of 12397 genes, 0.1% | 1 |
| intracellular ligand-gated calcium channel activity | 10 out of 10541 genes, 0.1% | 11 out of 12397 genes, 0.1% | 1 |
| glycosaminoglycan binding | 10 out of 10541 genes, 0.1% | 11 out of 12397 genes, 0.1% | 1 |
| phosphofructokinase activity | 10 out of 10541 genes, 0.1% | 11 out of 12397 genes, 0.1% | 1 |
| peptide receptor activity, G-protein coupled | 10 out of 10541 genes, 0.1% | 11 out of 12397 genes, 0.1% | 1 |
| calcium-release channel activity | 10 out of 10541 genes, 0.1% | 11 out of 12397 genes, 0.1% | 1 |
| nucleobase, nucleoside, nucleotide and nucleic acid transmembrane transporter activity | 10 out of 10541 genes, 0.1% | 11 out of 12397 genes, 0.1% | 1 |
| C-acyltransferase activity | 10 out of 10541 genes, 0.1% | 11 out of 12397 genes, 0.1% | 1 |
| intramolecular oxidoreductase activity, transposing C=C bonds | 10 out of 10541 genes, 0.1% | 11 out of 12397 genes, 0.1% | 1 |
| neurotransmitter binding | 10 out of 10541 genes, 0.1% | 11 out of 12397 genes, 0.1% | 1 |
| hormone receptor binding | 10 out of 10541 genes, 0.1% | 11 out of 12397 genes, 0.1% | 1 |
| mannosidase activity | 27 out of 10541 genes, 0.3% | 31 out of 12397 genes, 0.3% | 1 |
| Ras GTPase binding | 27 out of 10541 genes, 0.3% | 31 out of 12397 genes, 0.3% | 1 |
| peptide binding | 27 out of 10541 genes, 0.3% | 31 out of 12397 genes, 0.3% | 1 |
| hydrolase activity | 2269 out of 10541 genes, 21.5% | 2668 out of 12397 genes, 21.5% | 1 |
| di-, tri-valent inorganic cation transmembrane transporter activity | 21 out of 10541 genes, 0.2% | 24 out of 12397 genes, 0.2% | 1 |
| transcription factor binding | 60 out of 10541 genes, 0.6% | 70 out of 12397 genes, 0.6% | 1 |
| chromatin binding | 15 out of 10541 genes, 0.1% | 17 out of 12397 genes, 0.1% | 1 |
| trehalase activity | 15 out of 10541 genes, 0.1% | 17 out of 12397 genes, 0.1% | 1 |
| protein complex binding | 15 out of 10541 genes, 0.1% | 17 out of 12397 genes, 0.1% | 1 |
| guanyl-nucleotide exchange factor activity | 88 out of 10541 genes, 0.8% | 103 out of 12397 genes, 0.8% | 1 |
| SNARE binding | 4 out of 10541 genes, 0.0% | 4 out of 12397 genes, 0.0% | 1 |
| DNA secondary structure binding | 4 out of 10541 genes, 0.0% | 4 out of 12397 genes, 0.0% | 1 |
| transcription elongation regulator activity | 4 out of 10541 genes, 0.0% | 4 out of 12397 genes, 0.0% | 1 |
| exodeoxyribonuclease activity | 4 out of 10541 genes, 0.0% | 4 out of 12397 genes, 0.0% | 1 |
| MAP kinase kinase activity | 4 out of 10541 genes, 0.0% | 4 out of 12397 genes, 0.0% | 1 |
| dopamine receptor activity | 4 out of 10541 genes, 0.0% | 4 out of 12397 genes, 0.0% | 1 |
| Rho guanyl-nucleotide exchange factor activity | 4 out of 10541 genes, 0.0% | 4 out of 12397 genes, 0.0% | 1 |
| GDP-dissociation inhibitor activity | 4 out of 10541 genes, 0.0% | 4 out of 12397 genes, 0.0% | 1 |
| water transmembrane transporter activity | 4 out of 10541 genes, 0.0% | 4 out of 12397 genes, 0.0% | 1 |
| cation:amino acid symporter activity | 4 out of 10541 genes, 0.0% | 4 out of 12397 genes, 0.0% | 1 |
| peptide disulfide oxidoreductase activity | 4 out of 10541 genes, 0.0% | 4 out of 12397 genes, 0.0% | 1 |
| acidic amino acid transmembrane transporter activity | 4 out of 10541 genes, 0.0% | 4 out of 12397 genes, 0.0% | 1 |
| AMP binding | 4 out of 10541 genes, 0.0% | 4 out of 12397 genes, 0.0% | 1 |
| acyl-[acyl-carrier-protein] hydrolase activity | 4 out of 10541 genes, 0.0% | 4 out of 12397 genes, 0.0% | 1 |
| hydrolase activity, acting on carbon-nitrogen (but not peptide) bonds, in linear amidines | 4 out of 10541 genes, 0.0% | 4 out of 12397 genes, 0.0% | 1 |
| hydrolase activity, acting on acid carbon-carbon bonds | 4 out of 10541 genes, 0.0% | 4 out of 12397 genes, 0.0% | 1 |
| hydrolase activity, acting on acid carbon-carbon bonds, in ketonic substances | 4 out of 10541 genes, 0.0% | 4 out of 12397 genes, 0.0% | 1 |
| endonuclease activity, active with either ribo- or deoxyribonucleic acids and producing 3'-phosphomonoesters | 4 out of 10541 genes, 0.0% | 4 out of 12397 genes, 0.0% | 1 |
| exodeoxyribonuclease activity, producing 5'-phosphomonoesters | 4 out of 10541 genes, 0.0% | 4 out of 12397 genes, 0.0% | 1 |
| GMP binding | 4 out of 10541 genes, 0.0% | 4 out of 12397 genes, 0.0% | 1 |
| histone kinase activity | 4 out of 10541 genes, 0.0% | 4 out of 12397 genes, 0.0% | 1 |
| dopamine binding | 4 out of 10541 genes, 0.0% | 4 out of 12397 genes, 0.0% | 1 |
| glutamate receptor binding | 4 out of 10541 genes, 0.0% | 4 out of 12397 genes, 0.0% | 1 |
| protein kinase A binding | 4 out of 10541 genes, 0.0% | 4 out of 12397 genes, 0.0% | 1 |
| peptidyl-lysine 5-dioxygenase activity | 4 out of 10541 genes, 0.0% | 4 out of 12397 genes, 0.0% | 1 |
| gated channel activity | 99 out of 10541 genes, 0.9% | 116 out of 12397 genes, 0.9% | 1 |
| ligand-gated ion channel activity | 76 out of 10541 genes, 0.7% | 89 out of 12397 genes, 0.7% | 1 |
| ligand-gated channel activity | 76 out of 10541 genes, 0.7% | 89 out of 12397 genes, 0.7% | 1 |
| DNA topoisomerase activity | 20 out of 10541 genes, 0.2% | 23 out of 12397 genes, 0.2% | 1 |
| hydro-lyase activity | 31 out of 10541 genes, 0.3% | 36 out of 12397 genes, 0.3% | 1 |
| microfilament motor activity | 9 out of 10541 genes, 0.1% | 10 out of 12397 genes, 0.1% | 1 |
| endodeoxyribonuclease activity | 9 out of 10541 genes, 0.1% | 10 out of 12397 genes, 0.1% | 1 |
| monovalent cation:hydrogen antiporter activity | 9 out of 10541 genes, 0.1% | 10 out of 12397 genes, 0.1% | 1 |
| anion:anion antiporter activity | 9 out of 10541 genes, 0.1% | 10 out of 12397 genes, 0.1% | 1 |
| neurotransmitter receptor activity | 9 out of 10541 genes, 0.1% | 10 out of 12397 genes, 0.1% | 1 |
| nuclear hormone receptor binding | 9 out of 10541 genes, 0.1% | 10 out of 12397 genes, 0.1% | 1 |
| tubulin binding | 53 out of 10541 genes, 0.5% | 62 out of 12397 genes, 0.5% | 1 |
| protein kinase regulator activity | 25 out of 10541 genes, 0.2% | 29 out of 12397 genes, 0.2% | 1 |
| RNA polymerase activity | 58 out of 10541 genes, 0.6% | 68 out of 12397 genes, 0.5% | 1 |
| protein transmembrane transporter activity | 14 out of 10541 genes, 0.1% | 16 out of 12397 genes, 0.1% | 1 |
| intramolecular oxidoreductase activity, interconverting aldoses and ketoses | 14 out of 10541 genes, 0.1% | 16 out of 12397 genes, 0.1% | 1 |
| macromolecule transmembrane transporter activity | 14 out of 10541 genes, 0.1% | 16 out of 12397 genes, 0.1% | 1 |
| histone methyltransferase activity | 14 out of 10541 genes, 0.1% | 16 out of 12397 genes, 0.1% | 1 |
| inositol or phosphatidylinositol kinase activity | 30 out of 10541 genes, 0.3% | 35 out of 12397 genes, 0.3% | 1 |
| mRNA binding | 19 out of 10541 genes, 0.2% | 22 out of 12397 genes, 0.2% | 1 |
| protein transporter activity | 19 out of 10541 genes, 0.2% | 22 out of 12397 genes, 0.2% | 1 |
| anion transmembrane transporter activity | 46 out of 10541 genes, 0.4% | 54 out of 12397 genes, 0.4% | 1 |
| lipid kinase activity | 24 out of 10541 genes, 0.2% | 28 out of 12397 genes, 0.2% | 1 |
| pattern binding | 24 out of 10541 genes, 0.2% | 28 out of 12397 genes, 0.2% | 1 |
| voltage-gated ion channel activity | 24 out of 10541 genes, 0.2% | 28 out of 12397 genes, 0.2% | 1 |
| voltage-gated channel activity | 24 out of 10541 genes, 0.2% | 28 out of 12397 genes, 0.2% | 1 |
| transition metal ion binding | 1285 out of 10541 genes, 12.2% | 1514 out of 12397 genes, 12.2% | 1 |
| exonuclease activity | 51 out of 10541 genes, 0.5% | 60 out of 12397 genes, 0.5% | 1 |
| metallopeptidase activity | 29 out of 10541 genes, 0.3% | 34 out of 12397 genes, 0.3% | 1 |
| succinate dehydrogenase activity | 8 out of 10541 genes, 0.1% | 9 out of 12397 genes, 0.1% | 1 |
| fatty acid synthase activity | 8 out of 10541 genes, 0.1% | 9 out of 12397 genes, 0.1% | 1 |
| tRNA methyltransferase activity | 8 out of 10541 genes, 0.1% | 9 out of 12397 genes, 0.1% | 1 |
| neuropeptide receptor activity | 8 out of 10541 genes, 0.1% | 9 out of 12397 genes, 0.1% | 1 |
| fucosyltransferase activity | 8 out of 10541 genes, 0.1% | 9 out of 12397 genes, 0.1% | 1 |
| oxidoreductase activity, acting on the CH-NH group of donors, NAD or NADP as acceptor | 8 out of 10541 genes, 0.1% | 9 out of 12397 genes, 0.1% | 1 |
| neuropeptide binding | 8 out of 10541 genes, 0.1% | 9 out of 12397 genes, 0.1% | 1 |
| hydrolase activity, acting on glycosyl bonds | 127 out of 10541 genes, 1.2% | 150 out of 12397 genes, 1.2% | 1 |
| lyase activity | 127 out of 10541 genes, 1.2% | 150 out of 12397 genes, 1.2% | 1 |
| transcription repressor activity | 13 out of 10541 genes, 0.1% | 15 out of 12397 genes, 0.1% | 1 |
| oxidoreductase activity, acting on the CH-CH group of donors, NAD or NADP as acceptor | 13 out of 10541 genes, 0.1% | 15 out of 12397 genes, 0.1% | 1 |
| phosphotransferase activity, nitrogenous group as acceptor | 13 out of 10541 genes, 0.1% | 15 out of 12397 genes, 0.1% | 1 |
| oxidized base lesion DNA N-glycosylase activity | 3 out of 10541 genes, 0.0% | 3 out of 12397 genes, 0.0% | 1 |
| inositol pyrophosphate synthase activity | 3 out of 10541 genes, 0.0% | 3 out of 12397 genes, 0.0% | 1 |
| alcohol dehydrogenase (NAD) activity | 3 out of 10541 genes, 0.0% | 3 out of 12397 genes, 0.0% | 1 |
| phospholipase A2 activity | 3 out of 10541 genes, 0.0% | 3 out of 12397 genes, 0.0% | 1 |
| transforming growth factor beta receptor binding | 3 out of 10541 genes, 0.0% | 3 out of 12397 genes, 0.0% | 1 |
| extracellular-glutamate-gated ion channel activity | 3 out of 10541 genes, 0.0% | 3 out of 12397 genes, 0.0% | 1 |
| sodium:amino acid symporter activity | 3 out of 10541 genes, 0.0% | 3 out of 12397 genes, 0.0% | 1 |
| dicarboxylic acid transmembrane transporter activity | 3 out of 10541 genes, 0.0% | 3 out of 12397 genes, 0.0% | 1 |
| neurotransmitter:sodium symporter activity | 3 out of 10541 genes, 0.0% | 3 out of 12397 genes, 0.0% | 1 |
| drug binding | 3 out of 10541 genes, 0.0% | 3 out of 12397 genes, 0.0% | 1 |
| C-methyltransferase activity | 3 out of 10541 genes, 0.0% | 3 out of 12397 genes, 0.0% | 1 |
| histone-arginine N-methyltransferase activity | 3 out of 10541 genes, 0.0% | 3 out of 12397 genes, 0.0% | 1 |
| drug transmembrane transporter activity | 3 out of 10541 genes, 0.0% | 3 out of 12397 genes, 0.0% | 1 |
| outward rectifier potassium channel activity | 3 out of 10541 genes, 0.0% | 3 out of 12397 genes, 0.0% | 1 |
| solute:hydrogen symporter activity | 3 out of 10541 genes, 0.0% | 3 out of 12397 genes, 0.0% | 1 |
| ammonia ligase activity | 3 out of 10541 genes, 0.0% | 3 out of 12397 genes, 0.0% | 1 |
| tRNA (guanine) methyltransferase activity | 3 out of 10541 genes, 0.0% | 3 out of 12397 genes, 0.0% | 1 |
| aldehyde-lyase activity | 3 out of 10541 genes, 0.0% | 3 out of 12397 genes, 0.0% | 1 |
| acid-ammonia (or amide) ligase activity | 3 out of 10541 genes, 0.0% | 3 out of 12397 genes, 0.0% | 1 |
| NAD-dependent histone deacetylase activity | 3 out of 10541 genes, 0.0% | 3 out of 12397 genes, 0.0% | 1 |
| phosphatase inhibitor activity | 3 out of 10541 genes, 0.0% | 3 out of 12397 genes, 0.0% | 1 |
| translation repressor activity | 3 out of 10541 genes, 0.0% | 3 out of 12397 genes, 0.0% | 1 |
| quinone cofactor methyltransferase activity | 3 out of 10541 genes, 0.0% | 3 out of 12397 genes, 0.0% | 1 |
| protein binding, bridging | 3 out of 10541 genes, 0.0% | 3 out of 12397 genes, 0.0% | 1 |
| demethylase activity | 3 out of 10541 genes, 0.0% | 3 out of 12397 genes, 0.0% | 1 |
| histone demethylase activity | 3 out of 10541 genes, 0.0% | 3 out of 12397 genes, 0.0% | 1 |
| steroid dehydrogenase activity, acting on the CH-CH group of donors | 3 out of 10541 genes, 0.0% | 3 out of 12397 genes, 0.0% | 1 |
| peptide N-acetyltransferase activity | 3 out of 10541 genes, 0.0% | 3 out of 12397 genes, 0.0% | 1 |
| NAD-dependent protein deacetylase activity | 3 out of 10541 genes, 0.0% | 3 out of 12397 genes, 0.0% | 1 |
| 5,10-methylenetetrahydrofolate-dependent methyltransferase activity | 3 out of 10541 genes, 0.0% | 3 out of 12397 genes, 0.0% | 1 |
| amide transmembrane transporter activity | 3 out of 10541 genes, 0.0% | 3 out of 12397 genes, 0.0% | 1 |
| translation regulator activity | 3 out of 10541 genes, 0.0% | 3 out of 12397 genes, 0.0% | 1 |
| NADP or NADPH binding | 3 out of 10541 genes, 0.0% | 3 out of 12397 genes, 0.0% | 1 |
| hydrogen ion transmembrane transporter activity | 39 out of 10541 genes, 0.4% | 46 out of 12397 genes, 0.4% | 1 |
| general RNA polymerase II transcription factor activity | 23 out of 10541 genes, 0.2% | 27 out of 12397 genes, 0.2% | 1 |
| cation transmembrane transporter activity | 291 out of 10541 genes, 2.8% | 344 out of 12397 genes, 2.8% | 1 |
| hydrolase activity, acting on ester bonds | 631 out of 10541 genes, 6.0% | 745 out of 12397 genes, 6.0% | 1 |
| motor activity | 92 out of 10541 genes, 0.9% | 109 out of 12397 genes, 0.9% | 1 |
| RNA polymerase II transcription factor activity | 38 out of 10541 genes, 0.4% | 45 out of 12397 genes, 0.4% | 1 |
| enzyme activator activity | 97 out of 10541 genes, 0.9% | 115 out of 12397 genes, 0.9% | 1 |
| O-acyltransferase activity | 12 out of 10541 genes, 0.1% | 14 out of 12397 genes, 0.1% | 1 |
| sodium ion transmembrane transporter activity | 12 out of 10541 genes, 0.1% | 14 out of 12397 genes, 0.1% | 1 |
| solute:sodium symporter activity | 12 out of 10541 genes, 0.1% | 14 out of 12397 genes, 0.1% | 1 |
| oxidoreductase activity, acting on the CH-NH group of donors | 12 out of 10541 genes, 0.1% | 14 out of 12397 genes, 0.1% | 1 |
| phosphoinositide 3-kinase activity | 12 out of 10541 genes, 0.1% | 14 out of 12397 genes, 0.1% | 1 |
| carboxylic acid transmembrane transporter activity | 12 out of 10541 genes, 0.1% | 14 out of 12397 genes, 0.1% | 1 |
| cyclic-nucleotide phosphodiesterase activity | 17 out of 10541 genes, 0.2% | 20 out of 12397 genes, 0.2% | 1 |
| monooxygenase activity | 17 out of 10541 genes, 0.2% | 20 out of 12397 genes, 0.2% | 1 |
| phosphatase regulator activity | 17 out of 10541 genes, 0.2% | 20 out of 12397 genes, 0.2% | 1 |
| amine binding | 22 out of 10541 genes, 0.2% | 26 out of 12397 genes, 0.2% | 1 |
| protein serine/threonine/tyrosine kinase activity | 7 out of 10541 genes, 0.1% | 8 out of 12397 genes, 0.1% | 1 |
| ubiquitin-protein ligase activity | 7 out of 10541 genes, 0.1% | 8 out of 12397 genes, 0.1% | 1 |
| amino acid transmembrane transporter activity | 7 out of 10541 genes, 0.1% | 8 out of 12397 genes, 0.1% | 1 |
| glutamate synthase activity | 7 out of 10541 genes, 0.1% | 8 out of 12397 genes, 0.1% | 1 |
| oxidoreductase activity, acting on paired donors, with incorporation or reduction of molecular oxygen, reduced ascorbate as one donor, and incorporation of one atom of oxygen | 7 out of 10541 genes, 0.1% | 8 out of 12397 genes, 0.1% | 1 |
| phosphatidylinositol bisphosphate phosphatase activity | 7 out of 10541 genes, 0.1% | 8 out of 12397 genes, 0.1% | 1 |
| GTPase activator activity | 53 out of 10541 genes, 0.5% | 63 out of 12397 genes, 0.5% | 1 |
| peptidase regulator activity | 32 out of 10541 genes, 0.3% | 38 out of 12397 genes, 0.3% | 1 |
| RNA binding | 330 out of 10541 genes, 3.1% | 391 out of 12397 genes, 3.2% | 1 |
| Ras GTPase activator activity | 47 out of 10541 genes, 0.4% | 56 out of 12397 genes, 0.5% | 1 |
| peptidase inhibitor activity | 21 out of 10541 genes, 0.2% | 25 out of 12397 genes, 0.2% | 1 |
| intracellular ligand-gated ion channel activity | 16 out of 10541 genes, 0.2% | 19 out of 12397 genes, 0.2% | 1 |
| protein phosphatase regulator activity | 16 out of 10541 genes, 0.2% | 19 out of 12397 genes, 0.2% | 1 |
| intramolecular oxidoreductase activity | 31 out of 10541 genes, 0.3% | 37 out of 12397 genes, 0.3% | 1 |
| GTPase regulator activity | 251 out of 10541 genes, 2.4% | 298 out of 12397 genes, 2.4% | 1 |
| nucleoside-triphosphatase regulator activity | 251 out of 10541 genes, 2.4% | 298 out of 12397 genes, 2.4% | 1 |
| isomerase activity | 153 out of 10541 genes, 1.5% | 182 out of 12397 genes, 1.5% | 1 |
| metal ion transmembrane transporter activity | 41 out of 10541 genes, 0.4% | 49 out of 12397 genes, 0.4% | 1 |
| inositol or phosphatidylinositol phosphatase activity | 11 out of 10541 genes, 0.1% | 13 out of 12397 genes, 0.1% | 1 |
| protein histidine kinase activity | 11 out of 10541 genes, 0.1% | 13 out of 12397 genes, 0.1% | 1 |
| steroid dehydrogenase activity | 11 out of 10541 genes, 0.1% | 13 out of 12397 genes, 0.1% | 1 |
| carbon-sulfur lyase activity | 11 out of 10541 genes, 0.1% | 13 out of 12397 genes, 0.1% | 1 |
| histone-lysine N-methyltransferase activity | 11 out of 10541 genes, 0.1% | 13 out of 12397 genes, 0.1% | 1 |
| nucleobase, nucleoside, nucleotide kinase activity | 11 out of 10541 genes, 0.1% | 13 out of 12397 genes, 0.1% | 1 |
| acid-amino acid ligase activity | 147 out of 10541 genes, 1.4% | 175 out of 12397 genes, 1.4% | 1 |
| iron ion binding | 82 out of 10541 genes, 0.8% | 98 out of 12397 genes, 0.8% | 1 |
| endopeptidase activity | 221 out of 10541 genes, 2.1% | 263 out of 12397 genes, 2.1% | 1 |
| receptor signaling protein activity | 35 out of 10541 genes, 0.3% | 42 out of 12397 genes, 0.3% | 1 |
| lipid binding | 108 out of 10541 genes, 1.0% | 129 out of 12397 genes, 1.0% | 1 |
| ligase activity | 401 out of 10541 genes, 3.8% | 476 out of 12397 genes, 3.8% | 1 |
| carboxy-lyase activity | 20 out of 10541 genes, 0.2% | 24 out of 12397 genes, 0.2% | 1 |
| iron ion transmembrane transporter activity | 6 out of 10541 genes, 0.1% | 7 out of 12397 genes, 0.1% | 1 |
| protein tyrosine/serine/threonine phosphatase activity | 6 out of 10541 genes, 0.1% | 7 out of 12397 genes, 0.1% | 1 |
| protein prenyltransferase activity | 6 out of 10541 genes, 0.1% | 7 out of 12397 genes, 0.1% | 1 |
| 5'-3' exonuclease activity | 6 out of 10541 genes, 0.1% | 7 out of 12397 genes, 0.1% | 1 |
| polyol transmembrane transporter activity | 6 out of 10541 genes, 0.1% | 7 out of 12397 genes, 0.1% | 1 |
| alcohol transmembrane transporter activity | 6 out of 10541 genes, 0.1% | 7 out of 12397 genes, 0.1% | 1 |
| arginine N-methyltransferase activity | 6 out of 10541 genes, 0.1% | 7 out of 12397 genes, 0.1% | 1 |
| protein-arginine N-methyltransferase activity | 6 out of 10541 genes, 0.1% | 7 out of 12397 genes, 0.1% | 1 |
| phosphatidylinositol phosphate kinase activity | 6 out of 10541 genes, 0.1% | 7 out of 12397 genes, 0.1% | 1 |
| transferase activity, transferring aldehyde or ketonic groups | 6 out of 10541 genes, 0.1% | 7 out of 12397 genes, 0.1% | 1 |
| transition metal ion transmembrane transporter activity | 6 out of 10541 genes, 0.1% | 7 out of 12397 genes, 0.1% | 1 |
| oxidoreductase activity, acting on the CH-NH2 group of donors | 15 out of 10541 genes, 0.1% | 18 out of 12397 genes, 0.1% | 1 |
| monosaccharide binding | 15 out of 10541 genes, 0.1% | 18 out of 12397 genes, 0.1% | 1 |
| inositol hexakisphosphate kinase activity | 2 out of 10541 genes, 0.0% | 2 out of 12397 genes, 0.0% | 1 |
| regulatory region DNA binding | 2 out of 10541 genes, 0.0% | 2 out of 12397 genes, 0.0% | 1 |
| regulatory region nucleic acid binding | 2 out of 10541 genes, 0.0% | 2 out of 12397 genes, 0.0% | 1 |
| dopamine receptor activity, coupled via Gi/Go | 2 out of 10541 genes, 0.0% | 2 out of 12397 genes, 0.0% | 1 |
| transcription termination factor activity | 2 out of 10541 genes, 0.0% | 2 out of 12397 genes, 0.0% | 1 |
| DNA-(apurinic or apyrimidinic site) lyase activity | 2 out of 10541 genes, 0.0% | 2 out of 12397 genes, 0.0% | 1 |
| DNA photolyase activity | 2 out of 10541 genes, 0.0% | 2 out of 12397 genes, 0.0% | 1 |
| aldo-keto reductase activity | 2 out of 10541 genes, 0.0% | 2 out of 12397 genes, 0.0% | 1 |
| farnesyltranstransferase activity | 2 out of 10541 genes, 0.0% | 2 out of 12397 genes, 0.0% | 1 |
| hexokinase activity | 2 out of 10541 genes, 0.0% | 2 out of 12397 genes, 0.0% | 1 |
| ionotropic glutamate receptor activity | 2 out of 10541 genes, 0.0% | 2 out of 12397 genes, 0.0% | 1 |
| transforming growth factor beta receptor activity | 2 out of 10541 genes, 0.0% | 2 out of 12397 genes, 0.0% | 1 |
| transmembrane receptor protein tyrosine kinase signaling protein activity | 2 out of 10541 genes, 0.0% | 2 out of 12397 genes, 0.0% | 1 |
| L-glutamate transmembrane transporter activity | 2 out of 10541 genes, 0.0% | 2 out of 12397 genes, 0.0% | 1 |
| retinoid binding | 2 out of 10541 genes, 0.0% | 2 out of 12397 genes, 0.0% | 1 |
| serine-type peptidase activity | 2 out of 10541 genes, 0.0% | 2 out of 12397 genes, 0.0% | 1 |
| 3'-5'-exodeoxyribonuclease activity | 2 out of 10541 genes, 0.0% | 2 out of 12397 genes, 0.0% | 1 |
| tetracycline transporter activity | 2 out of 10541 genes, 0.0% | 2 out of 12397 genes, 0.0% | 1 |
| rRNA methyltransferase activity | 2 out of 10541 genes, 0.0% | 2 out of 12397 genes, 0.0% | 1 |
| mannose-phosphate guanylyltransferase activity | 2 out of 10541 genes, 0.0% | 2 out of 12397 genes, 0.0% | 1 |
| promoter binding | 2 out of 10541 genes, 0.0% | 2 out of 12397 genes, 0.0% | 1 |
| glutathione disulfide oxidoreductase activity | 2 out of 10541 genes, 0.0% | 2 out of 12397 genes, 0.0% | 1 |
| organic cation transmembrane transporter activity | 2 out of 10541 genes, 0.0% | 2 out of 12397 genes, 0.0% | 1 |
| tricarboxylic acid transmembrane transporter activity | 2 out of 10541 genes, 0.0% | 2 out of 12397 genes, 0.0% | 1 |
| L-amino acid transmembrane transporter activity | 2 out of 10541 genes, 0.0% | 2 out of 12397 genes, 0.0% | 1 |
| carnitine O-acyltransferase activity | 2 out of 10541 genes, 0.0% | 2 out of 12397 genes, 0.0% | 1 |
| S-acetyltransferase activity | 2 out of 10541 genes, 0.0% | 2 out of 12397 genes, 0.0% | 1 |
| tRNA (adenine) methyltransferase activity | 2 out of 10541 genes, 0.0% | 2 out of 12397 genes, 0.0% | 1 |
| cyclin-dependent protein kinase regulator activity | 2 out of 10541 genes, 0.0% | 2 out of 12397 genes, 0.0% | 1 |
| oxidoreductase activity, acting on the CH-CH group of donors, quinone or related compound as acceptor | 2 out of 10541 genes, 0.0% | 2 out of 12397 genes, 0.0% | 1 |
| oxidoreductase activity, acting on the CH-NH2 group of donors, oxygen as acceptor | 2 out of 10541 genes, 0.0% | 2 out of 12397 genes, 0.0% | 1 |
| oxidoreductase activity, acting on the CH-NH group of donors, quinone or similar compound as acceptor | 2 out of 10541 genes, 0.0% | 2 out of 12397 genes, 0.0% | 1 |
| oxidoreductase activity, acting on NADH or NADPH, nitrogenous group as acceptor | 2 out of 10541 genes, 0.0% | 2 out of 12397 genes, 0.0% | 1 |
| oxidoreductase activity, acting on paired donors, with incorporation or reduction of molecular oxygen, reduced pteridine as one donor, and incorporation of one atom of oxygen | 2 out of 10541 genes, 0.0% | 2 out of 12397 genes, 0.0% | 1 |
| trialkylsulfonium hydrolase activity | 2 out of 10541 genes, 0.0% | 2 out of 12397 genes, 0.0% | 1 |
| endodeoxyribonuclease activity, producing 3'-phosphomonoesters | 2 out of 10541 genes, 0.0% | 2 out of 12397 genes, 0.0% | 1 |
| endoribonuclease activity, producing 3'-phosphomonoesters | 2 out of 10541 genes, 0.0% | 2 out of 12397 genes, 0.0% | 1 |
| GABA receptor activity | 2 out of 10541 genes, 0.0% | 2 out of 12397 genes, 0.0% | 1 |
| serine hydrolase activity | 2 out of 10541 genes, 0.0% | 2 out of 12397 genes, 0.0% | 1 |
| kinase activator activity | 2 out of 10541 genes, 0.0% | 2 out of 12397 genes, 0.0% | 1 |
| isoprenoid binding | 2 out of 10541 genes, 0.0% | 2 out of 12397 genes, 0.0% | 1 |
| syntaxin binding | 2 out of 10541 genes, 0.0% | 2 out of 12397 genes, 0.0% | 1 |
| cytokine binding | 2 out of 10541 genes, 0.0% | 2 out of 12397 genes, 0.0% | 1 |
| interferon binding | 2 out of 10541 genes, 0.0% | 2 out of 12397 genes, 0.0% | 1 |
| protein kinase activator activity | 2 out of 10541 genes, 0.0% | 2 out of 12397 genes, 0.0% | 1 |
| alpha(1,2)-fucosyltransferase activity | 2 out of 10541 genes, 0.0% | 2 out of 12397 genes, 0.0% | 1 |
| lipoic acid binding | 2 out of 10541 genes, 0.0% | 2 out of 12397 genes, 0.0% | 1 |
| opioid receptor binding | 2 out of 10541 genes, 0.0% | 2 out of 12397 genes, 0.0% | 1 |
| single base insertion or deletion binding | 2 out of 10541 genes, 0.0% | 2 out of 12397 genes, 0.0% | 1 |
| histone demethylase activity (H3-K4 specific) | 2 out of 10541 genes, 0.0% | 2 out of 12397 genes, 0.0% | 1 |
| apolipoprotein binding | 2 out of 10541 genes, 0.0% | 2 out of 12397 genes, 0.0% | 1 |
| histone serine kinase activity | 2 out of 10541 genes, 0.0% | 2 out of 12397 genes, 0.0% | 1 |
| histone threonine kinase activity | 2 out of 10541 genes, 0.0% | 2 out of 12397 genes, 0.0% | 1 |
| MHC protein binding | 2 out of 10541 genes, 0.0% | 2 out of 12397 genes, 0.0% | 1 |
| antibiotic transporter activity | 2 out of 10541 genes, 0.0% | 2 out of 12397 genes, 0.0% | 1 |
| neurotrophin binding | 2 out of 10541 genes, 0.0% | 2 out of 12397 genes, 0.0% | 1 |
| ubiquitin binding | 2 out of 10541 genes, 0.0% | 2 out of 12397 genes, 0.0% | 1 |
| anion binding | 2 out of 10541 genes, 0.0% | 2 out of 12397 genes, 0.0% | 1 |
| transcription regulatory region DNA binding | 2 out of 10541 genes, 0.0% | 2 out of 12397 genes, 0.0% | 1 |
| protein phosphorylated amino acid binding | 2 out of 10541 genes, 0.0% | 2 out of 12397 genes, 0.0% | 1 |
| alpha(1,6)-fucosyltransferase activity | 2 out of 10541 genes, 0.0% | 2 out of 12397 genes, 0.0% | 1 |
| 3',5'-cyclic-GMP phosphodiesterase activity | 2 out of 10541 genes, 0.0% | 2 out of 12397 genes, 0.0% | 1 |
| alanine-oxo-acid transaminase activity | 2 out of 10541 genes, 0.0% | 2 out of 12397 genes, 0.0% | 1 |
| quinone binding | 2 out of 10541 genes, 0.0% | 2 out of 12397 genes, 0.0% | 1 |
| cofactor transporter activity | 2 out of 10541 genes, 0.0% | 2 out of 12397 genes, 0.0% | 1 |
| phosphoprotein binding | 2 out of 10541 genes, 0.0% | 2 out of 12397 genes, 0.0% | 1 |
| protein methylesterase activity | 2 out of 10541 genes, 0.0% | 2 out of 12397 genes, 0.0% | 1 |
| oxidoreductase activity, acting on a sulfur group of donors | 34 out of 10541 genes, 0.3% | 41 out of 12397 genes, 0.3% | 1 |
| O-methyltransferase activity | 10 out of 10541 genes, 0.1% | 12 out of 12397 genes, 0.1% | 1 |
| endopeptidase regulator activity | 24 out of 10541 genes, 0.2% | 29 out of 12397 genes, 0.2% | 1 |
| double-stranded DNA binding | 19 out of 10541 genes, 0.2% | 23 out of 12397 genes, 0.2% | 1 |
| phosphotransferase activity, phosphate group as acceptor | 19 out of 10541 genes, 0.2% | 23 out of 12397 genes, 0.2% | 1 |
| protein dimerization activity | 38 out of 10541 genes, 0.4% | 46 out of 12397 genes, 0.4% | 1 |
| nuclease activity | 157 out of 10541 genes, 1.5% | 188 out of 12397 genes, 1.5% | 1 |
| intramolecular transferase activity | 52 out of 10541 genes, 0.5% | 63 out of 12397 genes, 0.5% | 1 |
| transferase activity, transferring acyl groups other than amino-acyl groups | 150 out of 10541 genes, 1.4% | 180 out of 12397 genes, 1.5% | 1 |
| nucleotidyltransferase activity | 187 out of 10541 genes, 1.8% | 224 out of 12397 genes, 1.8% | 1 |
| translation release factor activity | 5 out of 10541 genes, 0.0% | 6 out of 12397 genes, 0.0% | 1 |
| transmembrane receptor protein serine/threonine kinase activity | 5 out of 10541 genes, 0.0% | 6 out of 12397 genes, 0.0% | 1 |
| sulfotransferase activity | 5 out of 10541 genes, 0.0% | 6 out of 12397 genes, 0.0% | 1 |
| carbon-nitrogen lyase activity | 5 out of 10541 genes, 0.0% | 6 out of 12397 genes, 0.0% | 1 |
| Rho GTPase binding | 5 out of 10541 genes, 0.0% | 6 out of 12397 genes, 0.0% | 1 |
| peptidase activity | 429 out of 10541 genes, 4.1% | 511 out of 12397 genes, 4.1% | 1 |
| symporter activity | 32 out of 10541 genes, 0.3% | 39 out of 12397 genes, 0.3% | 1 |
| transferase activity, transferring alkyl or aryl (other than methyl) groups | 32 out of 10541 genes, 0.3% | 39 out of 12397 genes, 0.3% | 1 |
| DNA-directed RNA polymerase activity | 9 out of 10541 genes, 0.1% | 11 out of 12397 genes, 0.1% | 1 |
| small protein activating enzyme activity | 9 out of 10541 genes, 0.1% | 11 out of 12397 genes, 0.1% | 1 |
| solute:hydrogen antiporter activity | 9 out of 10541 genes, 0.1% | 11 out of 12397 genes, 0.1% | 1 |
| CoA desaturase activity | 9 out of 10541 genes, 0.1% | 11 out of 12397 genes, 0.1% | 1 |
| malate dehydrogenase activity | 9 out of 10541 genes, 0.1% | 11 out of 12397 genes, 0.1% | 1 |
| oxidoreductase activity, acting on the aldehyde or oxo group of donors, disulfide as acceptor | 9 out of 10541 genes, 0.1% | 11 out of 12397 genes, 0.1% | 1 |
| oxidoreductase activity, acting on paired donors, with oxidation of a pair of donors resulting in the reduction of molecular oxygen to two molecules of water | 9 out of 10541 genes, 0.1% | 11 out of 12397 genes, 0.1% | 1 |
| solute:cation symporter activity | 27 out of 10541 genes, 0.3% | 33 out of 12397 genes, 0.3% | 1 |
| inorganic cation transmembrane transporter activity | 97 out of 10541 genes, 0.9% | 117 out of 12397 genes, 0.9% | 1 |
| endopeptidase inhibitor activity | 13 out of 10541 genes, 0.1% | 16 out of 12397 genes, 0.1% | 1 |
| glucosidase activity | 13 out of 10541 genes, 0.1% | 16 out of 12397 genes, 0.1% | 1 |
| CoA carboxylase activity | 13 out of 10541 genes, 0.1% | 16 out of 12397 genes, 0.1% | 1 |
| ligase activity, forming carbon-carbon bonds | 13 out of 10541 genes, 0.1% | 16 out of 12397 genes, 0.1% | 1 |
| 3'-5' exonuclease activity | 22 out of 10541 genes, 0.2% | 27 out of 12397 genes, 0.2% | 1 |
| acyltransferase activity | 111 out of 10541 genes, 1.1% | 134 out of 12397 genes, 1.1% | 1 |
| amino acid binding | 17 out of 10541 genes, 0.2% | 21 out of 12397 genes, 0.2% | 1 |
| ligase activity, forming carbon-sulfur bonds | 17 out of 10541 genes, 0.2% | 21 out of 12397 genes, 0.2% | 1 |
| oxidoreductase activity, acting on NADH or NADPH | 50 out of 10541 genes, 0.5% | 61 out of 12397 genes, 0.5% | 1 |
| N-acyltransferase activity | 35 out of 10541 genes, 0.3% | 43 out of 12397 genes, 0.3% | 1 |
| actin binding | 30 out of 10541 genes, 0.3% | 37 out of 12397 genes, 0.3% | 1 |
| translation termination factor activity | 8 out of 10541 genes, 0.1% | 10 out of 12397 genes, 0.1% | 1 |
| glucuronosyltransferase activity | 8 out of 10541 genes, 0.1% | 10 out of 12397 genes, 0.1% | 1 |
| steroid dehydrogenase activity, acting on the CH-OH group of donors, NAD or NADP as acceptor | 8 out of 10541 genes, 0.1% | 10 out of 12397 genes, 0.1% | 1 |
| small conjugating protein-specific protease activity | 12 out of 10541 genes, 0.1% | 15 out of 12397 genes, 0.1% | 1 |
| cation binding | 2000 out of 10541 genes, 19.0% | 2369 out of 12397 genes, 19.1% | 1 |
| protein geranylgeranyltransferase activity | 4 out of 10541 genes, 0.0% | 5 out of 12397 genes, 0.0% | 1 |
| palmitoyltransferase activity | 4 out of 10541 genes, 0.0% | 5 out of 12397 genes, 0.0% | 1 |
| S-acyltransferase activity | 4 out of 10541 genes, 0.0% | 5 out of 12397 genes, 0.0% | 1 |
| oxidoreductase activity, acting on a sulfur group of donors, disulfide as acceptor | 4 out of 10541 genes, 0.0% | 5 out of 12397 genes, 0.0% | 1 |
| oxidoreductase activity, acting on paired donors, with incorporation or reduction of molecular oxygen, NADH or NADPH as one donor, and incorporation of one atom of oxygen | 4 out of 10541 genes, 0.0% | 5 out of 12397 genes, 0.0% | 1 |
| hydroxymethyl-, formyl- and related transferase activity | 4 out of 10541 genes, 0.0% | 5 out of 12397 genes, 0.0% | 1 |
| protein phosphatase binding | 4 out of 10541 genes, 0.0% | 5 out of 12397 genes, 0.0% | 1 |
| identical protein binding | 72 out of 10541 genes, 0.7% | 88 out of 12397 genes, 0.7% | 1 |
| oxidoreductase activity, acting on the CH-OH group of donors, NAD or NADP as acceptor | 62 out of 10541 genes, 0.6% | 76 out of 12397 genes, 0.6% | 1 |
| N-acetyltransferase activity | 33 out of 10541 genes, 0.3% | 41 out of 12397 genes, 0.3% | 1 |
| carbon-carbon lyase activity | 33 out of 10541 genes, 0.3% | 41 out of 12397 genes, 0.3% | 1 |
| monovalent inorganic cation transmembrane transporter activity | 76 out of 10541 genes, 0.7% | 93 out of 12397 genes, 0.8% | 1 |
| succinate-CoA ligase activity | 11 out of 10541 genes, 0.1% | 14 out of 12397 genes, 0.1% | 1 |
| acid-thiol ligase activity | 11 out of 10541 genes, 0.1% | 14 out of 12397 genes, 0.1% | 1 |
| ATPase activity, coupled to transmembrane movement of ions, phosphorylative mechanism | 15 out of 10541 genes, 0.1% | 19 out of 12397 genes, 0.2% | 1 |
| CoA-ligase activity | 15 out of 10541 genes, 0.1% | 19 out of 12397 genes, 0.2% | 1 |
| transferase activity, transferring amino-acyl groups | 15 out of 10541 genes, 0.1% | 19 out of 12397 genes, 0.2% | 1 |
| tRNA-specific ribonuclease activity | 7 out of 10541 genes, 0.1% | 9 out of 12397 genes, 0.1% | 1 |
| diphosphotransferase activity | 7 out of 10541 genes, 0.1% | 9 out of 12397 genes, 0.1% | 1 |
| racemase and epimerase activity | 7 out of 10541 genes, 0.1% | 9 out of 12397 genes, 0.1% | 1 |
| intramolecular oxidoreductase activity, interconverting keto- and enol-groups | 7 out of 10541 genes, 0.1% | 9 out of 12397 genes, 0.1% | 1 |
| guanyl ribonucleotide binding | 306 out of 10541 genes, 2.9% | 368 out of 12397 genes, 3.0% | 1 |
| oxidoreductase activity | 443 out of 10541 genes, 4.2% | 531 out of 12397 genes, 4.3% | 1 |
| metal ion binding | 1783 out of 10541 genes, 16.9% | 2116 out of 12397 genes, 17.1% | 1 |
| cyclase activity | 23 out of 10541 genes, 0.2% | 29 out of 12397 genes, 0.2% | 1 |
| prenyltransferase activity | 14 out of 10541 genes, 0.1% | 18 out of 12397 genes, 0.1% | 1 |
| guanyl nucleotide binding | 306 out of 10541 genes, 2.9% | 369 out of 12397 genes, 3.0% | 1 |
| receptor signaling protein serine/threonine kinase activity | 26 out of 10541 genes, 0.2% | 33 out of 12397 genes, 0.3% | 1 |
| anion channel activity | 26 out of 10541 genes, 0.2% | 33 out of 12397 genes, 0.3% | 1 |
| carboxypeptidase activity | 3 out of 10541 genes, 0.0% | 4 out of 12397 genes, 0.0% | 1 |
| malic enzyme activity | 3 out of 10541 genes, 0.0% | 4 out of 12397 genes, 0.0% | 1 |
| nucleotide diphosphatase activity | 3 out of 10541 genes, 0.0% | 4 out of 12397 genes, 0.0% | 1 |
| phosphoenolpyruvate carboxykinase activity | 3 out of 10541 genes, 0.0% | 4 out of 12397 genes, 0.0% | 1 |
| protein kinase C activity | 3 out of 10541 genes, 0.0% | 4 out of 12397 genes, 0.0% | 1 |
| nucleoside transmembrane transporter activity | 3 out of 10541 genes, 0.0% | 4 out of 12397 genes, 0.0% | 1 |
| myo-inositol transmembrane transporter activity | 3 out of 10541 genes, 0.0% | 4 out of 12397 genes, 0.0% | 1 |
| sulfuric ester hydrolase activity | 3 out of 10541 genes, 0.0% | 4 out of 12397 genes, 0.0% | 1 |
| electron carrier activity | 3 out of 10541 genes, 0.0% | 4 out of 12397 genes, 0.0% | 1 |
| purine nucleoside transmembrane transporter activity | 3 out of 10541 genes, 0.0% | 4 out of 12397 genes, 0.0% | 1 |
| galactosidase activity | 3 out of 10541 genes, 0.0% | 4 out of 12397 genes, 0.0% | 1 |
| fucosidase activity | 3 out of 10541 genes, 0.0% | 4 out of 12397 genes, 0.0% | 1 |
| oxidoreductase activity, acting on a sulfur group of donors, NAD or NADP as acceptor | 3 out of 10541 genes, 0.0% | 4 out of 12397 genes, 0.0% | 1 |
| succinyltransferase activity | 3 out of 10541 genes, 0.0% | 4 out of 12397 genes, 0.0% | 1 |
| amidine-lyase activity | 3 out of 10541 genes, 0.0% | 4 out of 12397 genes, 0.0% | 1 |
| endodeoxyribonuclease activity, producing 5'-phosphomonoesters | 3 out of 10541 genes, 0.0% | 4 out of 12397 genes, 0.0% | 1 |
| oxygen binding | 3 out of 10541 genes, 0.0% | 4 out of 12397 genes, 0.0% | 1 |
| growth factor binding | 3 out of 10541 genes, 0.0% | 4 out of 12397 genes, 0.0% | 1 |
| heparan sulfate sulfotransferase activity | 3 out of 10541 genes, 0.0% | 4 out of 12397 genes, 0.0% | 1 |
| cytidylyltransferase activity | 3 out of 10541 genes, 0.0% | 4 out of 12397 genes, 0.0% | 1 |
| nuclear export signal receptor activity | 6 out of 10541 genes, 0.1% | 8 out of 12397 genes, 0.1% | 1 |
| mismatched DNA binding | 6 out of 10541 genes, 0.1% | 8 out of 12397 genes, 0.1% | 1 |
| growth factor receptor binding | 6 out of 10541 genes, 0.1% | 8 out of 12397 genes, 0.1% | 1 |
| ligase activity, forming carbon-nitrogen bonds | 210 out of 10541 genes, 2.0% | 255 out of 12397 genes, 2.1% | 1 |
| calcium channel activity | 17 out of 10541 genes, 0.2% | 22 out of 12397 genes, 0.2% | 1 |
| mannosyl-oligosaccharide mannosidase activity | 13 out of 10541 genes, 0.1% | 17 out of 12397 genes, 0.1% | 1 |
| transferase activity, transferring acyl groups, acyl groups converted into alkyl on transfer | 13 out of 10541 genes, 0.1% | 17 out of 12397 genes, 0.1% | 1 |
| cysteine-type peptidase activity | 38 out of 10541 genes, 0.4% | 48 out of 12397 genes, 0.4% | 1 |
| acetyltransferase activity | 56 out of 10541 genes, 0.5% | 70 out of 12397 genes, 0.6% | 1 |
| lysine N-acetyltransferase activity | 29 out of 10541 genes, 0.3% | 37 out of 12397 genes, 0.3% | 1 |
| ion binding | 2032 out of 10541 genes, 19.3% | 2414 out of 12397 genes, 19.5% | 1 |
| transferase activity, transferring acyl groups | 182 out of 10541 genes, 1.7% | 222 out of 12397 genes, 1.8% | 1 |
| oxidoreductase activity, acting on NADH or NADPH, quinone or similar compound as acceptor | 28 out of 10541 genes, 0.3% | 36 out of 12397 genes, 0.3% | 1 |
| NADH dehydrogenase (quinone) activity | 28 out of 10541 genes, 0.3% | 36 out of 12397 genes, 0.3% | 1 |
| potassium ion transmembrane transporter activity | 12 out of 10541 genes, 0.1% | 16 out of 12397 genes, 0.1% | 1 |
| channel regulator activity | 5 out of 10541 genes, 0.0% | 7 out of 12397 genes, 0.1% | 1 |
| racemase and epimerase activity, acting on carbohydrates and derivatives | 5 out of 10541 genes, 0.0% | 7 out of 12397 genes, 0.1% | 1 |
| carbohydrate phosphatase activity | 5 out of 10541 genes, 0.0% | 7 out of 12397 genes, 0.1% | 1 |
| DNA insertion or deletion binding | 5 out of 10541 genes, 0.0% | 7 out of 12397 genes, 0.1% | 1 |
| lipoprotein receptor binding | 5 out of 10541 genes, 0.0% | 7 out of 12397 genes, 0.1% | 1 |
| endonuclease activity | 36 out of 10541 genes, 0.3% | 46 out of 12397 genes, 0.4% | 1 |
| carboxylic acid binding | 36 out of 10541 genes, 0.3% | 46 out of 12397 genes, 0.4% | 1 |
| carbon-nitrogen ligase activity, with glutamine as amido-N-donor | 8 out of 10541 genes, 0.1% | 11 out of 12397 genes, 0.1% | 1 |
| thiolester hydrolase activity | 90 out of 10541 genes, 0.9% | 112 out of 12397 genes, 0.9% | 1 |
| cation:sugar symporter activity | 11 out of 10541 genes, 0.1% | 15 out of 12397 genes, 0.1% | 1 |
| intramolecular transferase activity, phosphotransferases | 11 out of 10541 genes, 0.1% | 15 out of 12397 genes, 0.1% | 1 |
| GTP cyclohydrolase activity | 2 out of 10541 genes, 0.0% | 3 out of 12397 genes, 0.0% | 1 |
| protein kinase inhibitor activity | 2 out of 10541 genes, 0.0% | 3 out of 12397 genes, 0.0% | 1 |
| extracellular matrix structural constituent | 2 out of 10541 genes, 0.0% | 3 out of 12397 genes, 0.0% | 1 |
| microtubule binding | 2 out of 10541 genes, 0.0% | 3 out of 12397 genes, 0.0% | 1 |
| monocarboxylic acid transmembrane transporter activity | 2 out of 10541 genes, 0.0% | 3 out of 12397 genes, 0.0% | 1 |
| S-methyltransferase activity | 2 out of 10541 genes, 0.0% | 3 out of 12397 genes, 0.0% | 1 |
| fatty acid ligase activity | 2 out of 10541 genes, 0.0% | 3 out of 12397 genes, 0.0% | 1 |
| CoA hydrolase activity | 2 out of 10541 genes, 0.0% | 3 out of 12397 genes, 0.0% | 1 |
| C-palmitoyltransferase activity | 2 out of 10541 genes, 0.0% | 3 out of 12397 genes, 0.0% | 1 |
| S-succinyltransferase activity | 2 out of 10541 genes, 0.0% | 3 out of 12397 genes, 0.0% | 1 |
| oxidoreductase activity, acting on the CH-OH group of donors, quinone or similar compound as acceptor | 2 out of 10541 genes, 0.0% | 3 out of 12397 genes, 0.0% | 1 |
| snRNA binding | 2 out of 10541 genes, 0.0% | 3 out of 12397 genes, 0.0% | 1 |
| kinase inhibitor activity | 2 out of 10541 genes, 0.0% | 3 out of 12397 genes, 0.0% | 1 |
| cyclohydrolase activity | 2 out of 10541 genes, 0.0% | 3 out of 12397 genes, 0.0% | 1 |
| deoxyribonucleotide binding | 2 out of 10541 genes, 0.0% | 3 out of 12397 genes, 0.0% | 1 |
| purine deoxyribonucleotide binding | 2 out of 10541 genes, 0.0% | 3 out of 12397 genes, 0.0% | 1 |
| adenyl deoxyribonucleotide binding | 2 out of 10541 genes, 0.0% | 3 out of 12397 genes, 0.0% | 1 |
| sterol binding | 2 out of 10541 genes, 0.0% | 3 out of 12397 genes, 0.0% | 1 |
| structural constituent of cuticle | 2 out of 10541 genes, 0.0% | 3 out of 12397 genes, 0.0% | 1 |
| actinin binding | 2 out of 10541 genes, 0.0% | 3 out of 12397 genes, 0.0% | 1 |
| retinoic acid receptor binding | 2 out of 10541 genes, 0.0% | 3 out of 12397 genes, 0.0% | 1 |
| lipoprotein particle binding | 2 out of 10541 genes, 0.0% | 3 out of 12397 genes, 0.0% | 1 |
| protein-lipid complex binding | 2 out of 10541 genes, 0.0% | 3 out of 12397 genes, 0.0% | 1 |
| transcription activator activity | 7 out of 10541 genes, 0.1% | 10 out of 12397 genes, 0.1% | 1 |
| cysteine-type endopeptidase activity | 21 out of 10541 genes, 0.2% | 28 out of 12397 genes, 0.2% | 1 |
| single-stranded RNA binding | 4 out of 10541 genes, 0.0% | 6 out of 12397 genes, 0.0% | 1 |
| acyl-CoA dehydrogenase activity | 4 out of 10541 genes, 0.0% | 6 out of 12397 genes, 0.0% | 1 |
| peroxidase activity | 4 out of 10541 genes, 0.0% | 6 out of 12397 genes, 0.0% | 1 |
| phosphoglycerate mutase activity | 4 out of 10541 genes, 0.0% | 6 out of 12397 genes, 0.0% | 1 |
| cyclic nucleotide-dependent protein kinase activity | 4 out of 10541 genes, 0.0% | 6 out of 12397 genes, 0.0% | 1 |
| MAP kinase activity | 4 out of 10541 genes, 0.0% | 6 out of 12397 genes, 0.0% | 1 |
| DNA-dependent ATPase activity | 4 out of 10541 genes, 0.0% | 6 out of 12397 genes, 0.0% | 1 |
| oxidoreductase activity, acting on peroxide as acceptor | 4 out of 10541 genes, 0.0% | 6 out of 12397 genes, 0.0% | 1 |
| oxidoreductase activity, acting on CH or CH2 groups | 4 out of 10541 genes, 0.0% | 6 out of 12397 genes, 0.0% | 1 |
| oxidoreductase activity, acting on CH or CH2 groups, disulfide as acceptor | 4 out of 10541 genes, 0.0% | 6 out of 12397 genes, 0.0% | 1 |
| SAP kinase activity | 4 out of 10541 genes, 0.0% | 6 out of 12397 genes, 0.0% | 1 |
| mismatch repair complex binding | 4 out of 10541 genes, 0.0% | 6 out of 12397 genes, 0.0% | 1 |
| oxidoreductase activity, acting on CH-OH group of donors | 81 out of 10541 genes, 0.8% | 102 out of 12397 genes, 0.8% | 1 |
| DNA polymerase activity | 67 out of 10541 genes, 0.6% | 85 out of 12397 genes, 0.7% | 1 |
| NADH dehydrogenase activity | 28 out of 10541 genes, 0.3% | 37 out of 12397 genes, 0.3% | 1 |
| antioxidant activity | 35 out of 10541 genes, 0.3% | 46 out of 12397 genes, 0.4% | 1 |
| transcription coactivator activity | 6 out of 10541 genes, 0.1% | 9 out of 12397 genes, 0.1% | 1 |
| nucleotide-sugar transmembrane transporter activity | 6 out of 10541 genes, 0.1% | 9 out of 12397 genes, 0.1% | 1 |
| potassium-transporting ATPase activity | 6 out of 10541 genes, 0.1% | 9 out of 12397 genes, 0.1% | 1 |
| pyrimidine nucleotide sugar transmembrane transporter activity | 6 out of 10541 genes, 0.1% | 9 out of 12397 genes, 0.1% | 1 |
| oxidoreductase activity, acting on the CH-NH2 group of donors, NAD or NADP as acceptor | 6 out of 10541 genes, 0.1% | 9 out of 12397 genes, 0.1% | 1 |
| histone acetyltransferase activity | 3 out of 10541 genes, 0.0% | 5 out of 12397 genes, 0.0% | 1 |
| protein serine/threonine phosphatase activity | 3 out of 10541 genes, 0.0% | 5 out of 12397 genes, 0.0% | 1 |
| hydrolase activity, acting on ether bonds | 3 out of 10541 genes, 0.0% | 5 out of 12397 genes, 0.0% | 1 |
| DNA helicase activity | 45 out of 10541 genes, 0.4% | 59 out of 12397 genes, 0.5% | 1 |
| carboxylesterase activity | 29 out of 10541 genes, 0.3% | 39 out of 12397 genes, 0.3% | 1 |
| steroid binding | 8 out of 10541 genes, 0.1% | 12 out of 12397 genes, 0.1% | 1 |
| ATP-dependent helicase activity | 5 out of 10541 genes, 0.0% | 8 out of 12397 genes, 0.1% | 1 |
| purine NTP-dependent helicase activity | 5 out of 10541 genes, 0.0% | 8 out of 12397 genes, 0.1% | 1 |
| chloride channel activity | 10 out of 10541 genes, 0.1% | 15 out of 12397 genes, 0.1% | 1 |
| oxidoreductase activity, acting on a sulfur group of donors, oxygen as acceptor | 7 out of 10541 genes, 0.1% | 11 out of 12397 genes, 0.1% | 1 |
| ribonuclease activity | 26 out of 10541 genes, 0.2% | 36 out of 12397 genes, 0.3% | 1 |
| ATP-dependent DNA helicase activity | 2 out of 10541 genes, 0.0% | 4 out of 12397 genes, 0.0% | 1 |
| poly-pyrimidine tract binding | 2 out of 10541 genes, 0.0% | 4 out of 12397 genes, 0.0% | 1 |
| dynein binding | 2 out of 10541 genes, 0.0% | 4 out of 12397 genes, 0.0% | 1 |
| fatty acid binding | 12 out of 10541 genes, 0.1% | 18 out of 12397 genes, 0.1% | 1 |
| monocarboxylic acid binding | 12 out of 10541 genes, 0.1% | 18 out of 12397 genes, 0.1% | 1 |
| carbohydrate transmembrane transporter activity | 9 out of 10541 genes, 0.1% | 14 out of 12397 genes, 0.1% | 1 |
| endoribonuclease activity | 18 out of 10541 genes, 0.2% | 26 out of 12397 genes, 0.2% | 1 |
| hydrolase activity, acting on carbon-nitrogen (but not peptide) bonds, in cyclic amidines | 6 out of 10541 genes, 0.1% | 10 out of 12397 genes, 0.1% | 1 |
| endonuclease activity, active with either ribo- or deoxyribonucleic acids and producing 5'-phosphomonoesters | 16 out of 10541 genes, 0.2% | 24 out of 12397 genes, 0.2% | 1 |
| nucleotidase activity | 6 out of 10541 genes, 0.1% | 11 out of 12397 genes, 0.1% | 1 |
| cytoskeletal protein binding | 269 out of 10541 genes, 2.6% | 338 out of 12397 genes, 2.7% | 1 |
| endoribonuclease activity, producing 5'-phosphomonoesters | 13 out of 10541 genes, 0.1% | 21 out of 12397 genes, 0.2% | 1 |
| structural molecule activity | 210 out of 10541 genes, 2.0% | 267 out of 12397 genes, 2.2% | 1 |
| receptor signaling complex scaffold activity | 3 out of 10541 genes, 0.0% | 7 out of 12397 genes, 0.1% | 1 |
| protein complex scaffold | 3 out of 10541 genes, 0.0% | 7 out of 12397 genes, 0.1% | 1 |

| Gene Ontology term | Genes annotated to the term |
| --- | --- |
| transferase activity, transferring hexosyl groups | Unigene41965\_Sample\_011046840, Unigene37281\_Sample\_011046840, Unigene31035\_Sample\_011046840, Unigene34281\_Sample\_011046840, Unigene24013\_Sample\_011046840, Unigene5650\_Sample\_011046840, Unigene9318\_Sample\_011046840, Unigene26707\_Sample\_011046840, Unigene29877\_Sample\_011046840, Unigene36981\_Sample\_011046840, Unigene40158\_Sample\_011046840, Unigene37582\_Sample\_011046840, Unigene36630\_Sample\_011046840, Unigene25024\_Sample\_011046840, Unigene39931\_Sample\_011046840, Unigene5652\_Sample\_011046840, Unigene15743\_Sample\_011046840, Unigene41823\_Sample\_011046840, Unigene25822\_Sample\_011046840, Unigene42326\_Sample\_011046840, Unigene19649\_Sample\_011046840, Unigene1610\_Sample\_011046840, Unigene32997\_Sample\_011046840, Unigene40727\_Sample\_011046840, Unigene32157\_Sample\_011046840, Unigene39413\_Sample\_011046840, Unigene29121\_Sample\_011046840, Unigene12202\_Sample\_011046840, Unigene37776\_Sample\_011046840, Unigene33190\_Sample\_011046840, Unigene23401\_Sample\_011046840, Unigene43051\_Sample\_011046840, Unigene28282\_Sample\_011046840, Unigene40655\_Sample\_011046840, Unigene20494\_Sample\_011046840, Unigene31911\_Sample\_011046840, Unigene40399\_Sample\_011046840, Unigene6446\_Sample\_011046840, Unigene4514\_Sample\_011046840, Unigene39202\_Sample\_011046840, Unigene42966\_Sample\_011046840, Unigene29095\_Sample\_011046840, Unigene37738\_Sample\_011046840, Unigene36144\_Sample\_011046840, Unigene35607\_Sample\_011046840, Unigene2197\_Sample\_011046840, Unigene19832\_Sample\_011046840, Unigene88\_Sample\_011046840, Unigene37439\_Sample\_011046840, Unigene36191\_Sample\_011046840, Unigene5305\_Sample\_011046840, Unigene20602\_Sample\_011046840, Unigene16386\_Sample\_011046840, Unigene42761\_Sample\_011046840, Unigene599\_Sample\_011046840, Unigene6287\_Sample\_011046840, Unigene34227\_Sample\_011046840, Unigene7352\_Sample\_011046840, Unigene42867\_Sample\_011046840, Unigene10638\_Sample\_011046840, Unigene39622\_Sample\_011046840, Unigene5388\_Sample\_011046840, Unigene37223\_Sample\_011046840, Unigene29655\_Sample\_011046840, Unigene32209\_Sample\_011046840, Unigene36547\_Sample\_011046840, Unigene34023\_Sample\_011046840, Unigene5415\_Sample\_011046840, Unigene15667\_Sample\_011046840, Unigene42202\_Sample\_011046840, Unigene31891\_Sample\_011046840, Unigene34200\_Sample\_011046840, Unigene18248\_Sample\_011046840, Unigene14077\_Sample\_011046840, Unigene16941\_Sample\_011046840, Unigene34638\_Sample\_011046840, Unigene31582\_Sample\_011046840, Unigene36463\_Sample\_011046840, Unigene12570\_Sample\_011046840, Unigene3237\_Sample\_011046840, Unigene29264\_Sample\_011046840, Unigene1557\_Sample\_011046840, Unigene30575\_Sample\_011046840, Unigene39953\_Sample\_011046840, Unigene27112\_Sample\_011046840, Unigene42094\_Sample\_011046840, Unigene40090\_Sample\_011046840, Unigene37669\_Sample\_011046840, Unigene41448\_Sample\_011046840, Unigene43482\_Sample\_011046840, Unigene40362\_Sample\_011046840, Unigene5278\_Sample\_011046840, Unigene39713\_Sample\_011046840, Unigene12126\_Sample\_011046840, Unigene39751\_Sample\_011046840, Unigene37790\_Sample\_011046840, Unigene39873\_Sample\_011046840, Unigene25584\_Sample\_011046840, Unigene4573\_Sample\_011046840, Unigene36251\_Sample\_011046840, Unigene43404\_Sample\_011046840, Unigene29367\_Sample\_011046840, Unigene42250\_Sample\_011046840, Unigene41663\_Sample\_011046840, Unigene7037\_Sample\_011046840, Unigene13209\_Sample\_011046840, Unigene3492\_Sample\_011046840, Unigene11976\_Sample\_011046840, Unigene41379\_Sample\_011046840, Unigene42552\_Sample\_011046840, Unigene40228\_Sample\_011046840, Unigene700\_Sample\_011046840, Unigene38573\_Sample\_011046840, Unigene38495\_Sample\_011046840, Unigene7141\_Sample\_011046840, Unigene32902\_Sample\_011046840, Unigene22626\_Sample\_011046840, Unigene5257\_Sample\_011046840, Unigene30051\_Sample\_011046840, Unigene34254\_Sample\_011046840, Unigene43464\_Sample\_011046840, Unigene36232\_Sample\_011046840, Unigene37398\_Sample\_011046840 |
| phosphoprotein phosphatase activity | Unigene30791\_Sample\_011046840, Unigene10803\_Sample\_011046840, Unigene29269\_Sample\_011046840, Unigene43013\_Sample\_011046840, Unigene1544\_Sample\_011046840, Unigene42869\_Sample\_011046840, Unigene42159\_Sample\_011046840, Unigene37304\_Sample\_011046840, Unigene14438\_Sample\_011046840, Unigene7132\_Sample\_011046840, Unigene24223\_Sample\_011046840, Unigene42074\_Sample\_011046840, Unigene3108\_Sample\_011046840, Unigene32488\_Sample\_011046840, Unigene37121\_Sample\_011046840, Unigene39315\_Sample\_011046840, Unigene33191\_Sample\_011046840, Unigene30604\_Sample\_011046840, Unigene37146\_Sample\_011046840, Unigene43355\_Sample\_011046840, Unigene39096\_Sample\_011046840, Unigene3682\_Sample\_011046840, Unigene26409\_Sample\_011046840, Unigene42607\_Sample\_011046840, Unigene29993\_Sample\_011046840, Unigene39685\_Sample\_011046840, Unigene10748\_Sample\_011046840, Unigene17640\_Sample\_011046840, Unigene1019\_Sample\_011046840, Unigene12436\_Sample\_011046840, Unigene2209\_Sample\_011046840, Unigene6735\_Sample\_011046840, Unigene29095\_Sample\_011046840, Unigene3151\_Sample\_011046840, Unigene42573\_Sample\_011046840, Unigene39006\_Sample\_011046840, Unigene6139\_Sample\_011046840, Unigene29586\_Sample\_011046840, Unigene3009\_Sample\_011046840, Unigene41003\_Sample\_011046840, Unigene43138\_Sample\_011046840, Unigene13576\_Sample\_011046840, Unigene35122\_Sample\_011046840, Unigene23528\_Sample\_011046840, Unigene29655\_Sample\_011046840, Unigene32138\_Sample\_011046840, Unigene41224\_Sample\_011046840, Unigene41918\_Sample\_011046840, Unigene14077\_Sample\_011046840, Unigene17482\_Sample\_011046840, Unigene35596\_Sample\_011046840, Unigene37235\_Sample\_011046840, Unigene43202\_Sample\_011046840, Unigene36688\_Sample\_011046840, Unigene14255\_Sample\_011046840, Unigene24618\_Sample\_011046840, Unigene40672\_Sample\_011046840, Unigene36910\_Sample\_011046840, Unigene31343\_Sample\_011046840, Unigene39237\_Sample\_011046840, Unigene13531\_Sample\_011046840, Unigene16605\_Sample\_011046840, Unigene42850\_Sample\_011046840, Unigene34076\_Sample\_011046840, Unigene7387\_Sample\_011046840, Unigene35213\_Sample\_011046840, Unigene36980\_Sample\_011046840, Unigene41617\_Sample\_011046840, Unigene2690\_Sample\_011046840, Unigene29521\_Sample\_011046840, Unigene38799\_Sample\_011046840, Unigene17084\_Sample\_011046840, Unigene39711\_Sample\_011046840, Unigene6373\_Sample\_011046840, Unigene42477\_Sample\_011046840, Unigene42149\_Sample\_011046840, Unigene27270\_Sample\_011046840, Unigene43417\_Sample\_011046840, Unigene6516\_Sample\_011046840, Unigene23875\_Sample\_011046840, Unigene40736\_Sample\_011046840, Unigene8055\_Sample\_011046840, Unigene41248\_Sample\_011046840, Unigene36503\_Sample\_011046840, Unigene21908\_Sample\_011046840, Unigene25217\_Sample\_011046840, Unigene16532\_Sample\_011046840, Unigene7523\_Sample\_011046840, Unigene16807\_Sample\_011046840, Unigene2718\_Sample\_011046840, Unigene35403\_Sample\_011046840, Unigene39384\_Sample\_011046840, Unigene28124\_Sample\_011046840, Unigene8156\_Sample\_011046840, Unigene37582\_Sample\_011046840, Unigene32610\_Sample\_011046840, Unigene40454\_Sample\_011046840, Unigene15205\_Sample\_011046840, Unigene29775\_Sample\_011046840, Unigene42728\_Sample\_011046840, Unigene34085\_Sample\_011046840, Unigene37248\_Sample\_011046840, Unigene1740\_Sample\_011046840, Unigene21914\_Sample\_011046840, Unigene13146\_Sample\_011046840, Unigene35942\_Sample\_011046840, Unigene25448\_Sample\_011046840, Unigene33579\_Sample\_011046840, Unigene38340\_Sample\_011046840, Unigene36137\_Sample\_011046840, Unigene30384\_Sample\_011046840, Unigene41420\_Sample\_011046840, Unigene13348\_Sample\_011046840, Unigene7974\_Sample\_011046840, Unigene40139\_Sample\_011046840, Unigene13067\_Sample\_011046840, Unigene38621\_Sample\_011046840, Unigene39188\_Sample\_011046840, Unigene7868\_Sample\_011046840, Unigene6088\_Sample\_011046840, Unigene15250\_Sample\_011046840, Unigene34967\_Sample\_011046840, Unigene1843\_Sample\_011046840, Unigene39291\_Sample\_011046840, Unigene42709\_Sample\_011046840, Unigene36662\_Sample\_011046840, Unigene13908\_Sample\_011046840, Unigene12710\_Sample\_011046840, Unigene10992\_Sample\_011046840, Unigene20174\_Sample\_011046840, Unigene25761\_Sample\_011046840, Unigene33865\_Sample\_011046840, Unigene2123\_Sample\_011046840, Unigene18059\_Sample\_011046840, Unigene41425\_Sample\_011046840, Unigene7408\_Sample\_011046840, Unigene42231\_Sample\_011046840, Unigene2295\_Sample\_011046840, Unigene34638\_Sample\_011046840, Unigene41204\_Sample\_011046840, Unigene36519\_Sample\_011046840, Unigene37605\_Sample\_011046840, Unigene33594\_Sample\_011046840, Unigene21311\_Sample\_011046840, Unigene1602\_Sample\_011046840, Unigene5294\_Sample\_011046840, Unigene26887\_Sample\_011046840, Unigene40146\_Sample\_011046840, Unigene20473\_Sample\_011046840, Unigene11615\_Sample\_011046840, Unigene37697\_Sample\_011046840, Unigene23356\_Sample\_011046840, Unigene41853\_Sample\_011046840, Unigene25603\_Sample\_011046840, Unigene38362\_Sample\_011046840, Unigene7144\_Sample\_011046840, Unigene39738\_Sample\_011046840, Unigene32001\_Sample\_011046840, Unigene12229\_Sample\_011046840, Unigene23373\_Sample\_011046840, Unigene4192\_Sample\_011046840, Unigene41024\_Sample\_011046840, Unigene4069\_Sample\_011046840, Unigene41388\_Sample\_011046840, Unigene33401\_Sample\_011046840, Unigene41059\_Sample\_011046840, Unigene37310\_Sample\_011046840, Unigene35687\_Sample\_011046840, Unigene1967\_Sample\_011046840 |
| active transmembrane transporter activity | Unigene32100\_Sample\_011046840, Unigene41332\_Sample\_011046840, Unigene32354\_Sample\_011046840, Unigene15192\_Sample\_011046840, Unigene17267\_Sample\_011046840, Unigene31662\_Sample\_011046840, Unigene39248\_Sample\_011046840, Unigene9443\_Sample\_011046840, Unigene5760\_Sample\_011046840, Unigene36088\_Sample\_011046840, Unigene41047\_Sample\_011046840, Unigene39204\_Sample\_011046840, Unigene8113\_Sample\_011046840, Unigene27519\_Sample\_011046840, Unigene35008\_Sample\_011046840, Unigene36965\_Sample\_011046840, Unigene14850\_Sample\_011046840, Unigene5901\_Sample\_011046840, Unigene14473\_Sample\_011046840, Unigene32197\_Sample\_011046840, Unigene12721\_Sample\_011046840, Unigene6313\_Sample\_011046840, Unigene17101\_Sample\_011046840, Unigene7477\_Sample\_011046840, Unigene40513\_Sample\_011046840, Unigene37745\_Sample\_011046840, Unigene15030\_Sample\_011046840, Unigene39499\_Sample\_011046840, Unigene34914\_Sample\_011046840, Unigene24674\_Sample\_011046840, Unigene38314\_Sample\_011046840, Unigene32614\_Sample\_011046840, Unigene26668\_Sample\_011046840, Unigene14116\_Sample\_011046840, Unigene40184\_Sample\_011046840, Unigene3892\_Sample\_011046840, Unigene41763\_Sample\_011046840, Unigene37928\_Sample\_011046840, Unigene41972\_Sample\_011046840, Unigene23663\_Sample\_011046840, Unigene34978\_Sample\_011046840, Unigene10979\_Sample\_011046840, Unigene2277\_Sample\_011046840, Unigene32158\_Sample\_011046840, Unigene39490\_Sample\_011046840, Unigene39632\_Sample\_011046840, Unigene42337\_Sample\_011046840, Unigene37068\_Sample\_011046840, Unigene14012\_Sample\_011046840, Unigene30270\_Sample\_011046840, Unigene31515\_Sample\_011046840, Unigene38720\_Sample\_011046840, Unigene31864\_Sample\_011046840, Unigene1053\_Sample\_011046840, Unigene17721\_Sample\_011046840, Unigene5219\_Sample\_011046840, Unigene39351\_Sample\_011046840, Unigene9682\_Sample\_011046840, Unigene26581\_Sample\_011046840, Unigene25781\_Sample\_011046840, Unigene43123\_Sample\_011046840, Unigene2152\_Sample\_011046840, Unigene36150\_Sample\_011046840, Unigene41683\_Sample\_011046840, Unigene11195\_Sample\_011046840, Unigene25\_Sample\_011046840, Unigene4089\_Sample\_011046840, Unigene39923\_Sample\_011046840, Unigene28467\_Sample\_011046840, Unigene38677\_Sample\_011046840, Unigene8051\_Sample\_011046840, Unigene34804\_Sample\_011046840, Unigene1742\_Sample\_011046840, Unigene37173\_Sample\_011046840, Unigene5914\_Sample\_011046840, Unigene12675\_Sample\_011046840, Unigene4481\_Sample\_011046840, Unigene41720\_Sample\_011046840, Unigene41628\_Sample\_011046840, Unigene29053\_Sample\_011046840, Unigene19865\_Sample\_011046840, Unigene29805\_Sample\_011046840, Unigene38472\_Sample\_011046840, Unigene7994\_Sample\_011046840, Unigene15903\_Sample\_011046840, Unigene29794\_Sample\_011046840, Unigene37786\_Sample\_011046840, Unigene6241\_Sample\_011046840, Unigene39273\_Sample\_011046840, Unigene30535\_Sample\_011046840, Unigene38609\_Sample\_011046840, Unigene8803\_Sample\_011046840, Unigene4518\_Sample\_011046840, Unigene33342\_Sample\_011046840, Unigene35976\_Sample\_011046840, Unigene26156\_Sample\_011046840, Unigene34506\_Sample\_011046840, Unigene12084\_Sample\_011046840, Unigene35594\_Sample\_011046840, Unigene10704\_Sample\_011046840, Unigene28857\_Sample\_011046840, Unigene3599\_Sample\_011046840, Unigene42769\_Sample\_011046840, Unigene38900\_Sample\_011046840, Unigene22068\_Sample\_011046840, Unigene27997\_Sample\_011046840, Unigene3791\_Sample\_011046840, Unigene34972\_Sample\_011046840, Unigene39755\_Sample\_011046840, Unigene28867\_Sample\_011046840, Unigene43573\_Sample\_011046840, Unigene25956\_Sample\_011046840, Unigene35951\_Sample\_011046840, Unigene15680\_Sample\_011046840, Unigene41788\_Sample\_011046840, Unigene20940\_Sample\_011046840, Unigene12410\_Sample\_011046840, Unigene40032\_Sample\_011046840, Unigene39608\_Sample\_011046840, Unigene7302\_Sample\_011046840, Unigene37979\_Sample\_011046840, Unigene32702\_Sample\_011046840, Unigene39249\_Sample\_011046840, Unigene32585\_Sample\_011046840, Unigene23158\_Sample\_011046840, Unigene43475\_Sample\_011046840, Unigene20758\_Sample\_011046840, Unigene21338\_Sample\_011046840, Unigene2822\_Sample\_011046840, Unigene14501\_Sample\_011046840, Unigene24937\_Sample\_011046840, Unigene15594\_Sample\_011046840, Unigene41582\_Sample\_011046840, Unigene30629\_Sample\_011046840, Unigene16244\_Sample\_011046840, Unigene6659\_Sample\_011046840, Unigene24801\_Sample\_011046840, Unigene36345\_Sample\_011046840, Unigene36141\_Sample\_011046840, Unigene31215\_Sample\_011046840, Unigene34105\_Sample\_011046840, Unigene16545\_Sample\_011046840, Unigene25046\_Sample\_011046840, Unigene39874\_Sample\_011046840, Unigene25612\_Sample\_011046840, Unigene30572\_Sample\_011046840, Unigene41653\_Sample\_011046840, Unigene35554\_Sample\_011046840, Unigene36371\_Sample\_011046840, Unigene1879\_Sample\_011046840, Unigene19647\_Sample\_011046840, Unigene32733\_Sample\_011046840, Unigene37353\_Sample\_011046840, Unigene22258\_Sample\_011046840, Unigene9893\_Sample\_011046840, Unigene37075\_Sample\_011046840, Unigene23727\_Sample\_011046840, Unigene41498\_Sample\_011046840, Unigene41669\_Sample\_011046840, Unigene31318\_Sample\_011046840, Unigene25769\_Sample\_011046840, Unigene38279\_Sample\_011046840, Unigene30451\_Sample\_011046840, Unigene33443\_Sample\_011046840, Unigene37336\_Sample\_011046840, Unigene5591\_Sample\_011046840, Unigene24034\_Sample\_011046840, Unigene34400\_Sample\_011046840, Unigene13\_Sample\_011046840, Unigene29386\_Sample\_011046840, Unigene36129\_Sample\_011046840, Unigene40445\_Sample\_011046840, Unigene6383\_Sample\_011046840, Unigene19704\_Sample\_011046840, Unigene5894\_Sample\_011046840, Unigene43001\_Sample\_011046840, Unigene27829\_Sample\_011046840, Unigene12033\_Sample\_011046840, Unigene43546\_Sample\_011046840, Unigene25408\_Sample\_011046840, Unigene43073\_Sample\_011046840, Unigene34358\_Sample\_011046840, Unigene42177\_Sample\_011046840, Unigene22824\_Sample\_011046840, Unigene36000\_Sample\_011046840, Unigene38028\_Sample\_011046840, Unigene37921\_Sample\_011046840, Unigene13862\_Sample\_011046840, Unigene31447\_Sample\_011046840, Unigene257\_Sample\_011046840, Unigene8019\_Sample\_011046840, Unigene42838\_Sample\_011046840, Unigene15362\_Sample\_011046840, Unigene9477\_Sample\_011046840, Unigene40428\_Sample\_011046840, Unigene7931\_Sample\_011046840, Unigene38147\_Sample\_011046840, Unigene40391\_Sample\_011046840, Unigene18919\_Sample\_011046840, Unigene33192\_Sample\_011046840, Unigene37868\_Sample\_011046840, Unigene41898\_Sample\_011046840, Unigene11142\_Sample\_011046840, Unigene7225\_Sample\_011046840, Unigene37347\_Sample\_011046840, Unigene43599\_Sample\_011046840, Unigene13797\_Sample\_011046840, Unigene4389\_Sample\_011046840, Unigene4500\_Sample\_011046840, Unigene28685\_Sample\_011046840, Unigene4327\_Sample\_011046840, Unigene2237\_Sample\_011046840, Unigene27257\_Sample\_011046840, Unigene35535\_Sample\_011046840, Unigene42621\_Sample\_011046840, Unigene35872\_Sample\_011046840, Unigene36260\_Sample\_011046840, Unigene14178\_Sample\_011046840, Unigene2310\_Sample\_011046840, Unigene31273\_Sample\_011046840, Unigene7606\_Sample\_011046840, Unigene32706\_Sample\_011046840, Unigene38081\_Sample\_011046840, Unigene12542\_Sample\_011046840, Unigene23213\_Sample\_011046840, Unigene2457\_Sample\_011046840, Unigene36762\_Sample\_011046840, Unigene32819\_Sample\_011046840, Unigene40687\_Sample\_011046840, Unigene7422\_Sample\_011046840, Unigene6547\_Sample\_011046840, Unigene39837\_Sample\_011046840, Unigene40205\_Sample\_011046840, Unigene26216\_Sample\_011046840, Unigene33823\_Sample\_011046840, Unigene29631\_Sample\_011046840 |
| phospholipid binding | Unigene7724\_Sample\_011046840, Unigene42722\_Sample\_011046840, Unigene39318\_Sample\_011046840, Unigene37087\_Sample\_011046840, Unigene3392\_Sample\_011046840, Unigene37026\_Sample\_011046840, Unigene34297\_Sample\_011046840, Unigene42774\_Sample\_011046840, Unigene18553\_Sample\_011046840, Unigene41652\_Sample\_011046840, Unigene42297\_Sample\_011046840, Unigene11494\_Sample\_011046840, Unigene28339\_Sample\_011046840, Unigene39076\_Sample\_011046840, Unigene32857\_Sample\_011046840, Unigene2822\_Sample\_011046840, Unigene8205\_Sample\_011046840, Unigene43615\_Sample\_011046840, Unigene13511\_Sample\_011046840, Unigene5714\_Sample\_011046840, Unigene31506\_Sample\_011046840, Unigene42892\_Sample\_011046840, Unigene23285\_Sample\_011046840, Unigene23607\_Sample\_011046840, Unigene34854\_Sample\_011046840, Unigene35477\_Sample\_011046840, Unigene40284\_Sample\_011046840, Unigene30267\_Sample\_011046840, Unigene25105\_Sample\_011046840, Unigene40399\_Sample\_011046840, Unigene19933\_Sample\_011046840, Unigene12541\_Sample\_011046840, Unigene26124\_Sample\_011046840, Unigene5271\_Sample\_011046840, Unigene42810\_Sample\_011046840, Unigene4147\_Sample\_011046840, Unigene37048\_Sample\_011046840, Unigene38322\_Sample\_011046840, Unigene5390\_Sample\_011046840, Unigene5388\_Sample\_011046840, Unigene42754\_Sample\_011046840, Unigene16314\_Sample\_011046840, Unigene6022\_Sample\_011046840, Unigene41520\_Sample\_011046840, Unigene30947\_Sample\_011046840, Unigene4279\_Sample\_011046840, Unigene37194\_Sample\_011046840, Unigene26320\_Sample\_011046840, Unigene8089\_Sample\_011046840, Unigene43499\_Sample\_011046840, Unigene37451\_Sample\_011046840, Unigene35561\_Sample\_011046840, Unigene35723\_Sample\_011046840, Unigene1756\_Sample\_011046840, Unigene5489\_Sample\_011046840, Unigene29538\_Sample\_011046840, Unigene41439\_Sample\_011046840, Unigene5969\_Sample\_011046840, Unigene21061\_Sample\_011046840, Unigene32547\_Sample\_011046840, Unigene19411\_Sample\_011046840, Unigene29255\_Sample\_011046840, Unigene1797\_Sample\_011046840, Unigene18382\_Sample\_011046840, Unigene35475\_Sample\_011046840, Unigene25695\_Sample\_011046840 |
| oxidoreductase activity, acting on paired donors, with incorporation or reduction of molecular oxygen, 2-oxoglutarate as one donor, and incorporation of one atom each of oxygen into both donors | Unigene43051\_Sample\_011046840, Unigene4595\_Sample\_011046840, Unigene40087\_Sample\_011046840, Unigene20821\_Sample\_011046840, Unigene42250\_Sample\_011046840, Unigene1619\_Sample\_011046840, Unigene3068\_Sample\_011046840, Unigene42972\_Sample\_011046840, Unigene23870\_Sample\_011046840, Unigene36713\_Sample\_011046840, Unigene13285\_Sample\_011046840, Unigene21804\_Sample\_011046840, Unigene43488\_Sample\_011046840, Unigene5876\_Sample\_011046840, Unigene41821\_Sample\_011046840, Unigene11684\_Sample\_011046840, Unigene30495\_Sample\_011046840, Unigene14590\_Sample\_011046840, Unigene30757\_Sample\_011046840, Unigene33029\_Sample\_011046840, Unigene35564\_Sample\_011046840, Unigene3029\_Sample\_011046840, Unigene33508\_Sample\_011046840, Unigene32005\_Sample\_011046840, Unigene31901\_Sample\_011046840, Unigene41448\_Sample\_011046840, Unigene32895\_Sample\_011046840, Unigene42557\_Sample\_011046840, Unigene41977\_Sample\_011046840, Unigene4570\_Sample\_011046840, Unigene7134\_Sample\_011046840 |
| amine transmembrane transporter activity | Unigene43123\_Sample\_011046840, Unigene32100\_Sample\_011046840, Unigene41683\_Sample\_011046840, Unigene41332\_Sample\_011046840, Unigene43599\_Sample\_011046840, Unigene37336\_Sample\_011046840, Unigene29386\_Sample\_011046840, Unigene23663\_Sample\_011046840, Unigene10979\_Sample\_011046840, Unigene5894\_Sample\_011046840, Unigene6383\_Sample\_011046840, Unigene41653\_Sample\_011046840, Unigene20940\_Sample\_011046840, Unigene1742\_Sample\_011046840, Unigene43073\_Sample\_011046840, Unigene27519\_Sample\_011046840, Unigene22824\_Sample\_011046840, Unigene35594\_Sample\_011046840, Unigene4481\_Sample\_011046840, Unigene38081\_Sample\_011046840, Unigene13862\_Sample\_011046840, Unigene8019\_Sample\_011046840, Unigene42838\_Sample\_011046840, Unigene39249\_Sample\_011046840, Unigene38147\_Sample\_011046840, Unigene28857\_Sample\_011046840, Unigene37075\_Sample\_011046840, Unigene21338\_Sample\_011046840, Unigene24937\_Sample\_011046840, Unigene38314\_Sample\_011046840 |
| vitamin binding | Unigene17098\_Sample\_011046840, Unigene42972\_Sample\_011046840, Unigene42131\_Sample\_011046840, Unigene36713\_Sample\_011046840, Unigene13285\_Sample\_011046840, Unigene40950\_Sample\_011046840, Unigene5876\_Sample\_011046840, Unigene40235\_Sample\_011046840, Unigene11684\_Sample\_011046840, Unigene14590\_Sample\_011046840, Unigene40724\_Sample\_011046840, Unigene3029\_Sample\_011046840, Unigene32005\_Sample\_011046840, Unigene13847\_Sample\_011046840, Unigene21529\_Sample\_011046840, Unigene40899\_Sample\_011046840, Unigene22482\_Sample\_011046840, Unigene31901\_Sample\_011046840, Unigene32256\_Sample\_011046840, Unigene41448\_Sample\_011046840, Unigene17329\_Sample\_011046840, Unigene33711\_Sample\_011046840, Unigene32895\_Sample\_011046840, Unigene41174\_Sample\_011046840, Unigene20757\_Sample\_011046840, Unigene32307\_Sample\_011046840, Unigene43051\_Sample\_011046840, Unigene42278\_Sample\_011046840, Unigene29743\_Sample\_011046840, Unigene32790\_Sample\_011046840, Unigene40234\_Sample\_011046840, Unigene17816\_Sample\_011046840, Unigene41239\_Sample\_011046840, Unigene26767\_Sample\_011046840, Unigene36942\_Sample\_011046840, Unigene16153\_Sample\_011046840, Unigene13335\_Sample\_011046840, Unigene40703\_Sample\_011046840, Unigene15010\_Sample\_011046840 |
| nucleoside binding | Unigene38023\_Sample\_011046840, Unigene26178\_Sample\_011046840, Unigene39461\_Sample\_011046840, Unigene20405\_Sample\_011046840, Unigene38706\_Sample\_011046840, Unigene9443\_Sample\_011046840, Unigene368\_Sample\_011046840, Unigene3429\_Sample\_011046840, Unigene37568\_Sample\_011046840, Unigene19401\_Sample\_011046840, Unigene41047\_Sample\_011046840, Unigene18730\_Sample\_011046840, Unigene14932\_Sample\_011046840, Unigene43154\_Sample\_011046840, Unigene902\_Sample\_011046840, Unigene43529\_Sample\_011046840, Unigene27378\_Sample\_011046840, Unigene22180\_Sample\_011046840, Unigene43365\_Sample\_011046840, Unigene20380\_Sample\_011046840, Unigene21224\_Sample\_011046840, Unigene27537\_Sample\_011046840, Unigene7557\_Sample\_011046840, Unigene42372\_Sample\_011046840, Unigene31094\_Sample\_011046840, Unigene41153\_Sample\_011046840, Unigene20314\_Sample\_011046840, Unigene32455\_Sample\_011046840, Unigene15132\_Sample\_011046840, Unigene42642\_Sample\_011046840, Unigene35962\_Sample\_011046840, Unigene4259\_Sample\_011046840, Unigene4677\_Sample\_011046840, Unigene36928\_Sample\_011046840, Unigene24531\_Sample\_011046840, Unigene32335\_Sample\_011046840, Unigene42401\_Sample\_011046840, Unigene25485\_Sample\_011046840, Unigene6748\_Sample\_011046840, Unigene34978\_Sample\_011046840, Unigene8081\_Sample\_011046840, Unigene34971\_Sample\_011046840, Unigene37185\_Sample\_011046840, Unigene30370\_Sample\_011046840, Unigene32811\_Sample\_011046840, Unigene12614\_Sample\_011046840, Unigene1458\_Sample\_011046840, Unigene17494\_Sample\_011046840, Unigene21883\_Sample\_011046840, Unigene37375\_Sample\_011046840, Unigene8178\_Sample\_011046840, Unigene36909\_Sample\_011046840, Unigene1132\_Sample\_011046840, Unigene21176\_Sample\_011046840, Unigene25680\_Sample\_011046840, Unigene38973\_Sample\_011046840, Unigene7529\_Sample\_011046840, Unigene22101\_Sample\_011046840, Unigene40181\_Sample\_011046840, Unigene39544\_Sample\_011046840, Unigene3849\_Sample\_011046840, Unigene38463\_Sample\_011046840, Unigene177\_Sample\_011046840, Unigene41833\_Sample\_011046840, Unigene4089\_Sample\_011046840, Unigene14294\_Sample\_011046840, Unigene30285\_Sample\_011046840, Unigene36764\_Sample\_011046840, Unigene37848\_Sample\_011046840, Unigene14393\_Sample\_011046840, Unigene5726\_Sample\_011046840, Unigene38504\_Sample\_011046840, Unigene36848\_Sample\_011046840, Unigene19725\_Sample\_011046840, Unigene37095\_Sample\_011046840, Unigene30893\_Sample\_011046840, Unigene36359\_Sample\_011046840, Unigene3319\_Sample\_011046840, Unigene13306\_Sample\_011046840, Unigene24816\_Sample\_011046840, Unigene20757\_Sample\_011046840, Unigene24103\_Sample\_011046840, Unigene33428\_Sample\_011046840, Unigene24254\_Sample\_011046840, Unigene7831\_Sample\_011046840, Unigene30203\_Sample\_011046840, Unigene893\_Sample\_011046840, Unigene43435\_Sample\_011046840, Unigene30471\_Sample\_011046840, Unigene40159\_Sample\_011046840, Unigene29784\_Sample\_011046840, Unigene39812\_Sample\_011046840, Unigene27998\_Sample\_011046840, Unigene19834\_Sample\_011046840, Unigene42253\_Sample\_011046840, Unigene29947\_Sample\_011046840, Unigene17955\_Sample\_011046840, Unigene34234\_Sample\_011046840, Unigene25555\_Sample\_011046840, Unigene42502\_Sample\_011046840, Unigene41104\_Sample\_011046840, Unigene11113\_Sample\_011046840, Unigene41150\_Sample\_011046840, Unigene4830\_Sample\_011046840, Unigene41496\_Sample\_011046840, Unigene16527\_Sample\_011046840, Unigene43077\_Sample\_011046840, Unigene40073\_Sample\_011046840, Unigene37822\_Sample\_011046840, Unigene9565\_Sample\_011046840, Unigene12101\_Sample\_011046840, Unigene5249\_Sample\_011046840, Unigene31599\_Sample\_011046840, Unigene22020\_Sample\_011046840, Unigene16161\_Sample\_011046840, Unigene5118\_Sample\_011046840, Unigene35603\_Sample\_011046840, Unigene16645\_Sample\_011046840, Unigene32978\_Sample\_011046840, Unigene42351\_Sample\_011046840, Unigene33208\_Sample\_011046840, Unigene36893\_Sample\_011046840, Unigene36637\_Sample\_011046840, Unigene30615\_Sample\_011046840, Unigene39662\_Sample\_011046840, Unigene18599\_Sample\_011046840, Unigene29270\_Sample\_011046840, Unigene13215\_Sample\_011046840, Unigene4578\_Sample\_011046840, Unigene15884\_Sample\_011046840, Unigene43230\_Sample\_011046840, Unigene42704\_Sample\_011046840, Unigene40406\_Sample\_011046840, Unigene37855\_Sample\_011046840, Unigene42242\_Sample\_011046840, Unigene20758\_Sample\_011046840, Unigene39911\_Sample\_011046840, Unigene36345\_Sample\_011046840, Unigene31215\_Sample\_011046840, Unigene41932\_Sample\_011046840, Unigene34105\_Sample\_011046840, Unigene20100\_Sample\_011046840, Unigene5001\_Sample\_011046840, Unigene39763\_Sample\_011046840, Unigene8801\_Sample\_011046840, Unigene37863\_Sample\_011046840, Unigene39780\_Sample\_011046840, Unigene8554\_Sample\_011046840, Unigene39218\_Sample\_011046840, Unigene41866\_Sample\_011046840, Unigene39982\_Sample\_011046840, Unigene20594\_Sample\_011046840, Unigene43537\_Sample\_011046840, Unigene20\_Sample\_011046840, Unigene33968\_Sample\_011046840, Unigene30239\_Sample\_011046840, Unigene37613\_Sample\_011046840, Unigene32733\_Sample\_011046840, Unigene37902\_Sample\_011046840, Unigene3770\_Sample\_011046840, Unigene1222\_Sample\_011046840, Unigene17734\_Sample\_011046840, Unigene38066\_Sample\_011046840, Unigene38847\_Sample\_011046840, Unigene26711\_Sample\_011046840, Unigene38443\_Sample\_011046840, Unigene18659\_Sample\_011046840, Unigene32337\_Sample\_011046840, Unigene20968\_Sample\_011046840, Unigene29802\_Sample\_011046840, Unigene42061\_Sample\_011046840, Unigene33983\_Sample\_011046840, Unigene6796\_Sample\_011046840, Unigene20491\_Sample\_011046840, Unigene35734\_Sample\_011046840, Unigene4994\_Sample\_011046840, Unigene33913\_Sample\_011046840, Unigene41476\_Sample\_011046840, Unigene13127\_Sample\_011046840, Unigene41389\_Sample\_011046840, Unigene4309\_Sample\_011046840, Unigene36619\_Sample\_011046840, Unigene16211\_Sample\_011046840, Unigene38206\_Sample\_011046840, Unigene9477\_Sample\_011046840, Unigene43332\_Sample\_011046840, Unigene41743\_Sample\_011046840, Unigene29953\_Sample\_011046840, Unigene38834\_Sample\_011046840, Unigene20493\_Sample\_011046840, Unigene32099\_Sample\_011046840, Unigene41898\_Sample\_011046840, Unigene37706\_Sample\_011046840, Unigene31090\_Sample\_011046840, Unigene3003\_Sample\_011046840, Unigene42374\_Sample\_011046840, Unigene39024\_Sample\_011046840, Unigene15465\_Sample\_011046840, Unigene4389\_Sample\_011046840, Unigene41997\_Sample\_011046840, Unigene38687\_Sample\_011046840, Unigene5116\_Sample\_011046840, Unigene40471\_Sample\_011046840, Unigene42605\_Sample\_011046840, Unigene41161\_Sample\_011046840, Unigene34519\_Sample\_011046840, Unigene33595\_Sample\_011046840, Unigene31273\_Sample\_011046840, Unigene29985\_Sample\_011046840, Unigene907\_Sample\_011046840, Unigene33768\_Sample\_011046840, Unigene10236\_Sample\_011046840, Unigene522\_Sample\_011046840, Unigene36897\_Sample\_011046840, Unigene29903\_Sample\_011046840, Unigene29839\_Sample\_011046840, Unigene2457\_Sample\_011046840, Unigene26813\_Sample\_011046840, Unigene6547\_Sample\_011046840, Unigene26216\_Sample\_011046840, Unigene34870\_Sample\_011046840, Unigene35061\_Sample\_011046840, Unigene279\_Sample\_011046840, Unigene42759\_Sample\_011046840, Unigene2459\_Sample\_011046840, Unigene36366\_Sample\_011046840, Unigene19628\_Sample\_011046840, Unigene38962\_Sample\_011046840, Unigene35913\_Sample\_011046840, Unigene34002\_Sample\_011046840, Unigene40216\_Sample\_011046840, Unigene41431\_Sample\_011046840, Unigene42859\_Sample\_011046840, Unigene30600\_Sample\_011046840, Unigene29629\_Sample\_011046840, Unigene43055\_Sample\_011046840, Unigene23556\_Sample\_011046840, Unigene15492\_Sample\_011046840, Unigene5087\_Sample\_011046840, Unigene20079\_Sample\_011046840, Unigene32197\_Sample\_011046840, Unigene6313\_Sample\_011046840, Unigene31572\_Sample\_011046840, Unigene27920\_Sample\_011046840, Unigene9002\_Sample\_011046840, Unigene39499\_Sample\_011046840, Unigene14420\_Sample\_011046840, Unigene39061\_Sample\_011046840, Unigene27158\_Sample\_011046840, Unigene24281\_Sample\_011046840, Unigene38441\_Sample\_011046840, Unigene10533\_Sample\_011046840, Unigene37904\_Sample\_011046840, Unigene43606\_Sample\_011046840, Unigene34854\_Sample\_011046840, Unigene37976\_Sample\_011046840, Unigene42547\_Sample\_011046840, Unigene10832\_Sample\_011046840, Unigene15502\_Sample\_011046840, Unigene2792\_Sample\_011046840, Unigene37545\_Sample\_011046840, Unigene41919\_Sample\_011046840, Unigene4779\_Sample\_011046840, Unigene10749\_Sample\_011046840, Unigene25548\_Sample\_011046840, Unigene14498\_Sample\_011046840, Unigene39972\_Sample\_011046840, Unigene41368\_Sample\_011046840, Unigene5372\_Sample\_011046840, Unigene38709\_Sample\_011046840, Unigene38720\_Sample\_011046840, Unigene31864\_Sample\_011046840, Unigene37214\_Sample\_011046840, Unigene43188\_Sample\_011046840, Unigene39660\_Sample\_011046840, Unigene36990\_Sample\_011046840, Unigene4344\_Sample\_011046840, Unigene39243\_Sample\_011046840, Unigene33012\_Sample\_011046840, Unigene37292\_Sample\_011046840, Unigene9682\_Sample\_011046840, Unigene43312\_Sample\_011046840, Unigene32292\_Sample\_011046840, Unigene35790\_Sample\_011046840, Unigene32209\_Sample\_011046840, Unigene16376\_Sample\_011046840, Unigene17229\_Sample\_011046840, Unigene30473\_Sample\_011046840, Unigene39165\_Sample\_011046840, Unigene15033\_Sample\_011046840, Unigene43000\_Sample\_011046840, Unigene30860\_Sample\_011046840, Unigene26740\_Sample\_011046840, Unigene16473\_Sample\_011046840, Unigene3551\_Sample\_011046840, Unigene1008\_Sample\_011046840, Unigene5570\_Sample\_011046840, Unigene40679\_Sample\_011046840, Unigene37870\_Sample\_011046840, Unigene26427\_Sample\_011046840, Unigene42499\_Sample\_011046840, Unigene16770\_Sample\_011046840, Unigene543\_Sample\_011046840, Unigene10730\_Sample\_011046840, Unigene31855\_Sample\_011046840, Unigene8201\_Sample\_011046840, Unigene29526\_Sample\_011046840, Unigene33605\_Sample\_011046840, Unigene33711\_Sample\_011046840, Unigene43632\_Sample\_011046840, Unigene34669\_Sample\_011046840, Unigene35906\_Sample\_011046840, Unigene898\_Sample\_011046840, Unigene43635\_Sample\_011046840, Unigene34301\_Sample\_011046840, Unigene35346\_Sample\_011046840, Unigene5439\_Sample\_011046840, Unigene38337\_Sample\_011046840, Unigene32844\_Sample\_011046840, Unigene1375\_Sample\_011046840, Unigene34436\_Sample\_011046840, Unigene1731\_Sample\_011046840, Unigene16611\_Sample\_011046840, Unigene8083\_Sample\_011046840, Unigene10704\_Sample\_011046840, Unigene26270\_Sample\_011046840, Unigene7744\_Sample\_011046840, Unigene43117\_Sample\_011046840, Unigene25644\_Sample\_011046840, Unigene27239\_Sample\_011046840, Unigene7065\_Sample\_011046840, Unigene15181\_Sample\_011046840, Unigene34833\_Sample\_011046840, Unigene7175\_Sample\_011046840, Unigene7395\_Sample\_011046840, Unigene23201\_Sample\_011046840, Unigene8227\_Sample\_011046840, Unigene22068\_Sample\_011046840, Unigene7410\_Sample\_011046840, Unigene39018\_Sample\_011046840, Unigene14074\_Sample\_011046840, Unigene42615\_Sample\_011046840, Unigene36773\_Sample\_011046840, Unigene17905\_Sample\_011046840, Unigene14139\_Sample\_011046840, Unigene8006\_Sample\_011046840, Unigene2797\_Sample\_011046840, Unigene40658\_Sample\_011046840, Unigene25347\_Sample\_011046840, Unigene40979\_Sample\_011046840, Unigene35547\_Sample\_011046840, Unigene20556\_Sample\_011046840, Unigene22514\_Sample\_011046840, Unigene30760\_Sample\_011046840, Unigene40427\_Sample\_011046840, Unigene2810\_Sample\_011046840, Unigene33019\_Sample\_011046840, Unigene40032\_Sample\_011046840, Unigene33870\_Sample\_011046840, Unigene37571\_Sample\_011046840, Unigene36877\_Sample\_011046840, Unigene15851\_Sample\_011046840, Unigene4839\_Sample\_011046840, Unigene39740\_Sample\_011046840, Unigene42384\_Sample\_011046840, Unigene27359\_Sample\_011046840, Unigene23158\_Sample\_011046840, Unigene42441\_Sample\_011046840, Unigene35031\_Sample\_011046840, Unigene20101\_Sample\_011046840, Unigene42659\_Sample\_011046840, Unigene37034\_Sample\_011046840, Unigene38074\_Sample\_011046840, Unigene40484\_Sample\_011046840, Unigene18009\_Sample\_011046840, Unigene36141\_Sample\_011046840, Unigene42234\_Sample\_011046840, Unigene23241\_Sample\_011046840, Unigene10778\_Sample\_011046840, Unigene39927\_Sample\_011046840, Unigene5564\_Sample\_011046840, Unigene36685\_Sample\_011046840, Unigene41585\_Sample\_011046840, Unigene15148\_Sample\_011046840, Unigene4032\_Sample\_011046840, Unigene33943\_Sample\_011046840, Unigene43082\_Sample\_011046840, Unigene36922\_Sample\_011046840, Unigene7796\_Sample\_011046840, Unigene5007\_Sample\_011046840, Unigene15874\_Sample\_011046840, Unigene34588\_Sample\_011046840, Unigene8203\_Sample\_011046840, Unigene38501\_Sample\_011046840, Unigene40793\_Sample\_011046840, Unigene22867\_Sample\_011046840, Unigene42340\_Sample\_011046840, Unigene41400\_Sample\_011046840, Unigene35494\_Sample\_011046840, Unigene12635\_Sample\_011046840, Unigene36375\_Sample\_011046840, Unigene24034\_Sample\_011046840, Unigene24711\_Sample\_011046840, Unigene2227\_Sample\_011046840, Unigene42189\_Sample\_011046840, Unigene24309\_Sample\_011046840, Unigene41899\_Sample\_011046840, Unigene33906\_Sample\_011046840, Unigene28095\_Sample\_011046840, Unigene36121\_Sample\_011046840, Unigene8240\_Sample\_011046840, Unigene27829\_Sample\_011046840, Unigene40541\_Sample\_011046840, Unigene18069\_Sample\_011046840, Unigene41399\_Sample\_011046840, Unigene12936\_Sample\_011046840, Unigene29046\_Sample\_011046840, Unigene40917\_Sample\_011046840, Unigene42075\_Sample\_011046840, Unigene38028\_Sample\_011046840, Unigene11264\_Sample\_011046840, Unigene41676\_Sample\_011046840, Unigene35179\_Sample\_011046840, Unigene17789\_Sample\_011046840, Unigene40221\_Sample\_011046840, Unigene12818\_Sample\_011046840, Unigene7262\_Sample\_011046840, Unigene42206\_Sample\_011046840, Unigene16016\_Sample\_011046840, Unigene12035\_Sample\_011046840, Unigene2808\_Sample\_011046840, Unigene40596\_Sample\_011046840, Unigene1771\_Sample\_011046840, Unigene972\_Sample\_011046840, Unigene8555\_Sample\_011046840, Unigene7148\_Sample\_011046840, Unigene2084\_Sample\_011046840, Unigene39583\_Sample\_011046840, Unigene38393\_Sample\_011046840, Unigene35532\_Sample\_011046840, Unigene32382\_Sample\_011046840, Unigene2815\_Sample\_011046840, Unigene7669\_Sample\_011046840, Unigene3631\_Sample\_011046840, Unigene31663\_Sample\_011046840, Unigene24311\_Sample\_011046840, Unigene14178\_Sample\_011046840, Unigene17776\_Sample\_011046840, Unigene29611\_Sample\_011046840, Unigene41927\_Sample\_011046840, Unigene29621\_Sample\_011046840, Unigene27942\_Sample\_011046840, Unigene41127\_Sample\_011046840, Unigene13984\_Sample\_011046840, Unigene34362\_Sample\_011046840, Unigene32819\_Sample\_011046840, Unigene30713\_Sample\_011046840, Unigene5552\_Sample\_011046840, Unigene15637\_Sample\_011046840, Unigene34780\_Sample\_011046840, Unigene7506\_Sample\_011046840, Unigene36827\_Sample\_011046840, Unigene19404\_Sample\_011046840, Unigene24938\_Sample\_011046840, Unigene31120\_Sample\_011046840, Unigene43143\_Sample\_011046840, Unigene9887\_Sample\_011046840, Unigene36413\_Sample\_011046840, Unigene11592\_Sample\_011046840, Unigene35789\_Sample\_011046840, Unigene15030\_Sample\_011046840, Unigene40822\_Sample\_011046840, Unigene42964\_Sample\_011046840, Unigene24453\_Sample\_011046840, Unigene42663\_Sample\_011046840, Unigene5963\_Sample\_011046840, Unigene13602\_Sample\_011046840, Unigene43397\_Sample\_011046840, Unigene19793\_Sample\_011046840, Unigene32158\_Sample\_011046840, Unigene42596\_Sample\_011046840, Unigene38864\_Sample\_011046840, Unigene20577\_Sample\_011046840, Unigene14302\_Sample\_011046840, Unigene31135\_Sample\_011046840, Unigene25783\_Sample\_011046840, Unigene25531\_Sample\_011046840, Unigene7067\_Sample\_011046840, Unigene43623\_Sample\_011046840, Unigene7349\_Sample\_011046840, Unigene3226\_Sample\_011046840, Unigene17721\_Sample\_011046840, Unigene31168\_Sample\_011046840, Unigene34071\_Sample\_011046840, Unigene41691\_Sample\_011046840, Unigene26581\_Sample\_011046840, Unigene28708\_Sample\_011046840, Unigene42865\_Sample\_011046840, Unigene6064\_Sample\_011046840, Unigene16389\_Sample\_011046840, Unigene3257\_Sample\_011046840, Unigene29534\_Sample\_011046840, Unigene22159\_Sample\_011046840, Unigene29452\_Sample\_011046840, Unigene32280\_Sample\_011046840, Unigene10176\_Sample\_011046840, Unigene33275\_Sample\_011046840, Unigene31074\_Sample\_011046840, Unigene15062\_Sample\_011046840, Unigene17700\_Sample\_011046840, Unigene6641\_Sample\_011046840, Unigene36699\_Sample\_011046840, Unigene28415\_Sample\_011046840, Unigene33858\_Sample\_011046840, Unigene38658\_Sample\_011046840, Unigene23820\_Sample\_011046840, Unigene7402\_Sample\_011046840, Unigene5375\_Sample\_011046840, Unigene10829\_Sample\_011046840, Unigene11629\_Sample\_011046840, Unigene43387\_Sample\_011046840, Unigene35909\_Sample\_011046840, Unigene26502\_Sample\_011046840, Unigene36268\_Sample\_011046840, Unigene29818\_Sample\_011046840, Unigene31458\_Sample\_011046840, Unigene30136\_Sample\_011046840, Unigene24201\_Sample\_011046840, Unigene8372\_Sample\_011046840, Unigene40709\_Sample\_011046840, Unigene35699\_Sample\_011046840, Unigene27865\_Sample\_011046840, Unigene40966\_Sample\_011046840, Unigene489\_Sample\_011046840, Unigene10957\_Sample\_011046840, Unigene4518\_Sample\_011046840, Unigene33342\_Sample\_011046840, Unigene26057\_Sample\_011046840, Unigene34506\_Sample\_011046840, Unigene20052\_Sample\_011046840, Unigene42097\_Sample\_011046840, Unigene24244\_Sample\_011046840, Unigene41398\_Sample\_011046840, Unigene7025\_Sample\_011046840, Unigene22635\_Sample\_011046840, Unigene27993\_Sample\_011046840, Unigene39020\_Sample\_011046840, Unigene40262\_Sample\_011046840, Unigene9922\_Sample\_011046840, Unigene24917\_Sample\_011046840, Unigene32947\_Sample\_011046840, Unigene32360\_Sample\_011046840, Unigene29103\_Sample\_011046840, Unigene39324\_Sample\_011046840, Unigene32594\_Sample\_011046840, Unigene22752\_Sample\_011046840, Unigene40551\_Sample\_011046840, Unigene41178\_Sample\_011046840, Unigene43627\_Sample\_011046840, Unigene33112\_Sample\_011046840, Unigene37999\_Sample\_011046840, Unigene6285\_Sample\_011046840, Unigene28867\_Sample\_011046840, Unigene33052\_Sample\_011046840, Unigene33999\_Sample\_011046840, Unigene31448\_Sample\_011046840, Unigene16829\_Sample\_011046840, Unigene29049\_Sample\_011046840, Unigene32831\_Sample\_011046840, Unigene26512\_Sample\_011046840, Unigene40783\_Sample\_011046840, Unigene42138\_Sample\_011046840, Unigene29397\_Sample\_011046840, Unigene37985\_Sample\_011046840, Unigene14485\_Sample\_011046840, Unigene36500\_Sample\_011046840, Unigene19235\_Sample\_011046840, Unigene20304\_Sample\_011046840, Unigene42585\_Sample\_011046840, Unigene29567\_Sample\_011046840, Unigene34472\_Sample\_011046840, Unigene15594\_Sample\_011046840, Unigene33393\_Sample\_011046840, Unigene31108\_Sample\_011046840, Unigene16321\_Sample\_011046840, Unigene8615\_Sample\_011046840, Unigene33196\_Sample\_011046840, Unigene19400\_Sample\_011046840, Unigene4619\_Sample\_011046840, Unigene24373\_Sample\_011046840, Unigene19647\_Sample\_011046840, Unigene34843\_Sample\_011046840, Unigene10033\_Sample\_011046840, Unigene40222\_Sample\_011046840, Unigene38165\_Sample\_011046840, Unigene33473\_Sample\_011046840, Unigene42190\_Sample\_011046840, Unigene41068\_Sample\_011046840, Unigene30539\_Sample\_011046840, Unigene39171\_Sample\_011046840, Unigene37353\_Sample\_011046840, Unigene42460\_Sample\_011046840, Unigene26767\_Sample\_011046840, Unigene16855\_Sample\_011046840, Unigene38564\_Sample\_011046840, Unigene25769\_Sample\_011046840, Unigene19809\_Sample\_011046840, Unigene39825\_Sample\_011046840, Unigene40368\_Sample\_011046840, Unigene6561\_Sample\_011046840, Unigene22844\_Sample\_011046840, Unigene10568\_Sample\_011046840, Unigene43614\_Sample\_011046840, Unigene41718\_Sample\_011046840, Unigene38534\_Sample\_011046840, Unigene36032\_Sample\_011046840, Unigene535\_Sample\_011046840, Unigene37644\_Sample\_011046840, Unigene32515\_Sample\_011046840, Unigene39951\_Sample\_011046840, Unigene38730\_Sample\_011046840, Unigene43450\_Sample\_011046840, Unigene40192\_Sample\_011046840, Unigene39935\_Sample\_011046840, Unigene473\_Sample\_011046840, Unigene39427\_Sample\_011046840, Unigene20351\_Sample\_011046840, Unigene25001\_Sample\_011046840, Unigene20367\_Sample\_011046840, Unigene31408\_Sample\_011046840, Unigene8211\_Sample\_011046840, Unigene4307\_Sample\_011046840, Unigene39007\_Sample\_011046840, Unigene39015\_Sample\_011046840, Unigene37487\_Sample\_011046840, Unigene38616\_Sample\_011046840, Unigene43281\_Sample\_011046840, Unigene815\_Sample\_011046840, Unigene13836\_Sample\_011046840, Unigene26820\_Sample\_011046840, Unigene42164\_Sample\_011046840, Unigene6530\_Sample\_011046840, Unigene412\_Sample\_011046840, Unigene38370\_Sample\_011046840, Unigene20078\_Sample\_011046840, Unigene38792\_Sample\_011046840, Unigene30813\_Sample\_011046840, Unigene43078\_Sample\_011046840, Unigene22254\_Sample\_011046840, Unigene34112\_Sample\_011046840, Unigene40971\_Sample\_011046840, Unigene43019\_Sample\_011046840, Unigene27313\_Sample\_011046840, Unigene17135\_Sample\_011046840, Unigene43311\_Sample\_011046840, Unigene43216\_Sample\_011046840, Unigene24243\_Sample\_011046840, Unigene34992\_Sample\_011046840, Unigene6409\_Sample\_011046840, Unigene22630\_Sample\_011046840, Unigene42997\_Sample\_011046840, Unigene42121\_Sample\_011046840, Unigene27337\_Sample\_011046840, Unigene9880\_Sample\_011046840, Unigene35787\_Sample\_011046840, Unigene34351\_Sample\_011046840, Unigene11464\_Sample\_011046840, Unigene39486\_Sample\_011046840, Unigene35792\_Sample\_011046840, Unigene6378\_Sample\_011046840, Unigene7938\_Sample\_011046840, Unigene33580\_Sample\_011046840, Unigene6293\_Sample\_011046840, Unigene28473\_Sample\_011046840, Unigene27358\_Sample\_011046840, Unigene37793\_Sample\_011046840, Unigene15491\_Sample\_011046840, Unigene32568\_Sample\_011046840, Unigene41588\_Sample\_011046840, Unigene28437\_Sample\_011046840, Unigene43176\_Sample\_011046840, Unigene36475\_Sample\_011046840, Unigene14116\_Sample\_011046840, Unigene37359\_Sample\_011046840, Unigene38852\_Sample\_011046840, Unigene13391\_Sample\_011046840, Unigene39348\_Sample\_011046840, Unigene11853\_Sample\_011046840, Unigene39080\_Sample\_011046840, Unigene38047\_Sample\_011046840, Unigene40881\_Sample\_011046840, Unigene12767\_Sample\_011046840, Unigene35476\_Sample\_011046840, Unigene29006\_Sample\_011046840, Unigene39136\_Sample\_011046840, Unigene15696\_Sample\_011046840, Unigene30322\_Sample\_011046840, Unigene5815\_Sample\_011046840, Unigene29131\_Sample\_011046840, Unigene39668\_Sample\_011046840, Unigene42406\_Sample\_011046840, Unigene40903\_Sample\_011046840, Unigene42227\_Sample\_011046840, Unigene40992\_Sample\_011046840, Unigene36427\_Sample\_011046840, Unigene16130\_Sample\_011046840, Unigene34102\_Sample\_011046840, Unigene36876\_Sample\_011046840, Unigene24004\_Sample\_011046840, Unigene39404\_Sample\_011046840, Unigene26969\_Sample\_011046840, Unigene34453\_Sample\_011046840, Unigene12655\_Sample\_011046840, Unigene37877\_Sample\_011046840, Unigene27975\_Sample\_011046840, Unigene14511\_Sample\_011046840, Unigene32134\_Sample\_011046840, Unigene32749\_Sample\_011046840, Unigene26815\_Sample\_011046840, Unigene26734\_Sample\_011046840, Unigene43062\_Sample\_011046840, Unigene29301\_Sample\_011046840, Unigene42383\_Sample\_011046840, Unigene28404\_Sample\_011046840, Unigene16269\_Sample\_011046840, Unigene40565\_Sample\_011046840, Unigene39381\_Sample\_011046840, Unigene2174\_Sample\_011046840, Unigene32865\_Sample\_011046840, Unigene27180\_Sample\_011046840, Unigene688\_Sample\_011046840, Unigene36400\_Sample\_011046840, Unigene43084\_Sample\_011046840, Unigene3156\_Sample\_011046840, Unigene35434\_Sample\_011046840, Unigene6180\_Sample\_011046840, Unigene33938\_Sample\_011046840, Unigene40865\_Sample\_011046840, Unigene39866\_Sample\_011046840, Unigene31310\_Sample\_011046840, Unigene23252\_Sample\_011046840, Unigene42296\_Sample\_011046840, Unigene22122\_Sample\_011046840, Unigene37628\_Sample\_011046840, Unigene11949\_Sample\_011046840, Unigene34464\_Sample\_011046840, Unigene36926\_Sample\_011046840, Unigene42789\_Sample\_011046840, Unigene13828\_Sample\_011046840, Unigene1963\_Sample\_011046840, Unigene39156\_Sample\_011046840, Unigene11179\_Sample\_011046840, Unigene40994\_Sample\_011046840, Unigene1256\_Sample\_011046840, Unigene6241\_Sample\_011046840, Unigene37705\_Sample\_011046840, Unigene16976\_Sample\_011046840, Unigene25184\_Sample\_011046840, Unigene31945\_Sample\_011046840, Unigene23357\_Sample\_011046840, Unigene23281\_Sample\_011046840, Unigene37392\_Sample\_011046840, Unigene39992\_Sample\_011046840, Unigene8218\_Sample\_011046840, Unigene10696\_Sample\_011046840, Unigene36706\_Sample\_011046840, Unigene43477\_Sample\_011046840, Unigene30048\_Sample\_011046840, Unigene41792\_Sample\_011046840, Unigene4723\_Sample\_011046840, Unigene30691\_Sample\_011046840, Unigene40837\_Sample\_011046840, Unigene14017\_Sample\_011046840, Unigene36112\_Sample\_011046840, Unigene20250\_Sample\_011046840, Unigene7503\_Sample\_011046840, Unigene30919\_Sample\_011046840, Unigene14611\_Sample\_011046840, Unigene41062\_Sample\_011046840, Unigene17216\_Sample\_011046840, Unigene40424\_Sample\_011046840, Unigene3599\_Sample\_011046840, Unigene23324\_Sample\_011046840, Unigene29427\_Sample\_011046840, Unigene17375\_Sample\_011046840, Unigene36567\_Sample\_011046840, Unigene15303\_Sample\_011046840, Unigene16035\_Sample\_011046840, Unigene27997\_Sample\_011046840, Unigene42864\_Sample\_011046840, Unigene36920\_Sample\_011046840, Unigene13276\_Sample\_011046840, Unigene39180\_Sample\_011046840, Unigene43536\_Sample\_011046840, Unigene34655\_Sample\_011046840, Unigene40818\_Sample\_011046840, Unigene38377\_Sample\_011046840, Unigene18440\_Sample\_011046840, Unigene28736\_Sample\_011046840, Unigene37830\_Sample\_011046840, Unigene33866\_Sample\_011046840, Unigene43607\_Sample\_011046840, Unigene21072\_Sample\_011046840, Unigene12713\_Sample\_011046840, Unigene22459\_Sample\_011046840, Unigene33926\_Sample\_011046840, Unigene37219\_Sample\_011046840, Unigene38559\_Sample\_011046840, Unigene11613\_Sample\_011046840, Unigene38360\_Sample\_011046840, Unigene38044\_Sample\_011046840, Unigene42007\_Sample\_011046840, Unigene30105\_Sample\_011046840, Unigene15831\_Sample\_011046840, Unigene41174\_Sample\_011046840, Unigene41740\_Sample\_011046840, Unigene432\_Sample\_011046840, Unigene17416\_Sample\_011046840, Unigene40346\_Sample\_011046840, Unigene35883\_Sample\_011046840, Unigene31610\_Sample\_011046840, Unigene37041\_Sample\_011046840, Unigene30572\_Sample\_011046840, Unigene23630\_Sample\_011046840, Unigene33760\_Sample\_011046840, Unigene38146\_Sample\_011046840, Unigene24246\_Sample\_011046840, Unigene31584\_Sample\_011046840, Unigene36376\_Sample\_011046840, Unigene30798\_Sample\_011046840, Unigene39838\_Sample\_011046840, Unigene3842\_Sample\_011046840, Unigene12743\_Sample\_011046840, Unigene31773\_Sample\_011046840, Unigene3928\_Sample\_011046840, Unigene41001\_Sample\_011046840, Unigene23856\_Sample\_011046840, Unigene43115\_Sample\_011046840, Unigene22258\_Sample\_011046840, Unigene17210\_Sample\_011046840, Unigene41498\_Sample\_011046840, Unigene31398\_Sample\_011046840, Unigene10770\_Sample\_011046840, Unigene42171\_Sample\_011046840, Unigene24889\_Sample\_011046840, Unigene9871\_Sample\_011046840, Unigene38932\_Sample\_011046840, Unigene33443\_Sample\_011046840, Unigene38152\_Sample\_011046840, Unigene14629\_Sample\_011046840, Unigene14756\_Sample\_011046840, Unigene42050\_Sample\_011046840, Unigene42835\_Sample\_011046840, Unigene5197\_Sample\_011046840, Unigene29880\_Sample\_011046840, Unigene7717\_Sample\_011046840, Unigene37049\_Sample\_011046840, Unigene5121\_Sample\_011046840, Unigene43147\_Sample\_011046840, Unigene38039\_Sample\_011046840, Unigene28861\_Sample\_011046840, Unigene42602\_Sample\_011046840, Unigene10135\_Sample\_011046840, Unigene22688\_Sample\_011046840, Unigene10802\_Sample\_011046840, Unigene40391\_Sample\_011046840, Unigene38670\_Sample\_011046840, Unigene29016\_Sample\_011046840, Unigene37540\_Sample\_011046840, Unigene21026\_Sample\_011046840, Unigene36690\_Sample\_011046840, Unigene39732\_Sample\_011046840, Unigene1959\_Sample\_011046840, Unigene18735\_Sample\_011046840, Unigene32368\_Sample\_011046840, Unigene19481\_Sample\_011046840, Unigene43025\_Sample\_011046840, Unigene41288\_Sample\_011046840, Unigene30094\_Sample\_011046840, Unigene25579\_Sample\_011046840, Unigene34053\_Sample\_011046840, Unigene28414\_Sample\_011046840, Unigene12587\_Sample\_011046840, Unigene39882\_Sample\_011046840, Unigene35872\_Sample\_011046840, Unigene10288\_Sample\_011046840, Unigene9718\_Sample\_011046840, Unigene39555\_Sample\_011046840, Unigene35927\_Sample\_011046840, Unigene5480\_Sample\_011046840, Unigene14666\_Sample\_011046840, Unigene36535\_Sample\_011046840, Unigene12679\_Sample\_011046840, Unigene40687\_Sample\_011046840, Unigene39837\_Sample\_011046840, Unigene23176\_Sample\_011046840, Unigene24594\_Sample\_011046840, Unigene41858\_Sample\_011046840, Unigene42683\_Sample\_011046840, Unigene14292\_Sample\_011046840, Unigene27724\_Sample\_011046840, Unigene19383\_Sample\_011046840, Unigene10601\_Sample\_011046840, Unigene33259\_Sample\_011046840, Unigene30862\_Sample\_011046840, Unigene41336\_Sample\_011046840, Unigene25871\_Sample\_011046840, Unigene33322\_Sample\_011046840, Unigene30573\_Sample\_011046840, Unigene38115\_Sample\_011046840, Unigene34746\_Sample\_011046840, Unigene36462\_Sample\_011046840, Unigene40812\_Sample\_011046840, Unigene40787\_Sample\_011046840, Unigene37745\_Sample\_011046840, Unigene24674\_Sample\_011046840, Unigene40184\_Sample\_011046840, Unigene28636\_Sample\_011046840, Unigene33889\_Sample\_011046840, Unigene28901\_Sample\_011046840, Unigene43453\_Sample\_011046840, Unigene39490\_Sample\_011046840, Unigene39632\_Sample\_011046840, Unigene11701\_Sample\_011046840, Unigene31638\_Sample\_011046840, Unigene11125\_Sample\_011046840, Unigene42030\_Sample\_011046840, Unigene28758\_Sample\_011046840, Unigene25431\_Sample\_011046840, Unigene4320\_Sample\_011046840, Unigene20420\_Sample\_011046840, Unigene28316\_Sample\_011046840, Unigene6563\_Sample\_011046840, Unigene36951\_Sample\_011046840, Unigene43031\_Sample\_011046840, Unigene12197\_Sample\_011046840, Unigene36090\_Sample\_011046840, Unigene22961\_Sample\_011046840, Unigene43363\_Sample\_011046840, Unigene42068\_Sample\_011046840, Unigene22232\_Sample\_011046840, Unigene38021\_Sample\_011046840, Unigene18262\_Sample\_011046840, Unigene41561\_Sample\_011046840, Unigene26967\_Sample\_011046840, Unigene12868\_Sample\_011046840, Unigene37710\_Sample\_011046840, Unigene41901\_Sample\_011046840, Unigene4043\_Sample\_011046840, Unigene25934\_Sample\_011046840, Unigene42079\_Sample\_011046840, Unigene165\_Sample\_011046840, Unigene43611\_Sample\_011046840, Unigene7072\_Sample\_011046840, Unigene3161\_Sample\_011046840, Unigene40387\_Sample\_011046840, Unigene21295\_Sample\_011046840, Unigene42944\_Sample\_011046840, Unigene5047\_Sample\_011046840, Unigene1859\_Sample\_011046840, Unigene4721\_Sample\_011046840, Unigene35151\_Sample\_011046840, Unigene35324\_Sample\_011046840, Unigene29879\_Sample\_011046840, Unigene5724\_Sample\_011046840, Unigene25629\_Sample\_011046840, Unigene29966\_Sample\_011046840, Unigene41467\_Sample\_011046840, Unigene16882\_Sample\_011046840, Unigene42154\_Sample\_011046840, Unigene38267\_Sample\_011046840, Unigene42047\_Sample\_011046840, Unigene25720\_Sample\_011046840, Unigene36496\_Sample\_011046840, Unigene14078\_Sample\_011046840, Unigene13016\_Sample\_011046840, Unigene39103\_Sample\_011046840, Unigene24184\_Sample\_011046840, Unigene3537\_Sample\_011046840, Unigene38522\_Sample\_011046840, Unigene6787\_Sample\_011046840, Unigene5393\_Sample\_011046840, Unigene37573\_Sample\_011046840, Unigene25466\_Sample\_011046840, Unigene31793\_Sample\_011046840, Unigene12386\_Sample\_011046840, Unigene29845\_Sample\_011046840, Unigene35118\_Sample\_011046840, Unigene42837\_Sample\_011046840, Unigene42475\_Sample\_011046840, Unigene42531\_Sample\_011046840, Unigene42204\_Sample\_011046840, Unigene2615\_Sample\_011046840, Unigene35939\_Sample\_011046840, Unigene34233\_Sample\_011046840, Unigene39958\_Sample\_011046840, Unigene23541\_Sample\_011046840, Unigene18141\_Sample\_011046840, Unigene42252\_Sample\_011046840, Unigene12084\_Sample\_011046840, Unigene40601\_Sample\_011046840, Unigene32188\_Sample\_011046840, Unigene39418\_Sample\_011046840, Unigene42200\_Sample\_011046840, Unigene21974\_Sample\_011046840, Unigene40208\_Sample\_011046840, Unigene37077\_Sample\_011046840, Unigene20828\_Sample\_011046840, Unigene30505\_Sample\_011046840, Unigene14237\_Sample\_011046840, Unigene42786\_Sample\_011046840, Unigene3395\_Sample\_011046840, Unigene35395\_Sample\_011046840, Unigene3814\_Sample\_011046840, Unigene42886\_Sample\_011046840, Unigene38215\_Sample\_011046840, Unigene33515\_Sample\_011046840, Unigene38121\_Sample\_011046840, Unigene41880\_Sample\_011046840, Unigene19448\_Sample\_011046840, Unigene3164\_Sample\_011046840, Unigene42133\_Sample\_011046840, Unigene30530\_Sample\_011046840, Unigene20634\_Sample\_011046840, Unigene7794\_Sample\_011046840, Unigene31\_Sample\_011046840, Unigene23873\_Sample\_011046840, Unigene33979\_Sample\_011046840, Unigene27545\_Sample\_011046840, Unigene38091\_Sample\_011046840, Unigene31822\_Sample\_011046840, Unigene41582\_Sample\_011046840, Unigene38462\_Sample\_011046840, Unigene34355\_Sample\_011046840, Unigene39505\_Sample\_011046840, Unigene8064\_Sample\_011046840, Unigene7983\_Sample\_011046840, Unigene37702\_Sample\_011046840, Unigene29411\_Sample\_011046840, Unigene43525\_Sample\_011046840, Unigene17079\_Sample\_011046840, Unigene5594\_Sample\_011046840, Unigene35068\_Sample\_011046840, Unigene30197\_Sample\_011046840, Unigene18134\_Sample\_011046840, Unigene35433\_Sample\_011046840, Unigene35554\_Sample\_011046840, Unigene25328\_Sample\_011046840, Unigene39870\_Sample\_011046840, Unigene9730\_Sample\_011046840, Unigene22550\_Sample\_011046840, Unigene24428\_Sample\_011046840, Unigene40465\_Sample\_011046840, Unigene25313\_Sample\_011046840, Unigene28259\_Sample\_011046840, Unigene37149\_Sample\_011046840, Unigene34500\_Sample\_011046840, Unigene29711\_Sample\_011046840, Unigene40223\_Sample\_011046840, Unigene6498\_Sample\_011046840, Unigene32116\_Sample\_011046840, Unigene20023\_Sample\_011046840, Unigene31318\_Sample\_011046840, Unigene39179\_Sample\_011046840, Unigene35234\_Sample\_011046840, Unigene3934\_Sample\_011046840, Unigene43422\_Sample\_011046840, Unigene14154\_Sample\_011046840, Unigene2408\_Sample\_011046840, Unigene42127\_Sample\_011046840, Unigene24187\_Sample\_011046840, Unigene23188\_Sample\_011046840, Unigene1039\_Sample\_011046840, Unigene31248\_Sample\_011046840, Unigene35633\_Sample\_011046840, Unigene29346\_Sample\_011046840, Unigene12033\_Sample\_011046840, Unigene17239\_Sample\_011046840, Unigene40016\_Sample\_011046840, Unigene35643\_Sample\_011046840, Unigene42177\_Sample\_011046840, Unigene33743\_Sample\_011046840, Unigene43636\_Sample\_011046840, Unigene15849\_Sample\_011046840, Unigene23811\_Sample\_011046840, Unigene41355\_Sample\_011046840, Unigene7643\_Sample\_011046840, Unigene41574\_Sample\_011046840, Unigene31649\_Sample\_011046840, Unigene33736\_Sample\_011046840, Unigene34555\_Sample\_011046840, Unigene10641\_Sample\_011046840, Unigene32875\_Sample\_011046840, Unigene11685\_Sample\_011046840, Unigene27895\_Sample\_011046840, Unigene4327\_Sample\_011046840, Unigene43095\_Sample\_011046840, Unigene12062\_Sample\_011046840, Unigene16063\_Sample\_011046840, Unigene30508\_Sample\_011046840, Unigene36187\_Sample\_011046840, Unigene5400\_Sample\_011046840, Unigene3339\_Sample\_011046840, Unigene36260\_Sample\_011046840, Unigene32706\_Sample\_011046840, Unigene37852\_Sample\_011046840, Unigene31684\_Sample\_011046840, Unigene28063\_Sample\_011046840, Unigene16650\_Sample\_011046840, Unigene39275\_Sample\_011046840, Unigene12701\_Sample\_011046840, Unigene14279\_Sample\_011046840, Unigene32354\_Sample\_011046840, Unigene30059\_Sample\_011046840, Unigene2326\_Sample\_011046840, Unigene10035\_Sample\_011046840, Unigene8904\_Sample\_011046840, Unigene36088\_Sample\_011046840, Unigene41771\_Sample\_011046840, Unigene41481\_Sample\_011046840, Unigene42569\_Sample\_011046840, Unigene18212\_Sample\_011046840, Unigene9721\_Sample\_011046840, Unigene8113\_Sample\_011046840, Unigene42574\_Sample\_011046840, Unigene25488\_Sample\_011046840, Unigene41935\_Sample\_011046840, Unigene4815\_Sample\_011046840, Unigene34896\_Sample\_011046840, Unigene14473\_Sample\_011046840, Unigene26865\_Sample\_011046840, Unigene23169\_Sample\_011046840, Unigene39391\_Sample\_011046840, Unigene2581\_Sample\_011046840, Unigene43556\_Sample\_011046840, Unigene41980\_Sample\_011046840, Unigene42325\_Sample\_011046840, Unigene42348\_Sample\_011046840, Unigene35074\_Sample\_011046840, Unigene39497\_Sample\_011046840, Unigene2218\_Sample\_011046840, Unigene39081\_Sample\_011046840, Unigene14503\_Sample\_011046840, Unigene41972\_Sample\_011046840, Unigene7458\_Sample\_011046840, Unigene21905\_Sample\_011046840, Unigene42768\_Sample\_011046840, Unigene12341\_Sample\_011046840, Unigene41855\_Sample\_011046840, Unigene41007\_Sample\_011046840, Unigene10729\_Sample\_011046840, Unigene19018\_Sample\_011046840, Unigene10318\_Sample\_011046840, Unigene3788\_Sample\_011046840, Unigene13393\_Sample\_011046840, Unigene41324\_Sample\_011046840, Unigene40612\_Sample\_011046840, Unigene6862\_Sample\_011046840, Unigene32621\_Sample\_011046840, Unigene23759\_Sample\_011046840, Unigene36342\_Sample\_011046840, Unigene33797\_Sample\_011046840, Unigene2687\_Sample\_011046840, Unigene21944\_Sample\_011046840, Unigene2151\_Sample\_011046840, Unigene42626\_Sample\_011046840, Unigene14750\_Sample\_011046840, Unigene31441\_Sample\_011046840, Unigene35459\_Sample\_011046840, Unigene43348\_Sample\_011046840, Unigene43412\_Sample\_011046840, Unigene2152\_Sample\_011046840, Unigene39717\_Sample\_011046840, Unigene4686\_Sample\_011046840, Unigene36544\_Sample\_011046840, Unigene17677\_Sample\_011046840, Unigene41261\_Sample\_011046840, Unigene10474\_Sample\_011046840, Unigene17194\_Sample\_011046840, Unigene15889\_Sample\_011046840, Unigene27951\_Sample\_011046840, Unigene31900\_Sample\_011046840, Unigene37045\_Sample\_011046840, Unigene4497\_Sample\_011046840, Unigene12353\_Sample\_011046840, Unigene2092\_Sample\_011046840, Unigene12675\_Sample\_011046840, Unigene34828\_Sample\_011046840, Unigene24005\_Sample\_011046840, Unigene22852\_Sample\_011046840, Unigene37741\_Sample\_011046840, Unigene19653\_Sample\_011046840, Unigene39559\_Sample\_011046840, Unigene40562\_Sample\_011046840, Unigene40053\_Sample\_011046840, Unigene32836\_Sample\_011046840, Unigene37404\_Sample\_011046840, Unigene32314\_Sample\_011046840, Unigene20154\_Sample\_011046840, Unigene39437\_Sample\_011046840, Unigene39720\_Sample\_011046840, Unigene5378\_Sample\_011046840, Unigene208\_Sample\_011046840, Unigene43258\_Sample\_011046840, Unigene19918\_Sample\_011046840, Unigene5436\_Sample\_011046840, Unigene20526\_Sample\_011046840, Unigene26338\_Sample\_011046840, Unigene40753\_Sample\_011046840, Unigene15987\_Sample\_011046840, Unigene40278\_Sample\_011046840, Unigene3127\_Sample\_011046840, Unigene26705\_Sample\_011046840, Unigene19593\_Sample\_011046840, Unigene6740\_Sample\_011046840, Unigene37517\_Sample\_011046840, Unigene30236\_Sample\_011046840, Unigene28852\_Sample\_011046840, Unigene33998\_Sample\_011046840, Unigene31994\_Sample\_011046840, Unigene26163\_Sample\_011046840, Unigene27886\_Sample\_011046840, Unigene18907\_Sample\_011046840, Unigene26846\_Sample\_011046840, Unigene5241\_Sample\_011046840, Unigene34039\_Sample\_011046840, Unigene38608\_Sample\_011046840, Unigene2543\_Sample\_011046840, Unigene35951\_Sample\_011046840, Unigene6760\_Sample\_011046840, Unigene21415\_Sample\_011046840, Unigene41637\_Sample\_011046840, Unigene20065\_Sample\_011046840, Unigene35555\_Sample\_011046840, Unigene28002\_Sample\_011046840, Unigene30702\_Sample\_011046840, Unigene20428\_Sample\_011046840, Unigene14691\_Sample\_011046840, Unigene42798\_Sample\_011046840, Unigene23889\_Sample\_011046840, Unigene40899\_Sample\_011046840, Unigene42418\_Sample\_011046840, Unigene31192\_Sample\_011046840, Unigene3075\_Sample\_011046840, Unigene21332\_Sample\_011046840, Unigene14501\_Sample\_011046840, Unigene42183\_Sample\_011046840, Unigene34524\_Sample\_011046840, Unigene30629\_Sample\_011046840, Unigene16244\_Sample\_011046840, Unigene42293\_Sample\_011046840, Unigene31880\_Sample\_011046840, Unigene32589\_Sample\_011046840, Unigene23234\_Sample\_011046840, Unigene2740\_Sample\_011046840, Unigene5453\_Sample\_011046840, Unigene21959\_Sample\_011046840, Unigene22620\_Sample\_011046840, Unigene41709\_Sample\_011046840, Unigene43416\_Sample\_011046840, Unigene34975\_Sample\_011046840, Unigene8660\_Sample\_011046840, Unigene37131\_Sample\_011046840, Unigene26023\_Sample\_011046840, Unigene16906\_Sample\_011046840, Unigene29661\_Sample\_011046840, Unigene40616\_Sample\_011046840, Unigene43186\_Sample\_011046840, Unigene29145\_Sample\_011046840, Unigene7789\_Sample\_011046840, Unigene26507\_Sample\_011046840, Unigene31407\_Sample\_011046840, Unigene42281\_Sample\_011046840, Unigene8121\_Sample\_011046840, Unigene34432\_Sample\_011046840, Unigene41419\_Sample\_011046840, Unigene22119\_Sample\_011046840, Unigene43273\_Sample\_011046840, Unigene37116\_Sample\_011046840, Unigene32645\_Sample\_011046840, Unigene7513\_Sample\_011046840, Unigene26212\_Sample\_011046840, Unigene2519\_Sample\_011046840, Unigene22727\_Sample\_011046840, Unigene41160\_Sample\_011046840, Unigene42088\_Sample\_011046840, Unigene34380\_Sample\_011046840, Unigene19472\_Sample\_011046840, Unigene34400\_Sample\_011046840, Unigene39994\_Sample\_011046840, Unigene17139\_Sample\_011046840, Unigene40445\_Sample\_011046840, Unigene19704\_Sample\_011046840, Unigene42738\_Sample\_011046840, Unigene43001\_Sample\_011046840, Unigene18270\_Sample\_011046840, Unigene41119\_Sample\_011046840, Unigene356\_Sample\_011046840, Unigene2068\_Sample\_011046840, Unigene34304\_Sample\_011046840, Unigene36231\_Sample\_011046840, Unigene39253\_Sample\_011046840, Unigene4276\_Sample\_011046840, Unigene11443\_Sample\_011046840, Unigene10448\_Sample\_011046840, Unigene7089\_Sample\_011046840, Unigene14521\_Sample\_011046840, Unigene40309\_Sample\_011046840, Unigene30053\_Sample\_011046840, Unigene18953\_Sample\_011046840, Unigene41695\_Sample\_011046840, Unigene39582\_Sample\_011046840, Unigene41799\_Sample\_011046840, Unigene16017\_Sample\_011046840, Unigene42249\_Sample\_011046840, Unigene28188\_Sample\_011046840, Unigene43217\_Sample\_011046840, Unigene22397\_Sample\_011046840, Unigene2237\_Sample\_011046840, Unigene42271\_Sample\_011046840, Unigene11994\_Sample\_011046840, Unigene31695\_Sample\_011046840, Unigene42116\_Sample\_011046840, Unigene11132\_Sample\_011046840, Unigene12950\_Sample\_011046840, Unigene8228\_Sample\_011046840, Unigene7856\_Sample\_011046840, Unigene42354\_Sample\_011046840, Unigene39818\_Sample\_011046840, Unigene36810\_Sample\_011046840, Unigene39399\_Sample\_011046840, Unigene43542\_Sample\_011046840, Unigene5443\_Sample\_011046840, Unigene38542\_Sample\_011046840, Unigene41337\_Sample\_011046840, Unigene27657\_Sample\_011046840, Unigene40404\_Sample\_011046840, Unigene29336\_Sample\_011046840, Unigene34874\_Sample\_011046840, Unigene35820\_Sample\_011046840, Unigene507\_Sample\_011046840, Unigene39628\_Sample\_011046840, Unigene8594\_Sample\_011046840, Unigene24129\_Sample\_011046840, Unigene6886\_Sample\_011046840, Unigene31662\_Sample\_011046840, Unigene20272\_Sample\_011046840, Unigene39204\_Sample\_011046840, Unigene28265\_Sample\_011046840, Unigene12153\_Sample\_011046840, Unigene35008\_Sample\_011046840, Unigene36013\_Sample\_011046840, Unigene9006\_Sample\_011046840, Unigene38540\_Sample\_011046840, Unigene25307\_Sample\_011046840, Unigene36128\_Sample\_011046840, Unigene38354\_Sample\_011046840, Unigene43631\_Sample\_011046840, Unigene31373\_Sample\_011046840, Unigene39649\_Sample\_011046840, Unigene7935\_Sample\_011046840, Unigene2986\_Sample\_011046840, Unigene31576\_Sample\_011046840, Unigene43486\_Sample\_011046840, Unigene27792\_Sample\_011046840, Unigene42666\_Sample\_011046840, Unigene11875\_Sample\_011046840, Unigene9715\_Sample\_011046840, Unigene7825\_Sample\_011046840, Unigene40602\_Sample\_011046840, Unigene27152\_Sample\_011046840, Unigene23601\_Sample\_011046840, Unigene15570\_Sample\_011046840, Unigene33901\_Sample\_011046840, Unigene15138\_Sample\_011046840, Unigene36025\_Sample\_011046840, Unigene19183\_Sample\_011046840, Unigene31646\_Sample\_011046840, Unigene35279\_Sample\_011046840, Unigene35252\_Sample\_011046840, Unigene29670\_Sample\_011046840, Unigene34909\_Sample\_011046840, Unigene4529\_Sample\_011046840, Unigene37829\_Sample\_011046840, Unigene41664\_Sample\_011046840, Unigene31732\_Sample\_011046840, Unigene39606\_Sample\_011046840, Unigene3153\_Sample\_011046840, Unigene42298\_Sample\_011046840, Unigene39674\_Sample\_011046840, Unigene17985\_Sample\_011046840, Unigene39633\_Sample\_011046840, Unigene42376\_Sample\_011046840, Unigene39748\_Sample\_011046840, Unigene16618\_Sample\_011046840, Unigene26435\_Sample\_011046840, Unigene41478\_Sample\_011046840, Unigene3258\_Sample\_011046840, Unigene5425\_Sample\_011046840, Unigene6039\_Sample\_011046840, Unigene40703\_Sample\_011046840, Unigene19931\_Sample\_011046840, Unigene7299\_Sample\_011046840, Unigene39226\_Sample\_011046840, Unigene43445\_Sample\_011046840, Unigene2524\_Sample\_011046840, Unigene36817\_Sample\_011046840, Unigene33539\_Sample\_011046840, Unigene12013\_Sample\_011046840, Unigene41090\_Sample\_011046840, Unigene42509\_Sample\_011046840, Unigene3219\_Sample\_011046840, Unigene42756\_Sample\_011046840, Unigene40031\_Sample\_011046840, Unigene15184\_Sample\_011046840, Unigene5791\_Sample\_011046840, Unigene40452\_Sample\_011046840, Unigene41720\_Sample\_011046840, Unigene23472\_Sample\_011046840, Unigene43516\_Sample\_011046840, Unigene40814\_Sample\_011046840, Unigene7994\_Sample\_011046840, Unigene39346\_Sample\_011046840, Unigene41163\_Sample\_011046840, Unigene2665\_Sample\_011046840, Unigene43231\_Sample\_011046840, Unigene30367\_Sample\_011046840, Unigene38801\_Sample\_011046840, Unigene13661\_Sample\_011046840, Unigene39273\_Sample\_011046840, Unigene37919\_Sample\_011046840, Unigene43210\_Sample\_011046840, Unigene31306\_Sample\_011046840, Unigene37192\_Sample\_011046840, Unigene29808\_Sample\_011046840, Unigene8217\_Sample\_011046840, Unigene40496\_Sample\_011046840, Unigene11931\_Sample\_011046840, Unigene41811\_Sample\_011046840, Unigene29362\_Sample\_011046840, Unigene13726\_Sample\_011046840, Unigene27185\_Sample\_011046840, Unigene38555\_Sample\_011046840, Unigene6925\_Sample\_011046840, Unigene38595\_Sample\_011046840, Unigene25467\_Sample\_011046840, Unigene39615\_Sample\_011046840, Unigene17602\_Sample\_011046840, Unigene32098\_Sample\_011046840, Unigene5385\_Sample\_011046840, Unigene42983\_Sample\_011046840, Unigene15428\_Sample\_011046840, Unigene40745\_Sample\_011046840, Unigene31705\_Sample\_011046840, Unigene35001\_Sample\_011046840, Unigene40260\_Sample\_011046840, Unigene41879\_Sample\_011046840, Unigene32783\_Sample\_011046840, Unigene32240\_Sample\_011046840, Unigene21551\_Sample\_011046840, Unigene5962\_Sample\_011046840, Unigene32573\_Sample\_011046840, Unigene34972\_Sample\_011046840, Unigene28616\_Sample\_011046840, Unigene33492\_Sample\_011046840, Unigene26448\_Sample\_011046840, Unigene37083\_Sample\_011046840, Unigene17486\_Sample\_011046840, Unigene43573\_Sample\_011046840, Unigene25450\_Sample\_011046840, Unigene7951\_Sample\_011046840, Unigene28129\_Sample\_011046840, Unigene7302\_Sample\_011046840, Unigene43120\_Sample\_011046840, Unigene39480\_Sample\_011046840, Unigene33745\_Sample\_011046840, Unigene3204\_Sample\_011046840, Unigene8085\_Sample\_011046840, Unigene31899\_Sample\_011046840, Unigene6101\_Sample\_011046840, Unigene19985\_Sample\_011046840, Unigene38217\_Sample\_011046840, Unigene43342\_Sample\_011046840, Unigene37484\_Sample\_011046840, Unigene19344\_Sample\_011046840, Unigene43502\_Sample\_011046840, Unigene27982\_Sample\_011046840, Unigene31409\_Sample\_011046840, Unigene33338\_Sample\_011046840, Unigene12677\_Sample\_011046840, Unigene26172\_Sample\_011046840, Unigene43410\_Sample\_011046840, Unigene28710\_Sample\_011046840, Unigene33618\_Sample\_011046840, Unigene19933\_Sample\_011046840, Unigene34693\_Sample\_011046840, Unigene34288\_Sample\_011046840, Unigene31198\_Sample\_011046840, Unigene43301\_Sample\_011046840, Unigene26124\_Sample\_011046840, Unigene38940\_Sample\_011046840, Unigene21513\_Sample\_011046840, Unigene41283\_Sample\_011046840, Unigene37828\_Sample\_011046840, Unigene36734\_Sample\_011046840, Unigene40253\_Sample\_011046840, Unigene1368\_Sample\_011046840, Unigene42041\_Sample\_011046840, Unigene41981\_Sample\_011046840, Unigene43405\_Sample\_011046840, Unigene29875\_Sample\_011046840, Unigene1872\_Sample\_011046840, Unigene4145\_Sample\_011046840, Unigene23682\_Sample\_011046840, Unigene8198\_Sample\_011046840, Unigene40895\_Sample\_011046840, Unigene31022\_Sample\_011046840, Unigene28610\_Sample\_011046840, Unigene40235\_Sample\_011046840, Unigene20869\_Sample\_011046840, Unigene39791\_Sample\_011046840, Unigene33873\_Sample\_011046840, Unigene40606\_Sample\_011046840, Unigene1379\_Sample\_011046840, Unigene41957\_Sample\_011046840, Unigene41383\_Sample\_011046840, Unigene26491\_Sample\_011046840, Unigene39843\_Sample\_011046840, Unigene3046\_Sample\_011046840, Unigene2709\_Sample\_011046840, Unigene37486\_Sample\_011046840, Unigene3212\_Sample\_011046840, Unigene3718\_Sample\_011046840, Unigene7203\_Sample\_011046840, Unigene13413\_Sample\_011046840, Unigene43289\_Sample\_011046840, Unigene36568\_Sample\_011046840, Unigene39778\_Sample\_011046840, Unigene22606\_Sample\_011046840, Unigene30720\_Sample\_011046840, Unigene14693\_Sample\_011046840, Unigene11201\_Sample\_011046840, Unigene15199\_Sample\_011046840, Unigene37213\_Sample\_011046840, Unigene7606\_Sample\_011046840, Unigene7813\_Sample\_011046840, Unigene332\_Sample\_011046840, Unigene7162\_Sample\_011046840, Unigene30599\_Sample\_011046840, Unigene36645\_Sample\_011046840, Unigene23950\_Sample\_011046840, Unigene36762\_Sample\_011046840, Unigene36732\_Sample\_011046840, Unigene41089\_Sample\_011046840, Unigene28034\_Sample\_011046840, Unigene24482\_Sample\_011046840, Unigene5166\_Sample\_011046840, Unigene20196\_Sample\_011046840, Unigene14589\_Sample\_011046840, Unigene19402\_Sample\_011046840, Unigene40547\_Sample\_011046840, Unigene39900\_Sample\_011046840, Unigene42906\_Sample\_011046840, Unigene16569\_Sample\_011046840, Unigene33705\_Sample\_011046840, Unigene38589\_Sample\_011046840, Unigene36323\_Sample\_011046840, Unigene36937\_Sample\_011046840, Unigene32067\_Sample\_011046840, Unigene40875\_Sample\_011046840, Unigene6764\_Sample\_011046840, Unigene20216\_Sample\_011046840, Unigene5336\_Sample\_011046840, Unigene36996\_Sample\_011046840, Unigene38250\_Sample\_011046840, Unigene33344\_Sample\_011046840, Unigene41584\_Sample\_011046840, Unigene27067\_Sample\_011046840, Unigene18456\_Sample\_011046840, Unigene4601\_Sample\_011046840, Unigene37506\_Sample\_011046840, Unigene39509\_Sample\_011046840, Unigene38767\_Sample\_011046840, Unigene5223\_Sample\_011046840, Unigene41805\_Sample\_011046840, Unigene38826\_Sample\_011046840, Unigene36211\_Sample\_011046840, Unigene43372\_Sample\_011046840, Unigene40587\_Sample\_011046840, Unigene39481\_Sample\_011046840, Unigene32614\_Sample\_011046840, Unigene26668\_Sample\_011046840, Unigene37136\_Sample\_011046840, Unigene29542\_Sample\_011046840, Unigene13327\_Sample\_011046840, Unigene42278\_Sample\_011046840, Unigene14602\_Sample\_011046840, Unigene40732\_Sample\_011046840, Unigene28853\_Sample\_011046840, Unigene8413\_Sample\_011046840, Unigene17765\_Sample\_011046840, Unigene42337\_Sample\_011046840, Unigene35167\_Sample\_011046840, Unigene41299\_Sample\_011046840, Unigene6283\_Sample\_011046840, Unigene27639\_Sample\_011046840, Unigene41061\_Sample\_011046840, Unigene11146\_Sample\_011046840, Unigene30715\_Sample\_011046840, Unigene37236\_Sample\_011046840, Unigene32566\_Sample\_011046840, Unigene33878\_Sample\_011046840, Unigene34566\_Sample\_011046840, Unigene26402\_Sample\_011046840, Unigene43585\_Sample\_011046840, Unigene38285\_Sample\_011046840, Unigene11248\_Sample\_011046840, Unigene14392\_Sample\_011046840, Unigene4139\_Sample\_011046840, Unigene36839\_Sample\_011046840, Unigene24400\_Sample\_011046840, Unigene36971\_Sample\_011046840, Unigene40950\_Sample\_011046840, Unigene31827\_Sample\_011046840, Unigene33088\_Sample\_011046840, Unigene36063\_Sample\_011046840, Unigene18251\_Sample\_011046840, Unigene42487\_Sample\_011046840, Unigene15903\_Sample\_011046840, Unigene35023\_Sample\_011046840, Unigene6842\_Sample\_011046840, Unigene32606\_Sample\_011046840, Unigene13197\_Sample\_011046840, Unigene5985\_Sample\_011046840, Unigene31283\_Sample\_011046840, Unigene41983\_Sample\_011046840, Unigene21387\_Sample\_011046840, Unigene27604\_Sample\_011046840, Unigene32554\_Sample\_011046840, Unigene17650\_Sample\_011046840, Unigene38526\_Sample\_011046840, Unigene35976\_Sample\_011046840, Unigene40764\_Sample\_011046840, Unigene27667\_Sample\_011046840, Unigene19027\_Sample\_011046840, Unigene7353\_Sample\_011046840, Unigene32180\_Sample\_011046840, Unigene20151\_Sample\_011046840, Unigene15237\_Sample\_011046840, Unigene40726\_Sample\_011046840, Unigene42638\_Sample\_011046840, Unigene19219\_Sample\_011046840, Unigene5707\_Sample\_011046840, Unigene17339\_Sample\_011046840, Unigene17541\_Sample\_011046840, Unigene35227\_Sample\_011046840, Unigene38242\_Sample\_011046840, Unigene33279\_Sample\_011046840, Unigene24546\_Sample\_011046840, Unigene43119\_Sample\_011046840, Unigene25298\_Sample\_011046840, Unigene31759\_Sample\_011046840, Unigene12410\_Sample\_011046840, Unigene38772\_Sample\_011046840, Unigene37440\_Sample\_011046840, Unigene28079\_Sample\_011046840, Unigene10436\_Sample\_011046840, Unigene43064\_Sample\_011046840, Unigene40520\_Sample\_011046840, Unigene32702\_Sample\_011046840, Unigene29405\_Sample\_011046840, Unigene39983\_Sample\_011046840, Unigene19991\_Sample\_011046840, Unigene42622\_Sample\_011046840, Unigene41793\_Sample\_011046840, Unigene39948\_Sample\_011046840, Unigene5658\_Sample\_011046840, Unigene33671\_Sample\_011046840, Unigene42763\_Sample\_011046840, Unigene38378\_Sample\_011046840, Unigene13641\_Sample\_011046840, Unigene6346\_Sample\_011046840, Unigene14981\_Sample\_011046840, Unigene3828\_Sample\_011046840, Unigene6395\_Sample\_011046840, Unigene2916\_Sample\_011046840, Unigene40716\_Sample\_011046840, Unigene29928\_Sample\_011046840, Unigene22209\_Sample\_011046840, Unigene11244\_Sample\_011046840, Unigene8189\_Sample\_011046840, Unigene40234\_Sample\_011046840, Unigene33379\_Sample\_011046840, Unigene39123\_Sample\_011046840, Unigene37694\_Sample\_011046840, Unigene12204\_Sample\_011046840, Unigene19740\_Sample\_011046840, Unigene4042\_Sample\_011046840, Unigene39967\_Sample\_011046840, Unigene43198\_Sample\_011046840, Unigene41086\_Sample\_011046840, Unigene11516\_Sample\_011046840, Unigene31973\_Sample\_011046840, Unigene39052\_Sample\_011046840, Unigene38279\_Sample\_011046840, Unigene25915\_Sample\_011046840, Unigene7631\_Sample\_011046840, Unigene30400\_Sample\_011046840, Unigene26572\_Sample\_011046840, Unigene41681\_Sample\_011046840, Unigene29592\_Sample\_011046840, Unigene3486\_Sample\_011046840, Unigene38262\_Sample\_011046840, Unigene34754\_Sample\_011046840, Unigene35053\_Sample\_011046840, Unigene42702\_Sample\_011046840, Unigene11883\_Sample\_011046840, Unigene42866\_Sample\_011046840, Unigene40010\_Sample\_011046840, Unigene37330\_Sample\_011046840, Unigene21296\_Sample\_011046840, Unigene7708\_Sample\_011046840, Unigene37921\_Sample\_011046840, Unigene20922\_Sample\_011046840, Unigene28301\_Sample\_011046840, Unigene41242\_Sample\_011046840, Unigene41510\_Sample\_011046840, Unigene27805\_Sample\_011046840, Unigene43165\_Sample\_011046840, Unigene32262\_Sample\_011046840, Unigene22393\_Sample\_011046840, Unigene16493\_Sample\_011046840, Unigene18168\_Sample\_011046840, Unigene11142\_Sample\_011046840, Unigene32307\_Sample\_011046840, Unigene42027\_Sample\_011046840, Unigene21554\_Sample\_011046840, Unigene8130\_Sample\_011046840, Unigene20146\_Sample\_011046840, Unigene7172\_Sample\_011046840, Unigene42096\_Sample\_011046840, Unigene5647\_Sample\_011046840, Unigene41869\_Sample\_011046840, Unigene19505\_Sample\_011046840, Unigene16302\_Sample\_011046840, Unigene36983\_Sample\_011046840, Unigene37827\_Sample\_011046840, Unigene24404\_Sample\_011046840, Unigene42692\_Sample\_011046840, Unigene40128\_Sample\_011046840, Unigene25020\_Sample\_011046840, Unigene43593\_Sample\_011046840, Unigene11571\_Sample\_011046840, Unigene28366\_Sample\_011046840, Unigene24019\_Sample\_011046840, Unigene7767\_Sample\_011046840, Unigene43201\_Sample\_011046840, Unigene41281\_Sample\_011046840, Unigene14437\_Sample\_011046840, Unigene7768\_Sample\_011046840, Unigene14257\_Sample\_011046840 |
| transmembrane transporter activity | Unigene5759\_Sample\_011046840, Unigene42993\_Sample\_011046840, Unigene32100\_Sample\_011046840, Unigene22255\_Sample\_011046840, Unigene15192\_Sample\_011046840, Unigene29099\_Sample\_011046840, Unigene9443\_Sample\_011046840, Unigene6332\_Sample\_011046840, Unigene41047\_Sample\_011046840, Unigene23426\_Sample\_011046840, Unigene43121\_Sample\_011046840, Unigene5485\_Sample\_011046840, Unigene36965\_Sample\_011046840, Unigene39213\_Sample\_011046840, Unigene5901\_Sample\_011046840, Unigene6964\_Sample\_011046840, Unigene12721\_Sample\_011046840, Unigene5625\_Sample\_011046840, Unigene14612\_Sample\_011046840, Unigene37745\_Sample\_011046840, Unigene24503\_Sample\_011046840, Unigene1295\_Sample\_011046840, Unigene24674\_Sample\_011046840, Unigene8271\_Sample\_011046840, Unigene40184\_Sample\_011046840, Unigene2490\_Sample\_011046840, Unigene20979\_Sample\_011046840, Unigene41763\_Sample\_011046840, Unigene43136\_Sample\_011046840, Unigene34978\_Sample\_011046840, Unigene38008\_Sample\_011046840, Unigene8179\_Sample\_011046840, Unigene39490\_Sample\_011046840, Unigene39632\_Sample\_011046840, Unigene37068\_Sample\_011046840, Unigene3403\_Sample\_011046840, Unigene27132\_Sample\_011046840, Unigene14012\_Sample\_011046840, Unigene39795\_Sample\_011046840, Unigene1053\_Sample\_011046840, Unigene15576\_Sample\_011046840, Unigene4881\_Sample\_011046840, Unigene24664\_Sample\_011046840, Unigene39978\_Sample\_011046840, Unigene2196\_Sample\_011046840, Unigene5219\_Sample\_011046840, Unigene39351\_Sample\_011046840, Unigene38610\_Sample\_011046840, Unigene39100\_Sample\_011046840, Unigene8034\_Sample\_011046840, Unigene25781\_Sample\_011046840, Unigene3889\_Sample\_011046840, Unigene41683\_Sample\_011046840, Unigene2784\_Sample\_011046840, Unigene4089\_Sample\_011046840, Unigene16615\_Sample\_011046840, Unigene7923\_Sample\_011046840, Unigene7086\_Sample\_011046840, Unigene7444\_Sample\_011046840, Unigene7954\_Sample\_011046840, Unigene20811\_Sample\_011046840, Unigene2663\_Sample\_011046840, Unigene12012\_Sample\_011046840, Unigene5914\_Sample\_011046840, Unigene18797\_Sample\_011046840, Unigene41628\_Sample\_011046840, Unigene42148\_Sample\_011046840, Unigene27749\_Sample\_011046840, Unigene29053\_Sample\_011046840, Unigene9004\_Sample\_011046840, Unigene42793\_Sample\_011046840, Unigene23365\_Sample\_011046840, Unigene6507\_Sample\_011046840, Unigene37786\_Sample\_011046840, Unigene4775\_Sample\_011046840, Unigene7612\_Sample\_011046840, Unigene31456\_Sample\_011046840, Unigene30535\_Sample\_011046840, Unigene38598\_Sample\_011046840, Unigene8803\_Sample\_011046840, Unigene26156\_Sample\_011046840, Unigene12084\_Sample\_011046840, Unigene41685\_Sample\_011046840, Unigene42530\_Sample\_011046840, Unigene4554\_Sample\_011046840, Unigene9481\_Sample\_011046840, Unigene41773\_Sample\_011046840, Unigene23758\_Sample\_011046840, Unigene16538\_Sample\_011046840, Unigene42122\_Sample\_011046840, Unigene13399\_Sample\_011046840, Unigene22996\_Sample\_011046840, Unigene6606\_Sample\_011046840, Unigene41788\_Sample\_011046840, Unigene35451\_Sample\_011046840, Unigene32675\_Sample\_011046840, Unigene37979\_Sample\_011046840, Unigene21470\_Sample\_011046840, Unigene5654\_Sample\_011046840, Unigene20758\_Sample\_011046840, Unigene23269\_Sample\_011046840, Unigene23610\_Sample\_011046840, Unigene20529\_Sample\_011046840, Unigene42901\_Sample\_011046840, Unigene41582\_Sample\_011046840, Unigene40178\_Sample\_011046840, Unigene36345\_Sample\_011046840, Unigene31215\_Sample\_011046840, Unigene13928\_Sample\_011046840, Unigene41412\_Sample\_011046840, Unigene19530\_Sample\_011046840, Unigene34105\_Sample\_011046840, Unigene33869\_Sample\_011046840, Unigene42723\_Sample\_011046840, Unigene39874\_Sample\_011046840, Unigene30452\_Sample\_011046840, Unigene7889\_Sample\_011046840, Unigene42395\_Sample\_011046840, Unigene3815\_Sample\_011046840, Unigene35554\_Sample\_011046840, Unigene42268\_Sample\_011046840, Unigene32733\_Sample\_011046840, Unigene39533\_Sample\_011046840, Unigene41689\_Sample\_011046840, Unigene23727\_Sample\_011046840, Unigene30044\_Sample\_011046840, Unigene31318\_Sample\_011046840, Unigene38188\_Sample\_011046840, Unigene5591\_Sample\_011046840, Unigene29386\_Sample\_011046840, Unigene30107\_Sample\_011046840, Unigene5894\_Sample\_011046840, Unigene31356\_Sample\_011046840, Unigene8452\_Sample\_011046840, Unigene43546\_Sample\_011046840, Unigene12033\_Sample\_011046840, Unigene21129\_Sample\_011046840, Unigene42177\_Sample\_011046840, Unigene28322\_Sample\_011046840, Unigene36000\_Sample\_011046840, Unigene5540\_Sample\_011046840, Unigene31447\_Sample\_011046840, Unigene31553\_Sample\_011046840, Unigene42838\_Sample\_011046840, Unigene9477\_Sample\_011046840, Unigene7931\_Sample\_011046840, Unigene93\_Sample\_011046840, Unigene31608\_Sample\_011046840, Unigene41898\_Sample\_011046840, Unigene36041\_Sample\_011046840, Unigene5831\_Sample\_011046840, Unigene7646\_Sample\_011046840, Unigene14113\_Sample\_011046840, Unigene4389\_Sample\_011046840, Unigene4327\_Sample\_011046840, Unigene36624\_Sample\_011046840, Unigene4123\_Sample\_011046840, Unigene36260\_Sample\_011046840, Unigene31273\_Sample\_011046840, Unigene36558\_Sample\_011046840, Unigene38081\_Sample\_011046840, Unigene32706\_Sample\_011046840, Unigene13699\_Sample\_011046840, Unigene7189\_Sample\_011046840, Unigene3851\_Sample\_011046840, Unigene2457\_Sample\_011046840, Unigene33119\_Sample\_011046840, Unigene36383\_Sample\_011046840, Unigene6547\_Sample\_011046840, Unigene26216\_Sample\_011046840, Unigene38987\_Sample\_011046840, Unigene31222\_Sample\_011046840, Unigene41332\_Sample\_011046840, Unigene32354\_Sample\_011046840, Unigene41117\_Sample\_011046840, Unigene39248\_Sample\_011046840, Unigene26961\_Sample\_011046840, Unigene36088\_Sample\_011046840, Unigene3532\_Sample\_011046840, Unigene19106\_Sample\_011046840, Unigene8113\_Sample\_011046840, Unigene40301\_Sample\_011046840, Unigene14473\_Sample\_011046840, Unigene1227\_Sample\_011046840, Unigene32197\_Sample\_011046840, Unigene3324\_Sample\_011046840, Unigene6313\_Sample\_011046840, Unigene17101\_Sample\_011046840, Unigene39499\_Sample\_011046840, Unigene34914\_Sample\_011046840, Unigene43384\_Sample\_011046840, Unigene6860\_Sample\_011046840, Unigene43583\_Sample\_011046840, Unigene8061\_Sample\_011046840, Unigene38145\_Sample\_011046840, Unigene41972\_Sample\_011046840, Unigene11308\_Sample\_011046840, Unigene23663\_Sample\_011046840, Unigene2277\_Sample\_011046840, Unigene30270\_Sample\_011046840, Unigene43110\_Sample\_011046840, Unigene38720\_Sample\_011046840, Unigene1312\_Sample\_011046840, Unigene31864\_Sample\_011046840, Unigene40826\_Sample\_011046840, Unigene22959\_Sample\_011046840, Unigene38118\_Sample\_011046840, Unigene9682\_Sample\_011046840, Unigene6500\_Sample\_011046840, Unigene33722\_Sample\_011046840, Unigene22499\_Sample\_011046840, Unigene33548\_Sample\_011046840, Unigene4452\_Sample\_011046840, Unigene2152\_Sample\_011046840, Unigene30788\_Sample\_011046840, Unigene2492\_Sample\_011046840, Unigene34278\_Sample\_011046840, Unigene39923\_Sample\_011046840, Unigene34993\_Sample\_011046840, Unigene8051\_Sample\_011046840, Unigene2422\_Sample\_011046840, Unigene34166\_Sample\_011046840, Unigene12675\_Sample\_011046840, Unigene369\_Sample\_011046840, Unigene37074\_Sample\_011046840, Unigene34807\_Sample\_011046840, Unigene8201\_Sample\_011046840, Unigene38472\_Sample\_011046840, Unigene41031\_Sample\_011046840, Unigene36847\_Sample\_011046840, Unigene34480\_Sample\_011046840, Unigene34930\_Sample\_011046840, Unigene24389\_Sample\_011046840, Unigene29794\_Sample\_011046840, Unigene33071\_Sample\_011046840, Unigene1884\_Sample\_011046840, Unigene31185\_Sample\_011046840, Unigene34612\_Sample\_011046840, Unigene6573\_Sample\_011046840, Unigene35412\_Sample\_011046840, Unigene19732\_Sample\_011046840, Unigene42421\_Sample\_011046840, Unigene41777\_Sample\_011046840, Unigene43041\_Sample\_011046840, Unigene30288\_Sample\_011046840, Unigene10704\_Sample\_011046840, Unigene28857\_Sample\_011046840, Unigene40574\_Sample\_011046840, Unigene42729\_Sample\_011046840, Unigene41449\_Sample\_011046840, Unigene33337\_Sample\_011046840, Unigene38900\_Sample\_011046840, Unigene22068\_Sample\_011046840, Unigene26485\_Sample\_011046840, Unigene4467\_Sample\_011046840, Unigene39121\_Sample\_011046840, Unigene30378\_Sample\_011046840, Unigene3791\_Sample\_011046840, Unigene39755\_Sample\_011046840, Unigene18838\_Sample\_011046840, Unigene21832\_Sample\_011046840, Unigene3188\_Sample\_011046840, Unigene6753\_Sample\_011046840, Unigene8148\_Sample\_011046840, Unigene35951\_Sample\_011046840, Unigene15680\_Sample\_011046840, Unigene20940\_Sample\_011046840, Unigene19531\_Sample\_011046840, Unigene1242\_Sample\_011046840, Unigene40032\_Sample\_011046840, Unigene42548\_Sample\_011046840, Unigene40403\_Sample\_011046840, Unigene23158\_Sample\_011046840, Unigene43475\_Sample\_011046840, Unigene1477\_Sample\_011046840, Unigene11476\_Sample\_011046840, Unigene21338\_Sample\_011046840, Unigene14501\_Sample\_011046840, Unigene37620\_Sample\_011046840, Unigene30629\_Sample\_011046840, Unigene16244\_Sample\_011046840, Unigene6659\_Sample\_011046840, Unigene36141\_Sample\_011046840, Unigene21938\_Sample\_011046840, Unigene15274\_Sample\_011046840, Unigene16545\_Sample\_011046840, Unigene5743\_Sample\_011046840, Unigene25046\_Sample\_011046840, Unigene35149\_Sample\_011046840, Unigene18176\_Sample\_011046840, Unigene27622\_Sample\_011046840, Unigene39777\_Sample\_011046840, Unigene4460\_Sample\_011046840, Unigene18522\_Sample\_011046840, Unigene39599\_Sample\_011046840, Unigene37075\_Sample\_011046840, Unigene40039\_Sample\_011046840, Unigene15163\_Sample\_011046840, Unigene37861\_Sample\_011046840, Unigene23926\_Sample\_011046840, Unigene6849\_Sample\_011046840, Unigene12708\_Sample\_011046840, Unigene24034\_Sample\_011046840, Unigene34400\_Sample\_011046840, Unigene1680\_Sample\_011046840, Unigene4371\_Sample\_011046840, Unigene36574\_Sample\_011046840, Unigene40445\_Sample\_011046840, Unigene6383\_Sample\_011046840, Unigene19704\_Sample\_011046840, Unigene43001\_Sample\_011046840, Unigene27829\_Sample\_011046840, Unigene43073\_Sample\_011046840, Unigene3854\_Sample\_011046840, Unigene34358\_Sample\_011046840, Unigene38028\_Sample\_011046840, Unigene257\_Sample\_011046840, Unigene8019\_Sample\_011046840, Unigene40428\_Sample\_011046840, Unigene38147\_Sample\_011046840, Unigene37992\_Sample\_011046840, Unigene7654\_Sample\_011046840, Unigene18919\_Sample\_011046840, Unigene7792\_Sample\_011046840, Unigene25057\_Sample\_011046840, Unigene975\_Sample\_011046840, Unigene17343\_Sample\_011046840, Unigene20070\_Sample\_011046840, Unigene2237\_Sample\_011046840, Unigene12950\_Sample\_011046840, Unigene42621\_Sample\_011046840, Unigene14178\_Sample\_011046840, Unigene38761\_Sample\_011046840, Unigene12111\_Sample\_011046840, Unigene23213\_Sample\_011046840, Unigene32819\_Sample\_011046840, Unigene40842\_Sample\_011046840, Unigene7422\_Sample\_011046840, Unigene36714\_Sample\_011046840, Unigene37249\_Sample\_011046840, Unigene3229\_Sample\_011046840, Unigene29631\_Sample\_011046840, Unigene43080\_Sample\_011046840, Unigene30232\_Sample\_011046840, Unigene17267\_Sample\_011046840, Unigene31662\_Sample\_011046840, Unigene8071\_Sample\_011046840, Unigene5760\_Sample\_011046840, Unigene41093\_Sample\_011046840, Unigene36158\_Sample\_011046840, Unigene39204\_Sample\_011046840, Unigene26951\_Sample\_011046840, Unigene25860\_Sample\_011046840, Unigene7804\_Sample\_011046840, Unigene14850\_Sample\_011046840, Unigene34737\_Sample\_011046840, Unigene35008\_Sample\_011046840, Unigene1022\_Sample\_011046840, Unigene9988\_Sample\_011046840, Unigene37629\_Sample\_011046840, Unigene28055\_Sample\_011046840, Unigene3539\_Sample\_011046840, Unigene40513\_Sample\_011046840, Unigene15030\_Sample\_011046840, Unigene37769\_Sample\_011046840, Unigene24456\_Sample\_011046840, Unigene13511\_Sample\_011046840, Unigene20042\_Sample\_011046840, Unigene38314\_Sample\_011046840, Unigene42904\_Sample\_011046840, Unigene3892\_Sample\_011046840, Unigene10979\_Sample\_011046840, Unigene32158\_Sample\_011046840, Unigene33360\_Sample\_011046840, Unigene7202\_Sample\_011046840, Unigene14977\_Sample\_011046840, Unigene28597\_Sample\_011046840, Unigene41252\_Sample\_011046840, Unigene17721\_Sample\_011046840, Unigene30566\_Sample\_011046840, Unigene13322\_Sample\_011046840, Unigene26581\_Sample\_011046840, Unigene41011\_Sample\_011046840, Unigene43123\_Sample\_011046840, Unigene36150\_Sample\_011046840, Unigene25\_Sample\_011046840, Unigene11195\_Sample\_011046840, Unigene43325\_Sample\_011046840, Unigene33313\_Sample\_011046840, Unigene33384\_Sample\_011046840, Unigene38677\_Sample\_011046840, Unigene34804\_Sample\_011046840, Unigene37173\_Sample\_011046840, Unigene39265\_Sample\_011046840, Unigene20790\_Sample\_011046840, Unigene40418\_Sample\_011046840, Unigene39541\_Sample\_011046840, Unigene18990\_Sample\_011046840, Unigene838\_Sample\_011046840, Unigene41720\_Sample\_011046840, Unigene19865\_Sample\_011046840, Unigene32932\_Sample\_011046840, Unigene6344\_Sample\_011046840, Unigene7994\_Sample\_011046840, Unigene11135\_Sample\_011046840, Unigene43028\_Sample\_011046840, Unigene18665\_Sample\_011046840, Unigene40729\_Sample\_011046840, Unigene39273\_Sample\_011046840, Unigene4518\_Sample\_011046840, Unigene33342\_Sample\_011046840, Unigene28640\_Sample\_011046840, Unigene23432\_Sample\_011046840, Unigene34506\_Sample\_011046840, Unigene35594\_Sample\_011046840, Unigene36653\_Sample\_011046840, Unigene39895\_Sample\_011046840, Unigene5915\_Sample\_011046840, Unigene29493\_Sample\_011046840, Unigene42846\_Sample\_011046840, Unigene34972\_Sample\_011046840, Unigene28867\_Sample\_011046840, Unigene25837\_Sample\_011046840, Unigene24133\_Sample\_011046840, Unigene21816\_Sample\_011046840, Unigene43573\_Sample\_011046840, Unigene16633\_Sample\_011046840, Unigene41642\_Sample\_011046840, Unigene7302\_Sample\_011046840, Unigene39608\_Sample\_011046840, Unigene39249\_Sample\_011046840, Unigene32585\_Sample\_011046840, Unigene3713\_Sample\_011046840, Unigene8190\_Sample\_011046840, Unigene39905\_Sample\_011046840, Unigene15594\_Sample\_011046840, Unigene35483\_Sample\_011046840, Unigene8109\_Sample\_011046840, Unigene22778\_Sample\_011046840, Unigene24801\_Sample\_011046840, Unigene43613\_Sample\_011046840, Unigene2195\_Sample\_011046840, Unigene38402\_Sample\_011046840, Unigene157\_Sample\_011046840, Unigene27719\_Sample\_011046840, Unigene36371\_Sample\_011046840, Unigene19647\_Sample\_011046840, Unigene36188\_Sample\_011046840, Unigene30334\_Sample\_011046840, Unigene4955\_Sample\_011046840, Unigene3827\_Sample\_011046840, Unigene40467\_Sample\_011046840, Unigene2245\_Sample\_011046840, Unigene4302\_Sample\_011046840, Unigene41462\_Sample\_011046840, Unigene37353\_Sample\_011046840, Unigene9893\_Sample\_011046840, Unigene38500\_Sample\_011046840, Unigene25769\_Sample\_011046840, Unigene30451\_Sample\_011046840, Unigene37336\_Sample\_011046840, Unigene36129\_Sample\_011046840, Unigene20538\_Sample\_011046840, Unigene13862\_Sample\_011046840, Unigene39065\_Sample\_011046840, Unigene2304\_Sample\_011046840, Unigene33192\_Sample\_011046840, Unigene36687\_Sample\_011046840, Unigene39683\_Sample\_011046840, Unigene37868\_Sample\_011046840, Unigene42439\_Sample\_011046840, Unigene13797\_Sample\_011046840, Unigene34527\_Sample\_011046840, Unigene43599\_Sample\_011046840, Unigene4500\_Sample\_011046840, Unigene26374\_Sample\_011046840, Unigene41305\_Sample\_011046840, Unigene35535\_Sample\_011046840, Unigene7606\_Sample\_011046840, Unigene21173\_Sample\_011046840, Unigene6919\_Sample\_011046840, Unigene7388\_Sample\_011046840, Unigene36762\_Sample\_011046840, Unigene12542\_Sample\_011046840, Unigene6351\_Sample\_011046840, Unigene37020\_Sample\_011046840, Unigene7411\_Sample\_011046840, Unigene25727\_Sample\_011046840, Unigene6317\_Sample\_011046840, Unigene17432\_Sample\_011046840, Unigene36673\_Sample\_011046840, Unigene1399\_Sample\_011046840, Unigene3307\_Sample\_011046840, Unigene27519\_Sample\_011046840, Unigene23275\_Sample\_011046840, Unigene4234\_Sample\_011046840, Unigene3926\_Sample\_011046840, Unigene34735\_Sample\_011046840, Unigene7477\_Sample\_011046840, Unigene5343\_Sample\_011046840, Unigene13113\_Sample\_011046840, Unigene27284\_Sample\_011046840, Unigene32614\_Sample\_011046840, Unigene26668\_Sample\_011046840, Unigene14116\_Sample\_011046840, Unigene5025\_Sample\_011046840, Unigene16000\_Sample\_011046840, Unigene32555\_Sample\_011046840, Unigene7123\_Sample\_011046840, Unigene37928\_Sample\_011046840, Unigene15064\_Sample\_011046840, Unigene4980\_Sample\_011046840, Unigene42337\_Sample\_011046840, Unigene28563\_Sample\_011046840, Unigene5643\_Sample\_011046840, Unigene31515\_Sample\_011046840, Unigene36488\_Sample\_011046840, Unigene42380\_Sample\_011046840, Unigene23331\_Sample\_011046840, Unigene16503\_Sample\_011046840, Unigene35374\_Sample\_011046840, Unigene18264\_Sample\_011046840, Unigene36661\_Sample\_011046840, Unigene2991\_Sample\_011046840, Unigene42048\_Sample\_011046840, Unigene29301\_Sample\_011046840, Unigene7412\_Sample\_011046840, Unigene24635\_Sample\_011046840, Unigene13869\_Sample\_011046840, Unigene28467\_Sample\_011046840, Unigene42873\_Sample\_011046840, Unigene1742\_Sample\_011046840, Unigene43564\_Sample\_011046840, Unigene4214\_Sample\_011046840, Unigene4481\_Sample\_011046840, Unigene23902\_Sample\_011046840, Unigene39912\_Sample\_011046840, Unigene36739\_Sample\_011046840, Unigene15860\_Sample\_011046840, Unigene36382\_Sample\_011046840, Unigene4368\_Sample\_011046840, Unigene29805\_Sample\_011046840, Unigene5979\_Sample\_011046840, Unigene39309\_Sample\_011046840, Unigene6202\_Sample\_011046840, Unigene26335\_Sample\_011046840, Unigene1671\_Sample\_011046840, Unigene15903\_Sample\_011046840, Unigene6318\_Sample\_011046840, Unigene5300\_Sample\_011046840, Unigene6241\_Sample\_011046840, Unigene38017\_Sample\_011046840, Unigene36473\_Sample\_011046840, Unigene31412\_Sample\_011046840, Unigene38609\_Sample\_011046840, Unigene39671\_Sample\_011046840, Unigene35976\_Sample\_011046840, Unigene34984\_Sample\_011046840, Unigene32198\_Sample\_011046840, Unigene5465\_Sample\_011046840, Unigene39342\_Sample\_011046840, Unigene2571\_Sample\_011046840, Unigene28681\_Sample\_011046840, Unigene3599\_Sample\_011046840, Unigene42769\_Sample\_011046840, Unigene15382\_Sample\_011046840, Unigene15932\_Sample\_011046840, Unigene25916\_Sample\_011046840, Unigene27997\_Sample\_011046840, Unigene31113\_Sample\_011046840, Unigene15659\_Sample\_011046840, Unigene2650\_Sample\_011046840, Unigene37511\_Sample\_011046840, Unigene25956\_Sample\_011046840, Unigene12410\_Sample\_011046840, Unigene28436\_Sample\_011046840, Unigene32702\_Sample\_011046840, Unigene20911\_Sample\_011046840, Unigene5974\_Sample\_011046840, Unigene2822\_Sample\_011046840, Unigene41688\_Sample\_011046840, Unigene24937\_Sample\_011046840, Unigene35507\_Sample\_011046840, Unigene38554\_Sample\_011046840, Unigene39285\_Sample\_011046840, Unigene40660\_Sample\_011046840, Unigene25612\_Sample\_011046840, Unigene30572\_Sample\_011046840, Unigene41653\_Sample\_011046840, Unigene34679\_Sample\_011046840, Unigene41359\_Sample\_011046840, Unigene19524\_Sample\_011046840, Unigene1879\_Sample\_011046840, Unigene5326\_Sample\_011046840, Unigene42616\_Sample\_011046840, Unigene43638\_Sample\_011046840, Unigene31698\_Sample\_011046840, Unigene22258\_Sample\_011046840, Unigene3344\_Sample\_011046840, Unigene41669\_Sample\_011046840, Unigene41498\_Sample\_011046840, Unigene38279\_Sample\_011046840, Unigene5628\_Sample\_011046840, Unigene33443\_Sample\_011046840, Unigene13\_Sample\_011046840, Unigene13452\_Sample\_011046840, Unigene25408\_Sample\_011046840, Unigene22824\_Sample\_011046840, Unigene37921\_Sample\_011046840, Unigene37833\_Sample\_011046840, Unigene42732\_Sample\_011046840, Unigene30240\_Sample\_011046840, Unigene6727\_Sample\_011046840, Unigene823\_Sample\_011046840, Unigene15362\_Sample\_011046840, Unigene40391\_Sample\_011046840, Unigene28898\_Sample\_011046840, Unigene11142\_Sample\_011046840, Unigene20710\_Sample\_011046840, Unigene7225\_Sample\_011046840, Unigene37347\_Sample\_011046840, Unigene40790\_Sample\_011046840, Unigene28685\_Sample\_011046840, Unigene5396\_Sample\_011046840, Unigene42218\_Sample\_011046840, Unigene7526\_Sample\_011046840, Unigene4462\_Sample\_011046840, Unigene27257\_Sample\_011046840, Unigene35435\_Sample\_011046840, Unigene35872\_Sample\_011046840, Unigene2310\_Sample\_011046840, Unigene5913\_Sample\_011046840, Unigene686\_Sample\_011046840, Unigene23327\_Sample\_011046840, Unigene40687\_Sample\_011046840, Unigene41415\_Sample\_011046840, Unigene40205\_Sample\_011046840, Unigene39837\_Sample\_011046840, Unigene33823\_Sample\_011046840, Unigene37371\_Sample\_011046840, Unigene18559\_Sample\_011046840, Unigene4735\_Sample\_011046840 |
| phosphoinositide binding | Unigene23607\_Sample\_011046840, Unigene35561\_Sample\_011046840, Unigene34854\_Sample\_011046840, Unigene16314\_Sample\_011046840, Unigene35477\_Sample\_011046840, Unigene41520\_Sample\_011046840, Unigene39318\_Sample\_011046840, Unigene25105\_Sample\_011046840, Unigene40399\_Sample\_011046840, Unigene34297\_Sample\_011046840, Unigene18553\_Sample\_011046840, Unigene21061\_Sample\_011046840, Unigene42297\_Sample\_011046840, Unigene5271\_Sample\_011046840, Unigene32547\_Sample\_011046840, Unigene28339\_Sample\_011046840, Unigene37194\_Sample\_011046840, Unigene32857\_Sample\_011046840, Unigene29255\_Sample\_011046840, Unigene8205\_Sample\_011046840, Unigene35475\_Sample\_011046840, Unigene18382\_Sample\_011046840, Unigene31506\_Sample\_011046840, Unigene8089\_Sample\_011046840, Unigene38322\_Sample\_011046840, Unigene42892\_Sample\_011046840, Unigene5390\_Sample\_011046840, Unigene23285\_Sample\_011046840, Unigene5388\_Sample\_011046840 |
| phosphoric ester hydrolase activity | Unigene30791\_Sample\_011046840, Unigene43390\_Sample\_011046840, Unigene42991\_Sample\_011046840, Unigene29269\_Sample\_011046840, Unigene6644\_Sample\_011046840, Unigene42869\_Sample\_011046840, Unigene42159\_Sample\_011046840, Unigene1548\_Sample\_011046840, Unigene7132\_Sample\_011046840, Unigene32488\_Sample\_011046840, Unigene23714\_Sample\_011046840, Unigene37121\_Sample\_011046840, Unigene37146\_Sample\_011046840, Unigene30415\_Sample\_011046840, Unigene35076\_Sample\_011046840, Unigene43355\_Sample\_011046840, Unigene4750\_Sample\_011046840, Unigene3682\_Sample\_011046840, Unigene23019\_Sample\_011046840, Unigene36859\_Sample\_011046840, Unigene42607\_Sample\_011046840, Unigene32062\_Sample\_011046840, Unigene17714\_Sample\_011046840, Unigene39685\_Sample\_011046840, Unigene17640\_Sample\_011046840, Unigene1019\_Sample\_011046840, Unigene31229\_Sample\_011046840, Unigene12436\_Sample\_011046840, Unigene2209\_Sample\_011046840, Unigene29095\_Sample\_011046840, Unigene3151\_Sample\_011046840, Unigene39006\_Sample\_011046840, Unigene6139\_Sample\_011046840, Unigene29586\_Sample\_011046840, Unigene3009\_Sample\_011046840, Unigene41003\_Sample\_011046840, Unigene42247\_Sample\_011046840, Unigene33837\_Sample\_011046840, Unigene31562\_Sample\_011046840, Unigene29655\_Sample\_011046840, Unigene41132\_Sample\_011046840, Unigene32138\_Sample\_011046840, Unigene14179\_Sample\_011046840, Unigene7554\_Sample\_011046840, Unigene41918\_Sample\_011046840, Unigene14077\_Sample\_011046840, Unigene42172\_Sample\_011046840, Unigene37235\_Sample\_011046840, Unigene36688\_Sample\_011046840, Unigene14255\_Sample\_011046840, Unigene40672\_Sample\_011046840, Unigene36910\_Sample\_011046840, Unigene31343\_Sample\_011046840, Unigene36359\_Sample\_011046840, Unigene39237\_Sample\_011046840, Unigene13531\_Sample\_011046840, Unigene6586\_Sample\_011046840, Unigene16605\_Sample\_011046840, Unigene42850\_Sample\_011046840, Unigene34076\_Sample\_011046840, Unigene7387\_Sample\_011046840, Unigene29065\_Sample\_011046840, Unigene35213\_Sample\_011046840, Unigene36980\_Sample\_011046840, Unigene41617\_Sample\_011046840, Unigene29521\_Sample\_011046840, Unigene2865\_Sample\_011046840, Unigene38799\_Sample\_011046840, Unigene17084\_Sample\_011046840, Unigene39711\_Sample\_011046840, Unigene6373\_Sample\_011046840, Unigene7926\_Sample\_011046840, Unigene42477\_Sample\_011046840, Unigene5969\_Sample\_011046840, Unigene27270\_Sample\_011046840, Unigene2383\_Sample\_011046840, Unigene6516\_Sample\_011046840, Unigene41960\_Sample\_011046840, Unigene40736\_Sample\_011046840, Unigene3044\_Sample\_011046840, Unigene35034\_Sample\_011046840, Unigene21908\_Sample\_011046840, Unigene7523\_Sample\_011046840, Unigene41250\_Sample\_011046840, Unigene16807\_Sample\_011046840, Unigene35403\_Sample\_011046840, Unigene6362\_Sample\_011046840, Unigene32978\_Sample\_011046840, Unigene33240\_Sample\_011046840, Unigene37582\_Sample\_011046840, Unigene15205\_Sample\_011046840, Unigene42728\_Sample\_011046840, Unigene34085\_Sample\_011046840, Unigene4477\_Sample\_011046840, Unigene13146\_Sample\_011046840, Unigene31514\_Sample\_011046840, Unigene15328\_Sample\_011046840, Unigene39046\_Sample\_011046840, Unigene34472\_Sample\_011046840, Unigene41420\_Sample\_011046840, Unigene28542\_Sample\_011046840, Unigene31314\_Sample\_011046840, Unigene7974\_Sample\_011046840, Unigene41582\_Sample\_011046840, Unigene40139\_Sample\_011046840, Unigene3812\_Sample\_011046840, Unigene41243\_Sample\_011046840, Unigene38621\_Sample\_011046840, Unigene39188\_Sample\_011046840, Unigene15250\_Sample\_011046840, Unigene34487\_Sample\_011046840, Unigene14227\_Sample\_011046840, Unigene27934\_Sample\_011046840, Unigene1843\_Sample\_011046840, Unigene39291\_Sample\_011046840, Unigene41218\_Sample\_011046840, Unigene40517\_Sample\_011046840, Unigene17052\_Sample\_011046840, Unigene634\_Sample\_011046840, Unigene33473\_Sample\_011046840, Unigene5877\_Sample\_011046840, Unigene36662\_Sample\_011046840, Unigene13908\_Sample\_011046840, Unigene38977\_Sample\_011046840, Unigene10992\_Sample\_011046840, Unigene7372\_Sample\_011046840, Unigene38646\_Sample\_011046840, Unigene25761\_Sample\_011046840, Unigene13900\_Sample\_011046840, Unigene29184\_Sample\_011046840, Unigene41425\_Sample\_011046840, Unigene7408\_Sample\_011046840, Unigene42231\_Sample\_011046840, Unigene4994\_Sample\_011046840, Unigene31356\_Sample\_011046840, Unigene4458\_Sample\_011046840, Unigene36623\_Sample\_011046840, Unigene43636\_Sample\_011046840, Unigene36519\_Sample\_011046840, Unigene34602\_Sample\_011046840, Unigene7207\_Sample\_011046840, Unigene29171\_Sample\_011046840, Unigene37605\_Sample\_011046840, Unigene8031\_Sample\_011046840, Unigene28046\_Sample\_011046840, Unigene3689\_Sample\_011046840, Unigene33594\_Sample\_011046840, Unigene18925\_Sample\_011046840, Unigene4466\_Sample\_011046840, Unigene28116\_Sample\_011046840, Unigene34624\_Sample\_011046840, Unigene4969\_Sample\_011046840, Unigene39328\_Sample\_011046840, Unigene38859\_Sample\_011046840, Unigene26887\_Sample\_011046840, Unigene36606\_Sample\_011046840, Unigene29334\_Sample\_011046840, Unigene40146\_Sample\_011046840, Unigene8094\_Sample\_011046840, Unigene37296\_Sample\_011046840, Unigene20473\_Sample\_011046840, Unigene37680\_Sample\_011046840, Unigene23356\_Sample\_011046840, Unigene7555\_Sample\_011046840, Unigene40045\_Sample\_011046840, Unigene41853\_Sample\_011046840, Unigene25603\_Sample\_011046840, Unigene7114\_Sample\_011046840, Unigene39730\_Sample\_011046840, Unigene39738\_Sample\_011046840, Unigene32001\_Sample\_011046840, Unigene12229\_Sample\_011046840, Unigene4192\_Sample\_011046840, Unigene4033\_Sample\_011046840, Unigene22971\_Sample\_011046840, Unigene22254\_Sample\_011046840, Unigene4069\_Sample\_011046840, Unigene36831\_Sample\_011046840, Unigene41059\_Sample\_011046840, Unigene11437\_Sample\_011046840, Unigene1967\_Sample\_011046840, Unigene10803\_Sample\_011046840, Unigene43013\_Sample\_011046840, Unigene1544\_Sample\_011046840, Unigene2080\_Sample\_011046840, Unigene37304\_Sample\_011046840, Unigene14438\_Sample\_011046840, Unigene42323\_Sample\_011046840, Unigene24223\_Sample\_011046840, Unigene28284\_Sample\_011046840, Unigene14845\_Sample\_011046840, Unigene42074\_Sample\_011046840, Unigene3108\_Sample\_011046840, Unigene16687\_Sample\_011046840, Unigene3448\_Sample\_011046840, Unigene26994\_Sample\_011046840, Unigene28344\_Sample\_011046840, Unigene39315\_Sample\_011046840, Unigene41818\_Sample\_011046840, Unigene33191\_Sample\_011046840, Unigene30604\_Sample\_011046840, Unigene39096\_Sample\_011046840, Unigene27425\_Sample\_011046840, Unigene27202\_Sample\_011046840, Unigene26409\_Sample\_011046840, Unigene29993\_Sample\_011046840, Unigene41488\_Sample\_011046840, Unigene10748\_Sample\_011046840, Unigene30703\_Sample\_011046840, Unigene26329\_Sample\_011046840, Unigene38876\_Sample\_011046840, Unigene42217\_Sample\_011046840, Unigene123\_Sample\_011046840, Unigene6735\_Sample\_011046840, Unigene40550\_Sample\_011046840, Unigene32318\_Sample\_011046840, Unigene42573\_Sample\_011046840, Unigene39660\_Sample\_011046840, Unigene41592\_Sample\_011046840, Unigene15\_Sample\_011046840, Unigene43138\_Sample\_011046840, Unigene13576\_Sample\_011046840, Unigene35122\_Sample\_011046840, Unigene23528\_Sample\_011046840, Unigene9862\_Sample\_011046840, Unigene41121\_Sample\_011046840, Unigene7010\_Sample\_011046840, Unigene19049\_Sample\_011046840, Unigene4352\_Sample\_011046840, Unigene2536\_Sample\_011046840, Unigene34993\_Sample\_011046840, Unigene41224\_Sample\_011046840, Unigene35596\_Sample\_011046840, Unigene17482\_Sample\_011046840, Unigene43202\_Sample\_011046840, Unigene4559\_Sample\_011046840, Unigene24618\_Sample\_011046840, Unigene6461\_Sample\_011046840, Unigene29181\_Sample\_011046840, Unigene7180\_Sample\_011046840, Unigene2690\_Sample\_011046840, Unigene4710\_Sample\_011046840, Unigene27625\_Sample\_011046840, Unigene38782\_Sample\_011046840, Unigene43265\_Sample\_011046840, Unigene19732\_Sample\_011046840, Unigene32855\_Sample\_011046840, Unigene4635\_Sample\_011046840, Unigene8053\_Sample\_011046840, Unigene42149\_Sample\_011046840, Unigene43417\_Sample\_011046840, Unigene3474\_Sample\_011046840, Unigene6926\_Sample\_011046840, Unigene23875\_Sample\_011046840, Unigene40617\_Sample\_011046840, Unigene28872\_Sample\_011046840, Unigene132\_Sample\_011046840, Unigene26046\_Sample\_011046840, Unigene22642\_Sample\_011046840, Unigene8055\_Sample\_011046840, Unigene41248\_Sample\_011046840, Unigene10400\_Sample\_011046840, Unigene36503\_Sample\_011046840, Unigene40057\_Sample\_011046840, Unigene25217\_Sample\_011046840, Unigene27233\_Sample\_011046840, Unigene16532\_Sample\_011046840, Unigene2718\_Sample\_011046840, Unigene28124\_Sample\_011046840, Unigene39384\_Sample\_011046840, Unigene8156\_Sample\_011046840, Unigene32610\_Sample\_011046840, Unigene40454\_Sample\_011046840, Unigene29775\_Sample\_011046840, Unigene24890\_Sample\_011046840, Unigene37248\_Sample\_011046840, Unigene1740\_Sample\_011046840, Unigene42297\_Sample\_011046840, Unigene21914\_Sample\_011046840, Unigene35942\_Sample\_011046840, Unigene25448\_Sample\_011046840, Unigene33579\_Sample\_011046840, Unigene38340\_Sample\_011046840, Unigene36137\_Sample\_011046840, Unigene30384\_Sample\_011046840, Unigene13348\_Sample\_011046840, Unigene41674\_Sample\_011046840, Unigene1533\_Sample\_011046840, Unigene13067\_Sample\_011046840, Unigene7868\_Sample\_011046840, Unigene32864\_Sample\_011046840, Unigene2891\_Sample\_011046840, Unigene6088\_Sample\_011046840, Unigene34967\_Sample\_011046840, Unigene2924\_Sample\_011046840, Unigene42709\_Sample\_011046840, Unigene41550\_Sample\_011046840, Unigene12710\_Sample\_011046840, Unigene20174\_Sample\_011046840, Unigene33865\_Sample\_011046840, Unigene2123\_Sample\_011046840, Unigene18059\_Sample\_011046840, Unigene5988\_Sample\_011046840, Unigene1129\_Sample\_011046840, Unigene2295\_Sample\_011046840, Unigene28759\_Sample\_011046840, Unigene40028\_Sample\_011046840, Unigene34638\_Sample\_011046840, Unigene41204\_Sample\_011046840, Unigene21758\_Sample\_011046840, Unigene8614\_Sample\_011046840, Unigene14213\_Sample\_011046840, Unigene21311\_Sample\_011046840, Unigene2654\_Sample\_011046840, Unigene5294\_Sample\_011046840, Unigene1602\_Sample\_011046840, Unigene39586\_Sample\_011046840, Unigene24606\_Sample\_011046840, Unigene14465\_Sample\_011046840, Unigene11615\_Sample\_011046840, Unigene37697\_Sample\_011046840, Unigene24621\_Sample\_011046840, Unigene27961\_Sample\_011046840, Unigene38362\_Sample\_011046840, Unigene39053\_Sample\_011046840, Unigene7144\_Sample\_011046840, Unigene28899\_Sample\_011046840, Unigene23373\_Sample\_011046840, Unigene41024\_Sample\_011046840, Unigene41388\_Sample\_011046840, Unigene33401\_Sample\_011046840, Unigene37310\_Sample\_011046840, Unigene35687\_Sample\_011046840 |
| purine nucleoside binding | Unigene38023\_Sample\_011046840, Unigene26178\_Sample\_011046840, Unigene39461\_Sample\_011046840, Unigene20405\_Sample\_011046840, Unigene38706\_Sample\_011046840, Unigene9443\_Sample\_011046840, Unigene368\_Sample\_011046840, Unigene3429\_Sample\_011046840, Unigene37568\_Sample\_011046840, Unigene19401\_Sample\_011046840, Unigene41047\_Sample\_011046840, Unigene18730\_Sample\_011046840, Unigene14932\_Sample\_011046840, Unigene43154\_Sample\_011046840, Unigene902\_Sample\_011046840, Unigene43529\_Sample\_011046840, Unigene27378\_Sample\_011046840, Unigene22180\_Sample\_011046840, Unigene43365\_Sample\_011046840, Unigene20380\_Sample\_011046840, Unigene21224\_Sample\_011046840, Unigene27537\_Sample\_011046840, Unigene7557\_Sample\_011046840, Unigene42372\_Sample\_011046840, Unigene31094\_Sample\_011046840, Unigene41153\_Sample\_011046840, Unigene20314\_Sample\_011046840, Unigene32455\_Sample\_011046840, Unigene15132\_Sample\_011046840, Unigene42642\_Sample\_011046840, Unigene35962\_Sample\_011046840, Unigene4259\_Sample\_011046840, Unigene4677\_Sample\_011046840, Unigene36928\_Sample\_011046840, Unigene24531\_Sample\_011046840, Unigene32335\_Sample\_011046840, Unigene42401\_Sample\_011046840, Unigene25485\_Sample\_011046840, Unigene6748\_Sample\_011046840, Unigene34978\_Sample\_011046840, Unigene8081\_Sample\_011046840, Unigene34971\_Sample\_011046840, Unigene37185\_Sample\_011046840, Unigene30370\_Sample\_011046840, Unigene32811\_Sample\_011046840, Unigene12614\_Sample\_011046840, Unigene1458\_Sample\_011046840, Unigene17494\_Sample\_011046840, Unigene21883\_Sample\_011046840, Unigene37375\_Sample\_011046840, Unigene8178\_Sample\_011046840, Unigene36909\_Sample\_011046840, Unigene1132\_Sample\_011046840, Unigene21176\_Sample\_011046840, Unigene25680\_Sample\_011046840, Unigene38973\_Sample\_011046840, Unigene7529\_Sample\_011046840, Unigene22101\_Sample\_011046840, Unigene40181\_Sample\_011046840, Unigene39544\_Sample\_011046840, Unigene3849\_Sample\_011046840, Unigene38463\_Sample\_011046840, Unigene177\_Sample\_011046840, Unigene41833\_Sample\_011046840, Unigene4089\_Sample\_011046840, Unigene14294\_Sample\_011046840, Unigene30285\_Sample\_011046840, Unigene36764\_Sample\_011046840, Unigene37848\_Sample\_011046840, Unigene14393\_Sample\_011046840, Unigene5726\_Sample\_011046840, Unigene38504\_Sample\_011046840, Unigene36848\_Sample\_011046840, Unigene19725\_Sample\_011046840, Unigene37095\_Sample\_011046840, Unigene30893\_Sample\_011046840, Unigene36359\_Sample\_011046840, Unigene3319\_Sample\_011046840, Unigene13306\_Sample\_011046840, Unigene24816\_Sample\_011046840, Unigene20757\_Sample\_011046840, Unigene24103\_Sample\_011046840, Unigene33428\_Sample\_011046840, Unigene24254\_Sample\_011046840, Unigene7831\_Sample\_011046840, Unigene30203\_Sample\_011046840, Unigene893\_Sample\_011046840, Unigene43435\_Sample\_011046840, Unigene30471\_Sample\_011046840, Unigene40159\_Sample\_011046840, Unigene29784\_Sample\_011046840, Unigene39812\_Sample\_011046840, Unigene27998\_Sample\_011046840, Unigene19834\_Sample\_011046840, Unigene42253\_Sample\_011046840, Unigene29947\_Sample\_011046840, Unigene17955\_Sample\_011046840, Unigene34234\_Sample\_011046840, Unigene25555\_Sample\_011046840, Unigene42502\_Sample\_011046840, Unigene41104\_Sample\_011046840, Unigene11113\_Sample\_011046840, Unigene41150\_Sample\_011046840, Unigene4830\_Sample\_011046840, Unigene41496\_Sample\_011046840, Unigene16527\_Sample\_011046840, Unigene43077\_Sample\_011046840, Unigene40073\_Sample\_011046840, Unigene37822\_Sample\_011046840, Unigene9565\_Sample\_011046840, Unigene12101\_Sample\_011046840, Unigene5249\_Sample\_011046840, Unigene31599\_Sample\_011046840, Unigene22020\_Sample\_011046840, Unigene16161\_Sample\_011046840, Unigene5118\_Sample\_011046840, Unigene35603\_Sample\_011046840, Unigene16645\_Sample\_011046840, Unigene32978\_Sample\_011046840, Unigene42351\_Sample\_011046840, Unigene33208\_Sample\_011046840, Unigene36893\_Sample\_011046840, Unigene36637\_Sample\_011046840, Unigene30615\_Sample\_011046840, Unigene18599\_Sample\_011046840, Unigene29270\_Sample\_011046840, Unigene13215\_Sample\_011046840, Unigene4578\_Sample\_011046840, Unigene15884\_Sample\_011046840, Unigene43230\_Sample\_011046840, Unigene42704\_Sample\_011046840, Unigene40406\_Sample\_011046840, Unigene37855\_Sample\_011046840, Unigene42242\_Sample\_011046840, Unigene20758\_Sample\_011046840, Unigene39911\_Sample\_011046840, Unigene36345\_Sample\_011046840, Unigene31215\_Sample\_011046840, Unigene41932\_Sample\_011046840, Unigene34105\_Sample\_011046840, Unigene20100\_Sample\_011046840, Unigene5001\_Sample\_011046840, Unigene39763\_Sample\_011046840, Unigene8801\_Sample\_011046840, Unigene37863\_Sample\_011046840, Unigene8554\_Sample\_011046840, Unigene39218\_Sample\_011046840, Unigene41866\_Sample\_011046840, Unigene39982\_Sample\_011046840, Unigene20594\_Sample\_011046840, Unigene43537\_Sample\_011046840, Unigene20\_Sample\_011046840, Unigene33968\_Sample\_011046840, Unigene30239\_Sample\_011046840, Unigene37613\_Sample\_011046840, Unigene32733\_Sample\_011046840, Unigene37902\_Sample\_011046840, Unigene3770\_Sample\_011046840, Unigene1222\_Sample\_011046840, Unigene17734\_Sample\_011046840, Unigene38066\_Sample\_011046840, Unigene38847\_Sample\_011046840, Unigene26711\_Sample\_011046840, Unigene38443\_Sample\_011046840, Unigene18659\_Sample\_011046840, Unigene32337\_Sample\_011046840, Unigene20968\_Sample\_011046840, Unigene29802\_Sample\_011046840, Unigene42061\_Sample\_011046840, Unigene33983\_Sample\_011046840, Unigene6796\_Sample\_011046840, Unigene20491\_Sample\_011046840, Unigene35734\_Sample\_011046840, Unigene4994\_Sample\_011046840, Unigene33913\_Sample\_011046840, Unigene41476\_Sample\_011046840, Unigene13127\_Sample\_011046840, Unigene41389\_Sample\_011046840, Unigene4309\_Sample\_011046840, Unigene36619\_Sample\_011046840, Unigene16211\_Sample\_011046840, Unigene38206\_Sample\_011046840, Unigene9477\_Sample\_011046840, Unigene43332\_Sample\_011046840, Unigene41743\_Sample\_011046840, Unigene29953\_Sample\_011046840, Unigene38834\_Sample\_011046840, Unigene20493\_Sample\_011046840, Unigene32099\_Sample\_011046840, Unigene41898\_Sample\_011046840, Unigene37706\_Sample\_011046840, Unigene31090\_Sample\_011046840, Unigene3003\_Sample\_011046840, Unigene42374\_Sample\_011046840, Unigene39024\_Sample\_011046840, Unigene15465\_Sample\_011046840, Unigene4389\_Sample\_011046840, Unigene41997\_Sample\_011046840, Unigene38687\_Sample\_011046840, Unigene5116\_Sample\_011046840, Unigene40471\_Sample\_011046840, Unigene42605\_Sample\_011046840, Unigene41161\_Sample\_011046840, Unigene34519\_Sample\_011046840, Unigene33595\_Sample\_011046840, Unigene31273\_Sample\_011046840, Unigene29985\_Sample\_011046840, Unigene907\_Sample\_011046840, Unigene33768\_Sample\_011046840, Unigene10236\_Sample\_011046840, Unigene522\_Sample\_011046840, Unigene36897\_Sample\_011046840, Unigene29903\_Sample\_011046840, Unigene29839\_Sample\_011046840, Unigene2457\_Sample\_011046840, Unigene26813\_Sample\_011046840, Unigene6547\_Sample\_011046840, Unigene26216\_Sample\_011046840, Unigene34870\_Sample\_011046840, Unigene35061\_Sample\_011046840, Unigene279\_Sample\_011046840, Unigene42759\_Sample\_011046840, Unigene2459\_Sample\_011046840, Unigene36366\_Sample\_011046840, Unigene19628\_Sample\_011046840, Unigene38962\_Sample\_011046840, Unigene35913\_Sample\_011046840, Unigene34002\_Sample\_011046840, Unigene40216\_Sample\_011046840, Unigene41431\_Sample\_011046840, Unigene42859\_Sample\_011046840, Unigene30600\_Sample\_011046840, Unigene29629\_Sample\_011046840, Unigene43055\_Sample\_011046840, Unigene23556\_Sample\_011046840, Unigene15492\_Sample\_011046840, Unigene5087\_Sample\_011046840, Unigene20079\_Sample\_011046840, Unigene32197\_Sample\_011046840, Unigene6313\_Sample\_011046840, Unigene31572\_Sample\_011046840, Unigene27920\_Sample\_011046840, Unigene9002\_Sample\_011046840, Unigene39499\_Sample\_011046840, Unigene14420\_Sample\_011046840, Unigene39061\_Sample\_011046840, Unigene27158\_Sample\_011046840, Unigene24281\_Sample\_011046840, Unigene38441\_Sample\_011046840, Unigene10533\_Sample\_011046840, Unigene37904\_Sample\_011046840, Unigene43606\_Sample\_011046840, Unigene34854\_Sample\_011046840, Unigene37976\_Sample\_011046840, Unigene42547\_Sample\_011046840, Unigene10832\_Sample\_011046840, Unigene15502\_Sample\_011046840, Unigene2792\_Sample\_011046840, Unigene37545\_Sample\_011046840, Unigene41919\_Sample\_011046840, Unigene4779\_Sample\_011046840, Unigene10749\_Sample\_011046840, Unigene25548\_Sample\_011046840, Unigene14498\_Sample\_011046840, Unigene39972\_Sample\_011046840, Unigene41368\_Sample\_011046840, Unigene5372\_Sample\_011046840, Unigene38709\_Sample\_011046840, Unigene38720\_Sample\_011046840, Unigene31864\_Sample\_011046840, Unigene37214\_Sample\_011046840, Unigene43188\_Sample\_011046840, Unigene39660\_Sample\_011046840, Unigene36990\_Sample\_011046840, Unigene4344\_Sample\_011046840, Unigene39243\_Sample\_011046840, Unigene33012\_Sample\_011046840, Unigene37292\_Sample\_011046840, Unigene9682\_Sample\_011046840, Unigene43312\_Sample\_011046840, Unigene32292\_Sample\_011046840, Unigene35790\_Sample\_011046840, Unigene32209\_Sample\_011046840, Unigene16376\_Sample\_011046840, Unigene17229\_Sample\_011046840, Unigene30473\_Sample\_011046840, Unigene39165\_Sample\_011046840, Unigene15033\_Sample\_011046840, Unigene43000\_Sample\_011046840, Unigene30860\_Sample\_011046840, Unigene26740\_Sample\_011046840, Unigene16473\_Sample\_011046840, Unigene3551\_Sample\_011046840, Unigene1008\_Sample\_011046840, Unigene5570\_Sample\_011046840, Unigene40679\_Sample\_011046840, Unigene37870\_Sample\_011046840, Unigene26427\_Sample\_011046840, Unigene42499\_Sample\_011046840, Unigene16770\_Sample\_011046840, Unigene543\_Sample\_011046840, Unigene10730\_Sample\_011046840, Unigene31855\_Sample\_011046840, Unigene8201\_Sample\_011046840, Unigene29526\_Sample\_011046840, Unigene33605\_Sample\_011046840, Unigene33711\_Sample\_011046840, Unigene43632\_Sample\_011046840, Unigene34669\_Sample\_011046840, Unigene35906\_Sample\_011046840, Unigene898\_Sample\_011046840, Unigene43635\_Sample\_011046840, Unigene34301\_Sample\_011046840, Unigene35346\_Sample\_011046840, Unigene5439\_Sample\_011046840, Unigene38337\_Sample\_011046840, Unigene32844\_Sample\_011046840, Unigene1375\_Sample\_011046840, Unigene34436\_Sample\_011046840, Unigene1731\_Sample\_011046840, Unigene16611\_Sample\_011046840, Unigene8083\_Sample\_011046840, Unigene10704\_Sample\_011046840, Unigene26270\_Sample\_011046840, Unigene7744\_Sample\_011046840, Unigene43117\_Sample\_011046840, Unigene25644\_Sample\_011046840, Unigene27239\_Sample\_011046840, Unigene7065\_Sample\_011046840, Unigene15181\_Sample\_011046840, Unigene34833\_Sample\_011046840, Unigene7175\_Sample\_011046840, Unigene7395\_Sample\_011046840, Unigene23201\_Sample\_011046840, Unigene8227\_Sample\_011046840, Unigene22068\_Sample\_011046840, Unigene7410\_Sample\_011046840, Unigene39018\_Sample\_011046840, Unigene14074\_Sample\_011046840, Unigene42615\_Sample\_011046840, Unigene36773\_Sample\_011046840, Unigene17905\_Sample\_011046840, Unigene14139\_Sample\_011046840, Unigene8006\_Sample\_011046840, Unigene2797\_Sample\_011046840, Unigene40658\_Sample\_011046840, Unigene25347\_Sample\_011046840, Unigene40979\_Sample\_011046840, Unigene35547\_Sample\_011046840, Unigene20556\_Sample\_011046840, Unigene22514\_Sample\_011046840, Unigene30760\_Sample\_011046840, Unigene40427\_Sample\_011046840, Unigene2810\_Sample\_011046840, Unigene33019\_Sample\_011046840, Unigene40032\_Sample\_011046840, Unigene33870\_Sample\_011046840, Unigene37571\_Sample\_011046840, Unigene36877\_Sample\_011046840, Unigene15851\_Sample\_011046840, Unigene4839\_Sample\_011046840, Unigene39740\_Sample\_011046840, Unigene42384\_Sample\_011046840, Unigene27359\_Sample\_011046840, Unigene23158\_Sample\_011046840, Unigene42441\_Sample\_011046840, Unigene35031\_Sample\_011046840, Unigene20101\_Sample\_011046840, Unigene42659\_Sample\_011046840, Unigene37034\_Sample\_011046840, Unigene38074\_Sample\_011046840, Unigene40484\_Sample\_011046840, Unigene18009\_Sample\_011046840, Unigene36141\_Sample\_011046840, Unigene42234\_Sample\_011046840, Unigene23241\_Sample\_011046840, Unigene10778\_Sample\_011046840, Unigene39927\_Sample\_011046840, Unigene5564\_Sample\_011046840, Unigene36685\_Sample\_011046840, Unigene41585\_Sample\_011046840, Unigene15148\_Sample\_011046840, Unigene4032\_Sample\_011046840, Unigene33943\_Sample\_011046840, Unigene43082\_Sample\_011046840, Unigene36922\_Sample\_011046840, Unigene7796\_Sample\_011046840, Unigene5007\_Sample\_011046840, Unigene15874\_Sample\_011046840, Unigene34588\_Sample\_011046840, Unigene8203\_Sample\_011046840, Unigene38501\_Sample\_011046840, Unigene40793\_Sample\_011046840, Unigene22867\_Sample\_011046840, Unigene42340\_Sample\_011046840, Unigene41400\_Sample\_011046840, Unigene35494\_Sample\_011046840, Unigene12635\_Sample\_011046840, Unigene36375\_Sample\_011046840, Unigene24034\_Sample\_011046840, Unigene24711\_Sample\_011046840, Unigene2227\_Sample\_011046840, Unigene42189\_Sample\_011046840, Unigene24309\_Sample\_011046840, Unigene41899\_Sample\_011046840, Unigene33906\_Sample\_011046840, Unigene28095\_Sample\_011046840, Unigene36121\_Sample\_011046840, Unigene8240\_Sample\_011046840, Unigene27829\_Sample\_011046840, Unigene40541\_Sample\_011046840, Unigene18069\_Sample\_011046840, Unigene41399\_Sample\_011046840, Unigene12936\_Sample\_011046840, Unigene29046\_Sample\_011046840, Unigene40917\_Sample\_011046840, Unigene42075\_Sample\_011046840, Unigene38028\_Sample\_011046840, Unigene11264\_Sample\_011046840, Unigene41676\_Sample\_011046840, Unigene35179\_Sample\_011046840, Unigene17789\_Sample\_011046840, Unigene40221\_Sample\_011046840, Unigene12818\_Sample\_011046840, Unigene7262\_Sample\_011046840, Unigene42206\_Sample\_011046840, Unigene16016\_Sample\_011046840, Unigene12035\_Sample\_011046840, Unigene2808\_Sample\_011046840, Unigene40596\_Sample\_011046840, Unigene1771\_Sample\_011046840, Unigene972\_Sample\_011046840, Unigene8555\_Sample\_011046840, Unigene7148\_Sample\_011046840, Unigene2084\_Sample\_011046840, Unigene39583\_Sample\_011046840, Unigene38393\_Sample\_011046840, Unigene35532\_Sample\_011046840, Unigene32382\_Sample\_011046840, Unigene2815\_Sample\_011046840, Unigene7669\_Sample\_011046840, Unigene3631\_Sample\_011046840, Unigene31663\_Sample\_011046840, Unigene24311\_Sample\_011046840, Unigene14178\_Sample\_011046840, Unigene17776\_Sample\_011046840, Unigene29611\_Sample\_011046840, Unigene41927\_Sample\_011046840, Unigene29621\_Sample\_011046840, Unigene27942\_Sample\_011046840, Unigene41127\_Sample\_011046840, Unigene13984\_Sample\_011046840, Unigene34362\_Sample\_011046840, Unigene32819\_Sample\_011046840, Unigene30713\_Sample\_011046840, Unigene5552\_Sample\_011046840, Unigene15637\_Sample\_011046840, Unigene34780\_Sample\_011046840, Unigene7506\_Sample\_011046840, Unigene36827\_Sample\_011046840, Unigene19404\_Sample\_011046840, Unigene24938\_Sample\_011046840, Unigene31120\_Sample\_011046840, Unigene43143\_Sample\_011046840, Unigene9887\_Sample\_011046840, Unigene36413\_Sample\_011046840, Unigene11592\_Sample\_011046840, Unigene35789\_Sample\_011046840, Unigene15030\_Sample\_011046840, Unigene40822\_Sample\_011046840, Unigene42964\_Sample\_011046840, Unigene24453\_Sample\_011046840, Unigene42663\_Sample\_011046840, Unigene5963\_Sample\_011046840, Unigene13602\_Sample\_011046840, Unigene43397\_Sample\_011046840, Unigene19793\_Sample\_011046840, Unigene32158\_Sample\_011046840, Unigene42596\_Sample\_011046840, Unigene38864\_Sample\_011046840, Unigene20577\_Sample\_011046840, Unigene14302\_Sample\_011046840, Unigene31135\_Sample\_011046840, Unigene25783\_Sample\_011046840, Unigene25531\_Sample\_011046840, Unigene7067\_Sample\_011046840, Unigene43623\_Sample\_011046840, Unigene7349\_Sample\_011046840, Unigene3226\_Sample\_011046840, Unigene17721\_Sample\_011046840, Unigene31168\_Sample\_011046840, Unigene34071\_Sample\_011046840, Unigene41691\_Sample\_011046840, Unigene26581\_Sample\_011046840, Unigene28708\_Sample\_011046840, Unigene42865\_Sample\_011046840, Unigene6064\_Sample\_011046840, Unigene16389\_Sample\_011046840, Unigene3257\_Sample\_011046840, Unigene29534\_Sample\_011046840, Unigene22159\_Sample\_011046840, Unigene29452\_Sample\_011046840, Unigene32280\_Sample\_011046840, Unigene10176\_Sample\_011046840, Unigene33275\_Sample\_011046840, Unigene31074\_Sample\_011046840, Unigene15062\_Sample\_011046840, Unigene17700\_Sample\_011046840, Unigene36699\_Sample\_011046840, Unigene28415\_Sample\_011046840, Unigene33858\_Sample\_011046840, Unigene38658\_Sample\_011046840, Unigene23820\_Sample\_011046840, Unigene7402\_Sample\_011046840, Unigene5375\_Sample\_011046840, Unigene10829\_Sample\_011046840, Unigene11629\_Sample\_011046840, Unigene43387\_Sample\_011046840, Unigene35909\_Sample\_011046840, Unigene26502\_Sample\_011046840, Unigene36268\_Sample\_011046840, Unigene29818\_Sample\_011046840, Unigene31458\_Sample\_011046840, Unigene30136\_Sample\_011046840, Unigene24201\_Sample\_011046840, Unigene8372\_Sample\_011046840, Unigene40709\_Sample\_011046840, Unigene35699\_Sample\_011046840, Unigene27865\_Sample\_011046840, Unigene40966\_Sample\_011046840, Unigene489\_Sample\_011046840, Unigene10957\_Sample\_011046840, Unigene4518\_Sample\_011046840, Unigene33342\_Sample\_011046840, Unigene26057\_Sample\_011046840, Unigene34506\_Sample\_011046840, Unigene20052\_Sample\_011046840, Unigene42097\_Sample\_011046840, Unigene24244\_Sample\_011046840, Unigene41398\_Sample\_011046840, Unigene7025\_Sample\_011046840, Unigene22635\_Sample\_011046840, Unigene27993\_Sample\_011046840, Unigene39020\_Sample\_011046840, Unigene40262\_Sample\_011046840, Unigene9922\_Sample\_011046840, Unigene24917\_Sample\_011046840, Unigene32947\_Sample\_011046840, Unigene32360\_Sample\_011046840, Unigene29103\_Sample\_011046840, Unigene39324\_Sample\_011046840, Unigene32594\_Sample\_011046840, Unigene22752\_Sample\_011046840, Unigene40551\_Sample\_011046840, Unigene41178\_Sample\_011046840, Unigene43627\_Sample\_011046840, Unigene33112\_Sample\_011046840, Unigene37999\_Sample\_011046840, Unigene6285\_Sample\_011046840, Unigene28867\_Sample\_011046840, Unigene33052\_Sample\_011046840, Unigene33999\_Sample\_011046840, Unigene31448\_Sample\_011046840, Unigene16829\_Sample\_011046840, Unigene29049\_Sample\_011046840, Unigene32831\_Sample\_011046840, Unigene26512\_Sample\_011046840, Unigene40783\_Sample\_011046840, Unigene42138\_Sample\_011046840, Unigene29397\_Sample\_011046840, Unigene37985\_Sample\_011046840, Unigene14485\_Sample\_011046840, Unigene36500\_Sample\_011046840, Unigene19235\_Sample\_011046840, Unigene20304\_Sample\_011046840, Unigene42585\_Sample\_011046840, Unigene29567\_Sample\_011046840, Unigene34472\_Sample\_011046840, Unigene15594\_Sample\_011046840, Unigene33393\_Sample\_011046840, Unigene31108\_Sample\_011046840, Unigene16321\_Sample\_011046840, Unigene8615\_Sample\_011046840, Unigene33196\_Sample\_011046840, Unigene19400\_Sample\_011046840, Unigene4619\_Sample\_011046840, Unigene24373\_Sample\_011046840, Unigene19647\_Sample\_011046840, Unigene34843\_Sample\_011046840, Unigene10033\_Sample\_011046840, Unigene40222\_Sample\_011046840, Unigene38165\_Sample\_011046840, Unigene33473\_Sample\_011046840, Unigene42190\_Sample\_011046840, Unigene41068\_Sample\_011046840, Unigene30539\_Sample\_011046840, Unigene39171\_Sample\_011046840, Unigene37353\_Sample\_011046840, Unigene42460\_Sample\_011046840, Unigene26767\_Sample\_011046840, Unigene16855\_Sample\_011046840, Unigene38564\_Sample\_011046840, Unigene25769\_Sample\_011046840, Unigene19809\_Sample\_011046840, Unigene39825\_Sample\_011046840, Unigene40368\_Sample\_011046840, Unigene6561\_Sample\_011046840, Unigene22844\_Sample\_011046840, Unigene10568\_Sample\_011046840, Unigene43614\_Sample\_011046840, Unigene41718\_Sample\_011046840, Unigene38534\_Sample\_011046840, Unigene36032\_Sample\_011046840, Unigene535\_Sample\_011046840, Unigene37644\_Sample\_011046840, Unigene32515\_Sample\_011046840, Unigene39951\_Sample\_011046840, Unigene38730\_Sample\_011046840, Unigene43450\_Sample\_011046840, Unigene40192\_Sample\_011046840, Unigene39935\_Sample\_011046840, Unigene473\_Sample\_011046840, Unigene39427\_Sample\_011046840, Unigene20351\_Sample\_011046840, Unigene25001\_Sample\_011046840, Unigene20367\_Sample\_011046840, Unigene31408\_Sample\_011046840, Unigene8211\_Sample\_011046840, Unigene4307\_Sample\_011046840, Unigene39007\_Sample\_011046840, Unigene39015\_Sample\_011046840, Unigene37487\_Sample\_011046840, Unigene38616\_Sample\_011046840, Unigene43281\_Sample\_011046840, Unigene815\_Sample\_011046840, Unigene13836\_Sample\_011046840, Unigene26820\_Sample\_011046840, Unigene42164\_Sample\_011046840, Unigene6530\_Sample\_011046840, Unigene412\_Sample\_011046840, Unigene38370\_Sample\_011046840, Unigene20078\_Sample\_011046840, Unigene38792\_Sample\_011046840, Unigene30813\_Sample\_011046840, Unigene43078\_Sample\_011046840, Unigene22254\_Sample\_011046840, Unigene34112\_Sample\_011046840, Unigene40971\_Sample\_011046840, Unigene43019\_Sample\_011046840, Unigene27313\_Sample\_011046840, Unigene17135\_Sample\_011046840, Unigene43311\_Sample\_011046840, Unigene43216\_Sample\_011046840, Unigene24243\_Sample\_011046840, Unigene34992\_Sample\_011046840, Unigene6409\_Sample\_011046840, Unigene22630\_Sample\_011046840, Unigene42997\_Sample\_011046840, Unigene42121\_Sample\_011046840, Unigene27337\_Sample\_011046840, Unigene9880\_Sample\_011046840, Unigene35787\_Sample\_011046840, Unigene34351\_Sample\_011046840, Unigene11464\_Sample\_011046840, Unigene39486\_Sample\_011046840, Unigene35792\_Sample\_011046840, Unigene6378\_Sample\_011046840, Unigene7938\_Sample\_011046840, Unigene33580\_Sample\_011046840, Unigene6293\_Sample\_011046840, Unigene28473\_Sample\_011046840, Unigene27358\_Sample\_011046840, Unigene37793\_Sample\_011046840, Unigene15491\_Sample\_011046840, Unigene32568\_Sample\_011046840, Unigene41588\_Sample\_011046840, Unigene28437\_Sample\_011046840, Unigene43176\_Sample\_011046840, Unigene36475\_Sample\_011046840, Unigene14116\_Sample\_011046840, Unigene37359\_Sample\_011046840, Unigene38852\_Sample\_011046840, Unigene13391\_Sample\_011046840, Unigene39348\_Sample\_011046840, Unigene11853\_Sample\_011046840, Unigene39080\_Sample\_011046840, Unigene38047\_Sample\_011046840, Unigene40881\_Sample\_011046840, Unigene12767\_Sample\_011046840, Unigene35476\_Sample\_011046840, Unigene29006\_Sample\_011046840, Unigene39136\_Sample\_011046840, Unigene15696\_Sample\_011046840, Unigene30322\_Sample\_011046840, Unigene5815\_Sample\_011046840, Unigene29131\_Sample\_011046840, Unigene39668\_Sample\_011046840, Unigene40903\_Sample\_011046840, Unigene42227\_Sample\_011046840, Unigene40992\_Sample\_011046840, Unigene36427\_Sample\_011046840, Unigene16130\_Sample\_011046840, Unigene34102\_Sample\_011046840, Unigene36876\_Sample\_011046840, Unigene24004\_Sample\_011046840, Unigene39404\_Sample\_011046840, Unigene26969\_Sample\_011046840, Unigene34453\_Sample\_011046840, Unigene12655\_Sample\_011046840, Unigene37877\_Sample\_011046840, Unigene27975\_Sample\_011046840, Unigene14511\_Sample\_011046840, Unigene32134\_Sample\_011046840, Unigene32749\_Sample\_011046840, Unigene26815\_Sample\_011046840, Unigene26734\_Sample\_011046840, Unigene43062\_Sample\_011046840, Unigene29301\_Sample\_011046840, Unigene42383\_Sample\_011046840, Unigene28404\_Sample\_011046840, Unigene16269\_Sample\_011046840, Unigene40565\_Sample\_011046840, Unigene39381\_Sample\_011046840, Unigene2174\_Sample\_011046840, Unigene32865\_Sample\_011046840, Unigene27180\_Sample\_011046840, Unigene688\_Sample\_011046840, Unigene36400\_Sample\_011046840, Unigene43084\_Sample\_011046840, Unigene3156\_Sample\_011046840, Unigene35434\_Sample\_011046840, Unigene6180\_Sample\_011046840, Unigene33938\_Sample\_011046840, Unigene40865\_Sample\_011046840, Unigene39866\_Sample\_011046840, Unigene31310\_Sample\_011046840, Unigene23252\_Sample\_011046840, Unigene42296\_Sample\_011046840, Unigene22122\_Sample\_011046840, Unigene37628\_Sample\_011046840, Unigene11949\_Sample\_011046840, Unigene34464\_Sample\_011046840, Unigene36926\_Sample\_011046840, Unigene42789\_Sample\_011046840, Unigene13828\_Sample\_011046840, Unigene1963\_Sample\_011046840, Unigene39156\_Sample\_011046840, Unigene11179\_Sample\_011046840, Unigene40994\_Sample\_011046840, Unigene1256\_Sample\_011046840, Unigene6241\_Sample\_011046840, Unigene37705\_Sample\_011046840, Unigene16976\_Sample\_011046840, Unigene25184\_Sample\_011046840, Unigene31945\_Sample\_011046840, Unigene23357\_Sample\_011046840, Unigene23281\_Sample\_011046840, Unigene37392\_Sample\_011046840, Unigene39992\_Sample\_011046840, Unigene8218\_Sample\_011046840, Unigene10696\_Sample\_011046840, Unigene36706\_Sample\_011046840, Unigene43477\_Sample\_011046840, Unigene30048\_Sample\_011046840, Unigene41792\_Sample\_011046840, Unigene4723\_Sample\_011046840, Unigene30691\_Sample\_011046840, Unigene40837\_Sample\_011046840, Unigene14017\_Sample\_011046840, Unigene36112\_Sample\_011046840, Unigene20250\_Sample\_011046840, Unigene7503\_Sample\_011046840, Unigene30919\_Sample\_011046840, Unigene14611\_Sample\_011046840, Unigene41062\_Sample\_011046840, Unigene17216\_Sample\_011046840, Unigene40424\_Sample\_011046840, Unigene3599\_Sample\_011046840, Unigene23324\_Sample\_011046840, Unigene29427\_Sample\_011046840, Unigene17375\_Sample\_011046840, Unigene36567\_Sample\_011046840, Unigene15303\_Sample\_011046840, Unigene16035\_Sample\_011046840, Unigene27997\_Sample\_011046840, Unigene42864\_Sample\_011046840, Unigene36920\_Sample\_011046840, Unigene13276\_Sample\_011046840, Unigene39180\_Sample\_011046840, Unigene43536\_Sample\_011046840, Unigene34655\_Sample\_011046840, Unigene40818\_Sample\_011046840, Unigene38377\_Sample\_011046840, Unigene18440\_Sample\_011046840, Unigene28736\_Sample\_011046840, Unigene37830\_Sample\_011046840, Unigene33866\_Sample\_011046840, Unigene43607\_Sample\_011046840, Unigene21072\_Sample\_011046840, Unigene12713\_Sample\_011046840, Unigene22459\_Sample\_011046840, Unigene33926\_Sample\_011046840, Unigene37219\_Sample\_011046840, Unigene38559\_Sample\_011046840, Unigene11613\_Sample\_011046840, Unigene38360\_Sample\_011046840, Unigene38044\_Sample\_011046840, Unigene42007\_Sample\_011046840, Unigene30105\_Sample\_011046840, Unigene41174\_Sample\_011046840, Unigene15831\_Sample\_011046840, Unigene41740\_Sample\_011046840, Unigene432\_Sample\_011046840, Unigene17416\_Sample\_011046840, Unigene40346\_Sample\_011046840, Unigene35883\_Sample\_011046840, Unigene31610\_Sample\_011046840, Unigene37041\_Sample\_011046840, Unigene30572\_Sample\_011046840, Unigene23630\_Sample\_011046840, Unigene33760\_Sample\_011046840, Unigene38146\_Sample\_011046840, Unigene24246\_Sample\_011046840, Unigene31584\_Sample\_011046840, Unigene36376\_Sample\_011046840, Unigene30798\_Sample\_011046840, Unigene39838\_Sample\_011046840, Unigene3842\_Sample\_011046840, Unigene12743\_Sample\_011046840, Unigene31773\_Sample\_011046840, Unigene3928\_Sample\_011046840, Unigene41001\_Sample\_011046840, Unigene23856\_Sample\_011046840, Unigene43115\_Sample\_011046840, Unigene22258\_Sample\_011046840, Unigene17210\_Sample\_011046840, Unigene41498\_Sample\_011046840, Unigene31398\_Sample\_011046840, Unigene10770\_Sample\_011046840, Unigene42171\_Sample\_011046840, Unigene24889\_Sample\_011046840, Unigene9871\_Sample\_011046840, Unigene38932\_Sample\_011046840, Unigene33443\_Sample\_011046840, Unigene38152\_Sample\_011046840, Unigene14629\_Sample\_011046840, Unigene14756\_Sample\_011046840, Unigene42050\_Sample\_011046840, Unigene42835\_Sample\_011046840, Unigene5197\_Sample\_011046840, Unigene29880\_Sample\_011046840, Unigene7717\_Sample\_011046840, Unigene5121\_Sample\_011046840, Unigene43147\_Sample\_011046840, Unigene38039\_Sample\_011046840, Unigene28861\_Sample\_011046840, Unigene42602\_Sample\_011046840, Unigene10135\_Sample\_011046840, Unigene22688\_Sample\_011046840, Unigene10802\_Sample\_011046840, Unigene40391\_Sample\_011046840, Unigene38670\_Sample\_011046840, Unigene29016\_Sample\_011046840, Unigene37540\_Sample\_011046840, Unigene21026\_Sample\_011046840, Unigene36690\_Sample\_011046840, Unigene39732\_Sample\_011046840, Unigene1959\_Sample\_011046840, Unigene18735\_Sample\_011046840, Unigene32368\_Sample\_011046840, Unigene19481\_Sample\_011046840, Unigene43025\_Sample\_011046840, Unigene41288\_Sample\_011046840, Unigene30094\_Sample\_011046840, Unigene25579\_Sample\_011046840, Unigene34053\_Sample\_011046840, Unigene28414\_Sample\_011046840, Unigene12587\_Sample\_011046840, Unigene39882\_Sample\_011046840, Unigene35872\_Sample\_011046840, Unigene10288\_Sample\_011046840, Unigene9718\_Sample\_011046840, Unigene39555\_Sample\_011046840, Unigene35927\_Sample\_011046840, Unigene5480\_Sample\_011046840, Unigene14666\_Sample\_011046840, Unigene36535\_Sample\_011046840, Unigene12679\_Sample\_011046840, Unigene40687\_Sample\_011046840, Unigene39837\_Sample\_011046840, Unigene23176\_Sample\_011046840, Unigene24594\_Sample\_011046840, Unigene41858\_Sample\_011046840, Unigene42683\_Sample\_011046840, Unigene14292\_Sample\_011046840, Unigene27724\_Sample\_011046840, Unigene19383\_Sample\_011046840, Unigene10601\_Sample\_011046840, Unigene33259\_Sample\_011046840, Unigene30862\_Sample\_011046840, Unigene41336\_Sample\_011046840, Unigene25871\_Sample\_011046840, Unigene33322\_Sample\_011046840, Unigene30573\_Sample\_011046840, Unigene38115\_Sample\_011046840, Unigene34746\_Sample\_011046840, Unigene36462\_Sample\_011046840, Unigene40812\_Sample\_011046840, Unigene40787\_Sample\_011046840, Unigene37745\_Sample\_011046840, Unigene24674\_Sample\_011046840, Unigene40184\_Sample\_011046840, Unigene28636\_Sample\_011046840, Unigene33889\_Sample\_011046840, Unigene28901\_Sample\_011046840, Unigene43453\_Sample\_011046840, Unigene39490\_Sample\_011046840, Unigene39632\_Sample\_011046840, Unigene11701\_Sample\_011046840, Unigene31638\_Sample\_011046840, Unigene11125\_Sample\_011046840, Unigene42030\_Sample\_011046840, Unigene28758\_Sample\_011046840, Unigene25431\_Sample\_011046840, Unigene4320\_Sample\_011046840, Unigene20420\_Sample\_011046840, Unigene28316\_Sample\_011046840, Unigene6563\_Sample\_011046840, Unigene36951\_Sample\_011046840, Unigene43031\_Sample\_011046840, Unigene12197\_Sample\_011046840, Unigene36090\_Sample\_011046840, Unigene22961\_Sample\_011046840, Unigene43363\_Sample\_011046840, Unigene42068\_Sample\_011046840, Unigene22232\_Sample\_011046840, Unigene38021\_Sample\_011046840, Unigene18262\_Sample\_011046840, Unigene41561\_Sample\_011046840, Unigene26967\_Sample\_011046840, Unigene12868\_Sample\_011046840, Unigene37710\_Sample\_011046840, Unigene41901\_Sample\_011046840, Unigene4043\_Sample\_011046840, Unigene25934\_Sample\_011046840, Unigene42079\_Sample\_011046840, Unigene165\_Sample\_011046840, Unigene43611\_Sample\_011046840, Unigene7072\_Sample\_011046840, Unigene3161\_Sample\_011046840, Unigene40387\_Sample\_011046840, Unigene21295\_Sample\_011046840, Unigene42944\_Sample\_011046840, Unigene5047\_Sample\_011046840, Unigene1859\_Sample\_011046840, Unigene4721\_Sample\_011046840, Unigene35151\_Sample\_011046840, Unigene35324\_Sample\_011046840, Unigene29879\_Sample\_011046840, Unigene25629\_Sample\_011046840, Unigene29966\_Sample\_011046840, Unigene41467\_Sample\_011046840, Unigene16882\_Sample\_011046840, Unigene42154\_Sample\_011046840, Unigene38267\_Sample\_011046840, Unigene42047\_Sample\_011046840, Unigene25720\_Sample\_011046840, Unigene36496\_Sample\_011046840, Unigene14078\_Sample\_011046840, Unigene13016\_Sample\_011046840, Unigene39103\_Sample\_011046840, Unigene24184\_Sample\_011046840, Unigene3537\_Sample\_011046840, Unigene38522\_Sample\_011046840, Unigene6787\_Sample\_011046840, Unigene5393\_Sample\_011046840, Unigene37573\_Sample\_011046840, Unigene25466\_Sample\_011046840, Unigene31793\_Sample\_011046840, Unigene12386\_Sample\_011046840, Unigene29845\_Sample\_011046840, Unigene35118\_Sample\_011046840, Unigene42837\_Sample\_011046840, Unigene42475\_Sample\_011046840, Unigene42531\_Sample\_011046840, Unigene42204\_Sample\_011046840, Unigene2615\_Sample\_011046840, Unigene35939\_Sample\_011046840, Unigene34233\_Sample\_011046840, Unigene39958\_Sample\_011046840, Unigene23541\_Sample\_011046840, Unigene18141\_Sample\_011046840, Unigene42252\_Sample\_011046840, Unigene12084\_Sample\_011046840, Unigene40601\_Sample\_011046840, Unigene32188\_Sample\_011046840, Unigene39418\_Sample\_011046840, Unigene42200\_Sample\_011046840, Unigene21974\_Sample\_011046840, Unigene40208\_Sample\_011046840, Unigene37077\_Sample\_011046840, Unigene20828\_Sample\_011046840, Unigene30505\_Sample\_011046840, Unigene14237\_Sample\_011046840, Unigene42786\_Sample\_011046840, Unigene3395\_Sample\_011046840, Unigene35395\_Sample\_011046840, Unigene3814\_Sample\_011046840, Unigene42886\_Sample\_011046840, Unigene38215\_Sample\_011046840, Unigene33515\_Sample\_011046840, Unigene38121\_Sample\_011046840, Unigene41880\_Sample\_011046840, Unigene19448\_Sample\_011046840, Unigene3164\_Sample\_011046840, Unigene42133\_Sample\_011046840, Unigene30530\_Sample\_011046840, Unigene20634\_Sample\_011046840, Unigene7794\_Sample\_011046840, Unigene31\_Sample\_011046840, Unigene23873\_Sample\_011046840, Unigene33979\_Sample\_011046840, Unigene27545\_Sample\_011046840, Unigene38091\_Sample\_011046840, Unigene31822\_Sample\_011046840, Unigene41582\_Sample\_011046840, Unigene38462\_Sample\_011046840, Unigene34355\_Sample\_011046840, Unigene39505\_Sample\_011046840, Unigene8064\_Sample\_011046840, Unigene7983\_Sample\_011046840, Unigene37702\_Sample\_011046840, Unigene29411\_Sample\_011046840, Unigene43525\_Sample\_011046840, Unigene17079\_Sample\_011046840, Unigene5594\_Sample\_011046840, Unigene35068\_Sample\_011046840, Unigene30197\_Sample\_011046840, Unigene18134\_Sample\_011046840, Unigene35433\_Sample\_011046840, Unigene35554\_Sample\_011046840, Unigene25328\_Sample\_011046840, Unigene39870\_Sample\_011046840, Unigene9730\_Sample\_011046840, Unigene22550\_Sample\_011046840, Unigene24428\_Sample\_011046840, Unigene40465\_Sample\_011046840, Unigene25313\_Sample\_011046840, Unigene28259\_Sample\_011046840, Unigene37149\_Sample\_011046840, Unigene34500\_Sample\_011046840, Unigene29711\_Sample\_011046840, Unigene40223\_Sample\_011046840, Unigene6498\_Sample\_011046840, Unigene32116\_Sample\_011046840, Unigene20023\_Sample\_011046840, Unigene31318\_Sample\_011046840, Unigene39179\_Sample\_011046840, Unigene35234\_Sample\_011046840, Unigene3934\_Sample\_011046840, Unigene43422\_Sample\_011046840, Unigene14154\_Sample\_011046840, Unigene2408\_Sample\_011046840, Unigene42127\_Sample\_011046840, Unigene24187\_Sample\_011046840, Unigene23188\_Sample\_011046840, Unigene1039\_Sample\_011046840, Unigene31248\_Sample\_011046840, Unigene35633\_Sample\_011046840, Unigene29346\_Sample\_011046840, Unigene12033\_Sample\_011046840, Unigene17239\_Sample\_011046840, Unigene40016\_Sample\_011046840, Unigene35643\_Sample\_011046840, Unigene42177\_Sample\_011046840, Unigene33743\_Sample\_011046840, Unigene43636\_Sample\_011046840, Unigene15849\_Sample\_011046840, Unigene23811\_Sample\_011046840, Unigene41355\_Sample\_011046840, Unigene7643\_Sample\_011046840, Unigene41574\_Sample\_011046840, Unigene31649\_Sample\_011046840, Unigene33736\_Sample\_011046840, Unigene34555\_Sample\_011046840, Unigene10641\_Sample\_011046840, Unigene32875\_Sample\_011046840, Unigene11685\_Sample\_011046840, Unigene27895\_Sample\_011046840, Unigene4327\_Sample\_011046840, Unigene43095\_Sample\_011046840, Unigene12062\_Sample\_011046840, Unigene16063\_Sample\_011046840, Unigene30508\_Sample\_011046840, Unigene36187\_Sample\_011046840, Unigene5400\_Sample\_011046840, Unigene3339\_Sample\_011046840, Unigene36260\_Sample\_011046840, Unigene32706\_Sample\_011046840, Unigene37852\_Sample\_011046840, Unigene31684\_Sample\_011046840, Unigene28063\_Sample\_011046840, Unigene16650\_Sample\_011046840, Unigene39275\_Sample\_011046840, Unigene12701\_Sample\_011046840, Unigene14279\_Sample\_011046840, Unigene32354\_Sample\_011046840, Unigene30059\_Sample\_011046840, Unigene2326\_Sample\_011046840, Unigene10035\_Sample\_011046840, Unigene8904\_Sample\_011046840, Unigene36088\_Sample\_011046840, Unigene41771\_Sample\_011046840, Unigene41481\_Sample\_011046840, Unigene42569\_Sample\_011046840, Unigene18212\_Sample\_011046840, Unigene9721\_Sample\_011046840, Unigene8113\_Sample\_011046840, Unigene42574\_Sample\_011046840, Unigene25488\_Sample\_011046840, Unigene41935\_Sample\_011046840, Unigene4815\_Sample\_011046840, Unigene34896\_Sample\_011046840, Unigene14473\_Sample\_011046840, Unigene26865\_Sample\_011046840, Unigene23169\_Sample\_011046840, Unigene39391\_Sample\_011046840, Unigene2581\_Sample\_011046840, Unigene43556\_Sample\_011046840, Unigene41980\_Sample\_011046840, Unigene42325\_Sample\_011046840, Unigene42348\_Sample\_011046840, Unigene35074\_Sample\_011046840, Unigene39497\_Sample\_011046840, Unigene2218\_Sample\_011046840, Unigene39081\_Sample\_011046840, Unigene14503\_Sample\_011046840, Unigene41972\_Sample\_011046840, Unigene7458\_Sample\_011046840, Unigene21905\_Sample\_011046840, Unigene42768\_Sample\_011046840, Unigene12341\_Sample\_011046840, Unigene41855\_Sample\_011046840, Unigene41007\_Sample\_011046840, Unigene10729\_Sample\_011046840, Unigene19018\_Sample\_011046840, Unigene10318\_Sample\_011046840, Unigene3788\_Sample\_011046840, Unigene13393\_Sample\_011046840, Unigene41324\_Sample\_011046840, Unigene40612\_Sample\_011046840, Unigene6862\_Sample\_011046840, Unigene32621\_Sample\_011046840, Unigene23759\_Sample\_011046840, Unigene36342\_Sample\_011046840, Unigene33797\_Sample\_011046840, Unigene2687\_Sample\_011046840, Unigene21944\_Sample\_011046840, Unigene2151\_Sample\_011046840, Unigene42626\_Sample\_011046840, Unigene14750\_Sample\_011046840, Unigene31441\_Sample\_011046840, Unigene35459\_Sample\_011046840, Unigene43348\_Sample\_011046840, Unigene43412\_Sample\_011046840, Unigene2152\_Sample\_011046840, Unigene39717\_Sample\_011046840, Unigene4686\_Sample\_011046840, Unigene36544\_Sample\_011046840, Unigene17677\_Sample\_011046840, Unigene41261\_Sample\_011046840, Unigene10474\_Sample\_011046840, Unigene17194\_Sample\_011046840, Unigene15889\_Sample\_011046840, Unigene27951\_Sample\_011046840, Unigene31900\_Sample\_011046840, Unigene37045\_Sample\_011046840, Unigene4497\_Sample\_011046840, Unigene12353\_Sample\_011046840, Unigene2092\_Sample\_011046840, Unigene12675\_Sample\_011046840, Unigene34828\_Sample\_011046840, Unigene24005\_Sample\_011046840, Unigene37741\_Sample\_011046840, Unigene19653\_Sample\_011046840, Unigene39559\_Sample\_011046840, Unigene40562\_Sample\_011046840, Unigene40053\_Sample\_011046840, Unigene32836\_Sample\_011046840, Unigene37404\_Sample\_011046840, Unigene32314\_Sample\_011046840, Unigene20154\_Sample\_011046840, Unigene39437\_Sample\_011046840, Unigene39720\_Sample\_011046840, Unigene5378\_Sample\_011046840, Unigene208\_Sample\_011046840, Unigene43258\_Sample\_011046840, Unigene19918\_Sample\_011046840, Unigene5436\_Sample\_011046840, Unigene20526\_Sample\_011046840, Unigene26338\_Sample\_011046840, Unigene40753\_Sample\_011046840, Unigene15987\_Sample\_011046840, Unigene40278\_Sample\_011046840, Unigene3127\_Sample\_011046840, Unigene26705\_Sample\_011046840, Unigene19593\_Sample\_011046840, Unigene6740\_Sample\_011046840, Unigene37517\_Sample\_011046840, Unigene30236\_Sample\_011046840, Unigene28852\_Sample\_011046840, Unigene33998\_Sample\_011046840, Unigene31994\_Sample\_011046840, Unigene26163\_Sample\_011046840, Unigene27886\_Sample\_011046840, Unigene18907\_Sample\_011046840, Unigene26846\_Sample\_011046840, Unigene5241\_Sample\_011046840, Unigene34039\_Sample\_011046840, Unigene38608\_Sample\_011046840, Unigene2543\_Sample\_011046840, Unigene35951\_Sample\_011046840, Unigene6760\_Sample\_011046840, Unigene21415\_Sample\_011046840, Unigene41637\_Sample\_011046840, Unigene20065\_Sample\_011046840, Unigene35555\_Sample\_011046840, Unigene28002\_Sample\_011046840, Unigene30702\_Sample\_011046840, Unigene20428\_Sample\_011046840, Unigene14691\_Sample\_011046840, Unigene42798\_Sample\_011046840, Unigene23889\_Sample\_011046840, Unigene40899\_Sample\_011046840, Unigene42418\_Sample\_011046840, Unigene31192\_Sample\_011046840, Unigene3075\_Sample\_011046840, Unigene21332\_Sample\_011046840, Unigene14501\_Sample\_011046840, Unigene42183\_Sample\_011046840, Unigene34524\_Sample\_011046840, Unigene30629\_Sample\_011046840, Unigene16244\_Sample\_011046840, Unigene42293\_Sample\_011046840, Unigene31880\_Sample\_011046840, Unigene32589\_Sample\_011046840, Unigene23234\_Sample\_011046840, Unigene2740\_Sample\_011046840, Unigene5453\_Sample\_011046840, Unigene21959\_Sample\_011046840, Unigene22620\_Sample\_011046840, Unigene41709\_Sample\_011046840, Unigene43416\_Sample\_011046840, Unigene34975\_Sample\_011046840, Unigene8660\_Sample\_011046840, Unigene37131\_Sample\_011046840, Unigene26023\_Sample\_011046840, Unigene16906\_Sample\_011046840, Unigene29661\_Sample\_011046840, Unigene40616\_Sample\_011046840, Unigene43186\_Sample\_011046840, Unigene29145\_Sample\_011046840, Unigene7789\_Sample\_011046840, Unigene26507\_Sample\_011046840, Unigene31407\_Sample\_011046840, Unigene42281\_Sample\_011046840, Unigene8121\_Sample\_011046840, Unigene34432\_Sample\_011046840, Unigene41419\_Sample\_011046840, Unigene22119\_Sample\_011046840, Unigene43273\_Sample\_011046840, Unigene37116\_Sample\_011046840, Unigene32645\_Sample\_011046840, Unigene7513\_Sample\_011046840, Unigene26212\_Sample\_011046840, Unigene2519\_Sample\_011046840, Unigene22727\_Sample\_011046840, Unigene41160\_Sample\_011046840, Unigene42088\_Sample\_011046840, Unigene34380\_Sample\_011046840, Unigene19472\_Sample\_011046840, Unigene34400\_Sample\_011046840, Unigene39994\_Sample\_011046840, Unigene17139\_Sample\_011046840, Unigene40445\_Sample\_011046840, Unigene19704\_Sample\_011046840, Unigene42738\_Sample\_011046840, Unigene43001\_Sample\_011046840, Unigene18270\_Sample\_011046840, Unigene41119\_Sample\_011046840, Unigene356\_Sample\_011046840, Unigene2068\_Sample\_011046840, Unigene34304\_Sample\_011046840, Unigene36231\_Sample\_011046840, Unigene39253\_Sample\_011046840, Unigene4276\_Sample\_011046840, Unigene11443\_Sample\_011046840, Unigene10448\_Sample\_011046840, Unigene7089\_Sample\_011046840, Unigene14521\_Sample\_011046840, Unigene40309\_Sample\_011046840, Unigene30053\_Sample\_011046840, Unigene18953\_Sample\_011046840, Unigene41695\_Sample\_011046840, Unigene39582\_Sample\_011046840, Unigene16017\_Sample\_011046840, Unigene28188\_Sample\_011046840, Unigene42249\_Sample\_011046840, Unigene43217\_Sample\_011046840, Unigene22397\_Sample\_011046840, Unigene2237\_Sample\_011046840, Unigene42271\_Sample\_011046840, Unigene11994\_Sample\_011046840, Unigene31695\_Sample\_011046840, Unigene42116\_Sample\_011046840, Unigene11132\_Sample\_011046840, Unigene12950\_Sample\_011046840, Unigene8228\_Sample\_011046840, Unigene7856\_Sample\_011046840, Unigene42354\_Sample\_011046840, Unigene39818\_Sample\_011046840, Unigene36810\_Sample\_011046840, Unigene39399\_Sample\_011046840, Unigene43542\_Sample\_011046840, Unigene5443\_Sample\_011046840, Unigene38542\_Sample\_011046840, Unigene41337\_Sample\_011046840, Unigene27657\_Sample\_011046840, Unigene40404\_Sample\_011046840, Unigene29336\_Sample\_011046840, Unigene34874\_Sample\_011046840, Unigene35820\_Sample\_011046840, Unigene507\_Sample\_011046840, Unigene39628\_Sample\_011046840, Unigene8594\_Sample\_011046840, Unigene24129\_Sample\_011046840, Unigene6886\_Sample\_011046840, Unigene31662\_Sample\_011046840, Unigene20272\_Sample\_011046840, Unigene39204\_Sample\_011046840, Unigene28265\_Sample\_011046840, Unigene12153\_Sample\_011046840, Unigene35008\_Sample\_011046840, Unigene36013\_Sample\_011046840, Unigene9006\_Sample\_011046840, Unigene38540\_Sample\_011046840, Unigene25307\_Sample\_011046840, Unigene36128\_Sample\_011046840, Unigene38354\_Sample\_011046840, Unigene43631\_Sample\_011046840, Unigene31373\_Sample\_011046840, Unigene39649\_Sample\_011046840, Unigene7935\_Sample\_011046840, Unigene2986\_Sample\_011046840, Unigene31576\_Sample\_011046840, Unigene43486\_Sample\_011046840, Unigene27792\_Sample\_011046840, Unigene42666\_Sample\_011046840, Unigene11875\_Sample\_011046840, Unigene9715\_Sample\_011046840, Unigene7825\_Sample\_011046840, Unigene40602\_Sample\_011046840, Unigene27152\_Sample\_011046840, Unigene23601\_Sample\_011046840, Unigene15570\_Sample\_011046840, Unigene33901\_Sample\_011046840, Unigene15138\_Sample\_011046840, Unigene36025\_Sample\_011046840, Unigene19183\_Sample\_011046840, Unigene31646\_Sample\_011046840, Unigene35279\_Sample\_011046840, Unigene35252\_Sample\_011046840, Unigene29670\_Sample\_011046840, Unigene34909\_Sample\_011046840, Unigene4529\_Sample\_011046840, Unigene37829\_Sample\_011046840, Unigene41664\_Sample\_011046840, Unigene31732\_Sample\_011046840, Unigene39606\_Sample\_011046840, Unigene3153\_Sample\_011046840, Unigene42298\_Sample\_011046840, Unigene39674\_Sample\_011046840, Unigene17985\_Sample\_011046840, Unigene39633\_Sample\_011046840, Unigene42376\_Sample\_011046840, Unigene39748\_Sample\_011046840, Unigene16618\_Sample\_011046840, Unigene26435\_Sample\_011046840, Unigene41478\_Sample\_011046840, Unigene3258\_Sample\_011046840, Unigene5425\_Sample\_011046840, Unigene6039\_Sample\_011046840, Unigene40703\_Sample\_011046840, Unigene19931\_Sample\_011046840, Unigene7299\_Sample\_011046840, Unigene39226\_Sample\_011046840, Unigene43445\_Sample\_011046840, Unigene2524\_Sample\_011046840, Unigene36817\_Sample\_011046840, Unigene33539\_Sample\_011046840, Unigene12013\_Sample\_011046840, Unigene41090\_Sample\_011046840, Unigene42509\_Sample\_011046840, Unigene3219\_Sample\_011046840, Unigene42756\_Sample\_011046840, Unigene40031\_Sample\_011046840, Unigene15184\_Sample\_011046840, Unigene5791\_Sample\_011046840, Unigene40452\_Sample\_011046840, Unigene41720\_Sample\_011046840, Unigene23472\_Sample\_011046840, Unigene43516\_Sample\_011046840, Unigene40814\_Sample\_011046840, Unigene7994\_Sample\_011046840, Unigene39346\_Sample\_011046840, Unigene41163\_Sample\_011046840, Unigene2665\_Sample\_011046840, Unigene43231\_Sample\_011046840, Unigene30367\_Sample\_011046840, Unigene38801\_Sample\_011046840, Unigene13661\_Sample\_011046840, Unigene39273\_Sample\_011046840, Unigene37919\_Sample\_011046840, Unigene43210\_Sample\_011046840, Unigene31306\_Sample\_011046840, Unigene37192\_Sample\_011046840, Unigene29808\_Sample\_011046840, Unigene8217\_Sample\_011046840, Unigene40496\_Sample\_011046840, Unigene11931\_Sample\_011046840, Unigene41811\_Sample\_011046840, Unigene29362\_Sample\_011046840, Unigene13726\_Sample\_011046840, Unigene27185\_Sample\_011046840, Unigene38555\_Sample\_011046840, Unigene6925\_Sample\_011046840, Unigene38595\_Sample\_011046840, Unigene39615\_Sample\_011046840, Unigene17602\_Sample\_011046840, Unigene25467\_Sample\_011046840, Unigene32098\_Sample\_011046840, Unigene5385\_Sample\_011046840, Unigene42983\_Sample\_011046840, Unigene15428\_Sample\_011046840, Unigene40745\_Sample\_011046840, Unigene31705\_Sample\_011046840, Unigene35001\_Sample\_011046840, Unigene40260\_Sample\_011046840, Unigene41879\_Sample\_011046840, Unigene32783\_Sample\_011046840, Unigene32240\_Sample\_011046840, Unigene21551\_Sample\_011046840, Unigene5962\_Sample\_011046840, Unigene32573\_Sample\_011046840, Unigene34972\_Sample\_011046840, Unigene28616\_Sample\_011046840, Unigene33492\_Sample\_011046840, Unigene26448\_Sample\_011046840, Unigene37083\_Sample\_011046840, Unigene17486\_Sample\_011046840, Unigene43573\_Sample\_011046840, Unigene25450\_Sample\_011046840, Unigene7951\_Sample\_011046840, Unigene28129\_Sample\_011046840, Unigene7302\_Sample\_011046840, Unigene43120\_Sample\_011046840, Unigene39480\_Sample\_011046840, Unigene33745\_Sample\_011046840, Unigene3204\_Sample\_011046840, Unigene8085\_Sample\_011046840, Unigene31899\_Sample\_011046840, Unigene6101\_Sample\_011046840, Unigene19985\_Sample\_011046840, Unigene38217\_Sample\_011046840, Unigene43342\_Sample\_011046840, Unigene37484\_Sample\_011046840, Unigene19344\_Sample\_011046840, Unigene43502\_Sample\_011046840, Unigene27982\_Sample\_011046840, Unigene31409\_Sample\_011046840, Unigene33338\_Sample\_011046840, Unigene12677\_Sample\_011046840, Unigene26172\_Sample\_011046840, Unigene43410\_Sample\_011046840, Unigene28710\_Sample\_011046840, Unigene33618\_Sample\_011046840, Unigene19933\_Sample\_011046840, Unigene34693\_Sample\_011046840, Unigene34288\_Sample\_011046840, Unigene31198\_Sample\_011046840, Unigene43301\_Sample\_011046840, Unigene26124\_Sample\_011046840, Unigene38940\_Sample\_011046840, Unigene21513\_Sample\_011046840, Unigene41283\_Sample\_011046840, Unigene37828\_Sample\_011046840, Unigene36734\_Sample\_011046840, Unigene40253\_Sample\_011046840, Unigene1368\_Sample\_011046840, Unigene42041\_Sample\_011046840, Unigene41981\_Sample\_011046840, Unigene43405\_Sample\_011046840, Unigene29875\_Sample\_011046840, Unigene1872\_Sample\_011046840, Unigene4145\_Sample\_011046840, Unigene23682\_Sample\_011046840, Unigene8198\_Sample\_011046840, Unigene40895\_Sample\_011046840, Unigene28610\_Sample\_011046840, Unigene40235\_Sample\_011046840, Unigene20869\_Sample\_011046840, Unigene39791\_Sample\_011046840, Unigene33873\_Sample\_011046840, Unigene40606\_Sample\_011046840, Unigene1379\_Sample\_011046840, Unigene41957\_Sample\_011046840, Unigene41383\_Sample\_011046840, Unigene26491\_Sample\_011046840, Unigene39843\_Sample\_011046840, Unigene3046\_Sample\_011046840, Unigene2709\_Sample\_011046840, Unigene37486\_Sample\_011046840, Unigene3212\_Sample\_011046840, Unigene3718\_Sample\_011046840, Unigene7203\_Sample\_011046840, Unigene13413\_Sample\_011046840, Unigene43289\_Sample\_011046840, Unigene36568\_Sample\_011046840, Unigene39778\_Sample\_011046840, Unigene22606\_Sample\_011046840, Unigene30720\_Sample\_011046840, Unigene14693\_Sample\_011046840, Unigene11201\_Sample\_011046840, Unigene15199\_Sample\_011046840, Unigene37213\_Sample\_011046840, Unigene7606\_Sample\_011046840, Unigene7813\_Sample\_011046840, Unigene332\_Sample\_011046840, Unigene7162\_Sample\_011046840, Unigene30599\_Sample\_011046840, Unigene36645\_Sample\_011046840, Unigene23950\_Sample\_011046840, Unigene36762\_Sample\_011046840, Unigene36732\_Sample\_011046840, Unigene41089\_Sample\_011046840, Unigene28034\_Sample\_011046840, Unigene24482\_Sample\_011046840, Unigene5166\_Sample\_011046840, Unigene20196\_Sample\_011046840, Unigene14589\_Sample\_011046840, Unigene19402\_Sample\_011046840, Unigene40547\_Sample\_011046840, Unigene39900\_Sample\_011046840, Unigene42906\_Sample\_011046840, Unigene16569\_Sample\_011046840, Unigene33705\_Sample\_011046840, Unigene38589\_Sample\_011046840, Unigene36323\_Sample\_011046840, Unigene36937\_Sample\_011046840, Unigene32067\_Sample\_011046840, Unigene40875\_Sample\_011046840, Unigene6764\_Sample\_011046840, Unigene20216\_Sample\_011046840, Unigene36996\_Sample\_011046840, Unigene38250\_Sample\_011046840, Unigene33344\_Sample\_011046840, Unigene41584\_Sample\_011046840, Unigene27067\_Sample\_011046840, Unigene18456\_Sample\_011046840, Unigene37506\_Sample\_011046840, Unigene39509\_Sample\_011046840, Unigene38767\_Sample\_011046840, Unigene5223\_Sample\_011046840, Unigene41805\_Sample\_011046840, Unigene38826\_Sample\_011046840, Unigene36211\_Sample\_011046840, Unigene43372\_Sample\_011046840, Unigene40587\_Sample\_011046840, Unigene39481\_Sample\_011046840, Unigene32614\_Sample\_011046840, Unigene26668\_Sample\_011046840, Unigene37136\_Sample\_011046840, Unigene29542\_Sample\_011046840, Unigene13327\_Sample\_011046840, Unigene42278\_Sample\_011046840, Unigene14602\_Sample\_011046840, Unigene40732\_Sample\_011046840, Unigene28853\_Sample\_011046840, Unigene8413\_Sample\_011046840, Unigene17765\_Sample\_011046840, Unigene42337\_Sample\_011046840, Unigene35167\_Sample\_011046840, Unigene41299\_Sample\_011046840, Unigene6283\_Sample\_011046840, Unigene27639\_Sample\_011046840, Unigene41061\_Sample\_011046840, Unigene11146\_Sample\_011046840, Unigene30715\_Sample\_011046840, Unigene37236\_Sample\_011046840, Unigene32566\_Sample\_011046840, Unigene33878\_Sample\_011046840, Unigene34566\_Sample\_011046840, Unigene26402\_Sample\_011046840, Unigene43585\_Sample\_011046840, Unigene38285\_Sample\_011046840, Unigene11248\_Sample\_011046840, Unigene14392\_Sample\_011046840, Unigene4139\_Sample\_011046840, Unigene36839\_Sample\_011046840, Unigene24400\_Sample\_011046840, Unigene36971\_Sample\_011046840, Unigene40950\_Sample\_011046840, Unigene31827\_Sample\_011046840, Unigene33088\_Sample\_011046840, Unigene36063\_Sample\_011046840, Unigene18251\_Sample\_011046840, Unigene42487\_Sample\_011046840, Unigene15903\_Sample\_011046840, Unigene35023\_Sample\_011046840, Unigene6842\_Sample\_011046840, Unigene32606\_Sample\_011046840, Unigene13197\_Sample\_011046840, Unigene5985\_Sample\_011046840, Unigene31283\_Sample\_011046840, Unigene41983\_Sample\_011046840, Unigene21387\_Sample\_011046840, Unigene27604\_Sample\_011046840, Unigene32554\_Sample\_011046840, Unigene17650\_Sample\_011046840, Unigene38526\_Sample\_011046840, Unigene35976\_Sample\_011046840, Unigene40764\_Sample\_011046840, Unigene27667\_Sample\_011046840, Unigene19027\_Sample\_011046840, Unigene7353\_Sample\_011046840, Unigene32180\_Sample\_011046840, Unigene15237\_Sample\_011046840, Unigene40726\_Sample\_011046840, Unigene42638\_Sample\_011046840, Unigene19219\_Sample\_011046840, Unigene5707\_Sample\_011046840, Unigene17339\_Sample\_011046840, Unigene35227\_Sample\_011046840, Unigene38242\_Sample\_011046840, Unigene33279\_Sample\_011046840, Unigene24546\_Sample\_011046840, Unigene25298\_Sample\_011046840, Unigene31759\_Sample\_011046840, Unigene12410\_Sample\_011046840, Unigene38772\_Sample\_011046840, Unigene37440\_Sample\_011046840, Unigene28079\_Sample\_011046840, Unigene10436\_Sample\_011046840, Unigene43064\_Sample\_011046840, Unigene40520\_Sample\_011046840, Unigene32702\_Sample\_011046840, Unigene29405\_Sample\_011046840, Unigene39983\_Sample\_011046840, Unigene19991\_Sample\_011046840, Unigene42622\_Sample\_011046840, Unigene41793\_Sample\_011046840, Unigene39948\_Sample\_011046840, Unigene5658\_Sample\_011046840, Unigene33671\_Sample\_011046840, Unigene42763\_Sample\_011046840, Unigene38378\_Sample\_011046840, Unigene13641\_Sample\_011046840, Unigene6346\_Sample\_011046840, Unigene14981\_Sample\_011046840, Unigene3828\_Sample\_011046840, Unigene6395\_Sample\_011046840, Unigene2916\_Sample\_011046840, Unigene40716\_Sample\_011046840, Unigene29928\_Sample\_011046840, Unigene22209\_Sample\_011046840, Unigene11244\_Sample\_011046840, Unigene8189\_Sample\_011046840, Unigene40234\_Sample\_011046840, Unigene33379\_Sample\_011046840, Unigene39123\_Sample\_011046840, Unigene37694\_Sample\_011046840, Unigene12204\_Sample\_011046840, Unigene19740\_Sample\_011046840, Unigene4042\_Sample\_011046840, Unigene39967\_Sample\_011046840, Unigene43198\_Sample\_011046840, Unigene41086\_Sample\_011046840, Unigene11516\_Sample\_011046840, Unigene31973\_Sample\_011046840, Unigene39052\_Sample\_011046840, Unigene38279\_Sample\_011046840, Unigene25915\_Sample\_011046840, Unigene7631\_Sample\_011046840, Unigene30400\_Sample\_011046840, Unigene41681\_Sample\_011046840, Unigene29592\_Sample\_011046840, Unigene3486\_Sample\_011046840, Unigene38262\_Sample\_011046840, Unigene34754\_Sample\_011046840, Unigene35053\_Sample\_011046840, Unigene42702\_Sample\_011046840, Unigene11883\_Sample\_011046840, Unigene42866\_Sample\_011046840, Unigene40010\_Sample\_011046840, Unigene37330\_Sample\_011046840, Unigene21296\_Sample\_011046840, Unigene7708\_Sample\_011046840, Unigene37921\_Sample\_011046840, Unigene20922\_Sample\_011046840, Unigene28301\_Sample\_011046840, Unigene41242\_Sample\_011046840, Unigene41510\_Sample\_011046840, Unigene27805\_Sample\_011046840, Unigene43165\_Sample\_011046840, Unigene32262\_Sample\_011046840, Unigene22393\_Sample\_011046840, Unigene16493\_Sample\_011046840, Unigene18168\_Sample\_011046840, Unigene11142\_Sample\_011046840, Unigene32307\_Sample\_011046840, Unigene42027\_Sample\_011046840, Unigene21554\_Sample\_011046840, Unigene8130\_Sample\_011046840, Unigene20146\_Sample\_011046840, Unigene7172\_Sample\_011046840, Unigene42096\_Sample\_011046840, Unigene5647\_Sample\_011046840, Unigene41869\_Sample\_011046840, Unigene19505\_Sample\_011046840, Unigene16302\_Sample\_011046840, Unigene36983\_Sample\_011046840, Unigene37827\_Sample\_011046840, Unigene24404\_Sample\_011046840, Unigene42692\_Sample\_011046840, Unigene40128\_Sample\_011046840, Unigene25020\_Sample\_011046840, Unigene43593\_Sample\_011046840, Unigene11571\_Sample\_011046840, Unigene28366\_Sample\_011046840, Unigene24019\_Sample\_011046840, Unigene7767\_Sample\_011046840, Unigene43201\_Sample\_011046840, Unigene41281\_Sample\_011046840, Unigene14437\_Sample\_011046840, Unigene7768\_Sample\_011046840, Unigene14257\_Sample\_011046840 |
| receptor activity | Unigene7411\_Sample\_011046840, Unigene6130\_Sample\_011046840, Unigene5229\_Sample\_011046840, Unigene8071\_Sample\_011046840, Unigene8904\_Sample\_011046840, Unigene43219\_Sample\_011046840, Unigene9833\_Sample\_011046840, Unigene6640\_Sample\_011046840, Unigene9447\_Sample\_011046840, Unigene35787\_Sample\_011046840, Unigene22291\_Sample\_011046840, Unigene24702\_Sample\_011046840, Unigene18019\_Sample\_011046840, Unigene14773\_Sample\_011046840, Unigene3514\_Sample\_011046840, Unigene39131\_Sample\_011046840, Unigene4082\_Sample\_011046840, Unigene28433\_Sample\_011046840, Unigene1227\_Sample\_011046840, Unigene36838\_Sample\_011046840, Unigene1022\_Sample\_011046840, Unigene41164\_Sample\_011046840, Unigene12089\_Sample\_011046840, Unigene31423\_Sample\_011046840, Unigene33816\_Sample\_011046840, Unigene991\_Sample\_011046840, Unigene43556\_Sample\_011046840, Unigene13511\_Sample\_011046840, Unigene42483\_Sample\_011046840, Unigene42712\_Sample\_011046840, Unigene8061\_Sample\_011046840, Unigene27684\_Sample\_011046840, Unigene16719\_Sample\_011046840, Unigene31791\_Sample\_011046840, Unigene24549\_Sample\_011046840, Unigene13947\_Sample\_011046840, Unigene39781\_Sample\_011046840, Unigene4544\_Sample\_011046840, Unigene32732\_Sample\_011046840, Unigene38931\_Sample\_011046840, Unigene23968\_Sample\_011046840, Unigene35833\_Sample\_011046840, Unigene43124\_Sample\_011046840, Unigene38611\_Sample\_011046840, Unigene11616\_Sample\_011046840, Unigene39891\_Sample\_011046840, Unigene29777\_Sample\_011046840, Unigene22858\_Sample\_011046840, Unigene23331\_Sample\_011046840, Unigene6225\_Sample\_011046840, Unigene12197\_Sample\_011046840, Unigene34704\_Sample\_011046840, Unigene24664\_Sample\_011046840, Unigene4724\_Sample\_011046840, Unigene39066\_Sample\_011046840, Unigene3510\_Sample\_011046840, Unigene14031\_Sample\_011046840, Unigene9168\_Sample\_011046840, Unigene7529\_Sample\_011046840, Unigene6500\_Sample\_011046840, Unigene24844\_Sample\_011046840, Unigene2505\_Sample\_011046840, Unigene1555\_Sample\_011046840, Unigene12964\_Sample\_011046840, Unigene2991\_Sample\_011046840, Unigene40896\_Sample\_011046840, Unigene19234\_Sample\_011046840, Unigene4138\_Sample\_011046840, Unigene2784\_Sample\_011046840, Unigene42371\_Sample\_011046840, Unigene2908\_Sample\_011046840, Unigene1830\_Sample\_011046840, Unigene34595\_Sample\_011046840, Unigene19342\_Sample\_011046840, Unigene35615\_Sample\_011046840, Unigene4668\_Sample\_011046840, Unigene15244\_Sample\_011046840, Unigene8010\_Sample\_011046840, Unigene6702\_Sample\_011046840, Unigene4464\_Sample\_011046840, Unigene8112\_Sample\_011046840, Unigene12012\_Sample\_011046840, Unigene34166\_Sample\_011046840, Unigene6292\_Sample\_011046840, Unigene27237\_Sample\_011046840, Unigene1814\_Sample\_011046840, Unigene33501\_Sample\_011046840, Unigene39912\_Sample\_011046840, Unigene33524\_Sample\_011046840, Unigene40286\_Sample\_011046840, Unigene4415\_Sample\_011046840, Unigene5178\_Sample\_011046840, Unigene34807\_Sample\_011046840, Unigene7904\_Sample\_011046840, Unigene42364\_Sample\_011046840, Unigene43240\_Sample\_011046840, Unigene37023\_Sample\_011046840, Unigene41643\_Sample\_011046840, Unigene33967\_Sample\_011046840, Unigene1495\_Sample\_011046840, Unigene6302\_Sample\_011046840, Unigene5527\_Sample\_011046840, Unigene1671\_Sample\_011046840, Unigene34759\_Sample\_011046840, Unigene28117\_Sample\_011046840, Unigene39350\_Sample\_011046840, Unigene42488\_Sample\_011046840, Unigene2864\_Sample\_011046840, Unigene43635\_Sample\_011046840, Unigene1290\_Sample\_011046840, Unigene1213\_Sample\_011046840, Unigene29758\_Sample\_011046840, Unigene21962\_Sample\_011046840, Unigene43472\_Sample\_011046840, Unigene39854\_Sample\_011046840, Unigene39895\_Sample\_011046840, Unigene6407\_Sample\_011046840, Unigene5429\_Sample\_011046840, Unigene43413\_Sample\_011046840, Unigene3935\_Sample\_011046840, Unigene6486\_Sample\_011046840, Unigene39296\_Sample\_011046840, Unigene26954\_Sample\_011046840, Unigene40074\_Sample\_011046840, Unigene34842\_Sample\_011046840, Unigene43174\_Sample\_011046840, Unigene7125\_Sample\_011046840, Unigene29066\_Sample\_011046840, Unigene22001\_Sample\_011046840, Unigene39133\_Sample\_011046840, Unigene30378\_Sample\_011046840, Unigene38605\_Sample\_011046840, Unigene31994\_Sample\_011046840, Unigene32025\_Sample\_011046840, Unigene7966\_Sample\_011046840, Unigene24546\_Sample\_011046840, Unigene19130\_Sample\_011046840, Unigene32809\_Sample\_011046840, Unigene6753\_Sample\_011046840, Unigene33208\_Sample\_011046840, Unigene21396\_Sample\_011046840, Unigene36100\_Sample\_011046840, Unigene28229\_Sample\_011046840, Unigene16578\_Sample\_011046840, Unigene30821\_Sample\_011046840, Unigene14691\_Sample\_011046840, Unigene35159\_Sample\_011046840, Unigene23913\_Sample\_011046840, Unigene38420\_Sample\_011046840, Unigene41234\_Sample\_011046840, Unigene34183\_Sample\_011046840, Unigene5658\_Sample\_011046840, Unigene41688\_Sample\_011046840, Unigene9954\_Sample\_011046840, Unigene37620\_Sample\_011046840, Unigene25203\_Sample\_011046840, Unigene2121\_Sample\_011046840, Unigene18939\_Sample\_011046840, Unigene7448\_Sample\_011046840, Unigene19514\_Sample\_011046840, Unigene3753\_Sample\_011046840, Unigene511\_Sample\_011046840, Unigene43160\_Sample\_011046840, Unigene7889\_Sample\_011046840, Unigene38003\_Sample\_011046840, Unigene8660\_Sample\_011046840, Unigene38778\_Sample\_011046840, Unigene37597\_Sample\_011046840, Unigene25323\_Sample\_011046840, Unigene41538\_Sample\_011046840, Unigene7682\_Sample\_011046840, Unigene38476\_Sample\_011046840, Unigene39533\_Sample\_011046840, Unigene41135\_Sample\_011046840, Unigene42460\_Sample\_011046840, Unigene3344\_Sample\_011046840, Unigene5122\_Sample\_011046840, Unigene4443\_Sample\_011046840, Unigene34920\_Sample\_011046840, Unigene1076\_Sample\_011046840, Unigene6245\_Sample\_011046840, Unigene20308\_Sample\_011046840, Unigene1039\_Sample\_011046840, Unigene35367\_Sample\_011046840, Unigene1790\_Sample\_011046840, Unigene32905\_Sample\_011046840, Unigene24378\_Sample\_011046840, Unigene8956\_Sample\_011046840, Unigene35556\_Sample\_011046840, Unigene28095\_Sample\_011046840, Unigene39894\_Sample\_011046840, Unigene34508\_Sample\_011046840, Unigene4591\_Sample\_011046840, Unigene32730\_Sample\_011046840, Unigene40917\_Sample\_011046840, Unigene40877\_Sample\_011046840, Unigene24508\_Sample\_011046840, Unigene2304\_Sample\_011046840, Unigene6727\_Sample\_011046840, Unigene39223\_Sample\_011046840, Unigene11150\_Sample\_011046840, Unigene12103\_Sample\_011046840, Unigene32285\_Sample\_011046840, Unigene42436\_Sample\_011046840, Unigene41319\_Sample\_011046840, Unigene26130\_Sample\_011046840, Unigene40539\_Sample\_011046840, Unigene13251\_Sample\_011046840, Unigene24573\_Sample\_011046840, Unigene32546\_Sample\_011046840, Unigene6828\_Sample\_011046840, Unigene38712\_Sample\_011046840, Unigene35839\_Sample\_011046840, Unigene42641\_Sample\_011046840, Unigene31614\_Sample\_011046840, Unigene18735\_Sample\_011046840, Unigene36041\_Sample\_011046840, Unigene24751\_Sample\_011046840, Unigene39259\_Sample\_011046840, Unigene2640\_Sample\_011046840, Unigene24163\_Sample\_011046840, Unigene40790\_Sample\_011046840, Unigene28292\_Sample\_011046840, Unigene7697\_Sample\_011046840, Unigene5647\_Sample\_011046840, Unigene7526\_Sample\_011046840, Unigene25202\_Sample\_011046840, Unigene8144\_Sample\_011046840, Unigene4123\_Sample\_011046840, Unigene4617\_Sample\_011046840, Unigene24796\_Sample\_011046840, Unigene412\_Sample\_011046840, Unigene21699\_Sample\_011046840, Unigene4674\_Sample\_011046840, Unigene42692\_Sample\_011046840, Unigene29789\_Sample\_011046840, Unigene36731\_Sample\_011046840, Unigene39399\_Sample\_011046840, Unigene24252\_Sample\_011046840, Unigene23327\_Sample\_011046840, Unigene29197\_Sample\_011046840, Unigene43146\_Sample\_011046840, Unigene17625\_Sample\_011046840, Unigene40205\_Sample\_011046840, Unigene5893\_Sample\_011046840, Unigene30504\_Sample\_011046840, Unigene2261\_Sample\_011046840, Unigene31437\_Sample\_011046840, Unigene7572\_Sample\_011046840, Unigene6624\_Sample\_011046840, Unigene35061\_Sample\_011046840, Unigene42555\_Sample\_011046840, Unigene4735\_Sample\_011046840 |
| enzyme binding | Unigene25304\_Sample\_011046840, Unigene36484\_Sample\_011046840, Unigene10050\_Sample\_011046840, Unigene39623\_Sample\_011046840, Unigene40547\_Sample\_011046840, Unigene26262\_Sample\_011046840, Unigene24250\_Sample\_011046840, Unigene13805\_Sample\_011046840, Unigene15881\_Sample\_011046840, Unigene29240\_Sample\_011046840, Unigene14438\_Sample\_011046840, Unigene31802\_Sample\_011046840, Unigene18354\_Sample\_011046840, Unigene33915\_Sample\_011046840, Unigene1549\_Sample\_011046840, Unigene3514\_Sample\_011046840, Unigene23133\_Sample\_011046840, Unigene6293\_Sample\_011046840, Unigene6863\_Sample\_011046840, Unigene32488\_Sample\_011046840, Unigene2986\_Sample\_011046840, Unigene35141\_Sample\_011046840, Unigene43556\_Sample\_011046840, Unigene24257\_Sample\_011046840, Unigene25180\_Sample\_011046840, Unigene34913\_Sample\_011046840, Unigene33356\_Sample\_011046840, Unigene39096\_Sample\_011046840, Unigene7825\_Sample\_011046840, Unigene29542\_Sample\_011046840, Unigene39752\_Sample\_011046840, Unigene26069\_Sample\_011046840, Unigene25523\_Sample\_011046840, Unigene28087\_Sample\_011046840, Unigene33395\_Sample\_011046840, Unigene43453\_Sample\_011046840, Unigene19793\_Sample\_011046840, Unigene29670\_Sample\_011046840, Unigene10776\_Sample\_011046840, Unigene9599\_Sample\_011046840, Unigene38722\_Sample\_011046840, Unigene31072\_Sample\_011046840, Unigene6033\_Sample\_011046840, Unigene37829\_Sample\_011046840, Unigene43300\_Sample\_011046840, Unigene7174\_Sample\_011046840, Unigene42298\_Sample\_011046840, Unigene19332\_Sample\_011046840, Unigene12197\_Sample\_011046840, Unigene41252\_Sample\_011046840, Unigene3258\_Sample\_011046840, Unigene33646\_Sample\_011046840, Unigene36437\_Sample\_011046840, Unigene7299\_Sample\_011046840, Unigene8171\_Sample\_011046840, Unigene27924\_Sample\_011046840, Unigene42110\_Sample\_011046840, Unigene5335\_Sample\_011046840, Unigene38381\_Sample\_011046840, Unigene3161\_Sample\_011046840, Unigene37014\_Sample\_011046840, Unigene7064\_Sample\_011046840, Unigene38254\_Sample\_011046840, Unigene4464\_Sample\_011046840, Unigene28660\_Sample\_011046840, Unigene4245\_Sample\_011046840, Unigene41562\_Sample\_011046840, Unigene16770\_Sample\_011046840, Unigene2672\_Sample\_011046840, Unigene33984\_Sample\_011046840, Unigene35368\_Sample\_011046840, Unigene39271\_Sample\_011046840, Unigene37404\_Sample\_011046840, Unigene33954\_Sample\_011046840, Unigene39950\_Sample\_011046840, Unigene37890\_Sample\_011046840, Unigene34402\_Sample\_011046840, Unigene35796\_Sample\_011046840, Unigene30694\_Sample\_011046840, Unigene11179\_Sample\_011046840, Unigene43371\_Sample\_011046840, Unigene40994\_Sample\_011046840, Unigene21171\_Sample\_011046840, Unigene43435\_Sample\_011046840, Unigene43423\_Sample\_011046840, Unigene37688\_Sample\_011046840, Unigene2180\_Sample\_011046840, Unigene4818\_Sample\_011046840, Unigene43303\_Sample\_011046840, Unigene40876\_Sample\_011046840, Unigene19732\_Sample\_011046840, Unigene6147\_Sample\_011046840, Unigene32180\_Sample\_011046840, Unigene29681\_Sample\_011046840, Unigene39224\_Sample\_011046840, Unigene1150\_Sample\_011046840, Unigene25527\_Sample\_011046840, Unigene28840\_Sample\_011046840, Unigene38030\_Sample\_011046840, Unigene25325\_Sample\_011046840, Unigene35929\_Sample\_011046840, Unigene31471\_Sample\_011046840, Unigene43077\_Sample\_011046840, Unigene43465\_Sample\_011046840, Unigene7175\_Sample\_011046840, Unigene37474\_Sample\_011046840, Unigene34369\_Sample\_011046840, Unigene1389\_Sample\_011046840, Unigene19915\_Sample\_011046840, Unigene43211\_Sample\_011046840, Unigene42927\_Sample\_011046840, Unigene6209\_Sample\_011046840, Unigene34039\_Sample\_011046840, Unigene34514\_Sample\_011046840, Unigene30767\_Sample\_011046840, Unigene41124\_Sample\_011046840, Unigene25450\_Sample\_011046840, Unigene15111\_Sample\_011046840, Unigene18234\_Sample\_011046840, Unigene32316\_Sample\_011046840, Unigene38328\_Sample\_011046840, Unigene43155\_Sample\_011046840, Unigene42335\_Sample\_011046840, Unigene23778\_Sample\_011046840, Unigene43358\_Sample\_011046840, Unigene2418\_Sample\_011046840, Unigene32858\_Sample\_011046840, Unigene19991\_Sample\_011046840, Unigene42798\_Sample\_011046840, Unigene33926\_Sample\_011046840, Unigene38795\_Sample\_011046840, Unigene11613\_Sample\_011046840, Unigene34472\_Sample\_011046840, Unigene31822\_Sample\_011046840, Unigene35007\_Sample\_011046840, Unigene43502\_Sample\_011046840, Unigene32023\_Sample\_011046840, Unigene36800\_Sample\_011046840, Unigene18009\_Sample\_011046840, Unigene33106\_Sample\_011046840, Unigene23234\_Sample\_011046840, Unigene31079\_Sample\_011046840, Unigene38612\_Sample\_011046840, Unigene34055\_Sample\_011046840, Unigene32620\_Sample\_011046840, Unigene8660\_Sample\_011046840, Unigene33260\_Sample\_011046840, Unigene31309\_Sample\_011046840, Unigene18652\_Sample\_011046840, Unigene32609\_Sample\_011046840, Unigene9133\_Sample\_011046840, Unigene43301\_Sample\_011046840, Unigene37728\_Sample\_011046840, Unigene43045\_Sample\_011046840, Unigene43407\_Sample\_011046840, Unigene25787\_Sample\_011046840, Unigene11904\_Sample\_011046840, Unigene14598\_Sample\_011046840, Unigene8203\_Sample\_011046840, Unigene42629\_Sample\_011046840, Unigene16406\_Sample\_011046840, Unigene41092\_Sample\_011046840, Unigene26711\_Sample\_011046840, Unigene16855\_Sample\_011046840, Unigene37207\_Sample\_011046840, Unigene43405\_Sample\_011046840, Unigene28752\_Sample\_011046840, Unigene8219\_Sample\_011046840, Unigene31598\_Sample\_011046840, Unigene42169\_Sample\_011046840, Unigene12936\_Sample\_011046840, Unigene40016\_Sample\_011046840, Unigene42905\_Sample\_011046840, Unigene27260\_Sample\_011046840, Unigene9733\_Sample\_011046840, Unigene29541\_Sample\_011046840, Unigene43330\_Sample\_011046840, Unigene34731\_Sample\_011046840, Unigene20330\_Sample\_011046840, Unigene40861\_Sample\_011046840, Unigene42838\_Sample\_011046840, Unigene35148\_Sample\_011046840, Unigene40606\_Sample\_011046840, Unigene41574\_Sample\_011046840, Unigene43455\_Sample\_011046840, Unigene3046\_Sample\_011046840, Unigene4825\_Sample\_011046840, Unigene31090\_Sample\_011046840, Unigene30936\_Sample\_011046840, Unigene43289\_Sample\_011046840, Unigene36431\_Sample\_011046840, Unigene15302\_Sample\_011046840, Unigene37680\_Sample\_011046840, Unigene9113\_Sample\_011046840, Unigene8144\_Sample\_011046840, Unigene412\_Sample\_011046840, Unigene21629\_Sample\_011046840, Unigene7664\_Sample\_011046840, Unigene39399\_Sample\_011046840, Unigene42173\_Sample\_011046840, Unigene42356\_Sample\_011046840, Unigene38453\_Sample\_011046840, Unigene23373\_Sample\_011046840, Unigene42301\_Sample\_011046840, Unigene22059\_Sample\_011046840, Unigene28034\_Sample\_011046840, Unigene43019\_Sample\_011046840 |
| RNA helicase activity | Unigene14503\_Sample\_011046840, Unigene25915\_Sample\_011046840, Unigene34380\_Sample\_011046840, Unigene38687\_Sample\_011046840, Unigene40716\_Sample\_011046840, Unigene42997\_Sample\_011046840, Unigene36773\_Sample\_011046840, Unigene4686\_Sample\_011046840, Unigene22397\_Sample\_011046840, Unigene34909\_Sample\_011046840, Unigene15696\_Sample\_011046840, Unigene4529\_Sample\_011046840, Unigene38658\_Sample\_011046840, Unigene38115\_Sample\_011046840, Unigene26865\_Sample\_011046840, Unigene7067\_Sample\_011046840, Unigene43165\_Sample\_011046840, Unigene32621\_Sample\_011046840, Unigene38370\_Sample\_011046840, Unigene42502\_Sample\_011046840, Unigene5223\_Sample\_011046840, Unigene37077\_Sample\_011046840, Unigene19985\_Sample\_011046840, Unigene11516\_Sample\_011046840, Unigene31458\_Sample\_011046840, Unigene31973\_Sample\_011046840, Unigene39732\_Sample\_011046840 |
| transferase activity, transferring one-carbon groups | Unigene35365\_Sample\_011046840, Unigene1374\_Sample\_011046840, Unigene19879\_Sample\_011046840, Unigene5289\_Sample\_011046840, Unigene18167\_Sample\_011046840, Unigene34118\_Sample\_011046840, Unigene40942\_Sample\_011046840, Unigene37584\_Sample\_011046840, Unigene14555\_Sample\_011046840, Unigene7660\_Sample\_011046840, Unigene19630\_Sample\_011046840, Unigene1115\_Sample\_011046840, Unigene23827\_Sample\_011046840, Unigene32249\_Sample\_011046840, Unigene31134\_Sample\_011046840, Unigene9531\_Sample\_011046840, Unigene5355\_Sample\_011046840, Unigene7455\_Sample\_011046840, Unigene39797\_Sample\_011046840, Unigene20455\_Sample\_011046840, Unigene32673\_Sample\_011046840, Unigene35900\_Sample\_011046840, Unigene33670\_Sample\_011046840, Unigene42990\_Sample\_011046840, Unigene37878\_Sample\_011046840, Unigene28341\_Sample\_011046840, Unigene18600\_Sample\_011046840, Unigene32400\_Sample\_011046840, Unigene23853\_Sample\_011046840, Unigene22543\_Sample\_011046840, Unigene20463\_Sample\_011046840, Unigene39864\_Sample\_011046840, Unigene28263\_Sample\_011046840, Unigene42308\_Sample\_011046840, Unigene41318\_Sample\_011046840, Unigene22491\_Sample\_011046840, Unigene43254\_Sample\_011046840, Unigene13709\_Sample\_011046840, Unigene14334\_Sample\_011046840, Unigene31928\_Sample\_011046840, Unigene33907\_Sample\_011046840, Unigene38468\_Sample\_011046840, Unigene16\_Sample\_011046840, Unigene36841\_Sample\_011046840, Unigene30744\_Sample\_011046840, Unigene11128\_Sample\_011046840, Unigene37229\_Sample\_011046840, Unigene23988\_Sample\_011046840, Unigene22424\_Sample\_011046840, Unigene17289\_Sample\_011046840, Unigene39760\_Sample\_011046840, Unigene28923\_Sample\_011046840, Unigene26208\_Sample\_011046840, Unigene24164\_Sample\_011046840, Unigene40277\_Sample\_011046840, Unigene17535\_Sample\_011046840, Unigene23982\_Sample\_011046840, Unigene16250\_Sample\_011046840, Unigene10938\_Sample\_011046840, Unigene29183\_Sample\_011046840, Unigene34136\_Sample\_011046840, Unigene18649\_Sample\_011046840, Unigene43511\_Sample\_011046840, Unigene41268\_Sample\_011046840, Unigene36089\_Sample\_011046840, Unigene32108\_Sample\_011046840, Unigene30304\_Sample\_011046840, Unigene41631\_Sample\_011046840, Unigene3223\_Sample\_011046840, Unigene37234\_Sample\_011046840, Unigene10859\_Sample\_011046840, Unigene10222\_Sample\_011046840, Unigene39512\_Sample\_011046840, Unigene19413\_Sample\_011046840, Unigene43238\_Sample\_011046840, Unigene27123\_Sample\_011046840, Unigene7405\_Sample\_011046840, Unigene37589\_Sample\_011046840, Unigene23432\_Sample\_011046840, Unigene38459\_Sample\_011046840, Unigene6960\_Sample\_011046840, Unigene36588\_Sample\_011046840, Unigene18930\_Sample\_011046840, Unigene43414\_Sample\_011046840, Unigene42939\_Sample\_011046840, Unigene31322\_Sample\_011046840, Unigene42382\_Sample\_011046840, Unigene38077\_Sample\_011046840, Unigene36028\_Sample\_011046840, Unigene36108\_Sample\_011046840, Unigene31950\_Sample\_011046840, Unigene34497\_Sample\_011046840, Unigene40355\_Sample\_011046840, Unigene1789\_Sample\_011046840, Unigene29510\_Sample\_011046840, Unigene19321\_Sample\_011046840, Unigene32980\_Sample\_011046840, Unigene25030\_Sample\_011046840, Unigene31913\_Sample\_011046840, Unigene41549\_Sample\_011046840, Unigene36185\_Sample\_011046840, Unigene16218\_Sample\_011046840, Unigene29786\_Sample\_011046840, Unigene20407\_Sample\_011046840, Unigene24992\_Sample\_011046840, Unigene8347\_Sample\_011046840, Unigene29641\_Sample\_011046840, Unigene16116\_Sample\_011046840, Unigene39836\_Sample\_011046840, Unigene21467\_Sample\_011046840, Unigene37796\_Sample\_011046840, Unigene40012\_Sample\_011046840, Unigene21991\_Sample\_011046840, Unigene30185\_Sample\_011046840, Unigene36929\_Sample\_011046840, Unigene8751\_Sample\_011046840, Unigene17837\_Sample\_011046840, Unigene20612\_Sample\_011046840, Unigene42731\_Sample\_011046840, Unigene40921\_Sample\_011046840, Unigene27856\_Sample\_011046840, Unigene41585\_Sample\_011046840, Unigene12776\_Sample\_011046840, Unigene43297\_Sample\_011046840, Unigene19595\_Sample\_011046840, Unigene42114\_Sample\_011046840, Unigene24318\_Sample\_011046840, Unigene32934\_Sample\_011046840, Unigene29746\_Sample\_011046840, Unigene40663\_Sample\_011046840, Unigene31492\_Sample\_011046840, Unigene40951\_Sample\_011046840, Unigene36605\_Sample\_011046840, Unigene24074\_Sample\_011046840, Unigene20327\_Sample\_011046840, Unigene36299\_Sample\_011046840, Unigene7894\_Sample\_011046840, Unigene16649\_Sample\_011046840, Unigene30181\_Sample\_011046840, Unigene39940\_Sample\_011046840, Unigene33698\_Sample\_011046840, Unigene35348\_Sample\_011046840, Unigene6312\_Sample\_011046840, Unigene41523\_Sample\_011046840, Unigene584\_Sample\_011046840, Unigene31598\_Sample\_011046840, Unigene322\_Sample\_011046840, Unigene11883\_Sample\_011046840, Unigene20860\_Sample\_011046840, Unigene6075\_Sample\_011046840, Unigene17585\_Sample\_011046840, Unigene36900\_Sample\_011046840, Unigene19182\_Sample\_011046840, Unigene32413\_Sample\_011046840, Unigene38376\_Sample\_011046840, Unigene40961\_Sample\_011046840, Unigene42554\_Sample\_011046840, Unigene10034\_Sample\_011046840, Unigene21352\_Sample\_011046840, Unigene33517\_Sample\_011046840, Unigene38953\_Sample\_011046840, Unigene9553\_Sample\_011046840, Unigene41716\_Sample\_011046840, Unigene27722\_Sample\_011046840, Unigene40573\_Sample\_011046840, Unigene43096\_Sample\_011046840, Unigene15302\_Sample\_011046840, Unigene36353\_Sample\_011046840, Unigene2307\_Sample\_011046840, Unigene41974\_Sample\_011046840, Unigene12534\_Sample\_011046840, Unigene30981\_Sample\_011046840, Unigene9937\_Sample\_011046840, Unigene32331\_Sample\_011046840, Unigene43473\_Sample\_011046840, Unigene33465\_Sample\_011046840, Unigene29113\_Sample\_011046840, Unigene41002\_Sample\_011046840, Unigene42620\_Sample\_011046840, Unigene39406\_Sample\_011046840, Unigene36942\_Sample\_011046840, Unigene34241\_Sample\_011046840, Unigene21268\_Sample\_011046840, Unigene14188\_Sample\_011046840, Unigene42306\_Sample\_011046840 |
| procollagen-proline dioxygenase activity | Unigene20821\_Sample\_011046840, Unigene1619\_Sample\_011046840, Unigene3068\_Sample\_011046840, Unigene42972\_Sample\_011046840, Unigene36713\_Sample\_011046840, Unigene13285\_Sample\_011046840, Unigene5876\_Sample\_011046840, Unigene41821\_Sample\_011046840, Unigene11684\_Sample\_011046840, Unigene30757\_Sample\_011046840, Unigene33029\_Sample\_011046840, Unigene3029\_Sample\_011046840, Unigene32005\_Sample\_011046840, Unigene31901\_Sample\_011046840, Unigene32895\_Sample\_011046840, Unigene4570\_Sample\_011046840, Unigene7134\_Sample\_011046840 |
| peptidyl-proline dioxygenase activity | Unigene20821\_Sample\_011046840, Unigene1619\_Sample\_011046840, Unigene3068\_Sample\_011046840, Unigene42972\_Sample\_011046840, Unigene36713\_Sample\_011046840, Unigene13285\_Sample\_011046840, Unigene5876\_Sample\_011046840, Unigene41821\_Sample\_011046840, Unigene11684\_Sample\_011046840, Unigene30757\_Sample\_011046840, Unigene33029\_Sample\_011046840, Unigene3029\_Sample\_011046840, Unigene32005\_Sample\_011046840, Unigene31901\_Sample\_011046840, Unigene32895\_Sample\_011046840, Unigene4570\_Sample\_011046840, Unigene7134\_Sample\_011046840 |
| adenyl nucleotide binding | Unigene38023\_Sample\_011046840, Unigene26178\_Sample\_011046840, Unigene39461\_Sample\_011046840, Unigene20405\_Sample\_011046840, Unigene38706\_Sample\_011046840, Unigene9443\_Sample\_011046840, Unigene368\_Sample\_011046840, Unigene3429\_Sample\_011046840, Unigene37568\_Sample\_011046840, Unigene19401\_Sample\_011046840, Unigene41047\_Sample\_011046840, Unigene18730\_Sample\_011046840, Unigene14932\_Sample\_011046840, Unigene43154\_Sample\_011046840, Unigene902\_Sample\_011046840, Unigene43529\_Sample\_011046840, Unigene27378\_Sample\_011046840, Unigene22180\_Sample\_011046840, Unigene43365\_Sample\_011046840, Unigene20380\_Sample\_011046840, Unigene21224\_Sample\_011046840, Unigene27537\_Sample\_011046840, Unigene7557\_Sample\_011046840, Unigene42372\_Sample\_011046840, Unigene31094\_Sample\_011046840, Unigene41153\_Sample\_011046840, Unigene20314\_Sample\_011046840, Unigene32455\_Sample\_011046840, Unigene15132\_Sample\_011046840, Unigene42642\_Sample\_011046840, Unigene35962\_Sample\_011046840, Unigene4259\_Sample\_011046840, Unigene4677\_Sample\_011046840, Unigene36928\_Sample\_011046840, Unigene24531\_Sample\_011046840, Unigene32335\_Sample\_011046840, Unigene42401\_Sample\_011046840, Unigene25485\_Sample\_011046840, Unigene6748\_Sample\_011046840, Unigene34978\_Sample\_011046840, Unigene8081\_Sample\_011046840, Unigene34971\_Sample\_011046840, Unigene37185\_Sample\_011046840, Unigene30370\_Sample\_011046840, Unigene32811\_Sample\_011046840, Unigene12614\_Sample\_011046840, Unigene1458\_Sample\_011046840, Unigene17494\_Sample\_011046840, Unigene21883\_Sample\_011046840, Unigene37375\_Sample\_011046840, Unigene8178\_Sample\_011046840, Unigene36909\_Sample\_011046840, Unigene1132\_Sample\_011046840, Unigene21176\_Sample\_011046840, Unigene25680\_Sample\_011046840, Unigene38973\_Sample\_011046840, Unigene7529\_Sample\_011046840, Unigene22101\_Sample\_011046840, Unigene40181\_Sample\_011046840, Unigene39544\_Sample\_011046840, Unigene3849\_Sample\_011046840, Unigene38463\_Sample\_011046840, Unigene177\_Sample\_011046840, Unigene41833\_Sample\_011046840, Unigene4089\_Sample\_011046840, Unigene14294\_Sample\_011046840, Unigene30285\_Sample\_011046840, Unigene36764\_Sample\_011046840, Unigene37848\_Sample\_011046840, Unigene14393\_Sample\_011046840, Unigene5726\_Sample\_011046840, Unigene38504\_Sample\_011046840, Unigene36848\_Sample\_011046840, Unigene19725\_Sample\_011046840, Unigene37095\_Sample\_011046840, Unigene30893\_Sample\_011046840, Unigene3319\_Sample\_011046840, Unigene13306\_Sample\_011046840, Unigene24816\_Sample\_011046840, Unigene20757\_Sample\_011046840, Unigene24103\_Sample\_011046840, Unigene33428\_Sample\_011046840, Unigene24254\_Sample\_011046840, Unigene7831\_Sample\_011046840, Unigene30203\_Sample\_011046840, Unigene893\_Sample\_011046840, Unigene43435\_Sample\_011046840, Unigene30471\_Sample\_011046840, Unigene40159\_Sample\_011046840, Unigene29784\_Sample\_011046840, Unigene39812\_Sample\_011046840, Unigene27998\_Sample\_011046840, Unigene19834\_Sample\_011046840, Unigene42253\_Sample\_011046840, Unigene29947\_Sample\_011046840, Unigene34234\_Sample\_011046840, Unigene25555\_Sample\_011046840, Unigene42502\_Sample\_011046840, Unigene41104\_Sample\_011046840, Unigene11113\_Sample\_011046840, Unigene41150\_Sample\_011046840, Unigene4830\_Sample\_011046840, Unigene41496\_Sample\_011046840, Unigene16527\_Sample\_011046840, Unigene43077\_Sample\_011046840, Unigene40073\_Sample\_011046840, Unigene37822\_Sample\_011046840, Unigene9565\_Sample\_011046840, Unigene12101\_Sample\_011046840, Unigene5249\_Sample\_011046840, Unigene31599\_Sample\_011046840, Unigene22020\_Sample\_011046840, Unigene16161\_Sample\_011046840, Unigene5118\_Sample\_011046840, Unigene35603\_Sample\_011046840, Unigene16645\_Sample\_011046840, Unigene32978\_Sample\_011046840, Unigene42351\_Sample\_011046840, Unigene33208\_Sample\_011046840, Unigene36893\_Sample\_011046840, Unigene36637\_Sample\_011046840, Unigene30615\_Sample\_011046840, Unigene18599\_Sample\_011046840, Unigene29270\_Sample\_011046840, Unigene13215\_Sample\_011046840, Unigene4578\_Sample\_011046840, Unigene15884\_Sample\_011046840, Unigene43230\_Sample\_011046840, Unigene42704\_Sample\_011046840, Unigene40406\_Sample\_011046840, Unigene37855\_Sample\_011046840, Unigene42242\_Sample\_011046840, Unigene20758\_Sample\_011046840, Unigene39911\_Sample\_011046840, Unigene36345\_Sample\_011046840, Unigene31215\_Sample\_011046840, Unigene41932\_Sample\_011046840, Unigene34105\_Sample\_011046840, Unigene20100\_Sample\_011046840, Unigene5001\_Sample\_011046840, Unigene39763\_Sample\_011046840, Unigene8801\_Sample\_011046840, Unigene37863\_Sample\_011046840, Unigene8554\_Sample\_011046840, Unigene39218\_Sample\_011046840, Unigene41866\_Sample\_011046840, Unigene39982\_Sample\_011046840, Unigene20594\_Sample\_011046840, Unigene43537\_Sample\_011046840, Unigene20\_Sample\_011046840, Unigene33968\_Sample\_011046840, Unigene30239\_Sample\_011046840, Unigene37613\_Sample\_011046840, Unigene32733\_Sample\_011046840, Unigene37902\_Sample\_011046840, Unigene3770\_Sample\_011046840, Unigene1222\_Sample\_011046840, Unigene17734\_Sample\_011046840, Unigene38066\_Sample\_011046840, Unigene38847\_Sample\_011046840, Unigene26711\_Sample\_011046840, Unigene38443\_Sample\_011046840, Unigene18659\_Sample\_011046840, Unigene32337\_Sample\_011046840, Unigene20968\_Sample\_011046840, Unigene29802\_Sample\_011046840, Unigene42061\_Sample\_011046840, Unigene33983\_Sample\_011046840, Unigene6796\_Sample\_011046840, Unigene20491\_Sample\_011046840, Unigene35734\_Sample\_011046840, Unigene4994\_Sample\_011046840, Unigene33913\_Sample\_011046840, Unigene41476\_Sample\_011046840, Unigene13127\_Sample\_011046840, Unigene41389\_Sample\_011046840, Unigene4309\_Sample\_011046840, Unigene36619\_Sample\_011046840, Unigene16211\_Sample\_011046840, Unigene38206\_Sample\_011046840, Unigene9477\_Sample\_011046840, Unigene43332\_Sample\_011046840, Unigene41743\_Sample\_011046840, Unigene29953\_Sample\_011046840, Unigene38834\_Sample\_011046840, Unigene20493\_Sample\_011046840, Unigene32099\_Sample\_011046840, Unigene41898\_Sample\_011046840, Unigene37706\_Sample\_011046840, Unigene31090\_Sample\_011046840, Unigene3003\_Sample\_011046840, Unigene42374\_Sample\_011046840, Unigene39024\_Sample\_011046840, Unigene15465\_Sample\_011046840, Unigene4389\_Sample\_011046840, Unigene41997\_Sample\_011046840, Unigene38687\_Sample\_011046840, Unigene5116\_Sample\_011046840, Unigene40471\_Sample\_011046840, Unigene42605\_Sample\_011046840, Unigene41161\_Sample\_011046840, Unigene34519\_Sample\_011046840, Unigene33595\_Sample\_011046840, Unigene31273\_Sample\_011046840, Unigene29985\_Sample\_011046840, Unigene907\_Sample\_011046840, Unigene33768\_Sample\_011046840, Unigene10236\_Sample\_011046840, Unigene522\_Sample\_011046840, Unigene36897\_Sample\_011046840, Unigene29903\_Sample\_011046840, Unigene29839\_Sample\_011046840, Unigene2457\_Sample\_011046840, Unigene26813\_Sample\_011046840, Unigene6547\_Sample\_011046840, Unigene26216\_Sample\_011046840, Unigene34870\_Sample\_011046840, Unigene35061\_Sample\_011046840, Unigene279\_Sample\_011046840, Unigene42759\_Sample\_011046840, Unigene2459\_Sample\_011046840, Unigene36366\_Sample\_011046840, Unigene19628\_Sample\_011046840, Unigene38962\_Sample\_011046840, Unigene34002\_Sample\_011046840, Unigene40216\_Sample\_011046840, Unigene41431\_Sample\_011046840, Unigene42859\_Sample\_011046840, Unigene30600\_Sample\_011046840, Unigene29629\_Sample\_011046840, Unigene43055\_Sample\_011046840, Unigene23556\_Sample\_011046840, Unigene15492\_Sample\_011046840, Unigene5087\_Sample\_011046840, Unigene20079\_Sample\_011046840, Unigene32197\_Sample\_011046840, Unigene6313\_Sample\_011046840, Unigene31572\_Sample\_011046840, Unigene27920\_Sample\_011046840, Unigene9002\_Sample\_011046840, Unigene39499\_Sample\_011046840, Unigene14420\_Sample\_011046840, Unigene39061\_Sample\_011046840, Unigene27158\_Sample\_011046840, Unigene24281\_Sample\_011046840, Unigene38441\_Sample\_011046840, Unigene10533\_Sample\_011046840, Unigene37904\_Sample\_011046840, Unigene43606\_Sample\_011046840, Unigene34854\_Sample\_011046840, Unigene37976\_Sample\_011046840, Unigene42547\_Sample\_011046840, Unigene10832\_Sample\_011046840, Unigene15502\_Sample\_011046840, Unigene2792\_Sample\_011046840, Unigene37545\_Sample\_011046840, Unigene41919\_Sample\_011046840, Unigene4779\_Sample\_011046840, Unigene10749\_Sample\_011046840, Unigene25548\_Sample\_011046840, Unigene14498\_Sample\_011046840, Unigene39972\_Sample\_011046840, Unigene41368\_Sample\_011046840, Unigene5372\_Sample\_011046840, Unigene38709\_Sample\_011046840, Unigene38720\_Sample\_011046840, Unigene31864\_Sample\_011046840, Unigene37214\_Sample\_011046840, Unigene43188\_Sample\_011046840, Unigene39660\_Sample\_011046840, Unigene36990\_Sample\_011046840, Unigene4344\_Sample\_011046840, Unigene39243\_Sample\_011046840, Unigene33012\_Sample\_011046840, Unigene37292\_Sample\_011046840, Unigene9682\_Sample\_011046840, Unigene43312\_Sample\_011046840, Unigene32292\_Sample\_011046840, Unigene35790\_Sample\_011046840, Unigene32209\_Sample\_011046840, Unigene16376\_Sample\_011046840, Unigene17229\_Sample\_011046840, Unigene30473\_Sample\_011046840, Unigene39165\_Sample\_011046840, Unigene15033\_Sample\_011046840, Unigene43000\_Sample\_011046840, Unigene30860\_Sample\_011046840, Unigene26740\_Sample\_011046840, Unigene16473\_Sample\_011046840, Unigene3551\_Sample\_011046840, Unigene1008\_Sample\_011046840, Unigene5570\_Sample\_011046840, Unigene40679\_Sample\_011046840, Unigene37870\_Sample\_011046840, Unigene26427\_Sample\_011046840, Unigene42499\_Sample\_011046840, Unigene16770\_Sample\_011046840, Unigene10730\_Sample\_011046840, Unigene31855\_Sample\_011046840, Unigene29526\_Sample\_011046840, Unigene33605\_Sample\_011046840, Unigene33711\_Sample\_011046840, Unigene43632\_Sample\_011046840, Unigene34669\_Sample\_011046840, Unigene35906\_Sample\_011046840, Unigene898\_Sample\_011046840, Unigene43635\_Sample\_011046840, Unigene34301\_Sample\_011046840, Unigene35346\_Sample\_011046840, Unigene5439\_Sample\_011046840, Unigene38337\_Sample\_011046840, Unigene32844\_Sample\_011046840, Unigene1375\_Sample\_011046840, Unigene34436\_Sample\_011046840, Unigene1731\_Sample\_011046840, Unigene16611\_Sample\_011046840, Unigene8083\_Sample\_011046840, Unigene10704\_Sample\_011046840, Unigene26270\_Sample\_011046840, Unigene7744\_Sample\_011046840, Unigene43117\_Sample\_011046840, Unigene25644\_Sample\_011046840, Unigene27239\_Sample\_011046840, Unigene7065\_Sample\_011046840, Unigene15181\_Sample\_011046840, Unigene34833\_Sample\_011046840, Unigene7175\_Sample\_011046840, Unigene7395\_Sample\_011046840, Unigene23201\_Sample\_011046840, Unigene8227\_Sample\_011046840, Unigene22068\_Sample\_011046840, Unigene7410\_Sample\_011046840, Unigene39018\_Sample\_011046840, Unigene14074\_Sample\_011046840, Unigene42615\_Sample\_011046840, Unigene36773\_Sample\_011046840, Unigene17905\_Sample\_011046840, Unigene8006\_Sample\_011046840, Unigene2797\_Sample\_011046840, Unigene40658\_Sample\_011046840, Unigene25347\_Sample\_011046840, Unigene40979\_Sample\_011046840, Unigene35547\_Sample\_011046840, Unigene20556\_Sample\_011046840, Unigene22514\_Sample\_011046840, Unigene30760\_Sample\_011046840, Unigene40427\_Sample\_011046840, Unigene2810\_Sample\_011046840, Unigene33019\_Sample\_011046840, Unigene40032\_Sample\_011046840, Unigene33870\_Sample\_011046840, Unigene37571\_Sample\_011046840, Unigene36877\_Sample\_011046840, Unigene15851\_Sample\_011046840, Unigene4839\_Sample\_011046840, Unigene39740\_Sample\_011046840, Unigene42384\_Sample\_011046840, Unigene27359\_Sample\_011046840, Unigene23158\_Sample\_011046840, Unigene42441\_Sample\_011046840, Unigene35031\_Sample\_011046840, Unigene20101\_Sample\_011046840, Unigene42659\_Sample\_011046840, Unigene37034\_Sample\_011046840, Unigene38074\_Sample\_011046840, Unigene40484\_Sample\_011046840, Unigene18009\_Sample\_011046840, Unigene36141\_Sample\_011046840, Unigene42234\_Sample\_011046840, Unigene23241\_Sample\_011046840, Unigene10778\_Sample\_011046840, Unigene39927\_Sample\_011046840, Unigene5564\_Sample\_011046840, Unigene36685\_Sample\_011046840, Unigene41585\_Sample\_011046840, Unigene15148\_Sample\_011046840, Unigene4032\_Sample\_011046840, Unigene33943\_Sample\_011046840, Unigene43082\_Sample\_011046840, Unigene36922\_Sample\_011046840, Unigene7796\_Sample\_011046840, Unigene5007\_Sample\_011046840, Unigene15874\_Sample\_011046840, Unigene34588\_Sample\_011046840, Unigene8203\_Sample\_011046840, Unigene38501\_Sample\_011046840, Unigene40793\_Sample\_011046840, Unigene22867\_Sample\_011046840, Unigene42340\_Sample\_011046840, Unigene41400\_Sample\_011046840, Unigene35494\_Sample\_011046840, Unigene12635\_Sample\_011046840, Unigene36375\_Sample\_011046840, Unigene24034\_Sample\_011046840, Unigene24711\_Sample\_011046840, Unigene2227\_Sample\_011046840, Unigene42189\_Sample\_011046840, Unigene24309\_Sample\_011046840, Unigene41899\_Sample\_011046840, Unigene33906\_Sample\_011046840, Unigene28095\_Sample\_011046840, Unigene36121\_Sample\_011046840, Unigene8240\_Sample\_011046840, Unigene27829\_Sample\_011046840, Unigene40541\_Sample\_011046840, Unigene18069\_Sample\_011046840, Unigene41399\_Sample\_011046840, Unigene12936\_Sample\_011046840, Unigene29046\_Sample\_011046840, Unigene40917\_Sample\_011046840, Unigene42075\_Sample\_011046840, Unigene38028\_Sample\_011046840, Unigene11264\_Sample\_011046840, Unigene41676\_Sample\_011046840, Unigene35179\_Sample\_011046840, Unigene17789\_Sample\_011046840, Unigene40221\_Sample\_011046840, Unigene12818\_Sample\_011046840, Unigene7262\_Sample\_011046840, Unigene42206\_Sample\_011046840, Unigene16016\_Sample\_011046840, Unigene12035\_Sample\_011046840, Unigene2808\_Sample\_011046840, Unigene40596\_Sample\_011046840, Unigene1771\_Sample\_011046840, Unigene972\_Sample\_011046840, Unigene8555\_Sample\_011046840, Unigene7148\_Sample\_011046840, Unigene2084\_Sample\_011046840, Unigene39583\_Sample\_011046840, Unigene38393\_Sample\_011046840, Unigene35532\_Sample\_011046840, Unigene32382\_Sample\_011046840, Unigene2815\_Sample\_011046840, Unigene7669\_Sample\_011046840, Unigene3631\_Sample\_011046840, Unigene31663\_Sample\_011046840, Unigene24311\_Sample\_011046840, Unigene14178\_Sample\_011046840, Unigene17776\_Sample\_011046840, Unigene29611\_Sample\_011046840, Unigene41927\_Sample\_011046840, Unigene29621\_Sample\_011046840, Unigene27942\_Sample\_011046840, Unigene41127\_Sample\_011046840, Unigene13984\_Sample\_011046840, Unigene34362\_Sample\_011046840, Unigene32819\_Sample\_011046840, Unigene30713\_Sample\_011046840, Unigene5552\_Sample\_011046840, Unigene15637\_Sample\_011046840, Unigene34780\_Sample\_011046840, Unigene7506\_Sample\_011046840, Unigene36827\_Sample\_011046840, Unigene19404\_Sample\_011046840, Unigene24938\_Sample\_011046840, Unigene31120\_Sample\_011046840, Unigene43143\_Sample\_011046840, Unigene9887\_Sample\_011046840, Unigene36413\_Sample\_011046840, Unigene11592\_Sample\_011046840, Unigene35789\_Sample\_011046840, Unigene15030\_Sample\_011046840, Unigene40822\_Sample\_011046840, Unigene42964\_Sample\_011046840, Unigene24453\_Sample\_011046840, Unigene42663\_Sample\_011046840, Unigene5963\_Sample\_011046840, Unigene13602\_Sample\_011046840, Unigene43397\_Sample\_011046840, Unigene19793\_Sample\_011046840, Unigene32158\_Sample\_011046840, Unigene42596\_Sample\_011046840, Unigene38864\_Sample\_011046840, Unigene20577\_Sample\_011046840, Unigene14302\_Sample\_011046840, Unigene31135\_Sample\_011046840, Unigene25783\_Sample\_011046840, Unigene25531\_Sample\_011046840, Unigene7067\_Sample\_011046840, Unigene43623\_Sample\_011046840, Unigene7349\_Sample\_011046840, Unigene3226\_Sample\_011046840, Unigene17721\_Sample\_011046840, Unigene31168\_Sample\_011046840, Unigene34071\_Sample\_011046840, Unigene41691\_Sample\_011046840, Unigene26581\_Sample\_011046840, Unigene28708\_Sample\_011046840, Unigene42865\_Sample\_011046840, Unigene6064\_Sample\_011046840, Unigene16389\_Sample\_011046840, Unigene3257\_Sample\_011046840, Unigene29534\_Sample\_011046840, Unigene22159\_Sample\_011046840, Unigene29452\_Sample\_011046840, Unigene32280\_Sample\_011046840, Unigene10176\_Sample\_011046840, Unigene33275\_Sample\_011046840, Unigene31074\_Sample\_011046840, Unigene15062\_Sample\_011046840, Unigene17700\_Sample\_011046840, Unigene36699\_Sample\_011046840, Unigene28415\_Sample\_011046840, Unigene33858\_Sample\_011046840, Unigene38658\_Sample\_011046840, Unigene23820\_Sample\_011046840, Unigene7402\_Sample\_011046840, Unigene5375\_Sample\_011046840, Unigene10829\_Sample\_011046840, Unigene11629\_Sample\_011046840, Unigene43387\_Sample\_011046840, Unigene35909\_Sample\_011046840, Unigene26502\_Sample\_011046840, Unigene36268\_Sample\_011046840, Unigene29818\_Sample\_011046840, Unigene31458\_Sample\_011046840, Unigene30136\_Sample\_011046840, Unigene24201\_Sample\_011046840, Unigene8372\_Sample\_011046840, Unigene40709\_Sample\_011046840, Unigene35699\_Sample\_011046840, Unigene27865\_Sample\_011046840, Unigene40966\_Sample\_011046840, Unigene489\_Sample\_011046840, Unigene10957\_Sample\_011046840, Unigene4518\_Sample\_011046840, Unigene33342\_Sample\_011046840, Unigene26057\_Sample\_011046840, Unigene34506\_Sample\_011046840, Unigene20052\_Sample\_011046840, Unigene42097\_Sample\_011046840, Unigene24244\_Sample\_011046840, Unigene41398\_Sample\_011046840, Unigene7025\_Sample\_011046840, Unigene22635\_Sample\_011046840, Unigene27993\_Sample\_011046840, Unigene39020\_Sample\_011046840, Unigene40262\_Sample\_011046840, Unigene9922\_Sample\_011046840, Unigene24917\_Sample\_011046840, Unigene32947\_Sample\_011046840, Unigene32360\_Sample\_011046840, Unigene29103\_Sample\_011046840, Unigene39324\_Sample\_011046840, Unigene32594\_Sample\_011046840, Unigene22752\_Sample\_011046840, Unigene40551\_Sample\_011046840, Unigene41178\_Sample\_011046840, Unigene43627\_Sample\_011046840, Unigene33112\_Sample\_011046840, Unigene37999\_Sample\_011046840, Unigene6285\_Sample\_011046840, Unigene28867\_Sample\_011046840, Unigene33052\_Sample\_011046840, Unigene33999\_Sample\_011046840, Unigene31448\_Sample\_011046840, Unigene16829\_Sample\_011046840, Unigene29049\_Sample\_011046840, Unigene32831\_Sample\_011046840, Unigene26512\_Sample\_011046840, Unigene40783\_Sample\_011046840, Unigene42138\_Sample\_011046840, Unigene29397\_Sample\_011046840, Unigene37985\_Sample\_011046840, Unigene14485\_Sample\_011046840, Unigene36500\_Sample\_011046840, Unigene19235\_Sample\_011046840, Unigene20304\_Sample\_011046840, Unigene42585\_Sample\_011046840, Unigene29567\_Sample\_011046840, Unigene34472\_Sample\_011046840, Unigene15594\_Sample\_011046840, Unigene33393\_Sample\_011046840, Unigene31108\_Sample\_011046840, Unigene16321\_Sample\_011046840, Unigene8615\_Sample\_011046840, Unigene33196\_Sample\_011046840, Unigene19400\_Sample\_011046840, Unigene4619\_Sample\_011046840, Unigene24373\_Sample\_011046840, Unigene19647\_Sample\_011046840, Unigene34843\_Sample\_011046840, Unigene10033\_Sample\_011046840, Unigene40222\_Sample\_011046840, Unigene38165\_Sample\_011046840, Unigene33473\_Sample\_011046840, Unigene42190\_Sample\_011046840, Unigene41068\_Sample\_011046840, Unigene30539\_Sample\_011046840, Unigene39171\_Sample\_011046840, Unigene37353\_Sample\_011046840, Unigene42460\_Sample\_011046840, Unigene26767\_Sample\_011046840, Unigene16855\_Sample\_011046840, Unigene38564\_Sample\_011046840, Unigene25769\_Sample\_011046840, Unigene19809\_Sample\_011046840, Unigene39825\_Sample\_011046840, Unigene40368\_Sample\_011046840, Unigene6561\_Sample\_011046840, Unigene22844\_Sample\_011046840, Unigene10568\_Sample\_011046840, Unigene43614\_Sample\_011046840, Unigene41718\_Sample\_011046840, Unigene38534\_Sample\_011046840, Unigene36032\_Sample\_011046840, Unigene535\_Sample\_011046840, Unigene37644\_Sample\_011046840, Unigene32515\_Sample\_011046840, Unigene39951\_Sample\_011046840, Unigene38730\_Sample\_011046840, Unigene43450\_Sample\_011046840, Unigene40192\_Sample\_011046840, Unigene39935\_Sample\_011046840, Unigene473\_Sample\_011046840, Unigene39427\_Sample\_011046840, Unigene25001\_Sample\_011046840, Unigene20367\_Sample\_011046840, Unigene31408\_Sample\_011046840, Unigene8211\_Sample\_011046840, Unigene4307\_Sample\_011046840, Unigene39007\_Sample\_011046840, Unigene39015\_Sample\_011046840, Unigene37487\_Sample\_011046840, Unigene38616\_Sample\_011046840, Unigene43281\_Sample\_011046840, Unigene815\_Sample\_011046840, Unigene13836\_Sample\_011046840, Unigene26820\_Sample\_011046840, Unigene42164\_Sample\_011046840, Unigene6530\_Sample\_011046840, Unigene412\_Sample\_011046840, Unigene38370\_Sample\_011046840, Unigene20078\_Sample\_011046840, Unigene38792\_Sample\_011046840, Unigene30813\_Sample\_011046840, Unigene43078\_Sample\_011046840, Unigene22254\_Sample\_011046840, Unigene34112\_Sample\_011046840, Unigene40971\_Sample\_011046840, Unigene43019\_Sample\_011046840, Unigene27313\_Sample\_011046840, Unigene17135\_Sample\_011046840, Unigene43311\_Sample\_011046840, Unigene43216\_Sample\_011046840, Unigene24243\_Sample\_011046840, Unigene34992\_Sample\_011046840, Unigene6409\_Sample\_011046840, Unigene22630\_Sample\_011046840, Unigene42997\_Sample\_011046840, Unigene42121\_Sample\_011046840, Unigene27337\_Sample\_011046840, Unigene9880\_Sample\_011046840, Unigene35787\_Sample\_011046840, Unigene34351\_Sample\_011046840, Unigene11464\_Sample\_011046840, Unigene39486\_Sample\_011046840, Unigene35792\_Sample\_011046840, Unigene6378\_Sample\_011046840, Unigene7938\_Sample\_011046840, Unigene33580\_Sample\_011046840, Unigene6293\_Sample\_011046840, Unigene28473\_Sample\_011046840, Unigene27358\_Sample\_011046840, Unigene37793\_Sample\_011046840, Unigene15491\_Sample\_011046840, Unigene32568\_Sample\_011046840, Unigene41588\_Sample\_011046840, Unigene28437\_Sample\_011046840, Unigene43176\_Sample\_011046840, Unigene36475\_Sample\_011046840, Unigene14116\_Sample\_011046840, Unigene37359\_Sample\_011046840, Unigene38852\_Sample\_011046840, Unigene13391\_Sample\_011046840, Unigene39348\_Sample\_011046840, Unigene11853\_Sample\_011046840, Unigene39080\_Sample\_011046840, Unigene38047\_Sample\_011046840, Unigene40881\_Sample\_011046840, Unigene12767\_Sample\_011046840, Unigene35476\_Sample\_011046840, Unigene29006\_Sample\_011046840, Unigene39136\_Sample\_011046840, Unigene15696\_Sample\_011046840, Unigene30322\_Sample\_011046840, Unigene5815\_Sample\_011046840, Unigene29131\_Sample\_011046840, Unigene39668\_Sample\_011046840, Unigene40903\_Sample\_011046840, Unigene42227\_Sample\_011046840, Unigene40992\_Sample\_011046840, Unigene36427\_Sample\_011046840, Unigene16130\_Sample\_011046840, Unigene34102\_Sample\_011046840, Unigene36876\_Sample\_011046840, Unigene24004\_Sample\_011046840, Unigene39404\_Sample\_011046840, Unigene26969\_Sample\_011046840, Unigene34453\_Sample\_011046840, Unigene12655\_Sample\_011046840, Unigene37877\_Sample\_011046840, Unigene27975\_Sample\_011046840, Unigene14511\_Sample\_011046840, Unigene32134\_Sample\_011046840, Unigene32749\_Sample\_011046840, Unigene26815\_Sample\_011046840, Unigene26734\_Sample\_011046840, Unigene43062\_Sample\_011046840, Unigene29301\_Sample\_011046840, Unigene42383\_Sample\_011046840, Unigene28404\_Sample\_011046840, Unigene16269\_Sample\_011046840, Unigene40565\_Sample\_011046840, Unigene39381\_Sample\_011046840, Unigene2174\_Sample\_011046840, Unigene32865\_Sample\_011046840, Unigene27180\_Sample\_011046840, Unigene688\_Sample\_011046840, Unigene36400\_Sample\_011046840, Unigene43084\_Sample\_011046840, Unigene3156\_Sample\_011046840, Unigene35434\_Sample\_011046840, Unigene6180\_Sample\_011046840, Unigene33938\_Sample\_011046840, Unigene40865\_Sample\_011046840, Unigene39866\_Sample\_011046840, Unigene31310\_Sample\_011046840, Unigene23252\_Sample\_011046840, Unigene42296\_Sample\_011046840, Unigene22122\_Sample\_011046840, Unigene37628\_Sample\_011046840, Unigene11949\_Sample\_011046840, Unigene34464\_Sample\_011046840, Unigene36926\_Sample\_011046840, Unigene42789\_Sample\_011046840, Unigene13828\_Sample\_011046840, Unigene1963\_Sample\_011046840, Unigene39156\_Sample\_011046840, Unigene11179\_Sample\_011046840, Unigene40994\_Sample\_011046840, Unigene1256\_Sample\_011046840, Unigene6241\_Sample\_011046840, Unigene37705\_Sample\_011046840, Unigene16976\_Sample\_011046840, Unigene25184\_Sample\_011046840, Unigene31945\_Sample\_011046840, Unigene23357\_Sample\_011046840, Unigene23281\_Sample\_011046840, Unigene37392\_Sample\_011046840, Unigene39992\_Sample\_011046840, Unigene8218\_Sample\_011046840, Unigene10696\_Sample\_011046840, Unigene36706\_Sample\_011046840, Unigene43477\_Sample\_011046840, Unigene30048\_Sample\_011046840, Unigene41792\_Sample\_011046840, Unigene4723\_Sample\_011046840, Unigene30691\_Sample\_011046840, Unigene40837\_Sample\_011046840, Unigene14017\_Sample\_011046840, Unigene36112\_Sample\_011046840, Unigene20250\_Sample\_011046840, Unigene7503\_Sample\_011046840, Unigene30919\_Sample\_011046840, Unigene14611\_Sample\_011046840, Unigene17216\_Sample\_011046840, Unigene40424\_Sample\_011046840, Unigene3599\_Sample\_011046840, Unigene23324\_Sample\_011046840, Unigene29427\_Sample\_011046840, Unigene17375\_Sample\_011046840, Unigene36567\_Sample\_011046840, Unigene15303\_Sample\_011046840, Unigene16035\_Sample\_011046840, Unigene27997\_Sample\_011046840, Unigene42864\_Sample\_011046840, Unigene36920\_Sample\_011046840, Unigene13276\_Sample\_011046840, Unigene39180\_Sample\_011046840, Unigene43536\_Sample\_011046840, Unigene34655\_Sample\_011046840, Unigene40818\_Sample\_011046840, Unigene38377\_Sample\_011046840, Unigene18440\_Sample\_011046840, Unigene28736\_Sample\_011046840, Unigene37830\_Sample\_011046840, Unigene33866\_Sample\_011046840, Unigene43607\_Sample\_011046840, Unigene21072\_Sample\_011046840, Unigene12713\_Sample\_011046840, Unigene22459\_Sample\_011046840, Unigene33926\_Sample\_011046840, Unigene37219\_Sample\_011046840, Unigene38559\_Sample\_011046840, Unigene11613\_Sample\_011046840, Unigene38360\_Sample\_011046840, Unigene38044\_Sample\_011046840, Unigene42007\_Sample\_011046840, Unigene30105\_Sample\_011046840, Unigene41174\_Sample\_011046840, Unigene15831\_Sample\_011046840, Unigene41740\_Sample\_011046840, Unigene432\_Sample\_011046840, Unigene17416\_Sample\_011046840, Unigene40346\_Sample\_011046840, Unigene35883\_Sample\_011046840, Unigene31610\_Sample\_011046840, Unigene37041\_Sample\_011046840, Unigene30572\_Sample\_011046840, Unigene23630\_Sample\_011046840, Unigene33760\_Sample\_011046840, Unigene38146\_Sample\_011046840, Unigene24246\_Sample\_011046840, Unigene31584\_Sample\_011046840, Unigene36376\_Sample\_011046840, Unigene30798\_Sample\_011046840, Unigene39838\_Sample\_011046840, Unigene3842\_Sample\_011046840, Unigene12743\_Sample\_011046840, Unigene31773\_Sample\_011046840, Unigene3928\_Sample\_011046840, Unigene41001\_Sample\_011046840, Unigene23856\_Sample\_011046840, Unigene43115\_Sample\_011046840, Unigene22258\_Sample\_011046840, Unigene17210\_Sample\_011046840, Unigene41498\_Sample\_011046840, Unigene31398\_Sample\_011046840, Unigene10770\_Sample\_011046840, Unigene42171\_Sample\_011046840, Unigene24889\_Sample\_011046840, Unigene9871\_Sample\_011046840, Unigene38932\_Sample\_011046840, Unigene33443\_Sample\_011046840, Unigene38152\_Sample\_011046840, Unigene14629\_Sample\_011046840, Unigene14756\_Sample\_011046840, Unigene42050\_Sample\_011046840, Unigene42835\_Sample\_011046840, Unigene5197\_Sample\_011046840, Unigene29880\_Sample\_011046840, Unigene7717\_Sample\_011046840, Unigene5121\_Sample\_011046840, Unigene43147\_Sample\_011046840, Unigene38039\_Sample\_011046840, Unigene28861\_Sample\_011046840, Unigene42602\_Sample\_011046840, Unigene10135\_Sample\_011046840, Unigene22688\_Sample\_011046840, Unigene10802\_Sample\_011046840, Unigene40391\_Sample\_011046840, Unigene38670\_Sample\_011046840, Unigene29016\_Sample\_011046840, Unigene37540\_Sample\_011046840, Unigene21026\_Sample\_011046840, Unigene36690\_Sample\_011046840, Unigene39732\_Sample\_011046840, Unigene1959\_Sample\_011046840, Unigene18735\_Sample\_011046840, Unigene32368\_Sample\_011046840, Unigene19481\_Sample\_011046840, Unigene43025\_Sample\_011046840, Unigene41288\_Sample\_011046840, Unigene30094\_Sample\_011046840, Unigene25579\_Sample\_011046840, Unigene34053\_Sample\_011046840, Unigene28414\_Sample\_011046840, Unigene12587\_Sample\_011046840, Unigene39882\_Sample\_011046840, Unigene35872\_Sample\_011046840, Unigene10288\_Sample\_011046840, Unigene9718\_Sample\_011046840, Unigene39555\_Sample\_011046840, Unigene35927\_Sample\_011046840, Unigene5480\_Sample\_011046840, Unigene14666\_Sample\_011046840, Unigene36535\_Sample\_011046840, Unigene12679\_Sample\_011046840, Unigene40687\_Sample\_011046840, Unigene39837\_Sample\_011046840, Unigene23176\_Sample\_011046840, Unigene24594\_Sample\_011046840, Unigene41858\_Sample\_011046840, Unigene42683\_Sample\_011046840, Unigene14292\_Sample\_011046840, Unigene27724\_Sample\_011046840, Unigene19383\_Sample\_011046840, Unigene10601\_Sample\_011046840, Unigene33259\_Sample\_011046840, Unigene30862\_Sample\_011046840, Unigene41336\_Sample\_011046840, Unigene25871\_Sample\_011046840, Unigene33322\_Sample\_011046840, Unigene30573\_Sample\_011046840, Unigene38115\_Sample\_011046840, Unigene34746\_Sample\_011046840, Unigene36462\_Sample\_011046840, Unigene40812\_Sample\_011046840, Unigene40787\_Sample\_011046840, Unigene37745\_Sample\_011046840, Unigene24674\_Sample\_011046840, Unigene40184\_Sample\_011046840, Unigene28636\_Sample\_011046840, Unigene33889\_Sample\_011046840, Unigene28901\_Sample\_011046840, Unigene43453\_Sample\_011046840, Unigene39490\_Sample\_011046840, Unigene39632\_Sample\_011046840, Unigene11701\_Sample\_011046840, Unigene31638\_Sample\_011046840, Unigene11125\_Sample\_011046840, Unigene42030\_Sample\_011046840, Unigene28758\_Sample\_011046840, Unigene25431\_Sample\_011046840, Unigene4320\_Sample\_011046840, Unigene20420\_Sample\_011046840, Unigene28316\_Sample\_011046840, Unigene6563\_Sample\_011046840, Unigene36951\_Sample\_011046840, Unigene43031\_Sample\_011046840, Unigene12197\_Sample\_011046840, Unigene36090\_Sample\_011046840, Unigene22961\_Sample\_011046840, Unigene43363\_Sample\_011046840, Unigene22232\_Sample\_011046840, Unigene38021\_Sample\_011046840, Unigene18262\_Sample\_011046840, Unigene41561\_Sample\_011046840, Unigene26967\_Sample\_011046840, Unigene12868\_Sample\_011046840, Unigene37710\_Sample\_011046840, Unigene41901\_Sample\_011046840, Unigene4043\_Sample\_011046840, Unigene25934\_Sample\_011046840, Unigene42079\_Sample\_011046840, Unigene165\_Sample\_011046840, Unigene43611\_Sample\_011046840, Unigene7072\_Sample\_011046840, Unigene3161\_Sample\_011046840, Unigene40387\_Sample\_011046840, Unigene21295\_Sample\_011046840, Unigene42944\_Sample\_011046840, Unigene5047\_Sample\_011046840, Unigene1859\_Sample\_011046840, Unigene4721\_Sample\_011046840, Unigene35151\_Sample\_011046840, Unigene35324\_Sample\_011046840, Unigene29879\_Sample\_011046840, Unigene25629\_Sample\_011046840, Unigene29966\_Sample\_011046840, Unigene41467\_Sample\_011046840, Unigene16882\_Sample\_011046840, Unigene42154\_Sample\_011046840, Unigene38267\_Sample\_011046840, Unigene42047\_Sample\_011046840, Unigene25720\_Sample\_011046840, Unigene36496\_Sample\_011046840, Unigene14078\_Sample\_011046840, Unigene13016\_Sample\_011046840, Unigene39103\_Sample\_011046840, Unigene24184\_Sample\_011046840, Unigene3537\_Sample\_011046840, Unigene38522\_Sample\_011046840, Unigene6787\_Sample\_011046840, Unigene5393\_Sample\_011046840, Unigene37573\_Sample\_011046840, Unigene25466\_Sample\_011046840, Unigene31793\_Sample\_011046840, Unigene12386\_Sample\_011046840, Unigene29845\_Sample\_011046840, Unigene35118\_Sample\_011046840, Unigene42837\_Sample\_011046840, Unigene42475\_Sample\_011046840, Unigene42531\_Sample\_011046840, Unigene42204\_Sample\_011046840, Unigene2615\_Sample\_011046840, Unigene35939\_Sample\_011046840, Unigene34233\_Sample\_011046840, Unigene39958\_Sample\_011046840, Unigene23541\_Sample\_011046840, Unigene18141\_Sample\_011046840, Unigene42252\_Sample\_011046840, Unigene12084\_Sample\_011046840, Unigene40601\_Sample\_011046840, Unigene32188\_Sample\_011046840, Unigene39418\_Sample\_011046840, Unigene42200\_Sample\_011046840, Unigene21974\_Sample\_011046840, Unigene40208\_Sample\_011046840, Unigene37077\_Sample\_011046840, Unigene20828\_Sample\_011046840, Unigene30505\_Sample\_011046840, Unigene14237\_Sample\_011046840, Unigene42786\_Sample\_011046840, Unigene3395\_Sample\_011046840, Unigene35395\_Sample\_011046840, Unigene3814\_Sample\_011046840, Unigene42886\_Sample\_011046840, Unigene38215\_Sample\_011046840, Unigene33515\_Sample\_011046840, Unigene38121\_Sample\_011046840, Unigene41880\_Sample\_011046840, Unigene19448\_Sample\_011046840, Unigene3164\_Sample\_011046840, Unigene42133\_Sample\_011046840, Unigene30530\_Sample\_011046840, Unigene20634\_Sample\_011046840, Unigene7794\_Sample\_011046840, Unigene31\_Sample\_011046840, Unigene23873\_Sample\_011046840, Unigene33979\_Sample\_011046840, Unigene27545\_Sample\_011046840, Unigene31822\_Sample\_011046840, Unigene41582\_Sample\_011046840, Unigene38462\_Sample\_011046840, Unigene34355\_Sample\_011046840, Unigene39505\_Sample\_011046840, Unigene8064\_Sample\_011046840, Unigene7983\_Sample\_011046840, Unigene37702\_Sample\_011046840, Unigene29411\_Sample\_011046840, Unigene43525\_Sample\_011046840, Unigene5594\_Sample\_011046840, Unigene35068\_Sample\_011046840, Unigene30197\_Sample\_011046840, Unigene18134\_Sample\_011046840, Unigene35433\_Sample\_011046840, Unigene35554\_Sample\_011046840, Unigene25328\_Sample\_011046840, Unigene39870\_Sample\_011046840, Unigene9730\_Sample\_011046840, Unigene22550\_Sample\_011046840, Unigene24428\_Sample\_011046840, Unigene40465\_Sample\_011046840, Unigene25313\_Sample\_011046840, Unigene28259\_Sample\_011046840, Unigene37149\_Sample\_011046840, Unigene34500\_Sample\_011046840, Unigene29711\_Sample\_011046840, Unigene40223\_Sample\_011046840, Unigene6498\_Sample\_011046840, Unigene32116\_Sample\_011046840, Unigene20023\_Sample\_011046840, Unigene31318\_Sample\_011046840, Unigene39179\_Sample\_011046840, Unigene35234\_Sample\_011046840, Unigene3934\_Sample\_011046840, Unigene43422\_Sample\_011046840, Unigene14154\_Sample\_011046840, Unigene2408\_Sample\_011046840, Unigene42127\_Sample\_011046840, Unigene24187\_Sample\_011046840, Unigene23188\_Sample\_011046840, Unigene1039\_Sample\_011046840, Unigene31248\_Sample\_011046840, Unigene35633\_Sample\_011046840, Unigene29346\_Sample\_011046840, Unigene12033\_Sample\_011046840, Unigene17239\_Sample\_011046840, Unigene40016\_Sample\_011046840, Unigene35643\_Sample\_011046840, Unigene42177\_Sample\_011046840, Unigene33743\_Sample\_011046840, Unigene43636\_Sample\_011046840, Unigene15849\_Sample\_011046840, Unigene23811\_Sample\_011046840, Unigene41355\_Sample\_011046840, Unigene7643\_Sample\_011046840, Unigene41574\_Sample\_011046840, Unigene31649\_Sample\_011046840, Unigene33736\_Sample\_011046840, Unigene34555\_Sample\_011046840, Unigene10641\_Sample\_011046840, Unigene32875\_Sample\_011046840, Unigene11685\_Sample\_011046840, Unigene27895\_Sample\_011046840, Unigene4327\_Sample\_011046840, Unigene43095\_Sample\_011046840, Unigene12062\_Sample\_011046840, Unigene16063\_Sample\_011046840, Unigene30508\_Sample\_011046840, Unigene36187\_Sample\_011046840, Unigene5400\_Sample\_011046840, Unigene3339\_Sample\_011046840, Unigene36260\_Sample\_011046840, Unigene32706\_Sample\_011046840, Unigene37852\_Sample\_011046840, Unigene31684\_Sample\_011046840, Unigene28063\_Sample\_011046840, Unigene16650\_Sample\_011046840, Unigene39275\_Sample\_011046840, Unigene12701\_Sample\_011046840, Unigene14279\_Sample\_011046840, Unigene32354\_Sample\_011046840, Unigene30059\_Sample\_011046840, Unigene10035\_Sample\_011046840, Unigene8904\_Sample\_011046840, Unigene36088\_Sample\_011046840, Unigene41771\_Sample\_011046840, Unigene41481\_Sample\_011046840, Unigene42569\_Sample\_011046840, Unigene18212\_Sample\_011046840, Unigene9721\_Sample\_011046840, Unigene8113\_Sample\_011046840, Unigene42574\_Sample\_011046840, Unigene25488\_Sample\_011046840, Unigene41935\_Sample\_011046840, Unigene4815\_Sample\_011046840, Unigene34896\_Sample\_011046840, Unigene14473\_Sample\_011046840, Unigene26865\_Sample\_011046840, Unigene23169\_Sample\_011046840, Unigene39391\_Sample\_011046840, Unigene2581\_Sample\_011046840, Unigene43556\_Sample\_011046840, Unigene41980\_Sample\_011046840, Unigene42325\_Sample\_011046840, Unigene42348\_Sample\_011046840, Unigene35074\_Sample\_011046840, Unigene39497\_Sample\_011046840, Unigene2218\_Sample\_011046840, Unigene39081\_Sample\_011046840, Unigene14503\_Sample\_011046840, Unigene41972\_Sample\_011046840, Unigene7458\_Sample\_011046840, Unigene21905\_Sample\_011046840, Unigene42768\_Sample\_011046840, Unigene12341\_Sample\_011046840, Unigene41855\_Sample\_011046840, Unigene41007\_Sample\_011046840, Unigene10729\_Sample\_011046840, Unigene19018\_Sample\_011046840, Unigene10318\_Sample\_011046840, Unigene3788\_Sample\_011046840, Unigene13393\_Sample\_011046840, Unigene41324\_Sample\_011046840, Unigene40612\_Sample\_011046840, Unigene6862\_Sample\_011046840, Unigene32621\_Sample\_011046840, Unigene23759\_Sample\_011046840, Unigene36342\_Sample\_011046840, Unigene33797\_Sample\_011046840, Unigene2687\_Sample\_011046840, Unigene21944\_Sample\_011046840, Unigene2151\_Sample\_011046840, Unigene42626\_Sample\_011046840, Unigene14750\_Sample\_011046840, Unigene31441\_Sample\_011046840, Unigene35459\_Sample\_011046840, Unigene43348\_Sample\_011046840, Unigene43412\_Sample\_011046840, Unigene2152\_Sample\_011046840, Unigene39717\_Sample\_011046840, Unigene4686\_Sample\_011046840, Unigene36544\_Sample\_011046840, Unigene17677\_Sample\_011046840, Unigene41261\_Sample\_011046840, Unigene10474\_Sample\_011046840, Unigene17194\_Sample\_011046840, Unigene15889\_Sample\_011046840, Unigene27951\_Sample\_011046840, Unigene31900\_Sample\_011046840, Unigene37045\_Sample\_011046840, Unigene4497\_Sample\_011046840, Unigene12353\_Sample\_011046840, Unigene2092\_Sample\_011046840, Unigene12675\_Sample\_011046840, Unigene34828\_Sample\_011046840, Unigene24005\_Sample\_011046840, Unigene19653\_Sample\_011046840, Unigene39559\_Sample\_011046840, Unigene40562\_Sample\_011046840, Unigene40053\_Sample\_011046840, Unigene32836\_Sample\_011046840, Unigene37404\_Sample\_011046840, Unigene32314\_Sample\_011046840, Unigene20154\_Sample\_011046840, Unigene39437\_Sample\_011046840, Unigene39720\_Sample\_011046840, Unigene5378\_Sample\_011046840, Unigene208\_Sample\_011046840, Unigene43258\_Sample\_011046840, Unigene19918\_Sample\_011046840, Unigene5436\_Sample\_011046840, Unigene20526\_Sample\_011046840, Unigene26338\_Sample\_011046840, Unigene40753\_Sample\_011046840, Unigene15987\_Sample\_011046840, Unigene40278\_Sample\_011046840, Unigene3127\_Sample\_011046840, Unigene26705\_Sample\_011046840, Unigene19593\_Sample\_011046840, Unigene6740\_Sample\_011046840, Unigene37517\_Sample\_011046840, Unigene30236\_Sample\_011046840, Unigene28852\_Sample\_011046840, Unigene33998\_Sample\_011046840, Unigene31994\_Sample\_011046840, Unigene26163\_Sample\_011046840, Unigene27886\_Sample\_011046840, Unigene18907\_Sample\_011046840, Unigene26846\_Sample\_011046840, Unigene5241\_Sample\_011046840, Unigene34039\_Sample\_011046840, Unigene38608\_Sample\_011046840, Unigene2543\_Sample\_011046840, Unigene35951\_Sample\_011046840, Unigene6760\_Sample\_011046840, Unigene21415\_Sample\_011046840, Unigene41637\_Sample\_011046840, Unigene20065\_Sample\_011046840, Unigene35555\_Sample\_011046840, Unigene28002\_Sample\_011046840, Unigene30702\_Sample\_011046840, Unigene20428\_Sample\_011046840, Unigene14691\_Sample\_011046840, Unigene42798\_Sample\_011046840, Unigene23889\_Sample\_011046840, Unigene40899\_Sample\_011046840, Unigene42418\_Sample\_011046840, Unigene31192\_Sample\_011046840, Unigene21332\_Sample\_011046840, Unigene14501\_Sample\_011046840, Unigene42183\_Sample\_011046840, Unigene34524\_Sample\_011046840, Unigene30629\_Sample\_011046840, Unigene16244\_Sample\_011046840, Unigene42293\_Sample\_011046840, Unigene31880\_Sample\_011046840, Unigene32589\_Sample\_011046840, Unigene23234\_Sample\_011046840, Unigene2740\_Sample\_011046840, Unigene5453\_Sample\_011046840, Unigene21959\_Sample\_011046840, Unigene22620\_Sample\_011046840, Unigene41709\_Sample\_011046840, Unigene43416\_Sample\_011046840, Unigene34975\_Sample\_011046840, Unigene8660\_Sample\_011046840, Unigene37131\_Sample\_011046840, Unigene26023\_Sample\_011046840, Unigene16906\_Sample\_011046840, Unigene29661\_Sample\_011046840, Unigene40616\_Sample\_011046840, Unigene43186\_Sample\_011046840, Unigene29145\_Sample\_011046840, Unigene7789\_Sample\_011046840, Unigene26507\_Sample\_011046840, Unigene31407\_Sample\_011046840, Unigene42281\_Sample\_011046840, Unigene8121\_Sample\_011046840, Unigene34432\_Sample\_011046840, Unigene41419\_Sample\_011046840, Unigene43273\_Sample\_011046840, Unigene37116\_Sample\_011046840, Unigene32645\_Sample\_011046840, Unigene7513\_Sample\_011046840, Unigene26212\_Sample\_011046840, Unigene2519\_Sample\_011046840, Unigene22727\_Sample\_011046840, Unigene41160\_Sample\_011046840, Unigene42088\_Sample\_011046840, Unigene34380\_Sample\_011046840, Unigene19472\_Sample\_011046840, Unigene34400\_Sample\_011046840, Unigene39994\_Sample\_011046840, Unigene17139\_Sample\_011046840, Unigene40445\_Sample\_011046840, Unigene19704\_Sample\_011046840, Unigene42738\_Sample\_011046840, Unigene43001\_Sample\_011046840, Unigene18270\_Sample\_011046840, Unigene41119\_Sample\_011046840, Unigene356\_Sample\_011046840, Unigene2068\_Sample\_011046840, Unigene34304\_Sample\_011046840, Unigene36231\_Sample\_011046840, Unigene39253\_Sample\_011046840, Unigene4276\_Sample\_011046840, Unigene11443\_Sample\_011046840, Unigene10448\_Sample\_011046840, Unigene7089\_Sample\_011046840, Unigene14521\_Sample\_011046840, Unigene40309\_Sample\_011046840, Unigene30053\_Sample\_011046840, Unigene18953\_Sample\_011046840, Unigene41695\_Sample\_011046840, Unigene39582\_Sample\_011046840, Unigene16017\_Sample\_011046840, Unigene28188\_Sample\_011046840, Unigene42249\_Sample\_011046840, Unigene43217\_Sample\_011046840, Unigene22397\_Sample\_011046840, Unigene2237\_Sample\_011046840, Unigene42271\_Sample\_011046840, Unigene11994\_Sample\_011046840, Unigene31695\_Sample\_011046840, Unigene42116\_Sample\_011046840, Unigene11132\_Sample\_011046840, Unigene12950\_Sample\_011046840, Unigene8228\_Sample\_011046840, Unigene7856\_Sample\_011046840, Unigene42354\_Sample\_011046840, Unigene39818\_Sample\_011046840, Unigene36810\_Sample\_011046840, Unigene39399\_Sample\_011046840, Unigene43542\_Sample\_011046840, Unigene5443\_Sample\_011046840, Unigene38542\_Sample\_011046840, Unigene41337\_Sample\_011046840, Unigene27657\_Sample\_011046840, Unigene40404\_Sample\_011046840, Unigene29336\_Sample\_011046840, Unigene34874\_Sample\_011046840, Unigene35820\_Sample\_011046840, Unigene507\_Sample\_011046840, Unigene39628\_Sample\_011046840, Unigene8594\_Sample\_011046840, Unigene24129\_Sample\_011046840, Unigene6886\_Sample\_011046840, Unigene31662\_Sample\_011046840, Unigene20272\_Sample\_011046840, Unigene39204\_Sample\_011046840, Unigene28265\_Sample\_011046840, Unigene12153\_Sample\_011046840, Unigene35008\_Sample\_011046840, Unigene36013\_Sample\_011046840, Unigene9006\_Sample\_011046840, Unigene38540\_Sample\_011046840, Unigene25307\_Sample\_011046840, Unigene36128\_Sample\_011046840, Unigene38354\_Sample\_011046840, Unigene43631\_Sample\_011046840, Unigene31373\_Sample\_011046840, Unigene39649\_Sample\_011046840, Unigene7935\_Sample\_011046840, Unigene2986\_Sample\_011046840, Unigene31576\_Sample\_011046840, Unigene43486\_Sample\_011046840, Unigene27792\_Sample\_011046840, Unigene42666\_Sample\_011046840, Unigene11875\_Sample\_011046840, Unigene9715\_Sample\_011046840, Unigene7825\_Sample\_011046840, Unigene40602\_Sample\_011046840, Unigene27152\_Sample\_011046840, Unigene23601\_Sample\_011046840, Unigene15570\_Sample\_011046840, Unigene33901\_Sample\_011046840, Unigene15138\_Sample\_011046840, Unigene36025\_Sample\_011046840, Unigene19183\_Sample\_011046840, Unigene31646\_Sample\_011046840, Unigene35279\_Sample\_011046840, Unigene35252\_Sample\_011046840, Unigene29670\_Sample\_011046840, Unigene34909\_Sample\_011046840, Unigene4529\_Sample\_011046840, Unigene37829\_Sample\_011046840, Unigene41664\_Sample\_011046840, Unigene31732\_Sample\_011046840, Unigene39606\_Sample\_011046840, Unigene3153\_Sample\_011046840, Unigene42298\_Sample\_011046840, Unigene39674\_Sample\_011046840, Unigene17985\_Sample\_011046840, Unigene39633\_Sample\_011046840, Unigene42376\_Sample\_011046840, Unigene39748\_Sample\_011046840, Unigene16618\_Sample\_011046840, Unigene26435\_Sample\_011046840, Unigene41478\_Sample\_011046840, Unigene3258\_Sample\_011046840, Unigene5425\_Sample\_011046840, Unigene6039\_Sample\_011046840, Unigene40703\_Sample\_011046840, Unigene19931\_Sample\_011046840, Unigene7299\_Sample\_011046840, Unigene39226\_Sample\_011046840, Unigene43445\_Sample\_011046840, Unigene2524\_Sample\_011046840, Unigene36817\_Sample\_011046840, Unigene33539\_Sample\_011046840, Unigene12013\_Sample\_011046840, Unigene41090\_Sample\_011046840, Unigene42509\_Sample\_011046840, Unigene3219\_Sample\_011046840, Unigene42756\_Sample\_011046840, Unigene40031\_Sample\_011046840, Unigene15184\_Sample\_011046840, Unigene5791\_Sample\_011046840, Unigene40452\_Sample\_011046840, Unigene41720\_Sample\_011046840, Unigene23472\_Sample\_011046840, Unigene43516\_Sample\_011046840, Unigene40814\_Sample\_011046840, Unigene7994\_Sample\_011046840, Unigene39346\_Sample\_011046840, Unigene41163\_Sample\_011046840, Unigene2665\_Sample\_011046840, Unigene43231\_Sample\_011046840, Unigene30367\_Sample\_011046840, Unigene38801\_Sample\_011046840, Unigene13661\_Sample\_011046840, Unigene39273\_Sample\_011046840, Unigene37919\_Sample\_011046840, Unigene43210\_Sample\_011046840, Unigene31306\_Sample\_011046840, Unigene37192\_Sample\_011046840, Unigene29808\_Sample\_011046840, Unigene8217\_Sample\_011046840, Unigene40496\_Sample\_011046840, Unigene11931\_Sample\_011046840, Unigene41811\_Sample\_011046840, Unigene29362\_Sample\_011046840, Unigene13726\_Sample\_011046840, Unigene27185\_Sample\_011046840, Unigene38555\_Sample\_011046840, Unigene6925\_Sample\_011046840, Unigene38595\_Sample\_011046840, Unigene39615\_Sample\_011046840, Unigene17602\_Sample\_011046840, Unigene25467\_Sample\_011046840, Unigene32098\_Sample\_011046840, Unigene5385\_Sample\_011046840, Unigene42983\_Sample\_011046840, Unigene15428\_Sample\_011046840, Unigene40745\_Sample\_011046840, Unigene31705\_Sample\_011046840, Unigene35001\_Sample\_011046840, Unigene40260\_Sample\_011046840, Unigene41879\_Sample\_011046840, Unigene32783\_Sample\_011046840, Unigene32240\_Sample\_011046840, Unigene21551\_Sample\_011046840, Unigene5962\_Sample\_011046840, Unigene32573\_Sample\_011046840, Unigene34972\_Sample\_011046840, Unigene28616\_Sample\_011046840, Unigene33492\_Sample\_011046840, Unigene26448\_Sample\_011046840, Unigene37083\_Sample\_011046840, Unigene17486\_Sample\_011046840, Unigene43573\_Sample\_011046840, Unigene25450\_Sample\_011046840, Unigene7951\_Sample\_011046840, Unigene28129\_Sample\_011046840, Unigene7302\_Sample\_011046840, Unigene43120\_Sample\_011046840, Unigene39480\_Sample\_011046840, Unigene33745\_Sample\_011046840, Unigene3204\_Sample\_011046840, Unigene8085\_Sample\_011046840, Unigene31899\_Sample\_011046840, Unigene6101\_Sample\_011046840, Unigene19985\_Sample\_011046840, Unigene38217\_Sample\_011046840, Unigene43342\_Sample\_011046840, Unigene37484\_Sample\_011046840, Unigene19344\_Sample\_011046840, Unigene43502\_Sample\_011046840, Unigene27982\_Sample\_011046840, Unigene31409\_Sample\_011046840, Unigene33338\_Sample\_011046840, Unigene12677\_Sample\_011046840, Unigene26172\_Sample\_011046840, Unigene43410\_Sample\_011046840, Unigene28710\_Sample\_011046840, Unigene33618\_Sample\_011046840, Unigene19933\_Sample\_011046840, Unigene34693\_Sample\_011046840, Unigene34288\_Sample\_011046840, Unigene31198\_Sample\_011046840, Unigene43301\_Sample\_011046840, Unigene26124\_Sample\_011046840, Unigene38940\_Sample\_011046840, Unigene21513\_Sample\_011046840, Unigene41283\_Sample\_011046840, Unigene37828\_Sample\_011046840, Unigene36734\_Sample\_011046840, Unigene40253\_Sample\_011046840, Unigene1368\_Sample\_011046840, Unigene42041\_Sample\_011046840, Unigene41981\_Sample\_011046840, Unigene43405\_Sample\_011046840, Unigene29875\_Sample\_011046840, Unigene1872\_Sample\_011046840, Unigene4145\_Sample\_011046840, Unigene23682\_Sample\_011046840, Unigene8198\_Sample\_011046840, Unigene40895\_Sample\_011046840, Unigene28610\_Sample\_011046840, Unigene40235\_Sample\_011046840, Unigene20869\_Sample\_011046840, Unigene39791\_Sample\_011046840, Unigene33873\_Sample\_011046840, Unigene40606\_Sample\_011046840, Unigene1379\_Sample\_011046840, Unigene41957\_Sample\_011046840, Unigene41383\_Sample\_011046840, Unigene26491\_Sample\_011046840, Unigene39843\_Sample\_011046840, Unigene3046\_Sample\_011046840, Unigene2709\_Sample\_011046840, Unigene37486\_Sample\_011046840, Unigene3212\_Sample\_011046840, Unigene3718\_Sample\_011046840, Unigene7203\_Sample\_011046840, Unigene13413\_Sample\_011046840, Unigene43289\_Sample\_011046840, Unigene36568\_Sample\_011046840, Unigene22606\_Sample\_011046840, Unigene39778\_Sample\_011046840, Unigene30720\_Sample\_011046840, Unigene14693\_Sample\_011046840, Unigene11201\_Sample\_011046840, Unigene15199\_Sample\_011046840, Unigene37213\_Sample\_011046840, Unigene7606\_Sample\_011046840, Unigene7813\_Sample\_011046840, Unigene332\_Sample\_011046840, Unigene7162\_Sample\_011046840, Unigene30599\_Sample\_011046840, Unigene36645\_Sample\_011046840, Unigene23950\_Sample\_011046840, Unigene36762\_Sample\_011046840, Unigene36732\_Sample\_011046840, Unigene41089\_Sample\_011046840, Unigene28034\_Sample\_011046840, Unigene24482\_Sample\_011046840, Unigene5166\_Sample\_011046840, Unigene20196\_Sample\_011046840, Unigene14589\_Sample\_011046840, Unigene19402\_Sample\_011046840, Unigene40547\_Sample\_011046840, Unigene39900\_Sample\_011046840, Unigene42906\_Sample\_011046840, Unigene16569\_Sample\_011046840, Unigene33705\_Sample\_011046840, Unigene38589\_Sample\_011046840, Unigene36323\_Sample\_011046840, Unigene36937\_Sample\_011046840, Unigene32067\_Sample\_011046840, Unigene40875\_Sample\_011046840, Unigene6764\_Sample\_011046840, Unigene20216\_Sample\_011046840, Unigene36996\_Sample\_011046840, Unigene38250\_Sample\_011046840, Unigene33344\_Sample\_011046840, Unigene41584\_Sample\_011046840, Unigene27067\_Sample\_011046840, Unigene18456\_Sample\_011046840, Unigene37506\_Sample\_011046840, Unigene39509\_Sample\_011046840, Unigene38767\_Sample\_011046840, Unigene5223\_Sample\_011046840, Unigene41805\_Sample\_011046840, Unigene38826\_Sample\_011046840, Unigene36211\_Sample\_011046840, Unigene43372\_Sample\_011046840, Unigene40587\_Sample\_011046840, Unigene39481\_Sample\_011046840, Unigene32614\_Sample\_011046840, Unigene26668\_Sample\_011046840, Unigene37136\_Sample\_011046840, Unigene29542\_Sample\_011046840, Unigene13327\_Sample\_011046840, Unigene42278\_Sample\_011046840, Unigene14602\_Sample\_011046840, Unigene40732\_Sample\_011046840, Unigene28853\_Sample\_011046840, Unigene8413\_Sample\_011046840, Unigene17765\_Sample\_011046840, Unigene42337\_Sample\_011046840, Unigene35167\_Sample\_011046840, Unigene41299\_Sample\_011046840, Unigene6283\_Sample\_011046840, Unigene27639\_Sample\_011046840, Unigene41061\_Sample\_011046840, Unigene11146\_Sample\_011046840, Unigene30715\_Sample\_011046840, Unigene37236\_Sample\_011046840, Unigene32566\_Sample\_011046840, Unigene33878\_Sample\_011046840, Unigene34566\_Sample\_011046840, Unigene26402\_Sample\_011046840, Unigene43585\_Sample\_011046840, Unigene38285\_Sample\_011046840, Unigene14392\_Sample\_011046840, Unigene4139\_Sample\_011046840, Unigene36839\_Sample\_011046840, Unigene24400\_Sample\_011046840, Unigene36971\_Sample\_011046840, Unigene40950\_Sample\_011046840, Unigene31827\_Sample\_011046840, Unigene33088\_Sample\_011046840, Unigene36063\_Sample\_011046840, Unigene18251\_Sample\_011046840, Unigene42487\_Sample\_011046840, Unigene15903\_Sample\_011046840, Unigene35023\_Sample\_011046840, Unigene6842\_Sample\_011046840, Unigene32606\_Sample\_011046840, Unigene13197\_Sample\_011046840, Unigene5985\_Sample\_011046840, Unigene31283\_Sample\_011046840, Unigene41983\_Sample\_011046840, Unigene21387\_Sample\_011046840, Unigene27604\_Sample\_011046840, Unigene17650\_Sample\_011046840, Unigene38526\_Sample\_011046840, Unigene35976\_Sample\_011046840, Unigene40764\_Sample\_011046840, Unigene27667\_Sample\_011046840, Unigene19027\_Sample\_011046840, Unigene7353\_Sample\_011046840, Unigene32180\_Sample\_011046840, Unigene15237\_Sample\_011046840, Unigene40726\_Sample\_011046840, Unigene42638\_Sample\_011046840, Unigene19219\_Sample\_011046840, Unigene5707\_Sample\_011046840, Unigene17339\_Sample\_011046840, Unigene35227\_Sample\_011046840, Unigene38242\_Sample\_011046840, Unigene33279\_Sample\_011046840, Unigene24546\_Sample\_011046840, Unigene25298\_Sample\_011046840, Unigene31759\_Sample\_011046840, Unigene12410\_Sample\_011046840, Unigene38772\_Sample\_011046840, Unigene37440\_Sample\_011046840, Unigene28079\_Sample\_011046840, Unigene10436\_Sample\_011046840, Unigene43064\_Sample\_011046840, Unigene40520\_Sample\_011046840, Unigene32702\_Sample\_011046840, Unigene29405\_Sample\_011046840, Unigene39983\_Sample\_011046840, Unigene19991\_Sample\_011046840, Unigene42622\_Sample\_011046840, Unigene41793\_Sample\_011046840, Unigene39948\_Sample\_011046840, Unigene5658\_Sample\_011046840, Unigene33671\_Sample\_011046840, Unigene42763\_Sample\_011046840, Unigene38378\_Sample\_011046840, Unigene13641\_Sample\_011046840, Unigene6346\_Sample\_011046840, Unigene14981\_Sample\_011046840, Unigene3828\_Sample\_011046840, Unigene6395\_Sample\_011046840, Unigene2916\_Sample\_011046840, Unigene40716\_Sample\_011046840, Unigene29928\_Sample\_011046840, Unigene22209\_Sample\_011046840, Unigene11244\_Sample\_011046840, Unigene8189\_Sample\_011046840, Unigene40234\_Sample\_011046840, Unigene33379\_Sample\_011046840, Unigene39123\_Sample\_011046840, Unigene37694\_Sample\_011046840, Unigene12204\_Sample\_011046840, Unigene19740\_Sample\_011046840, Unigene4042\_Sample\_011046840, Unigene39967\_Sample\_011046840, Unigene43198\_Sample\_011046840, Unigene41086\_Sample\_011046840, Unigene11516\_Sample\_011046840, Unigene31973\_Sample\_011046840, Unigene39052\_Sample\_011046840, Unigene38279\_Sample\_011046840, Unigene25915\_Sample\_011046840, Unigene7631\_Sample\_011046840, Unigene30400\_Sample\_011046840, Unigene41681\_Sample\_011046840, Unigene29592\_Sample\_011046840, Unigene3486\_Sample\_011046840, Unigene38262\_Sample\_011046840, Unigene34754\_Sample\_011046840, Unigene35053\_Sample\_011046840, Unigene42702\_Sample\_011046840, Unigene11883\_Sample\_011046840, Unigene42866\_Sample\_011046840, Unigene40010\_Sample\_011046840, Unigene37330\_Sample\_011046840, Unigene21296\_Sample\_011046840, Unigene7708\_Sample\_011046840, Unigene37921\_Sample\_011046840, Unigene20922\_Sample\_011046840, Unigene28301\_Sample\_011046840, Unigene41242\_Sample\_011046840, Unigene41510\_Sample\_011046840, Unigene27805\_Sample\_011046840, Unigene43165\_Sample\_011046840, Unigene32262\_Sample\_011046840, Unigene22393\_Sample\_011046840, Unigene16493\_Sample\_011046840, Unigene18168\_Sample\_011046840, Unigene11142\_Sample\_011046840, Unigene32307\_Sample\_011046840, Unigene42027\_Sample\_011046840, Unigene21554\_Sample\_011046840, Unigene8130\_Sample\_011046840, Unigene20146\_Sample\_011046840, Unigene7172\_Sample\_011046840, Unigene42096\_Sample\_011046840, Unigene5647\_Sample\_011046840, Unigene41869\_Sample\_011046840, Unigene19505\_Sample\_011046840, Unigene16302\_Sample\_011046840, Unigene36983\_Sample\_011046840, Unigene37827\_Sample\_011046840, Unigene24404\_Sample\_011046840, Unigene42692\_Sample\_011046840, Unigene40128\_Sample\_011046840, Unigene25020\_Sample\_011046840, Unigene43593\_Sample\_011046840, Unigene11571\_Sample\_011046840, Unigene28366\_Sample\_011046840, Unigene24019\_Sample\_011046840, Unigene7767\_Sample\_011046840, Unigene43201\_Sample\_011046840, Unigene41281\_Sample\_011046840, Unigene14437\_Sample\_011046840, Unigene7768\_Sample\_011046840, Unigene14257\_Sample\_011046840 |
| adenyl ribonucleotide binding | Unigene38023\_Sample\_011046840, Unigene39461\_Sample\_011046840, Unigene20405\_Sample\_011046840, Unigene38706\_Sample\_011046840, Unigene9443\_Sample\_011046840, Unigene368\_Sample\_011046840, Unigene3429\_Sample\_011046840, Unigene37568\_Sample\_011046840, Unigene19401\_Sample\_011046840, Unigene41047\_Sample\_011046840, Unigene18730\_Sample\_011046840, Unigene14932\_Sample\_011046840, Unigene43154\_Sample\_011046840, Unigene902\_Sample\_011046840, Unigene43529\_Sample\_011046840, Unigene27378\_Sample\_011046840, Unigene22180\_Sample\_011046840, Unigene43365\_Sample\_011046840, Unigene20380\_Sample\_011046840, Unigene21224\_Sample\_011046840, Unigene27537\_Sample\_011046840, Unigene7557\_Sample\_011046840, Unigene42372\_Sample\_011046840, Unigene31094\_Sample\_011046840, Unigene41153\_Sample\_011046840, Unigene20314\_Sample\_011046840, Unigene32455\_Sample\_011046840, Unigene15132\_Sample\_011046840, Unigene35962\_Sample\_011046840, Unigene4259\_Sample\_011046840, Unigene4677\_Sample\_011046840, Unigene36928\_Sample\_011046840, Unigene24531\_Sample\_011046840, Unigene32335\_Sample\_011046840, Unigene42401\_Sample\_011046840, Unigene25485\_Sample\_011046840, Unigene6748\_Sample\_011046840, Unigene34978\_Sample\_011046840, Unigene8081\_Sample\_011046840, Unigene34971\_Sample\_011046840, Unigene37185\_Sample\_011046840, Unigene30370\_Sample\_011046840, Unigene32811\_Sample\_011046840, Unigene12614\_Sample\_011046840, Unigene1458\_Sample\_011046840, Unigene17494\_Sample\_011046840, Unigene21883\_Sample\_011046840, Unigene37375\_Sample\_011046840, Unigene8178\_Sample\_011046840, Unigene36909\_Sample\_011046840, Unigene1132\_Sample\_011046840, Unigene21176\_Sample\_011046840, Unigene25680\_Sample\_011046840, Unigene38973\_Sample\_011046840, Unigene7529\_Sample\_011046840, Unigene22101\_Sample\_011046840, Unigene40181\_Sample\_011046840, Unigene39544\_Sample\_011046840, Unigene3849\_Sample\_011046840, Unigene38463\_Sample\_011046840, Unigene177\_Sample\_011046840, Unigene41833\_Sample\_011046840, Unigene4089\_Sample\_011046840, Unigene14294\_Sample\_011046840, Unigene30285\_Sample\_011046840, Unigene36764\_Sample\_011046840, Unigene37848\_Sample\_011046840, Unigene14393\_Sample\_011046840, Unigene5726\_Sample\_011046840, Unigene38504\_Sample\_011046840, Unigene36848\_Sample\_011046840, Unigene19725\_Sample\_011046840, Unigene37095\_Sample\_011046840, Unigene30893\_Sample\_011046840, Unigene3319\_Sample\_011046840, Unigene13306\_Sample\_011046840, Unigene24816\_Sample\_011046840, Unigene20757\_Sample\_011046840, Unigene33428\_Sample\_011046840, Unigene24254\_Sample\_011046840, Unigene7831\_Sample\_011046840, Unigene893\_Sample\_011046840, Unigene43435\_Sample\_011046840, Unigene30471\_Sample\_011046840, Unigene40159\_Sample\_011046840, Unigene29784\_Sample\_011046840, Unigene39812\_Sample\_011046840, Unigene27998\_Sample\_011046840, Unigene19834\_Sample\_011046840, Unigene42253\_Sample\_011046840, Unigene29947\_Sample\_011046840, Unigene34234\_Sample\_011046840, Unigene25555\_Sample\_011046840, Unigene42502\_Sample\_011046840, Unigene41104\_Sample\_011046840, Unigene11113\_Sample\_011046840, Unigene41150\_Sample\_011046840, Unigene4830\_Sample\_011046840, Unigene41496\_Sample\_011046840, Unigene16527\_Sample\_011046840, Unigene43077\_Sample\_011046840, Unigene40073\_Sample\_011046840, Unigene37822\_Sample\_011046840, Unigene9565\_Sample\_011046840, Unigene5249\_Sample\_011046840, Unigene31599\_Sample\_011046840, Unigene22020\_Sample\_011046840, Unigene16161\_Sample\_011046840, Unigene5118\_Sample\_011046840, Unigene35603\_Sample\_011046840, Unigene16645\_Sample\_011046840, Unigene32978\_Sample\_011046840, Unigene42351\_Sample\_011046840, Unigene33208\_Sample\_011046840, Unigene36893\_Sample\_011046840, Unigene36637\_Sample\_011046840, Unigene30615\_Sample\_011046840, Unigene29270\_Sample\_011046840, Unigene13215\_Sample\_011046840, Unigene4578\_Sample\_011046840, Unigene15884\_Sample\_011046840, Unigene43230\_Sample\_011046840, Unigene42704\_Sample\_011046840, Unigene40406\_Sample\_011046840, Unigene37855\_Sample\_011046840, Unigene42242\_Sample\_011046840, Unigene20758\_Sample\_011046840, Unigene39911\_Sample\_011046840, Unigene36345\_Sample\_011046840, Unigene31215\_Sample\_011046840, Unigene41932\_Sample\_011046840, Unigene34105\_Sample\_011046840, Unigene20100\_Sample\_011046840, Unigene5001\_Sample\_011046840, Unigene39763\_Sample\_011046840, Unigene8801\_Sample\_011046840, Unigene37863\_Sample\_011046840, Unigene8554\_Sample\_011046840, Unigene39218\_Sample\_011046840, Unigene41866\_Sample\_011046840, Unigene39982\_Sample\_011046840, Unigene20594\_Sample\_011046840, Unigene43537\_Sample\_011046840, Unigene20\_Sample\_011046840, Unigene33968\_Sample\_011046840, Unigene30239\_Sample\_011046840, Unigene37613\_Sample\_011046840, Unigene32733\_Sample\_011046840, Unigene37902\_Sample\_011046840, Unigene3770\_Sample\_011046840, Unigene1222\_Sample\_011046840, Unigene17734\_Sample\_011046840, Unigene38066\_Sample\_011046840, Unigene38847\_Sample\_011046840, Unigene26711\_Sample\_011046840, Unigene38443\_Sample\_011046840, Unigene18659\_Sample\_011046840, Unigene20968\_Sample\_011046840, Unigene29802\_Sample\_011046840, Unigene42061\_Sample\_011046840, Unigene33983\_Sample\_011046840, Unigene6796\_Sample\_011046840, Unigene20491\_Sample\_011046840, Unigene35734\_Sample\_011046840, Unigene4994\_Sample\_011046840, Unigene33913\_Sample\_011046840, Unigene41476\_Sample\_011046840, Unigene13127\_Sample\_011046840, Unigene41389\_Sample\_011046840, Unigene4309\_Sample\_011046840, Unigene36619\_Sample\_011046840, Unigene16211\_Sample\_011046840, Unigene38206\_Sample\_011046840, Unigene9477\_Sample\_011046840, Unigene43332\_Sample\_011046840, Unigene41743\_Sample\_011046840, Unigene29953\_Sample\_011046840, Unigene38834\_Sample\_011046840, Unigene20493\_Sample\_011046840, Unigene32099\_Sample\_011046840, Unigene41898\_Sample\_011046840, Unigene37706\_Sample\_011046840, Unigene31090\_Sample\_011046840, Unigene3003\_Sample\_011046840, Unigene42374\_Sample\_011046840, Unigene15465\_Sample\_011046840, Unigene4389\_Sample\_011046840, Unigene41997\_Sample\_011046840, Unigene38687\_Sample\_011046840, Unigene5116\_Sample\_011046840, Unigene40471\_Sample\_011046840, Unigene42605\_Sample\_011046840, Unigene41161\_Sample\_011046840, Unigene34519\_Sample\_011046840, Unigene33595\_Sample\_011046840, Unigene31273\_Sample\_011046840, Unigene29985\_Sample\_011046840, Unigene907\_Sample\_011046840, Unigene33768\_Sample\_011046840, Unigene10236\_Sample\_011046840, Unigene36897\_Sample\_011046840, Unigene29839\_Sample\_011046840, Unigene2457\_Sample\_011046840, Unigene26813\_Sample\_011046840, Unigene6547\_Sample\_011046840, Unigene26216\_Sample\_011046840, Unigene35061\_Sample\_011046840, Unigene279\_Sample\_011046840, Unigene42759\_Sample\_011046840, Unigene2459\_Sample\_011046840, Unigene36366\_Sample\_011046840, Unigene19628\_Sample\_011046840, Unigene38962\_Sample\_011046840, Unigene34002\_Sample\_011046840, Unigene40216\_Sample\_011046840, Unigene41431\_Sample\_011046840, Unigene42859\_Sample\_011046840, Unigene30600\_Sample\_011046840, Unigene29629\_Sample\_011046840, Unigene43055\_Sample\_011046840, Unigene23556\_Sample\_011046840, Unigene15492\_Sample\_011046840, Unigene5087\_Sample\_011046840, Unigene20079\_Sample\_011046840, Unigene32197\_Sample\_011046840, Unigene6313\_Sample\_011046840, Unigene31572\_Sample\_011046840, Unigene27920\_Sample\_011046840, Unigene9002\_Sample\_011046840, Unigene39499\_Sample\_011046840, Unigene14420\_Sample\_011046840, Unigene39061\_Sample\_011046840, Unigene27158\_Sample\_011046840, Unigene24281\_Sample\_011046840, Unigene38441\_Sample\_011046840, Unigene10533\_Sample\_011046840, Unigene37904\_Sample\_011046840, Unigene43606\_Sample\_011046840, Unigene34854\_Sample\_011046840, Unigene37976\_Sample\_011046840, Unigene10832\_Sample\_011046840, Unigene15502\_Sample\_011046840, Unigene41919\_Sample\_011046840, Unigene4779\_Sample\_011046840, Unigene10749\_Sample\_011046840, Unigene25548\_Sample\_011046840, Unigene14498\_Sample\_011046840, Unigene39972\_Sample\_011046840, Unigene41368\_Sample\_011046840, Unigene5372\_Sample\_011046840, Unigene38709\_Sample\_011046840, Unigene38720\_Sample\_011046840, Unigene31864\_Sample\_011046840, Unigene37214\_Sample\_011046840, Unigene43188\_Sample\_011046840, Unigene39660\_Sample\_011046840, Unigene36990\_Sample\_011046840, Unigene4344\_Sample\_011046840, Unigene39243\_Sample\_011046840, Unigene33012\_Sample\_011046840, Unigene37292\_Sample\_011046840, Unigene9682\_Sample\_011046840, Unigene43312\_Sample\_011046840, Unigene32292\_Sample\_011046840, Unigene35790\_Sample\_011046840, Unigene32209\_Sample\_011046840, Unigene16376\_Sample\_011046840, Unigene17229\_Sample\_011046840, Unigene39165\_Sample\_011046840, Unigene15033\_Sample\_011046840, Unigene43000\_Sample\_011046840, Unigene30860\_Sample\_011046840, Unigene26740\_Sample\_011046840, Unigene16473\_Sample\_011046840, Unigene3551\_Sample\_011046840, Unigene5570\_Sample\_011046840, Unigene40679\_Sample\_011046840, Unigene37870\_Sample\_011046840, Unigene26427\_Sample\_011046840, Unigene42499\_Sample\_011046840, Unigene16770\_Sample\_011046840, Unigene10730\_Sample\_011046840, Unigene31855\_Sample\_011046840, Unigene29526\_Sample\_011046840, Unigene33605\_Sample\_011046840, Unigene33711\_Sample\_011046840, Unigene34669\_Sample\_011046840, Unigene35906\_Sample\_011046840, Unigene898\_Sample\_011046840, Unigene43635\_Sample\_011046840, Unigene34301\_Sample\_011046840, Unigene35346\_Sample\_011046840, Unigene5439\_Sample\_011046840, Unigene38337\_Sample\_011046840, Unigene32844\_Sample\_011046840, Unigene1375\_Sample\_011046840, Unigene34436\_Sample\_011046840, Unigene1731\_Sample\_011046840, Unigene16611\_Sample\_011046840, Unigene8083\_Sample\_011046840, Unigene10704\_Sample\_011046840, Unigene26270\_Sample\_011046840, Unigene7744\_Sample\_011046840, Unigene25644\_Sample\_011046840, Unigene27239\_Sample\_011046840, Unigene7065\_Sample\_011046840, Unigene15181\_Sample\_011046840, Unigene34833\_Sample\_011046840, Unigene7175\_Sample\_011046840, Unigene7395\_Sample\_011046840, Unigene23201\_Sample\_011046840, Unigene8227\_Sample\_011046840, Unigene22068\_Sample\_011046840, Unigene7410\_Sample\_011046840, Unigene39018\_Sample\_011046840, Unigene14074\_Sample\_011046840, Unigene42615\_Sample\_011046840, Unigene36773\_Sample\_011046840, Unigene17905\_Sample\_011046840, Unigene8006\_Sample\_011046840, Unigene2797\_Sample\_011046840, Unigene40658\_Sample\_011046840, Unigene25347\_Sample\_011046840, Unigene35547\_Sample\_011046840, Unigene20556\_Sample\_011046840, Unigene22514\_Sample\_011046840, Unigene30760\_Sample\_011046840, Unigene40427\_Sample\_011046840, Unigene2810\_Sample\_011046840, Unigene33019\_Sample\_011046840, Unigene40032\_Sample\_011046840, Unigene33870\_Sample\_011046840, Unigene37571\_Sample\_011046840, Unigene36877\_Sample\_011046840, Unigene15851\_Sample\_011046840, Unigene39740\_Sample\_011046840, Unigene42384\_Sample\_011046840, Unigene27359\_Sample\_011046840, Unigene23158\_Sample\_011046840, Unigene42441\_Sample\_011046840, Unigene35031\_Sample\_011046840, Unigene20101\_Sample\_011046840, Unigene42659\_Sample\_011046840, Unigene37034\_Sample\_011046840, Unigene38074\_Sample\_011046840, Unigene40484\_Sample\_011046840, Unigene18009\_Sample\_011046840, Unigene36141\_Sample\_011046840, Unigene42234\_Sample\_011046840, Unigene23241\_Sample\_011046840, Unigene10778\_Sample\_011046840, Unigene39927\_Sample\_011046840, Unigene5564\_Sample\_011046840, Unigene36685\_Sample\_011046840, Unigene41585\_Sample\_011046840, Unigene15148\_Sample\_011046840, Unigene4032\_Sample\_011046840, Unigene43082\_Sample\_011046840, Unigene36922\_Sample\_011046840, Unigene7796\_Sample\_011046840, Unigene5007\_Sample\_011046840, Unigene15874\_Sample\_011046840, Unigene34588\_Sample\_011046840, Unigene8203\_Sample\_011046840, Unigene38501\_Sample\_011046840, Unigene40793\_Sample\_011046840, Unigene22867\_Sample\_011046840, Unigene42340\_Sample\_011046840, Unigene41400\_Sample\_011046840, Unigene35494\_Sample\_011046840, Unigene12635\_Sample\_011046840, Unigene36375\_Sample\_011046840, Unigene24034\_Sample\_011046840, Unigene24711\_Sample\_011046840, Unigene42189\_Sample\_011046840, Unigene24309\_Sample\_011046840, Unigene41899\_Sample\_011046840, Unigene33906\_Sample\_011046840, Unigene28095\_Sample\_011046840, Unigene36121\_Sample\_011046840, Unigene8240\_Sample\_011046840, Unigene27829\_Sample\_011046840, Unigene40541\_Sample\_011046840, Unigene18069\_Sample\_011046840, Unigene41399\_Sample\_011046840, Unigene12936\_Sample\_011046840, Unigene29046\_Sample\_011046840, Unigene40917\_Sample\_011046840, Unigene42075\_Sample\_011046840, Unigene38028\_Sample\_011046840, Unigene11264\_Sample\_011046840, Unigene41676\_Sample\_011046840, Unigene35179\_Sample\_011046840, Unigene17789\_Sample\_011046840, Unigene40221\_Sample\_011046840, Unigene12818\_Sample\_011046840, Unigene7262\_Sample\_011046840, Unigene42206\_Sample\_011046840, Unigene16016\_Sample\_011046840, Unigene12035\_Sample\_011046840, Unigene2808\_Sample\_011046840, Unigene40596\_Sample\_011046840, Unigene1771\_Sample\_011046840, Unigene972\_Sample\_011046840, Unigene8555\_Sample\_011046840, Unigene7148\_Sample\_011046840, Unigene2084\_Sample\_011046840, Unigene39583\_Sample\_011046840, Unigene38393\_Sample\_011046840, Unigene35532\_Sample\_011046840, Unigene32382\_Sample\_011046840, Unigene2815\_Sample\_011046840, Unigene7669\_Sample\_011046840, Unigene31663\_Sample\_011046840, Unigene24311\_Sample\_011046840, Unigene14178\_Sample\_011046840, Unigene17776\_Sample\_011046840, Unigene29611\_Sample\_011046840, Unigene41927\_Sample\_011046840, Unigene29621\_Sample\_011046840, Unigene27942\_Sample\_011046840, Unigene41127\_Sample\_011046840, Unigene13984\_Sample\_011046840, Unigene34362\_Sample\_011046840, Unigene32819\_Sample\_011046840, Unigene30713\_Sample\_011046840, Unigene5552\_Sample\_011046840, Unigene15637\_Sample\_011046840, Unigene34780\_Sample\_011046840, Unigene7506\_Sample\_011046840, Unigene36827\_Sample\_011046840, Unigene19404\_Sample\_011046840, Unigene43143\_Sample\_011046840, Unigene9887\_Sample\_011046840, Unigene36413\_Sample\_011046840, Unigene11592\_Sample\_011046840, Unigene35789\_Sample\_011046840, Unigene15030\_Sample\_011046840, Unigene40822\_Sample\_011046840, Unigene24453\_Sample\_011046840, Unigene42663\_Sample\_011046840, Unigene5963\_Sample\_011046840, Unigene13602\_Sample\_011046840, Unigene43397\_Sample\_011046840, Unigene19793\_Sample\_011046840, Unigene32158\_Sample\_011046840, Unigene42596\_Sample\_011046840, Unigene38864\_Sample\_011046840, Unigene20577\_Sample\_011046840, Unigene14302\_Sample\_011046840, Unigene31135\_Sample\_011046840, Unigene25783\_Sample\_011046840, Unigene25531\_Sample\_011046840, Unigene7067\_Sample\_011046840, Unigene43623\_Sample\_011046840, Unigene7349\_Sample\_011046840, Unigene3226\_Sample\_011046840, Unigene17721\_Sample\_011046840, Unigene31168\_Sample\_011046840, Unigene34071\_Sample\_011046840, Unigene41691\_Sample\_011046840, Unigene26581\_Sample\_011046840, Unigene28708\_Sample\_011046840, Unigene42865\_Sample\_011046840, Unigene6064\_Sample\_011046840, Unigene16389\_Sample\_011046840, Unigene3257\_Sample\_011046840, Unigene29534\_Sample\_011046840, Unigene22159\_Sample\_011046840, Unigene29452\_Sample\_011046840, Unigene32280\_Sample\_011046840, Unigene10176\_Sample\_011046840, Unigene31074\_Sample\_011046840, Unigene15062\_Sample\_011046840, Unigene17700\_Sample\_011046840, Unigene36699\_Sample\_011046840, Unigene28415\_Sample\_011046840, Unigene33858\_Sample\_011046840, Unigene38658\_Sample\_011046840, Unigene23820\_Sample\_011046840, Unigene7402\_Sample\_011046840, Unigene5375\_Sample\_011046840, Unigene10829\_Sample\_011046840, Unigene11629\_Sample\_011046840, Unigene43387\_Sample\_011046840, Unigene35909\_Sample\_011046840, Unigene26502\_Sample\_011046840, Unigene36268\_Sample\_011046840, Unigene29818\_Sample\_011046840, Unigene31458\_Sample\_011046840, Unigene30136\_Sample\_011046840, Unigene24201\_Sample\_011046840, Unigene8372\_Sample\_011046840, Unigene40709\_Sample\_011046840, Unigene35699\_Sample\_011046840, Unigene40966\_Sample\_011046840, Unigene489\_Sample\_011046840, Unigene10957\_Sample\_011046840, Unigene4518\_Sample\_011046840, Unigene33342\_Sample\_011046840, Unigene26057\_Sample\_011046840, Unigene34506\_Sample\_011046840, Unigene20052\_Sample\_011046840, Unigene42097\_Sample\_011046840, Unigene24244\_Sample\_011046840, Unigene41398\_Sample\_011046840, Unigene7025\_Sample\_011046840, Unigene22635\_Sample\_011046840, Unigene27993\_Sample\_011046840, Unigene39020\_Sample\_011046840, Unigene40262\_Sample\_011046840, Unigene9922\_Sample\_011046840, Unigene24917\_Sample\_011046840, Unigene32947\_Sample\_011046840, Unigene32360\_Sample\_011046840, Unigene39324\_Sample\_011046840, Unigene32594\_Sample\_011046840, Unigene22752\_Sample\_011046840, Unigene40551\_Sample\_011046840, Unigene41178\_Sample\_011046840, Unigene43627\_Sample\_011046840, Unigene33112\_Sample\_011046840, Unigene37999\_Sample\_011046840, Unigene6285\_Sample\_011046840, Unigene28867\_Sample\_011046840, Unigene33999\_Sample\_011046840, Unigene31448\_Sample\_011046840, Unigene29049\_Sample\_011046840, Unigene32831\_Sample\_011046840, Unigene26512\_Sample\_011046840, Unigene42138\_Sample\_011046840, Unigene37985\_Sample\_011046840, Unigene14485\_Sample\_011046840, Unigene36500\_Sample\_011046840, Unigene19235\_Sample\_011046840, Unigene20304\_Sample\_011046840, Unigene42585\_Sample\_011046840, Unigene34472\_Sample\_011046840, Unigene15594\_Sample\_011046840, Unigene33393\_Sample\_011046840, Unigene31108\_Sample\_011046840, Unigene16321\_Sample\_011046840, Unigene8615\_Sample\_011046840, Unigene33196\_Sample\_011046840, Unigene19400\_Sample\_011046840, Unigene4619\_Sample\_011046840, Unigene24373\_Sample\_011046840, Unigene19647\_Sample\_011046840, Unigene34843\_Sample\_011046840, Unigene10033\_Sample\_011046840, Unigene40222\_Sample\_011046840, Unigene38165\_Sample\_011046840, Unigene33473\_Sample\_011046840, Unigene30539\_Sample\_011046840, Unigene39171\_Sample\_011046840, Unigene37353\_Sample\_011046840, Unigene42460\_Sample\_011046840, Unigene26767\_Sample\_011046840, Unigene16855\_Sample\_011046840, Unigene38564\_Sample\_011046840, Unigene25769\_Sample\_011046840, Unigene19809\_Sample\_011046840, Unigene39825\_Sample\_011046840, Unigene40368\_Sample\_011046840, Unigene6561\_Sample\_011046840, Unigene22844\_Sample\_011046840, Unigene10568\_Sample\_011046840, Unigene43614\_Sample\_011046840, Unigene41718\_Sample\_011046840, Unigene38534\_Sample\_011046840, Unigene36032\_Sample\_011046840, Unigene535\_Sample\_011046840, Unigene32515\_Sample\_011046840, Unigene39951\_Sample\_011046840, Unigene38730\_Sample\_011046840, Unigene43450\_Sample\_011046840, Unigene40192\_Sample\_011046840, Unigene39935\_Sample\_011046840, Unigene473\_Sample\_011046840, Unigene39427\_Sample\_011046840, Unigene25001\_Sample\_011046840, Unigene20367\_Sample\_011046840, Unigene31408\_Sample\_011046840, Unigene8211\_Sample\_011046840, Unigene4307\_Sample\_011046840, Unigene39007\_Sample\_011046840, Unigene39015\_Sample\_011046840, Unigene37487\_Sample\_011046840, Unigene38616\_Sample\_011046840, Unigene43281\_Sample\_011046840, Unigene815\_Sample\_011046840, Unigene13836\_Sample\_011046840, Unigene26820\_Sample\_011046840, Unigene42164\_Sample\_011046840, Unigene6530\_Sample\_011046840, Unigene412\_Sample\_011046840, Unigene38370\_Sample\_011046840, Unigene38792\_Sample\_011046840, Unigene30813\_Sample\_011046840, Unigene43078\_Sample\_011046840, Unigene22254\_Sample\_011046840, Unigene34112\_Sample\_011046840, Unigene40971\_Sample\_011046840, Unigene43019\_Sample\_011046840, Unigene27313\_Sample\_011046840, Unigene17135\_Sample\_011046840, Unigene43311\_Sample\_011046840, Unigene43216\_Sample\_011046840, Unigene24243\_Sample\_011046840, Unigene34992\_Sample\_011046840, Unigene6409\_Sample\_011046840, Unigene42997\_Sample\_011046840, Unigene42121\_Sample\_011046840, Unigene27337\_Sample\_011046840, Unigene9880\_Sample\_011046840, Unigene35787\_Sample\_011046840, Unigene11464\_Sample\_011046840, Unigene39486\_Sample\_011046840, Unigene35792\_Sample\_011046840, Unigene6378\_Sample\_011046840, Unigene7938\_Sample\_011046840, Unigene33580\_Sample\_011046840, Unigene6293\_Sample\_011046840, Unigene28473\_Sample\_011046840, Unigene27358\_Sample\_011046840, Unigene37793\_Sample\_011046840, Unigene15491\_Sample\_011046840, Unigene32568\_Sample\_011046840, Unigene41588\_Sample\_011046840, Unigene43176\_Sample\_011046840, Unigene36475\_Sample\_011046840, Unigene14116\_Sample\_011046840, Unigene37359\_Sample\_011046840, Unigene38852\_Sample\_011046840, Unigene13391\_Sample\_011046840, Unigene39348\_Sample\_011046840, Unigene11853\_Sample\_011046840, Unigene39080\_Sample\_011046840, Unigene38047\_Sample\_011046840, Unigene40881\_Sample\_011046840, Unigene12767\_Sample\_011046840, Unigene35476\_Sample\_011046840, Unigene29006\_Sample\_011046840, Unigene39136\_Sample\_011046840, Unigene15696\_Sample\_011046840, Unigene30322\_Sample\_011046840, Unigene5815\_Sample\_011046840, Unigene29131\_Sample\_011046840, Unigene39668\_Sample\_011046840, Unigene40903\_Sample\_011046840, Unigene42227\_Sample\_011046840, Unigene40992\_Sample\_011046840, Unigene36427\_Sample\_011046840, Unigene16130\_Sample\_011046840, Unigene34102\_Sample\_011046840, Unigene36876\_Sample\_011046840, Unigene24004\_Sample\_011046840, Unigene39404\_Sample\_011046840, Unigene26969\_Sample\_011046840, Unigene34453\_Sample\_011046840, Unigene12655\_Sample\_011046840, Unigene37877\_Sample\_011046840, Unigene27975\_Sample\_011046840, Unigene14511\_Sample\_011046840, Unigene32134\_Sample\_011046840, Unigene32749\_Sample\_011046840, Unigene26734\_Sample\_011046840, Unigene43062\_Sample\_011046840, Unigene29301\_Sample\_011046840, Unigene42383\_Sample\_011046840, Unigene28404\_Sample\_011046840, Unigene16269\_Sample\_011046840, Unigene40565\_Sample\_011046840, Unigene39381\_Sample\_011046840, Unigene2174\_Sample\_011046840, Unigene32865\_Sample\_011046840, Unigene27180\_Sample\_011046840, Unigene688\_Sample\_011046840, Unigene36400\_Sample\_011046840, Unigene43084\_Sample\_011046840, Unigene3156\_Sample\_011046840, Unigene35434\_Sample\_011046840, Unigene6180\_Sample\_011046840, Unigene33938\_Sample\_011046840, Unigene40865\_Sample\_011046840, Unigene39866\_Sample\_011046840, Unigene31310\_Sample\_011046840, Unigene23252\_Sample\_011046840, Unigene42296\_Sample\_011046840, Unigene22122\_Sample\_011046840, Unigene37628\_Sample\_011046840, Unigene11949\_Sample\_011046840, Unigene34464\_Sample\_011046840, Unigene36926\_Sample\_011046840, Unigene42789\_Sample\_011046840, Unigene13828\_Sample\_011046840, Unigene1963\_Sample\_011046840, Unigene39156\_Sample\_011046840, Unigene11179\_Sample\_011046840, Unigene40994\_Sample\_011046840, Unigene1256\_Sample\_011046840, Unigene6241\_Sample\_011046840, Unigene37705\_Sample\_011046840, Unigene16976\_Sample\_011046840, Unigene31945\_Sample\_011046840, Unigene23357\_Sample\_011046840, Unigene23281\_Sample\_011046840, Unigene37392\_Sample\_011046840, Unigene39992\_Sample\_011046840, Unigene8218\_Sample\_011046840, Unigene36706\_Sample\_011046840, Unigene41792\_Sample\_011046840, Unigene4723\_Sample\_011046840, Unigene30691\_Sample\_011046840, Unigene40837\_Sample\_011046840, Unigene14017\_Sample\_011046840, Unigene36112\_Sample\_011046840, Unigene20250\_Sample\_011046840, Unigene7503\_Sample\_011046840, Unigene30919\_Sample\_011046840, Unigene14611\_Sample\_011046840, Unigene17216\_Sample\_011046840, Unigene40424\_Sample\_011046840, Unigene3599\_Sample\_011046840, Unigene23324\_Sample\_011046840, Unigene29427\_Sample\_011046840, Unigene17375\_Sample\_011046840, Unigene36567\_Sample\_011046840, Unigene15303\_Sample\_011046840, Unigene16035\_Sample\_011046840, Unigene27997\_Sample\_011046840, Unigene42864\_Sample\_011046840, Unigene36920\_Sample\_011046840, Unigene13276\_Sample\_011046840, Unigene39180\_Sample\_011046840, Unigene43536\_Sample\_011046840, Unigene34655\_Sample\_011046840, Unigene40818\_Sample\_011046840, Unigene38377\_Sample\_011046840, Unigene18440\_Sample\_011046840, Unigene37830\_Sample\_011046840, Unigene33866\_Sample\_011046840, Unigene43607\_Sample\_011046840, Unigene21072\_Sample\_011046840, Unigene12713\_Sample\_011046840, Unigene22459\_Sample\_011046840, Unigene33926\_Sample\_011046840, Unigene37219\_Sample\_011046840, Unigene38559\_Sample\_011046840, Unigene11613\_Sample\_011046840, Unigene38360\_Sample\_011046840, Unigene38044\_Sample\_011046840, Unigene42007\_Sample\_011046840, Unigene41174\_Sample\_011046840, Unigene15831\_Sample\_011046840, Unigene41740\_Sample\_011046840, Unigene432\_Sample\_011046840, Unigene17416\_Sample\_011046840, Unigene40346\_Sample\_011046840, Unigene35883\_Sample\_011046840, Unigene31610\_Sample\_011046840, Unigene37041\_Sample\_011046840, Unigene30572\_Sample\_011046840, Unigene23630\_Sample\_011046840, Unigene33760\_Sample\_011046840, Unigene38146\_Sample\_011046840, Unigene24246\_Sample\_011046840, Unigene31584\_Sample\_011046840, Unigene36376\_Sample\_011046840, Unigene30798\_Sample\_011046840, Unigene39838\_Sample\_011046840, Unigene3842\_Sample\_011046840, Unigene12743\_Sample\_011046840, Unigene31773\_Sample\_011046840, Unigene3928\_Sample\_011046840, Unigene41001\_Sample\_011046840, Unigene23856\_Sample\_011046840, Unigene43115\_Sample\_011046840, Unigene22258\_Sample\_011046840, Unigene17210\_Sample\_011046840, Unigene41498\_Sample\_011046840, Unigene31398\_Sample\_011046840, Unigene10770\_Sample\_011046840, Unigene42171\_Sample\_011046840, Unigene24889\_Sample\_011046840, Unigene9871\_Sample\_011046840, Unigene38932\_Sample\_011046840, Unigene33443\_Sample\_011046840, Unigene38152\_Sample\_011046840, Unigene14629\_Sample\_011046840, Unigene14756\_Sample\_011046840, Unigene42050\_Sample\_011046840, Unigene42835\_Sample\_011046840, Unigene5197\_Sample\_011046840, Unigene29880\_Sample\_011046840, Unigene7717\_Sample\_011046840, Unigene5121\_Sample\_011046840, Unigene43147\_Sample\_011046840, Unigene38039\_Sample\_011046840, Unigene28861\_Sample\_011046840, Unigene42602\_Sample\_011046840, Unigene10135\_Sample\_011046840, Unigene22688\_Sample\_011046840, Unigene10802\_Sample\_011046840, Unigene40391\_Sample\_011046840, Unigene38670\_Sample\_011046840, Unigene29016\_Sample\_011046840, Unigene37540\_Sample\_011046840, Unigene21026\_Sample\_011046840, Unigene36690\_Sample\_011046840, Unigene39732\_Sample\_011046840, Unigene18735\_Sample\_011046840, Unigene32368\_Sample\_011046840, Unigene19481\_Sample\_011046840, Unigene43025\_Sample\_011046840, Unigene41288\_Sample\_011046840, Unigene30094\_Sample\_011046840, Unigene25579\_Sample\_011046840, Unigene34053\_Sample\_011046840, Unigene28414\_Sample\_011046840, Unigene12587\_Sample\_011046840, Unigene39882\_Sample\_011046840, Unigene35872\_Sample\_011046840, Unigene10288\_Sample\_011046840, Unigene9718\_Sample\_011046840, Unigene39555\_Sample\_011046840, Unigene35927\_Sample\_011046840, Unigene5480\_Sample\_011046840, Unigene14666\_Sample\_011046840, Unigene36535\_Sample\_011046840, Unigene12679\_Sample\_011046840, Unigene40687\_Sample\_011046840, Unigene39837\_Sample\_011046840, Unigene23176\_Sample\_011046840, Unigene24594\_Sample\_011046840, Unigene41858\_Sample\_011046840, Unigene14292\_Sample\_011046840, Unigene27724\_Sample\_011046840, Unigene19383\_Sample\_011046840, Unigene10601\_Sample\_011046840, Unigene33259\_Sample\_011046840, Unigene30862\_Sample\_011046840, Unigene41336\_Sample\_011046840, Unigene25871\_Sample\_011046840, Unigene33322\_Sample\_011046840, Unigene30573\_Sample\_011046840, Unigene38115\_Sample\_011046840, Unigene34746\_Sample\_011046840, Unigene36462\_Sample\_011046840, Unigene40812\_Sample\_011046840, Unigene40787\_Sample\_011046840, Unigene37745\_Sample\_011046840, Unigene24674\_Sample\_011046840, Unigene40184\_Sample\_011046840, Unigene28636\_Sample\_011046840, Unigene33889\_Sample\_011046840, Unigene28901\_Sample\_011046840, Unigene43453\_Sample\_011046840, Unigene39490\_Sample\_011046840, Unigene39632\_Sample\_011046840, Unigene11701\_Sample\_011046840, Unigene31638\_Sample\_011046840, Unigene11125\_Sample\_011046840, Unigene42030\_Sample\_011046840, Unigene28758\_Sample\_011046840, Unigene25431\_Sample\_011046840, Unigene4320\_Sample\_011046840, Unigene20420\_Sample\_011046840, Unigene28316\_Sample\_011046840, Unigene6563\_Sample\_011046840, Unigene36951\_Sample\_011046840, Unigene43031\_Sample\_011046840, Unigene12197\_Sample\_011046840, Unigene36090\_Sample\_011046840, Unigene22961\_Sample\_011046840, Unigene43363\_Sample\_011046840, Unigene22232\_Sample\_011046840, Unigene38021\_Sample\_011046840, Unigene18262\_Sample\_011046840, Unigene41561\_Sample\_011046840, Unigene12868\_Sample\_011046840, Unigene37710\_Sample\_011046840, Unigene41901\_Sample\_011046840, Unigene4043\_Sample\_011046840, Unigene42079\_Sample\_011046840, Unigene165\_Sample\_011046840, Unigene7072\_Sample\_011046840, Unigene43611\_Sample\_011046840, Unigene3161\_Sample\_011046840, Unigene21295\_Sample\_011046840, Unigene42944\_Sample\_011046840, Unigene5047\_Sample\_011046840, Unigene1859\_Sample\_011046840, Unigene4721\_Sample\_011046840, Unigene35151\_Sample\_011046840, Unigene29879\_Sample\_011046840, Unigene35324\_Sample\_011046840, Unigene25629\_Sample\_011046840, Unigene29966\_Sample\_011046840, Unigene41467\_Sample\_011046840, Unigene16882\_Sample\_011046840, Unigene42154\_Sample\_011046840, Unigene38267\_Sample\_011046840, Unigene42047\_Sample\_011046840, Unigene25720\_Sample\_011046840, Unigene36496\_Sample\_011046840, Unigene14078\_Sample\_011046840, Unigene39103\_Sample\_011046840, Unigene24184\_Sample\_011046840, Unigene3537\_Sample\_011046840, Unigene38522\_Sample\_011046840, Unigene6787\_Sample\_011046840, Unigene37573\_Sample\_011046840, Unigene25466\_Sample\_011046840, Unigene31793\_Sample\_011046840, Unigene12386\_Sample\_011046840, Unigene29845\_Sample\_011046840, Unigene35118\_Sample\_011046840, Unigene42837\_Sample\_011046840, Unigene42475\_Sample\_011046840, Unigene42531\_Sample\_011046840, Unigene42204\_Sample\_011046840, Unigene2615\_Sample\_011046840, Unigene35939\_Sample\_011046840, Unigene34233\_Sample\_011046840, Unigene39958\_Sample\_011046840, Unigene23541\_Sample\_011046840, Unigene18141\_Sample\_011046840, Unigene42252\_Sample\_011046840, Unigene12084\_Sample\_011046840, Unigene40601\_Sample\_011046840, Unigene32188\_Sample\_011046840, Unigene39418\_Sample\_011046840, Unigene42200\_Sample\_011046840, Unigene21974\_Sample\_011046840, Unigene40208\_Sample\_011046840, Unigene37077\_Sample\_011046840, Unigene20828\_Sample\_011046840, Unigene30505\_Sample\_011046840, Unigene14237\_Sample\_011046840, Unigene42786\_Sample\_011046840, Unigene3395\_Sample\_011046840, Unigene3814\_Sample\_011046840, Unigene42886\_Sample\_011046840, Unigene38215\_Sample\_011046840, Unigene33515\_Sample\_011046840, Unigene38121\_Sample\_011046840, Unigene41880\_Sample\_011046840, Unigene19448\_Sample\_011046840, Unigene42133\_Sample\_011046840, Unigene3164\_Sample\_011046840, Unigene30530\_Sample\_011046840, Unigene20634\_Sample\_011046840, Unigene7794\_Sample\_011046840, Unigene31\_Sample\_011046840, Unigene23873\_Sample\_011046840, Unigene33979\_Sample\_011046840, Unigene27545\_Sample\_011046840, Unigene31822\_Sample\_011046840, Unigene41582\_Sample\_011046840, Unigene38462\_Sample\_011046840, Unigene34355\_Sample\_011046840, Unigene39505\_Sample\_011046840, Unigene8064\_Sample\_011046840, Unigene7983\_Sample\_011046840, Unigene37702\_Sample\_011046840, Unigene29411\_Sample\_011046840, Unigene43525\_Sample\_011046840, Unigene5594\_Sample\_011046840, Unigene35068\_Sample\_011046840, Unigene30197\_Sample\_011046840, Unigene18134\_Sample\_011046840, Unigene35433\_Sample\_011046840, Unigene35554\_Sample\_011046840, Unigene25328\_Sample\_011046840, Unigene39870\_Sample\_011046840, Unigene9730\_Sample\_011046840, Unigene22550\_Sample\_011046840, Unigene24428\_Sample\_011046840, Unigene25313\_Sample\_011046840, Unigene28259\_Sample\_011046840, Unigene37149\_Sample\_011046840, Unigene34500\_Sample\_011046840, Unigene29711\_Sample\_011046840, Unigene40223\_Sample\_011046840, Unigene6498\_Sample\_011046840, Unigene32116\_Sample\_011046840, Unigene31318\_Sample\_011046840, Unigene39179\_Sample\_011046840, Unigene35234\_Sample\_011046840, Unigene3934\_Sample\_011046840, Unigene43422\_Sample\_011046840, Unigene14154\_Sample\_011046840, Unigene2408\_Sample\_011046840, Unigene42127\_Sample\_011046840, Unigene24187\_Sample\_011046840, Unigene23188\_Sample\_011046840, Unigene1039\_Sample\_011046840, Unigene31248\_Sample\_011046840, Unigene35633\_Sample\_011046840, Unigene29346\_Sample\_011046840, Unigene12033\_Sample\_011046840, Unigene40016\_Sample\_011046840, Unigene35643\_Sample\_011046840, Unigene42177\_Sample\_011046840, Unigene33743\_Sample\_011046840, Unigene43636\_Sample\_011046840, Unigene15849\_Sample\_011046840, Unigene23811\_Sample\_011046840, Unigene41355\_Sample\_011046840, Unigene41574\_Sample\_011046840, Unigene31649\_Sample\_011046840, Unigene33736\_Sample\_011046840, Unigene34555\_Sample\_011046840, Unigene10641\_Sample\_011046840, Unigene32875\_Sample\_011046840, Unigene11685\_Sample\_011046840, Unigene4327\_Sample\_011046840, Unigene43095\_Sample\_011046840, Unigene12062\_Sample\_011046840, Unigene16063\_Sample\_011046840, Unigene30508\_Sample\_011046840, Unigene36187\_Sample\_011046840, Unigene5400\_Sample\_011046840, Unigene3339\_Sample\_011046840, Unigene36260\_Sample\_011046840, Unigene32706\_Sample\_011046840, Unigene37852\_Sample\_011046840, Unigene31684\_Sample\_011046840, Unigene28063\_Sample\_011046840, Unigene16650\_Sample\_011046840, Unigene39275\_Sample\_011046840, Unigene12701\_Sample\_011046840, Unigene14279\_Sample\_011046840, Unigene30059\_Sample\_011046840, Unigene32354\_Sample\_011046840, Unigene10035\_Sample\_011046840, Unigene8904\_Sample\_011046840, Unigene36088\_Sample\_011046840, Unigene41771\_Sample\_011046840, Unigene41481\_Sample\_011046840, Unigene42569\_Sample\_011046840, Unigene18212\_Sample\_011046840, Unigene9721\_Sample\_011046840, Unigene8113\_Sample\_011046840, Unigene42574\_Sample\_011046840, Unigene25488\_Sample\_011046840, Unigene41935\_Sample\_011046840, Unigene4815\_Sample\_011046840, Unigene34896\_Sample\_011046840, Unigene14473\_Sample\_011046840, Unigene26865\_Sample\_011046840, Unigene39391\_Sample\_011046840, Unigene23169\_Sample\_011046840, Unigene2581\_Sample\_011046840, Unigene43556\_Sample\_011046840, Unigene41980\_Sample\_011046840, Unigene42348\_Sample\_011046840, Unigene35074\_Sample\_011046840, Unigene39497\_Sample\_011046840, Unigene2218\_Sample\_011046840, Unigene39081\_Sample\_011046840, Unigene14503\_Sample\_011046840, Unigene41972\_Sample\_011046840, Unigene7458\_Sample\_011046840, Unigene21905\_Sample\_011046840, Unigene42768\_Sample\_011046840, Unigene12341\_Sample\_011046840, Unigene41855\_Sample\_011046840, Unigene41007\_Sample\_011046840, Unigene10729\_Sample\_011046840, Unigene19018\_Sample\_011046840, Unigene10318\_Sample\_011046840, Unigene3788\_Sample\_011046840, Unigene13393\_Sample\_011046840, Unigene6862\_Sample\_011046840, Unigene32621\_Sample\_011046840, Unigene23759\_Sample\_011046840, Unigene33797\_Sample\_011046840, Unigene36342\_Sample\_011046840, Unigene2687\_Sample\_011046840, Unigene21944\_Sample\_011046840, Unigene2151\_Sample\_011046840, Unigene42626\_Sample\_011046840, Unigene14750\_Sample\_011046840, Unigene31441\_Sample\_011046840, Unigene35459\_Sample\_011046840, Unigene43348\_Sample\_011046840, Unigene43412\_Sample\_011046840, Unigene2152\_Sample\_011046840, Unigene39717\_Sample\_011046840, Unigene4686\_Sample\_011046840, Unigene17677\_Sample\_011046840, Unigene36544\_Sample\_011046840, Unigene41261\_Sample\_011046840, Unigene17194\_Sample\_011046840, Unigene15889\_Sample\_011046840, Unigene27951\_Sample\_011046840, Unigene31900\_Sample\_011046840, Unigene37045\_Sample\_011046840, Unigene4497\_Sample\_011046840, Unigene2092\_Sample\_011046840, Unigene12675\_Sample\_011046840, Unigene34828\_Sample\_011046840, Unigene24005\_Sample\_011046840, Unigene19653\_Sample\_011046840, Unigene39559\_Sample\_011046840, Unigene40053\_Sample\_011046840, Unigene32836\_Sample\_011046840, Unigene37404\_Sample\_011046840, Unigene32314\_Sample\_011046840, Unigene39437\_Sample\_011046840, Unigene39720\_Sample\_011046840, Unigene5378\_Sample\_011046840, Unigene208\_Sample\_011046840, Unigene43258\_Sample\_011046840, Unigene19918\_Sample\_011046840, Unigene5436\_Sample\_011046840, Unigene20526\_Sample\_011046840, Unigene26338\_Sample\_011046840, Unigene40753\_Sample\_011046840, Unigene15987\_Sample\_011046840, Unigene40278\_Sample\_011046840, Unigene3127\_Sample\_011046840, Unigene26705\_Sample\_011046840, Unigene19593\_Sample\_011046840, Unigene6740\_Sample\_011046840, Unigene37517\_Sample\_011046840, Unigene30236\_Sample\_011046840, Unigene28852\_Sample\_011046840, Unigene33998\_Sample\_011046840, Unigene31994\_Sample\_011046840, Unigene26163\_Sample\_011046840, Unigene27886\_Sample\_011046840, Unigene18907\_Sample\_011046840, Unigene26846\_Sample\_011046840, Unigene5241\_Sample\_011046840, Unigene34039\_Sample\_011046840, Unigene38608\_Sample\_011046840, Unigene2543\_Sample\_011046840, Unigene35951\_Sample\_011046840, Unigene6760\_Sample\_011046840, Unigene21415\_Sample\_011046840, Unigene41637\_Sample\_011046840, Unigene20065\_Sample\_011046840, Unigene28002\_Sample\_011046840, Unigene30702\_Sample\_011046840, Unigene20428\_Sample\_011046840, Unigene14691\_Sample\_011046840, Unigene42798\_Sample\_011046840, Unigene23889\_Sample\_011046840, Unigene40899\_Sample\_011046840, Unigene42418\_Sample\_011046840, Unigene31192\_Sample\_011046840, Unigene21332\_Sample\_011046840, Unigene14501\_Sample\_011046840, Unigene42183\_Sample\_011046840, Unigene34524\_Sample\_011046840, Unigene30629\_Sample\_011046840, Unigene16244\_Sample\_011046840, Unigene42293\_Sample\_011046840, Unigene31880\_Sample\_011046840, Unigene32589\_Sample\_011046840, Unigene23234\_Sample\_011046840, Unigene2740\_Sample\_011046840, Unigene5453\_Sample\_011046840, Unigene21959\_Sample\_011046840, Unigene22620\_Sample\_011046840, Unigene41709\_Sample\_011046840, Unigene43416\_Sample\_011046840, Unigene34975\_Sample\_011046840, Unigene8660\_Sample\_011046840, Unigene37131\_Sample\_011046840, Unigene26023\_Sample\_011046840, Unigene16906\_Sample\_011046840, Unigene29661\_Sample\_011046840, Unigene40616\_Sample\_011046840, Unigene43186\_Sample\_011046840, Unigene29145\_Sample\_011046840, Unigene7789\_Sample\_011046840, Unigene26507\_Sample\_011046840, Unigene31407\_Sample\_011046840, Unigene42281\_Sample\_011046840, Unigene8121\_Sample\_011046840, Unigene34432\_Sample\_011046840, Unigene43273\_Sample\_011046840, Unigene37116\_Sample\_011046840, Unigene32645\_Sample\_011046840, Unigene7513\_Sample\_011046840, Unigene26212\_Sample\_011046840, Unigene2519\_Sample\_011046840, Unigene22727\_Sample\_011046840, Unigene41160\_Sample\_011046840, Unigene42088\_Sample\_011046840, Unigene34380\_Sample\_011046840, Unigene19472\_Sample\_011046840, Unigene34400\_Sample\_011046840, Unigene39994\_Sample\_011046840, Unigene40445\_Sample\_011046840, Unigene19704\_Sample\_011046840, Unigene42738\_Sample\_011046840, Unigene43001\_Sample\_011046840, Unigene18270\_Sample\_011046840, Unigene41119\_Sample\_011046840, Unigene356\_Sample\_011046840, Unigene2068\_Sample\_011046840, Unigene34304\_Sample\_011046840, Unigene36231\_Sample\_011046840, Unigene39253\_Sample\_011046840, Unigene4276\_Sample\_011046840, Unigene11443\_Sample\_011046840, Unigene10448\_Sample\_011046840, Unigene7089\_Sample\_011046840, Unigene14521\_Sample\_011046840, Unigene40309\_Sample\_011046840, Unigene30053\_Sample\_011046840, Unigene18953\_Sample\_011046840, Unigene41695\_Sample\_011046840, Unigene39582\_Sample\_011046840, Unigene16017\_Sample\_011046840, Unigene28188\_Sample\_011046840, Unigene42249\_Sample\_011046840, Unigene43217\_Sample\_011046840, Unigene22397\_Sample\_011046840, Unigene2237\_Sample\_011046840, Unigene42271\_Sample\_011046840, Unigene11994\_Sample\_011046840, Unigene31695\_Sample\_011046840, Unigene42116\_Sample\_011046840, Unigene11132\_Sample\_011046840, Unigene12950\_Sample\_011046840, Unigene8228\_Sample\_011046840, Unigene7856\_Sample\_011046840, Unigene42354\_Sample\_011046840, Unigene39818\_Sample\_011046840, Unigene36810\_Sample\_011046840, Unigene39399\_Sample\_011046840, Unigene43542\_Sample\_011046840, Unigene5443\_Sample\_011046840, Unigene38542\_Sample\_011046840, Unigene41337\_Sample\_011046840, Unigene27657\_Sample\_011046840, Unigene40404\_Sample\_011046840, Unigene29336\_Sample\_011046840, Unigene34874\_Sample\_011046840, Unigene35820\_Sample\_011046840, Unigene507\_Sample\_011046840, Unigene39628\_Sample\_011046840, Unigene8594\_Sample\_011046840, Unigene24129\_Sample\_011046840, Unigene31662\_Sample\_011046840, Unigene20272\_Sample\_011046840, Unigene39204\_Sample\_011046840, Unigene28265\_Sample\_011046840, Unigene12153\_Sample\_011046840, Unigene35008\_Sample\_011046840, Unigene36013\_Sample\_011046840, Unigene9006\_Sample\_011046840, Unigene38540\_Sample\_011046840, Unigene25307\_Sample\_011046840, Unigene36128\_Sample\_011046840, Unigene38354\_Sample\_011046840, Unigene43631\_Sample\_011046840, Unigene31373\_Sample\_011046840, Unigene39649\_Sample\_011046840, Unigene7935\_Sample\_011046840, Unigene2986\_Sample\_011046840, Unigene43486\_Sample\_011046840, Unigene27792\_Sample\_011046840, Unigene42666\_Sample\_011046840, Unigene11875\_Sample\_011046840, Unigene7825\_Sample\_011046840, Unigene40602\_Sample\_011046840, Unigene27152\_Sample\_011046840, Unigene23601\_Sample\_011046840, Unigene15570\_Sample\_011046840, Unigene15138\_Sample\_011046840, Unigene36025\_Sample\_011046840, Unigene19183\_Sample\_011046840, Unigene31646\_Sample\_011046840, Unigene35279\_Sample\_011046840, Unigene35252\_Sample\_011046840, Unigene29670\_Sample\_011046840, Unigene34909\_Sample\_011046840, Unigene4529\_Sample\_011046840, Unigene37829\_Sample\_011046840, Unigene41664\_Sample\_011046840, Unigene31732\_Sample\_011046840, Unigene39606\_Sample\_011046840, Unigene3153\_Sample\_011046840, Unigene42298\_Sample\_011046840, Unigene39674\_Sample\_011046840, Unigene17985\_Sample\_011046840, Unigene39633\_Sample\_011046840, Unigene42376\_Sample\_011046840, Unigene39748\_Sample\_011046840, Unigene16618\_Sample\_011046840, Unigene26435\_Sample\_011046840, Unigene41478\_Sample\_011046840, Unigene3258\_Sample\_011046840, Unigene5425\_Sample\_011046840, Unigene6039\_Sample\_011046840, Unigene40703\_Sample\_011046840, Unigene19931\_Sample\_011046840, Unigene7299\_Sample\_011046840, Unigene39226\_Sample\_011046840, Unigene43445\_Sample\_011046840, Unigene2524\_Sample\_011046840, Unigene36817\_Sample\_011046840, Unigene33539\_Sample\_011046840, Unigene41090\_Sample\_011046840, Unigene42509\_Sample\_011046840, Unigene3219\_Sample\_011046840, Unigene42756\_Sample\_011046840, Unigene40031\_Sample\_011046840, Unigene15184\_Sample\_011046840, Unigene5791\_Sample\_011046840, Unigene40452\_Sample\_011046840, Unigene41720\_Sample\_011046840, Unigene23472\_Sample\_011046840, Unigene43516\_Sample\_011046840, Unigene40814\_Sample\_011046840, Unigene7994\_Sample\_011046840, Unigene39346\_Sample\_011046840, Unigene41163\_Sample\_011046840, Unigene2665\_Sample\_011046840, Unigene43231\_Sample\_011046840, Unigene30367\_Sample\_011046840, Unigene38801\_Sample\_011046840, Unigene13661\_Sample\_011046840, Unigene39273\_Sample\_011046840, Unigene37919\_Sample\_011046840, Unigene43210\_Sample\_011046840, Unigene31306\_Sample\_011046840, Unigene37192\_Sample\_011046840, Unigene29808\_Sample\_011046840, Unigene8217\_Sample\_011046840, Unigene40496\_Sample\_011046840, Unigene11931\_Sample\_011046840, Unigene41811\_Sample\_011046840, Unigene29362\_Sample\_011046840, Unigene13726\_Sample\_011046840, Unigene27185\_Sample\_011046840, Unigene6925\_Sample\_011046840, Unigene38595\_Sample\_011046840, Unigene39615\_Sample\_011046840, Unigene17602\_Sample\_011046840, Unigene25467\_Sample\_011046840, Unigene32098\_Sample\_011046840, Unigene5385\_Sample\_011046840, Unigene42983\_Sample\_011046840, Unigene15428\_Sample\_011046840, Unigene40745\_Sample\_011046840, Unigene31705\_Sample\_011046840, Unigene35001\_Sample\_011046840, Unigene40260\_Sample\_011046840, Unigene41879\_Sample\_011046840, Unigene32783\_Sample\_011046840, Unigene32240\_Sample\_011046840, Unigene21551\_Sample\_011046840, Unigene5962\_Sample\_011046840, Unigene32573\_Sample\_011046840, Unigene34972\_Sample\_011046840, Unigene28616\_Sample\_011046840, Unigene33492\_Sample\_011046840, Unigene26448\_Sample\_011046840, Unigene37083\_Sample\_011046840, Unigene17486\_Sample\_011046840, Unigene43573\_Sample\_011046840, Unigene25450\_Sample\_011046840, Unigene7951\_Sample\_011046840, Unigene28129\_Sample\_011046840, Unigene7302\_Sample\_011046840, Unigene43120\_Sample\_011046840, Unigene39480\_Sample\_011046840, Unigene33745\_Sample\_011046840, Unigene3204\_Sample\_011046840, Unigene8085\_Sample\_011046840, Unigene6101\_Sample\_011046840, Unigene19985\_Sample\_011046840, Unigene38217\_Sample\_011046840, Unigene43342\_Sample\_011046840, Unigene19344\_Sample\_011046840, Unigene37484\_Sample\_011046840, Unigene43502\_Sample\_011046840, Unigene27982\_Sample\_011046840, Unigene31409\_Sample\_011046840, Unigene33338\_Sample\_011046840, Unigene12677\_Sample\_011046840, Unigene26172\_Sample\_011046840, Unigene43410\_Sample\_011046840, Unigene28710\_Sample\_011046840, Unigene33618\_Sample\_011046840, Unigene19933\_Sample\_011046840, Unigene34693\_Sample\_011046840, Unigene34288\_Sample\_011046840, Unigene31198\_Sample\_011046840, Unigene43301\_Sample\_011046840, Unigene26124\_Sample\_011046840, Unigene38940\_Sample\_011046840, Unigene21513\_Sample\_011046840, Unigene41283\_Sample\_011046840, Unigene37828\_Sample\_011046840, Unigene36734\_Sample\_011046840, Unigene40253\_Sample\_011046840, Unigene1368\_Sample\_011046840, Unigene42041\_Sample\_011046840, Unigene41981\_Sample\_011046840, Unigene43405\_Sample\_011046840, Unigene29875\_Sample\_011046840, Unigene1872\_Sample\_011046840, Unigene4145\_Sample\_011046840, Unigene23682\_Sample\_011046840, Unigene8198\_Sample\_011046840, Unigene40895\_Sample\_011046840, Unigene28610\_Sample\_011046840, Unigene40235\_Sample\_011046840, Unigene20869\_Sample\_011046840, Unigene39791\_Sample\_011046840, Unigene33873\_Sample\_011046840, Unigene40606\_Sample\_011046840, Unigene1379\_Sample\_011046840, Unigene41957\_Sample\_011046840, Unigene41383\_Sample\_011046840, Unigene26491\_Sample\_011046840, Unigene39843\_Sample\_011046840, Unigene3046\_Sample\_011046840, Unigene2709\_Sample\_011046840, Unigene37486\_Sample\_011046840, Unigene3212\_Sample\_011046840, Unigene3718\_Sample\_011046840, Unigene7203\_Sample\_011046840, Unigene13413\_Sample\_011046840, Unigene43289\_Sample\_011046840, Unigene36568\_Sample\_011046840, Unigene22606\_Sample\_011046840, Unigene39778\_Sample\_011046840, Unigene30720\_Sample\_011046840, Unigene14693\_Sample\_011046840, Unigene11201\_Sample\_011046840, Unigene15199\_Sample\_011046840, Unigene37213\_Sample\_011046840, Unigene7606\_Sample\_011046840, Unigene7813\_Sample\_011046840, Unigene332\_Sample\_011046840, Unigene7162\_Sample\_011046840, Unigene30599\_Sample\_011046840, Unigene36645\_Sample\_011046840, Unigene23950\_Sample\_011046840, Unigene36762\_Sample\_011046840, Unigene36732\_Sample\_011046840, Unigene41089\_Sample\_011046840, Unigene28034\_Sample\_011046840, Unigene24482\_Sample\_011046840, Unigene5166\_Sample\_011046840, Unigene20196\_Sample\_011046840, Unigene14589\_Sample\_011046840, Unigene19402\_Sample\_011046840, Unigene40547\_Sample\_011046840, Unigene39900\_Sample\_011046840, Unigene42906\_Sample\_011046840, Unigene16569\_Sample\_011046840, Unigene33705\_Sample\_011046840, Unigene36937\_Sample\_011046840, Unigene32067\_Sample\_011046840, Unigene6764\_Sample\_011046840, Unigene20216\_Sample\_011046840, Unigene36996\_Sample\_011046840, Unigene38250\_Sample\_011046840, Unigene33344\_Sample\_011046840, Unigene41584\_Sample\_011046840, Unigene27067\_Sample\_011046840, Unigene18456\_Sample\_011046840, Unigene37506\_Sample\_011046840, Unigene39509\_Sample\_011046840, Unigene38767\_Sample\_011046840, Unigene5223\_Sample\_011046840, Unigene41805\_Sample\_011046840, Unigene38826\_Sample\_011046840, Unigene36211\_Sample\_011046840, Unigene43372\_Sample\_011046840, Unigene40587\_Sample\_011046840, Unigene39481\_Sample\_011046840, Unigene32614\_Sample\_011046840, Unigene26668\_Sample\_011046840, Unigene37136\_Sample\_011046840, Unigene29542\_Sample\_011046840, Unigene13327\_Sample\_011046840, Unigene42278\_Sample\_011046840, Unigene14602\_Sample\_011046840, Unigene40732\_Sample\_011046840, Unigene28853\_Sample\_011046840, Unigene8413\_Sample\_011046840, Unigene17765\_Sample\_011046840, Unigene42337\_Sample\_011046840, Unigene35167\_Sample\_011046840, Unigene41299\_Sample\_011046840, Unigene6283\_Sample\_011046840, Unigene27639\_Sample\_011046840, Unigene41061\_Sample\_011046840, Unigene11146\_Sample\_011046840, Unigene30715\_Sample\_011046840, Unigene37236\_Sample\_011046840, Unigene32566\_Sample\_011046840, Unigene33878\_Sample\_011046840, Unigene34566\_Sample\_011046840, Unigene26402\_Sample\_011046840, Unigene43585\_Sample\_011046840, Unigene38285\_Sample\_011046840, Unigene14392\_Sample\_011046840, Unigene4139\_Sample\_011046840, Unigene36839\_Sample\_011046840, Unigene24400\_Sample\_011046840, Unigene36971\_Sample\_011046840, Unigene40950\_Sample\_011046840, Unigene31827\_Sample\_011046840, Unigene33088\_Sample\_011046840, Unigene36063\_Sample\_011046840, Unigene18251\_Sample\_011046840, Unigene15903\_Sample\_011046840, Unigene35023\_Sample\_011046840, Unigene42487\_Sample\_011046840, Unigene6842\_Sample\_011046840, Unigene32606\_Sample\_011046840, Unigene13197\_Sample\_011046840, Unigene5985\_Sample\_011046840, Unigene31283\_Sample\_011046840, Unigene41983\_Sample\_011046840, Unigene21387\_Sample\_011046840, Unigene27604\_Sample\_011046840, Unigene17650\_Sample\_011046840, Unigene38526\_Sample\_011046840, Unigene35976\_Sample\_011046840, Unigene40764\_Sample\_011046840, Unigene27667\_Sample\_011046840, Unigene19027\_Sample\_011046840, Unigene7353\_Sample\_011046840, Unigene32180\_Sample\_011046840, Unigene15237\_Sample\_011046840, Unigene40726\_Sample\_011046840, Unigene42638\_Sample\_011046840, Unigene19219\_Sample\_011046840, Unigene5707\_Sample\_011046840, Unigene17339\_Sample\_011046840, Unigene35227\_Sample\_011046840, Unigene38242\_Sample\_011046840, Unigene24546\_Sample\_011046840, Unigene25298\_Sample\_011046840, Unigene31759\_Sample\_011046840, Unigene12410\_Sample\_011046840, Unigene38772\_Sample\_011046840, Unigene37440\_Sample\_011046840, Unigene28079\_Sample\_011046840, Unigene10436\_Sample\_011046840, Unigene43064\_Sample\_011046840, Unigene40520\_Sample\_011046840, Unigene29405\_Sample\_011046840, Unigene32702\_Sample\_011046840, Unigene39983\_Sample\_011046840, Unigene19991\_Sample\_011046840, Unigene42622\_Sample\_011046840, Unigene41793\_Sample\_011046840, Unigene39948\_Sample\_011046840, Unigene5658\_Sample\_011046840, Unigene33671\_Sample\_011046840, Unigene42763\_Sample\_011046840, Unigene38378\_Sample\_011046840, Unigene6346\_Sample\_011046840, Unigene14981\_Sample\_011046840, Unigene3828\_Sample\_011046840, Unigene6395\_Sample\_011046840, Unigene2916\_Sample\_011046840, Unigene40716\_Sample\_011046840, Unigene29928\_Sample\_011046840, Unigene22209\_Sample\_011046840, Unigene11244\_Sample\_011046840, Unigene8189\_Sample\_011046840, Unigene40234\_Sample\_011046840, Unigene33379\_Sample\_011046840, Unigene37694\_Sample\_011046840, Unigene12204\_Sample\_011046840, Unigene19740\_Sample\_011046840, Unigene4042\_Sample\_011046840, Unigene39967\_Sample\_011046840, Unigene43198\_Sample\_011046840, Unigene41086\_Sample\_011046840, Unigene11516\_Sample\_011046840, Unigene31973\_Sample\_011046840, Unigene39052\_Sample\_011046840, Unigene38279\_Sample\_011046840, Unigene25915\_Sample\_011046840, Unigene7631\_Sample\_011046840, Unigene30400\_Sample\_011046840, Unigene41681\_Sample\_011046840, Unigene29592\_Sample\_011046840, Unigene3486\_Sample\_011046840, Unigene38262\_Sample\_011046840, Unigene34754\_Sample\_011046840, Unigene35053\_Sample\_011046840, Unigene42702\_Sample\_011046840, Unigene11883\_Sample\_011046840, Unigene42866\_Sample\_011046840, Unigene40010\_Sample\_011046840, Unigene37330\_Sample\_011046840, Unigene21296\_Sample\_011046840, Unigene7708\_Sample\_011046840, Unigene37921\_Sample\_011046840, Unigene20922\_Sample\_011046840, Unigene28301\_Sample\_011046840, Unigene41242\_Sample\_011046840, Unigene41510\_Sample\_011046840, Unigene27805\_Sample\_011046840, Unigene43165\_Sample\_011046840, Unigene32262\_Sample\_011046840, Unigene22393\_Sample\_011046840, Unigene16493\_Sample\_011046840, Unigene18168\_Sample\_011046840, Unigene11142\_Sample\_011046840, Unigene32307\_Sample\_011046840, Unigene42027\_Sample\_011046840, Unigene21554\_Sample\_011046840, Unigene8130\_Sample\_011046840, Unigene20146\_Sample\_011046840, Unigene7172\_Sample\_011046840, Unigene42096\_Sample\_011046840, Unigene5647\_Sample\_011046840, Unigene41869\_Sample\_011046840, Unigene19505\_Sample\_011046840, Unigene16302\_Sample\_011046840, Unigene36983\_Sample\_011046840, Unigene37827\_Sample\_011046840, Unigene24404\_Sample\_011046840, Unigene42692\_Sample\_011046840, Unigene40128\_Sample\_011046840, Unigene25020\_Sample\_011046840, Unigene43593\_Sample\_011046840, Unigene11571\_Sample\_011046840, Unigene28366\_Sample\_011046840, Unigene24019\_Sample\_011046840, Unigene7767\_Sample\_011046840, Unigene43201\_Sample\_011046840, Unigene41281\_Sample\_011046840, Unigene14437\_Sample\_011046840, Unigene7768\_Sample\_011046840, Unigene14257\_Sample\_011046840 |
[truncated: 2,219,278 more chars]
