# Supplementary material for: Identification of Dirofilaria immitis miRNA using illumina deep sequencing
Source: Vet Res. 2013 Jan 18;44(1):3. doi: 10.1186/1297-9716-44-3 (PMC3598945; doi:10.1186/1297-9716-44-3)
Supplement: Additional file 8 — The GO annotations on putative target genes (Biological process). 9 357 target genes were assigned to 2 235 Go-terms which belong to “Biological process” ontology. “Gene Ontology term” means GO terms with P-value as good or better than 1. “Cluster frequency” represents number and frequency of target genes related to this term. “Genome frequency of use” represents number and frequency of coding genes related to this term. [file 1297-9716-44-3-S8.zip › index.htm/Additional file 8. Biological process.html]

Terms for Dirofilaria\_immites\_P


## Terms for Dirofilaria\_immites\_P

---


### Result Table

|  |
| --- |
| **Terms from the Process Ontology with p-value as good or better than 1** |

| Gene Ontology term | Cluster frequency | Genome frequency of use | Corrected P-value |
| --- | --- | --- | --- |
| multicellular organismal process | 3733 out of 9357 genes, 39.9% | 4274 out of 10903 genes, 39.2% | 0.29426 |
| anatomical structure development | 2160 out of 9357 genes, 23.1% | 2465 out of 10903 genes, 22.6% | 1 |
| system development | 1440 out of 9357 genes, 15.4% | 1634 out of 10903 genes, 15.0% | 1 |
| tube development | 66 out of 9357 genes, 0.7% | 68 out of 10903 genes, 0.6% | 1 |
| cellular component organization at cellular level | 995 out of 9357 genes, 10.6% | 1123 out of 10903 genes, 10.3% | 1 |
| cellular component organization or biogenesis at cellular level | 1092 out of 9357 genes, 11.7% | 1235 out of 10903 genes, 11.3% | 1 |
| organ development | 1125 out of 9357 genes, 12.0% | 1273 out of 10903 genes, 11.7% | 1 |
| biological regulation | 3246 out of 9357 genes, 34.7% | 3726 out of 10903 genes, 34.2% | 1 |
| induction of apoptosis | 37 out of 9357 genes, 0.4% | 37 out of 10903 genes, 0.3% | 1 |
| regulation of macromolecule metabolic process | 889 out of 9357 genes, 9.5% | 1003 out of 10903 genes, 9.2% | 1 |
| reproduction | 1235 out of 9357 genes, 13.2% | 1402 out of 10903 genes, 12.9% | 1 |
| regulation of gene expression | 745 out of 9357 genes, 8.0% | 839 out of 10903 genes, 7.7% | 1 |
| developmental process | 3555 out of 9357 genes, 38.0% | 4089 out of 10903 genes, 37.5% | 1 |
| regulation of transcription from RNA polymerase II promoter | 142 out of 9357 genes, 1.5% | 153 out of 10903 genes, 1.4% | 1 |
| neurological system process | 313 out of 9357 genes, 3.3% | 346 out of 10903 genes, 3.2% | 1 |
| phosphate metabolic process | 260 out of 9357 genes, 2.8% | 286 out of 10903 genes, 2.6% | 1 |
| regulation of metabolic process | 1088 out of 9357 genes, 11.6% | 1234 out of 10903 genes, 11.3% | 1 |
| regulation of biological process | 2974 out of 9357 genes, 31.8% | 3416 out of 10903 genes, 31.3% | 1 |
| embryonic morphogenesis | 218 out of 9357 genes, 2.3% | 239 out of 10903 genes, 2.2% | 1 |
| anatomical structure morphogenesis | 1348 out of 9357 genes, 14.4% | 1535 out of 10903 genes, 14.1% | 1 |
| multicellular organismal development | 3262 out of 9357 genes, 34.9% | 3752 out of 10903 genes, 34.4% | 1 |
| regulation of transcription | 292 out of 9357 genes, 3.1% | 323 out of 10903 genes, 3.0% | 1 |
| phosphorus metabolic process | 262 out of 9357 genes, 2.8% | 289 out of 10903 genes, 2.7% | 1 |
| positive regulation of apoptosis | 44 out of 9357 genes, 0.5% | 45 out of 10903 genes, 0.4% | 1 |
| regulation of primary metabolic process | 496 out of 9357 genes, 5.3% | 556 out of 10903 genes, 5.1% | 1 |
| organ morphogenesis | 199 out of 9357 genes, 2.1% | 218 out of 10903 genes, 2.0% | 1 |
| tube morphogenesis | 54 out of 9357 genes, 0.6% | 56 out of 10903 genes, 0.5% | 1 |
| regulation of cellular macromolecule biosynthetic process | 322 out of 9357 genes, 3.4% | 358 out of 10903 genes, 3.3% | 1 |
| regulation of cellular biosynthetic process | 329 out of 9357 genes, 3.5% | 366 out of 10903 genes, 3.4% | 1 |
| tissue development | 578 out of 9357 genes, 6.2% | 651 out of 10903 genes, 6.0% | 1 |
| regulation of cellular metabolic process | 536 out of 9357 genes, 5.7% | 603 out of 10903 genes, 5.5% | 1 |
| system process | 402 out of 9357 genes, 4.3% | 450 out of 10903 genes, 4.1% | 1 |
| generation of a signal involved in cell-cell signaling | 39 out of 9357 genes, 0.4% | 40 out of 10903 genes, 0.4% | 1 |
| signal release | 39 out of 9357 genes, 0.4% | 40 out of 10903 genes, 0.4% | 1 |
| reproductive process | 1183 out of 9357 genes, 12.6% | 1349 out of 10903 genes, 12.4% | 1 |
| growth | 486 out of 9357 genes, 5.2% | 547 out of 10903 genes, 5.0% | 1 |
| regulation of developmental process | 149 out of 9357 genes, 1.6% | 163 out of 10903 genes, 1.5% | 1 |
| embryonic organ morphogenesis | 25 out of 9357 genes, 0.3% | 25 out of 10903 genes, 0.2% | 1 |
| body morphogenesis | 74 out of 9357 genes, 0.8% | 79 out of 10903 genes, 0.7% | 1 |
| regulation of transcription, DNA-dependent | 257 out of 9357 genes, 2.7% | 286 out of 10903 genes, 2.6% | 1 |
| cellular component organization or biogenesis | 2028 out of 9357 genes, 21.7% | 2329 out of 10903 genes, 21.4% | 1 |
| imaginal disc development | 90 out of 9357 genes, 1.0% | 97 out of 10903 genes, 0.9% | 1 |
| cell proliferation | 106 out of 9357 genes, 1.1% | 115 out of 10903 genes, 1.1% | 1 |
| regulation of multicellular organismal process | 190 out of 9357 genes, 2.0% | 210 out of 10903 genes, 1.9% | 1 |
| regulation of RNA metabolic process | 262 out of 9357 genes, 2.8% | 292 out of 10903 genes, 2.7% | 1 |
| organelle organization | 793 out of 9357 genes, 8.5% | 902 out of 10903 genes, 8.3% | 1 |
| regulation of mitosis | 34 out of 9357 genes, 0.4% | 35 out of 10903 genes, 0.3% | 1 |
| regulation of nuclear division | 34 out of 9357 genes, 0.4% | 35 out of 10903 genes, 0.3% | 1 |
| epithelial tube morphogenesis | 34 out of 9357 genes, 0.4% | 35 out of 10903 genes, 0.3% | 1 |
| cellular component organization | 1949 out of 9357 genes, 20.8% | 2239 out of 10903 genes, 20.5% | 1 |
| genitalia development | 580 out of 9357 genes, 6.2% | 657 out of 10903 genes, 6.0% | 1 |
| regulation of nucleobase, nucleoside, nucleotide and nucleic acid metabolic process | 384 out of 9357 genes, 4.1% | 432 out of 10903 genes, 4.0% | 1 |
| regulation of nitrogen compound metabolic process | 384 out of 9357 genes, 4.1% | 432 out of 10903 genes, 4.0% | 1 |
| cell differentiation | 699 out of 9357 genes, 7.5% | 794 out of 10903 genes, 7.3% | 1 |
| phosphorylation | 103 out of 9357 genes, 1.1% | 112 out of 10903 genes, 1.0% | 1 |
| sex differentiation | 651 out of 9357 genes, 7.0% | 739 out of 10903 genes, 6.8% | 1 |
| regulation of gene-specific transcription | 43 out of 9357 genes, 0.5% | 45 out of 10903 genes, 0.4% | 1 |
| establishment of spindle localization | 61 out of 9357 genes, 0.7% | 65 out of 10903 genes, 0.6% | 1 |
| spindle localization | 61 out of 9357 genes, 0.7% | 65 out of 10903 genes, 0.6% | 1 |
| eye morphogenesis | 52 out of 9357 genes, 0.6% | 55 out of 10903 genes, 0.5% | 1 |
| tissue morphogenesis | 422 out of 9357 genes, 4.5% | 476 out of 10903 genes, 4.4% | 1 |
| regulation of cellular process | 1335 out of 9357 genes, 14.3% | 1529 out of 10903 genes, 14.0% | 1 |
| regulation of cell differentiation | 109 out of 9357 genes, 1.2% | 119 out of 10903 genes, 1.1% | 1 |
| developmental process involved in reproduction | 844 out of 9357 genes, 9.0% | 962 out of 10903 genes, 8.8% | 1 |
| cell-cell signaling | 234 out of 9357 genes, 2.5% | 261 out of 10903 genes, 2.4% | 1 |
| morphogenesis of an epithelium | 101 out of 9357 genes, 1.1% | 110 out of 10903 genes, 1.0% | 1 |
| neurotransmitter transport | 42 out of 9357 genes, 0.4% | 44 out of 10903 genes, 0.4% | 1 |
| cell motility | 385 out of 9357 genes, 4.1% | 434 out of 10903 genes, 4.0% | 1 |
| localization of cell | 385 out of 9357 genes, 4.1% | 434 out of 10903 genes, 4.0% | 1 |
| response to stimulus | 1454 out of 9357 genes, 15.5% | 1668 out of 10903 genes, 15.3% | 1 |
| cell communication | 273 out of 9357 genes, 2.9% | 306 out of 10903 genes, 2.8% | 1 |
| cellular process involved in reproduction | 99 out of 9357 genes, 1.1% | 108 out of 10903 genes, 1.0% | 1 |
| pigmentation | 20 out of 9357 genes, 0.2% | 20 out of 10903 genes, 0.2% | 1 |
| establishment of mitotic spindle localization | 31 out of 9357 genes, 0.3% | 32 out of 10903 genes, 0.3% | 1 |
| localization | 2776 out of 9357 genes, 29.7% | 3202 out of 10903 genes, 29.4% | 1 |
| defense response | 49 out of 9357 genes, 0.5% | 52 out of 10903 genes, 0.5% | 1 |
| coenzyme biosynthetic process | 49 out of 9357 genes, 0.5% | 52 out of 10903 genes, 0.5% | 1 |
| cognition | 49 out of 9357 genes, 0.5% | 52 out of 10903 genes, 0.5% | 1 |
| epithelium development | 143 out of 9357 genes, 1.5% | 158 out of 10903 genes, 1.4% | 1 |
| protein phosphorylation | 90 out of 9357 genes, 1.0% | 98 out of 10903 genes, 0.9% | 1 |
| chromosome organization | 236 out of 9357 genes, 2.5% | 264 out of 10903 genes, 2.4% | 1 |
| neurotransmitter secretion | 30 out of 9357 genes, 0.3% | 31 out of 10903 genes, 0.3% | 1 |
| regulation of Notch signaling pathway | 30 out of 9357 genes, 0.3% | 31 out of 10903 genes, 0.3% | 1 |
| post-embryonic morphogenesis | 97 out of 9357 genes, 1.0% | 106 out of 10903 genes, 1.0% | 1 |
| learning or memory | 48 out of 9357 genes, 0.5% | 51 out of 10903 genes, 0.5% | 1 |
| embryo development | 1953 out of 9357 genes, 20.9% | 2248 out of 10903 genes, 20.6% | 1 |
| regulation of vesicle-mediated transport | 39 out of 9357 genes, 0.4% | 41 out of 10903 genes, 0.4% | 1 |
| regulation of macromolecule biosynthetic process | 350 out of 9357 genes, 3.7% | 395 out of 10903 genes, 3.6% | 1 |
| eye development | 72 out of 9357 genes, 0.8% | 78 out of 10903 genes, 0.7% | 1 |
| lipid localization | 294 out of 9357 genes, 3.1% | 331 out of 10903 genes, 3.0% | 1 |
| chromatin modification | 95 out of 9357 genes, 1.0% | 104 out of 10903 genes, 1.0% | 1 |
| multicellular organism growth | 348 out of 9357 genes, 3.7% | 393 out of 10903 genes, 3.6% | 1 |
| regulation of hormone levels | 18 out of 9357 genes, 0.2% | 18 out of 10903 genes, 0.2% | 1 |
| cellular component movement | 388 out of 9357 genes, 4.1% | 439 out of 10903 genes, 4.0% | 1 |
| regulation of synaptic transmission | 55 out of 9357 genes, 0.6% | 59 out of 10903 genes, 0.5% | 1 |
| cell development | 474 out of 9357 genes, 5.1% | 538 out of 10903 genes, 4.9% | 1 |
| cellular process | 6375 out of 9357 genes, 68.1% | 7398 out of 10903 genes, 67.9% | 1 |
| cellular developmental process | 785 out of 9357 genes, 8.4% | 897 out of 10903 genes, 8.2% | 1 |
| secretion by cell | 54 out of 9357 genes, 0.6% | 58 out of 10903 genes, 0.5% | 1 |
| extracellular structure organization | 54 out of 9357 genes, 0.6% | 58 out of 10903 genes, 0.5% | 1 |
| instar larval or pupal development | 70 out of 9357 genes, 0.7% | 76 out of 10903 genes, 0.7% | 1 |
| regulation of cell cycle process | 70 out of 9357 genes, 0.7% | 76 out of 10903 genes, 0.7% | 1 |
| positive regulation of cell death | 70 out of 9357 genes, 0.7% | 76 out of 10903 genes, 0.7% | 1 |
| positive regulation of programmed cell death | 70 out of 9357 genes, 0.7% | 76 out of 10903 genes, 0.7% | 1 |
| induction of programmed cell death | 62 out of 9357 genes, 0.7% | 67 out of 10903 genes, 0.6% | 1 |
| cellular response to stress | 249 out of 9357 genes, 2.7% | 280 out of 10903 genes, 2.6% | 1 |
| skeletal system development | 17 out of 9357 genes, 0.2% | 17 out of 10903 genes, 0.2% | 1 |
| cotranslational protein targeting to membrane | 17 out of 9357 genes, 0.2% | 17 out of 10903 genes, 0.2% | 1 |
| signal transmission via phosphorylation event | 100 out of 9357 genes, 1.1% | 110 out of 10903 genes, 1.0% | 1 |
| ameboidal cell migration | 45 out of 9357 genes, 0.5% | 48 out of 10903 genes, 0.4% | 1 |
| regulation of transport | 107 out of 9357 genes, 1.1% | 118 out of 10903 genes, 1.1% | 1 |
| regulation of endocytosis | 27 out of 9357 genes, 0.3% | 28 out of 10903 genes, 0.3% | 1 |
| regulation of neurological system process | 61 out of 9357 genes, 0.7% | 66 out of 10903 genes, 0.6% | 1 |
| appendage morphogenesis | 61 out of 9357 genes, 0.7% | 66 out of 10903 genes, 0.6% | 1 |
| peptidyl-amino acid modification | 36 out of 9357 genes, 0.4% | 38 out of 10903 genes, 0.3% | 1 |
| central nervous system development | 121 out of 9357 genes, 1.3% | 134 out of 10903 genes, 1.2% | 1 |
| cofactor biosynthetic process | 76 out of 9357 genes, 0.8% | 83 out of 10903 genes, 0.8% | 1 |
| muscle tissue development | 44 out of 9357 genes, 0.5% | 47 out of 10903 genes, 0.4% | 1 |
| germ cell development | 68 out of 9357 genes, 0.7% | 74 out of 10903 genes, 0.7% | 1 |
| regulation of transmission of nerve impulse | 60 out of 9357 genes, 0.6% | 65 out of 10903 genes, 0.6% | 1 |
| sensory organ development | 98 out of 9357 genes, 1.0% | 108 out of 10903 genes, 1.0% | 1 |
| induction of apoptosis by intracellular signals | 16 out of 9357 genes, 0.2% | 16 out of 10903 genes, 0.1% | 1 |
| tube formation | 16 out of 9357 genes, 0.2% | 16 out of 10903 genes, 0.1% | 1 |
| regulation of gene-specific transcription from RNA polymerase II promoter | 26 out of 9357 genes, 0.3% | 27 out of 10903 genes, 0.2% | 1 |
| cellular component disassembly | 26 out of 9357 genes, 0.3% | 27 out of 10903 genes, 0.2% | 1 |
| regulation of muscle organ development | 26 out of 9357 genes, 0.3% | 27 out of 10903 genes, 0.2% | 1 |
| microtubule cytoskeleton organization | 141 out of 9357 genes, 1.5% | 157 out of 10903 genes, 1.4% | 1 |
| locomotion | 685 out of 9357 genes, 7.3% | 783 out of 10903 genes, 7.2% | 1 |
| metamorphosis | 67 out of 9357 genes, 0.7% | 73 out of 10903 genes, 0.7% | 1 |
| instar larval or pupal morphogenesis | 67 out of 9357 genes, 0.7% | 73 out of 10903 genes, 0.7% | 1 |
| appendage development | 67 out of 9357 genes, 0.7% | 73 out of 10903 genes, 0.7% | 1 |
| regulation of biosynthetic process | 358 out of 9357 genes, 3.8% | 406 out of 10903 genes, 3.7% | 1 |
| striated muscle tissue development | 43 out of 9357 genes, 0.5% | 46 out of 10903 genes, 0.4% | 1 |
| anatomical structure formation involved in morphogenesis | 196 out of 9357 genes, 2.1% | 220 out of 10903 genes, 2.0% | 1 |
| multicellular organism reproduction | 548 out of 9357 genes, 5.9% | 625 out of 10903 genes, 5.7% | 1 |
| multicellular organismal reproductive process | 548 out of 9357 genes, 5.9% | 625 out of 10903 genes, 5.7% | 1 |
| positive regulation of catalytic activity | 59 out of 9357 genes, 0.6% | 64 out of 10903 genes, 0.6% | 1 |
| regulation of cell communication | 118 out of 9357 genes, 1.3% | 131 out of 10903 genes, 1.2% | 1 |
| transmission of nerve impulse | 195 out of 9357 genes, 2.1% | 219 out of 10903 genes, 2.0% | 1 |
| DNA recombination | 34 out of 9357 genes, 0.4% | 36 out of 10903 genes, 0.3% | 1 |
| imaginal disc morphogenesis | 66 out of 9357 genes, 0.7% | 72 out of 10903 genes, 0.7% | 1 |
| post-embryonic organ morphogenesis | 66 out of 9357 genes, 0.7% | 72 out of 10903 genes, 0.7% | 1 |
| morphogenesis of a branching structure | 25 out of 9357 genes, 0.3% | 26 out of 10903 genes, 0.2% | 1 |
| cellular component disassembly at cellular level | 25 out of 9357 genes, 0.3% | 26 out of 10903 genes, 0.2% | 1 |
| nervous system development | 487 out of 9357 genes, 5.2% | 555 out of 10903 genes, 5.1% | 1 |
| urogenital system development | 15 out of 9357 genes, 0.2% | 15 out of 10903 genes, 0.1% | 1 |
| internal protein amino acid acetylation | 15 out of 9357 genes, 0.2% | 15 out of 10903 genes, 0.1% | 1 |
| peptide transport | 15 out of 9357 genes, 0.2% | 15 out of 10903 genes, 0.1% | 1 |
| histone acetylation | 15 out of 9357 genes, 0.2% | 15 out of 10903 genes, 0.1% | 1 |
| internal peptidyl-lysine acetylation | 15 out of 9357 genes, 0.2% | 15 out of 10903 genes, 0.1% | 1 |
| peptidyl-lysine acetylation | 15 out of 9357 genes, 0.2% | 15 out of 10903 genes, 0.1% | 1 |
| intracellular protein kinase cascade | 80 out of 9357 genes, 0.9% | 88 out of 10903 genes, 0.8% | 1 |
| regulation of multicellular organismal development | 80 out of 9357 genes, 0.9% | 88 out of 10903 genes, 0.8% | 1 |
| synaptic transmission | 179 out of 9357 genes, 1.9% | 201 out of 10903 genes, 1.8% | 1 |
| cell junction organization | 57 out of 9357 genes, 0.6% | 62 out of 10903 genes, 0.6% | 1 |
| cellular response to stimulus | 273 out of 9357 genes, 2.9% | 309 out of 10903 genes, 2.8% | 1 |
| cell recognition | 24 out of 9357 genes, 0.3% | 25 out of 10903 genes, 0.2% | 1 |
| macromolecular complex disassembly | 24 out of 9357 genes, 0.3% | 25 out of 10903 genes, 0.2% | 1 |
| chemotaxis | 157 out of 9357 genes, 1.7% | 176 out of 10903 genes, 1.6% | 1 |
| response to chemical stimulus | 495 out of 9357 genes, 5.3% | 565 out of 10903 genes, 5.2% | 1 |
| embryonic heart tube morphogenesis | 14 out of 9357 genes, 0.1% | 14 out of 10903 genes, 0.1% | 1 |
| extracellular matrix organization | 14 out of 9357 genes, 0.1% | 14 out of 10903 genes, 0.1% | 1 |
| embryonic heart tube development | 14 out of 9357 genes, 0.1% | 14 out of 10903 genes, 0.1% | 1 |
| immune system development | 56 out of 9357 genes, 0.6% | 61 out of 10903 genes, 0.6% | 1 |
| sensory perception | 71 out of 9357 genes, 0.8% | 78 out of 10903 genes, 0.7% | 1 |
| chromatin organization | 163 out of 9357 genes, 1.7% | 183 out of 10903 genes, 1.7% | 1 |
| response to DNA damage stimulus | 135 out of 9357 genes, 1.4% | 151 out of 10903 genes, 1.4% | 1 |
| cell junction assembly | 40 out of 9357 genes, 0.4% | 43 out of 10903 genes, 0.4% | 1 |
| tRNA metabolic process | 176 out of 9357 genes, 1.9% | 198 out of 10903 genes, 1.8% | 1 |
| epithelial cell development | 23 out of 9357 genes, 0.2% | 24 out of 10903 genes, 0.2% | 1 |
| germarium-derived egg chamber formation | 23 out of 9357 genes, 0.2% | 24 out of 10903 genes, 0.2% | 1 |
| sensory perception of chemical stimulus | 23 out of 9357 genes, 0.2% | 24 out of 10903 genes, 0.2% | 1 |
| cellular macromolecular complex disassembly | 23 out of 9357 genes, 0.2% | 24 out of 10903 genes, 0.2% | 1 |
| membrane organization | 780 out of 9357 genes, 8.3% | 895 out of 10903 genes, 8.2% | 1 |
| ubiquitin-dependent protein catabolic process | 70 out of 9357 genes, 0.7% | 77 out of 10903 genes, 0.7% | 1 |
| RNA splicing, via transesterification reactions | 134 out of 9357 genes, 1.4% | 150 out of 10903 genes, 1.4% | 1 |
| RNA splicing, via transesterification reactions with bulged adenosine as nucleophile | 134 out of 9357 genes, 1.4% | 150 out of 10903 genes, 1.4% | 1 |
| cellular membrane organization | 779 out of 9357 genes, 8.3% | 894 out of 10903 genes, 8.2% | 1 |
| sexual reproduction | 282 out of 9357 genes, 3.0% | 320 out of 10903 genes, 2.9% | 1 |
| cell fate specification | 47 out of 9357 genes, 0.5% | 51 out of 10903 genes, 0.5% | 1 |
| regulation of immune system process | 31 out of 9357 genes, 0.3% | 33 out of 10903 genes, 0.3% | 1 |
| cell fate commitment | 119 out of 9357 genes, 1.3% | 133 out of 10903 genes, 1.2% | 1 |
| response to nutrient | 13 out of 9357 genes, 0.1% | 13 out of 10903 genes, 0.1% | 1 |
| dorsal/ventral pattern formation | 13 out of 9357 genes, 0.1% | 13 out of 10903 genes, 0.1% | 1 |
| respiratory tube development | 13 out of 9357 genes, 0.1% | 13 out of 10903 genes, 0.1% | 1 |
| cellular pigmentation | 13 out of 9357 genes, 0.1% | 13 out of 10903 genes, 0.1% | 1 |
| regulation of hormone secretion | 13 out of 9357 genes, 0.1% | 13 out of 10903 genes, 0.1% | 1 |
| cellular amino acid metabolic process | 200 out of 9357 genes, 2.1% | 226 out of 10903 genes, 2.1% | 1 |
| regulation of cellular component organization | 173 out of 9357 genes, 1.8% | 195 out of 10903 genes, 1.8% | 1 |
| endocytosis | 724 out of 9357 genes, 7.7% | 831 out of 10903 genes, 7.6% | 1 |
| membrane invagination | 724 out of 9357 genes, 7.7% | 831 out of 10903 genes, 7.6% | 1 |
| regulation of striated muscle tissue development | 22 out of 9357 genes, 0.2% | 23 out of 10903 genes, 0.2% | 1 |
| peptidyl-lysine modification | 22 out of 9357 genes, 0.2% | 23 out of 10903 genes, 0.2% | 1 |
| limb morphogenesis | 22 out of 9357 genes, 0.2% | 23 out of 10903 genes, 0.2% | 1 |
| protein complex disassembly | 22 out of 9357 genes, 0.2% | 23 out of 10903 genes, 0.2% | 1 |
| regulation of developmental growth | 22 out of 9357 genes, 0.2% | 23 out of 10903 genes, 0.2% | 1 |
| muscle fiber development | 22 out of 9357 genes, 0.2% | 23 out of 10903 genes, 0.2% | 1 |
| regulation of synapse structure and activity | 22 out of 9357 genes, 0.2% | 23 out of 10903 genes, 0.2% | 1 |
| regulation of synapse organization | 22 out of 9357 genes, 0.2% | 23 out of 10903 genes, 0.2% | 1 |
| vesicle localization | 22 out of 9357 genes, 0.2% | 23 out of 10903 genes, 0.2% | 1 |
| regulation of peptidase activity | 22 out of 9357 genes, 0.2% | 23 out of 10903 genes, 0.2% | 1 |
| limb development | 22 out of 9357 genes, 0.2% | 23 out of 10903 genes, 0.2% | 1 |
| behavior | 539 out of 9357 genes, 5.8% | 617 out of 10903 genes, 5.7% | 1 |
| cell migration | 332 out of 9357 genes, 3.5% | 378 out of 10903 genes, 3.5% | 1 |
| transition metal ion transport | 38 out of 9357 genes, 0.4% | 41 out of 10903 genes, 0.4% | 1 |
| cellular component assembly at cellular level | 232 out of 9357 genes, 2.5% | 263 out of 10903 genes, 2.4% | 1 |
| taxis | 165 out of 9357 genes, 1.8% | 186 out of 10903 genes, 1.7% | 1 |
| striated muscle cell development | 53 out of 9357 genes, 0.6% | 58 out of 10903 genes, 0.5% | 1 |
| post-embryonic development | 1641 out of 9357 genes, 17.5% | 1895 out of 10903 genes, 17.4% | 1 |
| regulation of protein metabolic process | 96 out of 9357 genes, 1.0% | 107 out of 10903 genes, 1.0% | 1 |
| wing disc morphogenesis | 60 out of 9357 genes, 0.6% | 66 out of 10903 genes, 0.6% | 1 |
| wing disc development | 60 out of 9357 genes, 0.6% | 66 out of 10903 genes, 0.6% | 1 |
| gamete generation | 264 out of 9357 genes, 2.8% | 300 out of 10903 genes, 2.8% | 1 |
| embryonic epithelial tube formation | 12 out of 9357 genes, 0.1% | 12 out of 10903 genes, 0.1% | 1 |
| regulation of peptide secretion | 12 out of 9357 genes, 0.1% | 12 out of 10903 genes, 0.1% | 1 |
| synaptic transmission, cholinergic | 12 out of 9357 genes, 0.1% | 12 out of 10903 genes, 0.1% | 1 |
| regulation of cell morphogenesis involved in differentiation | 12 out of 9357 genes, 0.1% | 12 out of 10903 genes, 0.1% | 1 |
| stem cell maintenance | 12 out of 9357 genes, 0.1% | 12 out of 10903 genes, 0.1% | 1 |
| regulation of cell morphogenesis | 12 out of 9357 genes, 0.1% | 12 out of 10903 genes, 0.1% | 1 |
| fin development | 12 out of 9357 genes, 0.1% | 12 out of 10903 genes, 0.1% | 1 |
| stem cell development | 12 out of 9357 genes, 0.1% | 12 out of 10903 genes, 0.1% | 1 |
| DNA conformation change | 12 out of 9357 genes, 0.1% | 12 out of 10903 genes, 0.1% | 1 |
| epithelial tube formation | 12 out of 9357 genes, 0.1% | 12 out of 10903 genes, 0.1% | 1 |
| regulation of peptide transport | 12 out of 9357 genes, 0.1% | 12 out of 10903 genes, 0.1% | 1 |
| regulation of peptide hormone secretion | 12 out of 9357 genes, 0.1% | 12 out of 10903 genes, 0.1% | 1 |
| neuron recognition | 21 out of 9357 genes, 0.2% | 22 out of 10903 genes, 0.2% | 1 |
| cellular protein complex disassembly | 21 out of 9357 genes, 0.2% | 22 out of 10903 genes, 0.2% | 1 |
| branching morphogenesis of a tube | 21 out of 9357 genes, 0.2% | 22 out of 10903 genes, 0.2% | 1 |
| establishment of vesicle localization | 21 out of 9357 genes, 0.2% | 22 out of 10903 genes, 0.2% | 1 |
| regulation of system process | 81 out of 9357 genes, 0.9% | 90 out of 10903 genes, 0.8% | 1 |
| cellular ion homeostasis | 102 out of 9357 genes, 1.1% | 114 out of 10903 genes, 1.0% | 1 |
| cellular amine metabolic process | 230 out of 9357 genes, 2.5% | 261 out of 10903 genes, 2.4% | 1 |
| alternative nuclear mRNA splicing, via spliceosome | 29 out of 9357 genes, 0.3% | 31 out of 10903 genes, 0.3% | 1 |
| morphogenesis of embryonic epithelium | 29 out of 9357 genes, 0.3% | 31 out of 10903 genes, 0.3% | 1 |
| embryonic organ development | 29 out of 9357 genes, 0.3% | 31 out of 10903 genes, 0.3% | 1 |
| cell surface receptor linked signaling pathway | 437 out of 9357 genes, 4.7% | 500 out of 10903 genes, 4.6% | 1 |
| metal ion transport | 216 out of 9357 genes, 2.3% | 245 out of 10903 genes, 2.2% | 1 |
| Golgi vesicle transport | 66 out of 9357 genes, 0.7% | 73 out of 10903 genes, 0.7% | 1 |
| amino acid activation | 108 out of 9357 genes, 1.2% | 121 out of 10903 genes, 1.1% | 1 |
| tRNA aminoacylation | 108 out of 9357 genes, 1.2% | 121 out of 10903 genes, 1.1% | 1 |
| chemical homeostasis | 162 out of 9357 genes, 1.7% | 183 out of 10903 genes, 1.7% | 1 |
| protein modification process | 1098 out of 9357 genes, 11.7% | 1266 out of 10903 genes, 11.6% | 1 |
| cytoskeleton organization | 345 out of 9357 genes, 3.7% | 394 out of 10903 genes, 3.6% | 1 |
| nuclear division | 51 out of 9357 genes, 0.5% | 56 out of 10903 genes, 0.5% | 1 |
| mitosis | 51 out of 9357 genes, 0.5% | 56 out of 10903 genes, 0.5% | 1 |
| gastrulation | 121 out of 9357 genes, 1.3% | 136 out of 10903 genes, 1.2% | 1 |
| cellular protein metabolic process | 1656 out of 9357 genes, 17.7% | 1914 out of 10903 genes, 17.6% | 1 |
| tRNA aminoacylation for protein translation | 107 out of 9357 genes, 1.1% | 120 out of 10903 genes, 1.1% | 1 |
| skeletal muscle tissue development | 20 out of 9357 genes, 0.2% | 21 out of 10903 genes, 0.2% | 1 |
| regulation of skeletal muscle tissue development | 20 out of 9357 genes, 0.2% | 21 out of 10903 genes, 0.2% | 1 |
| skeletal muscle fiber development | 20 out of 9357 genes, 0.2% | 21 out of 10903 genes, 0.2% | 1 |
| regulation of skeletal muscle fiber development | 20 out of 9357 genes, 0.2% | 21 out of 10903 genes, 0.2% | 1 |
| negative regulation of transport | 20 out of 9357 genes, 0.2% | 21 out of 10903 genes, 0.2% | 1 |
| regulation of muscle cell differentiation | 20 out of 9357 genes, 0.2% | 21 out of 10903 genes, 0.2% | 1 |
| regulation of striated muscle cell differentiation | 20 out of 9357 genes, 0.2% | 21 out of 10903 genes, 0.2% | 1 |
| skeletal muscle organ development | 20 out of 9357 genes, 0.2% | 21 out of 10903 genes, 0.2% | 1 |
| regulation of protein phosphorylation | 28 out of 9357 genes, 0.3% | 30 out of 10903 genes, 0.3% | 1 |
| brain development | 79 out of 9357 genes, 0.8% | 88 out of 10903 genes, 0.8% | 1 |
| microtubule-based process | 220 out of 9357 genes, 2.4% | 250 out of 10903 genes, 2.3% | 1 |
| macromolecule modification | 1213 out of 9357 genes, 13.0% | 1400 out of 10903 genes, 12.8% | 1 |
| regulation of localization | 120 out of 9357 genes, 1.3% | 135 out of 10903 genes, 1.2% | 1 |
| Wnt receptor signaling pathway | 43 out of 9357 genes, 0.5% | 47 out of 10903 genes, 0.4% | 1 |
| hemopoietic or lymphoid organ development | 43 out of 9357 genes, 0.5% | 47 out of 10903 genes, 0.4% | 1 |
| neuron development | 259 out of 9357 genes, 2.8% | 295 out of 10903 genes, 2.7% | 1 |
| intracellular receptor mediated signaling pathway | 11 out of 9357 genes, 0.1% | 11 out of 10903 genes, 0.1% | 1 |
| response to vitamin | 11 out of 9357 genes, 0.1% | 11 out of 10903 genes, 0.1% | 1 |
| neuron fate commitment | 11 out of 9357 genes, 0.1% | 11 out of 10903 genes, 0.1% | 1 |
| regulation of insulin secretion | 11 out of 9357 genes, 0.1% | 11 out of 10903 genes, 0.1% | 1 |
| pigment granule localization | 11 out of 9357 genes, 0.1% | 11 out of 10903 genes, 0.1% | 1 |
| establishment of pigment granule localization | 11 out of 9357 genes, 0.1% | 11 out of 10903 genes, 0.1% | 1 |
| signaling pathway | 843 out of 9357 genes, 9.0% | 971 out of 10903 genes, 8.9% | 1 |
| proteasomal protein catabolic process | 50 out of 9357 genes, 0.5% | 55 out of 10903 genes, 0.5% | 1 |
| generation of neurons | 329 out of 9357 genes, 3.5% | 376 out of 10903 genes, 3.4% | 1 |
| positive regulation of cellular process | 199 out of 9357 genes, 2.1% | 226 out of 10903 genes, 2.1% | 1 |
| response to biotic stimulus | 64 out of 9357 genes, 0.7% | 71 out of 10903 genes, 0.7% | 1 |
| G-protein coupled receptor protein signaling pathway | 78 out of 9357 genes, 0.8% | 87 out of 10903 genes, 0.8% | 1 |
| neurogenesis | 354 out of 9357 genes, 3.8% | 405 out of 10903 genes, 3.7% | 1 |
| cell morphogenesis involved in neuron differentiation | 205 out of 9357 genes, 2.2% | 233 out of 10903 genes, 2.1% | 1 |
| cellular localization | 456 out of 9357 genes, 4.9% | 523 out of 10903 genes, 4.8% | 1 |
| mRNA processing | 91 out of 9357 genes, 1.0% | 102 out of 10903 genes, 0.9% | 1 |
| protein acetylation | 19 out of 9357 genes, 0.2% | 20 out of 10903 genes, 0.2% | 1 |
| glial cell differentiation | 19 out of 9357 genes, 0.2% | 20 out of 10903 genes, 0.2% | 1 |
| defense response to bacterium | 19 out of 9357 genes, 0.2% | 20 out of 10903 genes, 0.2% | 1 |
| protein acylation | 19 out of 9357 genes, 0.2% | 20 out of 10903 genes, 0.2% | 1 |
| covalent chromatin modification | 56 out of 9357 genes, 0.6% | 62 out of 10903 genes, 0.6% | 1 |
| histone modification | 56 out of 9357 genes, 0.6% | 62 out of 10903 genes, 0.6% | 1 |
| striated muscle cell differentiation | 56 out of 9357 genes, 0.6% | 62 out of 10903 genes, 0.6% | 1 |
| DNA repair | 111 out of 9357 genes, 1.2% | 125 out of 10903 genes, 1.1% | 1 |
| muscle cell differentiation | 63 out of 9357 genes, 0.7% | 70 out of 10903 genes, 0.6% | 1 |
| regulation of ligase activity | 34 out of 9357 genes, 0.4% | 37 out of 10903 genes, 0.3% | 1 |
| regulation of ubiquitin-protein ligase activity | 34 out of 9357 genes, 0.4% | 37 out of 10903 genes, 0.3% | 1 |
| amine metabolic process | 261 out of 9357 genes, 2.8% | 298 out of 10903 genes, 2.7% | 1 |
| establishment of localization in cell | 421 out of 9357 genes, 4.5% | 483 out of 10903 genes, 4.4% | 1 |
| neuron differentiation | 293 out of 9357 genes, 3.1% | 335 out of 10903 genes, 3.1% | 1 |
| kidney development | 10 out of 9357 genes, 0.1% | 10 out of 10903 genes, 0.1% | 1 |
| adaptive immune response | 10 out of 9357 genes, 0.1% | 10 out of 10903 genes, 0.1% | 1 |
| signal complex assembly | 10 out of 9357 genes, 0.1% | 10 out of 10903 genes, 0.1% | 1 |
| imaginal disc pattern formation | 10 out of 9357 genes, 0.1% | 10 out of 10903 genes, 0.1% | 1 |
| aspartate family amino acid metabolic process | 10 out of 9357 genes, 0.1% | 10 out of 10903 genes, 0.1% | 1 |
| hormone transport | 10 out of 9357 genes, 0.1% | 10 out of 10903 genes, 0.1% | 1 |
| negative regulation of cell cycle process | 10 out of 9357 genes, 0.1% | 10 out of 10903 genes, 0.1% | 1 |
| melanosome localization | 10 out of 9357 genes, 0.1% | 10 out of 10903 genes, 0.1% | 1 |
| establishment of melanosome localization | 10 out of 9357 genes, 0.1% | 10 out of 10903 genes, 0.1% | 1 |
| maintenance of protein location in cell | 10 out of 9357 genes, 0.1% | 10 out of 10903 genes, 0.1% | 1 |
| positive regulation of protein kinase activity | 10 out of 9357 genes, 0.1% | 10 out of 10903 genes, 0.1% | 1 |
| photoreceptor cell fate commitment | 10 out of 9357 genes, 0.1% | 10 out of 10903 genes, 0.1% | 1 |
| renal system development | 10 out of 9357 genes, 0.1% | 10 out of 10903 genes, 0.1% | 1 |
| cell morphogenesis involved in differentiation | 228 out of 9357 genes, 2.4% | 260 out of 10903 genes, 2.4% | 1 |
| proteasomal ubiquitin-dependent protein catabolic process | 48 out of 9357 genes, 0.5% | 53 out of 10903 genes, 0.5% | 1 |
| organelle fission | 55 out of 9357 genes, 0.6% | 61 out of 10903 genes, 0.6% | 1 |
| mRNA metabolic process | 123 out of 9357 genes, 1.3% | 139 out of 10903 genes, 1.3% | 1 |
| ion homeostasis | 123 out of 9357 genes, 1.3% | 139 out of 10903 genes, 1.3% | 1 |
| compound eye morphogenesis | 26 out of 9357 genes, 0.3% | 28 out of 10903 genes, 0.3% | 1 |
| cell activation | 26 out of 9357 genes, 0.3% | 28 out of 10903 genes, 0.3% | 1 |
| gliogenesis | 26 out of 9357 genes, 0.3% | 28 out of 10903 genes, 0.3% | 1 |
| leukocyte activation | 26 out of 9357 genes, 0.3% | 28 out of 10903 genes, 0.3% | 1 |
| lymphocyte activation | 26 out of 9357 genes, 0.3% | 28 out of 10903 genes, 0.3% | 1 |
| compound eye development | 26 out of 9357 genes, 0.3% | 28 out of 10903 genes, 0.3% | 1 |
| cell cycle process | 432 out of 9357 genes, 4.6% | 496 out of 10903 genes, 4.5% | 1 |
| cellular component morphogenesis | 362 out of 9357 genes, 3.9% | 415 out of 10903 genes, 3.8% | 1 |
| morphogenesis of a polarized epithelium | 18 out of 9357 genes, 0.2% | 19 out of 10903 genes, 0.2% | 1 |
| regulation of synaptic growth at neuromuscular junction | 18 out of 9357 genes, 0.2% | 19 out of 10903 genes, 0.2% | 1 |
| positive regulation of transferase activity | 18 out of 9357 genes, 0.2% | 19 out of 10903 genes, 0.2% | 1 |
| cell cycle switching, mitotic to meiotic cell cycle | 18 out of 9357 genes, 0.2% | 19 out of 10903 genes, 0.2% | 1 |
| regulation of synaptogenesis | 18 out of 9357 genes, 0.2% | 19 out of 10903 genes, 0.2% | 1 |
| cell cycle switching | 18 out of 9357 genes, 0.2% | 19 out of 10903 genes, 0.2% | 1 |
| protein metabolic process | 2001 out of 9357 genes, 21.4% | 2318 out of 10903 genes, 21.3% | 1 |
| learning | 33 out of 9357 genes, 0.4% | 36 out of 10903 genes, 0.3% | 1 |
| cellular di-, tri-valent inorganic cation homeostasis | 33 out of 9357 genes, 0.4% | 36 out of 10903 genes, 0.3% | 1 |
| negative regulation of molecular function | 33 out of 9357 genes, 0.4% | 36 out of 10903 genes, 0.3% | 1 |
| cell-cell junction organization | 33 out of 9357 genes, 0.4% | 36 out of 10903 genes, 0.3% | 1 |
| regulation of ubiquitin-protein ligase activity involved in mitotic cell cycle | 33 out of 9357 genes, 0.4% | 36 out of 10903 genes, 0.3% | 1 |
| female gamete generation | 155 out of 9357 genes, 1.7% | 176 out of 10903 genes, 1.6% | 1 |
| muscle structure development | 75 out of 9357 genes, 0.8% | 84 out of 10903 genes, 0.8% | 1 |
| regulation of apoptosis | 102 out of 9357 genes, 1.1% | 115 out of 10903 genes, 1.1% | 1 |
| cellular chemical homeostasis | 102 out of 9357 genes, 1.1% | 115 out of 10903 genes, 1.1% | 1 |
| regulation of cell development | 61 out of 9357 genes, 0.7% | 68 out of 10903 genes, 0.6% | 1 |
| embryonic development via the syncytial blastoderm | 47 out of 9357 genes, 0.5% | 52 out of 10903 genes, 0.5% | 1 |
| regulation of cellular protein metabolic process | 88 out of 9357 genes, 0.9% | 99 out of 10903 genes, 0.9% | 1 |
| vesicle-mediated transport | 942 out of 9357 genes, 10.1% | 1088 out of 10903 genes, 10.0% | 1 |
| regulation of cell proliferation | 25 out of 9357 genes, 0.3% | 27 out of 10903 genes, 0.2% | 1 |
| establishment or maintenance of cell polarity | 74 out of 9357 genes, 0.8% | 83 out of 10903 genes, 0.8% | 1 |
| actin filament-based process | 166 out of 9357 genes, 1.8% | 189 out of 10903 genes, 1.7% | 1 |
| pyridine nucleotide metabolic process | 32 out of 9357 genes, 0.3% | 35 out of 10903 genes, 0.3% | 1 |
| nicotinamide nucleotide metabolic process | 32 out of 9357 genes, 0.3% | 35 out of 10903 genes, 0.3% | 1 |
| respiratory system development | 46 out of 9357 genes, 0.5% | 51 out of 10903 genes, 0.5% | 1 |
| pyrimidine nucleotide biosynthetic process | 9 out of 9357 genes, 0.1% | 9 out of 10903 genes, 0.1% | 1 |
| glycolipid metabolic process | 9 out of 9357 genes, 0.1% | 9 out of 10903 genes, 0.1% | 1 |
| nucleoside diphosphate metabolic process | 9 out of 9357 genes, 0.1% | 9 out of 10903 genes, 0.1% | 1 |
| response to ionizing radiation | 9 out of 9357 genes, 0.1% | 9 out of 10903 genes, 0.1% | 1 |
| negative regulation of intracellular protein kinase cascade | 9 out of 9357 genes, 0.1% | 9 out of 10903 genes, 0.1% | 1 |
| organic alcohol transport | 9 out of 9357 genes, 0.1% | 9 out of 10903 genes, 0.1% | 1 |
| steroid hormone receptor signaling pathway | 9 out of 9357 genes, 0.1% | 9 out of 10903 genes, 0.1% | 1 |
| response to vitamin A | 9 out of 9357 genes, 0.1% | 9 out of 10903 genes, 0.1% | 1 |
| detection of stimulus involved in sensory perception | 9 out of 9357 genes, 0.1% | 9 out of 10903 genes, 0.1% | 1 |
| heart morphogenesis | 17 out of 9357 genes, 0.2% | 18 out of 10903 genes, 0.2% | 1 |
| metallo-sulfur cluster assembly | 17 out of 9357 genes, 0.2% | 18 out of 10903 genes, 0.2% | 1 |
| positive regulation of kinase activity | 17 out of 9357 genes, 0.2% | 18 out of 10903 genes, 0.2% | 1 |
| establishment of localization | 2298 out of 9357 genes, 24.6% | 2665 out of 10903 genes, 24.4% | 1 |
| larval development | 1574 out of 9357 genes, 16.8% | 1823 out of 10903 genes, 16.7% | 1 |
| axon guidance | 73 out of 9357 genes, 0.8% | 82 out of 10903 genes, 0.8% | 1 |
| signaling | 1277 out of 9357 genes, 13.6% | 1478 out of 10903 genes, 13.6% | 1 |
| cell division | 222 out of 9357 genes, 2.4% | 254 out of 10903 genes, 2.3% | 1 |
| cellular calcium ion homeostasis | 24 out of 9357 genes, 0.3% | 26 out of 10903 genes, 0.2% | 1 |
| serine family amino acid metabolic process | 24 out of 9357 genes, 0.3% | 26 out of 10903 genes, 0.2% | 1 |
| calcium ion homeostasis | 24 out of 9357 genes, 0.3% | 26 out of 10903 genes, 0.2% | 1 |
| cell part morphogenesis | 241 out of 9357 genes, 2.6% | 276 out of 10903 genes, 2.5% | 1 |
| cell projection morphogenesis | 241 out of 9357 genes, 2.6% | 276 out of 10903 genes, 2.5% | 1 |
| positive regulation of biological process | 279 out of 9357 genes, 3.0% | 320 out of 10903 genes, 2.9% | 1 |
| tRNA processing | 45 out of 9357 genes, 0.5% | 50 out of 10903 genes, 0.5% | 1 |
| cell-cell junction assembly | 31 out of 9357 genes, 0.3% | 34 out of 10903 genes, 0.3% | 1 |
| cell morphogenesis | 285 out of 9357 genes, 3.0% | 327 out of 10903 genes, 3.0% | 1 |
| immune system process | 118 out of 9357 genes, 1.3% | 134 out of 10903 genes, 1.2% | 1 |
| protein modification by small protein conjugation or removal | 118 out of 9357 genes, 1.3% | 134 out of 10903 genes, 1.2% | 1 |
| neuron projection development | 221 out of 9357 genes, 2.4% | 253 out of 10903 genes, 2.3% | 1 |
| regulation of protein modification process | 65 out of 9357 genes, 0.7% | 73 out of 10903 genes, 0.7% | 1 |
| regulation of biological quality | 585 out of 9357 genes, 6.3% | 675 out of 10903 genes, 6.2% | 1 |
| nuclear mRNA splicing, via spliceosome | 58 out of 9357 genes, 0.6% | 65 out of 10903 genes, 0.6% | 1 |
| regulation of mitotic cell cycle | 58 out of 9357 genes, 0.6% | 65 out of 10903 genes, 0.6% | 1 |
| transmembrane receptor protein tyrosine kinase signaling pathway | 78 out of 9357 genes, 0.8% | 88 out of 10903 genes, 0.8% | 1 |
| positive regulation of molecular function | 78 out of 9357 genes, 0.8% | 88 out of 10903 genes, 0.8% | 1 |
| pyrimidine nucleotide metabolic process | 16 out of 9357 genes, 0.2% | 17 out of 10903 genes, 0.2% | 1 |
| ensheathment of neurons | 16 out of 9357 genes, 0.2% | 17 out of 10903 genes, 0.2% | 1 |
| response to temperature stimulus | 16 out of 9357 genes, 0.2% | 17 out of 10903 genes, 0.2% | 1 |
| regulation of action potential in neuron | 16 out of 9357 genes, 0.2% | 17 out of 10903 genes, 0.2% | 1 |
| positive regulation of signaling pathway | 16 out of 9357 genes, 0.2% | 17 out of 10903 genes, 0.2% | 1 |
| protein depolymerization | 16 out of 9357 genes, 0.2% | 17 out of 10903 genes, 0.2% | 1 |
| oogenesis | 51 out of 9357 genes, 0.5% | 57 out of 10903 genes, 0.5% | 1 |
| translation | 257 out of 9357 genes, 2.7% | 295 out of 10903 genes, 2.7% | 1 |
| regulation of anatomical structure morphogenesis | 30 out of 9357 genes, 0.3% | 33 out of 10903 genes, 0.3% | 1 |
| rhythmic process | 30 out of 9357 genes, 0.3% | 33 out of 10903 genes, 0.3% | 1 |
| compound eye photoreceptor fate commitment | 8 out of 9357 genes, 0.1% | 8 out of 10903 genes, 0.1% | 1 |
| leukocyte mediated immunity | 8 out of 9357 genes, 0.1% | 8 out of 10903 genes, 0.1% | 1 |
| lymphocyte mediated immunity | 8 out of 9357 genes, 0.1% | 8 out of 10903 genes, 0.1% | 1 |
| chromatin remodeling | 8 out of 9357 genes, 0.1% | 8 out of 10903 genes, 0.1% | 1 |
| GPI anchor metabolic process | 8 out of 9357 genes, 0.1% | 8 out of 10903 genes, 0.1% | 1 |
| GPI anchor biosynthetic process | 8 out of 9357 genes, 0.1% | 8 out of 10903 genes, 0.1% | 1 |
| response to unfolded protein | 8 out of 9357 genes, 0.1% | 8 out of 10903 genes, 0.1% | 1 |
| regulation of S phase of mitotic cell cycle | 8 out of 9357 genes, 0.1% | 8 out of 10903 genes, 0.1% | 1 |
| ovarian nurse cell to oocyte transport | 8 out of 9357 genes, 0.1% | 8 out of 10903 genes, 0.1% | 1 |
| leg disc morphogenesis | 8 out of 9357 genes, 0.1% | 8 out of 10903 genes, 0.1% | 1 |
| protein dealkylation | 8 out of 9357 genes, 0.1% | 8 out of 10903 genes, 0.1% | 1 |
| pyrimidine ribonucleotide metabolic process | 8 out of 9357 genes, 0.1% | 8 out of 10903 genes, 0.1% | 1 |
| nerve development | 8 out of 9357 genes, 0.1% | 8 out of 10903 genes, 0.1% | 1 |
| regulation of mitotic metaphase/anaphase transition | 8 out of 9357 genes, 0.1% | 8 out of 10903 genes, 0.1% | 1 |
| lymphocyte differentiation | 8 out of 9357 genes, 0.1% | 8 out of 10903 genes, 0.1% | 1 |
| regulation of cyclase activity | 8 out of 9357 genes, 0.1% | 8 out of 10903 genes, 0.1% | 1 |
| positive regulation of cellular biosynthetic process | 8 out of 9357 genes, 0.1% | 8 out of 10903 genes, 0.1% | 1 |
| negative regulation of protein complex assembly | 8 out of 9357 genes, 0.1% | 8 out of 10903 genes, 0.1% | 1 |
| negative regulation of cellular protein metabolic process | 8 out of 9357 genes, 0.1% | 8 out of 10903 genes, 0.1% | 1 |
| positive regulation of Ras GTPase activity | 8 out of 9357 genes, 0.1% | 8 out of 10903 genes, 0.1% | 1 |
| regulation of S phase | 8 out of 9357 genes, 0.1% | 8 out of 10903 genes, 0.1% | 1 |
| fin morphogenesis | 8 out of 9357 genes, 0.1% | 8 out of 10903 genes, 0.1% | 1 |
| leg morphogenesis | 8 out of 9357 genes, 0.1% | 8 out of 10903 genes, 0.1% | 1 |
| embryonic appendage morphogenesis | 8 out of 9357 genes, 0.1% | 8 out of 10903 genes, 0.1% | 1 |
| leg disc development | 8 out of 9357 genes, 0.1% | 8 out of 10903 genes, 0.1% | 1 |
| T cell activation | 8 out of 9357 genes, 0.1% | 8 out of 10903 genes, 0.1% | 1 |
| eye photoreceptor cell fate commitment | 8 out of 9357 genes, 0.1% | 8 out of 10903 genes, 0.1% | 1 |
| camera-type eye development | 8 out of 9357 genes, 0.1% | 8 out of 10903 genes, 0.1% | 1 |
| positive regulation of GTPase activity | 8 out of 9357 genes, 0.1% | 8 out of 10903 genes, 0.1% | 1 |
| phosphoinositide biosynthetic process | 8 out of 9357 genes, 0.1% | 8 out of 10903 genes, 0.1% | 1 |
| developmental pigmentation | 8 out of 9357 genes, 0.1% | 8 out of 10903 genes, 0.1% | 1 |
| digestive tract development | 8 out of 9357 genes, 0.1% | 8 out of 10903 genes, 0.1% | 1 |
| vitamin transport | 8 out of 9357 genes, 0.1% | 8 out of 10903 genes, 0.1% | 1 |
| negative regulation of protein metabolic process | 8 out of 9357 genes, 0.1% | 8 out of 10903 genes, 0.1% | 1 |
| digestive system development | 8 out of 9357 genes, 0.1% | 8 out of 10903 genes, 0.1% | 1 |
| protein localization in endoplasmic reticulum | 8 out of 9357 genes, 0.1% | 8 out of 10903 genes, 0.1% | 1 |
| regulation of phosphorylation | 70 out of 9357 genes, 0.7% | 79 out of 10903 genes, 0.7% | 1 |
| eating behavior | 70 out of 9357 genes, 0.7% | 79 out of 10903 genes, 0.7% | 1 |
| neuron projection morphogenesis | 211 out of 9357 genes, 2.3% | 242 out of 10903 genes, 2.2% | 1 |
| response to radiation | 63 out of 9357 genes, 0.7% | 71 out of 10903 genes, 0.7% | 1 |
| homeostatic process | 255 out of 9357 genes, 2.7% | 293 out of 10903 genes, 2.7% | 1 |
| cellular component assembly involved in morphogenesis | 76 out of 9357 genes, 0.8% | 86 out of 10903 genes, 0.8% | 1 |
| actin cytoskeleton organization | 147 out of 9357 genes, 1.6% | 168 out of 10903 genes, 1.5% | 1 |
| embryo development ending in birth or egg hatching | 102 out of 9357 genes, 1.1% | 116 out of 10903 genes, 1.1% | 1 |
| macromolecule localization | 759 out of 9357 genes, 8.1% | 878 out of 10903 genes, 8.1% | 1 |
| amine transport | 36 out of 9357 genes, 0.4% | 40 out of 10903 genes, 0.4% | 1 |
| imaginal disc-derived appendage morphogenesis | 36 out of 9357 genes, 0.4% | 40 out of 10903 genes, 0.4% | 1 |
| post-embryonic appendage morphogenesis | 36 out of 9357 genes, 0.4% | 40 out of 10903 genes, 0.4% | 1 |
| imaginal disc-derived appendage development | 36 out of 9357 genes, 0.4% | 40 out of 10903 genes, 0.4% | 1 |
| cell cycle | 523 out of 9357 genes, 5.6% | 604 out of 10903 genes, 5.5% | 1 |
| regulation of programmed cell death | 121 out of 9357 genes, 1.3% | 138 out of 10903 genes, 1.3% | 1 |
| regulation of cell cycle | 121 out of 9357 genes, 1.3% | 138 out of 10903 genes, 1.3% | 1 |
| mitotic cell cycle | 292 out of 9357 genes, 3.1% | 336 out of 10903 genes, 3.1% | 1 |
| embryonic axis specification | 15 out of 9357 genes, 0.2% | 16 out of 10903 genes, 0.1% | 1 |
| synaptic vesicle exocytosis | 15 out of 9357 genes, 0.2% | 16 out of 10903 genes, 0.1% | 1 |
| axon choice point recognition | 15 out of 9357 genes, 0.2% | 16 out of 10903 genes, 0.1% | 1 |
| glial cell development | 15 out of 9357 genes, 0.2% | 16 out of 10903 genes, 0.1% | 1 |
| regulation of locomotion | 15 out of 9357 genes, 0.2% | 16 out of 10903 genes, 0.1% | 1 |
| regulation of protein complex assembly | 15 out of 9357 genes, 0.2% | 16 out of 10903 genes, 0.1% | 1 |
| axon extension | 15 out of 9357 genes, 0.2% | 16 out of 10903 genes, 0.1% | 1 |
| detection of stimulus | 15 out of 9357 genes, 0.2% | 16 out of 10903 genes, 0.1% | 1 |
| lipid homeostasis | 15 out of 9357 genes, 0.2% | 16 out of 10903 genes, 0.1% | 1 |
| developmental growth involved in morphogenesis | 15 out of 9357 genes, 0.2% | 16 out of 10903 genes, 0.1% | 1 |
| negative regulation of developmental process | 29 out of 9357 genes, 0.3% | 32 out of 10903 genes, 0.3% | 1 |
| establishment of spindle orientation | 22 out of 9357 genes, 0.2% | 24 out of 10903 genes, 0.2% | 1 |
| establishment of organelle localization | 114 out of 9357 genes, 1.2% | 130 out of 10903 genes, 1.2% | 1 |
| regulation of growth | 1175 out of 9357 genes, 12.6% | 1362 out of 10903 genes, 12.5% | 1 |
| cellular amino acid and derivative metabolic process | 228 out of 9357 genes, 2.4% | 262 out of 10903 genes, 2.4% | 1 |
| transport | 2129 out of 9357 genes, 22.8% | 2472 out of 10903 genes, 22.7% | 1 |
| one-carbon metabolic process | 55 out of 9357 genes, 0.6% | 62 out of 10903 genes, 0.6% | 1 |
| actomyosin structure organization | 55 out of 9357 genes, 0.6% | 62 out of 10903 genes, 0.6% | 1 |
| muscle cell development | 55 out of 9357 genes, 0.6% | 62 out of 10903 genes, 0.6% | 1 |
| sister chromatid segregation | 35 out of 9357 genes, 0.4% | 39 out of 10903 genes, 0.4% | 1 |
| cytokinesis | 35 out of 9357 genes, 0.4% | 39 out of 10903 genes, 0.4% | 1 |
| exocytosis | 35 out of 9357 genes, 0.4% | 39 out of 10903 genes, 0.4% | 1 |
| RNA splicing | 151 out of 9357 genes, 1.6% | 173 out of 10903 genes, 1.6% | 1 |
| regulation of neurotransmitter levels | 87 out of 9357 genes, 0.9% | 99 out of 10903 genes, 0.9% | 1 |
| MAPKKK cascade | 48 out of 9357 genes, 0.5% | 54 out of 10903 genes, 0.5% | 1 |
| cell projection organization | 295 out of 9357 genes, 3.2% | 340 out of 10903 genes, 3.1% | 1 |
| muscle organ development | 28 out of 9357 genes, 0.3% | 31 out of 10903 genes, 0.3% | 1 |
| negative regulation of cell differentiation | 28 out of 9357 genes, 0.3% | 31 out of 10903 genes, 0.3% | 1 |
| regulation of secretion | 28 out of 9357 genes, 0.3% | 31 out of 10903 genes, 0.3% | 1 |
| positive regulation of hydrolase activity | 28 out of 9357 genes, 0.3% | 31 out of 10903 genes, 0.3% | 1 |
| non-recombinational repair | 7 out of 9357 genes, 0.1% | 7 out of 10903 genes, 0.1% | 1 |
| adaptive immune response based on somatic recombination of immune receptors built from immunoglobulin superfamily domains | 7 out of 9357 genes, 0.1% | 7 out of 10903 genes, 0.1% | 1 |
| peptide secretion | 7 out of 9357 genes, 0.1% | 7 out of 10903 genes, 0.1% | 1 |
| polyamine metabolic process | 7 out of 9357 genes, 0.1% | 7 out of 10903 genes, 0.1% | 1 |
| dorsal/ventral pattern formation, imaginal disc | 7 out of 9357 genes, 0.1% | 7 out of 10903 genes, 0.1% | 1 |
| hemostasis | 7 out of 9357 genes, 0.1% | 7 out of 10903 genes, 0.1% | 1 |
| ribonucleoside metabolic process | 7 out of 9357 genes, 0.1% | 7 out of 10903 genes, 0.1% | 1 |
| negative regulation of catabolic process | 7 out of 9357 genes, 0.1% | 7 out of 10903 genes, 0.1% | 1 |
| lipid storage | 7 out of 9357 genes, 0.1% | 7 out of 10903 genes, 0.1% | 1 |
| neural tube development | 7 out of 9357 genes, 0.1% | 7 out of 10903 genes, 0.1% | 1 |
| peptide hormone secretion | 7 out of 9357 genes, 0.1% | 7 out of 10903 genes, 0.1% | 1 |
| embryonic limb morphogenesis | 7 out of 9357 genes, 0.1% | 7 out of 10903 genes, 0.1% | 1 |
| negative regulation of actin filament polymerization | 7 out of 9357 genes, 0.1% | 7 out of 10903 genes, 0.1% | 1 |
| positive regulation of cyclase activity | 7 out of 9357 genes, 0.1% | 7 out of 10903 genes, 0.1% | 1 |
| negative regulation of protein polymerization | 7 out of 9357 genes, 0.1% | 7 out of 10903 genes, 0.1% | 1 |
| positive regulation of Rho GTPase activity | 7 out of 9357 genes, 0.1% | 7 out of 10903 genes, 0.1% | 1 |
| tube fusion | 7 out of 9357 genes, 0.1% | 7 out of 10903 genes, 0.1% | 1 |
| ear morphogenesis | 7 out of 9357 genes, 0.1% | 7 out of 10903 genes, 0.1% | 1 |
| positive regulation of MAP kinase activity | 7 out of 9357 genes, 0.1% | 7 out of 10903 genes, 0.1% | 1 |
| negative regulation of MAPKKK cascade | 7 out of 9357 genes, 0.1% | 7 out of 10903 genes, 0.1% | 1 |
| negative regulation of mitosis | 7 out of 9357 genes, 0.1% | 7 out of 10903 genes, 0.1% | 1 |
| negative regulation of mitotic metaphase/anaphase transition | 7 out of 9357 genes, 0.1% | 7 out of 10903 genes, 0.1% | 1 |
| hormone secretion | 7 out of 9357 genes, 0.1% | 7 out of 10903 genes, 0.1% | 1 |
| actin filament bundle assembly | 7 out of 9357 genes, 0.1% | 7 out of 10903 genes, 0.1% | 1 |
| negative regulation of hydrolase activity | 7 out of 9357 genes, 0.1% | 7 out of 10903 genes, 0.1% | 1 |
| negative regulation of nuclear division | 7 out of 9357 genes, 0.1% | 7 out of 10903 genes, 0.1% | 1 |
| positive regulation of protein serine/threonine kinase activity | 7 out of 9357 genes, 0.1% | 7 out of 10903 genes, 0.1% | 1 |
| protein targeting to membrane | 21 out of 9357 genes, 0.2% | 23 out of 10903 genes, 0.2% | 1 |
| ovarian follicle cell migration | 21 out of 9357 genes, 0.2% | 23 out of 10903 genes, 0.2% | 1 |
| oxidoreduction coenzyme metabolic process | 41 out of 9357 genes, 0.4% | 46 out of 10903 genes, 0.4% | 1 |
| response to other organism | 54 out of 9357 genes, 0.6% | 61 out of 10903 genes, 0.6% | 1 |
| regulation of cytokine production | 14 out of 9357 genes, 0.1% | 15 out of 10903 genes, 0.1% | 1 |
| positive regulation of immune system process | 14 out of 9357 genes, 0.1% | 15 out of 10903 genes, 0.1% | 1 |
| glycine metabolic process | 14 out of 9357 genes, 0.1% | 15 out of 10903 genes, 0.1% | 1 |
| microtubule depolymerization | 14 out of 9357 genes, 0.1% | 15 out of 10903 genes, 0.1% | 1 |
| positive regulation of peptidase activity | 14 out of 9357 genes, 0.1% | 15 out of 10903 genes, 0.1% | 1 |
| positive regulation of response to stimulus | 14 out of 9357 genes, 0.1% | 15 out of 10903 genes, 0.1% | 1 |
| regulation of endopeptidase activity | 14 out of 9357 genes, 0.1% | 15 out of 10903 genes, 0.1% | 1 |
| ncRNA metabolic process | 206 out of 9357 genes, 2.2% | 237 out of 10903 genes, 2.2% | 1 |
| enzyme linked receptor protein signaling pathway | 162 out of 9357 genes, 1.7% | 186 out of 10903 genes, 1.7% | 1 |
| secretion | 162 out of 9357 genes, 1.7% | 186 out of 10903 genes, 1.7% | 1 |
| cellular macromolecular complex subunit organization | 124 out of 9357 genes, 1.3% | 142 out of 10903 genes, 1.3% | 1 |
| imaginal disc-derived wing morphogenesis | 34 out of 9357 genes, 0.4% | 38 out of 10903 genes, 0.3% | 1 |
| response to bacterium | 34 out of 9357 genes, 0.4% | 38 out of 10903 genes, 0.3% | 1 |
| regulation of membrane potential | 34 out of 9357 genes, 0.4% | 38 out of 10903 genes, 0.3% | 1 |
| RNA processing | 361 out of 9357 genes, 3.9% | 417 out of 10903 genes, 3.8% | 1 |
| phosphoinositide metabolic process | 47 out of 9357 genes, 0.5% | 53 out of 10903 genes, 0.5% | 1 |
| regulation of nervous system development | 47 out of 9357 genes, 0.5% | 53 out of 10903 genes, 0.5% | 1 |
| regulation of molecular function | 230 out of 9357 genes, 2.5% | 265 out of 10903 genes, 2.4% | 1 |
| positive regulation of cellular component organization | 27 out of 9357 genes, 0.3% | 30 out of 10903 genes, 0.3% | 1 |
| protein glycosylation | 53 out of 9357 genes, 0.6% | 60 out of 10903 genes, 0.6% | 1 |
| macromolecule glycosylation | 53 out of 9357 genes, 0.6% | 60 out of 10903 genes, 0.6% | 1 |
| glycosylation | 53 out of 9357 genes, 0.6% | 60 out of 10903 genes, 0.6% | 1 |
| feeding behavior | 72 out of 9357 genes, 0.8% | 82 out of 10903 genes, 0.8% | 1 |
| regulation of phosphate metabolic process | 72 out of 9357 genes, 0.8% | 82 out of 10903 genes, 0.8% | 1 |
| regulation of phosphorus metabolic process | 72 out of 9357 genes, 0.8% | 82 out of 10903 genes, 0.8% | 1 |
| translational initiation | 20 out of 9357 genes, 0.2% | 22 out of 10903 genes, 0.2% | 1 |
| water-soluble vitamin metabolic process | 20 out of 9357 genes, 0.2% | 22 out of 10903 genes, 0.2% | 1 |
| negative regulation of signaling pathway | 20 out of 9357 genes, 0.2% | 22 out of 10903 genes, 0.2% | 1 |
| aerobic respiration | 46 out of 9357 genes, 0.5% | 52 out of 10903 genes, 0.5% | 1 |
| regulation of cell death | 122 out of 9357 genes, 1.3% | 140 out of 10903 genes, 1.3% | 1 |
| heart development | 33 out of 9357 genes, 0.4% | 37 out of 10903 genes, 0.3% | 1 |
| hemopoiesis | 33 out of 9357 genes, 0.4% | 37 out of 10903 genes, 0.3% | 1 |
| di-, tri-valent inorganic cation homeostasis | 33 out of 9357 genes, 0.4% | 37 out of 10903 genes, 0.3% | 1 |
| axonogenesis | 178 out of 9357 genes, 1.9% | 205 out of 10903 genes, 1.9% | 1 |
| cellular protein complex assembly | 65 out of 9357 genes, 0.7% | 74 out of 10903 genes, 0.7% | 1 |
| reproductive behavior | 308 out of 9357 genes, 3.3% | 356 out of 10903 genes, 3.3% | 1 |
| response to acid | 13 out of 9357 genes, 0.1% | 14 out of 10903 genes, 0.1% | 1 |
| somatic diversification of immune receptors | 13 out of 9357 genes, 0.1% | 14 out of 10903 genes, 0.1% | 1 |
| epidermis development | 13 out of 9357 genes, 0.1% | 14 out of 10903 genes, 0.1% | 1 |
| regulation of cellular ketone metabolic process | 13 out of 9357 genes, 0.1% | 14 out of 10903 genes, 0.1% | 1 |
| microtubule organizing center organization | 13 out of 9357 genes, 0.1% | 14 out of 10903 genes, 0.1% | 1 |
| positive regulation of caspase activity | 13 out of 9357 genes, 0.1% | 14 out of 10903 genes, 0.1% | 1 |
| regulation of caspase activity | 13 out of 9357 genes, 0.1% | 14 out of 10903 genes, 0.1% | 1 |
| response to peptide hormone stimulus | 13 out of 9357 genes, 0.1% | 14 out of 10903 genes, 0.1% | 1 |
| response to estrogen stimulus | 13 out of 9357 genes, 0.1% | 14 out of 10903 genes, 0.1% | 1 |
| stem cell differentiation | 13 out of 9357 genes, 0.1% | 14 out of 10903 genes, 0.1% | 1 |
| regulation of body fluid levels | 13 out of 9357 genes, 0.1% | 14 out of 10903 genes, 0.1% | 1 |
| positive regulation of multicellular organismal process | 13 out of 9357 genes, 0.1% | 14 out of 10903 genes, 0.1% | 1 |
| interphase | 13 out of 9357 genes, 0.1% | 14 out of 10903 genes, 0.1% | 1 |
| ncRNA processing | 71 out of 9357 genes, 0.8% | 81 out of 10903 genes, 0.7% | 1 |
| multicellular organismal reproductive behavior | 301 out of 9357 genes, 3.2% | 348 out of 10903 genes, 3.2% | 1 |
| membrane lipid metabolic process | 26 out of 9357 genes, 0.3% | 29 out of 10903 genes, 0.3% | 1 |
| open tracheal system development | 26 out of 9357 genes, 0.3% | 29 out of 10903 genes, 0.3% | 1 |
| associative learning | 26 out of 9357 genes, 0.3% | 29 out of 10903 genes, 0.3% | 1 |
| carboxylic acid metabolic process | 350 out of 9357 genes, 3.7% | 405 out of 10903 genes, 3.7% | 1 |
| regulation of DNA recombination | 6 out of 9357 genes, 0.1% | 6 out of 10903 genes, 0.1% | 1 |
| activation of MAPK activity | 6 out of 9357 genes, 0.1% | 6 out of 10903 genes, 0.1% | 1 |
| telomere maintenance | 6 out of 9357 genes, 0.1% | 6 out of 10903 genes, 0.1% | 1 |
| neural tube formation | 6 out of 9357 genes, 0.1% | 6 out of 10903 genes, 0.1% | 1 |
| immunoglobulin production | 6 out of 9357 genes, 0.1% | 6 out of 10903 genes, 0.1% | 1 |
| regulation of leukocyte activation | 6 out of 9357 genes, 0.1% | 6 out of 10903 genes, 0.1% | 1 |
| chitin metabolic process | 6 out of 9357 genes, 0.1% | 6 out of 10903 genes, 0.1% | 1 |
| acetyl-CoA biosynthetic process | 6 out of 9357 genes, 0.1% | 6 out of 10903 genes, 0.1% | 1 |
| DNA packaging | 6 out of 9357 genes, 0.1% | 6 out of 10903 genes, 0.1% | 1 |
| protein demethylation | 6 out of 9357 genes, 0.1% | 6 out of 10903 genes, 0.1% | 1 |
| protein monoubiquitination | 6 out of 9357 genes, 0.1% | 6 out of 10903 genes, 0.1% | 1 |
| smooth muscle contraction | 6 out of 9357 genes, 0.1% | 6 out of 10903 genes, 0.1% | 1 |
| epidermal growth factor receptor signaling pathway | 6 out of 9357 genes, 0.1% | 6 out of 10903 genes, 0.1% | 1 |
| G-protein signaling, coupled to cAMP nucleotide second messenger | 6 out of 9357 genes, 0.1% | 6 out of 10903 genes, 0.1% | 1 |
| negative regulation of cell proliferation | 6 out of 9357 genes, 0.1% | 6 out of 10903 genes, 0.1% | 1 |
| fibroblast growth factor receptor signaling pathway | 6 out of 9357 genes, 0.1% | 6 out of 10903 genes, 0.1% | 1 |
| nucleoside diphosphate catabolic process | 6 out of 9357 genes, 0.1% | 6 out of 10903 genes, 0.1% | 1 |
| pyrimidine nucleoside diphosphate metabolic process | 6 out of 9357 genes, 0.1% | 6 out of 10903 genes, 0.1% | 1 |
| response to toxin | 6 out of 9357 genes, 0.1% | 6 out of 10903 genes, 0.1% | 1 |
| negative regulation of peptidase activity | 6 out of 9357 genes, 0.1% | 6 out of 10903 genes, 0.1% | 1 |
| regulation of metal ion transport | 6 out of 9357 genes, 0.1% | 6 out of 10903 genes, 0.1% | 1 |
| primary neural tube formation | 6 out of 9357 genes, 0.1% | 6 out of 10903 genes, 0.1% | 1 |
| phospholipid transport | 6 out of 9357 genes, 0.1% | 6 out of 10903 genes, 0.1% | 1 |
| somatic diversification of immunoglobulins | 6 out of 9357 genes, 0.1% | 6 out of 10903 genes, 0.1% | 1 |
| 4-hydroxyproline metabolic process | 6 out of 9357 genes, 0.1% | 6 out of 10903 genes, 0.1% | 1 |
| cAMP-mediated signaling | 6 out of 9357 genes, 0.1% | 6 out of 10903 genes, 0.1% | 1 |
| regulation of Wnt receptor signaling pathway | 6 out of 9357 genes, 0.1% | 6 out of 10903 genes, 0.1% | 1 |
| regulation of cyclic nucleotide metabolic process | 6 out of 9357 genes, 0.1% | 6 out of 10903 genes, 0.1% | 1 |
| regulation of cyclic nucleotide biosynthetic process | 6 out of 9357 genes, 0.1% | 6 out of 10903 genes, 0.1% | 1 |
| regulation of nucleotide biosynthetic process | 6 out of 9357 genes, 0.1% | 6 out of 10903 genes, 0.1% | 1 |
| regulation of cAMP metabolic process | 6 out of 9357 genes, 0.1% | 6 out of 10903 genes, 0.1% | 1 |
| regulation of cAMP biosynthetic process | 6 out of 9357 genes, 0.1% | 6 out of 10903 genes, 0.1% | 1 |
| telomere organization | 6 out of 9357 genes, 0.1% | 6 out of 10903 genes, 0.1% | 1 |
| regulation of synaptic transmission, GABAergic | 6 out of 9357 genes, 0.1% | 6 out of 10903 genes, 0.1% | 1 |
| negative regulation of intracellular transport | 6 out of 9357 genes, 0.1% | 6 out of 10903 genes, 0.1% | 1 |
| carbohydrate homeostasis | 6 out of 9357 genes, 0.1% | 6 out of 10903 genes, 0.1% | 1 |
| endocrine system development | 6 out of 9357 genes, 0.1% | 6 out of 10903 genes, 0.1% | 1 |
| purine nucleoside metabolic process | 6 out of 9357 genes, 0.1% | 6 out of 10903 genes, 0.1% | 1 |
| cellular biogenic amine biosynthetic process | 6 out of 9357 genes, 0.1% | 6 out of 10903 genes, 0.1% | 1 |
| serotonin metabolic process | 6 out of 9357 genes, 0.1% | 6 out of 10903 genes, 0.1% | 1 |
| regulation of eye photoreceptor cell development | 6 out of 9357 genes, 0.1% | 6 out of 10903 genes, 0.1% | 1 |
| neuron maturation | 6 out of 9357 genes, 0.1% | 6 out of 10903 genes, 0.1% | 1 |
| regulation of myeloid cell differentiation | 6 out of 9357 genes, 0.1% | 6 out of 10903 genes, 0.1% | 1 |
| regulation of adenylate cyclase activity | 6 out of 9357 genes, 0.1% | 6 out of 10903 genes, 0.1% | 1 |
| regulation of photoreceptor cell differentiation | 6 out of 9357 genes, 0.1% | 6 out of 10903 genes, 0.1% | 1 |
| synapse organization | 6 out of 9357 genes, 0.1% | 6 out of 10903 genes, 0.1% | 1 |
| regulation of cell activation | 6 out of 9357 genes, 0.1% | 6 out of 10903 genes, 0.1% | 1 |
| regulation of lymphocyte activation | 6 out of 9357 genes, 0.1% | 6 out of 10903 genes, 0.1% | 1 |
| regulation of lyase activity | 6 out of 9357 genes, 0.1% | 6 out of 10903 genes, 0.1% | 1 |
| demethylation | 6 out of 9357 genes, 0.1% | 6 out of 10903 genes, 0.1% | 1 |
| Rho protein signal transduction | 64 out of 9357 genes, 0.7% | 73 out of 10903 genes, 0.7% | 1 |
| M phase | 207 out of 9357 genes, 2.2% | 239 out of 10903 genes, 2.2% | 1 |
| cellular homeostasis | 176 out of 9357 genes, 1.9% | 203 out of 10903 genes, 1.9% | 1 |
| methylation | 32 out of 9357 genes, 0.3% | 36 out of 10903 genes, 0.3% | 1 |
| regulation of binding | 32 out of 9357 genes, 0.3% | 36 out of 10903 genes, 0.3% | 1 |
| microtubule polymerization or depolymerization | 19 out of 9357 genes, 0.2% | 21 out of 10903 genes, 0.2% | 1 |
| synaptic vesicle transport | 19 out of 9357 genes, 0.2% | 21 out of 10903 genes, 0.2% | 1 |
| protein modification by small protein removal | 19 out of 9357 genes, 0.2% | 21 out of 10903 genes, 0.2% | 1 |
| epithelial cell differentiation | 44 out of 9357 genes, 0.5% | 50 out of 10903 genes, 0.5% | 1 |
| eye photoreceptor cell differentiation | 25 out of 9357 genes, 0.3% | 28 out of 10903 genes, 0.3% | 1 |
| ER-nucleus signaling pathway | 25 out of 9357 genes, 0.3% | 28 out of 10903 genes, 0.3% | 1 |
| negative regulation of macromolecule biosynthetic process | 25 out of 9357 genes, 0.3% | 28 out of 10903 genes, 0.3% | 1 |
| regulation of growth rate | 1103 out of 9357 genes, 11.8% | 1282 out of 10903 genes, 11.8% | 1 |
| regulation of cellular localization | 50 out of 9357 genes, 0.5% | 57 out of 10903 genes, 0.5% | 1 |
| DNA catabolic process, endonucleolytic | 12 out of 9357 genes, 0.1% | 13 out of 10903 genes, 0.1% | 1 |
| in utero embryonic development | 12 out of 9357 genes, 0.1% | 13 out of 10903 genes, 0.1% | 1 |
| establishment of planar polarity | 12 out of 9357 genes, 0.1% | 13 out of 10903 genes, 0.1% | 1 |
| leukocyte differentiation | 12 out of 9357 genes, 0.1% | 13 out of 10903 genes, 0.1% | 1 |
| NADP metabolic process | 12 out of 9357 genes, 0.1% | 13 out of 10903 genes, 0.1% | 1 |
| establishment of tissue polarity | 12 out of 9357 genes, 0.1% | 13 out of 10903 genes, 0.1% | 1 |
| I-kappaB kinase/NF-kappaB cascade | 12 out of 9357 genes, 0.1% | 13 out of 10903 genes, 0.1% | 1 |
| rhythmic behavior | 12 out of 9357 genes, 0.1% | 13 out of 10903 genes, 0.1% | 1 |
| secondary metabolic process | 12 out of 9357 genes, 0.1% | 13 out of 10903 genes, 0.1% | 1 |
| regulation of ion transport | 12 out of 9357 genes, 0.1% | 13 out of 10903 genes, 0.1% | 1 |
| positive regulation of endocytosis | 12 out of 9357 genes, 0.1% | 13 out of 10903 genes, 0.1% | 1 |
| regulation of organ morphogenesis | 12 out of 9357 genes, 0.1% | 13 out of 10903 genes, 0.1% | 1 |
| regulation of catalytic activity | 186 out of 9357 genes, 2.0% | 215 out of 10903 genes, 2.0% | 1 |
| nuclear-transcribed mRNA catabolic process | 31 out of 9357 genes, 0.3% | 35 out of 10903 genes, 0.3% | 1 |
| mRNA catabolic process | 31 out of 9357 genes, 0.3% | 35 out of 10903 genes, 0.3% | 1 |
| protein N-linked glycosylation | 31 out of 9357 genes, 0.3% | 35 out of 10903 genes, 0.3% | 1 |
| establishment of cell polarity | 31 out of 9357 genes, 0.3% | 35 out of 10903 genes, 0.3% | 1 |
| macromolecule methylation | 31 out of 9357 genes, 0.3% | 35 out of 10903 genes, 0.3% | 1 |
| cellular cation homeostasis | 62 out of 9357 genes, 0.7% | 71 out of 10903 genes, 0.7% | 1 |
| compound eye photoreceptor cell differentiation | 18 out of 9357 genes, 0.2% | 20 out of 10903 genes, 0.2% | 1 |
| gas transport | 18 out of 9357 genes, 0.2% | 20 out of 10903 genes, 0.2% | 1 |
| regulation of nucleocytoplasmic transport | 18 out of 9357 genes, 0.2% | 20 out of 10903 genes, 0.2% | 1 |
| positive regulation of binding | 18 out of 9357 genes, 0.2% | 20 out of 10903 genes, 0.2% | 1 |
| regulation of DNA binding | 18 out of 9357 genes, 0.2% | 20 out of 10903 genes, 0.2% | 1 |
| meiosis | 43 out of 9357 genes, 0.5% | 49 out of 10903 genes, 0.4% | 1 |
| M phase of meiotic cell cycle | 43 out of 9357 genes, 0.5% | 49 out of 10903 genes, 0.4% | 1 |
| vesicle coating | 24 out of 9357 genes, 0.3% | 27 out of 10903 genes, 0.2% | 1 |
| positive regulation of cell communication | 24 out of 9357 genes, 0.3% | 27 out of 10903 genes, 0.2% | 1 |
| ovarian follicle cell development | 24 out of 9357 genes, 0.3% | 27 out of 10903 genes, 0.2% | 1 |
| negative regulation of cellular macromolecule biosynthetic process | 24 out of 9357 genes, 0.3% | 27 out of 10903 genes, 0.2% | 1 |
| pharyngeal pumping | 55 out of 9357 genes, 0.6% | 63 out of 10903 genes, 0.6% | 1 |
| protein modification by small protein conjugation | 98 out of 9357 genes, 1.0% | 113 out of 10903 genes, 1.0% | 1 |
| response to external stimulus | 275 out of 9357 genes, 2.9% | 319 out of 10903 genes, 2.9% | 1 |
| vacuole organization | 30 out of 9357 genes, 0.3% | 34 out of 10903 genes, 0.3% | 1 |
| protein alkylation | 30 out of 9357 genes, 0.3% | 34 out of 10903 genes, 0.3% | 1 |
| positive regulation of cellular metabolic process | 30 out of 9357 genes, 0.3% | 34 out of 10903 genes, 0.3% | 1 |
| organic acid metabolic process | 360 out of 9357 genes, 3.8% | 418 out of 10903 genes, 3.8% | 1 |
| RNA methylation | 5 out of 9357 genes, 0.1% | 5 out of 10903 genes, 0.0% | 1 |
| gastrulation with mouth forming first | 5 out of 9357 genes, 0.1% | 5 out of 10903 genes, 0.0% | 1 |
| oenocyte differentiation | 5 out of 9357 genes, 0.1% | 5 out of 10903 genes, 0.0% | 1 |
| aggressive behavior | 5 out of 9357 genes, 0.1% | 5 out of 10903 genes, 0.0% | 1 |
| endothelium development | 5 out of 9357 genes, 0.1% | 5 out of 10903 genes, 0.0% | 1 |
| fructose metabolic process | 5 out of 9357 genes, 0.1% | 5 out of 10903 genes, 0.0% | 1 |
| 2-oxoglutarate metabolic process | 5 out of 9357 genes, 0.1% | 5 out of 10903 genes, 0.0% | 1 |
| regulation of carbohydrate metabolic process | 5 out of 9357 genes, 0.1% | 5 out of 10903 genes, 0.0% | 1 |
| DNA strand elongation involved in DNA replication | 5 out of 9357 genes, 0.1% | 5 out of 10903 genes, 0.0% | 1 |
| chromatin silencing | 5 out of 9357 genes, 0.1% | 5 out of 10903 genes, 0.0% | 1 |
| regulation of translational elongation | 5 out of 9357 genes, 0.1% | 5 out of 10903 genes, 0.0% | 1 |
| SRP-dependent cotranslational protein targeting to membrane | 5 out of 9357 genes, 0.1% | 5 out of 10903 genes, 0.0% | 1 |
| glycosphingolipid metabolic process | 5 out of 9357 genes, 0.1% | 5 out of 10903 genes, 0.0% | 1 |
| NADH metabolic process | 5 out of 9357 genes, 0.1% | 5 out of 10903 genes, 0.0% | 1 |
| calcium ion transport | 5 out of 9357 genes, 0.1% | 5 out of 10903 genes, 0.0% | 1 |
| JAK-STAT cascade | 5 out of 9357 genes, 0.1% | 5 out of 10903 genes, 0.0% | 1 |
| imaginal disc-derived leg morphogenesis | 5 out of 9357 genes, 0.1% | 5 out of 10903 genes, 0.0% | 1 |
| visual perception | 5 out of 9357 genes, 0.1% | 5 out of 10903 genes, 0.0% | 1 |
| phototransduction | 5 out of 9357 genes, 0.1% | 5 out of 10903 genes, 0.0% | 1 |
| phototransduction, visible light | 5 out of 9357 genes, 0.1% | 5 out of 10903 genes, 0.0% | 1 |
| regulation of G-protein coupled receptor protein signaling pathway | 5 out of 9357 genes, 0.1% | 5 out of 10903 genes, 0.0% | 1 |
| imaginal disc-derived wing margin morphogenesis | 5 out of 9357 genes, 0.1% | 5 out of 10903 genes, 0.0% | 1 |
| DNA damage response, signal transduction resulting in induction of apoptosis | 5 out of 9357 genes, 0.1% | 5 out of 10903 genes, 0.0% | 1 |
| pyrimidine nucleoside monophosphate metabolic process | 5 out of 9357 genes, 0.1% | 5 out of 10903 genes, 0.0% | 1 |
| pyrimidine nucleoside monophosphate biosynthetic process | 5 out of 9357 genes, 0.1% | 5 out of 10903 genes, 0.0% | 1 |
| purine nucleoside diphosphate metabolic process | 5 out of 9357 genes, 0.1% | 5 out of 10903 genes, 0.0% | 1 |
| nucleoside biosynthetic process | 5 out of 9357 genes, 0.1% | 5 out of 10903 genes, 0.0% | 1 |
| purine ribonucleoside diphosphate metabolic process | 5 out of 9357 genes, 0.1% | 5 out of 10903 genes, 0.0% | 1 |
| ribonucleoside diphosphate metabolic process | 5 out of 9357 genes, 0.1% | 5 out of 10903 genes, 0.0% | 1 |
| pyrimidine deoxyribonucleotide biosynthetic process | 5 out of 9357 genes, 0.1% | 5 out of 10903 genes, 0.0% | 1 |
| deoxyribonucleotide biosynthetic process | 5 out of 9357 genes, 0.1% | 5 out of 10903 genes, 0.0% | 1 |
| 2'-deoxyribonucleotide biosynthetic process | 5 out of 9357 genes, 0.1% | 5 out of 10903 genes, 0.0% | 1 |
| rhodopsin mediated phototransduction | 5 out of 9357 genes, 0.1% | 5 out of 10903 genes, 0.0% | 1 |
| regulation of glycoprotein biosynthetic process | 5 out of 9357 genes, 0.1% | 5 out of 10903 genes, 0.0% | 1 |
| positive regulation of glycoprotein biosynthetic process | 5 out of 9357 genes, 0.1% | 5 out of 10903 genes, 0.0% | 1 |
| regulation of cellular carbohydrate metabolic process | 5 out of 9357 genes, 0.1% | 5 out of 10903 genes, 0.0% | 1 |
| regulation of glucose metabolic process | 5 out of 9357 genes, 0.1% | 5 out of 10903 genes, 0.0% | 1 |
| organic anion transport | 5 out of 9357 genes, 0.1% | 5 out of 10903 genes, 0.0% | 1 |
| monoamine transport | 5 out of 9357 genes, 0.1% | 5 out of 10903 genes, 0.0% | 1 |
| bis(5'-nucleosidyl) oligophosphate metabolic process | 5 out of 9357 genes, 0.1% | 5 out of 10903 genes, 0.0% | 1 |
| bis(5'-nucleosidyl) oligophosphate biosynthetic process | 5 out of 9357 genes, 0.1% | 5 out of 10903 genes, 0.0% | 1 |
| diadenosine polyphosphate metabolic process | 5 out of 9357 genes, 0.1% | 5 out of 10903 genes, 0.0% | 1 |
| diadenosine polyphosphate biosynthetic process | 5 out of 9357 genes, 0.1% | 5 out of 10903 genes, 0.0% | 1 |
| regulation of calcium ion-dependent exocytosis | 5 out of 9357 genes, 0.1% | 5 out of 10903 genes, 0.0% | 1 |
| B cell mediated immunity | 5 out of 9357 genes, 0.1% | 5 out of 10903 genes, 0.0% | 1 |
| cranial nerve development | 5 out of 9357 genes, 0.1% | 5 out of 10903 genes, 0.0% | 1 |
| DNA strand elongation | 5 out of 9357 genes, 0.1% | 5 out of 10903 genes, 0.0% | 1 |
| lamellipodium assembly | 5 out of 9357 genes, 0.1% | 5 out of 10903 genes, 0.0% | 1 |
| regulation of proteolysis | 5 out of 9357 genes, 0.1% | 5 out of 10903 genes, 0.0% | 1 |
| chromosome condensation | 5 out of 9357 genes, 0.1% | 5 out of 10903 genes, 0.0% | 1 |
| negative regulation of cellular catabolic process | 5 out of 9357 genes, 0.1% | 5 out of 10903 genes, 0.0% | 1 |
| positive regulation of cellular catabolic process | 5 out of 9357 genes, 0.1% | 5 out of 10903 genes, 0.0% | 1 |
| positive regulation of cell projection organization | 5 out of 9357 genes, 0.1% | 5 out of 10903 genes, 0.0% | 1 |
| DNA geometric change | 5 out of 9357 genes, 0.1% | 5 out of 10903 genes, 0.0% | 1 |
| regulation of chemokine production | 5 out of 9357 genes, 0.1% | 5 out of 10903 genes, 0.0% | 1 |
| positive regulation of chemokine production | 5 out of 9357 genes, 0.1% | 5 out of 10903 genes, 0.0% | 1 |
| cellular response to hormone stimulus | 5 out of 9357 genes, 0.1% | 5 out of 10903 genes, 0.0% | 1 |
| cytokinesis after meiosis | 5 out of 9357 genes, 0.1% | 5 out of 10903 genes, 0.0% | 1 |
| cellular response to unfolded protein | 5 out of 9357 genes, 0.1% | 5 out of 10903 genes, 0.0% | 1 |
| negative regulation of transmembrane transport | 5 out of 9357 genes, 0.1% | 5 out of 10903 genes, 0.0% | 1 |
| imaginal disc-derived limb morphogenesis | 5 out of 9357 genes, 0.1% | 5 out of 10903 genes, 0.0% | 1 |
| post-embryonic limb morphogenesis | 5 out of 9357 genes, 0.1% | 5 out of 10903 genes, 0.0% | 1 |
| cell migration involved in gastrulation | 5 out of 9357 genes, 0.1% | 5 out of 10903 genes, 0.0% | 1 |
| hormone metabolic process | 5 out of 9357 genes, 0.1% | 5 out of 10903 genes, 0.0% | 1 |
| purine nucleoside biosynthetic process | 5 out of 9357 genes, 0.1% | 5 out of 10903 genes, 0.0% | 1 |
| ribonucleoside biosynthetic process | 5 out of 9357 genes, 0.1% | 5 out of 10903 genes, 0.0% | 1 |
| protein targeting to ER | 5 out of 9357 genes, 0.1% | 5 out of 10903 genes, 0.0% | 1 |
| positive regulation of adenylate cyclase activity | 5 out of 9357 genes, 0.1% | 5 out of 10903 genes, 0.0% | 1 |
| negative regulation of gene expression, epigenetic | 5 out of 9357 genes, 0.1% | 5 out of 10903 genes, 0.0% | 1 |
| positive regulation of nucleobase, nucleoside, nucleotide and nucleic acid metabolic process | 5 out of 9357 genes, 0.1% | 5 out of 10903 genes, 0.0% | 1 |
| purine ribonucleoside metabolic process | 5 out of 9357 genes, 0.1% | 5 out of 10903 genes, 0.0% | 1 |
| purine ribonucleoside biosynthetic process | 5 out of 9357 genes, 0.1% | 5 out of 10903 genes, 0.0% | 1 |
| negative regulation of nucleocytoplasmic transport | 5 out of 9357 genes, 0.1% | 5 out of 10903 genes, 0.0% | 1 |
| positive regulation of viral reproduction | 5 out of 9357 genes, 0.1% | 5 out of 10903 genes, 0.0% | 1 |
| embryonic skeletal system morphogenesis | 5 out of 9357 genes, 0.1% | 5 out of 10903 genes, 0.0% | 1 |
| skeletal system morphogenesis | 5 out of 9357 genes, 0.1% | 5 out of 10903 genes, 0.0% | 1 |
| embryonic skeletal system development | 5 out of 9357 genes, 0.1% | 5 out of 10903 genes, 0.0% | 1 |
| detection of light stimulus involved in visual perception | 5 out of 9357 genes, 0.1% | 5 out of 10903 genes, 0.0% | 1 |
| sensory perception of taste | 5 out of 9357 genes, 0.1% | 5 out of 10903 genes, 0.0% | 1 |
| detection of light stimulus involved in sensory perception | 5 out of 9357 genes, 0.1% | 5 out of 10903 genes, 0.0% | 1 |
| negative regulation of binding | 5 out of 9357 genes, 0.1% | 5 out of 10903 genes, 0.0% | 1 |
| positive regulation of nitrogen compound metabolic process | 5 out of 9357 genes, 0.1% | 5 out of 10903 genes, 0.0% | 1 |
| cartilage development | 5 out of 9357 genes, 0.1% | 5 out of 10903 genes, 0.0% | 1 |
| negative regulation of protein transport | 5 out of 9357 genes, 0.1% | 5 out of 10903 genes, 0.0% | 1 |
| G1 phase | 5 out of 9357 genes, 0.1% | 5 out of 10903 genes, 0.0% | 1 |
| positive regulation of lyase activity | 5 out of 9357 genes, 0.1% | 5 out of 10903 genes, 0.0% | 1 |
| regulation of meiotic cell cycle | 5 out of 9357 genes, 0.1% | 5 out of 10903 genes, 0.0% | 1 |
| catecholamine transport | 5 out of 9357 genes, 0.1% | 5 out of 10903 genes, 0.0% | 1 |
| cellular response to biotic stimulus | 5 out of 9357 genes, 0.1% | 5 out of 10903 genes, 0.0% | 1 |
| cellular response to protein stimulus | 5 out of 9357 genes, 0.1% | 5 out of 10903 genes, 0.0% | 1 |
| cellular response to endogenous stimulus | 5 out of 9357 genes, 0.1% | 5 out of 10903 genes, 0.0% | 1 |
| negative regulation of intracellular protein transport | 5 out of 9357 genes, 0.1% | 5 out of 10903 genes, 0.0% | 1 |
| negative regulation of cellular metabolic process | 36 out of 9357 genes, 0.4% | 41 out of 10903 genes, 0.4% | 1 |
| cellular macromolecule metabolic process | 2912 out of 9357 genes, 31.1% | 3391 out of 10903 genes, 31.1% | 1 |
| oxoacid metabolic process | 359 out of 9357 genes, 3.8% | 417 out of 10903 genes, 3.8% | 1 |
| cellular nitrogen compound metabolic process | 1797 out of 9357 genes, 19.2% | 2092 out of 10903 genes, 19.2% | 1 |
| cellular metal ion homeostasis | 48 out of 9357 genes, 0.5% | 55 out of 10903 genes, 0.5% | 1 |
| protein-based cuticle development | 109 out of 9357 genes, 1.2% | 126 out of 10903 genes, 1.2% | 1 |
| nitrogen compound metabolic process | 1832 out of 9357 genes, 19.6% | 2133 out of 10903 genes, 19.6% | 1 |
| immune effector process | 11 out of 9357 genes, 0.1% | 12 out of 10903 genes, 0.1% | 1 |
| dorsal closure | 11 out of 9357 genes, 0.1% | 12 out of 10903 genes, 0.1% | 1 |
| phospholipid biosynthetic process | 11 out of 9357 genes, 0.1% | 12 out of 10903 genes, 0.1% | 1 |
| positive regulation of catabolic process | 11 out of 9357 genes, 0.1% | 12 out of 10903 genes, 0.1% | 1 |
| regulation of fatty acid metabolic process | 11 out of 9357 genes, 0.1% | 12 out of 10903 genes, 0.1% | 1 |
| regulation of actin filament polymerization | 11 out of 9357 genes, 0.1% | 12 out of 10903 genes, 0.1% | 1 |
| regulation of protein polymerization | 11 out of 9357 genes, 0.1% | 12 out of 10903 genes, 0.1% | 1 |
| regulation of tube size, open tracheal system | 11 out of 9357 genes, 0.1% | 12 out of 10903 genes, 0.1% | 1 |
| regulation of tube architecture, open tracheal system | 11 out of 9357 genes, 0.1% | 12 out of 10903 genes, 0.1% | 1 |
| neurotransmitter metabolic process | 11 out of 9357 genes, 0.1% | 12 out of 10903 genes, 0.1% | 1 |
| maintenance of protein location | 11 out of 9357 genes, 0.1% | 12 out of 10903 genes, 0.1% | 1 |
| autophagic cell death | 11 out of 9357 genes, 0.1% | 12 out of 10903 genes, 0.1% | 1 |
| regulation of immune response | 11 out of 9357 genes, 0.1% | 12 out of 10903 genes, 0.1% | 1 |
| sensory perception of light stimulus | 11 out of 9357 genes, 0.1% | 12 out of 10903 genes, 0.1% | 1 |
| maintenance of location in cell | 11 out of 9357 genes, 0.1% | 12 out of 10903 genes, 0.1% | 1 |
| cofactor metabolic process | 133 out of 9357 genes, 1.4% | 154 out of 10903 genes, 1.4% | 1 |
| glycerolipid metabolic process | 72 out of 9357 genes, 0.8% | 83 out of 10903 genes, 0.8% | 1 |
| response to carbohydrate stimulus | 17 out of 9357 genes, 0.2% | 19 out of 10903 genes, 0.2% | 1 |
| telencephalon development | 17 out of 9357 genes, 0.2% | 19 out of 10903 genes, 0.2% | 1 |
| hindbrain development | 17 out of 9357 genes, 0.2% | 19 out of 10903 genes, 0.2% | 1 |
| segmentation | 17 out of 9357 genes, 0.2% | 19 out of 10903 genes, 0.2% | 1 |
| pigment metabolic process | 17 out of 9357 genes, 0.2% | 19 out of 10903 genes, 0.2% | 1 |
| regulation of neuron differentiation | 17 out of 9357 genes, 0.2% | 19 out of 10903 genes, 0.2% | 1 |
| protein methylation | 23 out of 9357 genes, 0.2% | 26 out of 10903 genes, 0.2% | 1 |
| immune response | 23 out of 9357 genes, 0.2% | 26 out of 10903 genes, 0.2% | 1 |
| chemosensory behavior | 23 out of 9357 genes, 0.2% | 26 out of 10903 genes, 0.2% | 1 |
| polyol metabolic process | 23 out of 9357 genes, 0.2% | 26 out of 10903 genes, 0.2% | 1 |
| Ras protein signal transduction | 96 out of 9357 genes, 1.0% | 111 out of 10903 genes, 1.0% | 1 |
| mitotic sister chromatid segregation | 29 out of 9357 genes, 0.3% | 33 out of 10903 genes, 0.3% | 1 |
| forebrain development | 29 out of 9357 genes, 0.3% | 33 out of 10903 genes, 0.3% | 1 |
| intracellular signaling pathway | 435 out of 9357 genes, 4.6% | 506 out of 10903 genes, 4.6% | 1 |
| meiotic cell cycle | 47 out of 9357 genes, 0.5% | 54 out of 10903 genes, 0.5% | 1 |
| cellular ketone metabolic process | 368 out of 9357 genes, 3.9% | 428 out of 10903 genes, 3.9% | 1 |
| glycerophospholipid metabolic process | 59 out of 9357 genes, 0.6% | 68 out of 10903 genes, 0.6% | 1 |
| phagocytosis | 65 out of 9357 genes, 0.7% | 75 out of 10903 genes, 0.7% | 1 |
| macromolecule biosynthetic process | 888 out of 9357 genes, 9.5% | 1034 out of 10903 genes, 9.5% | 1 |
| programmed cell death | 167 out of 9357 genes, 1.8% | 194 out of 10903 genes, 1.8% | 1 |
| protein lipidation | 40 out of 9357 genes, 0.4% | 46 out of 10903 genes, 0.4% | 1 |
| lipoprotein metabolic process | 40 out of 9357 genes, 0.4% | 46 out of 10903 genes, 0.4% | 1 |
| lipoprotein biosynthetic process | 40 out of 9357 genes, 0.4% | 46 out of 10903 genes, 0.4% | 1 |
| positive regulation of transport | 34 out of 9357 genes, 0.4% | 39 out of 10903 genes, 0.4% | 1 |
| response to stress | 492 out of 9357 genes, 5.3% | 573 out of 10903 genes, 5.3% | 1 |
| coenzyme metabolic process | 106 out of 9357 genes, 1.1% | 123 out of 10903 genes, 1.1% | 1 |
| negative regulation of macromolecule metabolic process | 136 out of 9357 genes, 1.5% | 158 out of 10903 genes, 1.4% | 1 |
| regulation of action potential | 16 out of 9357 genes, 0.2% | 18 out of 10903 genes, 0.2% | 1 |
| DNA-dependent DNA replication initiation | 16 out of 9357 genes, 0.2% | 18 out of 10903 genes, 0.2% | 1 |
| tRNA modification | 16 out of 9357 genes, 0.2% | 18 out of 10903 genes, 0.2% | 1 |
| protein deacetylation | 16 out of 9357 genes, 0.2% | 18 out of 10903 genes, 0.2% | 1 |
| blastoderm segmentation | 16 out of 9357 genes, 0.2% | 18 out of 10903 genes, 0.2% | 1 |
| amine biosynthetic process | 16 out of 9357 genes, 0.2% | 18 out of 10903 genes, 0.2% | 1 |
| protein deacylation | 16 out of 9357 genes, 0.2% | 18 out of 10903 genes, 0.2% | 1 |
| negative regulation of catalytic activity | 16 out of 9357 genes, 0.2% | 18 out of 10903 genes, 0.2% | 1 |
| cellular component maintenance | 16 out of 9357 genes, 0.2% | 18 out of 10903 genes, 0.2% | 1 |
| regulation of DNA metabolic process | 16 out of 9357 genes, 0.2% | 18 out of 10903 genes, 0.2% | 1 |
| cytosolic calcium ion homeostasis | 16 out of 9357 genes, 0.2% | 18 out of 10903 genes, 0.2% | 1 |
| modification-dependent protein catabolic process | 202 out of 9357 genes, 2.2% | 235 out of 10903 genes, 2.2% | 1 |
| modification-dependent macromolecule catabolic process | 202 out of 9357 genes, 2.2% | 235 out of 10903 genes, 2.2% | 1 |
| recombinational repair | 10 out of 9357 genes, 0.1% | 11 out of 10903 genes, 0.1% | 1 |
| ossification | 10 out of 9357 genes, 0.1% | 11 out of 10903 genes, 0.1% | 1 |
| positive regulation of cytokine production | 10 out of 9357 genes, 0.1% | 11 out of 10903 genes, 0.1% | 1 |
| blastocyst development | 10 out of 9357 genes, 0.1% | 11 out of 10903 genes, 0.1% | 1 |
| tRNA wobble base modification | 10 out of 9357 genes, 0.1% | 11 out of 10903 genes, 0.1% | 1 |
| epithelial cell morphogenesis | 10 out of 9357 genes, 0.1% | 11 out of 10903 genes, 0.1% | 1 |
| pentose-phosphate shunt | 10 out of 9357 genes, 0.1% | 11 out of 10903 genes, 0.1% | 1 |
| indolalkylamine metabolic process | 10 out of 9357 genes, 0.1% | 11 out of 10903 genes, 0.1% | 1 |
| NADPH regeneration | 10 out of 9357 genes, 0.1% | 11 out of 10903 genes, 0.1% | 1 |
| vesicle targeting | 10 out of 9357 genes, 0.1% | 11 out of 10903 genes, 0.1% | 1 |
| circadian rhythm | 10 out of 9357 genes, 0.1% | 11 out of 10903 genes, 0.1% | 1 |
| vitamin biosynthetic process | 10 out of 9357 genes, 0.1% | 11 out of 10903 genes, 0.1% | 1 |
| nucleobase metabolic process | 10 out of 9357 genes, 0.1% | 11 out of 10903 genes, 0.1% | 1 |
| sterol transport | 10 out of 9357 genes, 0.1% | 11 out of 10903 genes, 0.1% | 1 |
| viral reproduction | 10 out of 9357 genes, 0.1% | 11 out of 10903 genes, 0.1% | 1 |
| nicotinamide nucleotide biosynthetic process | 10 out of 9357 genes, 0.1% | 11 out of 10903 genes, 0.1% | 1 |
| pyridine nucleotide biosynthetic process | 10 out of 9357 genes, 0.1% | 11 out of 10903 genes, 0.1% | 1 |
| viral reproductive process | 10 out of 9357 genes, 0.1% | 11 out of 10903 genes, 0.1% | 1 |
| water-soluble vitamin biosynthetic process | 10 out of 9357 genes, 0.1% | 11 out of 10903 genes, 0.1% | 1 |
| indole and derivative metabolic process | 10 out of 9357 genes, 0.1% | 11 out of 10903 genes, 0.1% | 1 |
| indole derivative metabolic process | 10 out of 9357 genes, 0.1% | 11 out of 10903 genes, 0.1% | 1 |
| glycerolipid biosynthetic process | 10 out of 9357 genes, 0.1% | 11 out of 10903 genes, 0.1% | 1 |
| glycerophospholipid biosynthetic process | 10 out of 9357 genes, 0.1% | 11 out of 10903 genes, 0.1% | 1 |
| ion transport | 382 out of 9357 genes, 4.1% | 445 out of 10903 genes, 4.1% | 1 |
| hexose metabolic process | 147 out of 9357 genes, 1.6% | 171 out of 10903 genes, 1.6% | 1 |
| cuticle development | 117 out of 9357 genes, 1.3% | 136 out of 10903 genes, 1.2% | 1 |
| regulation of signaling pathway | 207 out of 9357 genes, 2.2% | 241 out of 10903 genes, 2.2% | 1 |
| post-embryonic organ development | 207 out of 9357 genes, 2.2% | 241 out of 10903 genes, 2.2% | 1 |
| response to inorganic substance | 75 out of 9357 genes, 0.8% | 87 out of 10903 genes, 0.8% | 1 |
| cellular component biogenesis | 429 out of 9357 genes, 4.6% | 500 out of 10903 genes, 4.6% | 1 |
| regulation of transferase activity | 45 out of 9357 genes, 0.5% | 52 out of 10903 genes, 0.5% | 1 |
| cellular metabolic process | 3955 out of 9357 genes, 42.3% | 4610 out of 10903 genes, 42.3% | 1 |
| nuclear-transcribed mRNA catabolic process, deadenylation-dependent decay | 4 out of 9357 genes, 0.0% | 4 out of 10903 genes, 0.0% | 1 |
| osteoblast differentiation | 4 out of 9357 genes, 0.0% | 4 out of 10903 genes, 0.0% | 1 |
| endothelial cell development | 4 out of 9357 genes, 0.0% | 4 out of 10903 genes, 0.0% | 1 |
| amino acid transmembrane transport | 4 out of 9357 genes, 0.0% | 4 out of 10903 genes, 0.0% | 1 |
| acetyl-CoA biosynthetic process from pyruvate | 4 out of 9357 genes, 0.0% | 4 out of 10903 genes, 0.0% | 1 |
| pyrimidine nucleotide catabolic process | 4 out of 9357 genes, 0.0% | 4 out of 10903 genes, 0.0% | 1 |
| protein O-linked glycosylation | 4 out of 9357 genes, 0.0% | 4 out of 10903 genes, 0.0% | 1 |
| glycoprotein catabolic process | 4 out of 9357 genes, 0.0% | 4 out of 10903 genes, 0.0% | 1 |
| alanine metabolic process | 4 out of 9357 genes, 0.0% | 4 out of 10903 genes, 0.0% | 1 |
| asparagine metabolic process | 4 out of 9357 genes, 0.0% | 4 out of 10903 genes, 0.0% | 1 |
| glutamate signaling pathway | 4 out of 9357 genes, 0.0% | 4 out of 10903 genes, 0.0% | 1 |
| tripartite regional subdivision | 4 out of 9357 genes, 0.0% | 4 out of 10903 genes, 0.0% | 1 |
| neuromuscular junction development | 4 out of 9357 genes, 0.0% | 4 out of 10903 genes, 0.0% | 1 |
| glial cell migration | 4 out of 9357 genes, 0.0% | 4 out of 10903 genes, 0.0% | 1 |
| regulation of cell shape | 4 out of 9357 genes, 0.0% | 4 out of 10903 genes, 0.0% | 1 |
| anterior/posterior axis specification, embryo | 4 out of 9357 genes, 0.0% | 4 out of 10903 genes, 0.0% | 1 |
| serine family amino acid biosynthetic process | 4 out of 9357 genes, 0.0% | 4 out of 10903 genes, 0.0% | 1 |
| pyruvate family amino acid metabolic process | 4 out of 9357 genes, 0.0% | 4 out of 10903 genes, 0.0% | 1 |
| purine nucleoside diphosphate catabolic process | 4 out of 9357 genes, 0.0% | 4 out of 10903 genes, 0.0% | 1 |
| pyrimidine nucleoside diphosphate catabolic process | 4 out of 9357 genes, 0.0% | 4 out of 10903 genes, 0.0% | 1 |
| pyrimidine nucleoside triphosphate biosynthetic process | 4 out of 9357 genes, 0.0% | 4 out of 10903 genes, 0.0% | 1 |
| purine ribonucleoside diphosphate catabolic process | 4 out of 9357 genes, 0.0% | 4 out of 10903 genes, 0.0% | 1 |
| ribonucleoside diphosphate catabolic process | 4 out of 9357 genes, 0.0% | 4 out of 10903 genes, 0.0% | 1 |
| pyrimidine ribonucleoside diphosphate metabolic process | 4 out of 9357 genes, 0.0% | 4 out of 10903 genes, 0.0% | 1 |
| pyrimidine ribonucleoside diphosphate catabolic process | 4 out of 9357 genes, 0.0% | 4 out of 10903 genes, 0.0% | 1 |
| pyrimidine ribonucleotide biosynthetic process | 4 out of 9357 genes, 0.0% | 4 out of 10903 genes, 0.0% | 1 |
| pyrimidine ribonucleotide catabolic process | 4 out of 9357 genes, 0.0% | 4 out of 10903 genes, 0.0% | 1 |
| detection of chemical stimulus | 4 out of 9357 genes, 0.0% | 4 out of 10903 genes, 0.0% | 1 |
| regulation of autophagy | 4 out of 9357 genes, 0.0% | 4 out of 10903 genes, 0.0% | 1 |
| negative regulation of autophagy | 4 out of 9357 genes, 0.0% | 4 out of 10903 genes, 0.0% | 1 |
| neuroblast differentiation | 4 out of 9357 genes, 0.0% | 4 out of 10903 genes, 0.0% | 1 |
| dopamine transport | 4 out of 9357 genes, 0.0% | 4 out of 10903 genes, 0.0% | 1 |
| immunoglobulin mediated immune response | 4 out of 9357 genes, 0.0% | 4 out of 10903 genes, 0.0% | 1 |
| iron-sulfur cluster assembly | 4 out of 9357 genes, 0.0% | 4 out of 10903 genes, 0.0% | 1 |
| histone phosphorylation | 4 out of 9357 genes, 0.0% | 4 out of 10903 genes, 0.0% | 1 |
| protein deubiquitination | 4 out of 9357 genes, 0.0% | 4 out of 10903 genes, 0.0% | 1 |
| stem cell division | 4 out of 9357 genes, 0.0% | 4 out of 10903 genes, 0.0% | 1 |
| peptidyl-serine modification | 4 out of 9357 genes, 0.0% | 4 out of 10903 genes, 0.0% | 1 |
| viral infectious cycle | 4 out of 9357 genes, 0.0% | 4 out of 10903 genes, 0.0% | 1 |
| forebrain generation of neurons | 4 out of 9357 genes, 0.0% | 4 out of 10903 genes, 0.0% | 1 |
| T cell differentiation | 4 out of 9357 genes, 0.0% | 4 out of 10903 genes, 0.0% | 1 |
| endoplasmic reticulum unfolded protein response | 4 out of 9357 genes, 0.0% | 4 out of 10903 genes, 0.0% | 1 |
| negative regulation of cell projection organization | 4 out of 9357 genes, 0.0% | 4 out of 10903 genes, 0.0% | 1 |
| negative regulation of defense response | 4 out of 9357 genes, 0.0% | 4 out of 10903 genes, 0.0% | 1 |
| regulation of type I interferon production | 4 out of 9357 genes, 0.0% | 4 out of 10903 genes, 0.0% | 1 |
| positive regulation of type I interferon production | 4 out of 9357 genes, 0.0% | 4 out of 10903 genes, 0.0% | 1 |
| endosome transport via multivesicular body sorting pathway | 4 out of 9357 genes, 0.0% | 4 out of 10903 genes, 0.0% | 1 |
| regulation of homeostatic process | 4 out of 9357 genes, 0.0% | 4 out of 10903 genes, 0.0% | 1 |
| transforming growth factor-beta2 production | 4 out of 9357 genes, 0.0% | 4 out of 10903 genes, 0.0% | 1 |
| microtubule anchoring | 4 out of 9357 genes, 0.0% | 4 out of 10903 genes, 0.0% | 1 |
| response to endoplasmic reticulum stress | 4 out of 9357 genes, 0.0% | 4 out of 10903 genes, 0.0% | 1 |
| negative regulation of protein import into nucleus | 4 out of 9357 genes, 0.0% | 4 out of 10903 genes, 0.0% | 1 |
| cellular metabolic compound salvage | 4 out of 9357 genes, 0.0% | 4 out of 10903 genes, 0.0% | 1 |
| receptor metabolic process | 4 out of 9357 genes, 0.0% | 4 out of 10903 genes, 0.0% | 1 |
| negative regulation of DNA binding | 4 out of 9357 genes, 0.0% | 4 out of 10903 genes, 0.0% | 1 |
| positive regulation of viral genome replication | 4 out of 9357 genes, 0.0% | 4 out of 10903 genes, 0.0% | 1 |
| meiotic chromosome segregation | 4 out of 9357 genes, 0.0% | 4 out of 10903 genes, 0.0% | 1 |
| endothelial cell differentiation | 4 out of 9357 genes, 0.0% | 4 out of 10903 genes, 0.0% | 1 |
| regulation of anti-apoptosis | 4 out of 9357 genes, 0.0% | 4 out of 10903 genes, 0.0% | 1 |
| negative regulation of endocytosis | 4 out of 9357 genes, 0.0% | 4 out of 10903 genes, 0.0% | 1 |
| nucleobase biosynthetic process | 4 out of 9357 genes, 0.0% | 4 out of 10903 genes, 0.0% | 1 |
| sperm competition | 4 out of 9357 genes, 0.0% | 4 out of 10903 genes, 0.0% | 1 |
| mesodermal cell differentiation | 4 out of 9357 genes, 0.0% | 4 out of 10903 genes, 0.0% | 1 |
| pigment cell differentiation | 4 out of 9357 genes, 0.0% | 4 out of 10903 genes, 0.0% | 1 |
| actin filament capping | 4 out of 9357 genes, 0.0% | 4 out of 10903 genes, 0.0% | 1 |
| regulation of calcium ion transport | 4 out of 9357 genes, 0.0% | 4 out of 10903 genes, 0.0% | 1 |
| retina development in camera-type eye | 4 out of 9357 genes, 0.0% | 4 out of 10903 genes, 0.0% | 1 |
| female mating behavior | 4 out of 9357 genes, 0.0% | 4 out of 10903 genes, 0.0% | 1 |
| branching involved in open tracheal system development | 4 out of 9357 genes, 0.0% | 4 out of 10903 genes, 0.0% | 1 |
| morphogenesis of a branching epithelium | 4 out of 9357 genes, 0.0% | 4 out of 10903 genes, 0.0% | 1 |
| basement membrane organization | 4 out of 9357 genes, 0.0% | 4 out of 10903 genes, 0.0% | 1 |
| regulation of ion homeostasis | 4 out of 9357 genes, 0.0% | 4 out of 10903 genes, 0.0% | 1 |
| positive regulation of reproductive process | 4 out of 9357 genes, 0.0% | 4 out of 10903 genes, 0.0% | 1 |
| cell projection assembly | 33 out of 9357 genes, 0.4% | 38 out of 10903 genes, 0.3% | 1 |
| nerve-nerve synaptic transmission | 27 out of 9357 genes, 0.3% | 31 out of 10903 genes, 0.3% | 1 |
| negative regulation of cell communication | 27 out of 9357 genes, 0.3% | 31 out of 10903 genes, 0.3% | 1 |
| organelle localization | 122 out of 9357 genes, 1.3% | 142 out of 10903 genes, 1.3% | 1 |
| ribonucleoprotein complex biogenesis | 110 out of 9357 genes, 1.2% | 128 out of 10903 genes, 1.2% | 1 |
| cellular component biogenesis at cellular level | 110 out of 9357 genes, 1.2% | 128 out of 10903 genes, 1.2% | 1 |
| sphingolipid metabolic process | 21 out of 9357 genes, 0.2% | 24 out of 10903 genes, 0.2% | 1 |
| vitamin metabolic process | 21 out of 9357 genes, 0.2% | 24 out of 10903 genes, 0.2% | 1 |
| negative regulation of transcription | 21 out of 9357 genes, 0.2% | 24 out of 10903 genes, 0.2% | 1 |
| adult behavior | 21 out of 9357 genes, 0.2% | 24 out of 10903 genes, 0.2% | 1 |
| regulation of ARF protein signal transduction | 21 out of 9357 genes, 0.2% | 24 out of 10903 genes, 0.2% | 1 |
| regulation of transmembrane transport | 21 out of 9357 genes, 0.2% | 24 out of 10903 genes, 0.2% | 1 |
| glycoprotein metabolic process | 68 out of 9357 genes, 0.7% | 79 out of 10903 genes, 0.7% | 1 |
| glycoprotein biosynthetic process | 62 out of 9357 genes, 0.7% | 72 out of 10903 genes, 0.7% | 1 |
| negative regulation of metabolic process | 145 out of 9357 genes, 1.5% | 169 out of 10903 genes, 1.6% | 1 |
| neutral lipid metabolic process | 15 out of 9357 genes, 0.2% | 17 out of 10903 genes, 0.2% | 1 |
| acylglycerol metabolic process | 15 out of 9357 genes, 0.2% | 17 out of 10903 genes, 0.2% | 1 |
| glycerol ether metabolic process | 15 out of 9357 genes, 0.2% | 17 out of 10903 genes, 0.2% | 1 |
| asymmetric cell division | 15 out of 9357 genes, 0.2% | 17 out of 10903 genes, 0.2% | 1 |
| response to hexose stimulus | 15 out of 9357 genes, 0.2% | 17 out of 10903 genes, 0.2% | 1 |
| response to organic cyclic substance | 15 out of 9357 genes, 0.2% | 17 out of 10903 genes, 0.2% | 1 |
| organic ether metabolic process | 15 out of 9357 genes, 0.2% | 17 out of 10903 genes, 0.2% | 1 |
| regulation of lipid metabolic process | 15 out of 9357 genes, 0.2% | 17 out of 10903 genes, 0.2% | 1 |
| pallium development | 15 out of 9357 genes, 0.2% | 17 out of 10903 genes, 0.2% | 1 |
| RNA 3'-end processing | 15 out of 9357 genes, 0.2% | 17 out of 10903 genes, 0.2% | 1 |
| response to monosaccharide stimulus | 15 out of 9357 genes, 0.2% | 17 out of 10903 genes, 0.2% | 1 |
| regulation of MAP kinase activity | 15 out of 9357 genes, 0.2% | 17 out of 10903 genes, 0.2% | 1 |
| negative regulation of cell cycle | 15 out of 9357 genes, 0.2% | 17 out of 10903 genes, 0.2% | 1 |
| metal ion homeostasis | 50 out of 9357 genes, 0.5% | 58 out of 10903 genes, 0.5% | 1 |
| cell cycle phase | 306 out of 9357 genes, 3.3% | 357 out of 10903 genes, 3.3% | 1 |
| regulation of catabolic process | 115 out of 9357 genes, 1.2% | 134 out of 10903 genes, 1.2% | 1 |
| response to metal ion | 44 out of 9357 genes, 0.5% | 51 out of 10903 genes, 0.5% | 1 |
| cardiovascular system development | 44 out of 9357 genes, 0.5% | 51 out of 10903 genes, 0.5% | 1 |
| circulatory system development | 44 out of 9357 genes, 0.5% | 51 out of 10903 genes, 0.5% | 1 |
| cellular biosynthetic process | 1159 out of 9357 genes, 12.4% | 1352 out of 10903 genes, 12.4% | 1 |
| cellular macromolecule biosynthetic process | 870 out of 9357 genes, 9.3% | 1015 out of 10903 genes, 9.3% | 1 |
| negative regulation of cellular process | 198 out of 9357 genes, 2.1% | 231 out of 10903 genes, 2.1% | 1 |
| cell death | 186 out of 9357 genes, 2.0% | 217 out of 10903 genes, 2.0% | 1 |
| death | 186 out of 9357 genes, 2.0% | 217 out of 10903 genes, 2.0% | 1 |
| monosaccharide metabolic process | 156 out of 9357 genes, 1.7% | 182 out of 10903 genes, 1.7% | 1 |
| multicellular organismal aging | 526 out of 9357 genes, 5.6% | 614 out of 10903 genes, 5.6% | 1 |
| spliceosome assembly | 9 out of 9357 genes, 0.1% | 10 out of 10903 genes, 0.1% | 1 |
| epithelial cell morphogenesis involved in gastrulation | 9 out of 9357 genes, 0.1% | 10 out of 10903 genes, 0.1% | 1 |
| DNA modification | 9 out of 9357 genes, 0.1% | 10 out of 10903 genes, 0.1% | 1 |
| DNA alkylation | 9 out of 9357 genes, 0.1% | 10 out of 10903 genes, 0.1% | 1 |
| male meiosis | 9 out of 9357 genes, 0.1% | 10 out of 10903 genes, 0.1% | 1 |
| positive regulation of intracellular protein kinase cascade | 9 out of 9357 genes, 0.1% | 10 out of 10903 genes, 0.1% | 1 |
| myeloid cell differentiation | 9 out of 9357 genes, 0.1% | 10 out of 10903 genes, 0.1% | 1 |
| cholesterol transport | 9 out of 9357 genes, 0.1% | 10 out of 10903 genes, 0.1% | 1 |
| regulation of transporter activity | 9 out of 9357 genes, 0.1% | 10 out of 10903 genes, 0.1% | 1 |
| photoreceptor cell development | 9 out of 9357 genes, 0.1% | 10 out of 10903 genes, 0.1% | 1 |
| interphase of mitotic cell cycle | 9 out of 9357 genes, 0.1% | 10 out of 10903 genes, 0.1% | 1 |
| response to protein stimulus | 9 out of 9357 genes, 0.1% | 10 out of 10903 genes, 0.1% | 1 |
| regulation of muscle system process | 9 out of 9357 genes, 0.1% | 10 out of 10903 genes, 0.1% | 1 |
| membrane budding | 26 out of 9357 genes, 0.3% | 30 out of 10903 genes, 0.3% | 1 |
| glucose metabolic process | 108 out of 9357 genes, 1.2% | 126 out of 10903 genes, 1.2% | 1 |
| biosynthetic process | 1204 out of 9357 genes, 12.9% | 1405 out of 10903 genes, 12.9% | 1 |
| oxidation reduction | 55 out of 9357 genes, 0.6% | 64 out of 10903 genes, 0.6% | 1 |
| divalent metal ion transport | 55 out of 9357 genes, 0.6% | 64 out of 10903 genes, 0.6% | 1 |
| regulation of response to stimulus | 96 out of 9357 genes, 1.0% | 112 out of 10903 genes, 1.0% | 1 |
| regulation of cellular component biogenesis | 49 out of 9357 genes, 0.5% | 57 out of 10903 genes, 0.5% | 1 |
| cellular nitrogen compound catabolic process | 49 out of 9357 genes, 0.5% | 57 out of 10903 genes, 0.5% | 1 |
| cation transport | 250 out of 9357 genes, 2.7% | 292 out of 10903 genes, 2.7% | 1 |
| phospholipid metabolic process | 84 out of 9357 genes, 0.9% | 98 out of 10903 genes, 0.9% | 1 |
| protein catabolic process | 214 out of 9357 genes, 2.3% | 250 out of 10903 genes, 2.3% | 1 |
| regulation of kinase activity | 43 out of 9357 genes, 0.5% | 50 out of 10903 genes, 0.5% | 1 |
| regulation of tube size | 20 out of 9357 genes, 0.2% | 23 out of 10903 genes, 0.2% | 1 |
| negative regulation of transcription, DNA-dependent | 20 out of 9357 genes, 0.2% | 23 out of 10903 genes, 0.2% | 1 |
| positive regulation of developmental process | 20 out of 9357 genes, 0.2% | 23 out of 10903 genes, 0.2% | 1 |
| cellular aromatic compound metabolic process | 37 out of 9357 genes, 0.4% | 43 out of 10903 genes, 0.4% | 1 |
| regulation of protein ubiquitination | 37 out of 9357 genes, 0.4% | 43 out of 10903 genes, 0.4% | 1 |
| gene silencing | 107 out of 9357 genes, 1.1% | 125 out of 10903 genes, 1.1% | 1 |
| nucleic acid metabolic process | 1375 out of 9357 genes, 14.7% | 1605 out of 10903 genes, 14.7% | 1 |
| organophosphate metabolic process | 89 out of 9357 genes, 1.0% | 104 out of 10903 genes, 1.0% | 1 |
| photoreceptor cell differentiation | 31 out of 9357 genes, 0.3% | 36 out of 10903 genes, 0.3% | 1 |
| RNA modification | 54 out of 9357 genes, 0.6% | 63 out of 10903 genes, 0.6% | 1 |
| ribonucleoside monophosphate biosynthetic process | 14 out of 9357 genes, 0.1% | 16 out of 10903 genes, 0.1% | 1 |
| ribonucleoside monophosphate metabolic process | 14 out of 9357 genes, 0.1% | 16 out of 10903 genes, 0.1% | 1 |
| histone methylation | 14 out of 9357 genes, 0.1% | 16 out of 10903 genes, 0.1% | 1 |
| regulation of protein import into nucleus | 14 out of 9357 genes, 0.1% | 16 out of 10903 genes, 0.1% | 1 |
| positive regulation of DNA binding | 14 out of 9357 genes, 0.1% | 16 out of 10903 genes, 0.1% | 1 |
| negative regulation of gene expression | 124 out of 9357 genes, 1.3% | 145 out of 10903 genes, 1.3% | 1 |
| apoptosis | 77 out of 9357 genes, 0.8% | 90 out of 10903 genes, 0.8% | 1 |
| positive regulation of biosynthetic process | 48 out of 9357 genes, 0.5% | 56 out of 10903 genes, 0.5% | 1 |
| response to nutrient levels | 48 out of 9357 genes, 0.5% | 56 out of 10903 genes, 0.5% | 1 |
| regulation of organelle organization | 71 out of 9357 genes, 0.8% | 83 out of 10903 genes, 0.8% | 1 |
| asymmetric protein localization | 25 out of 9357 genes, 0.3% | 29 out of 10903 genes, 0.3% | 1 |
| glutamine family amino acid metabolic process | 25 out of 9357 genes, 0.3% | 29 out of 10903 genes, 0.3% | 1 |
| negative regulation of cellular biosynthetic process | 25 out of 9357 genes, 0.3% | 29 out of 10903 genes, 0.3% | 1 |
| regulation of cell projection organization | 25 out of 9357 genes, 0.3% | 29 out of 10903 genes, 0.3% | 1 |
| regulation of intracellular transport | 25 out of 9357 genes, 0.3% | 29 out of 10903 genes, 0.3% | 1 |
| regulation of protein kinase activity | 25 out of 9357 genes, 0.3% | 29 out of 10903 genes, 0.3% | 1 |
| negative regulation of cellular component organization | 25 out of 9357 genes, 0.3% | 29 out of 10903 genes, 0.3% | 1 |
| signaling process | 873 out of 9357 genes, 9.3% | 1020 out of 10903 genes, 9.4% | 1 |
| cellular biogenic amine metabolic process | 36 out of 9357 genes, 0.4% | 42 out of 10903 genes, 0.4% | 1 |
| dicarboxylic acid metabolic process | 36 out of 9357 genes, 0.4% | 42 out of 10903 genes, 0.4% | 1 |
| intracellular transport | 264 out of 9357 genes, 2.8% | 309 out of 10903 genes, 2.8% | 1 |
| ribonucleotide biosynthetic process | 111 out of 9357 genes, 1.2% | 130 out of 10903 genes, 1.2% | 1 |
| nucleobase, nucleoside, nucleotide and nucleic acid metabolic process | 1639 out of 9357 genes, 17.5% | 1914 out of 10903 genes, 17.6% | 1 |
| cation homeostasis | 76 out of 9357 genes, 0.8% | 89 out of 10903 genes, 0.8% | 1 |
| regulation of translation | 19 out of 9357 genes, 0.2% | 22 out of 10903 genes, 0.2% | 1 |
| amino acid transport | 19 out of 9357 genes, 0.2% | 22 out of 10903 genes, 0.2% | 1 |
| alditol metabolic process | 19 out of 9357 genes, 0.2% | 22 out of 10903 genes, 0.2% | 1 |
| regulation of intracellular protein transport | 19 out of 9357 genes, 0.2% | 22 out of 10903 genes, 0.2% | 1 |
| positive regulation of cell differentiation | 19 out of 9357 genes, 0.2% | 22 out of 10903 genes, 0.2% | 1 |
| regulation of protein transport | 19 out of 9357 genes, 0.2% | 22 out of 10903 genes, 0.2% | 1 |
| regulation of establishment of protein localization | 19 out of 9357 genes, 0.2% | 22 out of 10903 genes, 0.2% | 1 |
| signal transmission | 871 out of 9357 genes, 9.3% | 1018 out of 10903 genes, 9.3% | 1 |
| nucleoside triphosphate biosynthetic process | 99 out of 9357 genes, 1.1% | 116 out of 10903 genes, 1.1% | 1 |
| nucleotide catabolic process | 47 out of 9357 genes, 0.5% | 55 out of 10903 genes, 0.5% | 1 |
| response to light stimulus | 47 out of 9357 genes, 0.5% | 55 out of 10903 genes, 0.5% | 1 |
| positive regulation of macromolecule biosynthetic process | 47 out of 9357 genes, 0.5% | 55 out of 10903 genes, 0.5% | 1 |
| nucleobase, nucleoside, nucleotide and nucleic acid catabolic process | 47 out of 9357 genes, 0.5% | 55 out of 10903 genes, 0.5% | 1 |
| nucleobase, nucleoside and nucleotide catabolic process | 47 out of 9357 genes, 0.5% | 55 out of 10903 genes, 0.5% | 1 |
| melanin metabolic process | 8 out of 9357 genes, 0.1% | 9 out of 10903 genes, 0.1% | 1 |
| catecholamine metabolic process | 8 out of 9357 genes, 0.1% | 9 out of 10903 genes, 0.1% | 1 |
| sphingomyelin metabolic process | 8 out of 9357 genes, 0.1% | 9 out of 10903 genes, 0.1% | 1 |
| autophagy | 8 out of 9357 genes, 0.1% | 9 out of 10903 genes, 0.1% | 1 |
| regulation of muscle contraction | 8 out of 9357 genes, 0.1% | 9 out of 10903 genes, 0.1% | 1 |
| detection of external stimulus | 8 out of 9357 genes, 0.1% | 9 out of 10903 genes, 0.1% | 1 |
| detection of abiotic stimulus | 8 out of 9357 genes, 0.1% | 9 out of 10903 genes, 0.1% | 1 |
| catechol metabolic process | 8 out of 9357 genes, 0.1% | 9 out of 10903 genes, 0.1% | 1 |
| histone ubiquitination | 8 out of 9357 genes, 0.1% | 9 out of 10903 genes, 0.1% | 1 |
| regulation of exocytosis | 8 out of 9357 genes, 0.1% | 9 out of 10903 genes, 0.1% | 1 |
| phenol metabolic process | 8 out of 9357 genes, 0.1% | 9 out of 10903 genes, 0.1% | 1 |
| aromatic compound biosynthetic process | 8 out of 9357 genes, 0.1% | 9 out of 10903 genes, 0.1% | 1 |
| NAD metabolic process | 8 out of 9357 genes, 0.1% | 9 out of 10903 genes, 0.1% | 1 |
| metencephalon development | 8 out of 9357 genes, 0.1% | 9 out of 10903 genes, 0.1% | 1 |
| regulation of defense response | 8 out of 9357 genes, 0.1% | 9 out of 10903 genes, 0.1% | 1 |
| response to insulin stimulus | 8 out of 9357 genes, 0.1% | 9 out of 10903 genes, 0.1% | 1 |
| diol metabolic process | 8 out of 9357 genes, 0.1% | 9 out of 10903 genes, 0.1% | 1 |
| pteridine and derivative metabolic process | 8 out of 9357 genes, 0.1% | 9 out of 10903 genes, 0.1% | 1 |
| pteridine and derivative biosynthetic process | 8 out of 9357 genes, 0.1% | 9 out of 10903 genes, 0.1% | 1 |
| positive regulation of MAPKKK cascade | 8 out of 9357 genes, 0.1% | 9 out of 10903 genes, 0.1% | 1 |
| centrosome organization | 8 out of 9357 genes, 0.1% | 9 out of 10903 genes, 0.1% | 1 |
| nucleic acid phosphodiester bond hydrolysis | 8 out of 9357 genes, 0.1% | 9 out of 10903 genes, 0.1% | 1 |
| chordate embryonic development | 41 out of 9357 genes, 0.4% | 48 out of 10903 genes, 0.4% | 1 |
| cellular macromolecular complex assembly | 81 out of 9357 genes, 0.9% | 95 out of 10903 genes, 0.9% | 1 |
| G1 phase of mitotic cell cycle | 3 out of 9357 genes, 0.0% | 3 out of 10903 genes, 0.0% | 1 |
| G2/M transition of mitotic cell cycle | 3 out of 9357 genes, 0.0% | 3 out of 10903 genes, 0.0% | 1 |
| metanephros development | 3 out of 9357 genes, 0.0% | 3 out of 10903 genes, 0.0% | 1 |
| optic placode formation | 3 out of 9357 genes, 0.0% | 3 out of 10903 genes, 0.0% | 1 |
| blastocyst formation | 3 out of 9357 genes, 0.0% | 3 out of 10903 genes, 0.0% | 1 |
| columnar/cuboidal epithelial cell differentiation | 3 out of 9357 genes, 0.0% | 3 out of 10903 genes, 0.0% | 1 |
| glandular epithelial cell differentiation | 3 out of 9357 genes, 0.0% | 3 out of 10903 genes, 0.0% | 1 |
| instar larval development | 3 out of 9357 genes, 0.0% | 3 out of 10903 genes, 0.0% | 1 |
| somatic recombination of immunoglobulin genes involved in immune response | 3 out of 9357 genes, 0.0% | 3 out of 10903 genes, 0.0% | 1 |
| somatic diversification of immunoglobulins involved in immune response | 3 out of 9357 genes, 0.0% | 3 out of 10903 genes, 0.0% | 1 |
| immunoglobulin production involved in immunoglobulin mediated immune response | 3 out of 9357 genes, 0.0% | 3 out of 10903 genes, 0.0% | 1 |
| positive regulation of leukocyte activation | 3 out of 9357 genes, 0.0% | 3 out of 10903 genes, 0.0% | 1 |
| regulation of cilium movement | 3 out of 9357 genes, 0.0% | 3 out of 10903 genes, 0.0% | 1 |
| neural retina development | 3 out of 9357 genes, 0.0% | 3 out of 10903 genes, 0.0% | 1 |
| regulation of glycogen biosynthetic process | 3 out of 9357 genes, 0.0% | 3 out of 10903 genes, 0.0% | 1 |
| uronic acid metabolic process | 3 out of 9357 genes, 0.0% | 3 out of 10903 genes, 0.0% | 1 |
| pyrimidine base metabolic process | 3 out of 9357 genes, 0.0% | 3 out of 10903 genes, 0.0% | 1 |
| RNA-dependent DNA replication | 3 out of 9357 genes, 0.0% | 3 out of 10903 genes, 0.0% | 1 |
| base-excision repair | 3 out of 9357 genes, 0.0% | 3 out of 10903 genes, 0.0% | 1 |
| chromatin assembly or disassembly | 3 out of 9357 genes, 0.0% | 3 out of 10903 genes, 0.0% | 1 |
| translational termination | 3 out of 9357 genes, 0.0% | 3 out of 10903 genes, 0.0% | 1 |
| cysteine metabolic process | 3 out of 9357 genes, 0.0% | 3 out of 10903 genes, 0.0% | 1 |
| glutamine metabolic process | 3 out of 9357 genes, 0.0% | 3 out of 10903 genes, 0.0% | 1 |
| histidine metabolic process | 3 out of 9357 genes, 0.0% | 3 out of 10903 genes, 0.0% | 1 |
| ethanolamine metabolic process | 3 out of 9357 genes, 0.0% | 3 out of 10903 genes, 0.0% | 1 |
| polyamine biosynthetic process | 3 out of 9357 genes, 0.0% | 3 out of 10903 genes, 0.0% | 1 |
| glycosylceramide metabolic process | 3 out of 9357 genes, 0.0% | 3 out of 10903 genes, 0.0% | 1 |
| terpenoid metabolic process | 3 out of 9357 genes, 0.0% | 3 out of 10903 genes, 0.0% | 1 |
| potassium ion transport | 3 out of 9357 genes, 0.0% | 3 out of 10903 genes, 0.0% | 1 |
| regulation of striated muscle contraction | 3 out of 9357 genes, 0.0% | 3 out of 10903 genes, 0.0% | 1 |
| hyperosmotic response | 3 out of 9357 genes, 0.0% | 3 out of 10903 genes, 0.0% | 1 |
| lysosome organization | 3 out of 9357 genes, 0.0% | 3 out of 10903 genes, 0.0% | 1 |
| meiotic prophase I | 3 out of 9357 genes, 0.0% | 3 out of 10903 genes, 0.0% | 1 |
| tyrosine phosphorylation of STAT protein | 3 out of 9357 genes, 0.0% | 3 out of 10903 genes, 0.0% | 1 |
| salivary gland morphogenesis | 3 out of 9357 genes, 0.0% | 3 out of 10903 genes, 0.0% | 1 |
| digestion | 3 out of 9357 genes, 0.0% | 3 out of 10903 genes, 0.0% | 1 |
| germ cell migration | 3 out of 9357 genes, 0.0% | 3 out of 10903 genes, 0.0% | 1 |
| histidine family amino acid metabolic process | 3 out of 9357 genes, 0.0% | 3 out of 10903 genes, 0.0% | 1 |
| nucleoside diphosphate biosynthetic process | 3 out of 9357 genes, 0.0% | 3 out of 10903 genes, 0.0% | 1 |
| deoxyribonucleoside monophosphate biosynthetic process | 3 out of 9357 genes, 0.0% | 3 out of 10903 genes, 0.0% | 1 |
| deoxyribonucleoside monophosphate metabolic process | 3 out of 9357 genes, 0.0% | 3 out of 10903 genes, 0.0% | 1 |
| pyrimidine deoxyribonucleoside monophosphate metabolic process | 3 out of 9357 genes, 0.0% | 3 out of 10903 genes, 0.0% | 1 |
| pyrimidine deoxyribonucleoside monophosphate biosynthetic process | 3 out of 9357 genes, 0.0% | 3 out of 10903 genes, 0.0% | 1 |
| cellular response to starvation | 3 out of 9357 genes, 0.0% | 3 out of 10903 genes, 0.0% | 1 |
| gamma-aminobutyric acid metabolic process | 3 out of 9357 genes, 0.0% | 3 out of 10903 genes, 0.0% | 1 |
| cardioblast differentiation | 3 out of 9357 genes, 0.0% | 3 out of 10903 genes, 0.0% | 1 |
| positive regulation of gene expression | 3 out of 9357 genes, 0.0% | 3 out of 10903 genes, 0.0% | 1 |
| regulation of glucan biosynthetic process | 3 out of 9357 genes, 0.0% | 3 out of 10903 genes, 0.0% | 1 |
| organic cation transport | 3 out of 9357 genes, 0.0% | 3 out of 10903 genes, 0.0% | 1 |
| quaternary ammonium group transport | 3 out of 9357 genes, 0.0% | 3 out of 10903 genes, 0.0% | 1 |
| acidic amino acid transport | 3 out of 9357 genes, 0.0% | 3 out of 10903 genes, 0.0% | 1 |
| thiamin transport | 3 out of 9357 genes, 0.0% | 3 out of 10903 genes, 0.0% | 1 |
| morphogenesis of follicular epithelium | 3 out of 9357 genes, 0.0% | 3 out of 10903 genes, 0.0% | 1 |
| establishment or maintenance of polarity of follicular epithelium | 3 out of 9357 genes, 0.0% | 3 out of 10903 genes, 0.0% | 1 |
| somatic recombination of immunoglobulin gene segments | 3 out of 9357 genes, 0.0% | 3 out of 10903 genes, 0.0% | 1 |
| histone demethylation | 3 out of 9357 genes, 0.0% | 3 out of 10903 genes, 0.0% | 1 |
| negative regulation of translation | 3 out of 9357 genes, 0.0% | 3 out of 10903 genes, 0.0% | 1 |
| peptidyl-diphthamide metabolic process | 3 out of 9357 genes, 0.0% | 3 out of 10903 genes, 0.0% | 1 |
| peptidyl-serine phosphorylation | 3 out of 9357 genes, 0.0% | 3 out of 10903 genes, 0.0% | 1 |
| peptidyl-tyrosine phosphorylation | 3 out of 9357 genes, 0.0% | 3 out of 10903 genes, 0.0% | 1 |
| protein nucleotidylation | 3 out of 9357 genes, 0.0% | 3 out of 10903 genes, 0.0% | 1 |
| peptidyl-histidine modification | 3 out of 9357 genes, 0.0% | 3 out of 10903 genes, 0.0% | 1 |
| peptidyl-tyrosine modification | 3 out of 9357 genes, 0.0% | 3 out of 10903 genes, 0.0% | 1 |
| protein prenylation | 3 out of 9357 genes, 0.0% | 3 out of 10903 genes, 0.0% | 1 |
| virus-host interaction | 3 out of 9357 genes, 0.0% | 3 out of 10903 genes, 0.0% | 1 |
| cysteine biosynthetic process | 3 out of 9357 genes, 0.0% | 3 out of 10903 genes, 0.0% | 1 |
| glucuronate metabolic process | 3 out of 9357 genes, 0.0% | 3 out of 10903 genes, 0.0% | 1 |
| antigen processing and presentation | 3 out of 9357 genes, 0.0% | 3 out of 10903 genes, 0.0% | 1 |
| subpallium development | 3 out of 9357 genes, 0.0% | 3 out of 10903 genes, 0.0% | 1 |
| rhombomere development | 3 out of 9357 genes, 0.0% | 3 out of 10903 genes, 0.0% | 1 |
| ventricular system development | 3 out of 9357 genes, 0.0% | 3 out of 10903 genes, 0.0% | 1 |
| cranial nerve morphogenesis | 3 out of 9357 genes, 0.0% | 3 out of 10903 genes, 0.0% | 1 |
| glial cell fate commitment | 3 out of 9357 genes, 0.0% | 3 out of 10903 genes, 0.0% | 1 |
| central nervous system neuron development | 3 out of 9357 genes, 0.0% | 3 out of 10903 genes, 0.0% | 1 |
| central nervous system neuron axonogenesis | 3 out of 9357 genes, 0.0% | 3 out of 10903 genes, 0.0% | 1 |
| molting cycle process | 3 out of 9357 genes, 0.0% | 3 out of 10903 genes, 0.0% | 1 |
| gland morphogenesis | 3 out of 9357 genes, 0.0% | 3 out of 10903 genes, 0.0% | 1 |
| negative regulation of Wnt receptor signaling pathway | 3 out of 9357 genes, 0.0% | 3 out of 10903 genes, 0.0% | 1 |
| regulation of ossification | 3 out of 9357 genes, 0.0% | 3 out of 10903 genes, 0.0% | 1 |
| lung development | 3 out of 9357 genes, 0.0% | 3 out of 10903 genes, 0.0% | 1 |
| BMP signaling pathway | 3 out of 9357 genes, 0.0% | 3 out of 10903 genes, 0.0% | 1 |
| eggshell formation | 3 out of 9357 genes, 0.0% | 3 out of 10903 genes, 0.0% | 1 |
| myosin filament assembly | 3 out of 9357 genes, 0.0% | 3 out of 10903 genes, 0.0% | 1 |
| positive regulation of defense response | 3 out of 9357 genes, 0.0% | 3 out of 10903 genes, 0.0% | 1 |
| N-terminal protein amino acid modification | 3 out of 9357 genes, 0.0% | 3 out of 10903 genes, 0.0% | 1 |
| spindle checkpoint | 3 out of 9357 genes, 0.0% | 3 out of 10903 genes, 0.0% | 1 |
| positive regulation of neurological system process | 3 out of 9357 genes, 0.0% | 3 out of 10903 genes, 0.0% | 1 |
| regulation of calcium ion transport via store-operated calcium channel activity | 3 out of 9357 genes, 0.0% | 3 out of 10903 genes, 0.0% | 1 |
| regulation of polysaccharide metabolic process | 3 out of 9357 genes, 0.0% | 3 out of 10903 genes, 0.0% | 1 |
| regulation of polysaccharide biosynthetic process | 3 out of 9357 genes, 0.0% | 3 out of 10903 genes, 0.0% | 1 |
| mononuclear cell proliferation | 3 out of 9357 genes, 0.0% | 3 out of 10903 genes, 0.0% | 1 |
| nucleotide-excision repair, DNA incision | 3 out of 9357 genes, 0.0% | 3 out of 10903 genes, 0.0% | 1 |
| cellular hormone metabolic process | 3 out of 9357 genes, 0.0% | 3 out of 10903 genes, 0.0% | 1 |
| melanization defense response | 3 out of 9357 genes, 0.0% | 3 out of 10903 genes, 0.0% | 1 |
| leading edge cell differentiation | 3 out of 9357 genes, 0.0% | 3 out of 10903 genes, 0.0% | 1 |
| maintenance of protein localization in endoplasmic reticulum | 3 out of 9357 genes, 0.0% | 3 out of 10903 genes, 0.0% | 1 |
| regulation of meiosis | 3 out of 9357 genes, 0.0% | 3 out of 10903 genes, 0.0% | 1 |
| cardioblast cell fate commitment | 3 out of 9357 genes, 0.0% | 3 out of 10903 genes, 0.0% | 1 |
| thiamin and derivative metabolic process | 3 out of 9357 genes, 0.0% | 3 out of 10903 genes, 0.0% | 1 |
| purine salvage | 3 out of 9357 genes, 0.0% | 3 out of 10903 genes, 0.0% | 1 |
| regulation of carbohydrate biosynthetic process | 3 out of 9357 genes, 0.0% | 3 out of 10903 genes, 0.0% | 1 |
| negative regulation of ion transport | 3 out of 9357 genes, 0.0% | 3 out of 10903 genes, 0.0% | 1 |
| regulation of generation of precursor metabolites and energy | 3 out of 9357 genes, 0.0% | 3 out of 10903 genes, 0.0% | 1 |
| histone H4 acetylation | 3 out of 9357 genes, 0.0% | 3 out of 10903 genes, 0.0% | 1 |
| innate immune response | 3 out of 9357 genes, 0.0% | 3 out of 10903 genes, 0.0% | 1 |
| regulation of T cell differentiation | 3 out of 9357 genes, 0.0% | 3 out of 10903 genes, 0.0% | 1 |
| positive regulation of T cell differentiation | 3 out of 9357 genes, 0.0% | 3 out of 10903 genes, 0.0% | 1 |
| regulation of lymphocyte differentiation | 3 out of 9357 genes, 0.0% | 3 out of 10903 genes, 0.0% | 1 |
| positive regulation of lymphocyte differentiation | 3 out of 9357 genes, 0.0% | 3 out of 10903 genes, 0.0% | 1 |
| positive regulation of myeloid cell differentiation | 3 out of 9357 genes, 0.0% | 3 out of 10903 genes, 0.0% | 1 |
| regulation of salivary gland boundary specification | 3 out of 9357 genes, 0.0% | 3 out of 10903 genes, 0.0% | 1 |
| regulation of angiogenesis | 3 out of 9357 genes, 0.0% | 3 out of 10903 genes, 0.0% | 1 |
| positive regulation of cell cycle | 3 out of 9357 genes, 0.0% | 3 out of 10903 genes, 0.0% | 1 |
| ethanolamine biosynthetic process | 3 out of 9357 genes, 0.0% | 3 out of 10903 genes, 0.0% | 1 |
| UDP-glucuronate metabolic process | 3 out of 9357 genes, 0.0% | 3 out of 10903 genes, 0.0% | 1 |
| regulation of organ growth | 3 out of 9357 genes, 0.0% | 3 out of 10903 genes, 0.0% | 1 |
| anatomical structure arrangement | 3 out of 9357 genes, 0.0% | 3 out of 10903 genes, 0.0% | 1 |
| oligodendrocyte differentiation | 3 out of 9357 genes, 0.0% | 3 out of 10903 genes, 0.0% | 1 |
| sensory system development | 3 out of 9357 genes, 0.0% | 3 out of 10903 genes, 0.0% | 1 |
| lateral line system development | 3 out of 9357 genes, 0.0% | 3 out of 10903 genes, 0.0% | 1 |
| regulation of phagocytosis | 3 out of 9357 genes, 0.0% | 3 out of 10903 genes, 0.0% | 1 |
| negative regulation of phagocytosis | 3 out of 9357 genes, 0.0% | 3 out of 10903 genes, 0.0% | 1 |
| positive regulation of phagocytosis | 3 out of 9357 genes, 0.0% | 3 out of 10903 genes, 0.0% | 1 |
| positive regulation of synaptic transmission | 3 out of 9357 genes, 0.0% | 3 out of 10903 genes, 0.0% | 1 |
| regulation of coagulation | 3 out of 9357 genes, 0.0% | 3 out of 10903 genes, 0.0% | 1 |
| regulation of T cell activation | 3 out of 9357 genes, 0.0% | 3 out of 10903 genes, 0.0% | 1 |
| positive regulation of cell activation | 3 out of 9357 genes, 0.0% | 3 out of 10903 genes, 0.0% | 1 |
| positive regulation of T cell activation | 3 out of 9357 genes, 0.0% | 3 out of 10903 genes, 0.0% | 1 |
| leukocyte migration | 3 out of 9357 genes, 0.0% | 3 out of 10903 genes, 0.0% | 1 |
| positive regulation of lymphocyte activation | 3 out of 9357 genes, 0.0% | 3 out of 10903 genes, 0.0% | 1 |
| positive regulation of RNA metabolic process | 3 out of 9357 genes, 0.0% | 3 out of 10903 genes, 0.0% | 1 |
| prophase | 3 out of 9357 genes, 0.0% | 3 out of 10903 genes, 0.0% | 1 |
| membrane depolarization | 3 out of 9357 genes, 0.0% | 3 out of 10903 genes, 0.0% | 1 |
| positive regulation of transmission of nerve impulse | 3 out of 9357 genes, 0.0% | 3 out of 10903 genes, 0.0% | 1 |
| regulation of cilium movement involved in cell motility | 3 out of 9357 genes, 0.0% | 3 out of 10903 genes, 0.0% | 1 |
| regulation of microtubule-based movement | 3 out of 9357 genes, 0.0% | 3 out of 10903 genes, 0.0% | 1 |
| ectodermal placode formation | 3 out of 9357 genes, 0.0% | 3 out of 10903 genes, 0.0% | 1 |
| cardiac cell fate commitment | 3 out of 9357 genes, 0.0% | 3 out of 10903 genes, 0.0% | 1 |
| histone lysine demethylation | 3 out of 9357 genes, 0.0% | 3 out of 10903 genes, 0.0% | 1 |
| leukocyte proliferation | 3 out of 9357 genes, 0.0% | 3 out of 10903 genes, 0.0% | 1 |
| regulation of glycogen metabolic process | 3 out of 9357 genes, 0.0% | 3 out of 10903 genes, 0.0% | 1 |
| ectodermal placode development | 3 out of 9357 genes, 0.0% | 3 out of 10903 genes, 0.0% | 1 |
| ectodermal placode morphogenesis | 3 out of 9357 genes, 0.0% | 3 out of 10903 genes, 0.0% | 1 |
| nephron development | 3 out of 9357 genes, 0.0% | 3 out of 10903 genes, 0.0% | 1 |
| cellular component assembly | 333 out of 9357 genes, 3.6% | 390 out of 10903 genes, 3.6% | 1 |
| cellular nitrogen compound biosynthetic process | 215 out of 9357 genes, 2.3% | 252 out of 10903 genes, 2.3% | 1 |
| protein localization | 392 out of 9357 genes, 4.2% | 459 out of 10903 genes, 4.2% | 1 |
| cellular protein catabolic process | 209 out of 9357 genes, 2.2% | 245 out of 10903 genes, 2.2% | 1 |
| myofibril assembly | 24 out of 9357 genes, 0.3% | 28 out of 10903 genes, 0.3% | 1 |
| cellular polysaccharide metabolic process | 24 out of 9357 genes, 0.3% | 28 out of 10903 genes, 0.3% | 1 |
| regionalization | 98 out of 9357 genes, 1.0% | 115 out of 10903 genes, 1.1% | 1 |
| cellular carbohydrate metabolic process | 244 out of 9357 genes, 2.6% | 286 out of 10903 genes, 2.6% | 1 |
| anion transport | 52 out of 9357 genes, 0.6% | 61 out of 10903 genes, 0.6% | 1 |
| nuclear migration | 52 out of 9357 genes, 0.6% | 61 out of 10903 genes, 0.6% | 1 |
| axis specification | 35 out of 9357 genes, 0.4% | 41 out of 10903 genes, 0.4% | 1 |
| cellular respiration | 92 out of 9357 genes, 1.0% | 108 out of 10903 genes, 1.0% | 1 |
| regulation of Ras protein signal transduction | 92 out of 9357 genes, 1.0% | 108 out of 10903 genes, 1.0% | 1 |
| regulation of small GTPase mediated signal transduction | 92 out of 9357 genes, 1.0% | 108 out of 10903 genes, 1.0% | 1 |
| sister chromatid cohesion | 13 out of 9357 genes, 0.1% | 15 out of 10903 genes, 0.1% | 1 |
| mRNA 3'-end processing | 13 out of 9357 genes, 0.1% | 15 out of 10903 genes, 0.1% | 1 |
| response to abiotic stimulus | 144 out of 9357 genes, 1.5% | 169 out of 10903 genes, 1.6% | 1 |
| nucleotide metabolic process | 255 out of 9357 genes, 2.7% | 299 out of 10903 genes, 2.7% | 1 |
| proteolysis involved in cellular protein catabolic process | 208 out of 9357 genes, 2.2% | 244 out of 10903 genes, 2.2% | 1 |
| organic substance transport | 103 out of 9357 genes, 1.1% | 121 out of 10903 genes, 1.1% | 1 |
| small GTPase mediated signal transduction | 126 out of 9357 genes, 1.3% | 148 out of 10903 genes, 1.4% | 1 |
| heterocycle biosynthetic process | 29 out of 9357 genes, 0.3% | 34 out of 10903 genes, 0.3% | 1 |
| membrane docking | 29 out of 9357 genes, 0.3% | 34 out of 10903 genes, 0.3% | 1 |
| transmembrane transport | 29 out of 9357 genes, 0.3% | 34 out of 10903 genes, 0.3% | 1 |
| ribonucleoside triphosphate biosynthetic process | 97 out of 9357 genes, 1.0% | 114 out of 10903 genes, 1.0% | 1 |
| cellular macromolecule catabolic process | 266 out of 9357 genes, 2.8% | 312 out of 10903 genes, 2.9% | 1 |
| nucleobase, nucleoside and nucleotide biosynthetic process | 178 out of 9357 genes, 1.9% | 209 out of 10903 genes, 1.9% | 1 |
| nucleobase, nucleoside, nucleotide and nucleic acid biosynthetic process | 178 out of 9357 genes, 1.9% | 209 out of 10903 genes, 1.9% | 1 |
| DNA-dependent DNA replication | 40 out of 9357 genes, 0.4% | 47 out of 10903 genes, 0.4% | 1 |
| cell cycle checkpoint | 18 out of 9357 genes, 0.2% | 21 out of 10903 genes, 0.2% | 1 |
| DNA catabolic process | 18 out of 9357 genes, 0.2% | 21 out of 10903 genes, 0.2% | 1 |
| cellular membrane fusion | 18 out of 9357 genes, 0.2% | 21 out of 10903 genes, 0.2% | 1 |
| cell cycle cytokinesis | 18 out of 9357 genes, 0.2% | 21 out of 10903 genes, 0.2% | 1 |
| dauer larval development | 18 out of 9357 genes, 0.2% | 21 out of 10903 genes, 0.2% | 1 |
| membrane fusion | 18 out of 9357 genes, 0.2% | 21 out of 10903 genes, 0.2% | 1 |
| regulation of cell cycle arrest | 18 out of 9357 genes, 0.2% | 21 out of 10903 genes, 0.2% | 1 |
| multi-organism process | 114 out of 9357 genes, 1.2% | 134 out of 10903 genes, 1.2% | 1 |
| aging | 531 out of 9357 genes, 5.7% | 622 out of 10903 genes, 5.7% | 1 |
| electron transport chain | 51 out of 9357 genes, 0.5% | 60 out of 10903 genes, 0.6% | 1 |
| macromolecule metabolic process | 3584 out of 9357 genes, 38.3% | 4184 out of 10903 genes, 38.4% | 1 |
| nucleoside phosphate metabolic process | 259 out of 9357 genes, 2.8% | 304 out of 10903 genes, 2.8% | 1 |
| di-, tri-valent inorganic cation transport | 62 out of 9357 genes, 0.7% | 73 out of 10903 genes, 0.7% | 1 |
| polysaccharide biosynthetic process | 23 out of 9357 genes, 0.2% | 27 out of 10903 genes, 0.2% | 1 |
| myosin filament assembly or disassembly | 23 out of 9357 genes, 0.2% | 27 out of 10903 genes, 0.2% | 1 |
| pattern specification process | 136 out of 9357 genes, 1.5% | 160 out of 10903 genes, 1.5% | 1 |
| polysaccharide metabolic process | 56 out of 9357 genes, 0.6% | 66 out of 10903 genes, 0.6% | 1 |
| posttranscriptional regulation of gene expression | 130 out of 9357 genes, 1.4% | 153 out of 10903 genes, 1.4% | 1 |
| purine ribonucleotide biosynthetic process | 107 out of 9357 genes, 1.1% | 126 out of 10903 genes, 1.2% | 1 |
| response to mechanical stimulus | 39 out of 9357 genes, 0.4% | 46 out of 10903 genes, 0.4% | 1 |
| regulation of neurogenesis | 28 out of 9357 genes, 0.3% | 33 out of 10903 genes, 0.3% | 1 |
| cellular response to chemical stimulus | 28 out of 9357 genes, 0.3% | 33 out of 10903 genes, 0.3% | 1 |
| intracellular signal transduction | 386 out of 9357 genes, 4.1% | 453 out of 10903 genes, 4.2% | 1 |
| small molecule metabolic process | 901 out of 9357 genes, 9.6% | 1055 out of 10903 genes, 9.7% | 1 |
| purine nucleoside triphosphate biosynthetic process | 95 out of 9357 genes, 1.0% | 112 out of 10903 genes, 1.0% | 1 |
| purine ribonucleoside triphosphate biosynthetic process | 95 out of 9357 genes, 1.0% | 112 out of 10903 genes, 1.0% | 1 |
| positive regulation of protein phosphorylation | 12 out of 9357 genes, 0.1% | 14 out of 10903 genes, 0.1% | 1 |
| acetyl-CoA metabolic process | 12 out of 9357 genes, 0.1% | 14 out of 10903 genes, 0.1% | 1 |
| triglyceride metabolic process | 12 out of 9357 genes, 0.1% | 14 out of 10903 genes, 0.1% | 1 |
| positive regulation of cell proliferation | 12 out of 9357 genes, 0.1% | 14 out of 10903 genes, 0.1% | 1 |
| nucleoside metabolic process | 12 out of 9357 genes, 0.1% | 14 out of 10903 genes, 0.1% | 1 |
| purine ribonucleoside monophosphate metabolic process | 12 out of 9357 genes, 0.1% | 14 out of 10903 genes, 0.1% | 1 |
| purine ribonucleoside monophosphate biosynthetic process | 12 out of 9357 genes, 0.1% | 14 out of 10903 genes, 0.1% | 1 |
| response to UV | 12 out of 9357 genes, 0.1% | 14 out of 10903 genes, 0.1% | 1 |
| positive regulation of phosphorus metabolic process | 12 out of 9357 genes, 0.1% | 14 out of 10903 genes, 0.1% | 1 |
| establishment or maintenance of apical/basal cell polarity | 12 out of 9357 genes, 0.1% | 14 out of 10903 genes, 0.1% | 1 |
| positive regulation of phosphorylation | 12 out of 9357 genes, 0.1% | 14 out of 10903 genes, 0.1% | 1 |
| response to starvation | 12 out of 9357 genes, 0.1% | 14 out of 10903 genes, 0.1% | 1 |
| ear development | 12 out of 9357 genes, 0.1% | 14 out of 10903 genes, 0.1% | 1 |
| positive regulation of phosphate metabolic process | 12 out of 9357 genes, 0.1% | 14 out of 10903 genes, 0.1% | 1 |
| lipoic acid metabolic process | 7 out of 9357 genes, 0.1% | 8 out of 10903 genes, 0.1% | 1 |
| activation of immune response | 7 out of 9357 genes, 0.1% | 8 out of 10903 genes, 0.1% | 1 |
| immune response-activating cell surface receptor signaling pathway | 7 out of 9357 genes, 0.1% | 8 out of 10903 genes, 0.1% | 1 |
| production of molecular mediator of immune response | 7 out of 9357 genes, 0.1% | 8 out of 10903 genes, 0.1% | 1 |
| somatic diversification of immune receptors via germline recombination within a single locus | 7 out of 9357 genes, 0.1% | 8 out of 10903 genes, 0.1% | 1 |
| immune response-activating signal transduction | 7 out of 9357 genes, 0.1% | 8 out of 10903 genes, 0.1% | 1 |
| immune response-regulating signaling pathway | 7 out of 9357 genes, 0.1% | 8 out of 10903 genes, 0.1% | 1 |
| immune response-regulating cell surface receptor signaling pathway | 7 out of 9357 genes, 0.1% | 8 out of 10903 genes, 0.1% | 1 |
| pyruvate metabolic process | 7 out of 9357 genes, 0.1% | 8 out of 10903 genes, 0.1% | 1 |
| acetylcholine metabolic process | 7 out of 9357 genes, 0.1% | 8 out of 10903 genes, 0.1% | 1 |
| axon ensheathment | 7 out of 9357 genes, 0.1% | 8 out of 10903 genes, 0.1% | 1 |
| detection of light stimulus | 7 out of 9357 genes, 0.1% | 8 out of 10903 genes, 0.1% | 1 |
| detection of visible light | 7 out of 9357 genes, 0.1% | 8 out of 10903 genes, 0.1% | 1 |
| regulation of phospholipase activity | 7 out of 9357 genes, 0.1% | 8 out of 10903 genes, 0.1% | 1 |
| somatic cell DNA recombination | 7 out of 9357 genes, 0.1% | 8 out of 10903 genes, 0.1% | 1 |
| negative regulation of kinase activity | 7 out of 9357 genes, 0.1% | 8 out of 10903 genes, 0.1% | 1 |
| cellular polysaccharide biosynthetic process | 7 out of 9357 genes, 0.1% | 8 out of 10903 genes, 0.1% | 1 |
| regulation of ion transmembrane transport | 7 out of 9357 genes, 0.1% | 8 out of 10903 genes, 0.1% | 1 |
| eye photoreceptor cell development | 7 out of 9357 genes, 0.1% | 8 out of 10903 genes, 0.1% | 1 |
| regulation of protein binding | 7 out of 9357 genes, 0.1% | 8 out of 10903 genes, 0.1% | 1 |
| pigment biosynthetic process | 7 out of 9357 genes, 0.1% | 8 out of 10903 genes, 0.1% | 1 |
| development of primary male sexual characteristics | 7 out of 9357 genes, 0.1% | 8 out of 10903 genes, 0.1% | 1 |
| male sex differentiation | 7 out of 9357 genes, 0.1% | 8 out of 10903 genes, 0.1% | 1 |
| positive regulation of immune response | 7 out of 9357 genes, 0.1% | 8 out of 10903 genes, 0.1% | 1 |
| antigen receptor-mediated signaling pathway | 7 out of 9357 genes, 0.1% | 8 out of 10903 genes, 0.1% | 1 |
| regulation of transcription factor activity | 7 out of 9357 genes, 0.1% | 8 out of 10903 genes, 0.1% | 1 |
| negative regulation of transferase activity | 7 out of 9357 genes, 0.1% | 8 out of 10903 genes, 0.1% | 1 |
| positive regulation of cytoskeleton organization | 7 out of 9357 genes, 0.1% | 8 out of 10903 genes, 0.1% | 1 |
| sterol homeostasis | 7 out of 9357 genes, 0.1% | 8 out of 10903 genes, 0.1% | 1 |
| regulation of lipase activity | 7 out of 9357 genes, 0.1% | 8 out of 10903 genes, 0.1% | 1 |
| regulation of transcription regulator activity | 7 out of 9357 genes, 0.1% | 8 out of 10903 genes, 0.1% | 1 |
| regulation of reproductive process | 7 out of 9357 genes, 0.1% | 8 out of 10903 genes, 0.1% | 1 |
| ribonucleotide metabolic process | 152 out of 9357 genes, 1.6% | 179 out of 10903 genes, 1.6% | 1 |
| response to endogenous stimulus | 72 out of 9357 genes, 0.8% | 85 out of 10903 genes, 0.8% | 1 |
| cellular macromolecule localization | 106 out of 9357 genes, 1.1% | 125 out of 10903 genes, 1.1% | 1 |
| alcohol metabolic process | 227 out of 9357 genes, 2.4% | 267 out of 10903 genes, 2.4% | 1 |
| purine nucleotide catabolic process | 44 out of 9357 genes, 0.5% | 52 out of 10903 genes, 0.5% | 1 |
| locomotory behavior | 44 out of 9357 genes, 0.5% | 52 out of 10903 genes, 0.5% | 1 |
| response to molecule of bacterial origin | 17 out of 9357 genes, 0.2% | 20 out of 10903 genes, 0.2% | 1 |
| negative regulation of signal transduction | 17 out of 9357 genes, 0.2% | 20 out of 10903 genes, 0.2% | 1 |
| negative regulation of signaling process | 17 out of 9357 genes, 0.2% | 20 out of 10903 genes, 0.2% | 1 |
| ARF protein signal transduction | 17 out of 9357 genes, 0.2% | 20 out of 10903 genes, 0.2% | 1 |
| nucleosome organization | 17 out of 9357 genes, 0.2% | 20 out of 10903 genes, 0.2% | 1 |
| monovalent inorganic cation homeostasis | 17 out of 9357 genes, 0.2% | 20 out of 10903 genes, 0.2% | 1 |
| regulation of protein serine/threonine kinase activity | 17 out of 9357 genes, 0.2% | 20 out of 10903 genes, 0.2% | 1 |
| embryonic pattern specification | 33 out of 9357 genes, 0.4% | 39 out of 10903 genes, 0.4% | 1 |
| carbohydrate metabolic process | 314 out of 9357 genes, 3.4% | 369 out of 10903 genes, 3.4% | 1 |
| energy derivation by oxidation of organic compounds | 117 out of 9357 genes, 1.3% | 138 out of 10903 genes, 1.3% | 1 |
| RNA export from nucleus | 22 out of 9357 genes, 0.2% | 26 out of 10903 genes, 0.2% | 1 |
| muscle contraction | 22 out of 9357 genes, 0.2% | 26 out of 10903 genes, 0.2% | 1 |
| response to hormone stimulus | 49 out of 9357 genes, 0.5% | 58 out of 10903 genes, 0.5% | 1 |
| lipid transport | 38 out of 9357 genes, 0.4% | 45 out of 10903 genes, 0.4% | 1 |
| spindle organization | 38 out of 9357 genes, 0.4% | 45 out of 10903 genes, 0.4% | 1 |
| vesicle organization | 38 out of 9357 genes, 0.4% | 45 out of 10903 genes, 0.4% | 1 |
| heterocycle metabolic process | 266 out of 9357 genes, 2.8% | 313 out of 10903 genes, 2.9% | 1 |
| protein complex subunit organization | 179 out of 9357 genes, 1.9% | 211 out of 10903 genes, 1.9% | 1 |
| negative regulation of biological process | 341 out of 9357 genes, 3.6% | 401 out of 10903 genes, 3.7% | 1 |
| positive regulation of macromolecule metabolic process | 65 out of 9357 genes, 0.7% | 77 out of 10903 genes, 0.7% | 1 |
| regulation of signal transduction | 213 out of 9357 genes, 2.3% | 251 out of 10903 genes, 2.3% | 1 |
| regulation of signaling process | 213 out of 9357 genes, 2.3% | 251 out of 10903 genes, 2.3% | 1 |
| cellular protein localization | 104 out of 9357 genes, 1.1% | 123 out of 10903 genes, 1.1% | 1 |
| signal transduction | 422 out of 9357 genes, 4.5% | 496 out of 10903 genes, 4.5% | 1 |
| proteolysis | 247 out of 9357 genes, 2.6% | 291 out of 10903 genes, 2.7% | 1 |
| M phase of mitotic cell cycle | 126 out of 9357 genes, 1.3% | 149 out of 10903 genes, 1.4% | 1 |
| generation of precursor metabolites and energy | 143 out of 9357 genes, 1.5% | 169 out of 10903 genes, 1.6% | 1 |
| hydrogen transport | 37 out of 9357 genes, 0.4% | 44 out of 10903 genes, 0.4% | 1 |
| sulfur amino acid metabolic process | 16 out of 9357 genes, 0.2% | 19 out of 10903 genes, 0.2% | 1 |
| nucleotide-excision repair | 16 out of 9357 genes, 0.2% | 19 out of 10903 genes, 0.2% | 1 |
| transforming growth factor beta receptor signaling pathway | 16 out of 9357 genes, 0.2% | 19 out of 10903 genes, 0.2% | 1 |
| positive regulation of cellular protein metabolic process | 16 out of 9357 genes, 0.2% | 19 out of 10903 genes, 0.2% | 1 |
| positive regulation of protein metabolic process | 16 out of 9357 genes, 0.2% | 19 out of 10903 genes, 0.2% | 1 |
| cytokine production | 11 out of 9357 genes, 0.1% | 13 out of 10903 genes, 0.1% | 1 |
| regulation of translational initiation | 11 out of 9357 genes, 0.1% | 13 out of 10903 genes, 0.1% | 1 |
| regulation of response to external stimulus | 11 out of 9357 genes, 0.1% | 13 out of 10903 genes, 0.1% | 1 |
| positive regulation of intracellular transport | 11 out of 9357 genes, 0.1% | 13 out of 10903 genes, 0.1% | 1 |
| inner ear development | 11 out of 9357 genes, 0.1% | 13 out of 10903 genes, 0.1% | 1 |
| mRNA transport | 11 out of 9357 genes, 0.1% | 13 out of 10903 genes, 0.1% | 1 |
| regulation of response to stress | 11 out of 9357 genes, 0.1% | 13 out of 10903 genes, 0.1% | 1 |
| regulation of gene expression, epigenetic | 103 out of 9357 genes, 1.1% | 122 out of 10903 genes, 1.1% | 1 |
| phagocytosis, engulfment | 21 out of 9357 genes, 0.2% | 25 out of 10903 genes, 0.2% | 1 |
| negative regulation of nucleobase, nucleoside, nucleotide and nucleic acid metabolic process | 21 out of 9357 genes, 0.2% | 25 out of 10903 genes, 0.2% | 1 |
| negative regulation of nitrogen compound metabolic process | 21 out of 9357 genes, 0.2% | 25 out of 10903 genes, 0.2% | 1 |
| purine ribonucleotide metabolic process | 148 out of 9357 genes, 1.6% | 175 out of 10903 genes, 1.6% | 1 |
| disaccharide metabolic process | 26 out of 9357 genes, 0.3% | 31 out of 10903 genes, 0.3% | 1 |
| aminoglycan metabolic process | 26 out of 9357 genes, 0.3% | 31 out of 10903 genes, 0.3% | 1 |
| rRNA processing | 26 out of 9357 genes, 0.3% | 31 out of 10903 genes, 0.3% | 1 |
| negative regulation of biosynthetic process | 26 out of 9357 genes, 0.3% | 31 out of 10903 genes, 0.3% | 1 |
| monovalent inorganic cation transport | 26 out of 9357 genes, 0.3% | 31 out of 10903 genes, 0.3% | 1 |
| RNA metabolic process | 878 out of 9357 genes, 9.4% | 1030 out of 10903 genes, 9.4% | 1 |
| cell growth | 58 out of 9357 genes, 0.6% | 69 out of 10903 genes, 0.6% | 1 |
| establishment of nucleus localization | 58 out of 9357 genes, 0.6% | 69 out of 10903 genes, 0.6% | 1 |
| regulation of cellular catabolic process | 80 out of 9357 genes, 0.9% | 95 out of 10903 genes, 0.9% | 1 |
| sex determination | 31 out of 9357 genes, 0.3% | 37 out of 10903 genes, 0.3% | 1 |
| germ-line sex determination | 31 out of 9357 genes, 0.3% | 37 out of 10903 genes, 0.3% | 1 |
| S phase of mitotic cell cycle | 2 out of 9357 genes, 0.0% | 2 out of 10903 genes, 0.0% | 1 |
| activation of MAPKK activity | 2 out of 9357 genes, 0.0% | 2 out of 10903 genes, 0.0% | 1 |
| contractile ring contraction involved in cell cycle cytokinesis | 2 out of 9357 genes, 0.0% | 2 out of 10903 genes, 0.0% | 1 |
| endoderm formation | 2 out of 9357 genes, 0.0% | 2 out of 10903 genes, 0.0% | 1 |
| negative regulation of cytokine production | 2 out of 9357 genes, 0.0% | 2 out of 10903 genes, 0.0% | 1 |
| receptor recycling | 2 out of 9357 genes, 0.0% | 2 out of 10903 genes, 0.0% | 1 |
| negative regulation of protein phosphorylation | 2 out of 9357 genes, 0.0% | 2 out of 10903 genes, 0.0% | 1 |
| columnar/cuboidal epithelial cell development | 2 out of 9357 genes, 0.0% | 2 out of 10903 genes, 0.0% | 1 |
| glandular epithelial cell development | 2 out of 9357 genes, 0.0% | 2 out of 10903 genes, 0.0% | 1 |
| lens development in camera-type eye | 2 out of 9357 genes, 0.0% | 2 out of 10903 genes, 0.0% | 1 |
| regulation of receptor internalization | 2 out of 9357 genes, 0.0% | 2 out of 10903 genes, 0.0% | 1 |
| cell activation involved in immune response | 2 out of 9357 genes, 0.0% | 2 out of 10903 genes, 0.0% | 1 |
| lymphocyte activation involved in immune response | 2 out of 9357 genes, 0.0% | 2 out of 10903 genes, 0.0% | 1 |
| leukocyte activation involved in immune response | 2 out of 9357 genes, 0.0% | 2 out of 10903 genes, 0.0% | 1 |
| negative regulation of immune system process | 2 out of 9357 genes, 0.0% | 2 out of 10903 genes, 0.0% | 1 |
| negative regulation of immune effector process | 2 out of 9357 genes, 0.0% | 2 out of 10903 genes, 0.0% | 1 |
| positive regulation of immune effector process | 2 out of 9357 genes, 0.0% | 2 out of 10903 genes, 0.0% | 1 |
| regulation of antimicrobial humoral response | 2 out of 9357 genes, 0.0% | 2 out of 10903 genes, 0.0% | 1 |
| regulation of myeloid leukocyte differentiation | 2 out of 9357 genes, 0.0% | 2 out of 10903 genes, 0.0% | 1 |
| regulation of humoral immune response | 2 out of 9357 genes, 0.0% | 2 out of 10903 genes, 0.0% | 1 |
| cardiac chamber development | 2 out of 9357 genes, 0.0% | 2 out of 10903 genes, 0.0% | 1 |
| cardiac chamber morphogenesis | 2 out of 9357 genes, 0.0% | 2 out of 10903 genes, 0.0% | 1 |
| ethanol metabolic process | 2 out of 9357 genes, 0.0% | 2 out of 10903 genes, 0.0% | 1 |
| succinyl-CoA metabolic process | 2 out of 9357 genes, 0.0% | 2 out of 10903 genes, 0.0% | 1 |
| regulation of gluconeogenesis | 2 out of 9357 genes, 0.0% | 2 out of 10903 genes, 0.0% | 1 |
| base-excision repair, AP site formation | 2 out of 9357 genes, 0.0% | 2 out of 10903 genes, 0.0% | 1 |
| double-strand break repair | 2 out of 9357 genes, 0.0% | 2 out of 10903 genes, 0.0% | 1 |
| transcription termination | 2 out of 9357 genes, 0.0% | 2 out of 10903 genes, 0.0% | 1 |
| mRNA cleavage | 2 out of 9357 genes, 0.0% | 2 out of 10903 genes, 0.0% | 1 |
| transcription from RNA polymerase III promoter | 2 out of 9357 genes, 0.0% | 2 out of 10903 genes, 0.0% | 1 |
| protein sulfation | 2 out of 9357 genes, 0.0% | 2 out of 10903 genes, 0.0% | 1 |
| polyamine catabolic process | 2 out of 9357 genes, 0.0% | 2 out of 10903 genes, 0.0% | 1 |
| protein targeting to mitochondrion | 2 out of 9357 genes, 0.0% | 2 out of 10903 genes, 0.0% | 1 |
| acyl-CoA metabolic process | 2 out of 9357 genes, 0.0% | 2 out of 10903 genes, 0.0% | 1 |
| riboflavin metabolic process | 2 out of 9357 genes, 0.0% | 2 out of 10903 genes, 0.0% | 1 |
| chloride transport | 2 out of 9357 genes, 0.0% | 2 out of 10903 genes, 0.0% | 1 |
| zinc ion transport | 2 out of 9357 genes, 0.0% | 2 out of 10903 genes, 0.0% | 1 |
| mitochondrial transport | 2 out of 9357 genes, 0.0% | 2 out of 10903 genes, 0.0% | 1 |
| cell volume homeostasis | 2 out of 9357 genes, 0.0% | 2 out of 10903 genes, 0.0% | 1 |
| post-Golgi vesicle-mediated transport | 2 out of 9357 genes, 0.0% | 2 out of 10903 genes, 0.0% | 1 |
| anti-apoptosis | 2 out of 9357 genes, 0.0% | 2 out of 10903 genes, 0.0% | 1 |
| telomere maintenance via telomerase | 2 out of 9357 genes, 0.0% | 2 out of 10903 genes, 0.0% | 1 |
| mitochondrial membrane organization | 2 out of 9357 genes, 0.0% | 2 out of 10903 genes, 0.0% | 1 |
| plasma membrane organization | 2 out of 9357 genes, 0.0% | 2 out of 10903 genes, 0.0% | 1 |
| reciprocal meiotic recombination | 2 out of 9357 genes, 0.0% | 2 out of 10903 genes, 0.0% | 1 |
| meiosis II | 2 out of 9357 genes, 0.0% | 2 out of 10903 genes, 0.0% | 1 |
| activation of adenylate cyclase activity by G-protein signaling pathway | 2 out of 9357 genes, 0.0% | 2 out of 10903 genes, 0.0% | 1 |
| activation of adenylate cyclase activity | 2 out of 9357 genes, 0.0% | 2 out of 10903 genes, 0.0% | 1 |
| spermatogenesis | 2 out of 9357 genes, 0.0% | 2 out of 10903 genes, 0.0% | 1 |
| oocyte anterior/posterior axis specification | 2 out of 9357 genes, 0.0% | 2 out of 10903 genes, 0.0% | 1 |
| pole plasm assembly | 2 out of 9357 genes, 0.0% | 2 out of 10903 genes, 0.0% | 1 |
| pole plasm RNA localization | 2 out of 9357 genes, 0.0% | 2 out of 10903 genes, 0.0% | 1 |
| single fertilization | 2 out of 9357 genes, 0.0% | 2 out of 10903 genes, 0.0% | 1 |
| segment specification | 2 out of 9357 genes, 0.0% | 2 out of 10903 genes, 0.0% | 1 |
| synapse assembly | 2 out of 9357 genes, 0.0% | 2 out of 10903 genes, 0.0% | 1 |
| anterior/posterior pattern formation, imaginal disc | 2 out of 9357 genes, 0.0% | 2 out of 10903 genes, 0.0% | 1 |
| imaginal disc-derived genitalia development | 2 out of 9357 genes, 0.0% | 2 out of 10903 genes, 0.0% | 1 |
| endoderm development | 2 out of 9357 genes, 0.0% | 2 out of 10903 genes, 0.0% | 1 |
| histolysis | 2 out of 9357 genes, 0.0% | 2 out of 10903 genes, 0.0% | 1 |
| negative regulation of female receptivity | 2 out of 9357 genes, 0.0% | 2 out of 10903 genes, 0.0% | 1 |
| ergosterol metabolic process | 2 out of 9357 genes, 0.0% | 2 out of 10903 genes, 0.0% | 1 |
| spermidine metabolic process | 2 out of 9357 genes, 0.0% | 2 out of 10903 genes, 0.0% | 1 |
| intracellular mRNA localization | 2 out of 9357 genes, 0.0% | 2 out of 10903 genes, 0.0% | 1 |
| histone mRNA metabolic process | 2 out of 9357 genes, 0.0% | 2 out of 10903 genes, 0.0% | 1 |
| maternal determination of anterior/posterior axis, embryo | 2 out of 9357 genes, 0.0% | 2 out of 10903 genes, 0.0% | 1 |
| respiratory chain complex IV assembly | 2 out of 9357 genes, 0.0% | 2 out of 10903 genes, 0.0% | 1 |
| serine family amino acid catabolic process | 2 out of 9357 genes, 0.0% | 2 out of 10903 genes, 0.0% | 1 |
| purine base biosynthetic process | 2 out of 9357 genes, 0.0% | 2 out of 10903 genes, 0.0% | 1 |
| pyrimidine nucleoside diphosphate biosynthetic process | 2 out of 9357 genes, 0.0% | 2 out of 10903 genes, 0.0% | 1 |
| pyrimidine ribonucleoside monophosphate metabolic process | 2 out of 9357 genes, 0.0% | 2 out of 10903 genes, 0.0% | 1 |
| pyrimidine ribonucleoside monophosphate biosynthetic process | 2 out of 9357 genes, 0.0% | 2 out of 10903 genes, 0.0% | 1 |
| deoxyribonucleoside diphosphate metabolic process | 2 out of 9357 genes, 0.0% | 2 out of 10903 genes, 0.0% | 1 |
| cyclic nucleotide metabolic process | 2 out of 9357 genes, 0.0% | 2 out of 10903 genes, 0.0% | 1 |
| deoxyribonucleoside diphosphate biosynthetic process | 2 out of 9357 genes, 0.0% | 2 out of 10903 genes, 0.0% | 1 |
| cyclic nucleotide biosynthetic process | 2 out of 9357 genes, 0.0% | 2 out of 10903 genes, 0.0% | 1 |
| pyrimidine deoxyribonucleoside diphosphate metabolic process | 2 out of 9357 genes, 0.0% | 2 out of 10903 genes, 0.0% | 1 |
| pyrimidine deoxyribonucleoside diphosphate biosynthetic process | 2 out of 9357 genes, 0.0% | 2 out of 10903 genes, 0.0% | 1 |
| deoxyribonucleoside triphosphate biosynthetic process | 2 out of 9357 genes, 0.0% | 2 out of 10903 genes, 0.0% | 1 |
| pyrimidine ribonucleoside triphosphate metabolic process | 2 out of 9357 genes, 0.0% | 2 out of 10903 genes, 0.0% | 1 |
| pyrimidine ribonucleoside triphosphate biosynthetic process | 2 out of 9357 genes, 0.0% | 2 out of 10903 genes, 0.0% | 1 |
| pyrimidine deoxyribonucleoside triphosphate biosynthetic process | 2 out of 9357 genes, 0.0% | 2 out of 10903 genes, 0.0% | 1 |
| detection of carbohydrate stimulus | 2 out of 9357 genes, 0.0% | 2 out of 10903 genes, 0.0% | 1 |
| detection of hexose stimulus | 2 out of 9357 genes, 0.0% | 2 out of 10903 genes, 0.0% | 1 |
| cell-cell recognition | 2 out of 9357 genes, 0.0% | 2 out of 10903 genes, 0.0% | 1 |
| negative regulation of cell fate specification | 2 out of 9357 genes, 0.0% | 2 out of 10903 genes, 0.0% | 1 |
| regulation of cell fate commitment | 2 out of 9357 genes, 0.0% | 2 out of 10903 genes, 0.0% | 1 |
| negative regulation of cell fate commitment | 2 out of 9357 genes, 0.0% | 2 out of 10903 genes, 0.0% | 1 |
| negative regulation of phosphorus metabolic process | 2 out of 9357 genes, 0.0% | 2 out of 10903 genes, 0.0% | 1 |
| regulation of adenylate cyclase activity involved in G-protein signaling pathway | 2 out of 9357 genes, 0.0% | 2 out of 10903 genes, 0.0% | 1 |
| positive regulation of adenylate cyclase activity by G-protein signaling pathway | 2 out of 9357 genes, 0.0% | 2 out of 10903 genes, 0.0% | 1 |
| regulation of lamellipodium assembly | 2 out of 9357 genes, 0.0% | 2 out of 10903 genes, 0.0% | 1 |
| epithelial structure maintenance | 2 out of 9357 genes, 0.0% | 2 out of 10903 genes, 0.0% | 1 |
| regulation of myotube differentiation | 2 out of 9357 genes, 0.0% | 2 out of 10903 genes, 0.0% | 1 |
| telomere maintenance via telomere lengthening | 2 out of 9357 genes, 0.0% | 2 out of 10903 genes, 0.0% | 1 |
| oligodendrocyte development | 2 out of 9357 genes, 0.0% | 2 out of 10903 genes, 0.0% | 1 |
| response to tropane | 2 out of 9357 genes, 0.0% | 2 out of 10903 genes, 0.0% | 1 |
| response to purine | 2 out of 9357 genes, 0.0% | 2 out of 10903 genes, 0.0% | 1 |
| smooth muscle cell migration | 2 out of 9357 genes, 0.0% | 2 out of 10903 genes, 0.0% | 1 |
| L-amino acid transport | 2 out of 9357 genes, 0.0% | 2 out of 10903 genes, 0.0% | 1 |
| energy coupled proton transport, against electrochemical gradient | 2 out of 9357 genes, 0.0% | 2 out of 10903 genes, 0.0% | 1 |
| deactivation of rhodopsin mediated signaling | 2 out of 9357 genes, 0.0% | 2 out of 10903 genes, 0.0% | 1 |
| prenol metabolic process | 2 out of 9357 genes, 0.0% | 2 out of 10903 genes, 0.0% | 1 |
| polyprenol metabolic process | 2 out of 9357 genes, 0.0% | 2 out of 10903 genes, 0.0% | 1 |
| diterpenoid metabolic process | 2 out of 9357 genes, 0.0% | 2 out of 10903 genes, 0.0% | 1 |
| phytosteroid metabolic process | 2 out of 9357 genes, 0.0% | 2 out of 10903 genes, 0.0% | 1 |
| tissue death | 2 out of 9357 genes, 0.0% | 2 out of 10903 genes, 0.0% | 1 |
| meiotic chromosome movement towards spindle pole | 2 out of 9357 genes, 0.0% | 2 out of 10903 genes, 0.0% | 1 |
| cytochrome complex assembly | 2 out of 9357 genes, 0.0% | 2 out of 10903 genes, 0.0% | 1 |
| protein nitrosylation | 2 out of 9357 genes, 0.0% | 2 out of 10903 genes, 0.0% | 1 |
| respiratory chain complex III assembly | 2 out of 9357 genes, 0.0% | 2 out of 10903 genes, 0.0% | 1 |
| calcium ion-dependent exocytosis | 2 out of 9357 genes, 0.0% | 2 out of 10903 genes, 0.0% | 1 |
| peptidyl-threonine phosphorylation | 2 out of 9357 genes, 0.0% | 2 out of 10903 genes, 0.0% | 1 |
| peptidyl-threonine modification | 2 out of 9357 genes, 0.0% | 2 out of 10903 genes, 0.0% | 1 |
| pole plasm mRNA localization | 2 out of 9357 genes, 0.0% | 2 out of 10903 genes, 0.0% | 1 |
| pyrimidine base biosynthetic process | 2 out of 9357 genes, 0.0% | 2 out of 10903 genes, 0.0% | 1 |
| antigen processing and presentation of exogenous antigen | 2 out of 9357 genes, 0.0% | 2 out of 10903 genes, 0.0% | 1 |
| pons development | 2 out of 9357 genes, 0.0% | 2 out of 10903 genes, 0.0% | 1 |
| medulla oblongata development | 2 out of 9357 genes, 0.0% | 2 out of 10903 genes, 0.0% | 1 |
| cranial nerve structural organization | 2 out of 9357 genes, 0.0% | 2 out of 10903 genes, 0.0% | 1 |
| forebrain neuron differentiation | 2 out of 9357 genes, 0.0% | 2 out of 10903 genes, 0.0% | 1 |
| pituitary gland development | 2 out of 9357 genes, 0.0% | 2 out of 10903 genes, 0.0% | 1 |
| central nervous system myelination | 2 out of 9357 genes, 0.0% | 2 out of 10903 genes, 0.0% | 1 |
| regulation of rhodopsin mediated signaling pathway | 2 out of 9357 genes, 0.0% | 2 out of 10903 genes, 0.0% | 1 |
| hair cycle process | 2 out of 9357 genes, 0.0% | 2 out of 10903 genes, 0.0% | 1 |
| ovulation cycle process | 2 out of 9357 genes, 0.0% | 2 out of 10903 genes, 0.0% | 1 |
| extracellular matrix disassembly | 2 out of 9357 genes, 0.0% | 2 out of 10903 genes, 0.0% | 1 |
| maintenance of cell polarity | 2 out of 9357 genes, 0.0% | 2 out of 10903 genes, 0.0% | 1 |
| insulin secretion | 2 out of 9357 genes, 0.0% | 2 out of 10903 genes, 0.0% | 1 |
| natural killer cell activation | 2 out of 9357 genes, 0.0% | 2 out of 10903 genes, 0.0% | 1 |
| estrogen receptor signaling pathway | 2 out of 9357 genes, 0.0% | 2 out of 10903 genes, 0.0% | 1 |
| regulation of epithelial cell differentiation | 2 out of 9357 genes, 0.0% | 2 out of 10903 genes, 0.0% | 1 |
| polarized epithelial cell differentiation | 2 out of 9357 genes, 0.0% | 2 out of 10903 genes, 0.0% | 1 |
| regulation of B cell proliferation | 2 out of 9357 genes, 0.0% | 2 out of 10903 genes, 0.0% | 1 |
| regulation of vesicle fusion | 2 out of 9357 genes, 0.0% | 2 out of 10903 genes, 0.0% | 1 |
| negative regulation of protein modification process | 2 out of 9357 genes, 0.0% | 2 out of 10903 genes, 0.0% | 1 |
| negative regulation of translation in response to stress | 2 out of 9357 genes, 0.0% | 2 out of 10903 genes, 0.0% | 1 |
| positive regulation of response to external stimulus | 2 out of 9357 genes, 0.0% | 2 out of 10903 genes, 0.0% | 1 |
| activation of protein kinase activity | 2 out of 9357 genes, 0.0% | 2 out of 10903 genes, 0.0% | 1 |
| regulation of actin filament bundle assembly | 2 out of 9357 genes, 0.0% | 2 out of 10903 genes, 0.0% | 1 |
| axon ensheathment in central nervous system | 2 out of 9357 genes, 0.0% | 2 out of 10903 genes, 0.0% | 1 |
| response to peptidoglycan | 2 out of 9357 genes, 0.0% | 2 out of 10903 genes, 0.0% | 1 |
| positive regulation of Rac GTPase activity | 2 out of 9357 genes, 0.0% | 2 out of 10903 genes, 0.0% | 1 |
| neurotrophin production | 2 out of 9357 genes, 0.0% | 2 out of 10903 genes, 0.0% | 1 |
| transforming growth factor-beta1 production | 2 out of 9357 genes, 0.0% | 2 out of 10903 genes, 0.0% | 1 |
| activin receptor signaling pathway | 2 out of 9357 genes, 0.0% | 2 out of 10903 genes, 0.0% | 1 |
| regulation of mononuclear cell proliferation | 2 out of 9357 genes, 0.0% | 2 out of 10903 genes, 0.0% | 1 |
| ribonucleoprotein complex disassembly | 2 out of 9357 genes, 0.0% | 2 out of 10903 genes, 0.0% | 1 |
| T cell differentiation in thymus | 2 out of 9357 genes, 0.0% | 2 out of 10903 genes, 0.0% | 1 |
| cell cycle comprising mitosis without cytokinesis | 2 out of 9357 genes, 0.0% | 2 out of 10903 genes, 0.0% | 1 |
| meiotic cell cycle checkpoint | 2 out of 9357 genes, 0.0% | 2 out of 10903 genes, 0.0% | 1 |
| cholesterol efflux | 2 out of 9357 genes, 0.0% | 2 out of 10903 genes, 0.0% | 1 |
| gas homeostasis | 2 out of 9357 genes, 0.0% | 2 out of 10903 genes, 0.0% | 1 |
| response to disaccharide stimulus | 2 out of 9357 genes, 0.0% | 2 out of 10903 genes, 0.0% | 1 |
| detection of monosaccharide stimulus | 2 out of 9357 genes, 0.0% | 2 out of 10903 genes, 0.0% | 1 |
| monohydric alcohol metabolic process | 2 out of 9357 genes, 0.0% | 2 out of 10903 genes, 0.0% | 1 |
| Arp2/3 complex-mediated actin nucleation | 2 out of 9357 genes, 0.0% | 2 out of 10903 genes, 0.0% | 1 |
| response to fluid shear stress | 2 out of 9357 genes, 0.0% | 2 out of 10903 genes, 0.0% | 1 |
| sperm-egg recognition | 2 out of 9357 genes, 0.0% | 2 out of 10903 genes, 0.0% | 1 |
| salivary gland histolysis | 2 out of 9357 genes, 0.0% | 2 out of 10903 genes, 0.0% | 1 |
| salivary gland cell autophagic cell death | 2 out of 9357 genes, 0.0% | 2 out of 10903 genes, 0.0% | 1 |
| epithelial cell type specification, open tracheal system | 2 out of 9357 genes, 0.0% | 2 out of 10903 genes, 0.0% | 1 |
| genital disc development | 2 out of 9357 genes, 0.0% | 2 out of 10903 genes, 0.0% | 1 |
| organ growth | 2 out of 9357 genes, 0.0% | 2 out of 10903 genes, 0.0% | 1 |
| fatty-acyl-CoA metabolic process | 2 out of 9357 genes, 0.0% | 2 out of 10903 genes, 0.0% | 1 |
| thioester metabolic process | 2 out of 9357 genes, 0.0% | 2 out of 10903 genes, 0.0% | 1 |
| histone-serine phosphorylation | 2 out of 9357 genes, 0.0% | 2 out of 10903 genes, 0.0% | 1 |
| histone-threonine phosphorylation | 2 out of 9357 genes, 0.0% | 2 out of 10903 genes, 0.0% | 1 |
| germ-line stem cell division | 2 out of 9357 genes, 0.0% | 2 out of 10903 genes, 0.0% | 1 |
| T cell proliferation | 2 out of 9357 genes, 0.0% | 2 out of 10903 genes, 0.0% | 1 |
| B cell activation | 2 out of 9357 genes, 0.0% | 2 out of 10903 genes, 0.0% | 1 |
| negative regulation of phosphorylation | 2 out of 9357 genes, 0.0% | 2 out of 10903 genes, 0.0% | 1 |
| eye pigment metabolic process | 2 out of 9357 genes, 0.0% | 2 out of 10903 genes, 0.0% | 1 |
| hormone biosynthetic process | 2 out of 9357 genes, 0.0% | 2 out of 10903 genes, 0.0% | 1 |
| inner ear morphogenesis | 2 out of 9357 genes, 0.0% | 2 out of 10903 genes, 0.0% | 1 |
| tyrosine phosphorylation of Stat3 protein | 2 out of 9357 genes, 0.0% | 2 out of 10903 genes, 0.0% | 1 |
| glucose homeostasis | 2 out of 9357 genes, 0.0% | 2 out of 10903 genes, 0.0% | 1 |
| hair cycle | 2 out of 9357 genes, 0.0% | 2 out of 10903 genes, 0.0% | 1 |
| regulation of cell fate specification | 2 out of 9357 genes, 0.0% | 2 out of 10903 genes, 0.0% | 1 |
| ovulation cycle | 2 out of 9357 genes, 0.0% | 2 out of 10903 genes, 0.0% | 1 |
| riboflavin and derivative metabolic process | 2 out of 9357 genes, 0.0% | 2 out of 10903 genes, 0.0% | 1 |
| amyloid precursor protein metabolic process | 2 out of 9357 genes, 0.0% | 2 out of 10903 genes, 0.0% | 1 |
| amyloid precursor protein catabolic process | 2 out of 9357 genes, 0.0% | 2 out of 10903 genes, 0.0% | 1 |
| purine base salvage | 2 out of 9357 genes, 0.0% | 2 out of 10903 genes, 0.0% | 1 |
| peptide catabolic process | 2 out of 9357 genes, 0.0% | 2 out of 10903 genes, 0.0% | 1 |
| positive regulation of gene-specific transcription | 2 out of 9357 genes, 0.0% | 2 out of 10903 genes, 0.0% | 1 |
| pigment metabolic process involved in developmental pigmentation | 2 out of 9357 genes, 0.0% | 2 out of 10903 genes, 0.0% | 1 |
| negative regulation of transcription factor activity | 2 out of 9357 genes, 0.0% | 2 out of 10903 genes, 0.0% | 1 |
| pigment metabolic process involved in pigmentation | 2 out of 9357 genes, 0.0% | 2 out of 10903 genes, 0.0% | 1 |
| regulation of RNA stability | 2 out of 9357 genes, 0.0% | 2 out of 10903 genes, 0.0% | 1 |
| regulation of mRNA stability | 2 out of 9357 genes, 0.0% | 2 out of 10903 genes, 0.0% | 1 |
| RNA stabilization | 2 out of 9357 genes, 0.0% | 2 out of 10903 genes, 0.0% | 1 |
| negative regulation of neuron apoptosis | 2 out of 9357 genes, 0.0% | 2 out of 10903 genes, 0.0% | 1 |
| regulation of lipid kinase activity | 2 out of 9357 genes, 0.0% | 2 out of 10903 genes, 0.0% | 1 |
| regulation of phosphoinositide 3-kinase activity | 2 out of 9357 genes, 0.0% | 2 out of 10903 genes, 0.0% | 1 |
| regulation of translation in response to stress | 2 out of 9357 genes, 0.0% | 2 out of 10903 genes, 0.0% | 1 |
| cellular amide metabolic process | 2 out of 9357 genes, 0.0% | 2 out of 10903 genes, 0.0% | 1 |
| photoreceptor cell fate specification | 2 out of 9357 genes, 0.0% | 2 out of 10903 genes, 0.0% | 1 |
| positive regulation of cellular component biogenesis | 2 out of 9357 genes, 0.0% | 2 out of 10903 genes, 0.0% | 1 |
| cellular alcohol metabolic process | 2 out of 9357 genes, 0.0% | 2 out of 10903 genes, 0.0% | 1 |
| multicellular organismal metabolic process | 2 out of 9357 genes, 0.0% | 2 out of 10903 genes, 0.0% | 1 |
| multicellular organismal macromolecule metabolic process | 2 out of 9357 genes, 0.0% | 2 out of 10903 genes, 0.0% | 1 |
| negative regulation of G-protein coupled receptor protein signaling pathway | 2 out of 9357 genes, 0.0% | 2 out of 10903 genes, 0.0% | 1 |
| positive regulation of transcription, DNA-dependent | 2 out of 9357 genes, 0.0% | 2 out of 10903 genes, 0.0% | 1 |
| regulation of female receptivity | 2 out of 9357 genes, 0.0% | 2 out of 10903 genes, 0.0% | 1 |
| negative regulation of phosphate metabolic process | 2 out of 9357 genes, 0.0% | 2 out of 10903 genes, 0.0% | 1 |
| positive regulation of transcription | 2 out of 9357 genes, 0.0% | 2 out of 10903 genes, 0.0% | 1 |
| pyrimidine ribonucleoside metabolic process | 2 out of 9357 genes, 0.0% | 2 out of 10903 genes, 0.0% | 1 |
| spermidine catabolic process | 2 out of 9357 genes, 0.0% | 2 out of 10903 genes, 0.0% | 1 |
| diacylglycerol metabolic process | 2 out of 9357 genes, 0.0% | 2 out of 10903 genes, 0.0% | 1 |
| regulation of alpha-beta T cell activation | 2 out of 9357 genes, 0.0% | 2 out of 10903 genes, 0.0% | 1 |
| positive regulation of alpha-beta T cell activation | 2 out of 9357 genes, 0.0% | 2 out of 10903 genes, 0.0% | 1 |
| regulation of alpha-beta T cell differentiation | 2 out of 9357 genes, 0.0% | 2 out of 10903 genes, 0.0% | 1 |
| positive regulation of alpha-beta T cell differentiation | 2 out of 9357 genes, 0.0% | 2 out of 10903 genes, 0.0% | 1 |
| lymphocyte proliferation | 2 out of 9357 genes, 0.0% | 2 out of 10903 genes, 0.0% | 1 |
| eye pigmentation | 2 out of 9357 genes, 0.0% | 2 out of 10903 genes, 0.0% | 1 |
| regulation of developmental pigmentation | 2 out of 9357 genes, 0.0% | 2 out of 10903 genes, 0.0% | 1 |
| germ-line cyst formation | 2 out of 9357 genes, 0.0% | 2 out of 10903 genes, 0.0% | 1 |
| mRNA stabilization | 2 out of 9357 genes, 0.0% | 2 out of 10903 genes, 0.0% | 1 |
| regulation of receptor-mediated endocytosis | 2 out of 9357 genes, 0.0% | 2 out of 10903 genes, 0.0% | 1 |
| regulation of post-embryonic development | 2 out of 9357 genes, 0.0% | 2 out of 10903 genes, 0.0% | 1 |
| neuron fate specification | 2 out of 9357 genes, 0.0% | 2 out of 10903 genes, 0.0% | 1 |
| pigment granule organization | 2 out of 9357 genes, 0.0% | 2 out of 10903 genes, 0.0% | 1 |
| tissue remodeling | 2 out of 9357 genes, 0.0% | 2 out of 10903 genes, 0.0% | 1 |
| formation of anatomical boundary | 2 out of 9357 genes, 0.0% | 2 out of 10903 genes, 0.0% | 1 |
| lateral line nerve development | 2 out of 9357 genes, 0.0% | 2 out of 10903 genes, 0.0% | 1 |
| chromosome localization | 2 out of 9357 genes, 0.0% | 2 out of 10903 genes, 0.0% | 1 |
| regulation of lymphocyte proliferation | 2 out of 9357 genes, 0.0% | 2 out of 10903 genes, 0.0% | 1 |
| regulation of epithelial cell proliferation | 2 out of 9357 genes, 0.0% | 2 out of 10903 genes, 0.0% | 1 |
| regulation of B cell activation | 2 out of 9357 genes, 0.0% | 2 out of 10903 genes, 0.0% | 1 |
| positive regulation of DNA metabolic process | 2 out of 9357 genes, 0.0% | 2 out of 10903 genes, 0.0% | 1 |
| chaperone-mediated protein complex assembly | 2 out of 9357 genes, 0.0% | 2 out of 10903 genes, 0.0% | 1 |
| spindle midzone assembly | 2 out of 9357 genes, 0.0% | 2 out of 10903 genes, 0.0% | 1 |
| regulation of sequestering of calcium ion | 2 out of 9357 genes, 0.0% | 2 out of 10903 genes, 0.0% | 1 |
| negative regulation of sequestering of calcium ion | 2 out of 9357 genes, 0.0% | 2 out of 10903 genes, 0.0% | 1 |
| establishment of chromosome localization | 2 out of 9357 genes, 0.0% | 2 out of 10903 genes, 0.0% | 1 |
| chromosome separation | 2 out of 9357 genes, 0.0% | 2 out of 10903 genes, 0.0% | 1 |
| chromosome movement towards spindle pole | 2 out of 9357 genes, 0.0% | 2 out of 10903 genes, 0.0% | 1 |
| attachment of spindle microtubules to chromosome | 2 out of 9357 genes, 0.0% | 2 out of 10903 genes, 0.0% | 1 |
| S phase | 2 out of 9357 genes, 0.0% | 2 out of 10903 genes, 0.0% | 1 |
| response to cAMP | 2 out of 9357 genes, 0.0% | 2 out of 10903 genes, 0.0% | 1 |
| sulfation | 2 out of 9357 genes, 0.0% | 2 out of 10903 genes, 0.0% | 1 |
| convergent extension | 2 out of 9357 genes, 0.0% | 2 out of 10903 genes, 0.0% | 1 |
| canonical Wnt receptor signaling pathway | 2 out of 9357 genes, 0.0% | 2 out of 10903 genes, 0.0% | 1 |
| lung epithelium development | 2 out of 9357 genes, 0.0% | 2 out of 10903 genes, 0.0% | 1 |
| lung cell differentiation | 2 out of 9357 genes, 0.0% | 2 out of 10903 genes, 0.0% | 1 |
| lung epithelial cell differentiation | 2 out of 9357 genes, 0.0% | 2 out of 10903 genes, 0.0% | 1 |
| cell fate commitment involved in formation of primary germ layers | 2 out of 9357 genes, 0.0% | 2 out of 10903 genes, 0.0% | 1 |
| intracellular mRNA localization involved in pattern specification process | 2 out of 9357 genes, 0.0% | 2 out of 10903 genes, 0.0% | 1 |
| intracellular mRNA localization involved in anterior/posterior axis specification | 2 out of 9357 genes, 0.0% | 2 out of 10903 genes, 0.0% | 1 |
| protein-DNA complex assembly | 2 out of 9357 genes, 0.0% | 2 out of 10903 genes, 0.0% | 1 |
| proton-transporting two-sector ATPase complex assembly | 2 out of 9357 genes, 0.0% | 2 out of 10903 genes, 0.0% | 1 |
| protein localization in mitochondrion | 2 out of 9357 genes, 0.0% | 2 out of 10903 genes, 0.0% | 1 |
| regulation of leukocyte proliferation | 2 out of 9357 genes, 0.0% | 2 out of 10903 genes, 0.0% | 1 |
| protein-DNA complex subunit organization | 2 out of 9357 genes, 0.0% | 2 out of 10903 genes, 0.0% | 1 |
| kidney mesenchyme development | 2 out of 9357 genes, 0.0% | 2 out of 10903 genes, 0.0% | 1 |
| metanephric mesenchyme development | 2 out of 9357 genes, 0.0% | 2 out of 10903 genes, 0.0% | 1 |
| vesicle uncoating | 2 out of 9357 genes, 0.0% | 2 out of 10903 genes, 0.0% | 1 |
| negative regulation of transcription regulator activity | 2 out of 9357 genes, 0.0% | 2 out of 10903 genes, 0.0% | 1 |
| metabolic process | 5097 out of 9357 genes, 54.5% | 5952 out of 10903 genes, 54.6% | 1 |
| cytokinetic cell separation | 6 out of 9357 genes, 0.1% | 7 out of 10903 genes, 0.1% | 1 |
| tryptophan metabolic process | 6 out of 9357 genes, 0.1% | 7 out of 10903 genes, 0.1% | 1 |
| ceramide metabolic process | 6 out of 9357 genes, 0.1% | 7 out of 10903 genes, 0.1% | 1 |
| memory | 6 out of 9357 genes, 0.1% | 7 out of 10903 genes, 0.1% | 1 |
| fertilization | 6 out of 9357 genes, 0.1% | 7 out of 10903 genes, 0.1% | 1 |
| NADH dehydrogenase complex assembly | 6 out of 9357 genes, 0.1% | 7 out of 10903 genes, 0.1% | 1 |
| regulation of receptor activity | 6 out of 9357 genes, 0.1% | 7 out of 10903 genes, 0.1% | 1 |
| response to isoquinoline alkaloid | 6 out of 9357 genes, 0.1% | 7 out of 10903 genes, 0.1% | 1 |
| spinal cord development | 6 out of 9357 genes, 0.1% | 7 out of 10903 genes, 0.1% | 1 |
| cerebellum development | 6 out of 9357 genes, 0.1% | 7 out of 10903 genes, 0.1% | 1 |
| pancreas development | 6 out of 9357 genes, 0.1% | 7 out of 10903 genes, 0.1% | 1 |
| positive regulation of protein complex assembly | 6 out of 9357 genes, 0.1% | 7 out of 10903 genes, 0.1% | 1 |
| chitin-based cuticle development | 6 out of 9357 genes, 0.1% | 7 out of 10903 genes, 0.1% | 1 |
| dopamine metabolic process | 6 out of 9357 genes, 0.1% | 7 out of 10903 genes, 0.1% | 1 |
| response to alkaloid | 6 out of 9357 genes, 0.1% | 7 out of 10903 genes, 0.1% | 1 |
| interspecies interaction between organisms | 6 out of 9357 genes, 0.1% | 7 out of 10903 genes, 0.1% | 1 |
| Golgi vesicle budding | 6 out of 9357 genes, 0.1% | 7 out of 10903 genes, 0.1% | 1 |
| vesicle targeting, to, from or within Golgi | 6 out of 9357 genes, 0.1% | 7 out of 10903 genes, 0.1% | 1 |
| Golgi transport vesicle coating | 6 out of 9357 genes, 0.1% | 7 out of 10903 genes, 0.1% | 1 |
| circadian behavior | 6 out of 9357 genes, 0.1% | 7 out of 10903 genes, 0.1% | 1 |
| regulation of viral reproduction | 6 out of 9357 genes, 0.1% | 7 out of 10903 genes, 0.1% | 1 |
| transforming growth factor-beta production | 6 out of 9357 genes, 0.1% | 7 out of 10903 genes, 0.1% | 1 |
| respiratory electron transport chain | 36 out of 9357 genes, 0.4% | 43 out of 10903 genes, 0.4% | 1 |
| nucleic acid transport | 36 out of 9357 genes, 0.4% | 43 out of 10903 genes, 0.4% | 1 |
| response to extracellular stimulus | 52 out of 9357 genes, 0.6% | 62 out of 10903 genes, 0.6% | 1 |
| male gamete generation | 52 out of 9357 genes, 0.6% | 62 out of 10903 genes, 0.6% | 1 |
| skeletal myofibril assembly | 20 out of 9357 genes, 0.2% | 24 out of 10903 genes, 0.2% | 1 |
| regulation of protein localization | 20 out of 9357 genes, 0.2% | 24 out of 10903 genes, 0.2% | 1 |
| sensory perception of mechanical stimulus | 20 out of 9357 genes, 0.2% | 24 out of 10903 genes, 0.2% | 1 |
| negative regulation of RNA metabolic process | 20 out of 9357 genes, 0.2% | 24 out of 10903 genes, 0.2% | 1 |
| actin filament organization | 46 out of 9357 genes, 0.5% | 55 out of 10903 genes, 0.5% | 1 |
| heterocycle catabolic process | 46 out of 9357 genes, 0.5% | 55 out of 10903 genes, 0.5% | 1 |
| negative regulation of transcription from RNA polymerase II promoter | 15 out of 9357 genes, 0.2% | 18 out of 10903 genes, 0.2% | 1 |
| response to reactive oxygen species | 15 out of 9357 genes, 0.2% | 18 out of 10903 genes, 0.2% | 1 |
| cell fate determination | 15 out of 9357 genes, 0.2% | 18 out of 10903 genes, 0.2% | 1 |
| positive regulation of protein modification process | 15 out of 9357 genes, 0.2% | 18 out of 10903 genes, 0.2% | 1 |
| cilium assembly | 15 out of 9357 genes, 0.2% | 18 out of 10903 genes, 0.2% | 1 |
| signal transduction in response to DNA damage | 15 out of 9357 genes, 0.2% | 18 out of 10903 genes, 0.2% | 1 |
| cilium morphogenesis | 15 out of 9357 genes, 0.2% | 18 out of 10903 genes, 0.2% | 1 |
| establishment or maintenance of bipolar cell polarity | 15 out of 9357 genes, 0.2% | 18 out of 10903 genes, 0.2% | 1 |
| organic acid transport | 25 out of 9357 genes, 0.3% | 30 out of 10903 genes, 0.3% | 1 |
| vesicle docking | 25 out of 9357 genes, 0.3% | 30 out of 10903 genes, 0.3% | 1 |
| gland development | 25 out of 9357 genes, 0.3% | 30 out of 10903 genes, 0.3% | 1 |
| ribonucleoside triphosphate metabolic process | 134 out of 9357 genes, 1.4% | 159 out of 10903 genes, 1.5% | 1 |
| hexose catabolic process | 62 out of 9357 genes, 0.7% | 74 out of 10903 genes, 0.7% | 1 |
| alcohol catabolic process | 62 out of 9357 genes, 0.7% | 74 out of 10903 genes, 0.7% | 1 |
| monosaccharide catabolic process | 62 out of 9357 genes, 0.7% | 74 out of 10903 genes, 0.7% | 1 |
| regulation of behavior | 62 out of 9357 genes, 0.7% | 74 out of 10903 genes, 0.7% | 1 |
| primary metabolic process | 4120 out of 9357 genes, 44.0% | 4815 out of 10903 genes, 44.2% | 1 |
| RNA transport | 35 out of 9357 genes, 0.4% | 42 out of 10903 genes, 0.4% | 1 |
| establishment of RNA localization | 35 out of 9357 genes, 0.4% | 42 out of 10903 genes, 0.4% | 1 |
| purine nucleotide metabolic process | 184 out of 9357 genes, 2.0% | 218 out of 10903 genes, 2.0% | 1 |
| nematode larval development | 281 out of 9357 genes, 3.0% | 332 out of 10903 genes, 3.0% | 1 |
| aminoglycan biosynthetic process | 10 out of 9357 genes, 0.1% | 12 out of 10903 genes, 0.1% | 1 |
| mRNA export from nucleus | 10 out of 9357 genes, 0.1% | 12 out of 10903 genes, 0.1% | 1 |
| glutamate metabolic process | 10 out of 9357 genes, 0.1% | 12 out of 10903 genes, 0.1% | 1 |
| periodic partitioning | 10 out of 9357 genes, 0.1% | 12 out of 10903 genes, 0.1% | 1 |
| cellular amino acid biosynthetic process | 10 out of 9357 genes, 0.1% | 12 out of 10903 genes, 0.1% | 1 |
| anterior/posterior axis specification | 10 out of 9357 genes, 0.1% | 12 out of 10903 genes, 0.1% | 1 |
| cyclic-nucleotide-mediated signaling | 10 out of 9357 genes, 0.1% | 12 out of 10903 genes, 0.1% | 1 |
| histone lysine methylation | 10 out of 9357 genes, 0.1% | 12 out of 10903 genes, 0.1% | 1 |
| cardiac cell differentiation | 10 out of 9357 genes, 0.1% | 12 out of 10903 genes, 0.1% | 1 |
| positive regulation of nucleocytoplasmic transport | 10 out of 9357 genes, 0.1% | 12 out of 10903 genes, 0.1% | 1 |
| regulation of synaptic plasticity | 10 out of 9357 genes, 0.1% | 12 out of 10903 genes, 0.1% | 1 |
| response to organic substance | 150 out of 9357 genes, 1.6% | 178 out of 10903 genes, 1.6% | 1 |
| gene expression | 1307 out of 9357 genes, 14.0% | 1533 out of 10903 genes, 14.1% | 1 |
| chromosome segregation | 94 out of 9357 genes, 1.0% | 112 out of 10903 genes, 1.0% | 1 |
| gene silencing by RNA | 94 out of 9357 genes, 1.0% | 112 out of 10903 genes, 1.0% | 1 |
| regulation of hydrolase activity | 94 out of 9357 genes, 1.0% | 112 out of 10903 genes, 1.0% | 1 |
| positive regulation of metabolic process | 72 out of 9357 genes, 0.8% | 86 out of 10903 genes, 0.8% | 1 |
| glucose catabolic process | 61 out of 9357 genes, 0.7% | 73 out of 10903 genes, 0.7% | 1 |
| cell-cell adhesion | 61 out of 9357 genes, 0.7% | 73 out of 10903 genes, 0.7% | 1 |
| regulation of GTPase activity | 61 out of 9357 genes, 0.7% | 73 out of 10903 genes, 0.7% | 1 |
| inorganic anion transport | 45 out of 9357 genes, 0.5% | 54 out of 10903 genes, 0.5% | 1 |
| nucleotide biosynthetic process | 166 out of 9357 genes, 1.8% | 197 out of 10903 genes, 1.8% | 1 |
| intracellular protein transport | 77 out of 9357 genes, 0.8% | 92 out of 10903 genes, 0.8% | 1 |
| purine nucleoside triphosphate metabolic process | 132 out of 9357 genes, 1.4% | 157 out of 10903 genes, 1.4% | 1 |
| purine ribonucleoside triphosphate metabolic process | 132 out of 9357 genes, 1.4% | 157 out of 10903 genes, 1.4% | 1 |
| transmembrane receptor protein serine/threonine kinase signaling pathway | 50 out of 9357 genes, 0.5% | 60 out of 10903 genes, 0.6% | 1 |
| molting cycle | 279 out of 9357 genes, 3.0% | 330 out of 10903 genes, 3.0% | 1 |
| nucleobase, nucleoside and nucleotide metabolic process | 273 out of 9357 genes, 2.9% | 323 out of 10903 genes, 3.0% | 1 |
| lipid catabolic process | 24 out of 9357 genes, 0.3% | 29 out of 10903 genes, 0.3% | 1 |
| hermaphrodite germ-line sex determination | 24 out of 9357 genes, 0.3% | 29 out of 10903 genes, 0.3% | 1 |
| cellular lipid catabolic process | 24 out of 9357 genes, 0.3% | 29 out of 10903 genes, 0.3% | 1 |
| carboxylic acid transport | 24 out of 9357 genes, 0.3% | 29 out of 10903 genes, 0.3% | 1 |
| organic acid biosynthetic process | 29 out of 9357 genes, 0.3% | 35 out of 10903 genes, 0.3% | 1 |
| carboxylic acid biosynthetic process | 29 out of 9357 genes, 0.3% | 35 out of 10903 genes, 0.3% | 1 |
| developmental cell growth | 29 out of 9357 genes, 0.3% | 35 out of 10903 genes, 0.3% | 1 |
| cellular response to organic substance | 19 out of 9357 genes, 0.2% | 23 out of 10903 genes, 0.2% | 1 |
| oligosaccharide metabolic process | 34 out of 9357 genes, 0.4% | 41 out of 10903 genes, 0.4% | 1 |
| developmental growth | 34 out of 9357 genes, 0.4% | 41 out of 10903 genes, 0.4% | 1 |
| macromolecule catabolic process | 324 out of 9357 genes, 3.5% | 383 out of 10903 genes, 3.5% | 1 |
| development of primary sexual characteristics | 98 out of 9357 genes, 1.0% | 117 out of 10903 genes, 1.1% | 1 |
| RNA catabolic process | 39 out of 9357 genes, 0.4% | 47 out of 10903 genes, 0.4% | 1 |
| purine ribonucleotide catabolic process | 39 out of 9357 genes, 0.4% | 47 out of 10903 genes, 0.4% | 1 |
| ribonucleotide catabolic process | 39 out of 9357 genes, 0.4% | 47 out of 10903 genes, 0.4% | 1 |
| positive regulation of signal transduction | 14 out of 9357 genes, 0.1% | 17 out of 10903 genes, 0.2% | 1 |
| positive regulation of signaling process | 14 out of 9357 genes, 0.1% | 17 out of 10903 genes, 0.2% | 1 |
| anatomical structure homeostasis | 14 out of 9357 genes, 0.1% | 17 out of 10903 genes, 0.2% | 1 |
| posttranscriptional gene silencing | 92 out of 9357 genes, 1.0% | 110 out of 10903 genes, 1.0% | 1 |
| posttranscriptional gene silencing by RNA | 92 out of 9357 genes, 1.0% | 110 out of 10903 genes, 1.0% | 1 |
| nucleoside triphosphate metabolic process | 136 out of 9357 genes, 1.5% | 162 out of 10903 genes, 1.5% | 1 |
| regulation of cell-matrix adhesion | 5 out of 9357 genes, 0.1% | 6 out of 10903 genes, 0.1% | 1 |
| regulation of immune effector process | 5 out of 9357 genes, 0.1% | 6 out of 10903 genes, 0.1% | 1 |
| regulation of response to biotic stimulus | 5 out of 9357 genes, 0.1% | 6 out of 10903 genes, 0.1% | 1 |
| purine base metabolic process | 5 out of 9357 genes, 0.1% | 6 out of 10903 genes, 0.1% | 1 |
| L-serine metabolic process | 5 out of 9357 genes, 0.1% | 6 out of 10903 genes, 0.1% | 1 |
| heme biosynthetic process | 5 out of 9357 genes, 0.1% | 6 out of 10903 genes, 0.1% | 1 |
| receptor-mediated endocytosis | 5 out of 9357 genes, 0.1% | 6 out of 10903 genes, 0.1% | 1 |
| vesicle docking involved in exocytosis | 5 out of 9357 genes, 0.1% | 6 out of 10903 genes, 0.1% | 1 |
| regulation of epidermal growth factor receptor activity | 5 out of 9357 genes, 0.1% | 6 out of 10903 genes, 0.1% | 1 |
| activation of phospholipase C activity by G-protein coupled receptor protein signaling pathway coupled to IP3 second messenger | 5 out of 9357 genes, 0.1% | 6 out of 10903 genes, 0.1% | 1 |
| activation of phospholipase C activity | 5 out of 9357 genes, 0.1% | 6 out of 10903 genes, 0.1% | 1 |
| body fluid secretion | 5 out of 9357 genes, 0.1% | 6 out of 10903 genes, 0.1% | 1 |
| visual behavior | 5 out of 9357 genes, 0.1% | 6 out of 10903 genes, 0.1% | 1 |
| regulation of smoothened signaling pathway | 5 out of 9357 genes, 0.1% | 6 out of 10903 genes, 0.1% | 1 |
| lipoic acid biosynthetic process | 5 out of 9357 genes, 0.1% | 6 out of 10903 genes, 0.1% | 1 |
| deoxyribonucleoside metabolic process | 5 out of 9357 genes, 0.1% | 6 out of 10903 genes, 0.1% | 1 |
| pyrimidine deoxyribonucleotide metabolic process | 5 out of 9357 genes, 0.1% | 6 out of 10903 genes, 0.1% | 1 |
| deoxyribonucleotide metabolic process | 5 out of 9357 genes, 0.1% | 6 out of 10903 genes, 0.1% | 1 |
| 2'-deoxyribonucleotide metabolic process | 5 out of 9357 genes, 0.1% | 6 out of 10903 genes, 0.1% | 1 |
| response to heat | 5 out of 9357 genes, 0.1% | 6 out of 10903 genes, 0.1% | 1 |
| positive regulation of phospholipase activity | 5 out of 9357 genes, 0.1% | 6 out of 10903 genes, 0.1% | 1 |
| regulation of cell-substrate adhesion | 5 out of 9357 genes, 0.1% | 6 out of 10903 genes, 0.1% | 1 |
| positive regulation of phospholipase C activity | 5 out of 9357 genes, 0.1% | 6 out of 10903 genes, 0.1% | 1 |
| cell differentiation in spinal cord | 5 out of 9357 genes, 0.1% | 6 out of 10903 genes, 0.1% | 1 |
| cell differentiation in hindbrain | 5 out of 9357 genes, 0.1% | 6 out of 10903 genes, 0.1% | 1 |
| cerebellar cortex development | 5 out of 9357 genes, 0.1% | 6 out of 10903 genes, 0.1% | 1 |
| circadian sleep/wake cycle process | 5 out of 9357 genes, 0.1% | 6 out of 10903 genes, 0.1% | 1 |
| regulation of transmembrane transporter activity | 5 out of 9357 genes, 0.1% | 6 out of 10903 genes, 0.1% | 1 |
| adaptation of signaling pathway | 5 out of 9357 genes, 0.1% | 6 out of 10903 genes, 0.1% | 1 |
| cellular monovalent inorganic cation homeostasis | 5 out of 9357 genes, 0.1% | 6 out of 10903 genes, 0.1% | 1 |
| microspike assembly | 5 out of 9357 genes, 0.1% | 6 out of 10903 genes, 0.1% | 1 |
| regulation of cellular pH | 5 out of 9357 genes, 0.1% | 6 out of 10903 genes, 0.1% | 1 |
| cellular response to extracellular stimulus | 5 out of 9357 genes, 0.1% | 6 out of 10903 genes, 0.1% | 1 |
| cellular response to nutrient levels | 5 out of 9357 genes, 0.1% | 6 out of 10903 genes, 0.1% | 1 |
| negative regulation of transporter activity | 5 out of 9357 genes, 0.1% | 6 out of 10903 genes, 0.1% | 1 |
| regulation of ion transmembrane transporter activity | 5 out of 9357 genes, 0.1% | 6 out of 10903 genes, 0.1% | 1 |
| response to lipid | 5 out of 9357 genes, 0.1% | 6 out of 10903 genes, 0.1% | 1 |
| cell junction maintenance | 5 out of 9357 genes, 0.1% | 6 out of 10903 genes, 0.1% | 1 |
| protein localization to chromosome | 5 out of 9357 genes, 0.1% | 6 out of 10903 genes, 0.1% | 1 |
| cellular response to oxidative stress | 5 out of 9357 genes, 0.1% | 6 out of 10903 genes, 0.1% | 1 |
| compound eye photoreceptor development | 5 out of 9357 genes, 0.1% | 6 out of 10903 genes, 0.1% | 1 |
| regulation of epidermal growth factor receptor signaling pathway | 5 out of 9357 genes, 0.1% | 6 out of 10903 genes, 0.1% | 1 |
| heme metabolic process | 5 out of 9357 genes, 0.1% | 6 out of 10903 genes, 0.1% | 1 |
| hemocyte differentiation | 5 out of 9357 genes, 0.1% | 6 out of 10903 genes, 0.1% | 1 |
| circadian sleep/wake cycle | 5 out of 9357 genes, 0.1% | 6 out of 10903 genes, 0.1% | 1 |
| peroxisomal transport | 5 out of 9357 genes, 0.1% | 6 out of 10903 genes, 0.1% | 1 |
| regulation of multi-organism process | 5 out of 9357 genes, 0.1% | 6 out of 10903 genes, 0.1% | 1 |
| symbiosis, encompassing mutualism through parasitism | 5 out of 9357 genes, 0.1% | 6 out of 10903 genes, 0.1% | 1 |
| regulation of centrosome cycle | 5 out of 9357 genes, 0.1% | 6 out of 10903 genes, 0.1% | 1 |
| phosphoinositide-mediated signaling | 5 out of 9357 genes, 0.1% | 6 out of 10903 genes, 0.1% | 1 |
| positive regulation of secretion | 5 out of 9357 genes, 0.1% | 6 out of 10903 genes, 0.1% | 1 |
| positive regulation of transcription factor activity | 5 out of 9357 genes, 0.1% | 6 out of 10903 genes, 0.1% | 1 |
| interaction with host | 5 out of 9357 genes, 0.1% | 6 out of 10903 genes, 0.1% | 1 |
| positive regulation of lipase activity | 5 out of 9357 genes, 0.1% | 6 out of 10903 genes, 0.1% | 1 |
| regulation of protein tyrosine kinase activity | 5 out of 9357 genes, 0.1% | 6 out of 10903 genes, 0.1% | 1 |
| cellular response to external stimulus | 5 out of 9357 genes, 0.1% | 6 out of 10903 genes, 0.1% | 1 |
| cellular component maintenance at cellular level | 5 out of 9357 genes, 0.1% | 6 out of 10903 genes, 0.1% | 1 |
| positive regulation of transcription regulator activity | 5 out of 9357 genes, 0.1% | 6 out of 10903 genes, 0.1% | 1 |
| regulation of nucleotide metabolic process | 75 out of 9357 genes, 0.8% | 90 out of 10903 genes, 0.8% | 1 |
| purine nucleotide biosynthetic process | 135 out of 9357 genes, 1.4% | 161 out of 10903 genes, 1.5% | 1 |
| small molecule catabolic process | 146 out of 9357 genes, 1.6% | 174 out of 10903 genes, 1.6% | 1 |
| protein maturation | 33 out of 9357 genes, 0.4% | 40 out of 10903 genes, 0.4% | 1 |
| response to osmotic stress | 23 out of 9357 genes, 0.2% | 28 out of 10903 genes, 0.3% | 1 |
| fatty acid catabolic process | 23 out of 9357 genes, 0.2% | 28 out of 10903 genes, 0.3% | 1 |
| nuclear export | 23 out of 9357 genes, 0.2% | 28 out of 10903 genes, 0.3% | 1 |
| monocarboxylic acid catabolic process | 23 out of 9357 genes, 0.2% | 28 out of 10903 genes, 0.3% | 1 |
| galactose metabolic process | 9 out of 9357 genes, 0.1% | 11 out of 10903 genes, 0.1% | 1 |
| transcription from RNA polymerase II promoter | 9 out of 9357 genes, 0.1% | 11 out of 10903 genes, 0.1% | 1 |
| ubiquinone metabolic process | 9 out of 9357 genes, 0.1% | 11 out of 10903 genes, 0.1% | 1 |
| G-protein signaling, coupled to cyclic nucleotide second messenger | 9 out of 9357 genes, 0.1% | 11 out of 10903 genes, 0.1% | 1 |
| positive regulation of organelle organization | 9 out of 9357 genes, 0.1% | 11 out of 10903 genes, 0.1% | 1 |
| regulation of neuron projection development | 9 out of 9357 genes, 0.1% | 11 out of 10903 genes, 0.1% | 1 |
| fatty acid oxidation | 9 out of 9357 genes, 0.1% | 11 out of 10903 genes, 0.1% | 1 |
| central nervous system neuron differentiation | 9 out of 9357 genes, 0.1% | 11 out of 10903 genes, 0.1% | 1 |
| regulation of Rho GTPase activity | 9 out of 9357 genes, 0.1% | 11 out of 10903 genes, 0.1% | 1 |
| regulation of microtubule-based process | 9 out of 9357 genes, 0.1% | 11 out of 10903 genes, 0.1% | 1 |
| lipid oxidation | 9 out of 9357 genes, 0.1% | 11 out of 10903 genes, 0.1% | 1 |
| regulation of Rho protein signal transduction | 9 out of 9357 genes, 0.1% | 11 out of 10903 genes, 0.1% | 1 |
| quinone cofactor metabolic process | 9 out of 9357 genes, 0.1% | 11 out of 10903 genes, 0.1% | 1 |
| sphingoid metabolic process | 9 out of 9357 genes, 0.1% | 11 out of 10903 genes, 0.1% | 1 |
| molting cycle, protein-based cuticle | 275 out of 9357 genes, 2.9% | 326 out of 10903 genes, 3.0% | 1 |
| muscle system process | 43 out of 9357 genes, 0.5% | 52 out of 10903 genes, 0.5% | 1 |
| excretion | 43 out of 9357 genes, 0.5% | 52 out of 10903 genes, 0.5% | 1 |
| glycogen metabolic process | 18 out of 9357 genes, 0.2% | 22 out of 10903 genes, 0.2% | 1 |
| cellular glucan metabolic process | 18 out of 9357 genes, 0.2% | 22 out of 10903 genes, 0.2% | 1 |
| energy reserve metabolic process | 18 out of 9357 genes, 0.2% | 22 out of 10903 genes, 0.2% | 1 |
| glucan metabolic process | 18 out of 9357 genes, 0.2% | 22 out of 10903 genes, 0.2% | 1 |
| regulation of GTP catabolic process | 69 out of 9357 genes, 0.7% | 83 out of 10903 genes, 0.8% | 1 |
| ribosome biogenesis | 85 out of 9357 genes, 0.9% | 102 out of 10903 genes, 0.9% | 1 |
| vasculature development | 13 out of 9357 genes, 0.1% | 16 out of 10903 genes, 0.1% | 1 |
| porphyrin metabolic process | 13 out of 9357 genes, 0.1% | 16 out of 10903 genes, 0.1% | 1 |
| porphyrin biosynthetic process | 13 out of 9357 genes, 0.1% | 16 out of 10903 genes, 0.1% | 1 |
| vacuolar transport | 13 out of 9357 genes, 0.1% | 16 out of 10903 genes, 0.1% | 1 |
| female meiosis | 13 out of 9357 genes, 0.1% | 16 out of 10903 genes, 0.1% | 1 |
| tetrapyrrole metabolic process | 13 out of 9357 genes, 0.1% | 16 out of 10903 genes, 0.1% | 1 |
| tetrapyrrole biosynthetic process | 13 out of 9357 genes, 0.1% | 16 out of 10903 genes, 0.1% | 1 |
| molting cycle, collagen and cuticulin-based cuticle | 37 out of 9357 genes, 0.4% | 45 out of 10903 genes, 0.4% | 1 |
| nucleobase, nucleoside, nucleotide and nucleic acid transport | 42 out of 9357 genes, 0.4% | 51 out of 10903 genes, 0.5% | 1 |
| formation of primary germ layer | 27 out of 9357 genes, 0.3% | 33 out of 10903 genes, 0.3% | 1 |
| protein localization to organelle | 27 out of 9357 genes, 0.3% | 33 out of 10903 genes, 0.3% | 1 |
| negative regulation of apoptosis | 47 out of 9357 genes, 0.5% | 57 out of 10903 genes, 0.5% | 1 |
| negative regulation of programmed cell death | 47 out of 9357 genes, 0.5% | 57 out of 10903 genes, 0.5% | 1 |
| negative regulation of cell death | 47 out of 9357 genes, 0.5% | 57 out of 10903 genes, 0.5% | 1 |
| cellular catabolic process | 374 out of 9357 genes, 4.0% | 443 out of 10903 genes, 4.1% | 1 |
| tail morphogenesis | 57 out of 9357 genes, 0.6% | 69 out of 10903 genes, 0.6% | 1 |
| cellular carbohydrate catabolic process | 62 out of 9357 genes, 0.7% | 75 out of 10903 genes, 0.7% | 1 |
| DNA replication | 83 out of 9357 genes, 0.9% | 100 out of 10903 genes, 0.9% | 1 |
| developmental maturation | 17 out of 9357 genes, 0.2% | 21 out of 10903 genes, 0.2% | 1 |
| DNA metabolic process | 435 out of 9357 genes, 4.6% | 515 out of 10903 genes, 4.7% | 1 |
| blood vessel development | 8 out of 9357 genes, 0.1% | 10 out of 10903 genes, 0.1% | 1 |
| tissue homeostasis | 8 out of 9357 genes, 0.1% | 10 out of 10903 genes, 0.1% | 1 |
| vascular process in circulatory system | 8 out of 9357 genes, 0.1% | 10 out of 10903 genes, 0.1% | 1 |
| aminoglycan catabolic process | 8 out of 9357 genes, 0.1% | 10 out of 10903 genes, 0.1% | 1 |
| glycerol metabolic process | 8 out of 9357 genes, 0.1% | 10 out of 10903 genes, 0.1% | 1 |
| translational elongation | 8 out of 9357 genes, 0.1% | 10 out of 10903 genes, 0.1% | 1 |
| fatty acid beta-oxidation | 8 out of 9357 genes, 0.1% | 10 out of 10903 genes, 0.1% | 1 |
| steroid biosynthetic process | 8 out of 9357 genes, 0.1% | 10 out of 10903 genes, 0.1% | 1 |
| regulation of pH | 8 out of 9357 genes, 0.1% | 10 out of 10903 genes, 0.1% | 1 |
| pentose metabolic process | 8 out of 9357 genes, 0.1% | 10 out of 10903 genes, 0.1% | 1 |
| blood vessel morphogenesis | 8 out of 9357 genes, 0.1% | 10 out of 10903 genes, 0.1% | 1 |
| neuromuscular process | 8 out of 9357 genes, 0.1% | 10 out of 10903 genes, 0.1% | 1 |
| positive regulation of protein transport | 8 out of 9357 genes, 0.1% | 10 out of 10903 genes, 0.1% | 1 |
| regulation of cell projection assembly | 8 out of 9357 genes, 0.1% | 10 out of 10903 genes, 0.1% | 1 |
| positive regulation of intracellular protein transport | 8 out of 9357 genes, 0.1% | 10 out of 10903 genes, 0.1% | 1 |
| vulval development | 141 out of 9357 genes, 1.5% | 169 out of 10903 genes, 1.6% | 1 |
| transcription | 421 out of 9357 genes, 4.5% | 499 out of 10903 genes, 4.6% | 1 |
| peptide metabolic process | 21 out of 9357 genes, 0.2% | 26 out of 10903 genes, 0.2% | 1 |
| ion transmembrane transport | 21 out of 9357 genes, 0.2% | 26 out of 10903 genes, 0.2% | 1 |
| trehalose metabolic process | 12 out of 9357 genes, 0.1% | 15 out of 10903 genes, 0.1% | 1 |
| syncytium formation | 12 out of 9357 genes, 0.1% | 15 out of 10903 genes, 0.1% | 1 |
| meiosis I | 12 out of 9357 genes, 0.1% | 15 out of 10903 genes, 0.1% | 1 |
| salivary gland development | 12 out of 9357 genes, 0.1% | 15 out of 10903 genes, 0.1% | 1 |
| cytokinetic process | 12 out of 9357 genes, 0.1% | 15 out of 10903 genes, 0.1% | 1 |
| exocrine system development | 12 out of 9357 genes, 0.1% | 15 out of 10903 genes, 0.1% | 1 |
| multicellular organismal homeostasis | 12 out of 9357 genes, 0.1% | 15 out of 10903 genes, 0.1% | 1 |
| carbohydrate catabolic process | 71 out of 9357 genes, 0.8% | 86 out of 10903 genes, 0.8% | 1 |
| mitochondrial fission | 4 out of 9357 genes, 0.0% | 5 out of 10903 genes, 0.0% | 1 |
| blastocyst growth | 4 out of 9357 genes, 0.0% | 5 out of 10903 genes, 0.0% | 1 |
| somatic diversification of immune receptors via somatic mutation | 4 out of 9357 genes, 0.0% | 5 out of 10903 genes, 0.0% | 1 |
| regulation of organ formation | 4 out of 9357 genes, 0.0% | 5 out of 10903 genes, 0.0% | 1 |
| amino sugar metabolic process | 4 out of 9357 genes, 0.0% | 5 out of 10903 genes, 0.0% | 1 |
| glucosamine metabolic process | 4 out of 9357 genes, 0.0% | 5 out of 10903 genes, 0.0% | 1 |
| pyrimidine nucleoside metabolic process | 4 out of 9357 genes, 0.0% | 5 out of 10903 genes, 0.0% | 1 |
| regulation of DNA replication | 4 out of 9357 genes, 0.0% | 5 out of 10903 genes, 0.0% | 1 |
| proline metabolic process | 4 out of 9357 genes, 0.0% | 5 out of 10903 genes, 0.0% | 1 |
| protein targeting to peroxisome | 4 out of 9357 genes, 0.0% | 5 out of 10903 genes, 0.0% | 1 |
| sodium ion transport | 4 out of 9357 genes, 0.0% | 5 out of 10903 genes, 0.0% | 1 |
| peroxisome organization | 4 out of 9357 genes, 0.0% | 5 out of 10903 genes, 0.0% | 1 |
| axon cargo transport | 4 out of 9357 genes, 0.0% | 5 out of 10903 genes, 0.0% | 1 |
| pyrimidine nucleoside triphosphate metabolic process | 4 out of 9357 genes, 0.0% | 5 out of 10903 genes, 0.0% | 1 |
| epidermal cell differentiation | 4 out of 9357 genes, 0.0% | 5 out of 10903 genes, 0.0% | 1 |
| regulation of glucose transport | 4 out of 9357 genes, 0.0% | 5 out of 10903 genes, 0.0% | 1 |
| RNA interference | 4 out of 9357 genes, 0.0% | 5 out of 10903 genes, 0.0% | 1 |
| positive regulation of actin filament polymerization | 4 out of 9357 genes, 0.0% | 5 out of 10903 genes, 0.0% | 1 |
| regulation of protein stability | 4 out of 9357 genes, 0.0% | 5 out of 10903 genes, 0.0% | 1 |
| positive regulation of protein polymerization | 4 out of 9357 genes, 0.0% | 5 out of 10903 genes, 0.0% | 1 |
| regulation of monooxygenase activity | 4 out of 9357 genes, 0.0% | 5 out of 10903 genes, 0.0% | 1 |
| directional locomotion | 4 out of 9357 genes, 0.0% | 5 out of 10903 genes, 0.0% | 1 |
| cellular response to reactive oxygen species | 4 out of 9357 genes, 0.0% | 5 out of 10903 genes, 0.0% | 1 |
| genitalia morphogenesis | 4 out of 9357 genes, 0.0% | 5 out of 10903 genes, 0.0% | 1 |
| post-embryonic hemopoiesis | 4 out of 9357 genes, 0.0% | 5 out of 10903 genes, 0.0% | 1 |
| larval lymph gland hemopoiesis | 4 out of 9357 genes, 0.0% | 5 out of 10903 genes, 0.0% | 1 |
| larval lymph gland hemocyte differentiation | 4 out of 9357 genes, 0.0% | 5 out of 10903 genes, 0.0% | 1 |
| lamellocyte differentiation | 4 out of 9357 genes, 0.0% | 5 out of 10903 genes, 0.0% | 1 |
| fluid transport | 4 out of 9357 genes, 0.0% | 5 out of 10903 genes, 0.0% | 1 |
| intercellular bridge organization | 4 out of 9357 genes, 0.0% | 5 out of 10903 genes, 0.0% | 1 |
| regulation of neuron apoptosis | 4 out of 9357 genes, 0.0% | 5 out of 10903 genes, 0.0% | 1 |
| actin nucleation | 4 out of 9357 genes, 0.0% | 5 out of 10903 genes, 0.0% | 1 |
| regulation of viral genome replication | 4 out of 9357 genes, 0.0% | 5 out of 10903 genes, 0.0% | 1 |
| pyrimidine deoxyribonucleoside metabolic process | 4 out of 9357 genes, 0.0% | 5 out of 10903 genes, 0.0% | 1 |
| phosphatidylcholine metabolic process | 4 out of 9357 genes, 0.0% | 5 out of 10903 genes, 0.0% | 1 |
| lymph gland development | 4 out of 9357 genes, 0.0% | 5 out of 10903 genes, 0.0% | 1 |
| regulation of oxidoreductase activity | 4 out of 9357 genes, 0.0% | 5 out of 10903 genes, 0.0% | 1 |
| neural precursor cell proliferation | 4 out of 9357 genes, 0.0% | 5 out of 10903 genes, 0.0% | 1 |
| ATP biosynthetic process | 45 out of 9357 genes, 0.5% | 55 out of 10903 genes, 0.5% | 1 |
| ATP metabolic process | 45 out of 9357 genes, 0.5% | 55 out of 10903 genes, 0.5% | 1 |
| RNA localization | 40 out of 9357 genes, 0.4% | 49 out of 10903 genes, 0.4% | 1 |
| nucleoside triphosphate catabolic process | 35 out of 9357 genes, 0.4% | 43 out of 10903 genes, 0.4% | 1 |
| purine nucleoside triphosphate catabolic process | 35 out of 9357 genes, 0.4% | 43 out of 10903 genes, 0.4% | 1 |
| ribonucleoside triphosphate catabolic process | 35 out of 9357 genes, 0.4% | 43 out of 10903 genes, 0.4% | 1 |
| purine ribonucleoside triphosphate catabolic process | 35 out of 9357 genes, 0.4% | 43 out of 10903 genes, 0.4% | 1 |
| nucleus localization | 60 out of 9357 genes, 0.6% | 73 out of 10903 genes, 0.7% | 1 |
| fatty acid biosynthetic process | 16 out of 9357 genes, 0.2% | 20 out of 10903 genes, 0.2% | 1 |
| specification of symmetry | 16 out of 9357 genes, 0.2% | 20 out of 10903 genes, 0.2% | 1 |
| determination of bilateral symmetry | 16 out of 9357 genes, 0.2% | 20 out of 10903 genes, 0.2% | 1 |
| negative regulation of organelle organization | 16 out of 9357 genes, 0.2% | 20 out of 10903 genes, 0.2% | 1 |
| energy coupled proton transport, down electrochemical gradient | 16 out of 9357 genes, 0.2% | 20 out of 10903 genes, 0.2% | 1 |
| ATP synthesis coupled proton transport | 16 out of 9357 genes, 0.2% | 20 out of 10903 genes, 0.2% | 1 |
| negative regulation of response to stimulus | 16 out of 9357 genes, 0.2% | 20 out of 10903 genes, 0.2% | 1 |
| reproductive structure development | 16 out of 9357 genes, 0.2% | 20 out of 10903 genes, 0.2% | 1 |
| ribonucleoprotein complex subunit organization | 16 out of 9357 genes, 0.2% | 20 out of 10903 genes, 0.2% | 1 |
| macromolecular complex subunit organization | 227 out of 9357 genes, 2.4% | 271 out of 10903 genes, 2.5% | 1 |
| establishment of protein localization | 277 out of 9357 genes, 3.0% | 330 out of 10903 genes, 3.0% | 1 |
| protein transport | 276 out of 9357 genes, 2.9% | 329 out of 10903 genes, 3.0% | 1 |
| sulfur compound metabolic process | 54 out of 9357 genes, 0.6% | 66 out of 10903 genes, 0.6% | 1 |
| nuclear transport | 59 out of 9357 genes, 0.6% | 72 out of 10903 genes, 0.7% | 1 |
| microtubule-based movement | 20 out of 9357 genes, 0.2% | 25 out of 10903 genes, 0.2% | 1 |
| proton transport | 20 out of 9357 genes, 0.2% | 25 out of 10903 genes, 0.2% | 1 |
| small molecule biosynthetic process | 214 out of 9357 genes, 2.3% | 256 out of 10903 genes, 2.3% | 1 |
| organic acid catabolic process | 34 out of 9357 genes, 0.4% | 42 out of 10903 genes, 0.4% | 1 |
| carboxylic acid catabolic process | 34 out of 9357 genes, 0.4% | 42 out of 10903 genes, 0.4% | 1 |
| regulation of nucleotide catabolic process | 69 out of 9357 genes, 0.7% | 84 out of 10903 genes, 0.8% | 1 |
| regulation of purine nucleotide catabolic process | 69 out of 9357 genes, 0.7% | 84 out of 10903 genes, 0.8% | 1 |
| copulation | 11 out of 9357 genes, 0.1% | 14 out of 10903 genes, 0.1% | 1 |
| cellular amino acid catabolic process | 11 out of 9357 genes, 0.1% | 14 out of 10903 genes, 0.1% | 1 |
| regulation of cell adhesion | 11 out of 9357 genes, 0.1% | 14 out of 10903 genes, 0.1% | 1 |
| DNA integrity checkpoint | 11 out of 9357 genes, 0.1% | 14 out of 10903 genes, 0.1% | 1 |
| gene silencing by miRNA | 11 out of 9357 genes, 0.1% | 14 out of 10903 genes, 0.1% | 1 |
| protein maturation by peptide bond cleavage | 11 out of 9357 genes, 0.1% | 14 out of 10903 genes, 0.1% | 1 |
| catabolic process | 499 out of 9357 genes, 5.3% | 592 out of 10903 genes, 5.4% | 1 |
| mitotic sister chromatid cohesion | 7 out of 9357 genes, 0.1% | 9 out of 10903 genes, 0.1% | 1 |
| smoothened signaling pathway | 7 out of 9357 genes, 0.1% | 9 out of 10903 genes, 0.1% | 1 |
| insemination | 7 out of 9357 genes, 0.1% | 9 out of 10903 genes, 0.1% | 1 |
| adult locomotory behavior | 7 out of 9357 genes, 0.1% | 9 out of 10903 genes, 0.1% | 1 |
| aromatic amino acid family metabolic process | 7 out of 9357 genes, 0.1% | 9 out of 10903 genes, 0.1% | 1 |
| actin filament-based movement | 7 out of 9357 genes, 0.1% | 9 out of 10903 genes, 0.1% | 1 |
| positive regulation of transmembrane transport | 7 out of 9357 genes, 0.1% | 9 out of 10903 genes, 0.1% | 1 |
| positive regulation of protein import into nucleus | 7 out of 9357 genes, 0.1% | 9 out of 10903 genes, 0.1% | 1 |
| mechanoreceptor differentiation | 7 out of 9357 genes, 0.1% | 9 out of 10903 genes, 0.1% | 1 |
| second-messenger-mediated signaling | 48 out of 9357 genes, 0.5% | 59 out of 10903 genes, 0.5% | 1 |
| response to oxidative stress | 24 out of 9357 genes, 0.3% | 30 out of 10903 genes, 0.3% | 1 |
| protein processing | 24 out of 9357 genes, 0.3% | 30 out of 10903 genes, 0.3% | 1 |
| protein targeting | 63 out of 9357 genes, 0.7% | 77 out of 10903 genes, 0.7% | 1 |
| ethanolamine and derivative metabolic process | 15 out of 9357 genes, 0.2% | 19 out of 10903 genes, 0.2% | 1 |
| cell maturation | 15 out of 9357 genes, 0.2% | 19 out of 10903 genes, 0.2% | 1 |
| maintenance of location | 15 out of 9357 genes, 0.2% | 19 out of 10903 genes, 0.2% | 1 |
| protein complex assembly | 157 out of 9357 genes, 1.7% | 189 out of 10903 genes, 1.7% | 1 |
| protein complex biogenesis | 157 out of 9357 genes, 1.7% | 189 out of 10903 genes, 1.7% | 1 |
| regulation of cell growth | 19 out of 9357 genes, 0.2% | 24 out of 10903 genes, 0.2% | 1 |
| cellular amino acid derivative metabolic process | 19 out of 9357 genes, 0.2% | 24 out of 10903 genes, 0.2% | 1 |
| nucleus organization | 19 out of 9357 genes, 0.2% | 24 out of 10903 genes, 0.2% | 1 |
| mitochondrion organization | 19 out of 9357 genes, 0.2% | 24 out of 10903 genes, 0.2% | 1 |
| microtubule-based transport | 19 out of 9357 genes, 0.2% | 24 out of 10903 genes, 0.2% | 1 |
| cytoskeleton-dependent intracellular transport | 19 out of 9357 genes, 0.2% | 24 out of 10903 genes, 0.2% | 1 |
| behavioral interaction between organisms | 28 out of 9357 genes, 0.3% | 35 out of 10903 genes, 0.3% | 1 |
| mating | 37 out of 9357 genes, 0.4% | 46 out of 10903 genes, 0.4% | 1 |
| regulation of actin cytoskeleton organization | 23 out of 9357 genes, 0.2% | 29 out of 10903 genes, 0.3% | 1 |
| regulation of actin filament-based process | 23 out of 9357 genes, 0.2% | 29 out of 10903 genes, 0.3% | 1 |
| regulation of cytoskeleton organization | 32 out of 9357 genes, 0.3% | 40 out of 10903 genes, 0.4% | 1 |
| cell adhesion | 144 out of 9357 genes, 1.5% | 174 out of 10903 genes, 1.6% | 1 |
| biological adhesion | 144 out of 9357 genes, 1.5% | 174 out of 10903 genes, 1.6% | 1 |
| regulation of Ras GTPase activity | 51 out of 9357 genes, 0.5% | 63 out of 10903 genes, 0.6% | 1 |
| fatty acid metabolic process | 81 out of 9357 genes, 0.9% | 99 out of 10903 genes, 0.9% | 1 |
| syncytium formation by plasma membrane fusion | 10 out of 9357 genes, 0.1% | 13 out of 10903 genes, 0.1% | 1 |
| glutathione metabolic process | 10 out of 9357 genes, 0.1% | 13 out of 10903 genes, 0.1% | 1 |
| mitotic spindle organization | 10 out of 9357 genes, 0.1% | 13 out of 10903 genes, 0.1% | 1 |
| courtship behavior | 10 out of 9357 genes, 0.1% | 13 out of 10903 genes, 0.1% | 1 |
| behavioral signaling | 10 out of 9357 genes, 0.1% | 13 out of 10903 genes, 0.1% | 1 |
| non-canonical Wnt receptor signaling pathway | 10 out of 9357 genes, 0.1% | 13 out of 10903 genes, 0.1% | 1 |
| mesenchyme development | 10 out of 9357 genes, 0.1% | 13 out of 10903 genes, 0.1% | 1 |
| actin polymerization or depolymerization | 14 out of 9357 genes, 0.1% | 18 out of 10903 genes, 0.2% | 1 |
| amine catabolic process | 14 out of 9357 genes, 0.1% | 18 out of 10903 genes, 0.2% | 1 |
| glycoside metabolic process | 14 out of 9357 genes, 0.1% | 18 out of 10903 genes, 0.2% | 1 |
| ribonucleoprotein complex assembly | 14 out of 9357 genes, 0.1% | 18 out of 10903 genes, 0.2% | 1 |
| spliceosomal snRNP assembly | 3 out of 9357 genes, 0.0% | 4 out of 10903 genes, 0.0% | 1 |
| RNA splicing, via endonucleolytic cleavage and ligation | 3 out of 9357 genes, 0.0% | 4 out of 10903 genes, 0.0% | 1 |
| cleavage involved in rRNA processing | 3 out of 9357 genes, 0.0% | 4 out of 10903 genes, 0.0% | 1 |
| maturation of LSU-rRNA | 3 out of 9357 genes, 0.0% | 4 out of 10903 genes, 0.0% | 1 |
| endothelial cell proliferation | 3 out of 9357 genes, 0.0% | 4 out of 10903 genes, 0.0% | 1 |
| mannose metabolic process | 3 out of 9357 genes, 0.0% | 4 out of 10903 genes, 0.0% | 1 |
| regulation of DNA repair | 3 out of 9357 genes, 0.0% | 4 out of 10903 genes, 0.0% | 1 |
| icosanoid metabolic process | 3 out of 9357 genes, 0.0% | 4 out of 10903 genes, 0.0% | 1 |
| substrate-dependent cell migration | 3 out of 9357 genes, 0.0% | 4 out of 10903 genes, 0.0% | 1 |
| cytoplasm organization | 3 out of 9357 genes, 0.0% | 4 out of 10903 genes, 0.0% | 1 |
| cell-matrix adhesion | 3 out of 9357 genes, 0.0% | 4 out of 10903 genes, 0.0% | 1 |
| neuroblast proliferation | 3 out of 9357 genes, 0.0% | 4 out of 10903 genes, 0.0% | 1 |
| peripheral nervous system development | 3 out of 9357 genes, 0.0% | 4 out of 10903 genes, 0.0% | 1 |
| regulation of blood pressure | 3 out of 9357 genes, 0.0% | 4 out of 10903 genes, 0.0% | 1 |
| folic acid and derivative biosynthetic process | 3 out of 9357 genes, 0.0% | 4 out of 10903 genes, 0.0% | 1 |
| energy taxis | 3 out of 9357 genes, 0.0% | 4 out of 10903 genes, 0.0% | 1 |
| response to gravity | 3 out of 9357 genes, 0.0% | 4 out of 10903 genes, 0.0% | 1 |
| photosynthetic electron transport chain | 3 out of 9357 genes, 0.0% | 4 out of 10903 genes, 0.0% | 1 |
| positive regulation of glucose transport | 3 out of 9357 genes, 0.0% | 4 out of 10903 genes, 0.0% | 1 |
| nucleoside transport | 3 out of 9357 genes, 0.0% | 4 out of 10903 genes, 0.0% | 1 |
| purine nucleoside transport | 3 out of 9357 genes, 0.0% | 4 out of 10903 genes, 0.0% | 1 |
| photosynthesis | 3 out of 9357 genes, 0.0% | 4 out of 10903 genes, 0.0% | 1 |
| D-ribose biosynthetic process | 3 out of 9357 genes, 0.0% | 4 out of 10903 genes, 0.0% | 1 |
| pentose biosynthetic process | 3 out of 9357 genes, 0.0% | 4 out of 10903 genes, 0.0% | 1 |
| aromatic compound catabolic process | 3 out of 9357 genes, 0.0% | 4 out of 10903 genes, 0.0% | 1 |
| photosynthesis, light reaction | 3 out of 9357 genes, 0.0% | 4 out of 10903 genes, 0.0% | 1 |
| regulation of cell-cell adhesion | 3 out of 9357 genes, 0.0% | 4 out of 10903 genes, 0.0% | 1 |
| negative regulation of cell-cell adhesion | 3 out of 9357 genes, 0.0% | 4 out of 10903 genes, 0.0% | 1 |
| border follicle cell delamination | 3 out of 9357 genes, 0.0% | 4 out of 10903 genes, 0.0% | 1 |
| positive regulation of protein ubiquitination | 3 out of 9357 genes, 0.0% | 4 out of 10903 genes, 0.0% | 1 |
| unsaturated fatty acid metabolic process | 3 out of 9357 genes, 0.0% | 4 out of 10903 genes, 0.0% | 1 |
| haltere disc development | 3 out of 9357 genes, 0.0% | 4 out of 10903 genes, 0.0% | 1 |
| wound healing | 3 out of 9357 genes, 0.0% | 4 out of 10903 genes, 0.0% | 1 |
| phototaxis | 3 out of 9357 genes, 0.0% | 4 out of 10903 genes, 0.0% | 1 |
| myelination | 3 out of 9357 genes, 0.0% | 4 out of 10903 genes, 0.0% | 1 |
| regulation of circadian sleep/wake cycle | 3 out of 9357 genes, 0.0% | 4 out of 10903 genes, 0.0% | 1 |
| regulation of circadian rhythm | 3 out of 9357 genes, 0.0% | 4 out of 10903 genes, 0.0% | 1 |
| pH reduction | 3 out of 9357 genes, 0.0% | 4 out of 10903 genes, 0.0% | 1 |
| synaptic vesicle endocytosis | 3 out of 9357 genes, 0.0% | 4 out of 10903 genes, 0.0% | 1 |
| regulation of defense response to virus | 3 out of 9357 genes, 0.0% | 4 out of 10903 genes, 0.0% | 1 |
| spindle assembly | 3 out of 9357 genes, 0.0% | 4 out of 10903 genes, 0.0% | 1 |
| negative regulation of multicellular organismal process | 3 out of 9357 genes, 0.0% | 4 out of 10903 genes, 0.0% | 1 |
| regulation of cellular component movement | 3 out of 9357 genes, 0.0% | 4 out of 10903 genes, 0.0% | 1 |
| delamination | 3 out of 9357 genes, 0.0% | 4 out of 10903 genes, 0.0% | 1 |
| regulation of cellular response to stress | 3 out of 9357 genes, 0.0% | 4 out of 10903 genes, 0.0% | 1 |
| regulation of cell motility | 3 out of 9357 genes, 0.0% | 4 out of 10903 genes, 0.0% | 1 |
| rRNA metabolic process | 27 out of 9357 genes, 0.3% | 34 out of 10903 genes, 0.3% | 1 |
| regulation of cell size | 70 out of 9357 genes, 0.7% | 86 out of 10903 genes, 0.8% | 1 |
| rRNA modification | 6 out of 9357 genes, 0.1% | 8 out of 10903 genes, 0.1% | 1 |
| angiogenesis | 6 out of 9357 genes, 0.1% | 8 out of 10903 genes, 0.1% | 1 |
| RNA elongation from RNA polymerase II promoter | 6 out of 9357 genes, 0.1% | 8 out of 10903 genes, 0.1% | 1 |
| iron ion transport | 6 out of 9357 genes, 0.1% | 8 out of 10903 genes, 0.1% | 1 |
| humoral immune response | 6 out of 9357 genes, 0.1% | 8 out of 10903 genes, 0.1% | 1 |
| cell cycle arrest | 6 out of 9357 genes, 0.1% | 8 out of 10903 genes, 0.1% | 1 |
| oocyte construction | 6 out of 9357 genes, 0.1% | 8 out of 10903 genes, 0.1% | 1 |
| female gonad development | 6 out of 9357 genes, 0.1% | 8 out of 10903 genes, 0.1% | 1 |
| proximal/distal axis specification | 6 out of 9357 genes, 0.1% | 8 out of 10903 genes, 0.1% | 1 |
| proximal/distal pattern formation | 6 out of 9357 genes, 0.1% | 8 out of 10903 genes, 0.1% | 1 |
| oocyte differentiation | 6 out of 9357 genes, 0.1% | 8 out of 10903 genes, 0.1% | 1 |
| heparan sulfate proteoglycan biosynthetic process | 6 out of 9357 genes, 0.1% | 8 out of 10903 genes, 0.1% | 1 |
| diencephalon development | 6 out of 9357 genes, 0.1% | 8 out of 10903 genes, 0.1% | 1 |
| heparan sulfate proteoglycan metabolic process | 6 out of 9357 genes, 0.1% | 8 out of 10903 genes, 0.1% | 1 |
| turning behavior | 6 out of 9357 genes, 0.1% | 8 out of 10903 genes, 0.1% | 1 |
| regulation of transcription factor import into nucleus | 6 out of 9357 genes, 0.1% | 8 out of 10903 genes, 0.1% | 1 |
| oocyte development | 6 out of 9357 genes, 0.1% | 8 out of 10903 genes, 0.1% | 1 |
| regulation of blood vessel size | 6 out of 9357 genes, 0.1% | 8 out of 10903 genes, 0.1% | 1 |
| protein homooligomerization | 6 out of 9357 genes, 0.1% | 8 out of 10903 genes, 0.1% | 1 |
| inner ear receptor cell differentiation | 6 out of 9357 genes, 0.1% | 8 out of 10903 genes, 0.1% | 1 |
| monocarboxylic acid metabolic process | 115 out of 9357 genes, 1.2% | 140 out of 10903 genes, 1.3% | 1 |
| regulation of intracellular protein kinase cascade | 26 out of 9357 genes, 0.3% | 33 out of 10903 genes, 0.3% | 1 |
| mesoderm migration involved in gastrulation | 13 out of 9357 genes, 0.1% | 17 out of 10903 genes, 0.2% | 1 |
| gonad development | 13 out of 9357 genes, 0.1% | 17 out of 10903 genes, 0.2% | 1 |
| nucleoside monophosphate metabolic process | 44 out of 9357 genes, 0.5% | 55 out of 10903 genes, 0.5% | 1 |
| nucleoside monophosphate biosynthetic process | 44 out of 9357 genes, 0.5% | 55 out of 10903 genes, 0.5% | 1 |
| circulatory system process | 17 out of 9357 genes, 0.2% | 22 out of 10903 genes, 0.2% | 1 |
| purine nucleoside monophosphate metabolic process | 17 out of 9357 genes, 0.2% | 22 out of 10903 genes, 0.2% | 1 |
| purine nucleoside monophosphate biosynthetic process | 17 out of 9357 genes, 0.2% | 22 out of 10903 genes, 0.2% | 1 |
| polysaccharide catabolic process | 9 out of 9357 genes, 0.1% | 12 out of 10903 genes, 0.1% | 1 |
| vesicle fusion | 9 out of 9357 genes, 0.1% | 12 out of 10903 genes, 0.1% | 1 |
| mesenchymal cell development | 9 out of 9357 genes, 0.1% | 12 out of 10903 genes, 0.1% | 1 |
| proteoglycan biosynthetic process | 9 out of 9357 genes, 0.1% | 12 out of 10903 genes, 0.1% | 1 |
| response to dsRNA | 9 out of 9357 genes, 0.1% | 12 out of 10903 genes, 0.1% | 1 |
| mesenchymal cell differentiation | 9 out of 9357 genes, 0.1% | 12 out of 10903 genes, 0.1% | 1 |
| mesoderm development | 21 out of 9357 genes, 0.2% | 27 out of 10903 genes, 0.2% | 1 |
| carbohydrate transport | 21 out of 9357 genes, 0.2% | 27 out of 10903 genes, 0.2% | 1 |
| endosome transport | 21 out of 9357 genes, 0.2% | 27 out of 10903 genes, 0.2% | 1 |
| regulation of actin filament length | 21 out of 9357 genes, 0.2% | 27 out of 10903 genes, 0.2% | 1 |
| mesoderm morphogenesis | 21 out of 9357 genes, 0.2% | 27 out of 10903 genes, 0.2% | 1 |
| protein oligomerization | 43 out of 9357 genes, 0.5% | 54 out of 10903 genes, 0.5% | 1 |
| sulfur compound biosynthetic process | 16 out of 9357 genes, 0.2% | 21 out of 10903 genes, 0.2% | 1 |
| protein import | 12 out of 9357 genes, 0.1% | 16 out of 10903 genes, 0.1% | 1 |
| lipid modification | 12 out of 9357 genes, 0.1% | 16 out of 10903 genes, 0.1% | 1 |
| negative regulation of behavior | 12 out of 9357 genes, 0.1% | 16 out of 10903 genes, 0.1% | 1 |
| sulfur amino acid biosynthetic process | 5 out of 9357 genes, 0.1% | 7 out of 10903 genes, 0.1% | 1 |
| meiotic spindle organization | 5 out of 9357 genes, 0.1% | 7 out of 10903 genes, 0.1% | 1 |
| epithelial to mesenchymal transition | 5 out of 9357 genes, 0.1% | 7 out of 10903 genes, 0.1% | 1 |
| D-ribose metabolic process | 5 out of 9357 genes, 0.1% | 7 out of 10903 genes, 0.1% | 1 |
| folic acid and derivative metabolic process | 5 out of 9357 genes, 0.1% | 7 out of 10903 genes, 0.1% | 1 |
| lysosomal transport | 5 out of 9357 genes, 0.1% | 7 out of 10903 genes, 0.1% | 1 |
| positive regulation of cell development | 5 out of 9357 genes, 0.1% | 7 out of 10903 genes, 0.1% | 1 |
| membrane protein proteolysis | 5 out of 9357 genes, 0.1% | 7 out of 10903 genes, 0.1% | 1 |
| cellular biogenic amine catabolic process | 5 out of 9357 genes, 0.1% | 7 out of 10903 genes, 0.1% | 1 |
| cell-cell signaling involved in cell fate commitment | 5 out of 9357 genes, 0.1% | 7 out of 10903 genes, 0.1% | 1 |
| regulation of neuronal synaptic plasticity | 5 out of 9357 genes, 0.1% | 7 out of 10903 genes, 0.1% | 1 |
| regulation of microtubule cytoskeleton organization | 5 out of 9357 genes, 0.1% | 7 out of 10903 genes, 0.1% | 1 |
| regulation of cellular component size | 95 out of 9357 genes, 1.0% | 117 out of 10903 genes, 1.1% | 1 |
| lipid biosynthetic process | 51 out of 9357 genes, 0.5% | 64 out of 10903 genes, 0.6% | 1 |
| protein folding | 28 out of 9357 genes, 0.3% | 36 out of 10903 genes, 0.3% | 1 |
| transcription initiation | 46 out of 9357 genes, 0.5% | 58 out of 10903 genes, 0.5% | 1 |
| cholesterol metabolic process | 8 out of 9357 genes, 0.1% | 11 out of 10903 genes, 0.1% | 1 |
| dsRNA fragmentation | 8 out of 9357 genes, 0.1% | 11 out of 10903 genes, 0.1% | 1 |
| development of primary female sexual characteristics | 8 out of 9357 genes, 0.1% | 11 out of 10903 genes, 0.1% | 1 |
| female sex differentiation | 8 out of 9357 genes, 0.1% | 11 out of 10903 genes, 0.1% | 1 |
| production of small RNA involved in gene silencing by RNA | 8 out of 9357 genes, 0.1% | 11 out of 10903 genes, 0.1% | 1 |
| cellular response to dsRNA | 8 out of 9357 genes, 0.1% | 11 out of 10903 genes, 0.1% | 1 |
| regulation of cyclin-dependent protein kinase activity | 2 out of 9357 genes, 0.0% | 3 out of 10903 genes, 0.0% | 1 |
| leukocyte mediated cytotoxicity | 2 out of 9357 genes, 0.0% | 3 out of 10903 genes, 0.0% | 1 |
| positive regulation of neurotransmitter secretion | 2 out of 9357 genes, 0.0% | 3 out of 10903 genes, 0.0% | 1 |
| myeloid leukocyte differentiation | 2 out of 9357 genes, 0.0% | 3 out of 10903 genes, 0.0% | 1 |
| DNA methylation | 2 out of 9357 genes, 0.0% | 3 out of 10903 genes, 0.0% | 1 |
| negative regulation of protein kinase activity | 2 out of 9357 genes, 0.0% | 3 out of 10903 genes, 0.0% | 1 |
| dolichol-linked oligosaccharide biosynthetic process | 2 out of 9357 genes, 0.0% | 3 out of 10903 genes, 0.0% | 1 |
| tryptophan catabolic process | 2 out of 9357 genes, 0.0% | 3 out of 10903 genes, 0.0% | 1 |
| betaine metabolic process | 2 out of 9357 genes, 0.0% | 3 out of 10903 genes, 0.0% | 1 |
| phosphatidylserine metabolic process | 2 out of 9357 genes, 0.0% | 3 out of 10903 genes, 0.0% | 1 |
| sphinganine metabolic process | 2 out of 9357 genes, 0.0% | 3 out of 10903 genes, 0.0% | 1 |
| sphingosine metabolic process | 2 out of 9357 genes, 0.0% | 3 out of 10903 genes, 0.0% | 1 |
| leukotriene metabolic process | 2 out of 9357 genes, 0.0% | 3 out of 10903 genes, 0.0% | 1 |
| nucleotide transport | 2 out of 9357 genes, 0.0% | 3 out of 10903 genes, 0.0% | 1 |
| inflammatory response | 2 out of 9357 genes, 0.0% | 3 out of 10903 genes, 0.0% | 1 |
| isoprenoid biosynthetic process | 2 out of 9357 genes, 0.0% | 3 out of 10903 genes, 0.0% | 1 |
| pyridoxine metabolic process | 2 out of 9357 genes, 0.0% | 3 out of 10903 genes, 0.0% | 1 |
| hexose transport | 2 out of 9357 genes, 0.0% | 3 out of 10903 genes, 0.0% | 1 |
| aromatic amino acid family catabolic process | 2 out of 9357 genes, 0.0% | 3 out of 10903 genes, 0.0% | 1 |
| deoxyribonucleoside triphosphate metabolic process | 2 out of 9357 genes, 0.0% | 3 out of 10903 genes, 0.0% | 1 |
| pyrimidine deoxyribonucleoside triphosphate metabolic process | 2 out of 9357 genes, 0.0% | 3 out of 10903 genes, 0.0% | 1 |
| oligosaccharide biosynthetic process | 2 out of 9357 genes, 0.0% | 3 out of 10903 genes, 0.0% | 1 |
| Schwann cell differentiation | 2 out of 9357 genes, 0.0% | 3 out of 10903 genes, 0.0% | 1 |
| muscle cell migration | 2 out of 9357 genes, 0.0% | 3 out of 10903 genes, 0.0% | 1 |
| monosaccharide transport | 2 out of 9357 genes, 0.0% | 3 out of 10903 genes, 0.0% | 1 |
| neutral amino acid transport | 2 out of 9357 genes, 0.0% | 3 out of 10903 genes, 0.0% | 1 |
| purine nucleotide transport | 2 out of 9357 genes, 0.0% | 3 out of 10903 genes, 0.0% | 1 |
| purine ribonucleotide transport | 2 out of 9357 genes, 0.0% | 3 out of 10903 genes, 0.0% | 1 |
| Rac protein signal transduction | 2 out of 9357 genes, 0.0% | 3 out of 10903 genes, 0.0% | 1 |
| ventral spinal cord development | 2 out of 9357 genes, 0.0% | 3 out of 10903 genes, 0.0% | 1 |
| spinal cord motor neuron differentiation | 2 out of 9357 genes, 0.0% | 3 out of 10903 genes, 0.0% | 1 |
| developmental induction | 2 out of 9357 genes, 0.0% | 3 out of 10903 genes, 0.0% | 1 |
| biomineral tissue development | 2 out of 9357 genes, 0.0% | 3 out of 10903 genes, 0.0% | 1 |
| negative regulation of ion transmembrane transporter activity | 2 out of 9357 genes, 0.0% | 3 out of 10903 genes, 0.0% | 1 |
| hermaphrodite somatic sex determination | 2 out of 9357 genes, 0.0% | 3 out of 10903 genes, 0.0% | 1 |
| glial cell growth | 2 out of 9357 genes, 0.0% | 3 out of 10903 genes, 0.0% | 1 |
| indole derivative catabolic process | 2 out of 9357 genes, 0.0% | 3 out of 10903 genes, 0.0% | 1 |
| vitamin B6 metabolic process | 2 out of 9357 genes, 0.0% | 3 out of 10903 genes, 0.0% | 1 |
| peptide biosynthetic process | 2 out of 9357 genes, 0.0% | 3 out of 10903 genes, 0.0% | 1 |
| negative regulation of MAP kinase activity | 2 out of 9357 genes, 0.0% | 3 out of 10903 genes, 0.0% | 1 |
| cellular alkene metabolic process | 2 out of 9357 genes, 0.0% | 3 out of 10903 genes, 0.0% | 1 |
| regulation of RNA splicing | 2 out of 9357 genes, 0.0% | 3 out of 10903 genes, 0.0% | 1 |
| wound healing, spreading of cells | 2 out of 9357 genes, 0.0% | 3 out of 10903 genes, 0.0% | 1 |
| fat cell differentiation | 2 out of 9357 genes, 0.0% | 3 out of 10903 genes, 0.0% | 1 |
| indolalkylamine catabolic process | 2 out of 9357 genes, 0.0% | 3 out of 10903 genes, 0.0% | 1 |
| regulation of neurotransmitter secretion | 2 out of 9357 genes, 0.0% | 3 out of 10903 genes, 0.0% | 1 |
| cardiac muscle tissue development | 2 out of 9357 genes, 0.0% | 3 out of 10903 genes, 0.0% | 1 |
| regulation of neurotransmitter transport | 2 out of 9357 genes, 0.0% | 3 out of 10903 genes, 0.0% | 1 |
| positive regulation of neurotransmitter transport | 2 out of 9357 genes, 0.0% | 3 out of 10903 genes, 0.0% | 1 |
| DNA biosynthetic process | 2 out of 9357 genes, 0.0% | 3 out of 10903 genes, 0.0% | 1 |
| negative regulation of protein serine/threonine kinase activity | 2 out of 9357 genes, 0.0% | 3 out of 10903 genes, 0.0% | 1 |
| RNA elongation | 27 out of 9357 genes, 0.3% | 35 out of 10903 genes, 0.3% | 1 |
| carbohydrate biosynthetic process | 31 out of 9357 genes, 0.3% | 40 out of 10903 genes, 0.4% | 1 |
| protein ubiquitination | 35 out of 9357 genes, 0.4% | 45 out of 10903 genes, 0.4% | 1 |
| cellular lipid metabolic process | 214 out of 9357 genes, 2.3% | 260 out of 10903 genes, 2.4% | 1 |
| mating behavior | 22 out of 9357 genes, 0.2% | 29 out of 10903 genes, 0.3% | 1 |
| mesoderm formation | 18 out of 9357 genes, 0.2% | 24 out of 10903 genes, 0.2% | 1 |
| lipid metabolic process | 304 out of 9357 genes, 3.2% | 367 out of 10903 genes, 3.4% | 1 |
| sterol metabolic process | 14 out of 9357 genes, 0.1% | 19 out of 10903 genes, 0.2% | 1 |
| tissue migration | 14 out of 9357 genes, 0.1% | 19 out of 10903 genes, 0.2% | 1 |
| DNA damage checkpoint | 7 out of 9357 genes, 0.1% | 10 out of 10903 genes, 0.1% | 1 |
| production of miRNAs involved in gene silencing by miRNA | 7 out of 9357 genes, 0.1% | 10 out of 10903 genes, 0.1% | 1 |
| regulation of protein complex disassembly | 7 out of 9357 genes, 0.1% | 10 out of 10903 genes, 0.1% | 1 |
| assembly of actomyosin apparatus involved in cell cycle cytokinesis | 4 out of 9357 genes, 0.0% | 6 out of 10903 genes, 0.1% | 1 |
| fucose metabolic process | 4 out of 9357 genes, 0.0% | 6 out of 10903 genes, 0.1% | 1 |
| misfolded or incompletely synthesized protein catabolic process | 4 out of 9357 genes, 0.0% | 6 out of 10903 genes, 0.1% | 1 |
| oocyte axis specification | 4 out of 9357 genes, 0.0% | 6 out of 10903 genes, 0.1% | 1 |
| male courtship behavior | 4 out of 9357 genes, 0.0% | 6 out of 10903 genes, 0.1% | 1 |
| positive regulation of transcription factor import into nucleus | 4 out of 9357 genes, 0.0% | 6 out of 10903 genes, 0.1% | 1 |
| proteoglycan metabolic process | 10 out of 9357 genes, 0.1% | 14 out of 10903 genes, 0.1% | 1 |
| organelle fusion | 10 out of 9357 genes, 0.1% | 14 out of 10903 genes, 0.1% | 1 |
| regulation of MAPKKK cascade | 21 out of 9357 genes, 0.2% | 28 out of 10903 genes, 0.3% | 1 |
| response to steroid hormone stimulus | 21 out of 9357 genes, 0.2% | 28 out of 10903 genes, 0.3% | 1 |
| regulation of actin polymerization or depolymerization | 17 out of 9357 genes, 0.2% | 23 out of 10903 genes, 0.2% | 1 |
| macromolecular complex assembly | 183 out of 9357 genes, 2.0% | 224 out of 10903 genes, 2.1% | 1 |
| cellular carbohydrate biosynthetic process | 13 out of 9357 genes, 0.1% | 18 out of 10903 genes, 0.2% | 1 |
| cell-substrate adhesion | 28 out of 9357 genes, 0.3% | 37 out of 10903 genes, 0.3% | 1 |
| regulation of heart contraction | 6 out of 9357 genes, 0.1% | 9 out of 10903 genes, 0.1% | 1 |
| nucleotide-sugar transport | 6 out of 9357 genes, 0.1% | 9 out of 10903 genes, 0.1% | 1 |
| pyrimidine nucleotide-sugar transport | 6 out of 9357 genes, 0.1% | 9 out of 10903 genes, 0.1% | 1 |
| actin filament polymerization | 9 out of 9357 genes, 0.1% | 13 out of 10903 genes, 0.1% | 1 |
| protein polymerization | 9 out of 9357 genes, 0.1% | 13 out of 10903 genes, 0.1% | 1 |
| steroid metabolic process | 35 out of 9357 genes, 0.4% | 46 out of 10903 genes, 0.4% | 1 |
| cell killing | 3 out of 9357 genes, 0.0% | 5 out of 10903 genes, 0.0% | 1 |
| L-fucose biosynthetic process | 3 out of 9357 genes, 0.0% | 5 out of 10903 genes, 0.0% | 1 |
| glycosaminoglycan biosynthetic process | 3 out of 9357 genes, 0.0% | 5 out of 10903 genes, 0.0% | 1 |
| striated muscle contraction | 3 out of 9357 genes, 0.0% | 5 out of 10903 genes, 0.0% | 1 |
| nuclear envelope organization | 3 out of 9357 genes, 0.0% | 5 out of 10903 genes, 0.0% | 1 |
| monocarboxylic acid transport | 3 out of 9357 genes, 0.0% | 5 out of 10903 genes, 0.0% | 1 |
| glycosaminoglycan metabolic process | 3 out of 9357 genes, 0.0% | 5 out of 10903 genes, 0.0% | 1 |
| fucose biosynthetic process | 3 out of 9357 genes, 0.0% | 5 out of 10903 genes, 0.0% | 1 |
| L-fucose metabolic process | 3 out of 9357 genes, 0.0% | 5 out of 10903 genes, 0.0% | 1 |
| epithelial cell proliferation | 3 out of 9357 genes, 0.0% | 5 out of 10903 genes, 0.0% | 1 |
| regulation of anatomical structure size | 121 out of 9357 genes, 1.3% | 151 out of 10903 genes, 1.4% | 1 |
| alcohol biosynthetic process | 8 out of 9357 genes, 0.1% | 12 out of 10903 genes, 0.1% | 1 |
| negative regulation of cytoskeleton organization | 8 out of 9357 genes, 0.1% | 12 out of 10903 genes, 0.1% | 1 |
| blood circulation | 11 out of 9357 genes, 0.1% | 16 out of 10903 genes, 0.1% | 1 |
| negative regulation of cell adhesion | 5 out of 9357 genes, 0.1% | 8 out of 10903 genes, 0.1% | 1 |
| primary sex determination | 5 out of 9357 genes, 0.1% | 8 out of 10903 genes, 0.1% | 1 |
| primary sex determination, germ-line | 5 out of 9357 genes, 0.1% | 8 out of 10903 genes, 0.1% | 1 |
| regulation of actin filament depolymerization | 5 out of 9357 genes, 0.1% | 8 out of 10903 genes, 0.1% | 1 |
| negative regulation of protein complex disassembly | 5 out of 9357 genes, 0.1% | 8 out of 10903 genes, 0.1% | 1 |
| nucleocytoplasmic transport | 33 out of 9357 genes, 0.4% | 44 out of 10903 genes, 0.4% | 1 |
| transcription, DNA-dependent | 333 out of 9357 genes, 3.6% | 405 out of 10903 genes, 3.7% | 1 |
| RNA biosynthetic process | 333 out of 9357 genes, 3.6% | 405 out of 10903 genes, 3.7% | 1 |
| isoprenoid metabolic process | 21 out of 9357 genes, 0.2% | 29 out of 10903 genes, 0.3% | 1 |
| anterior/posterior pattern formation | 24 out of 9357 genes, 0.3% | 33 out of 10903 genes, 0.3% | 1 |
| response to corticosteroid stimulus | 7 out of 9357 genes, 0.1% | 11 out of 10903 genes, 0.1% | 1 |
| positive regulation of cell size | 7 out of 9357 genes, 0.1% | 11 out of 10903 genes, 0.1% | 1 |
| monosaccharide biosynthetic process | 7 out of 9357 genes, 0.1% | 11 out of 10903 genes, 0.1% | 1 |
| nuclear import | 7 out of 9357 genes, 0.1% | 11 out of 10903 genes, 0.1% | 1 |
| response to oxygen radical | 2 out of 9357 genes, 0.0% | 4 out of 10903 genes, 0.0% | 1 |
| Notch signaling pathway | 2 out of 9357 genes, 0.0% | 4 out of 10903 genes, 0.0% | 1 |
| regulation of phosphatase activity | 2 out of 9357 genes, 0.0% | 4 out of 10903 genes, 0.0% | 1 |
| regulation of gliogenesis | 2 out of 9357 genes, 0.0% | 4 out of 10903 genes, 0.0% | 1 |
| snRNA metabolic process | 2 out of 9357 genes, 0.0% | 4 out of 10903 genes, 0.0% | 1 |
| larval behavior | 2 out of 9357 genes, 0.0% | 4 out of 10903 genes, 0.0% | 1 |
| regulation of chromosome organization | 2 out of 9357 genes, 0.0% | 4 out of 10903 genes, 0.0% | 1 |
| regulation of dephosphorylation | 2 out of 9357 genes, 0.0% | 4 out of 10903 genes, 0.0% | 1 |
| protein tetramerization | 2 out of 9357 genes, 0.0% | 4 out of 10903 genes, 0.0% | 1 |
| 'de novo' protein folding | 4 out of 9357 genes, 0.0% | 7 out of 10903 genes, 0.1% | 1 |
| somatic sex determination | 4 out of 9357 genes, 0.0% | 7 out of 10903 genes, 0.1% | 1 |
| hexose biosynthetic process | 4 out of 9357 genes, 0.0% | 7 out of 10903 genes, 0.1% | 1 |
| negative regulation of actin filament depolymerization | 4 out of 9357 genes, 0.0% | 7 out of 10903 genes, 0.1% | 1 |
| response to cytokine stimulus | 9 out of 9357 genes, 0.1% | 14 out of 10903 genes, 0.1% | 1 |
| protein import into nucleus | 6 out of 9357 genes, 0.1% | 10 out of 10903 genes, 0.1% | 1 |
| oxygen and reactive oxygen species metabolic process | 6 out of 9357 genes, 0.1% | 10 out of 10903 genes, 0.1% | 1 |
| positive regulation of cell growth | 6 out of 9357 genes, 0.1% | 10 out of 10903 genes, 0.1% | 1 |
| protein localization to nucleus | 6 out of 9357 genes, 0.1% | 10 out of 10903 genes, 0.1% | 1 |
| intermediate filament-based process | 6 out of 9357 genes, 0.1% | 10 out of 10903 genes, 0.1% | 1 |
| intermediate filament cytoskeleton organization | 6 out of 9357 genes, 0.1% | 10 out of 10903 genes, 0.1% | 1 |
| male mating behavior | 11 out of 9357 genes, 0.1% | 17 out of 10903 genes, 0.2% | 1 |
| positive regulation of growth | 14 out of 9357 genes, 0.1% | 21 out of 10903 genes, 0.2% | 1 |
| response to growth factor stimulus | 3 out of 9357 genes, 0.0% | 6 out of 10903 genes, 0.1% | 1 |
| negative regulation of growth | 4 out of 9357 genes, 0.0% | 8 out of 10903 genes, 0.1% | 1 |
| organelle assembly | 4 out of 9357 genes, 0.0% | 8 out of 10903 genes, 0.1% | 1 |
| 'de novo' posttranslational protein folding | 2 out of 9357 genes, 0.0% | 5 out of 10903 genes, 0.0% | 1 |
| response to wounding | 12 out of 9357 genes, 0.1% | 20 out of 10903 genes, 0.2% | 1 |

| Gene Ontology term | Genes annotated to the term |
| --- | --- |
| multicellular organismal process | Unigene43390\_Sample\_011046840, Unigene29380\_Sample\_011046840, Unigene9443\_Sample\_011046840, Unigene43221\_Sample\_011046840, Unigene38541\_Sample\_011046840, Unigene37021\_Sample\_011046840, Unigene34041\_Sample\_011046840, Unigene34674\_Sample\_011046840, Unigene7110\_Sample\_011046840, Unigene23426\_Sample\_011046840, Unigene31281\_Sample\_011046840, Unigene20846\_Sample\_011046840, Unigene23713\_Sample\_011046840, Unigene27378\_Sample\_011046840, Unigene41823\_Sample\_011046840, Unigene25728\_Sample\_011046840, Unigene42856\_Sample\_011046840, Unigene21224\_Sample\_011046840, Unigene5625\_Sample\_011046840, Unigene30311\_Sample\_011046840, Unigene1376\_Sample\_011046840, Unigene7455\_Sample\_011046840, Unigene42372\_Sample\_011046840, Unigene25276\_Sample\_011046840, Unigene41366\_Sample\_011046840, Unigene42870\_Sample\_011046840, Unigene33388\_Sample\_011046840, Unigene26709\_Sample\_011046840, Unigene36928\_Sample\_011046840, Unigene24531\_Sample\_011046840, Unigene7453\_Sample\_011046840, Unigene3171\_Sample\_011046840, Unigene29813\_Sample\_011046840, Unigene13947\_Sample\_011046840, Unigene40446\_Sample\_011046840, Unigene43136\_Sample\_011046840, Unigene28087\_Sample\_011046840, Unigene32526\_Sample\_011046840, Unigene2135\_Sample\_011046840, Unigene13736\_Sample\_011046840, Unigene40261\_Sample\_011046840, Unigene42772\_Sample\_011046840, Unigene29635\_Sample\_011046840, Unigene12614\_Sample\_011046840, Unigene38611\_Sample\_011046840, Unigene43070\_Sample\_011046840, Unigene37375\_Sample\_011046840, Unigene40497\_Sample\_011046840, Unigene39891\_Sample\_011046840, Unigene24030\_Sample\_011046840, Unigene36909\_Sample\_011046840, Unigene41739\_Sample\_011046840, Unigene19291\_Sample\_011046840, Unigene31238\_Sample\_011046840, Unigene2743\_Sample\_011046840, Unigene2196\_Sample\_011046840, Unigene25680\_Sample\_011046840, Unigene7529\_Sample\_011046840, Unigene43466\_Sample\_011046840, Unigene39330\_Sample\_011046840, Unigene19368\_Sample\_011046840, Unigene1555\_Sample\_011046840, Unigene35531\_Sample\_011046840, Unigene4570\_Sample\_011046840, Unigene43118\_Sample\_011046840, Unigene38179\_Sample\_011046840, Unigene3849\_Sample\_011046840, Unigene38463\_Sample\_011046840, Unigene30678\_Sample\_011046840, Unigene7923\_Sample\_011046840, Unigene26558\_Sample\_011046840, Unigene7086\_Sample\_011046840, Unigene19342\_Sample\_011046840, Unigene36764\_Sample\_011046840, Unigene14179\_Sample\_011046840, Unigene40371\_Sample\_011046840, Unigene14077\_Sample\_011046840, Unigene38177\_Sample\_011046840, Unigene40938\_Sample\_011046840, Unigene33318\_Sample\_011046840, Unigene42480\_Sample\_011046840, Unigene2100\_Sample\_011046840, Unigene43061\_Sample\_011046840, Unigene29483\_Sample\_011046840, Unigene14124\_Sample\_011046840, Unigene29090\_Sample\_011046840, Unigene43496\_Sample\_011046840, Unigene7965\_Sample\_011046840, Unigene14797\_Sample\_011046840, Unigene19928\_Sample\_011046840, Unigene39758\_Sample\_011046840, Unigene11588\_Sample\_011046840, Unigene5804\_Sample\_011046840, Unigene43189\_Sample\_011046840, Unigene4511\_Sample\_011046840, Unigene20735\_Sample\_011046840, Unigene41617\_Sample\_011046840, Unigene41107\_Sample\_011046840, Unigene32562\_Sample\_011046840, Unigene10585\_Sample\_011046840, Unigene1756\_Sample\_011046840, Unigene7468\_Sample\_011046840, Unigene24254\_Sample\_011046840, Unigene39350\_Sample\_011046840, Unigene18753\_Sample\_011046840, Unigene30471\_Sample\_011046840, Unigene12001\_Sample\_011046840, Unigene3867\_Sample\_011046840, Unigene38598\_Sample\_011046840, Unigene9780\_Sample\_011046840, Unigene31067\_Sample\_011046840, Unigene11967\_Sample\_011046840, Unigene34024\_Sample\_011046840, Unigene42477\_Sample\_011046840, Unigene5389\_Sample\_011046840, Unigene6949\_Sample\_011046840, Unigene8231\_Sample\_011046840, Unigene24135\_Sample\_011046840, Unigene41915\_Sample\_011046840, Unigene42141\_Sample\_011046840, Unigene38592\_Sample\_011046840, Unigene2602\_Sample\_011046840, Unigene22269\_Sample\_011046840, Unigene22041\_Sample\_011046840, Unigene4830\_Sample\_011046840, Unigene39563\_Sample\_011046840, Unigene3322\_Sample\_011046840, Unigene43077\_Sample\_011046840, Unigene3469\_Sample\_011046840, Unigene39208\_Sample\_011046840, Unigene12913\_Sample\_011046840, Unigene41470\_Sample\_011046840, Unigene36987\_Sample\_011046840, Unigene37233\_Sample\_011046840, Unigene9565\_Sample\_011046840, Unigene19915\_Sample\_011046840, Unigene39197\_Sample\_011046840, Unigene31274\_Sample\_011046840, Unigene12101\_Sample\_011046840, Unigene4094\_Sample\_011046840, Unigene36578\_Sample\_011046840, Unigene7622\_Sample\_011046840, Unigene22810\_Sample\_011046840, Unigene14350\_Sample\_011046840, Unigene31913\_Sample\_011046840, Unigene16161\_Sample\_011046840, Unigene16645\_Sample\_011046840, Unigene6665\_Sample\_011046840, Unigene37582\_Sample\_011046840, Unigene23457\_Sample\_011046840, Unigene4155\_Sample\_011046840, Unigene41939\_Sample\_011046840, Unigene30615\_Sample\_011046840, Unigene27300\_Sample\_011046840, Unigene42728\_Sample\_011046840, Unigene35451\_Sample\_011046840, Unigene880\_Sample\_011046840, Unigene37979\_Sample\_011046840, Unigene4846\_Sample\_011046840, Unigene40406\_Sample\_011046840, Unigene28674\_Sample\_011046840, Unigene5888\_Sample\_011046840, Unigene23913\_Sample\_011046840, Unigene18001\_Sample\_011046840, Unigene24734\_Sample\_011046840, Unigene34664\_Sample\_011046840, Unigene38371\_Sample\_011046840, Unigene31308\_Sample\_011046840, Unigene40659\_Sample\_011046840, Unigene38092\_Sample\_011046840, Unigene33106\_Sample\_011046840, Unigene41412\_Sample\_011046840, Unigene42971\_Sample\_011046840, Unigene39780\_Sample\_011046840, Unigene34460\_Sample\_011046840, Unigene41357\_Sample\_011046840, Unigene27019\_Sample\_011046840, Unigene40374\_Sample\_011046840, Unigene12699\_Sample\_011046840, Unigene6042\_Sample\_011046840, Unigene32733\_Sample\_011046840, Unigene27788\_Sample\_011046840, Unigene41048\_Sample\_011046840, Unigene37902\_Sample\_011046840, Unigene19757\_Sample\_011046840, Unigene41135\_Sample\_011046840, Unigene22924\_Sample\_011046840, Unigene38847\_Sample\_011046840, Unigene26711\_Sample\_011046840, Unigene3415\_Sample\_011046840, Unigene43530\_Sample\_011046840, Unigene32179\_Sample\_011046840, Unigene30184\_Sample\_011046840, Unigene19898\_Sample\_011046840, Unigene33983\_Sample\_011046840, Unigene41425\_Sample\_011046840, Unigene6796\_Sample\_011046840, Unigene35734\_Sample\_011046840, Unigene38904\_Sample\_011046840, Unigene976\_Sample\_011046840, Unigene24807\_Sample\_011046840, Unigene43546\_Sample\_011046840, Unigene37502\_Sample\_011046840, Unigene7969\_Sample\_011046840, Unigene36619\_Sample\_011046840, Unigene36414\_Sample\_011046840, Unigene28220\_Sample\_011046840, Unigene20904\_Sample\_011046840, Unigene24826\_Sample\_011046840, Unigene30474\_Sample\_011046840, Unigene13988\_Sample\_011046840, Unigene43429\_Sample\_011046840, Unigene31656\_Sample\_011046840, Unigene4738\_Sample\_011046840, Unigene41759\_Sample\_011046840, Unigene43455\_Sample\_011046840, Unigene37905\_Sample\_011046840, Unigene22950\_Sample\_011046840, Unigene865\_Sample\_011046840, Unigene5831\_Sample\_011046840, Unigene37128\_Sample\_011046840, Unigene42374\_Sample\_011046840, Unigene23958\_Sample\_011046840, Unigene4341\_Sample\_011046840, Unigene12163\_Sample\_011046840, Unigene10385\_Sample\_011046840, Unigene40471\_Sample\_011046840, Unigene37950\_Sample\_011046840, Unigene40141\_Sample\_011046840, Unigene10711\_Sample\_011046840, Unigene41912\_Sample\_011046840, Unigene40045\_Sample\_011046840, Unigene38173\_Sample\_011046840, Unigene41161\_Sample\_011046840, Unigene20905\_Sample\_011046840, Unigene39012\_Sample\_011046840, Unigene41209\_Sample\_011046840, Unigene35917\_Sample\_011046840, Unigene36731\_Sample\_011046840, Unigene24252\_Sample\_011046840, Unigene18003\_Sample\_011046840, Unigene40914\_Sample\_011046840, Unigene11361\_Sample\_011046840, Unigene29839\_Sample\_011046840, Unigene6936\_Sample\_011046840, Unigene43099\_Sample\_011046840, Unigene42076\_Sample\_011046840, Unigene4447\_Sample\_011046840, Unigene35902\_Sample\_011046840, Unigene40217\_Sample\_011046840, Unigene32734\_Sample\_011046840, Unigene8078\_Sample\_011046840, Unigene2459\_Sample\_011046840, Unigene36366\_Sample\_011046840, Unigene43140\_Sample\_011046840, Unigene35200\_Sample\_011046840, Unigene29902\_Sample\_011046840, Unigene37304\_Sample\_011046840, Unigene43379\_Sample\_011046840, Unigene39770\_Sample\_011046840, Unigene34297\_Sample\_011046840, Unigene13262\_Sample\_011046840, Unigene40216\_Sample\_011046840, Unigene42859\_Sample\_011046840, Unigene37153\_Sample\_011046840, Unigene40011\_Sample\_011046840, Unigene20079\_Sample\_011046840, Unigene32197\_Sample\_011046840, Unigene5441\_Sample\_011046840, Unigene35688\_Sample\_011046840, Unigene38455\_Sample\_011046840, Unigene39096\_Sample\_011046840, Unigene15724\_Sample\_011046840, Unigene17123\_Sample\_011046840, Unigene29981\_Sample\_011046840, Unigene2780\_Sample\_011046840, Unigene42547\_Sample\_011046840, Unigene18600\_Sample\_011046840, Unigene10749\_Sample\_011046840, Unigene36679\_Sample\_011046840, Unigene14498\_Sample\_011046840, Unigene38931\_Sample\_011046840, Unigene9599\_Sample\_011046840, Unigene37043\_Sample\_011046840, Unigene42025\_Sample\_011046840, Unigene6354\_Sample\_011046840, Unigene35379\_Sample\_011046840, Unigene42004\_Sample\_011046840, Unigene42388\_Sample\_011046840, Unigene21548\_Sample\_011046840, Unigene37714\_Sample\_011046840, Unigene37214\_Sample\_011046840, Unigene34291\_Sample\_011046840, Unigene38118\_Sample\_011046840, Unigene35063\_Sample\_011046840, Unigene37292\_Sample\_011046840, Unigene3914\_Sample\_011046840, Unigene5310\_Sample\_011046840, Unigene38690\_Sample\_011046840, Unigene1574\_Sample\_011046840, Unigene8077\_Sample\_011046840, Unigene19307\_Sample\_011046840, Unigene35790\_Sample\_011046840, Unigene2492\_Sample\_011046840, Unigene15667\_Sample\_011046840, Unigene3551\_Sample\_011046840, Unigene35596\_Sample\_011046840, Unigene38663\_Sample\_011046840, Unigene4668\_Sample\_011046840, Unigene25317\_Sample\_011046840, Unigene33665\_Sample\_011046840, Unigene15244\_Sample\_011046840, Unigene31160\_Sample\_011046840, Unigene37870\_Sample\_011046840, Unigene28429\_Sample\_011046840, Unigene10107\_Sample\_011046840, Unigene543\_Sample\_011046840, Unigene31855\_Sample\_011046840, Unigene22373\_Sample\_011046840, Unigene41643\_Sample\_011046840, Unigene43632\_Sample\_011046840, Unigene7337\_Sample\_011046840, Unigene29021\_Sample\_011046840, Unigene31693\_Sample\_011046840, Unigene41631\_Sample\_011046840, Unigene3206\_Sample\_011046840, Unigene8713\_Sample\_011046840, Unigene14813\_Sample\_011046840, Unigene39687\_Sample\_011046840, Unigene8163\_Sample\_011046840, Unigene7097\_Sample\_011046840, Unigene37909\_Sample\_011046840, Unigene10222\_Sample\_011046840, Unigene10962\_Sample\_011046840, Unigene1884\_Sample\_011046840, Unigene43635\_Sample\_011046840, Unigene43362\_Sample\_011046840, Unigene43234\_Sample\_011046840, Unigene33207\_Sample\_011046840, Unigene35370\_Sample\_011046840, Unigene15115\_Sample\_011046840, Unigene384\_Sample\_011046840, Unigene35463\_Sample\_011046840, Unigene34258\_Sample\_011046840, Unigene22051\_Sample\_011046840, Unigene19732\_Sample\_011046840, Unigene22643\_Sample\_011046840, Unigene39854\_Sample\_011046840, Unigene22177\_Sample\_011046840, Unigene41826\_Sample\_011046840, Unigene33927\_Sample\_011046840, Unigene41831\_Sample\_011046840, Unigene40044\_Sample\_011046840, Unigene43417\_Sample\_011046840, Unigene41777\_Sample\_011046840, Unigene1731\_Sample\_011046840, Unigene21308\_Sample\_011046840, Unigene37462\_Sample\_011046840, Unigene26270\_Sample\_011046840, Unigene7521\_Sample\_011046840, Unigene4403\_Sample\_011046840, Unigene14265\_Sample\_011046840, Unigene27239\_Sample\_011046840, Unigene40574\_Sample\_011046840, Unigene26954\_Sample\_011046840, Unigene34833\_Sample\_011046840, Unigene38797\_Sample\_011046840, Unigene7395\_Sample\_011046840, Unigene41734\_Sample\_011046840, Unigene39405\_Sample\_011046840, Unigene42070\_Sample\_011046840, Unigene12915\_Sample\_011046840, Unigene5383\_Sample\_011046840, Unigene25182\_Sample\_011046840, Unigene7966\_Sample\_011046840, Unigene41967\_Sample\_011046840, Unigene6753\_Sample\_011046840, Unigene14139\_Sample\_011046840, Unigene8006\_Sample\_011046840, Unigene30041\_Sample\_011046840, Unigene2810\_Sample\_011046840, Unigene33019\_Sample\_011046840, Unigene2829\_Sample\_011046840, Unigene35732\_Sample\_011046840, Unigene23348\_Sample\_011046840, Unigene36852\_Sample\_011046840, Unigene33029\_Sample\_011046840, Unigene40308\_Sample\_011046840, Unigene23095\_Sample\_011046840, Unigene40648\_Sample\_011046840, Unigene42157\_Sample\_011046840, Unigene1561\_Sample\_011046840, Unigene35586\_Sample\_011046840, Unigene30384\_Sample\_011046840, Unigene16873\_Sample\_011046840, Unigene25597\_Sample\_011046840, Unigene36685\_Sample\_011046840, Unigene41711\_Sample\_011046840, Unigene32674\_Sample\_011046840, Unigene15148\_Sample\_011046840, Unigene12978\_Sample\_011046840, Unigene43598\_Sample\_011046840, Unigene27478\_Sample\_011046840, Unigene40678\_Sample\_011046840, Unigene4032\_Sample\_011046840, Unigene43082\_Sample\_011046840, Unigene42706\_Sample\_011046840, Unigene5259\_Sample\_011046840, Unigene19620\_Sample\_011046840, Unigene29328\_Sample\_011046840, Unigene1096\_Sample\_011046840, Unigene22777\_Sample\_011046840, Unigene33197\_Sample\_011046840, Unigene41341\_Sample\_011046840, Unigene7925\_Sample\_011046840, Unigene32304\_Sample\_011046840, Unigene8203\_Sample\_011046840, Unigene38680\_Sample\_011046840, Unigene32944\_Sample\_011046840, Unigene17693\_Sample\_011046840, Unigene38269\_Sample\_011046840, Unigene38501\_Sample\_011046840, Unigene39808\_Sample\_011046840, Unigene36467\_Sample\_011046840, Unigene24359\_Sample\_011046840, Unigene37727\_Sample\_011046840, Unigene37223\_Sample\_011046840, Unigene42189\_Sample\_011046840, Unigene39264\_Sample\_011046840, Unigene31926\_Sample\_011046840, Unigene37939\_Sample\_011046840, Unigene41302\_Sample\_011046840, Unigene41399\_Sample\_011046840, Unigene43181\_Sample\_011046840, Unigene40917\_Sample\_011046840, Unigene37761\_Sample\_011046840, Unigene28594\_Sample\_011046840, Unigene26480\_Sample\_011046840, Unigene1005\_Sample\_011046840, Unigene12818\_Sample\_011046840, Unigene7262\_Sample\_011046840, Unigene5464\_Sample\_011046840, Unigene5834\_Sample\_011046840, Unigene42276\_Sample\_011046840, Unigene28965\_Sample\_011046840, Unigene39713\_Sample\_011046840, Unigene28350\_Sample\_011046840, Unigene40068\_Sample\_011046840, Unigene38031\_Sample\_011046840, Unigene35632\_Sample\_011046840, Unigene37162\_Sample\_011046840, Unigene2808\_Sample\_011046840, Unigene43629\_Sample\_011046840, Unigene1771\_Sample\_011046840, Unigene34416\_Sample\_011046840, Unigene972\_Sample\_011046840, Unigene41191\_Sample\_011046840, Unigene8555\_Sample\_011046840, Unigene38774\_Sample\_011046840, Unigene39583\_Sample\_011046840, Unigene36715\_Sample\_011046840, Unigene33028\_Sample\_011046840, Unigene35532\_Sample\_011046840, Unigene43406\_Sample\_011046840, Unigene32382\_Sample\_011046840, Unigene38071\_Sample\_011046840, Unigene33115\_Sample\_011046840, Unigene3631\_Sample\_011046840, Unigene13007\_Sample\_011046840, Unigene41037\_Sample\_011046840, Unigene14072\_Sample\_011046840, Unigene42450\_Sample\_011046840, Unigene42672\_Sample\_011046840, Unigene35981\_Sample\_011046840, Unigene28899\_Sample\_011046840, Unigene38573\_Sample\_011046840, Unigene38416\_Sample\_011046840, Unigene19411\_Sample\_011046840, Unigene39949\_Sample\_011046840, Unigene37483\_Sample\_011046840, Unigene29197\_Sample\_011046840, Unigene41593\_Sample\_011046840, Unigene40009\_Sample\_011046840, Unigene37785\_Sample\_011046840, Unigene38523\_Sample\_011046840, Unigene11650\_Sample\_011046840, Unigene34241\_Sample\_011046840, Unigene37310\_Sample\_011046840, Unigene15637\_Sample\_011046840, Unigene26624\_Sample\_011046840, Unigene4180\_Sample\_011046840, Unigene7506\_Sample\_011046840, Unigene29269\_Sample\_011046840, Unigene17267\_Sample\_011046840, Unigene31672\_Sample\_011046840, Unigene33597\_Sample\_011046840, Unigene14882\_Sample\_011046840, Unigene37042\_Sample\_011046840, Unigene22818\_Sample\_011046840, Unigene43470\_Sample\_011046840, Unigene37367\_Sample\_011046840, Unigene24938\_Sample\_011046840, Unigene5665\_Sample\_011046840, Unigene43338\_Sample\_011046840, Unigene36655\_Sample\_011046840, Unigene42187\_Sample\_011046840, Unigene36838\_Sample\_011046840, Unigene37629\_Sample\_011046840, Unigene3539\_Sample\_011046840, Unigene40868\_Sample\_011046840, Unigene40283\_Sample\_011046840, Unigene2011\_Sample\_011046840, Unigene42264\_Sample\_011046840, Unigene7319\_Sample\_011046840, Unigene41977\_Sample\_011046840, Unigene41508\_Sample\_011046840, Unigene42892\_Sample\_011046840, Unigene37361\_Sample\_011046840, Unigene34533\_Sample\_011046840, Unigene32227\_Sample\_011046840, Unigene24453\_Sample\_011046840, Unigene38059\_Sample\_011046840, Unigene36176\_Sample\_011046840, Unigene18282\_Sample\_011046840, Unigene25428\_Sample\_011046840, Unigene28006\_Sample\_011046840, Unigene15047\_Sample\_011046840, Unigene22023\_Sample\_011046840, Unigene38864\_Sample\_011046840, Unigene5683\_Sample\_011046840, Unigene8223\_Sample\_011046840, Unigene43124\_Sample\_011046840, Unigene7174\_Sample\_011046840, Unigene31928\_Sample\_011046840, Unigene25793\_Sample\_011046840, Unigene15213\_Sample\_011046840, Unigene40473\_Sample\_011046840, Unigene42745\_Sample\_011046840, Unigene42744\_Sample\_011046840, Unigene40953\_Sample\_011046840, Unigene39049\_Sample\_011046840, Unigene39918\_Sample\_011046840, Unigene33136\_Sample\_011046840, Unigene41691\_Sample\_011046840, Unigene42082\_Sample\_011046840, Unigene21539\_Sample\_011046840, Unigene42237\_Sample\_011046840, Unigene42865\_Sample\_011046840, Unigene33969\_Sample\_011046840, Unigene30879\_Sample\_011046840, Unigene39491\_Sample\_011046840, Unigene22159\_Sample\_011046840, Unigene32280\_Sample\_011046840, Unigene24277\_Sample\_011046840, Unigene31467\_Sample\_011046840, Unigene31422\_Sample\_011046840, Unigene33275\_Sample\_011046840, Unigene15062\_Sample\_011046840, Unigene6641\_Sample\_011046840, Unigene20790\_Sample\_011046840, Unigene4464\_Sample\_011046840, Unigene39237\_Sample\_011046840, Unigene41136\_Sample\_011046840, Unigene7402\_Sample\_011046840, Unigene5375\_Sample\_011046840, Unigene23730\_Sample\_011046840, Unigene43387\_Sample\_011046840, Unigene42669\_Sample\_011046840, Unigene33442\_Sample\_011046840, Unigene37570\_Sample\_011046840, Unigene13808\_Sample\_011046840, Unigene20104\_Sample\_011046840, Unigene36268\_Sample\_011046840, Unigene29568\_Sample\_011046840, Unigene31458\_Sample\_011046840, Unigene36089\_Sample\_011046840, Unigene35699\_Sample\_011046840, Unigene27865\_Sample\_011046840, Unigene36246\_Sample\_011046840, Unigene27711\_Sample\_011046840, Unigene38064\_Sample\_011046840, Unigene1619\_Sample\_011046840, Unigene10148\_Sample\_011046840, Unigene489\_Sample\_011046840, Unigene7785\_Sample\_011046840, Unigene40020\_Sample\_011046840, Unigene41040\_Sample\_011046840, Unigene7496\_Sample\_011046840, Unigene41188\_Sample\_011046840, Unigene23432\_Sample\_011046840, Unigene40537\_Sample\_011046840, Unigene24280\_Sample\_011046840, Unigene3897\_Sample\_011046840, Unigene38025\_Sample\_011046840, Unigene22635\_Sample\_011046840, Unigene7818\_Sample\_011046840, Unigene7964\_Sample\_011046840, Unigene29103\_Sample\_011046840, Unigene35359\_Sample\_011046840, Unigene32594\_Sample\_011046840, Unigene28786\_Sample\_011046840, Unigene33403\_Sample\_011046840, Unigene38351\_Sample\_011046840, Unigene41457\_Sample\_011046840, Unigene40060\_Sample\_011046840, Unigene41269\_Sample\_011046840, Unigene30281\_Sample\_011046840, Unigene33999\_Sample\_011046840, Unigene16829\_Sample\_011046840, Unigene37581\_Sample\_011046840, Unigene36659\_Sample\_011046840, Unigene43491\_Sample\_011046840, Unigene41424\_Sample\_011046840, Unigene22380\_Sample\_011046840, Unigene36819\_Sample\_011046840, Unigene1162\_Sample\_011046840, Unigene16633\_Sample\_011046840, Unigene29267\_Sample\_011046840, Unigene4456\_Sample\_011046840, Unigene7030\_Sample\_011046840, Unigene19649\_Sample\_011046840, Unigene8304\_Sample\_011046840, Unigene16385\_Sample\_011046840, Unigene40727\_Sample\_011046840, Unigene25620\_Sample\_011046840, Unigene20304\_Sample\_011046840, Unigene31514\_Sample\_011046840, Unigene13223\_Sample\_011046840, Unigene29294\_Sample\_011046840, Unigene3713\_Sample\_011046840, Unigene40548\_Sample\_011046840, Unigene36291\_Sample\_011046840, Unigene37160\_Sample\_011046840, Unigene41420\_Sample\_011046840, Unigene22778\_Sample\_011046840, Unigene22019\_Sample\_011046840, Unigene33393\_Sample\_011046840, Unigene34733\_Sample\_011046840, Unigene36800\_Sample\_011046840, Unigene42484\_Sample\_011046840, Unigene24023\_Sample\_011046840, Unigene40993\_Sample\_011046840, Unigene39775\_Sample\_011046840, Unigene32802\_Sample\_011046840, Unigene31108\_Sample\_011046840, Unigene32394\_Sample\_011046840, Unigene11460\_Sample\_011046840, Unigene7287\_Sample\_011046840, Unigene33196\_Sample\_011046840, Unigene11148\_Sample\_011046840, Unigene7398\_Sample\_011046840, Unigene42989\_Sample\_011046840, Unigene17368\_Sample\_011046840, Unigene4785\_Sample\_011046840, Unigene7682\_Sample\_011046840, Unigene40448\_Sample\_011046840, Unigene40607\_Sample\_011046840, Unigene34286\_Sample\_011046840, Unigene7018\_Sample\_011046840, Unigene36846\_Sample\_011046840, Unigene11904\_Sample\_011046840, Unigene41068\_Sample\_011046840, Unigene35591\_Sample\_011046840, Unigene37353\_Sample\_011046840, Unigene42460\_Sample\_011046840, Unigene32803\_Sample\_011046840, Unigene38500\_Sample\_011046840, Unigene36526\_Sample\_011046840, Unigene35874\_Sample\_011046840, Unigene23363\_Sample\_011046840, Unigene42677\_Sample\_011046840, Unigene36889\_Sample\_011046840, Unigene6245\_Sample\_011046840, Unigene31742\_Sample\_011046840, Unigene4095\_Sample\_011046840, Unigene35367\_Sample\_011046840, Unigene24378\_Sample\_011046840, Unigene42259\_Sample\_011046840, Unigene40052\_Sample\_011046840, Unigene34927\_Sample\_011046840, Unigene40368\_Sample\_011046840, Unigene26424\_Sample\_011046840, Unigene25992\_Sample\_011046840, Unigene8084\_Sample\_011046840, Unigene22186\_Sample\_011046840, Unigene8865\_Sample\_011046840, Unigene40400\_Sample\_011046840, Unigene41519\_Sample\_011046840, Unigene30365\_Sample\_011046840, Unigene2304\_Sample\_011046840, Unigene12728\_Sample\_011046840, Unigene7589\_Sample\_011046840, Unigene3029\_Sample\_011046840, Unigene473\_Sample\_011046840, Unigene40539\_Sample\_011046840, Unigene12616\_Sample\_011046840, Unigene29390\_Sample\_011046840, Unigene30972\_Sample\_011046840, Unigene38136\_Sample\_011046840, Unigene31408\_Sample\_011046840, Unigene19090\_Sample\_011046840, Unigene24751\_Sample\_011046840, Unigene4573\_Sample\_011046840, Unigene27023\_Sample\_011046840, Unigene17674\_Sample\_011046840, Unigene4307\_Sample\_011046840, Unigene39015\_Sample\_011046840, Unigene33692\_Sample\_011046840, Unigene42407\_Sample\_011046840, Unigene38116\_Sample\_011046840, Unigene4271\_Sample\_011046840, Unigene443\_Sample\_011046840, Unigene19281\_Sample\_011046840, Unigene3664\_Sample\_011046840, Unigene41989\_Sample\_011046840, Unigene40697\_Sample\_011046840, Unigene4074\_Sample\_011046840, Unigene412\_Sample\_011046840, Unigene18283\_Sample\_011046840, Unigene32001\_Sample\_011046840, Unigene39420\_Sample\_011046840, Unigene24457\_Sample\_011046840, Unigene3218\_Sample\_011046840, Unigene38370\_Sample\_011046840, Unigene37533\_Sample\_011046840, Unigene30813\_Sample\_011046840, Unigene22254\_Sample\_011046840, Unigene13304\_Sample\_011046840, Unigene37821\_Sample\_011046840, Unigene27313\_Sample\_011046840, Unigene7411\_Sample\_011046840, Unigene42997\_Sample\_011046840, Unigene22277\_Sample\_011046840, Unigene23433\_Sample\_011046840, Unigene9880\_Sample\_011046840, Unigene38844\_Sample\_011046840, Unigene35646\_Sample\_011046840, Unigene39107\_Sample\_011046840, Unigene18019\_Sample\_011046840, Unigene1399\_Sample\_011046840, Unigene20126\_Sample\_011046840, Unigene9230\_Sample\_011046840, Unigene6378\_Sample\_011046840, Unigene18553\_Sample\_011046840, Unigene33580\_Sample\_011046840, Unigene6293\_Sample\_011046840, Unigene6863\_Sample\_011046840, Unigene42560\_Sample\_011046840, Unigene39297\_Sample\_011046840, Unigene24719\_Sample\_011046840, Unigene5343\_Sample\_011046840, Unigene30604\_Sample\_011046840, Unigene33409\_Sample\_011046840, Unigene15093\_Sample\_011046840, Unigene41588\_Sample\_011046840, Unigene21293\_Sample\_011046840, Unigene40410\_Sample\_011046840, Unigene41301\_Sample\_011046840, Unigene27425\_Sample\_011046840, Unigene31906\_Sample\_011046840, Unigene32088\_Sample\_011046840, Unigene11853\_Sample\_011046840, Unigene41565\_Sample\_011046840, Unigene19655\_Sample\_011046840, Unigene35402\_Sample\_011046840, Unigene43392\_Sample\_011046840, Unigene37230\_Sample\_011046840, Unigene35476\_Sample\_011046840, Unigene42286\_Sample\_011046840, Unigene15696\_Sample\_011046840, Unigene42267\_Sample\_011046840, Unigene38049\_Sample\_011046840, Unigene42406\_Sample\_011046840, Unigene41976\_Sample\_011046840, Unigene35259\_Sample\_011046840, Unigene6735\_Sample\_011046840, Unigene7990\_Sample\_011046840, Unigene62\_Sample\_011046840, Unigene42227\_Sample\_011046840, Unigene42573\_Sample\_011046840, Unigene25063\_Sample\_011046840, Unigene29393\_Sample\_011046840, Unigene29512\_Sample\_011046840, Unigene26969\_Sample\_011046840, Unigene5227\_Sample\_011046840, Unigene4596\_Sample\_011046840, Unigene31789\_Sample\_011046840, Unigene10037\_Sample\_011046840, Unigene30222\_Sample\_011046840, Unigene22661\_Sample\_011046840, Unigene32134\_Sample\_011046840, Unigene1791\_Sample\_011046840, Unigene37027\_Sample\_011046840, Unigene38182\_Sample\_011046840, Unigene32118\_Sample\_011046840, Unigene10532\_Sample\_011046840, Unigene37721\_Sample\_011046840, Unigene37612\_Sample\_011046840, Unigene43062\_Sample\_011046840, Unigene29301\_Sample\_011046840, Unigene37050\_Sample\_011046840, Unigene40565\_Sample\_011046840, Unigene2908\_Sample\_011046840, Unigene28467\_Sample\_011046840, Unigene42873\_Sample\_011046840, Unigene39321\_Sample\_011046840, Unigene27180\_Sample\_011046840, Unigene30019\_Sample\_011046840, Unigene12611\_Sample\_011046840, Unigene36400\_Sample\_011046840, Unigene43084\_Sample\_011046840, Unigene24149\_Sample\_011046840, Unigene28660\_Sample\_011046840, Unigene1814\_Sample\_011046840, Unigene7778\_Sample\_011046840, Unigene40949\_Sample\_011046840, Unigene18949\_Sample\_011046840, Unigene10182\_Sample\_011046840, Unigene37431\_Sample\_011046840, Unigene37331\_Sample\_011046840, Unigene6202\_Sample\_011046840, Unigene34464\_Sample\_011046840, Unigene1404\_Sample\_011046840, Unigene17327\_Sample\_011046840, Unigene42789\_Sample\_011046840, Unigene8029\_Sample\_011046840, Unigene30636\_Sample\_011046840, Unigene34114\_Sample\_011046840, Unigene7617\_Sample\_011046840, Unigene13980\_Sample\_011046840, Unigene21171\_Sample\_011046840, Unigene36038\_Sample\_011046840, Unigene42473\_Sample\_011046840, Unigene35027\_Sample\_011046840, Unigene37705\_Sample\_011046840, Unigene31658\_Sample\_011046840, Unigene7891\_Sample\_011046840, Unigene38609\_Sample\_011046840, Unigene42360\_Sample\_011046840, Unigene36706\_Sample\_011046840, Unigene36498\_Sample\_011046840, Unigene30691\_Sample\_011046840, Unigene8185\_Sample\_011046840, Unigene22085\_Sample\_011046840, Unigene43414\_Sample\_011046840, Unigene43637\_Sample\_011046840, Unigene7503\_Sample\_011046840, Unigene8056\_Sample\_011046840, Unigene16274\_Sample\_011046840, Unigene27887\_Sample\_011046840, Unigene38630\_Sample\_011046840, Unigene43465\_Sample\_011046840, Unigene39865\_Sample\_011046840, Unigene40273\_Sample\_011046840, Unigene28061\_Sample\_011046840, Unigene32375\_Sample\_011046840, Unigene16035\_Sample\_011046840, Unigene25217\_Sample\_011046840, Unigene39694\_Sample\_011046840, Unigene39556\_Sample\_011046840, Unigene40411\_Sample\_011046840, Unigene42046\_Sample\_011046840, Unigene6593\_Sample\_011046840, Unigene13276\_Sample\_011046840, Unigene7743\_Sample\_011046840, Unigene41658\_Sample\_011046840, Unigene35238\_Sample\_011046840, Unigene7160\_Sample\_011046840, Unigene32984\_Sample\_011046840, Unigene25956\_Sample\_011046840, Unigene38377\_Sample\_011046840, Unigene40244\_Sample\_011046840, Unigene40056\_Sample\_011046840, Unigene37436\_Sample\_011046840, Unigene33866\_Sample\_011046840, Unigene20911\_Sample\_011046840, Unigene43607\_Sample\_011046840, Unigene21072\_Sample\_011046840, Unigene35505\_Sample\_011046840, Unigene22459\_Sample\_011046840, Unigene41033\_Sample\_011046840, Unigene16778\_Sample\_011046840, Unigene11831\_Sample\_011046840, Unigene41358\_Sample\_011046840, Unigene34658\_Sample\_011046840, Unigene2822\_Sample\_011046840, Unigene38044\_Sample\_011046840, Unigene30105\_Sample\_011046840, Unigene36704\_Sample\_011046840, Unigene30185\_Sample\_011046840, Unigene1808\_Sample\_011046840, Unigene7314\_Sample\_011046840, Unigene511\_Sample\_011046840, Unigene7868\_Sample\_011046840, Unigene37041\_Sample\_011046840, Unigene38326\_Sample\_011046840, Unigene42114\_Sample\_011046840, Unigene30572\_Sample\_011046840, Unigene41653\_Sample\_011046840, Unigene30946\_Sample\_011046840, Unigene37774\_Sample\_011046840, Unigene12994\_Sample\_011046840, Unigene38224\_Sample\_011046840, Unigene37728\_Sample\_011046840, Unigene40269\_Sample\_011046840, Unigene25413\_Sample\_011046840, Unigene35002\_Sample\_011046840, Unigene42943\_Sample\_011046840, Unigene6148\_Sample\_011046840, Unigene3655\_Sample\_011046840, Unigene27654\_Sample\_011046840, Unigene17719\_Sample\_011046840, Unigene17210\_Sample\_011046840, Unigene31398\_Sample\_011046840, Unigene34920\_Sample\_011046840, Unigene31798\_Sample\_011046840, Unigene25025\_Sample\_011046840, Unigene38963\_Sample\_011046840, Unigene39800\_Sample\_011046840, Unigene23890\_Sample\_011046840, Unigene34237\_Sample\_011046840, Unigene35348\_Sample\_011046840, Unigene42543\_Sample\_011046840, Unigene38297\_Sample\_011046840, Unigene1924\_Sample\_011046840, Unigene13452\_Sample\_011046840, Unigene37049\_Sample\_011046840, Unigene35038\_Sample\_011046840, Unigene22824\_Sample\_011046840, Unigene43147\_Sample\_011046840, Unigene37187\_Sample\_011046840, Unigene34490\_Sample\_011046840, Unigene38039\_Sample\_011046840, Unigene25041\_Sample\_011046840, Unigene6727\_Sample\_011046840, Unigene40577\_Sample\_011046840, Unigene40391\_Sample\_011046840, Unigene20326\_Sample\_011046840, Unigene6439\_Sample\_011046840, Unigene1089\_Sample\_011046840, Unigene39732\_Sample\_011046840, Unigene7134\_Sample\_011046840, Unigene1959\_Sample\_011046840, Unigene5214\_Sample\_011046840, Unigene8142\_Sample\_011046840, Unigene20821\_Sample\_011046840, Unigene28884\_Sample\_011046840, Unigene41766\_Sample\_011046840, Unigene4689\_Sample\_011046840, Unigene36458\_Sample\_011046840, Unigene32758\_Sample\_011046840, Unigene28668\_Sample\_011046840, Unigene10614\_Sample\_011046840, Unigene28414\_Sample\_011046840, Unigene14465\_Sample\_011046840, Unigene33488\_Sample\_011046840, Unigene37126\_Sample\_011046840, Unigene38046\_Sample\_011046840, Unigene39882\_Sample\_011046840, Unigene21804\_Sample\_011046840, Unigene7689\_Sample\_011046840, Unigene41260\_Sample\_011046840, Unigene29198\_Sample\_011046840, Unigene3986\_Sample\_011046840, Unigene17861\_Sample\_011046840, Unigene30757\_Sample\_011046840, Unigene23327\_Sample\_011046840, Unigene2733\_Sample\_011046840, Unigene15193\_Sample\_011046840, Unigene12679\_Sample\_011046840, Unigene35891\_Sample\_011046840, Unigene37574\_Sample\_011046840, Unigene39837\_Sample\_011046840, Unigene36621\_Sample\_011046840, Unigene33823\_Sample\_011046840, Unigene38877\_Sample\_011046840, Unigene18559\_Sample\_011046840, Unigene27392\_Sample\_011046840, Unigene38001\_Sample\_011046840, Unigene1995\_Sample\_011046840, Unigene14292\_Sample\_011046840, Unigene27724\_Sample\_011046840, Unigene38261\_Sample\_011046840, Unigene3959\_Sample\_011046840, Unigene40049\_Sample\_011046840, Unigene24925\_Sample\_011046840, Unigene36220\_Sample\_011046840, Unigene43559\_Sample\_011046840, Unigene23126\_Sample\_011046840, Unigene30862\_Sample\_011046840, Unigene42369\_Sample\_011046840, Unigene15803\_Sample\_011046840, Unigene38115\_Sample\_011046840, Unigene42326\_Sample\_011046840, Unigene42090\_Sample\_011046840, Unigene40127\_Sample\_011046840, Unigene42430\_Sample\_011046840, Unigene42349\_Sample\_011046840, Unigene14612\_Sample\_011046840, Unigene40302\_Sample\_011046840, Unigene26987\_Sample\_011046840, Unigene30393\_Sample\_011046840, Unigene30445\_Sample\_011046840, Unigene41699\_Sample\_011046840, Unigene28636\_Sample\_011046840, Unigene40806\_Sample\_011046840, Unigene28901\_Sample\_011046840, Unigene2013\_Sample\_011046840, Unigene1019\_Sample\_011046840, Unigene14012\_Sample\_011046840, Unigene38316\_Sample\_011046840, Unigene20681\_Sample\_011046840, Unigene39011\_Sample\_011046840, Unigene15872\_Sample\_011046840, Unigene2234\_Sample\_011046840, Unigene36951\_Sample\_011046840, Unigene35314\_Sample\_011046840, Unigene36090\_Sample\_011046840, Unigene38933\_Sample\_011046840, Unigene39978\_Sample\_011046840, Unigene7044\_Sample\_011046840, Unigene38021\_Sample\_011046840, Unigene34684\_Sample\_011046840, Unigene28200\_Sample\_011046840, Unigene35485\_Sample\_011046840, Unigene7976\_Sample\_011046840, Unigene40828\_Sample\_011046840, Unigene37710\_Sample\_011046840, Unigene36437\_Sample\_011046840, Unigene23988\_Sample\_011046840, Unigene38996\_Sample\_011046840, Unigene38381\_Sample\_011046840, Unigene30586\_Sample\_011046840, Unigene30195\_Sample\_011046840, Unigene35082\_Sample\_011046840, Unigene19602\_Sample\_011046840, Unigene31891\_Sample\_011046840, Unigene26821\_Sample\_011046840, Unigene2850\_Sample\_011046840, Unigene41566\_Sample\_011046840, Unigene24067\_Sample\_011046840, Unigene25979\_Sample\_011046840, Unigene34302\_Sample\_011046840, Unigene16882\_Sample\_011046840, Unigene42047\_Sample\_011046840, Unigene42154\_Sample\_011046840, Unigene24706\_Sample\_011046840, Unigene3537\_Sample\_011046840, Unigene37765\_Sample\_011046840, Unigene28163\_Sample\_011046840, Unigene18649\_Sample\_011046840, Unigene19522\_Sample\_011046840, Unigene28960\_Sample\_011046840, Unigene8038\_Sample\_011046840, Unigene15446\_Sample\_011046840, Unigene38984\_Sample\_011046840, Unigene34521\_Sample\_011046840, Unigene42209\_Sample\_011046840, Unigene24921\_Sample\_011046840, Unigene39540\_Sample\_011046840, Unigene42588\_Sample\_011046840, Unigene39958\_Sample\_011046840, Unigene30535\_Sample\_011046840, Unigene30803\_Sample\_011046840, Unigene4993\_Sample\_011046840, Unigene43303\_Sample\_011046840, Unigene16996\_Sample\_011046840, Unigene25969\_Sample\_011046840, Unigene42770\_Sample\_011046840, Unigene12125\_Sample\_011046840, Unigene12084\_Sample\_011046840, Unigene4662\_Sample\_011046840, Unigene37036\_Sample\_011046840, Unigene38030\_Sample\_011046840, Unigene9931\_Sample\_011046840, Unigene41960\_Sample\_011046840, Unigene37077\_Sample\_011046840, Unigene28352\_Sample\_011046840, Unigene31322\_Sample\_011046840, Unigene24172\_Sample\_011046840, Unigene23304\_Sample\_011046840, Unigene10449\_Sample\_011046840, Unigene30505\_Sample\_011046840, Unigene33255\_Sample\_011046840, Unigene30144\_Sample\_011046840, Unigene39133\_Sample\_011046840, Unigene35081\_Sample\_011046840, Unigene35015\_Sample\_011046840, Unigene43592\_Sample\_011046840, Unigene35395\_Sample\_011046840, Unigene4981\_Sample\_011046840, Unigene36682\_Sample\_011046840, Unigene38215\_Sample\_011046840, Unigene13399\_Sample\_011046840, Unigene25439\_Sample\_011046840, Unigene27539\_Sample\_011046840, Unigene22996\_Sample\_011046840, Unigene43209\_Sample\_011046840, Unigene35138\_Sample\_011046840, Unigene6606\_Sample\_011046840, Unigene29658\_Sample\_011046840, Unigene32316\_Sample\_011046840, Unigene34165\_Sample\_011046840, Unigene37994\_Sample\_011046840, Unigene7096\_Sample\_011046840, Unigene36487\_Sample\_011046840, Unigene21470\_Sample\_011046840, Unigene35564\_Sample\_011046840, Unigene35642\_Sample\_011046840, Unigene37719\_Sample\_011046840, Unigene23269\_Sample\_011046840, Unigene21991\_Sample\_011046840, Unigene31822\_Sample\_011046840, Unigene41582\_Sample\_011046840, Unigene19624\_Sample\_011046840, Unigene7290\_Sample\_011046840, Unigene25873\_Sample\_011046840, Unigene39990\_Sample\_011046840, Unigene6792\_Sample\_011046840, Unigene31936\_Sample\_011046840, Unigene20729\_Sample\_011046840, Unigene33260\_Sample\_011046840, Unigene1181\_Sample\_011046840, Unigene7806\_Sample\_011046840, Unigene27768\_Sample\_011046840, Unigene40465\_Sample\_011046840, Unigene21963\_Sample\_011046840, Unigene42900\_Sample\_011046840, Unigene29843\_Sample\_011046840, Unigene43407\_Sample\_011046840, Unigene43153\_Sample\_011046840, Unigene35284\_Sample\_011046840, Unigene34500\_Sample\_011046840, Unigene39533\_Sample\_011046840, Unigene6498\_Sample\_011046840, Unigene6287\_Sample\_011046840, Unigene34227\_Sample\_011046840, Unigene39179\_Sample\_011046840, Unigene914\_Sample\_011046840, Unigene3934\_Sample\_011046840, Unigene41947\_Sample\_011046840, Unigene5308\_Sample\_011046840, Unigene42127\_Sample\_011046840, Unigene41531\_Sample\_011046840, Unigene2538\_Sample\_011046840, Unigene36075\_Sample\_011046840, Unigene26432\_Sample\_011046840, Unigene33939\_Sample\_011046840, Unigene30107\_Sample\_011046840, Unigene5894\_Sample\_011046840, Unigene40210\_Sample\_011046840, Unigene18336\_Sample\_011046840, Unigene42336\_Sample\_011046840, Unigene39894\_Sample\_011046840, Unigene4280\_Sample\_011046840, Unigene23686\_Sample\_011046840, Unigene36509\_Sample\_011046840, Unigene29810\_Sample\_011046840, Unigene27260\_Sample\_011046840, Unigene25330\_Sample\_011046840, Unigene2241\_Sample\_011046840, Unigene41034\_Sample\_011046840, Unigene43347\_Sample\_011046840, Unigene6551\_Sample\_011046840, Unigene36772\_Sample\_011046840, Unigene28654\_Sample\_011046840, Unigene15939\_Sample\_011046840, Unigene42861\_Sample\_011046840, Unigene31756\_Sample\_011046840, Unigene35797\_Sample\_011046840, Unigene43617\_Sample\_011046840, Unigene9798\_Sample\_011046840, Unigene31676\_Sample\_011046840, Unigene27895\_Sample\_011046840, Unigene17153\_Sample\_011046840, Unigene43095\_Sample\_011046840, Unigene34652\_Sample\_011046840, Unigene23749\_Sample\_011046840, Unigene25826\_Sample\_011046840, Unigene4860\_Sample\_011046840, Unigene4960\_Sample\_011046840, Unigene18602\_Sample\_011046840, Unigene13277\_Sample\_011046840, Unigene34059\_Sample\_011046840, Unigene25669\_Sample\_011046840, Unigene5098\_Sample\_011046840, Unigene36485\_Sample\_011046840, Unigene17954\_Sample\_011046840, Unigene43590\_Sample\_011046840, Unigene10383\_Sample\_011046840, Unigene30059\_Sample\_011046840, Unigene2326\_Sample\_011046840, Unigene10035\_Sample\_011046840, Unigene7559\_Sample\_011046840, Unigene12797\_Sample\_011046840, Unigene24250\_Sample\_011046840, Unigene35263\_Sample\_011046840, Unigene8904\_Sample\_011046840, Unigene36617\_Sample\_011046840, Unigene27774\_Sample\_011046840, Unigene41481\_Sample\_011046840, Unigene39423\_Sample\_011046840, Unigene21868\_Sample\_011046840, Unigene31384\_Sample\_011046840, Unigene28192\_Sample\_011046840, Unigene25488\_Sample\_011046840, Unigene39829\_Sample\_011046840, Unigene29917\_Sample\_011046840, Unigene14473\_Sample\_011046840, Unigene34896\_Sample\_011046840, Unigene41164\_Sample\_011046840, Unigene42697\_Sample\_011046840, Unigene39804\_Sample\_011046840, Unigene42639\_Sample\_011046840, Unigene27502\_Sample\_011046840, Unigene42008\_Sample\_011046840, Unigene19427\_Sample\_011046840, Unigene43556\_Sample\_011046840, Unigene43384\_Sample\_011046840, Unigene41850\_Sample\_011046840, Unigene6860\_Sample\_011046840, Unigene19854\_Sample\_011046840, Unigene41225\_Sample\_011046840, Unigene33377\_Sample\_011046840, Unigene8061\_Sample\_011046840, Unigene32168\_Sample\_011046840, Unigene39631\_Sample\_011046840, Unigene23663\_Sample\_011046840, Unigene18729\_Sample\_011046840, Unigene26268\_Sample\_011046840, Unigene24420\_Sample\_011046840, Unigene41708\_Sample\_011046840, Unigene19018\_Sample\_011046840, Unigene38034\_Sample\_011046840, Unigene19750\_Sample\_011046840, Unigene3788\_Sample\_011046840, Unigene29777\_Sample\_011046840, Unigene41324\_Sample\_011046840, Unigene38082\_Sample\_011046840, Unigene25534\_Sample\_011046840, Unigene33797\_Sample\_011046840, Unigene3510\_Sample\_011046840, Unigene4324\_Sample\_011046840, Unigene6500\_Sample\_011046840, Unigene37845\_Sample\_011046840, Unigene14750\_Sample\_011046840, Unigene2505\_Sample\_011046840, Unigene40503\_Sample\_011046840, Unigene42626\_Sample\_011046840, Unigene21366\_Sample\_011046840, Unigene8162\_Sample\_011046840, Unigene41966\_Sample\_011046840, Unigene38572\_Sample\_011046840, Unigene4686\_Sample\_011046840, Unigene41224\_Sample\_011046840, Unigene41261\_Sample\_011046840, Unigene37984\_Sample\_011046840, Unigene31107\_Sample\_011046840, Unigene23892\_Sample\_011046840, Unigene36726\_Sample\_011046840, Unigene4101\_Sample\_011046840, Unigene29660\_Sample\_011046840, Unigene12353\_Sample\_011046840, Unigene41784\_Sample\_011046840, Unigene32027\_Sample\_011046840, Unigene41113\_Sample\_011046840, Unigene28846\_Sample\_011046840, Unigene40765\_Sample\_011046840, Unigene43481\_Sample\_011046840, Unigene34294\_Sample\_011046840, Unigene38383\_Sample\_011046840, Unigene9305\_Sample\_011046840, Unigene33984\_Sample\_011046840, Unigene32791\_Sample\_011046840, Unigene40130\_Sample\_011046840, Unigene25300\_Sample\_011046840, Unigene39835\_Sample\_011046840, Unigene36905\_Sample\_011046840, Unigene39559\_Sample\_011046840, Unigene40053\_Sample\_011046840, Unigene33967\_Sample\_011046840, Unigene21661\_Sample\_011046840, Unigene33954\_Sample\_011046840, Unigene3573\_Sample\_011046840, Unigene31558\_Sample\_011046840, Unigene8141\_Sample\_011046840, Unigene29794\_Sample\_011046840, Unigene208\_Sample\_011046840, Unigene43439\_Sample\_011046840, Unigene2087\_Sample\_011046840, Unigene43258\_Sample\_011046840, Unigene42488\_Sample\_011046840, Unigene2124\_Sample\_011046840, Unigene15316\_Sample\_011046840, Unigene40278\_Sample\_011046840, Unigene38689\_Sample\_011046840, Unigene24898\_Sample\_011046840, Unigene38428\_Sample\_011046840, Unigene5906\_Sample\_011046840, Unigene38679\_Sample\_011046840, Unigene4898\_Sample\_011046840, Unigene19593\_Sample\_011046840, Unigene3935\_Sample\_011046840, Unigene38495\_Sample\_011046840, Unigene27282\_Sample\_011046840, Unigene42729\_Sample\_011046840, Unigene41810\_Sample\_011046840, Unigene30989\_Sample\_011046840, Unigene31513\_Sample\_011046840, Unigene15836\_Sample\_011046840, Unigene28852\_Sample\_011046840, Unigene41870\_Sample\_011046840, Unigene3177\_Sample\_011046840, Unigene14093\_Sample\_011046840, Unigene3950\_Sample\_011046840, Unigene42809\_Sample\_011046840, Unigene16797\_Sample\_011046840, Unigene37338\_Sample\_011046840, Unigene38608\_Sample\_011046840, Unigene28124\_Sample\_011046840, Unigene2498\_Sample\_011046840, Unigene41882\_Sample\_011046840, Unigene694\_Sample\_011046840, Unigene40686\_Sample\_011046840, Unigene43337\_Sample\_011046840, Unigene29570\_Sample\_011046840, Unigene40454\_Sample\_011046840, Unigene7153\_Sample\_011046840, Unigene26719\_Sample\_011046840, Unigene24464\_Sample\_011046840, Unigene42930\_Sample\_011046840, Unigene1242\_Sample\_011046840, Unigene20065\_Sample\_011046840, Unigene4520\_Sample\_011046840, Unigene36374\_Sample\_011046840, Unigene37348\_Sample\_011046840, Unigene16037\_Sample\_011046840, Unigene2655\_Sample\_011046840, Unigene8069\_Sample\_011046840, Unigene259\_Sample\_011046840, Unigene41377\_Sample\_011046840, Unigene42352\_Sample\_011046840, Unigene42798\_Sample\_011046840, Unigene6824\_Sample\_011046840, Unigene27745\_Sample\_011046840, Unigene9889\_Sample\_011046840, Unigene38857\_Sample\_011046840, Unigene20264\_Sample\_011046840, Unigene42293\_Sample\_011046840, Unigene33267\_Sample\_011046840, Unigene23234\_Sample\_011046840, Unigene20485\_Sample\_011046840, Unigene38612\_Sample\_011046840, Unigene2740\_Sample\_011046840, Unigene41709\_Sample\_011046840, Unigene34975\_Sample\_011046840, Unigene24318\_Sample\_011046840, Unigene42665\_Sample\_011046840, Unigene32622\_Sample\_011046840, Unigene37488\_Sample\_011046840, Unigene41885\_Sample\_011046840, Unigene21784\_Sample\_011046840, Unigene35086\_Sample\_011046840, Unigene41973\_Sample\_011046840, Unigene35656\_Sample\_011046840, Unigene5006\_Sample\_011046840, Unigene31648\_Sample\_011046840, Unigene25551\_Sample\_011046840, Unigene36362\_Sample\_011046840, Unigene31407\_Sample\_011046840, Unigene40585\_Sample\_011046840, Unigene36042\_Sample\_011046840, Unigene42909\_Sample\_011046840, Unigene22119\_Sample\_011046840, Unigene29398\_Sample\_011046840, Unigene29394\_Sample\_011046840, Unigene22727\_Sample\_011046840, Unigene8569\_Sample\_011046840, Unigene38742\_Sample\_011046840, Unigene34380\_Sample\_011046840, Unigene34400\_Sample\_011046840, Unigene39701\_Sample\_011046840, Unigene34198\_Sample\_011046840, Unigene30356\_Sample\_011046840, Unigene40445\_Sample\_011046840, Unigene39577\_Sample\_011046840, Unigene20616\_Sample\_011046840, Unigene43001\_Sample\_011046840, Unigene41119\_Sample\_011046840, Unigene34584\_Sample\_011046840, Unigene37492\_Sample\_011046840, Unigene8114\_Sample\_011046840, Unigene7551\_Sample\_011046840, Unigene33831\_Sample\_011046840, Unigene29712\_Sample\_011046840, Unigene36231\_Sample\_011046840, Unigene15823\_Sample\_011046840, Unigene23089\_Sample\_011046840, Unigene4300\_Sample\_011046840, Unigene28019\_Sample\_011046840, Unigene14521\_Sample\_011046840, Unigene38147\_Sample\_011046840, Unigene40140\_Sample\_011046840, Unigene13251\_Sample\_011046840, Unigene30053\_Sample\_011046840, Unigene39184\_Sample\_011046840, Unigene23906\_Sample\_011046840, Unigene43148\_Sample\_011046840, Unigene15931\_Sample\_011046840, Unigene36603\_Sample\_011046840, Unigene38317\_Sample\_011046840, Unigene41928\_Sample\_011046840, Unigene34963\_Sample\_011046840, Unigene16017\_Sample\_011046840, Unigene24314\_Sample\_011046840, Unigene42249\_Sample\_011046840, Unigene42317\_Sample\_011046840, Unigene2237\_Sample\_011046840, Unigene22809\_Sample\_011046840, Unigene42628\_Sample\_011046840, Unigene42185\_Sample\_011046840, Unigene39818\_Sample\_011046840, Unigene670\_Sample\_011046840, Unigene33542\_Sample\_011046840, Unigene42614\_Sample\_011046840, Unigene41216\_Sample\_011046840, Unigene31437\_Sample\_011046840, Unigene7853\_Sample\_011046840, Unigene5003\_Sample\_011046840, Unigene42434\_Sample\_011046840, Unigene10050\_Sample\_011046840, Unigene31531\_Sample\_011046840, Unigene28275\_Sample\_011046840, Unigene8071\_Sample\_011046840, Unigene30429\_Sample\_011046840, Unigene26951\_Sample\_011046840, Unigene3379\_Sample\_011046840, Unigene5586\_Sample\_011046840, Unigene8762\_Sample\_011046840, Unigene37133\_Sample\_011046840, Unigene441\_Sample\_011046840, Unigene38540\_Sample\_011046840, Unigene41984\_Sample\_011046840, Unigene15651\_Sample\_011046840, Unigene39613\_Sample\_011046840, Unigene43355\_Sample\_011046840, Unigene4172\_Sample\_011046840, Unigene11875\_Sample\_011046840, Unigene42965\_Sample\_011046840, Unigene18027\_Sample\_011046840, Unigene24698\_Sample\_011046840, Unigene37789\_Sample\_011046840, Unigene10979\_Sample\_011046840, Unigene41638\_Sample\_011046840, Unigene33395\_Sample\_011046840, Unigene35279\_Sample\_011046840, Unigene29670\_Sample\_011046840, Unigene35252\_Sample\_011046840, Unigene27635\_Sample\_011046840, Unigene43052\_Sample\_011046840, Unigene31260\_Sample\_011046840, Unigene28451\_Sample\_011046840, Unigene36771\_Sample\_011046840, Unigene29095\_Sample\_011046840, Unigene38643\_Sample\_011046840, Unigene18934\_Sample\_011046840, Unigene31732\_Sample\_011046840, Unigene40067\_Sample\_011046840, Unigene43449\_Sample\_011046840, Unigene39748\_Sample\_011046840, Unigene10332\_Sample\_011046840, Unigene41478\_Sample\_011046840, Unigene40388\_Sample\_011046840, Unigene16\_Sample\_011046840, Unigene5425\_Sample\_011046840, Unigene4108\_Sample\_011046840, Unigene40703\_Sample\_011046840, Unigene34817\_Sample\_011046840, Unigene19931\_Sample\_011046840, Unigene7299\_Sample\_011046840, Unigene39355\_Sample\_011046840, Unigene828\_Sample\_011046840, Unigene36817\_Sample\_011046840, Unigene24581\_Sample\_011046840, Unigene40609\_Sample\_011046840, Unigene43500\_Sample\_011046840, Unigene30092\_Sample\_011046840, Unigene1830\_Sample\_011046840, Unigene31643\_Sample\_011046840, Unigene21957\_Sample\_011046840, Unigene42701\_Sample\_011046840, Unigene38677\_Sample\_011046840, Unigene33384\_Sample\_011046840, Unigene38254\_Sample\_011046840, Unigene19631\_Sample\_011046840, Unigene41501\_Sample\_011046840, Unigene40031\_Sample\_011046840, Unigene15184\_Sample\_011046840, Unigene42052\_Sample\_011046840, Unigene14995\_Sample\_011046840, Unigene22845\_Sample\_011046840, Unigene33524\_Sample\_011046840, Unigene33567\_Sample\_011046840, Unigene2132\_Sample\_011046840, Unigene6929\_Sample\_011046840, Unigene39550\_Sample\_011046840, Unigene25804\_Sample\_011046840, Unigene36638\_Sample\_011046840, Unigene40814\_Sample\_011046840, Unigene3802\_Sample\_011046840, Unigene17857\_Sample\_011046840, Unigene2002\_Sample\_011046840, Unigene7782\_Sample\_011046840, Unigene38801\_Sample\_011046840, Unigene37919\_Sample\_011046840, Unigene42999\_Sample\_011046840, Unigene25085\_Sample\_011046840, Unigene34752\_Sample\_011046840, Unigene31306\_Sample\_011046840, Unigene29808\_Sample\_011046840, Unigene29937\_Sample\_011046840, Unigene18930\_Sample\_011046840, Unigene30290\_Sample\_011046840, Unigene27185\_Sample\_011046840, Unigene5148\_Sample\_011046840, Unigene25467\_Sample\_011046840, Unigene32098\_Sample\_011046840, Unigene3694\_Sample\_011046840, Unigene971\_Sample\_011046840, Unigene40260\_Sample\_011046840, Unigene42780\_Sample\_011046840, Unigene37398\_Sample\_011046840, Unigene33188\_Sample\_011046840, Unigene37686\_Sample\_011046840, Unigene43331\_Sample\_011046840, Unigene34331\_Sample\_011046840, Unigene5962\_Sample\_011046840, Unigene3360\_Sample\_011046840, Unigene25604\_Sample\_011046840, Unigene26448\_Sample\_011046840, Unigene28616\_Sample\_011046840, Unigene33020\_Sample\_011046840, Unigene6626\_Sample\_011046840, Unigene6861\_Sample\_011046840, Unigene31181\_Sample\_011046840, Unigene6991\_Sample\_011046840, Unigene36824\_Sample\_011046840, Unigene38328\_Sample\_011046840, Unigene32274\_Sample\_011046840, Unigene23645\_Sample\_011046840, Unigene43358\_Sample\_011046840, Unigene5312\_Sample\_011046840, Unigene39608\_Sample\_011046840, Unigene43152\_Sample\_011046840, Unigene42294\_Sample\_011046840, Unigene38420\_Sample\_011046840, Unigene19985\_Sample\_011046840, Unigene38795\_Sample\_011046840, Unigene43068\_Sample\_011046840, Unigene34183\_Sample\_011046840, Unigene4100\_Sample\_011046840, Unigene21932\_Sample\_011046840, Unigene31024\_Sample\_011046840, Unigene38002\_Sample\_011046840, Unigene43502\_Sample\_011046840, Unigene42740\_Sample\_011046840, Unigene39542\_Sample\_011046840, Unigene14245\_Sample\_011046840, Unigene43519\_Sample\_011046840, Unigene38134\_Sample\_011046840, Unigene41243\_Sample\_011046840, Unigene43523\_Sample\_011046840, Unigene18148\_Sample\_011046840, Unigene39678\_Sample\_011046840, Unigene41829\_Sample\_011046840, Unigene42879\_Sample\_011046840, Unigene9708\_Sample\_011046840, Unigene20966\_Sample\_011046840, Unigene32377\_Sample\_011046840, Unigene6230\_Sample\_011046840, Unigene43410\_Sample\_011046840, Unigene19933\_Sample\_011046840, Unigene6873\_Sample\_011046840, Unigene25323\_Sample\_011046840, Unigene26710\_Sample\_011046840, Unigene17472\_Sample\_011046840, Unigene38940\_Sample\_011046840, Unigene38980\_Sample\_011046840, Unigene8658\_Sample\_011046840, Unigene26198\_Sample\_011046840, Unigene31655\_Sample\_011046840, Unigene1368\_Sample\_011046840, Unigene34694\_Sample\_011046840, Unigene34730\_Sample\_011046840, Unigene42041\_Sample\_011046840, Unigene29910\_Sample\_011046840, Unigene39019\_Sample\_011046840, Unigene37892\_Sample\_011046840, Unigene36547\_Sample\_011046840, Unigene37336\_Sample\_011046840, Unigene39699\_Sample\_011046840, Unigene8088\_Sample\_011046840, Unigene39956\_Sample\_011046840, Unigene34520\_Sample\_011046840, Unigene31022\_Sample\_011046840, Unigene20538\_Sample\_011046840, Unigene29967\_Sample\_011046840, Unigene37842\_Sample\_011046840, Unigene36320\_Sample\_011046840, Unigene1379\_Sample\_011046840, Unigene43023\_Sample\_011046840, Unigene41968\_Sample\_011046840, Unigene2872\_Sample\_011046840, Unigene38789\_Sample\_011046840, Unigene18925\_Sample\_011046840, Unigene41072\_Sample\_011046840, Unigene34218\_Sample\_011046840, Unigene34025\_Sample\_011046840, Unigene33046\_Sample\_011046840, Unigene8009\_Sample\_011046840, Unigene18706\_Sample\_011046840, Unigene27243\_Sample\_011046840, Unigene7553\_Sample\_011046840, Unigene38830\_Sample\_011046840, Unigene8164\_Sample\_011046840, Unigene9937\_Sample\_011046840, Unigene7606\_Sample\_011046840, Unigene37213\_Sample\_011046840, Unigene43291\_Sample\_011046840, Unigene36645\_Sample\_011046840, Unigene36780\_Sample\_011046840, Unigene17625\_Sample\_011046840, Unigene36232\_Sample\_011046840, Unigene19207\_Sample\_011046840, Unigene4855\_Sample\_011046840, Unigene12129\_Sample\_011046840, Unigene32514\_Sample\_011046840, Unigene13914\_Sample\_011046840, Unigene43373\_Sample\_011046840, Unigene38334\_Sample\_011046840, Unigene31613\_Sample\_011046840, Unigene29983\_Sample\_011046840, Unigene20131\_Sample\_011046840, Unigene9558\_Sample\_011046840, Unigene38589\_Sample\_011046840, Unigene29877\_Sample\_011046840, Unigene25457\_Sample\_011046840, Unigene40453\_Sample\_011046840, Unigene43016\_Sample\_011046840, Unigene34677\_Sample\_011046840, Unigene27032\_Sample\_011046840, Unigene40962\_Sample\_011046840, Unigene7061\_Sample\_011046840, Unigene32576\_Sample\_011046840, Unigene31726\_Sample\_011046840, Unigene4849\_Sample\_011046840, Unigene41940\_Sample\_011046840, Unigene3888\_Sample\_011046840, Unigene3456\_Sample\_011046840, Unigene41584\_Sample\_011046840, Unigene11026\_Sample\_011046840, Unigene40781\_Sample\_011046840, Unigene38452\_Sample\_011046840, Unigene41670\_Sample\_011046840, Unigene31523\_Sample\_011046840, Unigene27118\_Sample\_011046840, Unigene36992\_Sample\_011046840, Unigene32614\_Sample\_011046840, Unigene37136\_Sample\_011046840, Unigene18191\_Sample\_011046840, Unigene19398\_Sample\_011046840, Unigene752\_Sample\_011046840, Unigene11279\_Sample\_011046840, Unigene24549\_Sample\_011046840, Unigene12492\_Sample\_011046840, Unigene42337\_Sample\_011046840, Unigene17765\_Sample\_011046840, Unigene41656\_Sample\_011046840, Unigene31218\_Sample\_011046840, Unigene41299\_Sample\_011046840, Unigene43521\_Sample\_011046840, Unigene12416\_Sample\_011046840, Unigene1568\_Sample\_011046840, Unigene37872\_Sample\_011046840, Unigene42236\_Sample\_011046840, Unigene11146\_Sample\_011046840, Unigene30715\_Sample\_011046840, Unigene37236\_Sample\_011046840, Unigene23668\_Sample\_011046840, Unigene5390\_Sample\_011046840, Unigene40105\_Sample\_011046840, Unigene27924\_Sample\_011046840, Unigene8023\_Sample\_011046840, Unigene10706\_Sample\_011046840, Unigene43604\_Sample\_011046840, Unigene7412\_Sample\_011046840, Unigene29385\_Sample\_011046840, Unigene27403\_Sample\_011046840, Unigene8195\_Sample\_011046840, Unigene22945\_Sample\_011046840, Unigene8192\_Sample\_011046840, Unigene41237\_Sample\_011046840, Unigene37255\_Sample\_011046840, Unigene41862\_Sample\_011046840, Unigene7588\_Sample\_011046840, Unigene5090\_Sample\_011046840, Unigene14847\_Sample\_011046840, Unigene42510\_Sample\_011046840, Unigene27611\_Sample\_011046840, Unigene28755\_Sample\_011046840, Unigene27902\_Sample\_011046840, Unigene8229\_Sample\_011046840, Unigene35673\_Sample\_011046840, Unigene27833\_Sample\_011046840, Unigene22486\_Sample\_011046840, Unigene16716\_Sample\_011046840, Unigene36628\_Sample\_011046840, Unigene39912\_Sample\_011046840, Unigene15922\_Sample\_011046840, Unigene40286\_Sample\_011046840, Unigene36382\_Sample\_011046840, Unigene6634\_Sample\_011046840, Unigene41391\_Sample\_011046840, Unigene42364\_Sample\_011046840, Unigene2522\_Sample\_011046840, Unigene37337\_Sample\_011046840, Unigene31983\_Sample\_011046840, Unigene1495\_Sample\_011046840, Unigene10747\_Sample\_011046840, Unigene42881\_Sample\_011046840, Unigene33015\_Sample\_011046840, Unigene15903\_Sample\_011046840, Unigene8931\_Sample\_011046840, Unigene43514\_Sample\_011046840, Unigene32606\_Sample\_011046840, Unigene13197\_Sample\_011046840, Unigene15140\_Sample\_011046840, Unigene32866\_Sample\_011046840, Unigene14307\_Sample\_011046840, Unigene32403\_Sample\_011046840, Unigene5097\_Sample\_011046840, Unigene27742\_Sample\_011046840, Unigene32770\_Sample\_011046840, Unigene6328\_Sample\_011046840, Unigene16639\_Sample\_011046840, Unigene7340\_Sample\_011046840, Unigene2160\_Sample\_011046840, Unigene25527\_Sample\_011046840, Unigene2401\_Sample\_011046840, Unigene25874\_Sample\_011046840, Unigene32618\_Sample\_011046840, Unigene36770\_Sample\_011046840, Unigene38674\_Sample\_011046840, Unigene35486\_Sample\_011046840, Unigene39426\_Sample\_011046840, Unigene40615\_Sample\_011046840, Unigene37474\_Sample\_011046840, Unigene43374\_Sample\_011046840, Unigene17386\_Sample\_011046840, Unigene29537\_Sample\_011046840, Unigene25916\_Sample\_011046840, Unigene5544\_Sample\_011046840, Unigene8208\_Sample\_011046840, Unigene30688\_Sample\_011046840, Unigene27233\_Sample\_011046840, Unigene6209\_Sample\_011046840, Unigene19390\_Sample\_011046840, Unigene25298\_Sample\_011046840, Unigene868\_Sample\_011046840, Unigene43119\_Sample\_011046840, Unigene41750\_Sample\_011046840, Unigene8541\_Sample\_011046840, Unigene21046\_Sample\_011046840, Unigene4254\_Sample\_011046840, Unigene34589\_Sample\_011046840, Unigene38154\_Sample\_011046840, Unigene34777\_Sample\_011046840, Unigene19991\_Sample\_011046840, Unigene38135\_Sample\_011046840, Unigene42622\_Sample\_011046840, Unigene35573\_Sample\_011046840, Unigene39948\_Sample\_011046840, Unigene40247\_Sample\_011046840, Unigene5658\_Sample\_011046840, Unigene6608\_Sample\_011046840, Unigene38378\_Sample\_011046840, Unigene40722\_Sample\_011046840, Unigene35507\_Sample\_011046840, Unigene15614\_Sample\_011046840, Unigene43051\_Sample\_011046840, Unigene31151\_Sample\_011046840, Unigene4412\_Sample\_011046840, Unigene36855\_Sample\_011046840, Unigene6689\_Sample\_011046840, Unigene7896\_Sample\_011046840, Unigene2891\_Sample\_011046840, Unigene5037\_Sample\_011046840, Unigene20699\_Sample\_011046840, Unigene40807\_Sample\_011046840, Unigene37225\_Sample\_011046840, Unigene1879\_Sample\_011046840, Unigene18652\_Sample\_011046840, Unigene29707\_Sample\_011046840, Unigene24564\_Sample\_011046840, Unigene43638\_Sample\_011046840, Unigene31698\_Sample\_011046840, Unigene11516\_Sample\_011046840, Unigene31539\_Sample\_011046840, Unigene34208\_Sample\_011046840, Unigene14097\_Sample\_011046840, Unigene42844\_Sample\_011046840, Unigene15558\_Sample\_011046840, Unigene38279\_Sample\_011046840, Unigene28752\_Sample\_011046840, Unigene41461\_Sample\_011046840, Unigene25915\_Sample\_011046840, Unigene126\_Sample\_011046840, Unigene26572\_Sample\_011046840, Unigene38262\_Sample\_011046840, Unigene37510\_Sample\_011046840, Unigene42972\_Sample\_011046840, Unigene2284\_Sample\_011046840, Unigene19092\_Sample\_011046840, Unigene40218\_Sample\_011046840, Unigene36364\_Sample\_011046840, Unigene35053\_Sample\_011046840, Unigene25408\_Sample\_011046840, Unigene17585\_Sample\_011046840, Unigene40086\_Sample\_011046840, Unigene38805\_Sample\_011046840, Unigene623\_Sample\_011046840, Unigene43612\_Sample\_011046840, Unigene7708\_Sample\_011046840, Unigene41242\_Sample\_011046840, Unigene21134\_Sample\_011046840, Unigene43165\_Sample\_011046840, Unigene35858\_Sample\_011046840, Unigene19948\_Sample\_011046840, Unigene32262\_Sample\_011046840, Unigene13460\_Sample\_011046840, Unigene41436\_Sample\_011046840, Unigene8230\_Sample\_011046840, Unigene4965\_Sample\_011046840, Unigene39751\_Sample\_011046840, Unigene15076\_Sample\_011046840, Unigene8000\_Sample\_011046840, Unigene29125\_Sample\_011046840, Unigene1602\_Sample\_011046840, Unigene2640\_Sample\_011046840, Unigene37684\_Sample\_011046840, Unigene38450\_Sample\_011046840, Unigene5995\_Sample\_011046840, Unigene42250\_Sample\_011046840, Unigene33876\_Sample\_011046840, Unigene39916\_Sample\_011046840, Unigene39979\_Sample\_011046840, Unigene7602\_Sample\_011046840, Unigene35435\_Sample\_011046840, Unigene5662\_Sample\_011046840, Unigene5904\_Sample\_011046840, Unigene33011\_Sample\_011046840, Unigene7144\_Sample\_011046840, Unigene27926\_Sample\_011046840, Unigene40128\_Sample\_011046840, Unigene14238\_Sample\_011046840, Unigene25020\_Sample\_011046840, Unigene39566\_Sample\_011046840, Unigene23373\_Sample\_011046840, Unigene31397\_Sample\_011046840, Unigene7767\_Sample\_011046840, Unigene33527\_Sample\_011046840, Unigene14437\_Sample\_011046840, Unigene1195\_Sample\_011046840, Unigene30961\_Sample\_011046840, Unigene34615\_Sample\_011046840, Unigene4287\_Sample\_011046840, Unigene32859\_Sample\_011046840, Unigene34686\_Sample\_011046840, Unigene15626\_Sample\_011046840, Unigene39604\_Sample\_011046840, Unigene38023\_Sample\_011046840, Unigene42993\_Sample\_011046840, Unigene32100\_Sample\_011046840, Unigene26641\_Sample\_011046840, Unigene42776\_Sample\_011046840, Unigene42869\_Sample\_011046840, Unigene42159\_Sample\_011046840, Unigene41910\_Sample\_011046840, Unigene31703\_Sample\_011046840, Unigene18730\_Sample\_011046840, Unigene29476\_Sample\_011046840, Unigene14932\_Sample\_011046840, Unigene24702\_Sample\_011046840, Unigene7132\_Sample\_011046840, Unigene30892\_Sample\_011046840, Unigene313\_Sample\_011046840, Unigene7193\_Sample\_011046840, Unigene43100\_Sample\_011046840, Unigene39267\_Sample\_011046840, Unigene908\_Sample\_011046840, Unigene21853\_Sample\_011046840, Unigene6964\_Sample\_011046840, Unigene35945\_Sample\_011046840, Unigene32488\_Sample\_011046840, Unigene7199\_Sample\_011046840, Unigene41153\_Sample\_011046840, Unigene32455\_Sample\_011046840, Unigene6629\_Sample\_011046840, Unigene33356\_Sample\_011046840, Unigene8271\_Sample\_011046840, Unigene40710\_Sample\_011046840, Unigene35431\_Sample\_011046840, Unigene20979\_Sample\_011046840, Unigene42401\_Sample\_011046840, Unigene1603\_Sample\_011046840, Unigene36398\_Sample\_011046840, Unigene43161\_Sample\_011046840, Unigene6748\_Sample\_011046840, Unigene14734\_Sample\_011046840, Unigene29029\_Sample\_011046840, Unigene40625\_Sample\_011046840, Unigene40773\_Sample\_011046840, Unigene37185\_Sample\_011046840, Unigene41737\_Sample\_011046840, Unigene36145\_Sample\_011046840, Unigene31072\_Sample\_011046840, Unigene41318\_Sample\_011046840, Unigene32811\_Sample\_011046840, Unigene34499\_Sample\_011046840, Unigene23767\_Sample\_011046840, Unigene34252\_Sample\_011046840, Unigene42895\_Sample\_011046840, Unigene42810\_Sample\_011046840, Unigene15576\_Sample\_011046840, Unigene21176\_Sample\_011046840, Unigene42496\_Sample\_011046840, Unigene29804\_Sample\_011046840, Unigene39351\_Sample\_011046840, Unigene18500\_Sample\_011046840, Unigene40943\_Sample\_011046840, Unigene34982\_Sample\_011046840, Unigene22101\_Sample\_011046840, Unigene32903\_Sample\_011046840, Unigene36598\_Sample\_011046840, Unigene41683\_Sample\_011046840, Unigene39858\_Sample\_011046840, Unigene2784\_Sample\_011046840, Unigene4089\_Sample\_011046840, Unigene11899\_Sample\_011046840, Unigene40955\_Sample\_011046840, Unigene35615\_Sample\_011046840, Unigene5726\_Sample\_011046840, Unigene38504\_Sample\_011046840, Unigene40734\_Sample\_011046840, Unigene15902\_Sample\_011046840, Unigene38492\_Sample\_011046840, Unigene36848\_Sample\_011046840, Unigene29264\_Sample\_011046840, Unigene5914\_Sample\_011046840, Unigene38201\_Sample\_011046840, Unigene30026\_Sample\_011046840, Unigene40672\_Sample\_011046840, Unigene27237\_Sample\_011046840, Unigene41628\_Sample\_011046840, Unigene29497\_Sample\_011046840, Unigene40779\_Sample\_011046840, Unigene1709\_Sample\_011046840, Unigene6614\_Sample\_011046840, Unigene40194\_Sample\_011046840, Unigene14153\_Sample\_011046840, Unigene35528\_Sample\_011046840, Unigene24478\_Sample\_011046840, Unigene26590\_Sample\_011046840, Unigene7831\_Sample\_011046840, Unigene86\_Sample\_011046840, Unigene42036\_Sample\_011046840, Unigene36934\_Sample\_011046840, Unigene39812\_Sample\_011046840, Unigene33676\_Sample\_011046840, Unigene27998\_Sample\_011046840, Unigene30726\_Sample\_011046840, Unigene39159\_Sample\_011046840, Unigene41748\_Sample\_011046840, Unigene33574\_Sample\_011046840, Unigene6407\_Sample\_011046840, Unigene39955\_Sample\_011046840, Unigene34234\_Sample\_011046840, Unigene21071\_Sample\_011046840, Unigene42134\_Sample\_011046840, Unigene42502\_Sample\_011046840, Unigene40900\_Sample\_011046840, Unigene35736\_Sample\_011046840, Unigene41621\_Sample\_011046840, Unigene40074\_Sample\_011046840, Unigene7975\_Sample\_011046840, Unigene20573\_Sample\_011046840, Unigene40879\_Sample\_011046840, Unigene18097\_Sample\_011046840, Unigene34842\_Sample\_011046840, Unigene37066\_Sample\_011046840, Unigene23758\_Sample\_011046840, Unigene607\_Sample\_011046840, Unigene37822\_Sample\_011046840, Unigene40796\_Sample\_011046840, Unigene4663\_Sample\_011046840, Unigene23772\_Sample\_011046840, Unigene41635\_Sample\_011046840, Unigene36643\_Sample\_011046840, Unigene33315\_Sample\_011046840, Unigene34514\_Sample\_011046840, Unigene43122\_Sample\_011046840, Unigene3799\_Sample\_011046840, Unigene41124\_Sample\_011046840, Unigene33208\_Sample\_011046840, Unigene4270\_Sample\_011046840, Unigene17212\_Sample\_011046840, Unigene32176\_Sample\_011046840, Unigene26308\_Sample\_011046840, Unigene29560\_Sample\_011046840, Unigene17807\_Sample\_011046840, Unigene25972\_Sample\_011046840, Unigene33944\_Sample\_011046840, Unigene5654\_Sample\_011046840, Unigene8445\_Sample\_011046840, Unigene34084\_Sample\_011046840, Unigene41900\_Sample\_011046840, Unigene35629\_Sample\_011046840, Unigene29916\_Sample\_011046840, Unigene22613\_Sample\_011046840, Unigene34070\_Sample\_011046840, Unigene16857\_Sample\_011046840, Unigene13928\_Sample\_011046840, Unigene41932\_Sample\_011046840, Unigene42342\_Sample\_011046840, Unigene32833\_Sample\_011046840, Unigene3749\_Sample\_011046840, Unigene7889\_Sample\_011046840, Unigene39589\_Sample\_011046840, Unigene40592\_Sample\_011046840, Unigene41221\_Sample\_011046840, Unigene38983\_Sample\_011046840, Unigene42907\_Sample\_011046840, Unigene32289\_Sample\_011046840, Unigene42811\_Sample\_011046840, Unigene38741\_Sample\_011046840, Unigene4787\_Sample\_011046840, Unigene5532\_Sample\_011046840, Unigene42193\_Sample\_011046840, Unigene18086\_Sample\_011046840, Unigene43104\_Sample\_011046840, Unigene20\_Sample\_011046840, Unigene26949\_Sample\_011046840, Unigene37613\_Sample\_011046840, Unigene26548\_Sample\_011046840, Unigene32909\_Sample\_011046840, Unigene41800\_Sample\_011046840, Unigene40746\_Sample\_011046840, Unigene18659\_Sample\_011046840, Unigene38631\_Sample\_011046840, Unigene42521\_Sample\_011046840, Unigene25227\_Sample\_011046840, Unigene29802\_Sample\_011046840, Unigene13794\_Sample\_011046840, Unigene42061\_Sample\_011046840, Unigene20491\_Sample\_011046840, Unigene37444\_Sample\_011046840, Unigene21006\_Sample\_011046840, Unigene40877\_Sample\_011046840, Unigene36623\_Sample\_011046840, Unigene29515\_Sample\_011046840, Unigene5146\_Sample\_011046840, Unigene35780\_Sample\_011046840, Unigene31301\_Sample\_011046840, Unigene42838\_Sample\_011046840, Unigene5263\_Sample\_011046840, Unigene11913\_Sample\_011046840, Unigene36035\_Sample\_011046840, Unigene38971\_Sample\_011046840, Unigene29410\_Sample\_011046840, Unigene26130\_Sample\_011046840, Unigene38834\_Sample\_011046840, Unigene41577\_Sample\_011046840, Unigene12220\_Sample\_011046840, Unigene43087\_Sample\_011046840, Unigene34176\_Sample\_011046840, Unigene7137\_Sample\_011046840, Unigene1345\_Sample\_011046840, Unigene4825\_Sample\_011046840, Unigene38733\_Sample\_011046840, Unigene36712\_Sample\_011046840, Unigene37451\_Sample\_011046840, Unigene7646\_Sample\_011046840, Unigene3990\_Sample\_011046840, Unigene3003\_Sample\_011046840, Unigene40004\_Sample\_011046840, Unigene15465\_Sample\_011046840, Unigene39282\_Sample\_011046840, Unigene30987\_Sample\_011046840, Unigene41997\_Sample\_011046840, Unigene37579\_Sample\_011046840, Unigene11897\_Sample\_011046840, Unigene15302\_Sample\_011046840, Unigene24322\_Sample\_011046840, Unigene30924\_Sample\_011046840, Unigene41985\_Sample\_011046840, Unigene38655\_Sample\_011046840, Unigene43473\_Sample\_011046840, Unigene29985\_Sample\_011046840, Unigene15608\_Sample\_011046840, Unigene27598\_Sample\_011046840, Unigene35019\_Sample\_011046840, Unigene28458\_Sample\_011046840, Unigene41843\_Sample\_011046840, Unigene40015\_Sample\_011046840, Unigene33849\_Sample\_011046840, Unigene35061\_Sample\_011046840, Unigene34870\_Sample\_011046840, Unigene43549\_Sample\_011046840, Unigene23221\_Sample\_011046840, Unigene13839\_Sample\_011046840, Unigene32632\_Sample\_011046840, Unigene4744\_Sample\_011046840, Unigene39479\_Sample\_011046840, Unigene41926\_Sample\_011046840, Unigene31211\_Sample\_011046840, Unigene38962\_Sample\_011046840, Unigene41721\_Sample\_011046840, Unigene43293\_Sample\_011046840, Unigene14773\_Sample\_011046840, Unigene1549\_Sample\_011046840, Unigene31629\_Sample\_011046840, Unigene43055\_Sample\_011046840, Unigene1581\_Sample\_011046840, Unigene39554\_Sample\_011046840, Unigene42338\_Sample\_011046840, Unigene28410\_Sample\_011046840, Unigene16994\_Sample\_011046840, Unigene23967\_Sample\_011046840, Unigene2314\_Sample\_011046840, Unigene39646\_Sample\_011046840, Unigene7962\_Sample\_011046840, Unigene39061\_Sample\_011046840, Unigene2243\_Sample\_011046840, Unigene3564\_Sample\_011046840, Unigene27844\_Sample\_011046840, Unigene39625\_Sample\_011046840, Unigene40680\_Sample\_011046840, Unigene41376\_Sample\_011046840, Unigene37545\_Sample\_011046840, Unigene20769\_Sample\_011046840, Unigene36084\_Sample\_011046840, Unigene8222\_Sample\_011046840, Unigene36718\_Sample\_011046840, Unigene42693\_Sample\_011046840, Unigene41368\_Sample\_011046840, Unigene34316\_Sample\_011046840, Unigene42378\_Sample\_011046840, Unigene35961\_Sample\_011046840, Unigene35833\_Sample\_011046840, Unigene11174\_Sample\_011046840, Unigene8137\_Sample\_011046840, Unigene22787\_Sample\_011046840, Unigene2197\_Sample\_011046840, Unigene7871\_Sample\_011046840, Unigene6225\_Sample\_011046840, Unigene37957\_Sample\_011046840, Unigene39660\_Sample\_011046840, Unigene39243\_Sample\_011046840, Unigene23639\_Sample\_011046840, Unigene36654\_Sample\_011046840, Unigene37871\_Sample\_011046840, Unigene43094\_Sample\_011046840, Unigene8067\_Sample\_011046840, Unigene42511\_Sample\_011046840, Unigene33646\_Sample\_011046840, Unigene36481\_Sample\_011046840, Unigene38785\_Sample\_011046840, Unigene2762\_Sample\_011046840, Unigene43312\_Sample\_011046840, Unigene37352\_Sample\_011046840, Unigene42754\_Sample\_011046840, Unigene16376\_Sample\_011046840, Unigene14843\_Sample\_011046840, Unigene34278\_Sample\_011046840, Unigene39923\_Sample\_011046840, Unigene17019\_Sample\_011046840, Unigene3112\_Sample\_011046840, Unigene37653\_Sample\_011046840, Unigene40940\_Sample\_011046840, Unigene43202\_Sample\_011046840, Unigene7821\_Sample\_011046840, Unigene42328\_Sample\_011046840, Unigene26070\_Sample\_011046840, Unigene2863\_Sample\_011046840, Unigene30575\_Sample\_011046840, Unigene41330\_Sample\_011046840, Unigene5178\_Sample\_011046840, Unigene35240\_Sample\_011046840, Unigene8201\_Sample\_011046840, Unigene31901\_Sample\_011046840, Unigene29526\_Sample\_011046840, Unigene40682\_Sample\_011046840, Unigene32895\_Sample\_011046840, Unigene41934\_Sample\_011046840, Unigene43240\_Sample\_011046840, Unigene34480\_Sample\_011046840, Unigene33638\_Sample\_011046840, Unigene40990\_Sample\_011046840, Unigene34052\_Sample\_011046840, Unigene35022\_Sample\_011046840, Unigene33222\_Sample\_011046840, Unigene38519\_Sample\_011046840, Unigene39238\_Sample\_011046840, Unigene17426\_Sample\_011046840, Unigene24389\_Sample\_011046840, Unigene1854\_Sample\_011046840, Unigene24119\_Sample\_011046840, Unigene26441\_Sample\_011046840, Unigene7250\_Sample\_011046840, Unigene31185\_Sample\_011046840, Unigene25892\_Sample\_011046840, Unigene34612\_Sample\_011046840, Unigene34301\_Sample\_011046840, Unigene29499\_Sample\_011046840, Unigene35346\_Sample\_011046840, Unigene789\_Sample\_011046840, Unigene43551\_Sample\_011046840, Unigene32433\_Sample\_011046840, Unigene23273\_Sample\_011046840, Unigene32844\_Sample\_011046840, Unigene14007\_Sample\_011046840, Unigene34436\_Sample\_011046840, Unigene29589\_Sample\_011046840, Unigene40466\_Sample\_011046840, Unigene43413\_Sample\_011046840, Unigene43492\_Sample\_011046840, Unigene40894\_Sample\_011046840, Unigene8083\_Sample\_011046840, Unigene7895\_Sample\_011046840, Unigene30024\_Sample\_011046840, Unigene28951\_Sample\_011046840, Unigene20160\_Sample\_011046840, Unigene30852\_Sample\_011046840, Unigene42272\_Sample\_011046840, Unigene15260\_Sample\_011046840, Unigene42713\_Sample\_011046840, Unigene34106\_Sample\_011046840, Unigene14407\_Sample\_011046840, Unigene43211\_Sample\_011046840, Unigene2820\_Sample\_011046840, Unigene32025\_Sample\_011046840, Unigene39018\_Sample\_011046840, Unigene14074\_Sample\_011046840, Unigene29764\_Sample\_011046840, Unigene5288\_Sample\_011046840, Unigene2272\_Sample\_011046840, Unigene37099\_Sample\_011046840, Unigene15680\_Sample\_011046840, Unigene35547\_Sample\_011046840, Unigene20556\_Sample\_011046840, Unigene29775\_Sample\_011046840, Unigene22514\_Sample\_011046840, Unigene30760\_Sample\_011046840, Unigene42334\_Sample\_011046840, Unigene1829\_Sample\_011046840, Unigene33870\_Sample\_011046840, Unigene32933\_Sample\_011046840, Unigene18703\_Sample\_011046840, Unigene40403\_Sample\_011046840, Unigene35206\_Sample\_011046840, Unigene32581\_Sample\_011046840, Unigene35159\_Sample\_011046840, Unigene7427\_Sample\_011046840, Unigene35031\_Sample\_011046840, Unigene7929\_Sample\_011046840, Unigene42659\_Sample\_011046840, Unigene37034\_Sample\_011046840, Unigene38922\_Sample\_011046840, Unigene42234\_Sample\_011046840, Unigene20494\_Sample\_011046840, Unigene25046\_Sample\_011046840, Unigene22462\_Sample\_011046840, Unigene24692\_Sample\_011046840, Unigene5809\_Sample\_011046840, Unigene20856\_Sample\_011046840, Unigene42889\_Sample\_011046840, Unigene41235\_Sample\_011046840, Unigene9133\_Sample\_011046840, Unigene30679\_Sample\_011046840, Unigene41538\_Sample\_011046840, Unigene37766\_Sample\_011046840, Unigene4460\_Sample\_011046840, Unigene17744\_Sample\_011046840, Unigene7561\_Sample\_011046840, Unigene34588\_Sample\_011046840, Unigene19249\_Sample\_011046840, Unigene2847\_Sample\_011046840, Unigene37075\_Sample\_011046840, Unigene28684\_Sample\_011046840, Unigene35670\_Sample\_011046840, Unigene31299\_Sample\_011046840, Unigene40305\_Sample\_011046840, Unigene7820\_Sample\_011046840, Unigene33045\_Sample\_011046840, Unigene1790\_Sample\_011046840, Unigene41899\_Sample\_011046840, Unigene4472\_Sample\_011046840, Unigene28095\_Sample\_011046840, Unigene8240\_Sample\_011046840, Unigene37960\_Sample\_011046840, Unigene43073\_Sample\_011046840, Unigene4591\_Sample\_011046840, Unigene39440\_Sample\_011046840, Unigene40123\_Sample\_011046840, Unigene38028\_Sample\_011046840, Unigene40677\_Sample\_011046840, Unigene8614\_Sample\_011046840, Unigene8019\_Sample\_011046840, Unigene40861\_Sample\_011046840, Unigene43508\_Sample\_011046840, Unigene37992\_Sample\_011046840, Unigene41717\_Sample\_011046840, Unigene18919\_Sample\_011046840, Unigene15528\_Sample\_011046840, Unigene42206\_Sample\_011046840, Unigene23419\_Sample\_011046840, Unigene10640\_Sample\_011046840, Unigene35342\_Sample\_011046840, Unigene40596\_Sample\_011046840, Unigene20936\_Sample\_011046840, Unigene33202\_Sample\_011046840, Unigene35286\_Sample\_011046840, Unigene15508\_Sample\_011046840, Unigene7296\_Sample\_011046840, Unigene24388\_Sample\_011046840, Unigene43236\_Sample\_011046840, Unigene41663\_Sample\_011046840, Unigene20070\_Sample\_011046840, Unigene4339\_Sample\_011046840, Unigene7669\_Sample\_011046840, Unigene36700\_Sample\_011046840, Unigene9113\_Sample\_011046840, Unigene19703\_Sample\_011046840, Unigene28572\_Sample\_011046840, Unigene27942\_Sample\_011046840, Unigene29255\_Sample\_011046840, Unigene28309\_Sample\_011046840, Unigene42071\_Sample\_011046840, Unigene36942\_Sample\_011046840, Unigene16663\_Sample\_011046840, Unigene23409\_Sample\_011046840, Unigene13799\_Sample\_011046840, Unigene30150\_Sample\_011046840, Unigene35687\_Sample\_011046840, Unigene36459\_Sample\_011046840, Unigene34780\_Sample\_011046840, Unigene29339\_Sample\_011046840, Unigene36813\_Sample\_011046840, Unigene39361\_Sample\_011046840, Unigene28409\_Sample\_011046840, Unigene40942\_Sample\_011046840, Unigene40211\_Sample\_011046840, Unigene14015\_Sample\_011046840, Unigene43385\_Sample\_011046840, Unigene43006\_Sample\_011046840, Unigene43434\_Sample\_011046840, Unigene22310\_Sample\_011046840, Unigene29477\_Sample\_011046840, Unigene29759\_Sample\_011046840, Unigene41485\_Sample\_011046840, Unigene35789\_Sample\_011046840, Unigene12547\_Sample\_011046840, Unigene40513\_Sample\_011046840, Unigene40822\_Sample\_011046840, Unigene22607\_Sample\_011046840, Unigene37627\_Sample\_011046840, Unigene20042\_Sample\_011046840, Unigene42483\_Sample\_011046840, Unigene14782\_Sample\_011046840, Unigene38112\_Sample\_011046840, Unigene41667\_Sample\_011046840, Unigene36317\_Sample\_011046840, Unigene40317\_Sample\_011046840, Unigene42663\_Sample\_011046840, Unigene21266\_Sample\_011046840, Unigene35799\_Sample\_011046840, Unigene41022\_Sample\_011046840, Unigene42596\_Sample\_011046840, Unigene2736\_Sample\_011046840, Unigene41023\_Sample\_011046840, Unigene42717\_Sample\_011046840, Unigene14302\_Sample\_011046840, Unigene41825\_Sample\_011046840, Unigene22354\_Sample\_011046840, Unigene7349\_Sample\_011046840, Unigene41233\_Sample\_011046840, Unigene42247\_Sample\_011046840, Unigene1724\_Sample\_011046840, Unigene8118\_Sample\_011046840, Unigene33697\_Sample\_011046840, Unigene31844\_Sample\_011046840, Unigene38387\_Sample\_011046840, Unigene32073\_Sample\_011046840, Unigene37687\_Sample\_011046840, Unigene3555\_Sample\_011046840, Unigene29655\_Sample\_011046840, Unigene41011\_Sample\_011046840, Unigene7979\_Sample\_011046840, Unigene38577\_Sample\_011046840, Unigene16389\_Sample\_011046840, Unigene29452\_Sample\_011046840, Unigene41539\_Sample\_011046840, Unigene24972\_Sample\_011046840, Unigene37235\_Sample\_011046840, Unigene39503\_Sample\_011046840, Unigene28415\_Sample\_011046840, Unigene10804\_Sample\_011046840, Unigene40718\_Sample\_011046840, Unigene11629\_Sample\_011046840, Unigene25773\_Sample\_011046840, Unigene2672\_Sample\_011046840, Unigene65\_Sample\_011046840, Unigene17329\_Sample\_011046840, Unigene41363\_Sample\_011046840, Unigene43535\_Sample\_011046840, Unigene39271\_Sample\_011046840, Unigene35396\_Sample\_011046840, Unigene8372\_Sample\_011046840, Unigene30155\_Sample\_011046840, Unigene36918\_Sample\_011046840, Unigene42455\_Sample\_011046840, Unigene11451\_Sample\_011046840, Unigene38045\_Sample\_011046840, Unigene43423\_Sample\_011046840, Unigene43547\_Sample\_011046840, Unigene34790\_Sample\_011046840, Unigene20679\_Sample\_011046840, Unigene39962\_Sample\_011046840, Unigene4818\_Sample\_011046840, Unigene21084\_Sample\_011046840, Unigene36114\_Sample\_011046840, Unigene38593\_Sample\_011046840, Unigene6445\_Sample\_011046840, Unigene42097\_Sample\_011046840, Unigene20052\_Sample\_011046840, Unigene5915\_Sample\_011046840, Unigene41081\_Sample\_011046840, Unigene43361\_Sample\_011046840, Unigene28840\_Sample\_011046840, Unigene26552\_Sample\_011046840, Unigene43533\_Sample\_011046840, Unigene38011\_Sample\_011046840, Unigene42934\_Sample\_011046840, Unigene7280\_Sample\_011046840, Unigene39389\_Sample\_011046840, Unigene184\_Sample\_011046840, Unigene15940\_Sample\_011046840, Unigene18382\_Sample\_011046840, Unigene43174\_Sample\_011046840, Unigene21475\_Sample\_011046840, Unigene33673\_Sample\_011046840, Unigene40551\_Sample\_011046840, Unigene35922\_Sample\_011046840, Unigene8063\_Sample\_011046840, Unigene15425\_Sample\_011046840, Unigene22669\_Sample\_011046840, Unigene5733\_Sample\_011046840, Unigene39063\_Sample\_011046840, Unigene40143\_Sample\_011046840, Unigene25735\_Sample\_011046840, Unigene37090\_Sample\_011046840, Unigene25837\_Sample\_011046840, Unigene37108\_Sample\_011046840, Unigene1215\_Sample\_011046840, Unigene15111\_Sample\_011046840, Unigene26170\_Sample\_011046840, Unigene25558\_Sample\_011046840, Unigene25542\_Sample\_011046840, Unigene25406\_Sample\_011046840, Unigene29397\_Sample\_011046840, Unigene28450\_Sample\_011046840, Unigene32876\_Sample\_011046840, Unigene34246\_Sample\_011046840, Unigene42270\_Sample\_011046840, Unigene40139\_Sample\_011046840, Unigene3812\_Sample\_011046840, Unigene3810\_Sample\_011046840, Unigene19260\_Sample\_011046840, Unigene7857\_Sample\_011046840, Unigene37607\_Sample\_011046840, Unigene33173\_Sample\_011046840, Unigene38912\_Sample\_011046840, Unigene28908\_Sample\_011046840, Unigene17885\_Sample\_011046840, Unigene17827\_Sample\_011046840, Unigene19400\_Sample\_011046840, Unigene23045\_Sample\_011046840, Unigene43425\_Sample\_011046840, Unigene33815\_Sample\_011046840, Unigene38667\_Sample\_011046840, Unigene39400\_Sample\_011046840, Unigene26414\_Sample\_011046840, Unigene18685\_Sample\_011046840, Unigene36060\_Sample\_011046840, Unigene38476\_Sample\_011046840, Unigene42818\_Sample\_011046840, Unigene27324\_Sample\_011046840, Unigene36662\_Sample\_011046840, Unigene41401\_Sample\_011046840, Unigene39645\_Sample\_011046840, Unigene4443\_Sample\_011046840, Unigene14800\_Sample\_011046840, Unigene42802\_Sample\_011046840, Unigene39743\_Sample\_011046840, Unigene7950\_Sample\_011046840, Unigene41000\_Sample\_011046840, Unigene31606\_Sample\_011046840, Unigene12004\_Sample\_011046840, Unigene36854\_Sample\_011046840, Unigene26880\_Sample\_011046840, Unigene39086\_Sample\_011046840, Unigene38822\_Sample\_011046840, Unigene9950\_Sample\_011046840, Unigene42905\_Sample\_011046840, Unigene38534\_Sample\_011046840, Unigene41512\_Sample\_011046840, Unigene20630\_Sample\_011046840, Unigene41258\_Sample\_011046840, Unigene38056\_Sample\_011046840, Unigene37644\_Sample\_011046840, Unigene30467\_Sample\_011046840, Unigene39951\_Sample\_011046840, Unigene38730\_Sample\_011046840, Unigene39515\_Sample\_011046840, Unigene25001\_Sample\_011046840, Unigene38735\_Sample\_011046840, Unigene35839\_Sample\_011046840, Unigene23020\_Sample\_011046840, Unigene34973\_Sample\_011046840, Unigene41679\_Sample\_011046840, Unigene34795\_Sample\_011046840, Unigene23590\_Sample\_011046840, Unigene39007\_Sample\_011046840, Unigene34360\_Sample\_011046840, Unigene41716\_Sample\_011046840, Unigene1226\_Sample\_011046840, Unigene37469\_Sample\_011046840, Unigene36671\_Sample\_011046840, Unigene11390\_Sample\_011046840, Unigene2307\_Sample\_011046840, Unigene12277\_Sample\_011046840, Unigene8144\_Sample\_011046840, Unigene26820\_Sample\_011046840, Unigene42164\_Sample\_011046840, Unigene39421\_Sample\_011046840, Unigene38675\_Sample\_011046840, Unigene6919\_Sample\_011046840, Unigene42173\_Sample\_011046840, Unigene32598\_Sample\_011046840, Unigene29603\_Sample\_011046840, Unigene41173\_Sample\_011046840, Unigene20078\_Sample\_011046840, Unigene40439\_Sample\_011046840, Unigene43078\_Sample\_011046840, Unigene4823\_Sample\_011046840, Unigene41059\_Sample\_011046840, Unigene27802\_Sample\_011046840, Unigene1967\_Sample\_011046840, Unigene37918\_Sample\_011046840, Unigene43216\_Sample\_011046840, Unigene34992\_Sample\_011046840, Unigene25727\_Sample\_011046840, Unigene6409\_Sample\_011046840, Unigene6181\_Sample\_011046840, Unigene29823\_Sample\_011046840, Unigene40704\_Sample\_011046840, Unigene18005\_Sample\_011046840, Unigene28676\_Sample\_011046840, Unigene43336\_Sample\_011046840, Unigene5566\_Sample\_011046840, Unigene41304\_Sample\_011046840, Unigene35787\_Sample\_011046840, Unigene41183\_Sample\_011046840, Unigene36482\_Sample\_011046840, Unigene36713\_Sample\_011046840, Unigene8098\_Sample\_011046840, Unigene3712\_Sample\_011046840, Unigene35792\_Sample\_011046840, Unigene29814\_Sample\_011046840, Unigene35916\_Sample\_011046840, Unigene25585\_Sample\_011046840, Unigene29363\_Sample\_011046840, Unigene42849\_Sample\_011046840, Unigene4234\_Sample\_011046840, Unigene42568\_Sample\_011046840, Unigene27358\_Sample\_011046840, Unigene13113\_Sample\_011046840, Unigene37793\_Sample\_011046840, Unigene20290\_Sample\_011046840, Unigene20546\_Sample\_011046840, Unigene19258\_Sample\_011046840, Unigene42597\_Sample\_011046840, Unigene2439\_Sample\_011046840, Unigene21153\_Sample\_011046840, Unigene35676\_Sample\_011046840, Unigene36475\_Sample\_011046840, Unigene34108\_Sample\_011046840, Unigene18863\_Sample\_011046840, Unigene38852\_Sample\_011046840, Unigene19079\_Sample\_011046840, Unigene34717\_Sample\_011046840, Unigene40655\_Sample\_011046840, Unigene7324\_Sample\_011046840, Unigene42962\_Sample\_011046840, Unigene23643\_Sample\_011046840, Unigene13709\_Sample\_011046840, Unigene3580\_Sample\_011046840, Unigene37738\_Sample\_011046840, Unigene32318\_Sample\_011046840, Unigene39725\_Sample\_011046840, Unigene16130\_Sample\_011046840, Unigene34102\_Sample\_011046840, Unigene42240\_Sample\_011046840, Unigene41729\_Sample\_011046840, Unigene27975\_Sample\_011046840, Unigene22532\_Sample\_011046840, Unigene14511\_Sample\_011046840, Unigene8171\_Sample\_011046840, Unigene20745\_Sample\_011046840, Unigene32749\_Sample\_011046840, Unigene12521\_Sample\_011046840, Unigene19887\_Sample\_011046840, Unigene40896\_Sample\_011046840, Unigene42377\_Sample\_011046840, Unigene42383\_Sample\_011046840, Unigene16269\_Sample\_011046840, Unigene28317\_Sample\_011046840, Unigene2536\_Sample\_011046840, Unigene32865\_Sample\_011046840, Unigene40065\_Sample\_011046840, Unigene34857\_Sample\_011046840, Unigene30627\_Sample\_011046840, Unigene4481\_Sample\_011046840, Unigene42935\_Sample\_011046840, Unigene39257\_Sample\_011046840, Unigene27808\_Sample\_011046840, Unigene39866\_Sample\_011046840, Unigene41562\_Sample\_011046840, Unigene23252\_Sample\_011046840, Unigene36642\_Sample\_011046840, Unigene37526\_Sample\_011046840, Unigene11499\_Sample\_011046840, Unigene22230\_Sample\_011046840, Unigene1963\_Sample\_011046840, Unigene34759\_Sample\_011046840, Unigene41228\_Sample\_011046840, Unigene31417\_Sample\_011046840, Unigene33765\_Sample\_011046840, Unigene34098\_Sample\_011046840, Unigene40994\_Sample\_011046840, Unigene41903\_Sample\_011046840, Unigene39655\_Sample\_011046840, Unigene6867\_Sample\_011046840, Unigene36936\_Sample\_011046840, Unigene5461\_Sample\_011046840, Unigene42457\_Sample\_011046840, Unigene42679\_Sample\_011046840, Unigene31945\_Sample\_011046840, Unigene23357\_Sample\_011046840, Unigene7665\_Sample\_011046840, Unigene8218\_Sample\_011046840, Unigene33047\_Sample\_011046840, Unigene30650\_Sample\_011046840, Unigene6147\_Sample\_011046840, Unigene41441\_Sample\_011046840, Unigene36112\_Sample\_011046840, Unigene13380\_Sample\_011046840, Unigene20250\_Sample\_011046840, Unigene27063\_Sample\_011046840, Unigene33426\_Sample\_011046840, Unigene14611\_Sample\_011046840, Unigene9299\_Sample\_011046840, Unigene40424\_Sample\_011046840, Unigene35475\_Sample\_011046840, Unigene37081\_Sample\_011046840, Unigene20219\_Sample\_011046840, Unigene5038\_Sample\_011046840, Unigene7945\_Sample\_011046840, Unigene16118\_Sample\_011046840, Unigene27997\_Sample\_011046840, Unigene40332\_Sample\_011046840, Unigene40124\_Sample\_011046840, Unigene8156\_Sample\_011046840, Unigene31258\_Sample\_011046840, Unigene20383\_Sample\_011046840, Unigene43431\_Sample\_011046840, Unigene32610\_Sample\_011046840, Unigene32885\_Sample\_011046840, Unigene36139\_Sample\_011046840, Unigene11483\_Sample\_011046840, Unigene28736\_Sample\_011046840, Unigene24992\_Sample\_011046840, Unigene4005\_Sample\_011046840, Unigene509\_Sample\_011046840, Unigene33613\_Sample\_011046840, Unigene14753\_Sample\_011046840, Unigene41432\_Sample\_011046840, Unigene35746\_Sample\_011046840, Unigene12713\_Sample\_011046840, Unigene42985\_Sample\_011046840, Unigene27799\_Sample\_011046840, Unigene41079\_Sample\_011046840, Unigene11613\_Sample\_011046840, Unigene26792\_Sample\_011046840, Unigene19906\_Sample\_011046840, Unigene41715\_Sample\_011046840, Unigene2117\_Sample\_011046840, Unigene20296\_Sample\_011046840, Unigene13071\_Sample\_011046840, Unigene40346\_Sample\_011046840, Unigene6473\_Sample\_011046840, Unigene32864\_Sample\_011046840, Unigene37626\_Sample\_011046840, Unigene34716\_Sample\_011046840, Unigene43526\_Sample\_011046840, Unigene31610\_Sample\_011046840, Unigene643\_Sample\_011046840, Unigene34967\_Sample\_011046840, Unigene21000\_Sample\_011046840, Unigene36284\_Sample\_011046840, Unigene6114\_Sample\_011046840, Unigene30699\_Sample\_011046840, Unigene5271\_Sample\_011046840, Unigene28122\_Sample\_011046840, Unigene39838\_Sample\_011046840, Unigene1920\_Sample\_011046840, Unigene2470\_Sample\_011046840, Unigene39517\_Sample\_011046840, Unigene3842\_Sample\_011046840, Unigene31321\_Sample\_011046840, Unigene34257\_Sample\_011046840, Unigene3928\_Sample\_011046840, Unigene23856\_Sample\_011046840, Unigene26789\_Sample\_011046840, Unigene22258\_Sample\_011046840, Unigene41998\_Sample\_011046840, Unigene41139\_Sample\_011046840, Unigene38322\_Sample\_011046840, Unigene35461\_Sample\_011046840, Unigene4492\_Sample\_011046840, Unigene16736\_Sample\_011046840, Unigene38932\_Sample\_011046840, Unigene20308\_Sample\_011046840, Unigene33698\_Sample\_011046840, Unigene32905\_Sample\_011046840, Unigene37858\_Sample\_011046840, Unigene40137\_Sample\_011046840, Unigene5197\_Sample\_011046840, Unigene16329\_Sample\_011046840, Unigene36330\_Sample\_011046840, Unigene31582\_Sample\_011046840, Unigene36301\_Sample\_011046840, Unigene37218\_Sample\_011046840, Unigene30518\_Sample\_011046840, Unigene15362\_Sample\_011046840, Unigene31941\_Sample\_011046840, Unigene26892\_Sample\_011046840, Unigene41448\_Sample\_011046840, Unigene41518\_Sample\_011046840, Unigene24591\_Sample\_011046840, Unigene36690\_Sample\_011046840, Unigene21026\_Sample\_011046840, Unigene43376\_Sample\_011046840, Unigene24163\_Sample\_011046840, Unigene35723\_Sample\_011046840, Unigene29679\_Sample\_011046840, Unigene39176\_Sample\_011046840, Unigene30094\_Sample\_011046840, Unigene31860\_Sample\_011046840, Unigene25579\_Sample\_011046840, Unigene34053\_Sample\_011046840, Unigene42977\_Sample\_011046840, Unigene22877\_Sample\_011046840, Unigene39899\_Sample\_011046840, Unigene37209\_Sample\_011046840, Unigene38216\_Sample\_011046840, Unigene30332\_Sample\_011046840, Unigene886\_Sample\_011046840, Unigene38362\_Sample\_011046840, Unigene2053\_Sample\_011046840, Unigene43558\_Sample\_011046840, Unigene7035\_Sample\_011046840, Unigene42591\_Sample\_011046840, Unigene32894\_Sample\_011046840, Unigene28241\_Sample\_011046840, Unigene27015\_Sample\_011046840, Unigene29789\_Sample\_011046840, Unigene7295\_Sample\_011046840, Unigene27879\_Sample\_011046840, Unigene38633\_Sample\_011046840, Unigene40973\_Sample\_011046840, Unigene19126\_Sample\_011046840, Unigene30504\_Sample\_011046840, Unigene23684\_Sample\_011046840, Unigene39591\_Sample\_011046840, Unigene24342\_Sample\_011046840, Unigene29099\_Sample\_011046840, Unigene31137\_Sample\_011046840, Unigene9987\_Sample\_011046840, Unigene20980\_Sample\_011046840, Unigene41746\_Sample\_011046840, Unigene16518\_Sample\_011046840, Unigene39500\_Sample\_011046840, Unigene41336\_Sample\_011046840, Unigene42778\_Sample\_011046840, Unigene30573\_Sample\_011046840, Unigene34746\_Sample\_011046840, Unigene8172\_Sample\_011046840, Unigene26273\_Sample\_011046840, Unigene39303\_Sample\_011046840, Unigene7914\_Sample\_011046840, Unigene43409\_Sample\_011046840, Unigene37745\_Sample\_011046840, Unigene22327\_Sample\_011046840, Unigene24674\_Sample\_011046840, Unigene36969\_Sample\_011046840, Unigene40184\_Sample\_011046840, Unigene23150\_Sample\_011046840, Unigene37365\_Sample\_011046840, Unigene42945\_Sample\_011046840, Unigene33078\_Sample\_011046840, Unigene25523\_Sample\_011046840, Unigene26849\_Sample\_011046840, Unigene31910\_Sample\_011046840, Unigene43453\_Sample\_011046840, Unigene41479\_Sample\_011046840, Unigene39490\_Sample\_011046840, Unigene6446\_Sample\_011046840, Unigene13410\_Sample\_011046840, Unigene38951\_Sample\_011046840, Unigene6632\_Sample\_011046840, Unigene20420\_Sample\_011046840, Unigene39795\_Sample\_011046840, Unigene30584\_Sample\_011046840, Unigene41765\_Sample\_011046840, Unigene29586\_Sample\_011046840, Unigene43031\_Sample\_011046840, Unigene6541\_Sample\_011046840, Unigene22232\_Sample\_011046840, Unigene27364\_Sample\_011046840, Unigene24776\_Sample\_011046840, Unigene42867\_Sample\_011046840, Unigene7149\_Sample\_011046840, Unigene40246\_Sample\_011046840, Unigene37244\_Sample\_011046840, Unigene37735\_Sample\_011046840, Unigene42079\_Sample\_011046840, Unigene16615\_Sample\_011046840, Unigene3161\_Sample\_011046840, Unigene40387\_Sample\_011046840, Unigene40901\_Sample\_011046840, Unigene37396\_Sample\_011046840, Unigene4721\_Sample\_011046840, Unigene7739\_Sample\_011046840, Unigene36688\_Sample\_011046840, Unigene29879\_Sample\_011046840, Unigene1732\_Sample\_011046840, Unigene1557\_Sample\_011046840, Unigene5782\_Sample\_011046840, Unigene693\_Sample\_011046840, Unigene33501\_Sample\_011046840, Unigene40988\_Sample\_011046840, Unigene4784\_Sample\_011046840, Unigene34434\_Sample\_011046840, Unigene35663\_Sample\_011046840, Unigene6787\_Sample\_011046840, Unigene5393\_Sample\_011046840, Unigene37573\_Sample\_011046840, Unigene4077\_Sample\_011046840, Unigene2420\_Sample\_011046840, Unigene21043\_Sample\_011046840, Unigene37879\_Sample\_011046840, Unigene42793\_Sample\_011046840, Unigene42837\_Sample\_011046840, Unigene23365\_Sample\_011046840, Unigene42475\_Sample\_011046840, Unigene39986\_Sample\_011046840, Unigene30897\_Sample\_011046840, Unigene39416\_Sample\_011046840, Unigene42204\_Sample\_011046840, Unigene20783\_Sample\_011046840, Unigene17312\_Sample\_011046840, Unigene32871\_Sample\_011046840, Unigene6035\_Sample\_011046840, Unigene16630\_Sample\_011046840, Unigene31324\_Sample\_011046840, Unigene22123\_Sample\_011046840, Unigene41821\_Sample\_011046840, Unigene41701\_Sample\_011046840, Unigene40059\_Sample\_011046840, Unigene11534\_Sample\_011046840, Unigene40878\_Sample\_011046840, Unigene40208\_Sample\_011046840, Unigene41803\_Sample\_011046840, Unigene35996\_Sample\_011046840, Unigene4554\_Sample\_011046840, Unigene13916\_Sample\_011046840, Unigene42836\_Sample\_011046840, Unigene14237\_Sample\_011046840, Unigene2626\_Sample\_011046840, Unigene3395\_Sample\_011046840, Unigene40296\_Sample\_011046840, Unigene19433\_Sample\_011046840, Unigene9361\_Sample\_011046840, Unigene42886\_Sample\_011046840, Unigene31563\_Sample\_011046840, Unigene28530\_Sample\_011046840, Unigene14705\_Sample\_011046840, Unigene35817\_Sample\_011046840, Unigene33515\_Sample\_011046840, Unigene3999\_Sample\_011046840, Unigene41046\_Sample\_011046840, Unigene31044\_Sample\_011046840, Unigene42289\_Sample\_011046840, Unigene3164\_Sample\_011046840, Unigene30530\_Sample\_011046840, Unigene35417\_Sample\_011046840, Unigene7794\_Sample\_011046840, Unigene31707\_Sample\_011046840, Unigene10460\_Sample\_011046840, Unigene39082\_Sample\_011046840, Unigene28418\_Sample\_011046840, Unigene20038\_Sample\_011046840, Unigene25687\_Sample\_011046840, Unigene42956\_Sample\_011046840, Unigene39505\_Sample\_011046840, Unigene41606\_Sample\_011046840, Unigene40456\_Sample\_011046840, Unigene37702\_Sample\_011046840, Unigene43525\_Sample\_011046840, Unigene6216\_Sample\_011046840, Unigene5594\_Sample\_011046840, Unigene1672\_Sample\_011046840, Unigene35068\_Sample\_011046840, Unigene33158\_Sample\_011046840, Unigene35198\_Sample\_011046840, Unigene34280\_Sample\_011046840, Unigene43083\_Sample\_011046840, Unigene32223\_Sample\_011046840, Unigene9730\_Sample\_011046840, Unigene42872\_Sample\_011046840, Unigene28259\_Sample\_011046840, Unigene43245\_Sample\_011046840, Unigene21379\_Sample\_011046840, Unigene38753\_Sample\_011046840, Unigene23000\_Sample\_011046840, Unigene5305\_Sample\_011046840, Unigene38000\_Sample\_011046840, Unigene36140\_Sample\_011046840, Unigene8385\_Sample\_011046840, Unigene32116\_Sample\_011046840, Unigene40297\_Sample\_011046840, Unigene42243\_Sample\_011046840, Unigene14154\_Sample\_011046840, Unigene35698\_Sample\_011046840, Unigene37391\_Sample\_011046840, Unigene32278\_Sample\_011046840, Unigene27950\_Sample\_011046840, Unigene1039\_Sample\_011046840, Unigene8214\_Sample\_011046840, Unigene5591\_Sample\_011046840, Unigene25093\_Sample\_011046840, Unigene29386\_Sample\_011046840, Unigene17105\_Sample\_011046840, Unigene43415\_Sample\_011046840, Unigene12636\_Sample\_011046840, Unigene8452\_Sample\_011046840, Unigene34508\_Sample\_011046840, Unigene7091\_Sample\_011046840, Unigene17239\_Sample\_011046840, Unigene143\_Sample\_011046840, Unigene42215\_Sample\_011046840, Unigene16751\_Sample\_011046840, Unigene1087\_Sample\_011046840, Unigene13934\_Sample\_011046840, Unigene15420\_Sample\_011046840, Unigene36636\_Sample\_011046840, Unigene33387\_Sample\_011046840, Unigene31608\_Sample\_011046840, Unigene42680\_Sample\_011046840, Unigene24398\_Sample\_011046840, Unigene6828\_Sample\_011046840, Unigene38712\_Sample\_011046840, Unigene30141\_Sample\_011046840, Unigene36041\_Sample\_011046840, Unigene34624\_Sample\_011046840, Unigene34555\_Sample\_011046840, Unigene7375\_Sample\_011046840, Unigene32875\_Sample\_011046840, Unigene32424\_Sample\_011046840, Unigene42386\_Sample\_011046840, Unigene7734\_Sample\_011046840, Unigene41853\_Sample\_011046840, Unigene41607\_Sample\_011046840, Unigene34509\_Sample\_011046840, Unigene3339\_Sample\_011046840, Unigene39832\_Sample\_011046840, Unigene1942\_Sample\_011046840, Unigene24166\_Sample\_011046840, Unigene32706\_Sample\_011046840, Unigene16236\_Sample\_011046840, Unigene29599\_Sample\_011046840, Unigene8359\_Sample\_011046840, Unigene38444\_Sample\_011046840, Unigene35269\_Sample\_011046840, Unigene7189\_Sample\_011046840, Unigene29696\_Sample\_011046840, Unigene34715\_Sample\_011046840, Unigene42808\_Sample\_011046840, Unigene19098\_Sample\_011046840, Unigene39429\_Sample\_011046840, Unigene13340\_Sample\_011046840, Unigene24700\_Sample\_011046840, Unigene36791\_Sample\_011046840, Unigene6624\_Sample\_011046840, Unigene40042\_Sample\_011046840, Unigene12663\_Sample\_011046840, Unigene28521\_Sample\_011046840, Unigene40344\_Sample\_011046840, Unigene32183\_Sample\_011046840, Unigene40795\_Sample\_011046840, Unigene29240\_Sample\_011046840, Unigene14438\_Sample\_011046840, Unigene42569\_Sample\_011046840, Unigene36007\_Sample\_011046840, Unigene41056\_Sample\_011046840, Unigene3514\_Sample\_011046840, Unigene6711\_Sample\_011046840, Unigene23891\_Sample\_011046840, Unigene42819\_Sample\_011046840, Unigene22942\_Sample\_011046840, Unigene4815\_Sample\_011046840, Unigene37267\_Sample\_011046840, Unigene26865\_Sample\_011046840, Unigene39332\_Sample\_011046840, Unigene32927\_Sample\_011046840, Unigene14273\_Sample\_011046840, Unigene29504\_Sample\_011046840, Unigene23169\_Sample\_011046840, Unigene33810\_Sample\_011046840, Unigene16223\_Sample\_011046840, Unigene2581\_Sample\_011046840, Unigene31435\_Sample\_011046840, Unigene41980\_Sample\_011046840, Unigene12911\_Sample\_011046840, Unigene43583\_Sample\_011046840, Unigene37776\_Sample\_011046840, Unigene34147\_Sample\_011046840, Unigene39157\_Sample\_011046840, Unigene42325\_Sample\_011046840, Unigene42936\_Sample\_011046840, Unigene43277\_Sample\_011046840, Unigene28501\_Sample\_011046840, Unigene39497\_Sample\_011046840, Unigene2218\_Sample\_011046840, Unigene37251\_Sample\_011046840, Unigene14503\_Sample\_011046840, Unigene38058\_Sample\_011046840, Unigene41356\_Sample\_011046840, Unigene39304\_Sample\_011046840, Unigene7458\_Sample\_011046840, Unigene33589\_Sample\_011046840, Unigene29637\_Sample\_011046840, Unigene38693\_Sample\_011046840, Unigene37370\_Sample\_011046840, Unigene33699\_Sample\_011046840, Unigene36391\_Sample\_011046840, Unigene38722\_Sample\_011046840, Unigene42217\_Sample\_011046840, Unigene12359\_Sample\_011046840, Unigene43577\_Sample\_011046840, Unigene26536\_Sample\_011046840, Unigene42269\_Sample\_011046840, Unigene34180\_Sample\_011046840, Unigene6862\_Sample\_011046840, Unigene24414\_Sample\_011046840, Unigene13357\_Sample\_011046840, Unigene2687\_Sample\_011046840, Unigene42634\_Sample\_011046840, Unigene39430\_Sample\_011046840, Unigene34605\_Sample\_011046840, Unigene35459\_Sample\_011046840, Unigene6135\_Sample\_011046840, Unigene43412\_Sample\_011046840, Unigene38760\_Sample\_011046840, Unigene31200\_Sample\_011046840, Unigene16550\_Sample\_011046840, Unigene39717\_Sample\_011046840, Unigene28549\_Sample\_011046840, Unigene30383\_Sample\_011046840, Unigene8010\_Sample\_011046840, Unigene38100\_Sample\_011046840, Unigene37467\_Sample\_011046840, Unigene14853\_Sample\_011046840, Unigene4497\_Sample\_011046840, Unigene42466\_Sample\_011046840, Unigene35821\_Sample\_011046840, Unigene24183\_Sample\_011046840, Unigene27894\_Sample\_011046840, Unigene37741\_Sample\_011046840, Unigene43282\_Sample\_011046840, Unigene25706\_Sample\_011046840, Unigene40193\_Sample\_011046840, Unigene38093\_Sample\_011046840, Unigene36531\_Sample\_011046840, Unigene2690\_Sample\_011046840, Unigene36308\_Sample\_011046840, Unigene27040\_Sample\_011046840, Unigene36219\_Sample\_011046840, Unigene19918\_Sample\_011046840, Unigene42519\_Sample\_011046840, Unigene3068\_Sample\_011046840, Unigene43238\_Sample\_011046840, Unigene33083\_Sample\_011046840, Unigene41943\_Sample\_011046840, Unigene43007\_Sample\_011046840, Unigene4028\_Sample\_011046840, Unigene15987\_Sample\_011046840, Unigene42315\_Sample\_011046840, Unigene20800\_Sample\_011046840, Unigene38422\_Sample\_011046840, Unigene41480\_Sample\_011046840, Unigene27473\_Sample\_011046840, Unigene26705\_Sample\_011046840, Unigene41345\_Sample\_011046840, Unigene38103\_Sample\_011046840, Unigene31527\_Sample\_011046840, Unigene38749\_Sample\_011046840, Unigene22626\_Sample\_011046840, Unigene30034\_Sample\_011046840, Unigene41030\_Sample\_011046840, Unigene37397\_Sample\_011046840, Unigene28872\_Sample\_011046840, Unigene37695\_Sample\_011046840, Unigene38900\_Sample\_011046840, Unigene4521\_Sample\_011046840, Unigene32476\_Sample\_011046840, Unigene13275\_Sample\_011046840, Unigene2380\_Sample\_011046840, Unigene9759\_Sample\_011046840, Unigene25850\_Sample\_011046840, Unigene30648\_Sample\_011046840, Unigene26846\_Sample\_011046840, Unigene9318\_Sample\_011046840, Unigene39519\_Sample\_011046840, Unigene6781\_Sample\_011046840, Unigene34017\_Sample\_011046840, Unigene20940\_Sample\_011046840, Unigene4143\_Sample\_011046840, Unigene21415\_Sample\_011046840, Unigene34691\_Sample\_011046840, Unigene6041\_Sample\_011046840, Unigene26726\_Sample\_011046840, Unigene42525\_Sample\_011046840, Unigene29975\_Sample\_011046840, Unigene41334\_Sample\_011046840, Unigene43283\_Sample\_011046840, Unigene102\_Sample\_011046840, Unigene25448\_Sample\_011046840, Unigene10\_Sample\_011046840, Unigene42418\_Sample\_011046840, Unigene35782\_Sample\_011046840, Unigene42481\_Sample\_011046840, Unigene6659\_Sample\_011046840, Unigene31880\_Sample\_011046840, Unigene7988\_Sample\_011046840, Unigene40738\_Sample\_011046840, Unigene7448\_Sample\_011046840, Unigene41329\_Sample\_011046840, Unigene13731\_Sample\_011046840, Unigene21144\_Sample\_011046840, Unigene31084\_Sample\_011046840, Unigene35477\_Sample\_011046840, Unigene5453\_Sample\_011046840, Unigene40204\_Sample\_011046840, Unigene25928\_Sample\_011046840, Unigene43416\_Sample\_011046840, Unigene32934\_Sample\_011046840, Unigene38648\_Sample\_011046840, Unigene40494\_Sample\_011046840, Unigene5669\_Sample\_011046840, Unigene29145\_Sample\_011046840, Unigene11522\_Sample\_011046840, Unigene4187\_Sample\_011046840, Unigene31914\_Sample\_011046840, Unigene6987\_Sample\_011046840, Unigene26212\_Sample\_011046840, Unigene41160\_Sample\_011046840, Unigene12708\_Sample\_011046840, Unigene19472\_Sample\_011046840, Unigene34304\_Sample\_011046840, Unigene32730\_Sample\_011046840, Unigene41544\_Sample\_011046840, Unigene41982\_Sample\_011046840, Unigene36238\_Sample\_011046840, Unigene34536\_Sample\_011046840, Unigene24508\_Sample\_011046840, Unigene39106\_Sample\_011046840, Unigene27013\_Sample\_011046840, Unigene38825\_Sample\_011046840, Unigene12524\_Sample\_011046840, Unigene21188\_Sample\_011046840, Unigene42094\_Sample\_011046840, Unigene40309\_Sample\_011046840, Unigene30435\_Sample\_011046840, Unigene1926\_Sample\_011046840, Unigene43489\_Sample\_011046840, Unigene42320\_Sample\_011046840, Unigene26065\_Sample\_011046840, Unigene42641\_Sample\_011046840, Unigene41695\_Sample\_011046840, Unigene41799\_Sample\_011046840, Unigene41795\_Sample\_011046840, Unigene24767\_Sample\_011046840, Unigene31512\_Sample\_011046840, Unigene13416\_Sample\_011046840, Unigene28292\_Sample\_011046840, Unigene18537\_Sample\_011046840, Unigene22397\_Sample\_011046840, Unigene6890\_Sample\_011046840, Unigene4626\_Sample\_011046840, Unigene20109\_Sample\_011046840, Unigene26932\_Sample\_011046840, Unigene33790\_Sample\_011046840, Unigene39702\_Sample\_011046840, Unigene31695\_Sample\_011046840, Unigene42116\_Sample\_011046840, Unigene43257\_Sample\_011046840, Unigene43495\_Sample\_011046840, Unigene29713\_Sample\_011046840, Unigene3492\_Sample\_011046840, Unigene42354\_Sample\_011046840, Unigene34328\_Sample\_011046840, Unigene39075\_Sample\_011046840, Unigene42862\_Sample\_011046840, Unigene7058\_Sample\_011046840, Unigene38329\_Sample\_011046840, Unigene22181\_Sample\_011046840, Unigene36070\_Sample\_011046840, Unigene34874\_Sample\_011046840, Unigene6793\_Sample\_011046840, Unigene1705\_Sample\_011046840, Unigene7525\_Sample\_011046840, Unigene32637\_Sample\_011046840, Unigene40064\_Sample\_011046840, Unigene24124\_Sample\_011046840, Unigene40586\_Sample\_011046840, Unigene28820\_Sample\_011046840, Unigene31662\_Sample\_011046840, Unigene13805\_Sample\_011046840, Unigene41093\_Sample\_011046840, Unigene4807\_Sample\_011046840, Unigene15087\_Sample\_011046840, Unigene92\_Sample\_011046840, Unigene39204\_Sample\_011046840, Unigene39588\_Sample\_011046840, Unigene7944\_Sample\_011046840, Unigene1271\_Sample\_011046840, Unigene38502\_Sample\_011046840, Unigene35860\_Sample\_011046840, Unigene26937\_Sample\_011046840, Unigene36561\_Sample\_011046840, Unigene35008\_Sample\_011046840, Unigene11193\_Sample\_011046840, Unigene4972\_Sample\_011046840, Unigene28433\_Sample\_011046840, Unigene17218\_Sample\_011046840, Unigene1022\_Sample\_011046840, Unigene29392\_Sample\_011046840, Unigene11820\_Sample\_011046840, Unigene39649\_Sample\_011046840, Unigene31424\_Sample\_011046840, Unigene31576\_Sample\_011046840, Unigene9628\_Sample\_011046840, Unigene2986\_Sample\_011046840, Unigene43490\_Sample\_011046840, Unigene19450\_Sample\_011046840, Unigene43486\_Sample\_011046840, Unigene35141\_Sample\_011046840, Unigene43218\_Sample\_011046840, Unigene10850\_Sample\_011046840, Unigene31037\_Sample\_011046840, Unigene7429\_Sample\_011046840, Unigene4491\_Sample\_011046840, Unigene14928\_Sample\_011046840, Unigene7825\_Sample\_011046840, Unigene34610\_Sample\_011046840, Unigene29972\_Sample\_011046840, Unigene35195\_Sample\_011046840, Unigene19183\_Sample\_011046840, Unigene35735\_Sample\_011046840, Unigene40357\_Sample\_011046840, Unigene33659\_Sample\_011046840, Unigene34909\_Sample\_011046840, Unigene22452\_Sample\_011046840, Unigene4529\_Sample\_011046840, Unigene33360\_Sample\_011046840, Unigene37829\_Sample\_011046840, Unigene21598\_Sample\_011046840, Unigene17520\_Sample\_011046840, Unigene41619\_Sample\_011046840, Unigene8133\_Sample\_011046840, Unigene7221\_Sample\_011046840, Unigene42376\_Sample\_011046840, Unigene35131\_Sample\_011046840, Unigene39644\_Sample\_011046840, Unigene16618\_Sample\_011046840, Unigene40459\_Sample\_011046840, Unigene30566\_Sample\_011046840, Unigene3258\_Sample\_011046840, Unigene18413\_Sample\_011046840, Unigene13322\_Sample\_011046840, Unigene11128\_Sample\_011046840, Unigene465\_Sample\_011046840, Unigene22032\_Sample\_011046840, Unigene34023\_Sample\_011046840, Unigene7064\_Sample\_011046840, Unigene39883\_Sample\_011046840, Unigene1475\_Sample\_011046840, Unigene41978\_Sample\_011046840, Unigene39541\_Sample\_011046840, Unigene1897\_Sample\_011046840, Unigene4652\_Sample\_011046840, Unigene5678\_Sample\_011046840, Unigene40452\_Sample\_011046840, Unigene13531\_Sample\_011046840, Unigene34137\_Sample\_011046840, Unigene7596\_Sample\_011046840, Unigene31604\_Sample\_011046840, Unigene19434\_Sample\_011046840, Unigene14811\_Sample\_011046840, Unigene42707\_Sample\_011046840, Unigene9554\_Sample\_011046840, Unigene19865\_Sample\_011046840, Unigene5482\_Sample\_011046840, Unigene39970\_Sample\_011046840, Unigene38669\_Sample\_011046840, Unigene5527\_Sample\_011046840, Unigene33228\_Sample\_011046840, Unigene31320\_Sample\_011046840, Unigene618\_Sample\_011046840, Unigene38106\_Sample\_011046840, Unigene43569\_Sample\_011046840, Unigene2665\_Sample\_011046840, Unigene39686\_Sample\_011046840, Unigene2864\_Sample\_011046840, Unigene34806\_Sample\_011046840, Unigene43231\_Sample\_011046840, Unigene43028\_Sample\_011046840, Unigene30367\_Sample\_011046840, Unigene4129\_Sample\_011046840, Unigene42266\_Sample\_011046840, Unigene39273\_Sample\_011046840, Unigene40519\_Sample\_011046840, Unigene29758\_Sample\_011046840, Unigene25902\_Sample\_011046840, Unigene37578\_Sample\_011046840, Unigene43210\_Sample\_011046840, Unigene37192\_Sample\_011046840, Unigene39938\_Sample\_011046840, Unigene2180\_Sample\_011046840, Unigene2072\_Sample\_011046840, Unigene31226\_Sample\_011046840, Unigene41811\_Sample\_011046840, Unigene28270\_Sample\_011046840, Unigene42992\_Sample\_011046840, Unigene39895\_Sample\_011046840, Unigene39234\_Sample\_011046840, Unigene32740\_Sample\_011046840, Unigene23189\_Sample\_011046840, Unigene8191\_Sample\_011046840, Unigene12842\_Sample\_011046840, Unigene39615\_Sample\_011046840, Unigene11646\_Sample\_011046840, Unigene37969\_Sample\_011046840, Unigene35768\_Sample\_011046840, Unigene30051\_Sample\_011046840, Unigene43126\_Sample\_011046840, Unigene31705\_Sample\_011046840, Unigene35001\_Sample\_011046840, Unigene33550\_Sample\_011046840, Unigene32240\_Sample\_011046840, Unigene37285\_Sample\_011046840, Unigene1389\_Sample\_011046840, Unigene25049\_Sample\_011046840, Unigene36875\_Sample\_011046840, Unigene34695\_Sample\_011046840, Unigene32573\_Sample\_011046840, Unigene37083\_Sample\_011046840, Unigene24133\_Sample\_011046840, Unigene43573\_Sample\_011046840, Unigene6203\_Sample\_011046840, Unigene30514\_Sample\_011046840, Unigene35784\_Sample\_011046840, Unigene39079\_Sample\_011046840, Unigene41642\_Sample\_011046840, Unigene7302\_Sample\_011046840, Unigene28934\_Sample\_011046840, Unigene37880\_Sample\_011046840, Unigene35174\_Sample\_011046840, Unigene3204\_Sample\_011046840, Unigene39836\_Sample\_011046840, Unigene34448\_Sample\_011046840, Unigene35483\_Sample\_011046840, Unigene37484\_Sample\_011046840, Unigene19344\_Sample\_011046840, Unigene13358\_Sample\_011046840, Unigene42073\_Sample\_011046840, Unigene21108\_Sample\_011046840, Unigene42142\_Sample\_011046840, Unigene43002\_Sample\_011046840, Unigene39602\_Sample\_011046840, Unigene41194\_Sample\_011046840, Unigene4096\_Sample\_011046840, Unigene42765\_Sample\_011046840, Unigene22976\_Sample\_011046840, Unigene42719\_Sample\_011046840, Unigene38841\_Sample\_011046840, Unigene43206\_Sample\_011046840, Unigene31611\_Sample\_011046840, Unigene15960\_Sample\_011046840, Unigene34565\_Sample\_011046840, Unigene37597\_Sample\_011046840, Unigene34288\_Sample\_011046840, Unigene14463\_Sample\_011046840, Unigene37869\_Sample\_011046840, Unigene31198\_Sample\_011046840, Unigene43301\_Sample\_011046840, Unigene26124\_Sample\_011046840, Unigene31457\_Sample\_011046840, Unigene28065\_Sample\_011046840, Unigene4051\_Sample\_011046840, Unigene16413\_Sample\_011046840, Unigene2245\_Sample\_011046840, Unigene32607\_Sample\_011046840, Unigene31196\_Sample\_011046840, Unigene33061\_Sample\_011046840, Unigene42960\_Sample\_011046840, Unigene37048\_Sample\_011046840, Unigene5122\_Sample\_011046840, Unigene2956\_Sample\_011046840, Unigene5402\_Sample\_011046840, Unigene42730\_Sample\_011046840, Unigene31482\_Sample\_011046840, Unigene13104\_Sample\_011046840, Unigene43405\_Sample\_011046840, Unigene1715\_Sample\_011046840, Unigene3917\_Sample\_011046840, Unigene43010\_Sample\_011046840, Unigene36223\_Sample\_011046840, Unigene16228\_Sample\_011046840, Unigene1872\_Sample\_011046840, Unigene42888\_Sample\_011046840, Unigene41520\_Sample\_011046840, Unigene41618\_Sample\_011046840, Unigene8198\_Sample\_011046840, Unigene35429\_Sample\_011046840, Unigene43408\_Sample\_011046840, Unigene36952\_Sample\_011046840, Unigene40235\_Sample\_011046840, Unigene42043\_Sample\_011046840, Unigene36018\_Sample\_011046840, Unigene20869\_Sample\_011046840, Unigene24891\_Sample\_011046840, Unigene39791\_Sample\_011046840, Unigene41383\_Sample\_011046840, Unigene41319\_Sample\_011046840, Unigene39843\_Sample\_011046840, Unigene29507\_Sample\_011046840, Unigene41589\_Sample\_011046840, Unigene37486\_Sample\_011046840, Unigene43599\_Sample\_011046840, Unigene4932\_Sample\_011046840, Unigene43289\_Sample\_011046840, Unigene31376\_Sample\_011046840, Unigene42385\_Sample\_011046840, Unigene42343\_Sample\_011046840, Unigene28459\_Sample\_011046840, Unigene34268\_Sample\_011046840, Unigene36568\_Sample\_011046840, Unigene15290\_Sample\_011046840, Unigene28153\_Sample\_011046840, Unigene33295\_Sample\_011046840, Unigene38259\_Sample\_011046840, Unigene2407\_Sample\_011046840, Unigene23318\_Sample\_011046840, Unigene42219\_Sample\_011046840, Unigene42552\_Sample\_011046840, Unigene1962\_Sample\_011046840, Unigene42130\_Sample\_011046840, Unigene7388\_Sample\_011046840, Unigene36762\_Sample\_011046840, Unigene36732\_Sample\_011046840, Unigene23400\_Sample\_011046840, Unigene6827\_Sample\_011046840, Unigene24482\_Sample\_011046840, Unigene5166\_Sample\_011046840, Unigene7998\_Sample\_011046840, Unigene20196\_Sample\_011046840, Unigene14589\_Sample\_011046840, Unigene31615\_Sample\_011046840, Unigene32130\_Sample\_011046840, Unigene5229\_Sample\_011046840, Unigene40547\_Sample\_011046840, Unigene40113\_Sample\_011046840, Unigene18228\_Sample\_011046840, Unigene16569\_Sample\_011046840, Unigene26583\_Sample\_011046840, Unigene43269\_Sample\_011046840, Unigene28693\_Sample\_011046840, Unigene40259\_Sample\_011046840, Unigene5336\_Sample\_011046840, Unigene27061\_Sample\_011046840, Unigene40153\_Sample\_011046840, Unigene2711\_Sample\_011046840, Unigene37308\_Sample\_011046840, Unigene5223\_Sample\_011046840, Unigene31423\_Sample\_011046840, Unigene20777\_Sample\_011046840, Unigene27109\_Sample\_011046840, Unigene38659\_Sample\_011046840, Unigene39734\_Sample\_011046840, Unigene26668\_Sample\_011046840, Unigene35544\_Sample\_011046840, Unigene29542\_Sample\_011046840, Unigene27684\_Sample\_011046840, Unigene16719\_Sample\_011046840, Unigene37246\_Sample\_011046840, Unigene32555\_Sample\_011046840, Unigene13188\_Sample\_011046840, Unigene1727\_Sample\_011046840, Unigene7677\_Sample\_011046840, Unigene17573\_Sample\_011046840, Unigene30267\_Sample\_011046840, Unigene27624\_Sample\_011046840, Unigene23983\_Sample\_011046840, Unigene28307\_Sample\_011046840, Unigene32732\_Sample\_011046840, Unigene26614\_Sample\_011046840, Unigene28263\_Sample\_011046840, Unigene30865\_Sample\_011046840, Unigene38430\_Sample\_011046840, Unigene7603\_Sample\_011046840, Unigene7893\_Sample\_011046840, Unigene43043\_Sample\_011046840, Unigene41917\_Sample\_011046840, Unigene37954\_Sample\_011046840, Unigene33126\_Sample\_011046840, Unigene23331\_Sample\_011046840, Unigene5793\_Sample\_011046840, Unigene41758\_Sample\_011046840, Unigene35052\_Sample\_011046840, Unigene36661\_Sample\_011046840, Unigene32566\_Sample\_011046840, Unigene40495\_Sample\_011046840, Unigene41781\_Sample\_011046840, Unigene36494\_Sample\_011046840, Unigene34566\_Sample\_011046840, Unigene38423\_Sample\_011046840, Unigene38491\_Sample\_011046840, Unigene43177\_Sample\_011046840, Unigene42533\_Sample\_011046840, Unigene26402\_Sample\_011046840, Unigene6648\_Sample\_011046840, Unigene38285\_Sample\_011046840, Unigene34595\_Sample\_011046840, Unigene4139\_Sample\_011046840, Unigene7120\_Sample\_011046840, Unigene36082\_Sample\_011046840, Unigene5876\_Sample\_011046840, Unigene34598\_Sample\_011046840, Unigene31827\_Sample\_011046840, Unigene27174\_Sample\_011046840, Unigene41534\_Sample\_011046840, Unigene31805\_Sample\_011046840, Unigene43272\_Sample\_011046840, Unigene42565\_Sample\_011046840, Unigene7508\_Sample\_011046840, Unigene33964\_Sample\_011046840, Unigene18251\_Sample\_011046840, Unigene43109\_Sample\_011046840, Unigene43029\_Sample\_011046840, Unigene35796\_Sample\_011046840, Unigene42487\_Sample\_011046840, Unigene41545\_Sample\_011046840, Unigene31980\_Sample\_011046840, Unigene42766\_Sample\_011046840, Unigene19340\_Sample\_011046840, Unigene28456\_Sample\_011046840, Unigene31283\_Sample\_011046840, Unigene43396\_Sample\_011046840, Unigene41983\_Sample\_011046840, Unigene5300\_Sample\_011046840, Unigene2443\_Sample\_011046840, Unigene4067\_Sample\_011046840, Unigene38782\_Sample\_011046840, Unigene28754\_Sample\_011046840, Unigene33408\_Sample\_011046840, Unigene38526\_Sample\_011046840, Unigene35976\_Sample\_011046840, Unigene43357\_Sample\_011046840, Unigene1504\_Sample\_011046840, Unigene30293\_Sample\_011046840, Unigene3201\_Sample\_011046840, Unigene39941\_Sample\_011046840, Unigene40922\_Sample\_011046840, Unigene34408\_Sample\_011046840, Unigene7927\_Sample\_011046840, Unigene35511\_Sample\_011046840, Unigene6414\_Sample\_011046840, Unigene6200\_Sample\_011046840, Unigene12175\_Sample\_011046840, Unigene34977\_Sample\_011046840, Unigene43308\_Sample\_011046840, Unigene38713\_Sample\_011046840, Unigene20234\_Sample\_011046840, Unigene40555\_Sample\_011046840, Unigene31953\_Sample\_011046840, Unigene36094\_Sample\_011046840, Unigene12410\_Sample\_011046840, Unigene36520\_Sample\_011046840, Unigene16879\_Sample\_011046840, Unigene30821\_Sample\_011046840, Unigene27469\_Sample\_011046840, Unigene2418\_Sample\_011046840, Unigene20967\_Sample\_011046840, Unigene12669\_Sample\_011046840, Unigene5754\_Sample\_011046840, Unigene16402\_Sample\_011046840, Unigene35362\_Sample\_011046840, Unigene28036\_Sample\_011046840, Unigene41234\_Sample\_011046840, Unigene41017\_Sample\_011046840, Unigene13348\_Sample\_011046840, Unigene25658\_Sample\_011046840, Unigene13877\_Sample\_011046840, Unigene35479\_Sample\_011046840, Unigene31027\_Sample\_011046840, Unigene27677\_Sample\_011046840, Unigene13641\_Sample\_011046840, Unigene36915\_Sample\_011046840, Unigene1533\_Sample\_011046840, Unigene40369\_Sample\_011046840, Unigene26417\_Sample\_011046840, Unigene27738\_Sample\_011046840, Unigene8189\_Sample\_011046840, Unigene35857\_Sample\_011046840, Unigene40070\_Sample\_011046840, Unigene40099\_Sample\_011046840, Unigene36449\_Sample\_011046840, Unigene18217\_Sample\_011046840, Unigene43183\_Sample\_011046840, Unigene33172\_Sample\_011046840, Unigene28488\_Sample\_011046840, Unigene3344\_Sample\_011046840, Unigene33585\_Sample\_011046840, Unigene3795\_Sample\_011046840, Unigene5202\_Sample\_011046840, Unigene7452\_Sample\_011046840, Unigene5628\_Sample\_011046840, Unigene33865\_Sample\_011046840, Unigene35111\_Sample\_011046840, Unigene41523\_Sample\_011046840, Unigene38614\_Sample\_011046840, Unigene39017\_Sample\_011046840, Unigene37647\_Sample\_011046840, Unigene36640\_Sample\_011046840, Unigene322\_Sample\_011046840, Unigene25369\_Sample\_011046840, Unigene34638\_Sample\_011046840, Unigene42866\_Sample\_011046840, Unigene38454\_Sample\_011046840, Unigene37012\_Sample\_011046840, Unigene37921\_Sample\_011046840, Unigene32413\_Sample\_011046840, Unigene37833\_Sample\_011046840, Unigene41513\_Sample\_011046840, Unigene823\_Sample\_011046840, Unigene41883\_Sample\_011046840, Unigene33629\_Sample\_011046840, Unigene42613\_Sample\_011046840, Unigene38409\_Sample\_011046840, Unigene24573\_Sample\_011046840, Unigene334\_Sample\_011046840, Unigene27885\_Sample\_011046840, Unigene169\_Sample\_011046840, Unigene7638\_Sample\_011046840, Unigene30985\_Sample\_011046840, Unigene11142\_Sample\_011046840, Unigene32307\_Sample\_011046840, Unigene4515\_Sample\_011046840, Unigene42027\_Sample\_011046840, Unigene38902\_Sample\_011046840, Unigene42139\_Sample\_011046840, Unigene30430\_Sample\_011046840, Unigene20146\_Sample\_011046840, Unigene41723\_Sample\_011046840, Unigene43479\_Sample\_011046840, Unigene42022\_Sample\_011046840, Unigene31621\_Sample\_011046840, Unigene35439\_Sample\_011046840, Unigene43096\_Sample\_011046840, Unigene7037\_Sample\_011046840, Unigene37781\_Sample\_011046840, Unigene26597\_Sample\_011046840, Unigene43232\_Sample\_011046840, Unigene31282\_Sample\_011046840, Unigene27470\_Sample\_011046840, Unigene33108\_Sample\_011046840, Unigene31886\_Sample\_011046840, Unigene37216\_Sample\_011046840, Unigene2310\_Sample\_011046840, Unigene40591\_Sample\_011046840, Unigene42497\_Sample\_011046840, Unigene13404\_Sample\_011046840, Unigene43151\_Sample\_011046840, Unigene40314\_Sample\_011046840, Unigene24899\_Sample\_011046840, Unigene40998\_Sample\_011046840, Unigene31286\_Sample\_011046840, Unigene41415\_Sample\_011046840, Unigene20609\_Sample\_011046840, Unigene22925\_Sample\_011046840 |
[truncated: 6,481,027 more chars]
