# Supplementary material for: Identification of Dirofilaria immitis miRNA using illumina deep sequencing
Source: Vet Res. 2013 Jan 18;44(1):3. doi: 10.1186/1297-9716-44-3 (PMC3598945; doi:10.1186/1297-9716-44-3)
Supplement: Additional file 9 — KEGG pathway of predicted target genes. 9 661 target genes were assigned to 250 KEGG pathways. “Target genes with pathway annotation” represents number and frequency of target genes related to this pathway. “All genes of the species with pathway annotation” represents number and frequency of reference genes related to this pathway. “Pvalue” and “Qvalue” represent P-value before correction and corrected P-value, respectively. [file 1297-9716-44-3-S9.zip › index.htm/Additonal file 9. KEGG pathway of predicted target genes.htm]

Dirofilaria\_immites

Pathway annotation of Sample Dirofilaria\_immites

| # | Pathway | Sample1 (9661) | Sample2 (11207) | Pvalue | Qvalue | Pathway ID |
| 1 | T cell receptor signaling pathway | 111 | 118 | 0.005295687 | 0.6669394 | ko04660 |
| 2 | Cell adhesion molecules (CAMs) | 68 | 71 | 0.00806796 | 0.6669394 | ko04514 |
| 3 | O-Glycan biosynthesis | 29 | 29 | 0.01342537 | 0.6669394 | ko00512 |
| 4 | Homologous recombination | 63 | 66 | 0.0139234 | 0.6669394 | ko03440 |
| 5 | MAPK signaling pathway - yeast | 41 | 42 | 0.01500737 | 0.6669394 | ko04011 |
| 6 | B cell receptor signaling pathway | 80 | 85 | 0.01718720 | 0.6669394 | ko04662 |
| 7 | Arginine and proline metabolism | 59 | 62 | 0.02132525 | 0.6669394 | ko00330 |
| 8 | Apoptosis | 49 | 51 | 0.02134206 | 0.6669394 | ko04210 |
| 9 | Natural killer cell mediated cytotoxicity | 84 | 90 | 0.02690684 | 0.7474122 | ko04650 |
| 10 | Hypertrophic cardiomyopathy (HCM) | 123 | 134 | 0.03296263 | 0.7816032 | ko05410 |
| 11 | Colorectal cancer | 98 | 106 | 0.03439054 | 0.7816032 | ko05210 |
| 12 | Axon guidance | 168 | 186 | 0.05757755 | 0.9994000 | ko04360 |
| 13 | Lysosome | 203 | 226 | 0.06317156 | 0.9994000 | ko04142 |
| 14 | Neurotrophin signaling pathway | 150 | 166 | 0.06856641 | 0.9994000 | ko04722 |
| 15 | Maturity onset diabetes of the young | 18 | 18 | 0.06896662 | 0.9994000 | ko04950 |
| 16 | Toll-like receptor signaling pathway | 72 | 78 | 0.07297783 | 0.9994000 | ko04620 |
| 17 | Basal cell carcinoma | 72 | 78 | 0.07297783 | 0.9994000 | ko05217 |
| 18 | Leishmaniasis | 28 | 29 | 0.07590985 | 0.9994000 | ko05140 |
| 19 | Pantothenate and CoA biosynthesis | 17 | 17 | 0.08002244 | 0.9994000 | ko00770 |
| 20 | Progesterone-mediated oocyte maturation | 154 | 171 | 0.08263875 | 0.9994000 | ko04914 |
| 21 | Renal cell carcinoma | 109 | 120 | 0.08427856 | 0.9994000 | ko05211 |
| 22 | Pathways in cancer | 377 | 426 | 0.08995473 | 0.9994000 | ko05200 |
| 23 | Endometrial cancer | 99 | 109 | 0.09825934 | 0.9994000 | ko05213 |
| 24 | Adipocytokine signaling pathway | 83 | 91 | 0.1034100 | 0.9994000 | ko04920 |
| 25 | Plant-pathogen interaction | 42 | 45 | 0.1146856 | 0.9994000 | ko04626 |
| 26 | p53 signaling pathway | 50 | 54 | 0.1167355 | 0.9994000 | ko04115 |
| 27 | Phosphatidylinositol signaling system | 140 | 156 | 0.1176768 | 0.9994000 | ko04070 |
| 28 | Drug metabolism - other enzymes | 41 | 44 | 0.1255888 | 0.9994000 | ko00983 |
| 29 | NOD-like receptor signaling pathway | 41 | 44 | 0.1255888 | 0.9994000 | ko04621 |
| 30 | Amyotrophic lateral sclerosis (ALS) | 40 | 43 | 0.1373771 | 0.9994000 | ko05014 |
| 31 | Neuroactive ligand-receptor interaction | 62 | 68 | 0.1538322 | 0.9994000 | ko04080 |
| 32 | Notch signaling pathway | 84 | 93 | 0.1568344 | 0.9994000 | ko04330 |
| 33 | Hematopoietic cell lineage | 38 | 41 | 0.1638077 | 0.9994000 | ko04640 |
| 34 | Glioma | 83 | 92 | 0.1665054 | 0.9994000 | ko05214 |
| 35 | Epithelial cell signaling in Helicobacter pylori infection | 97 | 108 | 0.1708083 | 0.9994000 | ko05120 |
| 36 | Base excision repair | 60 | 66 | 0.1766284 | 0.9994000 | ko03410 |
| 37 | Complement and coagulation cascades | 37 | 40 | 0.1785477 | 0.9994000 | ko04610 |
| 38 | ErbB signaling pathway | 138 | 155 | 0.1823644 | 0.9994000 | ko04012 |
| 39 | Adherens junction | 117 | 131 | 0.1825601 | 0.9994000 | ko04520 |
| 40 | Alanine, aspartate and glutamate metabolism | 52 | 57 | 0.1832014 | 0.9994000 | ko00250 |
| 41 | Dorso-ventral axis formation | 74 | 82 | 0.1846967 | 0.9994000 | ko04320 |
| 42 | Insulin signaling pathway | 247 | 280 | 0.1851137 | 0.9994000 | ko04910 |
| 43 | RNA degradation | 144 | 162 | 0.1901014 | 0.9994000 | ko03018 |
| 44 | Inositol phosphate metabolism | 102 | 114 | 0.1910668 | 0.9994000 | ko00562 |
| 45 | Cell cycle | 239 | 271 | 0.1931796 | 0.9994000 | ko04110 |
| 46 | Riboflavin metabolism | 42 | 46 | 0.2204018 | 0.9994000 | ko00740 |
| 47 | Histidine metabolism | 18 | 19 | 0.2404925 | 0.9994000 | ko00340 |
| 48 | Chronic myeloid leukemia | 97 | 109 | 0.2447602 | 0.9994000 | ko05220 |
| 49 | Hedgehog signaling pathway | 76 | 85 | 0.2472765 | 0.9994000 | ko04340 |
| 50 | Non-homologous end-joining | 33 | 36 | 0.2487104 | 0.9994000 | ko03450 |
| 51 | Dilated cardiomyopathy | 117 | 132 | 0.2508708 | 0.9994000 | ko05414 |
| 52 | Prostate cancer | 103 | 116 | 0.2548222 | 0.9994000 | ko05215 |
| 53 | Protein export | 47 | 52 | 0.2589316 | 0.9994000 | ko03060 |
| 54 | MAPK signaling pathway | 256 | 292 | 0.2615167 | 0.9994000 | ko04010 |
| 55 | Glycosaminoglycan biosynthesis - keratan sulfate | 9 | 9 | 0.2627671 | 0.9994000 | ko00533 |
| 56 | Long-term potentiation | 95 | 107 | 0.2688522 | 0.9994000 | ko04720 |
| 57 | Selenoamino acid metabolism | 39 | 43 | 0.2741507 | 0.9994000 | ko00450 |
| 58 | Cytokine-cytokine receptor interaction | 39 | 43 | 0.2741507 | 0.9994000 | ko04060 |
| 59 | gamma-Hexachlorocyclohexane degradation | 24 | 26 | 0.2838973 | 0.9994000 | ko00361 |
| 60 | ABC transporters | 73 | 82 | 0.2891102 | 0.9994000 | ko02010 |
| 61 | Taurine and hypotaurine metabolism | 8 | 8 | 0.3048512 | 0.9994000 | ko00430 |
| 62 | Regulation of actin cytoskeleton | 270 | 309 | 0.3054073 | 0.9994000 | ko04810 |
| 63 | Arrhythmogenic right ventricular cardiomyopathy (ARVC) | 65 | 73 | 0.3070053 | 0.9994000 | ko05412 |
| 64 | Acute myeloid leukemia | 58 | 65 | 0.3097795 | 0.9994000 | ko05221 |
| 65 | Tight junction | 243 | 278 | 0.3132917 | 0.9994000 | ko04530 |
| 66 | Cytosolic DNA-sensing pathway | 30 | 33 | 0.3140315 | 0.9994000 | ko04623 |
| 67 | Carbon fixation in photosynthetic organisms | 37 | 41 | 0.3148933 | 0.9994000 | ko00710 |
| 68 | Lysine degradation | 91 | 103 | 0.3214111 | 0.9994000 | ko00310 |
| 69 | Pancreatic cancer | 77 | 87 | 0.3304892 | 0.9994000 | ko05212 |
| 70 | Chagas disease | 82 | 93 | 0.3555955 | 0.9994000 | ko05142 |
| 71 | Fc epsilon RI signaling pathway | 75 | 85 | 0.361707 | 0.9994000 | ko04664 |
| 72 | Cell cycle - yeast | 231 | 265 | 0.3621982 | 0.9994000 | ko04111 |
| 73 | Melanoma | 55 | 62 | 0.3633031 | 0.9994000 | ko05218 |
| 74 | Tyrosine metabolism | 55 | 62 | 0.3633031 | 0.9994000 | ko00350 |
| 75 | Glycosaminoglycan biosynthesis - chondroitin sulfate | 21 | 23 | 0.3669514 | 0.9994000 | ko00532 |
| 76 | Pyruvate metabolism | 68 | 77 | 0.3681477 | 0.9994000 | ko00620 |
| 77 | Basal transcription factors | 81 | 92 | 0.3709694 | 0.9994000 | ko03022 |
| 78 | Starch and sucrose metabolism | 81 | 92 | 0.3709694 | 0.9994000 | ko00500 |
| 79 | Wnt signaling pathway | 204 | 234 | 0.3740558 | 0.9994000 | ko04310 |
| 80 | Leukocyte transendothelial migration | 152 | 174 | 0.378453 | 0.9994000 | ko04670 |
| 81 | Valine, leucine and isoleucine biosynthesis | 34 | 38 | 0.3832869 | 0.9994000 | ko00290 |
| 82 | Tropane, piperidine and pyridine alkaloid biosynthesis | 13 | 14 | 0.4054051 | 0.9994000 | ko00960 |
| 83 | Ascorbate and aldarate metabolism | 6 | 6 | 0.4103016 | 0.9994000 | ko00053 |
| 84 | Olfactory transduction | 26 | 29 | 0.4185283 | 0.9994000 | ko04740 |
| 85 | Aminoacyl-tRNA biosynthesis | 129 | 148 | 0.4236295 | 0.9994000 | ko00970 |
| 86 | Glycolysis / Gluconeogenesis | 90 | 103 | 0.4324493 | 0.9994000 | ko00010 |
| 87 | MAPK signaling pathway - fly | 51 | 58 | 0.4420013 | 0.9994000 | ko04013 |
| 88 | Fatty acid biosynthesis | 25 | 28 | 0.4475755 | 0.9994000 | ko00061 |
| 89 | Phototransduction | 25 | 28 | 0.4475755 | 0.9994000 | ko04744 |
| 90 | Meiosis - yeast | 177 | 204 | 0.4570679 | 0.9994000 | ko04113 |
| 91 | Porphyrin and chlorophyll metabolism | 31 | 35 | 0.4597208 | 0.9994000 | ko00860 |
| 92 | Melanogenesis | 107 | 123 | 0.4633607 | 0.9994000 | ko04916 |
| 93 | Pathogenic Escherichia coli infection | 107 | 123 | 0.4633607 | 0.9994000 | ko05130 |
| 94 | Bladder cancer | 37 | 42 | 0.4692913 | 0.9994000 | ko05219 |
| 95 | Mismatch repair | 56 | 64 | 0.4698276 | 0.9994000 | ko03430 |
| 96 | 2,4-Dichlorobenzoate degradation | 5 | 5 | 0.475994 | 0.9994000 | ko00623 |
| 97 | Biosynthesis of vancomycin group antibiotics | 5 | 5 | 0.475994 | 0.9994000 | ko01055 |
| 98 | Viral myocarditis | 118 | 136 | 0.4857673 | 0.9994000 | ko05416 |
| 99 | Cyanoamino acid metabolism | 11 | 12 | 0.49174 | 0.9994000 | ko00460 |
| 100 | Naphthalene and anthracene degradation | 17 | 19 | 0.501543 | 0.9994000 | ko00626 |
| 101 | TGF-beta signaling pathway | 85 | 98 | 0.5118213 | 0.9994000 | ko04350 |
| 102 | Glycosaminoglycan biosynthesis - heparan sulfate | 29 | 33 | 0.5143817 | 0.9994000 | ko00534 |
| 103 | Renin-angiotensin system | 29 | 33 | 0.5143817 | 0.9994000 | ko04614 |
| 104 | Thyroid cancer | 35 | 40 | 0.5191885 | 0.9994000 | ko05216 |
| 105 | Ether lipid metabolism | 35 | 40 | 0.5191885 | 0.9994000 | ko00565 |
| 106 | mTOR signaling pathway | 96 | 111 | 0.5337555 | 0.9994000 | ko04150 |
| 107 | N-Glycan biosynthesis | 108 | 125 | 0.5388822 | 0.9994000 | ko00510 |
| 108 | ECM-receptor interaction | 108 | 125 | 0.5388822 | 0.9994000 | ko04512 |
| 109 | Thiamine metabolism | 16 | 18 | 0.5389338 | 0.9994000 | ko00730 |
| 110 | Bacterial secretion system | 10 | 11 | 0.5392094 | 0.9994000 | ko03070 |
| 111 | Citrate cycle (TCA cycle) | 83 | 96 | 0.5444229 | 0.9994000 | ko00020 |
| 112 | Glycine, serine and threonine metabolism | 34 | 39 | 0.544743 | 0.9994000 | ko00260 |
| 113 | Spliceosome | 386 | 448 | 0.5450071 | 0.9994000 | ko03040 |
| 114 | Chemokine signaling pathway | 163 | 189 | 0.5460094 | 0.9994000 | ko04062 |
| 115 | Amino sugar and nucleotide sugar metabolism | 95 | 110 | 0.5490176 | 0.9994000 | ko00520 |
| 116 | Glycosylphosphatidylinositol(GPI)-anchor biosynthesis | 46 | 53 | 0.5490779 | 0.9994000 | ko00563 |
| 117 | Zeatin biosynthesis | 4 | 4 | 0.5521965 | 0.9994000 | ko00908 |
| 118 | Flavone and flavonol biosynthesis | 4 | 4 | 0.5521965 | 0.9994000 | ko00944 |
| 119 | 3-Chloroacrylic acid degradation | 4 | 4 | 0.5521965 | 0.9994000 | ko00641 |
| 120 | GnRH signaling pathway | 106 | 123 | 0.567639 | 0.9994000 | ko04912 |
| 121 | Sphingolipid metabolism | 57 | 66 | 0.5728121 | 0.9994000 | ko00600 |
| 122 | Glyoxylate and dicarboxylate metabolism | 15 | 17 | 0.5774866 | 0.9994000 | ko00630 |
| 123 | Oocyte meiosis | 196 | 228 | 0.5888987 | 0.9994000 | ko04114 |
| 124 | Two-component system | 9 | 10 | 0.5892666 | 0.9994000 | ko02020 |
| 125 | Fc gamma R-mediated phagocytosis | 110 | 128 | 0.5971782 | 0.9994000 | ko04666 |
| 126 | Collecting duct acid secretion | 43 | 50 | 0.615527 | 0.9994000 | ko04966 |
| 127 | Autoimmune thyroid disease | 14 | 16 | 0.6169448 | 0.9994000 | ko05320 |
| 128 | Arachidonic acid metabolism | 31 | 36 | 0.6227286 | 0.9994000 | ko00590 |
| 129 | Vasopressin-regulated water reabsorption | 84 | 98 | 0.625671 | 0.9994000 | ko04962 |
| 130 | Non-small cell lung cancer | 78 | 91 | 0.6262561 | 0.9994000 | ko05223 |
| 131 | Glycerolipid metabolism | 60 | 70 | 0.6296639 | 0.9994000 | ko00561 |
| 132 | Antigen processing and presentation | 42 | 49 | 0.6376597 | 0.9994000 | ko04612 |
| 133 | Benzoate degradation via hydroxylation | 3 | 3 | 0.6405891 | 0.9994000 | ko00362 |
| 134 | Atrazine degradation | 3 | 3 | 0.6405891 | 0.9994000 | ko00791 |
| 135 | Biotin metabolism | 3 | 3 | 0.6405891 | 0.9994000 | ko00780 |
| 136 | Sulfur metabolism | 8 | 9 | 0.6415242 | 0.9994000 | ko00920 |
| 137 | Prion diseases | 30 | 35 | 0.6488105 | 0.9994000 | ko05020 |
| 138 | Pentose phosphate pathway | 53 | 62 | 0.6510626 | 0.9994000 | ko00030 |
| 139 | Bacterial invasion of epithelial cells | 141 | 165 | 0.6618926 | 0.9994000 | ko05100 |
| 140 | RIG-I-like receptor signaling pathway | 35 | 41 | 0.6660577 | 0.9994000 | ko04622 |
| 141 | Vibrio cholerae infection | 93 | 109 | 0.6684914 | 0.9994000 | ko05110 |
| 142 | Calcium signaling pathway | 158 | 185 | 0.6721601 | 0.9994000 | ko04020 |
| 143 | alpha-Linolenic acid metabolism | 18 | 21 | 0.672565 | 0.9994000 | ko00592 |
| 144 | Fatty acid elongation in mitochondria | 18 | 21 | 0.672565 | 0.9994000 | ko00062 |
| 145 | Jak-STAT signaling pathway | 69 | 81 | 0.6772133 | 0.9994000 | ko04630 |
| 146 | Endocytosis | 282 | 330 | 0.6901688 | 0.9994000 | ko04144 |
| 147 | Tetrachloroethene degradation | 7 | 8 | 0.6954044 | 0.9994000 | ko00625 |
| 148 | Glycosaminoglycan degradation | 12 | 14 | 0.6972505 | 0.9994000 | ko00531 |
| 149 | Phosphonate and phosphinate metabolism | 12 | 14 | 0.6972505 | 0.9994000 | ko00440 |
| 150 | Phenylpropanoid biosynthesis | 12 | 14 | 0.6972505 | 0.9994000 | ko00940 |
| 151 | VEGF signaling pathway | 85 | 100 | 0.6993119 | 0.9994000 | ko04370 |
| 152 | Aldosterone-regulated sodium reabsorption | 56 | 66 | 0.7023905 | 0.9994000 | ko04960 |
| 153 | Pyrimidine metabolism | 160 | 188 | 0.713785 | 0.9994000 | ko00240 |
| 154 | Type II diabetes mellitus | 44 | 52 | 0.7152869 | 0.9994000 | ko04930 |
| 155 | Nicotinate and nicotinamide metabolism | 22 | 26 | 0.7155838 | 0.9994000 | ko00760 |
| 156 | Purine metabolism | 229 | 269 | 0.732335 | 0.9994000 | ko00230 |
| 157 | Primary immunodeficiency | 11 | 13 | 0.737275 | 0.9994000 | ko05340 |
| 158 | Nucleotide excision repair | 111 | 131 | 0.7380636 | 0.9994000 | ko03420 |
| 159 | Betalain biosynthesis | 16 | 19 | 0.7383515 | 0.9994000 | ko00965 |
| 160 | Flavonoid biosynthesis | 2 | 2 | 0.7431205 | 0.9994000 | ko00941 |
| 161 | Styrene degradation | 2 | 2 | 0.7431205 | 0.9994000 | ko00643 |
| 162 | Lysine biosynthesis | 2 | 2 | 0.7431205 | 0.9994000 | ko00300 |
| 163 | Circadian rhythm - plant | 2 | 2 | 0.7431205 | 0.9994000 | ko04712 |
| 164 | Pentose and glucuronate interconversions | 21 | 25 | 0.743588 | 0.9994000 | ko00040 |
| 165 | Phenylalanine metabolism | 21 | 25 | 0.743588 | 0.9994000 | ko00360 |
| 166 | Long-term depression | 76 | 90 | 0.7457761 | 0.9994000 | ko04730 |
| 167 | Indole alkaloid biosynthesis | 6 | 7 | 0.7500889 | 0.9994000 | ko00901 |
| 168 | Type I diabetes mellitus | 6 | 7 | 0.7500889 | 0.9994000 | ko04940 |
| 169 | Vascular smooth muscle contraction | 155 | 183 | 0.7631132 | 0.9994000 | ko04270 |
| 170 | One carbon pool by folate | 15 | 18 | 0.7702509 | 0.9994000 | ko00670 |
| 171 | Isoquinoline alkaloid biosynthesis | 20 | 24 | 0.7708715 | 0.9994000 | ko00950 |
| 172 | Butanoate metabolism | 41 | 49 | 0.772433 | 0.9994000 | ko00650 |
| 173 | DNA replication | 102 | 121 | 0.7759188 | 0.9994000 | ko03030 |
| 174 | Bisphenol A degradation | 10 | 12 | 0.7765565 | 0.9994000 | ko00363 |
| 175 | Focal adhesion | 365 | 429 | 0.7782326 | 0.9994000 | ko04510 |
| 176 | Glycerophospholipid metabolism | 96 | 114 | 0.7797196 | 0.9994000 | ko00564 |
| 177 | Ubiquinone and other terpenoid-quinone biosynthesis | 19 | 23 | 0.7972415 | 0.9994000 | ko00130 |
| 178 | Reductive carboxylate cycle (CO2 fixation) | 19 | 23 | 0.7972415 | 0.9994000 | ko00720 |
| 179 | Cysteine and methionine metabolism | 24 | 29 | 0.7976033 | 0.9994000 | ko00270 |
| 180 | Retinol metabolism | 24 | 29 | 0.7976033 | 0.9994000 | ko00830 |
| 181 | Stilbenoid, diarylheptanoid and gingerol biosynthesis | 5 | 6 | 0.8044563 | 0.9994000 | ko00945 |
| 182 | Biosynthesis of ansamycins | 5 | 6 | 0.8044563 | 0.9994000 | ko01051 |
| 183 | RNA polymerase | 55 | 66 | 0.8078721 | 0.9994000 | ko03020 |
| 184 | D-Glutamine and D-glutamate metabolism | 9 | 11 | 0.8145242 | 0.9994000 | ko00471 |
| 185 | Folate biosynthesis | 9 | 11 | 0.8145242 | 0.9994000 | ko00790 |
| 186 | Gap junction | 114 | 136 | 0.8263937 | 0.9994000 | ko04540 |
| 187 | Nitrogen metabolism | 27 | 33 | 0.8392427 | 0.9994000 | ko00910 |
| 188 | Peroxisome | 101 | 121 | 0.843821 | 0.9994000 | ko04146 |
| 189 | Linoleic acid metabolism | 17 | 21 | 0.846481 | 0.9994000 | ko00591 |
| 190 | Geraniol degradation | 8 | 10 | 0.8505542 | 0.9994000 | ko00281 |
| 191 | Asthma | 4 | 5 | 0.8570062 | 0.9994000 | ko05310 |
| 192 | Phenylalanine, tyrosine and tryptophan biosynthesis | 4 | 5 | 0.8570062 | 0.9994000 | ko00400 |
| 193 | Synthesis and degradation of ketone bodies | 4 | 5 | 0.8570062 | 0.9994000 | ko00072 |
| 194 | Vitamin B6 metabolism | 4 | 5 | 0.8570062 | 0.9994000 | ko00750 |
| 195 | Novobiocin biosynthesis | 4 | 5 | 0.8570062 | 0.9994000 | ko00401 |
| 196 | 1,2-Dichloroethane degradation | 1 | 1 | 0.8620505 | 0.9994000 | ko00631 |
| 197 | D-Arginine and D-ornithine metabolism | 1 | 1 | 0.8620505 | 0.9994000 | ko00472 |
| 198 | Intestinal immune network for IgA production | 1 | 1 | 0.8620505 | 0.9994000 | ko04672 |
| 199 | Penicillin and cephalosporin biosynthesis | 1 | 1 | 0.8620505 | 0.9994000 | ko00311 |
| 200 | Ubiquitin mediated proteolysis | 268 | 318 | 0.8624086 | 0.9994000 | ko04120 |
| 201 | Fatty acid metabolism | 56 | 68 | 0.863309 | 0.9994000 | ko00071 |
| 202 | Tryptophan metabolism | 45 | 55 | 0.8713885 | 0.9994000 | ko00380 |
| 203 | PPAR signaling pathway | 71 | 86 | 0.8715438 | 0.9994000 | ko03320 |
| 204 | Circadian rhythm - fly | 20 | 25 | 0.8800055 | 0.9994000 | ko04711 |
| 205 | Lipoic acid metabolism | 7 | 9 | 0.8839852 | 0.9994000 | ko00785 |
| 206 | Glycosphingolipid biosynthesis - lacto and neolacto series | 11 | 14 | 0.8840316 | 0.9994000 | ko00601 |
| 207 | Proximal tubule bicarbonate reclamation | 34 | 42 | 0.8842742 | 0.9994000 | ko04964 |
| 208 | Other glycan degradation | 24 | 30 | 0.8905114 | 0.9994000 | ko00511 |
| 209 | Proteasome | 95 | 115 | 0.8934512 | 0.9994000 | ko03050 |
| 210 | Valine, leucine and isoleucine degradation | 48 | 59 | 0.8946975 | 0.9994000 | ko00280 |
| 211 | SNARE interactions in vesicular transport | 23 | 29 | 0.9053567 | 0.9994000 | ko04130 |
| 212 | O-Mannosyl glycan biosynthesis | 3 | 4 | 0.9057669 | 0.9994000 | ko00514 |
| 213 | Circadian rhythm - mammal | 10 | 13 | 0.907495 | 0.9994000 | ko04710 |
| 214 | Primary bile acid biosynthesis | 10 | 13 | 0.907495 | 0.9994000 | ko00120 |
| 215 | Cell cycle - Caulobacter | 10 | 13 | 0.907495 | 0.9994000 | ko04112 |
| 216 | Fructose and mannose metabolism | 72 | 88 | 0.9081011 | 0.9994000 | ko00051 |
| 217 | Biosynthesis of unsaturated fatty acids | 32 | 40 | 0.909169 | 0.9994000 | ko01040 |
| 218 | Galactose metabolism | 56 | 69 | 0.913712 | 0.9994000 | ko00052 |
| 219 | Polyketide sugar unit biosynthesis | 6 | 8 | 0.9141425 | 0.9994000 | ko00523 |
| 220 | Steroid biosynthesis | 6 | 8 | 0.9141425 | 0.9994000 | ko00100 |
| 221 | Benzoate degradation via CoA ligation | 13 | 17 | 0.9262414 | 0.9994000 | ko00632 |
| 222 | Alzheimer's disease | 225 | 270 | 0.9269375 | 0.9994000 | ko05010 |
| 223 | Glutathione metabolism | 58 | 72 | 0.9357076 | 0.9994000 | ko00480 |
| 224 | Metabolic pathways | 1487 | 1748 | 0.9369044 | 0.9994000 | ko01100 |
| 225 | Small cell lung cancer | 88 | 108 | 0.9371073 | 0.9994000 | ko05222 |
| 226 | Propanoate metabolism | 48 | 60 | 0.937411 | 0.9994000 | ko00640 |
| 227 | Caprolactam degradation | 12 | 16 | 0.9415367 | 0.9994000 | ko00930 |
| 228 | 1- and 2-Methylnaphthalene degradation | 8 | 11 | 0.9466342 | 0.9994000 | ko00624 |
| 229 | Regulation of autophagy | 32 | 41 | 0.9519007 | 0.9994000 | ko04140 |
| 230 | Drug metabolism - cytochrome P450 | 15 | 20 | 0.9525247 | 0.9994000 | ko00982 |
| 231 | Metabolism of xenobiotics by cytochrome P450 | 11 | 15 | 0.95484 | 0.9994000 | ko00980 |
| 232 | Steroid hormone biosynthesis | 11 | 15 | 0.95484 | 0.9994000 | ko00140 |
| 233 | Biosynthesis of secondary metabolites | 365 | 437 | 0.9554791 | 0.9994000 | ko01110 |
| 234 | beta-Alanine metabolism | 22 | 29 | 0.9619219 | 0.9994000 | ko00410 |
| 235 | Glycosphingolipid biosynthesis - ganglio series | 7 | 10 | 0.9619907 | 0.9994000 | ko00604 |
| 236 | Butirosin and neomycin biosynthesis | 7 | 10 | 0.9619907 | 0.9994000 | ko00524 |
| 237 | High-mannose type N-glycan biosynthesis | 4 | 6 | 0.962106 | 0.9994000 | ko00513 |
| 238 | Taste transduction | 21 | 28 | 0.9687726 | 0.9994000 | ko04742 |
| 239 | Ribosome | 75 | 94 | 0.9700484 | 0.9994000 | ko03010 |
| 240 | Limonene and pinene degradation | 13 | 18 | 0.9710838 | 0.9994000 | ko00903 |
| 241 | Ethylbenzene degradation | 1 | 2 | 0.9809805 | 0.9994000 | ko00642 |
| 242 | Streptomycin biosynthesis | 22 | 30 | 0.9833388 | 0.9994000 | ko00521 |
| 243 | Glycosphingolipid biosynthesis - globo series | 5 | 8 | 0.9840952 | 0.9994000 | ko00603 |
| 244 | Cardiac muscle contraction | 60 | 77 | 0.984397 | 0.9994000 | ko04260 |
| 245 | Methane metabolism | 14 | 20 | 0.9858841 | 0.9994000 | ko00680 |
| 246 | Oxidative phosphorylation | 175 | 217 | 0.9917358 | 0.9994000 | ko00190 |
| 247 | Terpenoid backbone biosynthesis | 22 | 31 | 0.9931623 | 0.9994000 | ko00900 |
| 248 | Systemic lupus erythematosus | 27 | 38 | 0.996154 | 0.9994000 | ko05322 |
| 249 | Parkinson's disease | 165 | 207 | 0.9965422 | 0.9994000 | ko05012 |
| 250 | Huntington's disease | 259 | 324 | 0.9994 | 0.9994000 | ko05016 |

| # | Pathway | Target genes involved in the pathway |
| --- | --- | --- |
| 1 | T cell receptor signaling pathway Back to summary table | Unigene2194\_Sample\_011046840, Unigene2690\_Sample\_011046840, Unigene4790\_Sample\_011046840, Unigene4993\_Sample\_011046840, Unigene8035\_Sample\_011046840, Unigene8046\_Sample\_011046840, Unigene15939\_Sample\_011046840, Unigene40260\_Sample\_011046840, Unigene40413\_Sample\_011046840, Unigene40471\_Sample\_011046840, Unigene41242\_Sample\_011046840, Unigene42127\_Sample\_011046840, Unigene42759\_Sample\_011046840, Unigene42766\_Sample\_011046840, Unigene42773\_Sample\_011046840, Unigene42900\_Sample\_011046840, Unigene43441\_Sample\_011046840, Unigene43496\_Sample\_011046840, Unigene1286\_Sample\_011046840, Unigene2986\_Sample\_011046840, Unigene3258\_Sample\_011046840, Unigene4779\_Sample\_011046840, Unigene6863\_Sample\_011046840, Unigene7868\_Sample\_011046840, Unigene10748\_Sample\_011046840, Unigene26575\_Sample\_011046840, Unigene32380\_Sample\_011046840, Unigene34085\_Sample\_011046840, Unigene34974\_Sample\_011046840, Unigene35122\_Sample\_011046840, Unigene38617\_Sample\_011046840, Unigene38659\_Sample\_011046840, Unigene38866\_Sample\_011046840, Unigene39843\_Sample\_011046840, Unigene40297\_Sample\_011046840, Unigene41370\_Sample\_011046840, Unigene41915\_Sample\_011046840, Unigene42164\_Sample\_011046840, Unigene42428\_Sample\_011046840, Unigene43301\_Sample\_011046840, Unigene43342\_Sample\_011046840, Unigene14932\_Sample\_011046840, Unigene34039\_Sample\_011046840, Unigene38847\_Sample\_011046840, Unigene43149\_Sample\_011046840, Unigene13789\_Sample\_011046840, Unigene29542\_Sample\_011046840, Unigene31699\_Sample\_011046840, Unigene4818\_Sample\_011046840, Unigene7408\_Sample\_011046840, Unigene13661\_Sample\_011046840, Unigene14485\_Sample\_011046840, Unigene20325\_Sample\_011046840, Unigene23045\_Sample\_011046840, Unigene28883\_Sample\_011046840, Unigene31079\_Sample\_011046840, Unigene35150\_Sample\_011046840, Unigene36320\_Sample\_011046840, Unigene36860\_Sample\_011046840, Unigene40335\_Sample\_011046840, Unigene40659\_Sample\_011046840, Unigene42079\_Sample\_011046840, Unigene42487\_Sample\_011046840, Unigene43068\_Sample\_011046840, Unigene43392\_Sample\_011046840, Unigene24869\_Sample\_011046840, Unigene28124\_Sample\_011046840, Unigene30141\_Sample\_011046840, Unigene33873\_Sample\_011046840, Unigene4139\_Sample\_011046840, Unigene18933\_Sample\_011046840, Unigene25548\_Sample\_011046840, Unigene26785\_Sample\_011046840, Unigene28751\_Sample\_011046840, Unigene29670\_Sample\_011046840, Unigene31705\_Sample\_011046840, Unigene31880\_Sample\_011046840, Unigene39503\_Sample\_011046840, Unigene40189\_Sample\_011046840, Unigene42008\_Sample\_011046840, Unigene43154\_Sample\_011046840, Unigene1368\_Sample\_011046840, Unigene6944\_Sample\_011046840, Unigene10749\_Sample\_011046840, Unigene17676\_Sample\_011046840, Unigene18009\_Sample\_011046840, Unigene19400\_Sample\_011046840, Unigene20896\_Sample\_011046840, Unigene29526\_Sample\_011046840, Unigene29660\_Sample\_011046840, Unigene32479\_Sample\_011046840, Unigene33870\_Sample\_011046840, Unigene38572\_Sample\_011046840, Unigene42811\_Sample\_011046840, Unigene5726\_Sample\_011046840, Unigene19127\_Sample\_011046840, Unigene21176\_Sample\_011046840, Unigene27623\_Sample\_011046840, Unigene31320\_Sample\_011046840, Unigene41560\_Sample\_011046840, Unigene10037\_Sample\_011046840, Unigene24184\_Sample\_011046840, Unigene32864\_Sample\_011046840, Unigene41704\_Sample\_011046840, Unigene4487\_Sample\_011046840, Unigene16035\_Sample\_011046840, Unigene32265\_Sample\_011046840, Unigene37491\_Sample\_011046840, Unigene41410\_Sample\_011046840, Unigene24889\_Sample\_011046840, Unigene31562\_Sample\_011046840 |
| 2 | Cell adhesion molecules (CAMs) Back to summary table | Unigene1287\_Sample\_011046840, Unigene1555\_Sample\_011046840, Unigene1982\_Sample\_011046840, Unigene7268\_Sample\_011046840, Unigene9987\_Sample\_011046840, Unigene25850\_Sample\_011046840, Unigene29477\_Sample\_011046840, Unigene31437\_Sample\_011046840, Unigene38712\_Sample\_011046840, Unigene40454\_Sample\_011046840, Unigene41507\_Sample\_011046840, Unigene42639\_Sample\_011046840, Unigene43141\_Sample\_011046840, Unigene43429\_Sample\_011046840, Unigene43553\_Sample\_011046840, Unigene1705\_Sample\_011046840, Unigene6185\_Sample\_011046840, Unigene7013\_Sample\_011046840, Unigene37715\_Sample\_011046840, Unigene39242\_Sample\_011046840, Unigene41221\_Sample\_011046840, Unigene41617\_Sample\_011046840, Unigene41853\_Sample\_011046840, Unigene41969\_Sample\_011046840, Unigene43535\_Sample\_011046840, Unigene4155\_Sample\_011046840, Unigene8133\_Sample\_011046840, Unigene25969\_Sample\_011046840, Unigene26548\_Sample\_011046840, Unigene28292\_Sample\_011046840, Unigene34302\_Sample\_011046840, Unigene42910\_Sample\_011046840, Unigene22181\_Sample\_011046840, Unigene28433\_Sample\_011046840, Unigene30719\_Sample\_011046840, Unigene43466\_Sample\_011046840, Unigene92\_Sample\_011046840, Unigene4823\_Sample\_011046840, Unigene5470\_Sample\_011046840, Unigene6516\_Sample\_011046840, Unigene7678\_Sample\_011046840, Unigene8173\_Sample\_011046840, Unigene31860\_Sample\_011046840, Unigene37257\_Sample\_011046840, Unigene39100\_Sample\_011046840, Unigene39625\_Sample\_011046840, Unigene40766\_Sample\_011046840, Unigene41345\_Sample\_011046840, Unigene42231\_Sample\_011046840, Unigene7035\_Sample\_011046840, Unigene27598\_Sample\_011046840, Unigene41758\_Sample\_011046840, Unigene693\_Sample\_011046840, Unigene31742\_Sample\_011046840, Unigene1790\_Sample\_011046840, Unigene36362\_Sample\_011046840, Unigene42677\_Sample\_011046840, Unigene27056\_Sample\_011046840, Unigene33045\_Sample\_011046840, Unigene35550\_Sample\_011046840, Unigene42839\_Sample\_011046840, Unigene3839\_Sample\_011046840, Unigene30821\_Sample\_011046840, Unigene36889\_Sample\_011046840, Unigene38423\_Sample\_011046840, Unigene37909\_Sample\_011046840, Unigene39350\_Sample\_011046840, Unigene3151\_Sample\_011046840 |
| 3 | O-Glycan biosynthesis Back to summary table | Unigene5517\_Sample\_011046840, Unigene7971\_Sample\_011046840, Unigene11155\_Sample\_011046840, Unigene18403\_Sample\_011046840, Unigene27232\_Sample\_011046840, Unigene36580\_Sample\_011046840, Unigene7509\_Sample\_011046840, Unigene11896\_Sample\_011046840, Unigene28039\_Sample\_011046840, Unigene35183\_Sample\_011046840, Unigene37282\_Sample\_011046840, Unigene4612\_Sample\_011046840, Unigene5278\_Sample\_011046840, Unigene42966\_Sample\_011046840, Unigene41693\_Sample\_011046840, Unigene333\_Sample\_011046840, Unigene5257\_Sample\_011046840, Unigene18248\_Sample\_011046840, Unigene36477\_Sample\_011046840, Unigene43464\_Sample\_011046840, Unigene37439\_Sample\_011046840, Unigene38070\_Sample\_011046840, Unigene16690\_Sample\_011046840, Unigene30889\_Sample\_011046840, Unigene31916\_Sample\_011046840, Unigene32591\_Sample\_011046840, Unigene41236\_Sample\_011046840, Unigene15781\_Sample\_011046840, Unigene19320\_Sample\_011046840 |
| 4 | Homologous recombination Back to summary table | Unigene29976\_Sample\_011046840, Unigene39339\_Sample\_011046840, Unigene41600\_Sample\_011046840, Unigene42357\_Sample\_011046840, Unigene2066\_Sample\_011046840, Unigene2740\_Sample\_011046840, Unigene7902\_Sample\_011046840, Unigene12550\_Sample\_011046840, Unigene20014\_Sample\_011046840, Unigene22265\_Sample\_011046840, Unigene29875\_Sample\_011046840, Unigene30400\_Sample\_011046840, Unigene34230\_Sample\_011046840, Unigene34573\_Sample\_011046840, Unigene38747\_Sample\_011046840, Unigene40049\_Sample\_011046840, Unigene40223\_Sample\_011046840, Unigene40625\_Sample\_011046840, Unigene41398\_Sample\_011046840, Unigene41880\_Sample\_011046840, Unigene7935\_Sample\_011046840, Unigene21033\_Sample\_011046840, Unigene40812\_Sample\_011046840, Unigene37705\_Sample\_011046840, Unigene3915\_Sample\_011046840, Unigene4723\_Sample\_011046840, Unigene8081\_Sample\_011046840, Unigene21136\_Sample\_011046840, Unigene24917\_Sample\_011046840, Unigene28451\_Sample\_011046840, Unigene31960\_Sample\_011046840, Unigene36117\_Sample\_011046840, Unigene38337\_Sample\_011046840, Unigene28631\_Sample\_011046840, Unigene31572\_Sample\_011046840, Unigene34828\_Sample\_011046840, Unigene35476\_Sample\_011046840, Unigene6961\_Sample\_011046840, Unigene19866\_Sample\_011046840, Unigene20787\_Sample\_011046840, Unigene34315\_Sample\_011046840, Unigene4108\_Sample\_011046840, Unigene7475\_Sample\_011046840, Unigene26800\_Sample\_011046840, Unigene31539\_Sample\_011046840, Unigene40571\_Sample\_011046840, Unigene43374\_Sample\_011046840, Unigene10700\_Sample\_011046840, Unigene18570\_Sample\_011046840, Unigene34002\_Sample\_011046840, Unigene40061\_Sample\_011046840, Unigene41104\_Sample\_011046840, Unigene34583\_Sample\_011046840, Unigene35167\_Sample\_011046840, Unigene41334\_Sample\_011046840, Unigene7882\_Sample\_011046840, Unigene16944\_Sample\_011046840, Unigene30448\_Sample\_011046840, Unigene30920\_Sample\_011046840, Unigene34691\_Sample\_011046840, Unigene41163\_Sample\_011046840, Unigene10159\_Sample\_011046840, Unigene11948\_Sample\_011046840 |
| 5 | MAPK signaling pathway - yeast Back to summary table | Unigene535\_Sample\_011046840, Unigene880\_Sample\_011046840, Unigene4993\_Sample\_011046840, Unigene30862\_Sample\_011046840, Unigene42127\_Sample\_011046840, Unigene42759\_Sample\_011046840, Unigene42889\_Sample\_011046840, Unigene3776\_Sample\_011046840, Unigene4101\_Sample\_011046840, Unigene5564\_Sample\_011046840, Unigene32467\_Sample\_011046840, Unigene36347\_Sample\_011046840, Unigene36905\_Sample\_011046840, Unigene39843\_Sample\_011046840, Unigene41370\_Sample\_011046840, Unigene41915\_Sample\_011046840, Unigene42479\_Sample\_011046840, Unigene42716\_Sample\_011046840, Unigene3608\_Sample\_011046840, Unigene14753\_Sample\_011046840, Unigene3161\_Sample\_011046840, Unigene6322\_Sample\_011046840, Unigene36839\_Sample\_011046840, Unigene40221\_Sample\_011046840, Unigene4807\_Sample\_011046840, Unigene40888\_Sample\_011046840, Unigene42487\_Sample\_011046840, Unigene33873\_Sample\_011046840, Unigene7073\_Sample\_011046840, Unigene29670\_Sample\_011046840, Unigene30801\_Sample\_011046840, Unigene40648\_Sample\_011046840, Unigene42385\_Sample\_011046840, Unigene33553\_Sample\_011046840, Unigene4849\_Sample\_011046840, Unigene19127\_Sample\_011046840, Unigene28122\_Sample\_011046840, Unigene37246\_Sample\_011046840, Unigene21815\_Sample\_011046840, Unigene33692\_Sample\_011046840, Unigene11201\_Sample\_011046840 |
| 6 | B cell receptor signaling pathway Back to summary table | Unigene228\_Sample\_011046840, Unigene2690\_Sample\_011046840, Unigene4790\_Sample\_011046840, Unigene7980\_Sample\_011046840, Unigene15939\_Sample\_011046840, Unigene41242\_Sample\_011046840, Unigene42773\_Sample\_011046840, Unigene43441\_Sample\_011046840, Unigene43496\_Sample\_011046840, Unigene1286\_Sample\_011046840, Unigene2986\_Sample\_011046840, Unigene4779\_Sample\_011046840, Unigene7868\_Sample\_011046840, Unigene10748\_Sample\_011046840, Unigene34085\_Sample\_011046840, Unigene34974\_Sample\_011046840, Unigene35122\_Sample\_011046840, Unigene38617\_Sample\_011046840, Unigene38866\_Sample\_011046840, Unigene39242\_Sample\_011046840, Unigene39883\_Sample\_011046840, Unigene42164\_Sample\_011046840, Unigene43301\_Sample\_011046840, Unigene5933\_Sample\_011046840, Unigene33835\_Sample\_011046840, Unigene38847\_Sample\_011046840, Unigene42910\_Sample\_011046840, Unigene43149\_Sample\_011046840, Unigene13789\_Sample\_011046840, Unigene29542\_Sample\_011046840, Unigene31699\_Sample\_011046840, Unigene7408\_Sample\_011046840, Unigene20325\_Sample\_011046840, Unigene23045\_Sample\_011046840, Unigene28883\_Sample\_011046840, Unigene35150\_Sample\_011046840, Unigene36320\_Sample\_011046840, Unigene36860\_Sample\_011046840, Unigene40335\_Sample\_011046840, Unigene43068\_Sample\_011046840, Unigene43392\_Sample\_011046840, Unigene24869\_Sample\_011046840, Unigene28124\_Sample\_011046840, Unigene30141\_Sample\_011046840, Unigene4139\_Sample\_011046840, Unigene18933\_Sample\_011046840, Unigene26785\_Sample\_011046840, Unigene28751\_Sample\_011046840, Unigene31705\_Sample\_011046840, Unigene31880\_Sample\_011046840, Unigene37830\_Sample\_011046840, Unigene39503\_Sample\_011046840, Unigene40189\_Sample\_011046840, Unigene42008\_Sample\_011046840, Unigene43154\_Sample\_011046840, Unigene1368\_Sample\_011046840, Unigene6944\_Sample\_011046840, Unigene17676\_Sample\_011046840, Unigene19400\_Sample\_011046840, Unigene20896\_Sample\_011046840, Unigene29526\_Sample\_011046840, Unigene29660\_Sample\_011046840, Unigene32479\_Sample\_011046840, Unigene33870\_Sample\_011046840, Unigene38572\_Sample\_011046840, Unigene42811\_Sample\_011046840, Unigene5726\_Sample\_011046840, Unigene21176\_Sample\_011046840, Unigene27623\_Sample\_011046840, Unigene37778\_Sample\_011046840, Unigene41560\_Sample\_011046840, Unigene10037\_Sample\_011046840, Unigene32864\_Sample\_011046840, Unigene41704\_Sample\_011046840, Unigene14930\_Sample\_011046840, Unigene16035\_Sample\_011046840, Unigene32265\_Sample\_011046840, Unigene41410\_Sample\_011046840, Unigene31562\_Sample\_011046840, Unigene39535\_Sample\_011046840 |
| 7 | Arginine and proline metabolism Back to summary table | Unigene1619\_Sample\_011046840, Unigene2741\_Sample\_011046840, Unigene4570\_Sample\_011046840, Unigene4663\_Sample\_011046840, Unigene7194\_Sample\_011046840, Unigene26766\_Sample\_011046840, Unigene30757\_Sample\_011046840, Unigene42701\_Sample\_011046840, Unigene865\_Sample\_011046840, Unigene7945\_Sample\_011046840, Unigene8475\_Sample\_011046840, Unigene11684\_Sample\_011046840, Unigene18383\_Sample\_011046840, Unigene23357\_Sample\_011046840, Unigene31901\_Sample\_011046840, Unigene32005\_Sample\_011046840, Unigene36713\_Sample\_011046840, Unigene38277\_Sample\_011046840, Unigene38380\_Sample\_011046840, Unigene40609\_Sample\_011046840, Unigene41805\_Sample\_011046840, Unigene41821\_Sample\_011046840, Unigene41961\_Sample\_011046840, Unigene42590\_Sample\_011046840, Unigene42972\_Sample\_011046840, Unigene43555\_Sample\_011046840, Unigene5876\_Sample\_011046840, Unigene7134\_Sample\_011046840, Unigene38355\_Sample\_011046840, Unigene4446\_Sample\_011046840, Unigene473\_Sample\_011046840, Unigene7751\_Sample\_011046840, Unigene20699\_Sample\_011046840, Unigene27797\_Sample\_011046840, Unigene33029\_Sample\_011046840, Unigene33797\_Sample\_011046840, Unigene35349\_Sample\_011046840, Unigene38972\_Sample\_011046840, Unigene39002\_Sample\_011046840, Unigene41087\_Sample\_011046840, Unigene43449\_Sample\_011046840, Unigene32895\_Sample\_011046840, Unigene1504\_Sample\_011046840, Unigene24657\_Sample\_011046840, Unigene3068\_Sample\_011046840, Unigene25675\_Sample\_011046840, Unigene3029\_Sample\_011046840, Unigene8122\_Sample\_011046840, Unigene32735\_Sample\_011046840, Unigene36053\_Sample\_011046840, Unigene41828\_Sample\_011046840, Unigene13285\_Sample\_011046840, Unigene20183\_Sample\_011046840, Unigene32020\_Sample\_011046840, Unigene10135\_Sample\_011046840, Unigene25488\_Sample\_011046840, Unigene32114\_Sample\_011046840, Unigene18659\_Sample\_011046840, Unigene27158\_Sample\_011046840 |
| 8 | Apoptosis Back to summary table | Unigene2690\_Sample\_011046840, Unigene8069\_Sample\_011046840, Unigene40979\_Sample\_011046840, Unigene7868\_Sample\_011046840, Unigene27254\_Sample\_011046840, Unigene34974\_Sample\_011046840, Unigene36749\_Sample\_011046840, Unigene38617\_Sample\_011046840, Unigene40128\_Sample\_011046840, Unigene42065\_Sample\_011046840, Unigene42164\_Sample\_011046840, Unigene7322\_Sample\_011046840, Unigene38847\_Sample\_011046840, Unigene43149\_Sample\_011046840, Unigene17259\_Sample\_011046840, Unigene31655\_Sample\_011046840, Unigene31945\_Sample\_011046840, Unigene36860\_Sample\_011046840, Unigene39081\_Sample\_011046840, Unigene42642\_Sample\_011046840, Unigene43068\_Sample\_011046840, Unigene8064\_Sample\_011046840, Unigene24869\_Sample\_011046840, Unigene28124\_Sample\_011046840, Unigene33392\_Sample\_011046840, Unigene4139\_Sample\_011046840, Unigene18933\_Sample\_011046840, Unigene26785\_Sample\_011046840, Unigene32974\_Sample\_011046840, Unigene42008\_Sample\_011046840, Unigene1368\_Sample\_011046840, Unigene6944\_Sample\_011046840, Unigene17676\_Sample\_011046840, Unigene20896\_Sample\_011046840, Unigene25457\_Sample\_011046840, Unigene32479\_Sample\_011046840, Unigene42811\_Sample\_011046840, Unigene5726\_Sample\_011046840, Unigene21176\_Sample\_011046840, Unigene32489\_Sample\_011046840, Unigene33634\_Sample\_011046840, Unigene9230\_Sample\_011046840, Unigene32864\_Sample\_011046840, Unigene41704\_Sample\_011046840, Unigene16035\_Sample\_011046840, Unigene32265\_Sample\_011046840, Unigene41410\_Sample\_011046840, Unigene31562\_Sample\_011046840, Unigene36611\_Sample\_011046840 |
| 9 | Natural killer cell mediated cytotoxicity Back to summary table | Unigene880\_Sample\_011046840, Unigene2690\_Sample\_011046840, Unigene4790\_Sample\_011046840, Unigene4993\_Sample\_011046840, Unigene15939\_Sample\_011046840, Unigene30862\_Sample\_011046840, Unigene39096\_Sample\_011046840, Unigene43496\_Sample\_011046840, Unigene2986\_Sample\_011046840, Unigene3258\_Sample\_011046840, Unigene4033\_Sample\_011046840, Unigene4101\_Sample\_011046840, Unigene7868\_Sample\_011046840, Unigene10748\_Sample\_011046840, Unigene34085\_Sample\_011046840, Unigene34974\_Sample\_011046840, Unigene35122\_Sample\_011046840, Unigene38617\_Sample\_011046840, Unigene38866\_Sample\_011046840, Unigene39843\_Sample\_011046840, Unigene39883\_Sample\_011046840, Unigene40208\_Sample\_011046840, Unigene41370\_Sample\_011046840, Unigene41584\_Sample\_011046840, Unigene41915\_Sample\_011046840, Unigene3608\_Sample\_011046840, Unigene14932\_Sample\_011046840, Unigene33835\_Sample\_011046840, Unigene36962\_Sample\_011046840, Unigene43149\_Sample\_011046840, Unigene3161\_Sample\_011046840, Unigene6322\_Sample\_011046840, Unigene4807\_Sample\_011046840, Unigene7408\_Sample\_011046840, Unigene8088\_Sample\_011046840, Unigene20325\_Sample\_011046840, Unigene23045\_Sample\_011046840, Unigene28708\_Sample\_011046840, Unigene28883\_Sample\_011046840, Unigene35150\_Sample\_011046840, Unigene36320\_Sample\_011046840, Unigene36860\_Sample\_011046840, Unigene42487\_Sample\_011046840, Unigene43068\_Sample\_011046840, Unigene43392\_Sample\_011046840, Unigene21908\_Sample\_011046840, Unigene24869\_Sample\_011046840, Unigene28124\_Sample\_011046840, Unigene30141\_Sample\_011046840, Unigene14255\_Sample\_011046840, Unigene16429\_Sample\_011046840, Unigene18933\_Sample\_011046840, Unigene26785\_Sample\_011046840, Unigene27378\_Sample\_011046840, Unigene28751\_Sample\_011046840, Unigene29670\_Sample\_011046840, Unigene31880\_Sample\_011046840, Unigene37830\_Sample\_011046840, Unigene39503\_Sample\_011046840, Unigene40189\_Sample\_011046840, Unigene40648\_Sample\_011046840, Unigene42008\_Sample\_011046840, Unigene42385\_Sample\_011046840, Unigene43154\_Sample\_011046840, Unigene4849\_Sample\_011046840, Unigene6944\_Sample\_011046840, Unigene17676\_Sample\_011046840, Unigene20896\_Sample\_011046840, Unigene32479\_Sample\_011046840, Unigene33870\_Sample\_011046840, Unigene38572\_Sample\_011046840, Unigene42811\_Sample\_011046840, Unigene2209\_Sample\_011046840, Unigene13067\_Sample\_011046840, Unigene27623\_Sample\_011046840, Unigene41560\_Sample\_011046840, Unigene32864\_Sample\_011046840, Unigene33692\_Sample\_011046840, Unigene41704\_Sample\_011046840, Unigene11201\_Sample\_011046840, Unigene14930\_Sample\_011046840, Unigene20420\_Sample\_011046840, Unigene32265\_Sample\_011046840, Unigene31562\_Sample\_011046840 |
| 10 | Hypertrophic cardiomyopathy (HCM) Back to summary table | Unigene259\_Sample\_011046840, Unigene409\_Sample\_011046840, Unigene4515\_Sample\_011046840, Unigene4981\_Sample\_011046840, Unigene5147\_Sample\_011046840, Unigene5831\_Sample\_011046840, Unigene6576\_Sample\_011046840, Unigene6630\_Sample\_011046840, Unigene8231\_Sample\_011046840, Unigene9987\_Sample\_011046840, Unigene23056\_Sample\_011046840, Unigene23133\_Sample\_011046840, Unigene25850\_Sample\_011046840, Unigene29452\_Sample\_011046840, Unigene31309\_Sample\_011046840, Unigene40596\_Sample\_011046840, Unigene41683\_Sample\_011046840, Unigene41900\_Sample\_011046840, Unigene42639\_Sample\_011046840, Unigene42826\_Sample\_011046840, Unigene42884\_Sample\_011046840, Unigene43073\_Sample\_011046840, Unigene43429\_Sample\_011046840, Unigene2230\_Sample\_011046840, Unigene3724\_Sample\_011046840, Unigene4028\_Sample\_011046840, Unigene4536\_Sample\_011046840, Unigene5214\_Sample\_011046840, Unigene5795\_Sample\_011046840, Unigene7704\_Sample\_011046840, Unigene8012\_Sample\_011046840, Unigene8114\_Sample\_011046840, Unigene17998\_Sample\_011046840, Unigene28163\_Sample\_011046840, Unigene29336\_Sample\_011046840, Unigene31398\_Sample\_011046840, Unigene32879\_Sample\_011046840, Unigene33926\_Sample\_011046840, Unigene38045\_Sample\_011046840, Unigene38281\_Sample\_011046840, Unigene41237\_Sample\_011046840, Unigene41630\_Sample\_011046840, Unigene41856\_Sample\_011046840, Unigene42336\_Sample\_011046840, Unigene42424\_Sample\_011046840, Unigene42819\_Sample\_011046840, Unigene42838\_Sample\_011046840, Unigene43004\_Sample\_011046840, Unigene43258\_Sample\_011046840, Unigene43312\_Sample\_011046840, Unigene43547\_Sample\_011046840, Unigene448\_Sample\_011046840, Unigene4511\_Sample\_011046840, Unigene8133\_Sample\_011046840, Unigene25969\_Sample\_011046840, Unigene29603\_Sample\_011046840, Unigene36775\_Sample\_011046840, Unigene42303\_Sample\_011046840, Unigene42603\_Sample\_011046840, Unigene23177\_Sample\_011046840, Unigene27657\_Sample\_011046840, Unigene36373\_Sample\_011046840, Unigene41719\_Sample\_011046840, Unigene43466\_Sample\_011046840, Unigene5364\_Sample\_011046840, Unigene25431\_Sample\_011046840, Unigene31285\_Sample\_011046840, Unigene31860\_Sample\_011046840, Unigene31889\_Sample\_011046840, Unigene34942\_Sample\_011046840, Unigene35114\_Sample\_011046840, Unigene35443\_Sample\_011046840, Unigene37336\_Sample\_011046840, Unigene39505\_Sample\_011046840, Unigene39625\_Sample\_011046840, Unigene41345\_Sample\_011046840, Unigene41485\_Sample\_011046840, Unigene42110\_Sample\_011046840, Unigene42913\_Sample\_011046840, Unigene3758\_Sample\_011046840, Unigene26711\_Sample\_011046840, Unigene28892\_Sample\_011046840, Unigene34520\_Sample\_011046840, Unigene38616\_Sample\_011046840, Unigene41881\_Sample\_011046840, Unigene43250\_Sample\_011046840, Unigene43309\_Sample\_011046840, Unigene50\_Sample\_011046840, Unigene6035\_Sample\_011046840, Unigene7196\_Sample\_011046840, Unigene24545\_Sample\_011046840, Unigene32118\_Sample\_011046840, Unigene36856\_Sample\_011046840, Unigene43015\_Sample\_011046840, Unigene26493\_Sample\_011046840, Unigene34848\_Sample\_011046840, Unigene42677\_Sample\_011046840, Unigene6846\_Sample\_011046840, Unigene42212\_Sample\_011046840, Unigene2815\_Sample\_011046840, Unigene3219\_Sample\_011046840, Unigene24388\_Sample\_011046840, Unigene36060\_Sample\_011046840, Unigene38423\_Sample\_011046840, Unigene40056\_Sample\_011046840, Unigene40892\_Sample\_011046840, Unigene12868\_Sample\_011046840, Unigene15237\_Sample\_011046840, Unigene31135\_Sample\_011046840, Unigene39360\_Sample\_011046840, Unigene4030\_Sample\_011046840, Unigene25727\_Sample\_011046840, Unigene29797\_Sample\_011046840, Unigene35039\_Sample\_011046840, Unigene35068\_Sample\_011046840, Unigene35925\_Sample\_011046840, Unigene37909\_Sample\_011046840, Unigene43398\_Sample\_011046840, Unigene5921\_Sample\_011046840, Unigene11113\_Sample\_011046840, Unigene21138\_Sample\_011046840, Unigene21744\_Sample\_011046840, Unigene43443\_Sample\_011046840 |
| 11 | Colorectal cancer Back to summary table | Unigene15939\_Sample\_011046840, Unigene40446\_Sample\_011046840, Unigene41242\_Sample\_011046840, Unigene41702\_Sample\_011046840, Unigene43118\_Sample\_011046840, Unigene43211\_Sample\_011046840, Unigene43441\_Sample\_011046840, Unigene2986\_Sample\_011046840, Unigene4254\_Sample\_011046840, Unigene4779\_Sample\_011046840, Unigene5197\_Sample\_011046840, Unigene6863\_Sample\_011046840, Unigene15111\_Sample\_011046840, Unigene24698\_Sample\_011046840, Unigene27254\_Sample\_011046840, Unigene29711\_Sample\_011046840, Unigene30370\_Sample\_011046840, Unigene30539\_Sample\_011046840, Unigene34974\_Sample\_011046840, Unigene38435\_Sample\_011046840, Unigene38534\_Sample\_011046840, Unigene38612\_Sample\_011046840, Unigene38617\_Sample\_011046840, Unigene39883\_Sample\_011046840, Unigene40208\_Sample\_011046840, Unigene41584\_Sample\_011046840, Unigene41949\_Sample\_011046840, Unigene42164\_Sample\_011046840, Unigene43301\_Sample\_011046840, Unigene43492\_Sample\_011046840, Unigene43502\_Sample\_011046840, Unigene26548\_Sample\_011046840, Unigene33835\_Sample\_011046840, Unigene33954\_Sample\_011046840, Unigene35961\_Sample\_011046840, Unigene38847\_Sample\_011046840, Unigene40065\_Sample\_011046840, Unigene43149\_Sample\_011046840, Unigene6921\_Sample\_011046840, Unigene21306\_Sample\_011046840, Unigene22181\_Sample\_011046840, Unigene28433\_Sample\_011046840, Unigene29542\_Sample\_011046840, Unigene31699\_Sample\_011046840, Unigene33984\_Sample\_011046840, Unigene4818\_Sample\_011046840, Unigene19401\_Sample\_011046840, Unigene28708\_Sample\_011046840, Unigene31860\_Sample\_011046840, Unigene34871\_Sample\_011046840, Unigene35699\_Sample\_011046840, Unigene35981\_Sample\_011046840, Unigene38655\_Sample\_011046840, Unigene41303\_Sample\_011046840, Unigene43068\_Sample\_011046840, Unigene24869\_Sample\_011046840, Unigene42346\_Sample\_011046840, Unigene4139\_Sample\_011046840, Unigene9938\_Sample\_011046840, Unigene13197\_Sample\_011046840, Unigene16429\_Sample\_011046840, Unigene18933\_Sample\_011046840, Unigene26785\_Sample\_011046840, Unigene27378\_Sample\_011046840, Unigene31705\_Sample\_011046840, Unigene31880\_Sample\_011046840, Unigene37702\_Sample\_011046840, Unigene39503\_Sample\_011046840, Unigene42008\_Sample\_011046840, Unigene43154\_Sample\_011046840, Unigene36362\_Sample\_011046840, Unigene1368\_Sample\_011046840, Unigene14237\_Sample\_011046840, Unigene19400\_Sample\_011046840, Unigene20896\_Sample\_011046840, Unigene25523\_Sample\_011046840, Unigene29526\_Sample\_011046840, Unigene29660\_Sample\_011046840, Unigene33870\_Sample\_011046840, Unigene38882\_Sample\_011046840, Unigene42811\_Sample\_011046840, Unigene5726\_Sample\_011046840, Unigene21176\_Sample\_011046840, Unigene30821\_Sample\_011046840, Unigene34360\_Sample\_011046840, Unigene10037\_Sample\_011046840, Unigene30201\_Sample\_011046840, Unigene35972\_Sample\_011046840, Unigene41704\_Sample\_011046840, Unigene1568\_Sample\_011046840, Unigene14930\_Sample\_011046840, Unigene16035\_Sample\_011046840, Unigene19481\_Sample\_011046840, Unigene20420\_Sample\_011046840, Unigene26719\_Sample\_011046840, Unigene32265\_Sample\_011046840, Unigene19593\_Sample\_011046840, Unigene37186\_Sample\_011046840 |
| 12 | Axon guidance Back to summary table | Unigene1555\_Sample\_011046840, Unigene2174\_Sample\_011046840, Unigene2690\_Sample\_011046840, Unigene4993\_Sample\_011046840, Unigene6378\_Sample\_011046840, Unigene8191\_Sample\_011046840, Unigene9987\_Sample\_011046840, Unigene10035\_Sample\_011046840, Unigene18283\_Sample\_011046840, Unigene25850\_Sample\_011046840, Unigene37793\_Sample\_011046840, Unigene38706\_Sample\_011046840, Unigene40286\_Sample\_011046840, Unigene41242\_Sample\_011046840, Unigene42475\_Sample\_011046840, Unigene42639\_Sample\_011046840, Unigene43311\_Sample\_011046840, Unigene43556\_Sample\_011046840, Unigene1039\_Sample\_011046840, Unigene1252\_Sample\_011046840, Unigene2986\_Sample\_011046840, Unigene3258\_Sample\_011046840, Unigene3903\_Sample\_011046840, Unigene4715\_Sample\_011046840, Unigene4779\_Sample\_011046840, Unigene5122\_Sample\_011046840, Unigene5259\_Sample\_011046840, Unigene5290\_Sample\_011046840, Unigene5665\_Sample\_011046840, Unigene6863\_Sample\_011046840, Unigene7002\_Sample\_011046840, Unigene7349\_Sample\_011046840, Unigene7394\_Sample\_011046840, Unigene7506\_Sample\_011046840, Unigene7868\_Sample\_011046840, Unigene7976\_Sample\_011046840, Unigene8010\_Sample\_011046840, Unigene8023\_Sample\_011046840, Unigene8063\_Sample\_011046840, Unigene8131\_Sample\_011046840, Unigene8185\_Sample\_011046840, Unigene32734\_Sample\_011046840, Unigene38291\_Sample\_011046840, Unigene38422\_Sample\_011046840, Unigene39160\_Sample\_011046840, Unigene39843\_Sample\_011046840, Unigene39883\_Sample\_011046840, Unigene40388\_Sample\_011046840, Unigene40570\_Sample\_011046840, Unigene41370\_Sample\_011046840, Unigene41501\_Sample\_011046840, Unigene41915\_Sample\_011046840, Unigene42646\_Sample\_011046840, Unigene43301\_Sample\_011046840, Unigene43342\_Sample\_011046840, Unigene43526\_Sample\_011046840, Unigene43543\_Sample\_011046840, Unigene43576\_Sample\_011046840, Unigene43578\_Sample\_011046840, Unigene1924\_Sample\_011046840, Unigene2247\_Sample\_011046840, Unigene3828\_Sample\_011046840, Unigene6402\_Sample\_011046840, Unigene22396\_Sample\_011046840, Unigene25969\_Sample\_011046840, Unigene26548\_Sample\_011046840, Unigene28292\_Sample\_011046840, Unigene33835\_Sample\_011046840, Unigene34584\_Sample\_011046840, Unigene36636\_Sample\_011046840, Unigene42641\_Sample\_011046840, Unigene14035\_Sample\_011046840, Unigene22038\_Sample\_011046840, Unigene22181\_Sample\_011046840, Unigene28433\_Sample\_011046840, Unigene29542\_Sample\_011046840, Unigene31699\_Sample\_011046840, Unigene37012\_Sample\_011046840, Unigene37705\_Sample\_011046840, Unigene42798\_Sample\_011046840, Unigene43466\_Sample\_011046840, Unigene43559\_Sample\_011046840, Unigene3735\_Sample\_011046840, Unigene4818\_Sample\_011046840, Unigene8040\_Sample\_011046840, Unigene8080\_Sample\_011046840, Unigene14485\_Sample\_011046840, Unigene19204\_Sample\_011046840, Unigene21554\_Sample\_011046840, Unigene30530\_Sample\_011046840, Unigene31079\_Sample\_011046840, Unigene31393\_Sample\_011046840, Unigene31860\_Sample\_011046840, Unigene33288\_Sample\_011046840, Unigene34871\_Sample\_011046840, Unigene35379\_Sample\_011046840, Unigene36817\_Sample\_011046840, Unigene36860\_Sample\_011046840, Unigene40211\_Sample\_011046840, Unigene40838\_Sample\_011046840, Unigene41967\_Sample\_011046840, Unigene42079\_Sample\_011046840, Unigene42487\_Sample\_011046840, Unigene42685\_Sample\_011046840, Unigene7314\_Sample\_011046840, Unigene14379\_Sample\_011046840, Unigene15148\_Sample\_011046840, Unigene28124\_Sample\_011046840, Unigene37128\_Sample\_011046840, Unigene5132\_Sample\_011046840, Unigene17368\_Sample\_011046840, Unigene29670\_Sample\_011046840, Unigene31705\_Sample\_011046840, Unigene32721\_Sample\_011046840, Unigene36897\_Sample\_011046840, Unigene39503\_Sample\_011046840, Unigene40473\_Sample\_011046840, Unigene42141\_Sample\_011046840, Unigene43154\_Sample\_011046840, Unigene22877\_Sample\_011046840, Unigene25534\_Sample\_011046840, Unigene36362\_Sample\_011046840, Unigene40259\_Sample\_011046840, Unigene2420\_Sample\_011046840, Unigene6944\_Sample\_011046840, Unigene12492\_Sample\_011046840, Unigene16224\_Sample\_011046840, Unigene17676\_Sample\_011046840, Unigene19400\_Sample\_011046840, Unigene24411\_Sample\_011046840, Unigene25317\_Sample\_011046840, Unigene29526\_Sample\_011046840, Unigene29917\_Sample\_011046840, Unigene32290\_Sample\_011046840, Unigene32479\_Sample\_011046840, Unigene33765\_Sample\_011046840, Unigene34894\_Sample\_011046840, Unigene36126\_Sample\_011046840, Unigene38675\_Sample\_011046840, Unigene41475\_Sample\_011046840, Unigene42839\_Sample\_011046840, Unigene7479\_Sample\_011046840, Unigene7796\_Sample\_011046840, Unigene7924\_Sample\_011046840, Unigene19127\_Sample\_011046840, Unigene25531\_Sample\_011046840, Unigene30821\_Sample\_011046840, Unigene34012\_Sample\_011046840, Unigene38423\_Sample\_011046840, Unigene42902\_Sample\_011046840, Unigene3861\_Sample\_011046840, Unigene10037\_Sample\_011046840, Unigene24184\_Sample\_011046840, Unigene32864\_Sample\_011046840, Unigene37254\_Sample\_011046840, Unigene38066\_Sample\_011046840, Unigene1256\_Sample\_011046840, Unigene4276\_Sample\_011046840, Unigene5044\_Sample\_011046840, Unigene14930\_Sample\_011046840, Unigene28102\_Sample\_011046840, Unigene31424\_Sample\_011046840, Unigene38476\_Sample\_011046840, Unigene42184\_Sample\_011046840, Unigene31562\_Sample\_011046840, Unigene31926\_Sample\_011046840, Unigene35401\_Sample\_011046840, Unigene37185\_Sample\_011046840 |
| 13 | Lysosome Back to summary table | Unigene2144\_Sample\_011046840, Unigene2176\_Sample\_011046840, Unigene2439\_Sample\_011046840, Unigene4623\_Sample\_011046840, Unigene5734\_Sample\_011046840, Unigene5772\_Sample\_011046840, Unigene6042\_Sample\_011046840, Unigene6705\_Sample\_011046840, Unigene7919\_Sample\_011046840, Unigene7966\_Sample\_011046840, Unigene8020\_Sample\_011046840, Unigene8180\_Sample\_011046840, Unigene8864\_Sample\_011046840, Unigene26216\_Sample\_011046840, Unigene28676\_Sample\_011046840, Unigene30678\_Sample\_011046840, Unigene35486\_Sample\_011046840, Unigene37193\_Sample\_011046840, Unigene38003\_Sample\_011046840, Unigene38106\_Sample\_011046840, Unigene38326\_Sample\_011046840, Unigene39923\_Sample\_011046840, Unigene40051\_Sample\_011046840, Unigene43124\_Sample\_011046840, Unigene43408\_Sample\_011046840, Unigene43475\_Sample\_011046840, Unigene1755\_Sample\_011046840, Unigene3983\_Sample\_011046840, Unigene4062\_Sample\_011046840, Unigene5625\_Sample\_011046840, Unigene6860\_Sample\_011046840, Unigene7024\_Sample\_011046840, Unigene7589\_Sample\_011046840, Unigene7881\_Sample\_011046840, Unigene8150\_Sample\_011046840, Unigene9112\_Sample\_011046840, Unigene9447\_Sample\_011046840, Unigene18477\_Sample\_011046840, Unigene23713\_Sample\_011046840, Unigene25194\_Sample\_011046840, Unigene26709\_Sample\_011046840, Unigene27226\_Sample\_011046840, Unigene28956\_Sample\_011046840, Unigene29525\_Sample\_011046840, Unigene30383\_Sample\_011046840, Unigene31044\_Sample\_011046840, Unigene31806\_Sample\_011046840, Unigene31812\_Sample\_011046840, Unigene31994\_Sample\_011046840, Unigene32285\_Sample\_011046840, Unigene32425\_Sample\_011046840, Unigene32590\_Sample\_011046840, Unigene32901\_Sample\_011046840, Unigene33184\_Sample\_011046840, Unigene33772\_Sample\_011046840, Unigene35341\_Sample\_011046840, Unigene35431\_Sample\_011046840, Unigene36758\_Sample\_011046840, Unigene36977\_Sample\_011046840, Unigene37754\_Sample\_011046840, Unigene38497\_Sample\_011046840, Unigene38813\_Sample\_011046840, Unigene38931\_Sample\_011046840, Unigene39609\_Sample\_011046840, Unigene39742\_Sample\_011046840, Unigene41128\_Sample\_011046840, Unigene41227\_Sample\_011046840, Unigene41234\_Sample\_011046840, Unigene41254\_Sample\_011046840, Unigene41378\_Sample\_011046840, Unigene41424\_Sample\_011046840, Unigene41642\_Sample\_011046840, Unigene41914\_Sample\_011046840, Unigene42320\_Sample\_011046840, Unigene42474\_Sample\_011046840, Unigene42651\_Sample\_011046840, Unigene43028\_Sample\_011046840, Unigene43032\_Sample\_011046840, Unigene43629\_Sample\_011046840, Unigene715\_Sample\_011046840, Unigene1399\_Sample\_011046840, Unigene4178\_Sample\_011046840, Unigene31980\_Sample\_011046840, Unigene34891\_Sample\_011046840, Unigene35086\_Sample\_011046840, Unigene37469\_Sample\_011046840, Unigene38490\_Sample\_011046840, Unigene3867\_Sample\_011046840, Unigene5664\_Sample\_011046840, Unigene8072\_Sample\_011046840, Unigene13717\_Sample\_011046840, Unigene20538\_Sample\_011046840, Unigene35138\_Sample\_011046840, Unigene38392\_Sample\_011046840, Unigene40243\_Sample\_011046840, Unigene42698\_Sample\_011046840, Unigene117\_Sample\_011046840, Unigene241\_Sample\_011046840, Unigene1463\_Sample\_011046840, Unigene2548\_Sample\_011046840, Unigene3218\_Sample\_011046840, Unigene3279\_Sample\_011046840, Unigene5034\_Sample\_011046840, Unigene7221\_Sample\_011046840, Unigene7638\_Sample\_011046840, Unigene10402\_Sample\_011046840, Unigene16112\_Sample\_011046840, Unigene19098\_Sample\_011046840, Unigene25781\_Sample\_011046840, Unigene25956\_Sample\_011046840, Unigene31085\_Sample\_011046840, Unigene31106\_Sample\_011046840, Unigene31864\_Sample\_011046840, Unigene33599\_Sample\_011046840, Unigene34972\_Sample\_011046840, Unigene36253\_Sample\_011046840, Unigene36718\_Sample\_011046840, Unigene37629\_Sample\_011046840, Unigene38605\_Sample\_011046840, Unigene40588\_Sample\_011046840, Unigene42488\_Sample\_011046840, Unigene13402\_Sample\_011046840, Unigene26130\_Sample\_011046840, Unigene27441\_Sample\_011046840, Unigene36382\_Sample\_011046840, Unigene42345\_Sample\_011046840, Unigene9645\_Sample\_011046840, Unigene12033\_Sample\_011046840, Unigene17166\_Sample\_011046840, Unigene22216\_Sample\_011046840, Unigene24546\_Sample\_011046840, Unigene27829\_Sample\_011046840, Unigene29690\_Sample\_011046840, Unigene30704\_Sample\_011046840, Unigene33547\_Sample\_011046840, Unigene34034\_Sample\_011046840, Unigene39343\_Sample\_011046840, Unigene39891\_Sample\_011046840, Unigene40924\_Sample\_011046840, Unigene41229\_Sample\_011046840, Unigene42977\_Sample\_011046840, Unigene9750\_Sample\_011046840, Unigene22103\_Sample\_011046840, Unigene27885\_Sample\_011046840, Unigene34041\_Sample\_011046840, Unigene37126\_Sample\_011046840, Unigene2318\_Sample\_011046840, Unigene6374\_Sample\_011046840, Unigene6841\_Sample\_011046840, Unigene10704\_Sample\_011046840, Unigene11899\_Sample\_011046840, Unigene14007\_Sample\_011046840, Unigene14691\_Sample\_011046840, Unigene30979\_Sample\_011046840, Unigene38461\_Sample\_011046840, Unigene38611\_Sample\_011046840, Unigene38900\_Sample\_011046840, Unigene39771\_Sample\_011046840, Unigene41973\_Sample\_011046840, Unigene42755\_Sample\_011046840, Unigene765\_Sample\_011046840, Unigene1574\_Sample\_011046840, Unigene19865\_Sample\_011046840, Unigene19996\_Sample\_011046840, Unigene22291\_Sample\_011046840, Unigene33326\_Sample\_011046840, Unigene33389\_Sample\_011046840, Unigene34595\_Sample\_011046840, Unigene34677\_Sample\_011046840, Unigene38774\_Sample\_011046840, Unigene38853\_Sample\_011046840, Unigene40520\_Sample\_011046840, Unigene40789\_Sample\_011046840, Unigene41158\_Sample\_011046840, Unigene42427\_Sample\_011046840, Unigene1317\_Sample\_011046840, Unigene14612\_Sample\_011046840, Unigene19647\_Sample\_011046840, Unigene30260\_Sample\_011046840, Unigene32870\_Sample\_011046840, Unigene35818\_Sample\_011046840, Unigene36577\_Sample\_011046840, Unigene42363\_Sample\_011046840, Unigene3001\_Sample\_011046840, Unigene14784\_Sample\_011046840, Unigene20157\_Sample\_011046840, Unigene29297\_Sample\_011046840, Unigene30726\_Sample\_011046840, Unigene31389\_Sample\_011046840, Unigene31743\_Sample\_011046840, Unigene39248\_Sample\_011046840, Unigene42157\_Sample\_011046840, Unigene1194\_Sample\_011046840, Unigene13340\_Sample\_011046840, Unigene20109\_Sample\_011046840, Unigene21495\_Sample\_011046840, Unigene24344\_Sample\_011046840, Unigene24378\_Sample\_011046840, Unigene30564\_Sample\_011046840, Unigene32555\_Sample\_011046840, Unigene34366\_Sample\_011046840, Unigene36661\_Sample\_011046840, Unigene39986\_Sample\_011046840 |
| 14 | Neurotrophin signaling pathway Back to summary table | Unigene439\_Sample\_011046840, Unigene4464\_Sample\_011046840, Unigene4790\_Sample\_011046840, Unigene7525\_Sample\_011046840, Unigene7563\_Sample\_011046840, Unigene8069\_Sample\_011046840, Unigene15939\_Sample\_011046840, Unigene33573\_Sample\_011046840, Unigene34317\_Sample\_011046840, Unigene38206\_Sample\_011046840, Unigene38826\_Sample\_011046840, Unigene39096\_Sample\_011046840, Unigene40484\_Sample\_011046840, Unigene41242\_Sample\_011046840, Unigene42127\_Sample\_011046840, Unigene42759\_Sample\_011046840, Unigene42766\_Sample\_011046840, Unigene43311\_Sample\_011046840, Unigene43332\_Sample\_011046840, Unigene43365\_Sample\_011046840, Unigene2418\_Sample\_011046840, Unigene2986\_Sample\_011046840, Unigene4033\_Sample\_011046840, Unigene4043\_Sample\_011046840, Unigene4779\_Sample\_011046840, Unigene5564\_Sample\_011046840, Unigene6863\_Sample\_011046840, Unigene7349\_Sample\_011046840, Unigene10748\_Sample\_011046840, Unigene28710\_Sample\_011046840, Unigene28752\_Sample\_011046840, Unigene28757\_Sample\_011046840, Unigene29711\_Sample\_011046840, Unigene30539\_Sample\_011046840, Unigene32180\_Sample\_011046840, Unigene34085\_Sample\_011046840, Unigene34974\_Sample\_011046840, Unigene36317\_Sample\_011046840, Unigene38617\_Sample\_011046840, Unigene38866\_Sample\_011046840, Unigene38923\_Sample\_011046840, Unigene39883\_Sample\_011046840, Unigene40208\_Sample\_011046840, Unigene40958\_Sample\_011046840, Unigene41096\_Sample\_011046840, Unigene41501\_Sample\_011046840, Unigene41584\_Sample\_011046840, Unigene42164\_Sample\_011046840, Unigene42197\_Sample\_011046840, Unigene43301\_Sample\_011046840, Unigene43313\_Sample\_011046840, Unigene1246\_Sample\_011046840, Unigene5166\_Sample\_011046840, Unigene33835\_Sample\_011046840, Unigene34039\_Sample\_011046840, Unigene38847\_Sample\_011046840, Unigene41695\_Sample\_011046840, Unigene43149\_Sample\_011046840, Unigene6921\_Sample\_011046840, Unigene29542\_Sample\_011046840, Unigene31699\_Sample\_011046840, Unigene36839\_Sample\_011046840, Unigene38648\_Sample\_011046840, Unigene39054\_Sample\_011046840, Unigene40221\_Sample\_011046840, Unigene41264\_Sample\_011046840, Unigene1475\_Sample\_011046840, Unigene4818\_Sample\_011046840, Unigene7408\_Sample\_011046840, Unigene7951\_Sample\_011046840, Unigene8088\_Sample\_011046840, Unigene12635\_Sample\_011046840, Unigene13215\_Sample\_011046840, Unigene20325\_Sample\_011046840, Unigene23045\_Sample\_011046840, Unigene26542\_Sample\_011046840, Unigene28708\_Sample\_011046840, Unigene31079\_Sample\_011046840, Unigene34117\_Sample\_011046840, Unigene36320\_Sample\_011046840, Unigene36788\_Sample\_011046840, Unigene39081\_Sample\_011046840, Unigene39621\_Sample\_011046840, Unigene40334\_Sample\_011046840, Unigene40659\_Sample\_011046840, Unigene41412\_Sample\_011046840, Unigene43068\_Sample\_011046840, Unigene334\_Sample\_011046840, Unigene21908\_Sample\_011046840, Unigene24869\_Sample\_011046840, Unigene26621\_Sample\_011046840, Unigene33873\_Sample\_011046840, Unigene35901\_Sample\_011046840, Unigene37954\_Sample\_011046840, Unigene4139\_Sample\_011046840, Unigene12147\_Sample\_011046840, Unigene14255\_Sample\_011046840, Unigene16429\_Sample\_011046840, Unigene18933\_Sample\_011046840, Unigene25548\_Sample\_011046840, Unigene26268\_Sample\_011046840, Unigene26785\_Sample\_011046840, Unigene27378\_Sample\_011046840, Unigene28751\_Sample\_011046840, Unigene31705\_Sample\_011046840, Unigene31880\_Sample\_011046840, Unigene36897\_Sample\_011046840, Unigene39503\_Sample\_011046840, Unigene40189\_Sample\_011046840, Unigene42008\_Sample\_011046840, Unigene43154\_Sample\_011046840, Unigene42027\_Sample\_011046840, Unigene1368\_Sample\_011046840, Unigene10749\_Sample\_011046840, Unigene14237\_Sample\_011046840, Unigene18009\_Sample\_011046840, Unigene19400\_Sample\_011046840, Unigene20896\_Sample\_011046840, Unigene29526\_Sample\_011046840, Unigene29660\_Sample\_011046840, Unigene33870\_Sample\_011046840, Unigene37041\_Sample\_011046840, Unigene37392\_Sample\_011046840, Unigene42811\_Sample\_011046840, Unigene670\_Sample\_011046840, Unigene2209\_Sample\_011046840, Unigene5726\_Sample\_011046840, Unigene13067\_Sample\_011046840, Unigene19127\_Sample\_011046840, Unigene21176\_Sample\_011046840, Unigene27623\_Sample\_011046840, Unigene39577\_Sample\_011046840, Unigene41560\_Sample\_011046840, Unigene103\_Sample\_011046840, Unigene2711\_Sample\_011046840, Unigene10037\_Sample\_011046840, Unigene41704\_Sample\_011046840, Unigene1568\_Sample\_011046840, Unigene14930\_Sample\_011046840, Unigene16035\_Sample\_011046840, Unigene20420\_Sample\_011046840, Unigene22185\_Sample\_011046840, Unigene29291\_Sample\_011046840, Unigene32265\_Sample\_011046840, Unigene41410\_Sample\_011046840, Unigene19593\_Sample\_011046840, Unigene24889\_Sample\_011046840, Unigene36545\_Sample\_011046840, Unigene37186\_Sample\_011046840, Unigene43443\_Sample\_011046840 |
| 15 | Maturity onset diabetes of the young Back to summary table | Unigene35615\_Sample\_011046840, Unigene43413\_Sample\_011046840, Unigene41706\_Sample\_011046840, Unigene41982\_Sample\_011046840, Unigene16223\_Sample\_011046840, Unigene42882\_Sample\_011046840, Unigene972\_Sample\_011046840, Unigene6407\_Sample\_011046840, Unigene34121\_Sample\_011046840, Unigene41752\_Sample\_011046840, Unigene40781\_Sample\_011046840, Unigene4724\_Sample\_011046840, Unigene40877\_Sample\_011046840, Unigene43217\_Sample\_011046840, Unigene1271\_Sample\_011046840, Unigene33524\_Sample\_011046840, Unigene11179\_Sample\_011046840, Unigene25793\_Sample\_011046840 |
| 16 | Toll-like receptor signaling pathway Back to summary table | Unigene4009\_Sample\_011046840, Unigene7341\_Sample\_011046840, Unigene8198\_Sample\_011046840, Unigene15939\_Sample\_011046840, Unigene40471\_Sample\_011046840, Unigene42127\_Sample\_011046840, Unigene42759\_Sample\_011046840, Unigene42766\_Sample\_011046840, Unigene43441\_Sample\_011046840, Unigene2986\_Sample\_011046840, Unigene4711\_Sample\_011046840, Unigene5616\_Sample\_011046840, Unigene5981\_Sample\_011046840, Unigene29711\_Sample\_011046840, Unigene30539\_Sample\_011046840, Unigene34974\_Sample\_011046840, Unigene38617\_Sample\_011046840, Unigene39004\_Sample\_011046840, Unigene39418\_Sample\_011046840, Unigene39883\_Sample\_011046840, Unigene42164\_Sample\_011046840, Unigene8115\_Sample\_011046840, Unigene14437\_Sample\_011046840, Unigene29880\_Sample\_011046840, Unigene33835\_Sample\_011046840, Unigene34039\_Sample\_011046840, Unigene38847\_Sample\_011046840, Unigene43149\_Sample\_011046840, Unigene6921\_Sample\_011046840, Unigene3218\_Sample\_011046840, Unigene13661\_Sample\_011046840, Unigene39081\_Sample\_011046840, Unigene40246\_Sample\_011046840, Unigene43068\_Sample\_011046840, Unigene43101\_Sample\_011046840, Unigene24869\_Sample\_011046840, Unigene33873\_Sample\_011046840, Unigene4139\_Sample\_011046840, Unigene11279\_Sample\_011046840, Unigene18933\_Sample\_011046840, Unigene25548\_Sample\_011046840, Unigene26785\_Sample\_011046840, Unigene31880\_Sample\_011046840, Unigene35532\_Sample\_011046840, Unigene39958\_Sample\_011046840, Unigene42008\_Sample\_011046840, Unigene43154\_Sample\_011046840, Unigene1368\_Sample\_011046840, Unigene6374\_Sample\_011046840, Unigene10749\_Sample\_011046840, Unigene14237\_Sample\_011046840, Unigene18009\_Sample\_011046840, Unigene20896\_Sample\_011046840, Unigene29660\_Sample\_011046840, Unigene33870\_Sample\_011046840, Unigene41475\_Sample\_011046840, Unigene42811\_Sample\_011046840, Unigene5726\_Sample\_011046840, Unigene21176\_Sample\_011046840, Unigene1317\_Sample\_011046840, Unigene36577\_Sample\_011046840, Unigene41704\_Sample\_011046840, Unigene1568\_Sample\_011046840, Unigene14930\_Sample\_011046840, Unigene16035\_Sample\_011046840, Unigene29621\_Sample\_011046840, Unigene32265\_Sample\_011046840, Unigene41410\_Sample\_011046840, Unigene19593\_Sample\_011046840, Unigene24889\_Sample\_011046840, Unigene37186\_Sample\_011046840, Unigene40053\_Sample\_011046840 |
| 17 | Basal cell carcinoma Back to summary table | Unigene4506\_Sample\_011046840, Unigene5429\_Sample\_011046840, Unigene41164\_Sample\_011046840, Unigene41242\_Sample\_011046840, Unigene41989\_Sample\_011046840, Unigene42654\_Sample\_011046840, Unigene43118\_Sample\_011046840, Unigene43124\_Sample\_011046840, Unigene43174\_Sample\_011046840, Unigene43211\_Sample\_011046840, Unigene4254\_Sample\_011046840, Unigene4668\_Sample\_011046840, Unigene4779\_Sample\_011046840, Unigene6245\_Sample\_011046840, Unigene7303\_Sample\_011046840, Unigene7893\_Sample\_011046840, Unigene16630\_Sample\_011046840, Unigene18652\_Sample\_011046840, Unigene22845\_Sample\_011046840, Unigene24751\_Sample\_011046840, Unigene30430\_Sample\_011046840, Unigene32730\_Sample\_011046840, Unigene38435\_Sample\_011046840, Unigene38612\_Sample\_011046840, Unigene40105\_Sample\_011046840, Unigene40332\_Sample\_011046840, Unigene41234\_Sample\_011046840, Unigene41949\_Sample\_011046840, Unigene42364\_Sample\_011046840, Unigene43222\_Sample\_011046840, Unigene43301\_Sample\_011046840, Unigene43492\_Sample\_011046840, Unigene4972\_Sample\_011046840, Unigene2604\_Sample\_011046840, Unigene21306\_Sample\_011046840, Unigene29542\_Sample\_011046840, Unigene31699\_Sample\_011046840, Unigene41130\_Sample\_011046840, Unigene7429\_Sample\_011046840, Unigene33501\_Sample\_011046840, Unigene34871\_Sample\_011046840, Unigene35839\_Sample\_011046840, Unigene35981\_Sample\_011046840, Unigene37994\_Sample\_011046840, Unigene41303\_Sample\_011046840, Unigene42488\_Sample\_011046840, Unigene35367\_Sample\_011046840, Unigene31705\_Sample\_011046840, Unigene39891\_Sample\_011046840, Unigene6629\_Sample\_011046840, Unigene15970\_Sample\_011046840, Unigene32732\_Sample\_011046840, Unigene43240\_Sample\_011046840, Unigene1495\_Sample\_011046840, Unigene19400\_Sample\_011046840, Unigene25523\_Sample\_011046840, Unigene29526\_Sample\_011046840, Unigene35833\_Sample\_011046840, Unigene38611\_Sample\_011046840, Unigene38882\_Sample\_011046840, Unigene39771\_Sample\_011046840, Unigene40539\_Sample\_011046840, Unigene14773\_Sample\_011046840, Unigene34677\_Sample\_011046840, Unigene10037\_Sample\_011046840, Unigene40680\_Sample\_011046840, Unigene34920\_Sample\_011046840, Unigene36617\_Sample\_011046840, Unigene40896\_Sample\_011046840, Unigene43308\_Sample\_011046840, Unigene24378\_Sample\_011046840, Unigene24508\_Sample\_011046840 |
| 18 | Leishmaniasis (no map in kegg database) Back to summary table | Unigene6551\_Sample\_011046840, Unigene7341\_Sample\_011046840, Unigene9987\_Sample\_011046840, Unigene25850\_Sample\_011046840, Unigene40029\_Sample\_011046840, Unigene40471\_Sample\_011046840, Unigene42127\_Sample\_011046840, Unigene42639\_Sample\_011046840, Unigene42759\_Sample\_011046840, Unigene43441\_Sample\_011046840, Unigene43496\_Sample\_011046840, Unigene2986\_Sample\_011046840, Unigene10748\_Sample\_011046840, Unigene34085\_Sample\_011046840, Unigene35122\_Sample\_011046840, Unigene43499\_Sample\_011046840, Unigene8133\_Sample\_011046840, Unigene25969\_Sample\_011046840, Unigene43466\_Sample\_011046840, Unigene7408\_Sample\_011046840, Unigene13661\_Sample\_011046840, Unigene39081\_Sample\_011046840, Unigene43101\_Sample\_011046840, Unigene33873\_Sample\_011046840, Unigene43154\_Sample\_011046840, Unigene29660\_Sample\_011046840, Unigene38423\_Sample\_011046840, Unigene41410\_Sample\_011046840 |
| 19 | Pantothenate and CoA biosynthesis Back to summary table | Unigene41389\_Sample\_011046840, Unigene41912\_Sample\_011046840, Unigene42259\_Sample\_011046840, Unigene6360\_Sample\_011046840, Unigene8023\_Sample\_011046840, Unigene24005\_Sample\_011046840, Unigene27178\_Sample\_011046840, Unigene29611\_Sample\_011046840, Unigene32708\_Sample\_011046840, Unigene33287\_Sample\_011046840, Unigene36197\_Sample\_011046840, Unigene3631\_Sample\_011046840, Unigene34532\_Sample\_011046840, Unigene36791\_Sample\_011046840, Unigene455\_Sample\_011046840, Unigene30281\_Sample\_011046840, Unigene29405\_Sample\_011046840 |
| 20 | Progesterone-mediated oocyte maturation Back to summary table | Unigene3123\_Sample\_011046840, Unigene7525\_Sample\_011046840, Unigene7647\_Sample\_011046840, Unigene8055\_Sample\_011046840, Unigene13399\_Sample\_011046840, Unigene15939\_Sample\_011046840, Unigene33337\_Sample\_011046840, Unigene33667\_Sample\_011046840, Unigene41132\_Sample\_011046840, Unigene42127\_Sample\_011046840, Unigene42759\_Sample\_011046840, Unigene42859\_Sample\_011046840, Unigene43439\_Sample\_011046840, Unigene2986\_Sample\_011046840, Unigene4320\_Sample\_011046840, Unigene4497\_Sample\_011046840, Unigene8203\_Sample\_011046840, Unigene14078\_Sample\_011046840, Unigene18467\_Sample\_011046840, Unigene27185\_Sample\_011046840, Unigene29711\_Sample\_011046840, Unigene29864\_Sample\_011046840, Unigene30539\_Sample\_011046840, Unigene32593\_Sample\_011046840, Unigene32679\_Sample\_011046840, Unigene34974\_Sample\_011046840, Unigene35491\_Sample\_011046840, Unigene36846\_Sample\_011046840, Unigene36911\_Sample\_011046840, Unigene37340\_Sample\_011046840, Unigene37890\_Sample\_011046840, Unigene38617\_Sample\_011046840, Unigene40128\_Sample\_011046840, Unigene40208\_Sample\_011046840, Unigene40685\_Sample\_011046840, Unigene41401\_Sample\_011046840, Unigene41444\_Sample\_011046840, Unigene41584\_Sample\_011046840, Unigene41621\_Sample\_011046840, Unigene41673\_Sample\_011046840, Unigene42065\_Sample\_011046840, Unigene42164\_Sample\_011046840, Unigene43162\_Sample\_011046840, Unigene43213\_Sample\_011046840, Unigene43395\_Sample\_011046840, Unigene43481\_Sample\_011046840, Unigene43635\_Sample\_011046840, Unigene2541\_Sample\_011046840, Unigene3954\_Sample\_011046840, Unigene27034\_Sample\_011046840, Unigene33638\_Sample\_011046840, Unigene38540\_Sample\_011046840, Unigene38847\_Sample\_011046840, Unigene41114\_Sample\_011046840, Unigene43149\_Sample\_011046840, Unigene4268\_Sample\_011046840, Unigene6921\_Sample\_011046840, Unigene7605\_Sample\_011046840, Unigene19991\_Sample\_011046840, Unigene24747\_Sample\_011046840, Unigene33396\_Sample\_011046840, Unigene33840\_Sample\_011046840, Unigene39778\_Sample\_011046840, Unigene41403\_Sample\_011046840, Unigene43111\_Sample\_011046840, Unigene3872\_Sample\_011046840, Unigene14373\_Sample\_011046840, Unigene22321\_Sample\_011046840, Unigene25450\_Sample\_011046840, Unigene28708\_Sample\_011046840, Unigene31945\_Sample\_011046840, Unigene33849\_Sample\_011046840, Unigene36800\_Sample\_011046840, Unigene38366\_Sample\_011046840, Unigene38504\_Sample\_011046840, Unigene42685\_Sample\_011046840, Unigene43068\_Sample\_011046840, Unigene16307\_Sample\_011046840, Unigene22643\_Sample\_011046840, Unigene24869\_Sample\_011046840, Unigene33222\_Sample\_011046840, Unigene33873\_Sample\_011046840, Unigene36619\_Sample\_011046840, Unigene37128\_Sample\_011046840, Unigene41125\_Sample\_011046840, Unigene42022\_Sample\_011046840, Unigene42693\_Sample\_011046840, Unigene115\_Sample\_011046840, Unigene3486\_Sample\_011046840, Unigene4139\_Sample\_011046840, Unigene16429\_Sample\_011046840, Unigene18933\_Sample\_011046840, Unigene21758\_Sample\_011046840, Unigene26785\_Sample\_011046840, Unigene27378\_Sample\_011046840, Unigene31880\_Sample\_011046840, Unigene32645\_Sample\_011046840, Unigene38902\_Sample\_011046840, Unigene39503\_Sample\_011046840, Unigene41425\_Sample\_011046840, Unigene41691\_Sample\_011046840, Unigene42008\_Sample\_011046840, Unigene43154\_Sample\_011046840, Unigene5852\_Sample\_011046840, Unigene12320\_Sample\_011046840, Unigene37680\_Sample\_011046840, Unigene40665\_Sample\_011046840, Unigene1368\_Sample\_011046840, Unigene14237\_Sample\_011046840, Unigene17776\_Sample\_011046840, Unigene20050\_Sample\_011046840, Unigene20896\_Sample\_011046840, Unigene24411\_Sample\_011046840, Unigene32355\_Sample\_011046840, Unigene33161\_Sample\_011046840, Unigene33870\_Sample\_011046840, Unigene36291\_Sample\_011046840, Unigene38455\_Sample\_011046840, Unigene42242\_Sample\_011046840, Unigene42811\_Sample\_011046840, Unigene2031\_Sample\_011046840, Unigene5726\_Sample\_011046840, Unigene21176\_Sample\_011046840, Unigene24021\_Sample\_011046840, Unigene29181\_Sample\_011046840, Unigene30829\_Sample\_011046840, Unigene31266\_Sample\_011046840, Unigene33722\_Sample\_011046840, Unigene36385\_Sample\_011046840, Unigene37505\_Sample\_011046840, Unigene40019\_Sample\_011046840, Unigene3861\_Sample\_011046840, Unigene10037\_Sample\_011046840, Unigene17478\_Sample\_011046840, Unigene20949\_Sample\_011046840, Unigene22924\_Sample\_011046840, Unigene41704\_Sample\_011046840, Unigene42173\_Sample\_011046840, Unigene1568\_Sample\_011046840, Unigene10612\_Sample\_011046840, Unigene16035\_Sample\_011046840, Unigene20420\_Sample\_011046840, Unigene20430\_Sample\_011046840, Unigene26500\_Sample\_011046840, Unigene29411\_Sample\_011046840, Unigene31090\_Sample\_011046840, Unigene32265\_Sample\_011046840, Unigene39224\_Sample\_011046840, Unigene41690\_Sample\_011046840, Unigene42473\_Sample\_011046840, Unigene2308\_Sample\_011046840, Unigene19593\_Sample\_011046840, Unigene37186\_Sample\_011046840, Unigene38610\_Sample\_011046840 |
| 21 | Renal cell carcinoma Back to summary table | Unigene2280\_Sample\_011046840, Unigene4790\_Sample\_011046840, Unigene4993\_Sample\_011046840, Unigene6378\_Sample\_011046840, Unigene15939\_Sample\_011046840, Unigene25743\_Sample\_011046840, Unigene26069\_Sample\_011046840, Unigene26863\_Sample\_011046840, Unigene32137\_Sample\_011046840, Unigene34317\_Sample\_011046840, Unigene36978\_Sample\_011046840, Unigene39096\_Sample\_011046840, Unigene43556\_Sample\_011046840, Unigene2986\_Sample\_011046840, Unigene10748\_Sample\_011046840, Unigene30694\_Sample\_011046840, Unigene34085\_Sample\_011046840, Unigene34658\_Sample\_011046840, Unigene34974\_Sample\_011046840, Unigene36317\_Sample\_011046840, Unigene37858\_Sample\_011046840, Unigene38617\_Sample\_011046840, Unigene38866\_Sample\_011046840, Unigene39285\_Sample\_011046840, Unigene39843\_Sample\_011046840, Unigene39883\_Sample\_011046840, Unigene40208\_Sample\_011046840, Unigene41370\_Sample\_011046840, Unigene41584\_Sample\_011046840, Unigene41915\_Sample\_011046840, Unigene41926\_Sample\_011046840, Unigene42164\_Sample\_011046840, Unigene42197\_Sample\_011046840, Unigene43342\_Sample\_011046840, Unigene976\_Sample\_011046840, Unigene33835\_Sample\_011046840, Unigene38847\_Sample\_011046840, Unigene43149\_Sample\_011046840, Unigene38648\_Sample\_011046840, Unigene7408\_Sample\_011046840, Unigene7625\_Sample\_011046840, Unigene14485\_Sample\_011046840, Unigene19944\_Sample\_011046840, Unigene20325\_Sample\_011046840, Unigene23045\_Sample\_011046840, Unigene24222\_Sample\_011046840, Unigene26542\_Sample\_011046840, Unigene28708\_Sample\_011046840, Unigene31079\_Sample\_011046840, Unigene36320\_Sample\_011046840, Unigene39621\_Sample\_011046840, Unigene40659\_Sample\_011046840, Unigene41977\_Sample\_011046840, Unigene42079\_Sample\_011046840, Unigene42487\_Sample\_011046840, Unigene43068\_Sample\_011046840, Unigene1680\_Sample\_011046840, Unigene21908\_Sample\_011046840, Unigene24869\_Sample\_011046840, Unigene26621\_Sample\_011046840, Unigene35901\_Sample\_011046840, Unigene37954\_Sample\_011046840, Unigene4139\_Sample\_011046840, Unigene12147\_Sample\_011046840, Unigene13839\_Sample\_011046840, Unigene14255\_Sample\_011046840, Unigene16429\_Sample\_011046840, Unigene18354\_Sample\_011046840, Unigene18933\_Sample\_011046840, Unigene26785\_Sample\_011046840, Unigene27378\_Sample\_011046840, Unigene28751\_Sample\_011046840, Unigene29670\_Sample\_011046840, Unigene30226\_Sample\_011046840, Unigene31880\_Sample\_011046840, Unigene32620\_Sample\_011046840, Unigene39503\_Sample\_011046840, Unigene40189\_Sample\_011046840, Unigene42008\_Sample\_011046840, Unigene43154\_Sample\_011046840, Unigene1368\_Sample\_011046840, Unigene8865\_Sample\_011046840, Unigene20896\_Sample\_011046840, Unigene29660\_Sample\_011046840, Unigene33870\_Sample\_011046840, Unigene34777\_Sample\_011046840, Unigene36190\_Sample\_011046840, Unigene42811\_Sample\_011046840, Unigene2209\_Sample\_011046840, Unigene5726\_Sample\_011046840, Unigene13067\_Sample\_011046840, Unigene19127\_Sample\_011046840, Unigene21176\_Sample\_011046840, Unigene25325\_Sample\_011046840, Unigene27623\_Sample\_011046840, Unigene30501\_Sample\_011046840, Unigene37207\_Sample\_011046840, Unigene41560\_Sample\_011046840, Unigene12908\_Sample\_011046840, Unigene14598\_Sample\_011046840, Unigene24184\_Sample\_011046840, Unigene26628\_Sample\_011046840, Unigene41704\_Sample\_011046840, Unigene14930\_Sample\_011046840, Unigene16035\_Sample\_011046840, Unigene20420\_Sample\_011046840, Unigene32265\_Sample\_011046840, Unigene41521\_Sample\_011046840, Unigene36545\_Sample\_011046840 |
| 22 | Pathways in cancer Back to summary table | Unigene463\_Sample\_011046840, Unigene880\_Sample\_011046840, Unigene1345\_Sample\_011046840, Unigene2132\_Sample\_011046840, Unigene4506\_Sample\_011046840, Unigene4790\_Sample\_011046840, Unigene4981\_Sample\_011046840, Unigene5429\_Sample\_011046840, Unigene6209\_Sample\_011046840, Unigene6378\_Sample\_011046840, Unigene7767\_Sample\_011046840, Unigene8069\_Sample\_011046840, Unigene8154\_Sample\_011046840, Unigene8167\_Sample\_011046840, Unigene9987\_Sample\_011046840, Unigene10035\_Sample\_011046840, Unigene15939\_Sample\_011046840, Unigene21166\_Sample\_011046840, Unigene24311\_Sample\_011046840, Unigene25743\_Sample\_011046840, Unigene25850\_Sample\_011046840, Unigene26069\_Sample\_011046840, Unigene26863\_Sample\_011046840, Unigene27844\_Sample\_011046840, Unigene29592\_Sample\_011046840, Unigene30862\_Sample\_011046840, Unigene32137\_Sample\_011046840, Unigene35439\_Sample\_011046840, Unigene37137\_Sample\_011046840, Unigene37793\_Sample\_011046840, Unigene38706\_Sample\_011046840, Unigene40260\_Sample\_011046840, Unigene40446\_Sample\_011046840, Unigene41164\_Sample\_011046840, Unigene41242\_Sample\_011046840, Unigene41355\_Sample\_011046840, Unigene41702\_Sample\_011046840, Unigene41989\_Sample\_011046840, Unigene42639\_Sample\_011046840, Unigene42654\_Sample\_011046840, Unigene43118\_Sample\_011046840, Unigene43124\_Sample\_011046840, Unigene43174\_Sample\_011046840, Unigene43176\_Sample\_011046840, Unigene43211\_Sample\_011046840, Unigene43311\_Sample\_011046840, Unigene43441\_Sample\_011046840, Unigene43445\_Sample\_011046840, Unigene43556\_Sample\_011046840, Unigene43623\_Sample\_011046840, Unigene2261\_Sample\_011046840, Unigene2615\_Sample\_011046840, Unigene2986\_Sample\_011046840, Unigene3580\_Sample\_011046840, Unigene3776\_Sample\_011046840, Unigene4033\_Sample\_011046840, Unigene4082\_Sample\_011046840, Unigene4101\_Sample\_011046840, Unigene4254\_Sample\_011046840, Unigene4320\_Sample\_011046840, Unigene4497\_Sample\_011046840, Unigene4668\_Sample\_011046840, Unigene4779\_Sample\_011046840, Unigene5197\_Sample\_011046840, Unigene5389\_Sample\_011046840, Unigene5425\_Sample\_011046840, Unigene6245\_Sample\_011046840, Unigene6593\_Sample\_011046840, Unigene6863\_Sample\_011046840, Unigene7303\_Sample\_011046840, Unigene7349\_Sample\_011046840, Unigene7529\_Sample\_011046840, Unigene7537\_Sample\_011046840, Unigene7819\_Sample\_011046840, Unigene7893\_Sample\_011046840, Unigene7979\_Sample\_011046840, Unigene8009\_Sample\_011046840, Unigene8138\_Sample\_011046840, Unigene11592\_Sample\_011046840, Unigene15111\_Sample\_011046840, Unigene16630\_Sample\_011046840, Unigene18652\_Sample\_011046840, Unigene22845\_Sample\_011046840, Unigene23143\_Sample\_011046840, Unigene24651\_Sample\_011046840, Unigene24698\_Sample\_011046840, Unigene24751\_Sample\_011046840, Unigene26575\_Sample\_011046840, Unigene27254\_Sample\_011046840, Unigene27291\_Sample\_011046840, Unigene28752\_Sample\_011046840, Unigene29711\_Sample\_011046840, Unigene30370\_Sample\_011046840, Unigene30430\_Sample\_011046840, Unigene30539\_Sample\_011046840, Unigene30694\_Sample\_011046840, Unigene30720\_Sample\_011046840, Unigene32380\_Sample\_011046840, Unigene32467\_Sample\_011046840, Unigene32730\_Sample\_011046840, Unigene33208\_Sample\_011046840, Unigene33370\_Sample\_011046840, Unigene33815\_Sample\_011046840, Unigene34035\_Sample\_011046840, Unigene34658\_Sample\_011046840, Unigene34974\_Sample\_011046840, Unigene36347\_Sample\_011046840, Unigene36690\_Sample\_011046840, Unigene37858\_Sample\_011046840, Unigene38435\_Sample\_011046840, Unigene38534\_Sample\_011046840, Unigene38612\_Sample\_011046840, Unigene38617\_Sample\_011046840, Unigene38866\_Sample\_011046840, Unigene39237\_Sample\_011046840, Unigene39282\_Sample\_011046840, Unigene39285\_Sample\_011046840, Unigene39437\_Sample\_011046840, Unigene39883\_Sample\_011046840, Unigene40105\_Sample\_011046840, Unigene40208\_Sample\_011046840, Unigene40332\_Sample\_011046840, Unigene40657\_Sample\_011046840, Unigene41234\_Sample\_011046840, Unigene41237\_Sample\_011046840, Unigene41501\_Sample\_011046840, Unigene41584\_Sample\_011046840, Unigene41658\_Sample\_011046840, Unigene41926\_Sample\_011046840, Unigene41949\_Sample\_011046840, Unigene42164\_Sample\_011046840, Unigene42327\_Sample\_011046840, Unigene42336\_Sample\_011046840, Unigene42364\_Sample\_011046840, Unigene42479\_Sample\_011046840, Unigene42716\_Sample\_011046840, Unigene43002\_Sample\_011046840, Unigene43210\_Sample\_011046840, Unigene43222\_Sample\_011046840, Unigene43301\_Sample\_011046840, Unigene43317\_Sample\_011046840, Unigene43492\_Sample\_011046840, Unigene43502\_Sample\_011046840, Unigene43635\_Sample\_011046840, Unigene30\_Sample\_011046840, Unigene976\_Sample\_011046840, Unigene3608\_Sample\_011046840, Unigene3954\_Sample\_011046840, Unigene4972\_Sample\_011046840, Unigene7684\_Sample\_011046840, Unigene7853\_Sample\_011046840, Unigene8058\_Sample\_011046840, Unigene8133\_Sample\_011046840, Unigene8372\_Sample\_011046840, Unigene8904\_Sample\_011046840, Unigene25969\_Sample\_011046840, Unigene26548\_Sample\_011046840, Unigene32594\_Sample\_011046840, Unigene32596\_Sample\_011046840, Unigene33835\_Sample\_011046840, Unigene33954\_Sample\_011046840, Unigene33983\_Sample\_011046840, Unigene35961\_Sample\_011046840, Unigene38834\_Sample\_011046840, Unigene38847\_Sample\_011046840, Unigene40065\_Sample\_011046840, Unigene42204\_Sample\_011046840, Unigene43149\_Sample\_011046840, Unigene169\_Sample\_011046840, Unigene2604\_Sample\_011046840, Unigene3161\_Sample\_011046840, Unigene4268\_Sample\_011046840, Unigene6322\_Sample\_011046840, Unigene6921\_Sample\_011046840, Unigene16050\_Sample\_011046840, Unigene21306\_Sample\_011046840, Unigene22181\_Sample\_011046840, Unigene28433\_Sample\_011046840, Unigene28604\_Sample\_011046840, Unigene29542\_Sample\_011046840, Unigene31699\_Sample\_011046840, Unigene33984\_Sample\_011046840, Unigene35727\_Sample\_011046840, Unigene37084\_Sample\_011046840, Unigene37792\_Sample\_011046840, Unigene38648\_Sample\_011046840, Unigene39778\_Sample\_011046840, Unigene41130\_Sample\_011046840, Unigene41734\_Sample\_011046840, Unigene43466\_Sample\_011046840, Unigene2808\_Sample\_011046840, Unigene4807\_Sample\_011046840, Unigene4818\_Sample\_011046840, Unigene7429\_Sample\_011046840, Unigene7625\_Sample\_011046840, Unigene7950\_Sample\_011046840, Unigene16958\_Sample\_011046840, Unigene19401\_Sample\_011046840, Unigene19944\_Sample\_011046840, Unigene20325\_Sample\_011046840, Unigene22423\_Sample\_011046840, Unigene23045\_Sample\_011046840, Unigene24669\_Sample\_011046840, Unigene28708\_Sample\_011046840, Unigene31860\_Sample\_011046840, Unigene33313\_Sample\_011046840, Unigene33501\_Sample\_011046840, Unigene34871\_Sample\_011046840, Unigene35699\_Sample\_011046840, Unigene35839\_Sample\_011046840, Unigene35981\_Sample\_011046840, Unigene36320\_Sample\_011046840, Unigene37994\_Sample\_011046840, Unigene38655\_Sample\_011046840, Unigene38694\_Sample\_011046840, Unigene39562\_Sample\_011046840, Unigene39625\_Sample\_011046840, Unigene40659\_Sample\_011046840, Unigene40784\_Sample\_011046840, Unigene40888\_Sample\_011046840, Unigene41303\_Sample\_011046840, Unigene41977\_Sample\_011046840, Unigene42298\_Sample\_011046840, Unigene42488\_Sample\_011046840, Unigene43068\_Sample\_011046840, Unigene43497\_Sample\_011046840, Unigene1680\_Sample\_011046840, Unigene8240\_Sample\_011046840, Unigene14379\_Sample\_011046840, Unigene21144\_Sample\_011046840, Unigene24869\_Sample\_011046840, Unigene26621\_Sample\_011046840, Unigene30155\_Sample\_011046840, Unigene31572\_Sample\_011046840, Unigene35367\_Sample\_011046840, Unigene35901\_Sample\_011046840, Unigene38216\_Sample\_011046840, Unigene38245\_Sample\_011046840, Unigene38301\_Sample\_011046840, Unigene39525\_Sample\_011046840, Unigene42345\_Sample\_011046840, Unigene42346\_Sample\_011046840, Unigene4139\_Sample\_011046840, Unigene7073\_Sample\_011046840, Unigene7125\_Sample\_011046840, Unigene9938\_Sample\_011046840, Unigene12153\_Sample\_011046840, Unigene13197\_Sample\_011046840, Unigene13839\_Sample\_011046840, Unigene16429\_Sample\_011046840, Unigene18354\_Sample\_011046840, Unigene18933\_Sample\_011046840, Unigene20787\_Sample\_011046840, Unigene24271\_Sample\_011046840, Unigene25551\_Sample\_011046840, Unigene26785\_Sample\_011046840, Unigene27378\_Sample\_011046840, Unigene28751\_Sample\_011046840, Unigene30226\_Sample\_011046840, Unigene31705\_Sample\_011046840, Unigene31880\_Sample\_011046840, Unigene32620\_Sample\_011046840, Unigene35787\_Sample\_011046840, Unigene36897\_Sample\_011046840, Unigene37702\_Sample\_011046840, Unigene38000\_Sample\_011046840, Unigene39503\_Sample\_011046840, Unigene39891\_Sample\_011046840, Unigene40189\_Sample\_011046840, Unigene40648\_Sample\_011046840, Unigene41033\_Sample\_011046840, Unigene42008\_Sample\_011046840, Unigene42385\_Sample\_011046840, Unigene43102\_Sample\_011046840, Unigene43154\_Sample\_011046840, Unigene43590\_Sample\_011046840, Unigene6629\_Sample\_011046840, Unigene15970\_Sample\_011046840, Unigene20803\_Sample\_011046840, Unigene32732\_Sample\_011046840, Unigene33553\_Sample\_011046840, Unigene36362\_Sample\_011046840, Unigene42677\_Sample\_011046840, Unigene43240\_Sample\_011046840, Unigene1368\_Sample\_011046840, Unigene1495\_Sample\_011046840, Unigene4849\_Sample\_011046840, Unigene8865\_Sample\_011046840, Unigene14237\_Sample\_011046840, Unigene15053\_Sample\_011046840, Unigene17776\_Sample\_011046840, Unigene19400\_Sample\_011046840, Unigene20896\_Sample\_011046840, Unigene22073\_Sample\_011046840, Unigene24124\_Sample\_011046840, Unigene25523\_Sample\_011046840, Unigene29526\_Sample\_011046840, Unigene29660\_Sample\_011046840, Unigene30246\_Sample\_011046840, Unigene30941\_Sample\_011046840, Unigene33870\_Sample\_011046840, Unigene34777\_Sample\_011046840, Unigene35833\_Sample\_011046840, Unigene36190\_Sample\_011046840, Unigene38611\_Sample\_011046840, Unigene38882\_Sample\_011046840, Unigene39771\_Sample\_011046840, Unigene40539\_Sample\_011046840, Unigene40855\_Sample\_011046840, Unigene42811\_Sample\_011046840, Unigene5726\_Sample\_011046840, Unigene14072\_Sample\_011046840, Unigene14773\_Sample\_011046840, Unigene18703\_Sample\_011046840, Unigene19127\_Sample\_011046840, Unigene19918\_Sample\_011046840, Unigene21176\_Sample\_011046840, Unigene25325\_Sample\_011046840, Unigene27623\_Sample\_011046840, Unigene30501\_Sample\_011046840, Unigene30821\_Sample\_011046840, Unigene31320\_Sample\_011046840, Unigene34360\_Sample\_011046840, Unigene34677\_Sample\_011046840, Unigene37207\_Sample\_011046840, Unigene38423\_Sample\_011046840, Unigene40056\_Sample\_011046840, Unigene41104\_Sample\_011046840, Unigene41560\_Sample\_011046840, Unigene10037\_Sample\_011046840, Unigene12908\_Sample\_011046840, Unigene14598\_Sample\_011046840, Unigene20949\_Sample\_011046840, Unigene21815\_Sample\_011046840, Unigene26628\_Sample\_011046840, Unigene30201\_Sample\_011046840, Unigene30685\_Sample\_011046840, Unigene33524\_Sample\_011046840, Unigene33692\_Sample\_011046840, Unigene35972\_Sample\_011046840, Unigene38066\_Sample\_011046840, Unigene38690\_Sample\_011046840, Unigene39360\_Sample\_011046840, Unigene39584\_Sample\_011046840, Unigene40680\_Sample\_011046840, Unigene41704\_Sample\_011046840, Unigene1256\_Sample\_011046840, Unigene1568\_Sample\_011046840, Unigene4276\_Sample\_011046840, Unigene9721\_Sample\_011046840, Unigene11201\_Sample\_011046840, Unigene14930\_Sample\_011046840, Unigene16035\_Sample\_011046840, Unigene19481\_Sample\_011046840, Unigene20420\_Sample\_011046840, Unigene24954\_Sample\_011046840, Unigene26719\_Sample\_011046840, Unigene31090\_Sample\_011046840, Unigene32265\_Sample\_011046840, Unigene34920\_Sample\_011046840, Unigene34975\_Sample\_011046840, Unigene36617\_Sample\_011046840, Unigene37491\_Sample\_011046840, Unigene37909\_Sample\_011046840, Unigene38841\_Sample\_011046840, Unigene40896\_Sample\_011046840, Unigene41201\_Sample\_011046840, Unigene41410\_Sample\_011046840, Unigene41521\_Sample\_011046840, Unigene43308\_Sample\_011046840, Unigene5544\_Sample\_011046840, Unigene19593\_Sample\_011046840, Unigene24378\_Sample\_011046840, Unigene24508\_Sample\_011046840, Unigene37186\_Sample\_011046840, Unigene38088\_Sample\_011046840, Unigene41562\_Sample\_011046840 |
| 23 | Endometrial cancer Back to summary table | Unigene4790\_Sample\_011046840, Unigene6551\_Sample\_011046840, Unigene7744\_Sample\_011046840, Unigene15939\_Sample\_011046840, Unigene41242\_Sample\_011046840, Unigene43118\_Sample\_011046840, Unigene43211\_Sample\_011046840, Unigene2418\_Sample\_011046840, Unigene2986\_Sample\_011046840, Unigene4254\_Sample\_011046840, Unigene4779\_Sample\_011046840, Unigene7979\_Sample\_011046840, Unigene8009\_Sample\_011046840, Unigene11592\_Sample\_011046840, Unigene24698\_Sample\_011046840, Unigene27291\_Sample\_011046840, Unigene28752\_Sample\_011046840, Unigene30370\_Sample\_011046840, Unigene33208\_Sample\_011046840, Unigene33370\_Sample\_011046840, Unigene34974\_Sample\_011046840, Unigene36690\_Sample\_011046840, Unigene38435\_Sample\_011046840, Unigene38612\_Sample\_011046840, Unigene38617\_Sample\_011046840, Unigene38866\_Sample\_011046840, Unigene39237\_Sample\_011046840, Unigene40208\_Sample\_011046840, Unigene41289\_Sample\_011046840, Unigene41584\_Sample\_011046840, Unigene41949\_Sample\_011046840, Unigene42164\_Sample\_011046840, Unigene43301\_Sample\_011046840, Unigene43492\_Sample\_011046840, Unigene33983\_Sample\_011046840, Unigene38834\_Sample\_011046840, Unigene38847\_Sample\_011046840, Unigene40065\_Sample\_011046840, Unigene42204\_Sample\_011046840, Unigene43149\_Sample\_011046840, Unigene21306\_Sample\_011046840, Unigene28604\_Sample\_011046840, Unigene29542\_Sample\_011046840, Unigene31699\_Sample\_011046840, Unigene35790\_Sample\_011046840, Unigene19401\_Sample\_011046840, Unigene20325\_Sample\_011046840, Unigene23045\_Sample\_011046840, Unigene28708\_Sample\_011046840, Unigene34871\_Sample\_011046840, Unigene35699\_Sample\_011046840, Unigene35981\_Sample\_011046840, Unigene36320\_Sample\_011046840, Unigene40784\_Sample\_011046840, Unigene41303\_Sample\_011046840, Unigene42509\_Sample\_011046840, Unigene43068\_Sample\_011046840, Unigene24869\_Sample\_011046840, Unigene30860\_Sample\_011046840, Unigene4139\_Sample\_011046840, Unigene12153\_Sample\_011046840, Unigene13197\_Sample\_011046840, Unigene16429\_Sample\_011046840, Unigene18933\_Sample\_011046840, Unigene26785\_Sample\_011046840, Unigene27378\_Sample\_011046840, Unigene28751\_Sample\_011046840, Unigene31705\_Sample\_011046840, Unigene31880\_Sample\_011046840, Unigene32098\_Sample\_011046840, Unigene35787\_Sample\_011046840, Unigene39503\_Sample\_011046840, Unigene40189\_Sample\_011046840, Unigene42008\_Sample\_011046840, Unigene42189\_Sample\_011046840, Unigene43154\_Sample\_011046840, Unigene43590\_Sample\_011046840, Unigene1368\_Sample\_011046840, Unigene19400\_Sample\_011046840, Unigene20896\_Sample\_011046840, Unigene25523\_Sample\_011046840, Unigene29526\_Sample\_011046840, Unigene30941\_Sample\_011046840, Unigene33870\_Sample\_011046840, Unigene38882\_Sample\_011046840, Unigene42811\_Sample\_011046840, Unigene5726\_Sample\_011046840, Unigene21176\_Sample\_011046840, Unigene27623\_Sample\_011046840, Unigene41560\_Sample\_011046840, Unigene10037\_Sample\_011046840, Unigene35972\_Sample\_011046840, Unigene41704\_Sample\_011046840, Unigene16035\_Sample\_011046840, Unigene19481\_Sample\_011046840, Unigene20420\_Sample\_011046840, Unigene32265\_Sample\_011046840, Unigene33028\_Sample\_011046840, Unigene33393\_Sample\_011046840 |
| 24 | Adipocytokine signaling pathway Back to summary table | Unigene409\_Sample\_011046840, Unigene4464\_Sample\_011046840, Unigene5241\_Sample\_011046840, Unigene24311\_Sample\_011046840, Unigene29452\_Sample\_011046840, Unigene29592\_Sample\_011046840, Unigene33573\_Sample\_011046840, Unigene38021\_Sample\_011046840, Unigene39096\_Sample\_011046840, Unigene42068\_Sample\_011046840, Unigene1655\_Sample\_011046840, Unigene2230\_Sample\_011046840, Unigene2261\_Sample\_011046840, Unigene3724\_Sample\_011046840, Unigene3948\_Sample\_011046840, Unigene4082\_Sample\_011046840, Unigene4536\_Sample\_011046840, Unigene4874\_Sample\_011046840, Unigene5425\_Sample\_011046840, Unigene5453\_Sample\_011046840, Unigene9985\_Sample\_011046840, Unigene10748\_Sample\_011046840, Unigene25801\_Sample\_011046840, Unigene28956\_Sample\_011046840, Unigene29711\_Sample\_011046840, Unigene30539\_Sample\_011046840, Unigene30720\_Sample\_011046840, Unigene31398\_Sample\_011046840, Unigene33926\_Sample\_011046840, Unigene34085\_Sample\_011046840, Unigene38281\_Sample\_011046840, Unigene38923\_Sample\_011046840, Unigene39285\_Sample\_011046840, Unigene40958\_Sample\_011046840, Unigene41039\_Sample\_011046840, Unigene42164\_Sample\_011046840, Unigene43100\_Sample\_011046840, Unigene43484\_Sample\_011046840, Unigene38847\_Sample\_011046840, Unigene6921\_Sample\_011046840, Unigene7408\_Sample\_011046840, Unigene8134\_Sample\_011046840, Unigene30119\_Sample\_011046840, Unigene31285\_Sample\_011046840, Unigene34117\_Sample\_011046840, Unigene36139\_Sample\_011046840, Unigene39505\_Sample\_011046840, Unigene1680\_Sample\_011046840, Unigene21908\_Sample\_011046840, Unigene26711\_Sample\_011046840, Unigene3170\_Sample\_011046840, Unigene4139\_Sample\_011046840, Unigene7125\_Sample\_011046840, Unigene14255\_Sample\_011046840, Unigene34784\_Sample\_011046840, Unigene1368\_Sample\_011046840, Unigene12591\_Sample\_011046840, Unigene14237\_Sample\_011046840, Unigene33577\_Sample\_011046840, Unigene43092\_Sample\_011046840, Unigene2209\_Sample\_011046840, Unigene5726\_Sample\_011046840, Unigene13067\_Sample\_011046840, Unigene21176\_Sample\_011046840, Unigene30462\_Sample\_011046840, Unigene37264\_Sample\_011046840, Unigene32870\_Sample\_011046840, Unigene33524\_Sample\_011046840, Unigene1375\_Sample\_011046840, Unigene1568\_Sample\_011046840, Unigene16035\_Sample\_011046840, Unigene35039\_Sample\_011046840, Unigene35068\_Sample\_011046840, Unigene40303\_Sample\_011046840, Unigene41410\_Sample\_011046840, Unigene42706\_Sample\_011046840, Unigene43393\_Sample\_011046840, Unigene8333\_Sample\_011046840, Unigene11113\_Sample\_011046840, Unigene19593\_Sample\_011046840, Unigene21138\_Sample\_011046840, Unigene37186\_Sample\_011046840, Unigene37898\_Sample\_011046840 |
| 25 | Plant-pathogen interaction Back to summary table | Unigene15939\_Sample\_011046840, Unigene37093\_Sample\_011046840, Unigene4320\_Sample\_011046840, Unigene5795\_Sample\_011046840, Unigene23103\_Sample\_011046840, Unigene2783\_Sample\_011046840, Unigene3954\_Sample\_011046840, Unigene8372\_Sample\_011046840, Unigene32594\_Sample\_011046840, Unigene33360\_Sample\_011046840, Unigene36509\_Sample\_011046840, Unigene4268\_Sample\_011046840, Unigene32357\_Sample\_011046840, Unigene35727\_Sample\_011046840, Unigene39054\_Sample\_011046840, Unigene12619\_Sample\_011046840, Unigene36788\_Sample\_011046840, Unigene334\_Sample\_011046840, Unigene8240\_Sample\_011046840, Unigene30107\_Sample\_011046840, Unigene242\_Sample\_011046840, Unigene31880\_Sample\_011046840, Unigene39865\_Sample\_011046840, Unigene13404\_Sample\_011046840, Unigene17776\_Sample\_011046840, Unigene33870\_Sample\_011046840, Unigene670\_Sample\_011046840, Unigene6582\_Sample\_011046840, Unigene19918\_Sample\_011046840, Unigene38742\_Sample\_011046840, Unigene39577\_Sample\_011046840, Unigene41103\_Sample\_011046840, Unigene2711\_Sample\_011046840, Unigene20949\_Sample\_011046840, Unigene26892\_Sample\_011046840, Unigene9721\_Sample\_011046840, Unigene13262\_Sample\_011046840, Unigene20960\_Sample\_011046840, Unigene23494\_Sample\_011046840, Unigene32156\_Sample\_011046840, Unigene34975\_Sample\_011046840, Unigene43443\_Sample\_011046840 |
| 26 | p53 signaling pathway Back to summary table | Unigene571\_Sample\_011046840, Unigene5556\_Sample\_011046840, Unigene8055\_Sample\_011046840, Unigene31074\_Sample\_011046840, Unigene35485\_Sample\_011046840, Unigene37534\_Sample\_011046840, Unigene40260\_Sample\_011046840, Unigene41510\_Sample\_011046840, Unigene42215\_Sample\_011046840, Unigene177\_Sample\_011046840, Unigene4497\_Sample\_011046840, Unigene7768\_Sample\_011046840, Unigene8203\_Sample\_011046840, Unigene24698\_Sample\_011046840, Unigene27254\_Sample\_011046840, Unigene28459\_Sample\_011046840, Unigene36749\_Sample\_011046840, Unigene39237\_Sample\_011046840, Unigene7322\_Sample\_011046840, Unigene27034\_Sample\_011046840, Unigene40818\_Sample\_011046840, Unigene19991\_Sample\_011046840, Unigene5867\_Sample\_011046840, Unigene25026\_Sample\_011046840, Unigene30502\_Sample\_011046840, Unigene33849\_Sample\_011046840, Unigene37485\_Sample\_011046840, Unigene38504\_Sample\_011046840, Unigene41713\_Sample\_011046840, Unigene43574\_Sample\_011046840, Unigene5439\_Sample\_011046840, Unigene8064\_Sample\_011046840, Unigene34998\_Sample\_011046840, Unigene3486\_Sample\_011046840, Unigene39430\_Sample\_011046840, Unigene30578\_Sample\_011046840, Unigene32586\_Sample\_011046840, Unigene40873\_Sample\_011046840, Unigene10037\_Sample\_011046840, Unigene13414\_Sample\_011046840, Unigene17478\_Sample\_011046840, Unigene22924\_Sample\_011046840, Unigene35972\_Sample\_011046840, Unigene14340\_Sample\_011046840, Unigene31090\_Sample\_011046840, Unigene35936\_Sample\_011046840, Unigene16930\_Sample\_011046840, Unigene24819\_Sample\_011046840, Unigene36506\_Sample\_011046840, Unigene36611\_Sample\_011046840 |
| 27 | Phosphatidylinositol signaling system Back to summary table | Unigene880\_Sample\_011046840, Unigene2822\_Sample\_011046840, Unigene4244\_Sample\_011046840, Unigene5489\_Sample\_011046840, Unigene6899\_Sample\_011046840, Unigene30862\_Sample\_011046840, Unigene33730\_Sample\_011046840, Unigene34179\_Sample\_011046840, Unigene34602\_Sample\_011046840, Unigene37948\_Sample\_011046840, Unigene40745\_Sample\_011046840, Unigene41587\_Sample\_011046840, Unigene43510\_Sample\_011046840, Unigene132\_Sample\_011046840, Unigene810\_Sample\_011046840, Unigene1533\_Sample\_011046840, Unigene2536\_Sample\_011046840, Unigene2924\_Sample\_011046840, Unigene3812\_Sample\_011046840, Unigene4033\_Sample\_011046840, Unigene4101\_Sample\_011046840, Unigene4969\_Sample\_011046840, Unigene5669\_Sample\_011046840, Unigene6147\_Sample\_011046840, Unigene6457\_Sample\_011046840, Unigene6500\_Sample\_011046840, Unigene7631\_Sample\_011046840, Unigene28519\_Sample\_011046840, Unigene31836\_Sample\_011046840, Unigene32141\_Sample\_011046840, Unigene34974\_Sample\_011046840, Unigene36899\_Sample\_011046840, Unigene38259\_Sample\_011046840, Unigene38617\_Sample\_011046840, Unigene38795\_Sample\_011046840, Unigene38876\_Sample\_011046840, Unigene39237\_Sample\_011046840, Unigene40251\_Sample\_011046840, Unigene40284\_Sample\_011046840, Unigene40359\_Sample\_011046840, Unigene40934\_Sample\_011046840, Unigene41194\_Sample\_011046840, Unigene41417\_Sample\_011046840, Unigene42217\_Sample\_011046840, Unigene42256\_Sample\_011046840, Unigene42799\_Sample\_011046840, Unigene42996\_Sample\_011046840, Unigene43390\_Sample\_011046840, Unigene3608\_Sample\_011046840, Unigene13195\_Sample\_011046840, Unigene34299\_Sample\_011046840, Unigene35076\_Sample\_011046840, Unigene42172\_Sample\_011046840, Unigene42241\_Sample\_011046840, Unigene43149\_Sample\_011046840, Unigene3161\_Sample\_011046840, Unigene6322\_Sample\_011046840, Unigene9080\_Sample\_011046840, Unigene28344\_Sample\_011046840, Unigene32981\_Sample\_011046840, Unigene39001\_Sample\_011046840, Unigene39054\_Sample\_011046840, Unigene40435\_Sample\_011046840, Unigene40726\_Sample\_011046840, Unigene123\_Sample\_011046840, Unigene2829\_Sample\_011046840, Unigene4635\_Sample\_011046840, Unigene4807\_Sample\_011046840, Unigene5969\_Sample\_011046840, Unigene6398\_Sample\_011046840, Unigene13900\_Sample\_011046840, Unigene15586\_Sample\_011046840, Unigene17036\_Sample\_011046840, Unigene21632\_Sample\_011046840, Unigene36788\_Sample\_011046840, Unigene40222\_Sample\_011046840, Unigene41243\_Sample\_011046840, Unigene43068\_Sample\_011046840, Unigene334\_Sample\_011046840, Unigene14179\_Sample\_011046840, Unigene21688\_Sample\_011046840, Unigene24869\_Sample\_011046840, Unigene28872\_Sample\_011046840, Unigene40143\_Sample\_011046840, Unigene42153\_Sample\_011046840, Unigene5877\_Sample\_011046840, Unigene6926\_Sample\_011046840, Unigene8004\_Sample\_011046840, Unigene18933\_Sample\_011046840, Unigene26785\_Sample\_011046840, Unigene32648\_Sample\_011046840, Unigene33328\_Sample\_011046840, Unigene39912\_Sample\_011046840, Unigene40648\_Sample\_011046840, Unigene40918\_Sample\_011046840, Unigene41137\_Sample\_011046840, Unigene42008\_Sample\_011046840, Unigene42323\_Sample\_011046840, Unigene42385\_Sample\_011046840, Unigene3689\_Sample\_011046840, Unigene26329\_Sample\_011046840, Unigene28542\_Sample\_011046840, Unigene4849\_Sample\_011046840, Unigene7441\_Sample\_011046840, Unigene13307\_Sample\_011046840, Unigene20896\_Sample\_011046840, Unigene42811\_Sample\_011046840, Unigene670\_Sample\_011046840, Unigene3044\_Sample\_011046840, Unigene8614\_Sample\_011046840, Unigene14465\_Sample\_011046840, Unigene17714\_Sample\_011046840, Unigene24036\_Sample\_011046840, Unigene29065\_Sample\_011046840, Unigene33665\_Sample\_011046840, Unigene39577\_Sample\_011046840, Unigene2711\_Sample\_011046840, Unigene3017\_Sample\_011046840, Unigene4493\_Sample\_011046840, Unigene13511\_Sample\_011046840, Unigene32062\_Sample\_011046840, Unigene33692\_Sample\_011046840, Unigene41488\_Sample\_011046840, Unigene41704\_Sample\_011046840, Unigene7388\_Sample\_011046840, Unigene9889\_Sample\_011046840, Unigene10814\_Sample\_011046840, Unigene11201\_Sample\_011046840, Unigene14938\_Sample\_011046840, Unigene28339\_Sample\_011046840, Unigene30077\_Sample\_011046840, Unigene32265\_Sample\_011046840, Unigene34487\_Sample\_011046840, Unigene36591\_Sample\_011046840, Unigene37794\_Sample\_011046840, Unigene38178\_Sample\_011046840, Unigene38668\_Sample\_011046840, Unigene41998\_Sample\_011046840, Unigene1548\_Sample\_011046840, Unigene43443\_Sample\_011046840 |
| 28 | Drug metabolism - other enzymes Back to summary table | Unigene5648\_Sample\_011046840, Unigene6559\_Sample\_011046840, Unigene43446\_Sample\_011046840, Unigene800\_Sample\_011046840, Unigene3386\_Sample\_011046840, Unigene4870\_Sample\_011046840, Unigene8023\_Sample\_011046840, Unigene26709\_Sample\_011046840, Unigene27907\_Sample\_011046840, Unigene30383\_Sample\_011046840, Unigene34102\_Sample\_011046840, Unigene35097\_Sample\_011046840, Unigene35431\_Sample\_011046840, Unigene514\_Sample\_011046840, Unigene38360\_Sample\_011046840, Unigene5470\_Sample\_011046840, Unigene5515\_Sample\_011046840, Unigene8173\_Sample\_011046840, Unigene15512\_Sample\_011046840, Unigene35434\_Sample\_011046840, Unigene36718\_Sample\_011046840, Unigene37628\_Sample\_011046840, Unigene40766\_Sample\_011046840, Unigene41940\_Sample\_011046840, Unigene29879\_Sample\_011046840, Unigene35332\_Sample\_011046840, Unigene40313\_Sample\_011046840, Unigene27920\_Sample\_011046840, Unigene7039\_Sample\_011046840, Unigene32865\_Sample\_011046840, Unigene22192\_Sample\_011046840, Unigene13306\_Sample\_011046840, Unigene13692\_Sample\_011046840, Unigene34024\_Sample\_011046840, Unigene2074\_Sample\_011046840, Unigene12275\_Sample\_011046840, Unigene13836\_Sample\_011046840, Unigene20765\_Sample\_011046840, Unigene29750\_Sample\_011046840, Unigene33605\_Sample\_011046840, Unigene39668\_Sample\_011046840 |
| 29 | NOD-like receptor signaling pathway Back to summary table | Unigene5306\_Sample\_011046840, Unigene40471\_Sample\_011046840, Unigene42127\_Sample\_011046840, Unigene42215\_Sample\_011046840, Unigene42759\_Sample\_011046840, Unigene2986\_Sample\_011046840, Unigene4320\_Sample\_011046840, Unigene24741\_Sample\_011046840, Unigene29711\_Sample\_011046840, Unigene30539\_Sample\_011046840, Unigene43423\_Sample\_011046840, Unigene3954\_Sample\_011046840, Unigene8372\_Sample\_011046840, Unigene32594\_Sample\_011046840, Unigene40086\_Sample\_011046840, Unigene4268\_Sample\_011046840, Unigene6921\_Sample\_011046840, Unigene27040\_Sample\_011046840, Unigene35727\_Sample\_011046840, Unigene3073\_Sample\_011046840, Unigene12619\_Sample\_011046840, Unigene13661\_Sample\_011046840, Unigene38112\_Sample\_011046840, Unigene8240\_Sample\_011046840, Unigene33873\_Sample\_011046840, Unigene43154\_Sample\_011046840, Unigene41115\_Sample\_011046840, Unigene14237\_Sample\_011046840, Unigene17776\_Sample\_011046840, Unigene35821\_Sample\_011046840, Unigene19918\_Sample\_011046840, Unigene30936\_Sample\_011046840, Unigene41103\_Sample\_011046840, Unigene20949\_Sample\_011046840, Unigene1568\_Sample\_011046840, Unigene9721\_Sample\_011046840, Unigene34975\_Sample\_011046840, Unigene41410\_Sample\_011046840, Unigene19593\_Sample\_011046840, Unigene37186\_Sample\_011046840, Unigene37618\_Sample\_011046840 |
| 30 | Amyotrophic lateral sclerosis (ALS) Back to summary table | Unigene2690\_Sample\_011046840, Unigene38206\_Sample\_011046840, Unigene42127\_Sample\_011046840, Unigene42759\_Sample\_011046840, Unigene43332\_Sample\_011046840, Unigene43365\_Sample\_011046840, Unigene2314\_Sample\_011046840, Unigene4067\_Sample\_011046840, Unigene4390\_Sample\_011046840, Unigene5894\_Sample\_011046840, Unigene7868\_Sample\_011046840, Unigene27254\_Sample\_011046840, Unigene28710\_Sample\_011046840, Unigene34242\_Sample\_011046840, Unigene36749\_Sample\_011046840, Unigene39883\_Sample\_011046840, Unigene6149\_Sample\_011046840, Unigene7322\_Sample\_011046840, Unigene14437\_Sample\_011046840, Unigene31553\_Sample\_011046840, Unigene33835\_Sample\_011046840, Unigene38402\_Sample\_011046840, Unigene7951\_Sample\_011046840, Unigene10979\_Sample\_011046840, Unigene12635\_Sample\_011046840, Unigene28898\_Sample\_011046840, Unigene36860\_Sample\_011046840, Unigene8064\_Sample\_011046840, Unigene28124\_Sample\_011046840, Unigene33873\_Sample\_011046840, Unigene39958\_Sample\_011046840, Unigene6944\_Sample\_011046840, Unigene10749\_Sample\_011046840, Unigene14139\_Sample\_011046840, Unigene17676\_Sample\_011046840, Unigene32479\_Sample\_011046840, Unigene32864\_Sample\_011046840, Unigene14930\_Sample\_011046840, Unigene31562\_Sample\_011046840, Unigene36611\_Sample\_011046840 |
| 31 | Neuroactive ligand-receptor interaction Back to summary table | Unigene838\_Sample\_011046840, Unigene1715\_Sample\_011046840, Unigene1883\_Sample\_011046840, Unigene4123\_Sample\_011046840, Unigene4674\_Sample\_011046840, Unigene7653\_Sample\_011046840, Unigene8071\_Sample\_011046840, Unigene36100\_Sample\_011046840, Unigene37620\_Sample\_011046840, Unigene42555\_Sample\_011046840, Unigene43160\_Sample\_011046840, Unigene43219\_Sample\_011046840, Unigene2195\_Sample\_011046840, Unigene2505\_Sample\_011046840, Unigene3025\_Sample\_011046840, Unigene6753\_Sample\_011046840, Unigene7697\_Sample\_011046840, Unigene7961\_Sample\_011046840, Unigene8112\_Sample\_011046840, Unigene18838\_Sample\_011046840, Unigene27429\_Sample\_011046840, Unigene34735\_Sample\_011046840, Unigene34930\_Sample\_011046840, Unigene37597\_Sample\_011046840, Unigene39259\_Sample\_011046840, Unigene42846\_Sample\_011046840, Unigene1076\_Sample\_011046840, Unigene2640\_Sample\_011046840, Unigene12089\_Sample\_011046840, Unigene29197\_Sample\_011046840, Unigene38407\_Sample\_011046840, Unigene39352\_Sample\_011046840, Unigene41688\_Sample\_011046840, Unigene41082\_Sample\_011046840, Unigene43434\_Sample\_011046840, Unigene2908\_Sample\_011046840, Unigene6292\_Sample\_011046840, Unigene32809\_Sample\_011046840, Unigene39213\_Sample\_011046840, Unigene21962\_Sample\_011046840, Unigene40790\_Sample\_011046840, Unigene4138\_Sample\_011046840, Unigene28229\_Sample\_011046840, Unigene38778\_Sample\_011046840, Unigene93\_Sample\_011046840, Unigene12012\_Sample\_011046840, Unigene30888\_Sample\_011046840, Unigene35435\_Sample\_011046840, Unigene34166\_Sample\_011046840, Unigene35556\_Sample\_011046840, Unigene30092\_Sample\_011046840, Unigene34807\_Sample\_011046840, Unigene39066\_Sample\_011046840, Unigene4735\_Sample\_011046840, Unigene21816\_Sample\_011046840, Unigene21396\_Sample\_011046840, Unigene26954\_Sample\_011046840, Unigene29777\_Sample\_011046840, Unigene353\_Sample\_011046840, Unigene6302\_Sample\_011046840, Unigene13947\_Sample\_011046840, Unigene15244\_Sample\_011046840 |
| 32 | Notch signaling pathway Back to summary table | Unigene3475\_Sample\_011046840, Unigene3726\_Sample\_011046840, Unigene6446\_Sample\_011046840, Unigene22626\_Sample\_011046840, Unigene35368\_Sample\_011046840, Unigene39589\_Sample\_011046840, Unigene41989\_Sample\_011046840, Unigene42071\_Sample\_011046840, Unigene43601\_Sample\_011046840, Unigene1179\_Sample\_011046840, Unigene1280\_Sample\_011046840, Unigene2005\_Sample\_011046840, Unigene4438\_Sample\_011046840, Unigene5085\_Sample\_011046840, Unigene5402\_Sample\_011046840, Unigene6173\_Sample\_011046840, Unigene8063\_Sample\_011046840, Unigene9825\_Sample\_011046840, Unigene15140\_Sample\_011046840, Unigene18537\_Sample\_011046840, Unigene18652\_Sample\_011046840, Unigene24376\_Sample\_011046840, Unigene24651\_Sample\_011046840, Unigene25944\_Sample\_011046840, Unigene28757\_Sample\_011046840, Unigene38954\_Sample\_011046840, Unigene39573\_Sample\_011046840, Unigene39735\_Sample\_011046840, Unigene39808\_Sample\_011046840, Unigene40002\_Sample\_011046840, Unigene40105\_Sample\_011046840, Unigene976\_Sample\_011046840, Unigene4662\_Sample\_011046840, Unigene38046\_Sample\_011046840, Unigene42562\_Sample\_011046840, Unigene1581\_Sample\_011046840, Unigene16050\_Sample\_011046840, Unigene37084\_Sample\_011046840, Unigene38523\_Sample\_011046840, Unigene38592\_Sample\_011046840, Unigene40411\_Sample\_011046840, Unigene40677\_Sample\_011046840, Unigene770\_Sample\_011046840, Unigene1475\_Sample\_011046840, Unigene14193\_Sample\_011046840, Unigene18000\_Sample\_011046840, Unigene21792\_Sample\_011046840, Unigene31393\_Sample\_011046840, Unigene33313\_Sample\_011046840, Unigene33403\_Sample\_011046840, Unigene34667\_Sample\_011046840, Unigene37994\_Sample\_011046840, Unigene39646\_Sample\_011046840, Unigene41412\_Sample\_011046840, Unigene41781\_Sample\_011046840, Unigene38301\_Sample\_011046840, Unigene11483\_Sample\_011046840, Unigene41341\_Sample\_011046840, Unigene7772\_Sample\_011046840, Unigene15970\_Sample\_011046840, Unigene6148\_Sample\_011046840, Unigene15053\_Sample\_011046840, Unigene30184\_Sample\_011046840, Unigene30246\_Sample\_011046840, Unigene30445\_Sample\_011046840, Unigene34284\_Sample\_011046840, Unigene38741\_Sample\_011046840, Unigene38996\_Sample\_011046840, Unigene40855\_Sample\_011046840, Unigene6181\_Sample\_011046840, Unigene14072\_Sample\_011046840, Unigene36704\_Sample\_011046840, Unigene40943\_Sample\_011046840, Unigene19841\_Sample\_011046840, Unigene20234\_Sample\_011046840, Unigene30685\_Sample\_011046840, Unigene38690\_Sample\_011046840, Unigene16736\_Sample\_011046840, Unigene38841\_Sample\_011046840, Unigene41201\_Sample\_011046840, Unigene43308\_Sample\_011046840, Unigene32619\_Sample\_011046840, Unigene35805\_Sample\_011046840, Unigene41562\_Sample\_011046840 |
| 33 | Hematopoietic cell lineage Back to summary table | Unigene7980\_Sample\_011046840, Unigene8107\_Sample\_011046840, Unigene42018\_Sample\_011046840, Unigene43193\_Sample\_011046840, Unigene43429\_Sample\_011046840, Unigene509\_Sample\_011046840, Unigene6996\_Sample\_011046840, Unigene7091\_Sample\_011046840, Unigene27053\_Sample\_011046840, Unigene28956\_Sample\_011046840, Unigene33011\_Sample\_011046840, Unigene39242\_Sample\_011046840, Unigene39938\_Sample\_011046840, Unigene40816\_Sample\_011046840, Unigene8133\_Sample\_011046840, Unigene42910\_Sample\_011046840, Unigene3335\_Sample\_011046840, Unigene32463\_Sample\_011046840, Unigene33876\_Sample\_011046840, Unigene39625\_Sample\_011046840, Unigene43101\_Sample\_011046840, Unigene7250\_Sample\_011046840, Unigene21594\_Sample\_011046840, Unigene30442\_Sample\_011046840, Unigene38196\_Sample\_011046840, Unigene39613\_Sample\_011046840, Unigene643\_Sample\_011046840, Unigene12234\_Sample\_011046840, Unigene24335\_Sample\_011046840, Unigene36081\_Sample\_011046840, Unigene37778\_Sample\_011046840, Unigene18122\_Sample\_011046840, Unigene32870\_Sample\_011046840, Unigene25726\_Sample\_011046840, Unigene28432\_Sample\_011046840, Unigene37909\_Sample\_011046840, Unigene26282\_Sample\_011046840, Unigene39535\_Sample\_011046840 |
| 34 | Glioma Back to summary table | Unigene880\_Sample\_011046840, Unigene4790\_Sample\_011046840, Unigene15939\_Sample\_011046840, Unigene24311\_Sample\_011046840, Unigene29592\_Sample\_011046840, Unigene30862\_Sample\_011046840, Unigene40260\_Sample\_011046840, Unigene2986\_Sample\_011046840, Unigene4033\_Sample\_011046840, Unigene4101\_Sample\_011046840, Unigene5425\_Sample\_011046840, Unigene8009\_Sample\_011046840, Unigene11592\_Sample\_011046840, Unigene24698\_Sample\_011046840, Unigene30720\_Sample\_011046840, Unigene33208\_Sample\_011046840, Unigene34974\_Sample\_011046840, Unigene36690\_Sample\_011046840, Unigene38617\_Sample\_011046840, Unigene38866\_Sample\_011046840, Unigene39237\_Sample\_011046840, Unigene40208\_Sample\_011046840, Unigene41584\_Sample\_011046840, Unigene42164\_Sample\_011046840, Unigene43635\_Sample\_011046840, Unigene3608\_Sample\_011046840, Unigene5166\_Sample\_011046840, Unigene33983\_Sample\_011046840, Unigene38834\_Sample\_011046840, Unigene38847\_Sample\_011046840, Unigene42204\_Sample\_011046840, Unigene43149\_Sample\_011046840, Unigene3161\_Sample\_011046840, Unigene6322\_Sample\_011046840, Unigene39054\_Sample\_011046840, Unigene39778\_Sample\_011046840, Unigene4807\_Sample\_011046840, Unigene8088\_Sample\_011046840, Unigene20325\_Sample\_011046840, Unigene23045\_Sample\_011046840, Unigene28708\_Sample\_011046840, Unigene36320\_Sample\_011046840, Unigene36788\_Sample\_011046840, Unigene43068\_Sample\_011046840, Unigene334\_Sample\_011046840, Unigene24869\_Sample\_011046840, Unigene38245\_Sample\_011046840, Unigene4139\_Sample\_011046840, Unigene12153\_Sample\_011046840, Unigene16429\_Sample\_011046840, Unigene18933\_Sample\_011046840, Unigene26785\_Sample\_011046840, Unigene27378\_Sample\_011046840, Unigene28751\_Sample\_011046840, Unigene31880\_Sample\_011046840, Unigene35787\_Sample\_011046840, Unigene39503\_Sample\_011046840, Unigene40189\_Sample\_011046840, Unigene40648\_Sample\_011046840, Unigene42008\_Sample\_011046840, Unigene42385\_Sample\_011046840, Unigene43154\_Sample\_011046840, Unigene1368\_Sample\_011046840, Unigene4849\_Sample\_011046840, Unigene20896\_Sample\_011046840, Unigene33870\_Sample\_011046840, Unigene37392\_Sample\_011046840, Unigene42811\_Sample\_011046840, Unigene670\_Sample\_011046840, Unigene5726\_Sample\_011046840, Unigene21176\_Sample\_011046840, Unigene27623\_Sample\_011046840, Unigene39577\_Sample\_011046840, Unigene41560\_Sample\_011046840, Unigene2711\_Sample\_011046840, Unigene33692\_Sample\_011046840, Unigene35972\_Sample\_011046840, Unigene41704\_Sample\_011046840, Unigene11201\_Sample\_011046840, Unigene16035\_Sample\_011046840, Unigene20420\_Sample\_011046840, Unigene32265\_Sample\_011046840, Unigene43443\_Sample\_011046840 |
| 35 | Epithelial cell signaling in Helicobacter pylori infection Back to summary table | Unigene1843\_Sample\_011046840, Unigene4993\_Sample\_011046840, Unigene6378\_Sample\_011046840, Unigene8198\_Sample\_011046840, Unigene36475\_Sample\_011046840, Unigene39096\_Sample\_011046840, Unigene39923\_Sample\_011046840, Unigene40184\_Sample\_011046840, Unigene40484\_Sample\_011046840, Unigene42127\_Sample\_011046840, Unigene42759\_Sample\_011046840, Unigene43094\_Sample\_011046840, Unigene43556\_Sample\_011046840, Unigene1602\_Sample\_011046840, Unigene4033\_Sample\_011046840, Unigene4438\_Sample\_011046840, Unigene10748\_Sample\_011046840, Unigene11592\_Sample\_011046840, Unigene29711\_Sample\_011046840, Unigene29794\_Sample\_011046840, Unigene30539\_Sample\_011046840, Unigene33208\_Sample\_011046840, Unigene33883\_Sample\_011046840, Unigene34085\_Sample\_011046840, Unigene36690\_Sample\_011046840, Unigene36762\_Sample\_011046840, Unigene39843\_Sample\_011046840, Unigene39883\_Sample\_011046840, Unigene41235\_Sample\_011046840, Unigene41370\_Sample\_011046840, Unigene41642\_Sample\_011046840, Unigene41915\_Sample\_011046840, Unigene1399\_Sample\_011046840, Unigene29880\_Sample\_011046840, Unigene30019\_Sample\_011046840, Unigene33835\_Sample\_011046840, Unigene33983\_Sample\_011046840, Unigene38834\_Sample\_011046840, Unigene41695\_Sample\_011046840, Unigene42204\_Sample\_011046840, Unigene4449\_Sample\_011046840, Unigene6921\_Sample\_011046840, Unigene20538\_Sample\_011046840, Unigene31844\_Sample\_011046840, Unigene35976\_Sample\_011046840, Unigene38028\_Sample\_011046840, Unigene7408\_Sample\_011046840, Unigene7678\_Sample\_011046840, Unigene15594\_Sample\_011046840, Unigene25781\_Sample\_011046840, Unigene25956\_Sample\_011046840, Unigene37629\_Sample\_011046840, Unigene38677\_Sample\_011046840, Unigene40513\_Sample\_011046840, Unigene42487\_Sample\_011046840, Unigene12084\_Sample\_011046840, Unigene21908\_Sample\_011046840, Unigene33873\_Sample\_011046840, Unigene36382\_Sample\_011046840, Unigene36687\_Sample\_011046840, Unigene39303\_Sample\_011046840, Unigene12153\_Sample\_011046840, Unigene14255\_Sample\_011046840, Unigene17166\_Sample\_011046840, Unigene29670\_Sample\_011046840, Unigene33547\_Sample\_011046840, Unigene35532\_Sample\_011046840, Unigene35787\_Sample\_011046840, Unigene42027\_Sample\_011046840, Unigene13322\_Sample\_011046840, Unigene14237\_Sample\_011046840, Unigene23323\_Sample\_011046840, Unigene24674\_Sample\_011046840, Unigene29660\_Sample\_011046840, Unigene38900\_Sample\_011046840, Unigene2209\_Sample\_011046840, Unigene13067\_Sample\_011046840, Unigene19127\_Sample\_011046840, Unigene19865\_Sample\_011046840, Unigene25046\_Sample\_011046840, Unigene31253\_Sample\_011046840, Unigene39062\_Sample\_011046840, Unigene14612\_Sample\_011046840, Unigene1568\_Sample\_011046840, Unigene14930\_Sample\_011046840, Unigene17721\_Sample\_011046840, Unigene20157\_Sample\_011046840, Unigene23699\_Sample\_011046840, Unigene28467\_Sample\_011046840, Unigene29621\_Sample\_011046840, Unigene41410\_Sample\_011046840, Unigene15362\_Sample\_011046840, Unigene19593\_Sample\_011046840, Unigene20109\_Sample\_011046840, Unigene32555\_Sample\_011046840, Unigene36661\_Sample\_011046840, Unigene37186\_Sample\_011046840 |
| 36 | Base excision repair Back to summary table | Unigene1920\_Sample\_011046840, Unigene18925\_Sample\_011046840, Unigene43375\_Sample\_011046840, Unigene1215\_Sample\_011046840, Unigene23759\_Sample\_011046840, Unigene24457\_Sample\_011046840, Unigene28929\_Sample\_011046840, Unigene30695\_Sample\_011046840, Unigene34596\_Sample\_011046840, Unigene35051\_Sample\_011046840, Unigene36032\_Sample\_011046840, Unigene38474\_Sample\_011046840, Unigene5316\_Sample\_011046840, Unigene16797\_Sample\_011046840, Unigene21598\_Sample\_011046840, Unigene27432\_Sample\_011046840, Unigene27931\_Sample\_011046840, Unigene42105\_Sample\_011046840, Unigene42569\_Sample\_011046840, Unigene3915\_Sample\_011046840, Unigene10754\_Sample\_011046840, Unigene13502\_Sample\_011046840, Unigene14018\_Sample\_011046840, Unigene35271\_Sample\_011046840, Unigene36117\_Sample\_011046840, Unigene37440\_Sample\_011046840, Unigene41259\_Sample\_011046840, Unigene42625\_Sample\_011046840, Unigene21911\_Sample\_011046840, Unigene29016\_Sample\_011046840, Unigene36772\_Sample\_011046840, Unigene37154\_Sample\_011046840, Unigene41479\_Sample\_011046840, Unigene42038\_Sample\_011046840, Unigene42776\_Sample\_011046840, Unigene15290\_Sample\_011046840, Unigene19866\_Sample\_011046840, Unigene20296\_Sample\_011046840, Unigene4108\_Sample\_011046840, Unigene26800\_Sample\_011046840, Unigene28109\_Sample\_011046840, Unigene31539\_Sample\_011046840, Unigene31638\_Sample\_011046840, Unigene36289\_Sample\_011046840, Unigene41291\_Sample\_011046840, Unigene39581\_Sample\_011046840, Unigene7669\_Sample\_011046840, Unigene25039\_Sample\_011046840, Unigene41334\_Sample\_011046840, Unigene16944\_Sample\_011046840, Unigene26640\_Sample\_011046840, Unigene27745\_Sample\_011046840, Unigene30448\_Sample\_011046840, Unigene32822\_Sample\_011046840, Unigene34691\_Sample\_011046840, Unigene10159\_Sample\_011046840, Unigene16663\_Sample\_011046840, Unigene25605\_Sample\_011046840, Unigene33837\_Sample\_011046840, Unigene37486\_Sample\_011046840 |
| 37 | Complement and coagulation cascades Back to summary table | Unigene36596\_Sample\_011046840, Unigene40029\_Sample\_011046840, Unigene2371\_Sample\_011046840, Unigene3663\_Sample\_011046840, Unigene5975\_Sample\_011046840, Unigene7045\_Sample\_011046840, Unigene7773\_Sample\_011046840, Unigene8104\_Sample\_011046840, Unigene18043\_Sample\_011046840, Unigene26769\_Sample\_011046840, Unigene36214\_Sample\_011046840, Unigene39242\_Sample\_011046840, Unigene42309\_Sample\_011046840, Unigene42678\_Sample\_011046840, Unigene43436\_Sample\_011046840, Unigene547\_Sample\_011046840, Unigene4942\_Sample\_011046840, Unigene6909\_Sample\_011046840, Unigene7165\_Sample\_011046840, Unigene29428\_Sample\_011046840, Unigene39131\_Sample\_011046840, Unigene43050\_Sample\_011046840, Unigene43563\_Sample\_011046840, Unigene18633\_Sample\_011046840, Unigene30665\_Sample\_011046840, Unigene41247\_Sample\_011046840, Unigene43226\_Sample\_011046840, Unigene1701\_Sample\_011046840, Unigene2146\_Sample\_011046840, Unigene7772\_Sample\_011046840, Unigene29454\_Sample\_011046840, Unigene20806\_Sample\_011046840, Unigene23687\_Sample\_011046840, Unigene30900\_Sample\_011046840, Unigene5569\_Sample\_011046840, Unigene7661\_Sample\_011046840, Unigene25119\_Sample\_011046840 |
| 38 | ErbB signaling pathway Back to summary table | Unigene880\_Sample\_011046840, Unigene2194\_Sample\_011046840, Unigene4790\_Sample\_011046840, Unigene4993\_Sample\_011046840, Unigene6551\_Sample\_011046840, Unigene8198\_Sample\_011046840, Unigene10035\_Sample\_011046840, Unigene15939\_Sample\_011046840, Unigene24311\_Sample\_011046840, Unigene29592\_Sample\_011046840, Unigene30862\_Sample\_011046840, Unigene36475\_Sample\_011046840, Unigene37793\_Sample\_011046840, Unigene38706\_Sample\_011046840, Unigene40413\_Sample\_011046840, Unigene41242\_Sample\_011046840, Unigene42766\_Sample\_011046840, Unigene42900\_Sample\_011046840, Unigene43311\_Sample\_011046840, Unigene2986\_Sample\_011046840, Unigene4033\_Sample\_011046840, Unigene4101\_Sample\_011046840, Unigene4779\_Sample\_011046840, Unigene5389\_Sample\_011046840, Unigene5425\_Sample\_011046840, Unigene7349\_Sample\_011046840, Unigene8009\_Sample\_011046840, Unigene11592\_Sample\_011046840, Unigene26575\_Sample\_011046840, Unigene29711\_Sample\_011046840, Unigene30539\_Sample\_011046840, Unigene30720\_Sample\_011046840, Unigene32380\_Sample\_011046840, Unigene33208\_Sample\_011046840, Unigene34974\_Sample\_011046840, Unigene36690\_Sample\_011046840, Unigene38617\_Sample\_011046840, Unigene38866\_Sample\_011046840, Unigene39843\_Sample\_011046840, Unigene40208\_Sample\_011046840, Unigene41370\_Sample\_011046840, Unigene41501\_Sample\_011046840, Unigene41584\_Sample\_011046840, Unigene41915\_Sample\_011046840, Unigene42164\_Sample\_011046840, Unigene42428\_Sample\_011046840, Unigene43301\_Sample\_011046840, Unigene43342\_Sample\_011046840, Unigene3608\_Sample\_011046840, Unigene5166\_Sample\_011046840, Unigene29880\_Sample\_011046840, Unigene33983\_Sample\_011046840, Unigene34039\_Sample\_011046840, Unigene38834\_Sample\_011046840, Unigene38847\_Sample\_011046840, Unigene42204\_Sample\_011046840, Unigene43149\_Sample\_011046840, Unigene3161\_Sample\_011046840, Unigene6322\_Sample\_011046840, Unigene6921\_Sample\_011046840, Unigene29542\_Sample\_011046840, Unigene31699\_Sample\_011046840, Unigene38648\_Sample\_011046840, Unigene4807\_Sample\_011046840, Unigene8088\_Sample\_011046840, Unigene14485\_Sample\_011046840, Unigene20325\_Sample\_011046840, Unigene23045\_Sample\_011046840, Unigene28708\_Sample\_011046840, Unigene31079\_Sample\_011046840, Unigene36320\_Sample\_011046840, Unigene40659\_Sample\_011046840, Unigene42079\_Sample\_011046840, Unigene42487\_Sample\_011046840, Unigene43068\_Sample\_011046840, Unigene14379\_Sample\_011046840, Unigene24869\_Sample\_011046840, Unigene26621\_Sample\_011046840, Unigene35901\_Sample\_011046840, Unigene4139\_Sample\_011046840, Unigene12153\_Sample\_011046840, Unigene16429\_Sample\_011046840, Unigene18933\_Sample\_011046840, Unigene25548\_Sample\_011046840, Unigene26785\_Sample\_011046840, Unigene27378\_Sample\_011046840, Unigene28751\_Sample\_011046840, Unigene29670\_Sample\_011046840, Unigene31705\_Sample\_011046840, Unigene31880\_Sample\_011046840, Unigene35532\_Sample\_011046840, Unigene35787\_Sample\_011046840, Unigene36897\_Sample\_011046840, Unigene38000\_Sample\_011046840, Unigene39503\_Sample\_011046840, Unigene40189\_Sample\_011046840, Unigene40648\_Sample\_011046840, Unigene42008\_Sample\_011046840, Unigene42385\_Sample\_011046840, Unigene43154\_Sample\_011046840, Unigene1368\_Sample\_011046840, Unigene4849\_Sample\_011046840, Unigene10749\_Sample\_011046840, Unigene14237\_Sample\_011046840, Unigene18009\_Sample\_011046840, Unigene19400\_Sample\_011046840, Unigene20896\_Sample\_011046840, Unigene22254\_Sample\_011046840, Unigene29526\_Sample\_011046840, Unigene29660\_Sample\_011046840, Unigene33870\_Sample\_011046840, Unigene35953\_Sample\_011046840, Unigene37392\_Sample\_011046840, Unigene42811\_Sample\_011046840, Unigene5726\_Sample\_011046840, Unigene21176\_Sample\_011046840, Unigene27623\_Sample\_011046840, Unigene31320\_Sample\_011046840, Unigene41560\_Sample\_011046840, Unigene10037\_Sample\_011046840, Unigene24184\_Sample\_011046840, Unigene33692\_Sample\_011046840, Unigene38066\_Sample\_011046840, Unigene41704\_Sample\_011046840, Unigene1256\_Sample\_011046840, Unigene1568\_Sample\_011046840, Unigene4276\_Sample\_011046840, Unigene11201\_Sample\_011046840, Unigene11765\_Sample\_011046840, Unigene16035\_Sample\_011046840, Unigene20420\_Sample\_011046840, Unigene28337\_Sample\_011046840, Unigene29621\_Sample\_011046840, Unigene32265\_Sample\_011046840, Unigene37491\_Sample\_011046840, Unigene19593\_Sample\_011046840, Unigene24889\_Sample\_011046840, Unigene37186\_Sample\_011046840 |
| 39 | Adherens junction Back to summary table | Unigene1715\_Sample\_011046840, Unigene2132\_Sample\_011046840, Unigene2194\_Sample\_011046840, Unigene4600\_Sample\_011046840, Unigene6378\_Sample\_011046840, Unigene6922\_Sample\_011046840, Unigene8035\_Sample\_011046840, Unigene10232\_Sample\_011046840, Unigene36475\_Sample\_011046840, Unigene40280\_Sample\_011046840, Unigene40446\_Sample\_011046840, Unigene40454\_Sample\_011046840, Unigene40471\_Sample\_011046840, Unigene41702\_Sample\_011046840, Unigene43211\_Sample\_011046840, Unigene43438\_Sample\_011046840, Unigene43496\_Sample\_011046840, Unigene43556\_Sample\_011046840, Unigene2986\_Sample\_011046840, Unigene3258\_Sample\_011046840, Unigene3776\_Sample\_011046840, Unigene6240\_Sample\_011046840, Unigene6863\_Sample\_011046840, Unigene7496\_Sample\_011046840, Unigene7529\_Sample\_011046840, Unigene7979\_Sample\_011046840, Unigene10748\_Sample\_011046840, Unigene11592\_Sample\_011046840, Unigene15111\_Sample\_011046840, Unigene16996\_Sample\_011046840, Unigene27291\_Sample\_011046840, Unigene28418\_Sample\_011046840, Unigene29916\_Sample\_011046840, Unigene30791\_Sample\_011046840, Unigene33208\_Sample\_011046840, Unigene33370\_Sample\_011046840, Unigene34085\_Sample\_011046840, Unigene35122\_Sample\_011046840, Unigene35657\_Sample\_011046840, Unigene36690\_Sample\_011046840, Unigene38045\_Sample\_011046840, Unigene38432\_Sample\_011046840, Unigene38612\_Sample\_011046840, Unigene39883\_Sample\_011046840, Unigene41853\_Sample\_011046840, Unigene43210\_Sample\_011046840, Unigene43258\_Sample\_011046840, Unigene43312\_Sample\_011046840, Unigene43492\_Sample\_011046840, Unigene43635\_Sample\_011046840, Unigene976\_Sample\_011046840, Unigene2156\_Sample\_011046840, Unigene8904\_Sample\_011046840, Unigene25979\_Sample\_011046840, Unigene33673\_Sample\_011046840, Unigene33835\_Sample\_011046840, Unigene33954\_Sample\_011046840, Unigene33983\_Sample\_011046840, Unigene34302\_Sample\_011046840, Unigene35961\_Sample\_011046840, Unigene38834\_Sample\_011046840, Unigene40469\_Sample\_011046840, Unigene42204\_Sample\_011046840, Unigene42227\_Sample\_011046840, Unigene7958\_Sample\_011046840, Unigene27657\_Sample\_011046840, Unigene28604\_Sample\_011046840, Unigene33984\_Sample\_011046840, Unigene39778\_Sample\_011046840, Unigene41082\_Sample\_011046840, Unigene4818\_Sample\_011046840, Unigene5364\_Sample\_011046840, Unigene6516\_Sample\_011046840, Unigene7408\_Sample\_011046840, Unigene13661\_Sample\_011046840, Unigene25431\_Sample\_011046840, Unigene35981\_Sample\_011046840, Unigene38655\_Sample\_011046840, Unigene38692\_Sample\_011046840, Unigene39100\_Sample\_011046840, Unigene39243\_Sample\_011046840, Unigene39375\_Sample\_011046840, Unigene40784\_Sample\_011046840, Unigene42231\_Sample\_011046840, Unigene2581\_Sample\_011046840, Unigene34563\_Sample\_011046840, Unigene38616\_Sample\_011046840, Unigene39303\_Sample\_011046840, Unigene41024\_Sample\_011046840, Unigene41881\_Sample\_011046840, Unigene42770\_Sample\_011046840, Unigene9938\_Sample\_011046840, Unigene12153\_Sample\_011046840, Unigene31742\_Sample\_011046840, Unigene35787\_Sample\_011046840, Unigene36662\_Sample\_011046840, Unigene43154\_Sample\_011046840, Unigene43590\_Sample\_011046840, Unigene6846\_Sample\_011046840, Unigene25523\_Sample\_011046840, Unigene30941\_Sample\_011046840, Unigene33045\_Sample\_011046840, Unigene35550\_Sample\_011046840, Unigene2815\_Sample\_011046840, Unigene3219\_Sample\_011046840, Unigene19127\_Sample\_011046840, Unigene39850\_Sample\_011046840, Unigene12868\_Sample\_011046840, Unigene15237\_Sample\_011046840, Unigene30201\_Sample\_011046840, Unigene31135\_Sample\_011046840, Unigene14930\_Sample\_011046840, Unigene24464\_Sample\_011046840, Unigene26719\_Sample\_011046840, Unigene3151\_Sample\_011046840, Unigene5921\_Sample\_011046840, Unigene39933\_Sample\_011046840 |
| 40 | Alanine, aspartate and glutamate metabolism Back to summary table | Unigene4663\_Sample\_011046840, Unigene7194\_Sample\_011046840, Unigene9554\_Sample\_011046840, Unigene25747\_Sample\_011046840, Unigene26710\_Sample\_011046840, Unigene32365\_Sample\_011046840, Unigene2227\_Sample\_011046840, Unigene8475\_Sample\_011046840, Unigene17139\_Sample\_011046840, Unigene23357\_Sample\_011046840, Unigene31660\_Sample\_011046840, Unigene36700\_Sample\_011046840, Unigene36774\_Sample\_011046840, Unigene37225\_Sample\_011046840, Unigene38380\_Sample\_011046840, Unigene38857\_Sample\_011046840, Unigene40609\_Sample\_011046840, Unigene40612\_Sample\_011046840, Unigene41843\_Sample\_011046840, Unigene43477\_Sample\_011046840, Unigene43479\_Sample\_011046840, Unigene43555\_Sample\_011046840, Unigene350\_Sample\_011046840, Unigene5021\_Sample\_011046840, Unigene32803\_Sample\_011046840, Unigene1672\_Sample\_011046840, Unigene473\_Sample\_011046840, Unigene7751\_Sample\_011046840, Unigene17327\_Sample\_011046840, Unigene20023\_Sample\_011046840, Unigene20699\_Sample\_011046840, Unigene27802\_Sample\_011046840, Unigene31776\_Sample\_011046840, Unigene35315\_Sample\_011046840, Unigene36533\_Sample\_011046840, Unigene40632\_Sample\_011046840, Unigene40938\_Sample\_011046840, Unigene43449\_Sample\_011046840, Unigene1504\_Sample\_011046840, Unigene7058\_Sample\_011046840, Unigene8122\_Sample\_011046840, Unigene20783\_Sample\_011046840, Unigene25628\_Sample\_011046840, Unigene29567\_Sample\_011046840, Unigene32735\_Sample\_011046840, Unigene20183\_Sample\_011046840, Unigene26590\_Sample\_011046840, Unigene29298\_Sample\_011046840, Unigene25488\_Sample\_011046840, Unigene39980\_Sample\_011046840, Unigene18659\_Sample\_011046840, Unigene30324\_Sample\_011046840 |
| 41 | Dorso-ventral axis formation Back to summary table | Unigene3475\_Sample\_011046840, Unigene3726\_Sample\_011046840, Unigene4790\_Sample\_011046840, Unigene6665\_Sample\_011046840, Unigene15939\_Sample\_011046840, Unigene43601\_Sample\_011046840, Unigene2005\_Sample\_011046840, Unigene2986\_Sample\_011046840, Unigene5085\_Sample\_011046840, Unigene5402\_Sample\_011046840, Unigene6065\_Sample\_011046840, Unigene6173\_Sample\_011046840, Unigene8063\_Sample\_011046840, Unigene11592\_Sample\_011046840, Unigene25944\_Sample\_011046840, Unigene27677\_Sample\_011046840, Unigene29864\_Sample\_011046840, Unigene30393\_Sample\_011046840, Unigene33208\_Sample\_011046840, Unigene35491\_Sample\_011046840, Unigene36690\_Sample\_011046840, Unigene37340\_Sample\_011046840, Unigene38866\_Sample\_011046840, Unigene38954\_Sample\_011046840, Unigene39573\_Sample\_011046840, Unigene39735\_Sample\_011046840, Unigene39808\_Sample\_011046840, Unigene40002\_Sample\_011046840, Unigene40208\_Sample\_011046840, Unigene41584\_Sample\_011046840, Unigene767\_Sample\_011046840, Unigene2541\_Sample\_011046840, Unigene4662\_Sample\_011046840, Unigene33983\_Sample\_011046840, Unigene38046\_Sample\_011046840, Unigene38834\_Sample\_011046840, Unigene42204\_Sample\_011046840, Unigene42562\_Sample\_011046840, Unigene1581\_Sample\_011046840, Unigene25043\_Sample\_011046840, Unigene770\_Sample\_011046840, Unigene14373\_Sample\_011046840, Unigene18000\_Sample\_011046840, Unigene20325\_Sample\_011046840, Unigene21792\_Sample\_011046840, Unigene23045\_Sample\_011046840, Unigene31393\_Sample\_011046840, Unigene34667\_Sample\_011046840, Unigene36320\_Sample\_011046840, Unigene37223\_Sample\_011046840, Unigene22643\_Sample\_011046840, Unigene40655\_Sample\_011046840, Unigene11483\_Sample\_011046840, Unigene12153\_Sample\_011046840, Unigene27378\_Sample\_011046840, Unigene28751\_Sample\_011046840, Unigene31880\_Sample\_011046840, Unigene35787\_Sample\_011046840, Unigene36251\_Sample\_011046840, Unigene39503\_Sample\_011046840, Unigene40189\_Sample\_011046840, Unigene7772\_Sample\_011046840, Unigene6148\_Sample\_011046840, Unigene33870\_Sample\_011046840, Unigene34284\_Sample\_011046840, Unigene38455\_Sample\_011046840, Unigene38741\_Sample\_011046840, Unigene42094\_Sample\_011046840, Unigene25406\_Sample\_011046840, Unigene27623\_Sample\_011046840, Unigene40943\_Sample\_011046840, Unigene41560\_Sample\_011046840, Unigene42473\_Sample\_011046840, Unigene35805\_Sample\_011046840 |
| 42 | Insulin signaling pathway Back to summary table | Unigene409\_Sample\_011046840, Unigene439\_Sample\_011046840, Unigene571\_Sample\_011046840, Unigene1129\_Sample\_011046840, Unigene1557\_Sample\_011046840, Unigene2194\_Sample\_011046840, Unigene2865\_Sample\_011046840, Unigene4464\_Sample\_011046840, Unigene4790\_Sample\_011046840, Unigene5557\_Sample\_011046840, Unigene6100\_Sample\_011046840, Unigene6551\_Sample\_011046840, Unigene6644\_Sample\_011046840, Unigene7744\_Sample\_011046840, Unigene8062\_Sample\_011046840, Unigene8140\_Sample\_011046840, Unigene15939\_Sample\_011046840, Unigene24311\_Sample\_011046840, Unigene29452\_Sample\_011046840, Unigene29592\_Sample\_011046840, Unigene33378\_Sample\_011046840, Unigene33573\_Sample\_011046840, Unigene34317\_Sample\_011046840, Unigene37951\_Sample\_011046840, Unigene37968\_Sample\_011046840, Unigene39527\_Sample\_011046840, Unigene40234\_Sample\_011046840, Unigene40454\_Sample\_011046840, Unigene41132\_Sample\_011046840, Unigene41242\_Sample\_011046840, Unigene41418\_Sample\_011046840, Unigene42068\_Sample\_011046840, Unigene42278\_Sample\_011046840, Unigene42552\_Sample\_011046840, Unigene43363\_Sample\_011046840, Unigene543\_Sample\_011046840, Unigene2230\_Sample\_011046840, Unigene2986\_Sample\_011046840, Unigene3474\_Sample\_011046840, Unigene3724\_Sample\_011046840, Unigene4403\_Sample\_011046840, Unigene4477\_Sample\_011046840, Unigene4536\_Sample\_011046840, Unigene4779\_Sample\_011046840, Unigene5425\_Sample\_011046840, Unigene5774\_Sample\_011046840, Unigene6139\_Sample\_011046840, Unigene6428\_Sample\_011046840, Unigene10033\_Sample\_011046840, Unigene19398\_Sample\_011046840, Unigene24934\_Sample\_011046840, Unigene26575\_Sample\_011046840, Unigene28752\_Sample\_011046840, Unigene29711\_Sample\_011046840, Unigene30539\_Sample\_011046840, Unigene30720\_Sample\_011046840, Unigene30791\_Sample\_011046840, Unigene31398\_Sample\_011046840, Unigene32368\_Sample\_011046840, Unigene32380\_Sample\_011046840, Unigene33926\_Sample\_011046840, Unigene34953\_Sample\_011046840, Unigene34974\_Sample\_011046840, Unigene35122\_Sample\_011046840, Unigene36252\_Sample\_011046840, Unigene36797\_Sample\_011046840, Unigene37006\_Sample\_011046840, Unigene37776\_Sample\_011046840, Unigene38281\_Sample\_011046840, Unigene38538\_Sample\_011046840, Unigene38573\_Sample\_011046840, Unigene38617\_Sample\_011046840, Unigene38772\_Sample\_011046840, Unigene38866\_Sample\_011046840, Unigene38923\_Sample\_011046840, Unigene40125\_Sample\_011046840, Unigene40128\_Sample\_011046840, Unigene40208\_Sample\_011046840, Unigene40899\_Sample\_011046840, Unigene40958\_Sample\_011046840, Unigene41550\_Sample\_011046840, Unigene41584\_Sample\_011046840, Unigene41853\_Sample\_011046840, Unigene42065\_Sample\_011046840, Unigene42164\_Sample\_011046840, Unigene42197\_Sample\_011046840, Unigene42284\_Sample\_011046840, Unigene42777\_Sample\_011046840, Unigene42867\_Sample\_011046840, Unigene42991\_Sample\_011046840, Unigene43301\_Sample\_011046840, Unigene43635\_Sample\_011046840, Unigene19624\_Sample\_011046840, Unigene26123\_Sample\_011046840, Unigene27667\_Sample\_011046840, Unigene38847\_Sample\_011046840, Unigene40950\_Sample\_011046840, Unigene43149\_Sample\_011046840, Unigene6921\_Sample\_011046840, Unigene28116\_Sample\_011046840, Unigene28404\_Sample\_011046840, Unigene29542\_Sample\_011046840, Unigene31699\_Sample\_011046840, Unigene38648\_Sample\_011046840, Unigene39054\_Sample\_011046840, Unigene43387\_Sample\_011046840, Unigene634\_Sample\_011046840, Unigene4610\_Sample\_011046840, Unigene6516\_Sample\_011046840, Unigene8088\_Sample\_011046840, Unigene10400\_Sample\_011046840, Unigene20325\_Sample\_011046840, Unigene23045\_Sample\_011046840, Unigene26542\_Sample\_011046840, Unigene28708\_Sample\_011046840, Unigene30502\_Sample\_011046840, Unigene31151\_Sample\_011046840, Unigene31285\_Sample\_011046840, Unigene31655\_Sample\_011046840, Unigene31945\_Sample\_011046840, Unigene32209\_Sample\_011046840, Unigene34117\_Sample\_011046840, Unigene34516\_Sample\_011046840, Unigene36320\_Sample\_011046840, Unigene36788\_Sample\_011046840, Unigene37698\_Sample\_011046840, Unigene38586\_Sample\_011046840, Unigene39100\_Sample\_011046840, Unigene39505\_Sample\_011046840, Unigene40478\_Sample\_011046840, Unigene40659\_Sample\_011046840, Unigene41174\_Sample\_011046840, Unigene41595\_Sample\_011046840, Unigene41713\_Sample\_011046840, Unigene42086\_Sample\_011046840, Unigene42509\_Sample\_011046840, Unigene42666\_Sample\_011046840, Unigene43068\_Sample\_011046840, Unigene334\_Sample\_011046840, Unigene7114\_Sample\_011046840, Unigene24869\_Sample\_011046840, Unigene26621\_Sample\_011046840, Unigene26711\_Sample\_011046840, Unigene30860\_Sample\_011046840, Unigene35901\_Sample\_011046840, Unigene39408\_Sample\_011046840, Unigene3170\_Sample\_011046840, Unigene4139\_Sample\_011046840, Unigene7372\_Sample\_011046840, Unigene7938\_Sample\_011046840, Unigene12147\_Sample\_011046840, Unigene16429\_Sample\_011046840, Unigene18933\_Sample\_011046840, Unigene21758\_Sample\_011046840, Unigene26785\_Sample\_011046840, Unigene27233\_Sample\_011046840, Unigene27378\_Sample\_011046840, Unigene28751\_Sample\_011046840, Unigene31705\_Sample\_011046840, Unigene31742\_Sample\_011046840, Unigene31880\_Sample\_011046840, Unigene33660\_Sample\_011046840, Unigene38267\_Sample\_011046840, Unigene39503\_Sample\_011046840, Unigene40189\_Sample\_011046840, Unigene42008\_Sample\_011046840, Unigene42326\_Sample\_011046840, Unigene43154\_Sample\_011046840, Unigene23714\_Sample\_011046840, Unigene30578\_Sample\_011046840, Unigene37680\_Sample\_011046840, Unigene40315\_Sample\_011046840, Unigene41909\_Sample\_011046840, Unigene1038\_Sample\_011046840, Unigene1368\_Sample\_011046840, Unigene4125\_Sample\_011046840, Unigene13531\_Sample\_011046840, Unigene14237\_Sample\_011046840, Unigene19400\_Sample\_011046840, Unigene20896\_Sample\_011046840, Unigene22254\_Sample\_011046840, Unigene25457\_Sample\_011046840, Unigene26767\_Sample\_011046840, Unigene29526\_Sample\_011046840, Unigene30703\_Sample\_011046840, Unigene33682\_Sample\_011046840, Unigene33870\_Sample\_011046840, Unigene35550\_Sample\_011046840, Unigene40235\_Sample\_011046840, Unigene40679\_Sample\_011046840, Unigene42811\_Sample\_011046840, Unigene42968\_Sample\_011046840, Unigene43092\_Sample\_011046840, Unigene670\_Sample\_011046840, Unigene5726\_Sample\_011046840, Unigene10083\_Sample\_011046840, Unigene11513\_Sample\_011046840, Unigene12229\_Sample\_011046840, Unigene19183\_Sample\_011046840, Unigene21176\_Sample\_011046840, Unigene27623\_Sample\_011046840, Unigene28202\_Sample\_011046840, Unigene29181\_Sample\_011046840, Unigene31320\_Sample\_011046840, Unigene32678\_Sample\_011046840, Unigene38781\_Sample\_011046840, Unigene39577\_Sample\_011046840, Unigene40703\_Sample\_011046840, Unigene41560\_Sample\_011046840, Unigene43217\_Sample\_011046840, Unigene2711\_Sample\_011046840, Unigene9230\_Sample\_011046840, Unigene10037\_Sample\_011046840, Unigene20757\_Sample\_011046840, Unigene26126\_Sample\_011046840, Unigene39880\_Sample\_011046840, Unigene41704\_Sample\_011046840, Unigene1568\_Sample\_011046840, Unigene4005\_Sample\_011046840, Unigene4352\_Sample\_011046840, Unigene7135\_Sample\_011046840, Unigene14227\_Sample\_011046840, Unigene15158\_Sample\_011046840, Unigene16035\_Sample\_011046840, Unigene17741\_Sample\_011046840, Unigene20420\_Sample\_011046840, Unigene23019\_Sample\_011046840, Unigene31471\_Sample\_011046840, Unigene32265\_Sample\_011046840, Unigene32307\_Sample\_011046840, Unigene33028\_Sample\_011046840, Unigene35039\_Sample\_011046840, Unigene35068\_Sample\_011046840, Unigene37491\_Sample\_011046840, Unigene11113\_Sample\_011046840, Unigene11179\_Sample\_011046840, Unigene16037\_Sample\_011046840, Unigene19593\_Sample\_011046840, Unigene20602\_Sample\_011046840, Unigene21138\_Sample\_011046840, Unigene31562\_Sample\_011046840, Unigene32453\_Sample\_011046840, Unigene33393\_Sample\_011046840, Unigene33711\_Sample\_011046840, Unigene36545\_Sample\_011046840, Unigene37186\_Sample\_011046840, Unigene43443\_Sample\_011046840 |
| 43 | RNA degradation Back to summary table | Unigene1815\_Sample\_011046840, Unigene7760\_Sample\_011046840, Unigene29404\_Sample\_011046840, Unigene31512\_Sample\_011046840, Unigene35459\_Sample\_011046840, Unigene39310\_Sample\_011046840, Unigene39357\_Sample\_011046840, Unigene41021\_Sample\_011046840, Unigene41866\_Sample\_011046840, Unigene1224\_Sample\_011046840, Unigene3641\_Sample\_011046840, Unigene5001\_Sample\_011046840, Unigene5076\_Sample\_011046840, Unigene6216\_Sample\_011046840, Unigene7208\_Sample\_011046840, Unigene7891\_Sample\_011046840, Unigene8187\_Sample\_011046840, Unigene8230\_Sample\_011046840, Unigene8313\_Sample\_011046840, Unigene12207\_Sample\_011046840, Unigene20083\_Sample\_011046840, Unigene20573\_Sample\_011046840, Unigene24761\_Sample\_011046840, Unigene25182\_Sample\_011046840, Unigene25775\_Sample\_011046840, Unigene27291\_Sample\_011046840, Unigene28697\_Sample\_011046840, Unigene29069\_Sample\_011046840, Unigene29637\_Sample\_011046840, Unigene30443\_Sample\_011046840, Unigene31068\_Sample\_011046840, Unigene31178\_Sample\_011046840, Unigene33370\_Sample\_011046840, Unigene33480\_Sample\_011046840, Unigene35428\_Sample\_011046840, Unigene36403\_Sample\_011046840, Unigene36749\_Sample\_011046840, Unigene37685\_Sample\_011046840, Unigene38801\_Sample\_011046840, Unigene40153\_Sample\_011046840, Unigene41392\_Sample\_011046840, Unigene41500\_Sample\_011046840, Unigene41735\_Sample\_011046840, Unigene42546\_Sample\_011046840, Unigene42789\_Sample\_011046840, Unigene43120\_Sample\_011046840, Unigene43381\_Sample\_011046840, Unigene17179\_Sample\_011046840, Unigene27520\_Sample\_011046840, Unigene31057\_Sample\_011046840, Unigene41432\_Sample\_011046840, Unigene42412\_Sample\_011046840, Unigene43113\_Sample\_011046840, Unigene12728\_Sample\_011046840, Unigene17011\_Sample\_011046840, Unigene19997\_Sample\_011046840, Unigene22066\_Sample\_011046840, Unigene28604\_Sample\_011046840, Unigene36610\_Sample\_011046840, Unigene38055\_Sample\_011046840, Unigene41084\_Sample\_011046840, Unigene6726\_Sample\_011046840, Unigene7130\_Sample\_011046840, Unigene14484\_Sample\_011046840, Unigene29507\_Sample\_011046840, Unigene31077\_Sample\_011046840, Unigene37260\_Sample\_011046840, Unigene37849\_Sample\_011046840, Unigene39570\_Sample\_011046840, Unigene40100\_Sample\_011046840, Unigene40784\_Sample\_011046840, Unigene41610\_Sample\_011046840, Unigene28548\_Sample\_011046840, Unigene30520\_Sample\_011046840, Unigene31137\_Sample\_011046840, Unigene33737\_Sample\_011046840, Unigene36833\_Sample\_011046840, Unigene39171\_Sample\_011046840, Unigene39700\_Sample\_011046840, Unigene43552\_Sample\_011046840, Unigene27975\_Sample\_011046840, Unigene28546\_Sample\_011046840, Unigene29951\_Sample\_011046840, Unigene32866\_Sample\_011046840, Unigene34164\_Sample\_011046840, Unigene36489\_Sample\_011046840, Unigene37871\_Sample\_011046840, Unigene38383\_Sample\_011046840, Unigene39234\_Sample\_011046840, Unigene6423\_Sample\_011046840, Unigene1608\_Sample\_011046840, Unigene2367\_Sample\_011046840, Unigene4562\_Sample\_011046840, Unigene11208\_Sample\_011046840, Unigene15548\_Sample\_011046840, Unigene19658\_Sample\_011046840, Unigene21248\_Sample\_011046840, Unigene21679\_Sample\_011046840, Unigene26919\_Sample\_011046840, Unigene29543\_Sample\_011046840, Unigene29992\_Sample\_011046840, Unigene30162\_Sample\_011046840, Unigene30941\_Sample\_011046840, Unigene31557\_Sample\_011046840, Unigene32012\_Sample\_011046840, Unigene32647\_Sample\_011046840, Unigene35784\_Sample\_011046840, Unigene37275\_Sample\_011046840, Unigene38914\_Sample\_011046840, Unigene39032\_Sample\_011046840, Unigene40324\_Sample\_011046840, Unigene41515\_Sample\_011046840, Unigene42583\_Sample\_011046840, Unigene43537\_Sample\_011046840, Unigene1309\_Sample\_011046840, Unigene5530\_Sample\_011046840, Unigene21410\_Sample\_011046840, Unigene33915\_Sample\_011046840, Unigene39279\_Sample\_011046840, Unigene39890\_Sample\_011046840, Unigene41616\_Sample\_011046840, Unigene41902\_Sample\_011046840, Unigene42193\_Sample\_011046840, Unigene28779\_Sample\_011046840, Unigene5461\_Sample\_011046840, Unigene10106\_Sample\_011046840, Unigene10531\_Sample\_011046840, Unigene19126\_Sample\_011046840, Unigene19216\_Sample\_011046840, Unigene20400\_Sample\_011046840, Unigene20445\_Sample\_011046840, Unigene21668\_Sample\_011046840, Unigene22645\_Sample\_011046840, Unigene28043\_Sample\_011046840, Unigene28881\_Sample\_011046840, Unigene30874\_Sample\_011046840, Unigene31663\_Sample\_011046840, Unigene32685\_Sample\_011046840, Unigene36458\_Sample\_011046840, Unigene18037\_Sample\_011046840, Unigene19260\_Sample\_011046840, Unigene24628\_Sample\_011046840, Unigene34715\_Sample\_011046840, Unigene37735\_Sample\_011046840 |
| 44 | Inositol phosphate metabolism Back to summary table | Unigene2822\_Sample\_011046840, Unigene5489\_Sample\_011046840, Unigene6899\_Sample\_011046840, Unigene34179\_Sample\_011046840, Unigene34602\_Sample\_011046840, Unigene37948\_Sample\_011046840, Unigene40745\_Sample\_011046840, Unigene41587\_Sample\_011046840, Unigene43510\_Sample\_011046840, Unigene132\_Sample\_011046840, Unigene810\_Sample\_011046840, Unigene1533\_Sample\_011046840, Unigene2536\_Sample\_011046840, Unigene2924\_Sample\_011046840, Unigene3812\_Sample\_011046840, Unigene4033\_Sample\_011046840, Unigene4969\_Sample\_011046840, Unigene5669\_Sample\_011046840, Unigene6457\_Sample\_011046840, Unigene7631\_Sample\_011046840, Unigene31836\_Sample\_011046840, Unigene32141\_Sample\_011046840, Unigene34974\_Sample\_011046840, Unigene36899\_Sample\_011046840, Unigene38259\_Sample\_011046840, Unigene38876\_Sample\_011046840, Unigene39237\_Sample\_011046840, Unigene40251\_Sample\_011046840, Unigene40284\_Sample\_011046840, Unigene40359\_Sample\_011046840, Unigene40934\_Sample\_011046840, Unigene41194\_Sample\_011046840, Unigene41417\_Sample\_011046840, Unigene42217\_Sample\_011046840, Unigene42256\_Sample\_011046840, Unigene42799\_Sample\_011046840, Unigene42996\_Sample\_011046840, Unigene43390\_Sample\_011046840, Unigene13195\_Sample\_011046840, Unigene34299\_Sample\_011046840, Unigene35076\_Sample\_011046840, Unigene42172\_Sample\_011046840, Unigene9080\_Sample\_011046840, Unigene28344\_Sample\_011046840, Unigene32981\_Sample\_011046840, Unigene39001\_Sample\_011046840, Unigene40435\_Sample\_011046840, Unigene40726\_Sample\_011046840, Unigene123\_Sample\_011046840, Unigene4635\_Sample\_011046840, Unigene5969\_Sample\_011046840, Unigene6398\_Sample\_011046840, Unigene13900\_Sample\_011046840, Unigene15586\_Sample\_011046840, Unigene17036\_Sample\_011046840, Unigene21632\_Sample\_011046840, Unigene40222\_Sample\_011046840, Unigene41243\_Sample\_011046840, Unigene43068\_Sample\_011046840, Unigene14179\_Sample\_011046840, Unigene24869\_Sample\_011046840, Unigene28872\_Sample\_011046840, Unigene40143\_Sample\_011046840, Unigene42153\_Sample\_011046840, Unigene5877\_Sample\_011046840, Unigene6926\_Sample\_011046840, Unigene26785\_Sample\_011046840, Unigene32648\_Sample\_011046840, Unigene40918\_Sample\_011046840, Unigene42008\_Sample\_011046840, Unigene42323\_Sample\_011046840, Unigene3689\_Sample\_011046840, Unigene26329\_Sample\_011046840, Unigene28542\_Sample\_011046840, Unigene7441\_Sample\_011046840, Unigene20896\_Sample\_011046840, Unigene42811\_Sample\_011046840, Unigene3044\_Sample\_011046840, Unigene8614\_Sample\_011046840, Unigene14465\_Sample\_011046840, Unigene14528\_Sample\_011046840, Unigene17714\_Sample\_011046840, Unigene24036\_Sample\_011046840, Unigene29065\_Sample\_011046840, Unigene33665\_Sample\_011046840, Unigene3017\_Sample\_011046840, Unigene4493\_Sample\_011046840, Unigene32062\_Sample\_011046840, Unigene41488\_Sample\_011046840, Unigene9889\_Sample\_011046840, Unigene10814\_Sample\_011046840, Unigene14938\_Sample\_011046840, Unigene28339\_Sample\_011046840, Unigene30077\_Sample\_011046840, Unigene32265\_Sample\_011046840, Unigene34487\_Sample\_011046840, Unigene36591\_Sample\_011046840, Unigene37794\_Sample\_011046840, Unigene38178\_Sample\_011046840, Unigene38668\_Sample\_011046840, Unigene41998\_Sample\_011046840, Unigene1548\_Sample\_011046840 |
| 45 | Cell cycle Back to summary table | Unigene2956\_Sample\_011046840, Unigene3123\_Sample\_011046840, Unigene7647\_Sample\_011046840, Unigene8055\_Sample\_011046840, Unigene13399\_Sample\_011046840, Unigene25448\_Sample\_011046840, Unigene25743\_Sample\_011046840, Unigene26863\_Sample\_011046840, Unigene27738\_Sample\_011046840, Unigene28917\_Sample\_011046840, Unigene31074\_Sample\_011046840, Unigene31248\_Sample\_011046840, Unigene33337\_Sample\_011046840, Unigene33631\_Sample\_011046840, Unigene33667\_Sample\_011046840, Unigene35895\_Sample\_011046840, Unigene35941\_Sample\_011046840, Unigene36784\_Sample\_011046840, Unigene38940\_Sample\_011046840, Unigene40260\_Sample\_011046840, Unigene40446\_Sample\_011046840, Unigene41242\_Sample\_011046840, Unigene41510\_Sample\_011046840, Unigene41512\_Sample\_011046840, Unigene41702\_Sample\_011046840, Unigene42859\_Sample\_011046840, Unigene43311\_Sample\_011046840, Unigene177\_Sample\_011046840, Unigene4497\_Sample\_011046840, Unigene4779\_Sample\_011046840, Unigene5746\_Sample\_011046840, Unigene7349\_Sample\_011046840, Unigene7768\_Sample\_011046840, Unigene8203\_Sample\_011046840, Unigene12359\_Sample\_011046840, Unigene14078\_Sample\_011046840, Unigene15111\_Sample\_011046840, Unigene16328\_Sample\_011046840, Unigene16882\_Sample\_011046840, Unigene18467\_Sample\_011046840, Unigene24698\_Sample\_011046840, Unigene25830\_Sample\_011046840, Unigene27185\_Sample\_011046840, Unigene30691\_Sample\_011046840, Unigene30813\_Sample\_011046840, Unigene32520\_Sample\_011046840, Unigene32593\_Sample\_011046840, Unigene32679\_Sample\_011046840, Unigene32791\_Sample\_011046840, Unigene36364\_Sample\_011046840, Unigene36846\_Sample\_011046840, Unigene36911\_Sample\_011046840, Unigene37219\_Sample\_011046840, Unigene37821\_Sample\_011046840, Unigene37890\_Sample\_011046840, Unigene37904\_Sample\_011046840, Unigene38463\_Sample\_011046840, Unigene39900\_Sample\_011046840, Unigene39948\_Sample\_011046840, Unigene40424\_Sample\_011046840, Unigene40685\_Sample\_011046840, Unigene41401\_Sample\_011046840, Unigene41444\_Sample\_011046840, Unigene41495\_Sample\_011046840, Unigene41501\_Sample\_011046840, Unigene41621\_Sample\_011046840, Unigene41673\_Sample\_011046840, Unigene42372\_Sample\_011046840, Unigene42510\_Sample\_011046840, Unigene43162\_Sample\_011046840, Unigene43273\_Sample\_011046840, Unigene43301\_Sample\_011046840, Unigene43395\_Sample\_011046840, Unigene43397\_Sample\_011046840, Unigene976\_Sample\_011046840, Unigene22946\_Sample\_011046840, Unigene23252\_Sample\_011046840, Unigene27034\_Sample\_011046840, Unigene33638\_Sample\_011046840, Unigene33954\_Sample\_011046840, Unigene35961\_Sample\_011046840, Unigene36400\_Sample\_011046840, Unigene36688\_Sample\_011046840, Unigene38540\_Sample\_011046840, Unigene40818\_Sample\_011046840, Unigene41114\_Sample\_011046840, Unigene42866\_Sample\_011046840, Unigene188\_Sample\_011046840, Unigene12743\_Sample\_011046840, Unigene16050\_Sample\_011046840, Unigene17494\_Sample\_011046840, Unigene19991\_Sample\_011046840, Unigene20800\_Sample\_011046840, Unigene24747\_Sample\_011046840, Unigene29542\_Sample\_011046840, Unigene31699\_Sample\_011046840, Unigene33396\_Sample\_011046840, Unigene33840\_Sample\_011046840, Unigene33984\_Sample\_011046840, Unigene37084\_Sample\_011046840, Unigene39200\_Sample\_011046840, Unigene41403\_Sample\_011046840, Unigene43111\_Sample\_011046840, Unigene5867\_Sample\_011046840, Unigene13449\_Sample\_011046840, Unigene13502\_Sample\_011046840, Unigene15502\_Sample\_011046840, Unigene16611\_Sample\_011046840, Unigene22321\_Sample\_011046840, Unigene22944\_Sample\_011046840, Unigene23873\_Sample\_011046840, Unigene24220\_Sample\_011046840, Unigene25328\_Sample\_011046840, Unigene25450\_Sample\_011046840, Unigene27478\_Sample\_011046840, Unigene27883\_Sample\_011046840, Unigene28259\_Sample\_011046840, Unigene28415\_Sample\_011046840, Unigene30865\_Sample\_011046840, Unigene32884\_Sample\_011046840, Unigene33849\_Sample\_011046840, Unigene36800\_Sample\_011046840, Unigene37121\_Sample\_011046840, Unigene38366\_Sample\_011046840, Unigene38504\_Sample\_011046840, Unigene38655\_Sample\_011046840, Unigene39040\_Sample\_011046840, Unigene41000\_Sample\_011046840, Unigene41337\_Sample\_011046840, Unigene41383\_Sample\_011046840, Unigene42169\_Sample\_011046840, Unigene43281\_Sample\_011046840, Unigene43486\_Sample\_011046840, Unigene5439\_Sample\_011046840, Unigene11573\_Sample\_011046840, Unigene16307\_Sample\_011046840, Unigene16618\_Sample\_011046840, Unigene22410\_Sample\_011046840, Unigene33222\_Sample\_011046840, Unigene34998\_Sample\_011046840, Unigene37919\_Sample\_011046840, Unigene38121\_Sample\_011046840, Unigene38245\_Sample\_011046840, Unigene38939\_Sample\_011046840, Unigene41125\_Sample\_011046840, Unigene42022\_Sample\_011046840, Unigene115\_Sample\_011046840, Unigene3084\_Sample\_011046840, Unigene3486\_Sample\_011046840, Unigene9938\_Sample\_011046840, Unigene11875\_Sample\_011046840, Unigene15135\_Sample\_011046840, Unigene18768\_Sample\_011046840, Unigene29122\_Sample\_011046840, Unigene29958\_Sample\_011046840, Unigene31705\_Sample\_011046840, Unigene32620\_Sample\_011046840, Unigene32645\_Sample\_011046840, Unigene32875\_Sample\_011046840, Unigene34436\_Sample\_011046840, Unigene36166\_Sample\_011046840, Unigene36897\_Sample\_011046840, Unigene38902\_Sample\_011046840, Unigene39391\_Sample\_011046840, Unigene41228\_Sample\_011046840, Unigene41425\_Sample\_011046840, Unigene41691\_Sample\_011046840, Unigene42927\_Sample\_011046840, Unigene5852\_Sample\_011046840, Unigene12320\_Sample\_011046840, Unigene30604\_Sample\_011046840, Unigene40665\_Sample\_011046840, Unigene8801\_Sample\_011046840, Unigene9678\_Sample\_011046840, Unigene19400\_Sample\_011046840, Unigene20050\_Sample\_011046840, Unigene22162\_Sample\_011046840, Unigene23923\_Sample\_011046840, Unigene25413\_Sample\_011046840, Unigene27358\_Sample\_011046840, Unigene29526\_Sample\_011046840, Unigene31648\_Sample\_011046840, Unigene32355\_Sample\_011046840, Unigene33161\_Sample\_011046840, Unigene36291\_Sample\_011046840, Unigene37605\_Sample\_011046840, Unigene39412\_Sample\_011046840, Unigene40038\_Sample\_011046840, Unigene42242\_Sample\_011046840, Unigene42293\_Sample\_011046840, Unigene2031\_Sample\_011046840, Unigene5383\_Sample\_011046840, Unigene14072\_Sample\_011046840, Unigene15271\_Sample\_011046840, Unigene24021\_Sample\_011046840, Unigene25871\_Sample\_011046840, Unigene25972\_Sample\_011046840, Unigene26435\_Sample\_011046840, Unigene30829\_Sample\_011046840, Unigene31266\_Sample\_011046840, Unigene33722\_Sample\_011046840, Unigene34096\_Sample\_011046840, Unigene36385\_Sample\_011046840, Unigene37505\_Sample\_011046840, Unigene37981\_Sample\_011046840, Unigene40019\_Sample\_011046840, Unigene40216\_Sample\_011046840, Unigene40873\_Sample\_011046840, Unigene10037\_Sample\_011046840, Unigene17478\_Sample\_011046840, Unigene22924\_Sample\_011046840, Unigene30201\_Sample\_011046840, Unigene35972\_Sample\_011046840, Unigene38690\_Sample\_011046840, Unigene42173\_Sample\_011046840, Unigene1344\_Sample\_011046840, Unigene10612\_Sample\_011046840, Unigene14154\_Sample\_011046840, Unigene16351\_Sample\_011046840, Unigene20430\_Sample\_011046840, Unigene21236\_Sample\_011046840, Unigene22078\_Sample\_011046840, Unigene22727\_Sample\_011046840, Unigene26500\_Sample\_011046840, Unigene26719\_Sample\_011046840, Unigene29411\_Sample\_011046840, Unigene31090\_Sample\_011046840, Unigene35789\_Sample\_011046840, Unigene38023\_Sample\_011046840, Unigene39224\_Sample\_011046840, Unigene41201\_Sample\_011046840, Unigene41690\_Sample\_011046840, Unigene2308\_Sample\_011046840, Unigene11742\_Sample\_011046840, Unigene15195\_Sample\_011046840, Unigene36506\_Sample\_011046840, Unigene36819\_Sample\_011046840, Unigene38610\_Sample\_011046840, Unigene41562\_Sample\_011046840 |
| 46 | Riboflavin metabolism Back to summary table | Unigene6088\_Sample\_011046840, Unigene7926\_Sample\_011046840, Unigene26088\_Sample\_011046840, Unigene28461\_Sample\_011046840, Unigene33240\_Sample\_011046840, Unigene40028\_Sample\_011046840, Unigene40674\_Sample\_011046840, Unigene42437\_Sample\_011046840, Unigene43265\_Sample\_011046840, Unigene43417\_Sample\_011046840, Unigene3749\_Sample\_011046840, Unigene6360\_Sample\_011046840, Unigene8031\_Sample\_011046840, Unigene26834\_Sample\_011046840, Unigene32708\_Sample\_011046840, Unigene33287\_Sample\_011046840, Unigene36197\_Sample\_011046840, Unigene41224\_Sample\_011046840, Unigene7264\_Sample\_011046840, Unigene39738\_Sample\_011046840, Unigene4758\_Sample\_011046840, Unigene7461\_Sample\_011046840, Unigene13827\_Sample\_011046840, Unigene3631\_Sample\_011046840, Unigene6461\_Sample\_011046840, Unigene29870\_Sample\_011046840, Unigene32318\_Sample\_011046840, Unigene28899\_Sample\_011046840, Unigene38646\_Sample\_011046840, Unigene13585\_Sample\_011046840, Unigene455\_Sample\_011046840, Unigene36976\_Sample\_011046840, Unigene40045\_Sample\_011046840, Unigene8079\_Sample\_011046840, Unigene21926\_Sample\_011046840, Unigene2383\_Sample\_011046840, Unigene2123\_Sample\_011046840, Unigene10233\_Sample\_011046840, Unigene29739\_Sample\_011046840, Unigene28070\_Sample\_011046840, Unigene31514\_Sample\_011046840, Unigene39685\_Sample\_011046840 |
| 47 | Histidine metabolism Back to summary table | Unigene21412\_Sample\_011046840, Unigene36841\_Sample\_011046840, Unigene38468\_Sample\_011046840, Unigene42263\_Sample\_011046840, Unigene3573\_Sample\_011046840, Unigene10034\_Sample\_011046840, Unigene41275\_Sample\_011046840, Unigene41585\_Sample\_011046840, Unigene24754\_Sample\_011046840, Unigene34616\_Sample\_011046840, Unigene4446\_Sample\_011046840, Unigene42521\_Sample\_011046840, Unigene7636\_Sample\_011046840, Unigene41484\_Sample\_011046840, Unigene7140\_Sample\_011046840, Unigene33907\_Sample\_011046840, Unigene16857\_Sample\_011046840, Unigene28923\_Sample\_011046840 |
| 48 | Chronic myeloid leukemia Back to summary table | Unigene1345\_Sample\_011046840, Unigene4790\_Sample\_011046840, Unigene8154\_Sample\_011046840, Unigene15939\_Sample\_011046840, Unigene37137\_Sample\_011046840, Unigene39096\_Sample\_011046840, Unigene40260\_Sample\_011046840, Unigene41702\_Sample\_011046840, Unigene43311\_Sample\_011046840, Unigene2615\_Sample\_011046840, Unigene2986\_Sample\_011046840, Unigene5389\_Sample\_011046840, Unigene7349\_Sample\_011046840, Unigene10748\_Sample\_011046840, Unigene24651\_Sample\_011046840, Unigene24698\_Sample\_011046840, Unigene26575\_Sample\_011046840, Unigene32380\_Sample\_011046840, Unigene34085\_Sample\_011046840, Unigene34974\_Sample\_011046840, Unigene38617\_Sample\_011046840, Unigene38866\_Sample\_011046840, Unigene40208\_Sample\_011046840, Unigene40657\_Sample\_011046840, Unigene41501\_Sample\_011046840, Unigene41584\_Sample\_011046840, Unigene41658\_Sample\_011046840, Unigene42164\_Sample\_011046840, Unigene43002\_Sample\_011046840, Unigene7853\_Sample\_011046840, Unigene8058\_Sample\_011046840, Unigene35961\_Sample\_011046840, Unigene38847\_Sample\_011046840, Unigene43149\_Sample\_011046840, Unigene16050\_Sample\_011046840, Unigene37084\_Sample\_011046840, Unigene38648\_Sample\_011046840, Unigene7408\_Sample\_011046840, Unigene8088\_Sample\_011046840, Unigene20325\_Sample\_011046840, Unigene23045\_Sample\_011046840, Unigene28708\_Sample\_011046840, Unigene33313\_Sample\_011046840, Unigene36320\_Sample\_011046840, Unigene38655\_Sample\_011046840, Unigene40659\_Sample\_011046840, Unigene43068\_Sample\_011046840, Unigene43497\_Sample\_011046840, Unigene21908\_Sample\_011046840, Unigene24869\_Sample\_011046840, Unigene26621\_Sample\_011046840, Unigene35901\_Sample\_011046840, Unigene38245\_Sample\_011046840, Unigene38301\_Sample\_011046840, Unigene4139\_Sample\_011046840, Unigene9938\_Sample\_011046840, Unigene14255\_Sample\_011046840, Unigene16429\_Sample\_011046840, Unigene18933\_Sample\_011046840, Unigene26785\_Sample\_011046840, Unigene27378\_Sample\_011046840, Unigene28751\_Sample\_011046840, Unigene31880\_Sample\_011046840, Unigene36897\_Sample\_011046840, Unigene38000\_Sample\_011046840, Unigene39503\_Sample\_011046840, Unigene40189\_Sample\_011046840, Unigene42008\_Sample\_011046840, Unigene43154\_Sample\_011046840, Unigene1368\_Sample\_011046840, Unigene15053\_Sample\_011046840, Unigene20896\_Sample\_011046840, Unigene30246\_Sample\_011046840, Unigene33870\_Sample\_011046840, Unigene42811\_Sample\_011046840, Unigene2209\_Sample\_011046840, Unigene5726\_Sample\_011046840, Unigene13067\_Sample\_011046840, Unigene14072\_Sample\_011046840, Unigene21176\_Sample\_011046840, Unigene27623\_Sample\_011046840, Unigene31320\_Sample\_011046840, Unigene41560\_Sample\_011046840, Unigene30201\_Sample\_011046840, Unigene30685\_Sample\_011046840, Unigene35972\_Sample\_011046840, Unigene38690\_Sample\_011046840, Unigene41704\_Sample\_011046840, Unigene16035\_Sample\_011046840, Unigene20420\_Sample\_011046840, Unigene26719\_Sample\_011046840, Unigene32265\_Sample\_011046840, Unigene37491\_Sample\_011046840, Unigene38841\_Sample\_011046840, Unigene41201\_Sample\_011046840, Unigene41410\_Sample\_011046840, Unigene41562\_Sample\_011046840 |
| 49 | Hedgehog signaling pathway Back to summary table | Unigene8223\_Sample\_011046840, Unigene35927\_Sample\_011046840, Unigene40124\_Sample\_011046840, Unigene41242\_Sample\_011046840, Unigene42343\_Sample\_011046840, Unigene42654\_Sample\_011046840, Unigene43124\_Sample\_011046840, Unigene43174\_Sample\_011046840, Unigene4668\_Sample\_011046840, Unigene4779\_Sample\_011046840, Unigene6245\_Sample\_011046840, Unigene7303\_Sample\_011046840, Unigene7893\_Sample\_011046840, Unigene16630\_Sample\_011046840, Unigene22845\_Sample\_011046840, Unigene24751\_Sample\_011046840, Unigene30430\_Sample\_011046840, Unigene32130\_Sample\_011046840, Unigene32730\_Sample\_011046840, Unigene35780\_Sample\_011046840, Unigene40128\_Sample\_011046840, Unigene40332\_Sample\_011046840, Unigene41234\_Sample\_011046840, Unigene41833\_Sample\_011046840, Unigene42065\_Sample\_011046840, Unigene42364\_Sample\_011046840, Unigene43301\_Sample\_011046840, Unigene4972\_Sample\_011046840, Unigene40404\_Sample\_011046840, Unigene2604\_Sample\_011046840, Unigene29542\_Sample\_011046840, Unigene31699\_Sample\_011046840, Unigene39804\_Sample\_011046840, Unigene41130\_Sample\_011046840, Unigene7429\_Sample\_011046840, Unigene11174\_Sample\_011046840, Unigene14624\_Sample\_011046840, Unigene14824\_Sample\_011046840, Unigene17905\_Sample\_011046840, Unigene26353\_Sample\_011046840, Unigene31945\_Sample\_011046840, Unigene35673\_Sample\_011046840, Unigene42488\_Sample\_011046840, Unigene42614\_Sample\_011046840, Unigene9530\_Sample\_011046840, Unigene35367\_Sample\_011046840, Unigene31705\_Sample\_011046840, Unigene37160\_Sample\_011046840, Unigene39891\_Sample\_011046840, Unigene6629\_Sample\_011046840, Unigene8569\_Sample\_011046840, Unigene43240\_Sample\_011046840, Unigene1495\_Sample\_011046840, Unigene11225\_Sample\_011046840, Unigene19400\_Sample\_011046840, Unigene26464\_Sample\_011046840, Unigene29526\_Sample\_011046840, Unigene32607\_Sample\_011046840, Unigene38611\_Sample\_011046840, Unigene39771\_Sample\_011046840, Unigene11390\_Sample\_011046840, Unigene34677\_Sample\_011046840, Unigene36070\_Sample\_011046840, Unigene10037\_Sample\_011046840, Unigene13188\_Sample\_011046840, Unigene35648\_Sample\_011046840, Unigene40680\_Sample\_011046840, Unigene11650\_Sample\_011046840, Unigene15316\_Sample\_011046840, Unigene19655\_Sample\_011046840, Unigene31611\_Sample\_011046840, Unigene36617\_Sample\_011046840, Unigene36628\_Sample\_011046840, Unigene40896\_Sample\_011046840, Unigene24378\_Sample\_011046840, Unigene29848\_Sample\_011046840 |
| 50 | Non-homologous end-joining Back to summary table | Unigene41600\_Sample\_011046840, Unigene42357\_Sample\_011046840, Unigene42875\_Sample\_011046840, Unigene43375\_Sample\_011046840, Unigene5041\_Sample\_011046840, Unigene7902\_Sample\_011046840, Unigene12550\_Sample\_011046840, Unigene20014\_Sample\_011046840, Unigene22265\_Sample\_011046840, Unigene34230\_Sample\_011046840, Unigene34573\_Sample\_011046840, Unigene38747\_Sample\_011046840, Unigene39275\_Sample\_011046840, Unigene40049\_Sample\_011046840, Unigene21598\_Sample\_011046840, Unigene42224\_Sample\_011046840, Unigene43246\_Sample\_011046840, Unigene37705\_Sample\_011046840, Unigene4723\_Sample\_011046840, Unigene8081\_Sample\_011046840, Unigene28451\_Sample\_011046840, Unigene40919\_Sample\_011046840, Unigene41288\_Sample\_011046840, Unigene35476\_Sample\_011046840, Unigene15290\_Sample\_011046840, Unigene38443\_Sample\_011046840, Unigene7475\_Sample\_011046840, Unigene25346\_Sample\_011046840, Unigene9887\_Sample\_011046840, Unigene25767\_Sample\_011046840, Unigene33479\_Sample\_011046840, Unigene38754\_Sample\_011046840, Unigene11948\_Sample\_011046840 |
| 51 | Dilated cardiomyopathy Back to summary table | Unigene259\_Sample\_011046840, Unigene4515\_Sample\_011046840, Unigene4981\_Sample\_011046840, Unigene5147\_Sample\_011046840, Unigene5831\_Sample\_011046840, Unigene6576\_Sample\_011046840, Unigene6630\_Sample\_011046840, Unigene8231\_Sample\_011046840, Unigene9987\_Sample\_011046840, Unigene23056\_Sample\_011046840, Unigene23133\_Sample\_011046840, Unigene25850\_Sample\_011046840, Unigene31309\_Sample\_011046840, Unigene40596\_Sample\_011046840, Unigene41683\_Sample\_011046840, Unigene41900\_Sample\_011046840, Unigene42639\_Sample\_011046840, Unigene42826\_Sample\_011046840, Unigene42884\_Sample\_011046840, Unigene43073\_Sample\_011046840, Unigene43429\_Sample\_011046840, Unigene43439\_Sample\_011046840, Unigene4028\_Sample\_011046840, Unigene5214\_Sample\_011046840, Unigene5795\_Sample\_011046840, Unigene7704\_Sample\_011046840, Unigene8012\_Sample\_011046840, Unigene28163\_Sample\_011046840, Unigene29336\_Sample\_011046840, Unigene32879\_Sample\_011046840, Unigene38045\_Sample\_011046840, Unigene40128\_Sample\_011046840, Unigene41237\_Sample\_011046840, Unigene41630\_Sample\_011046840, Unigene41856\_Sample\_011046840, Unigene42065\_Sample\_011046840, Unigene42336\_Sample\_011046840, Unigene42424\_Sample\_011046840, Unigene42819\_Sample\_011046840, Unigene42838\_Sample\_011046840, Unigene43004\_Sample\_011046840, Unigene43213\_Sample\_011046840, Unigene43258\_Sample\_011046840, Unigene43312\_Sample\_011046840, Unigene43481\_Sample\_011046840, Unigene43547\_Sample\_011046840, Unigene448\_Sample\_011046840, Unigene4511\_Sample\_011046840, Unigene8133\_Sample\_011046840, Unigene25969\_Sample\_011046840, Unigene29603\_Sample\_011046840, Unigene36775\_Sample\_011046840, Unigene42303\_Sample\_011046840, Unigene42603\_Sample\_011046840, Unigene7605\_Sample\_011046840, Unigene23177\_Sample\_011046840, Unigene27657\_Sample\_011046840, Unigene36373\_Sample\_011046840, Unigene41719\_Sample\_011046840, Unigene43466\_Sample\_011046840, Unigene3872\_Sample\_011046840, Unigene5364\_Sample\_011046840, Unigene25431\_Sample\_011046840, Unigene31860\_Sample\_011046840, Unigene31889\_Sample\_011046840, Unigene31945\_Sample\_011046840, Unigene34942\_Sample\_011046840, Unigene35114\_Sample\_011046840, Unigene35443\_Sample\_011046840, Unigene37336\_Sample\_011046840, Unigene39625\_Sample\_011046840, Unigene41345\_Sample\_011046840, Unigene41485\_Sample\_011046840, Unigene42110\_Sample\_011046840, Unigene42913\_Sample\_011046840, Unigene3758\_Sample\_011046840, Unigene28892\_Sample\_011046840, Unigene34520\_Sample\_011046840, Unigene36619\_Sample\_011046840, Unigene38616\_Sample\_011046840, Unigene41881\_Sample\_011046840, Unigene42693\_Sample\_011046840, Unigene43250\_Sample\_011046840, Unigene43309\_Sample\_011046840, Unigene50\_Sample\_011046840, Unigene6035\_Sample\_011046840, Unigene7196\_Sample\_011046840, Unigene24545\_Sample\_011046840, Unigene32118\_Sample\_011046840, Unigene36856\_Sample\_011046840, Unigene43015\_Sample\_011046840, Unigene26493\_Sample\_011046840, Unigene34848\_Sample\_011046840, Unigene42677\_Sample\_011046840, Unigene6846\_Sample\_011046840, Unigene42212\_Sample\_011046840, Unigene2815\_Sample\_011046840, Unigene3219\_Sample\_011046840, Unigene24388\_Sample\_011046840, Unigene36060\_Sample\_011046840, Unigene38423\_Sample\_011046840, Unigene40056\_Sample\_011046840, Unigene40892\_Sample\_011046840, Unigene12868\_Sample\_011046840, Unigene15237\_Sample\_011046840, Unigene31135\_Sample\_011046840, Unigene39360\_Sample\_011046840, Unigene4030\_Sample\_011046840, Unigene25727\_Sample\_011046840, Unigene29797\_Sample\_011046840, Unigene35925\_Sample\_011046840, Unigene37909\_Sample\_011046840, Unigene43398\_Sample\_011046840, Unigene5921\_Sample\_011046840, Unigene21744\_Sample\_011046840, Unigene40453\_Sample\_011046840, Unigene43443\_Sample\_011046840 |
| 52 | Prostate cancer Back to summary table | Unigene2132\_Sample\_011046840, Unigene4790\_Sample\_011046840, Unigene7744\_Sample\_011046840, Unigene15939\_Sample\_011046840, Unigene24311\_Sample\_011046840, Unigene29592\_Sample\_011046840, Unigene41242\_Sample\_011046840, Unigene43211\_Sample\_011046840, Unigene2986\_Sample\_011046840, Unigene4320\_Sample\_011046840, Unigene4497\_Sample\_011046840, Unigene4779\_Sample\_011046840, Unigene5425\_Sample\_011046840, Unigene7529\_Sample\_011046840, Unigene8009\_Sample\_011046840, Unigene11592\_Sample\_011046840, Unigene24698\_Sample\_011046840, Unigene28752\_Sample\_011046840, Unigene30720\_Sample\_011046840, Unigene33208\_Sample\_011046840, Unigene34974\_Sample\_011046840, Unigene36690\_Sample\_011046840, Unigene38612\_Sample\_011046840, Unigene38617\_Sample\_011046840, Unigene38866\_Sample\_011046840, Unigene39237\_Sample\_011046840, Unigene39437\_Sample\_011046840, Unigene40208\_Sample\_011046840, Unigene41584\_Sample\_011046840, Unigene42164\_Sample\_011046840, Unigene43210\_Sample\_011046840, Unigene43301\_Sample\_011046840, Unigene43492\_Sample\_011046840, Unigene43635\_Sample\_011046840, Unigene976\_Sample\_011046840, Unigene3954\_Sample\_011046840, Unigene8372\_Sample\_011046840, Unigene8904\_Sample\_011046840, Unigene32594\_Sample\_011046840, Unigene33983\_Sample\_011046840, Unigene38834\_Sample\_011046840, Unigene38847\_Sample\_011046840, Unigene42204\_Sample\_011046840, Unigene43149\_Sample\_011046840, Unigene4268\_Sample\_011046840, Unigene29542\_Sample\_011046840, Unigene31699\_Sample\_011046840, Unigene35727\_Sample\_011046840, Unigene39571\_Sample\_011046840, Unigene39778\_Sample\_011046840, Unigene2808\_Sample\_011046840, Unigene20325\_Sample\_011046840, Unigene23045\_Sample\_011046840, Unigene28708\_Sample\_011046840, Unigene35981\_Sample\_011046840, Unigene36320\_Sample\_011046840, Unigene42509\_Sample\_011046840, Unigene43068\_Sample\_011046840, Unigene8240\_Sample\_011046840, Unigene24869\_Sample\_011046840, Unigene25701\_Sample\_011046840, Unigene30860\_Sample\_011046840, Unigene38245\_Sample\_011046840, Unigene4139\_Sample\_011046840, Unigene12153\_Sample\_011046840, Unigene16429\_Sample\_011046840, Unigene18933\_Sample\_011046840, Unigene26785\_Sample\_011046840, Unigene27378\_Sample\_011046840, Unigene28751\_Sample\_011046840, Unigene31705\_Sample\_011046840, Unigene31880\_Sample\_011046840, Unigene35787\_Sample\_011046840, Unigene39503\_Sample\_011046840, Unigene40189\_Sample\_011046840, Unigene42008\_Sample\_011046840, Unigene43154\_Sample\_011046840, Unigene1368\_Sample\_011046840, Unigene17776\_Sample\_011046840, Unigene19400\_Sample\_011046840, Unigene20896\_Sample\_011046840, Unigene25523\_Sample\_011046840, Unigene29526\_Sample\_011046840, Unigene33870\_Sample\_011046840, Unigene42811\_Sample\_011046840, Unigene5726\_Sample\_011046840, Unigene19918\_Sample\_011046840, Unigene21176\_Sample\_011046840, Unigene27623\_Sample\_011046840, Unigene41560\_Sample\_011046840, Unigene10037\_Sample\_011046840, Unigene20949\_Sample\_011046840, Unigene35972\_Sample\_011046840, Unigene41704\_Sample\_011046840, Unigene9721\_Sample\_011046840, Unigene16035\_Sample\_011046840, Unigene20420\_Sample\_011046840, Unigene31090\_Sample\_011046840, Unigene32265\_Sample\_011046840, Unigene33028\_Sample\_011046840, Unigene34975\_Sample\_011046840, Unigene41410\_Sample\_011046840, Unigene33393\_Sample\_011046840 |
| 53 | Protein export Back to summary table | Unigene5915\_Sample\_011046840, Unigene8084\_Sample\_011046840, Unigene13497\_Sample\_011046840, Unigene35913\_Sample\_011046840, Unigene40530\_Sample\_011046840, Unigene6129\_Sample\_011046840, Unigene6394\_Sample\_011046840, Unigene21153\_Sample\_011046840, Unigene23468\_Sample\_011046840, Unigene34833\_Sample\_011046840, Unigene42198\_Sample\_011046840, Unigene1278\_Sample\_011046840, Unigene14530\_Sample\_011046840, Unigene31281\_Sample\_011046840, Unigene38001\_Sample\_011046840, Unigene16269\_Sample\_011046840, Unigene29099\_Sample\_011046840, Unigene35505\_Sample\_011046840, Unigene8733\_Sample\_011046840, Unigene19142\_Sample\_011046840, Unigene20351\_Sample\_011046840, Unigene28104\_Sample\_011046840, Unigene35905\_Sample\_011046840, Unigene38739\_Sample\_011046840, Unigene42194\_Sample\_011046840, Unigene8452\_Sample\_011046840, Unigene11248\_Sample\_011046840, Unigene16959\_Sample\_011046840, Unigene9442\_Sample\_011046840, Unigene5956\_Sample\_011046840, Unigene28670\_Sample\_011046840, Unigene32612\_Sample\_011046840, Unigene38065\_Sample\_011046840, Unigene39033\_Sample\_011046840, Unigene2196\_Sample\_011046840, Unigene13746\_Sample\_011046840, Unigene37224\_Sample\_011046840, Unigene41042\_Sample\_011046840, Unigene30831\_Sample\_011046840, Unigene35090\_Sample\_011046840, Unigene11668\_Sample\_011046840, Unigene12506\_Sample\_011046840, Unigene18559\_Sample\_011046840, Unigene31467\_Sample\_011046840, Unigene32745\_Sample\_011046840, Unigene41077\_Sample\_011046840, Unigene21157\_Sample\_011046840 |
| 54 | MAPK signaling pathway Back to summary table | Unigene228\_Sample\_011046840, Unigene259\_Sample\_011046840, Unigene535\_Sample\_011046840, Unigene880\_Sample\_011046840, Unigene1345\_Sample\_011046840, Unigene2132\_Sample\_011046840, Unigene2690\_Sample\_011046840, Unigene4790\_Sample\_011046840, Unigene4993\_Sample\_011046840, Unigene5831\_Sample\_011046840, Unigene6209\_Sample\_011046840, Unigene6551\_Sample\_011046840, Unigene7525\_Sample\_011046840, Unigene7856\_Sample\_011046840, Unigene8069\_Sample\_011046840, Unigene8198\_Sample\_011046840, Unigene15939\_Sample\_011046840, Unigene16605\_Sample\_011046840, Unigene30862\_Sample\_011046840, Unigene33750\_Sample\_011046840, Unigene34362\_Sample\_011046840, Unigene37697\_Sample\_011046840, Unigene38206\_Sample\_011046840, Unigene38826\_Sample\_011046840, Unigene39833\_Sample\_011046840, Unigene40471\_Sample\_011046840, Unigene42127\_Sample\_011046840, Unigene42658\_Sample\_011046840, Unigene42759\_Sample\_011046840, Unigene42766\_Sample\_011046840, Unigene42826\_Sample\_011046840, Unigene42844\_Sample\_011046840, Unigene43095\_Sample\_011046840, Unigene43176\_Sample\_011046840, Unigene43319\_Sample\_011046840, Unigene43330\_Sample\_011046840, Unigene43332\_Sample\_011046840, Unigene43365\_Sample\_011046840, Unigene43441\_Sample\_011046840, Unigene789\_Sample\_011046840, Unigene2986\_Sample\_011046840, Unigene4017\_Sample\_011046840, Unigene4043\_Sample\_011046840, Unigene4101\_Sample\_011046840, Unigene4561\_Sample\_011046840, Unigene4775\_Sample\_011046840, Unigene5059\_Sample\_011046840, Unigene5564\_Sample\_011046840, Unigene7132\_Sample\_011046840, Unigene7232\_Sample\_011046840, Unigene7299\_Sample\_011046840, Unigene7529\_Sample\_011046840, Unigene7868\_Sample\_011046840, Unigene8009\_Sample\_011046840, Unigene10033\_Sample\_011046840, Unigene11592\_Sample\_011046840, Unigene14894\_Sample\_011046840, Unigene19620\_Sample\_011046840, Unigene28710\_Sample\_011046840, Unigene29457\_Sample\_011046840, Unigene29711\_Sample\_011046840, Unigene30539\_Sample\_011046840, Unigene30767\_Sample\_011046840, Unigene32180\_Sample\_011046840, Unigene32368\_Sample\_011046840, Unigene33208\_Sample\_011046840, Unigene33865\_Sample\_011046840, Unigene33939\_Sample\_011046840, Unigene34147\_Sample\_011046840, Unigene35796\_Sample\_011046840, Unigene36317\_Sample\_011046840, Unigene36690\_Sample\_011046840, Unigene37326\_Sample\_011046840, Unigene37579\_Sample\_011046840, Unigene38866\_Sample\_011046840, Unigene38946\_Sample\_011046840, Unigene39437\_Sample\_011046840, Unigene39843\_Sample\_011046840, Unigene39883\_Sample\_011046840, Unigene40128\_Sample\_011046840, Unigene40208\_Sample\_011046840, Unigene40322\_Sample\_011046840, Unigene40414\_Sample\_011046840, Unigene40570\_Sample\_011046840, Unigene40972\_Sample\_011046840, Unigene41204\_Sample\_011046840, Unigene41370\_Sample\_011046840, Unigene41584\_Sample\_011046840, Unigene41915\_Sample\_011046840, Unigene42065\_Sample\_011046840, Unigene42164\_Sample\_011046840, Unigene42607\_Sample\_011046840, Unigene43002\_Sample\_011046840, Unigene43046\_Sample\_011046840, Unigene43138\_Sample\_011046840, Unigene43210\_Sample\_011046840, Unigene43305\_Sample\_011046840, Unigene43421\_Sample\_011046840, Unigene43435\_Sample\_011046840, Unigene43578\_Sample\_011046840, Unigene43582\_Sample\_011046840, Unigene143\_Sample\_011046840, Unigene1246\_Sample\_011046840, Unigene3608\_Sample\_011046840, Unigene7144\_Sample\_011046840, Unigene8904\_Sample\_011046840, Unigene14437\_Sample\_011046840, Unigene23432\_Sample\_011046840, Unigene29880\_Sample\_011046840, Unigene33579\_Sample\_011046840, Unigene33835\_Sample\_011046840, Unigene33983\_Sample\_011046840, Unigene34039\_Sample\_011046840, Unigene38834\_Sample\_011046840, Unigene38847\_Sample\_011046840, Unigene42204\_Sample\_011046840, Unigene42227\_Sample\_011046840, Unigene43638\_Sample\_011046840, Unigene1549\_Sample\_011046840, Unigene3161\_Sample\_011046840, Unigene6322\_Sample\_011046840, Unigene6735\_Sample\_011046840, Unigene6921\_Sample\_011046840, Unigene16645\_Sample\_011046840, Unigene25603\_Sample\_011046840, Unigene29090\_Sample\_011046840, Unigene36839\_Sample\_011046840, Unigene36951\_Sample\_011046840, Unigene38648\_Sample\_011046840, Unigene40221\_Sample\_011046840, Unigene2808\_Sample\_011046840, Unigene3691\_Sample\_011046840, Unigene4069\_Sample\_011046840, Unigene4807\_Sample\_011046840, Unigene7951\_Sample\_011046840, Unigene12635\_Sample\_011046840, Unigene13215\_Sample\_011046840, Unigene13661\_Sample\_011046840, Unigene13908\_Sample\_011046840, Unigene14856\_Sample\_011046840, Unigene20325\_Sample\_011046840, Unigene20824\_Sample\_011046840, Unigene23045\_Sample\_011046840, Unigene25579\_Sample\_011046840, Unigene27023\_Sample\_011046840, Unigene28708\_Sample\_011046840, Unigene30049\_Sample\_011046840, Unigene30600\_Sample\_011046840, Unigene31079\_Sample\_011046840, Unigene31703\_Sample\_011046840, Unigene31945\_Sample\_011046840, Unigene33646\_Sample\_011046840, Unigene36320\_Sample\_011046840, Unigene36860\_Sample\_011046840, Unigene37235\_Sample\_011046840, Unigene38756\_Sample\_011046840, Unigene39243\_Sample\_011046840, Unigene39621\_Sample\_011046840, Unigene40659\_Sample\_011046840, Unigene40838\_Sample\_011046840, Unigene41059\_Sample\_011046840, Unigene41269\_Sample\_011046840, Unigene41539\_Sample\_011046840, Unigene41579\_Sample\_011046840, Unigene41854\_Sample\_011046840, Unigene42268\_Sample\_011046840, Unigene42487\_Sample\_011046840, Unigene42666\_Sample\_011046840, Unigene42707\_Sample\_011046840, Unigene5791\_Sample\_011046840, Unigene26621\_Sample\_011046840, Unigene28124\_Sample\_011046840, Unigene33873\_Sample\_011046840, Unigene35503\_Sample\_011046840, Unigene35901\_Sample\_011046840, Unigene37954\_Sample\_011046840, Unigene3212\_Sample\_011046840, Unigene4139\_Sample\_011046840, Unigene12153\_Sample\_011046840, Unigene16429\_Sample\_011046840, Unigene16876\_Sample\_011046840, Unigene22839\_Sample\_011046840, Unigene24891\_Sample\_011046840, Unigene25548\_Sample\_011046840, Unigene27378\_Sample\_011046840, Unigene28751\_Sample\_011046840, Unigene29670\_Sample\_011046840, Unigene31802\_Sample\_011046840, Unigene31880\_Sample\_011046840, Unigene35532\_Sample\_011046840, Unigene35787\_Sample\_011046840, Unigene36910\_Sample\_011046840, Unigene39503\_Sample\_011046840, Unigene39958\_Sample\_011046840, Unigene40189\_Sample\_011046840, Unigene40501\_Sample\_011046840, Unigene40648\_Sample\_011046840, Unigene41425\_Sample\_011046840, Unigene42385\_Sample\_011046840, Unigene43154\_Sample\_011046840, Unigene2706\_Sample\_011046840, Unigene36227\_Sample\_011046840, Unigene1368\_Sample\_011046840, Unigene1740\_Sample\_011046840, Unigene4849\_Sample\_011046840, Unigene6944\_Sample\_011046840, Unigene10749\_Sample\_011046840, Unigene14237\_Sample\_011046840, Unigene17676\_Sample\_011046840, Unigene18009\_Sample\_011046840, Unigene26398\_Sample\_011046840, Unigene26552\_Sample\_011046840, Unigene29660\_Sample\_011046840, Unigene32479\_Sample\_011046840, Unigene33870\_Sample\_011046840, Unigene34894\_Sample\_011046840, Unigene37041\_Sample\_011046840, Unigene39294\_Sample\_011046840, Unigene39723\_Sample\_011046840, Unigene5726\_Sample\_011046840, Unigene19127\_Sample\_011046840, Unigene21176\_Sample\_011046840, Unigene22338\_Sample\_011046840, Unigene27623\_Sample\_011046840, Unigene41560\_Sample\_011046840, Unigene32864\_Sample\_011046840, Unigene33692\_Sample\_011046840, Unigene38608\_Sample\_011046840, Unigene39271\_Sample\_011046840, Unigene1568\_Sample\_011046840, Unigene11201\_Sample\_011046840, Unigene14930\_Sample\_011046840, Unigene16035\_Sample\_011046840, Unigene18949\_Sample\_011046840, Unigene20038\_Sample\_011046840, Unigene20420\_Sample\_011046840, Unigene23668\_Sample\_011046840, Unigene25727\_Sample\_011046840, Unigene29621\_Sample\_011046840, Unigene30094\_Sample\_011046840, Unigene30573\_Sample\_011046840, Unigene32011\_Sample\_011046840, Unigene33594\_Sample\_011046840, Unigene35687\_Sample\_011046840, Unigene41410\_Sample\_011046840, Unigene41689\_Sample\_011046840, Unigene1379\_Sample\_011046840, Unigene16016\_Sample\_011046840, Unigene19593\_Sample\_011046840, Unigene24889\_Sample\_011046840, Unigene31562\_Sample\_011046840, Unigene33809\_Sample\_011046840, Unigene37186\_Sample\_011046840, Unigene37304\_Sample\_011046840, Unigene37517\_Sample\_011046840, Unigene38088\_Sample\_011046840 |
| 55 | Glycosaminoglycan biosynthesis - keratan sulfate (no map in kegg database) Back to summary table | Unigene29877\_Sample\_011046840, Unigene34282\_Sample\_011046840, Unigene36547\_Sample\_011046840, Unigene20494\_Sample\_011046840, Unigene21897\_Sample\_011046840, Unigene42959\_Sample\_011046840, Unigene779\_Sample\_011046840, Unigene39751\_Sample\_011046840, Unigene40727\_Sample\_011046840 |
| 56 | Long-term potentiation Back to summary table | Unigene880\_Sample\_011046840, Unigene1129\_Sample\_011046840, Unigene2690\_Sample\_011046840, Unigene2865\_Sample\_011046840, Unigene5662\_Sample\_011046840, Unigene5831\_Sample\_011046840, Unigene6644\_Sample\_011046840, Unigene7525\_Sample\_011046840, Unigene8140\_Sample\_011046840, Unigene15939\_Sample\_011046840, Unigene30862\_Sample\_011046840, Unigene34179\_Sample\_011046840, Unigene43180\_Sample\_011046840, Unigene2986\_Sample\_011046840, Unigene3474\_Sample\_011046840, Unigene3812\_Sample\_011046840, Unigene4101\_Sample\_011046840, Unigene4477\_Sample\_011046840, Unigene6139\_Sample\_011046840, Unigene6500\_Sample\_011046840, Unigene7868\_Sample\_011046840, Unigene36317\_Sample\_011046840, Unigene40128\_Sample\_011046840, Unigene40208\_Sample\_011046840, Unigene41550\_Sample\_011046840, Unigene41584\_Sample\_011046840, Unigene42065\_Sample\_011046840, Unigene42217\_Sample\_011046840, Unigene42284\_Sample\_011046840, Unigene42477\_Sample\_011046840, Unigene42991\_Sample\_011046840, Unigene43481\_Sample\_011046840, Unigene976\_Sample\_011046840, Unigene3608\_Sample\_011046840, Unigene5166\_Sample\_011046840, Unigene3161\_Sample\_011046840, Unigene6322\_Sample\_011046840, Unigene7605\_Sample\_011046840, Unigene28116\_Sample\_011046840, Unigene39054\_Sample\_011046840, Unigene634\_Sample\_011046840, Unigene4610\_Sample\_011046840, Unigene4807\_Sample\_011046840, Unigene10400\_Sample\_011046840, Unigene28708\_Sample\_011046840, Unigene31945\_Sample\_011046840, Unigene36788\_Sample\_011046840, Unigene36860\_Sample\_011046840, Unigene39621\_Sample\_011046840, Unigene334\_Sample\_011046840, Unigene14179\_Sample\_011046840, Unigene28124\_Sample\_011046840, Unigene28872\_Sample\_011046840, Unigene37954\_Sample\_011046840, Unigene7372\_Sample\_011046840, Unigene16429\_Sample\_011046840, Unigene27233\_Sample\_011046840, Unigene27378\_Sample\_011046840, Unigene31880\_Sample\_011046840, Unigene39503\_Sample\_011046840, Unigene39912\_Sample\_011046840, Unigene40648\_Sample\_011046840, Unigene42385\_Sample\_011046840, Unigene43154\_Sample\_011046840, Unigene23714\_Sample\_011046840, Unigene41909\_Sample\_011046840, Unigene4125\_Sample\_011046840, Unigene4849\_Sample\_011046840, Unigene6944\_Sample\_011046840, Unigene13531\_Sample\_011046840, Unigene17676\_Sample\_011046840, Unigene22818\_Sample\_011046840, Unigene30703\_Sample\_011046840, Unigene32479\_Sample\_011046840, Unigene33870\_Sample\_011046840, Unigene37392\_Sample\_011046840, Unigene670\_Sample\_011046840, Unigene8614\_Sample\_011046840, Unigene12229\_Sample\_011046840, Unigene39577\_Sample\_011046840, Unigene2711\_Sample\_011046840, Unigene13511\_Sample\_011046840, Unigene32864\_Sample\_011046840, Unigene33692\_Sample\_011046840, Unigene37218\_Sample\_011046840, Unigene7388\_Sample\_011046840, Unigene11201\_Sample\_011046840, Unigene14227\_Sample\_011046840, Unigene20420\_Sample\_011046840, Unigene23019\_Sample\_011046840, Unigene34487\_Sample\_011046840, Unigene15244\_Sample\_011046840, Unigene31562\_Sample\_011046840, Unigene36447\_Sample\_011046840, Unigene43443\_Sample\_011046840 |
| 57 | Selenoamino acid metabolism Back to summary table | Unigene6749\_Sample\_011046840, Unigene36841\_Sample\_011046840, Unigene36918\_Sample\_011046840, Unigene38468\_Sample\_011046840, Unigene39991\_Sample\_011046840, Unigene41261\_Sample\_011046840, Unigene43348\_Sample\_011046840, Unigene3288\_Sample\_011046840, Unigene6826\_Sample\_011046840, Unigene7262\_Sample\_011046840, Unigene10034\_Sample\_011046840, Unigene20848\_Sample\_011046840, Unigene29145\_Sample\_011046840, Unigene40541\_Sample\_011046840, Unigene41275\_Sample\_011046840, Unigene41585\_Sample\_011046840, Unigene43069\_Sample\_011046840, Unigene24754\_Sample\_011046840, Unigene30367\_Sample\_011046840, Unigene34616\_Sample\_011046840, Unigene2180\_Sample\_011046840, Unigene25716\_Sample\_011046840, Unigene7636\_Sample\_011046840, Unigene41484\_Sample\_011046840, Unigene7140\_Sample\_011046840, Unigene4045\_Sample\_011046840, Unigene33907\_Sample\_011046840, Unigene36922\_Sample\_011046840, Unigene5168\_Sample\_011046840, Unigene20968\_Sample\_011046840, Unigene41140\_Sample\_011046840, Unigene985\_Sample\_011046840, Unigene26805\_Sample\_011046840, Unigene28923\_Sample\_011046840, Unigene38934\_Sample\_011046840, Unigene42596\_Sample\_011046840, Unigene10829\_Sample\_011046840, Unigene24148\_Sample\_011046840, Unigene30592\_Sample\_011046840 |
| 58 | Cytokine-cytokine receptor interaction Back to summary table | Unigene5379\_Sample\_011046840, Unigene6378\_Sample\_011046840, Unigene28188\_Sample\_011046840, Unigene41643\_Sample\_011046840, Unigene43193\_Sample\_011046840, Unigene43556\_Sample\_011046840, Unigene6996\_Sample\_011046840, Unigene7091\_Sample\_011046840, Unigene8009\_Sample\_011046840, Unigene11592\_Sample\_011046840, Unigene33208\_Sample\_011046840, Unigene36690\_Sample\_011046840, Unigene39938\_Sample\_011046840, Unigene42460\_Sample\_011046840, Unigene43225\_Sample\_011046840, Unigene8904\_Sample\_011046840, Unigene33983\_Sample\_011046840, Unigene35074\_Sample\_011046840, Unigene38834\_Sample\_011046840, Unigene42204\_Sample\_011046840, Unigene43115\_Sample\_011046840, Unigene3335\_Sample\_011046840, Unigene32463\_Sample\_011046840, Unigene7250\_Sample\_011046840, Unigene14323\_Sample\_011046840, Unigene15148\_Sample\_011046840, Unigene12153\_Sample\_011046840, Unigene21594\_Sample\_011046840, Unigene35061\_Sample\_011046840, Unigene35787\_Sample\_011046840, Unigene39613\_Sample\_011046840, Unigene643\_Sample\_011046840, Unigene12234\_Sample\_011046840, Unigene24335\_Sample\_011046840, Unigene18122\_Sample\_011046840, Unigene4487\_Sample\_011046840, Unigene30136\_Sample\_011046840, Unigene34843\_Sample\_011046840, Unigene19027\_Sample\_011046840 |
| 59 | gamma-Hexachlorocyclohexane degradation Back to summary table | Unigene5648\_Sample\_011046840, Unigene27934\_Sample\_011046840, Unigene30845\_Sample\_011046840, Unigene33240\_Sample\_011046840, Unigene40028\_Sample\_011046840, Unigene43265\_Sample\_011046840, Unigene8031\_Sample\_011046840, Unigene39738\_Sample\_011046840, Unigene27109\_Sample\_011046840, Unigene36523\_Sample\_011046840, Unigene6461\_Sample\_011046840, Unigene32318\_Sample\_011046840, Unigene28899\_Sample\_011046840, Unigene38646\_Sample\_011046840, Unigene28894\_Sample\_011046840, Unigene14381\_Sample\_011046840, Unigene17097\_Sample\_011046840, Unigene27664\_Sample\_011046840, Unigene31775\_Sample\_011046840, Unigene40045\_Sample\_011046840, Unigene2383\_Sample\_011046840, Unigene28199\_Sample\_011046840, Unigene9792\_Sample\_011046840, Unigene31514\_Sample\_011046840 |
| 60 | ABC transporters Back to summary table | Unigene2421\_Sample\_011046840, Unigene4623\_Sample\_011046840, Unigene7895\_Sample\_011046840, Unigene7952\_Sample\_011046840, Unigene26216\_Sample\_011046840, Unigene36141\_Sample\_011046840, Unigene38720\_Sample\_011046840, Unigene39795\_Sample\_011046840, Unigene41047\_Sample\_011046840, Unigene4955\_Sample\_011046840, Unigene5249\_Sample\_011046840, Unigene7230\_Sample\_011046840, Unigene8036\_Sample\_011046840, Unigene8113\_Sample\_011046840, Unigene9443\_Sample\_011046840, Unigene11441\_Sample\_011046840, Unigene26581\_Sample\_011046840, Unigene31273\_Sample\_011046840, Unigene35008\_Sample\_011046840, Unigene35951\_Sample\_011046840, Unigene37200\_Sample\_011046840, Unigene37353\_Sample\_011046840, Unigene37754\_Sample\_011046840, Unigene39499\_Sample\_011046840, Unigene40032\_Sample\_011046840, Unigene42647\_Sample\_011046840, Unigene3599\_Sample\_011046840, Unigene41867\_Sample\_011046840, Unigene6313\_Sample\_011046840, Unigene6547\_Sample\_011046840, Unigene5880\_Sample\_011046840, Unigene14116\_Sample\_011046840, Unigene25020\_Sample\_011046840, Unigene31864\_Sample\_011046840, Unigene34972\_Sample\_011046840, Unigene37745\_Sample\_011046840, Unigene38585\_Sample\_011046840, Unigene40445\_Sample\_011046840, Unigene40687\_Sample\_011046840, Unigene3064\_Sample\_011046840, Unigene4389\_Sample\_011046840, Unigene36345\_Sample\_011046840, Unigene36983\_Sample\_011046840, Unigene41626\_Sample\_011046840, Unigene5013\_Sample\_011046840, Unigene12033\_Sample\_011046840, Unigene27829\_Sample\_011046840, Unigene40236\_Sample\_011046840, Unigene41352\_Sample\_011046840, Unigene20428\_Sample\_011046840, Unigene8211\_Sample\_011046840, Unigene10704\_Sample\_011046840, Unigene26930\_Sample\_011046840, Unigene36150\_Sample\_011046840, Unigene41476\_Sample\_011046840, Unigene5066\_Sample\_011046840, Unigene15030\_Sample\_011046840, Unigene23158\_Sample\_011046840, Unigene34400\_Sample\_011046840, Unigene37921\_Sample\_011046840, Unigene40520\_Sample\_011046840, Unigene41858\_Sample\_011046840, Unigene41898\_Sample\_011046840, Unigene19647\_Sample\_011046840, Unigene36088\_Sample\_011046840, Unigene31318\_Sample\_011046840, Unigene31732\_Sample\_011046840, Unigene39490\_Sample\_011046840, Unigene42530\_Sample\_011046840, Unigene4518\_Sample\_011046840, Unigene24115\_Sample\_011046840, Unigene29805\_Sample\_011046840, Unigene39351\_Sample\_011046840 |
| 61 | Taurine and hypotaurine metabolism Back to summary table | Unigene36918\_Sample\_011046840, Unigene3288\_Sample\_011046840, Unigene6826\_Sample\_011046840, Unigene41442\_Sample\_011046840, Unigene7058\_Sample\_011046840, Unigene5168\_Sample\_011046840, Unigene26805\_Sample\_011046840, Unigene30592\_Sample\_011046840 |
| 62 | Regulation of actin cytoskeleton Back to summary table | Unigene1129\_Sample\_011046840, Unigene2132\_Sample\_011046840, Unigene2865\_Sample\_011046840, Unigene4341\_Sample\_011046840, Unigene4875\_Sample\_011046840, Unigene4993\_Sample\_011046840, Unigene6209\_Sample\_011046840, Unigene6644\_Sample\_011046840, Unigene7195\_Sample\_011046840, Unigene7975\_Sample\_011046840, Unigene8050\_Sample\_011046840, Unigene8140\_Sample\_011046840, Unigene8154\_Sample\_011046840, Unigene9987\_Sample\_011046840, Unigene10035\_Sample\_011046840, Unigene10232\_Sample\_011046840, Unigene15939\_Sample\_011046840, Unigene23133\_Sample\_011046840, Unigene25850\_Sample\_011046840, Unigene27834\_Sample\_011046840, Unigene27840\_Sample\_011046840, Unigene32514\_Sample\_011046840, Unigene35023\_Sample\_011046840, Unigene37793\_Sample\_011046840, Unigene38706\_Sample\_011046840, Unigene39384\_Sample\_011046840, Unigene40152\_Sample\_011046840, Unigene40484\_Sample\_011046840, Unigene40745\_Sample\_011046840, Unigene41220\_Sample\_011046840, Unigene42639\_Sample\_011046840, Unigene42844\_Sample\_011046840, Unigene42956\_Sample\_011046840, Unigene43118\_Sample\_011046840, Unigene43429\_Sample\_011046840, Unigene43470\_Sample\_011046840, Unigene43514\_Sample\_011046840, Unigene1487\_Sample\_011046840, Unigene2986\_Sample\_011046840, Unigene3158\_Sample\_011046840, Unigene3474\_Sample\_011046840, Unigene3903\_Sample\_011046840, Unigene4057\_Sample\_011046840, Unigene4477\_Sample\_011046840, Unigene6065\_Sample\_011046840, Unigene6139\_Sample\_011046840, Unigene6863\_Sample\_011046840, Unigene7529\_Sample\_011046840, Unigene7631\_Sample\_011046840, Unigene8009\_Sample\_011046840, Unigene8185\_Sample\_011046840, Unigene11592\_Sample\_011046840, Unigene16996\_Sample\_011046840, Unigene27902\_Sample\_011046840, Unigene28418\_Sample\_011046840, Unigene28742\_Sample\_011046840, Unigene29916\_Sample\_011046840, Unigene33208\_Sample\_011046840, Unigene33883\_Sample\_011046840, Unigene34974\_Sample\_011046840, Unigene36690\_Sample\_011046840, Unigene38045\_Sample\_011046840, Unigene38435\_Sample\_011046840, Unigene38617\_Sample\_011046840, Unigene38866\_Sample\_011046840, Unigene39220\_Sample\_011046840, Unigene39437\_Sample\_011046840, Unigene39714\_Sample\_011046840, Unigene39843\_Sample\_011046840, Unigene39883\_Sample\_011046840, Unigene40208\_Sample\_011046840, Unigene40273\_Sample\_011046840, Unigene40388\_Sample\_011046840, Unigene41370\_Sample\_011046840, Unigene41550\_Sample\_011046840, Unigene41584\_Sample\_011046840, Unigene41856\_Sample\_011046840, Unigene41915\_Sample\_011046840, Unigene41949\_Sample\_011046840, Unigene41968\_Sample\_011046840, Unigene41996\_Sample\_011046840, Unigene42067\_Sample\_011046840, Unigene42284\_Sample\_011046840, Unigene42349\_Sample\_011046840, Unigene42356\_Sample\_011046840, Unigene42477\_Sample\_011046840, Unigene42812\_Sample\_011046840, Unigene42991\_Sample\_011046840, Unigene42996\_Sample\_011046840, Unigene43018\_Sample\_011046840, Unigene43202\_Sample\_011046840, Unigene43210\_Sample\_011046840, Unigene43258\_Sample\_011046840, Unigene43312\_Sample\_011046840, Unigene43342\_Sample\_011046840, Unigene43557\_Sample\_011046840, Unigene43576\_Sample\_011046840, Unigene2247\_Sample\_011046840, Unigene6402\_Sample\_011046840, Unigene8133\_Sample\_011046840, Unigene8904\_Sample\_011046840, Unigene25969\_Sample\_011046840, Unigene25979\_Sample\_011046840, Unigene26624\_Sample\_011046840, Unigene33673\_Sample\_011046840, Unigene33835\_Sample\_011046840, Unigene33983\_Sample\_011046840, Unigene38834\_Sample\_011046840, Unigene40469\_Sample\_011046840, Unigene41695\_Sample\_011046840, Unigene42204\_Sample\_011046840, Unigene43149\_Sample\_011046840, Unigene43608\_Sample\_011046840, Unigene1053\_Sample\_011046840, Unigene4449\_Sample\_011046840, Unigene6869\_Sample\_011046840, Unigene7782\_Sample\_011046840, Unigene21306\_Sample\_011046840, Unigene22038\_Sample\_011046840, Unigene27657\_Sample\_011046840, Unigene28116\_Sample\_011046840, Unigene35929\_Sample\_011046840, Unigene37705\_Sample\_011046840, Unigene38648\_Sample\_011046840, Unigene40726\_Sample\_011046840, Unigene42798\_Sample\_011046840, Unigene43466\_Sample\_011046840, Unigene634\_Sample\_011046840, Unigene2808\_Sample\_011046840, Unigene4279\_Sample\_011046840, Unigene4610\_Sample\_011046840, Unigene4818\_Sample\_011046840, Unigene5364\_Sample\_011046840, Unigene10400\_Sample\_011046840, Unigene13908\_Sample\_011046840, Unigene14485\_Sample\_011046840, Unigene18272\_Sample\_011046840, Unigene19332\_Sample\_011046840, Unigene20325\_Sample\_011046840, Unigene21554\_Sample\_011046840, Unigene25431\_Sample\_011046840, Unigene28708\_Sample\_011046840, Unigene28883\_Sample\_011046840, Unigene30530\_Sample\_011046840, Unigene31079\_Sample\_011046840, Unigene33288\_Sample\_011046840, Unigene35150\_Sample\_011046840, Unigene35443\_Sample\_011046840, Unigene36320\_Sample\_011046840, Unigene36734\_Sample\_011046840, Unigene39625\_Sample\_011046840, Unigene40659\_Sample\_011046840, Unigene41303\_Sample\_011046840, Unigene41345\_Sample\_011046840, Unigene41539\_Sample\_011046840, Unigene42079\_Sample\_011046840, Unigene42487\_Sample\_011046840, Unigene42871\_Sample\_011046840, Unigene43068\_Sample\_011046840, Unigene43392\_Sample\_011046840, Unigene14379\_Sample\_011046840, Unigene24869\_Sample\_011046840, Unigene26621\_Sample\_011046840, Unigene30141\_Sample\_011046840, Unigene31181\_Sample\_011046840, Unigene34563\_Sample\_011046840, Unigene35901\_Sample\_011046840, Unigene38616\_Sample\_011046840, Unigene41881\_Sample\_011046840, Unigene42142\_Sample\_011046840, Unigene42770\_Sample\_011046840, Unigene7372\_Sample\_011046840, Unigene12153\_Sample\_011046840, Unigene16429\_Sample\_011046840, Unigene17368\_Sample\_011046840, Unigene18933\_Sample\_011046840, Unigene24257\_Sample\_011046840, Unigene26785\_Sample\_011046840, Unigene27233\_Sample\_011046840, Unigene27378\_Sample\_011046840, Unigene28610\_Sample\_011046840, Unigene28751\_Sample\_011046840, Unigene29282\_Sample\_011046840, Unigene29670\_Sample\_011046840, Unigene29722\_Sample\_011046840, Unigene31880\_Sample\_011046840, Unigene32648\_Sample\_011046840, Unigene35787\_Sample\_011046840, Unigene36662\_Sample\_011046840, Unigene36952\_Sample\_011046840, Unigene36992\_Sample\_011046840, Unigene39503\_Sample\_011046840, Unigene40189\_Sample\_011046840, Unigene42008\_Sample\_011046840, Unigene43154\_Sample\_011046840, Unigene2706\_Sample\_011046840, Unigene13348\_Sample\_011046840, Unigene23714\_Sample\_011046840, Unigene32609\_Sample\_011046840, Unigene34490\_Sample\_011046840, Unigene40259\_Sample\_011046840, Unigene40960\_Sample\_011046840, Unigene41909\_Sample\_011046840, Unigene42027\_Sample\_011046840, Unigene42677\_Sample\_011046840, Unigene4125\_Sample\_011046840, Unigene6846\_Sample\_011046840, Unigene13531\_Sample\_011046840, Unigene19307\_Sample\_011046840, Unigene20896\_Sample\_011046840, Unigene22818\_Sample\_011046840, Unigene24972\_Sample\_011046840, Unigene25317\_Sample\_011046840, Unigene28829\_Sample\_011046840, Unigene30703\_Sample\_011046840, Unigene33870\_Sample\_011046840, Unigene34466\_Sample\_011046840, Unigene35904\_Sample\_011046840, Unigene38572\_Sample\_011046840, Unigene38882\_Sample\_011046840, Unigene41947\_Sample\_011046840, Unigene42811\_Sample\_011046840, Unigene2815\_Sample\_011046840, Unigene3219\_Sample\_011046840, Unigene12229\_Sample\_011046840, Unigene15260\_Sample\_011046840, Unigene19127\_Sample\_011046840, Unigene25531\_Sample\_011046840, Unigene27623\_Sample\_011046840, Unigene35081\_Sample\_011046840, Unigene38423\_Sample\_011046840, Unigene41560\_Sample\_011046840, Unigene42805\_Sample\_011046840, Unigene42902\_Sample\_011046840, Unigene5106\_Sample\_011046840, Unigene12163\_Sample\_011046840, Unigene12868\_Sample\_011046840, Unigene15237\_Sample\_011046840, Unigene22950\_Sample\_011046840, Unigene24184\_Sample\_011046840, Unigene25527\_Sample\_011046840, Unigene26406\_Sample\_011046840, Unigene31135\_Sample\_011046840, Unigene37218\_Sample\_011046840, Unigene38066\_Sample\_011046840, Unigene41704\_Sample\_011046840, Unigene1256\_Sample\_011046840, Unigene4276\_Sample\_011046840, Unigene10814\_Sample\_011046840, Unigene14227\_Sample\_011046840, Unigene14930\_Sample\_011046840, Unigene17677\_Sample\_011046840, Unigene20420\_Sample\_011046840, Unigene21308\_Sample\_011046840, Unigene23019\_Sample\_011046840, Unigene23699\_Sample\_011046840, Unigene24464\_Sample\_011046840, Unigene25276\_Sample\_011046840, Unigene28339\_Sample\_011046840, Unigene32265\_Sample\_011046840, Unigene34059\_Sample\_011046840, Unigene37909\_Sample\_011046840, Unigene41056\_Sample\_011046840, Unigene42184\_Sample\_011046840, Unigene5921\_Sample\_011046840, Unigene18706\_Sample\_011046840, Unigene31562\_Sample\_011046840, Unigene31926\_Sample\_011046840, Unigene36447\_Sample\_011046840, Unigene37185\_Sample\_011046840 |
| 63 | Arrhythmogenic right ventricular cardiomyopathy (ARVC) Back to summary table | Unigene259\_Sample\_011046840, Unigene4515\_Sample\_011046840, Unigene4981\_Sample\_011046840, Unigene5831\_Sample\_011046840, Unigene9987\_Sample\_011046840, Unigene25850\_Sample\_011046840, Unigene41683\_Sample\_011046840, Unigene42639\_Sample\_011046840, Unigene42826\_Sample\_011046840, Unigene43073\_Sample\_011046840, Unigene43211\_Sample\_011046840, Unigene43429\_Sample\_011046840, Unigene7979\_Sample\_011046840, Unigene8012\_Sample\_011046840, Unigene27291\_Sample\_011046840, Unigene28418\_Sample\_011046840, Unigene33370\_Sample\_011046840, Unigene38045\_Sample\_011046840, Unigene38612\_Sample\_011046840, Unigene41237\_Sample\_011046840, Unigene42334\_Sample\_011046840, Unigene42336\_Sample\_011046840, Unigene42838\_Sample\_011046840, Unigene43258\_Sample\_011046840, Unigene43312\_Sample\_011046840, Unigene43492\_Sample\_011046840, Unigene8133\_Sample\_011046840, Unigene25969\_Sample\_011046840, Unigene33673\_Sample\_011046840, Unigene42603\_Sample\_011046840, Unigene27657\_Sample\_011046840, Unigene28604\_Sample\_011046840, Unigene41719\_Sample\_011046840, Unigene43466\_Sample\_011046840, Unigene5364\_Sample\_011046840, Unigene25431\_Sample\_011046840, Unigene31889\_Sample\_011046840, Unigene35981\_Sample\_011046840, Unigene37336\_Sample\_011046840, Unigene39625\_Sample\_011046840, Unigene40784\_Sample\_011046840, Unigene41345\_Sample\_011046840, Unigene3758\_Sample\_011046840, Unigene34520\_Sample\_011046840, Unigene38616\_Sample\_011046840, Unigene41881\_Sample\_011046840, Unigene28241\_Sample\_011046840, Unigene36662\_Sample\_011046840, Unigene43590\_Sample\_011046840, Unigene42677\_Sample\_011046840, Unigene6846\_Sample\_011046840, Unigene25523\_Sample\_011046840, Unigene30941\_Sample\_011046840, Unigene2815\_Sample\_011046840, Unigene3219\_Sample\_011046840, Unigene36060\_Sample\_011046840, Unigene38423\_Sample\_011046840, Unigene40056\_Sample\_011046840, Unigene12868\_Sample\_011046840, Unigene15237\_Sample\_011046840, Unigene31135\_Sample\_011046840, Unigene24464\_Sample\_011046840, Unigene25727\_Sample\_011046840, Unigene37909\_Sample\_011046840, Unigene5921\_Sample\_011046840 |
| 64 | Acute myeloid leukemia Back to summary table | Unigene4790\_Sample\_011046840, Unigene15939\_Sample\_011046840, Unigene24311\_Sample\_011046840, Unigene27951\_Sample\_011046840, Unigene29592\_Sample\_011046840, Unigene2615\_Sample\_011046840, Unigene2986\_Sample\_011046840, Unigene5389\_Sample\_011046840, Unigene5425\_Sample\_011046840, Unigene24698\_Sample\_011046840, Unigene30720\_Sample\_011046840, Unigene34974\_Sample\_011046840, Unigene38617\_Sample\_011046840, Unigene38866\_Sample\_011046840, Unigene40208\_Sample\_011046840, Unigene41584\_Sample\_011046840, Unigene42164\_Sample\_011046840, Unigene43509\_Sample\_011046840, Unigene38847\_Sample\_011046840, Unigene43149\_Sample\_011046840, Unigene20325\_Sample\_011046840, Unigene23045\_Sample\_011046840, Unigene23820\_Sample\_011046840, Unigene28708\_Sample\_011046840, Unigene36320\_Sample\_011046840, Unigene43068\_Sample\_011046840, Unigene24869\_Sample\_011046840, Unigene4139\_Sample\_011046840, Unigene16429\_Sample\_011046840, Unigene18933\_Sample\_011046840, Unigene26785\_Sample\_011046840, Unigene27378\_Sample\_011046840, Unigene28751\_Sample\_011046840, Unigene31880\_Sample\_011046840, Unigene38000\_Sample\_011046840, Unigene39503\_Sample\_011046840, Unigene40189\_Sample\_011046840, Unigene42008\_Sample\_011046840, Unigene43154\_Sample\_011046840, Unigene332\_Sample\_011046840, Unigene1368\_Sample\_011046840, Unigene20896\_Sample\_011046840, Unigene22254\_Sample\_011046840, Unigene25523\_Sample\_011046840, Unigene33870\_Sample\_011046840, Unigene42811\_Sample\_011046840, Unigene5726\_Sample\_011046840, Unigene21176\_Sample\_011046840, Unigene27623\_Sample\_011046840, Unigene29928\_Sample\_011046840, Unigene41560\_Sample\_011046840, Unigene35972\_Sample\_011046840, Unigene41704\_Sample\_011046840, Unigene16035\_Sample\_011046840, Unigene20420\_Sample\_011046840, Unigene26172\_Sample\_011046840, Unigene32265\_Sample\_011046840, Unigene41410\_Sample\_011046840 |
| 65 | Tight junction Back to summary table | Unigene880\_Sample\_011046840, Unigene1715\_Sample\_011046840, Unigene2914\_Sample\_011046840, Unigene7153\_Sample\_011046840, Unigene7825\_Sample\_011046840, Unigene8035\_Sample\_011046840, Unigene29549\_Sample\_011046840, Unigene30862\_Sample\_011046840, Unigene32488\_Sample\_011046840, Unigene35027\_Sample\_011046840, Unigene36474\_Sample\_011046840, Unigene36475\_Sample\_011046840, Unigene38498\_Sample\_011046840, Unigene38511\_Sample\_011046840, Unigene38728\_Sample\_011046840, Unigene38729\_Sample\_011046840, Unigene40260\_Sample\_011046840, Unigene40280\_Sample\_011046840, Unigene41600\_Sample\_011046840, Unigene42144\_Sample\_011046840, Unigene43188\_Sample\_011046840, Unigene43211\_Sample\_011046840, Unigene43216\_Sample\_011046840, Unigene43345\_Sample\_011046840, Unigene43493\_Sample\_011046840, Unigene43577\_Sample\_011046840, Unigene3205\_Sample\_011046840, Unigene3454\_Sample\_011046840, Unigene3740\_Sample\_011046840, Unigene3776\_Sample\_011046840, Unigene4101\_Sample\_011046840, Unigene4695\_Sample\_011046840, Unigene6240\_Sample\_011046840, Unigene6863\_Sample\_011046840, Unigene7382\_Sample\_011046840, Unigene7460\_Sample\_011046840, Unigene7709\_Sample\_011046840, Unigene7784\_Sample\_011046840, Unigene7979\_Sample\_011046840, Unigene8145\_Sample\_011046840, Unigene8185\_Sample\_011046840, Unigene11146\_Sample\_011046840, Unigene13250\_Sample\_011046840, Unigene15987\_Sample\_011046840, Unigene17210\_Sample\_011046840, Unigene20491\_Sample\_011046840, Unigene23826\_Sample\_011046840, Unigene27291\_Sample\_011046840, Unigene28418\_Sample\_011046840, Unigene29211\_Sample\_011046840, Unigene30842\_Sample\_011046840, Unigene33356\_Sample\_011046840, Unigene33370\_Sample\_011046840, Unigene34234\_Sample\_011046840, Unigene35044\_Sample\_011046840, Unigene35657\_Sample\_011046840, Unigene36568\_Sample\_011046840, Unigene37526\_Sample\_011046840, Unigene38045\_Sample\_011046840, Unigene38269\_Sample\_011046840, Unigene38432\_Sample\_011046840, Unigene38612\_Sample\_011046840, Unigene38973\_Sample\_011046840, Unigene39237\_Sample\_011046840, Unigene40157\_Sample\_011046840, Unigene41707\_Sample\_011046840, Unigene41924\_Sample\_011046840, Unigene42164\_Sample\_011046840, Unigene42237\_Sample\_011046840, Unigene42340\_Sample\_011046840, Unigene42479\_Sample\_011046840, Unigene42573\_Sample\_011046840, Unigene42704\_Sample\_011046840, Unigene42716\_Sample\_011046840, Unigene42833\_Sample\_011046840, Unigene43044\_Sample\_011046840, Unigene43175\_Sample\_011046840, Unigene43258\_Sample\_011046840, Unigene43312\_Sample\_011046840, Unigene43433\_Sample\_011046840, Unigene43468\_Sample\_011046840, Unigene43476\_Sample\_011046840, Unigene43492\_Sample\_011046840, Unigene43517\_Sample\_011046840, Unigene43540\_Sample\_011046840, Unigene43544\_Sample\_011046840, Unigene43558\_Sample\_011046840, Unigene2080\_Sample\_011046840, Unigene2156\_Sample\_011046840, Unigene3608\_Sample\_011046840, Unigene13248\_Sample\_011046840, Unigene33383\_Sample\_011046840, Unigene33673\_Sample\_011046840, Unigene35869\_Sample\_011046840, Unigene37037\_Sample\_011046840, Unigene38847\_Sample\_011046840, Unigene41589\_Sample\_011046840, Unigene3161\_Sample\_011046840, Unigene6322\_Sample\_011046840, Unigene6764\_Sample\_011046840, Unigene7958\_Sample\_011046840, Unigene27657\_Sample\_011046840, Unigene28087\_Sample\_011046840, Unigene28604\_Sample\_011046840, Unigene36038\_Sample\_011046840, Unigene36299\_Sample\_011046840, Unigene36467\_Sample\_011046840, Unigene37705\_Sample\_011046840, Unigene37716\_Sample\_011046840, Unigene38670\_Sample\_011046840, Unigene40250\_Sample\_011046840, Unigene40565\_Sample\_011046840, Unigene41082\_Sample\_011046840, Unigene43387\_Sample\_011046840, Unigene1249\_Sample\_011046840, Unigene3718\_Sample\_011046840, Unigene4596\_Sample\_011046840, Unigene4807\_Sample\_011046840, Unigene4818\_Sample\_011046840, Unigene5364\_Sample\_011046840, Unigene6495\_Sample\_011046840, Unigene7678\_Sample\_011046840, Unigene10107\_Sample\_011046840, Unigene11481\_Sample\_011046840, Unigene13127\_Sample\_011046840, Unigene14395\_Sample\_011046840, Unigene17971\_Sample\_011046840, Unigene25431\_Sample\_011046840, Unigene29294\_Sample\_011046840, Unigene32340\_Sample\_011046840, Unigene35981\_Sample\_011046840, Unigene36566\_Sample\_011046840, Unigene37301\_Sample\_011046840, Unigene37437\_Sample\_011046840, Unigene37551\_Sample\_011046840, Unigene38692\_Sample\_011046840, Unigene38794\_Sample\_011046840, Unigene39375\_Sample\_011046840, Unigene40784\_Sample\_011046840, Unigene40888\_Sample\_011046840, Unigene41246\_Sample\_011046840, Unigene41539\_Sample\_011046840, Unigene41873\_Sample\_011046840, Unigene42211\_Sample\_011046840, Unigene42531\_Sample\_011046840, Unigene42685\_Sample\_011046840, Unigene43224\_Sample\_011046840, Unigene43455\_Sample\_011046840, Unigene43473\_Sample\_011046840, Unigene2581\_Sample\_011046840, Unigene7822\_Sample\_011046840, Unigene8074\_Sample\_011046840, Unigene10956\_Sample\_011046840, Unigene22765\_Sample\_011046840, Unigene33409\_Sample\_011046840, Unigene37128\_Sample\_011046840, Unigene38616\_Sample\_011046840, Unigene38962\_Sample\_011046840, Unigene39303\_Sample\_011046840, Unigene41881\_Sample\_011046840, Unigene956\_Sample\_011046840, Unigene4139\_Sample\_011046840, Unigene7073\_Sample\_011046840, Unigene20146\_Sample\_011046840, Unigene25382\_Sample\_011046840, Unigene32413\_Sample\_011046840, Unigene35781\_Sample\_011046840, Unigene36662\_Sample\_011046840, Unigene37510\_Sample\_011046840, Unigene39503\_Sample\_011046840, Unigene40256\_Sample\_011046840, Unigene40648\_Sample\_011046840, Unigene42385\_Sample\_011046840, Unigene43532\_Sample\_011046840, Unigene43590\_Sample\_011046840, Unigene2706\_Sample\_011046840, Unigene7089\_Sample\_011046840, Unigene14082\_Sample\_011046840, Unigene23541\_Sample\_011046840, Unigene36227\_Sample\_011046840, Unigene43428\_Sample\_011046840, Unigene1368\_Sample\_011046840, Unigene4849\_Sample\_011046840, Unigene6846\_Sample\_011046840, Unigene10832\_Sample\_011046840, Unigene22459\_Sample\_011046840, Unigene24411\_Sample\_011046840, Unigene27954\_Sample\_011046840, Unigene30222\_Sample\_011046840, Unigene30941\_Sample\_011046840, Unigene31900\_Sample\_011046840, Unigene32176\_Sample\_011046840, Unigene32319\_Sample\_011046840, Unigene34966\_Sample\_011046840, Unigene39887\_Sample\_011046840, Unigene40751\_Sample\_011046840, Unigene40944\_Sample\_011046840, Unigene43338\_Sample\_011046840, Unigene2815\_Sample\_011046840, Unigene3219\_Sample\_011046840, Unigene5726\_Sample\_011046840, Unigene19127\_Sample\_011046840, Unigene19183\_Sample\_011046840, Unigene21176\_Sample\_011046840, Unigene30892\_Sample\_011046840, Unigene39850\_Sample\_011046840, Unigene42423\_Sample\_011046840, Unigene42902\_Sample\_011046840, Unigene42967\_Sample\_011046840, Unigene3861\_Sample\_011046840, Unigene4092\_Sample\_011046840, Unigene5569\_Sample\_011046840, Unigene12868\_Sample\_011046840, Unigene15237\_Sample\_011046840, Unigene16211\_Sample\_011046840, Unigene22950\_Sample\_011046840, Unigene23373\_Sample\_011046840, Unigene28952\_Sample\_011046840, Unigene31135\_Sample\_011046840, Unigene33307\_Sample\_011046840, Unigene33692\_Sample\_011046840, Unigene3046\_Sample\_011046840, Unigene8509\_Sample\_011046840, Unigene11201\_Sample\_011046840, Unigene14438\_Sample\_011046840, Unigene15057\_Sample\_011046840, Unigene15851\_Sample\_011046840, Unigene16035\_Sample\_011046840, Unigene18758\_Sample\_011046840, Unigene20606\_Sample\_011046840, Unigene22685\_Sample\_011046840, Unigene23260\_Sample\_011046840, Unigene24464\_Sample\_011046840, Unigene30242\_Sample\_011046840, Unigene42506\_Sample\_011046840, Unigene5921\_Sample\_011046840, Unigene26023\_Sample\_011046840, Unigene27955\_Sample\_011046840, Unigene29269\_Sample\_011046840, Unigene34398\_Sample\_011046840, Unigene35799\_Sample\_011046840, Unigene38852\_Sample\_011046840, Unigene41952\_Sample\_011046840 |
| 66 | Cytosolic DNA-sensing pathway Back to summary table | Unigene5467\_Sample\_011046840, Unigene37277\_Sample\_011046840, Unigene39662\_Sample\_011046840, Unigene4474\_Sample\_011046840, Unigene4601\_Sample\_011046840, Unigene6641\_Sample\_011046840, Unigene7727\_Sample\_011046840, Unigene34982\_Sample\_011046840, Unigene39418\_Sample\_011046840, Unigene40865\_Sample\_011046840, Unigene41799\_Sample\_011046840, Unigene42104\_Sample\_011046840, Unigene43089\_Sample\_011046840, Unigene43119\_Sample\_011046840, Unigene5724\_Sample\_011046840, Unigene12771\_Sample\_011046840, Unigene36333\_Sample\_011046840, Unigene17567\_Sample\_011046840, Unigene43337\_Sample\_011046840, Unigene17541\_Sample\_011046840, Unigene1151\_Sample\_011046840, Unigene5308\_Sample\_011046840, Unigene12677\_Sample\_011046840, Unigene13775\_Sample\_011046840, Unigene17861\_Sample\_011046840, Unigene23155\_Sample\_011046840, Unigene35859\_Sample\_011046840, Unigene41410\_Sample\_011046840, Unigene39486\_Sample\_011046840, Unigene40053\_Sample\_011046840 |
| 67 | Carbon fixation in photosynthetic organisms Back to summary table | Unigene5738\_Sample\_011046840, Unigene7194\_Sample\_011046840, Unigene32365\_Sample\_011046840, Unigene33042\_Sample\_011046840, Unigene43191\_Sample\_011046840, Unigene979\_Sample\_011046840, Unigene8475\_Sample\_011046840, Unigene13053\_Sample\_011046840, Unigene14254\_Sample\_011046840, Unigene32079\_Sample\_011046840, Unigene4670\_Sample\_011046840, Unigene38096\_Sample\_011046840, Unigene20274\_Sample\_011046840, Unigene36533\_Sample\_011046840, Unigene37309\_Sample\_011046840, Unigene40393\_Sample\_011046840, Unigene40632\_Sample\_011046840, Unigene41283\_Sample\_011046840, Unigene7114\_Sample\_011046840, Unigene23606\_Sample\_011046840, Unigene35603\_Sample\_011046840, Unigene2384\_Sample\_011046840, Unigene41344\_Sample\_011046840, Unigene17098\_Sample\_011046840, Unigene19312\_Sample\_011046840, Unigene29743\_Sample\_011046840, Unigene18224\_Sample\_011046840, Unigene32735\_Sample\_011046840, Unigene37359\_Sample\_011046840, Unigene841\_Sample\_011046840, Unigene31845\_Sample\_011046840, Unigene4352\_Sample\_011046840, Unigene19328\_Sample\_011046840, Unigene3777\_Sample\_011046840, Unigene16784\_Sample\_011046840, Unigene30324\_Sample\_011046840, Unigene33008\_Sample\_011046840 |
| 68 | Lysine degradation Back to summary table | Unigene5394\_Sample\_011046840, Unigene21015\_Sample\_011046840, Unigene21857\_Sample\_011046840, Unigene27750\_Sample\_011046840, Unigene32859\_Sample\_011046840, Unigene38139\_Sample\_011046840, Unigene38335\_Sample\_011046840, Unigene38376\_Sample\_011046840, Unigene40280\_Sample\_011046840, Unigene40638\_Sample\_011046840, Unigene40951\_Sample\_011046840, Unigene42131\_Sample\_011046840, Unigene42250\_Sample\_011046840, Unigene42466\_Sample\_011046840, Unigene43051\_Sample\_011046840, Unigene487\_Sample\_011046840, Unigene2498\_Sample\_011046840, Unigene6075\_Sample\_011046840, Unigene7784\_Sample\_011046840, Unigene7913\_Sample\_011046840, Unigene23826\_Sample\_011046840, Unigene31913\_Sample\_011046840, Unigene35511\_Sample\_011046840, Unigene36028\_Sample\_011046840, Unigene38077\_Sample\_011046840, Unigene38459\_Sample\_011046840, Unigene41096\_Sample\_011046840, Unigene42306\_Sample\_011046840, Unigene42382\_Sample\_011046840, Unigene42637\_Sample\_011046840, Unigene42919\_Sample\_011046840, Unigene43239\_Sample\_011046840, Unigene18600\_Sample\_011046840, Unigene24340\_Sample\_011046840, Unigene41448\_Sample\_011046840, Unigene797\_Sample\_011046840, Unigene4446\_Sample\_011046840, Unigene23987\_Sample\_011046840, Unigene34180\_Sample\_011046840, Unigene36299\_Sample\_011046840, Unigene13731\_Sample\_011046840, Unigene20463\_Sample\_011046840, Unigene22392\_Sample\_011046840, Unigene31928\_Sample\_011046840, Unigene34265\_Sample\_011046840, Unigene35365\_Sample\_011046840, Unigene37589\_Sample\_011046840, Unigene39836\_Sample\_011046840, Unigene43473\_Sample\_011046840, Unigene43586\_Sample\_011046840, Unigene17329\_Sample\_011046840, Unigene24992\_Sample\_011046840, Unigene41037\_Sample\_011046840, Unigene41714\_Sample\_011046840, Unigene41976\_Sample\_011046840, Unigene43291\_Sample\_011046840, Unigene956\_Sample\_011046840, Unigene2538\_Sample\_011046840, Unigene18167\_Sample\_011046840, Unigene32413\_Sample\_011046840, Unigene32790\_Sample\_011046840, Unigene40304\_Sample\_011046840, Unigene40724\_Sample\_011046840, Unigene40961\_Sample\_011046840, Unigene41549\_Sample\_011046840, Unigene12534\_Sample\_011046840, Unigene17816\_Sample\_011046840, Unigene3810\_Sample\_011046840, Unigene13847\_Sample\_011046840, Unigene16153\_Sample\_011046840, Unigene17585\_Sample\_011046840, Unigene31775\_Sample\_011046840, Unigene40136\_Sample\_011046840, Unigene41995\_Sample\_011046840, Unigene20612\_Sample\_011046840, Unigene22945\_Sample\_011046840, Unigene25904\_Sample\_011046840, Unigene30750\_Sample\_011046840, Unigene32057\_Sample\_011046840, Unigene34118\_Sample\_011046840, Unigene40137\_Sample\_011046840, Unigene9531\_Sample\_011046840, Unigene10572\_Sample\_011046840, Unigene13335\_Sample\_011046840, Unigene13709\_Sample\_011046840, Unigene14590\_Sample\_011046840, Unigene17569\_Sample\_011046840, Unigene22482\_Sample\_011046840, Unigene42114\_Sample\_011046840, Unigene11392\_Sample\_011046840, Unigene24318\_Sample\_011046840 |
| 69 | Pancreatic cancer Back to summary table | Unigene15939\_Sample\_011046840, Unigene27834\_Sample\_011046840, Unigene40260\_Sample\_011046840, Unigene40446\_Sample\_011046840, Unigene41702\_Sample\_011046840, Unigene2986\_Sample\_011046840, Unigene8009\_Sample\_011046840, Unigene11592\_Sample\_011046840, Unigene15111\_Sample\_011046840, Unigene24698\_Sample\_011046840, Unigene29711\_Sample\_011046840, Unigene30539\_Sample\_011046840, Unigene33208\_Sample\_011046840, Unigene34974\_Sample\_011046840, Unigene36690\_Sample\_011046840, Unigene38617\_Sample\_011046840, Unigene39282\_Sample\_011046840, Unigene39883\_Sample\_011046840, Unigene40208\_Sample\_011046840, Unigene41584\_Sample\_011046840, Unigene42164\_Sample\_011046840, Unigene32596\_Sample\_011046840, Unigene33835\_Sample\_011046840, Unigene33954\_Sample\_011046840, Unigene33983\_Sample\_011046840, Unigene35961\_Sample\_011046840, Unigene38834\_Sample\_011046840, Unigene38847\_Sample\_011046840, Unigene42204\_Sample\_011046840, Unigene43149\_Sample\_011046840, Unigene6921\_Sample\_011046840, Unigene33984\_Sample\_011046840, Unigene22423\_Sample\_011046840, Unigene24669\_Sample\_011046840, Unigene28708\_Sample\_011046840, Unigene38655\_Sample\_011046840, Unigene43068\_Sample\_011046840, Unigene24869\_Sample\_011046840, Unigene30155\_Sample\_011046840, Unigene31572\_Sample\_011046840, Unigene38245\_Sample\_011046840, Unigene42346\_Sample\_011046840, Unigene4139\_Sample\_011046840, Unigene9938\_Sample\_011046840, Unigene12153\_Sample\_011046840, Unigene16429\_Sample\_011046840, Unigene18933\_Sample\_011046840, Unigene20787\_Sample\_011046840, Unigene26785\_Sample\_011046840, Unigene27378\_Sample\_011046840, Unigene31880\_Sample\_011046840, Unigene35787\_Sample\_011046840, Unigene39503\_Sample\_011046840, Unigene42008\_Sample\_011046840, Unigene43154\_Sample\_011046840, Unigene1368\_Sample\_011046840, Unigene14237\_Sample\_011046840, Unigene20896\_Sample\_011046840, Unigene22073\_Sample\_011046840, Unigene33870\_Sample\_011046840, Unigene42811\_Sample\_011046840, Unigene5726\_Sample\_011046840, Unigene19127\_Sample\_011046840, Unigene21176\_Sample\_011046840, Unigene41104\_Sample\_011046840, Unigene30201\_Sample\_011046840, Unigene35972\_Sample\_011046840, Unigene41704\_Sample\_011046840, Unigene1568\_Sample\_011046840, Unigene14930\_Sample\_011046840, Unigene16035\_Sample\_011046840, Unigene20420\_Sample\_011046840, Unigene26719\_Sample\_011046840, Unigene32265\_Sample\_011046840, Unigene41410\_Sample\_011046840, Unigene19593\_Sample\_011046840, Unigene37186\_Sample\_011046840 |
| 70 | Chagas disease (no map in kegg database) Back to summary table | Unigene4412\_Sample\_011046840, Unigene5662\_Sample\_011046840, Unigene7341\_Sample\_011046840, Unigene8198\_Sample\_011046840, Unigene32488\_Sample\_011046840, Unigene34179\_Sample\_011046840, Unigene40029\_Sample\_011046840, Unigene40446\_Sample\_011046840, Unigene42127\_Sample\_011046840, Unigene42759\_Sample\_011046840, Unigene43441\_Sample\_011046840, Unigene43493\_Sample\_011046840, Unigene2326\_Sample\_011046840, Unigene2986\_Sample\_011046840, Unigene3812\_Sample\_011046840, Unigene4695\_Sample\_011046840, Unigene8114\_Sample\_011046840, Unigene15111\_Sample\_011046840, Unigene17998\_Sample\_011046840, Unigene29711\_Sample\_011046840, Unigene30539\_Sample\_011046840, Unigene34974\_Sample\_011046840, Unigene38617\_Sample\_011046840, Unigene42164\_Sample\_011046840, Unigene42217\_Sample\_011046840, Unigene42573\_Sample\_011046840, Unigene43481\_Sample\_011046840, Unigene43517\_Sample\_011046840, Unigene2080\_Sample\_011046840, Unigene6414\_Sample\_011046840, Unigene29880\_Sample\_011046840, Unigene33954\_Sample\_011046840, Unigene38847\_Sample\_011046840, Unigene43149\_Sample\_011046840, Unigene6921\_Sample\_011046840, Unigene7605\_Sample\_011046840, Unigene33984\_Sample\_011046840, Unigene36467\_Sample\_011046840, Unigene7110\_Sample\_011046840, Unigene39081\_Sample\_011046840, Unigene42685\_Sample\_011046840, Unigene43068\_Sample\_011046840, Unigene43101\_Sample\_011046840, Unigene6793\_Sample\_011046840, Unigene14179\_Sample\_011046840, Unigene22765\_Sample\_011046840, Unigene24869\_Sample\_011046840, Unigene28872\_Sample\_011046840, Unigene33873\_Sample\_011046840, Unigene37128\_Sample\_011046840, Unigene4139\_Sample\_011046840, Unigene18933\_Sample\_011046840, Unigene23590\_Sample\_011046840, Unigene26785\_Sample\_011046840, Unigene35532\_Sample\_011046840, Unigene42008\_Sample\_011046840, Unigene1368\_Sample\_011046840, Unigene14237\_Sample\_011046840, Unigene20896\_Sample\_011046840, Unigene24411\_Sample\_011046840, Unigene29660\_Sample\_011046840, Unigene42811\_Sample\_011046840, Unigene2762\_Sample\_011046840, Unigene5726\_Sample\_011046840, Unigene8614\_Sample\_011046840, Unigene21176\_Sample\_011046840, Unigene3861\_Sample\_011046840, Unigene23373\_Sample\_011046840, Unigene41704\_Sample\_011046840, Unigene1568\_Sample\_011046840, Unigene14438\_Sample\_011046840, Unigene16035\_Sample\_011046840, Unigene20606\_Sample\_011046840, Unigene29621\_Sample\_011046840, Unigene30242\_Sample\_011046840, Unigene32265\_Sample\_011046840, Unigene34487\_Sample\_011046840, Unigene41410\_Sample\_011046840, Unigene19593\_Sample\_011046840, Unigene29269\_Sample\_011046840, Unigene37186\_Sample\_011046840, Unigene40453\_Sample\_011046840 |
| 71 | Fc epsilon RI signaling pathway Back to summary table | Unigene4790\_Sample\_011046840, Unigene8198\_Sample\_011046840, Unigene15939\_Sample\_011046840, Unigene42127\_Sample\_011046840, Unigene42759\_Sample\_011046840, Unigene42766\_Sample\_011046840, Unigene2986\_Sample\_011046840, Unigene3258\_Sample\_011046840, Unigene4033\_Sample\_011046840, Unigene5059\_Sample\_011046840, Unigene7232\_Sample\_011046840, Unigene29711\_Sample\_011046840, Unigene30539\_Sample\_011046840, Unigene34974\_Sample\_011046840, Unigene38617\_Sample\_011046840, Unigene38866\_Sample\_011046840, Unigene39883\_Sample\_011046840, Unigene42164\_Sample\_011046840, Unigene43421\_Sample\_011046840, Unigene14437\_Sample\_011046840, Unigene29880\_Sample\_011046840, Unigene33835\_Sample\_011046840, Unigene34039\_Sample\_011046840, Unigene38847\_Sample\_011046840, Unigene43149\_Sample\_011046840, Unigene6921\_Sample\_011046840, Unigene20325\_Sample\_011046840, Unigene23045\_Sample\_011046840, Unigene28883\_Sample\_011046840, Unigene35150\_Sample\_011046840, Unigene36320\_Sample\_011046840, Unigene42268\_Sample\_011046840, Unigene43068\_Sample\_011046840, Unigene43392\_Sample\_011046840, Unigene24869\_Sample\_011046840, Unigene30141\_Sample\_011046840, Unigene33873\_Sample\_011046840, Unigene4139\_Sample\_011046840, Unigene18933\_Sample\_011046840, Unigene25548\_Sample\_011046840, Unigene26785\_Sample\_011046840, Unigene28751\_Sample\_011046840, Unigene31880\_Sample\_011046840, Unigene35532\_Sample\_011046840, Unigene37830\_Sample\_011046840, Unigene39503\_Sample\_011046840, Unigene39958\_Sample\_011046840, Unigene40189\_Sample\_011046840, Unigene42008\_Sample\_011046840, Unigene43154\_Sample\_011046840, Unigene1368\_Sample\_011046840, Unigene10749\_Sample\_011046840, Unigene14237\_Sample\_011046840, Unigene18009\_Sample\_011046840, Unigene20896\_Sample\_011046840, Unigene26398\_Sample\_011046840, Unigene33870\_Sample\_011046840, Unigene38572\_Sample\_011046840, Unigene42811\_Sample\_011046840, Unigene5726\_Sample\_011046840, Unigene21176\_Sample\_011046840, Unigene27623\_Sample\_011046840, Unigene41560\_Sample\_011046840, Unigene41704\_Sample\_011046840, Unigene1568\_Sample\_011046840, Unigene14930\_Sample\_011046840, Unigene16035\_Sample\_011046840, Unigene29621\_Sample\_011046840, Unigene32011\_Sample\_011046840, Unigene32265\_Sample\_011046840, Unigene41689\_Sample\_011046840, Unigene19593\_Sample\_011046840, Unigene24889\_Sample\_011046840, Unigene33809\_Sample\_011046840, Unigene37186\_Sample\_011046840 |
| 72 | Cell cycle - yeast Back to summary table | Unigene2956\_Sample\_011046840, Unigene3123\_Sample\_011046840, Unigene6976\_Sample\_011046840, Unigene7647\_Sample\_011046840, Unigene8055\_Sample\_011046840, Unigene13399\_Sample\_011046840, Unigene25448\_Sample\_011046840, Unigene25743\_Sample\_011046840, Unigene26863\_Sample\_011046840, Unigene27738\_Sample\_011046840, Unigene28917\_Sample\_011046840, Unigene31074\_Sample\_011046840, Unigene31248\_Sample\_011046840, Unigene32488\_Sample\_011046840, Unigene33337\_Sample\_011046840, Unigene33667\_Sample\_011046840, Unigene35895\_Sample\_011046840, Unigene37234\_Sample\_011046840, Unigene38139\_Sample\_011046840, Unigene39036\_Sample\_011046840, Unigene41423\_Sample\_011046840, Unigene41510\_Sample\_011046840, Unigene41512\_Sample\_011046840, Unigene42144\_Sample\_011046840, Unigene43493\_Sample\_011046840, Unigene2084\_Sample\_011046840, Unigene4695\_Sample\_011046840, Unigene15428\_Sample\_011046840, Unigene15936\_Sample\_011046840, Unigene16328\_Sample\_011046840, Unigene16882\_Sample\_011046840, Unigene18467\_Sample\_011046840, Unigene24973\_Sample\_011046840, Unigene25830\_Sample\_011046840, Unigene26194\_Sample\_011046840, Unigene27185\_Sample\_011046840, Unigene27812\_Sample\_011046840, Unigene30466\_Sample\_011046840, Unigene30691\_Sample\_011046840, Unigene30813\_Sample\_011046840, Unigene31375\_Sample\_011046840, Unigene32520\_Sample\_011046840, Unigene32593\_Sample\_011046840, Unigene32679\_Sample\_011046840, Unigene32791\_Sample\_011046840, Unigene36364\_Sample\_011046840, Unigene36417\_Sample\_011046840, Unigene36846\_Sample\_011046840, Unigene36911\_Sample\_011046840, Unigene37219\_Sample\_011046840, Unigene37890\_Sample\_011046840, Unigene37904\_Sample\_011046840, Unigene38089\_Sample\_011046840, Unigene38463\_Sample\_011046840, Unigene39900\_Sample\_011046840, Unigene39948\_Sample\_011046840, Unigene40424\_Sample\_011046840, Unigene40658\_Sample\_011046840, Unigene40685\_Sample\_011046840, Unigene41401\_Sample\_011046840, Unigene41444\_Sample\_011046840, Unigene41495\_Sample\_011046840, Unigene41621\_Sample\_011046840, Unigene41673\_Sample\_011046840, Unigene41771\_Sample\_011046840, Unigene41862\_Sample\_011046840, Unigene42030\_Sample\_011046840, Unigene42372\_Sample\_011046840, Unigene42510\_Sample\_011046840, Unigene42573\_Sample\_011046840, Unigene43162\_Sample\_011046840, Unigene43273\_Sample\_011046840, Unigene43352\_Sample\_011046840, Unigene43395\_Sample\_011046840, Unigene43397\_Sample\_011046840, Unigene43517\_Sample\_011046840, Unigene2080\_Sample\_011046840, Unigene23252\_Sample\_011046840, Unigene33638\_Sample\_011046840, Unigene36400\_Sample\_011046840, Unigene36688\_Sample\_011046840, Unigene36929\_Sample\_011046840, Unigene38540\_Sample\_011046840, Unigene40601\_Sample\_011046840, Unigene41114\_Sample\_011046840, Unigene42800\_Sample\_011046840, Unigene42866\_Sample\_011046840, Unigene43081\_Sample\_011046840, Unigene188\_Sample\_011046840, Unigene12743\_Sample\_011046840, Unigene17494\_Sample\_011046840, Unigene20800\_Sample\_011046840, Unigene23526\_Sample\_011046840, Unigene24747\_Sample\_011046840, Unigene33396\_Sample\_011046840, Unigene36467\_Sample\_011046840, Unigene39200\_Sample\_011046840, Unigene41403\_Sample\_011046840, Unigene5867\_Sample\_011046840, Unigene7238\_Sample\_011046840, Unigene13449\_Sample\_011046840, Unigene16264\_Sample\_011046840, Unigene16611\_Sample\_011046840, Unigene22321\_Sample\_011046840, Unigene22944\_Sample\_011046840, Unigene23873\_Sample\_011046840, Unigene25328\_Sample\_011046840, Unigene27478\_Sample\_011046840, Unigene27883\_Sample\_011046840, Unigene28259\_Sample\_011046840, Unigene28415\_Sample\_011046840, Unigene30865\_Sample\_011046840, Unigene33950\_Sample\_011046840, Unigene35572\_Sample\_011046840, Unigene36800\_Sample\_011046840, Unigene37121\_Sample\_011046840, Unigene37437\_Sample\_011046840, Unigene38794\_Sample\_011046840, Unigene39846\_Sample\_011046840, Unigene41000\_Sample\_011046840, Unigene41337\_Sample\_011046840, Unigene41383\_Sample\_011046840, Unigene41747\_Sample\_011046840, Unigene42169\_Sample\_011046840, Unigene42384\_Sample\_011046840, Unigene42554\_Sample\_011046840, Unigene42702\_Sample\_011046840, Unigene43281\_Sample\_011046840, Unigene43486\_Sample\_011046840, Unigene43594\_Sample\_011046840, Unigene14685\_Sample\_011046840, Unigene16307\_Sample\_011046840, Unigene16618\_Sample\_011046840, Unigene22765\_Sample\_011046840, Unigene33222\_Sample\_011046840, Unigene34998\_Sample\_011046840, Unigene37919\_Sample\_011046840, Unigene38121\_Sample\_011046840, Unigene38575\_Sample\_011046840, Unigene38939\_Sample\_011046840, Unigene41125\_Sample\_011046840, Unigene115\_Sample\_011046840, Unigene3084\_Sample\_011046840, Unigene11875\_Sample\_011046840, Unigene18768\_Sample\_011046840, Unigene29122\_Sample\_011046840, Unigene29958\_Sample\_011046840, Unigene32620\_Sample\_011046840, Unigene34436\_Sample\_011046840, Unigene35580\_Sample\_011046840, Unigene38902\_Sample\_011046840, Unigene39391\_Sample\_011046840, Unigene39967\_Sample\_011046840, Unigene40083\_Sample\_011046840, Unigene41228\_Sample\_011046840, Unigene42818\_Sample\_011046840, Unigene42927\_Sample\_011046840, Unigene3380\_Sample\_011046840, Unigene5852\_Sample\_011046840, Unigene12320\_Sample\_011046840, Unigene30604\_Sample\_011046840, Unigene35654\_Sample\_011046840, Unigene40665\_Sample\_011046840, Unigene3908\_Sample\_011046840, Unigene4251\_Sample\_011046840, Unigene8801\_Sample\_011046840, Unigene9678\_Sample\_011046840, Unigene23923\_Sample\_011046840, Unigene25413\_Sample\_011046840, Unigene27358\_Sample\_011046840, Unigene31648\_Sample\_011046840, Unigene32355\_Sample\_011046840, Unigene33161\_Sample\_011046840, Unigene36291\_Sample\_011046840, Unigene37155\_Sample\_011046840, Unigene37605\_Sample\_011046840, Unigene39412\_Sample\_011046840, Unigene40038\_Sample\_011046840, Unigene42242\_Sample\_011046840, Unigene42293\_Sample\_011046840, Unigene2031\_Sample\_011046840, Unigene5383\_Sample\_011046840, Unigene15271\_Sample\_011046840, Unigene20410\_Sample\_011046840, Unigene23028\_Sample\_011046840, Unigene24021\_Sample\_011046840, Unigene25871\_Sample\_011046840, Unigene25972\_Sample\_011046840, Unigene26435\_Sample\_011046840, Unigene30829\_Sample\_011046840, Unigene33722\_Sample\_011046840, Unigene34096\_Sample\_011046840, Unigene36385\_Sample\_011046840, Unigene37505\_Sample\_011046840, Unigene40019\_Sample\_011046840, Unigene40216\_Sample\_011046840, Unigene40873\_Sample\_011046840, Unigene41052\_Sample\_011046840, Unigene23373\_Sample\_011046840, Unigene30872\_Sample\_011046840, Unigene41793\_Sample\_011046840, Unigene42173\_Sample\_011046840, Unigene1344\_Sample\_011046840, Unigene10612\_Sample\_011046840, Unigene14154\_Sample\_011046840, Unigene14438\_Sample\_011046840, Unigene16351\_Sample\_011046840, Unigene20430\_Sample\_011046840, Unigene20606\_Sample\_011046840, Unigene22180\_Sample\_011046840, Unigene22727\_Sample\_011046840, Unigene24954\_Sample\_011046840, Unigene30242\_Sample\_011046840, Unigene35200\_Sample\_011046840, Unigene35789\_Sample\_011046840, Unigene36006\_Sample\_011046840, Unigene38023\_Sample\_011046840, Unigene39224\_Sample\_011046840, Unigene40756\_Sample\_011046840, Unigene41006\_Sample\_011046840, Unigene2308\_Sample\_011046840, Unigene8297\_Sample\_011046840, Unigene11742\_Sample\_011046840, Unigene15195\_Sample\_011046840, Unigene26057\_Sample\_011046840, Unigene29269\_Sample\_011046840, Unigene31966\_Sample\_011046840, Unigene36280\_Sample\_011046840, Unigene36506\_Sample\_011046840, Unigene36819\_Sample\_011046840, Unigene38610\_Sample\_011046840 |
| 73 | Melanoma Back to summary table | Unigene2132\_Sample\_011046840, Unigene6209\_Sample\_011046840, Unigene6378\_Sample\_011046840, Unigene15939\_Sample\_011046840, Unigene40260\_Sample\_011046840, Unigene43556\_Sample\_011046840, Unigene2986\_Sample\_011046840, Unigene7529\_Sample\_011046840, Unigene7819\_Sample\_011046840, Unigene8009\_Sample\_011046840, Unigene11592\_Sample\_011046840, Unigene24698\_Sample\_011046840, Unigene33208\_Sample\_011046840, Unigene34974\_Sample\_011046840, Unigene36690\_Sample\_011046840, Unigene38617\_Sample\_011046840, Unigene39237\_Sample\_011046840, Unigene40208\_Sample\_011046840, Unigene41584\_Sample\_011046840, Unigene42164\_Sample\_011046840, Unigene43210\_Sample\_011046840, Unigene43635\_Sample\_011046840, Unigene8904\_Sample\_011046840, Unigene33983\_Sample\_011046840, Unigene38834\_Sample\_011046840, Unigene38847\_Sample\_011046840, Unigene42204\_Sample\_011046840, Unigene43149\_Sample\_011046840, Unigene39778\_Sample\_011046840, Unigene28708\_Sample\_011046840, Unigene43068\_Sample\_011046840, Unigene24869\_Sample\_011046840, Unigene38245\_Sample\_011046840, Unigene4139\_Sample\_011046840, Unigene12153\_Sample\_011046840, Unigene16429\_Sample\_011046840, Unigene18933\_Sample\_011046840, Unigene26785\_Sample\_011046840, Unigene27378\_Sample\_011046840, Unigene31880\_Sample\_011046840, Unigene35787\_Sample\_011046840, Unigene39503\_Sample\_011046840, Unigene42008\_Sample\_011046840, Unigene43154\_Sample\_011046840, Unigene1368\_Sample\_011046840, Unigene20896\_Sample\_011046840, Unigene33870\_Sample\_011046840, Unigene42811\_Sample\_011046840, Unigene5726\_Sample\_011046840, Unigene21176\_Sample\_011046840, Unigene35972\_Sample\_011046840, Unigene41704\_Sample\_011046840, Unigene16035\_Sample\_011046840, Unigene20420\_Sample\_011046840, Unigene32265\_Sample\_011046840 |
| 74 | Tyrosine metabolism Back to summary table | Unigene7194\_Sample\_011046840, Unigene21412\_Sample\_011046840, Unigene36841\_Sample\_011046840, Unigene38468\_Sample\_011046840, Unigene40674\_Sample\_011046840, Unigene42263\_Sample\_011046840, Unigene43193\_Sample\_011046840, Unigene3573\_Sample\_011046840, Unigene3749\_Sample\_011046840, Unigene6996\_Sample\_011046840, Unigene7091\_Sample\_011046840, Unigene8475\_Sample\_011046840, Unigene10034\_Sample\_011046840, Unigene26834\_Sample\_011046840, Unigene39938\_Sample\_011046840, Unigene41275\_Sample\_011046840, Unigene41585\_Sample\_011046840, Unigene42860\_Sample\_011046840, Unigene7264\_Sample\_011046840, Unigene24754\_Sample\_011046840, Unigene34616\_Sample\_011046840, Unigene3335\_Sample\_011046840, Unigene42521\_Sample\_011046840, Unigene3223\_Sample\_011046840, Unigene4758\_Sample\_011046840, Unigene7090\_Sample\_011046840, Unigene7145\_Sample\_011046840, Unigene7461\_Sample\_011046840, Unigene7636\_Sample\_011046840, Unigene13827\_Sample\_011046840, Unigene32463\_Sample\_011046840, Unigene41484\_Sample\_011046840, Unigene7140\_Sample\_011046840, Unigene7250\_Sample\_011046840, Unigene21594\_Sample\_011046840, Unigene13585\_Sample\_011046840, Unigene39613\_Sample\_011046840, Unigene643\_Sample\_011046840, Unigene12234\_Sample\_011046840, Unigene17049\_Sample\_011046840, Unigene24335\_Sample\_011046840, Unigene32735\_Sample\_011046840, Unigene33907\_Sample\_011046840, Unigene36976\_Sample\_011046840, Unigene10471\_Sample\_011046840, Unigene21926\_Sample\_011046840, Unigene38185\_Sample\_011046840, Unigene42713\_Sample\_011046840, Unigene16857\_Sample\_011046840, Unigene18122\_Sample\_011046840, Unigene27136\_Sample\_011046840, Unigene28923\_Sample\_011046840, Unigene34208\_Sample\_011046840, Unigene35036\_Sample\_011046840, Unigene28070\_Sample\_011046840 |
| 75 | Glycosaminoglycan biosynthesis - chondroitin sulfate Back to summary table | Unigene22177\_Sample\_011046840, Unigene39945\_Sample\_011046840, Unigene3990\_Sample\_011046840, Unigene15667\_Sample\_011046840, Unigene19860\_Sample\_011046840, Unigene30989\_Sample\_011046840, Unigene33659\_Sample\_011046840, Unigene10638\_Sample\_011046840, Unigene31891\_Sample\_011046840, Unigene40807\_Sample\_011046840, Unigene36144\_Sample\_011046840, Unigene39416\_Sample\_011046840, Unigene37738\_Sample\_011046840, Unigene1610\_Sample\_011046840, Unigene7785\_Sample\_011046840, Unigene34023\_Sample\_011046840, Unigene35064\_Sample\_011046840, Unigene19649\_Sample\_011046840, Unigene35417\_Sample\_011046840, Unigene1089\_Sample\_011046840, Unigene35531\_Sample\_011046840 |
| 76 | Pyruvate metabolism Back to summary table | Unigene5738\_Sample\_011046840, Unigene33042\_Sample\_011046840, Unigene38139\_Sample\_011046840, Unigene40234\_Sample\_011046840, Unigene42068\_Sample\_011046840, Unigene42278\_Sample\_011046840, Unigene43191\_Sample\_011046840, Unigene2009\_Sample\_011046840, Unigene5057\_Sample\_011046840, Unigene6534\_Sample\_011046840, Unigene15303\_Sample\_011046840, Unigene17744\_Sample\_011046840, Unigene31096\_Sample\_011046840, Unigene32767\_Sample\_011046840, Unigene33542\_Sample\_011046840, Unigene33706\_Sample\_011046840, Unigene38031\_Sample\_011046840, Unigene40899\_Sample\_011046840, Unigene42598\_Sample\_011046840, Unigene42964\_Sample\_011046840, Unigene24816\_Sample\_011046840, Unigene40725\_Sample\_011046840, Unigene40950\_Sample\_011046840, Unigene4446\_Sample\_011046840, Unigene4670\_Sample\_011046840, Unigene5545\_Sample\_011046840, Unigene41917\_Sample\_011046840, Unigene29476\_Sample\_011046840, Unigene37309\_Sample\_011046840, Unigene37311\_Sample\_011046840, Unigene40393\_Sample\_011046840, Unigene41174\_Sample\_011046840, Unigene41225\_Sample\_011046840, Unigene41283\_Sample\_011046840, Unigene42994\_Sample\_011046840, Unigene5509\_Sample\_011046840, Unigene16699\_Sample\_011046840, Unigene30786\_Sample\_011046840, Unigene35603\_Sample\_011046840, Unigene3170\_Sample\_011046840, Unigene6873\_Sample\_011046840, Unigene10696\_Sample\_011046840, Unigene30801\_Sample\_011046840, Unigene41344\_Sample\_011046840, Unigene41540\_Sample\_011046840, Unigene42516\_Sample\_011046840, Unigene23121\_Sample\_011046840, Unigene35683\_Sample\_011046840, Unigene3810\_Sample\_011046840, Unigene14678\_Sample\_011046840, Unigene18224\_Sample\_011046840, Unigene26767\_Sample\_011046840, Unigene37359\_Sample\_011046840, Unigene40235\_Sample\_011046840, Unigene43092\_Sample\_011046840, Unigene841\_Sample\_011046840, Unigene17239\_Sample\_011046840, Unigene31576\_Sample\_011046840, Unigene31845\_Sample\_011046840, Unigene32723\_Sample\_011046840, Unigene40703\_Sample\_011046840, Unigene20757\_Sample\_011046840, Unigene28129\_Sample\_011046840, Unigene23472\_Sample\_011046840, Unigene32307\_Sample\_011046840, Unigene26228\_Sample\_011046840, Unigene33008\_Sample\_011046840, Unigene33711\_Sample\_011046840 |
| 77 | Basal transcription factors Back to summary table | Unigene8142\_Sample\_011046840, Unigene11124\_Sample\_011046840, Unigene23122\_Sample\_011046840, Unigene23636\_Sample\_011046840, Unigene32931\_Sample\_011046840, Unigene38295\_Sample\_011046840, Unigene42216\_Sample\_011046840, Unigene42387\_Sample\_011046840, Unigene42889\_Sample\_011046840, Unigene43610\_Sample\_011046840, Unigene1653\_Sample\_011046840, Unigene5097\_Sample\_011046840, Unigene5353\_Sample\_011046840, Unigene6216\_Sample\_011046840, Unigene6627\_Sample\_011046840, Unigene12416\_Sample\_011046840, Unigene15902\_Sample\_011046840, Unigene19074\_Sample\_011046840, Unigene21660\_Sample\_011046840, Unigene22122\_Sample\_011046840, Unigene26170\_Sample\_011046840, Unigene28979\_Sample\_011046840, Unigene32009\_Sample\_011046840, Unigene32547\_Sample\_011046840, Unigene33708\_Sample\_011046840, Unigene35437\_Sample\_011046840, Unigene35780\_Sample\_011046840, Unigene37637\_Sample\_011046840, Unigene39404\_Sample\_011046840, Unigene40936\_Sample\_011046840, Unigene41958\_Sample\_011046840, Unigene43278\_Sample\_011046840, Unigene6127\_Sample\_011046840, Unigene42762\_Sample\_011046840, Unigene22560\_Sample\_011046840, Unigene27570\_Sample\_011046840, Unigene27784\_Sample\_011046840, Unigene4860\_Sample\_011046840, Unigene5533\_Sample\_011046840, Unigene6254\_Sample\_011046840, Unigene7391\_Sample\_011046840, Unigene11502\_Sample\_011046840, Unigene14623\_Sample\_011046840, Unigene28456\_Sample\_011046840, Unigene35126\_Sample\_011046840, Unigene39263\_Sample\_011046840, Unigene39570\_Sample\_011046840, Unigene41074\_Sample\_011046840, Unigene41812\_Sample\_011046840, Unigene42653\_Sample\_011046840, Unigene42775\_Sample\_011046840, Unigene40661\_Sample\_011046840, Unigene573\_Sample\_011046840, Unigene3914\_Sample\_011046840, Unigene18542\_Sample\_011046840, Unigene32608\_Sample\_011046840, Unigene39402\_Sample\_011046840, Unigene40042\_Sample\_011046840, Unigene11906\_Sample\_011046840, Unigene39774\_Sample\_011046840, Unigene2602\_Sample\_011046840, Unigene26460\_Sample\_011046840, Unigene28273\_Sample\_011046840, Unigene29398\_Sample\_011046840, Unigene29629\_Sample\_011046840, Unigene33196\_Sample\_011046840, Unigene35874\_Sample\_011046840, Unigene39032\_Sample\_011046840, Unigene13863\_Sample\_011046840, Unigene17976\_Sample\_011046840, Unigene22310\_Sample\_011046840, Unigene29679\_Sample\_011046840, Unigene2917\_Sample\_011046840, Unigene23645\_Sample\_011046840, Unigene30963\_Sample\_011046840, Unigene41436\_Sample\_011046840, Unigene13672\_Sample\_011046840, Unigene26110\_Sample\_011046840, Unigene27683\_Sample\_011046840, Unigene32172\_Sample\_011046840, Unigene42230\_Sample\_011046840 |
| 78 | Starch and sucrose metabolism Back to summary table | Unigene1557\_Sample\_011046840, Unigene2087\_Sample\_011046840, Unigene35247\_Sample\_011046840, Unigene39027\_Sample\_011046840, Unigene42492\_Sample\_011046840, Unigene42552\_Sample\_011046840, Unigene2100\_Sample\_011046840, Unigene3386\_Sample\_011046840, Unigene3712\_Sample\_011046840, Unigene3950\_Sample\_011046840, Unigene4573\_Sample\_011046840, Unigene4870\_Sample\_011046840, Unigene6360\_Sample\_011046840, Unigene6689\_Sample\_011046840, Unigene7739\_Sample\_011046840, Unigene26709\_Sample\_011046840, Unigene30383\_Sample\_011046840, Unigene32708\_Sample\_011046840, Unigene32933\_Sample\_011046840, Unigene33287\_Sample\_011046840, Unigene35431\_Sample\_011046840, Unigene36197\_Sample\_011046840, Unigene36398\_Sample\_011046840, Unigene37776\_Sample\_011046840, Unigene38059\_Sample\_011046840, Unigene38452\_Sample\_011046840, Unigene38573\_Sample\_011046840, Unigene38772\_Sample\_011046840, Unigene42651\_Sample\_011046840, Unigene42867\_Sample\_011046840, Unigene43376\_Sample\_011046840, Unigene4283\_Sample\_011046840, Unigene27667\_Sample\_011046840, Unigene43098\_Sample\_011046840, Unigene3477\_Sample\_011046840, Unigene28404\_Sample\_011046840, Unigene35242\_Sample\_011046840, Unigene39953\_Sample\_011046840, Unigene441\_Sample\_011046840, Unigene7943\_Sample\_011046840, Unigene9446\_Sample\_011046840, Unigene15558\_Sample\_011046840, Unigene16468\_Sample\_011046840, Unigene31435\_Sample\_011046840, Unigene32209\_Sample\_011046840, Unigene36718\_Sample\_011046840, Unigene43060\_Sample\_011046840, Unigene3631\_Sample\_011046840, Unigene20745\_Sample\_011046840, Unigene36401\_Sample\_011046840, Unigene40313\_Sample\_011046840, Unigene42139\_Sample\_011046840, Unigene861\_Sample\_011046840, Unigene7938\_Sample\_011046840, Unigene20007\_Sample\_011046840, Unigene31805\_Sample\_011046840, Unigene39079\_Sample\_011046840, Unigene40763\_Sample\_011046840, Unigene41030\_Sample\_011046840, Unigene42326\_Sample\_011046840, Unigene28458\_Sample\_011046840, Unigene32394\_Sample\_011046840, Unigene455\_Sample\_011046840, Unigene2850\_Sample\_011046840, Unigene6867\_Sample\_011046840, Unigene27469\_Sample\_011046840, Unigene29497\_Sample\_011046840, Unigene35342\_Sample\_011046840, Unigene40679\_Sample\_011046840, Unigene43217\_Sample\_011046840, Unigene13577\_Sample\_011046840, Unigene23438\_Sample\_011046840, Unigene28904\_Sample\_011046840, Unigene36138\_Sample\_011046840, Unigene10962\_Sample\_011046840, Unigene11179\_Sample\_011046840, Unigene20602\_Sample\_011046840, Unigene20815\_Sample\_011046840, Unigene31829\_Sample\_011046840, Unigene35772\_Sample\_011046840, Unigene40242\_Sample\_011046840 |
| 79 | Wnt signaling pathway Back to summary table | Unigene880\_Sample\_011046840, Unigene2690\_Sample\_011046840, Unigene4506\_Sample\_011046840, Unigene5429\_Sample\_011046840, Unigene7503\_Sample\_011046840, Unigene25743\_Sample\_011046840, Unigene26863\_Sample\_011046840, Unigene28917\_Sample\_011046840, Unigene30862\_Sample\_011046840, Unigene32488\_Sample\_011046840, Unigene34179\_Sample\_011046840, Unigene35485\_Sample\_011046840, Unigene40446\_Sample\_011046840, Unigene40471\_Sample\_011046840, Unigene40589\_Sample\_011046840, Unigene41164\_Sample\_011046840, Unigene41242\_Sample\_011046840, Unigene41637\_Sample\_011046840, Unigene41702\_Sample\_011046840, Unigene41989\_Sample\_011046840, Unigene43118\_Sample\_011046840, Unigene43211\_Sample\_011046840, Unigene43493\_Sample\_011046840, Unigene3794\_Sample\_011046840, Unigene3812\_Sample\_011046840, Unigene4096\_Sample\_011046840, Unigene4101\_Sample\_011046840, Unigene4241\_Sample\_011046840, Unigene4254\_Sample\_011046840, Unigene4695\_Sample\_011046840, Unigene4779\_Sample\_011046840, Unigene6863\_Sample\_011046840, Unigene7868\_Sample\_011046840, Unigene7893\_Sample\_011046840, Unigene8185\_Sample\_011046840, Unigene15111\_Sample\_011046840, Unigene18537\_Sample\_011046840, Unigene18652\_Sample\_011046840, Unigene22845\_Sample\_011046840, Unigene24651\_Sample\_011046840, Unigene24698\_Sample\_011046840, Unigene25944\_Sample\_011046840, Unigene28459\_Sample\_011046840, Unigene28757\_Sample\_011046840, Unigene29711\_Sample\_011046840, Unigene30539\_Sample\_011046840, Unigene31843\_Sample\_011046840, Unigene32520\_Sample\_011046840, Unigene35780\_Sample\_011046840, Unigene36284\_Sample\_011046840, Unigene36875\_Sample\_011046840, Unigene38435\_Sample\_011046840, Unigene38612\_Sample\_011046840, Unigene39638\_Sample\_011046840, Unigene39883\_Sample\_011046840, Unigene40105\_Sample\_011046840, Unigene40128\_Sample\_011046840, Unigene40388\_Sample\_011046840, Unigene40936\_Sample\_011046840, Unigene41156\_Sample\_011046840, Unigene41388\_Sample\_011046840, Unigene41398\_Sample\_011046840, Unigene41949\_Sample\_011046840, Unigene42065\_Sample\_011046840, Unigene42217\_Sample\_011046840, Unigene43222\_Sample\_011046840, Unigene43301\_Sample\_011046840, Unigene43492\_Sample\_011046840, Unigene43517\_Sample\_011046840, Unigene976\_Sample\_011046840, Unigene2080\_Sample\_011046840, Unigene3608\_Sample\_011046840, Unigene4972\_Sample\_011046840, Unigene5166\_Sample\_011046840, Unigene8166\_Sample\_011046840, Unigene33835\_Sample\_011046840, Unigene33954\_Sample\_011046840, Unigene35961\_Sample\_011046840, Unigene40404\_Sample\_011046840, Unigene42227\_Sample\_011046840, Unigene3161\_Sample\_011046840, Unigene6322\_Sample\_011046840, Unigene6921\_Sample\_011046840, Unigene21306\_Sample\_011046840, Unigene22038\_Sample\_011046840, Unigene27061\_Sample\_011046840, Unigene29542\_Sample\_011046840, Unigene31699\_Sample\_011046840, Unigene33984\_Sample\_011046840, Unigene36063\_Sample\_011046840, Unigene37705\_Sample\_011046840, Unigene38523\_Sample\_011046840, Unigene42798\_Sample\_011046840, Unigene1475\_Sample\_011046840, Unigene4689\_Sample\_011046840, Unigene4807\_Sample\_011046840, Unigene4818\_Sample\_011046840, Unigene7429\_Sample\_011046840, Unigene11174\_Sample\_011046840, Unigene13661\_Sample\_011046840, Unigene14824\_Sample\_011046840, Unigene21554\_Sample\_011046840, Unigene30530\_Sample\_011046840, Unigene31945\_Sample\_011046840, Unigene33288\_Sample\_011046840, Unigene33313\_Sample\_011046840, Unigene33501\_Sample\_011046840, Unigene34871\_Sample\_011046840, Unigene35839\_Sample\_011046840, Unigene35981\_Sample\_011046840, Unigene36860\_Sample\_011046840, Unigene37994\_Sample\_011046840, Unigene38655\_Sample\_011046840, Unigene38674\_Sample\_011046840, Unigene39243\_Sample\_011046840, Unigene39375\_Sample\_011046840, Unigene41303\_Sample\_011046840, Unigene41412\_Sample\_011046840, Unigene42169\_Sample\_011046840, Unigene42240\_Sample\_011046840, Unigene2464\_Sample\_011046840, Unigene2581\_Sample\_011046840, Unigene7626\_Sample\_011046840, Unigene11648\_Sample\_011046840, Unigene14179\_Sample\_011046840, Unigene22765\_Sample\_011046840, Unigene28124\_Sample\_011046840, Unigene28872\_Sample\_011046840, Unigene38301\_Sample\_011046840, Unigene40718\_Sample\_011046840, Unigene9938\_Sample\_011046840, Unigene24624\_Sample\_011046840, Unigene31705\_Sample\_011046840, Unigene32620\_Sample\_011046840, Unigene40648\_Sample\_011046840, Unigene42385\_Sample\_011046840, Unigene42927\_Sample\_011046840, Unigene43154\_Sample\_011046840, Unigene6629\_Sample\_011046840, Unigene15970\_Sample\_011046840, Unigene32732\_Sample\_011046840, Unigene33574\_Sample\_011046840, Unigene4849\_Sample\_011046840, Unigene6944\_Sample\_011046840, Unigene11225\_Sample\_011046840, Unigene14237\_Sample\_011046840, Unigene15053\_Sample\_011046840, Unigene17676\_Sample\_011046840, Unigene19400\_Sample\_011046840, Unigene20679\_Sample\_011046840, Unigene25070\_Sample\_011046840, Unigene25523\_Sample\_011046840, Unigene29526\_Sample\_011046840, Unigene29660\_Sample\_011046840, Unigene30246\_Sample\_011046840, Unigene32479\_Sample\_011046840, Unigene32607\_Sample\_011046840, Unigene35833\_Sample\_011046840, Unigene36452\_Sample\_011046840, Unigene37392\_Sample\_011046840, Unigene38882\_Sample\_011046840, Unigene40539\_Sample\_011046840, Unigene8614\_Sample\_011046840, Unigene14773\_Sample\_011046840, Unigene25531\_Sample\_011046840, Unigene26520\_Sample\_011046840, Unigene42902\_Sample\_011046840, Unigene10037\_Sample\_011046840, Unigene10196\_Sample\_011046840, Unigene11574\_Sample\_011046840, Unigene19741\_Sample\_011046840, Unigene23373\_Sample\_011046840, Unigene30201\_Sample\_011046840, Unigene30685\_Sample\_011046840, Unigene32864\_Sample\_011046840, Unigene33692\_Sample\_011046840, Unigene35972\_Sample\_011046840, Unigene41015\_Sample\_011046840, Unigene1568\_Sample\_011046840, Unigene11201\_Sample\_011046840, Unigene11650\_Sample\_011046840, Unigene14438\_Sample\_011046840, Unigene14930\_Sample\_011046840, Unigene16736\_Sample\_011046840, Unigene19655\_Sample\_011046840, Unigene20606\_Sample\_011046840, Unigene24573\_Sample\_011046840, Unigene26719\_Sample\_011046840, Unigene34487\_Sample\_011046840, Unigene34920\_Sample\_011046840, Unigene36617\_Sample\_011046840, Unigene36659\_Sample\_011046840, Unigene38841\_Sample\_011046840, Unigene42184\_Sample\_011046840, Unigene43308\_Sample\_011046840, Unigene19593\_Sample\_011046840, Unigene24508\_Sample\_011046840, Unigene26491\_Sample\_011046840, Unigene27174\_Sample\_011046840, Unigene29269\_Sample\_011046840, Unigene31562\_Sample\_011046840, Unigene37185\_Sample\_011046840, Unigene37186\_Sample\_011046840, Unigene40227\_Sample\_011046840 |
| 80 | Leukocyte transendothelial migration Back to summary table | Unigene463\_Sample\_011046840, Unigene880\_Sample\_011046840, Unigene6165\_Sample\_011046840, Unigene6378\_Sample\_011046840, Unigene8035\_Sample\_011046840, Unigene8154\_Sample\_011046840, Unigene9987\_Sample\_011046840, Unigene10035\_Sample\_011046840, Unigene25850\_Sample\_011046840, Unigene27840\_Sample\_011046840, Unigene30862\_Sample\_011046840, Unigene37793\_Sample\_011046840, Unigene38706\_Sample\_011046840, Unigene39096\_Sample\_011046840, Unigene40152\_Sample\_011046840, Unigene40280\_Sample\_011046840, Unigene41220\_Sample\_011046840, Unigene42127\_Sample\_011046840, Unigene42639\_Sample\_011046840, Unigene42759\_Sample\_011046840, Unigene43131\_Sample\_011046840, Unigene43180\_Sample\_011046840, Unigene43211\_Sample\_011046840, Unigene43514\_Sample\_011046840, Unigene20\_Sample\_011046840, Unigene3776\_Sample\_011046840, Unigene4033\_Sample\_011046840, Unigene4101\_Sample\_011046840, Unigene6240\_Sample\_011046840, Unigene6863\_Sample\_011046840, Unigene7496\_Sample\_011046840, Unigene7979\_Sample\_011046840, Unigene8185\_Sample\_011046840, Unigene10748\_Sample\_011046840, Unigene16996\_Sample\_011046840, Unigene27291\_Sample\_011046840, Unigene28418\_Sample\_011046840, Unigene29916\_Sample\_011046840, Unigene33370\_Sample\_011046840, Unigene34085\_Sample\_011046840, Unigene34974\_Sample\_011046840, Unigene35657\_Sample\_011046840, Unigene36317\_Sample\_011046840, Unigene38045\_Sample\_011046840, Unigene38612\_Sample\_011046840, Unigene38617\_Sample\_011046840, Unigene39883\_Sample\_011046840, Unigene40388\_Sample\_011046840, Unigene43258\_Sample\_011046840, Unigene43312\_Sample\_011046840, Unigene43492\_Sample\_011046840, Unigene43499\_Sample\_011046840, Unigene2156\_Sample\_011046840, Unigene3608\_Sample\_011046840, Unigene8133\_Sample\_011046840, Unigene25969\_Sample\_011046840, Unigene25979\_Sample\_011046840, Unigene26624\_Sample\_011046840, Unigene33673\_Sample\_011046840, Unigene33835\_Sample\_011046840, Unigene42910\_Sample\_011046840, Unigene43149\_Sample\_011046840, Unigene3161\_Sample\_011046840, Unigene6322\_Sample\_011046840, Unigene7782\_Sample\_011046840, Unigene7958\_Sample\_011046840, Unigene22038\_Sample\_011046840, Unigene27657\_Sample\_011046840, Unigene28604\_Sample\_011046840, Unigene37705\_Sample\_011046840, Unigene42798\_Sample\_011046840, Unigene43466\_Sample\_011046840, Unigene4279\_Sample\_011046840, Unigene4807\_Sample\_011046840, Unigene4818\_Sample\_011046840, Unigene5364\_Sample\_011046840, Unigene7408\_Sample\_011046840, Unigene7678\_Sample\_011046840, Unigene21554\_Sample\_011046840, Unigene25431\_Sample\_011046840, Unigene28883\_Sample\_011046840, Unigene30530\_Sample\_011046840, Unigene33288\_Sample\_011046840, Unigene35150\_Sample\_011046840, Unigene35981\_Sample\_011046840, Unigene38692\_Sample\_011046840, Unigene38694\_Sample\_011046840, Unigene39621\_Sample\_011046840, Unigene40659\_Sample\_011046840, Unigene40784\_Sample\_011046840, Unigene42685\_Sample\_011046840, Unigene43068\_Sample\_011046840, Unigene43392\_Sample\_011046840, Unigene14379\_Sample\_011046840, Unigene21908\_Sample\_011046840, Unigene24869\_Sample\_011046840, Unigene30141\_Sample\_011046840, Unigene33873\_Sample\_011046840, Unigene37128\_Sample\_011046840, Unigene37954\_Sample\_011046840, Unigene38616\_Sample\_011046840, Unigene41881\_Sample\_011046840, Unigene42142\_Sample\_011046840, Unigene42770\_Sample\_011046840, Unigene3385\_Sample\_011046840, Unigene14255\_Sample\_011046840, Unigene18933\_Sample\_011046840, Unigene26785\_Sample\_011046840, Unigene36662\_Sample\_011046840, Unigene40648\_Sample\_011046840, Unigene42008\_Sample\_011046840, Unigene42385\_Sample\_011046840, Unigene43590\_Sample\_011046840, Unigene34490\_Sample\_011046840, Unigene40960\_Sample\_011046840, Unigene4849\_Sample\_011046840, Unigene6846\_Sample\_011046840, Unigene20896\_Sample\_011046840, Unigene24411\_Sample\_011046840, Unigene28829\_Sample\_011046840, Unigene30941\_Sample\_011046840, Unigene38572\_Sample\_011046840, Unigene42811\_Sample\_011046840, Unigene2209\_Sample\_011046840, Unigene2815\_Sample\_011046840, Unigene3219\_Sample\_011046840, Unigene13067\_Sample\_011046840, Unigene19127\_Sample\_011046840, Unigene25531\_Sample\_011046840, Unigene38423\_Sample\_011046840, Unigene39850\_Sample\_011046840, Unigene42902\_Sample\_011046840, Unigene3861\_Sample\_011046840, Unigene12868\_Sample\_011046840, Unigene15237\_Sample\_011046840, Unigene22950\_Sample\_011046840, Unigene31135\_Sample\_011046840, Unigene33692\_Sample\_011046840, Unigene38066\_Sample\_011046840, Unigene41704\_Sample\_011046840, Unigene1256\_Sample\_011046840, Unigene4276\_Sample\_011046840, Unigene11201\_Sample\_011046840, Unigene14930\_Sample\_011046840, Unigene24464\_Sample\_011046840, Unigene25276\_Sample\_011046840, Unigene32265\_Sample\_011046840, Unigene34059\_Sample\_011046840, Unigene41056\_Sample\_011046840, Unigene42184\_Sample\_011046840, Unigene5921\_Sample\_011046840, Unigene37185\_Sample\_011046840 |
| 81 | Valine, leucine and isoleucine biosynthesis Back to summary table | Unigene5121\_Sample\_011046840, Unigene33671\_Sample\_011046840, Unigene36975\_Sample\_011046840, Unigene40966\_Sample\_011046840, Unigene42259\_Sample\_011046840, Unigene42944\_Sample\_011046840, Unigene1222\_Sample\_011046840, Unigene2009\_Sample\_011046840, Unigene4145\_Sample\_011046840, Unigene7203\_Sample\_011046840, Unigene32767\_Sample\_011046840, Unigene33706\_Sample\_011046840, Unigene38031\_Sample\_011046840, Unigene41061\_Sample\_011046840, Unigene41697\_Sample\_011046840, Unigene5545\_Sample\_011046840, Unigene41162\_Sample\_011046840, Unigene41917\_Sample\_011046840, Unigene27982\_Sample\_011046840, Unigene40793\_Sample\_011046840, Unigene7410\_Sample\_011046840, Unigene30786\_Sample\_011046840, Unigene34532\_Sample\_011046840, Unigene5436\_Sample\_011046840, Unigene6873\_Sample\_011046840, Unigene41540\_Sample\_011046840, Unigene23121\_Sample\_011046840, Unigene23889\_Sample\_011046840, Unigene37571\_Sample\_011046840, Unigene25555\_Sample\_011046840, Unigene43450\_Sample\_011046840, Unigene19404\_Sample\_011046840, Unigene38522\_Sample\_011046840, Unigene22688\_Sample\_011046840 |
| 82 | Tropane, piperidine and pyridine alkaloid biosynthesis Back to summary table | Unigene7194\_Sample\_011046840, Unigene800\_Sample\_011046840, Unigene8475\_Sample\_011046840, Unigene27907\_Sample\_011046840, Unigene514\_Sample\_011046840, Unigene5470\_Sample\_011046840, Unigene8173\_Sample\_011046840, Unigene39822\_Sample\_011046840, Unigene40766\_Sample\_011046840, Unigene35332\_Sample\_011046840, Unigene17049\_Sample\_011046840, Unigene32735\_Sample\_011046840, Unigene37206\_Sample\_011046840 |
| 83 | Ascorbate and aldarate metabolism Back to summary table | Unigene3386\_Sample\_011046840, Unigene4870\_Sample\_011046840, Unigene7739\_Sample\_011046840, Unigene4446\_Sample\_011046840, Unigene40313\_Sample\_011046840, Unigene39079\_Sample\_011046840 |
| 84 | Olfactory transduction Back to summary table | Unigene4184\_Sample\_011046840, Unigene8201\_Sample\_011046840, Unigene39065\_Sample\_011046840, Unigene40128\_Sample\_011046840, Unigene40863\_Sample\_011046840, Unigene42065\_Sample\_011046840, Unigene42116\_Sample\_011046840, Unigene5166\_Sample\_011046840, Unigene39054\_Sample\_011046840, Unigene28128\_Sample\_011046840, Unigene31698\_Sample\_011046840, Unigene31945\_Sample\_011046840, Unigene35280\_Sample\_011046840, Unigene36788\_Sample\_011046840, Unigene37034\_Sample\_011046840, Unigene334\_Sample\_011046840, Unigene8164\_Sample\_011046840, Unigene14498\_Sample\_011046840, Unigene37392\_Sample\_011046840, Unigene40035\_Sample\_011046840, Unigene41292\_Sample\_011046840, Unigene670\_Sample\_011046840, Unigene19156\_Sample\_011046840, Unigene39577\_Sample\_011046840, Unigene2711\_Sample\_011046840, Unigene43443\_Sample\_011046840 |
| 85 | Aminoacyl-tRNA biosynthesis Back to summary table | Unigene5121\_Sample\_011046840, Unigene5385\_Sample\_011046840, Unigene7162\_Sample\_011046840, Unigene8045\_Sample\_011046840, Unigene8217\_Sample\_011046840, Unigene12328\_Sample\_011046840, Unigene16976\_Sample\_011046840, Unigene20585\_Sample\_011046840, Unigene28316\_Sample\_011046840, Unigene29953\_Sample\_011046840, Unigene31192\_Sample\_011046840, Unigene33671\_Sample\_011046840, Unigene35118\_Sample\_011046840, Unigene36975\_Sample\_011046840, Unigene38354\_Sample\_011046840, Unigene39509\_Sample\_011046840, Unigene39740\_Sample\_011046840, Unigene39983\_Sample\_011046840, Unigene40966\_Sample\_011046840, Unigene41261\_Sample\_011046840, Unigene41927\_Sample\_011046840, Unigene42585\_Sample\_011046840, Unigene42944\_Sample\_011046840, Unigene43064\_Sample\_011046840, Unigene43348\_Sample\_011046840, Unigene1222\_Sample\_011046840, Unigene1294\_Sample\_011046840, Unigene3127\_Sample\_011046840, Unigene4145\_Sample\_011046840, Unigene7203\_Sample\_011046840, Unigene7468\_Sample\_011046840, Unigene18953\_Sample\_011046840, Unigene21295\_Sample\_011046840, Unigene26846\_Sample\_011046840, Unigene27110\_Sample\_011046840, Unigene29076\_Sample\_011046840, Unigene30713\_Sample\_011046840, Unigene31599\_Sample\_011046840, Unigene33618\_Sample\_011046840, Unigene33705\_Sample\_011046840, Unigene36128\_Sample\_011046840, Unigene37863\_Sample\_011046840, Unigene39628\_Sample\_011046840, Unigene39674\_Sample\_011046840, Unigene40541\_Sample\_011046840, Unigene41061\_Sample\_011046840, Unigene41086\_Sample\_011046840, Unigene41150\_Sample\_011046840, Unigene41561\_Sample\_011046840, Unigene41697\_Sample\_011046840, Unigene41981\_Sample\_011046840, Unigene42508\_Sample\_011046840, Unigene42942\_Sample\_011046840, Unigene43611\_Sample\_011046840, Unigene12655\_Sample\_011046840, Unigene24255\_Sample\_011046840, Unigene30367\_Sample\_011046840, Unigene39825\_Sample\_011046840, Unigene40488\_Sample\_011046840, Unigene2519\_Sample\_011046840, Unigene37877\_Sample\_011046840, Unigene38393\_Sample\_011046840, Unigene38542\_Sample\_011046840, Unigene41162\_Sample\_011046840, Unigene2092\_Sample\_011046840, Unigene7962\_Sample\_011046840, Unigene11244\_Sample\_011046840, Unigene16100\_Sample\_011046840, Unigene16473\_Sample\_011046840, Unigene20526\_Sample\_011046840, Unigene27982\_Sample\_011046840, Unigene29784\_Sample\_011046840, Unigene31759\_Sample\_011046840, Unigene35581\_Sample\_011046840, Unigene36810\_Sample\_011046840, Unigene39080\_Sample\_011046840, Unigene40793\_Sample\_011046840, Unigene40992\_Sample\_011046840, Unigene41676\_Sample\_011046840, Unigene5541\_Sample\_011046840, Unigene7410\_Sample\_011046840, Unigene10176\_Sample\_011046840, Unigene41935\_Sample\_011046840, Unigene815\_Sample\_011046840, Unigene2151\_Sample\_011046840, Unigene5436\_Sample\_011046840, Unigene10729\_Sample\_011046840, Unigene20828\_Sample\_011046840, Unigene35962\_Sample\_011046840, Unigene23889\_Sample\_011046840, Unigene32292\_Sample\_011046840, Unigene37571\_Sample\_011046840, Unigene3226\_Sample\_011046840, Unigene10957\_Sample\_011046840, Unigene17216\_Sample\_011046840, Unigene18262\_Sample\_011046840, Unigene25555\_Sample\_011046840, Unigene26858\_Sample\_011046840, Unigene32961\_Sample\_011046840, Unigene33112\_Sample\_011046840, Unigene33259\_Sample\_011046840, Unigene43450\_Sample\_011046840, Unigene15199\_Sample\_011046840, Unigene19404\_Sample\_011046840, Unigene20968\_Sample\_011046840, Unigene29049\_Sample\_011046840, Unigene34053\_Sample\_011046840, Unigene36990\_Sample\_011046840, Unigene38522\_Sample\_011046840, Unigene41616\_Sample\_011046840, Unigene43186\_Sample\_011046840, Unigene43422\_Sample\_011046840, Unigene3770\_Sample\_011046840, Unigene10288\_Sample\_011046840, Unigene36971\_Sample\_011046840, Unigene13726\_Sample\_011046840, Unigene15184\_Sample\_011046840, Unigene22393\_Sample\_011046840, Unigene22688\_Sample\_011046840, Unigene26734\_Sample\_011046840, Unigene34992\_Sample\_011046840, Unigene35270\_Sample\_011046840, Unigene36926\_Sample\_011046840, Unigene38462\_Sample\_011046840, Unigene39156\_Sample\_011046840, Unigene39555\_Sample\_011046840, Unigene42596\_Sample\_011046840, Unigene21226\_Sample\_011046840, Unigene29427\_Sample\_011046840 |
| 86 | Glycolysis / Gluconeogenesis Back to summary table | Unigene5466\_Sample\_011046840, Unigene12797\_Sample\_011046840, Unigene26994\_Sample\_011046840, Unigene35056\_Sample\_011046840, Unigene42068\_Sample\_011046840, Unigene2009\_Sample\_011046840, Unigene2100\_Sample\_011046840, Unigene5057\_Sample\_011046840, Unigene6925\_Sample\_011046840, Unigene13253\_Sample\_011046840, Unigene15303\_Sample\_011046840, Unigene17744\_Sample\_011046840, Unigene25182\_Sample\_011046840, Unigene31096\_Sample\_011046840, Unigene32767\_Sample\_011046840, Unigene33706\_Sample\_011046840, Unigene38031\_Sample\_011046840, Unigene38452\_Sample\_011046840, Unigene38772\_Sample\_011046840, Unigene40602\_Sample\_011046840, Unigene43058\_Sample\_011046840, Unigene43376\_Sample\_011046840, Unigene17179\_Sample\_011046840, Unigene24816\_Sample\_011046840, Unigene27667\_Sample\_011046840, Unigene40725\_Sample\_011046840, Unigene4446\_Sample\_011046840, Unigene5545\_Sample\_011046840, Unigene15651\_Sample\_011046840, Unigene28404\_Sample\_011046840, Unigene35242\_Sample\_011046840, Unigene38096\_Sample\_011046840, Unigene41917\_Sample\_011046840, Unigene42253\_Sample\_011046840, Unigene9446\_Sample\_011046840, Unigene9628\_Sample\_011046840, Unigene16468\_Sample\_011046840, Unigene20274\_Sample\_011046840, Unigene29476\_Sample\_011046840, Unigene30627\_Sample\_011046840, Unigene36578\_Sample\_011046840, Unigene41225\_Sample\_011046840, Unigene42835\_Sample\_011046840, Unigene43606\_Sample\_011046840, Unigene5490\_Sample\_011046840, Unigene5509\_Sample\_011046840, Unigene7114\_Sample\_011046840, Unigene20745\_Sample\_011046840, Unigene30786\_Sample\_011046840, Unigene35603\_Sample\_011046840, Unigene2384\_Sample\_011046840, Unigene3170\_Sample\_011046840, Unigene6873\_Sample\_011046840, Unigene7938\_Sample\_011046840, Unigene10696\_Sample\_011046840, Unigene31805\_Sample\_011046840, Unigene39042\_Sample\_011046840, Unigene41344\_Sample\_011046840, Unigene41540\_Sample\_011046840, Unigene12699\_Sample\_011046840, Unigene23121\_Sample\_011046840, Unigene28458\_Sample\_011046840, Unigene30648\_Sample\_011046840, Unigene35683\_Sample\_011046840, Unigene36699\_Sample\_011046840, Unigene1608\_Sample\_011046840, Unigene14678\_Sample\_011046840, Unigene18224\_Sample\_011046840, Unigene40679\_Sample\_011046840, Unigene43092\_Sample\_011046840, Unigene9555\_Sample\_011046840, Unigene17239\_Sample\_011046840, Unigene31576\_Sample\_011046840, Unigene31845\_Sample\_011046840, Unigene33267\_Sample\_011046840, Unigene36493\_Sample\_011046840, Unigene42713\_Sample\_011046840, Unigene43217\_Sample\_011046840, Unigene13577\_Sample\_011046840, Unigene28129\_Sample\_011046840, Unigene4352\_Sample\_011046840, Unigene16855\_Sample\_011046840, Unigene23472\_Sample\_011046840, Unigene28904\_Sample\_011046840, Unigene34208\_Sample\_011046840, Unigene35036\_Sample\_011046840, Unigene11179\_Sample\_011046840, Unigene16784\_Sample\_011046840, Unigene26228\_Sample\_011046840, Unigene40242\_Sample\_011046840 |
| 87 | MAPK signaling pathway - fly Back to summary table | Unigene4790\_Sample\_011046840, Unigene6665\_Sample\_011046840, Unigene7767\_Sample\_011046840, Unigene15939\_Sample\_011046840, Unigene39096\_Sample\_011046840, Unigene40596\_Sample\_011046840, Unigene43319\_Sample\_011046840, Unigene2986\_Sample\_011046840, Unigene10748\_Sample\_011046840, Unigene11592\_Sample\_011046840, Unigene11799\_Sample\_011046840, Unigene32116\_Sample\_011046840, Unigene33208\_Sample\_011046840, Unigene34085\_Sample\_011046840, Unigene36690\_Sample\_011046840, Unigene38866\_Sample\_011046840, Unigene39437\_Sample\_011046840, Unigene40208\_Sample\_011046840, Unigene41584\_Sample\_011046840, Unigene42864\_Sample\_011046840, Unigene29839\_Sample\_011046840, Unigene33983\_Sample\_011046840, Unigene38834\_Sample\_011046840, Unigene42204\_Sample\_011046840, Unigene6322\_Sample\_011046840, Unigene28852\_Sample\_011046840, Unigene7408\_Sample\_011046840, Unigene20325\_Sample\_011046840, Unigene23045\_Sample\_011046840, Unigene36320\_Sample\_011046840, Unigene40814\_Sample\_011046840, Unigene14393\_Sample\_011046840, Unigene21908\_Sample\_011046840, Unigene12153\_Sample\_011046840, Unigene14255\_Sample\_011046840, Unigene27378\_Sample\_011046840, Unigene28751\_Sample\_011046840, Unigene31880\_Sample\_011046840, Unigene35787\_Sample\_011046840, Unigene39503\_Sample\_011046840, Unigene40189\_Sample\_011046840, Unigene13276\_Sample\_011046840, Unigene17985\_Sample\_011046840, Unigene33870\_Sample\_011046840, Unigene35252\_Sample\_011046840, Unigene2209\_Sample\_011046840, Unigene13067\_Sample\_011046840, Unigene27623\_Sample\_011046840, Unigene38122\_Sample\_011046840, Unigene41560\_Sample\_011046840, Unigene18921\_Sample\_011046840 |
| 88 | Fatty acid biosynthesis Back to summary table | Unigene5557\_Sample\_011046840, Unigene8062\_Sample\_011046840, Unigene37951\_Sample\_011046840, Unigene39527\_Sample\_011046840, Unigene40234\_Sample\_011046840, Unigene42278\_Sample\_011046840, Unigene5774\_Sample\_011046840, Unigene24934\_Sample\_011046840, Unigene38880\_Sample\_011046840, Unigene40899\_Sample\_011046840, Unigene40950\_Sample\_011046840, Unigene34516\_Sample\_011046840, Unigene40478\_Sample\_011046840, Unigene41174\_Sample\_011046840, Unigene42086\_Sample\_011046840, Unigene14787\_Sample\_011046840, Unigene25342\_Sample\_011046840, Unigene26283\_Sample\_011046840, Unigene26767\_Sample\_011046840, Unigene40235\_Sample\_011046840, Unigene40703\_Sample\_011046840, Unigene20757\_Sample\_011046840, Unigene32307\_Sample\_011046840, Unigene42891\_Sample\_011046840, Unigene33711\_Sample\_011046840 |
| 89 | Phototransduction (no map in kegg database) Back to summary table | Unigene39057\_Sample\_011046840, Unigene4017\_Sample\_011046840, Unigene34108\_Sample\_011046840, Unigene36905\_Sample\_011046840, Unigene4520\_Sample\_011046840, Unigene14753\_Sample\_011046840, Unigene23006\_Sample\_011046840, Unigene39054\_Sample\_011046840, Unigene31703\_Sample\_011046840, Unigene35922\_Sample\_011046840, Unigene36788\_Sample\_011046840, Unigene41060\_Sample\_011046840, Unigene334\_Sample\_011046840, Unigene8164\_Sample\_011046840, Unigene23643\_Sample\_011046840, Unigene26552\_Sample\_011046840, Unigene31301\_Sample\_011046840, Unigene670\_Sample\_011046840, Unigene1614\_Sample\_011046840, Unigene18624\_Sample\_011046840, Unigene37246\_Sample\_011046840, Unigene39577\_Sample\_011046840, Unigene2711\_Sample\_011046840, Unigene18949\_Sample\_011046840, Unigene43443\_Sample\_011046840 |
| 90 | Meiosis - yeast Back to summary table | Unigene1129\_Sample\_011046840, Unigene2865\_Sample\_011046840, Unigene3123\_Sample\_011046840, Unigene6644\_Sample\_011046840, Unigene8140\_Sample\_011046840, Unigene13399\_Sample\_011046840, Unigene25448\_Sample\_011046840, Unigene27738\_Sample\_011046840, Unigene31074\_Sample\_011046840, Unigene31248\_Sample\_011046840, Unigene32488\_Sample\_011046840, Unigene33337\_Sample\_011046840, Unigene35895\_Sample\_011046840, Unigene40589\_Sample\_011046840, Unigene41510\_Sample\_011046840, Unigene43493\_Sample\_011046840, Unigene3474\_Sample\_011046840, Unigene4459\_Sample\_011046840, Unigene4477\_Sample\_011046840, Unigene4695\_Sample\_011046840, Unigene6139\_Sample\_011046840, Unigene8006\_Sample\_011046840, Unigene16328\_Sample\_011046840, Unigene18467\_Sample\_011046840, Unigene25524\_Sample\_011046840, Unigene25830\_Sample\_011046840, Unigene27185\_Sample\_011046840, Unigene30466\_Sample\_011046840, Unigene30691\_Sample\_011046840, Unigene30813\_Sample\_011046840, Unigene32593\_Sample\_011046840, Unigene32791\_Sample\_011046840, Unigene36364\_Sample\_011046840, Unigene36846\_Sample\_011046840, Unigene36911\_Sample\_011046840, Unigene37890\_Sample\_011046840, Unigene37904\_Sample\_011046840, Unigene38463\_Sample\_011046840, Unigene39900\_Sample\_011046840, Unigene39948\_Sample\_011046840, Unigene40128\_Sample\_011046840, Unigene40424\_Sample\_011046840, Unigene40685\_Sample\_011046840, Unigene41388\_Sample\_011046840, Unigene41444\_Sample\_011046840, Unigene41495\_Sample\_011046840, Unigene41550\_Sample\_011046840, Unigene41621\_Sample\_011046840, Unigene41673\_Sample\_011046840, Unigene42065\_Sample\_011046840, Unigene42284\_Sample\_011046840, Unigene42372\_Sample\_011046840, Unigene42872\_Sample\_011046840, Unigene42991\_Sample\_011046840, Unigene43162\_Sample\_011046840, Unigene43213\_Sample\_011046840, Unigene43273\_Sample\_011046840, Unigene43395\_Sample\_011046840, Unigene43397\_Sample\_011046840, Unigene43517\_Sample\_011046840, Unigene2080\_Sample\_011046840, Unigene23252\_Sample\_011046840, Unigene33638\_Sample\_011046840, Unigene36400\_Sample\_011046840, Unigene36688\_Sample\_011046840, Unigene38540\_Sample\_011046840, Unigene41114\_Sample\_011046840, Unigene42866\_Sample\_011046840, Unigene188\_Sample\_011046840, Unigene12743\_Sample\_011046840, Unigene17494\_Sample\_011046840, Unigene24747\_Sample\_011046840, Unigene28116\_Sample\_011046840, Unigene33396\_Sample\_011046840, Unigene39200\_Sample\_011046840, Unigene634\_Sample\_011046840, Unigene3872\_Sample\_011046840, Unigene4610\_Sample\_011046840, Unigene10400\_Sample\_011046840, Unigene13449\_Sample\_011046840, Unigene16611\_Sample\_011046840, Unigene22321\_Sample\_011046840, Unigene22944\_Sample\_011046840, Unigene23873\_Sample\_011046840, Unigene25328\_Sample\_011046840, Unigene27478\_Sample\_011046840, Unigene28259\_Sample\_011046840, Unigene28415\_Sample\_011046840, Unigene30865\_Sample\_011046840, Unigene31945\_Sample\_011046840, Unigene36800\_Sample\_011046840, Unigene37121\_Sample\_011046840, Unigene41000\_Sample\_011046840, Unigene41337\_Sample\_011046840, Unigene41383\_Sample\_011046840, Unigene41747\_Sample\_011046840, Unigene42240\_Sample\_011046840, Unigene43281\_Sample\_011046840, Unigene43486\_Sample\_011046840, Unigene16307\_Sample\_011046840, Unigene16618\_Sample\_011046840, Unigene22765\_Sample\_011046840, Unigene33222\_Sample\_011046840, Unigene34998\_Sample\_011046840, Unigene37919\_Sample\_011046840, Unigene38121\_Sample\_011046840, Unigene38939\_Sample\_011046840, Unigene41125\_Sample\_011046840, Unigene7372\_Sample\_011046840, Unigene11875\_Sample\_011046840, Unigene18768\_Sample\_011046840, Unigene27233\_Sample\_011046840, Unigene29122\_Sample\_011046840, Unigene29958\_Sample\_011046840, Unigene34436\_Sample\_011046840, Unigene38902\_Sample\_011046840, Unigene39391\_Sample\_011046840, Unigene3380\_Sample\_011046840, Unigene12320\_Sample\_011046840, Unigene23714\_Sample\_011046840, Unigene30604\_Sample\_011046840, Unigene40665\_Sample\_011046840, Unigene41909\_Sample\_011046840, Unigene4125\_Sample\_011046840, Unigene8801\_Sample\_011046840, Unigene9678\_Sample\_011046840, Unigene13531\_Sample\_011046840, Unigene25413\_Sample\_011046840, Unigene27358\_Sample\_011046840, Unigene30703\_Sample\_011046840, Unigene31648\_Sample\_011046840, Unigene32355\_Sample\_011046840, Unigene33161\_Sample\_011046840, Unigene36291\_Sample\_011046840, Unigene37605\_Sample\_011046840, Unigene39412\_Sample\_011046840, Unigene40038\_Sample\_011046840, Unigene42242\_Sample\_011046840, Unigene42293\_Sample\_011046840, Unigene5383\_Sample\_011046840, Unigene12229\_Sample\_011046840, Unigene15271\_Sample\_011046840, Unigene24021\_Sample\_011046840, Unigene25871\_Sample\_011046840, Unigene26435\_Sample\_011046840, Unigene26520\_Sample\_011046840, Unigene33722\_Sample\_011046840, Unigene34096\_Sample\_011046840, Unigene36385\_Sample\_011046840, Unigene37505\_Sample\_011046840, Unigene40019\_Sample\_011046840, Unigene40216\_Sample\_011046840, Unigene10196\_Sample\_011046840, Unigene23373\_Sample\_011046840, Unigene42173\_Sample\_011046840, Unigene1344\_Sample\_011046840, Unigene9002\_Sample\_011046840, Unigene10612\_Sample\_011046840, Unigene14154\_Sample\_011046840, Unigene14227\_Sample\_011046840, Unigene14438\_Sample\_011046840, Unigene20430\_Sample\_011046840, Unigene20606\_Sample\_011046840, Unigene22727\_Sample\_011046840, Unigene23019\_Sample\_011046840, Unigene35789\_Sample\_011046840, Unigene38023\_Sample\_011046840, Unigene39224\_Sample\_011046840, Unigene2308\_Sample\_011046840, Unigene11742\_Sample\_011046840, Unigene15195\_Sample\_011046840, Unigene29269\_Sample\_011046840, Unigene31562\_Sample\_011046840, Unigene36506\_Sample\_011046840, Unigene36819\_Sample\_011046840, Unigene38610\_Sample\_011046840, Unigene40227\_Sample\_011046840 |
| 91 | Porphyrin and chlorophyll metabolism Back to summary table | Unigene8217\_Sample\_011046840, Unigene16709\_Sample\_011046840, Unigene41927\_Sample\_011046840, Unigene3386\_Sample\_011046840, Unigene4870\_Sample\_011046840, Unigene16252\_Sample\_011046840, Unigene26709\_Sample\_011046840, Unigene28329\_Sample\_011046840, Unigene30383\_Sample\_011046840, Unigene30713\_Sample\_011046840, Unigene33172\_Sample\_011046840, Unigene35431\_Sample\_011046840, Unigene36128\_Sample\_011046840, Unigene41981\_Sample\_011046840, Unigene8022\_Sample\_011046840, Unigene31278\_Sample\_011046840, Unigene36718\_Sample\_011046840, Unigene42378\_Sample\_011046840, Unigene11867\_Sample\_011046840, Unigene36896\_Sample\_011046840, Unigene40313\_Sample\_011046840, Unigene2151\_Sample\_011046840, Unigene35110\_Sample\_011046840, Unigene26858\_Sample\_011046840, Unigene24901\_Sample\_011046840, Unigene28993\_Sample\_011046840, Unigene43186\_Sample\_011046840, Unigene35223\_Sample\_011046840, Unigene13726\_Sample\_011046840, Unigene1625\_Sample\_011046840, Unigene16873\_Sample\_011046840 |
| 92 | Melanogenesis Back to summary table | Unigene880\_Sample\_011046840, Unigene4412\_Sample\_011046840, Unigene4506\_Sample\_011046840, Unigene5429\_Sample\_011046840, Unigene5662\_Sample\_011046840, Unigene15939\_Sample\_011046840, Unigene30862\_Sample\_011046840, Unigene34179\_Sample\_011046840, Unigene40674\_Sample\_011046840, Unigene41164\_Sample\_011046840, Unigene41242\_Sample\_011046840, Unigene41989\_Sample\_011046840, Unigene43211\_Sample\_011046840, Unigene43439\_Sample\_011046840, Unigene2326\_Sample\_011046840, Unigene2986\_Sample\_011046840, Unigene3749\_Sample\_011046840, Unigene3812\_Sample\_011046840, Unigene4101\_Sample\_011046840, Unigene4779\_Sample\_011046840, Unigene7819\_Sample\_011046840, Unigene7893\_Sample\_011046840, Unigene18652\_Sample\_011046840, Unigene22845\_Sample\_011046840, Unigene26834\_Sample\_011046840, Unigene38612\_Sample\_011046840, Unigene40105\_Sample\_011046840, Unigene40128\_Sample\_011046840, Unigene42065\_Sample\_011046840, Unigene42217\_Sample\_011046840, Unigene43213\_Sample\_011046840, Unigene43222\_Sample\_011046840, Unigene43301\_Sample\_011046840, Unigene43481\_Sample\_011046840, Unigene43492\_Sample\_011046840, Unigene976\_Sample\_011046840, Unigene3608\_Sample\_011046840, Unigene4972\_Sample\_011046840, Unigene5166\_Sample\_011046840, Unigene6414\_Sample\_011046840, Unigene7264\_Sample\_011046840, Unigene3161\_Sample\_011046840, Unigene6322\_Sample\_011046840, Unigene7605\_Sample\_011046840, Unigene29542\_Sample\_011046840, Unigene31699\_Sample\_011046840, Unigene39054\_Sample\_011046840, Unigene39571\_Sample\_011046840, Unigene3872\_Sample\_011046840, Unigene4758\_Sample\_011046840, Unigene4807\_Sample\_011046840, Unigene7110\_Sample\_011046840, Unigene7429\_Sample\_011046840, Unigene7461\_Sample\_011046840, Unigene13827\_Sample\_011046840, Unigene31945\_Sample\_011046840, Unigene33501\_Sample\_011046840, Unigene35839\_Sample\_011046840, Unigene35981\_Sample\_011046840, Unigene36788\_Sample\_011046840, Unigene37994\_Sample\_011046840, Unigene42685\_Sample\_011046840, Unigene334\_Sample\_011046840, Unigene6793\_Sample\_011046840, Unigene14179\_Sample\_011046840, Unigene28872\_Sample\_011046840, Unigene36619\_Sample\_011046840, Unigene37128\_Sample\_011046840, Unigene42693\_Sample\_011046840, Unigene31705\_Sample\_011046840, Unigene31880\_Sample\_011046840, Unigene39503\_Sample\_011046840, Unigene40648\_Sample\_011046840, Unigene42385\_Sample\_011046840, Unigene43154\_Sample\_011046840, Unigene6629\_Sample\_011046840, Unigene13585\_Sample\_011046840, Unigene15970\_Sample\_011046840, Unigene32732\_Sample\_011046840, Unigene4849\_Sample\_011046840, Unigene19400\_Sample\_011046840, Unigene24411\_Sample\_011046840, Unigene25523\_Sample\_011046840, Unigene29526\_Sample\_011046840, Unigene33870\_Sample\_011046840, Unigene35833\_Sample\_011046840, Unigene36976\_Sample\_011046840, Unigene37392\_Sample\_011046840, Unigene40539\_Sample\_011046840, Unigene670\_Sample\_011046840, Unigene8614\_Sample\_011046840, Unigene14773\_Sample\_011046840, Unigene21926\_Sample\_011046840, Unigene39577\_Sample\_011046840, Unigene2711\_Sample\_011046840, Unigene3861\_Sample\_011046840, Unigene10037\_Sample\_011046840, Unigene33692\_Sample\_011046840, Unigene11201\_Sample\_011046840, Unigene34487\_Sample\_011046840, Unigene34920\_Sample\_011046840, Unigene36617\_Sample\_011046840, Unigene43308\_Sample\_011046840, Unigene24508\_Sample\_011046840, Unigene28070\_Sample\_011046840, Unigene40453\_Sample\_011046840, Unigene43443\_Sample\_011046840 |
| 93 | Pathogenic Escherichia coli infection Back to summary table | Unigene2194\_Sample\_011046840, Unigene4666\_Sample\_011046840, Unigene7341\_Sample\_011046840, Unigene9987\_Sample\_011046840, Unigene10232\_Sample\_011046840, Unigene12449\_Sample\_011046840, Unigene25850\_Sample\_011046840, Unigene29549\_Sample\_011046840, Unigene32514\_Sample\_011046840, Unigene40413\_Sample\_011046840, Unigene42639\_Sample\_011046840, Unigene42680\_Sample\_011046840, Unigene42900\_Sample\_011046840, Unigene43211\_Sample\_011046840, Unigene43311\_Sample\_011046840, Unigene43316\_Sample\_011046840, Unigene43514\_Sample\_011046840, Unigene1791\_Sample\_011046840, Unigene3258\_Sample\_011046840, Unigene5441\_Sample\_011046840, Unigene6354\_Sample\_011046840, Unigene6863\_Sample\_011046840, Unigene7349\_Sample\_011046840, Unigene8185\_Sample\_011046840, Unigene12257\_Sample\_011046840, Unigene27902\_Sample\_011046840, Unigene28742\_Sample\_011046840, Unigene31196\_Sample\_011046840, Unigene38045\_Sample\_011046840, Unigene38612\_Sample\_011046840, Unigene40388\_Sample\_011046840, Unigene41501\_Sample\_011046840, Unigene42428\_Sample\_011046840, Unigene43258\_Sample\_011046840, Unigene43312\_Sample\_011046840, Unigene43492\_Sample\_011046840, Unigene1953\_Sample\_011046840, Unigene25969\_Sample\_011046840, Unigene33383\_Sample\_011046840, Unigene40469\_Sample\_011046840, Unigene22038\_Sample\_011046840, Unigene24766\_Sample\_011046840, Unigene27657\_Sample\_011046840, Unigene31211\_Sample\_011046840, Unigene37705\_Sample\_011046840, Unigene42798\_Sample\_011046840, Unigene43466\_Sample\_011046840, Unigene4818\_Sample\_011046840, Unigene4862\_Sample\_011046840, Unigene5364\_Sample\_011046840, Unigene6056\_Sample\_011046840, Unigene9804\_Sample\_011046840, Unigene15094\_Sample\_011046840, Unigene18272\_Sample\_011046840, Unigene21554\_Sample\_011046840, Unigene25431\_Sample\_011046840, Unigene25718\_Sample\_011046840, Unigene29294\_Sample\_011046840, Unigene30530\_Sample\_011046840, Unigene33288\_Sample\_011046840, Unigene35981\_Sample\_011046840, Unigene36734\_Sample\_011046840, Unigene42871\_Sample\_011046840, Unigene8359\_Sample\_011046840, Unigene8592\_Sample\_011046840, Unigene21865\_Sample\_011046840, Unigene23967\_Sample\_011046840, Unigene26420\_Sample\_011046840, Unigene31181\_Sample\_011046840, Unigene34563\_Sample\_011046840, Unigene38616\_Sample\_011046840, Unigene41881\_Sample\_011046840, Unigene200\_Sample\_011046840, Unigene956\_Sample\_011046840, Unigene30144\_Sample\_011046840, Unigene30618\_Sample\_011046840, Unigene36897\_Sample\_011046840, Unigene36992\_Sample\_011046840, Unigene38641\_Sample\_011046840, Unigene40256\_Sample\_011046840, Unigene6846\_Sample\_011046840, Unigene24972\_Sample\_011046840, Unigene35082\_Sample\_011046840, Unigene38093\_Sample\_011046840, Unigene2815\_Sample\_011046840, Unigene3219\_Sample\_011046840, Unigene7329\_Sample\_011046840, Unigene13487\_Sample\_011046840, Unigene15260\_Sample\_011046840, Unigene19127\_Sample\_011046840, Unigene25531\_Sample\_011046840, Unigene38423\_Sample\_011046840, Unigene42902\_Sample\_011046840, Unigene12163\_Sample\_011046840, Unigene12868\_Sample\_011046840, Unigene15237\_Sample\_011046840, Unigene31135\_Sample\_011046840, Unigene33307\_Sample\_011046840, Unigene33796\_Sample\_011046840, Unigene12584\_Sample\_011046840, Unigene17472\_Sample\_011046840, Unigene17677\_Sample\_011046840, Unigene34059\_Sample\_011046840, Unigene42184\_Sample\_011046840, Unigene5921\_Sample\_011046840, Unigene35799\_Sample\_011046840, Unigene37185\_Sample\_011046840 |
| 94 | Bladder cancer Back to summary table | Unigene463\_Sample\_011046840, Unigene15939\_Sample\_011046840, Unigene21166\_Sample\_011046840, Unigene35439\_Sample\_011046840, Unigene40260\_Sample\_011046840, Unigene41355\_Sample\_011046840, Unigene43445\_Sample\_011046840, Unigene43623\_Sample\_011046840, Unigene2986\_Sample\_011046840, Unigene7529\_Sample\_011046840, Unigene8009\_Sample\_011046840, Unigene8138\_Sample\_011046840, Unigene11592\_Sample\_011046840, Unigene24698\_Sample\_011046840, Unigene33208\_Sample\_011046840, Unigene36690\_Sample\_011046840, Unigene40208\_Sample\_011046840, Unigene41584\_Sample\_011046840, Unigene43210\_Sample\_011046840, Unigene33983\_Sample\_011046840, Unigene38834\_Sample\_011046840, Unigene42204\_Sample\_011046840, Unigene37792\_Sample\_011046840, Unigene28708\_Sample\_011046840, Unigene38694\_Sample\_011046840, Unigene42298\_Sample\_011046840, Unigene38245\_Sample\_011046840, Unigene12153\_Sample\_011046840, Unigene16429\_Sample\_011046840, Unigene27378\_Sample\_011046840, Unigene31880\_Sample\_011046840, Unigene35787\_Sample\_011046840, Unigene39503\_Sample\_011046840, Unigene43154\_Sample\_011046840, Unigene33870\_Sample\_011046840, Unigene35972\_Sample\_011046840, Unigene20420\_Sample\_011046840 |
| 95 | Mismatch repair Back to summary table | Unigene368\_Sample\_011046840, Unigene38306\_Sample\_011046840, Unigene41302\_Sample\_011046840, Unigene43375\_Sample\_011046840, Unigene5197\_Sample\_011046840, Unigene23759\_Sample\_011046840, Unigene30370\_Sample\_011046840, Unigene36032\_Sample\_011046840, Unigene38534\_Sample\_011046840, Unigene40625\_Sample\_011046840, Unigene41709\_Sample\_011046840, Unigene42075\_Sample\_011046840, Unigene42138\_Sample\_011046840, Unigene43502\_Sample\_011046840, Unigene40065\_Sample\_011046840, Unigene30599\_Sample\_011046840, Unigene3915\_Sample\_011046840, Unigene13502\_Sample\_011046840, Unigene19401\_Sample\_011046840, Unigene21136\_Sample\_011046840, Unigene27604\_Sample\_011046840, Unigene31960\_Sample\_011046840, Unigene33322\_Sample\_011046840, Unigene35699\_Sample\_011046840, Unigene36117\_Sample\_011046840, Unigene39649\_Sample\_011046840, Unigene24187\_Sample\_011046840, Unigene27672\_Sample\_011046840, Unigene28265\_Sample\_011046840, Unigene28631\_Sample\_011046840, Unigene29016\_Sample\_011046840, Unigene38136\_Sample\_011046840, Unigene13197\_Sample\_011046840, Unigene19866\_Sample\_011046840, Unigene32099\_Sample\_011046840, Unigene36303\_Sample\_011046840, Unigene37702\_Sample\_011046840, Unigene4108\_Sample\_011046840, Unigene7488\_Sample\_011046840, Unigene18212\_Sample\_011046840, Unigene21696\_Sample\_011046840, Unigene26800\_Sample\_011046840, Unigene31539\_Sample\_011046840, Unigene31638\_Sample\_011046840, Unigene43374\_Sample\_011046840, Unigene7669\_Sample\_011046840, Unigene41334\_Sample\_011046840, Unigene16944\_Sample\_011046840, Unigene19481\_Sample\_011046840, Unigene21415\_Sample\_011046840, Unigene24019\_Sample\_011046840, Unigene30448\_Sample\_011046840, Unigene30760\_Sample\_011046840, Unigene34691\_Sample\_011046840, Unigene36764\_Sample\_011046840, Unigene10159\_Sample\_011046840 |
| 96 | 2,4-Dichlorobenzoate degradation Back to summary table | Unigene800\_Sample\_011046840, Unigene514\_Sample\_011046840, Unigene5470\_Sample\_011046840, Unigene39822\_Sample\_011046840, Unigene37206\_Sample\_011046840 |
| 97 | Biosynthesis of vancomycin group antibiotics Back to summary table | Unigene7613\_Sample\_011046840, Unigene38428\_Sample\_011046840, Unigene6867\_Sample\_011046840, Unigene27469\_Sample\_011046840, Unigene28317\_Sample\_011046840 |
| 98 | Viral myocarditis Back to summary table | Unigene4515\_Sample\_011046840, Unigene4981\_Sample\_011046840, Unigene41600\_Sample\_011046840, Unigene41683\_Sample\_011046840, Unigene42144\_Sample\_011046840, Unigene43073\_Sample\_011046840, Unigene43188\_Sample\_011046840, Unigene43216\_Sample\_011046840, Unigene43311\_Sample\_011046840, Unigene43345\_Sample\_011046840, Unigene3205\_Sample\_011046840, Unigene3258\_Sample\_011046840, Unigene3454\_Sample\_011046840, Unigene3776\_Sample\_011046840, Unigene7349\_Sample\_011046840, Unigene7382\_Sample\_011046840, Unigene8012\_Sample\_011046840, Unigene8145\_Sample\_011046840, Unigene8185\_Sample\_011046840, Unigene11146\_Sample\_011046840, Unigene12189\_Sample\_011046840, Unigene13250\_Sample\_011046840, Unigene15987\_Sample\_011046840, Unigene17210\_Sample\_011046840, Unigene17280\_Sample\_011046840, Unigene20491\_Sample\_011046840, Unigene24698\_Sample\_011046840, Unigene27254\_Sample\_011046840, Unigene34234\_Sample\_011046840, Unigene36568\_Sample\_011046840, Unigene37526\_Sample\_011046840, Unigene38045\_Sample\_011046840, Unigene38973\_Sample\_011046840, Unigene39883\_Sample\_011046840, Unigene40157\_Sample\_011046840, Unigene41237\_Sample\_011046840, Unigene41501\_Sample\_011046840, Unigene41707\_Sample\_011046840, Unigene41924\_Sample\_011046840, Unigene42336\_Sample\_011046840, Unigene42340\_Sample\_011046840, Unigene42479\_Sample\_011046840, Unigene42704\_Sample\_011046840, Unigene42716\_Sample\_011046840, Unigene42838\_Sample\_011046840, Unigene43044\_Sample\_011046840, Unigene43175\_Sample\_011046840, Unigene43258\_Sample\_011046840, Unigene43312\_Sample\_011046840, Unigene43468\_Sample\_011046840, Unigene43544\_Sample\_011046840, Unigene13248\_Sample\_011046840, Unigene33835\_Sample\_011046840, Unigene37037\_Sample\_011046840, Unigene41589\_Sample\_011046840, Unigene42603\_Sample\_011046840, Unigene14347\_Sample\_011046840, Unigene27657\_Sample\_011046840, Unigene37705\_Sample\_011046840, Unigene38670\_Sample\_011046840, Unigene40565\_Sample\_011046840, Unigene41719\_Sample\_011046840, Unigene1249\_Sample\_011046840, Unigene5364\_Sample\_011046840, Unigene13127\_Sample\_011046840, Unigene25431\_Sample\_011046840, Unigene31889\_Sample\_011046840, Unigene37216\_Sample\_011046840, Unigene37301\_Sample\_011046840, Unigene37336\_Sample\_011046840, Unigene37437\_Sample\_011046840, Unigene38794\_Sample\_011046840, Unigene40888\_Sample\_011046840, Unigene41873\_Sample\_011046840, Unigene42531\_Sample\_011046840, Unigene43224\_Sample\_011046840, Unigene43455\_Sample\_011046840, Unigene3758\_Sample\_011046840, Unigene7822\_Sample\_011046840, Unigene8074\_Sample\_011046840, Unigene22064\_Sample\_011046840, Unigene34520\_Sample\_011046840, Unigene38616\_Sample\_011046840, Unigene38962\_Sample\_011046840, Unigene41881\_Sample\_011046840, Unigene7073\_Sample\_011046840, Unigene20146\_Sample\_011046840, Unigene23363\_Sample\_011046840, Unigene36897\_Sample\_011046840, Unigene6846\_Sample\_011046840, Unigene10832\_Sample\_011046840, Unigene22459\_Sample\_011046840, Unigene30222\_Sample\_011046840, Unigene31140\_Sample\_011046840, Unigene31900\_Sample\_011046840, Unigene40751\_Sample\_011046840, Unigene40944\_Sample\_011046840, Unigene2815\_Sample\_011046840, Unigene3219\_Sample\_011046840, Unigene36060\_Sample\_011046840, Unigene40056\_Sample\_011046840, Unigene42423\_Sample\_011046840, Unigene42902\_Sample\_011046840, Unigene42967\_Sample\_011046840, Unigene5569\_Sample\_011046840, Unigene12868\_Sample\_011046840, Unigene15237\_Sample\_011046840, Unigene16211\_Sample\_011046840, Unigene31135\_Sample\_011046840, Unigene35972\_Sample\_011046840, Unigene14930\_Sample\_011046840, Unigene15851\_Sample\_011046840, Unigene42506\_Sample\_011046840, Unigene5921\_Sample\_011046840, Unigene26023\_Sample\_011046840, Unigene34398\_Sample\_011046840, Unigene38852\_Sample\_011046840, Unigene41952\_Sample\_011046840 |
| 99 | Cyanoamino acid metabolism Back to summary table | Unigene31322\_Sample\_011046840, Unigene36918\_Sample\_011046840, Unigene3288\_Sample\_011046840, Unigene6826\_Sample\_011046840, Unigene38355\_Sample\_011046840, Unigene41828\_Sample\_011046840, Unigene5168\_Sample\_011046840, Unigene26805\_Sample\_011046840, Unigene9937\_Sample\_011046840, Unigene11128\_Sample\_011046840, Unigene30592\_Sample\_011046840 |
| 100 | Naphthalene and anthracene degradation Back to summary table | Unigene5648\_Sample\_011046840, Unigene36841\_Sample\_011046840, Unigene38468\_Sample\_011046840, Unigene10034\_Sample\_011046840, Unigene41275\_Sample\_011046840, Unigene41585\_Sample\_011046840, Unigene24754\_Sample\_011046840, Unigene34616\_Sample\_011046840, Unigene7636\_Sample\_011046840, Unigene41484\_Sample\_011046840, Unigene7140\_Sample\_011046840, Unigene14381\_Sample\_011046840, Unigene27664\_Sample\_011046840, Unigene31775\_Sample\_011046840, Unigene33907\_Sample\_011046840, Unigene28923\_Sample\_011046840, Unigene9792\_Sample\_011046840 |
| 101 | TGF-beta signaling pathway Back to summary table | Unigene25743\_Sample\_011046840, Unigene26863\_Sample\_011046840, Unigene28917\_Sample\_011046840, Unigene32488\_Sample\_011046840, Unigene33631\_Sample\_011046840, Unigene35941\_Sample\_011046840, Unigene38567\_Sample\_011046840, Unigene40446\_Sample\_011046840, Unigene41643\_Sample\_011046840, Unigene41702\_Sample\_011046840, Unigene43493\_Sample\_011046840, Unigene1778\_Sample\_011046840, Unigene2986\_Sample\_011046840, Unigene4172\_Sample\_011046840, Unigene4266\_Sample\_011046840, Unigene4695\_Sample\_011046840, Unigene5746\_Sample\_011046840, Unigene6863\_Sample\_011046840, Unigene7773\_Sample\_011046840, Unigene8185\_Sample\_011046840, Unigene12359\_Sample\_011046840, Unigene15111\_Sample\_011046840, Unigene16630\_Sample\_011046840, Unigene32520\_Sample\_011046840, Unigene33090\_Sample\_011046840, Unigene34268\_Sample\_011046840, Unigene38768\_Sample\_011046840, Unigene40388\_Sample\_011046840, Unigene42460\_Sample\_011046840, Unigene43517\_Sample\_011046840, Unigene976\_Sample\_011046840, Unigene2080\_Sample\_011046840, Unigene22946\_Sample\_011046840, Unigene33954\_Sample\_011046840, Unigene35961\_Sample\_011046840, Unigene2604\_Sample\_011046840, Unigene22038\_Sample\_011046840, Unigene33984\_Sample\_011046840, Unigene37705\_Sample\_011046840, Unigene42798\_Sample\_011046840, Unigene4818\_Sample\_011046840, Unigene8080\_Sample\_011046840, Unigene21554\_Sample\_011046840, Unigene24220\_Sample\_011046840, Unigene26353\_Sample\_011046840, Unigene28596\_Sample\_011046840, Unigene30530\_Sample\_011046840, Unigene32884\_Sample\_011046840, Unigene33288\_Sample\_011046840, Unigene38655\_Sample\_011046840, Unigene39040\_Sample\_011046840, Unigene39274\_Sample\_011046840, Unigene42169\_Sample\_011046840, Unigene11573\_Sample\_011046840, Unigene22410\_Sample\_011046840, Unigene22765\_Sample\_011046840, Unigene38830\_Sample\_011046840, Unigene875\_Sample\_011046840, Unigene9938\_Sample\_011046840, Unigene15135\_Sample\_011046840, Unigene26862\_Sample\_011046840, Unigene32620\_Sample\_011046840, Unigene35931\_Sample\_011046840, Unigene36166\_Sample\_011046840, Unigene42927\_Sample\_011046840, Unigene16402\_Sample\_011046840, Unigene41375\_Sample\_011046840, Unigene22254\_Sample\_011046840, Unigene26464\_Sample\_011046840, Unigene25531\_Sample\_011046840, Unigene39624\_Sample\_011046840, Unigene42902\_Sample\_011046840, Unigene9113\_Sample\_011046840, Unigene23373\_Sample\_011046840, Unigene30201\_Sample\_011046840, Unigene35648\_Sample\_011046840, Unigene12911\_Sample\_011046840, Unigene14438\_Sample\_011046840, Unigene20606\_Sample\_011046840, Unigene21236\_Sample\_011046840, Unigene26719\_Sample\_011046840, Unigene42184\_Sample\_011046840, Unigene29269\_Sample\_011046840, Unigene29848\_Sample\_011046840, Unigene37185\_Sample\_011046840 |
| 102 | Glycosaminoglycan biosynthesis - heparan sulfate Back to summary table | Unigene7352\_Sample\_011046840, Unigene36232\_Sample\_011046840, Unigene39945\_Sample\_011046840, Unigene6203\_Sample\_011046840, Unigene15667\_Sample\_011046840, Unigene33659\_Sample\_011046840, Unigene39168\_Sample\_011046840, Unigene40973\_Sample\_011046840, Unigene10638\_Sample\_011046840, Unigene31891\_Sample\_011046840, Unigene34227\_Sample\_011046840, Unigene36144\_Sample\_011046840, Unigene20131\_Sample\_011046840, Unigene36499\_Sample\_011046840, Unigene35029\_Sample\_011046840, Unigene37738\_Sample\_011046840, Unigene1610\_Sample\_011046840, Unigene7785\_Sample\_011046840, Unigene9747\_Sample\_011046840, Unigene34023\_Sample\_011046840, Unigene35064\_Sample\_011046840, Unigene19649\_Sample\_011046840, Unigene40578\_Sample\_011046840, Unigene43579\_Sample\_011046840, Unigene24103\_Sample\_011046840, Unigene38257\_Sample\_011046840, Unigene34884\_Sample\_011046840, Unigene41265\_Sample\_011046840, Unigene40598\_Sample\_011046840 |
| 103 | Renin-angiotensin system Back to summary table | Unigene2144\_Sample\_011046840, Unigene8107\_Sample\_011046840, Unigene42018\_Sample\_011046840, Unigene8114\_Sample\_011046840, Unigene17998\_Sample\_011046840, Unigene27053\_Sample\_011046840, Unigene27226\_Sample\_011046840, Unigene31345\_Sample\_011046840, Unigene31806\_Sample\_011046840, Unigene33011\_Sample\_011046840, Unigene33184\_Sample\_011046840, Unigene36977\_Sample\_011046840, Unigene40816\_Sample\_011046840, Unigene41254\_Sample\_011046840, Unigene41914\_Sample\_011046840, Unigene241\_Sample\_011046840, Unigene31106\_Sample\_011046840, Unigene33599\_Sample\_011046840, Unigene18633\_Sample\_011046840, Unigene22103\_Sample\_011046840, Unigene30442\_Sample\_011046840, Unigene38196\_Sample\_011046840, Unigene36081\_Sample\_011046840, Unigene40789\_Sample\_011046840, Unigene30260\_Sample\_011046840, Unigene25726\_Sample\_011046840, Unigene28432\_Sample\_011046840, Unigene1194\_Sample\_011046840, Unigene26282\_Sample\_011046840 |
| 104 | Thyroid cancer Back to summary table | Unigene7767\_Sample\_011046840, Unigene8069\_Sample\_011046840, Unigene15939\_Sample\_011046840, Unigene43211\_Sample\_011046840, Unigene2261\_Sample\_011046840, Unigene2986\_Sample\_011046840, Unigene3776\_Sample\_011046840, Unigene4082\_Sample\_011046840, Unigene23143\_Sample\_011046840, Unigene24698\_Sample\_011046840, Unigene32467\_Sample\_011046840, Unigene36347\_Sample\_011046840, Unigene38612\_Sample\_011046840, Unigene40208\_Sample\_011046840, Unigene41584\_Sample\_011046840, Unigene42479\_Sample\_011046840, Unigene42716\_Sample\_011046840, Unigene43492\_Sample\_011046840, Unigene28708\_Sample\_011046840, Unigene35981\_Sample\_011046840, Unigene40888\_Sample\_011046840, Unigene7073\_Sample\_011046840, Unigene7125\_Sample\_011046840, Unigene16429\_Sample\_011046840, Unigene27378\_Sample\_011046840, Unigene31880\_Sample\_011046840, Unigene39503\_Sample\_011046840, Unigene33553\_Sample\_011046840, Unigene25523\_Sample\_011046840, Unigene33870\_Sample\_011046840, Unigene21815\_Sample\_011046840, Unigene33524\_Sample\_011046840, Unigene35972\_Sample\_011046840, Unigene39360\_Sample\_011046840, Unigene20420\_Sample\_011046840 |
| 105 | Ether lipid metabolism Back to summary table | Unigene8120\_Sample\_011046840, Unigene32403\_Sample\_011046840, Unigene42267\_Sample\_011046840, Unigene42936\_Sample\_011046840, Unigene1756\_Sample\_011046840, Unigene5059\_Sample\_011046840, Unigene7232\_Sample\_011046840, Unigene8053\_Sample\_011046840, Unigene36508\_Sample\_011046840, Unigene36947\_Sample\_011046840, Unigene40204\_Sample\_011046840, Unigene41631\_Sample\_011046840, Unigene43421\_Sample\_011046840, Unigene42417\_Sample\_011046840, Unigene42\_Sample\_011046840, Unigene39053\_Sample\_011046840, Unigene40803\_Sample\_011046840, Unigene42268\_Sample\_011046840, Unigene40013\_Sample\_011046840, Unigene41439\_Sample\_011046840, Unigene29397\_Sample\_011046840, Unigene34997\_Sample\_011046840, Unigene42547\_Sample\_011046840, Unigene30105\_Sample\_011046840, Unigene455\_Sample\_011046840, Unigene20078\_Sample\_011046840, Unigene26398\_Sample\_011046840, Unigene34624\_Sample\_011046840, Unigene34694\_Sample\_011046840, Unigene16716\_Sample\_011046840, Unigene38082\_Sample\_011046840, Unigene28369\_Sample\_011046840, Unigene32011\_Sample\_011046840, Unigene41689\_Sample\_011046840, Unigene33809\_Sample\_011046840 |
| 106 | mTOR signaling pathway Back to summary table | Unigene571\_Sample\_011046840, Unigene3238\_Sample\_011046840, Unigene6456\_Sample\_011046840, Unigene7525\_Sample\_011046840, Unigene7744\_Sample\_011046840, Unigene24311\_Sample\_011046840, Unigene26869\_Sample\_011046840, Unigene29452\_Sample\_011046840, Unigene29592\_Sample\_011046840, Unigene32137\_Sample\_011046840, Unigene37968\_Sample\_011046840, Unigene38127\_Sample\_011046840, Unigene543\_Sample\_011046840, Unigene2986\_Sample\_011046840, Unigene4101\_Sample\_011046840, Unigene4403\_Sample\_011046840, Unigene5425\_Sample\_011046840, Unigene5453\_Sample\_011046840, Unigene5958\_Sample\_011046840, Unigene9193\_Sample\_011046840, Unigene19398\_Sample\_011046840, Unigene27207\_Sample\_011046840, Unigene30720\_Sample\_011046840, Unigene31398\_Sample\_011046840, Unigene34658\_Sample\_011046840, Unigene34953\_Sample\_011046840, Unigene34974\_Sample\_011046840, Unigene36797\_Sample\_011046840, Unigene37006\_Sample\_011046840, Unigene37858\_Sample\_011046840, Unigene38617\_Sample\_011046840, Unigene40208\_Sample\_011046840, Unigene40709\_Sample\_011046840, Unigene40832\_Sample\_011046840, Unigene41584\_Sample\_011046840, Unigene42164\_Sample\_011046840, Unigene8178\_Sample\_011046840, Unigene19624\_Sample\_011046840, Unigene38847\_Sample\_011046840, Unigene43149\_Sample\_011046840, Unigene28962\_Sample\_011046840, Unigene14521\_Sample\_011046840, Unigene25309\_Sample\_011046840, Unigene28708\_Sample\_011046840, Unigene30239\_Sample\_011046840, Unigene30502\_Sample\_011046840, Unigene31151\_Sample\_011046840, Unigene37698\_Sample\_011046840, Unigene39346\_Sample\_011046840, Unigene39505\_Sample\_011046840, Unigene41713\_Sample\_011046840, Unigene42509\_Sample\_011046840, Unigene43068\_Sample\_011046840, Unigene16554\_Sample\_011046840, Unigene18168\_Sample\_011046840, Unigene24869\_Sample\_011046840, Unigene30860\_Sample\_011046840, Unigene32548\_Sample\_011046840, Unigene39408\_Sample\_011046840, Unigene4139\_Sample\_011046840, Unigene16429\_Sample\_011046840, Unigene18933\_Sample\_011046840, Unigene26785\_Sample\_011046840, Unigene27378\_Sample\_011046840, Unigene28636\_Sample\_011046840, Unigene42008\_Sample\_011046840, Unigene30578\_Sample\_011046840, Unigene31830\_Sample\_011046840, Unigene33272\_Sample\_011046840, Unigene40315\_Sample\_011046840, Unigene1368\_Sample\_011046840, Unigene20896\_Sample\_011046840, Unigene22254\_Sample\_011046840, Unigene34777\_Sample\_011046840, Unigene42811\_Sample\_011046840, Unigene42968\_Sample\_011046840, Unigene5726\_Sample\_011046840, Unigene21176\_Sample\_011046840, Unigene30501\_Sample\_011046840, Unigene32678\_Sample\_011046840, Unigene41704\_Sample\_011046840, Unigene4005\_Sample\_011046840, Unigene7135\_Sample\_011046840, Unigene9669\_Sample\_011046840, Unigene16035\_Sample\_011046840, Unigene17741\_Sample\_011046840, Unigene20420\_Sample\_011046840, Unigene31471\_Sample\_011046840, Unigene32265\_Sample\_011046840, Unigene33028\_Sample\_011046840, Unigene35068\_Sample\_011046840, Unigene41521\_Sample\_011046840, Unigene11113\_Sample\_011046840, Unigene16037\_Sample\_011046840, Unigene20634\_Sample\_011046840, Unigene33393\_Sample\_011046840 |
| 107 | N-Glycan biosynthesis Back to summary table | Unigene5305\_Sample\_011046840, Unigene5392\_Sample\_011046840, Unigene5650\_Sample\_011046840, Unigene6070\_Sample\_011046840, Unigene25584\_Sample\_011046840, Unigene29264\_Sample\_011046840, Unigene34663\_Sample\_011046840, Unigene36191\_Sample\_011046840, Unigene37772\_Sample\_011046840, Unigene38470\_Sample\_011046840, Unigene38752\_Sample\_011046840, Unigene40090\_Sample\_011046840, Unigene42492\_Sample\_011046840, Unigene43385\_Sample\_011046840, Unigene29877\_Sample\_011046840, Unigene30961\_Sample\_011046840, Unigene31362\_Sample\_011046840, Unigene32997\_Sample\_011046840, Unigene36630\_Sample\_011046840, Unigene39931\_Sample\_011046840, Unigene41823\_Sample\_011046840, Unigene42817\_Sample\_011046840, Unigene42976\_Sample\_011046840, Unigene43454\_Sample\_011046840, Unigene4324\_Sample\_011046840, Unigene7141\_Sample\_011046840, Unigene19832\_Sample\_011046840, Unigene24873\_Sample\_011046840, Unigene32526\_Sample\_011046840, Unigene34282\_Sample\_011046840, Unigene36547\_Sample\_011046840, Unigene39713\_Sample\_011046840, Unigene41965\_Sample\_011046840, Unigene43404\_Sample\_011046840, Unigene2396\_Sample\_011046840, Unigene16941\_Sample\_011046840, Unigene22982\_Sample\_011046840, Unigene29121\_Sample\_011046840, Unigene38495\_Sample\_011046840, Unigene38705\_Sample\_011046840, Unigene39622\_Sample\_011046840, Unigene1454\_Sample\_011046840, Unigene3237\_Sample\_011046840, Unigene6591\_Sample\_011046840, Unigene7652\_Sample\_011046840, Unigene15743\_Sample\_011046840, Unigene17552\_Sample\_011046840, Unigene20494\_Sample\_011046840, Unigene20998\_Sample\_011046840, Unigene21897\_Sample\_011046840, Unigene23401\_Sample\_011046840, Unigene26707\_Sample\_011046840, Unigene27231\_Sample\_011046840, Unigene28282\_Sample\_011046840, Unigene32223\_Sample\_011046840, Unigene33361\_Sample\_011046840, Unigene36107\_Sample\_011046840, Unigene37669\_Sample\_011046840, Unigene37719\_Sample\_011046840, Unigene40158\_Sample\_011046840, Unigene40228\_Sample\_011046840, Unigene43241\_Sample\_011046840, Unigene2963\_Sample\_011046840, Unigene5652\_Sample\_011046840, Unigene34254\_Sample\_011046840, Unigene34435\_Sample\_011046840, Unigene39202\_Sample\_011046840, Unigene40257\_Sample\_011046840, Unigene4387\_Sample\_011046840, Unigene29159\_Sample\_011046840, Unigene30650\_Sample\_011046840, Unigene30684\_Sample\_011046840, Unigene37581\_Sample\_011046840, Unigene39368\_Sample\_011046840, Unigene907\_Sample\_011046840, Unigene11726\_Sample\_011046840, Unigene36981\_Sample\_011046840, Unigene38488\_Sample\_011046840, Unigene779\_Sample\_011046840, Unigene9976\_Sample\_011046840, Unigene35567\_Sample\_011046840, Unigene35609\_Sample\_011046840, Unigene35966\_Sample\_011046840, Unigene37124\_Sample\_011046840, Unigene39751\_Sample\_011046840, Unigene40362\_Sample\_011046840, Unigene40727\_Sample\_011046840, Unigene7838\_Sample\_011046840, Unigene29106\_Sample\_011046840, Unigene30575\_Sample\_011046840, Unigene31270\_Sample\_011046840, Unigene31478\_Sample\_011046840, Unigene31582\_Sample\_011046840, Unigene34281\_Sample\_011046840, Unigene36463\_Sample\_011046840, Unigene36852\_Sample\_011046840, Unigene12570\_Sample\_011046840, Unigene14396\_Sample\_011046840, Unigene23735\_Sample\_011046840, Unigene30051\_Sample\_011046840, Unigene3206\_Sample\_011046840, Unigene19380\_Sample\_011046840, Unigene27376\_Sample\_011046840, Unigene31035\_Sample\_011046840, Unigene31911\_Sample\_011046840, Unigene35607\_Sample\_011046840, Unigene41379\_Sample\_011046840, Unigene42761\_Sample\_011046840 |
| 108 | ECM-receptor interaction Back to summary table | Unigene4837\_Sample\_011046840, Unigene4981\_Sample\_011046840, Unigene8167\_Sample\_011046840, Unigene9987\_Sample\_011046840, Unigene25850\_Sample\_011046840, Unigene27007\_Sample\_011046840, Unigene27844\_Sample\_011046840, Unigene35231\_Sample\_011046840, Unigene37905\_Sample\_011046840, Unigene40296\_Sample\_011046840, Unigene41577\_Sample\_011046840, Unigene41711\_Sample\_011046840, Unigene42639\_Sample\_011046840, Unigene42718\_Sample\_011046840, Unigene43087\_Sample\_011046840, Unigene43429\_Sample\_011046840, Unigene509\_Sample\_011046840, Unigene1705\_Sample\_011046840, Unigene3580\_Sample\_011046840, Unigene4172\_Sample\_011046840, Unigene4266\_Sample\_011046840, Unigene5215\_Sample\_011046840, Unigene6593\_Sample\_011046840, Unigene6698\_Sample\_011046840, Unigene7150\_Sample\_011046840, Unigene7537\_Sample\_011046840, Unigene7773\_Sample\_011046840, Unigene8052\_Sample\_011046840, Unigene25944\_Sample\_011046840, Unigene28956\_Sample\_011046840, Unigene29525\_Sample\_011046840, Unigene33815\_Sample\_011046840, Unigene36347\_Sample\_011046840, Unigene41237\_Sample\_011046840, Unigene41240\_Sample\_011046840, Unigene41700\_Sample\_011046840, Unigene42070\_Sample\_011046840, Unigene42327\_Sample\_011046840, Unigene42336\_Sample\_011046840, Unigene42352\_Sample\_011046840, Unigene43317\_Sample\_011046840, Unigene43362\_Sample\_011046840, Unigene43637\_Sample\_011046840, Unigene30\_Sample\_011046840, Unigene7684\_Sample\_011046840, Unigene8133\_Sample\_011046840, Unigene25969\_Sample\_011046840, Unigene169\_Sample\_011046840, Unigene17237\_Sample\_011046840, Unigene26924\_Sample\_011046840, Unigene36414\_Sample\_011046840, Unigene41734\_Sample\_011046840, Unigene43466\_Sample\_011046840, Unigene1249\_Sample\_011046840, Unigene6909\_Sample\_011046840, Unigene7319\_Sample\_011046840, Unigene7950\_Sample\_011046840, Unigene8080\_Sample\_011046840, Unigene16958\_Sample\_011046840, Unigene27324\_Sample\_011046840, Unigene28530\_Sample\_011046840, Unigene33876\_Sample\_011046840, Unigene39562\_Sample\_011046840, Unigene39625\_Sample\_011046840, Unigene40795\_Sample\_011046840, Unigene41345\_Sample\_011046840, Unigene42120\_Sample\_011046840, Unigene42315\_Sample\_011046840, Unigene43101\_Sample\_011046840, Unigene21144\_Sample\_011046840, Unigene34520\_Sample\_011046840, Unigene38216\_Sample\_011046840, Unigene38830\_Sample\_011046840, Unigene41366\_Sample\_011046840, Unigene42345\_Sample\_011046840, Unigene24271\_Sample\_011046840, Unigene25551\_Sample\_011046840, Unigene41033\_Sample\_011046840, Unigene43006\_Sample\_011046840, Unigene43102\_Sample\_011046840, Unigene1701\_Sample\_011046840, Unigene20803\_Sample\_011046840, Unigene39257\_Sample\_011046840, Unigene42677\_Sample\_011046840, Unigene7256\_Sample\_011046840, Unigene13910\_Sample\_011046840, Unigene24124\_Sample\_011046840, Unigene27056\_Sample\_011046840, Unigene29917\_Sample\_011046840, Unigene35736\_Sample\_011046840, Unigene40855\_Sample\_011046840, Unigene18703\_Sample\_011046840, Unigene23687\_Sample\_011046840, Unigene28036\_Sample\_011046840, Unigene28674\_Sample\_011046840, Unigene36060\_Sample\_011046840, Unigene38423\_Sample\_011046840, Unigene40056\_Sample\_011046840, Unigene42630\_Sample\_011046840, Unigene32870\_Sample\_011046840, Unigene6436\_Sample\_011046840, Unigene25119\_Sample\_011046840, Unigene32848\_Sample\_011046840, Unigene35857\_Sample\_011046840, Unigene37909\_Sample\_011046840, Unigene38979\_Sample\_011046840, Unigene5544\_Sample\_011046840, Unigene29910\_Sample\_011046840 |
| 109 | Thiamine metabolism Back to summary table | Unigene6088\_Sample\_011046840, Unigene7926\_Sample\_011046840, Unigene26088\_Sample\_011046840, Unigene28461\_Sample\_011046840, Unigene38363\_Sample\_011046840, Unigene43417\_Sample\_011046840, Unigene11049\_Sample\_011046840, Unigene26338\_Sample\_011046840, Unigene41224\_Sample\_011046840, Unigene43612\_Sample\_011046840, Unigene40576\_Sample\_011046840, Unigene40593\_Sample\_011046840, Unigene1018\_Sample\_011046840, Unigene2123\_Sample\_011046840, Unigene29739\_Sample\_011046840, Unigene39685\_Sample\_011046840 |
| 110 | Bacterial secretion system Back to summary table | Unigene35913\_Sample\_011046840, Unigene14530\_Sample\_011046840, Unigene20351\_Sample\_011046840, Unigene28104\_Sample\_011046840, Unigene38739\_Sample\_011046840, Unigene11248\_Sample\_011046840, Unigene13746\_Sample\_011046840, Unigene41042\_Sample\_011046840, Unigene35090\_Sample\_011046840, Unigene41077\_Sample\_011046840 |
| 111 | Citrate cycle (TCA cycle) Back to summary table | Unigene33042\_Sample\_011046840, Unigene38764\_Sample\_011046840, Unigene41068\_Sample\_011046840, Unigene42068\_Sample\_011046840, Unigene42131\_Sample\_011046840, Unigene43191\_Sample\_011046840, Unigene2009\_Sample\_011046840, Unigene12353\_Sample\_011046840, Unigene17744\_Sample\_011046840, Unigene32767\_Sample\_011046840, Unigene33706\_Sample\_011046840, Unigene37553\_Sample\_011046840, Unigene38031\_Sample\_011046840, Unigene38464\_Sample\_011046840, Unigene39916\_Sample\_011046840, Unigene3826\_Sample\_011046840, Unigene33539\_Sample\_011046840, Unigene33585\_Sample\_011046840, Unigene35179\_Sample\_011046840, Unigene39708\_Sample\_011046840, Unigene40451\_Sample\_011046840, Unigene40725\_Sample\_011046840, Unigene40731\_Sample\_011046840, Unigene797\_Sample\_011046840, Unigene4670\_Sample\_011046840, Unigene5545\_Sample\_011046840, Unigene37694\_Sample\_011046840, Unigene41917\_Sample\_011046840, Unigene43181\_Sample\_011046840, Unigene9361\_Sample\_011046840, Unigene19944\_Sample\_011046840, Unigene25934\_Sample\_011046840, Unigene29476\_Sample\_011046840, Unigene35743\_Sample\_011046840, Unigene36103\_Sample\_011046840, Unigene36187\_Sample\_011046840, Unigene40629\_Sample\_011046840, Unigene41225\_Sample\_011046840, Unigene30786\_Sample\_011046840, Unigene36462\_Sample\_011046840, Unigene40465\_Sample\_011046840, Unigene3170\_Sample\_011046840, Unigene6873\_Sample\_011046840, Unigene10696\_Sample\_011046840, Unigene13839\_Sample\_011046840, Unigene27895\_Sample\_011046840, Unigene32790\_Sample\_011046840, Unigene33275\_Sample\_011046840, Unigene35324\_Sample\_011046840, Unigene36759\_Sample\_011046840, Unigene37975\_Sample\_011046840, Unigene40724\_Sample\_011046840, Unigene41540\_Sample\_011046840, Unigene17816\_Sample\_011046840, Unigene23121\_Sample\_011046840, Unigene33338\_Sample\_011046840, Unigene35395\_Sample\_011046840, Unigene35683\_Sample\_011046840, Unigene37545\_Sample\_011046840, Unigene40437\_Sample\_011046840, Unigene8865\_Sample\_011046840, Unigene16153\_Sample\_011046840, Unigene31775\_Sample\_011046840, Unigene40136\_Sample\_011046840, Unigene43092\_Sample\_011046840, Unigene13641\_Sample\_011046840, Unigene17239\_Sample\_011046840, Unigene22844\_Sample\_011046840, Unigene26868\_Sample\_011046840, Unigene26967\_Sample\_011046840, Unigene31576\_Sample\_011046840, Unigene8445\_Sample\_011046840, Unigene12191\_Sample\_011046840, Unigene13335\_Sample\_011046840, Unigene19369\_Sample\_011046840, Unigene20154\_Sample\_011046840, Unigene22482\_Sample\_011046840, Unigene27805\_Sample\_011046840, Unigene11392\_Sample\_011046840, Unigene24938\_Sample\_011046840, Unigene26228\_Sample\_011046840, Unigene33008\_Sample\_011046840, Unigene37285\_Sample\_011046840 |
| 112 | Glycine, serine and threonine metabolism Back to summary table | Unigene1737\_Sample\_011046840, Unigene6749\_Sample\_011046840, Unigene31322\_Sample\_011046840, Unigene7894\_Sample\_011046840, Unigene16252\_Sample\_011046840, Unigene20848\_Sample\_011046840, Unigene26208\_Sample\_011046840, Unigene4839\_Sample\_011046840, Unigene40725\_Sample\_011046840, Unigene2180\_Sample\_011046840, Unigene5148\_Sample\_011046840, Unigene7751\_Sample\_011046840, Unigene37614\_Sample\_011046840, Unigene41215\_Sample\_011046840, Unigene37274\_Sample\_011046840, Unigene10696\_Sample\_011046840, Unigene23572\_Sample\_011046840, Unigene32167\_Sample\_011046840, Unigene4538\_Sample\_011046840, Unigene37346\_Sample\_011046840, Unigene13016\_Sample\_011046840, Unigene40663\_Sample\_011046840, Unigene42073\_Sample\_011046840, Unigene17239\_Sample\_011046840, Unigene26178\_Sample\_011046840, Unigene28993\_Sample\_011046840, Unigene31576\_Sample\_011046840, Unigene32748\_Sample\_011046840, Unigene985\_Sample\_011046840, Unigene9937\_Sample\_011046840, Unigene24649\_Sample\_011046840, Unigene11128\_Sample\_011046840, Unigene22543\_Sample\_011046840, Unigene26228\_Sample\_011046840 |
| 113 | Spliceosome Back to summary table | Unigene5058\_Sample\_011046840, Unigene8120\_Sample\_011046840, Unigene8181\_Sample\_011046840, Unigene8555\_Sample\_011046840, Unigene23108\_Sample\_011046840, Unigene24379\_Sample\_011046840, Unigene25925\_Sample\_011046840, Unigene26884\_Sample\_011046840, Unigene27621\_Sample\_011046840, Unigene29985\_Sample\_011046840, Unigene31973\_Sample\_011046840, Unigene33237\_Sample\_011046840, Unigene34927\_Sample\_011046840, Unigene35692\_Sample\_011046840, Unigene36192\_Sample\_011046840, Unigene36505\_Sample\_011046840, Unigene36934\_Sample\_011046840, Unigene37077\_Sample\_011046840, Unigene37867\_Sample\_011046840, Unigene39598\_Sample\_011046840, Unigene40472\_Sample\_011046840, Unigene41440\_Sample\_011046840, Unigene41458\_Sample\_011046840, Unigene41679\_Sample\_011046840, Unigene41930\_Sample\_011046840, Unigene41980\_Sample\_011046840, Unigene42007\_Sample\_011046840, Unigene42533\_Sample\_011046840, Unigene42544\_Sample\_011046840, Unigene42663\_Sample\_011046840, Unigene42936\_Sample\_011046840, Unigene43372\_Sample\_011046840, Unigene43610\_Sample\_011046840, Unigene789\_Sample\_011046840, Unigene2459\_Sample\_011046840, Unigene2805\_Sample\_011046840, Unigene3974\_Sample\_011046840, Unigene3999\_Sample\_011046840, Unigene4561\_Sample\_011046840, Unigene4640\_Sample\_011046840, Unigene6626\_Sample\_011046840, Unigene7127\_Sample\_011046840, Unigene7988\_Sample\_011046840, Unigene9825\_Sample\_011046840, Unigene9927\_Sample\_011046840, Unigene14147\_Sample\_011046840, Unigene14525\_Sample\_011046840, Unigene14705\_Sample\_011046840, Unigene14993\_Sample\_011046840, Unigene15140\_Sample\_011046840, Unigene15931\_Sample\_011046840, Unigene16389\_Sample\_011046840, Unigene17573\_Sample\_011046840, Unigene21223\_Sample\_011046840, Unigene22644\_Sample\_011046840, Unigene23324\_Sample\_011046840, Unigene24612\_Sample\_011046840, Unigene25343\_Sample\_011046840, Unigene25921\_Sample\_011046840, Unigene27458\_Sample\_011046840, Unigene27532\_Sample\_011046840, Unigene27677\_Sample\_011046840, Unigene27777\_Sample\_011046840, Unigene29518\_Sample\_011046840, Unigene30556\_Sample\_011046840, Unigene30708\_Sample\_011046840, Unigene31695\_Sample\_011046840, Unigene32621\_Sample\_011046840, Unigene33982\_Sample\_011046840, Unigene34408\_Sample\_011046840, Unigene34929\_Sample\_011046840, Unigene35835\_Sample\_011046840, Unigene36025\_Sample\_011046840, Unigene36211\_Sample\_011046840, Unigene36773\_Sample\_011046840, Unigene36936\_Sample\_011046840, Unigene37502\_Sample\_011046840, Unigene37674\_Sample\_011046840, Unigene37836\_Sample\_011046840, Unigene37976\_Sample\_011046840, Unigene38227\_Sample\_011046840, Unigene38478\_Sample\_011046840, Unigene38944\_Sample\_011046840, Unigene39147\_Sample\_011046840, Unigene39195\_Sample\_011046840, Unigene39409\_Sample\_011046840, Unigene39583\_Sample\_011046840, Unigene39604\_Sample\_011046840, Unigene39807\_Sample\_011046840, Unigene39979\_Sample\_011046840, Unigene40082\_Sample\_011046840, Unigene40495\_Sample\_011046840, Unigene40505\_Sample\_011046840, Unigene40890\_Sample\_011046840, Unigene40949\_Sample\_011046840, Unigene41005\_Sample\_011046840, Unigene41043\_Sample\_011046840, Unigene41189\_Sample\_011046840, Unigene41563\_Sample\_011046840, Unigene42050\_Sample\_011046840, Unigene42097\_Sample\_011046840, Unigene42154\_Sample\_011046840, Unigene42369\_Sample\_011046840, Unigene42453\_Sample\_011046840, Unigene43043\_Sample\_011046840, Unigene43066\_Sample\_011046840, Unigene43314\_Sample\_011046840, Unigene43444\_Sample\_011046840, Unigene43521\_Sample\_011046840, Unigene43585\_Sample\_011046840, Unigene43591\_Sample\_011046840, Unigene43593\_Sample\_011046840, Unigene5782\_Sample\_011046840, Unigene7740\_Sample\_011046840, Unigene10357\_Sample\_011046840, Unigene18134\_Sample\_011046840, Unigene19402\_Sample\_011046840, Unigene28690\_Sample\_011046840, Unigene30053\_Sample\_011046840, Unigene31513\_Sample\_011046840, Unigene32028\_Sample\_011046840, Unigene34726\_Sample\_011046840, Unigene38104\_Sample\_011046840, Unigene38687\_Sample\_011046840, Unigene39421\_Sample\_011046840, Unigene39633\_Sample\_011046840, Unigene40988\_Sample\_011046840, Unigene41178\_Sample\_011046840, Unigene41336\_Sample\_011046840, Unigene42200\_Sample\_011046840, Unigene42408\_Sample\_011046840, Unigene42412\_Sample\_011046840, Unigene42520\_Sample\_011046840, Unigene42591\_Sample\_011046840, Unigene42764\_Sample\_011046840, Unigene43093\_Sample\_011046840, Unigene4914\_Sample\_011046840, Unigene7018\_Sample\_011046840, Unigene7702\_Sample\_011046840, Unigene25043\_Sample\_011046840, Unigene30617\_Sample\_011046840, Unigene32144\_Sample\_011046840, Unigene34591\_Sample\_011046840, Unigene34760\_Sample\_011046840, Unigene35279\_Sample\_011046840, Unigene35986\_Sample\_011046840, Unigene38115\_Sample\_011046840, Unigene43091\_Sample\_011046840, Unigene43294\_Sample\_011046840, Unigene43335\_Sample\_011046840, Unigene1456\_Sample\_011046840, Unigene4465\_Sample\_011046840, Unigene4815\_Sample\_011046840, Unigene5094\_Sample\_011046840, Unigene5223\_Sample\_011046840, Unigene7044\_Sample\_011046840, Unigene7427\_Sample\_011046840, Unigene8520\_Sample\_011046840, Unigene10054\_Sample\_011046840, Unigene14503\_Sample\_011046840, Unigene16154\_Sample\_011046840, Unigene16493\_Sample\_011046840, Unigene17650\_Sample\_011046840, Unigene17942\_Sample\_011046840, Unigene19239\_Sample\_011046840, Unigene20222\_Sample\_011046840, Unigene20936\_Sample\_011046840, Unigene22269\_Sample\_011046840, Unigene22770\_Sample\_011046840, Unigene25915\_Sample\_011046840, Unigene26219\_Sample\_011046840, Unigene26472\_Sample\_011046840, Unigene26680\_Sample\_011046840, Unigene26865\_Sample\_011046840, Unigene27879\_Sample\_011046840, Unigene28153\_Sample\_011046840, Unigene28315\_Sample\_011046840, Unigene29635\_Sample\_011046840, Unigene29890\_Sample\_011046840, Unigene31408\_Sample\_011046840, Unigene32382\_Sample\_011046840, Unigene32573\_Sample\_011046840, Unigene32899\_Sample\_011046840, Unigene34288\_Sample\_011046840, Unigene34294\_Sample\_011046840, Unigene34533\_Sample\_011046840, Unigene34674\_Sample\_011046840, Unigene35749\_Sample\_011046840, Unigene37781\_Sample\_011046840, Unigene38329\_Sample\_011046840, Unigene38370\_Sample\_011046840, Unigene38526\_Sample\_011046840, Unigene38998\_Sample\_011046840, Unigene39017\_Sample\_011046840, Unigene39546\_Sample\_011046840, Unigene39570\_Sample\_011046840, Unigene39832\_Sample\_011046840, Unigene39970\_Sample\_011046840, Unigene40031\_Sample\_011046840, Unigene40239\_Sample\_011046840, Unigene40494\_Sample\_011046840, Unigene40825\_Sample\_011046840, Unigene41413\_Sample\_011046840, Unigene42034\_Sample\_011046840, Unigene42047\_Sample\_011046840, Unigene42388\_Sample\_011046840, Unigene42551\_Sample\_011046840, Unigene42622\_Sample\_011046840, Unigene42995\_Sample\_011046840, Unigene43251\_Sample\_011046840, Unigene43315\_Sample\_011046840, Unigene43426\_Sample\_011046840, Unigene1995\_Sample\_011046840, Unigene4686\_Sample\_011046840, Unigene5791\_Sample\_011046840, Unigene14430\_Sample\_011046840, Unigene16906\_Sample\_011046840, Unigene17194\_Sample\_011046840, Unigene21071\_Sample\_011046840, Unigene23630\_Sample\_011046840, Unigene23767\_Sample\_011046840, Unigene25873\_Sample\_011046840, Unigene26150\_Sample\_011046840, Unigene30697\_Sample\_011046840, Unigene31777\_Sample\_011046840, Unigene32606\_Sample\_011046840, Unigene32944\_Sample\_011046840, Unigene33136\_Sample\_011046840, Unigene38550\_Sample\_011046840, Unigene38680\_Sample\_011046840, Unigene38904\_Sample\_011046840, Unigene39171\_Sample\_011046840, Unigene40401\_Sample\_011046840, Unigene337\_Sample\_011046840, Unigene956\_Sample\_011046840, Unigene3212\_Sample\_011046840, Unigene4920\_Sample\_011046840, Unigene7507\_Sample\_011046840, Unigene9431\_Sample\_011046840, Unigene16569\_Sample\_011046840, Unigene16898\_Sample\_011046840, Unigene21026\_Sample\_011046840, Unigene21970\_Sample\_011046840, Unigene22397\_Sample\_011046840, Unigene22752\_Sample\_011046840, Unigene25552\_Sample\_011046840, Unigene28429\_Sample\_011046840, Unigene32154\_Sample\_011046840, Unigene33046\_Sample\_011046840, Unigene33521\_Sample\_011046840, Unigene34137\_Sample\_011046840, Unigene34163\_Sample\_011046840, Unigene34566\_Sample\_011046840, Unigene34918\_Sample\_011046840, Unigene35916\_Sample\_011046840, Unigene36366\_Sample\_011046840, Unigene37704\_Sample\_011046840, Unigene39061\_Sample\_011046840, Unigene39286\_Sample\_011046840, Unigene41341\_Sample\_011046840, Unigene43367\_Sample\_011046840, Unigene6861\_Sample\_011046840, Unigene13035\_Sample\_011046840, Unigene15062\_Sample\_011046840, Unigene17146\_Sample\_011046840, Unigene18863\_Sample\_011046840, Unigene21399\_Sample\_011046840, Unigene22322\_Sample\_011046840, Unigene24312\_Sample\_011046840, Unigene27863\_Sample\_011046840, Unigene32250\_Sample\_011046840, Unigene41743\_Sample\_011046840, Unigene2367\_Sample\_011046840, Unigene3185\_Sample\_011046840, Unigene6929\_Sample\_011046840, Unigene8753\_Sample\_011046840, Unigene8931\_Sample\_011046840, Unigene12130\_Sample\_011046840, Unigene13388\_Sample\_011046840, Unigene15455\_Sample\_011046840, Unigene15465\_Sample\_011046840, Unigene16746\_Sample\_011046840, Unigene17397\_Sample\_011046840, Unigene22186\_Sample\_011046840, Unigene22530\_Sample\_011046840, Unigene22669\_Sample\_011046840, Unigene23174\_Sample\_011046840, Unigene25515\_Sample\_011046840, Unigene25629\_Sample\_011046840, Unigene26168\_Sample\_011046840, Unigene27300\_Sample\_011046840, Unigene28171\_Sample\_011046840, Unigene32377\_Sample\_011046840, Unigene32484\_Sample\_011046840, Unigene33595\_Sample\_011046840, Unigene34158\_Sample\_011046840, Unigene34458\_Sample\_011046840, Unigene34909\_Sample\_011046840, Unigene36246\_Sample\_011046840, Unigene36969\_Sample\_011046840, Unigene38085\_Sample\_011046840, Unigene38825\_Sample\_011046840, Unigene39830\_Sample\_011046840, Unigene40015\_Sample\_011046840, Unigene40433\_Sample\_011046840, Unigene41536\_Sample\_011046840, Unigene42140\_Sample\_011046840, Unigene42286\_Sample\_011046840, Unigene42583\_Sample\_011046840, Unigene42997\_Sample\_011046840, Unigene12028\_Sample\_011046840, Unigene12491\_Sample\_011046840, Unigene13533\_Sample\_011046840, Unigene15696\_Sample\_011046840, Unigene15710\_Sample\_011046840, Unigene17719\_Sample\_011046840, Unigene19931\_Sample\_011046840, Unigene21963\_Sample\_011046840, Unigene22004\_Sample\_011046840, Unigene22116\_Sample\_011046840, Unigene23850\_Sample\_011046840, Unigene29712\_Sample\_011046840, Unigene31024\_Sample\_011046840, Unigene33510\_Sample\_011046840, Unigene33915\_Sample\_011046840, Unigene35461\_Sample\_011046840, Unigene35947\_Sample\_011046840, Unigene36075\_Sample\_011046840, Unigene42183\_Sample\_011046840, Unigene5348\_Sample\_011046840, Unigene13700\_Sample\_011046840, Unigene20234\_Sample\_011046840, Unigene22748\_Sample\_011046840, Unigene25337\_Sample\_011046840, Unigene27816\_Sample\_011046840, Unigene29764\_Sample\_011046840, Unigene30608\_Sample\_011046840, Unigene34316\_Sample\_011046840, Unigene36203\_Sample\_011046840, Unigene37565\_Sample\_011046840, Unigene38113\_Sample\_011046840, Unigene40416\_Sample\_011046840, Unigene43459\_Sample\_011046840, Unigene5998\_Sample\_011046840, Unigene8605\_Sample\_011046840, Unigene9973\_Sample\_011046840, Unigene10460\_Sample\_011046840, Unigene11516\_Sample\_011046840, Unigene12978\_Sample\_011046840, Unigene16247\_Sample\_011046840, Unigene19905\_Sample\_011046840, Unigene20400\_Sample\_011046840, Unigene20599\_Sample\_011046840, Unigene22726\_Sample\_011046840, Unigene25018\_Sample\_011046840, Unigene25999\_Sample\_011046840, Unigene26614\_Sample\_011046840, Unigene27348\_Sample\_011046840, Unigene30033\_Sample\_011046840, Unigene30573\_Sample\_011046840, Unigene31669\_Sample\_011046840, Unigene32583\_Sample\_011046840, Unigene32741\_Sample\_011046840, Unigene33205\_Sample\_011046840, Unigene34216\_Sample\_011046840, Unigene34604\_Sample\_011046840, Unigene34783\_Sample\_011046840, Unigene36230\_Sample\_011046840, Unigene36458\_Sample\_011046840, Unigene38631\_Sample\_011046840, Unigene38811\_Sample\_011046840, Unigene39138\_Sample\_011046840, Unigene39687\_Sample\_011046840, Unigene40179\_Sample\_011046840, Unigene1379\_Sample\_011046840, Unigene15420\_Sample\_011046840, Unigene16016\_Sample\_011046840, Unigene25429\_Sample\_011046840, Unigene26538\_Sample\_011046840, Unigene26789\_Sample\_011046840, Unigene27180\_Sample\_011046840, Unigene29125\_Sample\_011046840, Unigene34589\_Sample\_011046840, Unigene36413\_Sample\_011046840, Unigene38146\_Sample\_011046840, Unigene41975\_Sample\_011046840 |
| 114 | Chemokine signaling pathway Back to summary table | Unigene1715\_Sample\_011046840, Unigene4790\_Sample\_011046840, Unigene4993\_Sample\_011046840, Unigene5379\_Sample\_011046840, Unigene8120\_Sample\_011046840, Unigene10035\_Sample\_011046840, Unigene10232\_Sample\_011046840, Unigene15939\_Sample\_011046840, Unigene23856\_Sample\_011046840, Unigene29947\_Sample\_011046840, Unigene32521\_Sample\_011046840, Unigene34179\_Sample\_011046840, Unigene37793\_Sample\_011046840, Unigene38706\_Sample\_011046840, Unigene40152\_Sample\_011046840, Unigene40484\_Sample\_011046840, Unigene41220\_Sample\_011046840, Unigene41242\_Sample\_011046840, Unigene43439\_Sample\_011046840, Unigene1487\_Sample\_011046840, Unigene1859\_Sample\_011046840, Unigene2418\_Sample\_011046840, Unigene2986\_Sample\_011046840, Unigene3812\_Sample\_011046840, Unigene4017\_Sample\_011046840, Unigene4779\_Sample\_011046840, Unigene5389\_Sample\_011046840, Unigene6863\_Sample\_011046840, Unigene8185\_Sample\_011046840, Unigene14981\_Sample\_011046840, Unigene28752\_Sample\_011046840, Unigene34974\_Sample\_011046840, Unigene36317\_Sample\_011046840, Unigene36484\_Sample\_011046840, Unigene36905\_Sample\_011046840, Unigene38617\_Sample\_011046840, Unigene38866\_Sample\_011046840, Unigene39843\_Sample\_011046840, Unigene39883\_Sample\_011046840, Unigene40128\_Sample\_011046840, Unigene40208\_Sample\_011046840, Unigene40388\_Sample\_011046840, Unigene40834\_Sample\_011046840, Unigene41370\_Sample\_011046840, Unigene41584\_Sample\_011046840, Unigene41915\_Sample\_011046840, Unigene42065\_Sample\_011046840, Unigene42164\_Sample\_011046840, Unigene42217\_Sample\_011046840, Unigene42699\_Sample\_011046840, Unigene43018\_Sample\_011046840, Unigene43213\_Sample\_011046840, Unigene43301\_Sample\_011046840, Unigene43481\_Sample\_011046840, Unigene43499\_Sample\_011046840, Unigene14753\_Sample\_011046840, Unigene26624\_Sample\_011046840, Unigene33835\_Sample\_011046840, Unigene38847\_Sample\_011046840, Unigene40469\_Sample\_011046840, Unigene41695\_Sample\_011046840, Unigene43149\_Sample\_011046840, Unigene7605\_Sample\_011046840, Unigene7782\_Sample\_011046840, Unigene22038\_Sample\_011046840, Unigene29542\_Sample\_011046840, Unigene31699\_Sample\_011046840, Unigene35377\_Sample\_011046840, Unigene37705\_Sample\_011046840, Unigene38648\_Sample\_011046840, Unigene41082\_Sample\_011046840, Unigene42798\_Sample\_011046840, Unigene3872\_Sample\_011046840, Unigene4279\_Sample\_011046840, Unigene4818\_Sample\_011046840, Unigene8088\_Sample\_011046840, Unigene9535\_Sample\_011046840, Unigene20325\_Sample\_011046840, Unigene21554\_Sample\_011046840, Unigene23045\_Sample\_011046840, Unigene28708\_Sample\_011046840, Unigene28883\_Sample\_011046840, Unigene30530\_Sample\_011046840, Unigene31703\_Sample\_011046840, Unigene31945\_Sample\_011046840, Unigene33288\_Sample\_011046840, Unigene35150\_Sample\_011046840, Unigene36320\_Sample\_011046840, Unigene39621\_Sample\_011046840, Unigene40659\_Sample\_011046840, Unigene42487\_Sample\_011046840, Unigene42685\_Sample\_011046840, Unigene43068\_Sample\_011046840, Unigene43392\_Sample\_011046840, Unigene14179\_Sample\_011046840, Unigene14379\_Sample\_011046840, Unigene24869\_Sample\_011046840, Unigene26621\_Sample\_011046840, Unigene28872\_Sample\_011046840, Unigene30141\_Sample\_011046840, Unigene34563\_Sample\_011046840, Unigene35901\_Sample\_011046840, Unigene36619\_Sample\_011046840, Unigene37128\_Sample\_011046840, Unigene37954\_Sample\_011046840, Unigene42142\_Sample\_011046840, Unigene42693\_Sample\_011046840, Unigene4139\_Sample\_011046840, Unigene16429\_Sample\_011046840, Unigene18933\_Sample\_011046840, Unigene19933\_Sample\_011046840, Unigene26785\_Sample\_011046840, Unigene27378\_Sample\_011046840, Unigene28751\_Sample\_011046840, Unigene29670\_Sample\_011046840, Unigene31705\_Sample\_011046840, Unigene31880\_Sample\_011046840, Unigene38000\_Sample\_011046840, Unigene39503\_Sample\_011046840, Unigene40189\_Sample\_011046840, Unigene42008\_Sample\_011046840, Unigene43154\_Sample\_011046840, Unigene34490\_Sample\_011046840, Unigene40960\_Sample\_011046840, Unigene42027\_Sample\_011046840, Unigene1368\_Sample\_011046840, Unigene19400\_Sample\_011046840, Unigene20896\_Sample\_011046840, Unigene24411\_Sample\_011046840, Unigene26552\_Sample\_011046840, Unigene28829\_Sample\_011046840, Unigene29526\_Sample\_011046840, Unigene33870\_Sample\_011046840, Unigene38572\_Sample\_011046840, Unigene40016\_Sample\_011046840, Unigene42811\_Sample\_011046840, Unigene5726\_Sample\_011046840, Unigene8614\_Sample\_011046840, Unigene19127\_Sample\_011046840, Unigene21176\_Sample\_011046840, Unigene25531\_Sample\_011046840, Unigene27623\_Sample\_011046840, Unigene28122\_Sample\_011046840, Unigene37246\_Sample\_011046840, Unigene41560\_Sample\_011046840, Unigene42902\_Sample\_011046840, Unigene3861\_Sample\_011046840, Unigene10037\_Sample\_011046840, Unigene38066\_Sample\_011046840, Unigene41704\_Sample\_011046840, Unigene1256\_Sample\_011046840, Unigene4276\_Sample\_011046840, Unigene14930\_Sample\_011046840, Unigene16035\_Sample\_011046840, Unigene18949\_Sample\_011046840, Unigene20420\_Sample\_011046840, Unigene25276\_Sample\_011046840, Unigene26124\_Sample\_011046840, Unigene32265\_Sample\_011046840, Unigene34487\_Sample\_011046840, Unigene41410\_Sample\_011046840, Unigene42184\_Sample\_011046840, Unigene37185\_Sample\_011046840 |
| 115 | Amino sugar and nucleotide sugar metabolism Back to summary table | Unigene2439\_Sample\_011046840, Unigene35007\_Sample\_011046840, Unigene35247\_Sample\_011046840, Unigene35656\_Sample\_011046840, Unigene35984\_Sample\_011046840, Unigene37643\_Sample\_011046840, Unigene2100\_Sample\_011046840, Unigene3492\_Sample\_011046840, Unigene6395\_Sample\_011046840, Unigene7037\_Sample\_011046840, Unigene7739\_Sample\_011046840, Unigene9318\_Sample\_011046840, Unigene15065\_Sample\_011046840, Unigene18001\_Sample\_011046840, Unigene19092\_Sample\_011046840, Unigene34026\_Sample\_011046840, Unigene37647\_Sample\_011046840, Unigene38452\_Sample\_011046840, Unigene38503\_Sample\_011046840, Unigene38772\_Sample\_011046840, Unigene39753\_Sample\_011046840, Unigene41843\_Sample\_011046840, Unigene42236\_Sample\_011046840, Unigene43376\_Sample\_011046840, Unigene5021\_Sample\_011046840, Unigene27667\_Sample\_011046840, Unigene31906\_Sample\_011046840, Unigene33568\_Sample\_011046840, Unigene33684\_Sample\_011046840, Unigene40272\_Sample\_011046840, Unigene1672\_Sample\_011046840, Unigene6033\_Sample\_011046840, Unigene28404\_Sample\_011046840, Unigene35242\_Sample\_011046840, Unigene41162\_Sample\_011046840, Unigene1485\_Sample\_011046840, Unigene5481\_Sample\_011046840, Unigene9446\_Sample\_011046840, Unigene9966\_Sample\_011046840, Unigene16468\_Sample\_011046840, Unigene24044\_Sample\_011046840, Unigene26315\_Sample\_011046840, Unigene35280\_Sample\_011046840, Unigene36629\_Sample\_011046840, Unigene41188\_Sample\_011046840, Unigene42618\_Sample\_011046840, Unigene42743\_Sample\_011046840, Unigene43038\_Sample\_011046840, Unigene20745\_Sample\_011046840, Unigene36525\_Sample\_011046840, Unigene861\_Sample\_011046840, Unigene1150\_Sample\_011046840, Unigene2197\_Sample\_011046840, Unigene7938\_Sample\_011046840, Unigene16550\_Sample\_011046840, Unigene31805\_Sample\_011046840, Unigene34615\_Sample\_011046840, Unigene39079\_Sample\_011046840, Unigene41030\_Sample\_011046840, Unigene41663\_Sample\_011046840, Unigene7774\_Sample\_011046840, Unigene28458\_Sample\_011046840, Unigene42511\_Sample\_011046840, Unigene6867\_Sample\_011046840, Unigene27469\_Sample\_011046840, Unigene29497\_Sample\_011046840, Unigene31587\_Sample\_011046840, Unigene38177\_Sample\_011046840, Unigene40679\_Sample\_011046840, Unigene5834\_Sample\_011046840, Unigene7664\_Sample\_011046840, Unigene13756\_Sample\_011046840, Unigene13889\_Sample\_011046840, Unigene26590\_Sample\_011046840, Unigene33747\_Sample\_011046840, Unigene40302\_Sample\_011046840, Unigene42103\_Sample\_011046840, Unigene43217\_Sample\_011046840, Unigene7340\_Sample\_011046840, Unigene13577\_Sample\_011046840, Unigene14928\_Sample\_011046840, Unigene22607\_Sample\_011046840, Unigene18146\_Sample\_011046840, Unigene21031\_Sample\_011046840, Unigene28689\_Sample\_011046840, Unigene28904\_Sample\_011046840, Unigene34351\_Sample\_011046840, Unigene37367\_Sample\_011046840, Unigene38053\_Sample\_011046840, Unigene41116\_Sample\_011046840, Unigene11179\_Sample\_011046840, Unigene20815\_Sample\_011046840, Unigene35148\_Sample\_011046840, Unigene37097\_Sample\_011046840, Unigene40242\_Sample\_011046840 |
| 116 | Glycosylphosphatidylinositol(GPI)-anchor biosynthesis Back to summary table | Unigene88\_Sample\_011046840, Unigene7963\_Sample\_011046840, Unigene27779\_Sample\_011046840, Unigene35146\_Sample\_011046840, Unigene43487\_Sample\_011046840, Unigene2204\_Sample\_011046840, Unigene7583\_Sample\_011046840, Unigene8205\_Sample\_011046840, Unigene36023\_Sample\_011046840, Unigene38398\_Sample\_011046840, Unigene42486\_Sample\_011046840, Unigene23730\_Sample\_011046840, Unigene34739\_Sample\_011046840, Unigene42897\_Sample\_011046840, Unigene38628\_Sample\_011046840, Unigene5324\_Sample\_011046840, Unigene5837\_Sample\_011046840, Unigene8268\_Sample\_011046840, Unigene21061\_Sample\_011046840, Unigene24744\_Sample\_011046840, Unigene25024\_Sample\_011046840, Unigene34236\_Sample\_011046840, Unigene40047\_Sample\_011046840, Unigene6878\_Sample\_011046840, Unigene9238\_Sample\_011046840, Unigene37281\_Sample\_011046840, Unigene43049\_Sample\_011046840, Unigene19504\_Sample\_011046840, Unigene26900\_Sample\_011046840, Unigene35334\_Sample\_011046840, Unigene10569\_Sample\_011046840, Unigene17372\_Sample\_011046840, Unigene26637\_Sample\_011046840, Unigene27922\_Sample\_011046840, Unigene31503\_Sample\_011046840, Unigene32157\_Sample\_011046840, Unigene36077\_Sample\_011046840, Unigene36229\_Sample\_011046840, Unigene7487\_Sample\_011046840, Unigene36543\_Sample\_011046840, Unigene13592\_Sample\_011046840, Unigene1107\_Sample\_011046840, Unigene26260\_Sample\_011046840, Unigene27153\_Sample\_011046840, Unigene34004\_Sample\_011046840, Unigene34015\_Sample\_011046840 |
| 117 | Zeatin biosynthesis Back to summary table | Unigene22232\_Sample\_011046840, Unigene41399\_Sample\_011046840, Unigene27313\_Sample\_011046840, Unigene28222\_Sample\_011046840 |
| 118 | Flavone and flavonol biosynthesis Back to summary table | Unigene26709\_Sample\_011046840, Unigene30383\_Sample\_011046840, Unigene35431\_Sample\_011046840, Unigene36718\_Sample\_011046840 |
| 119 | 3-Chloroacrylic acid degradation Back to summary table | Unigene4446\_Sample\_011046840, Unigene42713\_Sample\_011046840, Unigene34208\_Sample\_011046840, Unigene35036\_Sample\_011046840 |
| 120 | GnRH signaling pathway Back to summary table | Unigene259\_Sample\_011046840, Unigene463\_Sample\_011046840, Unigene535\_Sample\_011046840, Unigene1883\_Sample\_011046840, Unigene4790\_Sample\_011046840, Unigene5662\_Sample\_011046840, Unigene5831\_Sample\_011046840, Unigene6551\_Sample\_011046840, Unigene8198\_Sample\_011046840, Unigene15939\_Sample\_011046840, Unigene34179\_Sample\_011046840, Unigene36475\_Sample\_011046840, Unigene42127\_Sample\_011046840, Unigene42759\_Sample\_011046840, Unigene42766\_Sample\_011046840, Unigene43439\_Sample\_011046840, Unigene1756\_Sample\_011046840, Unigene2986\_Sample\_011046840, Unigene3025\_Sample\_011046840, Unigene3812\_Sample\_011046840, Unigene5059\_Sample\_011046840, Unigene5564\_Sample\_011046840, Unigene6500\_Sample\_011046840, Unigene7232\_Sample\_011046840, Unigene8112\_Sample\_011046840, Unigene11592\_Sample\_011046840, Unigene27429\_Sample\_011046840, Unigene29711\_Sample\_011046840, Unigene30539\_Sample\_011046840, Unigene33208\_Sample\_011046840, Unigene36690\_Sample\_011046840, Unigene38866\_Sample\_011046840, Unigene40128\_Sample\_011046840, Unigene42065\_Sample\_011046840, Unigene42217\_Sample\_011046840, Unigene43213\_Sample\_011046840, Unigene43421\_Sample\_011046840, Unigene43435\_Sample\_011046840, Unigene43481\_Sample\_011046840, Unigene5166\_Sample\_011046840, Unigene14437\_Sample\_011046840, Unigene29880\_Sample\_011046840, Unigene33983\_Sample\_011046840, Unigene34039\_Sample\_011046840, Unigene38834\_Sample\_011046840, Unigene42204\_Sample\_011046840, Unigene6921\_Sample\_011046840, Unigene7605\_Sample\_011046840, Unigene36839\_Sample\_011046840, Unigene39054\_Sample\_011046840, Unigene40221\_Sample\_011046840, Unigene43434\_Sample\_011046840, Unigene3872\_Sample\_011046840, Unigene20325\_Sample\_011046840, Unigene23045\_Sample\_011046840, Unigene31945\_Sample\_011046840, Unigene36320\_Sample\_011046840, Unigene36788\_Sample\_011046840, Unigene38694\_Sample\_011046840, Unigene42268\_Sample\_011046840, Unigene334\_Sample\_011046840, Unigene14179\_Sample\_011046840, Unigene21962\_Sample\_011046840, Unigene28872\_Sample\_011046840, Unigene33873\_Sample\_011046840, Unigene36619\_Sample\_011046840, Unigene41439\_Sample\_011046840, Unigene42693\_Sample\_011046840, Unigene12153\_Sample\_011046840, Unigene25548\_Sample\_011046840, Unigene28751\_Sample\_011046840, Unigene31880\_Sample\_011046840, Unigene35532\_Sample\_011046840, Unigene35787\_Sample\_011046840, Unigene39503\_Sample\_011046840, Unigene39912\_Sample\_011046840, Unigene39958\_Sample\_011046840, Unigene40189\_Sample\_011046840, Unigene43154\_Sample\_011046840, Unigene10749\_Sample\_011046840, Unigene14237\_Sample\_011046840, Unigene18009\_Sample\_011046840, Unigene26398\_Sample\_011046840, Unigene29660\_Sample\_011046840, Unigene33870\_Sample\_011046840, Unigene37392\_Sample\_011046840, Unigene670\_Sample\_011046840, Unigene8614\_Sample\_011046840, Unigene19127\_Sample\_011046840, Unigene27623\_Sample\_011046840, Unigene39577\_Sample\_011046840, Unigene41560\_Sample\_011046840, Unigene2711\_Sample\_011046840, Unigene13511\_Sample\_011046840, Unigene1568\_Sample\_011046840, Unigene7388\_Sample\_011046840, Unigene29621\_Sample\_011046840, Unigene32011\_Sample\_011046840, Unigene34487\_Sample\_011046840, Unigene41689\_Sample\_011046840, Unigene19593\_Sample\_011046840, Unigene24889\_Sample\_011046840, Unigene33809\_Sample\_011046840, Unigene37186\_Sample\_011046840, Unigene40453\_Sample\_011046840, Unigene43443\_Sample\_011046840 |
| 121 | Sphingolipid metabolism Back to summary table | Unigene6705\_Sample\_011046840, Unigene7919\_Sample\_011046840, Unigene39857\_Sample\_011046840, Unigene8053\_Sample\_011046840, Unigene32901\_Sample\_011046840, Unigene35693\_Sample\_011046840, Unigene36508\_Sample\_011046840, Unigene36947\_Sample\_011046840, Unigene37282\_Sample\_011046840, Unigene39235\_Sample\_011046840, Unigene39609\_Sample\_011046840, Unigene39742\_Sample\_011046840, Unigene41335\_Sample\_011046840, Unigene42214\_Sample\_011046840, Unigene43177\_Sample\_011046840, Unigene36152\_Sample\_011046840, Unigene42417\_Sample\_011046840, Unigene3867\_Sample\_011046840, Unigene30746\_Sample\_011046840, Unigene35138\_Sample\_011046840, Unigene38481\_Sample\_011046840, Unigene42282\_Sample\_011046840, Unigene7859\_Sample\_011046840, Unigene10296\_Sample\_011046840, Unigene13288\_Sample\_011046840, Unigene31369\_Sample\_011046840, Unigene32964\_Sample\_011046840, Unigene35885\_Sample\_011046840, Unigene37900\_Sample\_011046840, Unigene39053\_Sample\_011046840, Unigene43231\_Sample\_011046840, Unigene35254\_Sample\_011046840, Unigene22230\_Sample\_011046840, Unigene29690\_Sample\_011046840, Unigene39343\_Sample\_011046840, Unigene40924\_Sample\_011046840, Unigene9750\_Sample\_011046840, Unigene12226\_Sample\_011046840, Unigene24336\_Sample\_011046840, Unigene38070\_Sample\_011046840, Unigene38387\_Sample\_011046840, Unigene29233\_Sample\_011046840, Unigene32443\_Sample\_011046840, Unigene32691\_Sample\_011046840, Unigene33959\_Sample\_011046840, Unigene34624\_Sample\_011046840, Unigene42076\_Sample\_011046840, Unigene4548\_Sample\_011046840, Unigene34642\_Sample\_011046840, Unigene36709\_Sample\_011046840, Unigene38317\_Sample\_011046840, Unigene9541\_Sample\_011046840, Unigene22528\_Sample\_011046840, Unigene31878\_Sample\_011046840, Unigene37580\_Sample\_011046840, Unigene37627\_Sample\_011046840, Unigene937\_Sample\_011046840 |
| 122 | Glyoxylate and dicarboxylate metabolism Back to summary table | Unigene33042\_Sample\_011046840, Unigene43191\_Sample\_011046840, Unigene1330\_Sample\_011046840, Unigene39916\_Sample\_011046840, Unigene33585\_Sample\_011046840, Unigene4670\_Sample\_011046840, Unigene43181\_Sample\_011046840, Unigene5226\_Sample\_011046840, Unigene37975\_Sample\_011046840, Unigene26868\_Sample\_011046840, Unigene12191\_Sample\_011046840, Unigene24649\_Sample\_011046840, Unigene33008\_Sample\_011046840, Unigene35275\_Sample\_011046840, Unigene37285\_Sample\_011046840 |
| 123 | Oocyte meiosis Back to summary table | Unigene1129\_Sample\_011046840, Unigene2690\_Sample\_011046840, Unigene2865\_Sample\_011046840, Unigene3123\_Sample\_011046840, Unigene6644\_Sample\_011046840, Unigene7525\_Sample\_011046840, Unigene8140\_Sample\_011046840, Unigene9558\_Sample\_011046840, Unigene13399\_Sample\_011046840, Unigene15939\_Sample\_011046840, Unigene25743\_Sample\_011046840, Unigene26863\_Sample\_011046840, Unigene28917\_Sample\_011046840, Unigene32488\_Sample\_011046840, Unigene33337\_Sample\_011046840, Unigene40589\_Sample\_011046840, Unigene42859\_Sample\_011046840, Unigene43019\_Sample\_011046840, Unigene43439\_Sample\_011046840, Unigene43493\_Sample\_011046840, Unigene2986\_Sample\_011046840, Unigene3474\_Sample\_011046840, Unigene4477\_Sample\_011046840, Unigene4497\_Sample\_011046840, Unigene4695\_Sample\_011046840, Unigene6139\_Sample\_011046840, Unigene6500\_Sample\_011046840, Unigene7868\_Sample\_011046840, Unigene8203\_Sample\_011046840, Unigene14078\_Sample\_011046840, Unigene18467\_Sample\_011046840, Unigene27185\_Sample\_011046840, Unigene29864\_Sample\_011046840, Unigene32520\_Sample\_011046840, Unigene32593\_Sample\_011046840, Unigene32791\_Sample\_011046840, Unigene35491\_Sample\_011046840, Unigene35780\_Sample\_011046840, Unigene36364\_Sample\_011046840, Unigene36846\_Sample\_011046840, Unigene36911\_Sample\_011046840, Unigene37340\_Sample\_011046840, Unigene37890\_Sample\_011046840, Unigene40128\_Sample\_011046840, Unigene40685\_Sample\_011046840, Unigene41388\_Sample\_011046840, Unigene41444\_Sample\_011046840, Unigene41550\_Sample\_011046840, Unigene41621\_Sample\_011046840, Unigene41673\_Sample\_011046840, Unigene42065\_Sample\_011046840, Unigene42284\_Sample\_011046840, Unigene42991\_Sample\_011046840, Unigene43162\_Sample\_011046840, Unigene43213\_Sample\_011046840, Unigene43273\_Sample\_011046840, Unigene43395\_Sample\_011046840, Unigene43397\_Sample\_011046840, Unigene43481\_Sample\_011046840, Unigene43517\_Sample\_011046840, Unigene43635\_Sample\_011046840, Unigene2080\_Sample\_011046840, Unigene2493\_Sample\_011046840, Unigene2541\_Sample\_011046840, Unigene5166\_Sample\_011046840, Unigene33638\_Sample\_011046840, Unigene38540\_Sample\_011046840, Unigene41114\_Sample\_011046840, Unigene42866\_Sample\_011046840, Unigene7605\_Sample\_011046840, Unigene19991\_Sample\_011046840, Unigene24747\_Sample\_011046840, Unigene28116\_Sample\_011046840, Unigene33396\_Sample\_011046840, Unigene33840\_Sample\_011046840, Unigene39054\_Sample\_011046840, Unigene39200\_Sample\_011046840, Unigene39778\_Sample\_011046840, Unigene43111\_Sample\_011046840, Unigene634\_Sample\_011046840, Unigene3872\_Sample\_011046840, Unigene4610\_Sample\_011046840, Unigene10400\_Sample\_011046840, Unigene11174\_Sample\_011046840, Unigene13449\_Sample\_011046840, Unigene14373\_Sample\_011046840, Unigene14824\_Sample\_011046840, Unigene22321\_Sample\_011046840, Unigene25450\_Sample\_011046840, Unigene26897\_Sample\_011046840, Unigene31945\_Sample\_011046840, Unigene33849\_Sample\_011046840, Unigene36788\_Sample\_011046840, Unigene36800\_Sample\_011046840, Unigene36860\_Sample\_011046840, Unigene38504\_Sample\_011046840, Unigene41337\_Sample\_011046840, Unigene42169\_Sample\_011046840, Unigene42240\_Sample\_011046840, Unigene334\_Sample\_011046840, Unigene16307\_Sample\_011046840, Unigene16618\_Sample\_011046840, Unigene22606\_Sample\_011046840, Unigene22643\_Sample\_011046840, Unigene22765\_Sample\_011046840, Unigene28124\_Sample\_011046840, Unigene33222\_Sample\_011046840, Unigene36619\_Sample\_011046840, Unigene38121\_Sample\_011046840, Unigene41125\_Sample\_011046840, Unigene42693\_Sample\_011046840, Unigene3486\_Sample\_011046840, Unigene7372\_Sample\_011046840, Unigene18768\_Sample\_011046840, Unigene27233\_Sample\_011046840, Unigene29958\_Sample\_011046840, Unigene31880\_Sample\_011046840, Unigene32620\_Sample\_011046840, Unigene32645\_Sample\_011046840, Unigene34436\_Sample\_011046840, Unigene38902\_Sample\_011046840, Unigene39912\_Sample\_011046840, Unigene41425\_Sample\_011046840, Unigene41691\_Sample\_011046840, Unigene42927\_Sample\_011046840, Unigene12320\_Sample\_011046840, Unigene23714\_Sample\_011046840, Unigene40665\_Sample\_011046840, Unigene41909\_Sample\_011046840, Unigene4125\_Sample\_011046840, Unigene6944\_Sample\_011046840, Unigene8801\_Sample\_011046840, Unigene9678\_Sample\_011046840, Unigene11225\_Sample\_011046840, Unigene13531\_Sample\_011046840, Unigene17676\_Sample\_011046840, Unigene27358\_Sample\_011046840, Unigene30703\_Sample\_011046840, Unigene31648\_Sample\_011046840, Unigene32355\_Sample\_011046840, Unigene32479\_Sample\_011046840, Unigene32607\_Sample\_011046840, Unigene33161\_Sample\_011046840, Unigene33870\_Sample\_011046840, Unigene36291\_Sample\_011046840, Unigene37392\_Sample\_011046840, Unigene38455\_Sample\_011046840, Unigene39412\_Sample\_011046840, Unigene42242\_Sample\_011046840, Unigene670\_Sample\_011046840, Unigene12229\_Sample\_011046840, Unigene15271\_Sample\_011046840, Unigene15570\_Sample\_011046840, Unigene24021\_Sample\_011046840, Unigene26520\_Sample\_011046840, Unigene31266\_Sample\_011046840, Unigene33722\_Sample\_011046840, Unigene34096\_Sample\_011046840, Unigene36385\_Sample\_011046840, Unigene37505\_Sample\_011046840, Unigene39577\_Sample\_011046840, Unigene40019\_Sample\_011046840, Unigene2711\_Sample\_011046840, Unigene10037\_Sample\_011046840, Unigene10196\_Sample\_011046840, Unigene13511\_Sample\_011046840, Unigene23373\_Sample\_011046840, Unigene29661\_Sample\_011046840, Unigene32864\_Sample\_011046840, Unigene42173\_Sample\_011046840, Unigene1344\_Sample\_011046840, Unigene7388\_Sample\_011046840, Unigene10612\_Sample\_011046840, Unigene11650\_Sample\_011046840, Unigene14227\_Sample\_011046840, Unigene14438\_Sample\_011046840, Unigene19655\_Sample\_011046840, Unigene20430\_Sample\_011046840, Unigene20606\_Sample\_011046840, Unigene23019\_Sample\_011046840, Unigene29411\_Sample\_011046840, Unigene31090\_Sample\_011046840, Unigene35493\_Sample\_011046840, Unigene35789\_Sample\_011046840, Unigene39224\_Sample\_011046840, Unigene42473\_Sample\_011046840, Unigene2308\_Sample\_011046840, Unigene2401\_Sample\_011046840, Unigene11742\_Sample\_011046840, Unigene15195\_Sample\_011046840, Unigene29269\_Sample\_011046840, Unigene31562\_Sample\_011046840, Unigene36819\_Sample\_011046840, Unigene38610\_Sample\_011046840, Unigene40227\_Sample\_011046840, Unigene43443\_Sample\_011046840 |
| 124 | Two-component system Back to summary table | Unigene38139\_Sample\_011046840, Unigene23357\_Sample\_011046840, Unigene33172\_Sample\_011046840, Unigene31278\_Sample\_011046840, Unigene3810\_Sample\_011046840, Unigene28199\_Sample\_011046840, Unigene25488\_Sample\_011046840, Unigene16873\_Sample\_011046840, Unigene18659\_Sample\_011046840 |
| 125 | Fc gamma R-mediated phagocytosis Back to summary table | Unigene880\_Sample\_011046840, Unigene4993\_Sample\_011046840, Unigene10232\_Sample\_011046840, Unigene15939\_Sample\_011046840, Unigene30862\_Sample\_011046840, Unigene32514\_Sample\_011046840, Unigene35141\_Sample\_011046840, Unigene40745\_Sample\_011046840, Unigene43135\_Sample\_011046840, Unigene43321\_Sample\_011046840, Unigene43470\_Sample\_011046840, Unigene1756\_Sample\_011046840, Unigene2986\_Sample\_011046840, Unigene4033\_Sample\_011046840, Unigene4101\_Sample\_011046840, Unigene7631\_Sample\_011046840, Unigene8053\_Sample\_011046840, Unigene27902\_Sample\_011046840, Unigene28742\_Sample\_011046840, Unigene34585\_Sample\_011046840, Unigene34974\_Sample\_011046840, Unigene36484\_Sample\_011046840, Unigene36508\_Sample\_011046840, Unigene36947\_Sample\_011046840, Unigene37892\_Sample\_011046840, Unigene38617\_Sample\_011046840, Unigene39843\_Sample\_011046840, Unigene39883\_Sample\_011046840, Unigene40297\_Sample\_011046840, Unigene40697\_Sample\_011046840, Unigene40878\_Sample\_011046840, Unigene41370\_Sample\_011046840, Unigene41915\_Sample\_011046840, Unigene41968\_Sample\_011046840, Unigene41996\_Sample\_011046840, Unigene42164\_Sample\_011046840, Unigene42996\_Sample\_011046840, Unigene43166\_Sample\_011046840, Unigene43499\_Sample\_011046840, Unigene3608\_Sample\_011046840, Unigene5933\_Sample\_011046840, Unigene33835\_Sample\_011046840, Unigene38847\_Sample\_011046840, Unigene40469\_Sample\_011046840, Unigene42417\_Sample\_011046840, Unigene43149\_Sample\_011046840, Unigene3161\_Sample\_011046840, Unigene6322\_Sample\_011046840, Unigene38648\_Sample\_011046840, Unigene4807\_Sample\_011046840, Unigene18272\_Sample\_011046840, Unigene28754\_Sample\_011046840, Unigene28883\_Sample\_011046840, Unigene35150\_Sample\_011046840, Unigene36665\_Sample\_011046840, Unigene36734\_Sample\_011046840, Unigene39053\_Sample\_011046840, Unigene40659\_Sample\_011046840, Unigene42487\_Sample\_011046840, Unigene42871\_Sample\_011046840, Unigene43068\_Sample\_011046840, Unigene43231\_Sample\_011046840, Unigene43392\_Sample\_011046840, Unigene24869\_Sample\_011046840, Unigene26621\_Sample\_011046840, Unigene28450\_Sample\_011046840, Unigene30141\_Sample\_011046840, Unigene31181\_Sample\_011046840, Unigene34563\_Sample\_011046840, Unigene35901\_Sample\_011046840, Unigene41439\_Sample\_011046840, Unigene42134\_Sample\_011046840, Unigene4139\_Sample\_011046840, Unigene17368\_Sample\_011046840, Unigene18933\_Sample\_011046840, Unigene26785\_Sample\_011046840, Unigene29670\_Sample\_011046840, Unigene31880\_Sample\_011046840, Unigene36992\_Sample\_011046840, Unigene37830\_Sample\_011046840, Unigene40648\_Sample\_011046840, Unigene42008\_Sample\_011046840, Unigene42385\_Sample\_011046840, Unigene43154\_Sample\_011046840, Unigene43533\_Sample\_011046840, Unigene40259\_Sample\_011046840, Unigene1368\_Sample\_011046840, Unigene4849\_Sample\_011046840, Unigene20896\_Sample\_011046840, Unigene22254\_Sample\_011046840, Unigene24972\_Sample\_011046840, Unigene25317\_Sample\_011046840, Unigene33870\_Sample\_011046840, Unigene34624\_Sample\_011046840, Unigene38572\_Sample\_011046840, Unigene42811\_Sample\_011046840, Unigene5726\_Sample\_011046840, Unigene15260\_Sample\_011046840, Unigene19127\_Sample\_011046840, Unigene21176\_Sample\_011046840, Unigene12163\_Sample\_011046840, Unigene33692\_Sample\_011046840, Unigene41704\_Sample\_011046840, Unigene11201\_Sample\_011046840, Unigene14930\_Sample\_011046840, Unigene16035\_Sample\_011046840, Unigene17677\_Sample\_011046840, Unigene32265\_Sample\_011046840, Unigene41056\_Sample\_011046840, Unigene31926\_Sample\_011046840 |
| 126 | Collecting duct acid secretion (no map in kegg database) Back to summary table | Unigene2245\_Sample\_011046840, Unigene2492\_Sample\_011046840, Unigene4681\_Sample\_011046840, Unigene6606\_Sample\_011046840, Unigene40184\_Sample\_011046840, Unigene41202\_Sample\_011046840, Unigene41628\_Sample\_011046840, Unigene8051\_Sample\_011046840, Unigene29794\_Sample\_011046840, Unigene36762\_Sample\_011046840, Unigene42758\_Sample\_011046840, Unigene1399\_Sample\_011046840, Unigene42421\_Sample\_011046840, Unigene20538\_Sample\_011046840, Unigene35976\_Sample\_011046840, Unigene38028\_Sample\_011046840, Unigene15594\_Sample\_011046840, Unigene25781\_Sample\_011046840, Unigene37629\_Sample\_011046840, Unigene38677\_Sample\_011046840, Unigene40513\_Sample\_011046840, Unigene42595\_Sample\_011046840, Unigene12084\_Sample\_011046840, Unigene28830\_Sample\_011046840, Unigene36382\_Sample\_011046840, Unigene36687\_Sample\_011046840, Unigene41035\_Sample\_011046840, Unigene17166\_Sample\_011046840, Unigene33547\_Sample\_011046840, Unigene13322\_Sample\_011046840, Unigene23323\_Sample\_011046840, Unigene24674\_Sample\_011046840, Unigene3539\_Sample\_011046840, Unigene25046\_Sample\_011046840, Unigene14612\_Sample\_011046840, Unigene17721\_Sample\_011046840, Unigene20157\_Sample\_011046840, Unigene28467\_Sample\_011046840, Unigene37938\_Sample\_011046840, Unigene15362\_Sample\_011046840, Unigene20109\_Sample\_011046840, Unigene32555\_Sample\_011046840, Unigene34612\_Sample\_011046840 |
| 127 | Autoimmune thyroid disease Back to summary table | Unigene43193\_Sample\_011046840, Unigene6996\_Sample\_011046840, Unigene7091\_Sample\_011046840, Unigene39938\_Sample\_011046840, Unigene3335\_Sample\_011046840, Unigene32463\_Sample\_011046840, Unigene7250\_Sample\_011046840, Unigene21594\_Sample\_011046840, Unigene39613\_Sample\_011046840, Unigene643\_Sample\_011046840, Unigene12234\_Sample\_011046840, Unigene24335\_Sample\_011046840, Unigene18122\_Sample\_011046840, Unigene26954\_Sample\_011046840 |
| 128 | Arachidonic acid metabolism Back to summary table | Unigene36918\_Sample\_011046840, Unigene37179\_Sample\_011046840, Unigene2776\_Sample\_011046840, Unigene3288\_Sample\_011046840, Unigene5059\_Sample\_011046840, Unigene6826\_Sample\_011046840, Unigene7232\_Sample\_011046840, Unigene13788\_Sample\_011046840, Unigene34541\_Sample\_011046840, Unigene37663\_Sample\_011046840, Unigene43421\_Sample\_011046840, Unigene11797\_Sample\_011046840, Unigene35123\_Sample\_011046840, Unigene41350\_Sample\_011046840, Unigene27109\_Sample\_011046840, Unigene41233\_Sample\_011046840, Unigene42268\_Sample\_011046840, Unigene28814\_Sample\_011046840, Unigene34167\_Sample\_011046840, Unigene19961\_Sample\_011046840, Unigene26398\_Sample\_011046840, Unigene5168\_Sample\_011046840, Unigene6728\_Sample\_011046840, Unigene26805\_Sample\_011046840, Unigene35266\_Sample\_011046840, Unigene9958\_Sample\_011046840, Unigene16168\_Sample\_011046840, Unigene32011\_Sample\_011046840, Unigene41689\_Sample\_011046840, Unigene30592\_Sample\_011046840, Unigene33809\_Sample\_011046840 |
| 129 | Vasopressin-regulated water reabsorption Back to summary table | Unigene1037\_Sample\_011046840, Unigene8202\_Sample\_011046840, Unigene35646\_Sample\_011046840, Unigene37192\_Sample\_011046840, Unigene38784\_Sample\_011046840, Unigene40040\_Sample\_011046840, Unigene43439\_Sample\_011046840, Unigene8208\_Sample\_011046840, Unigene14420\_Sample\_011046840, Unigene25215\_Sample\_011046840, Unigene27013\_Sample\_011046840, Unigene29170\_Sample\_011046840, Unigene33999\_Sample\_011046840, Unigene36050\_Sample\_011046840, Unigene37822\_Sample\_011046840, Unigene39692\_Sample\_011046840, Unigene40128\_Sample\_011046840, Unigene40129\_Sample\_011046840, Unigene40157\_Sample\_011046840, Unigene40532\_Sample\_011046840, Unigene41100\_Sample\_011046840, Unigene41481\_Sample\_011046840, Unigene42065\_Sample\_011046840, Unigene42099\_Sample\_011046840, Unigene42383\_Sample\_011046840, Unigene42865\_Sample\_011046840, Unigene42950\_Sample\_011046840, Unigene2072\_Sample\_011046840, Unigene30615\_Sample\_011046840, Unigene35768\_Sample\_011046840, Unigene36702\_Sample\_011046840, Unigene43344\_Sample\_011046840, Unigene23512\_Sample\_011046840, Unigene26956\_Sample\_011046840, Unigene39571\_Sample\_011046840, Unigene42837\_Sample\_011046840, Unigene3872\_Sample\_011046840, Unigene6498\_Sample\_011046840, Unigene7671\_Sample\_011046840, Unigene12679\_Sample\_011046840, Unigene21224\_Sample\_011046840, Unigene25001\_Sample\_011046840, Unigene31945\_Sample\_011046840, Unigene39015\_Sample\_011046840, Unigene40217\_Sample\_011046840, Unigene40369\_Sample\_011046840, Unigene41281\_Sample\_011046840, Unigene43396\_Sample\_011046840, Unigene43491\_Sample\_011046840, Unigene7458\_Sample\_011046840, Unigene36619\_Sample\_011046840, Unigene40704\_Sample\_011046840, Unigene42693\_Sample\_011046840, Unigene23148\_Sample\_011046840, Unigene24531\_Sample\_011046840, Unigene36598\_Sample\_011046840, Unigene43021\_Sample\_011046840, Unigene43607\_Sample\_011046840, Unigene14139\_Sample\_011046840, Unigene16017\_Sample\_011046840, Unigene16130\_Sample\_011046840, Unigene21028\_Sample\_011046840, Unigene24453\_Sample\_011046840, Unigene29983\_Sample\_011046840, Unigene11451\_Sample\_011046840, Unigene18730\_Sample\_011046840, Unigene26708\_Sample\_011046840, Unigene30866\_Sample\_011046840, Unigene38215\_Sample\_011046840, Unigene2810\_Sample\_011046840, Unigene9880\_Sample\_011046840, Unigene25149\_Sample\_011046840, Unigene28952\_Sample\_011046840, Unigene34963\_Sample\_011046840, Unigene164\_Sample\_011046840, Unigene15637\_Sample\_011046840, Unigene19387\_Sample\_011046840, Unigene22185\_Sample\_011046840, Unigene29291\_Sample\_011046840, Unigene17979\_Sample\_011046840, Unigene27340\_Sample\_011046840, Unigene30628\_Sample\_011046840, Unigene34304\_Sample\_011046840, Unigene40453\_Sample\_011046840 |
| 130 | Non-small cell lung cancer Back to summary table | Unigene880\_Sample\_011046840, Unigene4790\_Sample\_011046840, Unigene7744\_Sample\_011046840, Unigene15939\_Sample\_011046840, Unigene30862\_Sample\_011046840, Unigene40260\_Sample\_011046840, Unigene43176\_Sample\_011046840, Unigene2261\_Sample\_011046840, Unigene2418\_Sample\_011046840, Unigene2986\_Sample\_011046840, Unigene4033\_Sample\_011046840, Unigene4082\_Sample\_011046840, Unigene4101\_Sample\_011046840, Unigene8009\_Sample\_011046840, Unigene11592\_Sample\_011046840, Unigene24698\_Sample\_011046840, Unigene28752\_Sample\_011046840, Unigene33208\_Sample\_011046840, Unigene34974\_Sample\_011046840, Unigene36690\_Sample\_011046840, Unigene38617\_Sample\_011046840, Unigene38866\_Sample\_011046840, Unigene40208\_Sample\_011046840, Unigene41584\_Sample\_011046840, Unigene42164\_Sample\_011046840, Unigene3608\_Sample\_011046840, Unigene33983\_Sample\_011046840, Unigene38834\_Sample\_011046840, Unigene38847\_Sample\_011046840, Unigene42204\_Sample\_011046840, Unigene43149\_Sample\_011046840, Unigene3161\_Sample\_011046840, Unigene6322\_Sample\_011046840, Unigene4807\_Sample\_011046840, Unigene20325\_Sample\_011046840, Unigene23045\_Sample\_011046840, Unigene28708\_Sample\_011046840, Unigene36320\_Sample\_011046840, Unigene42509\_Sample\_011046840, Unigene43068\_Sample\_011046840, Unigene24869\_Sample\_011046840, Unigene30860\_Sample\_011046840, Unigene38245\_Sample\_011046840, Unigene4139\_Sample\_011046840, Unigene7125\_Sample\_011046840, Unigene12153\_Sample\_011046840, Unigene16429\_Sample\_011046840, Unigene18933\_Sample\_011046840, Unigene26785\_Sample\_011046840, Unigene27378\_Sample\_011046840, Unigene28751\_Sample\_011046840, Unigene31880\_Sample\_011046840, Unigene35787\_Sample\_011046840, Unigene39503\_Sample\_011046840, Unigene40189\_Sample\_011046840, Unigene40648\_Sample\_011046840, Unigene42008\_Sample\_011046840, Unigene42385\_Sample\_011046840, Unigene43154\_Sample\_011046840, Unigene1368\_Sample\_011046840, Unigene4849\_Sample\_011046840, Unigene20896\_Sample\_011046840, Unigene33870\_Sample\_011046840, Unigene42811\_Sample\_011046840, Unigene5726\_Sample\_011046840, Unigene21176\_Sample\_011046840, Unigene27623\_Sample\_011046840, Unigene41560\_Sample\_011046840, Unigene33524\_Sample\_011046840, Unigene33692\_Sample\_011046840, Unigene35972\_Sample\_011046840, Unigene41704\_Sample\_011046840, Unigene11201\_Sample\_011046840, Unigene16035\_Sample\_011046840, Unigene20420\_Sample\_011046840, Unigene32265\_Sample\_011046840, Unigene33028\_Sample\_011046840, Unigene33393\_Sample\_011046840 |
| 131 | Glycerolipid metabolism Back to summary table | Unigene4244\_Sample\_011046840, Unigene29235\_Sample\_011046840, Unigene33730\_Sample\_011046840, Unigene37093\_Sample\_011046840, Unigene37768\_Sample\_011046840, Unigene1829\_Sample\_011046840, Unigene6147\_Sample\_011046840, Unigene7024\_Sample\_011046840, Unigene8053\_Sample\_011046840, Unigene14093\_Sample\_011046840, Unigene23103\_Sample\_011046840, Unigene25305\_Sample\_011046840, Unigene27236\_Sample\_011046840, Unigene28519\_Sample\_011046840, Unigene31096\_Sample\_011046840, Unigene32030\_Sample\_011046840, Unigene33542\_Sample\_011046840, Unigene35618\_Sample\_011046840, Unigene36293\_Sample\_011046840, Unigene36508\_Sample\_011046840, Unigene36947\_Sample\_011046840, Unigene38795\_Sample\_011046840, Unigene39742\_Sample\_011046840, Unigene40060\_Sample\_011046840, Unigene41136\_Sample\_011046840, Unigene41796\_Sample\_011046840, Unigene42598\_Sample\_011046840, Unigene2783\_Sample\_011046840, Unigene4178\_Sample\_011046840, Unigene17873\_Sample\_011046840, Unigene33360\_Sample\_011046840, Unigene42241\_Sample\_011046840, Unigene42417\_Sample\_011046840, Unigene4446\_Sample\_011046840, Unigene6970\_Sample\_011046840, Unigene7428\_Sample\_011046840, Unigene10123\_Sample\_011046840, Unigene30964\_Sample\_011046840, Unigene39053\_Sample\_011046840, Unigene5509\_Sample\_011046840, Unigene16699\_Sample\_011046840, Unigene21688\_Sample\_011046840, Unigene30107\_Sample\_011046840, Unigene8004\_Sample\_011046840, Unigene29690\_Sample\_011046840, Unigene33328\_Sample\_011046840, Unigene41137\_Sample\_011046840, Unigene42302\_Sample\_011046840, Unigene14678\_Sample\_011046840, Unigene34624\_Sample\_011046840, Unigene37422\_Sample\_011046840, Unigene42489\_Sample\_011046840, Unigene13802\_Sample\_011046840, Unigene30699\_Sample\_011046840, Unigene36457\_Sample\_011046840, Unigene24649\_Sample\_011046840, Unigene32156\_Sample\_011046840, Unigene1572\_Sample\_011046840, Unigene20546\_Sample\_011046840, Unigene36298\_Sample\_011046840 |
| 132 | Antigen processing and presentation Back to summary table | Unigene8020\_Sample\_011046840, Unigene8864\_Sample\_011046840, Unigene789\_Sample\_011046840, Unigene4320\_Sample\_011046840, Unigene4561\_Sample\_011046840, Unigene5047\_Sample\_011046840, Unigene32598\_Sample\_011046840, Unigene35341\_Sample\_011046840, Unigene40007\_Sample\_011046840, Unigene41717\_Sample\_011046840, Unigene42398\_Sample\_011046840, Unigene3954\_Sample\_011046840, Unigene9502\_Sample\_011046840, Unigene13794\_Sample\_011046840, Unigene4268\_Sample\_011046840, Unigene8072\_Sample\_011046840, Unigene3218\_Sample\_011046840, Unigene17426\_Sample\_011046840, Unigene30457\_Sample\_011046840, Unigene36326\_Sample\_011046840, Unigene38770\_Sample\_011046840, Unigene39401\_Sample\_011046840, Unigene40590\_Sample\_011046840, Unigene5791\_Sample\_011046840, Unigene3212\_Sample\_011046840, Unigene23590\_Sample\_011046840, Unigene26070\_Sample\_011046840, Unigene6374\_Sample\_011046840, Unigene17776\_Sample\_011046840, Unigene2762\_Sample\_011046840, Unigene19996\_Sample\_011046840, Unigene26597\_Sample\_011046840, Unigene42427\_Sample\_011046840, Unigene1317\_Sample\_011046840, Unigene20949\_Sample\_011046840, Unigene35818\_Sample\_011046840, Unigene14784\_Sample\_011046840, Unigene30573\_Sample\_011046840, Unigene31389\_Sample\_011046840, Unigene1379\_Sample\_011046840, Unigene16016\_Sample\_011046840, Unigene34366\_Sample\_011046840 |
| 133 | Benzoate degradation via hydroxylation Back to summary table | Unigene37244\_Sample\_011046840, Unigene37714\_Sample\_011046840, Unigene32278\_Sample\_011046840 |
| 134 | Atrazine degradation Back to summary table | Unigene2202\_Sample\_011046840, Unigene40779\_Sample\_011046840, Unigene38982\_Sample\_011046840 |
| 135 | Biotin metabolism Back to summary table | Unigene43560\_Sample\_011046840, Unigene43291\_Sample\_011046840, Unigene2538\_Sample\_011046840 |
| 136 | Sulfur metabolism Back to summary table | Unigene26807\_Sample\_011046840, Unigene39586\_Sample\_011046840, Unigene7348\_Sample\_011046840, Unigene14213\_Sample\_011046840, Unigene9307\_Sample\_011046840, Unigene42323\_Sample\_011046840, Unigene32268\_Sample\_011046840, Unigene37885\_Sample\_011046840 |
| 137 | Prion diseases Back to summary table | Unigene6551\_Sample\_011046840, Unigene7268\_Sample\_011046840, Unigene8167\_Sample\_011046840, Unigene15939\_Sample\_011046840, Unigene29477\_Sample\_011046840, Unigene1705\_Sample\_011046840, Unigene2986\_Sample\_011046840, Unigene3258\_Sample\_011046840, Unigene13603\_Sample\_011046840, Unigene34833\_Sample\_011046840, Unigene40128\_Sample\_011046840, Unigene40559\_Sample\_011046840, Unigene41221\_Sample\_011046840, Unigene42065\_Sample\_011046840, Unigene42327\_Sample\_011046840, Unigene4155\_Sample\_011046840, Unigene7684\_Sample\_011046840, Unigene16269\_Sample\_011046840, Unigene7678\_Sample\_011046840, Unigene15508\_Sample\_011046840, Unigene31945\_Sample\_011046840, Unigene37846\_Sample\_011046840, Unigene39131\_Sample\_011046840, Unigene7035\_Sample\_011046840, Unigene693\_Sample\_011046840, Unigene31880\_Sample\_011046840, Unigene7772\_Sample\_011046840, Unigene33870\_Sample\_011046840, Unigene40261\_Sample\_011046840, Unigene36889\_Sample\_011046840 |
| 138 | Pentose phosphate pathway Back to summary table | Unigene6231\_Sample\_011046840, Unigene19075\_Sample\_011046840, Unigene35056\_Sample\_011046840, Unigene42885\_Sample\_011046840, Unigene2100\_Sample\_011046840, Unigene4158\_Sample\_011046840, Unigene6925\_Sample\_011046840, Unigene13053\_Sample\_011046840, Unigene14254\_Sample\_011046840, Unigene21826\_Sample\_011046840, Unigene32079\_Sample\_011046840, Unigene38452\_Sample\_011046840, Unigene39835\_Sample\_011046840, Unigene40602\_Sample\_011046840, Unigene43376\_Sample\_011046840, Unigene29390\_Sample\_011046840, Unigene33748\_Sample\_011046840, Unigene35242\_Sample\_011046840, Unigene38096\_Sample\_011046840, Unigene42253\_Sample\_011046840, Unigene9446\_Sample\_011046840, Unigene16468\_Sample\_011046840, Unigene42835\_Sample\_011046840, Unigene43606\_Sample\_011046840, Unigene5490\_Sample\_011046840, Unigene7114\_Sample\_011046840, Unigene7157\_Sample\_011046840, Unigene20745\_Sample\_011046840, Unigene23606\_Sample\_011046840, Unigene38657\_Sample\_011046840, Unigene42845\_Sample\_011046840, Unigene25274\_Sample\_011046840, Unigene31805\_Sample\_011046840, Unigene37810\_Sample\_011046840, Unigene17098\_Sample\_011046840, Unigene19312\_Sample\_011046840, Unigene28458\_Sample\_011046840, Unigene29743\_Sample\_011046840, Unigene36699\_Sample\_011046840, Unigene18027\_Sample\_011046840, Unigene25660\_Sample\_011046840, Unigene35963\_Sample\_011046840, Unigene9555\_Sample\_011046840, Unigene36493\_Sample\_011046840, Unigene13577\_Sample\_011046840, Unigene34226\_Sample\_011046840, Unigene4352\_Sample\_011046840, Unigene16855\_Sample\_011046840, Unigene19328\_Sample\_011046840, Unigene28904\_Sample\_011046840, Unigene3777\_Sample\_011046840, Unigene16784\_Sample\_011046840, Unigene40242\_Sample\_011046840 |
| 139 | Bacterial invasion of epithelial cells (no map in kegg database) Back to summary table | Unigene6378\_Sample\_011046840, Unigene9987\_Sample\_011046840, Unigene10035\_Sample\_011046840, Unigene10232\_Sample\_011046840, Unigene25850\_Sample\_011046840, Unigene29549\_Sample\_011046840, Unigene32514\_Sample\_011046840, Unigene35141\_Sample\_011046840, Unigene36475\_Sample\_011046840, Unigene37793\_Sample\_011046840, Unigene38706\_Sample\_011046840, Unigene40152\_Sample\_011046840, Unigene41220\_Sample\_011046840, Unigene42639\_Sample\_011046840, Unigene43211\_Sample\_011046840, Unigene43429\_Sample\_011046840, Unigene43556\_Sample\_011046840, Unigene6863\_Sample\_011046840, Unigene7979\_Sample\_011046840, Unigene9112\_Sample\_011046840, Unigene16996\_Sample\_011046840, Unigene18477\_Sample\_011046840, Unigene26575\_Sample\_011046840, Unigene27291\_Sample\_011046840, Unigene27902\_Sample\_011046840, Unigene28742\_Sample\_011046840, Unigene29916\_Sample\_011046840, Unigene32380\_Sample\_011046840, Unigene33370\_Sample\_011046840, Unigene34974\_Sample\_011046840, Unigene37892\_Sample\_011046840, Unigene38045\_Sample\_011046840, Unigene38612\_Sample\_011046840, Unigene38617\_Sample\_011046840, Unigene39883\_Sample\_011046840, Unigene40697\_Sample\_011046840, Unigene40834\_Sample\_011046840, Unigene40878\_Sample\_011046840, Unigene41128\_Sample\_011046840, Unigene41289\_Sample\_011046840, Unigene42356\_Sample\_011046840, Unigene42699\_Sample\_011046840, Unigene43258\_Sample\_011046840, Unigene43312\_Sample\_011046840, Unigene43492\_Sample\_011046840, Unigene25969\_Sample\_011046840, Unigene25979\_Sample\_011046840, Unigene26624\_Sample\_011046840, Unigene33383\_Sample\_011046840, Unigene33835\_Sample\_011046840, Unigene35086\_Sample\_011046840, Unigene40469\_Sample\_011046840, Unigene43149\_Sample\_011046840, Unigene6024\_Sample\_011046840, Unigene7782\_Sample\_011046840, Unigene7802\_Sample\_011046840, Unigene27657\_Sample\_011046840, Unigene28604\_Sample\_011046840, Unigene35377\_Sample\_011046840, Unigene35790\_Sample\_011046840, Unigene35929\_Sample\_011046840, Unigene38648\_Sample\_011046840, Unigene43466\_Sample\_011046840, Unigene4279\_Sample\_011046840, Unigene4818\_Sample\_011046840, Unigene5364\_Sample\_011046840, Unigene8088\_Sample\_011046840, Unigene18272\_Sample\_011046840, Unigene19332\_Sample\_011046840, Unigene25431\_Sample\_011046840, Unigene28754\_Sample\_011046840, Unigene29294\_Sample\_011046840, Unigene34305\_Sample\_011046840, Unigene35150\_Sample\_011046840, Unigene35981\_Sample\_011046840, Unigene36253\_Sample\_011046840, Unigene36734\_Sample\_011046840, Unigene40659\_Sample\_011046840, Unigene40784\_Sample\_011046840, Unigene42280\_Sample\_011046840, Unigene42871\_Sample\_011046840, Unigene43068\_Sample\_011046840, Unigene14379\_Sample\_011046840, Unigene24869\_Sample\_011046840, Unigene26621\_Sample\_011046840, Unigene28450\_Sample\_011046840, Unigene31181\_Sample\_011046840, Unigene34563\_Sample\_011046840, Unigene35901\_Sample\_011046840, Unigene38616\_Sample\_011046840, Unigene41881\_Sample\_011046840, Unigene42142\_Sample\_011046840, Unigene42770\_Sample\_011046840, Unigene956\_Sample\_011046840, Unigene18933\_Sample\_011046840, Unigene26785\_Sample\_011046840, Unigene32098\_Sample\_011046840, Unigene36992\_Sample\_011046840, Unigene40256\_Sample\_011046840, Unigene42008\_Sample\_011046840, Unigene42189\_Sample\_011046840, Unigene43533\_Sample\_011046840, Unigene43590\_Sample\_011046840, Unigene27885\_Sample\_011046840, Unigene32609\_Sample\_011046840, Unigene34041\_Sample\_011046840, Unigene34490\_Sample\_011046840, Unigene40960\_Sample\_011046840, Unigene6846\_Sample\_011046840, Unigene11899\_Sample\_011046840, Unigene20896\_Sample\_011046840, Unigene22073\_Sample\_011046840, Unigene24972\_Sample\_011046840, Unigene28829\_Sample\_011046840, Unigene30941\_Sample\_011046840, Unigene42811\_Sample\_011046840, Unigene2815\_Sample\_011046840, Unigene3219\_Sample\_011046840, Unigene15260\_Sample\_011046840, Unigene19127\_Sample\_011046840, Unigene31320\_Sample\_011046840, Unigene33389\_Sample\_011046840, Unigene38423\_Sample\_011046840, Unigene41158\_Sample\_011046840, Unigene12163\_Sample\_011046840, Unigene12868\_Sample\_011046840, Unigene15237\_Sample\_011046840, Unigene31135\_Sample\_011046840, Unigene33307\_Sample\_011046840, Unigene38066\_Sample\_011046840, Unigene41704\_Sample\_011046840, Unigene1256\_Sample\_011046840, Unigene4276\_Sample\_011046840, Unigene14930\_Sample\_011046840, Unigene17677\_Sample\_011046840, Unigene25276\_Sample\_011046840, Unigene31743\_Sample\_011046840, Unigene32265\_Sample\_011046840, Unigene37491\_Sample\_011046840, Unigene5921\_Sample\_011046840, Unigene35799\_Sample\_011046840 |
| 140 | RIG-I-like receptor signaling pathway Back to summary table | Unigene5467\_Sample\_011046840, Unigene40309\_Sample\_011046840, Unigene40471\_Sample\_011046840, Unigene42127\_Sample\_011046840, Unigene42759\_Sample\_011046840, Unigene43082\_Sample\_011046840, Unigene462\_Sample\_011046840, Unigene4474\_Sample\_011046840, Unigene16947\_Sample\_011046840, Unigene27236\_Sample\_011046840, Unigene29711\_Sample\_011046840, Unigene30539\_Sample\_011046840, Unigene39418\_Sample\_011046840, Unigene40865\_Sample\_011046840, Unigene6921\_Sample\_011046840, Unigene4529\_Sample\_011046840, Unigene13661\_Sample\_011046840, Unigene15839\_Sample\_011046840, Unigene41160\_Sample\_011046840, Unigene4713\_Sample\_011046840, Unigene33873\_Sample\_011046840, Unigene27\_Sample\_011046840, Unigene41530\_Sample\_011046840, Unigene42302\_Sample\_011046840, Unigene43154\_Sample\_011046840, Unigene14237\_Sample\_011046840, Unigene12677\_Sample\_011046840, Unigene29731\_Sample\_011046840, Unigene32604\_Sample\_011046840, Unigene1568\_Sample\_011046840, Unigene41410\_Sample\_011046840, Unigene19593\_Sample\_011046840, Unigene37186\_Sample\_011046840, Unigene39486\_Sample\_011046840, Unigene40053\_Sample\_011046840 |
| 141 | Vibrio cholerae infection Back to summary table | Unigene880\_Sample\_011046840, Unigene5915\_Sample\_011046840, Unigene27473\_Sample\_011046840, Unigene30862\_Sample\_011046840, Unigene39923\_Sample\_011046840, Unigene40184\_Sample\_011046840, Unigene43439\_Sample\_011046840, Unigene43577\_Sample\_011046840, Unigene4033\_Sample\_011046840, Unigene4101\_Sample\_011046840, Unigene6204\_Sample\_011046840, Unigene29794\_Sample\_011046840, Unigene31120\_Sample\_011046840, Unigene36762\_Sample\_011046840, Unigene38045\_Sample\_011046840, Unigene40128\_Sample\_011046840, Unigene41642\_Sample\_011046840, Unigene42065\_Sample\_011046840, Unigene42721\_Sample\_011046840, Unigene43258\_Sample\_011046840, Unigene43312\_Sample\_011046840, Unigene1399\_Sample\_011046840, Unigene3608\_Sample\_011046840, Unigene37068\_Sample\_011046840, Unigene40089\_Sample\_011046840, Unigene3161\_Sample\_011046840, Unigene6322\_Sample\_011046840, Unigene20538\_Sample\_011046840, Unigene27657\_Sample\_011046840, Unigene29099\_Sample\_011046840, Unigene35976\_Sample\_011046840, Unigene38028\_Sample\_011046840, Unigene4807\_Sample\_011046840, Unigene5364\_Sample\_011046840, Unigene15594\_Sample\_011046840, Unigene25431\_Sample\_011046840, Unigene25781\_Sample\_011046840, Unigene25956\_Sample\_011046840, Unigene31945\_Sample\_011046840, Unigene37629\_Sample\_011046840, Unigene38677\_Sample\_011046840, Unigene40513\_Sample\_011046840, Unigene8452\_Sample\_011046840, Unigene12084\_Sample\_011046840, Unigene28830\_Sample\_011046840, Unigene36382\_Sample\_011046840, Unigene36619\_Sample\_011046840, Unigene36687\_Sample\_011046840, Unigene38616\_Sample\_011046840, Unigene39303\_Sample\_011046840, Unigene41035\_Sample\_011046840, Unigene41881\_Sample\_011046840, Unigene42693\_Sample\_011046840, Unigene3760\_Sample\_011046840, Unigene17166\_Sample\_011046840, Unigene33547\_Sample\_011046840, Unigene40648\_Sample\_011046840, Unigene42385\_Sample\_011046840, Unigene522\_Sample\_011046840, Unigene2419\_Sample\_011046840, Unigene4849\_Sample\_011046840, Unigene6846\_Sample\_011046840, Unigene13322\_Sample\_011046840, Unigene13862\_Sample\_011046840, Unigene15087\_Sample\_011046840, Unigene16531\_Sample\_011046840, Unigene23323\_Sample\_011046840, Unigene24674\_Sample\_011046840, Unigene33749\_Sample\_011046840, Unigene38900\_Sample\_011046840, Unigene41078\_Sample\_011046840, Unigene2196\_Sample\_011046840, Unigene2815\_Sample\_011046840, Unigene3219\_Sample\_011046840, Unigene19865\_Sample\_011046840, Unigene23676\_Sample\_011046840, Unigene25046\_Sample\_011046840, Unigene12868\_Sample\_011046840, Unigene14612\_Sample\_011046840, Unigene15237\_Sample\_011046840, Unigene31135\_Sample\_011046840, Unigene33692\_Sample\_011046840, Unigene11201\_Sample\_011046840, Unigene17721\_Sample\_011046840, Unigene18559\_Sample\_011046840, Unigene20157\_Sample\_011046840, Unigene28467\_Sample\_011046840, Unigene5921\_Sample\_011046840, Unigene15362\_Sample\_011046840, Unigene20109\_Sample\_011046840, Unigene32555\_Sample\_011046840, Unigene36661\_Sample\_011046840, Unigene40453\_Sample\_011046840 |
| 142 | Calcium signaling pathway Back to summary table | Unigene259\_Sample\_011046840, Unigene880\_Sample\_011046840, Unigene1879\_Sample\_011046840, Unigene2690\_Sample\_011046840, Unigene4875\_Sample\_011046840, Unigene5343\_Sample\_011046840, Unigene5662\_Sample\_011046840, Unigene5831\_Sample\_011046840, Unigene6100\_Sample\_011046840, Unigene23133\_Sample\_011046840, Unigene24801\_Sample\_011046840, Unigene30862\_Sample\_011046840, Unigene32706\_Sample\_011046840, Unigene34179\_Sample\_011046840, Unigene35023\_Sample\_011046840, Unigene35688\_Sample\_011046840, Unigene42287\_Sample\_011046840, Unigene43363\_Sample\_011046840, Unigene43439\_Sample\_011046840, Unigene2924\_Sample\_011046840, Unigene3812\_Sample\_011046840, Unigene4033\_Sample\_011046840, Unigene4057\_Sample\_011046840, Unigene4101\_Sample\_011046840, Unigene4234\_Sample\_011046840, Unigene5591\_Sample\_011046840, Unigene5795\_Sample\_011046840, Unigene6428\_Sample\_011046840, Unigene6500\_Sample\_011046840, Unigene7302\_Sample\_011046840, Unigene7868\_Sample\_011046840, Unigene11592\_Sample\_011046840, Unigene33208\_Sample\_011046840, Unigene34930\_Sample\_011046840, Unigene36252\_Sample\_011046840, Unigene36690\_Sample\_011046840, Unigene36724\_Sample\_011046840, Unigene38876\_Sample\_011046840, Unigene39220\_Sample\_011046840, Unigene40128\_Sample\_011046840, Unigene40273\_Sample\_011046840, Unigene41669\_Sample\_011046840, Unigene41856\_Sample\_011046840, Unigene42065\_Sample\_011046840, Unigene42217\_Sample\_011046840, Unigene42777\_Sample\_011046840, Unigene42873\_Sample\_011046840, Unigene43481\_Sample\_011046840, Unigene43557\_Sample\_011046840, Unigene2310\_Sample\_011046840, Unigene3608\_Sample\_011046840, Unigene5166\_Sample\_011046840, Unigene7373\_Sample\_011046840, Unigene11142\_Sample\_011046840, Unigene14012\_Sample\_011046840, Unigene23432\_Sample\_011046840, Unigene26123\_Sample\_011046840, Unigene33983\_Sample\_011046840, Unigene38834\_Sample\_011046840, Unigene39204\_Sample\_011046840, Unigene41011\_Sample\_011046840, Unigene42172\_Sample\_011046840, Unigene42204\_Sample\_011046840, Unigene3161\_Sample\_011046840, Unigene6322\_Sample\_011046840, Unigene7605\_Sample\_011046840, Unigene28344\_Sample\_011046840, Unigene39054\_Sample\_011046840, Unigene39273\_Sample\_011046840, Unigene39660\_Sample\_011046840, Unigene123\_Sample\_011046840, Unigene4635\_Sample\_011046840, Unigene4807\_Sample\_011046840, Unigene5969\_Sample\_011046840, Unigene6292\_Sample\_011046840, Unigene30572\_Sample\_011046840, Unigene31945\_Sample\_011046840, Unigene35443\_Sample\_011046840, Unigene36673\_Sample\_011046840, Unigene36788\_Sample\_011046840, Unigene36860\_Sample\_011046840, Unigene38586\_Sample\_011046840, Unigene41391\_Sample\_011046840, Unigene42218\_Sample\_011046840, Unigene43001\_Sample\_011046840, Unigene43231\_Sample\_011046840, Unigene334\_Sample\_011046840, Unigene14179\_Sample\_011046840, Unigene15903\_Sample\_011046840, Unigene28124\_Sample\_011046840, Unigene28872\_Sample\_011046840, Unigene31662\_Sample\_011046840, Unigene36619\_Sample\_011046840, Unigene41773\_Sample\_011046840, Unigene42693\_Sample\_011046840, Unigene5914\_Sample\_011046840, Unigene12153\_Sample\_011046840, Unigene24257\_Sample\_011046840, Unigene28610\_Sample\_011046840, Unigene33660\_Sample\_011046840, Unigene35787\_Sample\_011046840, Unigene35815\_Sample\_011046840, Unigene38267\_Sample\_011046840, Unigene38778\_Sample\_011046840, Unigene39912\_Sample\_011046840, Unigene40648\_Sample\_011046840, Unigene42385\_Sample\_011046840, Unigene42769\_Sample\_011046840, Unigene32238\_Sample\_011046840, Unigene39755\_Sample\_011046840, Unigene1038\_Sample\_011046840, Unigene4849\_Sample\_011046840, Unigene6944\_Sample\_011046840, Unigene17676\_Sample\_011046840, Unigene26668\_Sample\_011046840, Unigene32479\_Sample\_011046840, Unigene34466\_Sample\_011046840, Unigene35953\_Sample\_011046840, Unigene37392\_Sample\_011046840, Unigene40391\_Sample\_011046840, Unigene670\_Sample\_011046840, Unigene3044\_Sample\_011046840, Unigene8614\_Sample\_011046840, Unigene29065\_Sample\_011046840, Unigene32197\_Sample\_011046840, Unigene37786\_Sample\_011046840, Unigene39577\_Sample\_011046840, Unigene42805\_Sample\_011046840, Unigene2711\_Sample\_011046840, Unigene4493\_Sample\_011046840, Unigene5106\_Sample\_011046840, Unigene13511\_Sample\_011046840, Unigene21816\_Sample\_011046840, Unigene26126\_Sample\_011046840, Unigene32062\_Sample\_011046840, Unigene32864\_Sample\_011046840, Unigene33692\_Sample\_011046840, Unigene38118\_Sample\_011046840, Unigene41488\_Sample\_011046840, Unigene7388\_Sample\_011046840, Unigene11201\_Sample\_011046840, Unigene11765\_Sample\_011046840, Unigene15158\_Sample\_011046840, Unigene25408\_Sample\_011046840, Unigene26432\_Sample\_011046840, Unigene26954\_Sample\_011046840, Unigene28337\_Sample\_011046840, Unigene34487\_Sample\_011046840, Unigene37249\_Sample\_011046840, Unigene15244\_Sample\_011046840, Unigene23365\_Sample\_011046840, Unigene24277\_Sample\_011046840, Unigene31562\_Sample\_011046840, Unigene32614\_Sample\_011046840, Unigene36221\_Sample\_011046840, Unigene36359\_Sample\_011046840, Unigene40453\_Sample\_011046840, Unigene43443\_Sample\_011046840 |
| 143 | alpha-Linolenic acid metabolism Back to summary table | Unigene5059\_Sample\_011046840, Unigene7232\_Sample\_011046840, Unigene30203\_Sample\_011046840, Unigene33901\_Sample\_011046840, Unigene40875\_Sample\_011046840, Unigene43421\_Sample\_011046840, Unigene33052\_Sample\_011046840, Unigene1485\_Sample\_011046840, Unigene34550\_Sample\_011046840, Unigene42268\_Sample\_011046840, Unigene26398\_Sample\_011046840, Unigene39024\_Sample\_011046840, Unigene32011\_Sample\_011046840, Unigene32337\_Sample\_011046840, Unigene34351\_Sample\_011046840, Unigene41689\_Sample\_011046840, Unigene42020\_Sample\_011046840, Unigene33809\_Sample\_011046840 |
| 144 | Fatty acid elongation in mitochondria Back to summary table | Unigene32859\_Sample\_011046840, Unigene35486\_Sample\_011046840, Unigene2498\_Sample\_011046840, Unigene31044\_Sample\_011046840, Unigene35511\_Sample\_011046840, Unigene37244\_Sample\_011046840, Unigene23987\_Sample\_011046840, Unigene42353\_Sample\_011046840, Unigene13731\_Sample\_011046840, Unigene22392\_Sample\_011046840, Unigene37714\_Sample\_011046840, Unigene41037\_Sample\_011046840, Unigene9645\_Sample\_011046840, Unigene27082\_Sample\_011046840, Unigene30750\_Sample\_011046840, Unigene17569\_Sample\_011046840, Unigene13340\_Sample\_011046840, Unigene32278\_Sample\_011046840 |
| 145 | Jak-STAT signaling pathway Back to summary table | Unigene4790\_Sample\_011046840, Unigene27951\_Sample\_011046840, Unigene39096\_Sample\_011046840, Unigene43193\_Sample\_011046840, Unigene43496\_Sample\_011046840, Unigene5389\_Sample\_011046840, Unigene6996\_Sample\_011046840, Unigene7091\_Sample\_011046840, Unigene10748\_Sample\_011046840, Unigene24698\_Sample\_011046840, Unigene26575\_Sample\_011046840, Unigene32380\_Sample\_011046840, Unigene34035\_Sample\_011046840, Unigene34085\_Sample\_011046840, Unigene34974\_Sample\_011046840, Unigene35122\_Sample\_011046840, Unigene38617\_Sample\_011046840, Unigene38866\_Sample\_011046840, Unigene39938\_Sample\_011046840, Unigene42164\_Sample\_011046840, Unigene43509\_Sample\_011046840, Unigene976\_Sample\_011046840, Unigene38847\_Sample\_011046840, Unigene43149\_Sample\_011046840, Unigene3335\_Sample\_011046840, Unigene7408\_Sample\_011046840, Unigene20325\_Sample\_011046840, Unigene23045\_Sample\_011046840, Unigene31893\_Sample\_011046840, Unigene32463\_Sample\_011046840, Unigene36320\_Sample\_011046840, Unigene43068\_Sample\_011046840, Unigene7250\_Sample\_011046840, Unigene21908\_Sample\_011046840, Unigene24869\_Sample\_011046840, Unigene25365\_Sample\_011046840, Unigene39525\_Sample\_011046840, Unigene4139\_Sample\_011046840, Unigene14255\_Sample\_011046840, Unigene18933\_Sample\_011046840, Unigene21594\_Sample\_011046840, Unigene26785\_Sample\_011046840, Unigene28751\_Sample\_011046840, Unigene38000\_Sample\_011046840, Unigene40189\_Sample\_011046840, Unigene42008\_Sample\_011046840, Unigene332\_Sample\_011046840, Unigene39613\_Sample\_011046840, Unigene643\_Sample\_011046840, Unigene1368\_Sample\_011046840, Unigene12234\_Sample\_011046840, Unigene20896\_Sample\_011046840, Unigene24335\_Sample\_011046840, Unigene42811\_Sample\_011046840, Unigene2209\_Sample\_011046840, Unigene5726\_Sample\_011046840, Unigene13067\_Sample\_011046840, Unigene21176\_Sample\_011046840, Unigene27623\_Sample\_011046840, Unigene31320\_Sample\_011046840, Unigene41560\_Sample\_011046840, Unigene18122\_Sample\_011046840, Unigene35972\_Sample\_011046840, Unigene39584\_Sample\_011046840, Unigene41704\_Sample\_011046840, Unigene16035\_Sample\_011046840, Unigene26172\_Sample\_011046840, Unigene32265\_Sample\_011046840, Unigene37491\_Sample\_011046840 |
[truncated: 224,327 more chars]
